# MelanomaDB: A Web Tool for Integrative Analysis of Melanoma Genomic Information to Identify Disease-Associated Molecular Pathways

**DOI:** 10.3389/fonc.2013.00184

**Published:** 2013-07-16

**Authors:** Alexander J. Trevarton, Michael B. Mann, Christoph Knapp, Hiromitsu Araki, Jonathan D. Wren, Steven Stones-Havas, Michael A. Black, Cristin G. Print

**Affiliations:** ^1^Department of Molecular Medicine and Pathology, School of Medical Sciences, University of Auckland, Auckland, New Zealand; ^2^Cancer Research Program, The Methodist Hospital Research Institute, Houston, TX, USA; ^3^Bioinformatics Institute, University of Auckland, Auckland, New Zealand; ^4^Department of Bioscience and Biotechnology, Faculty of Agriculture, Kyushu University, Fukuoka, Japan; ^5^Arthritis and Clinical Immunology Research Program, Oklahoma Medical Research Foundation, Oklahoma City, OK, USA; ^6^Department of Biochemistry and Molecular Biology, University of Oklahoma Health Sciences Center, Oklahoma City, OK, USA; ^7^Biomatters Ltd., Auckland, New Zealand; ^8^Department of Biochemistry, University of Otago, Dunedin, New Zealand; ^9^Maurice Wilkins Centre, Auckland, New Zealand

**Keywords:** melanoma, mutation, molecular pathway, MelanomaDB, gene set analysis, BaseSpace

## Abstract

Despite on-going research, metastatic melanoma survival rates remain low and treatment options are limited. Researchers can now access a rapidly growing amount of molecular and clinical information about melanoma. This information is becoming difficult to assemble and interpret due to its dispersed nature, yet as it grows it becomes increasingly valuable for understanding melanoma. Integration of this information into a comprehensive resource to aid rational experimental design and patient stratification is needed. As an initial step in this direction, we have assembled a web-accessible melanoma database, MelanomaDB, which incorporates clinical and molecular data from publically available sources, which will be regularly updated as new information becomes available. This database allows complex links to be drawn between many different aspects of melanoma biology: genetic changes (e.g., mutations) in individual melanomas revealed by DNA sequencing, associations between gene expression and patient survival, data concerning drug targets, biomarkers, druggability, and clinical trials, as well as our own statistical analysis of relationships between molecular pathways and clinical parameters that have been produced using these data sets. The database is freely available at http://genesetdb.auckland.ac.nz/melanomadb/about.html. A subset of the information in the database can also be accessed through a freely available web application in the Illumina genomic cloud computing platform BaseSpace at http://www.biomatters.com/apps/melanoma-profiler-for-research. The MelanomaDB database illustrates dysregulation of specific signaling pathways across 310 exome-sequenced melanomas and in individual tumors and identifies the distribution of somatic variants in melanoma. We suggest that MelanomaDB can provide a context in which to interpret the tumor molecular profiles of individual melanoma patients relative to biological information and available drug therapies.

## Introduction

### The growth and complexity of melanoma genomic data

**Figure 1:**
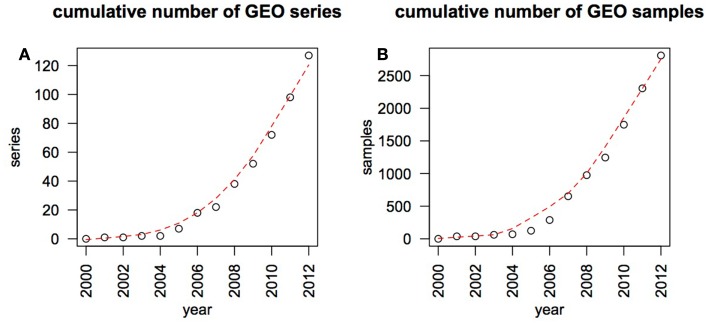
**Growth of melanoma genomic data in the GEO database**. The GEO database was searched on a year by year basis, using the MESH term “melanoma” and excluding records containing the phrase “cell line.” By the end of January 2013 GEO contained 128 data series made up of 2819 samples that match these search criteria. The cumulative number of data series (submitted experiments) **(A)** and individual samples **(B)** are plotted as black circles, overlaid by a red trend line fitted over this data using the loess method.

Melanoma researchers are faced with a rapidly growing amount of useful molecular and clinical data, particularly gene expression information. This rapid growth can be illustrated by surveying the Gene Expression Omnibus (GEO) (1), an international repository that contains a large subset of the published gene expression data (Figure 1). Largely based on genomic data, our understanding of the genes involved in melanoma progression has advanced from focused investigations of candidate genes to studies on a whole-genome scale (2). The advent of next-generation sequencing (NGS) in particular has opened up a floodgate of data, from the published sequence of the first melanoma genome in the beginning of 2010 (3), to more recent whole-exome studies sequencing more than one hundred tumors (4, 5). Melanoma genomic data is poised to grow rapidly with the advent of large-scale initiatives such as Australia’s Melanoma Genome Project^1^, melanoma analysis in The Cancer Genome Atlas (TCGA) project^2^ as well as the melanoma sequencing projects underway at several individual institutions.

### Limitations of current techniques

Unfortunately, information pertinent to melanoma exists in a diverse range of formats and locations. For example, relevant data about a single gene of interest may include information about the encoded protein’s structure, cellular location, and function, contribution to molecular pathways, drugs that target the protein, the gene, or protein’s utility as a biomarker, genome-wide association studies, mutation frequency, chromosomal aberrations, as well as RNA expression associations with metastasis, treatment response and patient survival, clinical SNP associations, and the results of literature mining. Even within the single data type of tumor DNA sequencing, a variety of methods have been used to implicate genes in melanoma initiation and progression, and these different methods produce data in differing formats. Ideally, all these diverse forms of data could be used by researchers in an integrated fashion to triangulate in on clinically important genes.

As a further challenge, genomic information in melanoma is particularly dense due to the high mutation rate found in melanomas of sun-exposed skin (6). This is likely to be due to both ultraviolet radiation-induced DNA damage and defects in DNA repair mechanisms (3). In addition, sequencing studies suggest that malignant melanoma is a relatively heterogeneous neoplasia with a range of driver mutations (5). Despite its potential value, coherent analysis of melanoma genomic information remains difficult for individual researchers. Data repositories such as Oncomine (7), Ingenuity Pathways Analysis^3^, the Catalogue of Somatic Mutations in Cancer (COSMIC) (8, 9), and the Broad Institute’s Melanoma Genomics Portal (10) bring together a massive amount of useful melanoma data. However, these disparate resources do not yet enable the full potential of integrated analysis of molecular pathways across different types of data associated with melanoma.

### Potential clinical use of molecular pathway data about individual tumors

Tumor development involves multiple genes encoding proteins and non-coding RNAs operating in molecular pathways. Therefore, inference of molecular pathway activity from tumor genomic data using methods such as gene set analysis (GSA) (11) is useful in oncology (12, 13). Gene sets used for analysis may consist of co-expressed genes downstream of a specific molecular pathway (14) or genes that share common transcription factor binding sites (15). Statistical summaries of these gene sets have been used to infer molecular pathway activity, and these gene sets are frequently conserved across species (16). GSA has identified several molecular pathways associated with melanoma (17, 18), and can be used to identify the putative functional changes caused by the mutation, DNA gain or loss, and/or altered expression of genes in a particular patient’s tumor. Popular GSA tools include GATHER (19), DAVID (20), GSEA (21), and GeneSetDB (22).

The number of clinically available targeted therapies for melanoma remains limited compared to the diverse genetic drivers of this tumor. Nevertheless, identification of drugs targeting a small number of melanoma drivers has been a major advance. For example, Vemurafenib targets the Mitogen Activated Protein Kinase (MAPK) pathway molecule BRAF (23). However, Vemurafenib is only indicated in *BRAF* V600E or V600K containing tumors and the majority of treated patients show relatively short term remission, with their relapse almost certainly caused by re-activation of the MAPK pathway, commonly through mutations in *NRAS* or *PDGFRB* (24). We propose that integration of molecular pathway data at both the patient population scale and individual tumor scale could help researchers better understand phenomena such as Vemurafenib resistance, and permit identification of rationally selected combinatorial therapies based on molecular stratification of patients.

### Experimental objectives

In the work described here, we have amalgamated a diverse range of genomic and clinical melanoma data, on the scales of both patient population and individual tumor into a single resource. This resource is provided as a downloadable file that can be searched and filtered using any spread sheet application. To facilitate use of this resource in the context of molecular pathways, we also provide a web-accessible SQL database named MelanomaDB, through which researchers can perform GSA using integrated melanoma data of several types. A subset of the information in the database can also be accessed through a freely available web application in the Illumina genomic cloud computing platform BaseSpace. While other disease-specific databases exist for other cancers such as lung (25) and ovarian (26) cancer, we know of no other database similar to ours dealing with melanoma. Furthermore, we believe that MelanomaDB’s breadth across sequence and microarray data, biological and pharmacological gene sets, and pathway information, in addition to its usability and its melanoma focus, make it unique. In this paper, we use information assembled in MelanomaDB in several downstream analyses to demonstrate the utility of this resource for finding relationships between molecular pathways and clinical parameters, including the mutational patterns of members of molecular pathways (27) in individual tumors. We hope this tool will prove increasingly useful as it expands when new tumor data becomes available. In particular, we hope that it will provide a context in which to interpret the tumor molecular profiles of individual melanoma patients.

## Materials and Methods

### Overview of the construction of melanoma gene sets

To facilitate an integrative analysis of melanoma information we combined a variety of melanoma data in the form of gene sets, attempting to collect information for all genes in the genome. These melanoma gene sets were groups of genes that shared biological or clinical relevance for melanoma, derived from five types of publically available information: drug and biomarker information, druggability, literature relationship strength, disease-specific survival, and somatic mutation data. Drug information includes information on compounds and the proteins they target, while Druggability information comprises of estimations of the degree to which proteins are amenable to targeting by drugs, and protein characteristics relevant to this. A detailed description of this information is available in Data Sheet 1 in Supplementary Material.

### Sources of specific information

Further explanations of the gene sets used are in the MelanomaDB help page at http://genesetdb.auckland.ac.nz/melanomadb/help.html

#### Drug and biomarker information

Drug information was taken from online databases DrugBank version 3 (28), KEGG DRUG (27), Therapeutic Targets Database (29), and ClinicalTrials.gov. Biomarker information was taken from published papers by Gould Rothberg et al. (30), Schramm and Mann (31), Utikal et al. (85), Mehta et al. (32), and from the database KEGG BRITE (27). It should be noted that gene sets such as those derived from DrugBank include all genes encoding proteins to which each drug binds, including both intended and unintended targets. However, metabolising enzymes, transporters and carrier proteins are excluded. For example, targets of the drug Cetuximab include the intended target (the human epidermal growth factor receptor) but also compliment components and Fc receptors, as is expected due to the nature of this drug as an antibody^4^. For further explanations of the gene sets used see the MelanomaDB help page at http://genesetdb.auckland.ac.nz/melanomadb/help.html

#### Druggability information

Druggability data was sourced from the Sophic Integrated Druggable Genome Database (33), EBI’s DrugEBIlity database (34), and published papers by Li and Lai (35) and Tiedemann et al. (36). Data on protein characteristics relevant to druggability were taken from Affymetrix annotations^5^, and online databases UniProt Consortium (37), Secreted Protein Database (38), and KinBase (39).

#### Literature and genomic data relationship strength information

Information on Literature Relationship strength was derived from the IRIDESCENT (40) and GAMMA (41) software packages. IRIDESCENT searches every published MEDLINE abstract for associations between objects, and creates a network of tentative relationships between these objects. Objects encompass genes, diseases, phenotypes, chemical compounds, drugs, and ontology categories. The relative strength of association between two objects is determined by the frequency in which they appear in the same abstract or sentence. Here, this network is used to score the strength of association between genes and the terms “melanoma” or “metastatic melanoma.”

GAMMA conducts a meta-analysis of gene expression behavior across 16,000 wide-ranging microarray experiments to identify genes that are consistently and specifically co-expressed across heterogeneous experimental conditions. In this way GAMMA extends the connections in IRIDESCENT’s association network to genes without any published associations to melanoma by identifying which of these genes are consistently co-expressed with multiple known melanoma genes. To date, GAMMA has been used successfully to identify phenotypes and/or disease relevance for several previously uncharacterized genes (42–45).

#### Disease-specific survival data

Strength of statistical associations between RNA abundance and melanoma-specific survival were gathered from several published studies, and from our additional statistical analysis of two published sets of linked microarray and clinical data. Associations between gene expression in melanomas and patient survival were taken directly from John et al. (46), Mandruzzato et al. (47), and Journe et al. (48), and associations between gene expression and metastasis were taken directly from Timar et al. (49). We performed our own analyses on the microarray data of Bogunovic et al. (50) and Jönsson et al. (51) based on patient survival data and Affymetrix CEL files retrieved from GEO. The Bogunovic study’s raw Affymetrix data was normalized using RMA normalization performed using the affy package in the R statistical software (52). The Illumina data from the Jonsson et al. study was obtained in a normalized format, however, we removed three patients for whom patient survival data was missing, and adjusted all microarray values by adding the minimum value in order to eliminate negative values. R was used to split the patients into two groups, create a survival object for each group and then compare these two survival objects using a Log Rank test. For each probe set this splitting was performed nine times, once at each RNA abundance decile across the patient population. R was also used to fit a Cox proportional hazards regression model for each probe set.

To facilitate the use of these data in exploratory analyses for hypothesis generation, we also generated additional gene sets in which we aggregated several different RNA associations with patient survival to allow broader surveys. For example, four gene sets were identified from the expression and survival data of Bogunovic et al. (50) using different statistical criteria.

#### Somatic variant data

Multiple studies reporting melanoma variants were collated for use with MelanomaDB. A literature review identified 11 exome sequencing studies suitable for inclusion (4–6, 53–60). In addition, the Cancer Cell Line Encyclopedia (61), and the Sanger Institute’s COSMIC (8, 9), and Matched Pair Cancer Cell Lines (3) were searched for mutations detected in melanoma cell lines. In total, we collected data on 58 established melanoma cell lines, 119 primary “short-passage” cell lines, 38 primary tumors, and 96 metastatic melanoma tumors. Non-silent variants were reported in 16,488 genes. With the exception of the 10 samples from the 2010 study of Berger et al. (53), and some of the samples from COSMIC, these samples have all been paired with matched normal samples to ensure that the variants reported are somatic. In the current iteration of this database only non-synonymous coding mutations, indels, splice-site mutations, and structural rearrangements (including gene fusions and read-through transcripts) are included. Synonymous coding mutations are not included. Presently, this somatic variant data includes more than 35,000 non-synonymous coding mutations, and more than 3,500 structural rearrangements and indels. We have not provided this somatic variant data as a supplementary file but instead invite readers to contact us to obtain the links to this data. We do this so we can ensure that access permission and ethical issues associated with this individual patient data are adhered to.

### Amalgamation of all data into gene sets

To facilitate the construction of gene sets, all data described above was combined into a single matrix, which is available as Data Sheet 2 in Supplementary Material. This matrix is gene-based and uses Entrez Gene ID as a unique index for each gene^6^. Every gene is represented by one row, and each column contains data from a single source. Columns annotating genes with references to other databases were derived from NCBI’s Gene database FTP directory^7^ and supplemented by Affymetrix annotations (see text footnote 5).

From this data matrix, a number of gene sets were derived. In most cases, columns of the matrix were converted directly into gene sets by including in that set every gene with an entry in that column. In some cases, such as statistical associations between RNA expression and patient survival, a cut-off was required for defining gene set membership. For example, only genes encoding proteins with positive DrugEBIlity ensemble scores were included in the gene set “DrugEBIlity: Positive ensemble scores.” A further description of the melanoma gene sets is available in Data Sheet 1 in Supplementary Material.

### SQL database generation

To facilitate access, combination, and filtering of different types of genomic data related to melanoma, and interpretation of this data in terms of molecular pathways and functional categories, the data matrix described above was used to generate a web-accessible SQL database named MelanomaDB. The web interface is implemented using Apache, PHP, Javascript, and HTML. The meta-gene set database GeneSetDB (22) was accessed from within MelanomaDB to identify the intersection between melanoma-specific gene sets and gene sets related to biological functions and molecular pathways. The R framework was used for statistical calculations. GSA was performed using the hyper-geometric distribution to calculate the probability of overrepresentation, followed by multiple testing correction using the Benjamini and Hochberg method (62).

### BaseSpace application preparation

A subset of the information in MelanomaDB is also included in a freely available Illumina BaseSpace application. This BaseSpace application retrieves a tumor and corresponding normal germ line sequence pair from the BaseSpace archive or the user’s own BaseSpace account as vcf files. Then, variants present in the tumor but the not normal germ line tissue of the patient are identified using the Genome Analysis Tool Kit’s SelectVariants java tool (63). This list of tumor variant genes is identified. Then, the molecular pathways these genes correspond to, along with any statistically significant pathway enrichment within the list of variant genes and targeting drugs, are retrieved from the GeneSetDB pathway analysis web tool (22). A diagram showing tumor variant genes in the context of molecular pathways is generated using the KEGG, Reactome, and Biocarta pathways included in the R graphite package (64), and a clustered heatmap showing how the genetic variants in the sample tumor compare to variants in the 310 tumors cataloged in MelanomaDB is generated. This clustered heatmap is generated: (i) using a modification of the heatmap.2 function from the R gtools package (see Data Sheet 5 in Supplementary Material) (65), using the “binary” method for distance calculation and the “single” method for clustering and (ii) as a reverse-orientation waterfall plot to illustrate patterns of somatic variant co-occurrence in melanoma.

### Assembly of information for individual tumors

From the exome and whole-genome sequencing information assembled above, we constructed a tumor-based matrix in which each row was a gene, each column was an individual tumor and each cell described any somatic variants present in a certain gene for a certain tumor. After duplicated tumors were removed, this somatic variant data included 310 samples, 183, and 72 of which had somatic alterations in the *BRAF* and *NRAS* genes, respectively. When multiple sequenced tumors or cell lines from the same patient were available, the union of somatic variants found in these samples was used. Links to the papers and their supplementary web sites used to construct this tumor-specific somatic variant data is available in Data Sheet 3 in Supplementary Material. The authors can assist researchers with the precise sources of information used to construct this resource.

### Visualization

The statistical software R was used to construct a clustered heat map of tumor variants for genes included in the KEGG “Melanoma” signalling pathway with a modified heatmap.2 function of the R package “gplots,”^8^ using the “binary” method for distance and the “single” method for clustering. R was also used to draw gene network diagrams. Molecular pathways were obtained from the pathways included in the graphite R package^9^ and were plotted using the graphite (see text footnote 9) R package.

**Figure 2:**
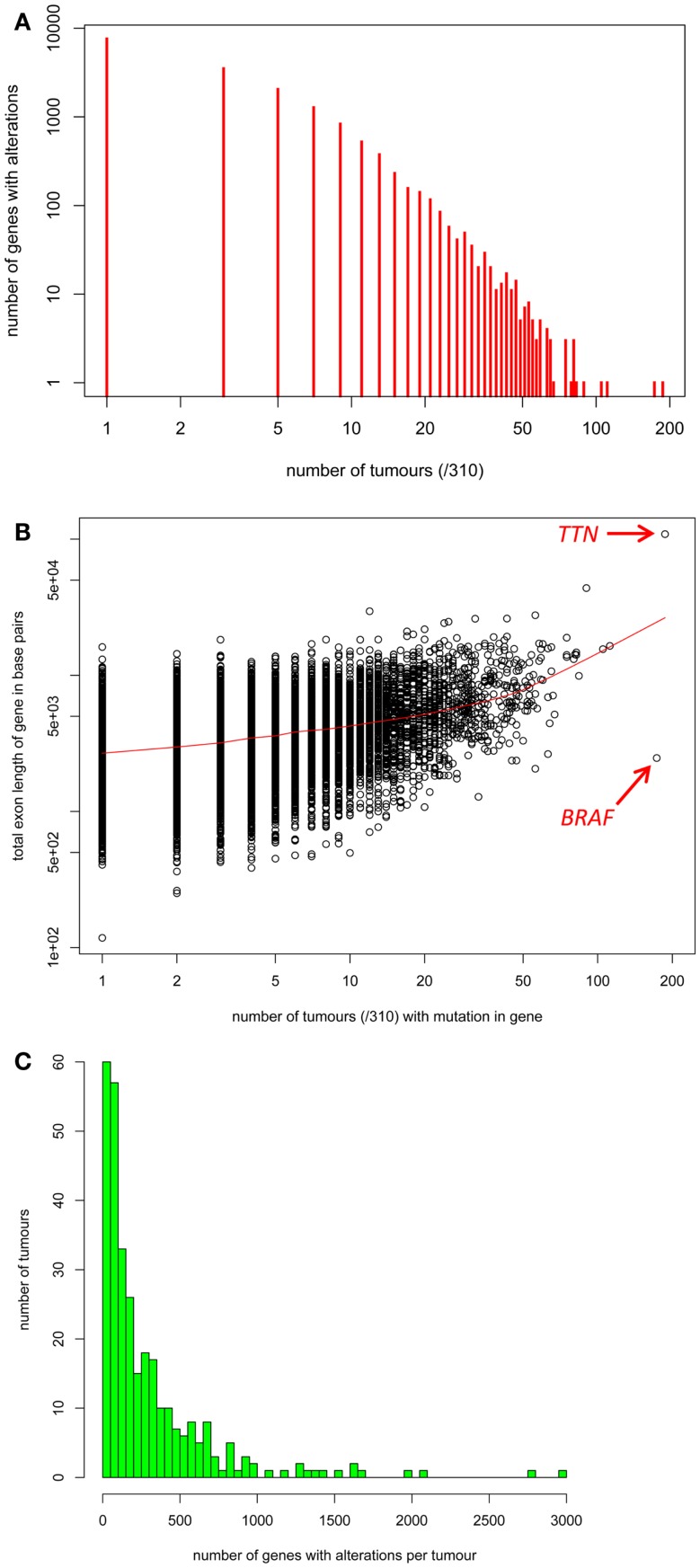
**(A)** The distribution of the number of tumors with somatic alterations in each individual gene. **(B)** Each gene’s total exon length in base pairs (*y*-axis) versus the number of the 310 tumors with a mutation in that gene (*x*-axis). **(C)** The distribution of the number of genes with somatic alterations in each individual tumor.

The R scripts used to generate Figures 2A–C as well as the pathway diagrams and heatmaps in Figures 4–7 are given in Data Sheet 5 in Supplementary Material.

## Results and Discussion

Here we describe the assembly and use of the MelanomaDB database.

### Assembly of melanoma genomic information from diverse sources into a melanoma data matrix

Firstly, a melanoma data matrix (Data Sheet 2 in Supplementary Material) was constructed, with genes (or genomic loci in some cases) as rows. The columns of this matrix represent diverse features of biological functions related to melanoma and are described in Data Sheet 1 in Supplementary Material. This melanoma data matrix can be utilized in a variety of ways. Most simply, researchers can access a variety of data pertaining to their particular gene of interest. The melanoma data matrix can also be manipulated with spread sheet software to sort, find, and filter information in order to generate gene lists useful for hypothesis generation.

### Assembly of somatic variant information for melanomas of individual patients

Next, we assembled as much information about somatic variation in individual exome-sequenced and genome-wide-sequenced melanomas as possible. We gathered information about somatic variations in 58 established melanoma cell lines, 119 primary “fresh” cell lines, 38 primary tumors, and 96 metastatic melanoma tumors, which was appended to the information matrix described above (Data Sheet 3 in Supplementary Material, Tab “Tables Used”). Information about non-synonymous coding mutations, structural rearrangements, and indels was included (intronic and synonymous coding mutations were excluded from the current iteration of this data resource). The information contained in Data Sheet 2 in Supplementary Material was read into the statistical environment R and visualized, as described in the Section “Materials and Methods” and Data Sheet 5 in Supplementary Material. Firstly, the distribution of somatic variations for individual genes is shown in Figure 2A. The majority of genes showed somatic variations in only small numbers of tumors. Comparison of each gene’s total exon length versus the number of tumors with a mutation in that gene using R (Figure 2B), revealed a statistically significant but weak correlation between somatic variation frequency and total exon length (Pearson’s correlation coefficient = 0.47, *p* ≤ 0.001). Although variations in large genes such as *Titan* (*TTN*) have been implicated as cancer drivers, these may also occur in so many melanomas due to large gene size increasing the likelihood of passenger mutations. However, the *BRAF* gene clearly stands out as frequently mutated in melanomas despite its moderate length. The distribution of the number of genes with somatic alterations in each individual tumor was performed using R and is shown in Figure 2C.

### Use of the combined melanoma information

**Figure 3:**
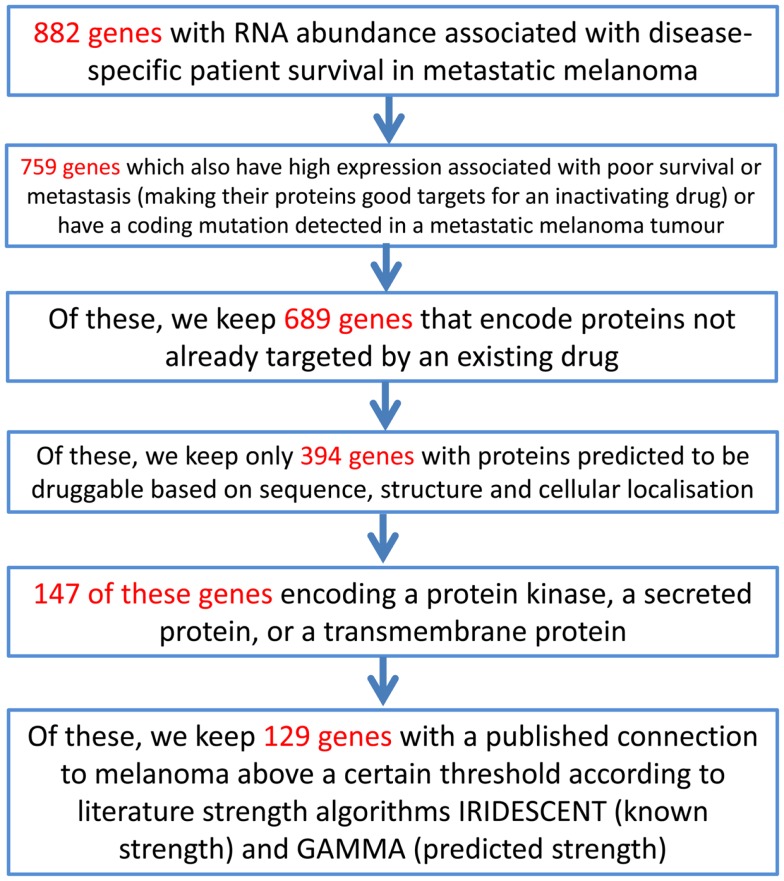
**An example of a process through which the melanoma data matrix (Data Sheet 2 in Supplementary Material) can be used to generate a short list of putative drug targets**. The initial gene list consists of those genes in the melanoma data matrix (Data Sheet 2 in Supplementary Material) that have an entry in any of the columns describing the data of the studies of Jönsson et al. (51), John et al. (46), Mandruzzato et al. (47), Journe et al. (48), or Bogunovic et al. (50). (Please note that this example is for use with the data matrix in Data Sheet 2 in Supplementary Material, rather than for the MelanomaDB web tool).

As an example of using the information assembled above, an approach to identifying novel candidate novel drug targets for melanoma using this melanoma data matrix (Data Sheet 2 in Supplementary Material) can be performed by filtering and sorting Data Sheet 2 in Supplementary Material in a spreadsheet application and is described in Figure 3.

This process generates a short list of 129 genes that can be examined more closely in order to select a final list of genes that may warrant investigation in the laboratory. A variant on this approach may be to place more weight on particular data, for example, on selected druggability measures. By using a spreadsheet application to take the 987 genes in Data Sheet 2 in Supplementary Material encoding proteins that have scored greater than 0.5 on either DrugEBIlity’s Ensemble score or Li and Lai’s druggability measure, and eliminating proteins already targeted by existing drugs, we have a list of 803 genes that are predicted to be probably druggable. Of these, 21 also have high RNA expression significantly associated with reduced disease-free survival in melanoma patients, making them possible new drug targets. These genes are *AKR7A2*, *AKR7A3*, *ARIH1*, *ARPC1A*, *CD163*, *DCT*, *DHRS11*, *DUS4L*, *FAH*, *FSCN1*, *HS3ST3A1*, *NRAS*, *NUP155*, *PANK2*, *PRMT3*, *QTRT1*, *RAD1*, *RAE1*, *SUV39H2*, *UPP1*, *USP13*. It is interesting to see *NRAS* on this list, which is a potential melanoma drug candidate but has proved remarkably resistant to drug development efforts to date (66). *CD163* expression on melanoma-infiltrating macrophages has been suggested as a prognostic marker in melanoma (67).

Similarly, a list of putative melanoma tumor suppressor genes or melanoma oncogenes can be generated using a spreadsheet application from this melanoma data matrix (Data Sheet 2 in Supplementary Material). For example, a list consisting of genes that are mutated in more than 10% of melanoma metastases and have shorter melanoma-free patient survival associated with their low (putative tumor suppressor) or high (oncogene) RNA expression. Known tumor suppressors and oncogenes that were identified by this strategy (*NRAS*, *KIT*, and *WNT* family members) were removed. This list of putative melanoma tumor suppressors and oncogenes that remains is shown in Table 1.

**Table 1:** **Four putative melanoma oncogenes and two putative tumor suppressor genes derived from the amalgamated data**.

Entrez gene	Gene symbol	Gene title	Chromosomal location	Putative tumor suppressor or oncogene?
7373	*COL14A1*	Collagen, type XIV, alpha 1	8q23	Tumor suppressor
387357	*THEMIS*	Thymocyte selection associated	6q22.33	Tumor suppressor
6299	*SALL1*	Sal-like 1 (*Drosophila*)	16q12.1	Oncogene
5069	*PAPPA*	Pregnancy-associated plasma protein A, pappalysin 1	9q33.2	Oncogene
26278	*SACS*	Spastic ataxia of Charlevoix-Saguenay (sacsin)	13q12	Oncogene
81832	*NETO1*	Neuropilin (NRP) and tolloid (TLL)-like 1	18q22.2	Oncogene

#### Combined melanoma information with gene set analysis

Combining this assembled melanoma information with statistical GSA can potentially provide additional insights. For example, with a spreadsheet application we could generate a list of 245 genes from Data Sheet 2 in Supplementary Material that have coding region mutations in more than 10% of melanoma metastases, and subject this list to gene set enrichment analysis in order to identify biological functions that may be commonly disrupted in melanoma. When submitted to the web tool GeneSetDB (a meta-database of biologically relevant sets of genes) for enrichment analysis (with false discovery rate set to 0.01), this list of 245 genes was found to be significantly enriched for several gene sets including sets associated with the extracellular matrix (ECM), cell adhesion, and collagen fibril organization. We encourage users to use a spreadsheet application and simple web tools such as GeneSetDB to perform their own exploration of Data Sheet 2 in Supplementary Material.

### Assembly of MelanomaDB – a web-accessible genomic melanoma SQL database, and of a corresponding BaseSpace app

In order to make use of this assembly of melanoma information and its regular updating easier, we converted this melanoma data matrix (Data Sheet 2 in Supplementary Material) into a web-accessible SQL database. This database, named MelanomaDB, features melanoma gene sets derived from Data Sheet 2 in Supplementary Material and directly links into a molecular pathway/GSA meta-database previously generated by our research group named GeneSetDB (22). Using MelanomaDB, a user can easily find the union or intersection between any number of melanoma gene sets (taken from the columns of Data Sheet 2 in Supplementary Material) and also their own user-submitted gene lists (copied and pasted, or uploaded from a file, using any of over 50 types of commonly used gene identifier), then interrogate the molecular pathways for which the genes in these lists are enriched. Multiple iterations are possible, so that a user might find the union of some melanoma-associated gene sets and then find the intersection of this union with other gene sets, which can finally be directly piped into the gene set meta-database GeneSetDB to identify enriched molecular pathways. MelanomaDB is available at http://genesetdb.auckland.ac.nz/melanomadb/about.html

A subset of the information in MelanomaDB was also included in a freely available Illumina BaseSpace application, which can be accessed at http://www.biomatters.com/apps/melanoma-profiler-for-research (click on “sample project” and navigate using green tabs at top of screen). This BaseSpace application performs variant calling against reference sequences for a user-defined tumor, then uses information from MelanomaDB to identify molecular pathways that genes which contain non-synonymous variants constitute. These pathways are visualized relative to targeting drugs and other clinically related information using pathway diagrams, heatmaps, and waterfall plots, in comparison to the 310 melanomas described above. We hope that this app may be of particular use to researchers involved in generating new melanoma tumor sequences.

### MelanomaDB facilitates assessment of functional relationships inherent in tumor somatic variants

The tumor gene sequence information included in MelanomaDB allows calculation of the proportion of melanomas that carry somatic variations in each gene/loci on a genome-wide scale. For example, by selecting gene sets using the MelanomaDB web tool, we identified those genes in which over 10% of the 96 sequenced metastatic melanomas currently in the database carried non-synonymous somatic variations. This list of 245 genes included genes that have been the focus of recent publications describing mutations in melanoma, such as *PREX2* (6), *GRM3* (57), and *ERBB4* (56) [other melanoma-associated genes such as *MAP3K5/9* (58), *MAP2K1/2* (54), and *RAC1* (4–6) are included as mutated genes in human tumors in MelanomaDB but fall outside this list of 245 genes]. As would be expected, this composite list featured genes also indicated as frequently mutated in melanoma by the larger sequencing studies (4, 5) that were used in its construction, for example, half of the genes identified by Berger et al. (6) as “significantly mutated” appear on our composite list. By selecting the option in MelanomaDB to pipe these 245 genes to the GeneSetDB web tool, we identified that these genes were significantly enriched for a small group of biological functions including cell adhesion, collagen fibril organization, and ECM. Cell adhesion is briefly mentioned in some of the sequencing studies’ discussions (4, 54), and the ECM is a focus for one study (55). However, other pathways emphasized by these sequencing studies, such as the glutamate pathway (60) or chromatin remodeling pathways (5), did not feature in the results of our analysis.

### Analysis of specific signaling pathways relevant to melanoma

**Figure 4:**
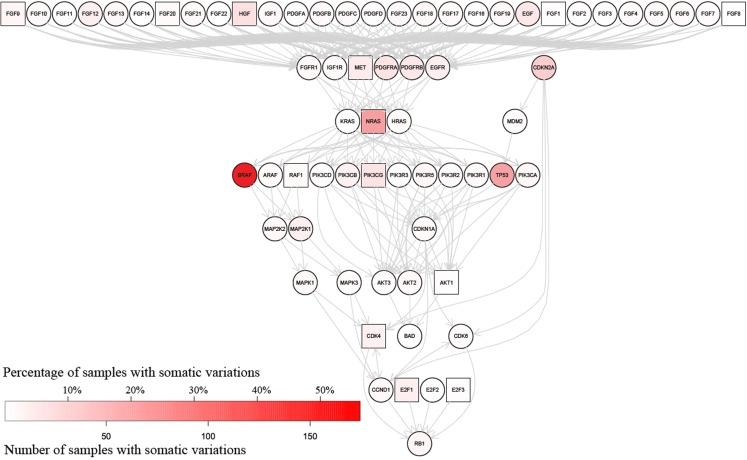
**Somatic variations in genes encoding proteins of the KEGG “Melanoma” signaling pathway**. The color of each gene’s node indicates the number of melanomas in which at least one non-synonymous somatic variation has been identified; white indicates no melanomas with reported somatic variation in the gene, while the degree of red saturation indicates the number of melanomas containing somatic variations in that gene (refer to color key in lower left). Square nodes indicate RNA expression in melanoma metastases significantly associated [*p* ≤ 0.05 no multiple testing correction applied, Cox proportion hazards model, Bogunovic et al. (50) data], with patient disease-free survival, while circular nodes indicate the absence of any significant association between RNA abundance and patient survival. This graph was generated using the pathwayGraph function to access the KEGG pathway information contained within the R graphite package.

The information in MelanomaDB can be used to annotate the signalling pathways contained within the R graphite package (27). This can be done either as a function of the MelanomaDB web tool, or using R scripts supplied in Data Sheet 5 in Supplementary Material. For example, Figure 4 shows the KEGG pathway named “Melanoma” with nodes colored in shades of red according to the frequency of non-synonymous somatic variations. Thirteen nodes were plotted as boxes rather than circles to indicate that the abundance of their encoded mRNA in melanoma metastases was significantly associated with patient survival in our analysis of the data of Bogunovic et al. (50) (Cox proportional hazards model, *p* ≤ 0.05, no multiple testing correction applied). Significantly more of the genes in the KEGG pathway named “Melanoma” carried more somatic variants than expected due to chance alone (Fisher’s exact test with right-tailed hyper-geometric distribution, *p* ≤ 0.002), in agreement with the known importance of the signaling events represented in this pathway to melanoma formation and progression.

### Analysis of melanoma signaling pathways in individual tumors

**Figure 5:**
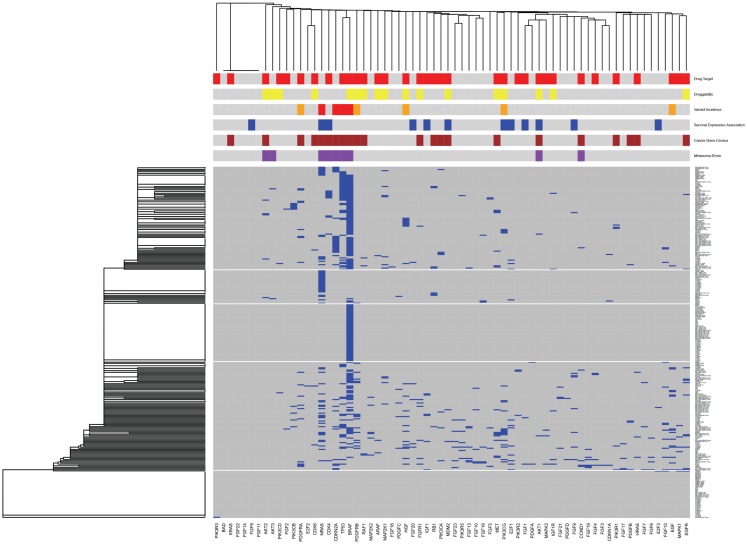
**Clustered heatmap for genes encoding proteins of the KEGG “Melanoma” signaling pathway**. Gene names are on the horizontal axis, individual melanoma tumor names are on the vertical axis. Blue blocks at the intersection of a gene and a tumor indicates the presence of a protein-altering somatic variant in that gene in that tumor. Clustering of genes and tumors using single linkage clustering with binary distance was performed based on this variant information. The clustered figure was then annotated with additional information above the heatmap. In the first row above the heatmap red blocks mark genes encoding known drug targets according to version 3 of the DrugBank database. In the second row yellow blocks mark genes encoding potentially druggable proteins, as indicated by the MelanomaDB gene set “Druggability: Sophic ENSEMBL list” (33). In the third orange and red blocks indicate genes mutated in ≥1 or ≥5% of the 310 melanomas in our database, respectively. In the fourth row blue blocks mark genes that encode RNAs with a significant association between expression and patient survival [*p* ≤ 0.05 no multiple testing correction applied, Cox proportion hazards model, Bogunovic et al. (50) data]. In the fifth row brown blocks indicate genes that are members of the Wellcome Trust Cosmic “Cancer Gene Census” gene set, as on 1st March 2013 (http://cancer.sanger.ac.uk/cancergenome/projects/census/). In the sixth row, purple blocks mark genes thought to be melanoma drivers when mutated [MelanomaDB gene set “Melanomagenesis Drivers” (84)]. This graph was generated using a modification of the heatmap.2 function of the gplots package in R.

As an example of how this pathway-specific information can be used to place the tumors of individual patients into the context of tumors from the patient population, as well as into the context of other information within MelanomaDB, we used the information assembled here to draw a clustered heat map for genes encoding molecules of the KEGG “Melanoma” signaling pathway (Figure 5). This clustered heatmap is annotated with gene-survival associations, druggability indices, current drug targets, COSMIC census genes, known melanoma driver mutations and somatic variant frequency in melanoma. This can be done either as a function of the MelanomaDB web tool, or using R scripts supplied in Data Sheet 5 in Supplementary Material. In this analysis, somatic variants in genes drive the tumor clustering and potentially stratify patients into those with common biological changes, which may be susceptible to particular pathway-targeted therapies. For instance, there is a cluster of tumors with *BRAF* as the only somatic variant in this pathway (middle horizontal block in Figure 5). Of these 51 *BRAF*-variant only melanomas, 42 carry the *BRAF* V600E mutation and may putatively be tumors for stratification to Vemurafenib therapy, given their lack of somatic variants in genes encoding other proteins in this signaling pathway that could potentially contribute to Vemurafenib resistance. Some tumors carry only *NRAS* mutations, while others have either more complex mutational patterns, or no somatic mutations in this pathway. This is in accordance with previous studies reporting that mutations in *NRAS* and *BRAF* tend to be mutually exclusive but collectively occur in approximately 90% of melanomas (68). To assist interpretation of the different mutations seen in each tumor and in clusters of genetically similar tumors, the heatmap has been annotated with information about inferred melanoma driver mutations, known drug targets, and potentially druggable proteins. This type of heat map can be generated for any molecular pathway or combination of pathways. Extending this analysis, a new patient’s mutation profile could be added to an established clustering analysis of large numbers of melanomas in order to identify which previously studied tumors were similar in mutation complement, which may assist prognostication and treatment stratification. In the future it will be interesting to use MelanomaDB to investigate the genomes of multiple samples from single melanomas to assess the intra-tumoral heterogeneity seen in this disease (69).

**Figure 6:**
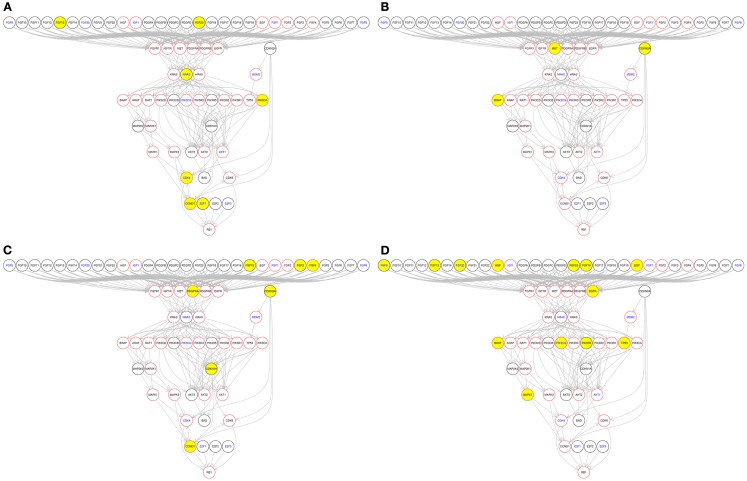
**Somatic variations in individual tumors of molecules in the KEGG “Melanoma” signaling pathway**. Yellow nodes indicate that the gene has a somatic variation in that particular tumor. Red node borders indicate that there is a drug available to target that gene’s encoded protein. Blue node text indicates that this gene’s RNA abundance is associated with patient survival in metastatic melanoma, in the data of Bogunovic et al. (50). Four individual tumors with different mutation profiles are shown as examples: **(A)** ME049 from Berger et al. (6); **(B)** 01T from Wei et al. (60); **(C)** melanoma reported by Turajlic et al. (59); **(D)** YUKLAB from Krauthammer et al. (5). This graph was generated using the pathwayGraph function and KEGG information contained within the R graphite package.

In addition, using a function in the MelanomaDB web tool of the R scripts supplied in Data Sheet 5 in Supplementary Material, somatic alteration of genes in specific molecular pathways can be drawn on a patient-by-patient basis (Figure 6). This allows visualization of protein-altering gene sequence variants in the context of the encoded protein’s position in molecular pathways relevant to specific targeted therapies. For instance, using a well-known example from other tumor types, the position in pathway diagrams of a genetic variant known to be activating (e.g., mutant *KRAS*), downstream of a drug (e.g., cetuximab) target (e.g., EGFR) may indicate potential for resistance to the drug.

**Figure 7:**
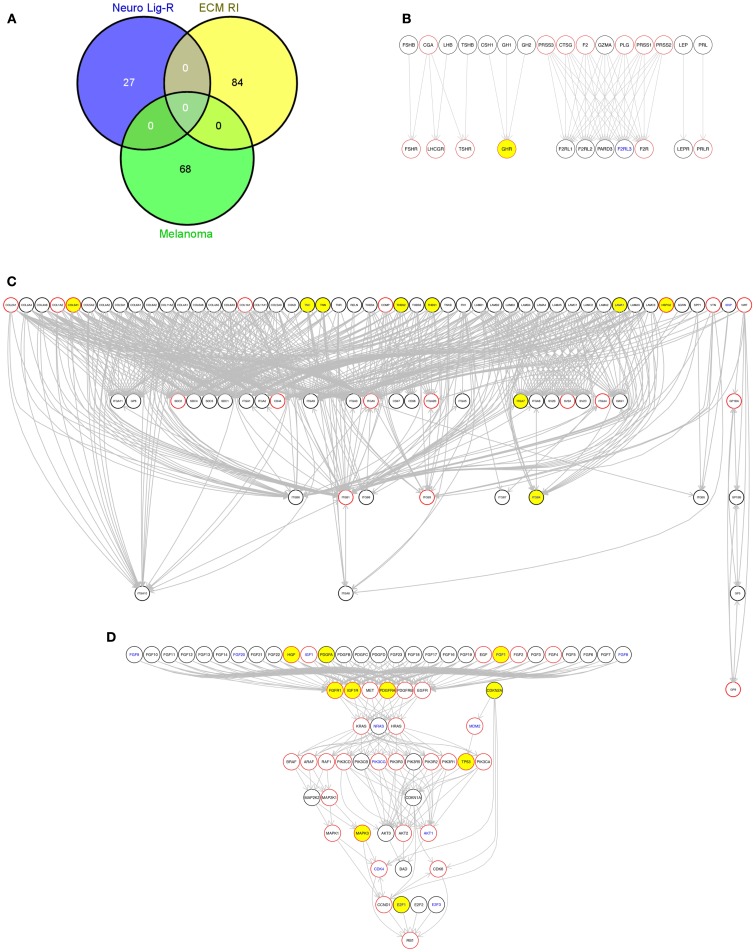
**(A)** Venn diagram showing the overlap of genes between these three pathways used in this figure, generated using the Venny web tool (http://bioinfogp.cnb.csic.es/tools/venny/index.html). “Melanoma,” “Neuro Lig-RI,” and “ECM RI” indicate members of the “Melanoma,” “Neuroactive ligand-receptor interaction,” and “Extracellular matrix (ECM) receptor interaction” KEGG pathways, respectively, contained in the R graphite package; **(B)** The KEGG “Neuroactive ligand-receptor interaction” pathway; **(C)** The KEGG “Extracellular matrix (ECM) receptor interaction pathway”; **(D)** The KEGG “Melanoma” pathway. Yellow fill color in nodes indicate genes with protein-altering somatic variations in this sample. Nodes with red borders represent genes that encode targets of existing drugs according to version 3 of the DrugBank database. Nodes with blue text indicate genes that encode RNAs with a significant association between expression and patient survival [*p* ≤ 0.05 no multiple testing correction applied, Cox proportion hazards model, Bogunovic et al. (50) data, see Materials and Methods]. This graph was generated using the pathwayGraph function and KEGG information contained within the R graphite package. Similar graphs can also be generated using the MelanomaDB web tool.

We then used an R script (Data Sheet 5 in Supplementary Material) to perform gene set enrichment analysis using the GATHER web tool^10^(19) to identify any KEGG pathways for which genes somatically altered in each tumor were significantly enriched (Data Sheet 4 in Supplementary Material). KEGG pathways that appeared as significantly enriched in individual tumors included the “ECM receptor interaction” and “Neuroactive ligand-receptor interaction” KEGG pathways. To illustrate this, we selected one sequenced metastatic melanoma, ME029 from the Berger et al. (6) cohort, and drew these two pathways along with the KEGG “Melamoma” pathway for this single tumor (Figure 7). Two of these pathways are drawn for all 310 tumors included in this study in: Presentation 1 (“Melanoma”) and Presentation 2 (“Neuroactive ligand-receptor interaction”).

### Limitations of our approach

The approach we have described, while already functioning in a useful way as a melanoma-focused integrated genomic database, provides a template for further development to address the limitations below: (i) It will be important to identify the likely effects of specific somatic variations in the sequenced tumors (e.g., loss of function, altered function, or activation of the encoded protein). In future iterations of MelanomaDB, based on larger numbers of tumors, we will include capacity to dissect the type of genetic alteration such as deletions, coding region mutations, promoter mutations, etc. The database may also be expanded to include the results of analyses from software that predict the effects of coding variants on protein function, such as SIFT (70), PolyPhen (71), or PROVEAN (72), as well as the known effects of specific mutations using resources such as COSMIC (8). (ii) Data on naevi and synonymous mutations can also be added. (iii) Information from model organisms such as mouse could also be added. (iv) Results from the ENCODE project (73) could be added along with whole genome sequencing of melanomas will allow inclusion of numerous additional functional genetic loci [e.g., ncRNAs, both general (74) and melanoma specific (75)] in the database. The ENCODE project suggests that mutations in regulatory regions such as distal enhancers can affect the expression of genes located hundreds of kilobases away (76); a way to include this in MelanomaDB could be to take a gene network approach to identify distant genes that have expression correlated with these mutations, as well as methods such as chromatin conformation capture (77). (v) Future additions to the database will also aim to incorporate data concerning the role of epigenetics, including methylation, in melanoma (78–80). (vi) There is also room to expand upon melanoma drivers, such as those highlighted in GISTIC (81), JISTIC (82), and CONEXIC (83). (vii) There is an inherent risk in any assembly or meta-analysis of data from several sources that errors in the original data are perpetuated. While it is possible that the intersection of multiple independent sources of similar types of information may reduce the change of propagating random errors, systematic errors co-occur in independent data sources. This risk affects any project of this sort and is difficult to control. Here we have attempted to minimize this risk by selecting constituent databases that are extensively used and have been peer reviewed, and on which we could perform spot checks. We consider these data sources to be the best possible choices, within our ability to assess them. (viii) The final limitation is that the molecular pathways used when assembling this database are limited by current knowledge, and overlap with one another. The database will be updated with new pathway information as it becomes available. Identifying the pathways that are not affected can be as useful as identifying those that are. The data we have generated using literature relationships with the IRREDESCENT and GAMMA methods has not been experimentally verified and is intended primarily for hypothesis generating.

## Conclusion

We have brought together a large collection of melanoma genomic data of several types from published studies and publicly available datasets into an easily utilized data matrix that can be analyzed using a spread sheet application. We also assembled data on tumors from individual patients. We then incorporated this information into a web-accessible SQL database, MelanomaDB, which researchers can use to perform molecular pathway and GSA of melanoma genomic data, and into a BaseSpace application. By way of illustration, we used this information to analyze the mutational and expression patterns of genes encoding proteins in specific directional signaling pathways within individual tumors, and annotated these visualizations with information about existing drugs, druggability, associations between RNA expression and survival, and driver mutations. We hope that this resource will prove increasingly useful when it expands as new tumor data becomes available. In particular, we hope it may provide a context in which to interpret the melanoma molecular profiles of new patients as well as patient-specific molecular pathway disruption. We have demonstrated possible uses of this integrated information, and encourage melanoma researchers to employ these resources.

## Conflict of Interest Statement

Steven Stones-Havas is an employee, and Cristin G. Print a paid consultant, of the company Biomatters Ltd., which works in the general field of genomic visualization. Biomatters Ltd., generated the freely available BaseSpace application described in this manuscript in a non-commercial collaboration with the other authors.

## Supplementary Material

The Supplementary Material for this article can be found online at http://www.frontiersin.org/Cancer_Genetics/10.3389/fonc.2013.00184/abstract

## Footnotes

^1^http://www.melanoma.org.au/research/melanoma-genome-project.html

^2^http://cancergenome.nih.gov

^3^http://ingenuity.com/products/pathways_analysis.html

^4^http://www.drugbank.ca/drugs/DB00002

^5^http://www.affymetrix.com/support/technical/annotationfilesmain.affx

^6^http://www.ncbi.nlm.nih.gov/gene, accessed on 30/7/12

^7^ftp://ftp.ncbi.nih.gov/gene/DATA/

^8^http://cran.r-project.org/web/packages/gplots/index.html

^9^http://www.bioconductor.org/packages/release/bioc/html/graphite.html

^10^http://gather.genome.duke.edu
