# Supplementary material for: MelanomaDB: A Web Tool for Integrative Analysis of Melanoma Genomic Information to Identify Disease-Associated Molecular Pathways
Source: Front Oncol. 2013 Jul 16;3:184. doi: 10.3389/fonc.2013.00184 (PMC3712543; doi:10.3389/fonc.2013.00184)

The two pages that follows are: (i) a clustered heatmap and (ii) a reverse waterfall plot for genes encoding proteins of the KEGG "Melanoma" signalling pathway. Gene names are on the horizontal axis, individual melanoma tumour names are on the vertical axis. Blue blocks at the intersection of a gene and a tumour indicates the presence of a protein-altering somatic variant in that gene in that tumour. Clustering of genes and tumours using single linkage clustering with binary distance was performed based on this variant information. The clustered figure was then annotated with additional information above the heatmap. in the first row above the heatmap red blocks mark genes encoding known drug targets according to version 3 of the DrugBank database. In the second row yellow blocks mark genes encoding potentially druggable proteins, as indicated by the MelanomaDB gene set "Druggability: Sophic ENSEMBL list"[31]. In the third orange and red blocks indicate genes mutated in ≥1% or ≥ 5% of the 310 melanomas in our database, respectively. In the fourth row blue blocks mark genes that encode RNAs with a significant association between expression and patient survival ($p \leq 0.05$ no multiple testing correction applied, Cox proportion hazards model, Bogunovic et al data[48]). In the fifth row brown blocks indicate genes that are members of the Wellcome Trust Cosmic "Cancer Gene Census" gene set, as on 1st March 2013 (http://cancer.sanger.ac.uk/cancergenome/projects/census/). In the sixth row, purple blocks mark genes thought to be melanoma drivers when mutated (MelanomaDB gene set "Melanomagenesis Drivers" [76]).the "Melanoma" KEGG pathway in an individual tumour.

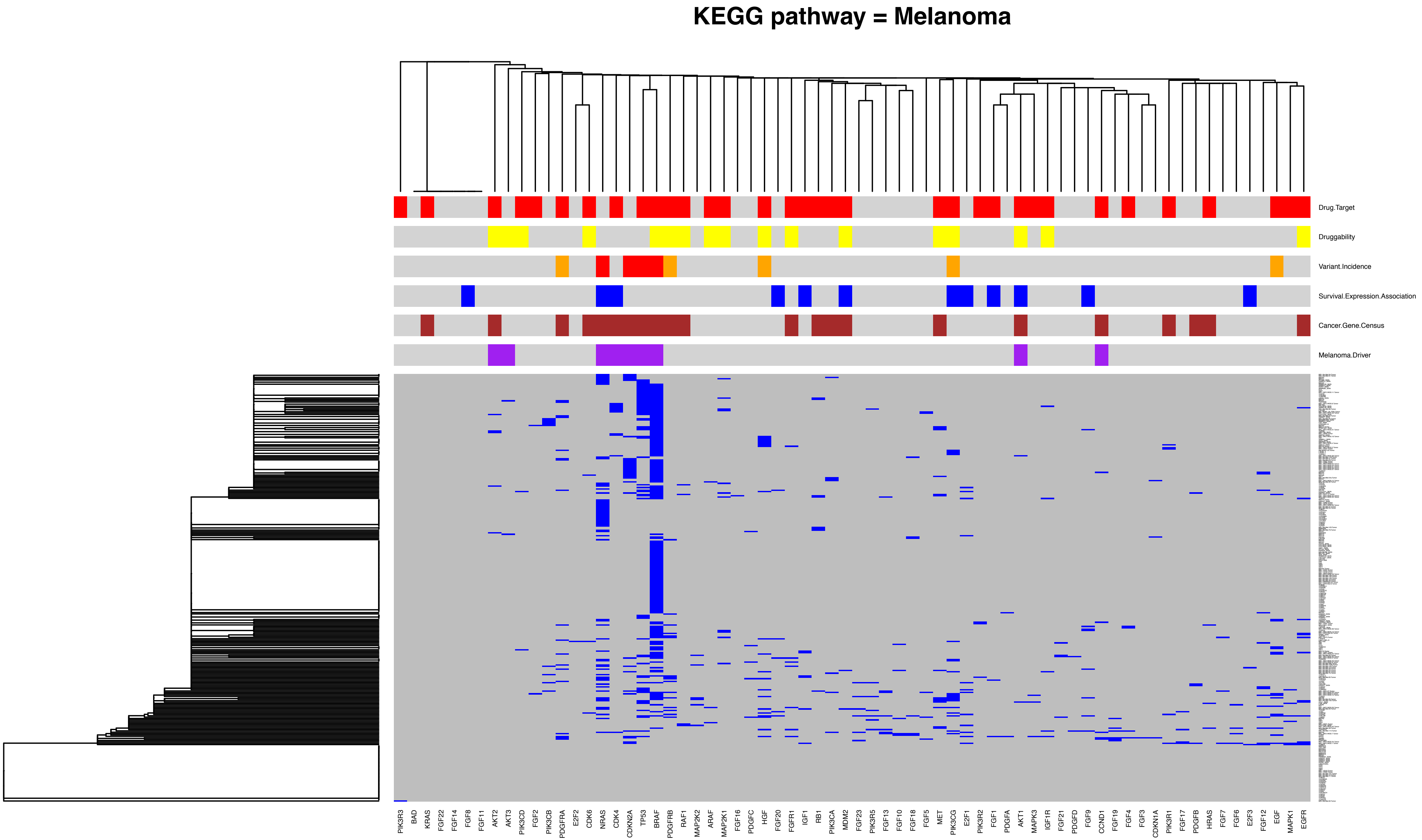

KEGG pathway = Melanoma
Drug.Target
Druggability
Variant.Incidence
Survival.Expression.Association
Cancer.Gene.Census
Melanoma.Driver
PIK3R3
BAD
KRAS
FGF22
FGF14
FGF8
FGF11
AKT2
AKT3
PIK3CD
FGF2
PIK3CB
PDGFRA
E2F2
CDK6
NRAS
CDK4
CDKN2A
TP53
BRAF
PDGFRB
RAF1
MAP2K2
ARAF
MAP2K1
FGF16
PDGFC
HGF
FGF20
FGFR1
IGF1
RB1
PIK3CA
MDM2
FGF23
PIK3R5
FGF13
FGF10
FGF18
FGF5
MET
PIK3CG
E2F1
PIK3R2
FGF1
PDGFA
AKT1
MAPK3
IGF1R
FGF21
PDGFD
FGF9
CCND1
FGF19
FGF4
FGF3
CDKN1A
PIK3R1
FGF17
PDGFB
HRAS
FGF7
FGF6
E2F3
FGF12
EGF
MAPK1
EGFR

KEGG pathway = Melanoma

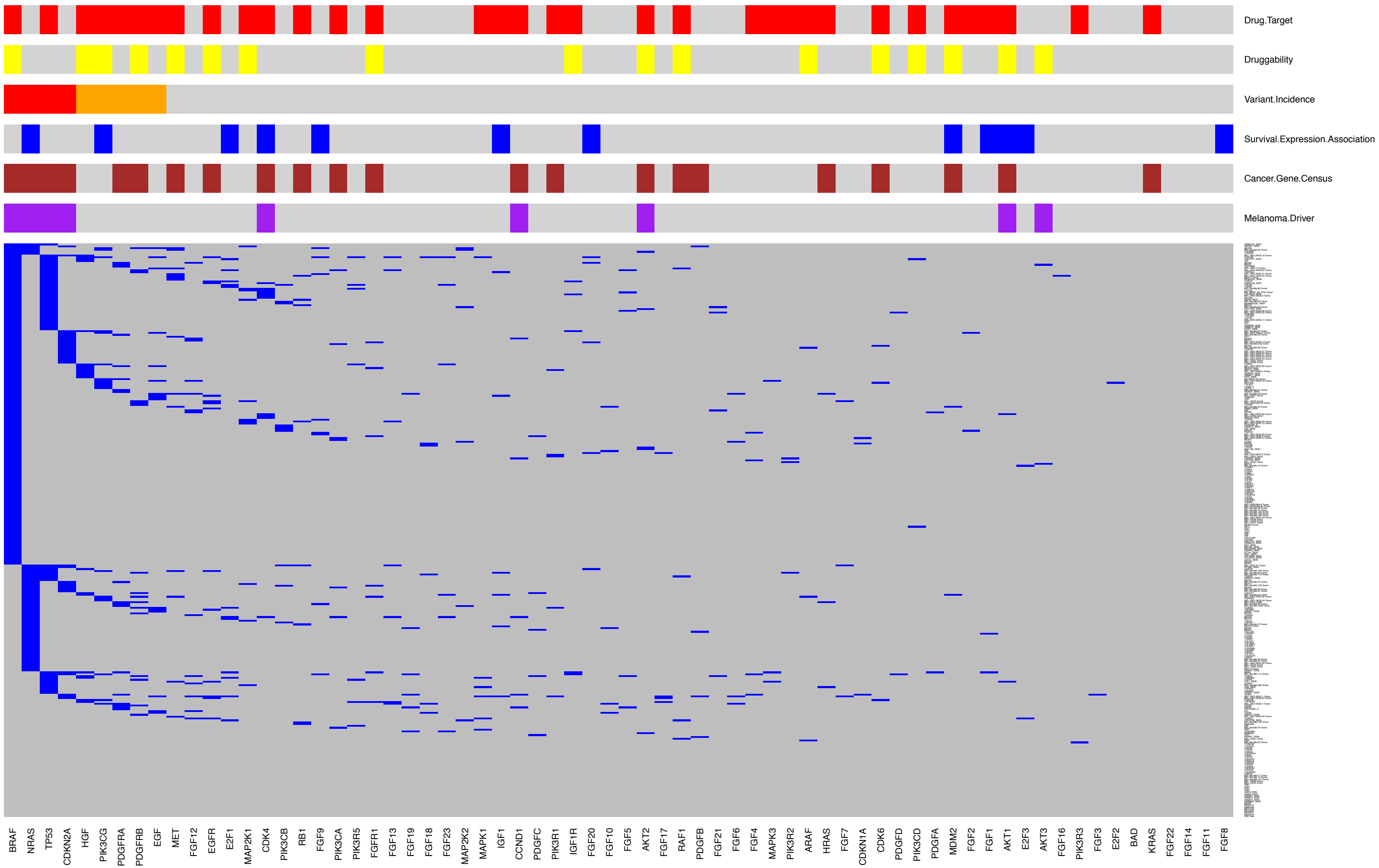

Each page that follows represents the “Melanoma” KEGG pathway in an individual tumour.

Yellow fill in nodes indicates that the gene has a somatic variation in that tumour.

Red border of nodes indicates that there is a drug available to target this gene’s encoded protein.

Blue text in nodes indicate genes that encode RNAs with a significant association between expression and patient survival ($p \leq 0.05$ no multiple testing correction applied, Cox proportion hazards model, Bogunovic et al data [48])

KEGG pathway = Melanoma : tumour = X501.Mel : Yellow Fill = gene variant, Blue Text = expression–survival association, Red Boarder = drug

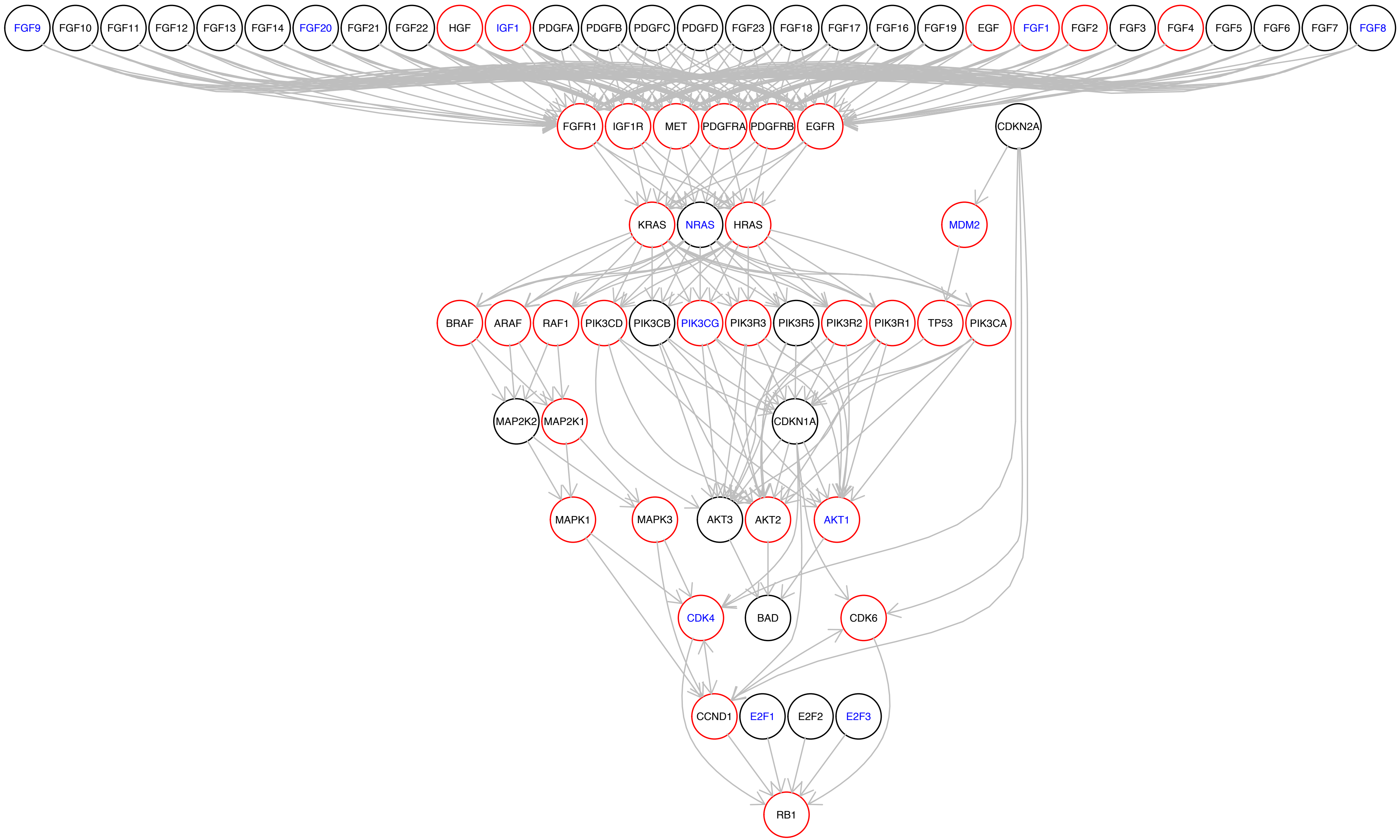

KEGG pathway = Melanoma : tumour = M000216 : Yellow Fill = gene variant, Blue Text = expression−survival association, Red Boarder = drug

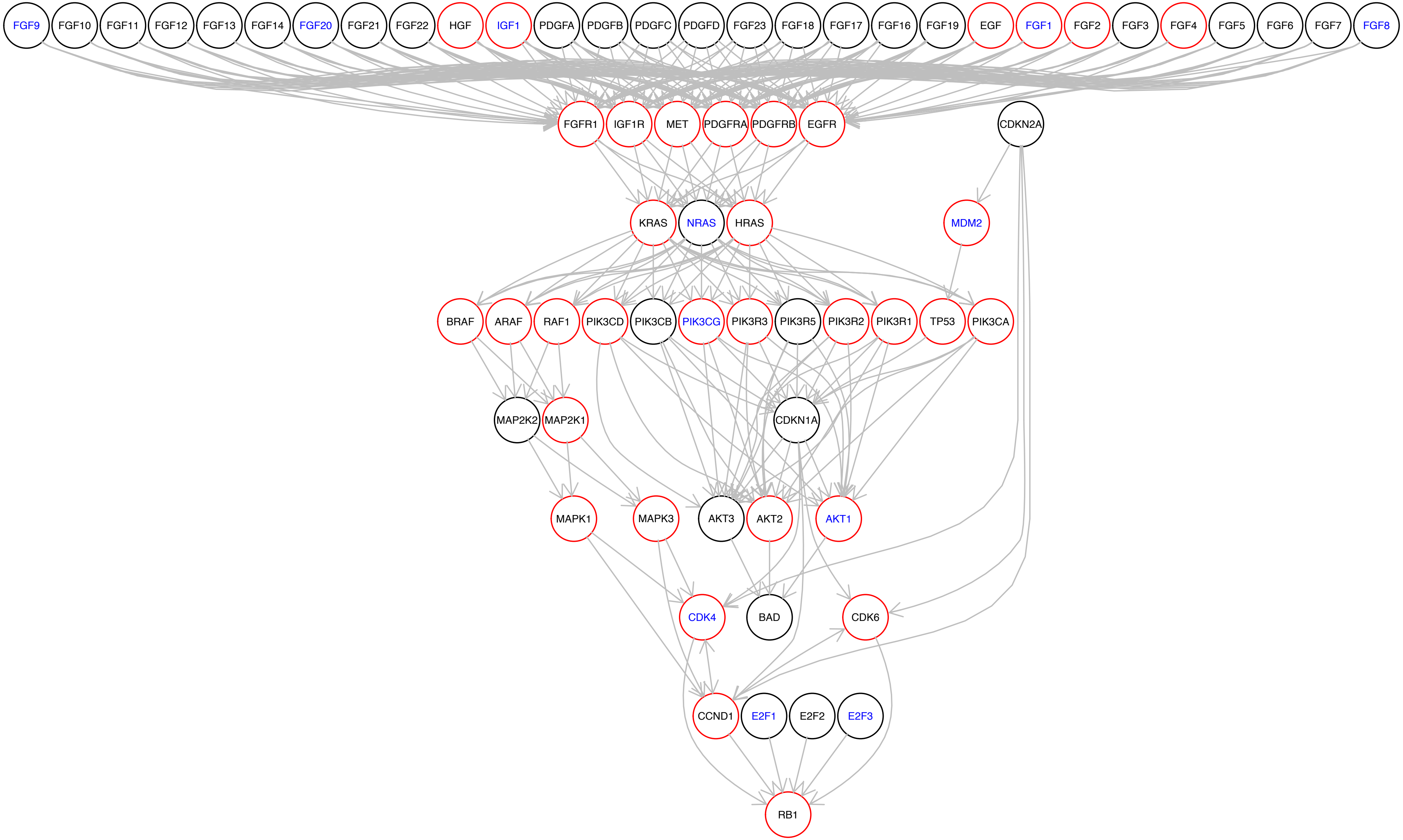

KEGG pathway = Melanoma :   tumour = M000921 :   Yellow Fill = gene variant, Blue Text = expression–survival association, Red Boarder = drug

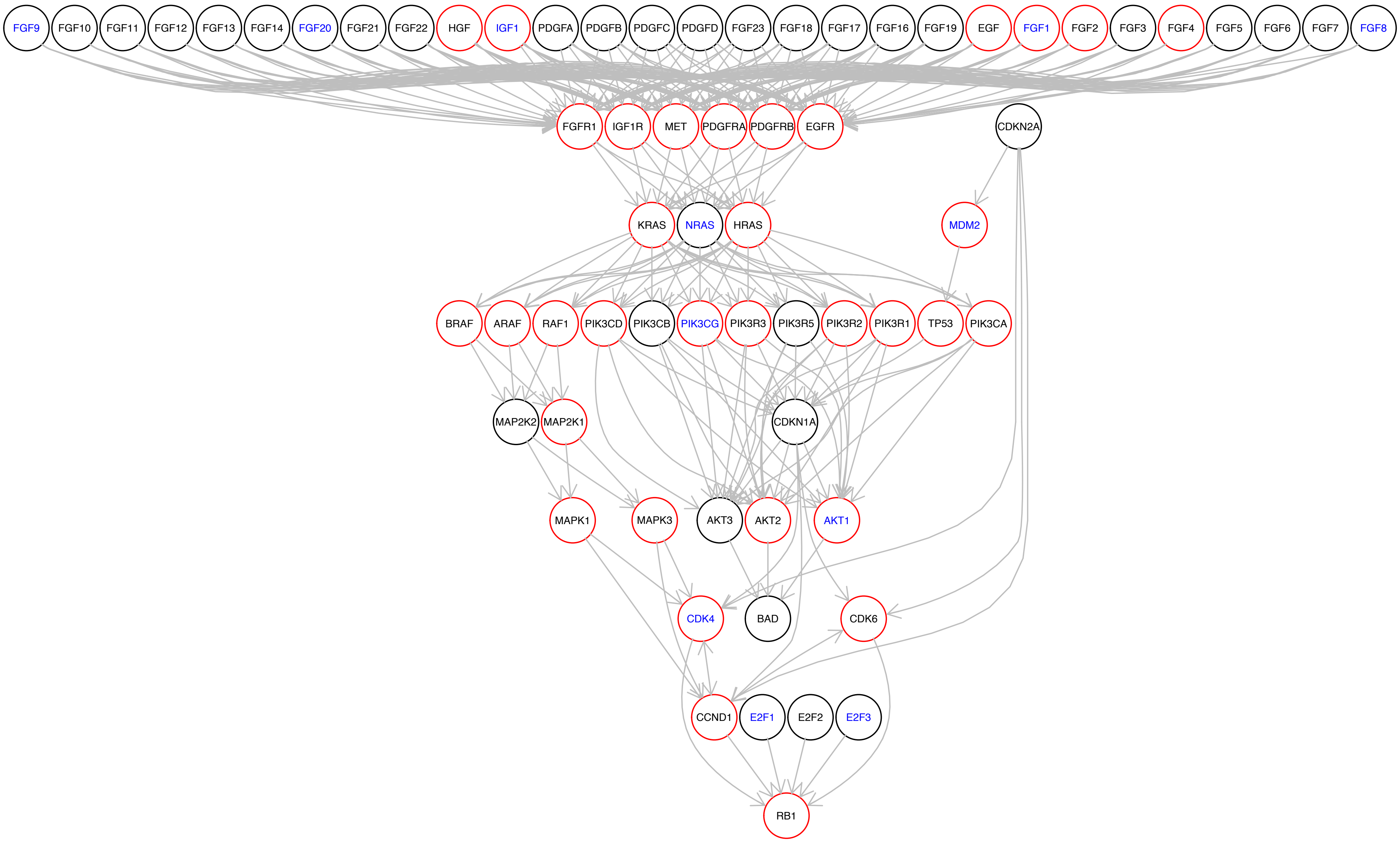

KEGG pathway = Melanoma :   tumour = M010403 :   Yellow Fill = gene variant, Blue Text = expression−survival association, Red Boarder = drug

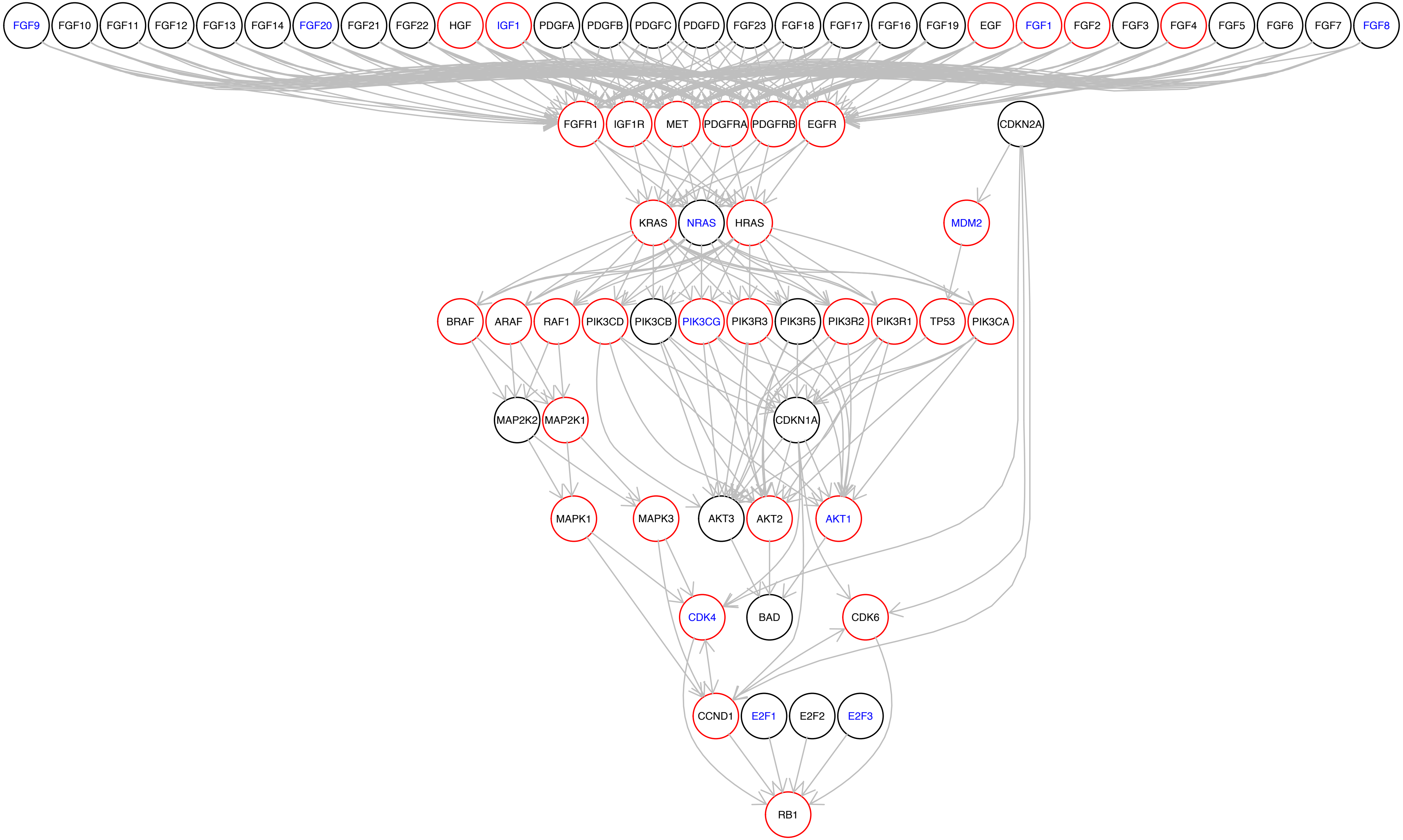

KEGG pathway = Melanoma :   tumour = M970109 :   Yellow Fill = gene variant, Blue Text = expression−survival association, Red Boarder = drug

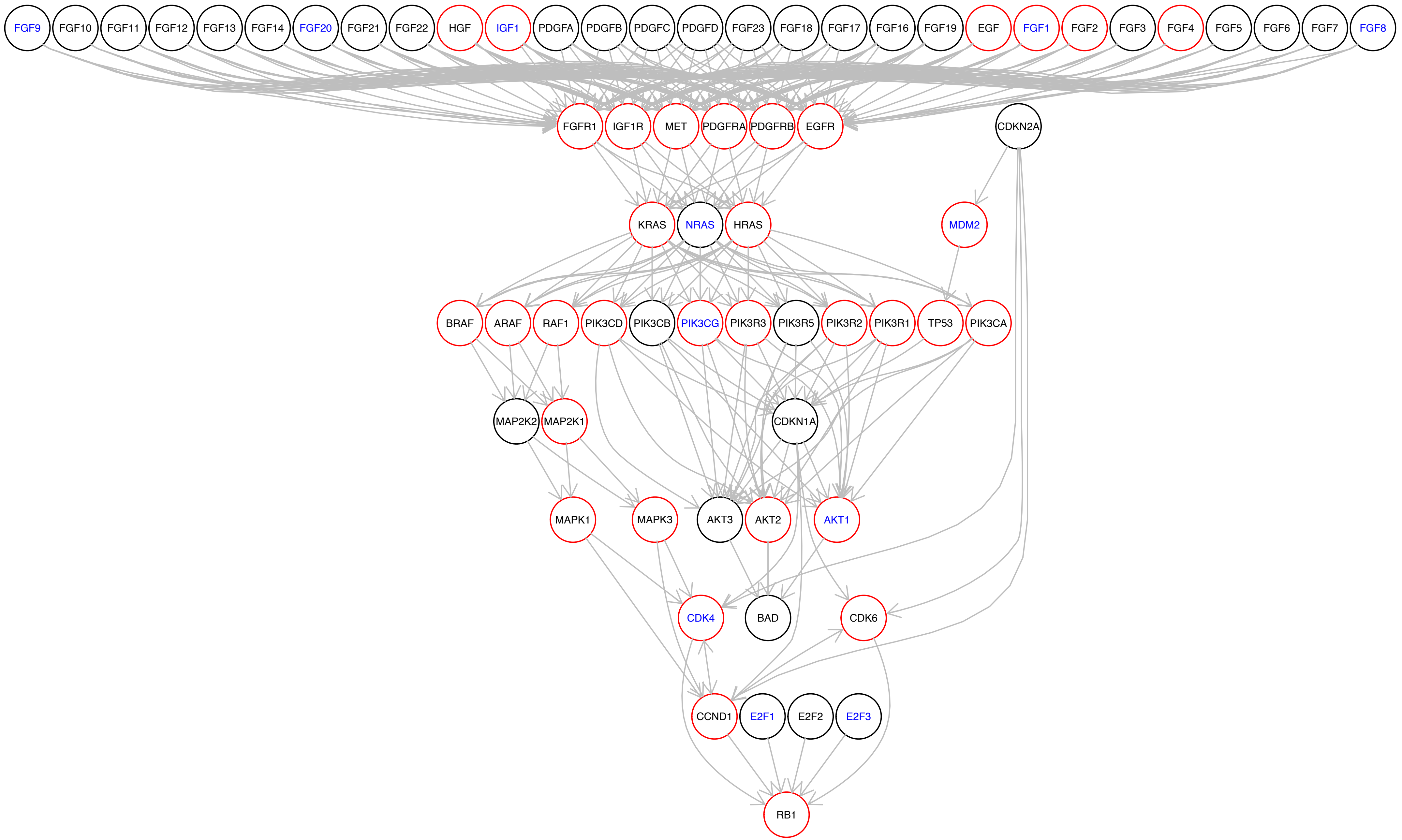

KEGG pathway = Melanoma : tumour = M980409 : Yellow Fill = gene variant, Blue Text = expression–survival association, Red Boarder = drug

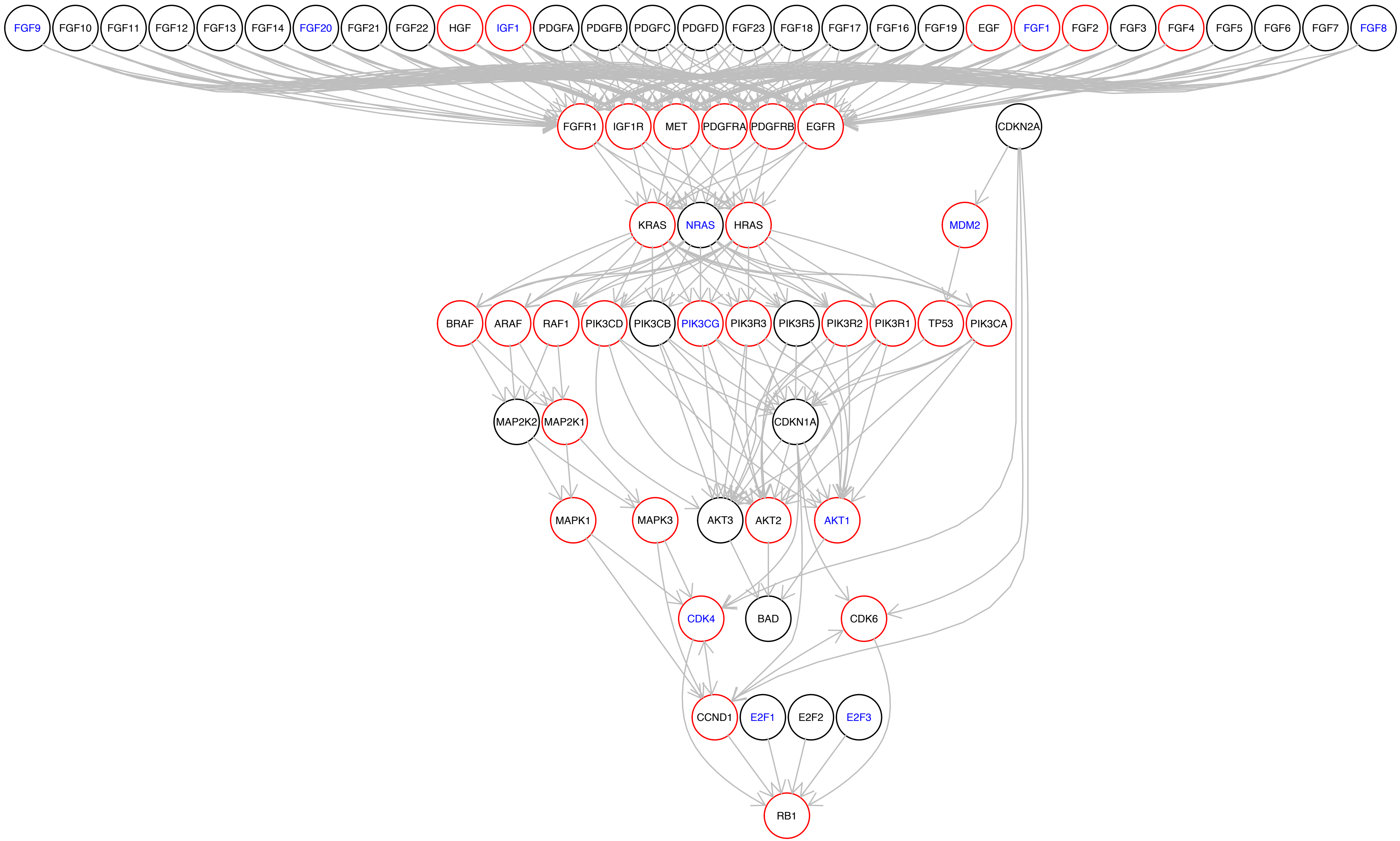

KEGG pathway = Melanoma : tumour = M980928 : Yellow Fill = gene variant, Blue Text = expression−survival association, Red Boarder = drug

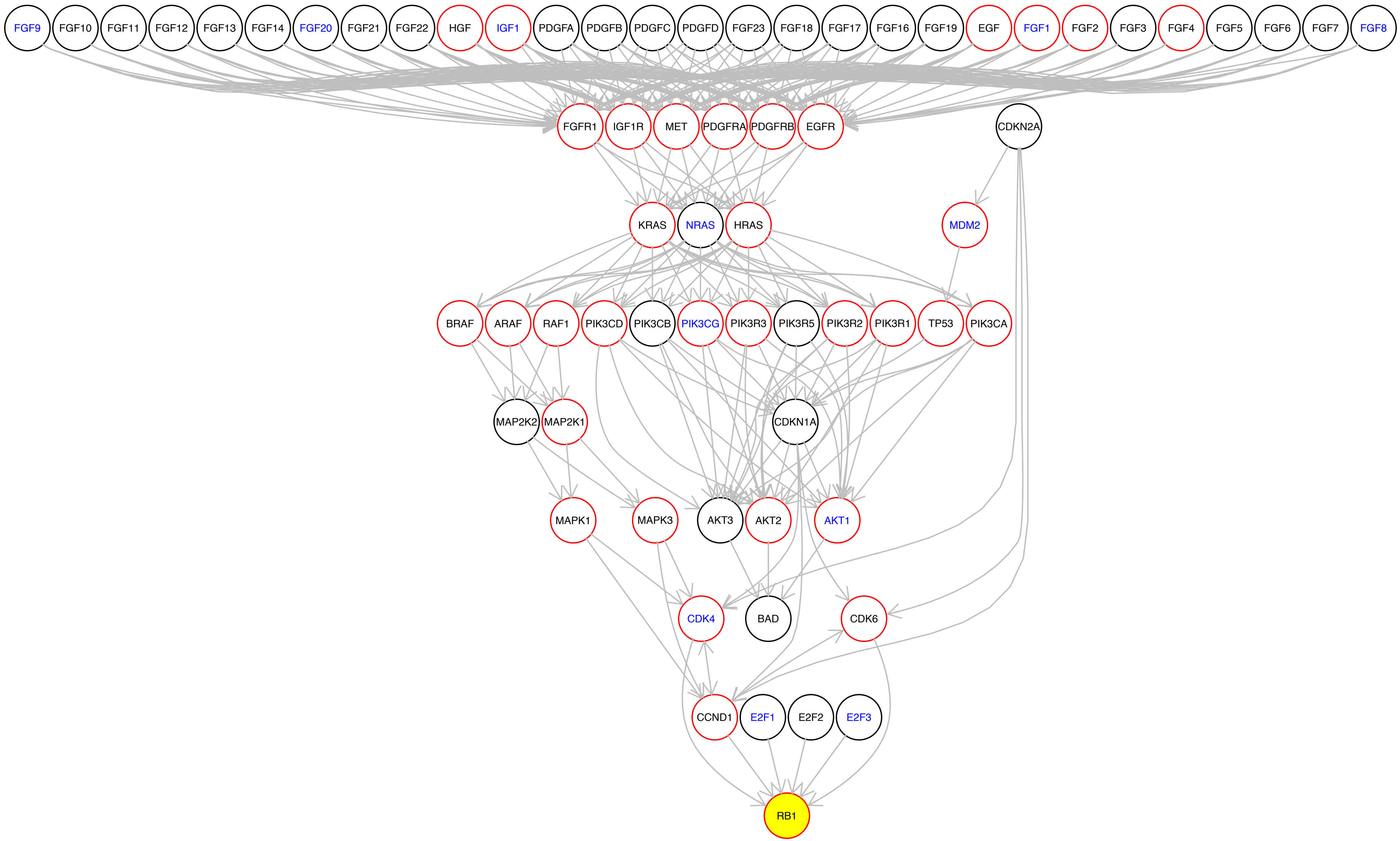

KEGG pathway = Melanoma : tumour = M990514 : Yellow Fill = gene variant, Blue Text = expression−survival association, Red Boarder = drug

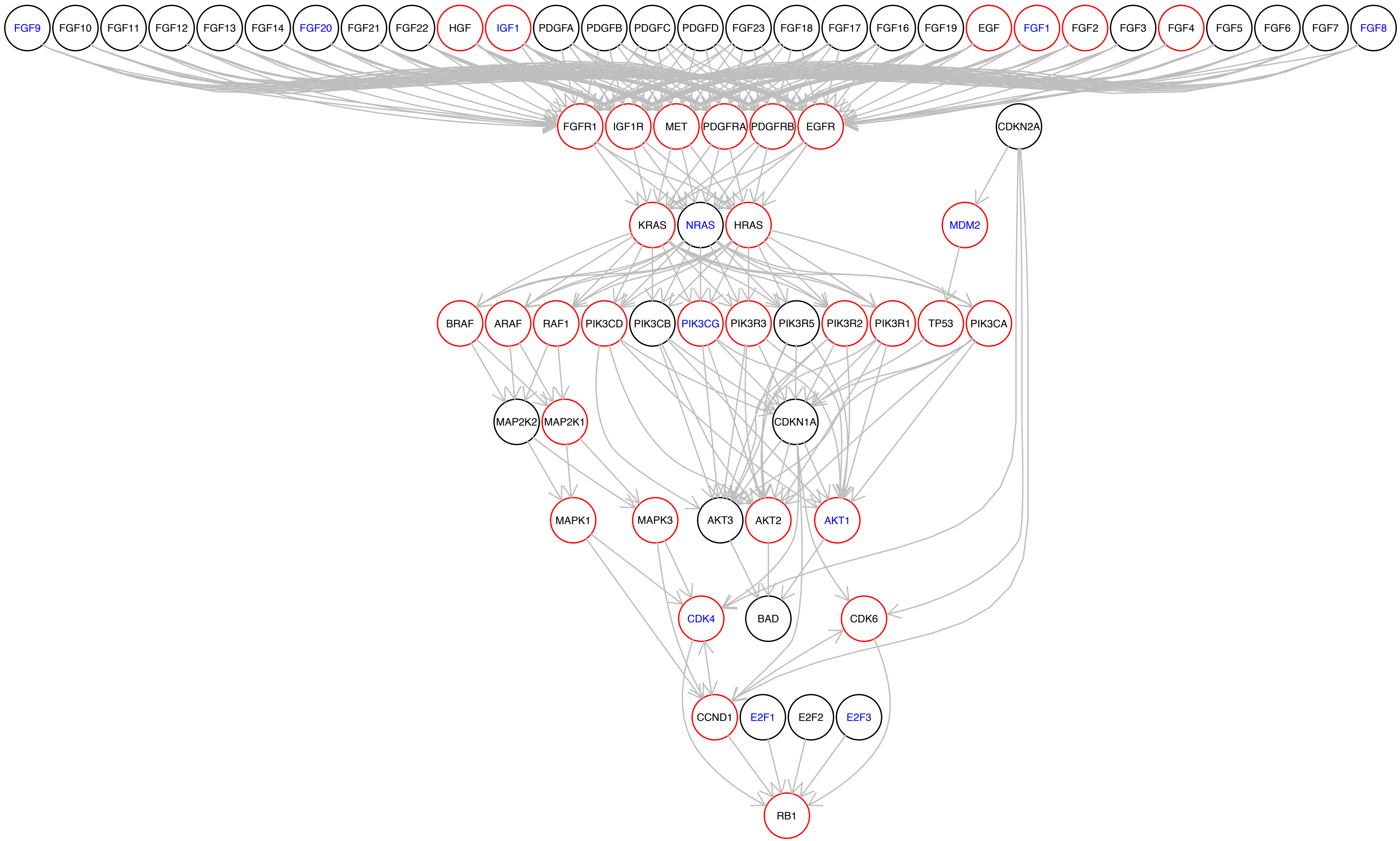

KEGG pathway = Melanoma :   tumour = M990802 :   Yellow Fill = gene variant, Blue Text = expression−survival association, Red Boarder = drug

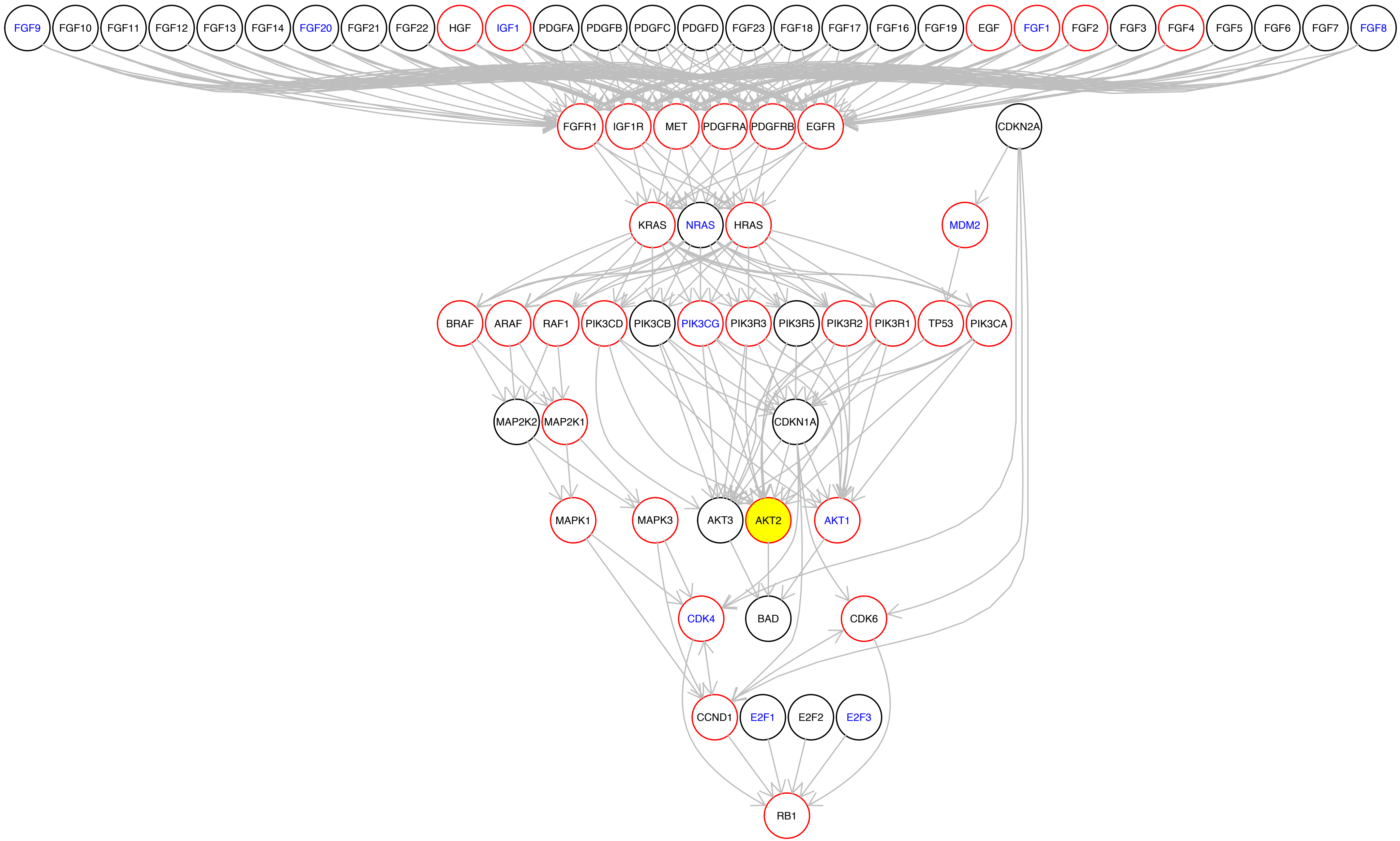

KEGG pathway = Melanoma :   tumour = MeWo :   Yellow Fill = gene variant, Blue Text = expression–survival association, Red Boarder = drug

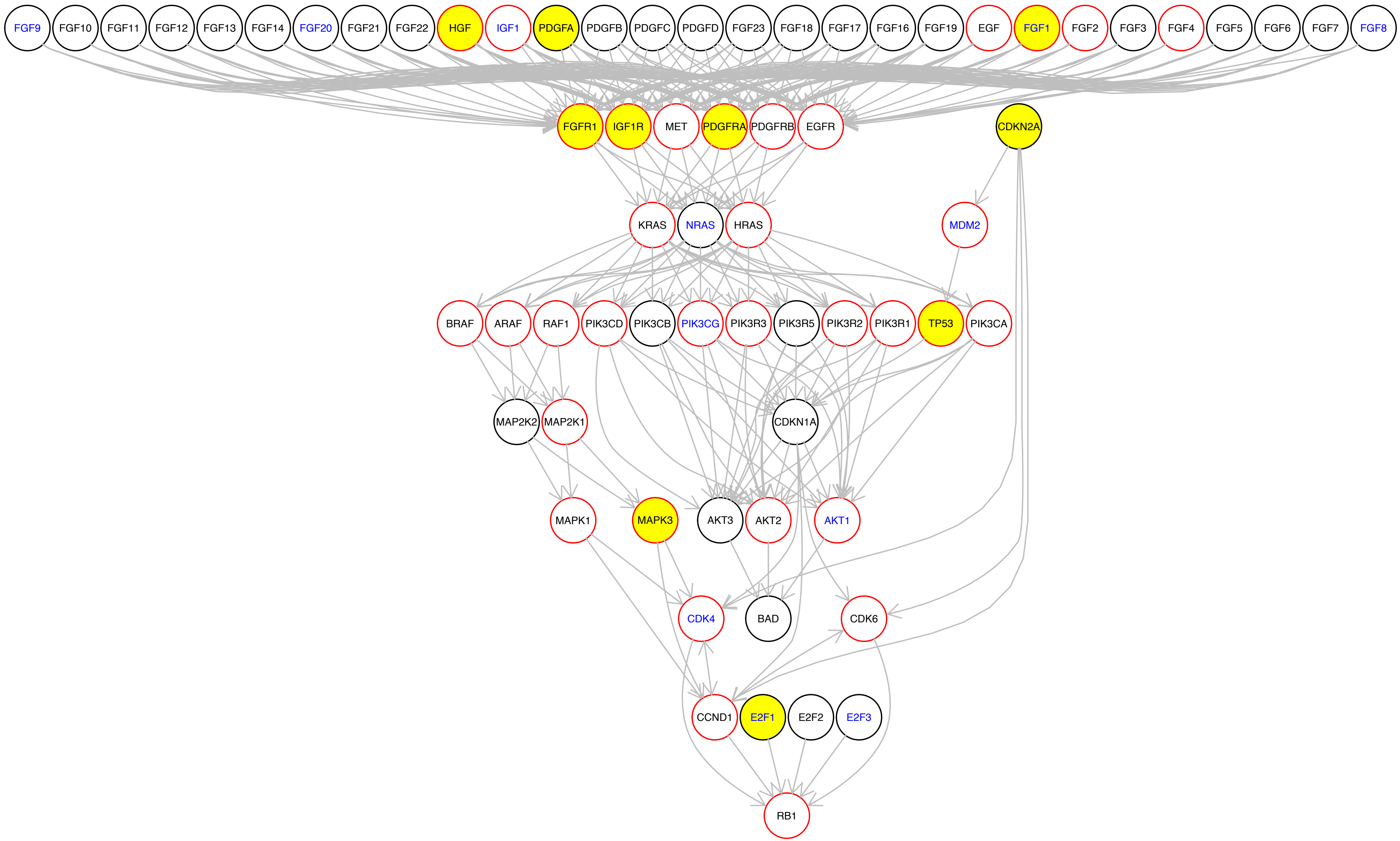

KEGG pathway = Melanoma : tumour = ME001 : Yellow Fill = gene variant, Blue Text = expression−survival association, Red Boarder = drug

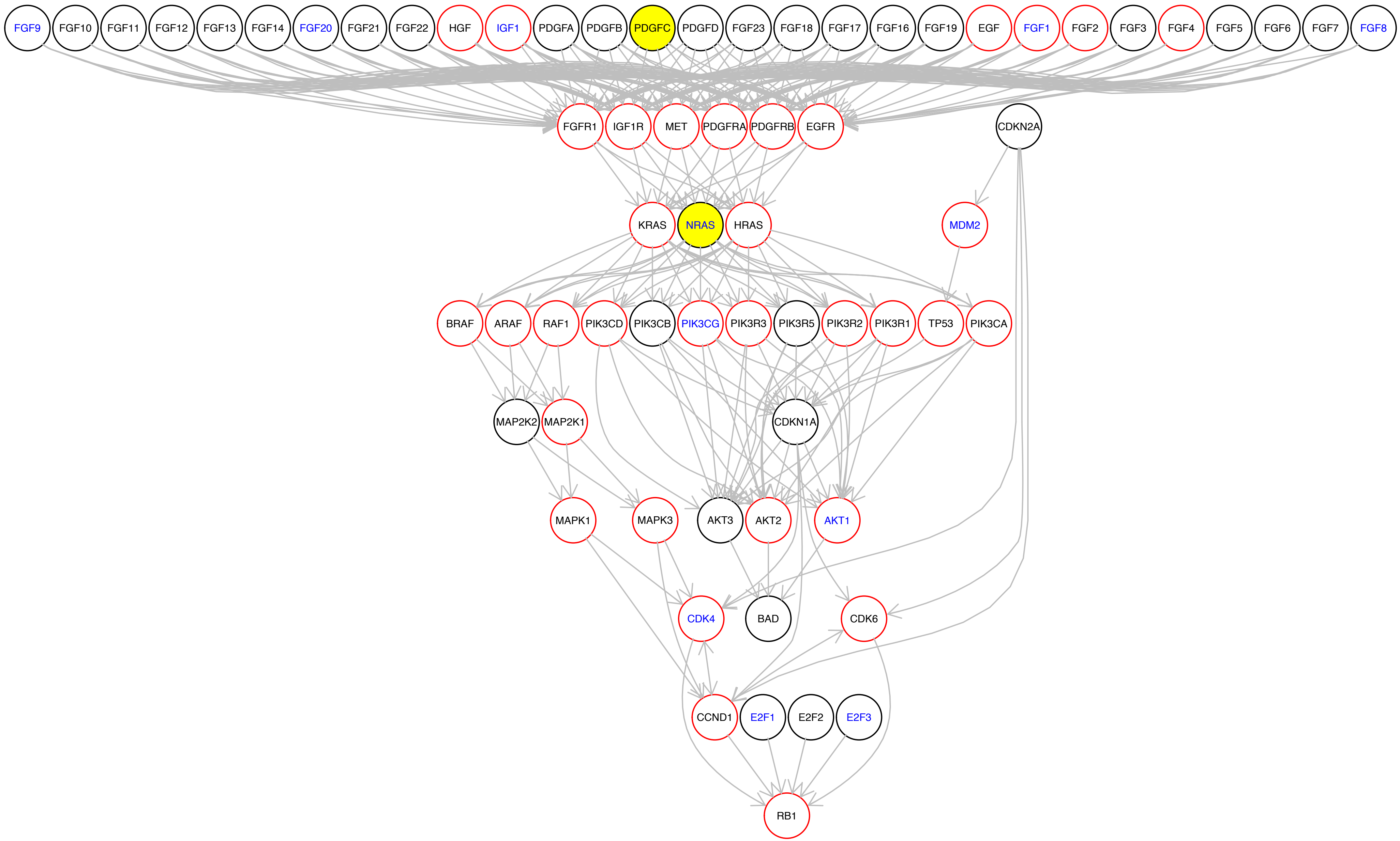

KEGG pathway = Melanoma :   tumour = ME002 :   Yellow Fill = gene variant, Blue Text = expression−survival association, Red Boarder = drug

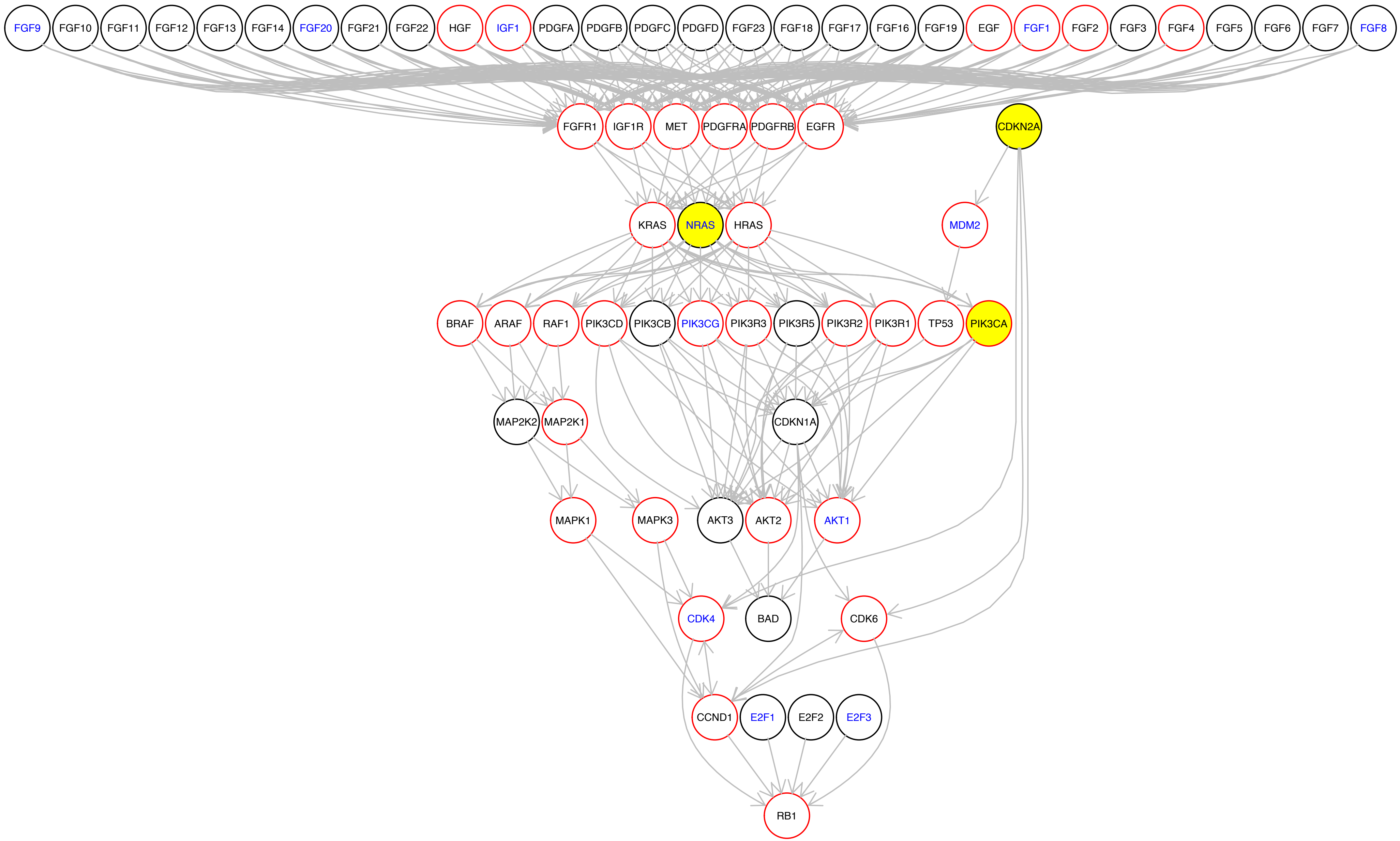

KEGG pathway = Melanoma : tumour = ME007 : Yellow Fill = gene variant, Blue Text = expression−survival association, Red Boarder = drug

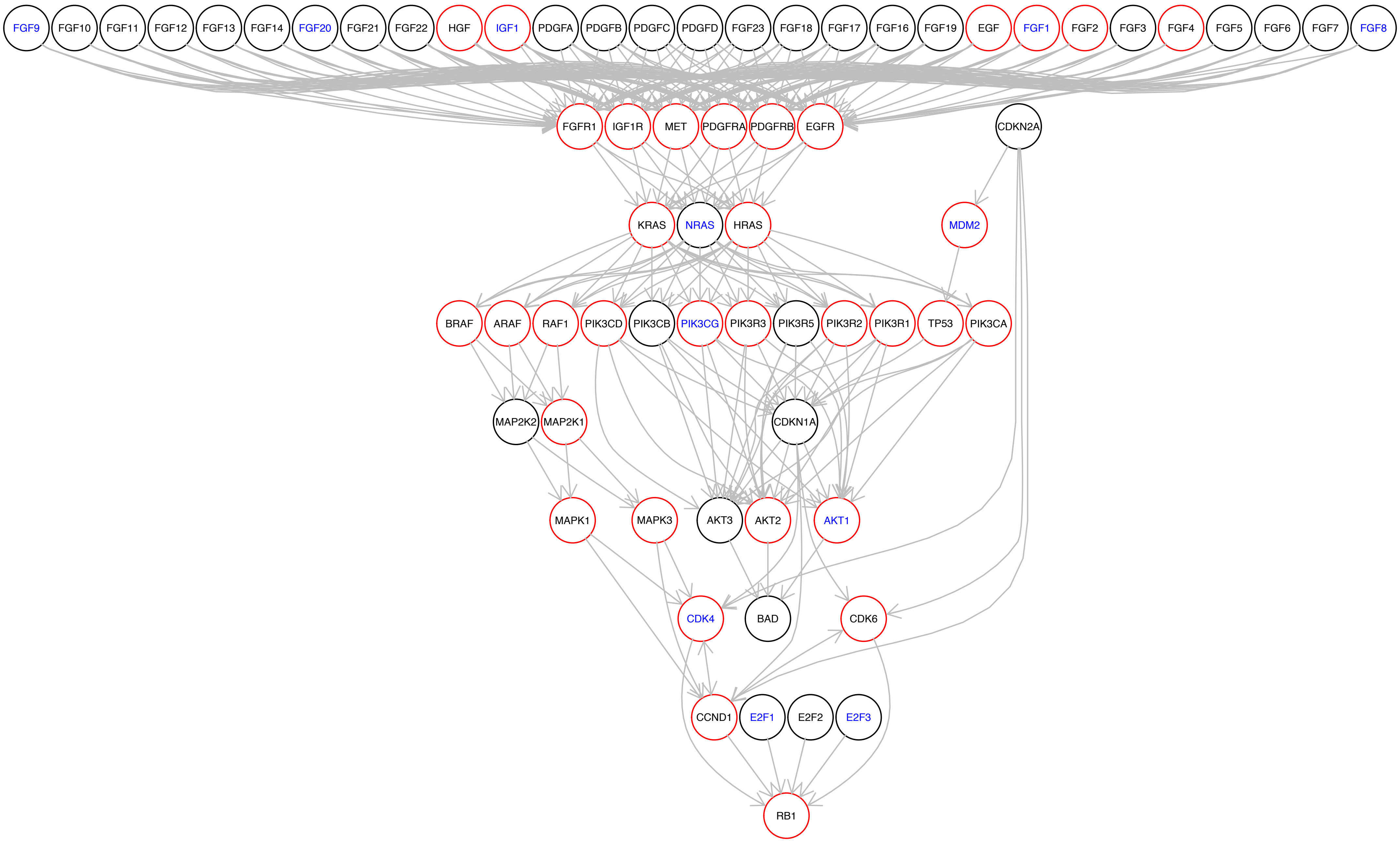

KEGG pathway = Melanoma : tumour = ME009 : Yellow Fill = gene variant, Blue Text = expression−survival association, Red Boarder = drug

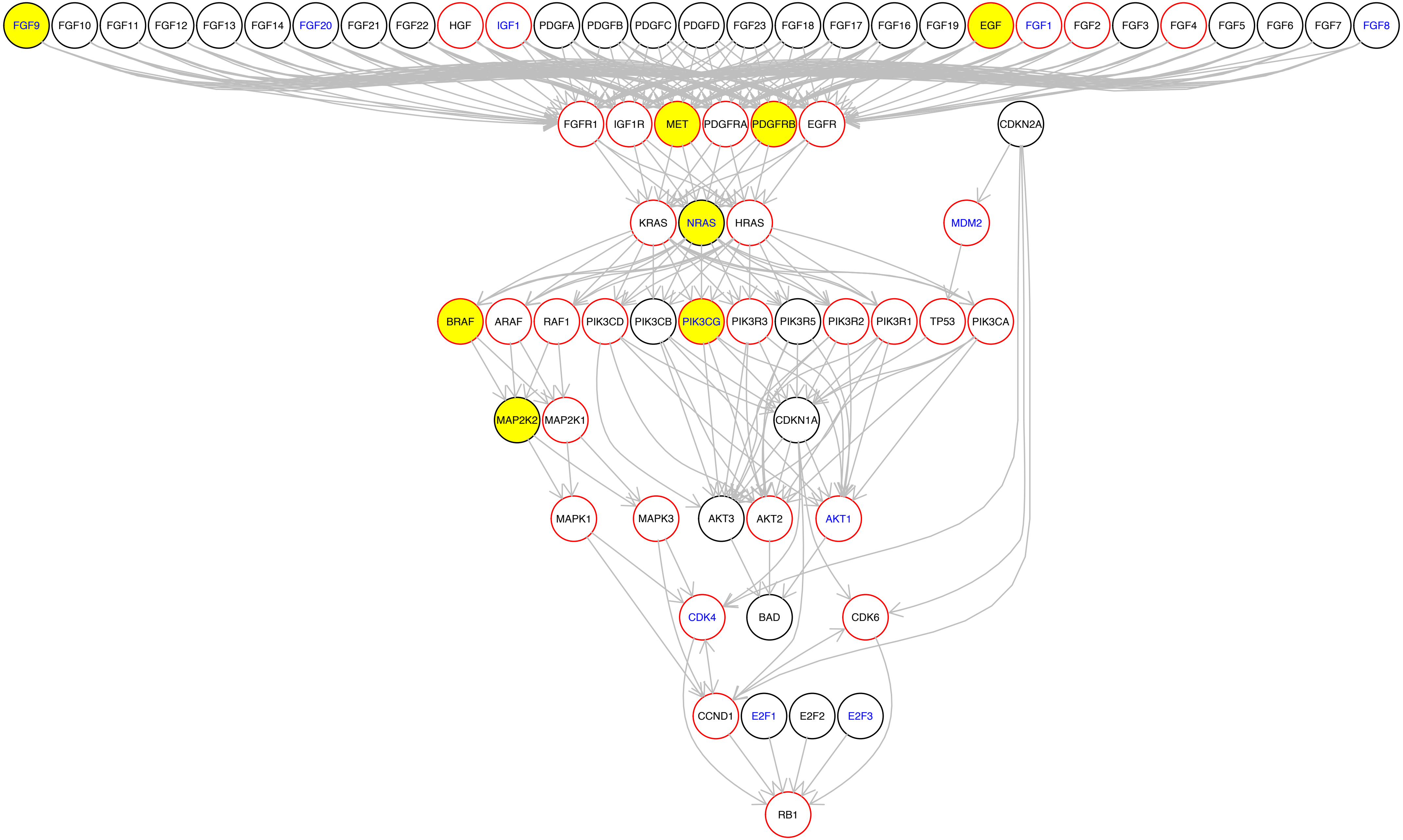

KEGG pathway = Melanoma :   tumour = ME011 :   Yellow Fill = gene variant, Blue Text = expression−survival association, Red Boarder = drug

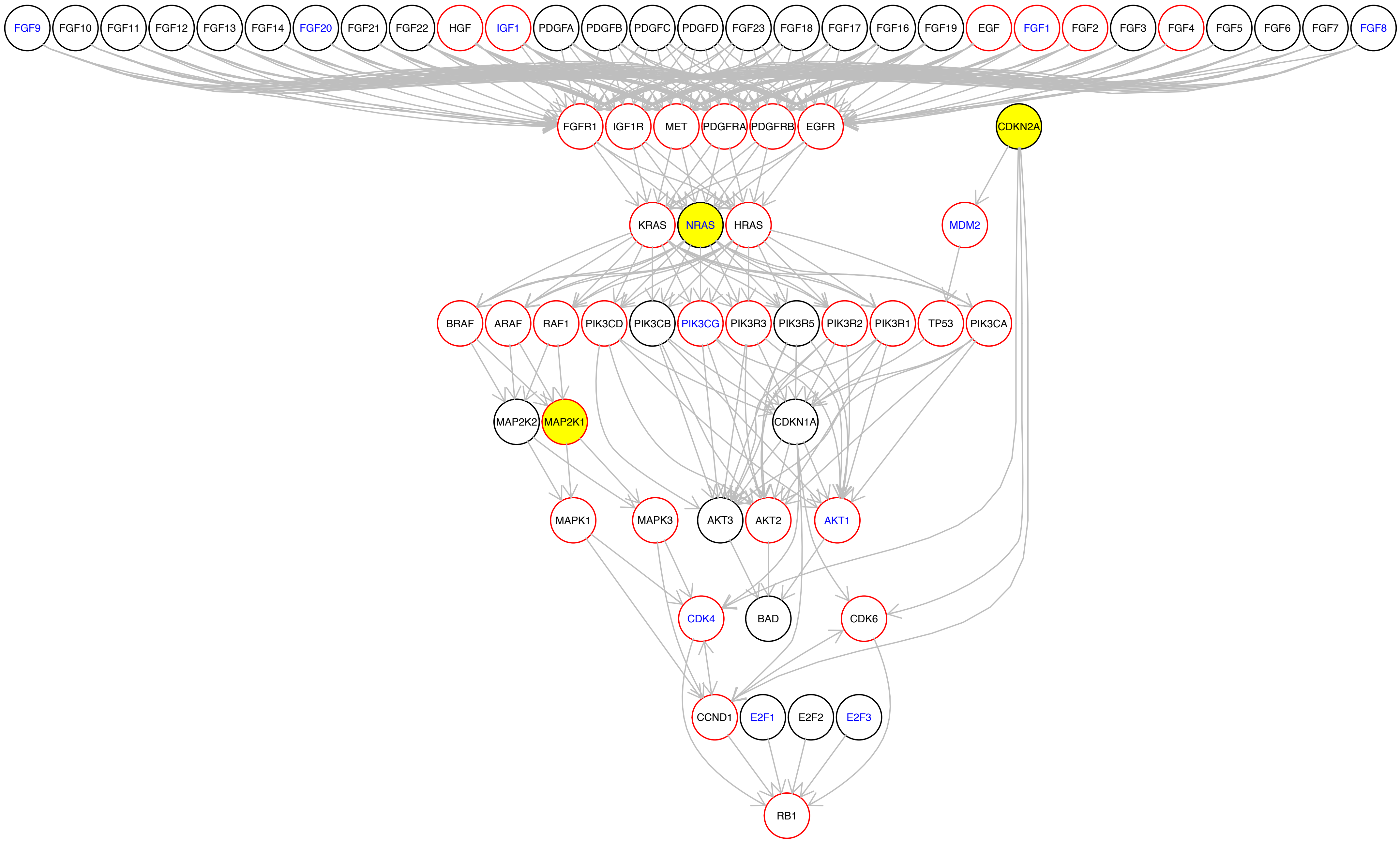

KEGG pathway = Melanoma : tumour = ME012 : Yellow Fill = gene variant, Blue Text = expression−survival association, Red Boarder = drug

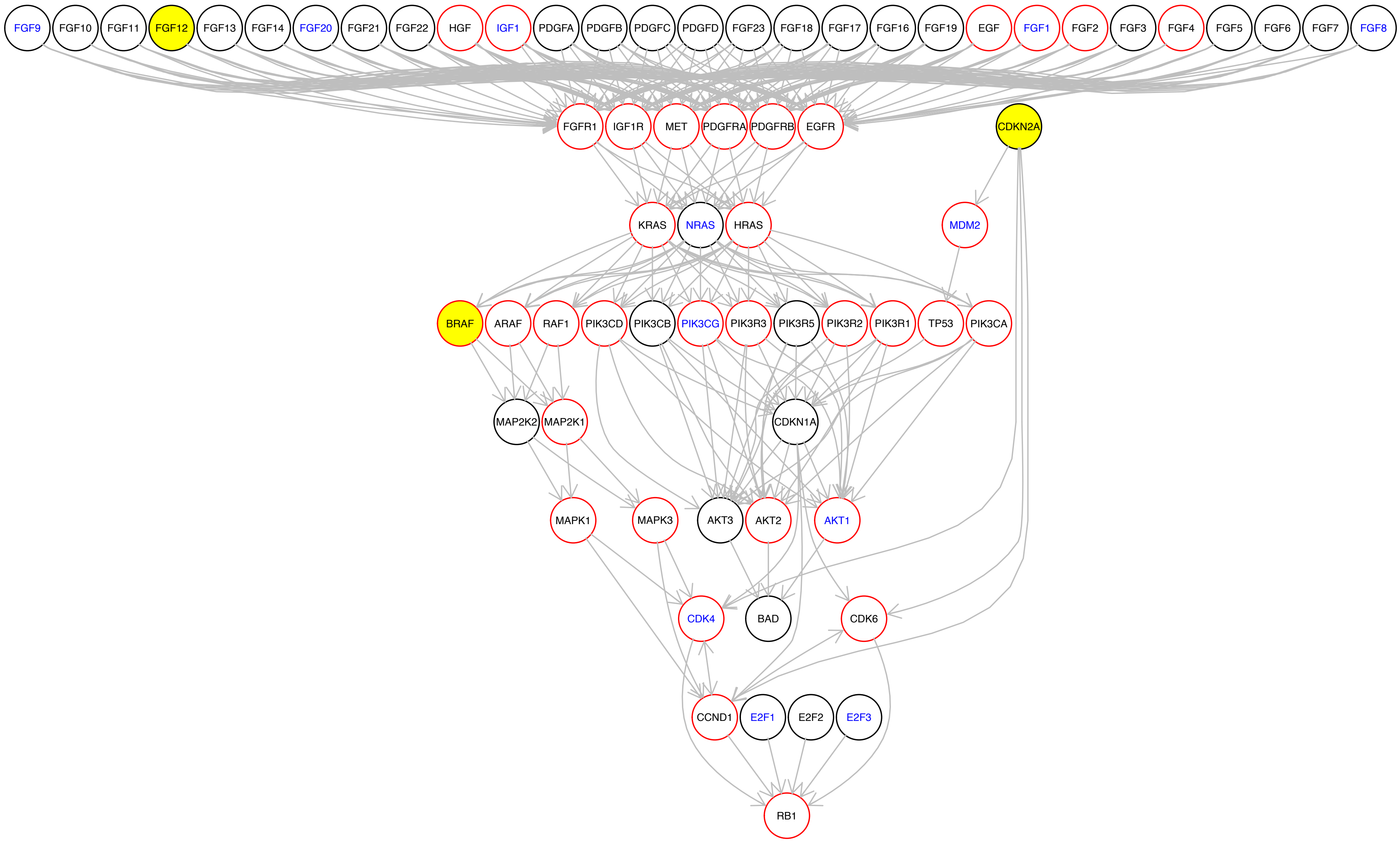

KEGG pathway = Melanoma : tumour = ME015 : Yellow Fill = gene variant, Blue Text = expression−survival association, Red Boarder = drug

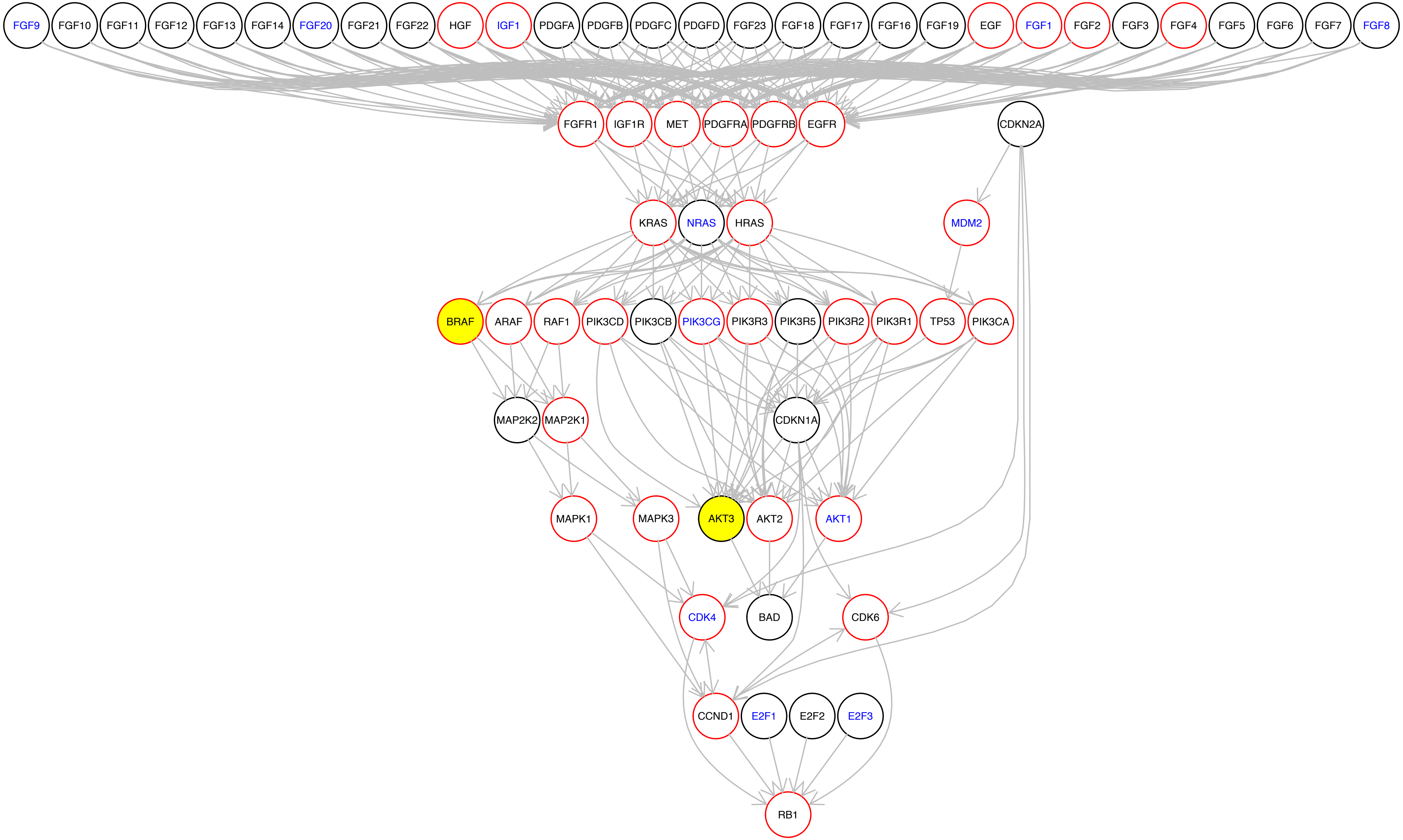

KEGG pathway = Melanoma : tumour = ME016 : Yellow Fill = gene variant, Blue Text = expression−survival association, Red Boarder = drug

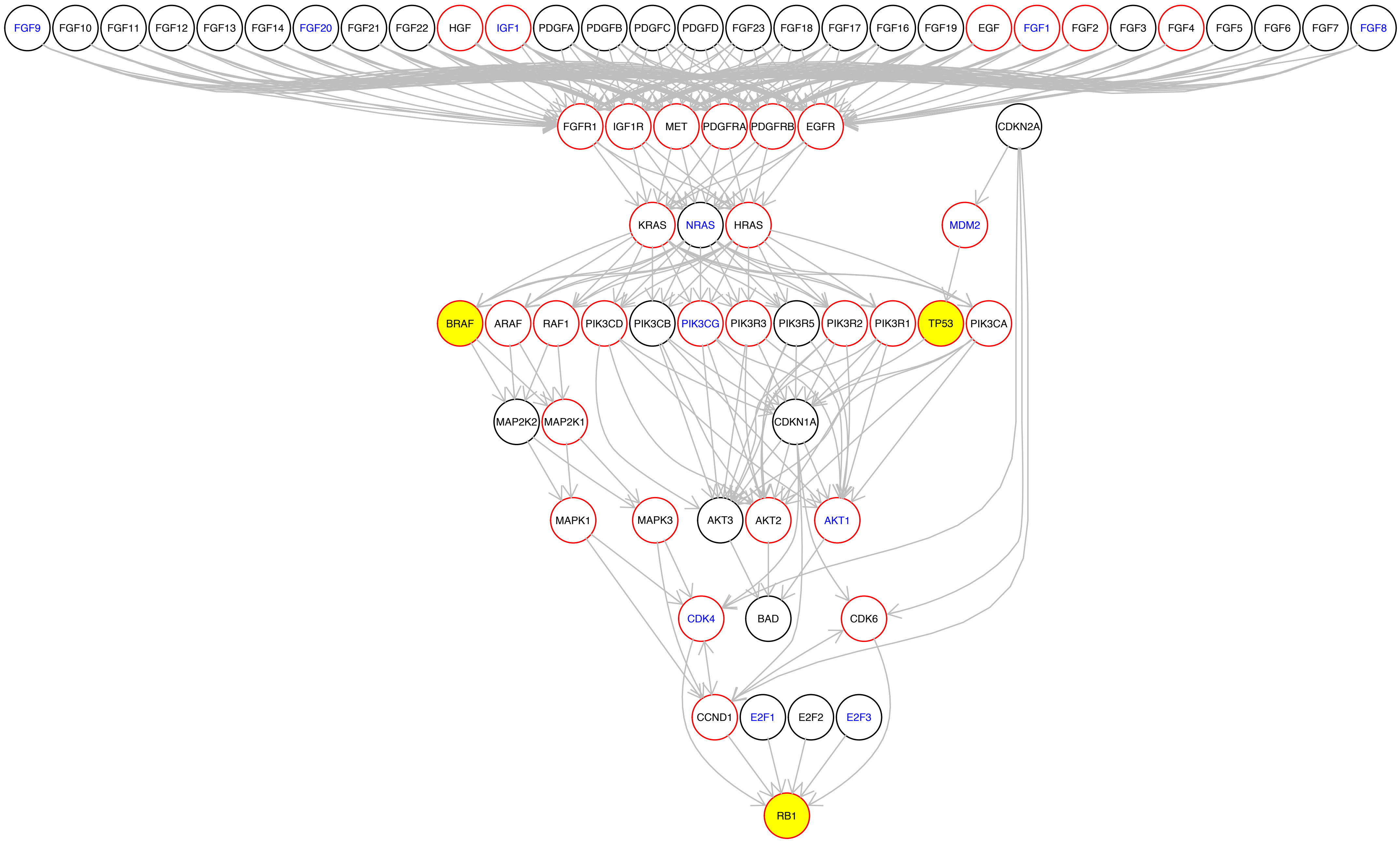

KEGG pathway = Melanoma : tumour = ME018 : Yellow Fill = gene variant, Blue Text = expression–survival association, Red Boarder = drug

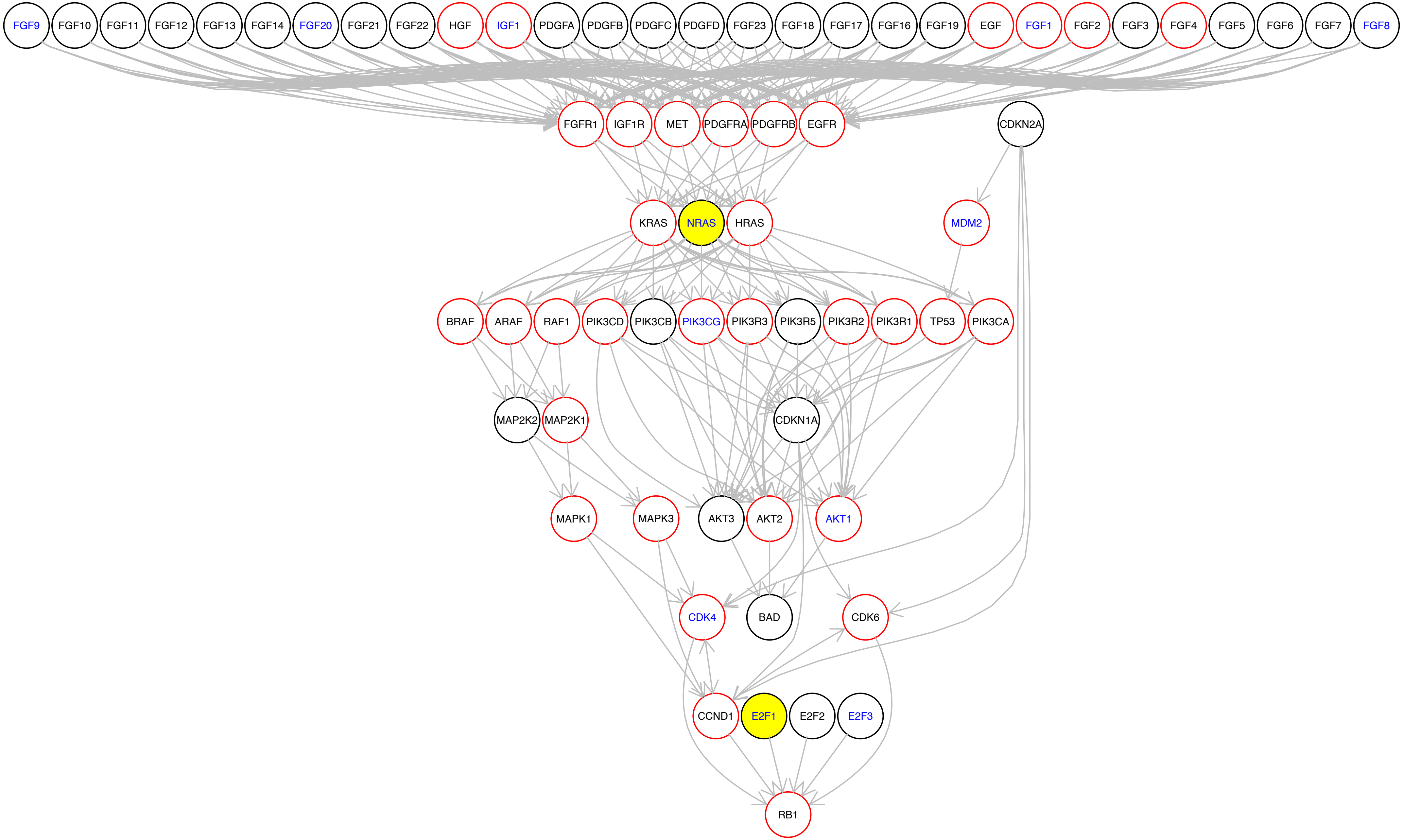

KEGG pathway = Melanoma : tumour = ME020 : Yellow Fill = gene variant, Blue Text = expression−survival association, Red Boarder = drug

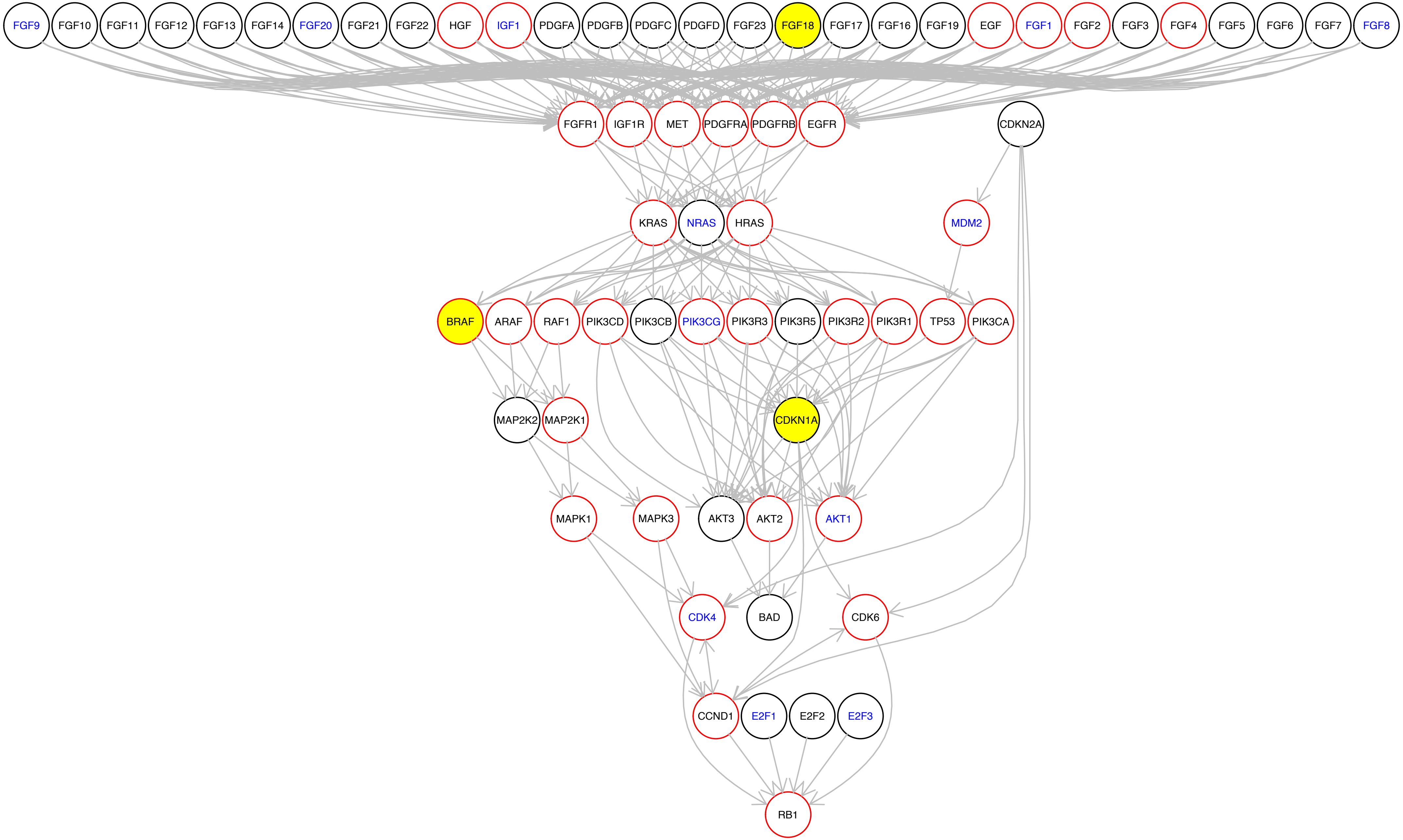

KEGG pathway = Melanoma : tumour = ME021 : Yellow Fill = gene variant, Blue Text = expression−survival association, Red Boarder = drug

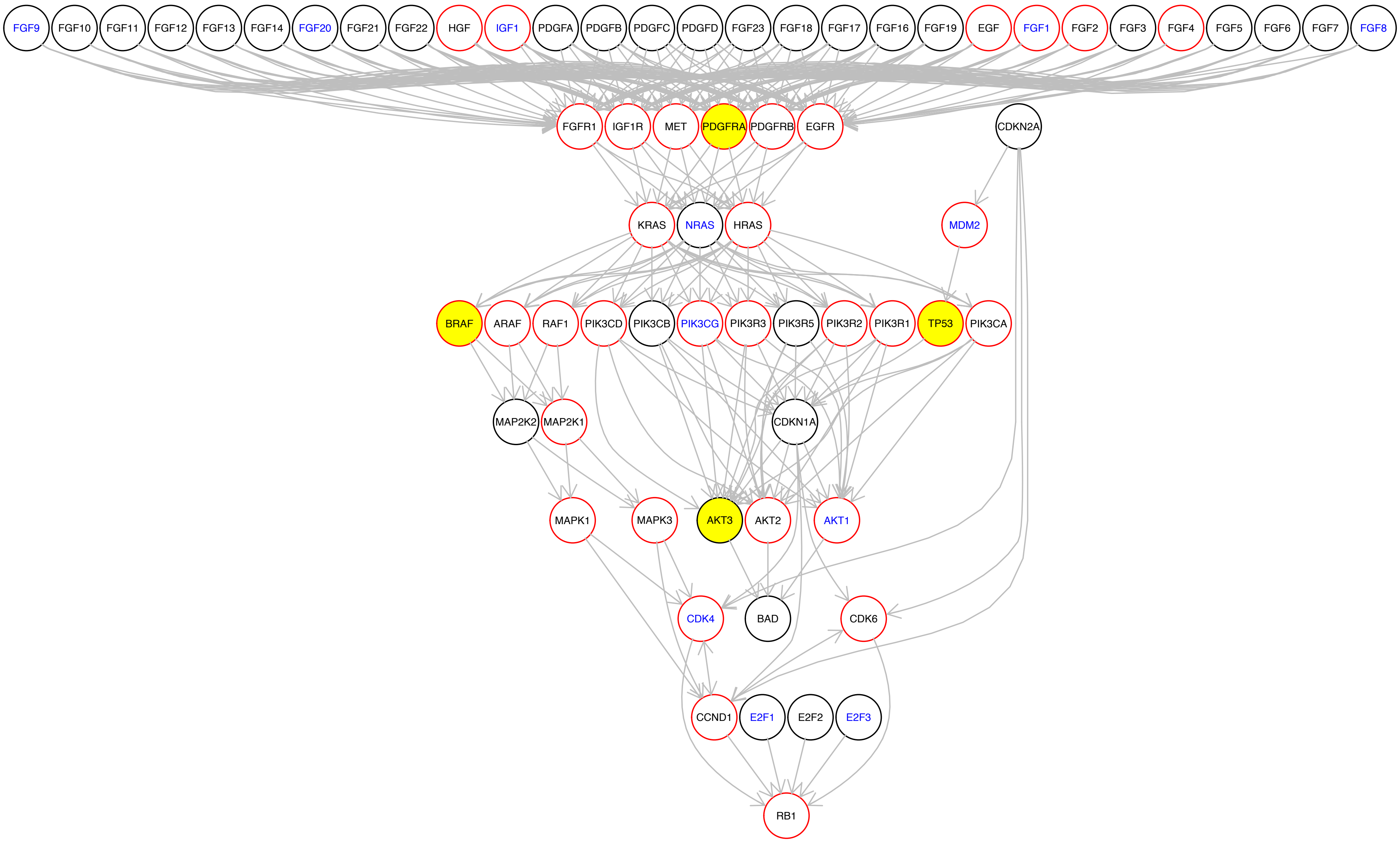

KEGG pathway = Melanoma : tumour = ME024 : Yellow Fill = gene variant, Blue Text = expression−survival association, Red Boarder = drug

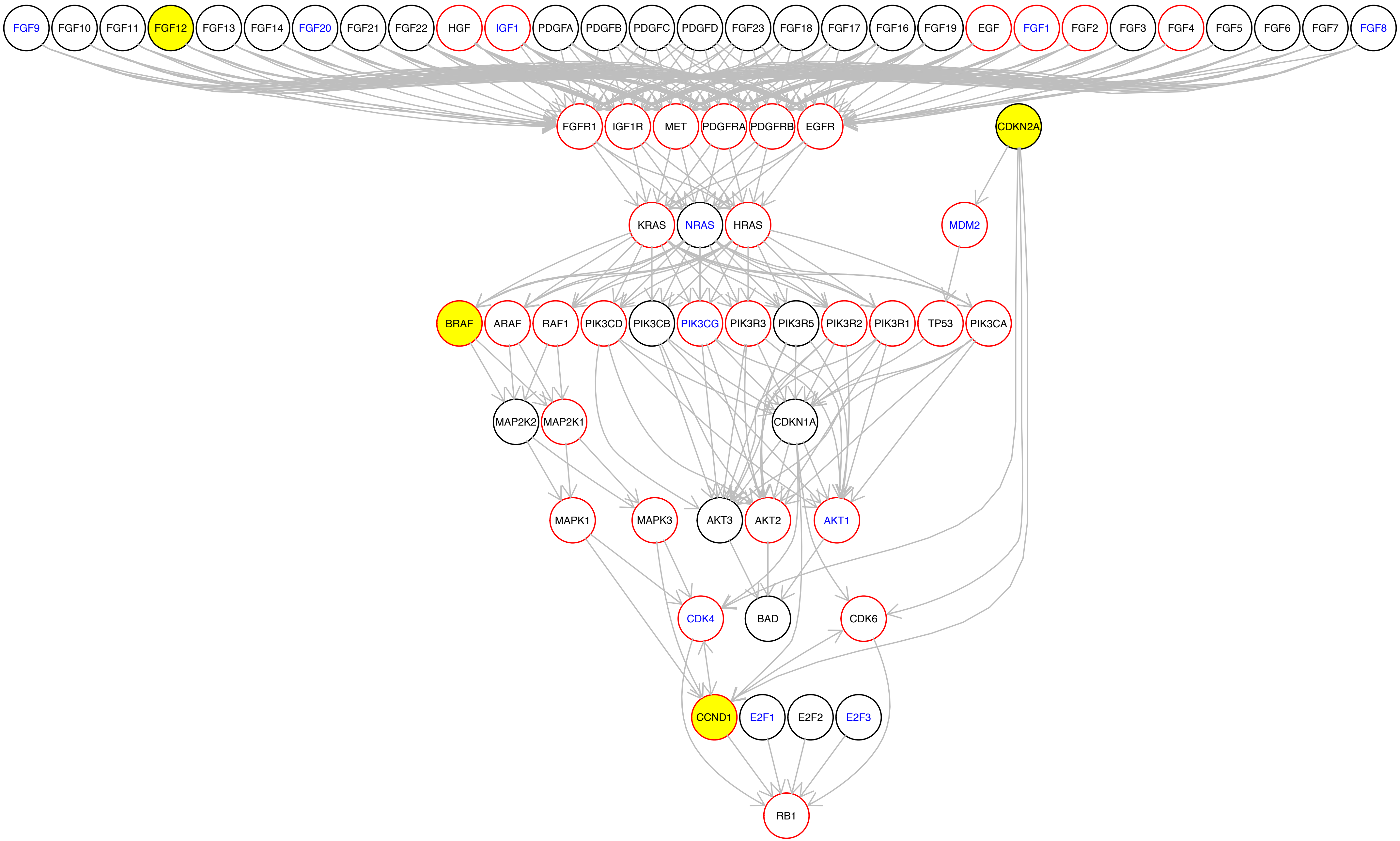

KEGG pathway = Melanoma : tumour = ME029 : Yellow Fill = gene variant, Blue Text = expression−survival association, Red Boarder = drug

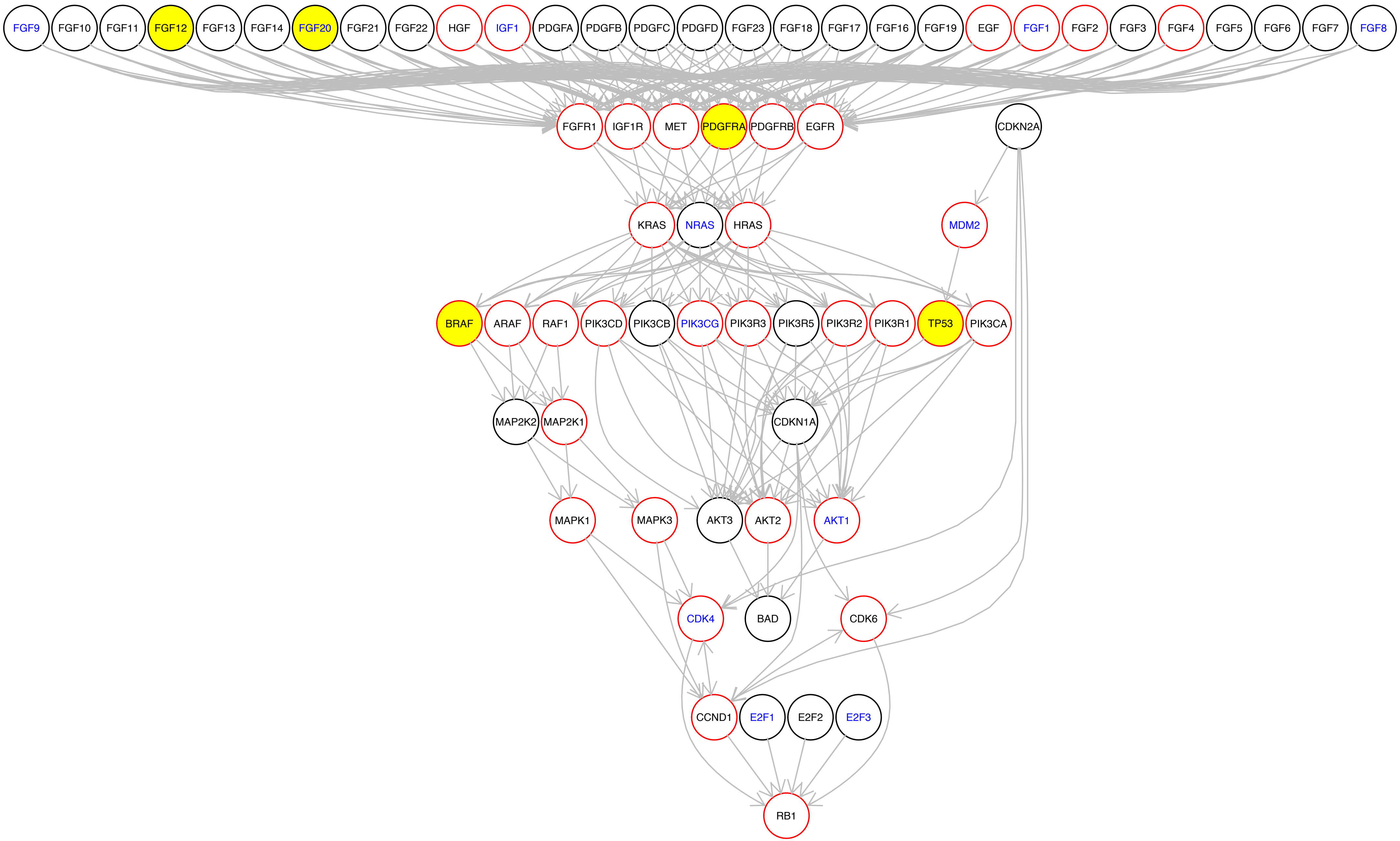

KEGG pathway = Melanoma : tumour = ME030 : Yellow Fill = gene variant, Blue Text = expression−survival association, Red Boarder = drug

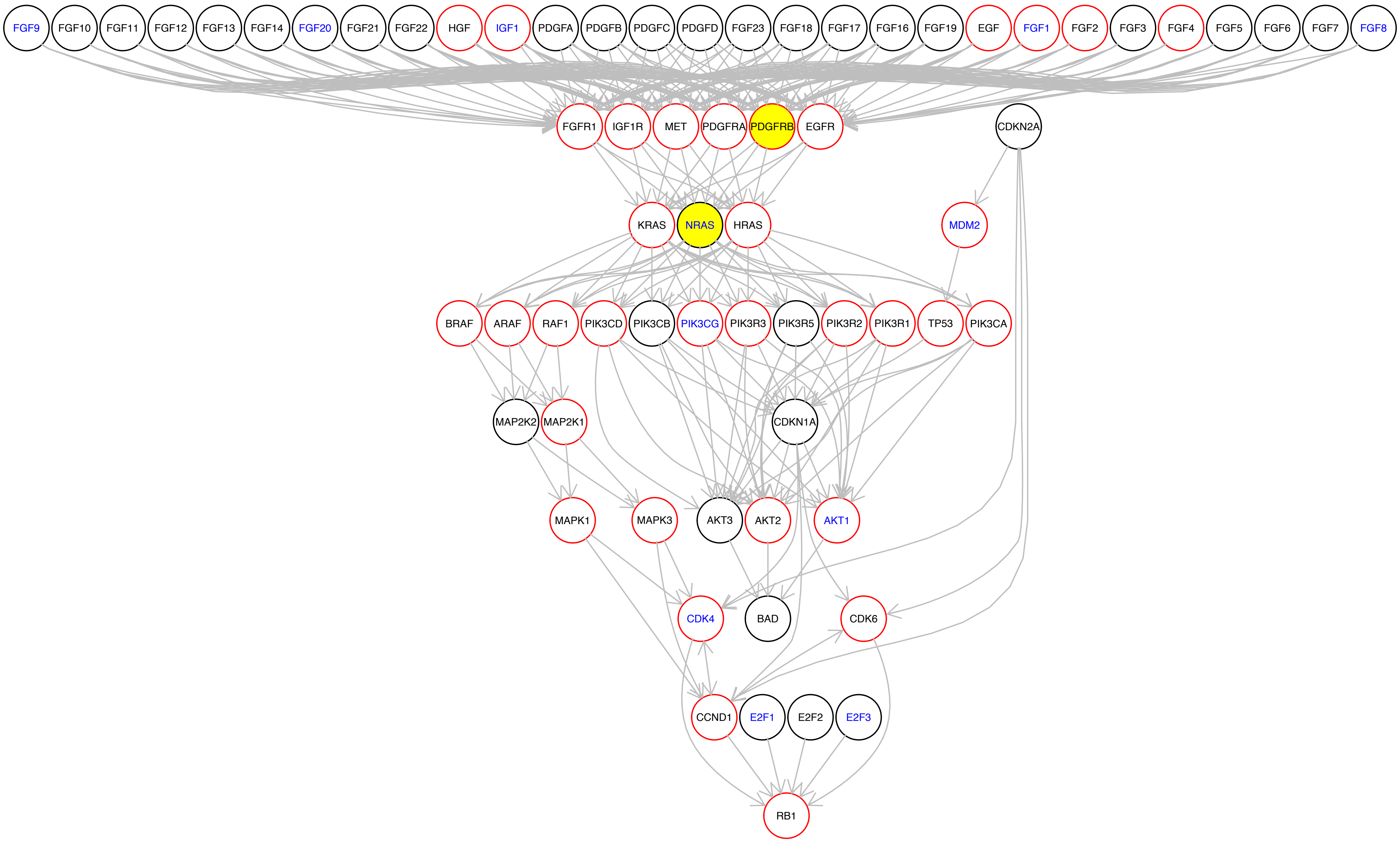

KEGG pathway = Melanoma : tumour = ME032 : Yellow Fill = gene variant, Blue Text = expression−survival association, Red Boarder = drug

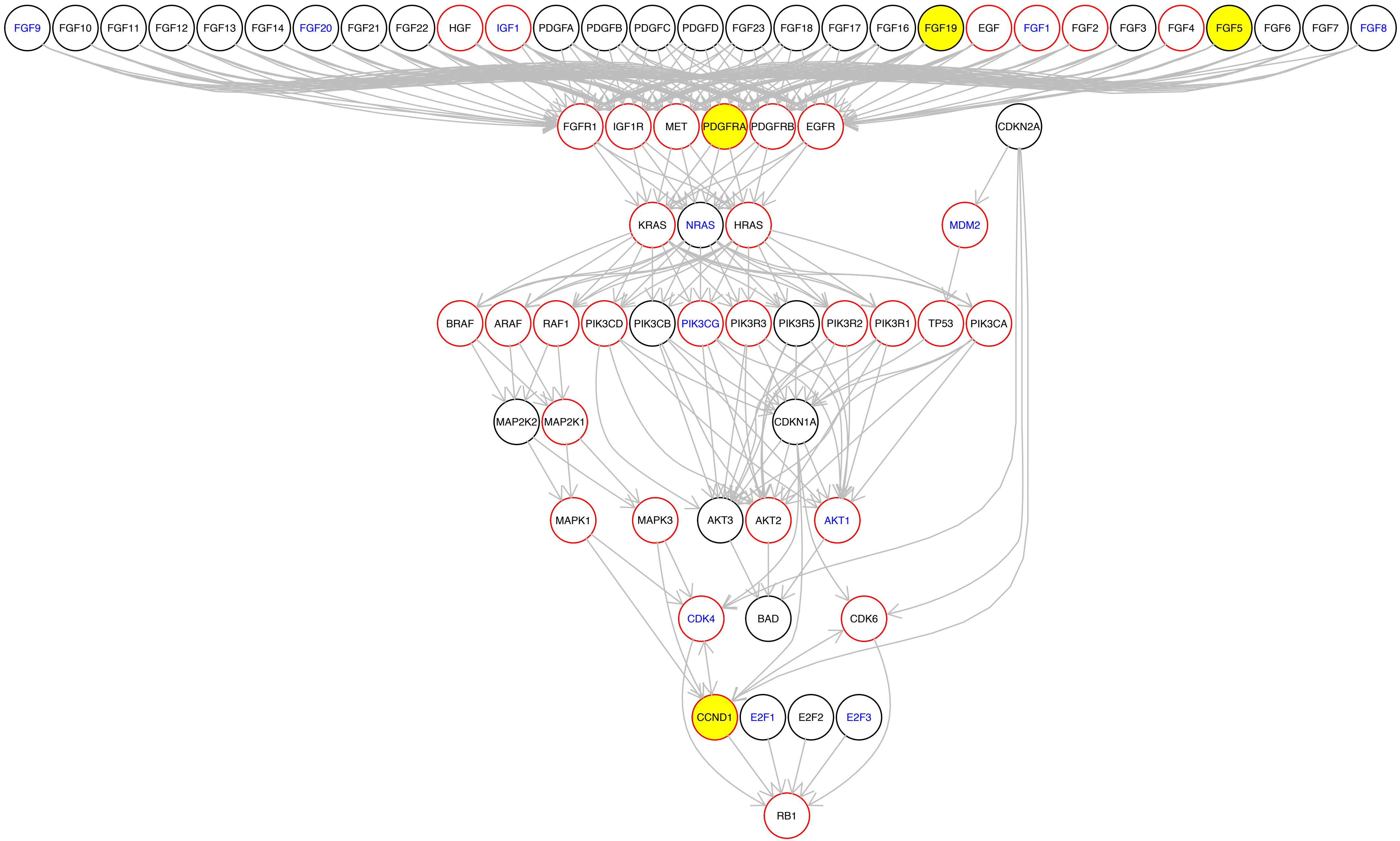

KEGG pathway = Melanoma : tumour = ME035 : Yellow Fill = gene variant, Blue Text = expression−survival association, Red Boarder = drug

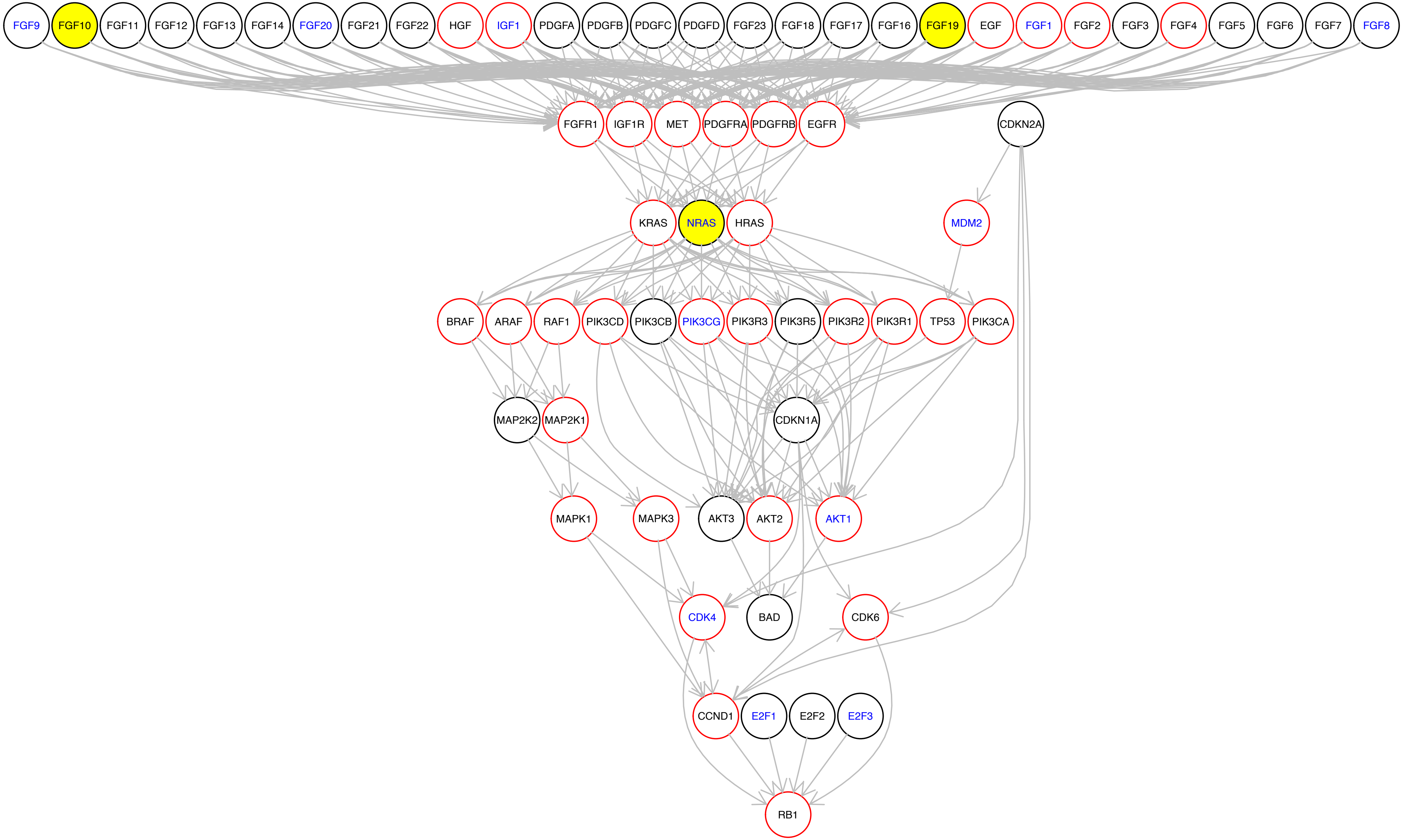

KEGG pathway = Melanoma : tumour = ME037 : Yellow Fill = gene variant, Blue Text = expression−survival association, Red Boarder = drug

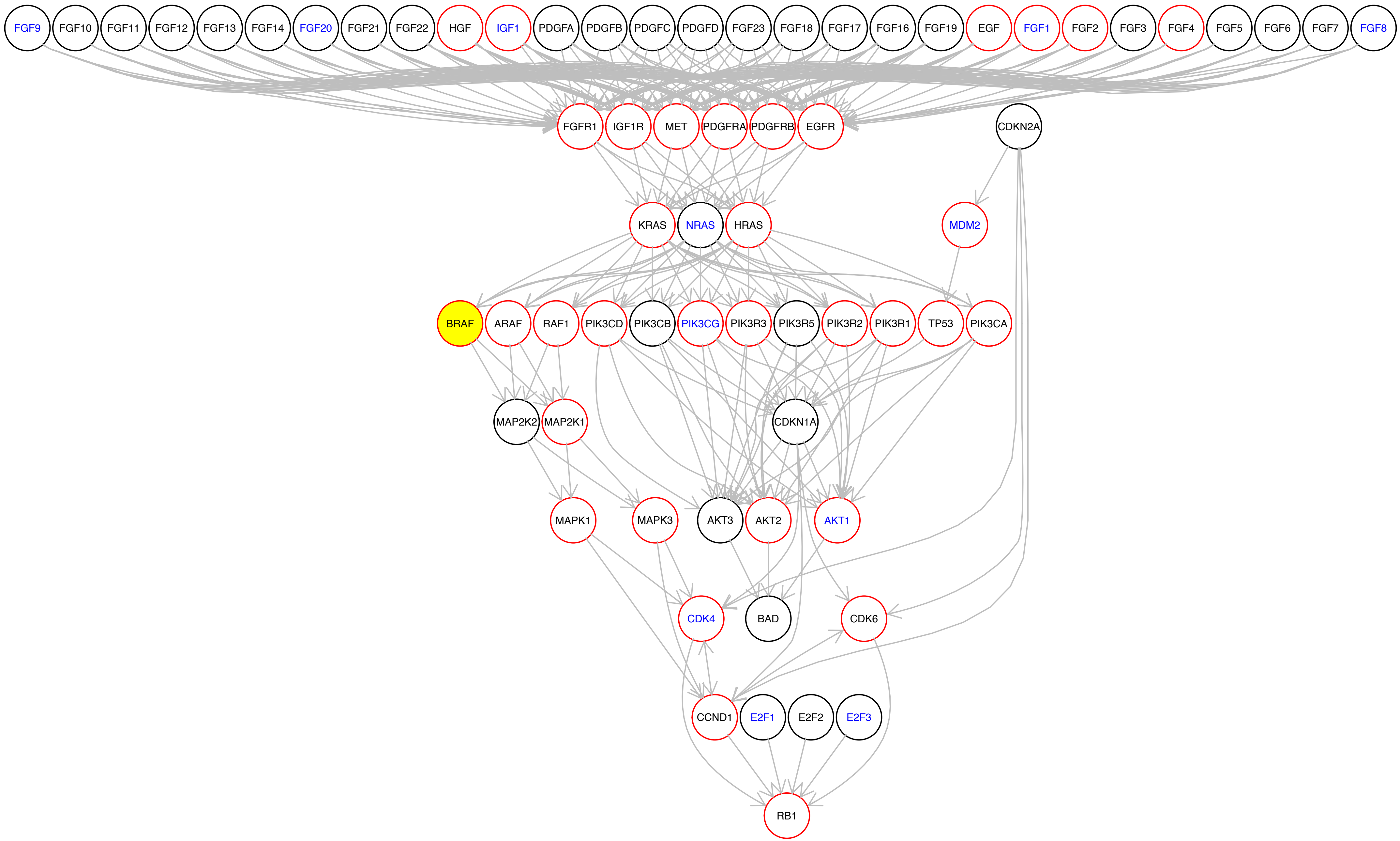

KEGG pathway = Melanoma : tumour = ME041 : Yellow Fill = gene variant, Blue Text = expression−survival association, Red Boarder = drug

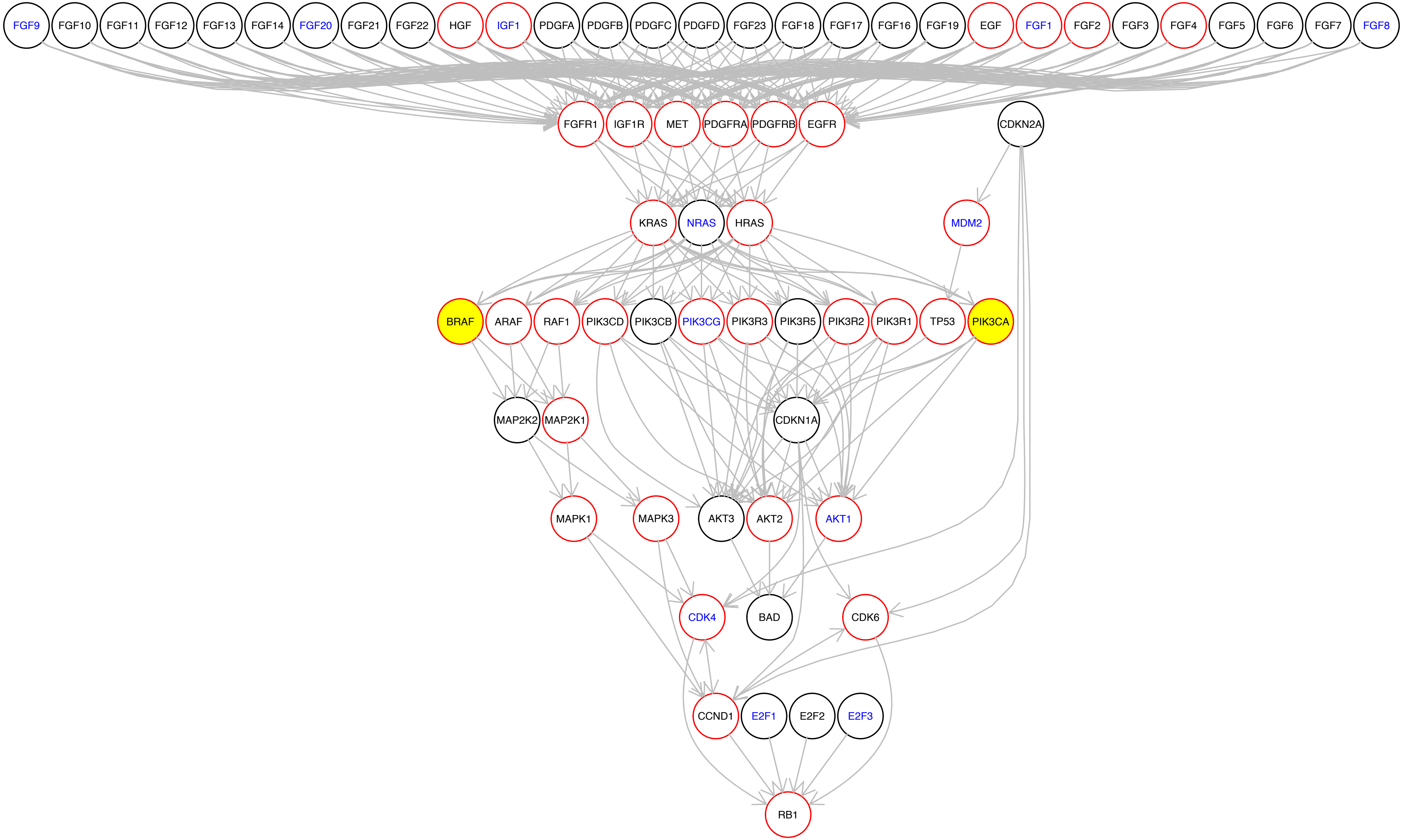

KEGG pathway = Melanoma : tumour = ME043 : Yellow Fill = gene variant, Blue Text = expression−survival association, Red Boarder = drug

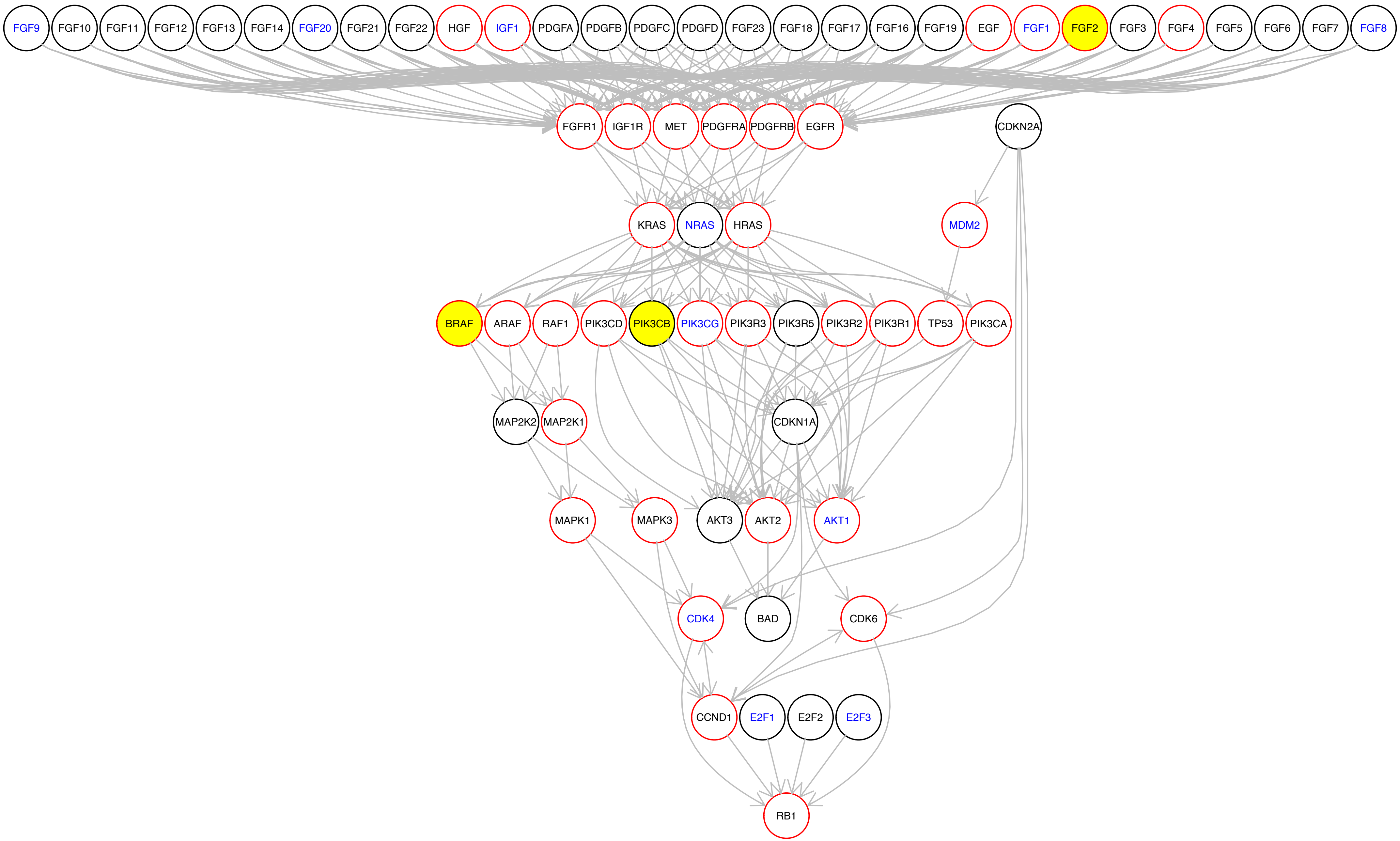

KEGG pathway = Melanoma : tumour = ME044 : Yellow Fill = gene variant, Blue Text = expression−survival association, Red Boarder = drug

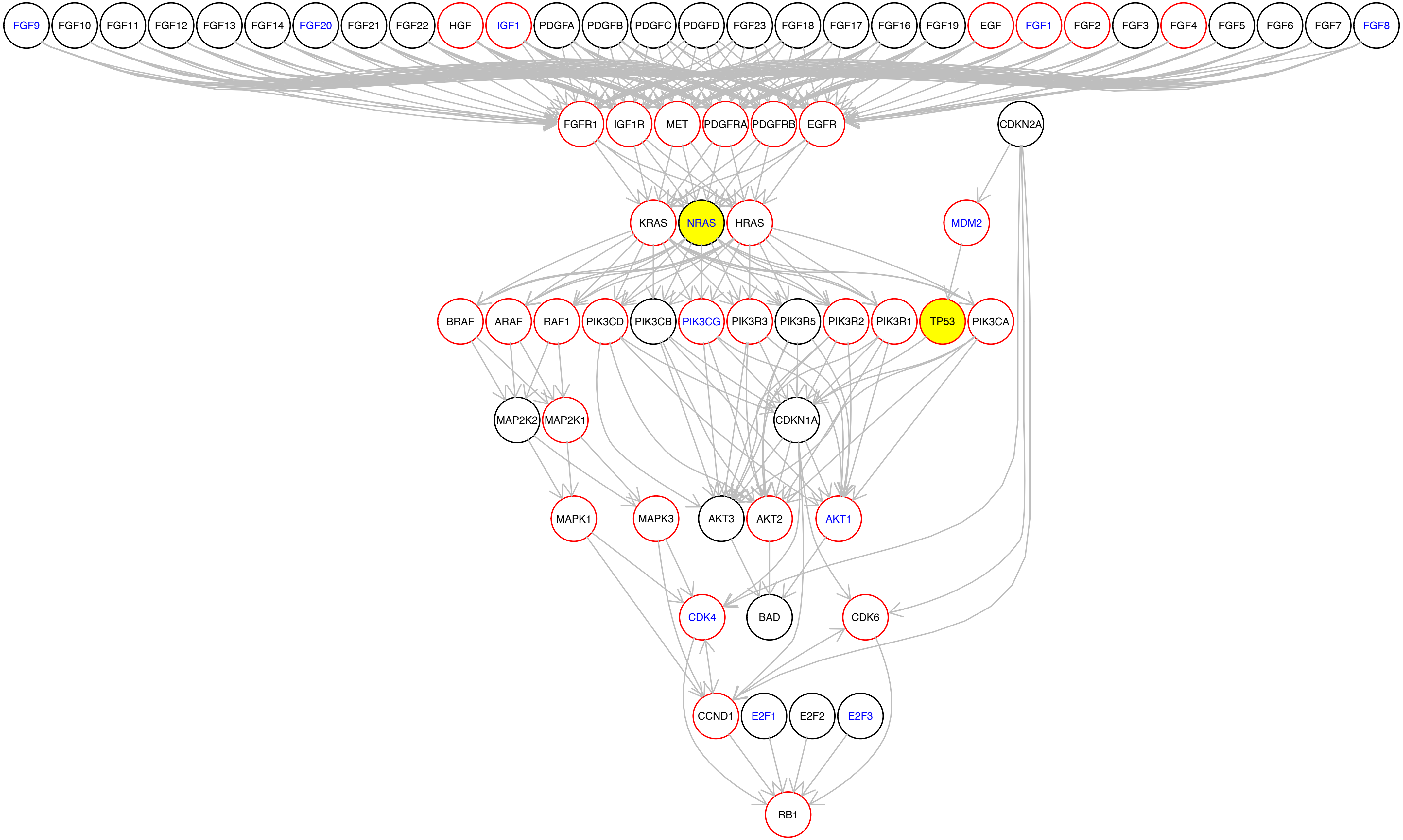

KEGG pathway = Melanoma : tumour = ME045 : Yellow Fill = gene variant, Blue Text = expression−survival association, Red Boarder = drug

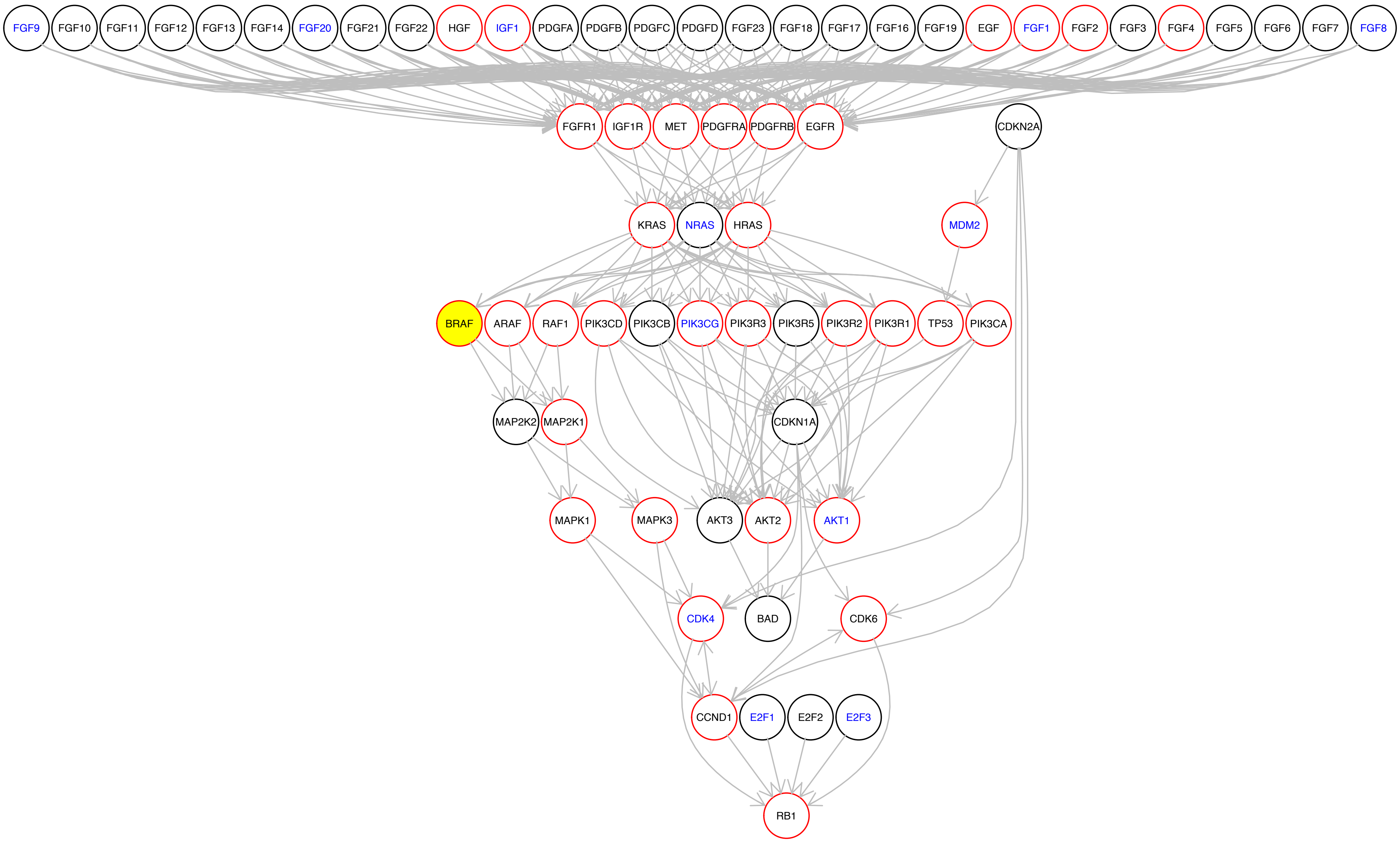

KEGG pathway = Melanoma : tumour = ME048 : Yellow Fill = gene variant, Blue Text = expression−survival association, Red Boarder = drug

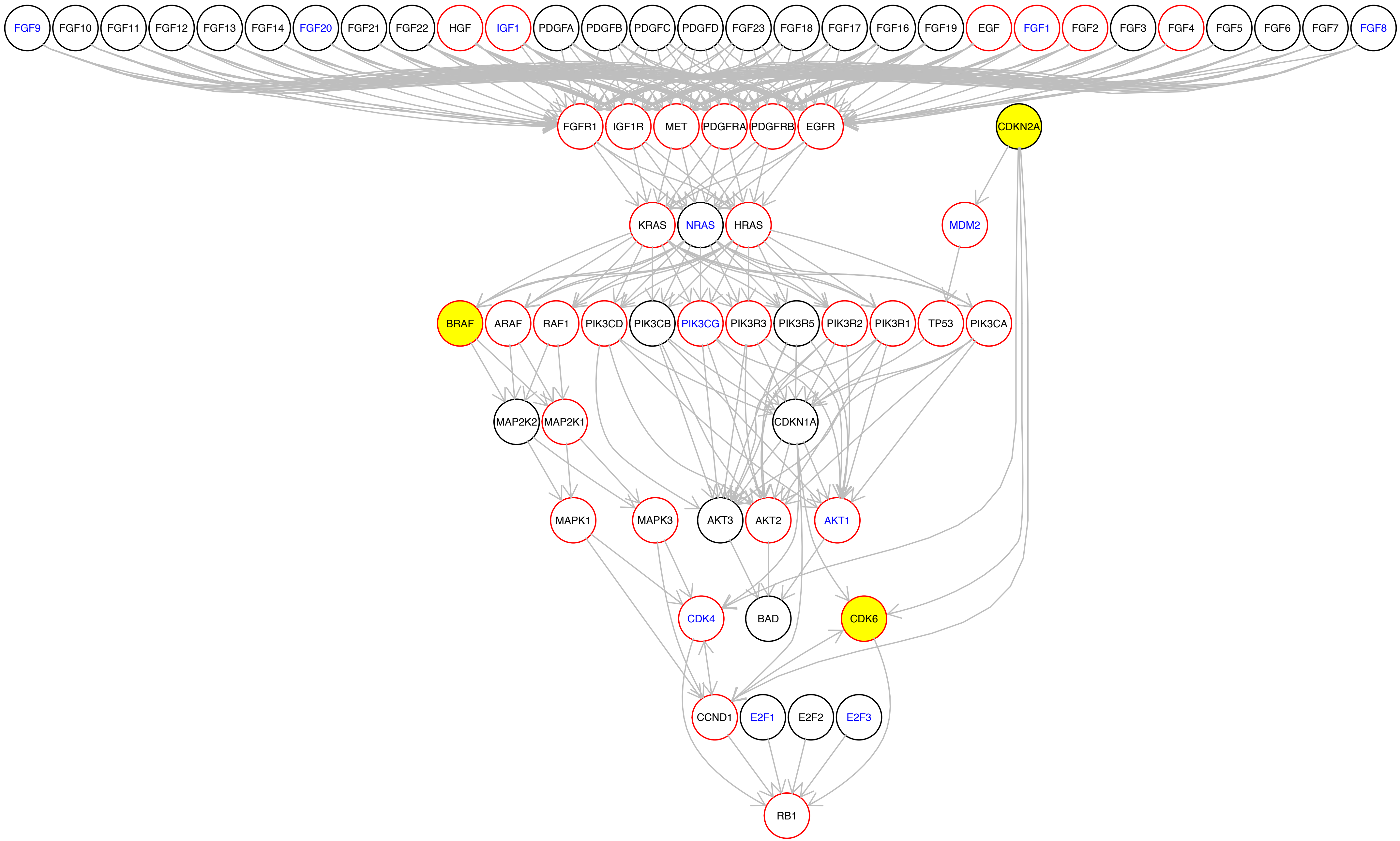

KEGG pathway = Melanoma : tumour = ME049 : Yellow Fill = gene variant, Blue Text = expression−survival association, Red Boarder = drug

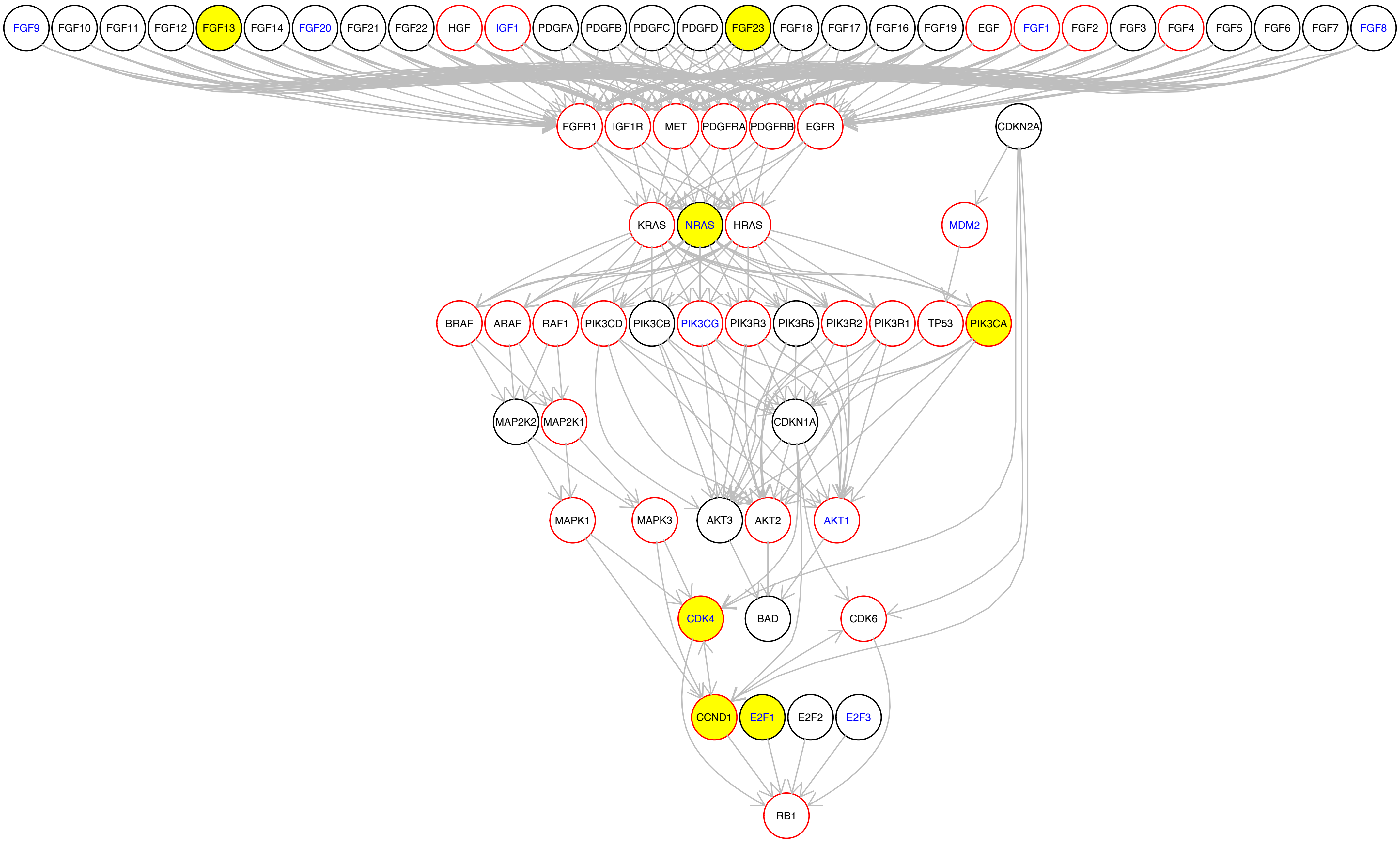

KEGG pathway = Melanoma : tumour = ME050 : Yellow Fill = gene variant, Blue Text = expression−survival association, Red Boarder = drug

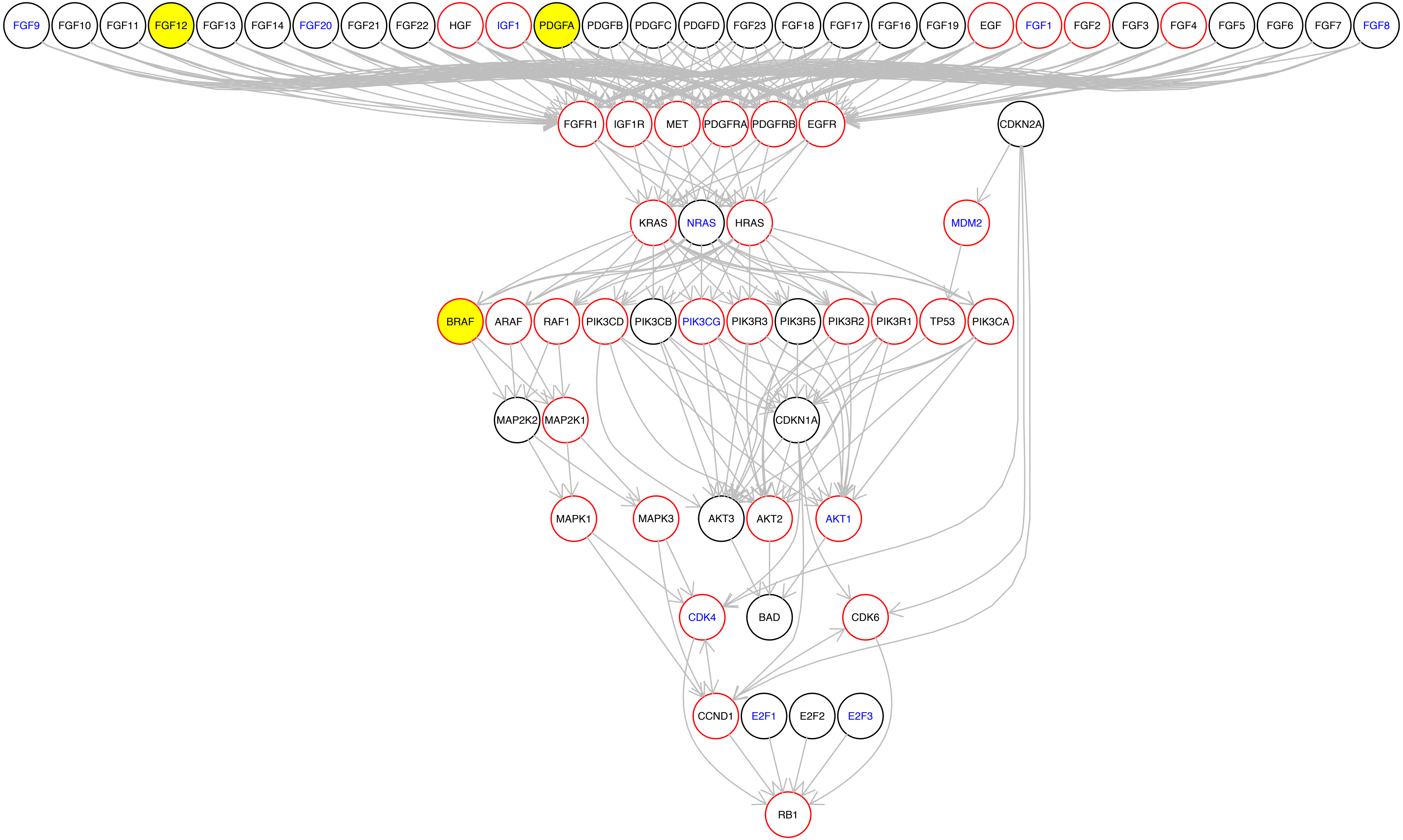

KEGG pathway = Melanoma : tumour = ME100L : Yellow Fill = gene variant, Blue Text = expression−survival association, Red Boarder = drug

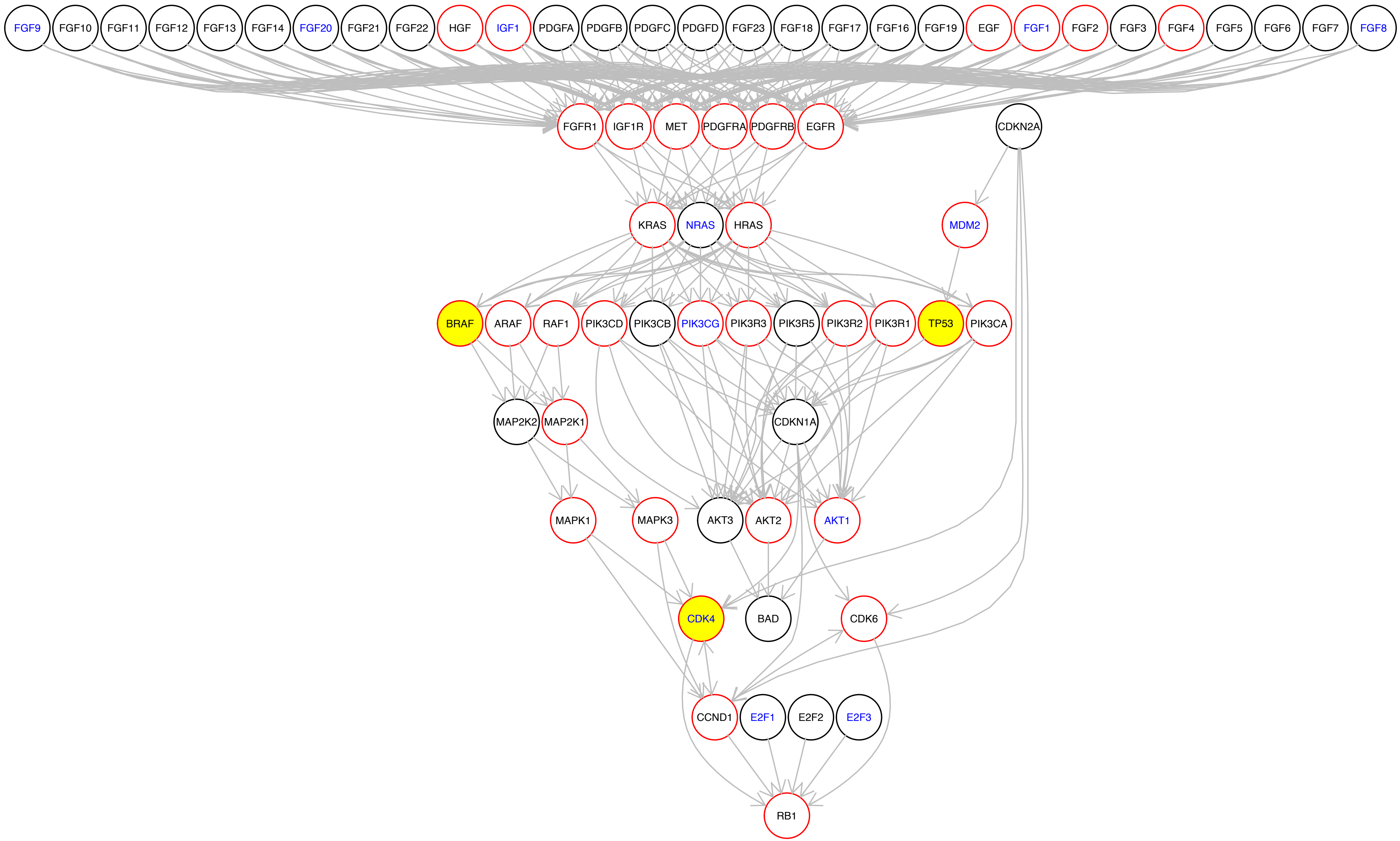

KEGG pathway = Melanoma : tumour = A101D_SKIN : Yellow Fill = gene variant, Blue Text = expression−survival association, Red Boarder = drug

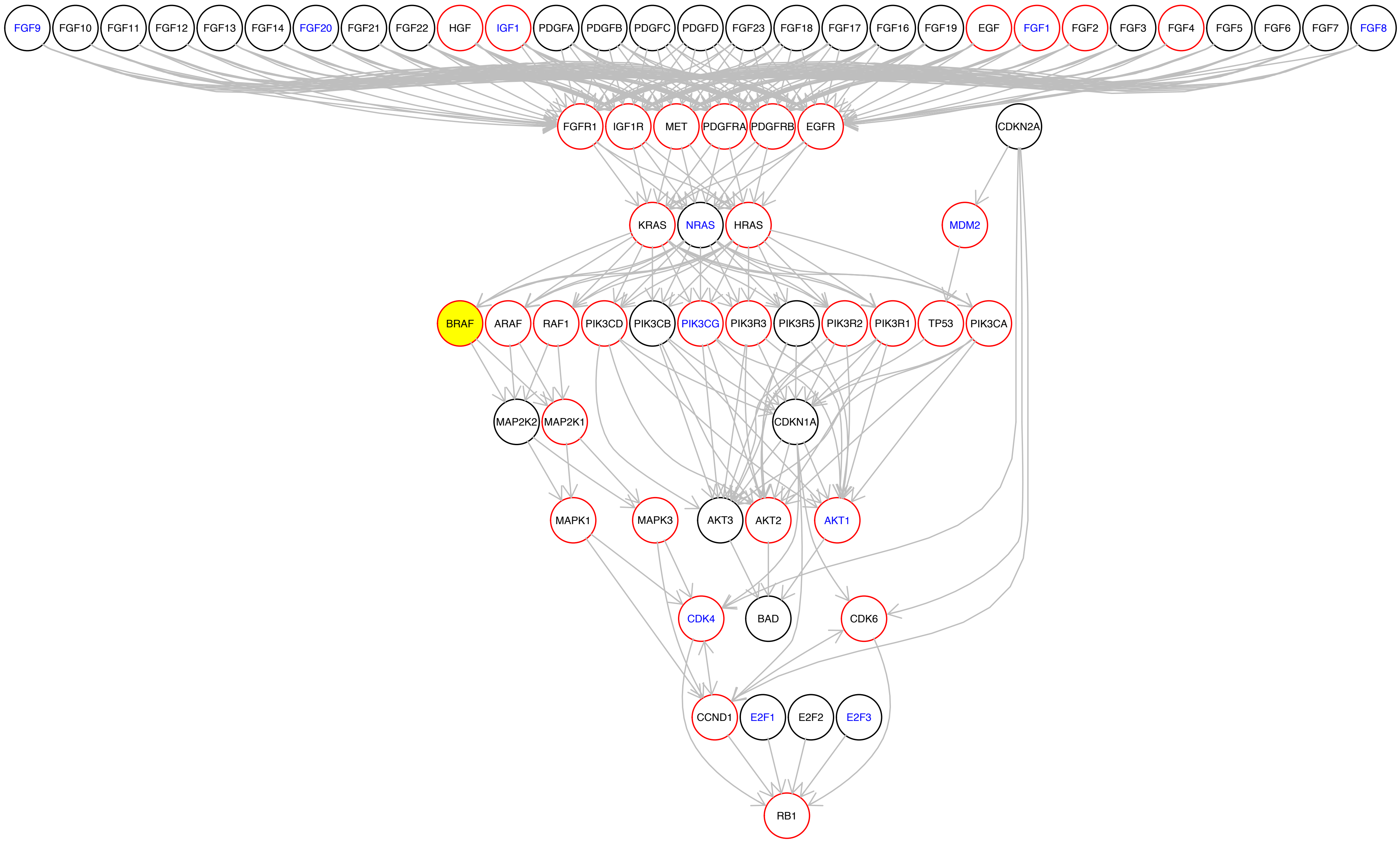

KEGG pathway = Melanoma :   tumour = A2058_SKIN :   Yellow Fill = gene variant, Blue Text = expression−survival association, Red Boarder = drug

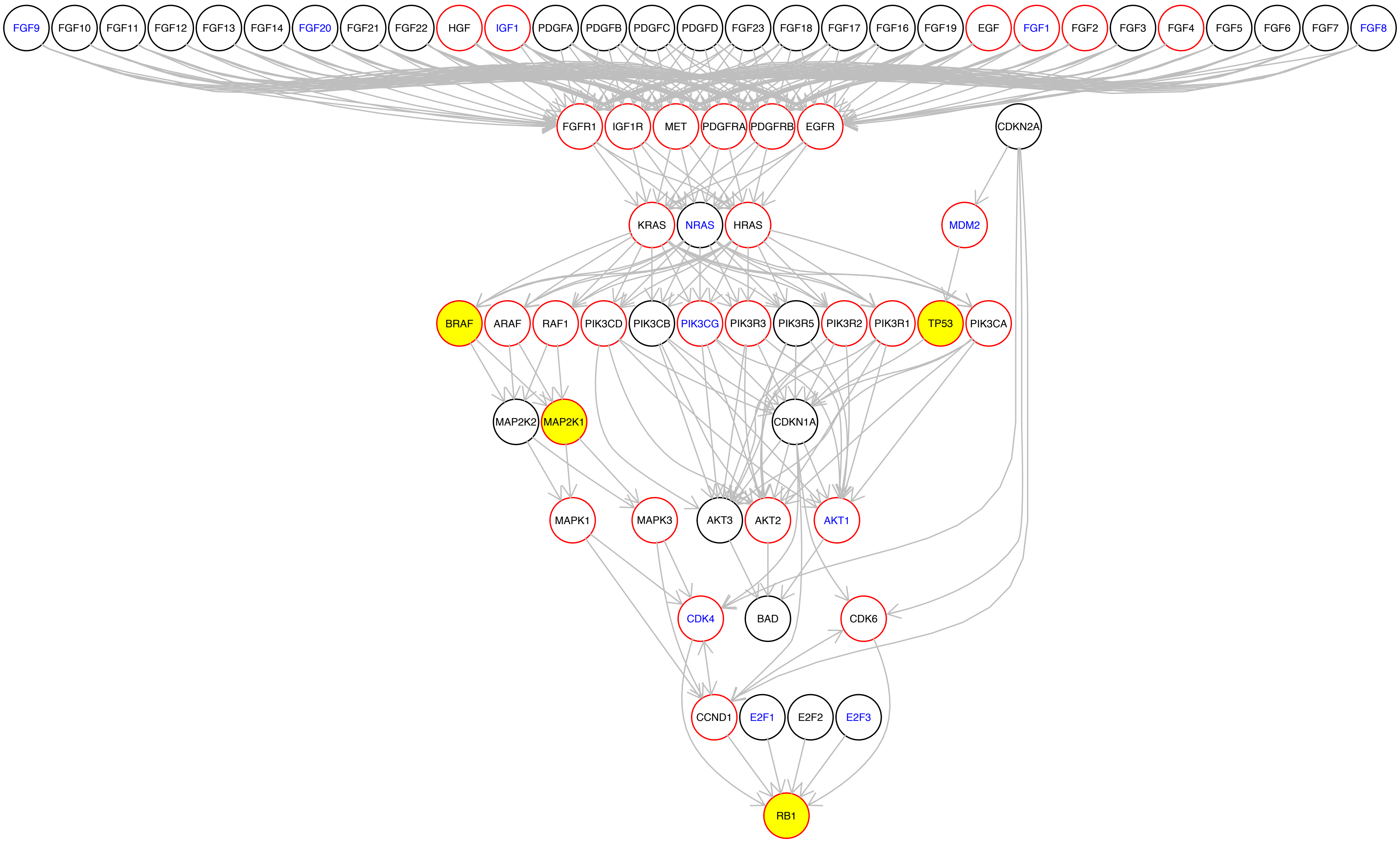

KEGG pathway = Melanoma : tumour = A375_SKIN : Yellow Fill = gene variant, Blue Text = expression−survival association, Red Boarder = drug

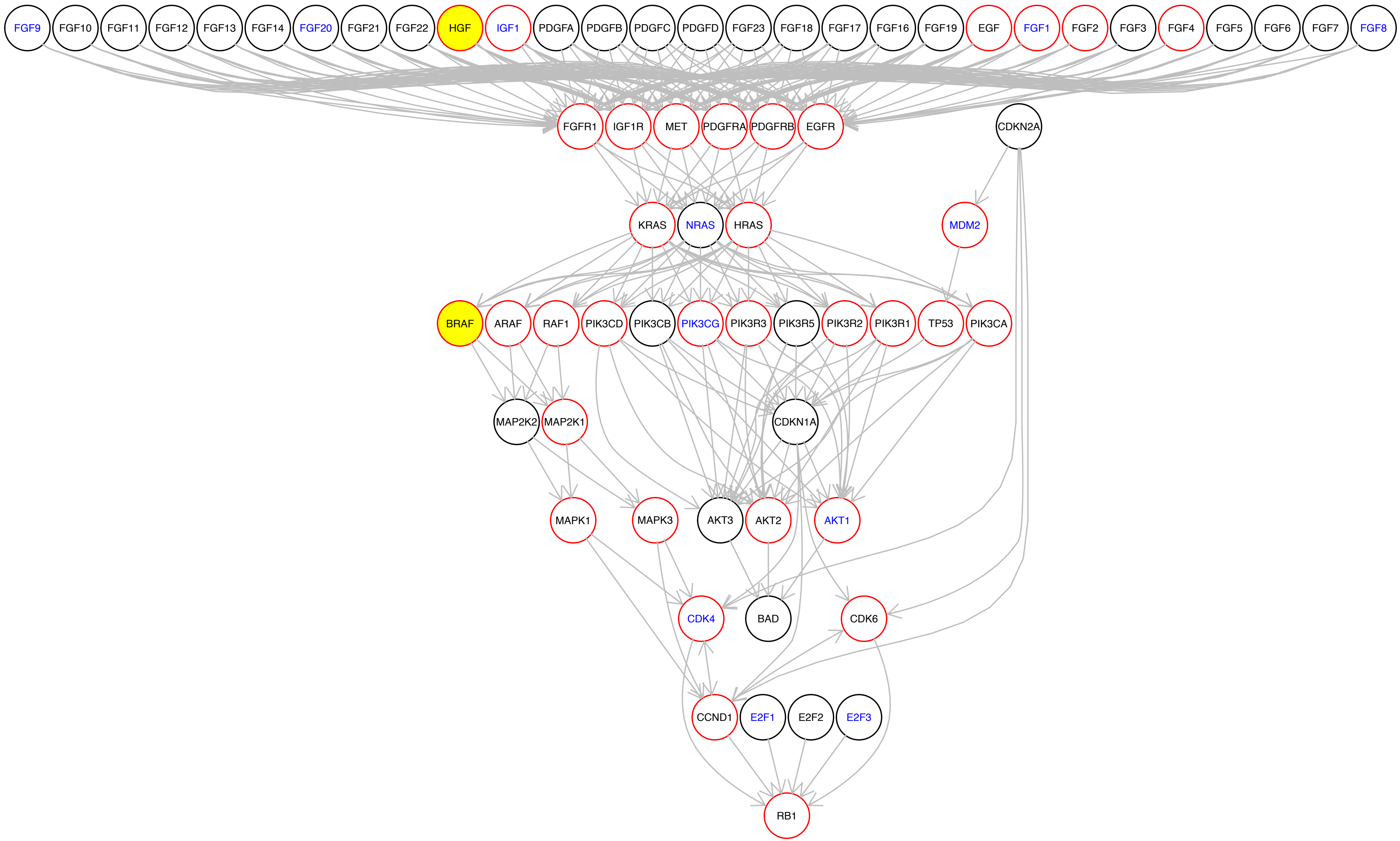

KEGG pathway = Melanoma : tumour = C32_SKIN : Yellow Fill = gene variant, Blue Text = expression−survival association, Red Boarder = drug

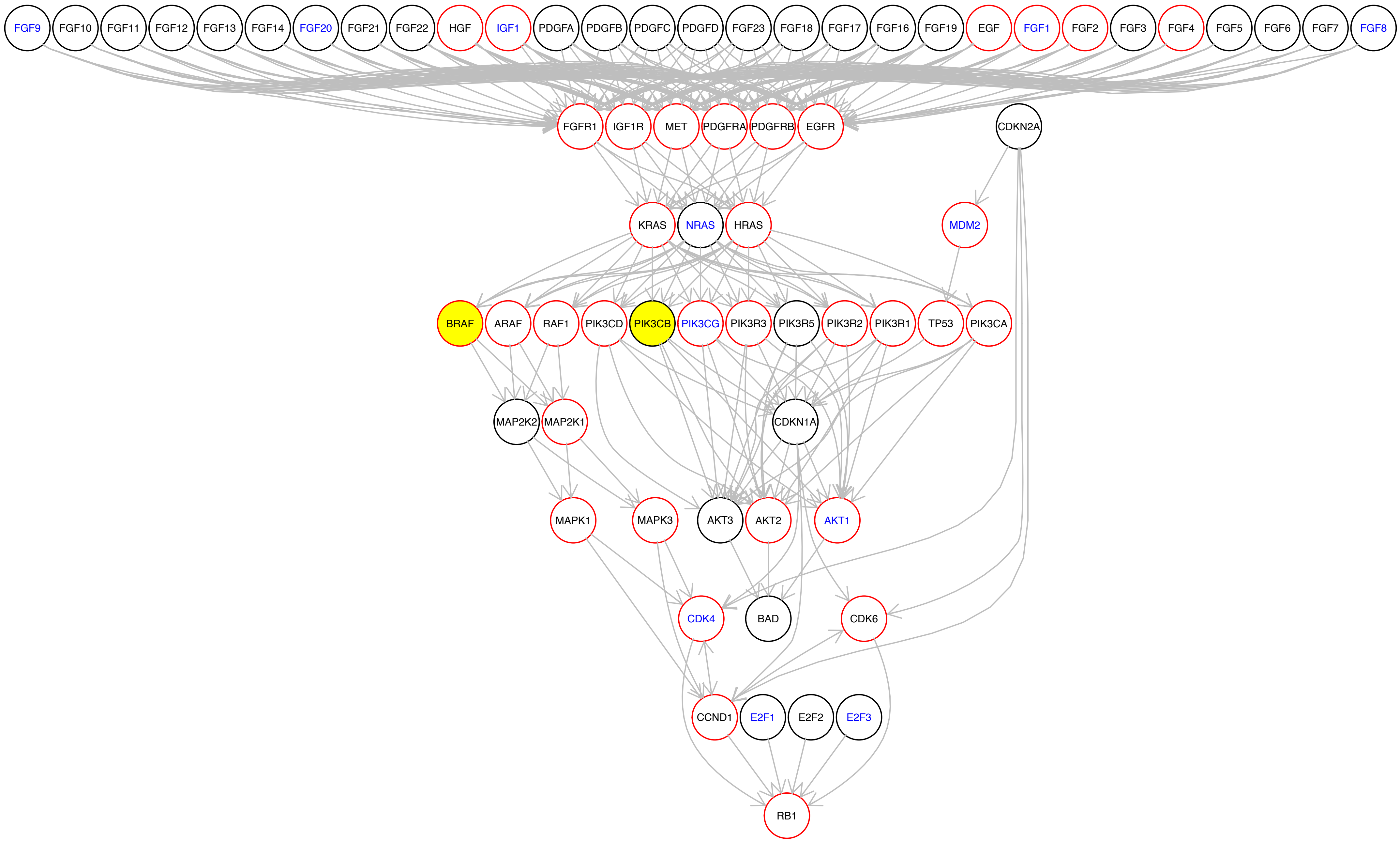

KEGG pathway = Melanoma : tumour = CHL1_SKIN : Yellow Fill = gene variant, Blue Text = expression−survival association, Red Boarder = drug

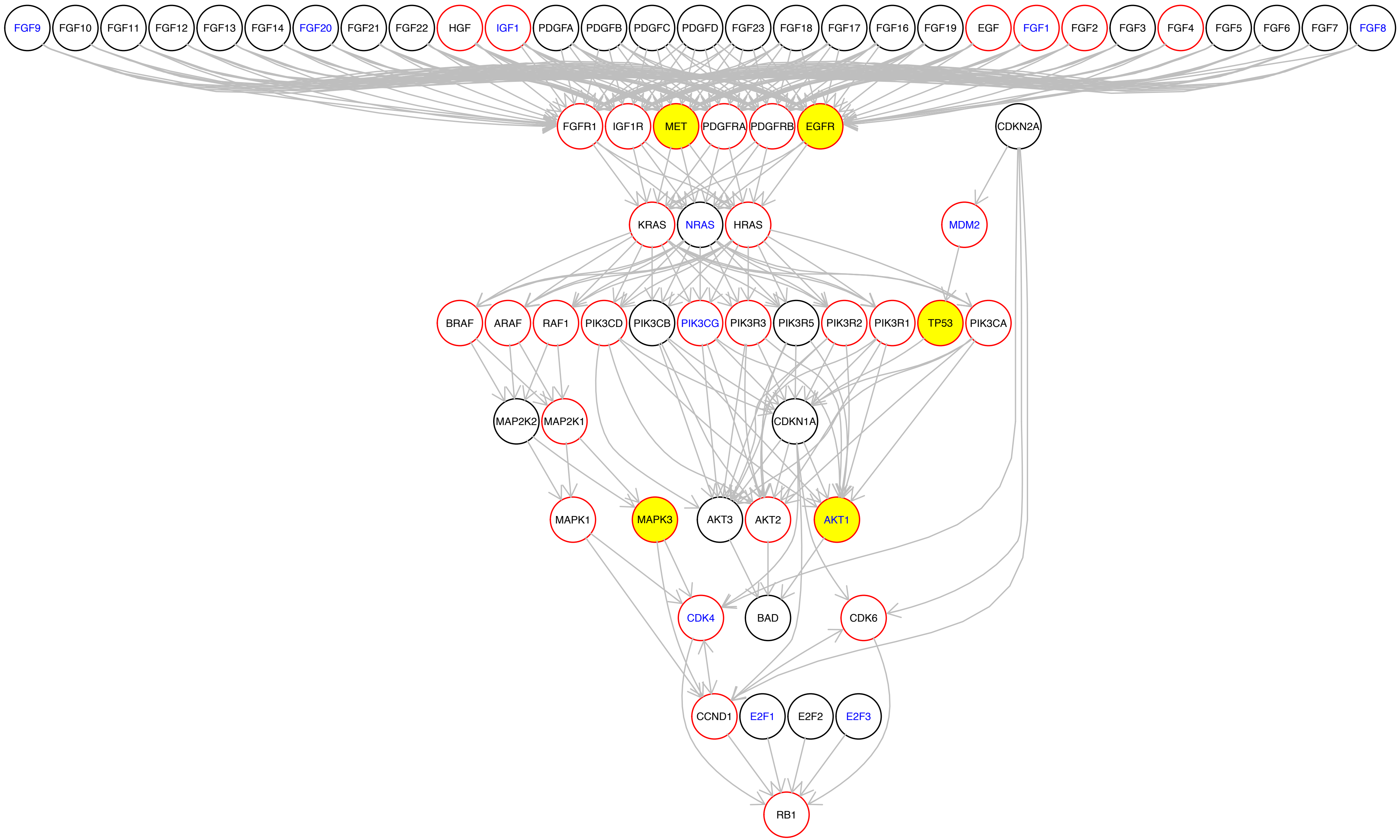

KEGG pathway = Melanoma : tumour = CJM_SKIN : Yellow Fill = gene variant, Blue Text = expression–survival association, Red Boarder = drug

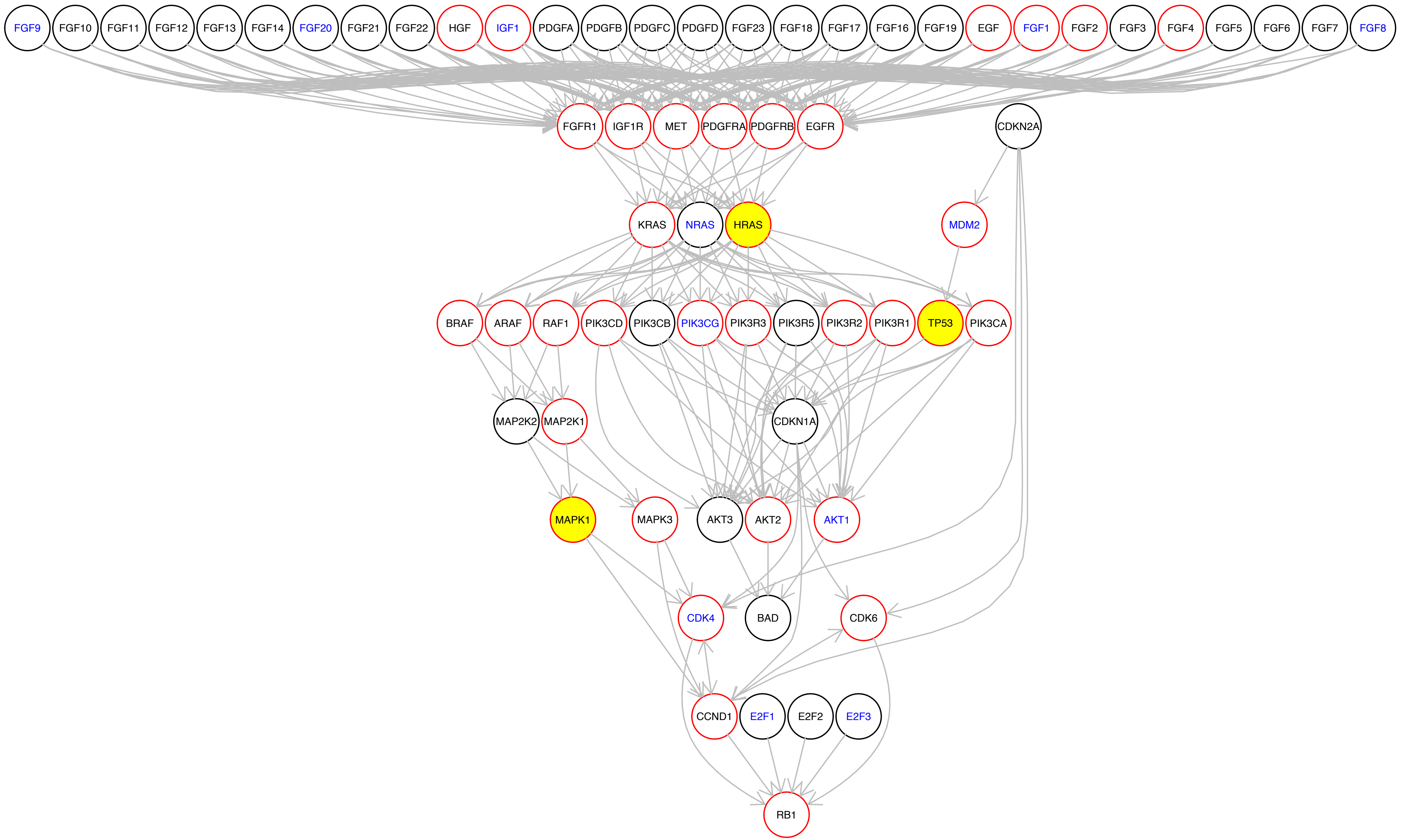

KEGG pathway = Melanoma : tumour = COLO679_SKIN : Yellow Fill = gene variant, Blue Text = expression−survival association, Red Boarder = drug

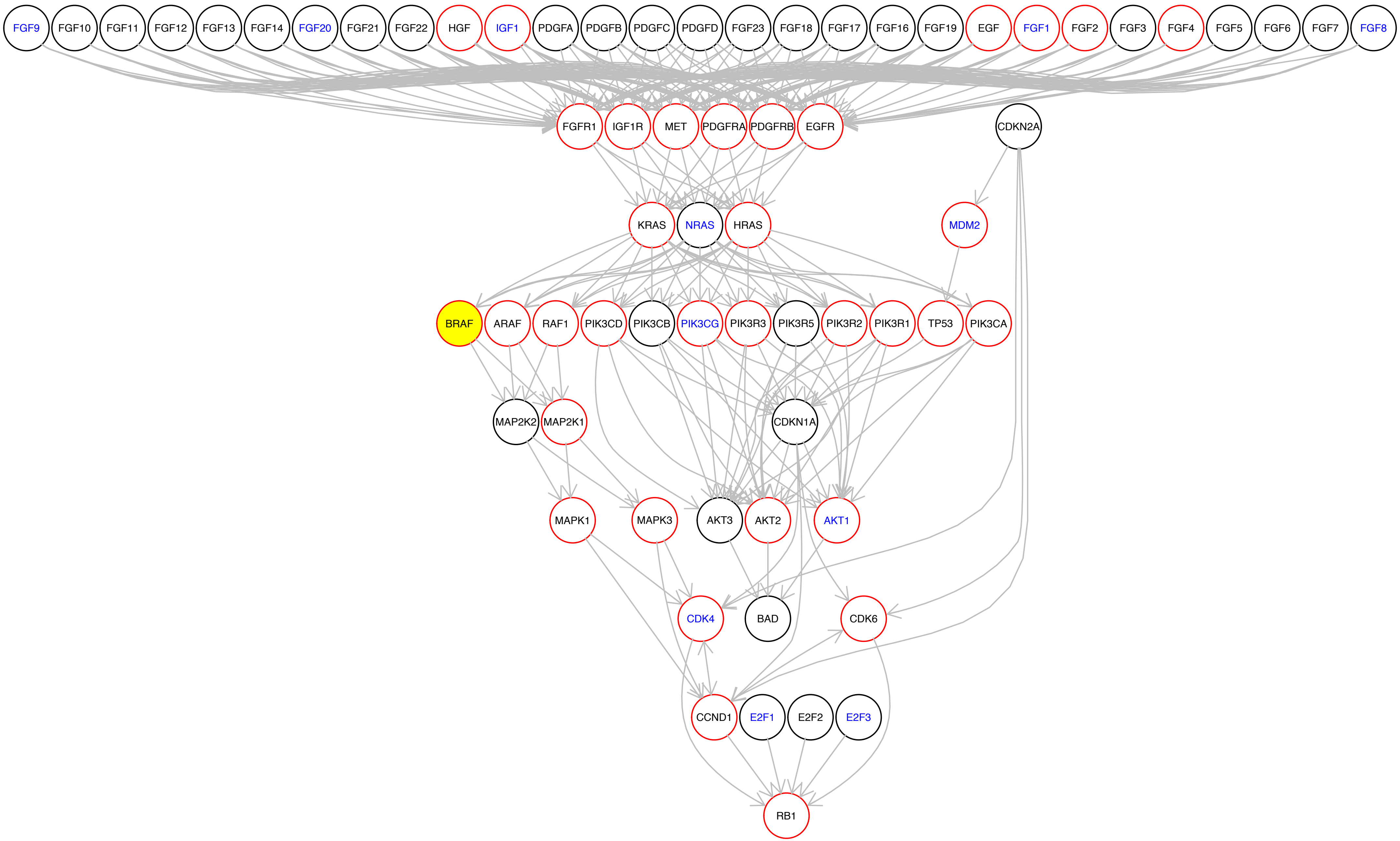

KEGG pathway = Melanoma : tumour = COLO741_SKIN : Yellow Fill = gene variant, Blue Text = expression−survival association, Red Boarder = drug

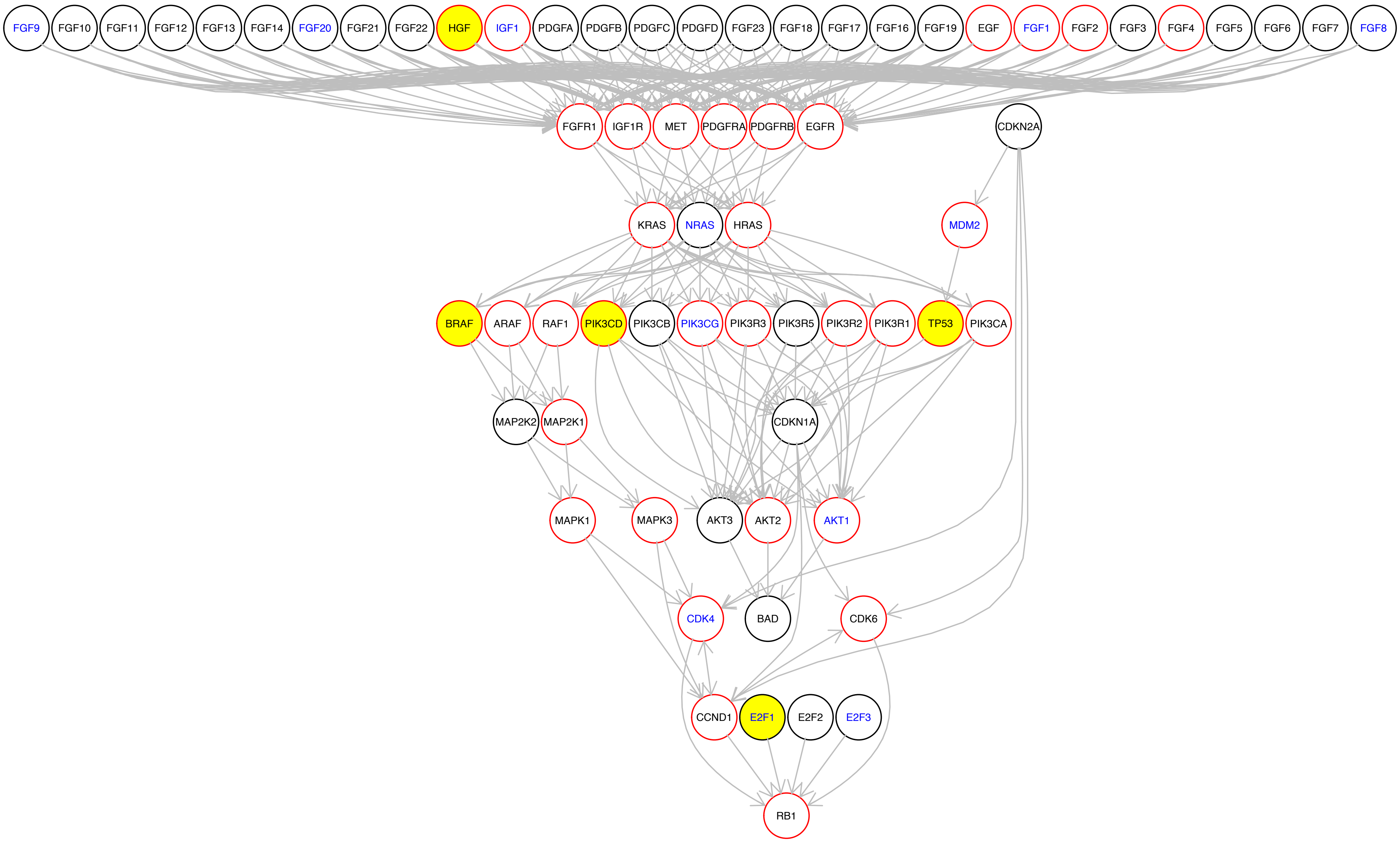

KEGG pathway = Melanoma : tumour = COLO783_SKIN : Yellow Fill = gene variant, Blue Text = expression−survival association, Red Boarder = drug

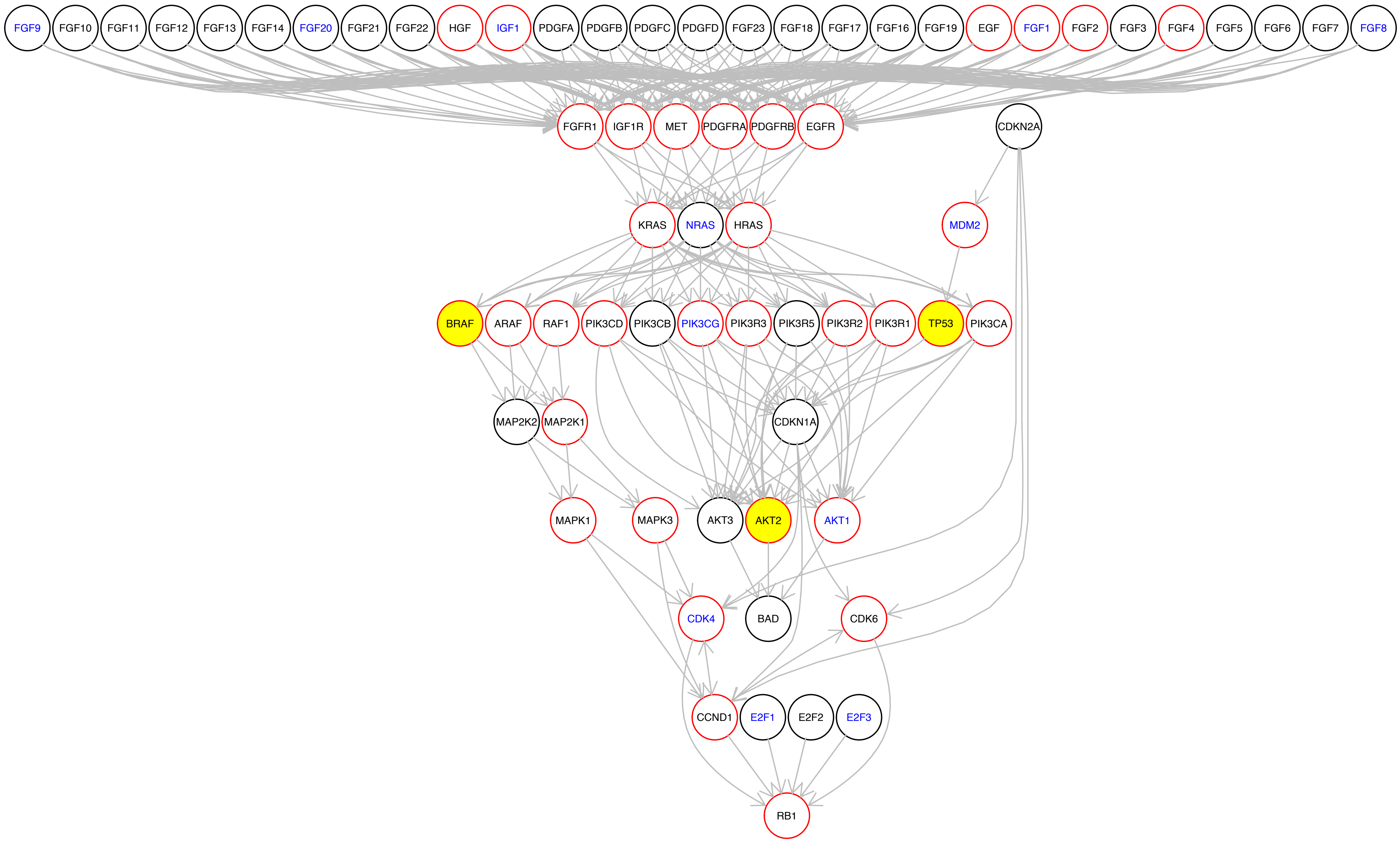

KEGG pathway = Melanoma : tumour = COLO792_SKIN : Yellow Fill = gene variant, Blue Text = expression-survival association, Red Boarder = drug

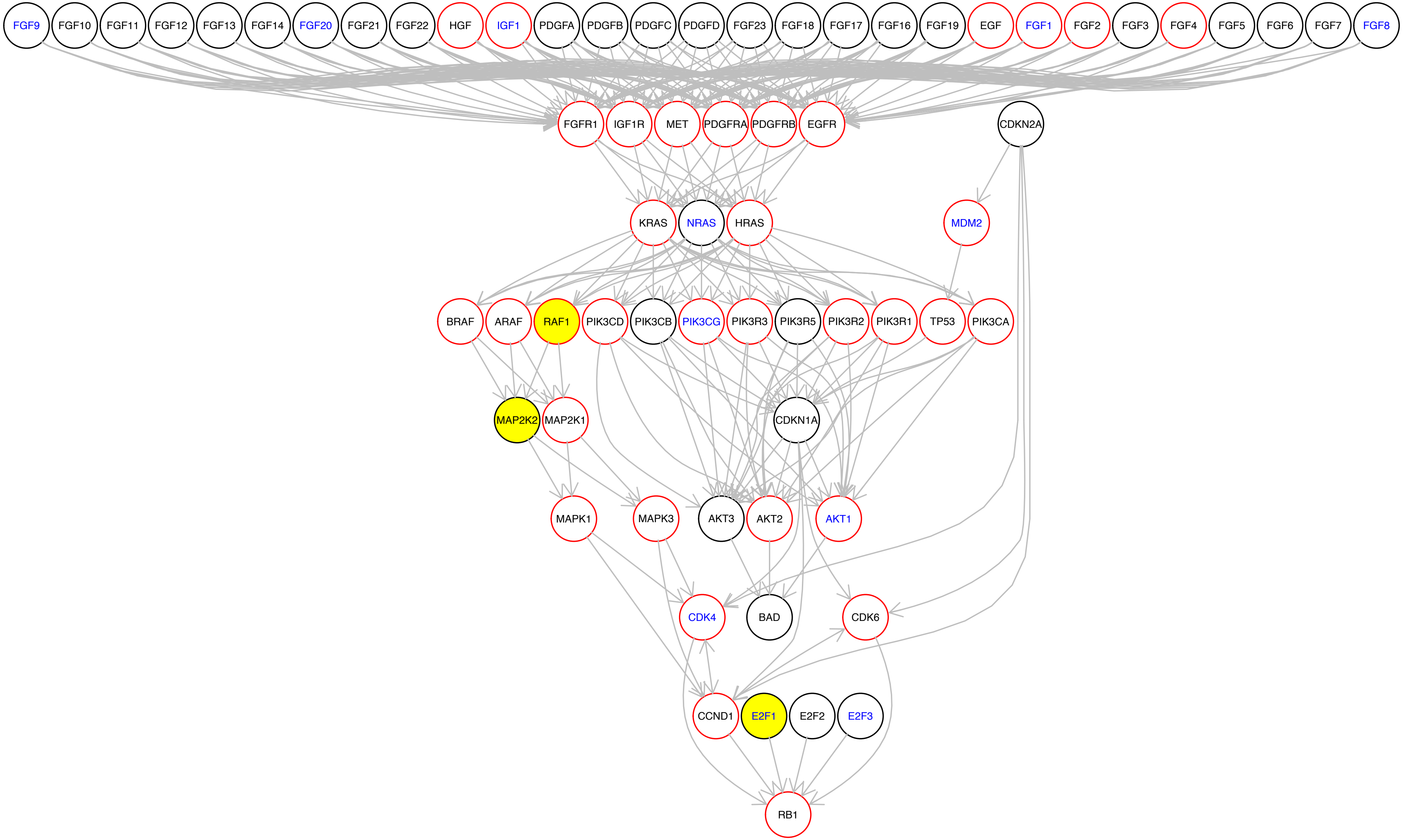

KEGG pathway = Melanoma : tumour = COLO818_SKIN : Yellow Fill = gene variant, Blue Text = expression−survival association, Red Boarder = drug

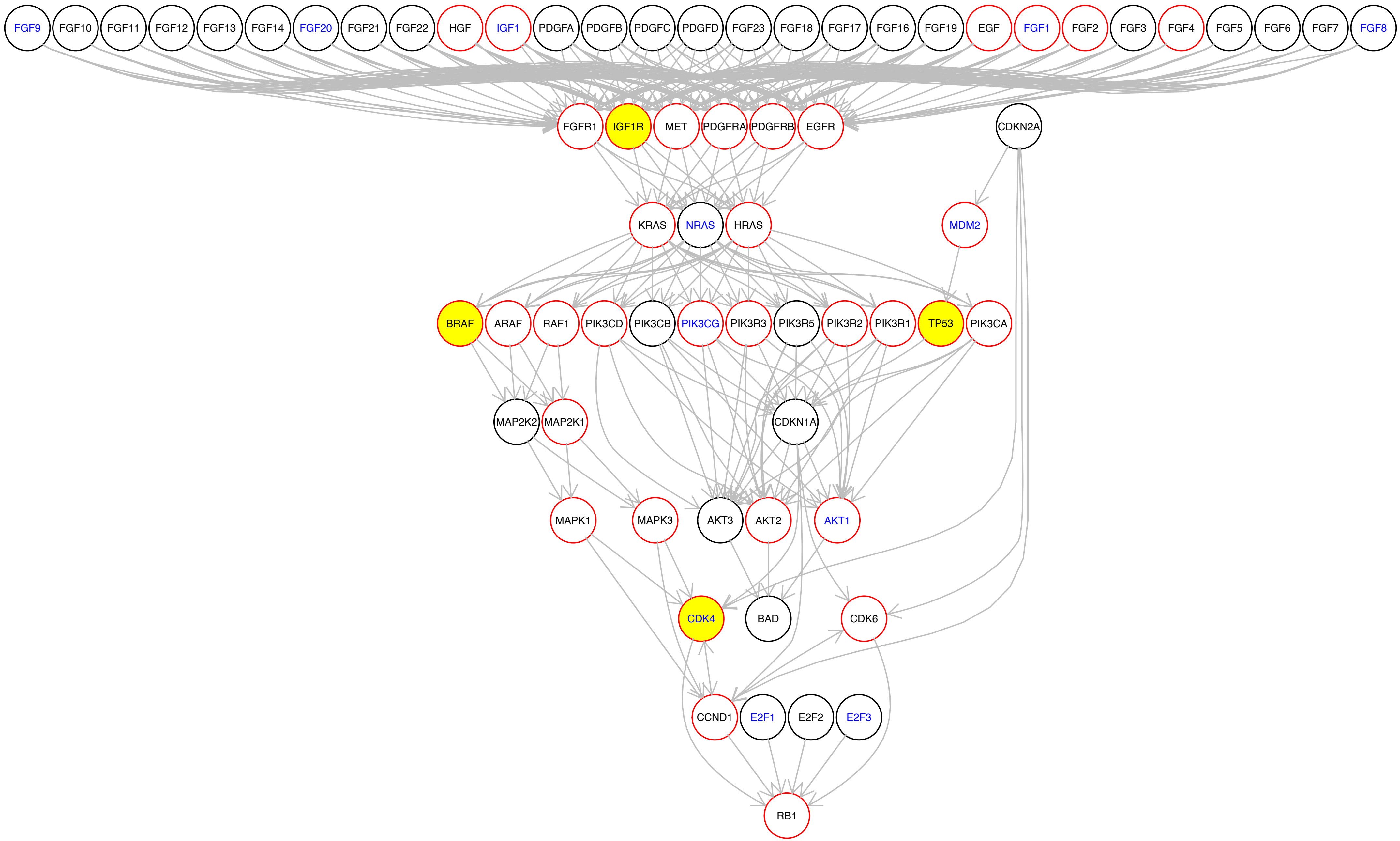

KEGG pathway = Melanoma : tumour = COLO829_SKIN : Yellow Fill = gene variant, Blue Text = expression−survival association, Red Boarder = drug

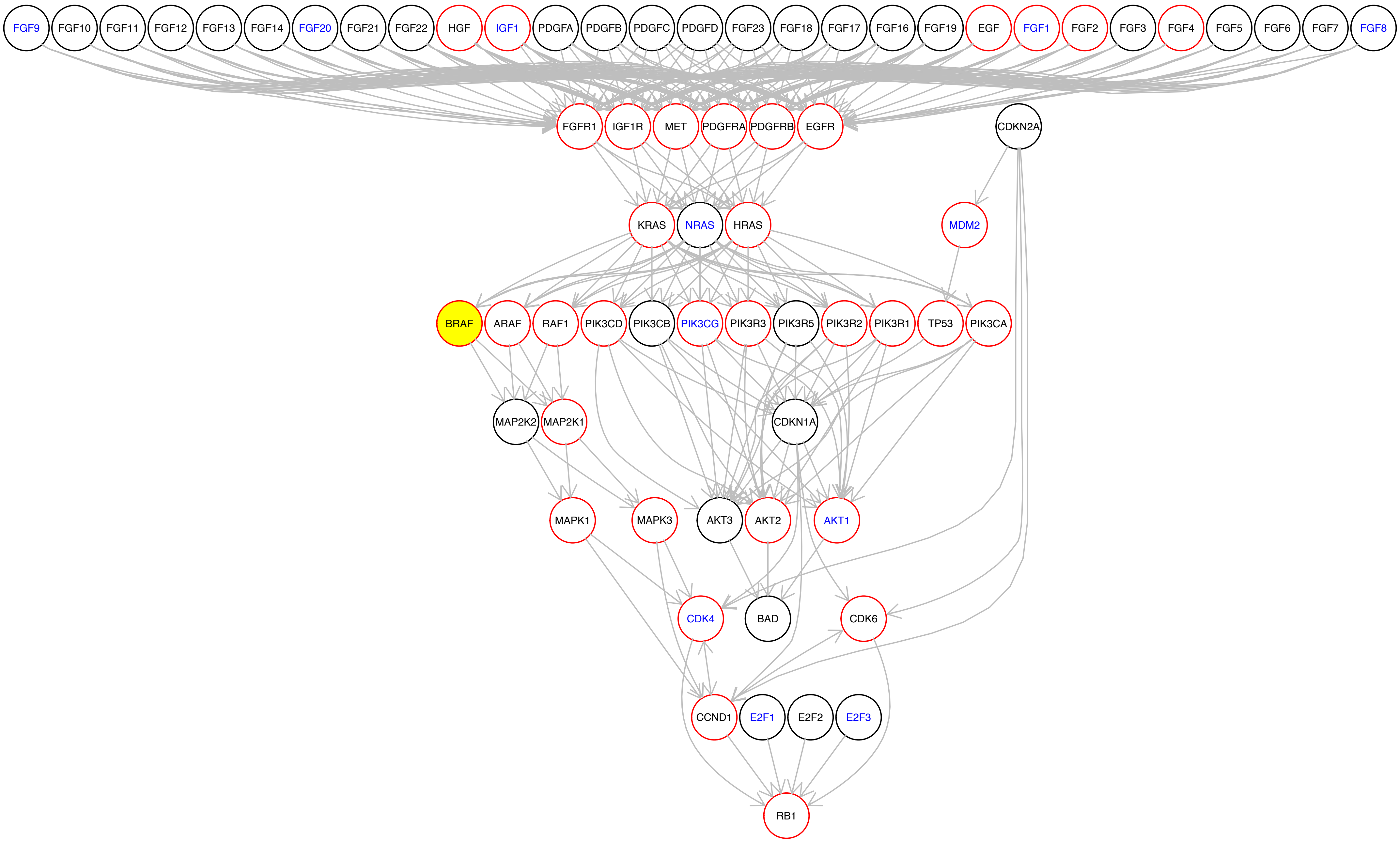

KEGG pathway = Melanoma : tumour = G361_SKIN : Yellow Fill = gene variant, Blue Text = expression−survival association, Red Boarder = drug

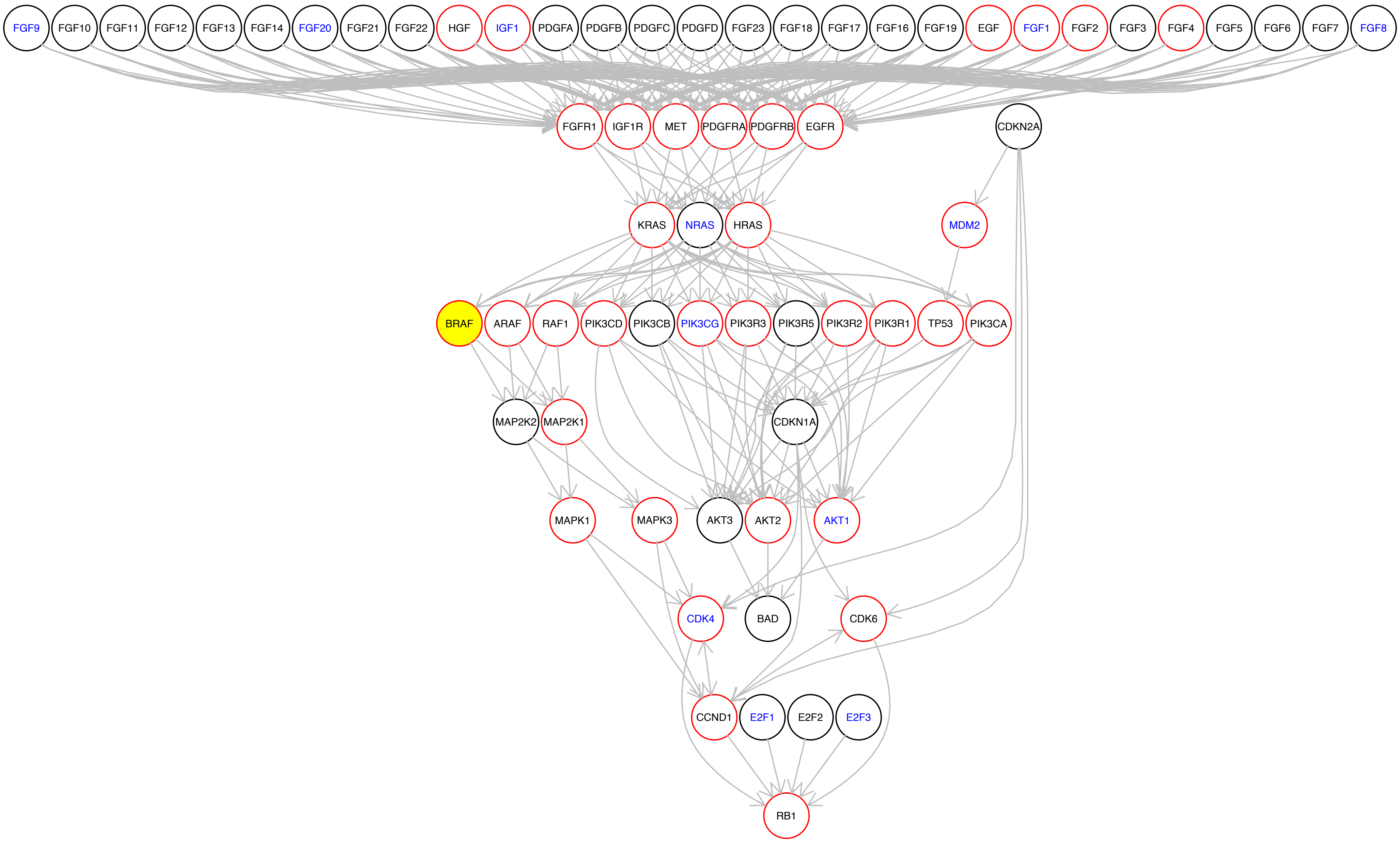

KEGG pathway = Melanoma : tumour = HS600T_SKIN : Yellow Fill = gene variant, Blue Text = expression−survival association, Red Boarder = drug

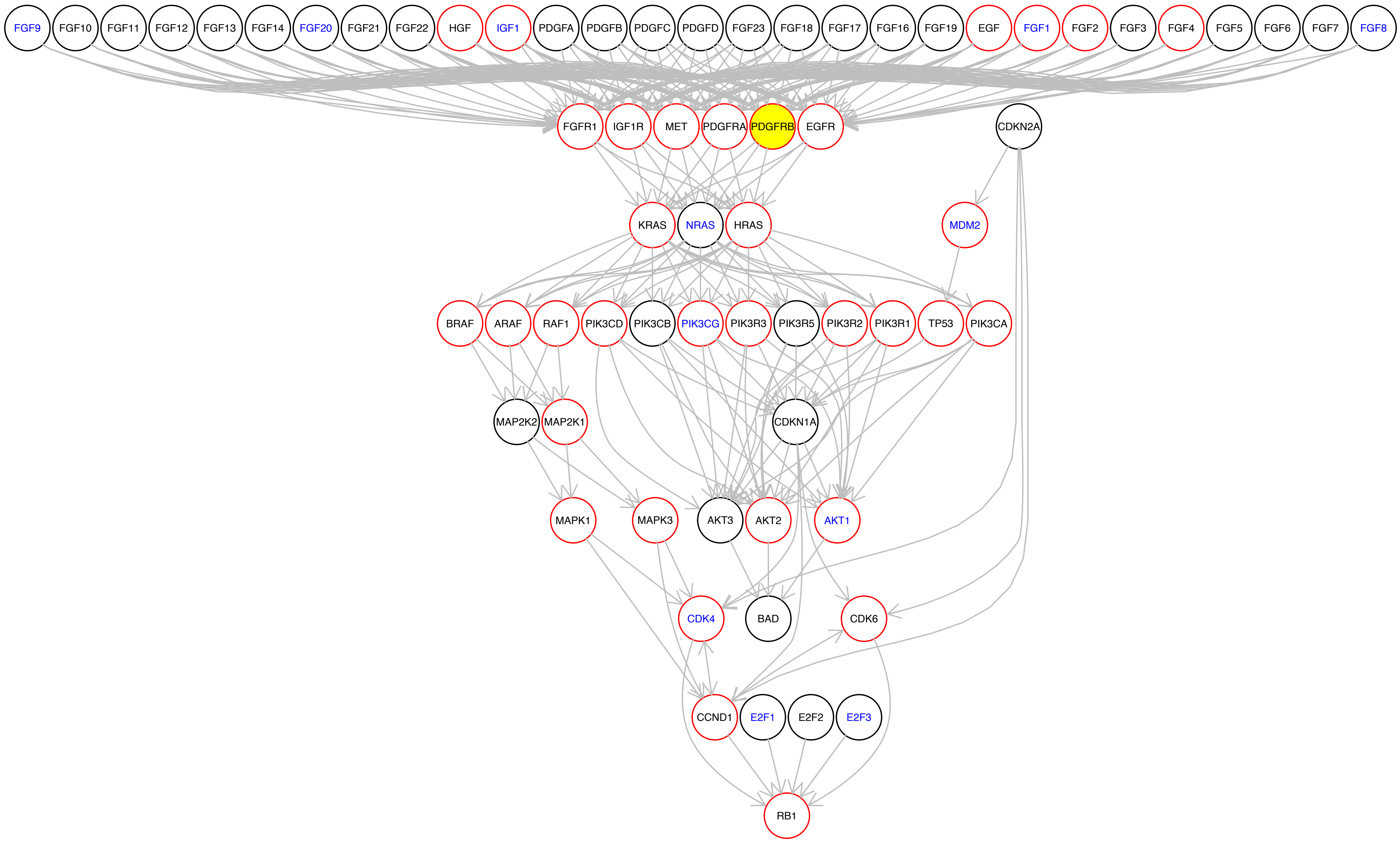

KEGG pathway = Melanoma : tumour = HS688AT_SKIN : Yellow Fill = gene variant, Blue Text = expression−survival association, Red Boarder = drug

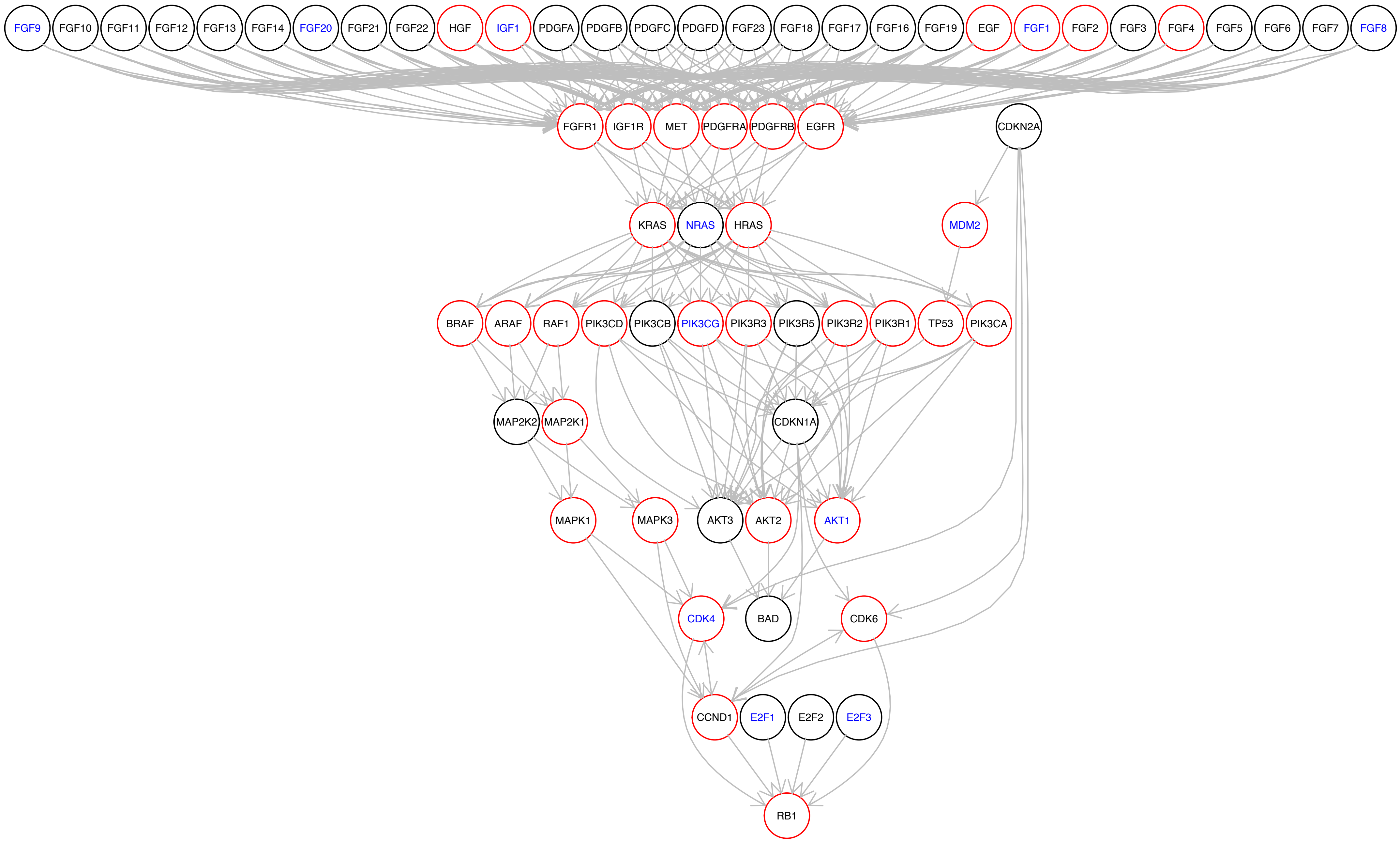

KEGG pathway = Melanoma :   tumour = HS695T_SKIN :   Yellow Fill = gene variant, Blue Text = expression−survival association, Red Boarder = drug

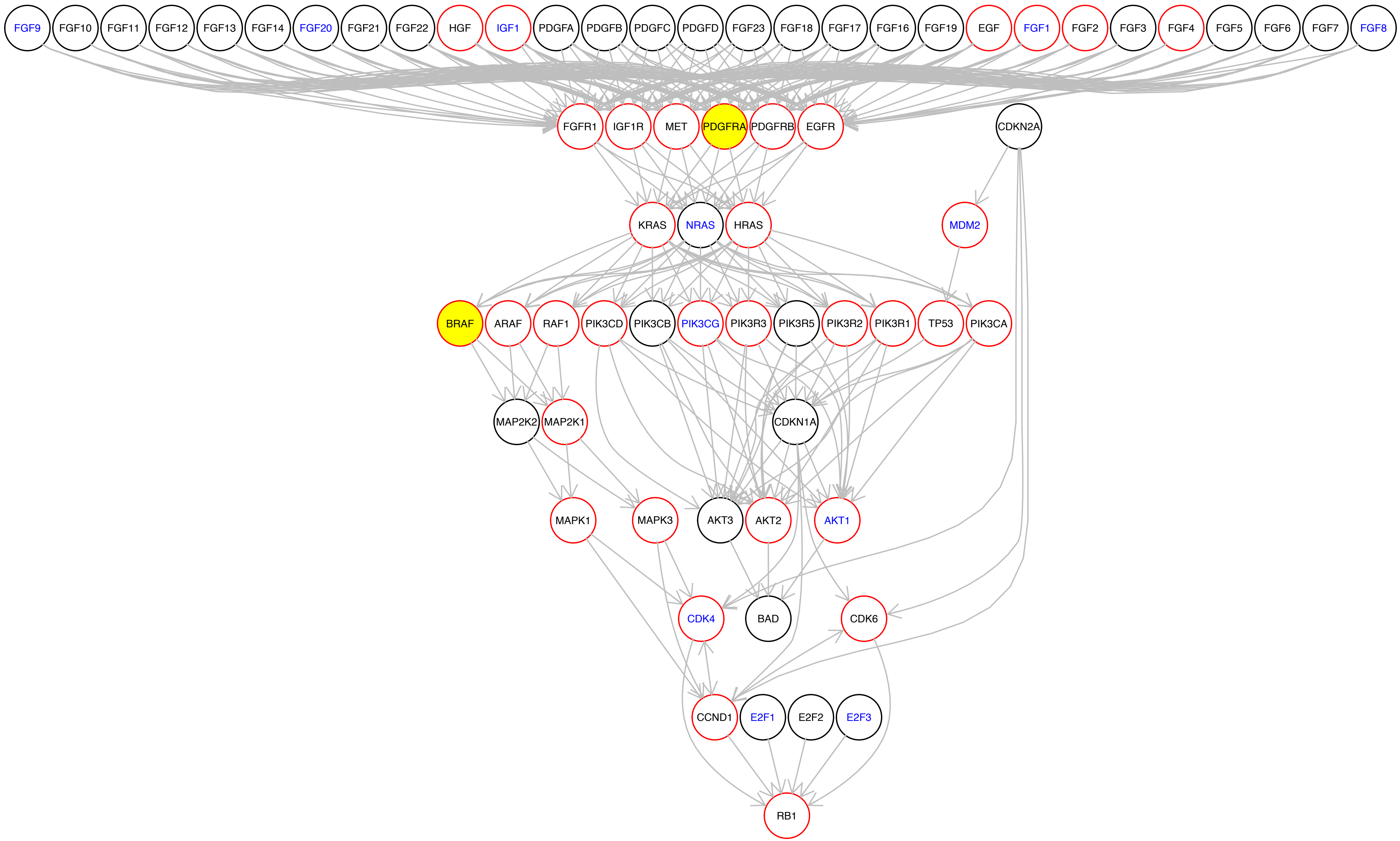

KEGG pathway = Melanoma : tumour = HS834T_SKIN : Yellow Fill = gene variant, Blue Text = expression−survival association, Red Boarder = drug

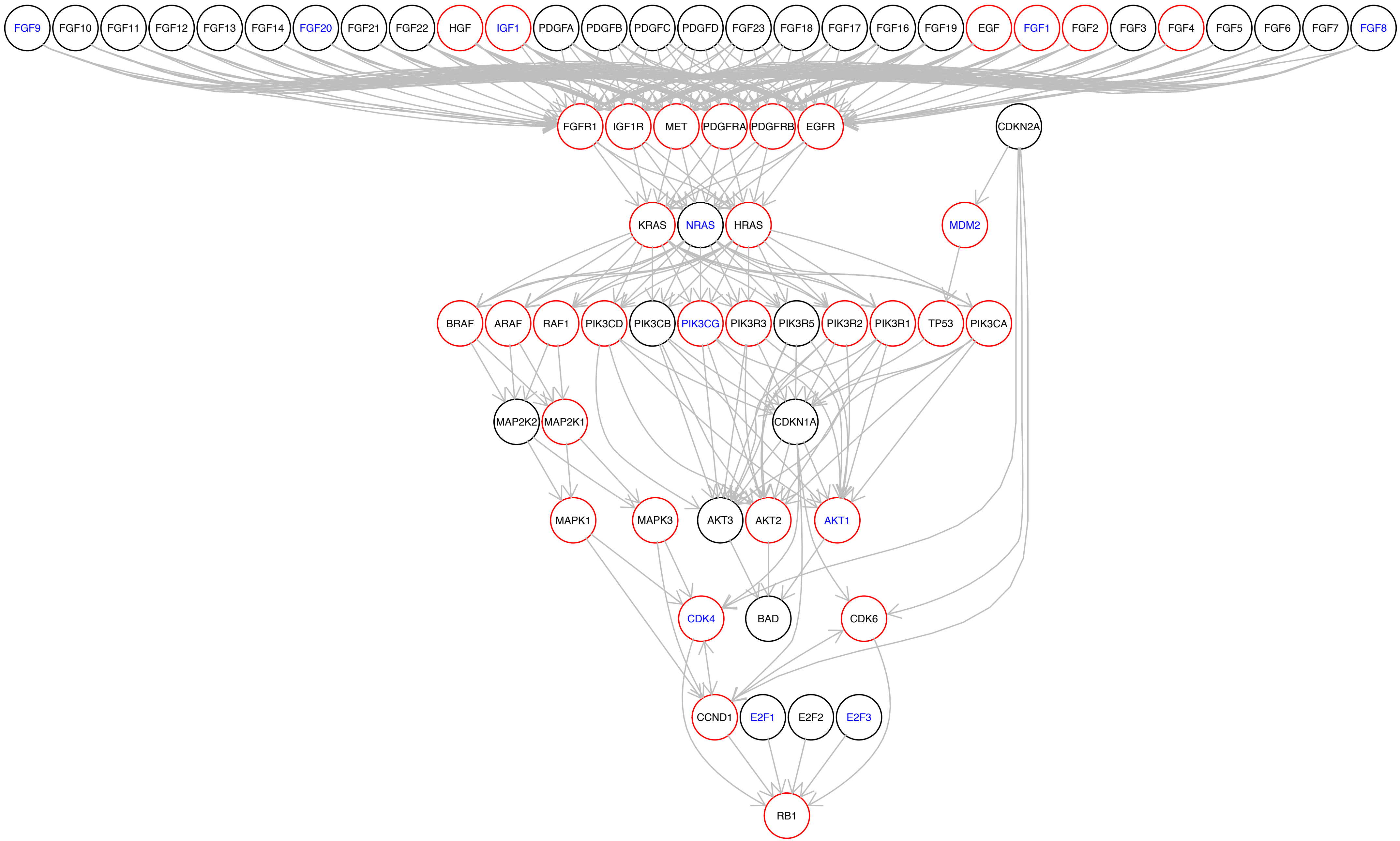

KEGG pathway = Melanoma : tumour = HS839T_SKIN : Yellow Fill = gene variant, Blue Text = expression–survival association, Red Boarder = drug

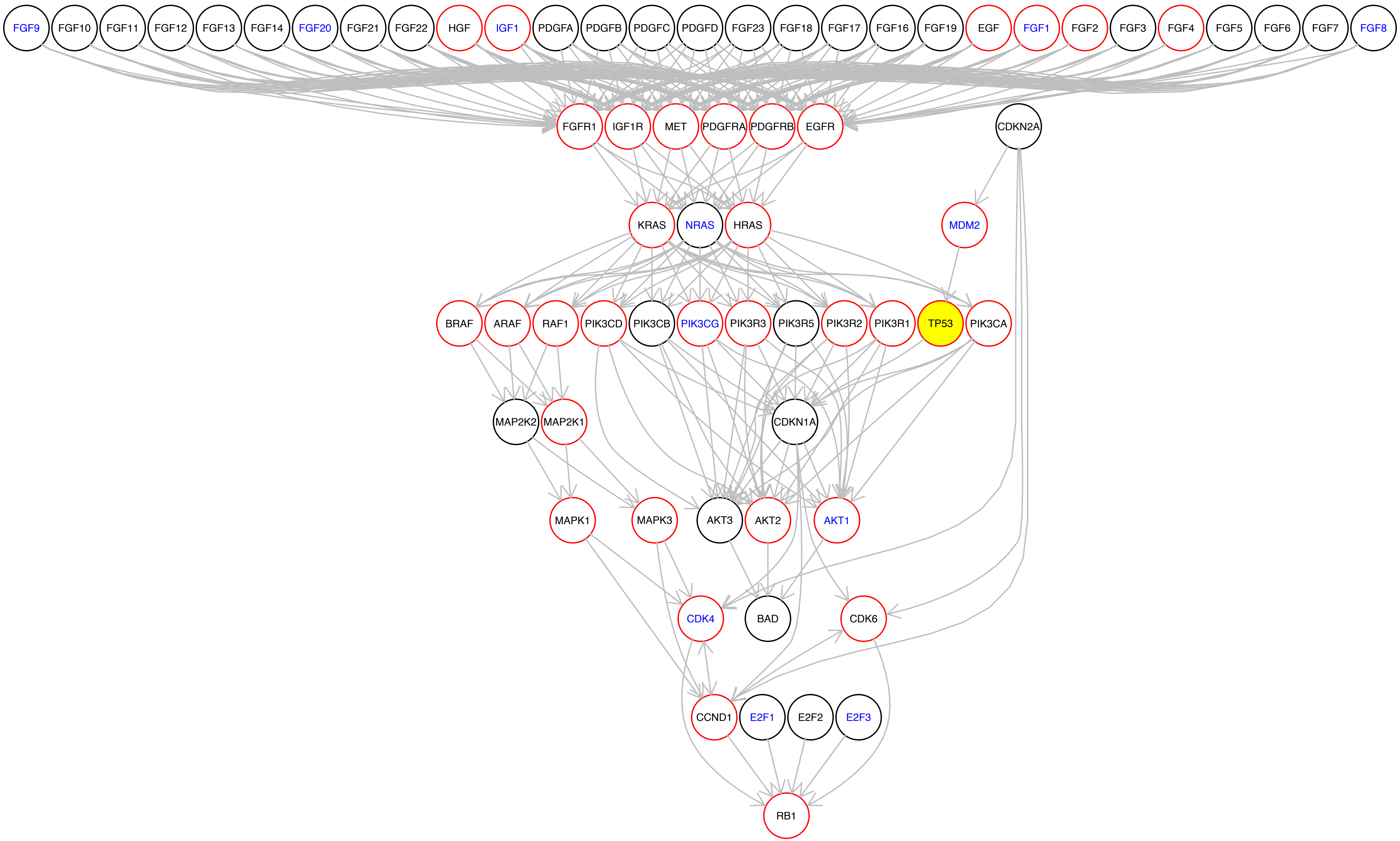

KEGG pathway = Melanoma : tumour = HS852T_SKIN : Yellow Fill = gene variant, Blue Text = expression–survival association, Red Boarder = drug

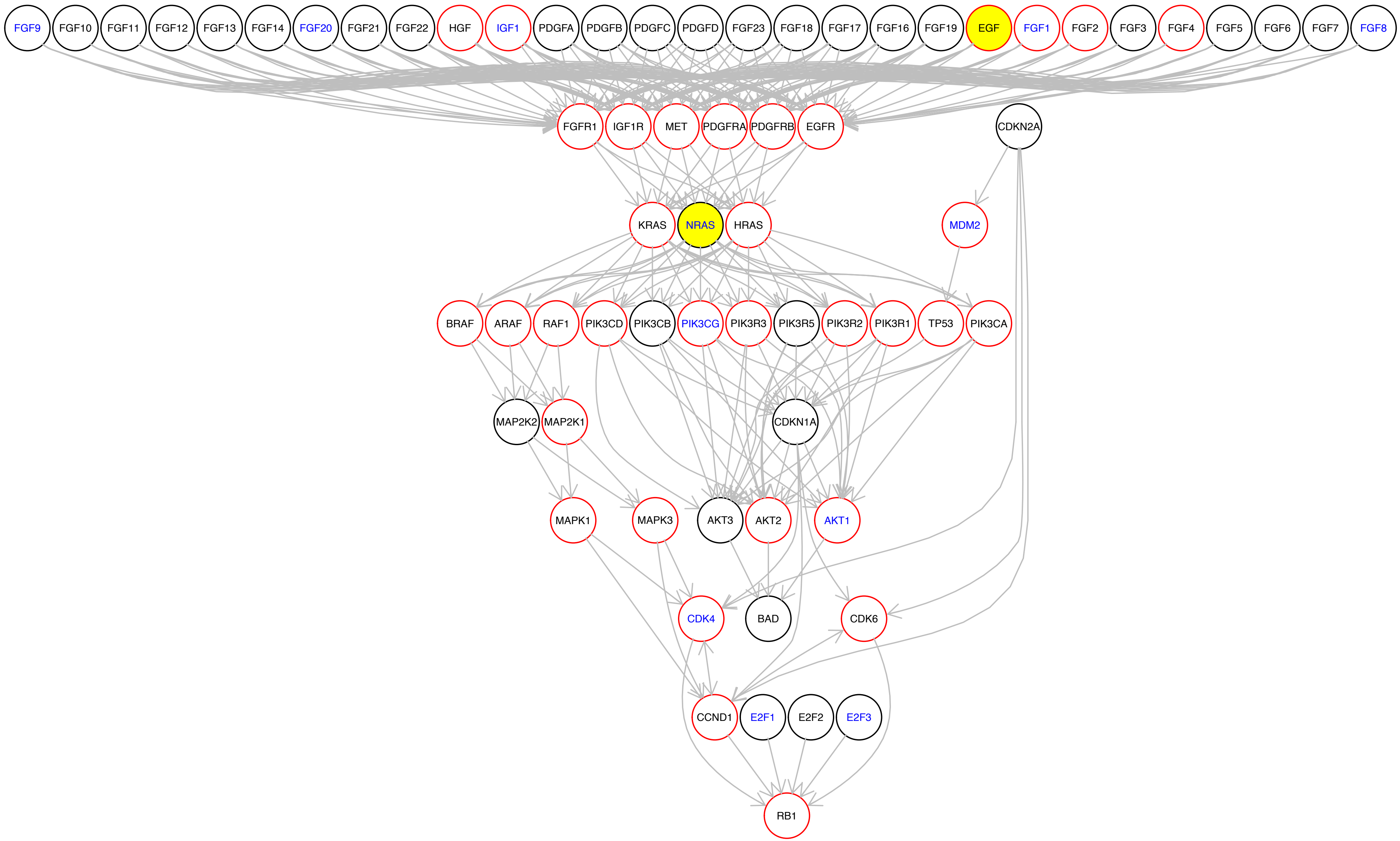

KEGG pathway = Melanoma : tumour = HS895T_SKIN : Yellow Fill = gene variant, Blue Text = expression−survival association, Red Boarder = drug

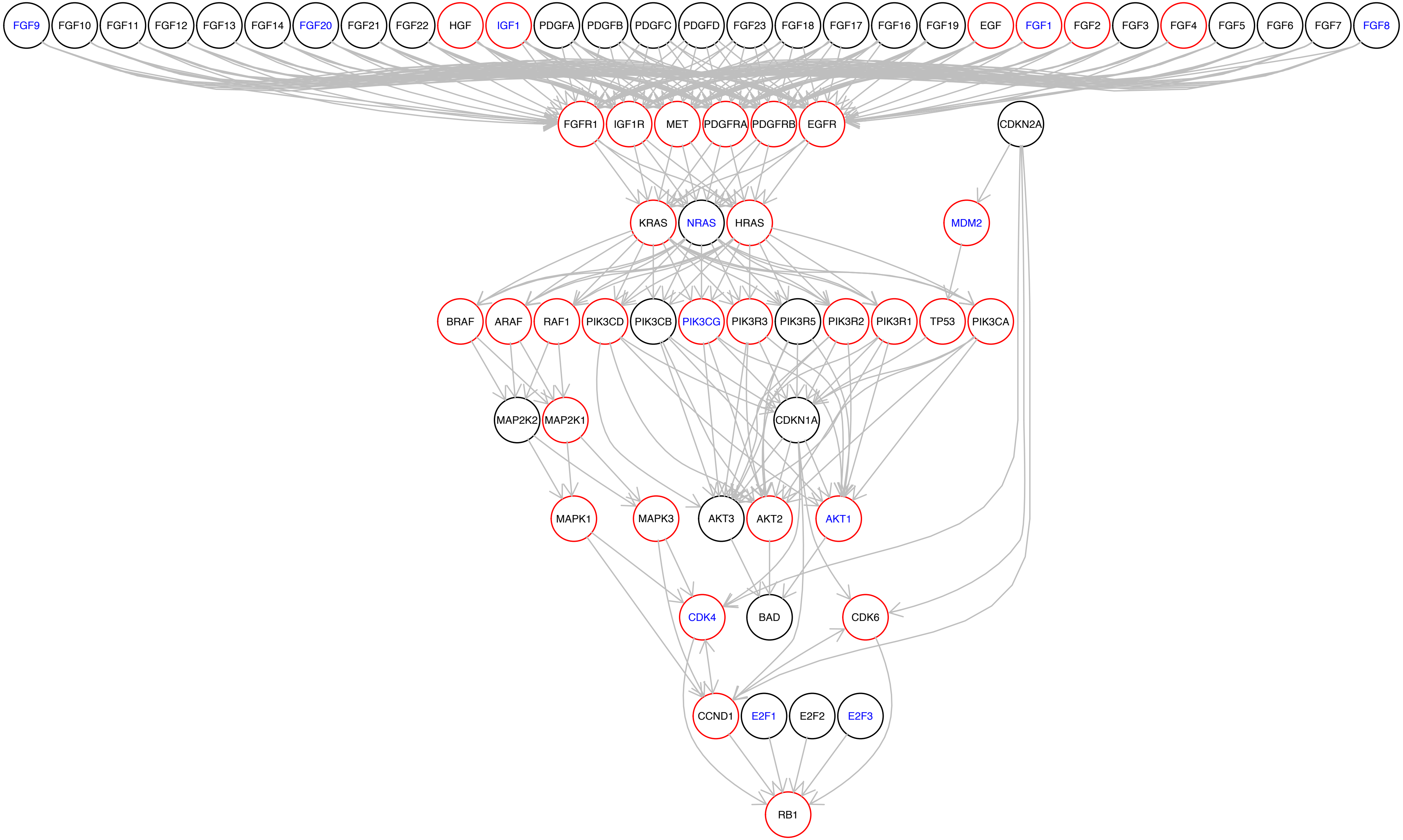

KEGG pathway = Melanoma : tumour = HS934T_SKIN : Yellow Fill = gene variant, Blue Text = expression−survival association, Red Boarder = drug

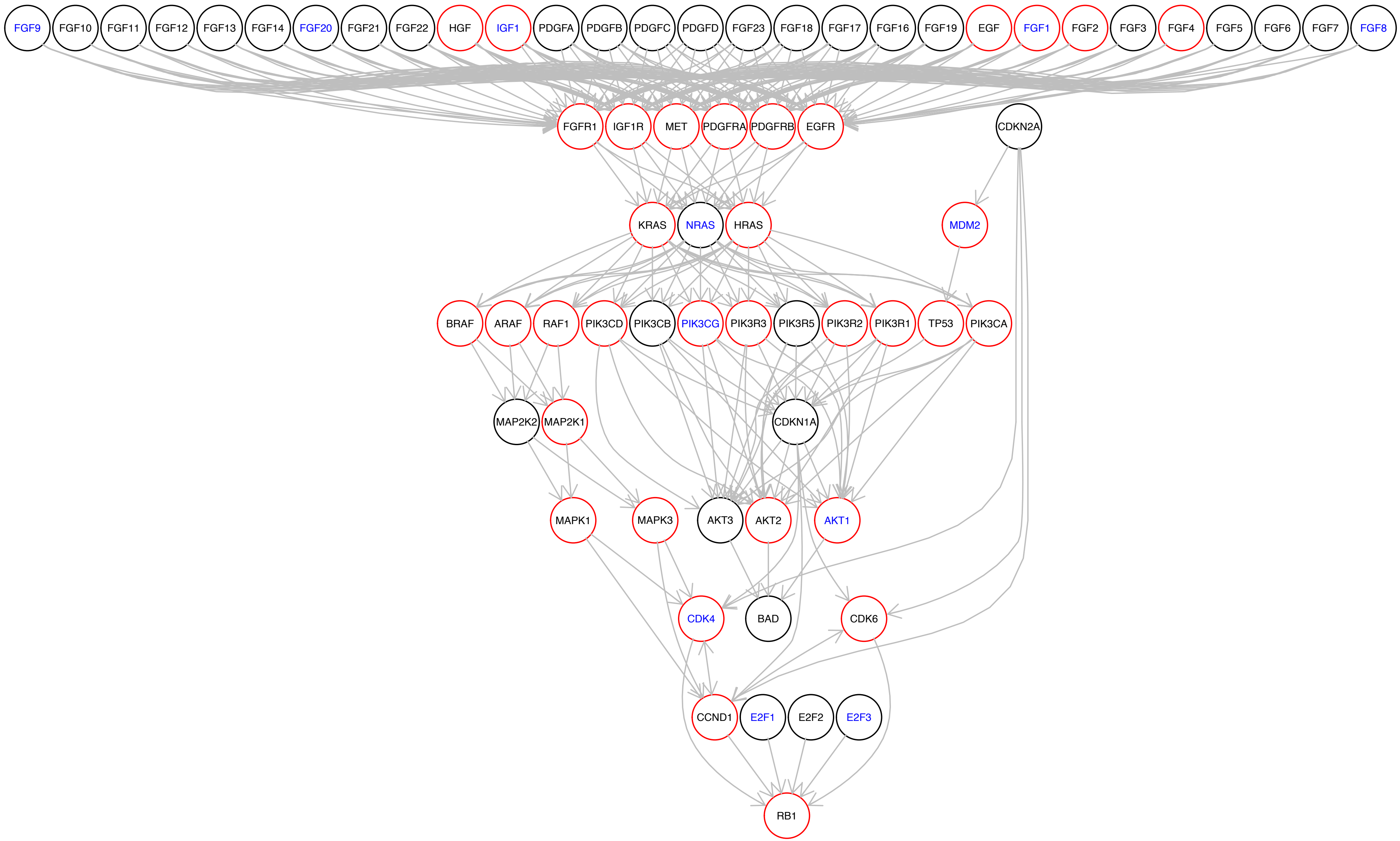

KEGG pathway = Melanoma : tumour = HS936T_SKIN : Yellow Fill = gene variant, Blue Text = expression−survival association, Red Boarder = drug

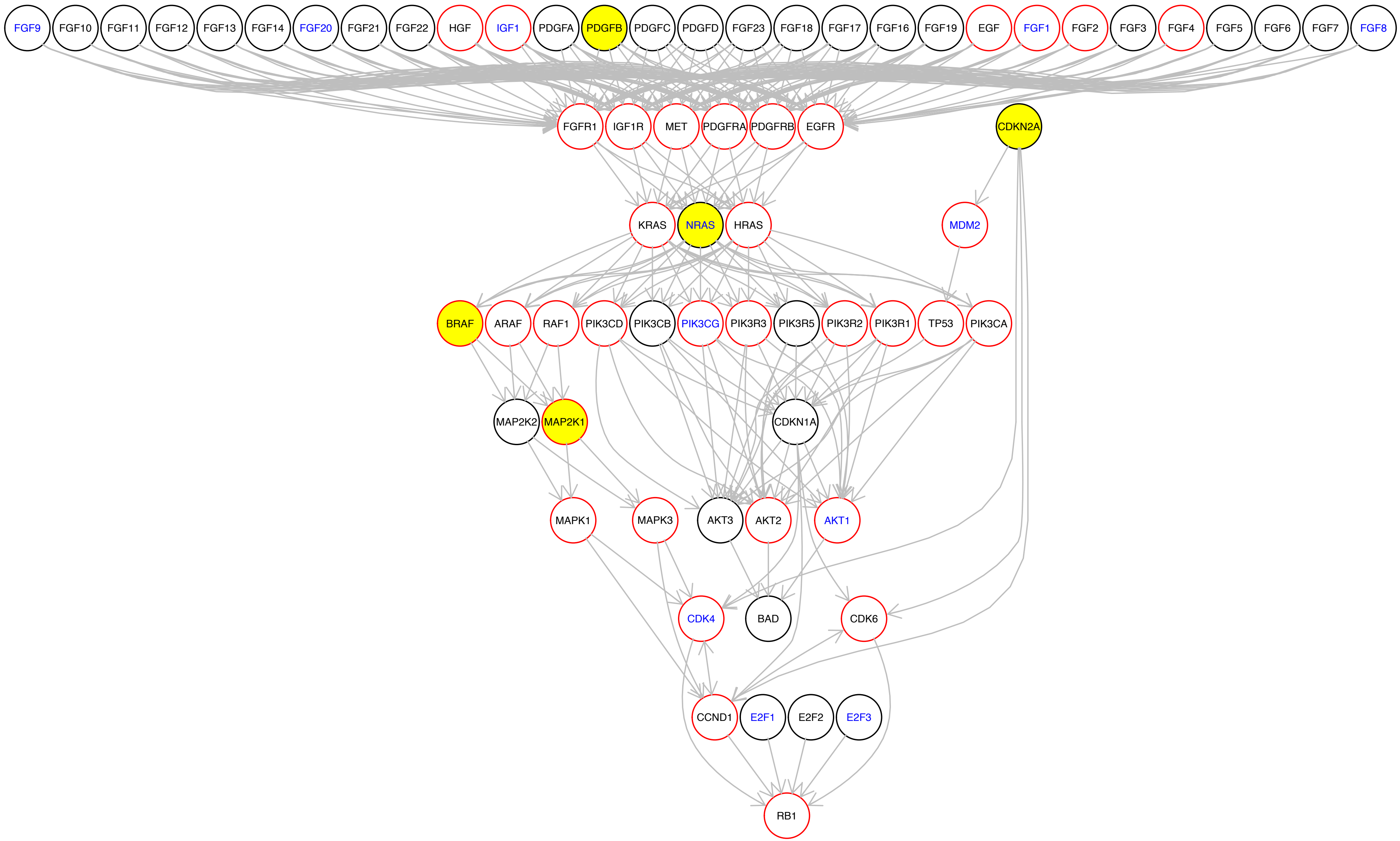

KEGG pathway = Melanoma :   tumour = HS940T_SKIN :   Yellow Fill = gene variant, Blue Text = expression–survival association, Red Boarder = drug

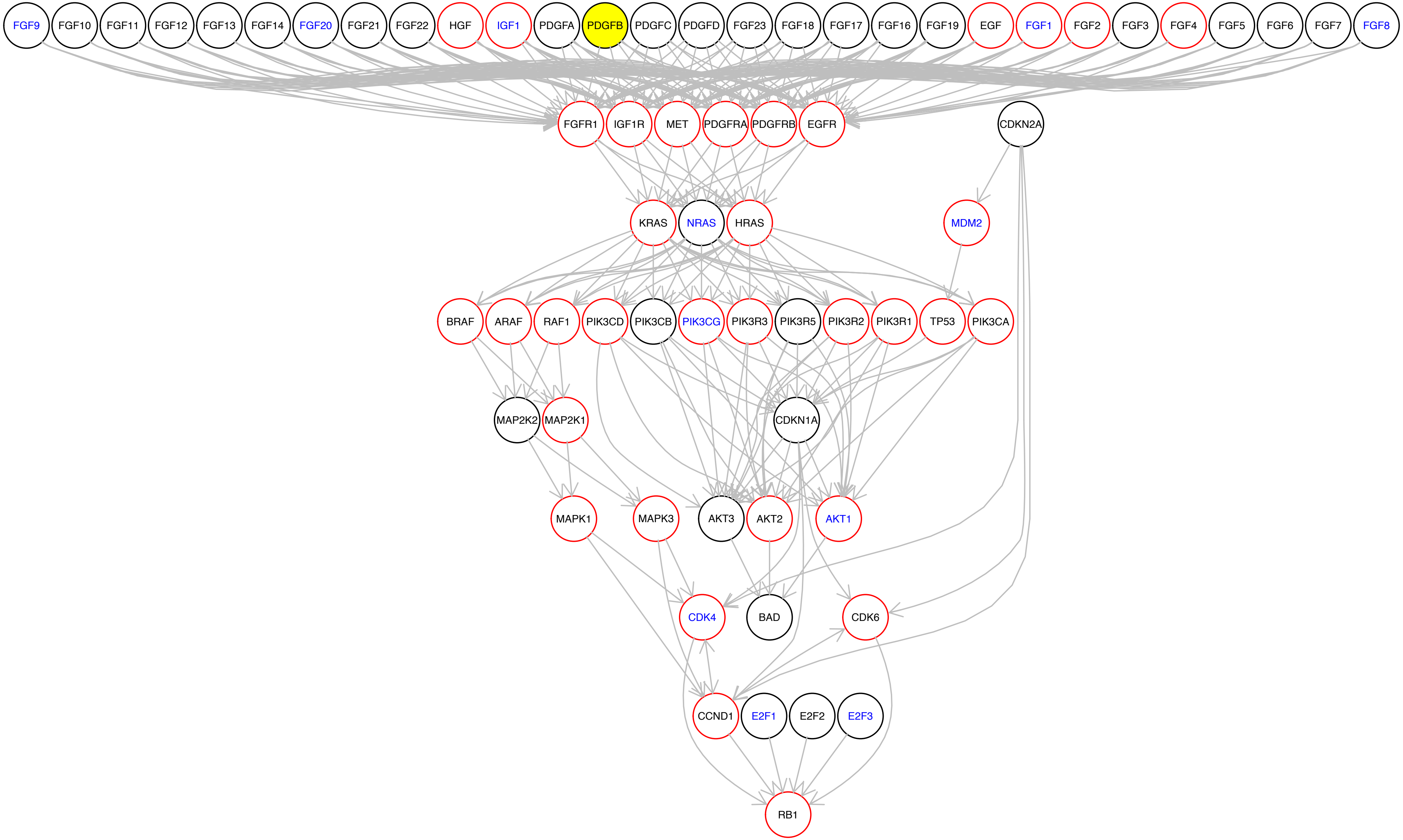

KEGG pathway = Melanoma :   tumour = HS944T_SKIN :   Yellow Fill = gene variant, Blue Text = expression−survival association, Red Boarder = drug

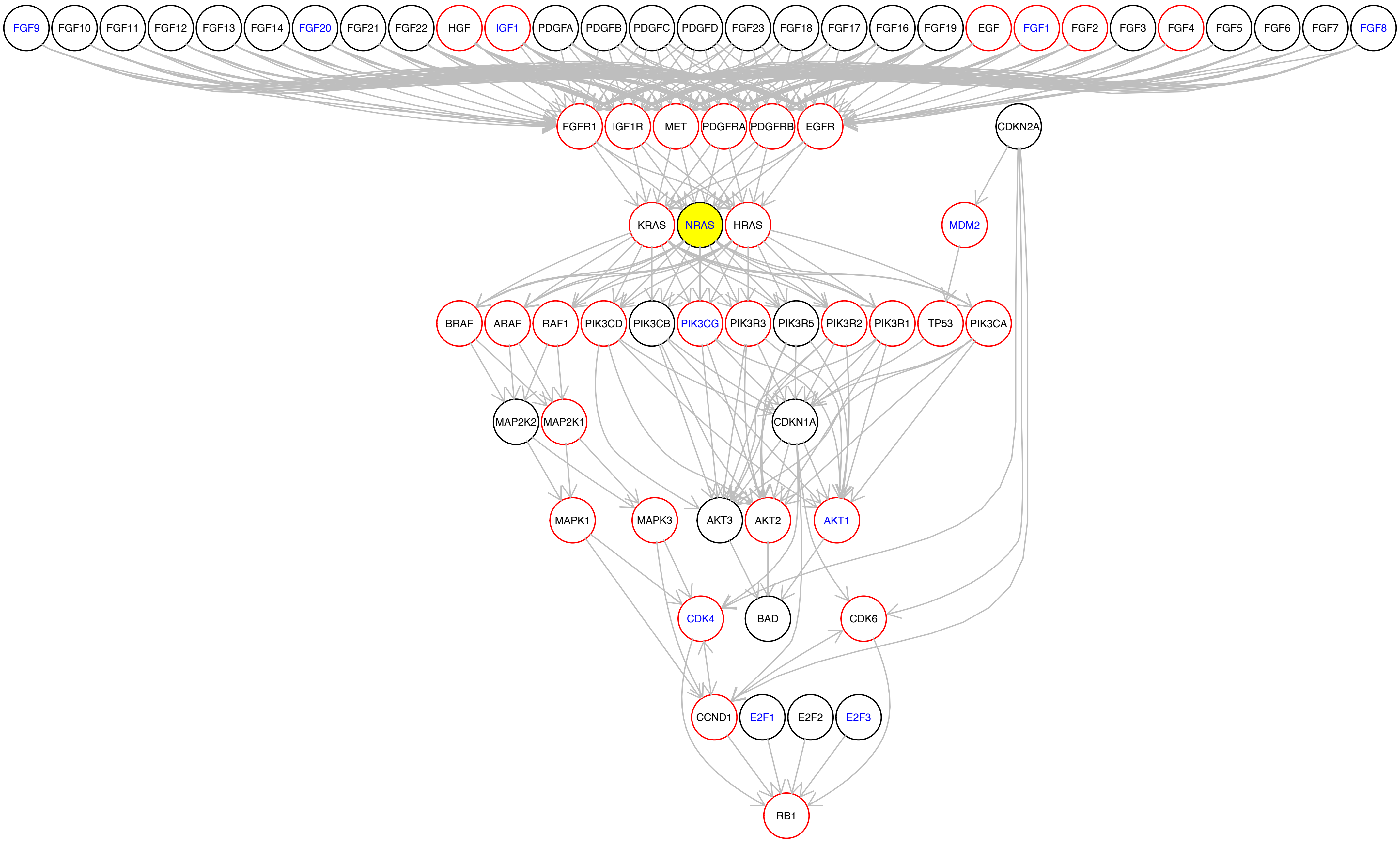

KEGG pathway = Melanoma : tumour = HT144_SKIN : Yellow Fill = gene variant, Blue Text = expression−survival association, Red Boarder = drug

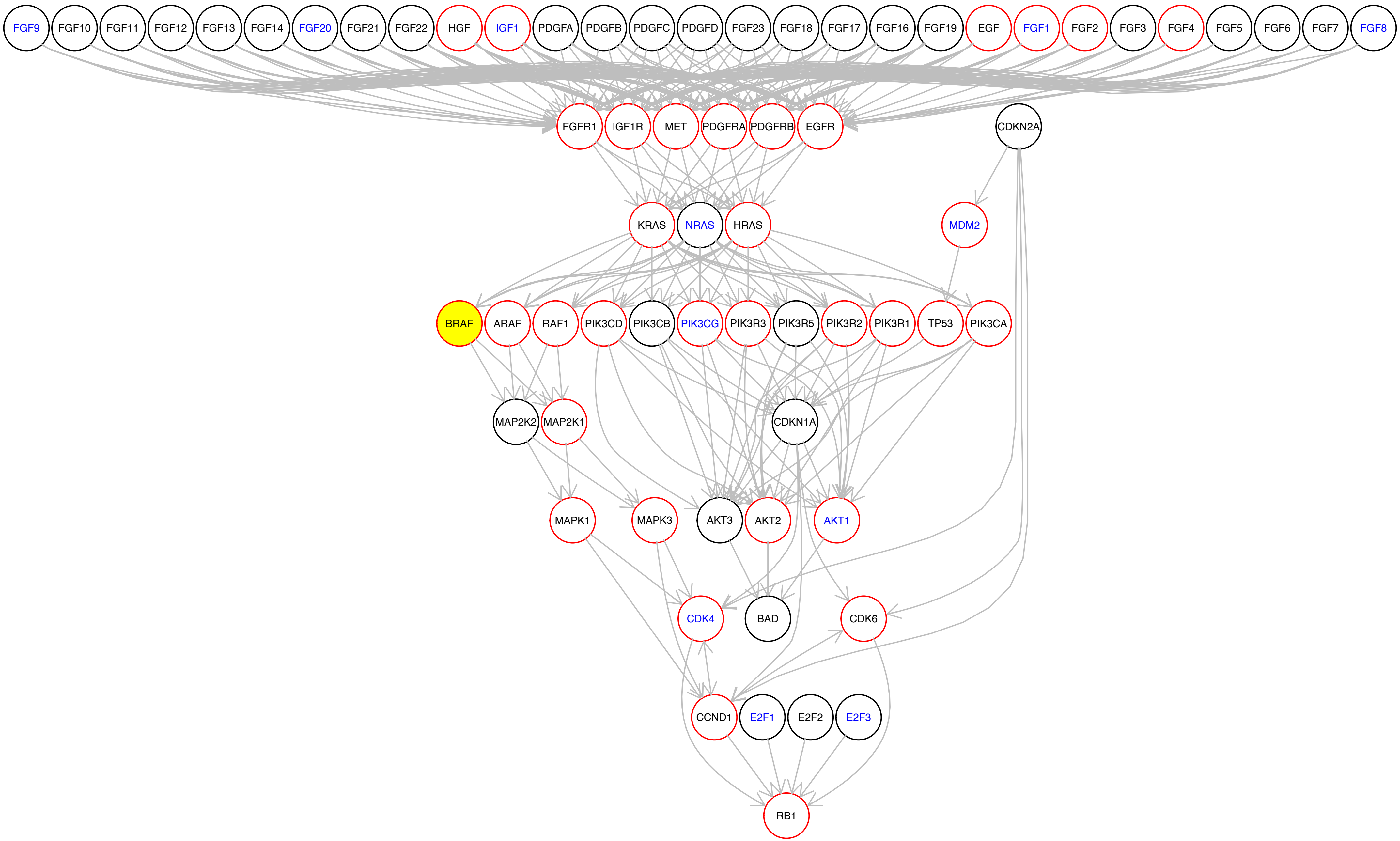

KEGG pathway = Melanoma : tumour = IGR37_SKIN : Yellow Fill = gene variant, Blue Text = expression−survival association, Red Boarder = drug

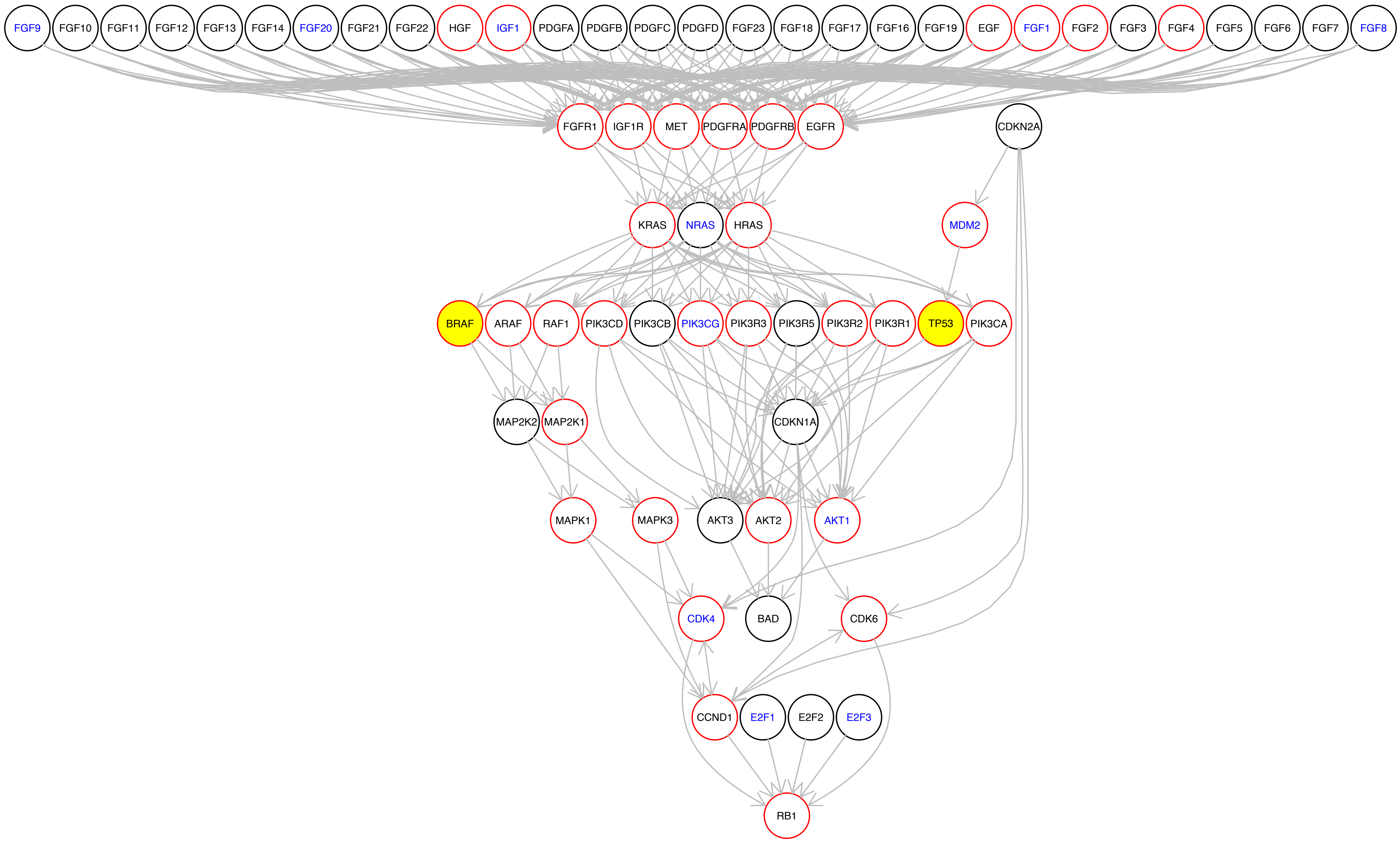

KEGG pathway = Melanoma : tumour = IGR39_SKIN : Yellow Fill = gene variant, Blue Text = expression−survival association, Red Boarder = drug

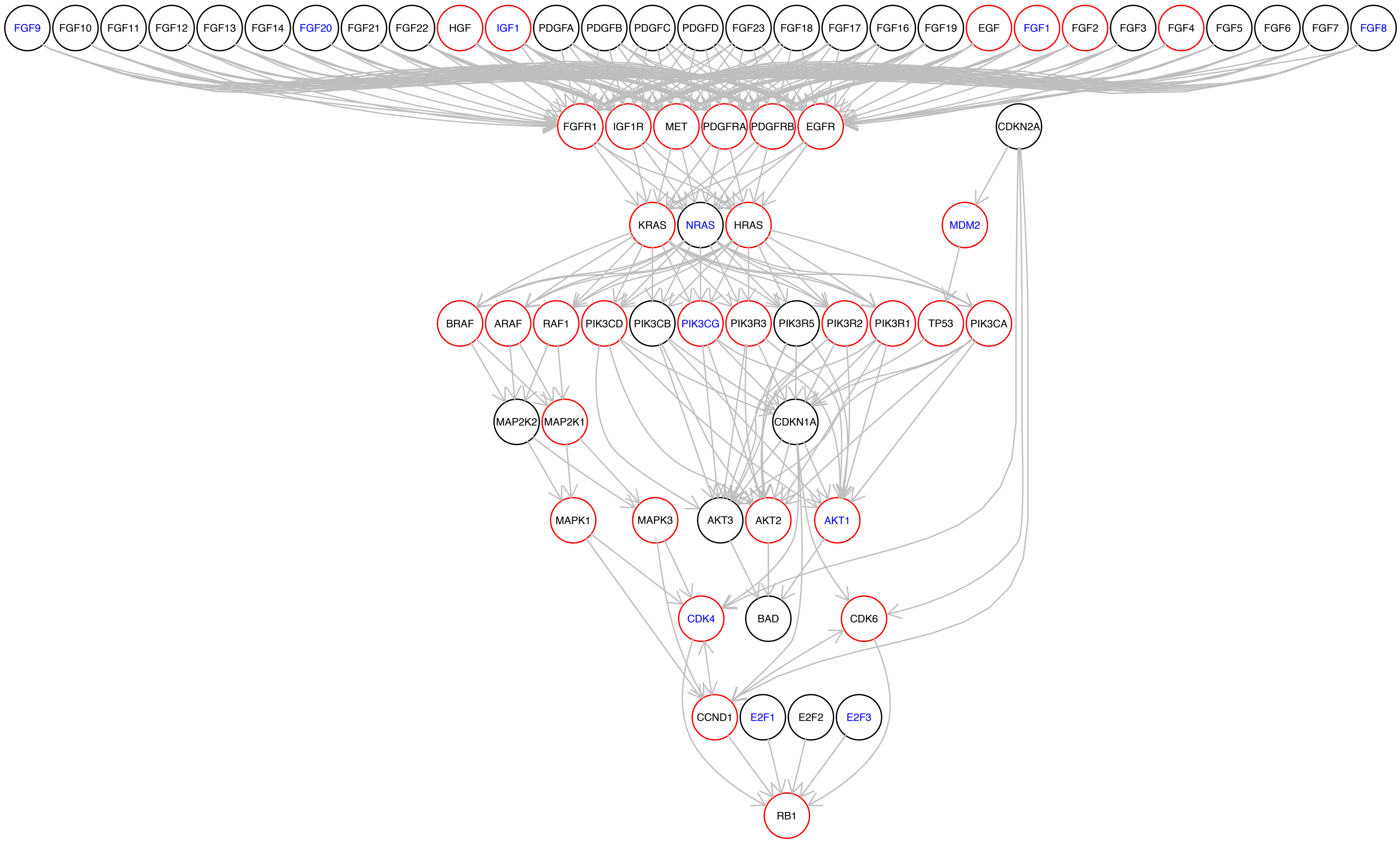

KEGG pathway = Melanoma : tumour = IPC298_SKIN : Yellow Fill = gene variant, Blue Text = expression−survival association, Red Boarder = drug

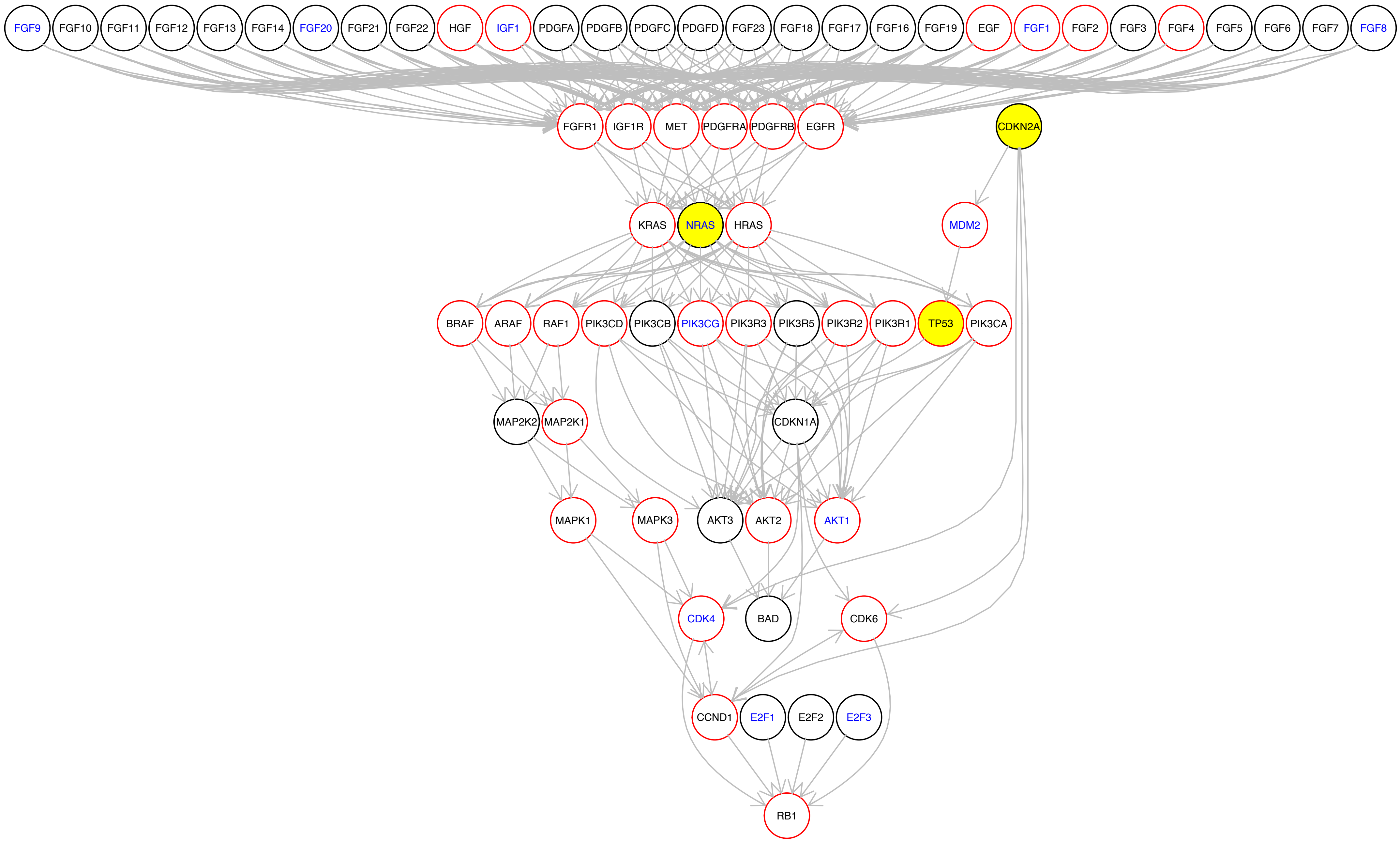

KEGG pathway = Melanoma : tumour = K029AX_SKIN : Yellow Fill = gene variant, Blue Text = expression−survival association, Red Boarder = drug

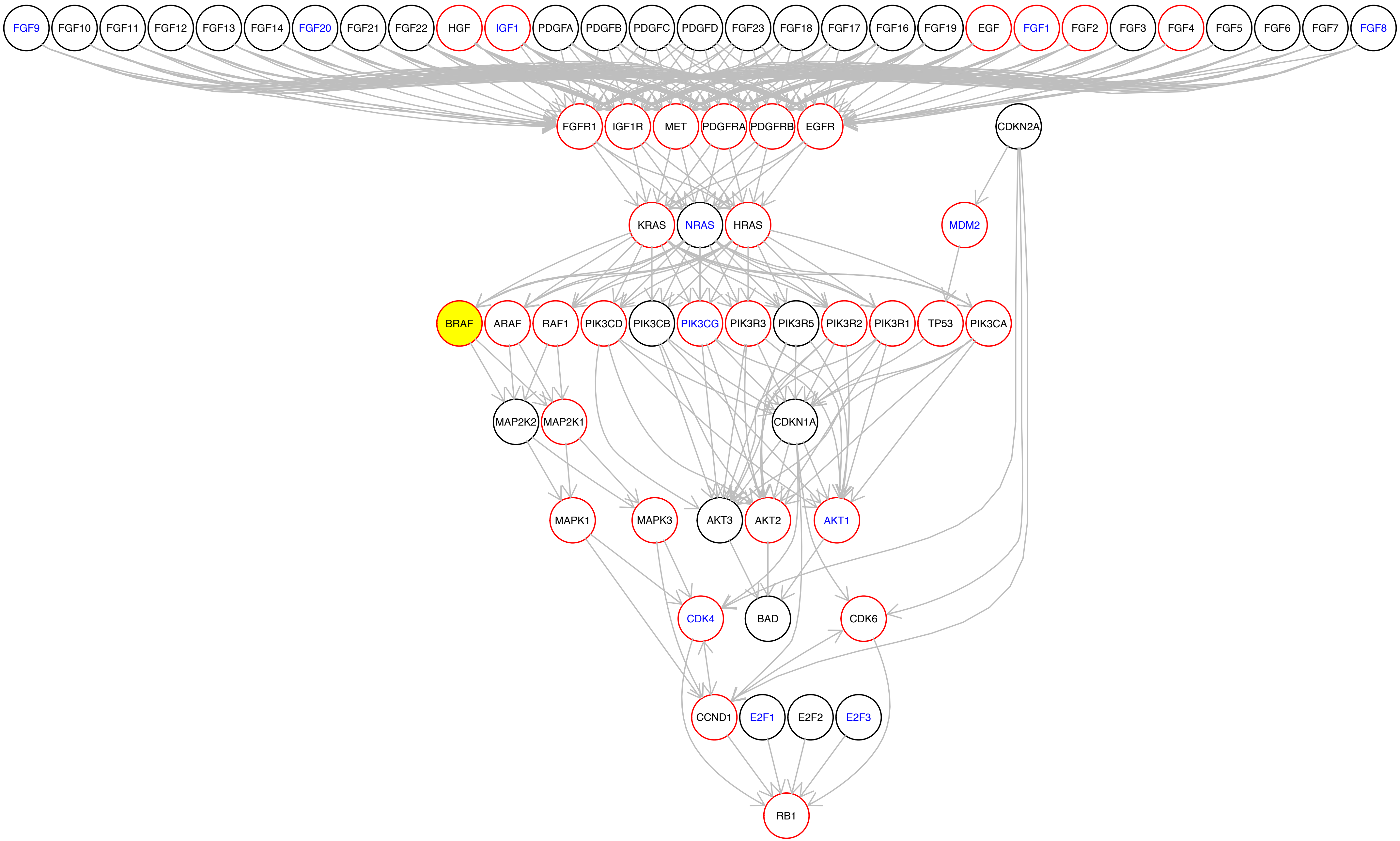

KEGG pathway = Melanoma : tumour = LOXIMVI_SKIN : Yellow Fill = gene variant, Blue Text = expression−survival association, Red Boarder = drug

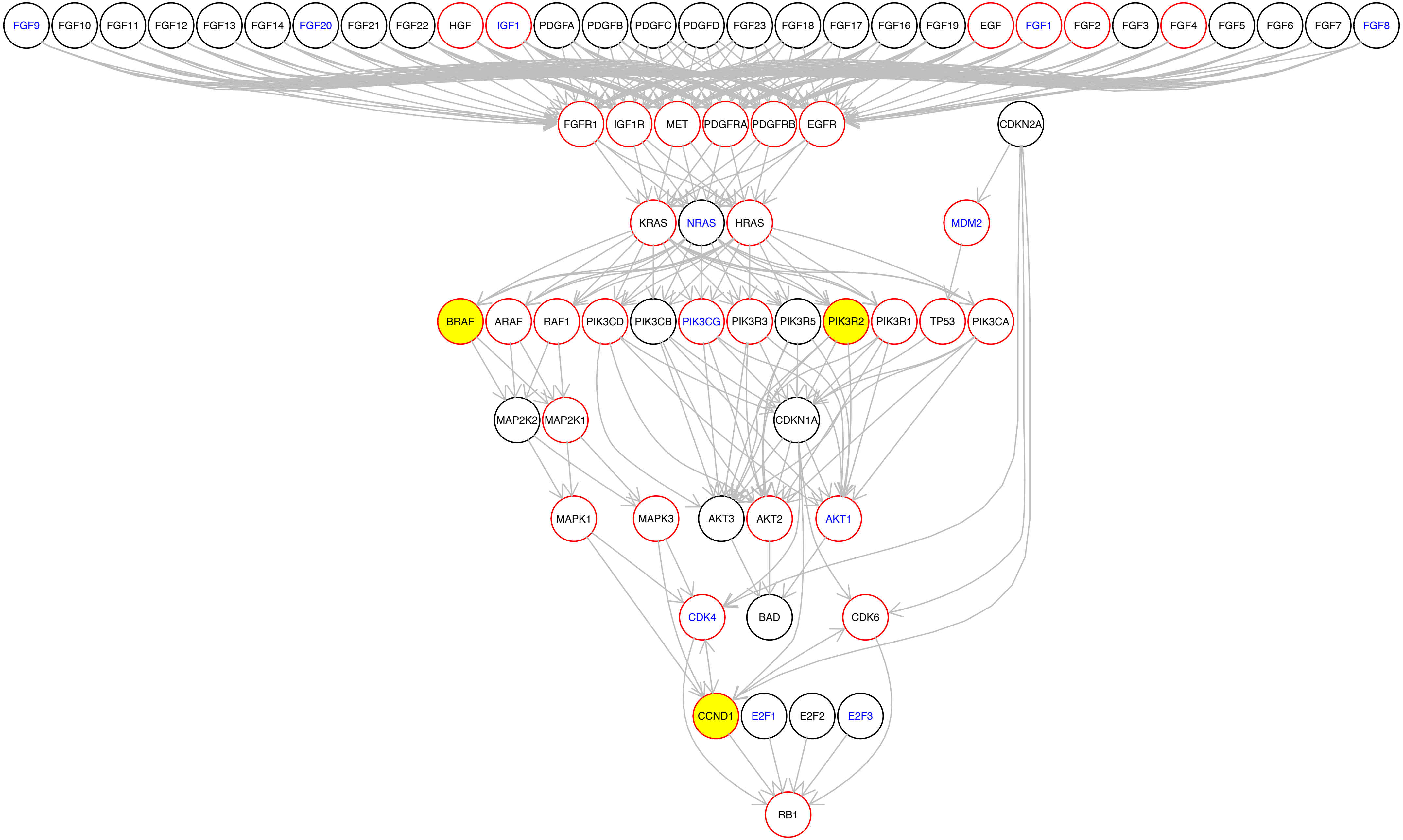

KEGG pathway = Melanoma : tumour = MALME3M_SKIN : Yellow Fill = gene variant, Blue Text = expression–survival association, Red Boarder = drug

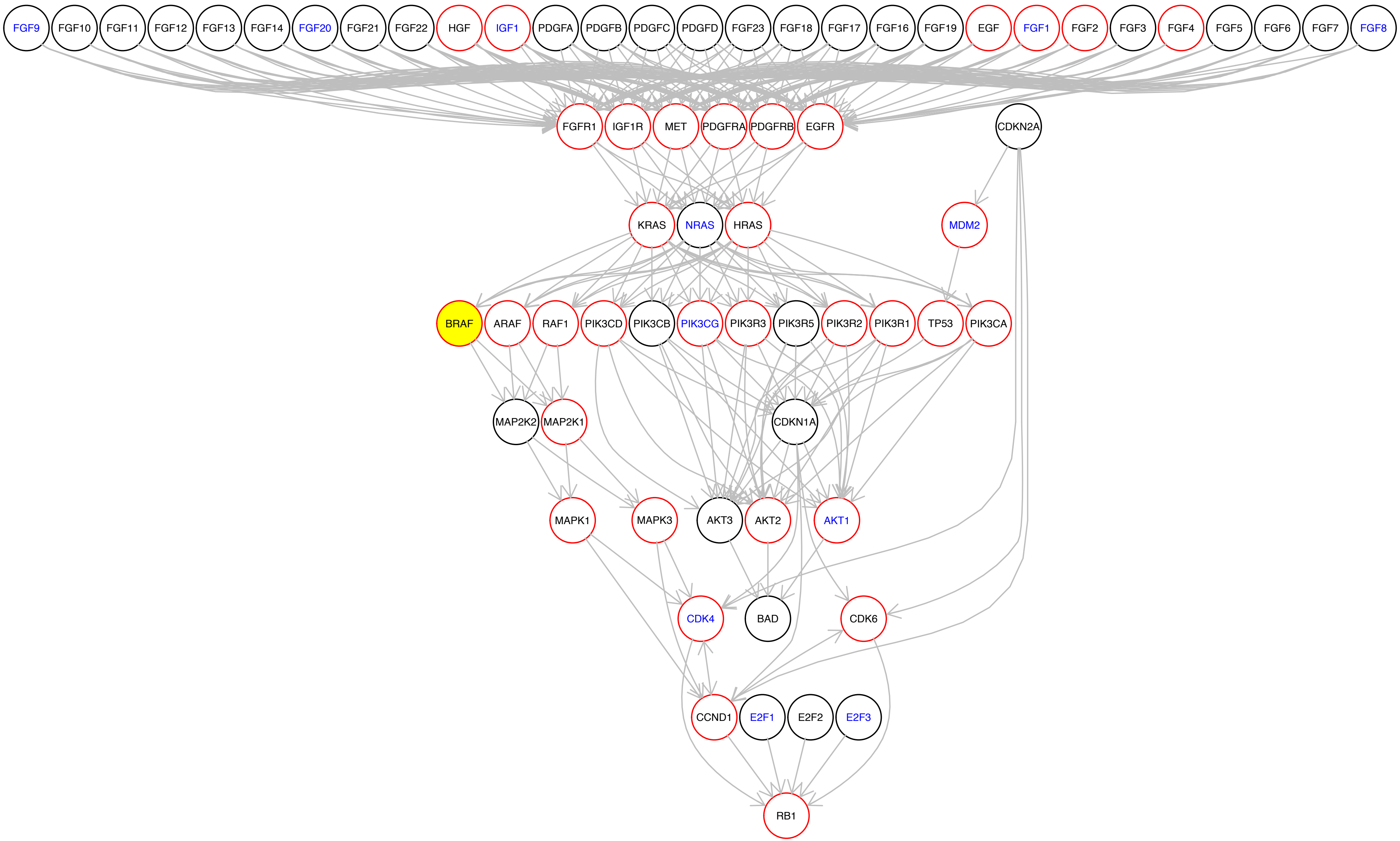

KEGG pathway = Melanoma : tumour = MDAMB435S_SKIN : Yellow Fill = gene variant, Blue Text = expression−survival association, Red Boarder = drug

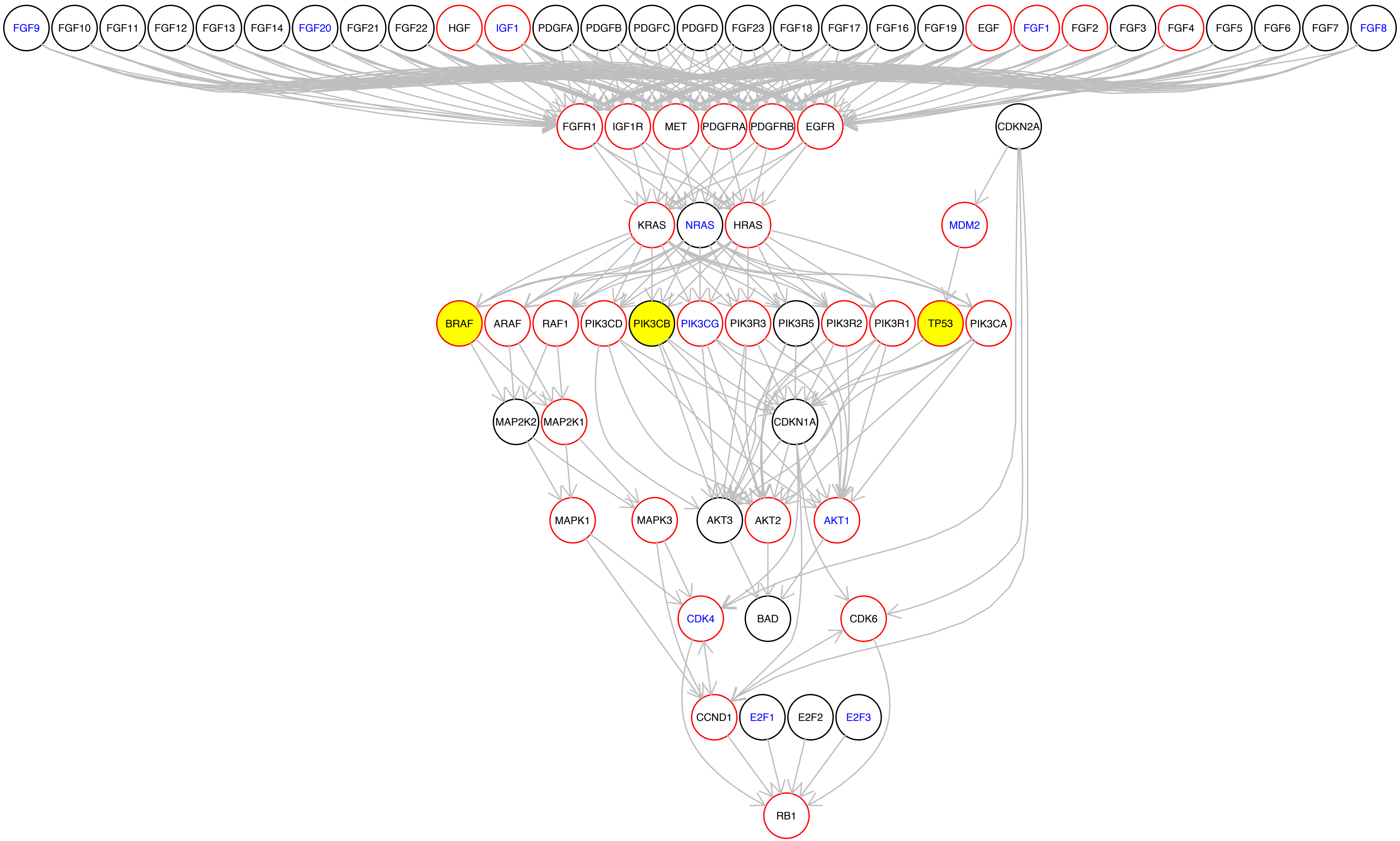

KEGG pathway = Melanoma :   tumour = MELHO_SKIN :   Yellow Fill = gene variant, Blue Text = expression−survival association, Red Boarder = drug

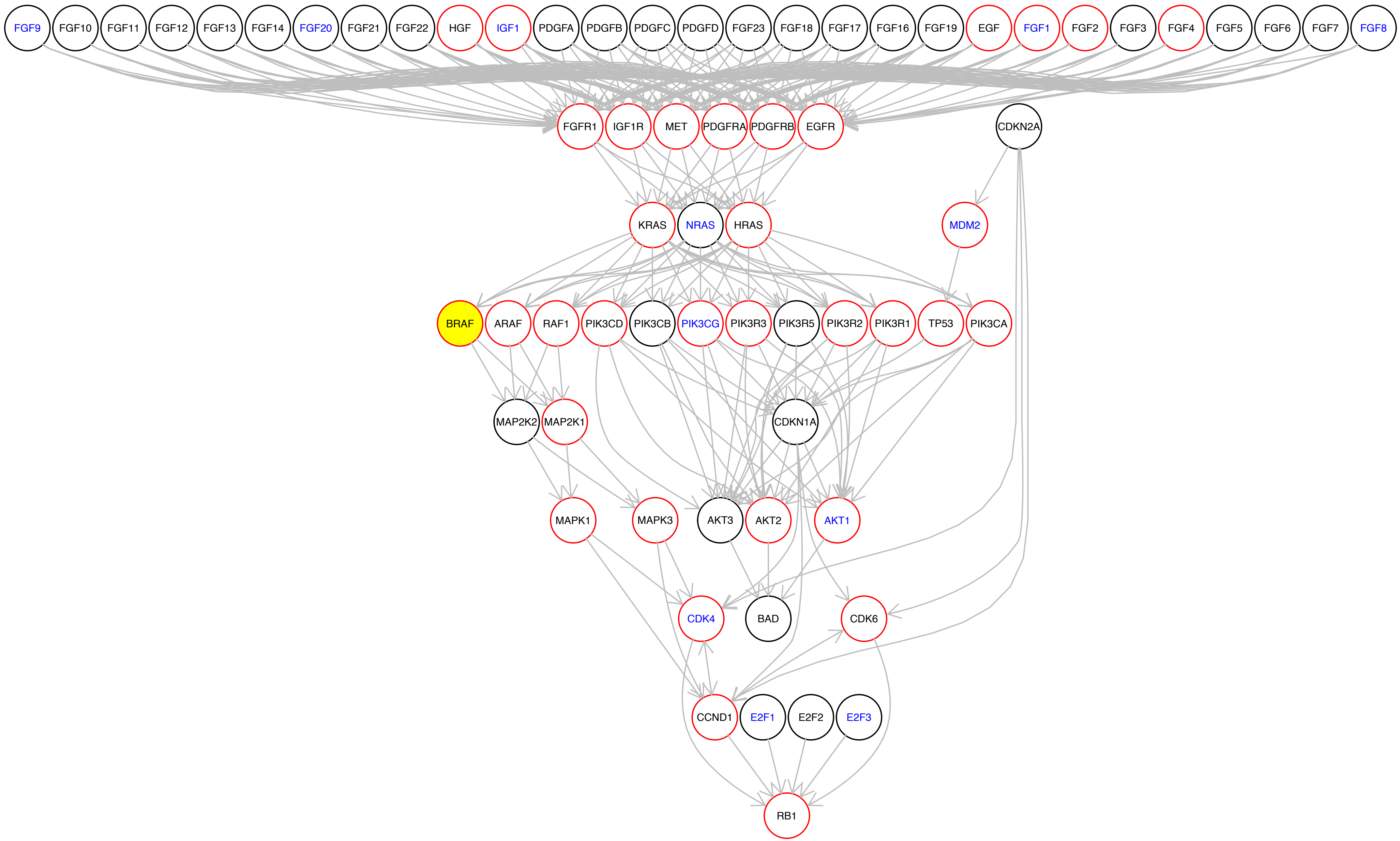

KEGG pathway = Melanoma :   tumour = MELJUSO_SKIN :   Yellow Fill = gene variant, Blue Text = expression–survival association, Red Boarder = drug

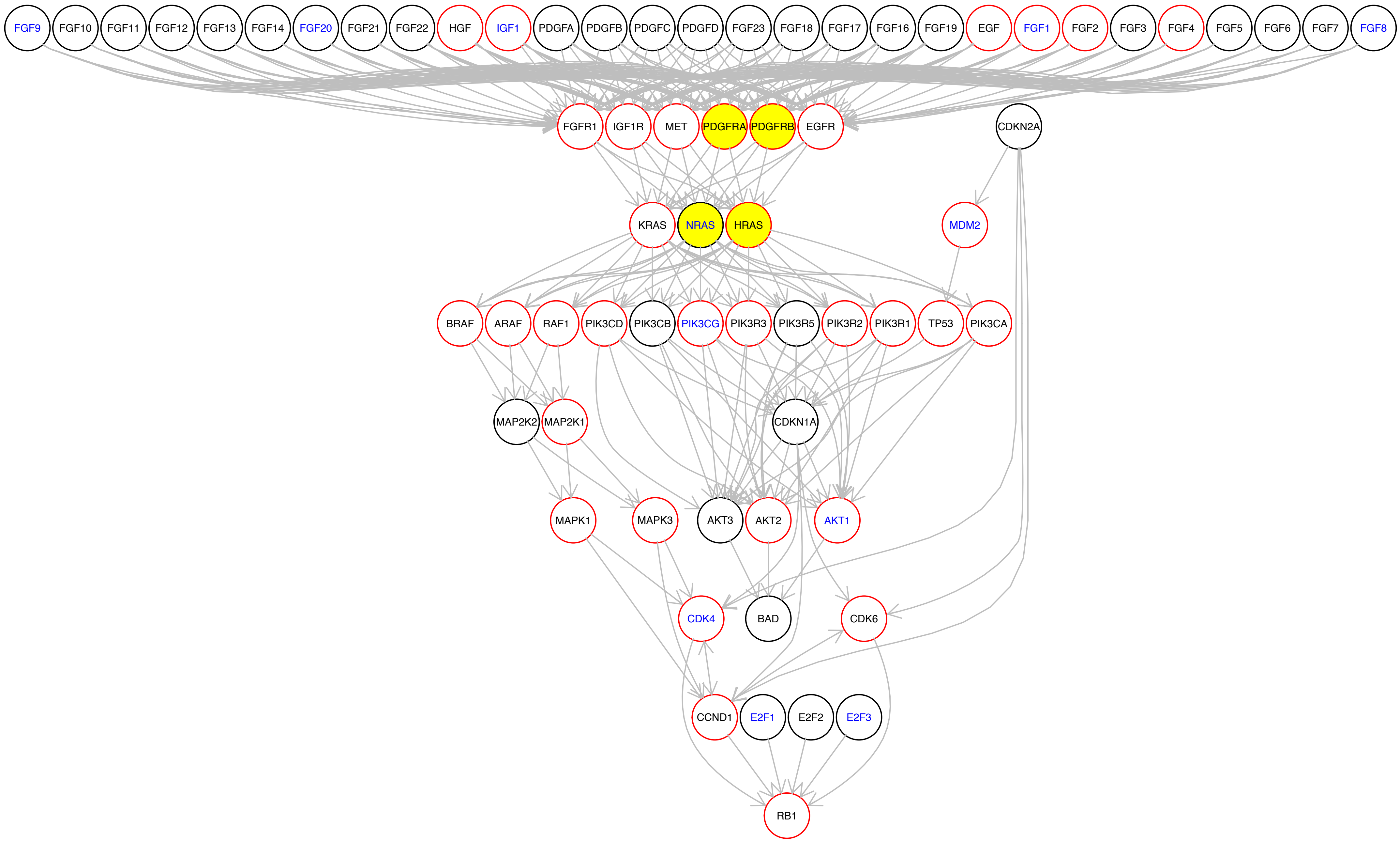

KEGG pathway = Melanoma : tumour = RPMI7951_SKIN : Yellow Fill = gene variant, Blue Text = expression−survival association, Red Boarder = drug

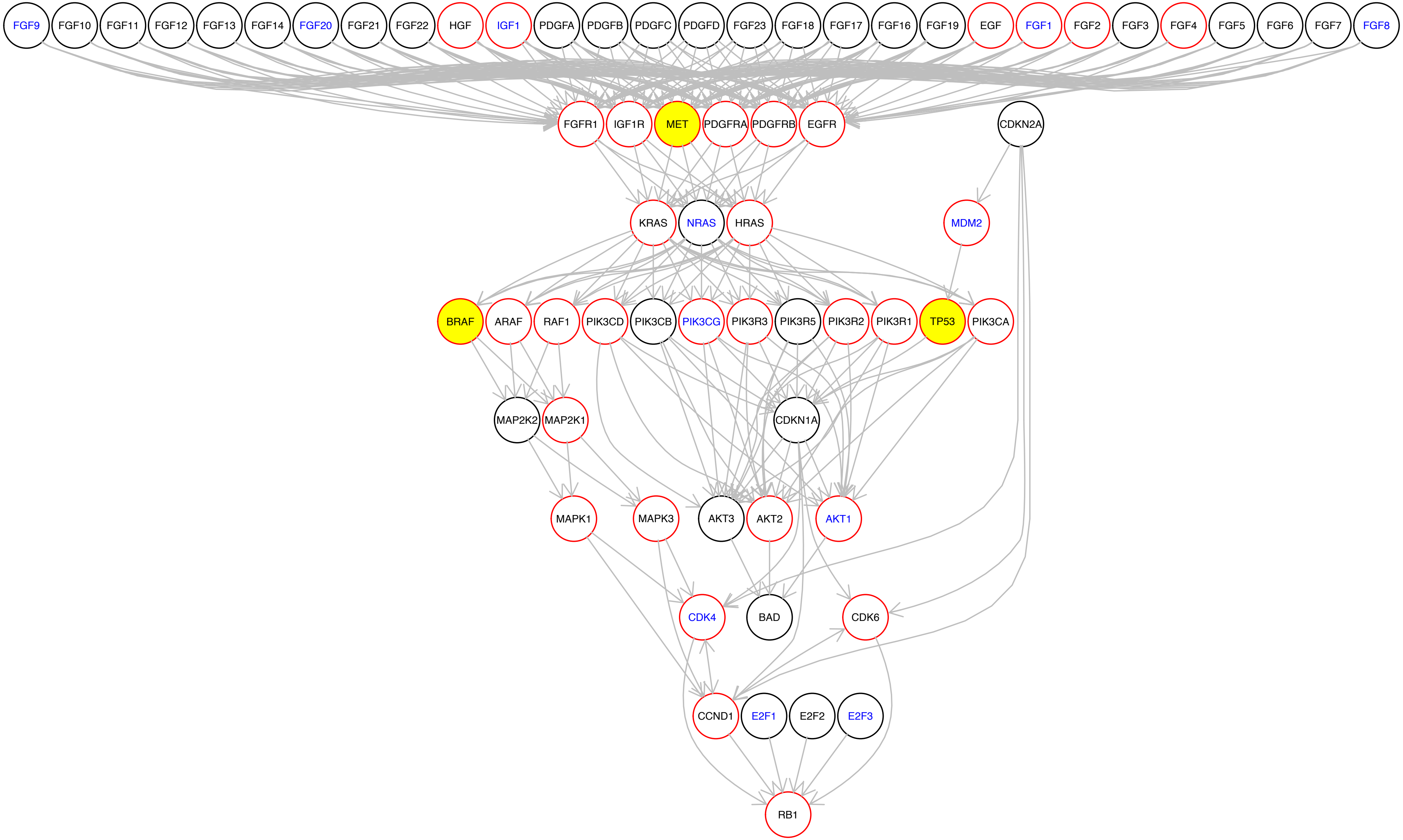

KEGG pathway = Melanoma : tumour = SH4_SKIN : Yellow Fill = gene variant, Blue Text = expression−survival association, Red Boarder = drug

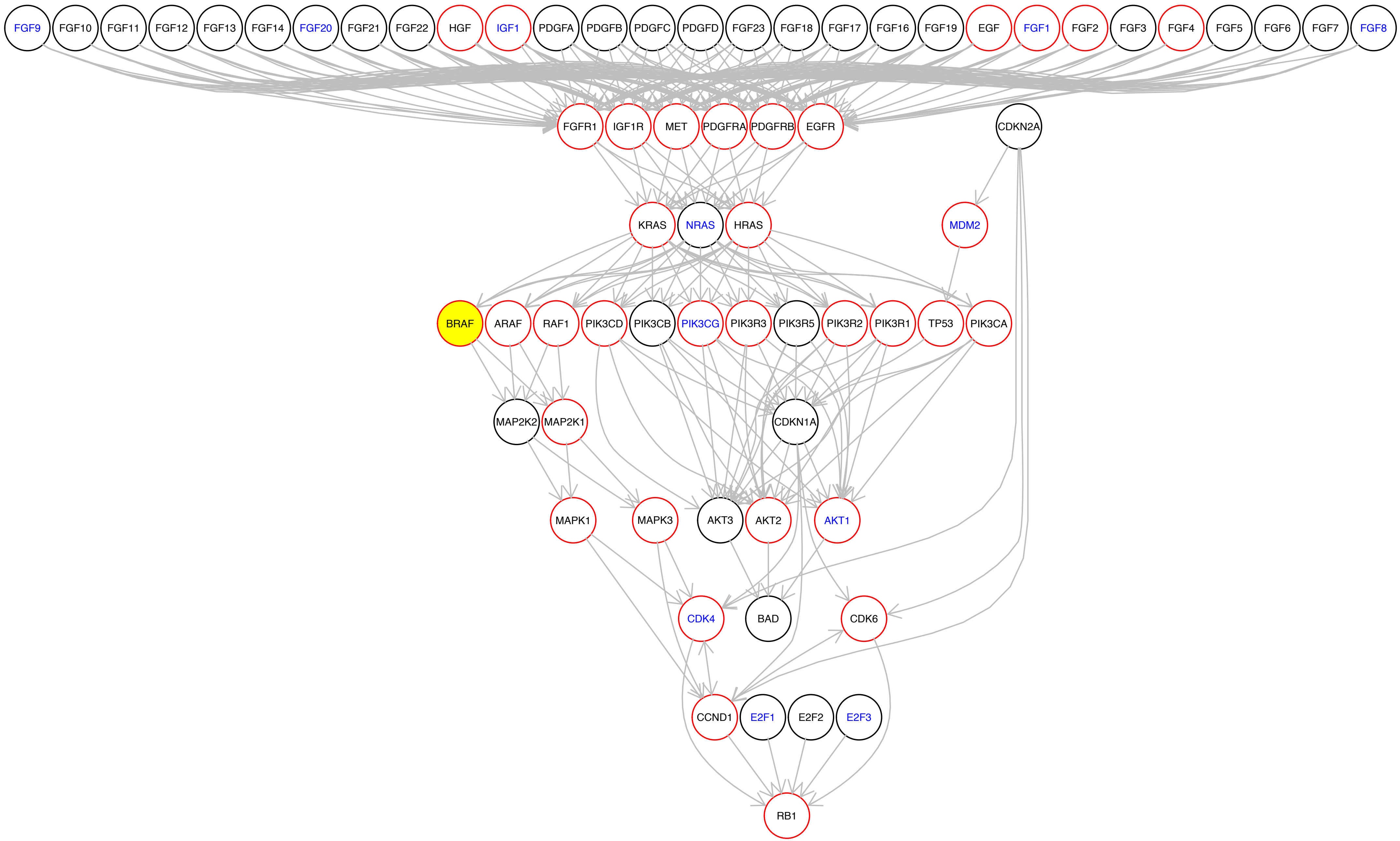

KEGG pathway = Melanoma : tumour = SKMEL1_SKIN : Yellow Fill = gene variant, Blue Text = expression−survival association, Red Boarder = drug

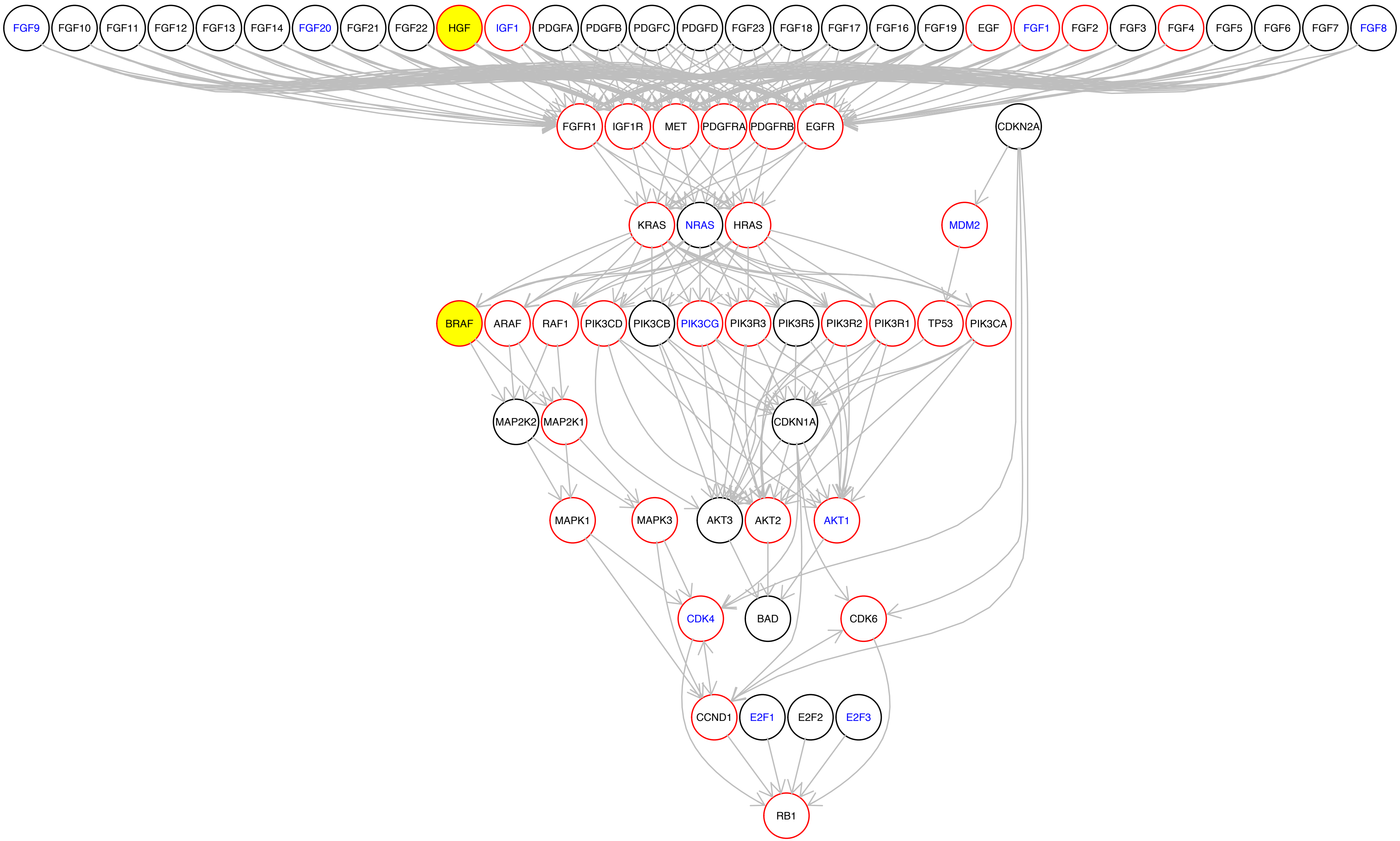

KEGG pathway = Melanoma : tumour = SKMEL2_SKIN : Yellow Fill = gene variant, Blue Text = expression−survival association, Red Boarder = drug

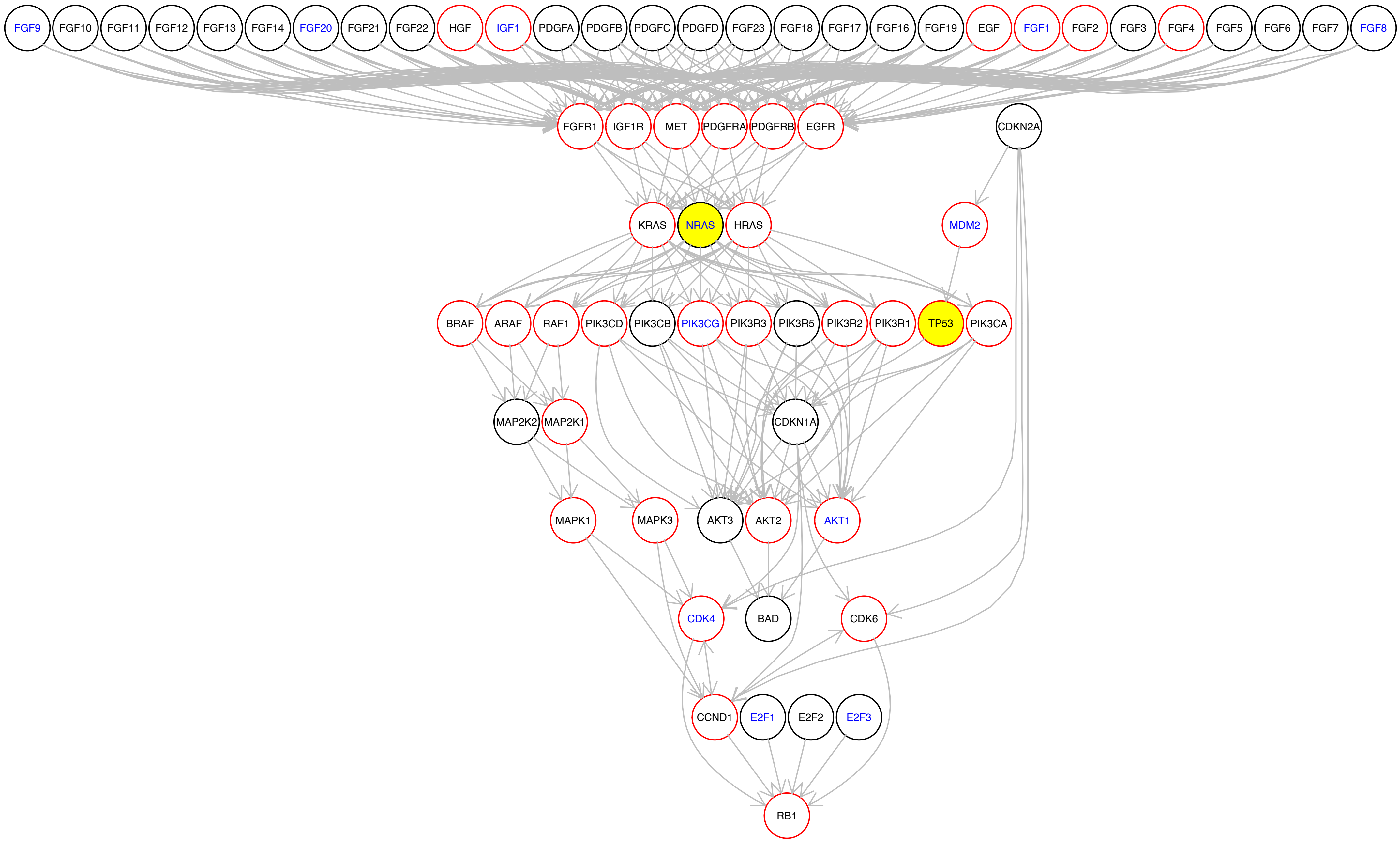

KEGG pathway = Melanoma : tumour = SKMEL24_SKIN : Yellow Fill = gene variant, Blue Text = expression−survival association, Red Boarder = drug

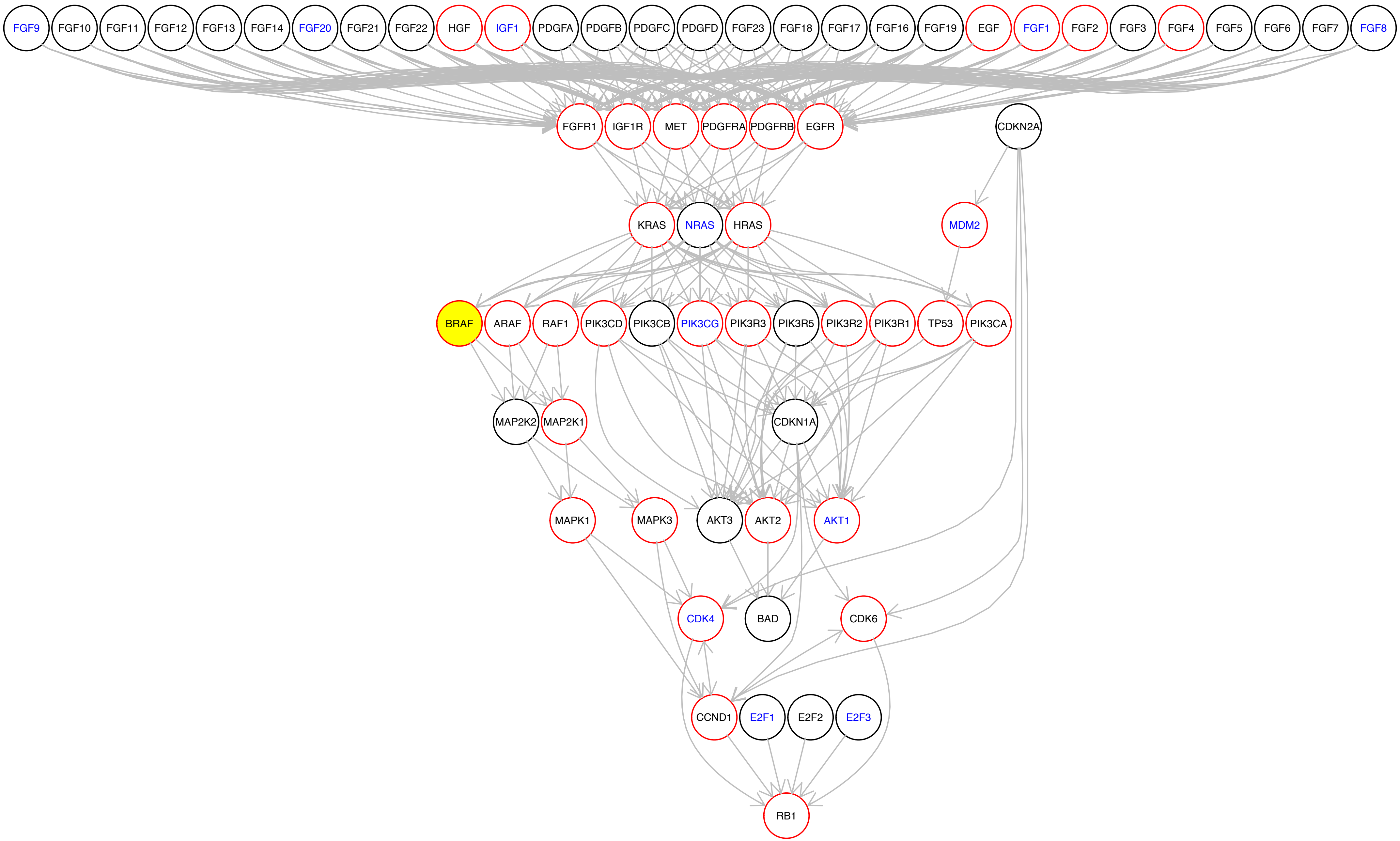

KEGG pathway = Melanoma : tumour = SKMEL28_SKIN : Yellow Fill = gene variant, Blue Text = expression−survival association, Red Boarder = drug

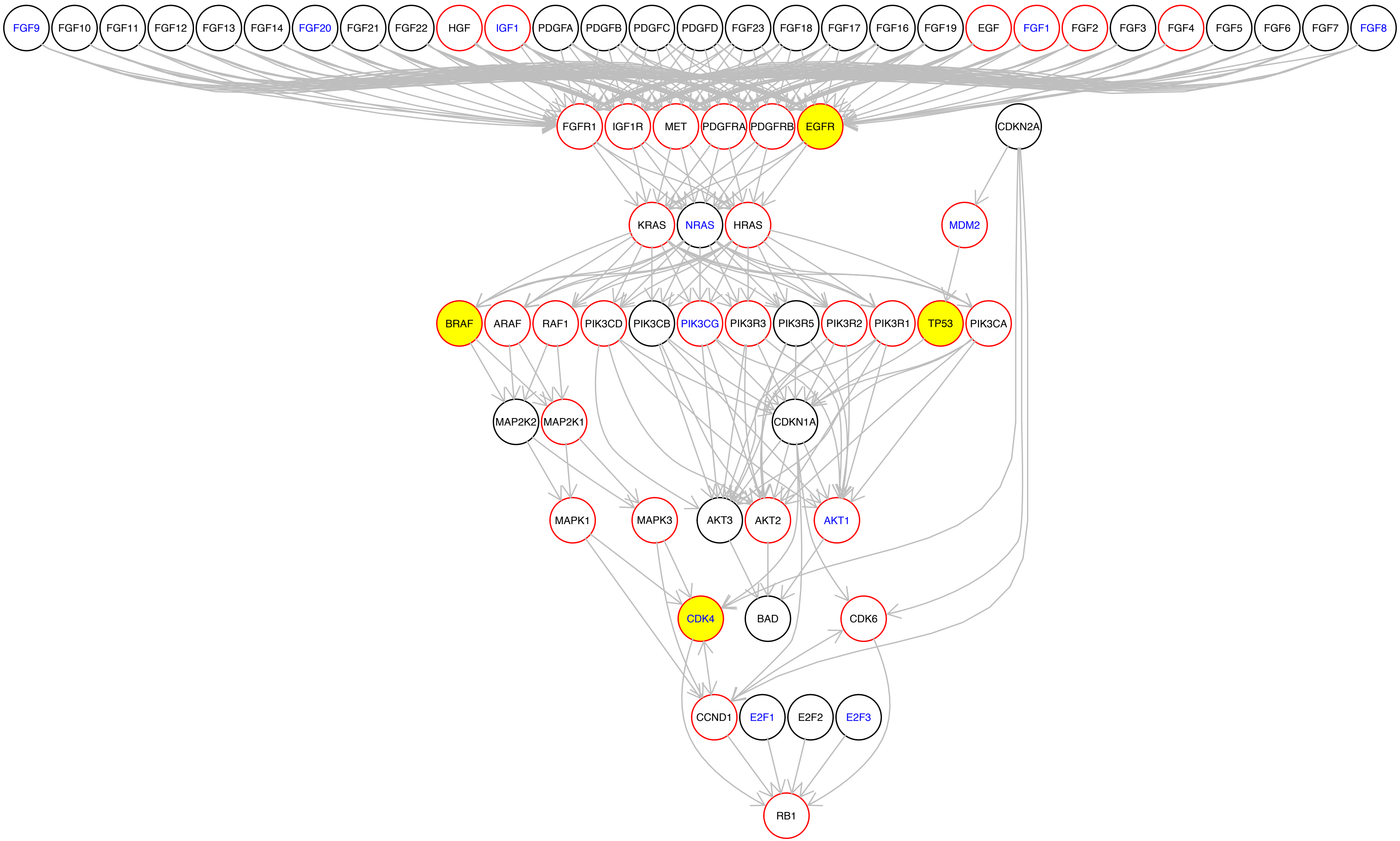

KEGG pathway = Melanoma : tumour = SKMEL3_SKIN : Yellow Fill = gene variant, Blue Text = expression−survival association, Red Boarder = drug

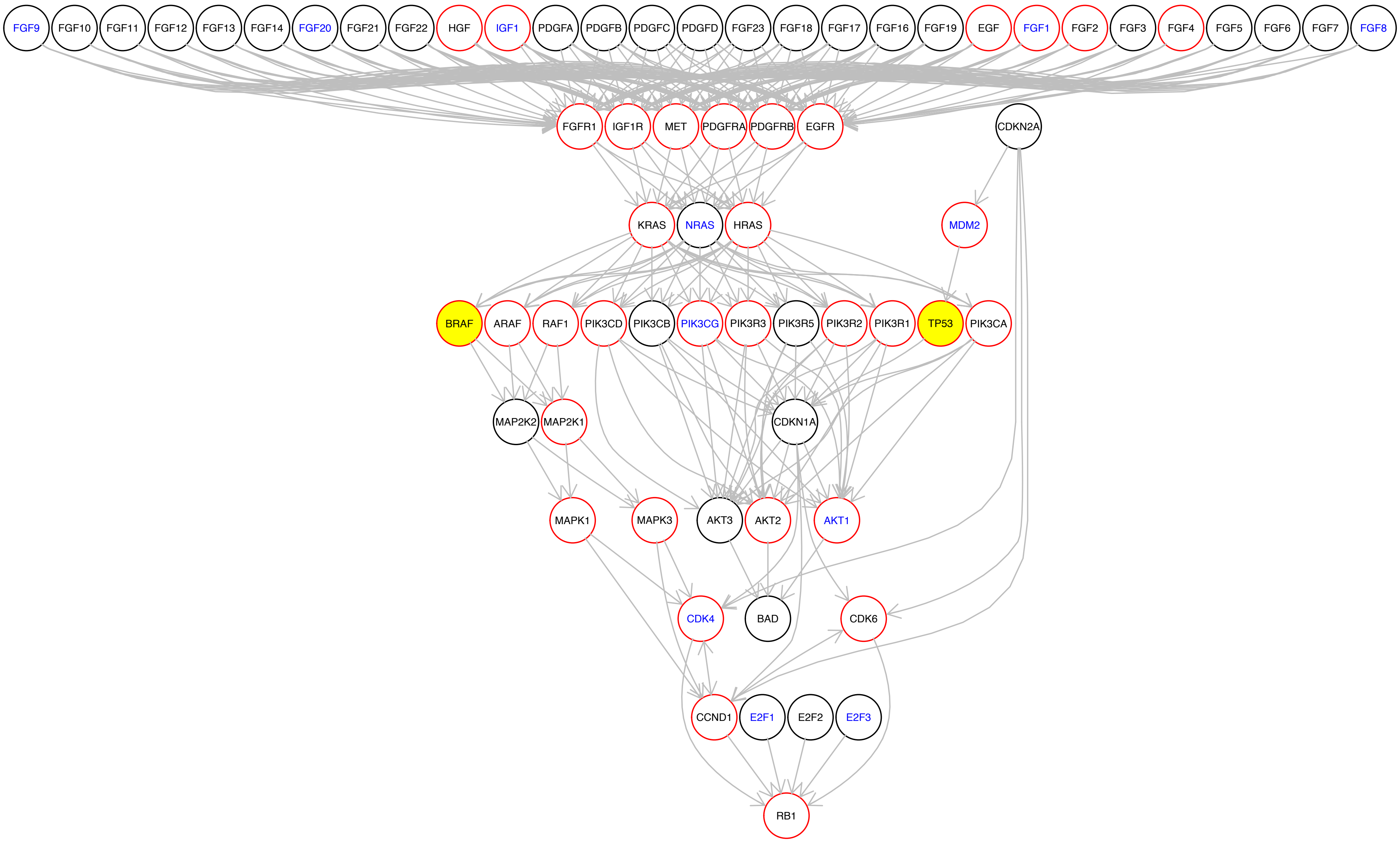

KEGG pathway = Melanoma : tumour = SKMEL30_SKIN : Yellow Fill = gene variant, Blue Text = expression−survival association, Red Boarder = drug

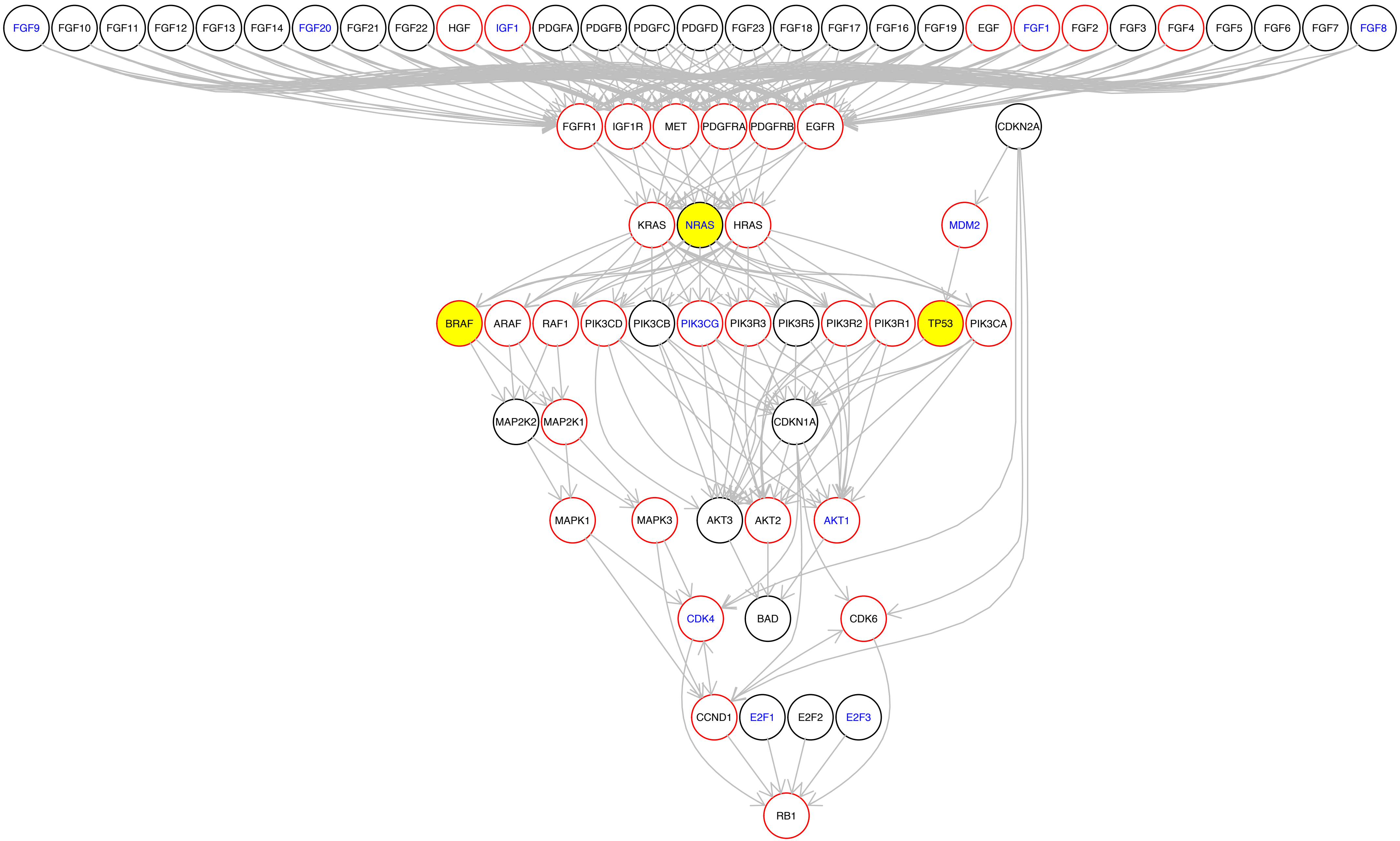

KEGG pathway = Melanoma : tumour = SKMEL5_SKIN : Yellow Fill = gene variant, Blue Text = expression−survival association, Red Boarder = drug

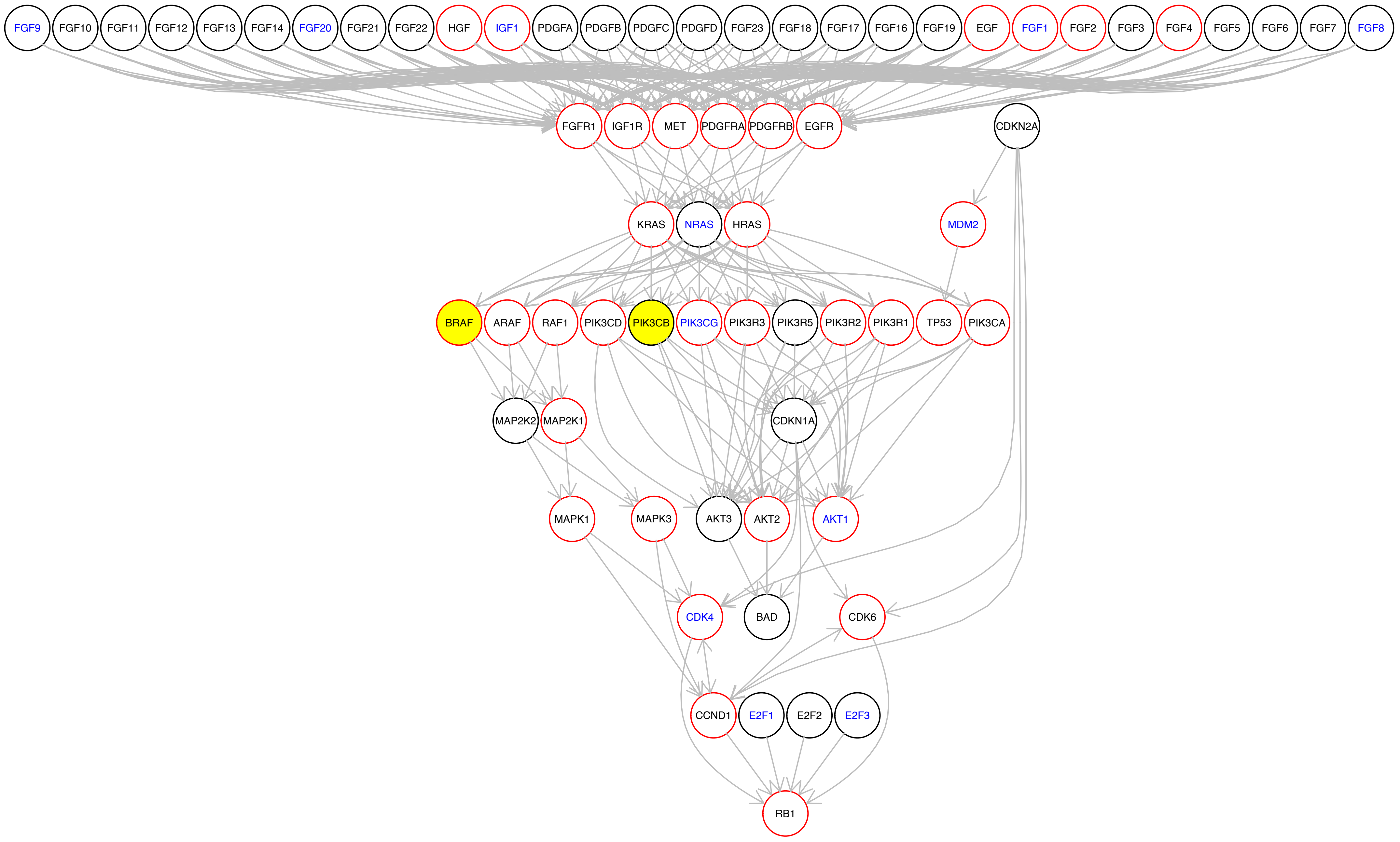

KEGG pathway = Melanoma : tumour = UACC257_SKIN : Yellow Fill = gene variant, Blue Text = expression−survival association, Red Boarder = drug

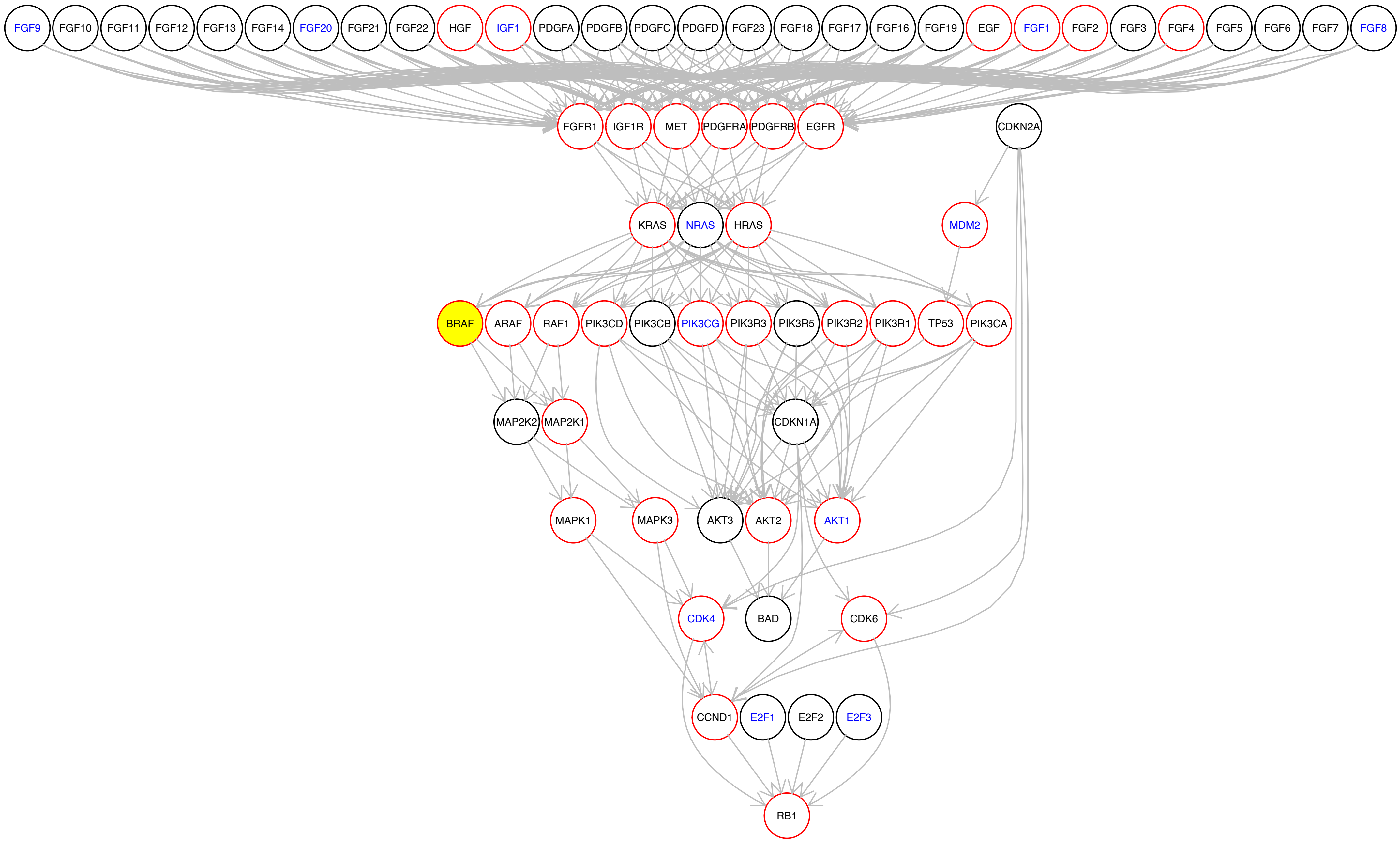

KEGG pathway = Melanoma : tumour = UACC62_SKIN : Yellow Fill = gene variant, Blue Text = expression−survival association, Red Boarder = drug

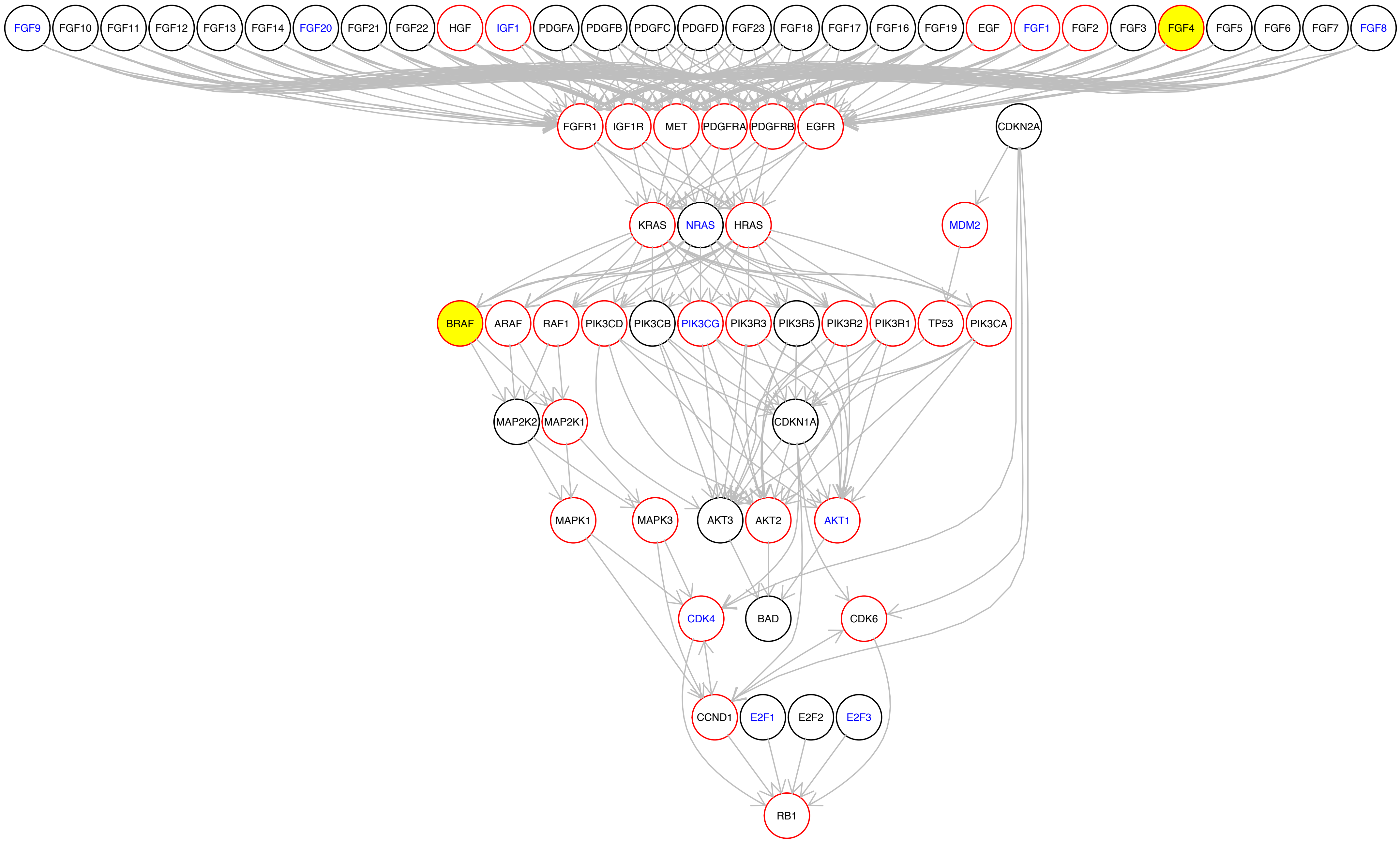

KEGG pathway = Melanoma : tumour = WM115_SKIN : Yellow Fill = gene variant, Blue Text = expression–survival association, Red Boarder = drug

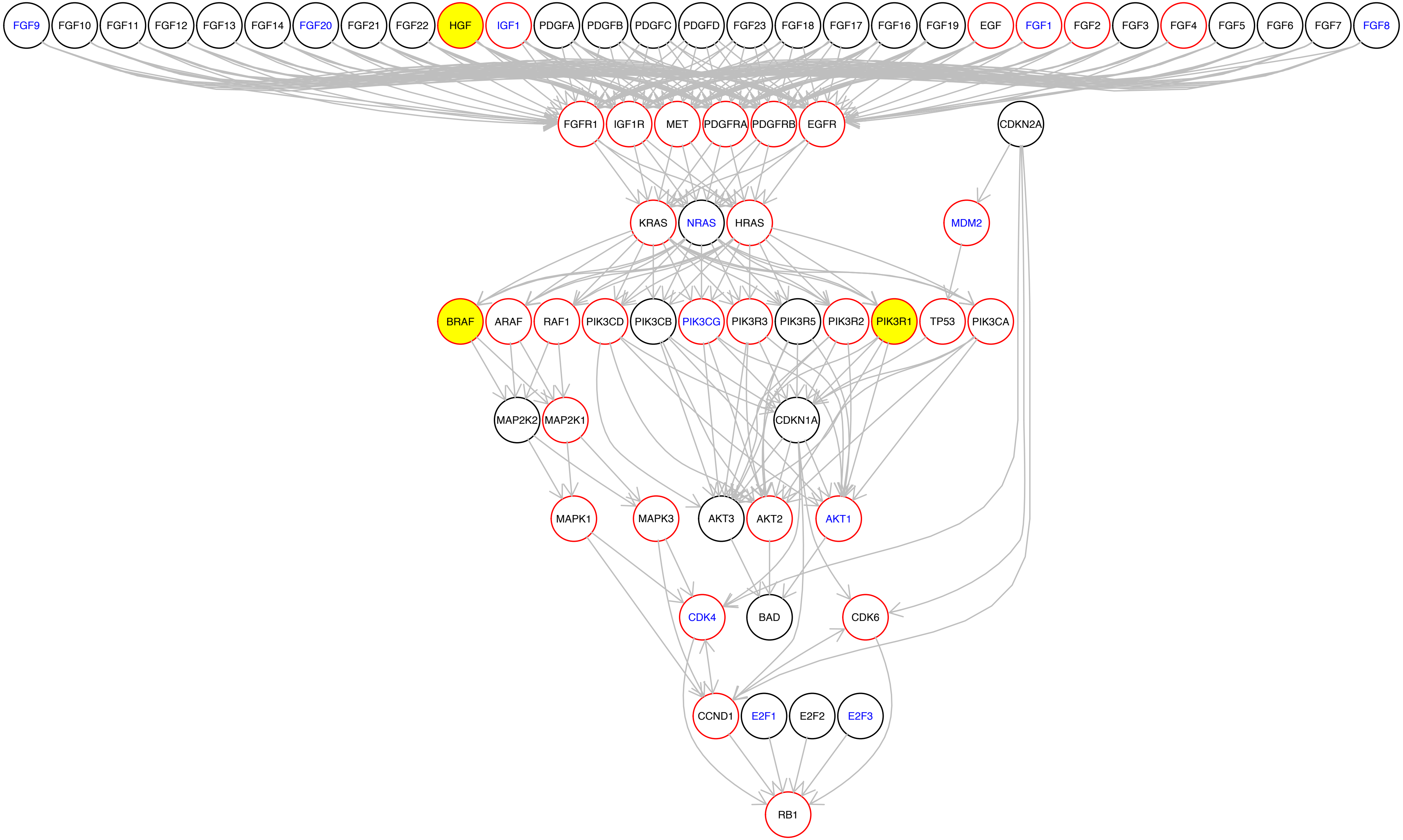

KEGG pathway = Melanoma : tumour = WM1799_SKIN : Yellow Fill = gene variant, Blue Text = expression−survival association, Red Boarder = drug

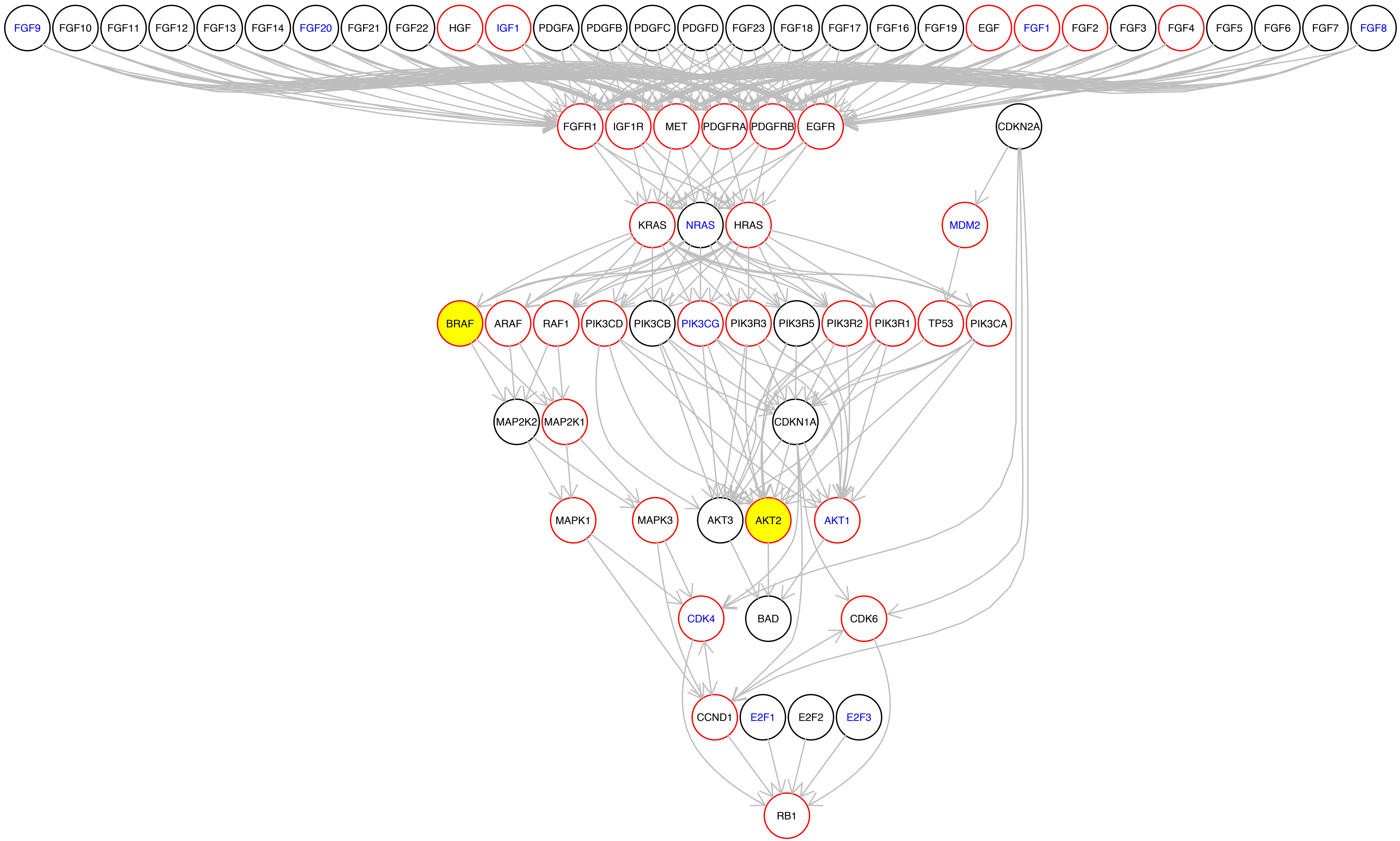

KEGG pathway = Melanoma : tumour = WM2664_SKIN : Yellow Fill = gene variant, Blue Text = expression−survival association, Red Boarder = drug

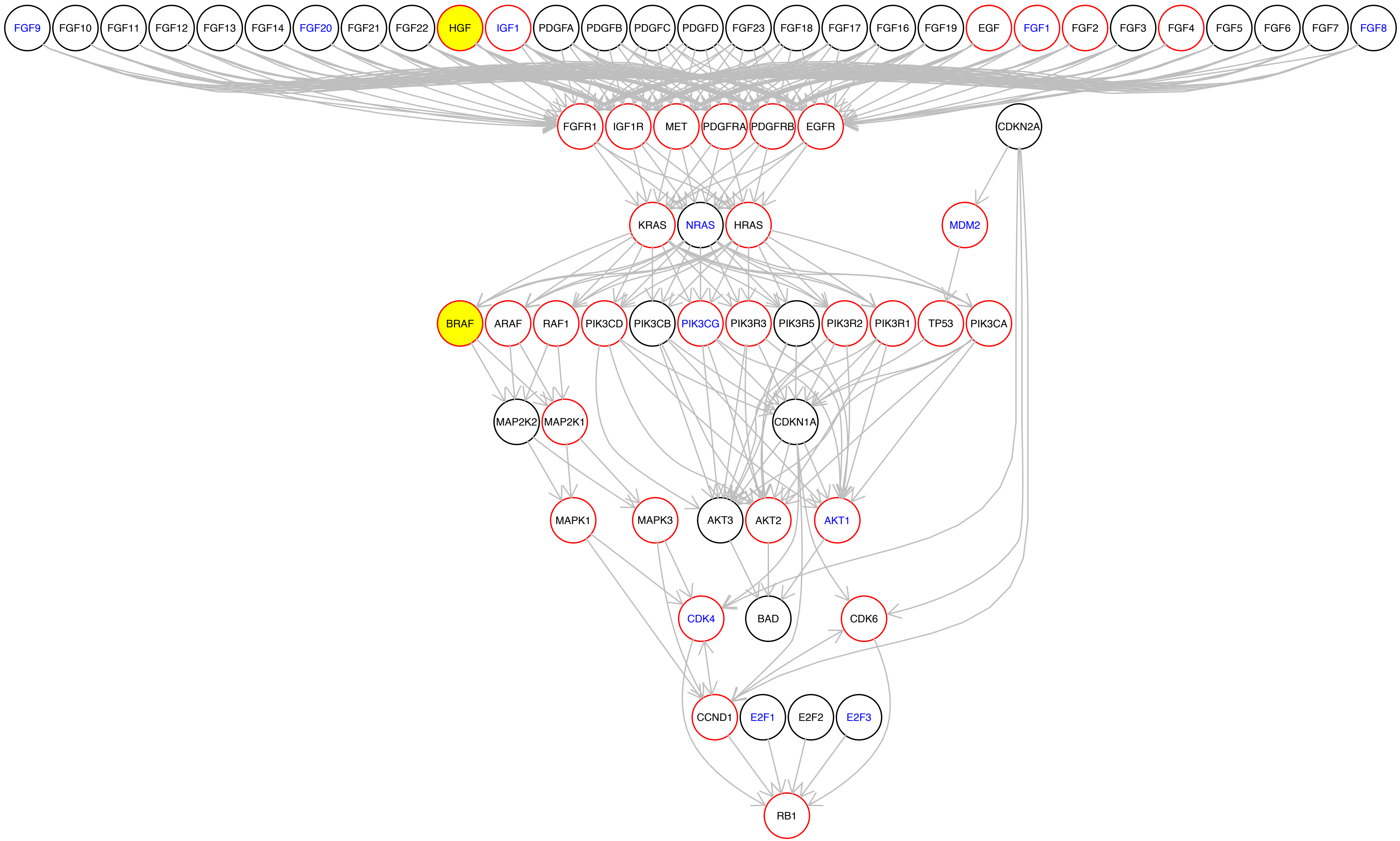

KEGG pathway = Melanoma : tumour = WM793_SKIN : Yellow Fill = gene variant, Blue Text = expression−survival association, Red Boarder = drug

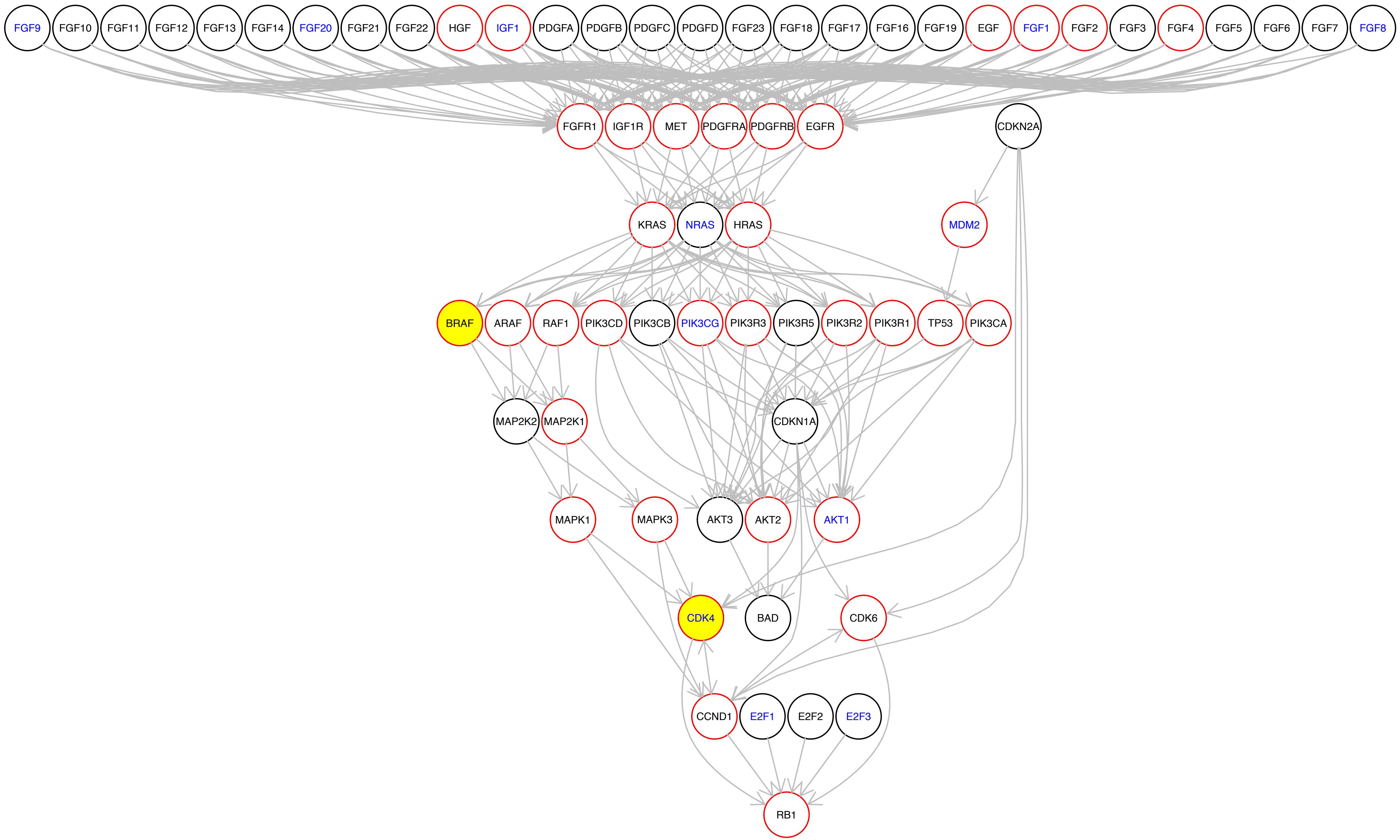

KEGG pathway = Melanoma : tumour = WM88_SKIN : Yellow Fill = gene variant, Blue Text = expression–survival association, Red Boarder = drug

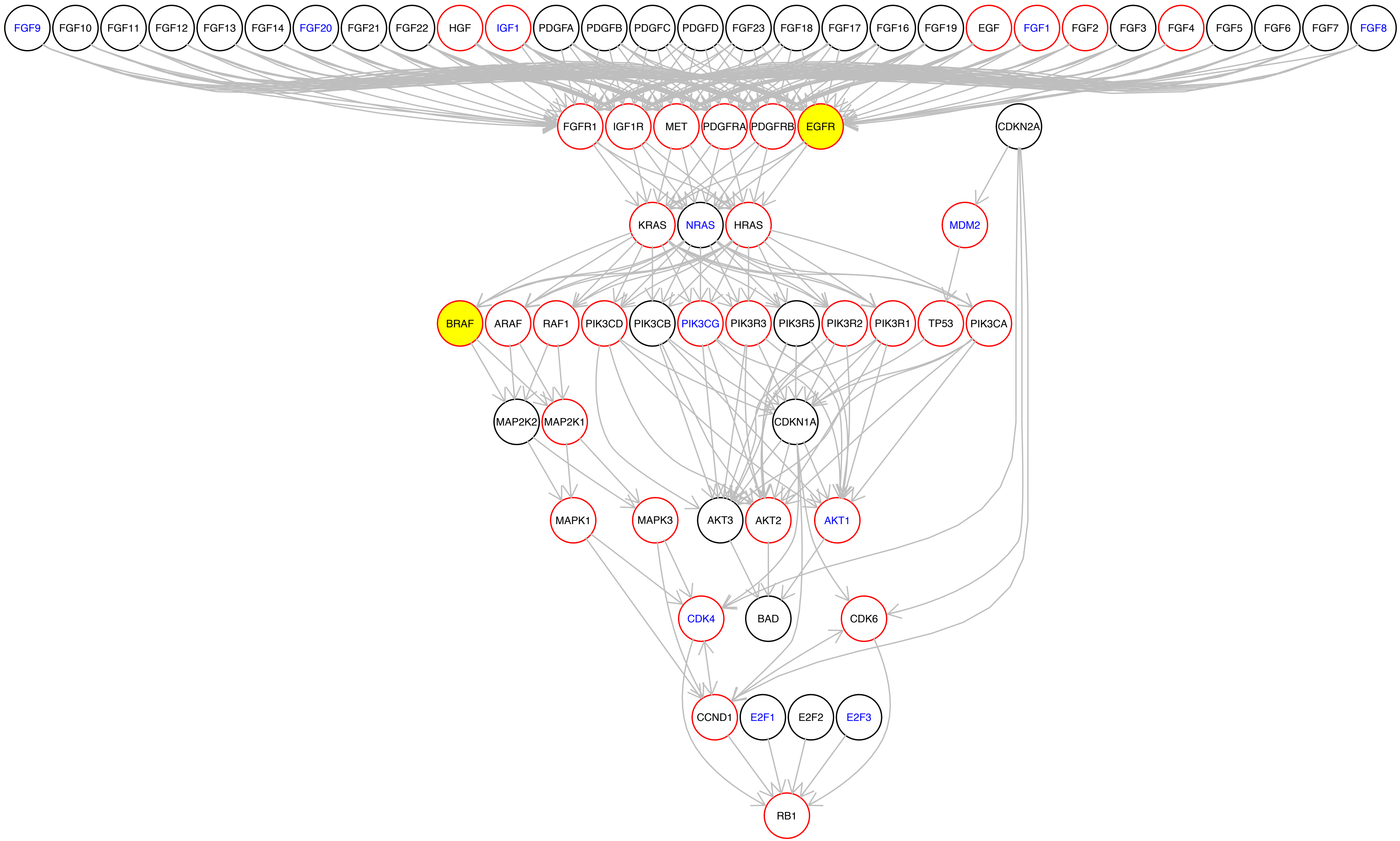

KEGG pathway = Melanoma : tumour = WM983B_SKIN : Yellow Fill = gene variant, Blue Text = expression−survival association, Red Boarder = drug

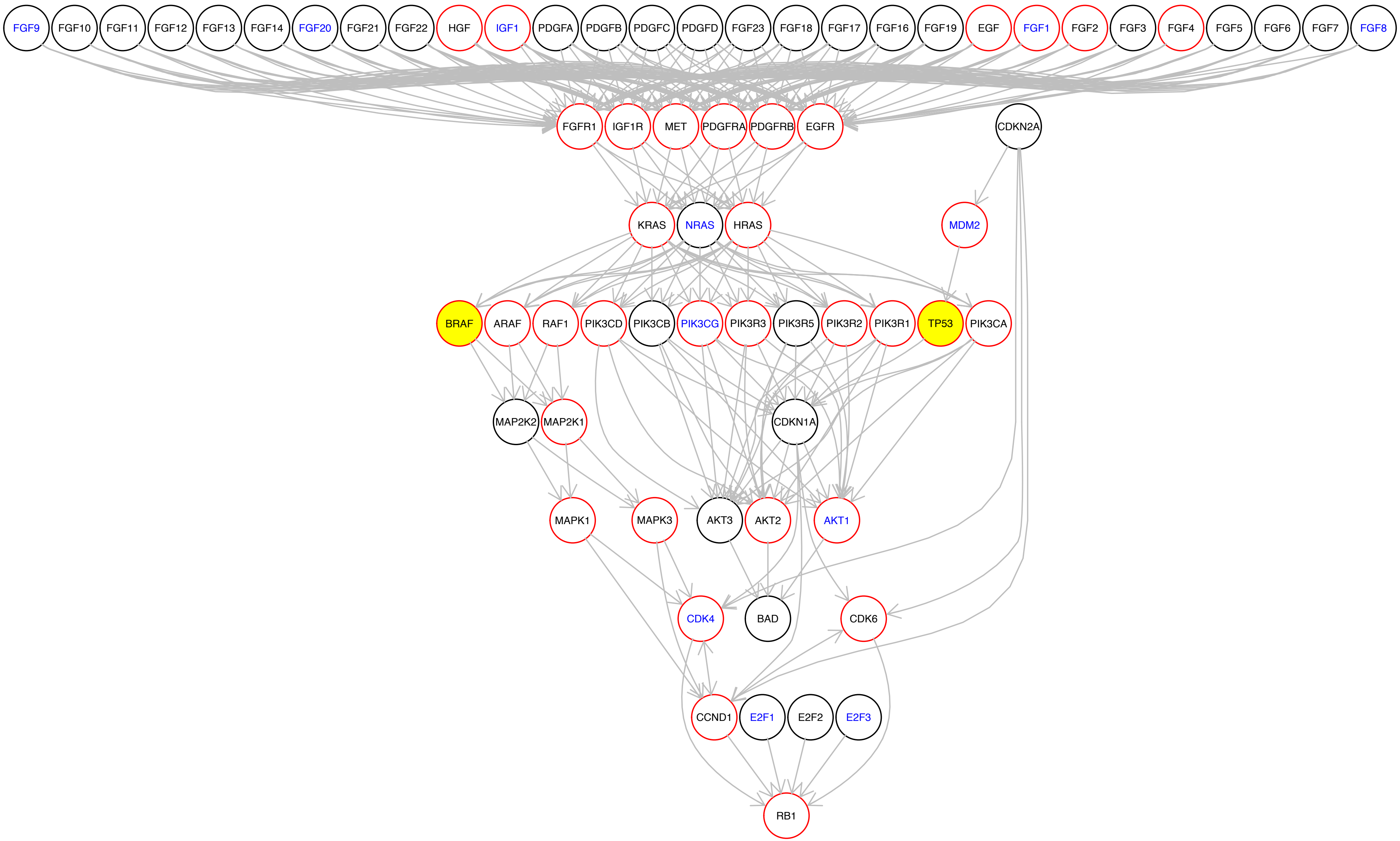

KEGG pathway = Melanoma : tumour = LAU108 : Yellow Fill = gene variant, Blue Text = expression−survival association, Red Boarder = drug

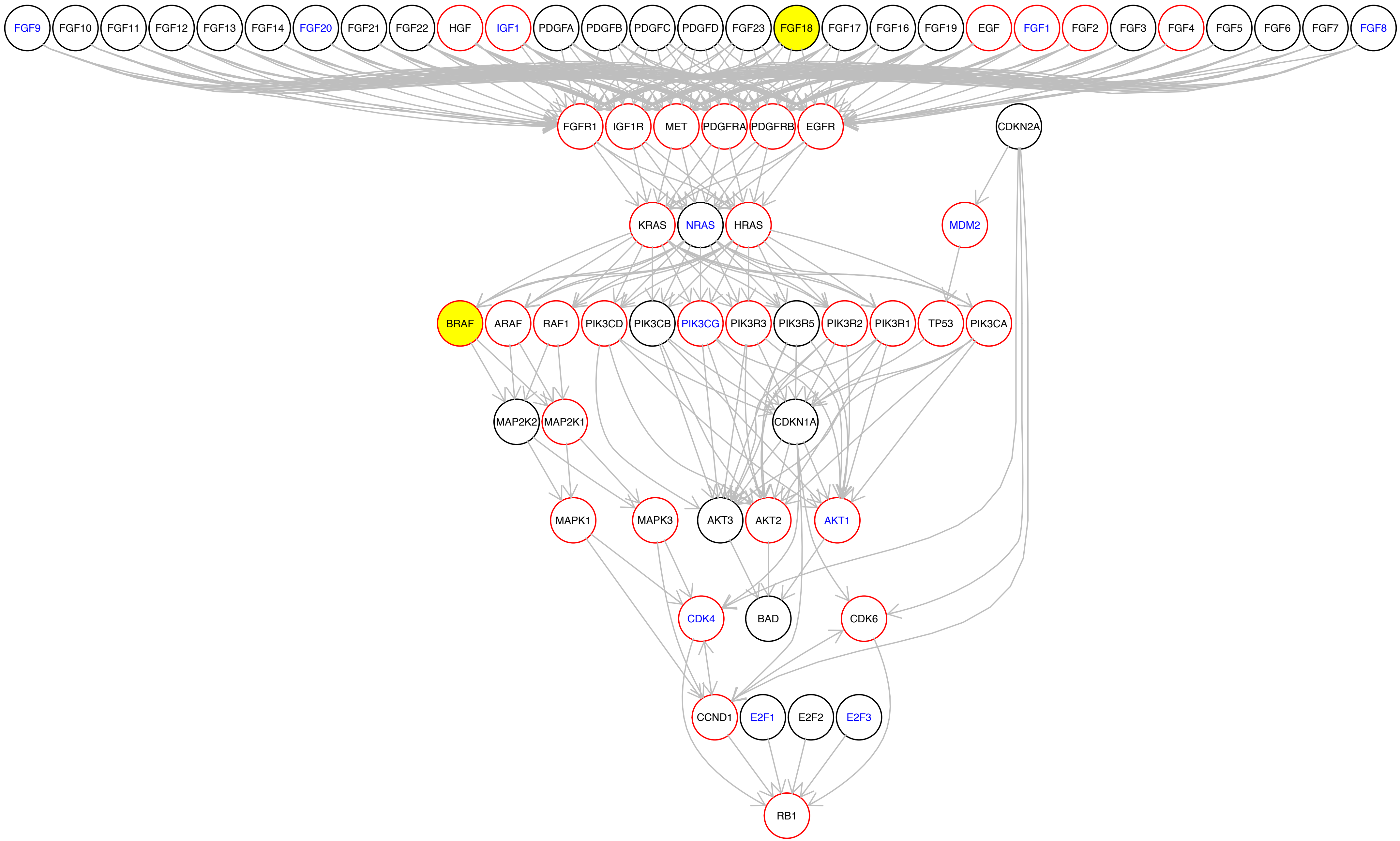

KEGG pathway = Melanoma : tumour = LAU149 : Yellow Fill = gene variant, Blue Text = expression−survival association, Red Boarder = drug

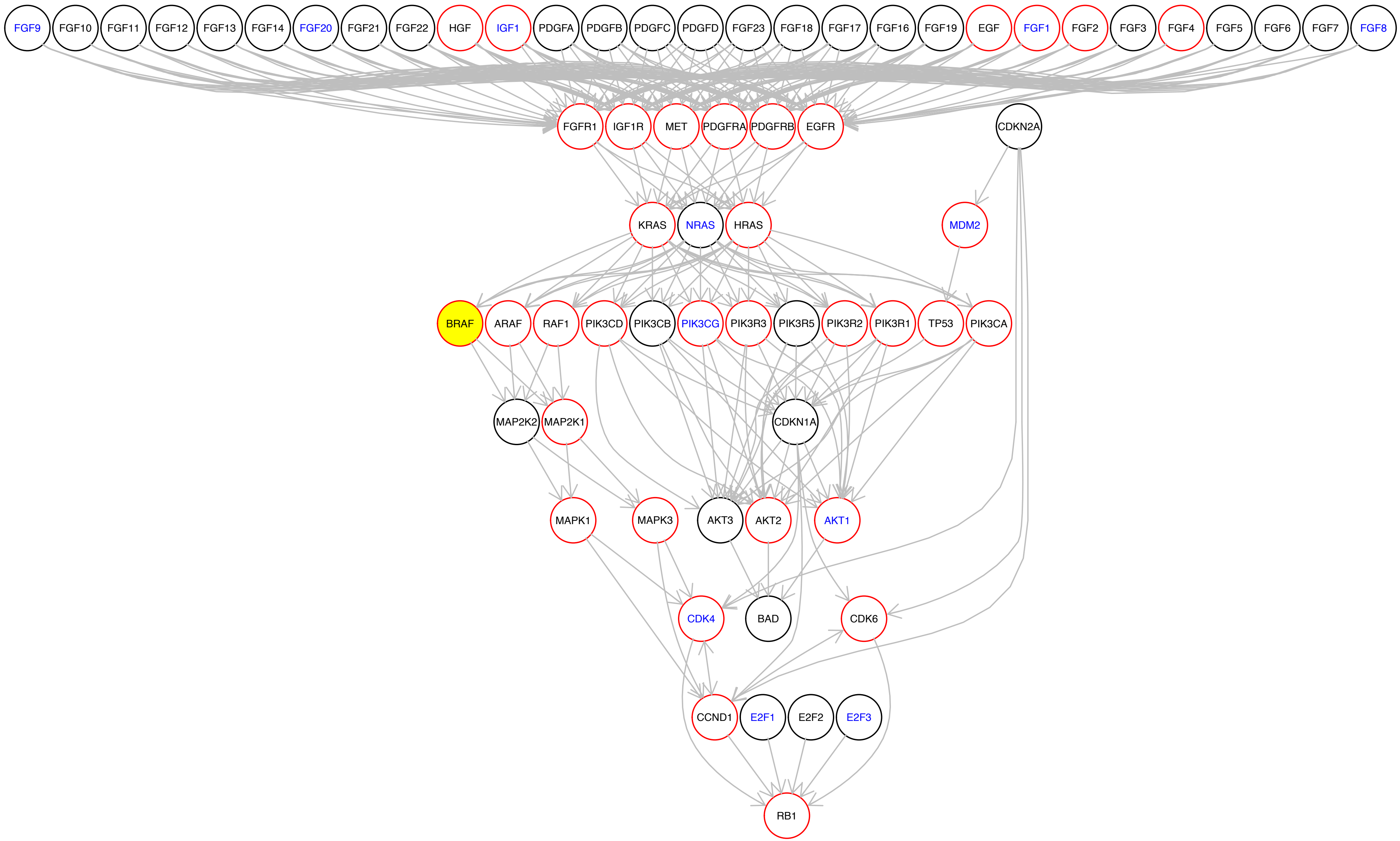

KEGG pathway = Melanoma :   tumour = LAU165 :   Yellow Fill = gene variant, Blue Text = expression−survival association, Red Boarder = drug

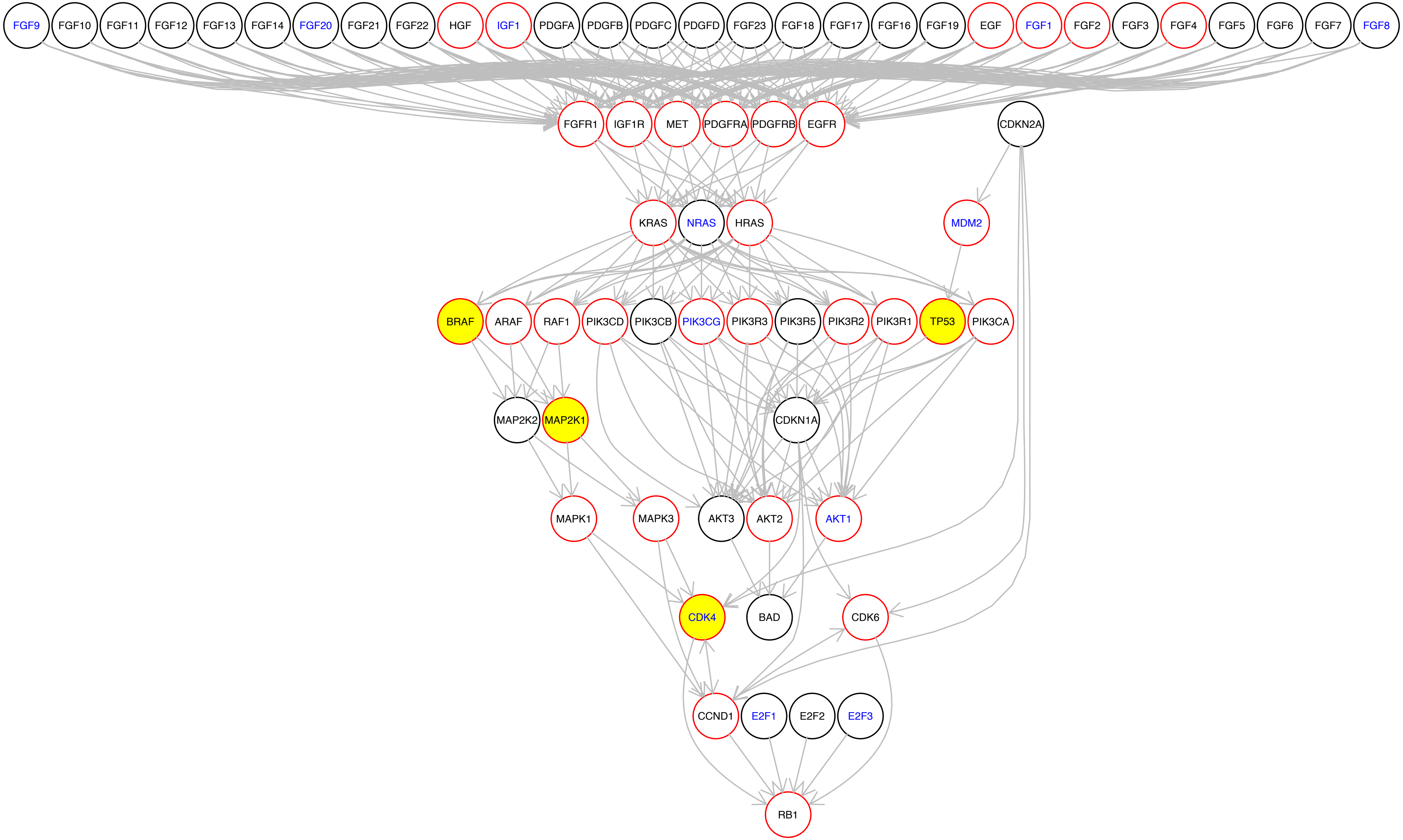

KEGG pathway = Melanoma :   tumour = LAU50_1 :   Yellow Fill = gene variant, Blue Text = expression−survival association, Red Boarder = drug

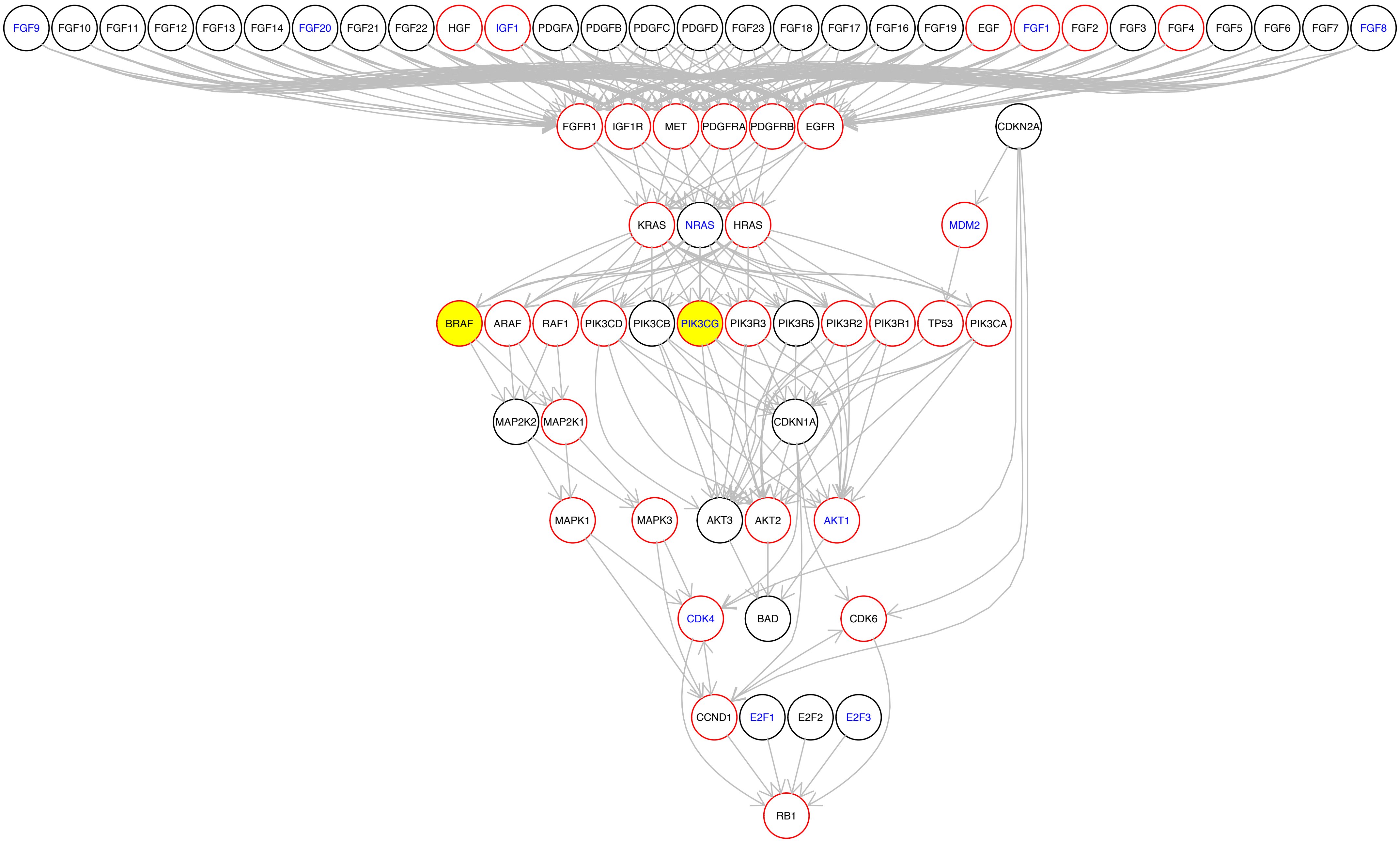

KEGG pathway = Melanoma : tumour = LAU50_2 : Yellow Fill = gene variant, Blue Text = expression−survival association, Red Boarder = drug

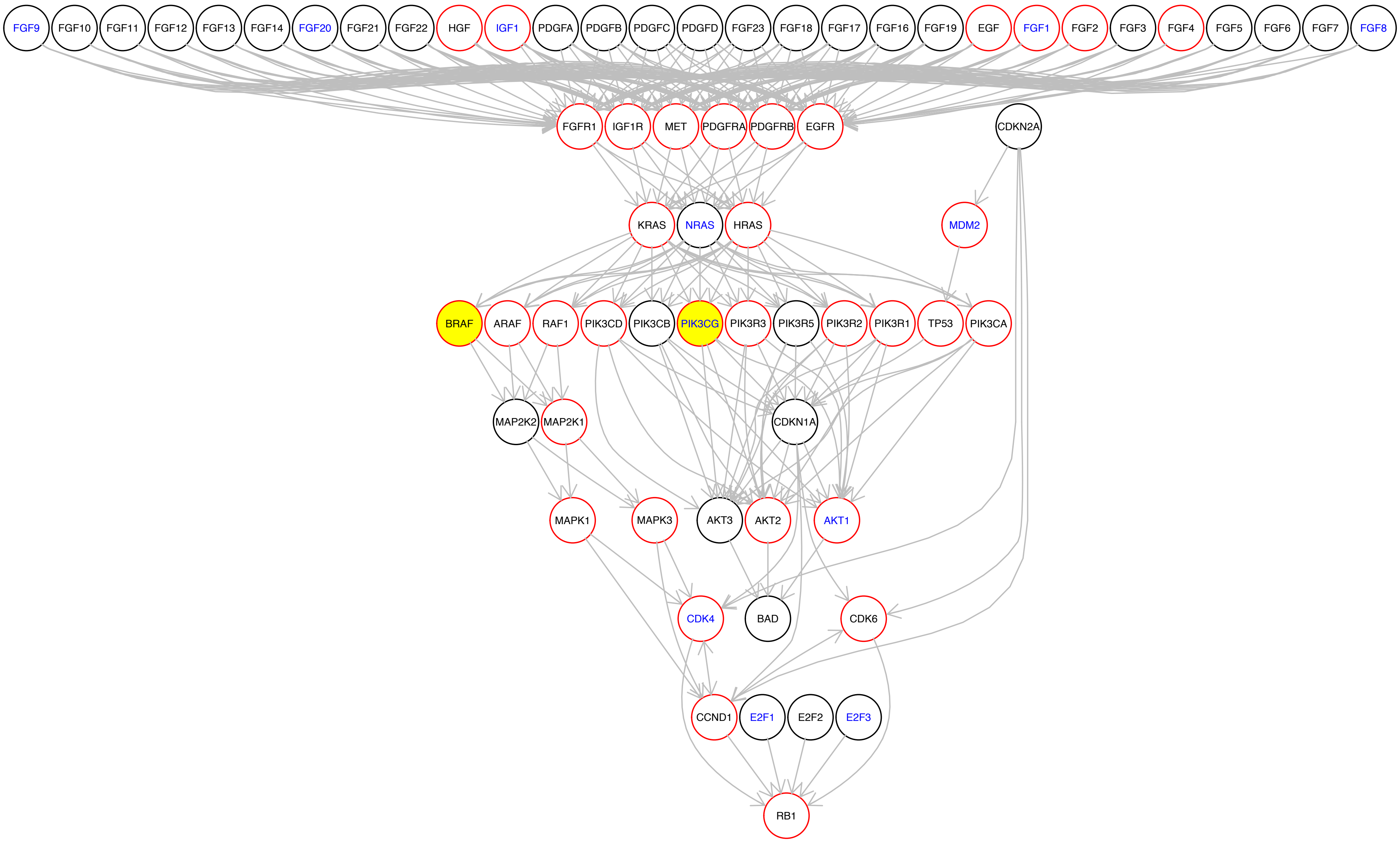

KEGG pathway = Melanoma : tumour = LAU618 : Yellow Fill = gene variant, Blue Text = expression−survival association, Red Boarder = drug

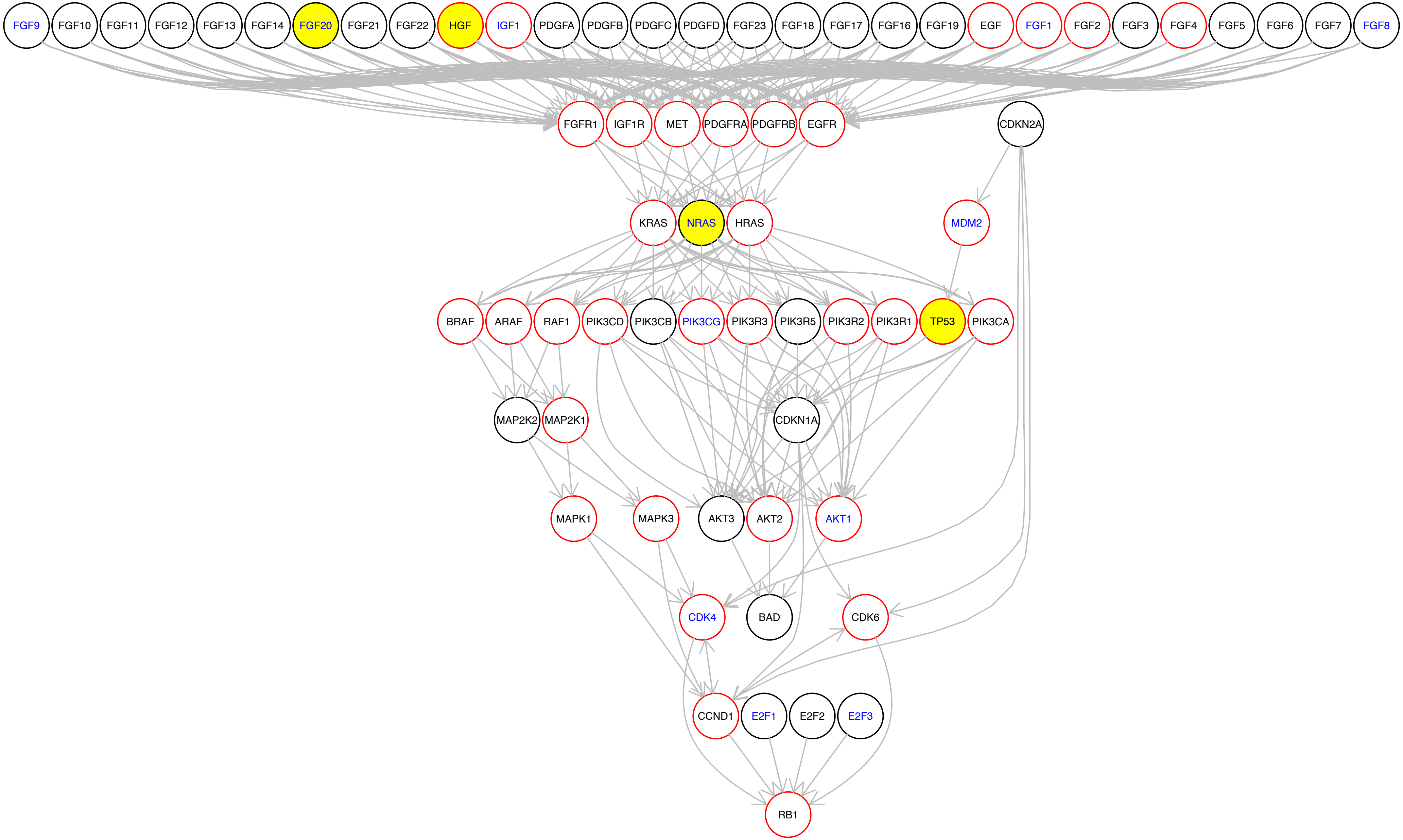

KEGG pathway = Melanoma : tumour = LAU63 : Yellow Fill = gene variant, Blue Text = expression−survival association, Red Boarder = drug

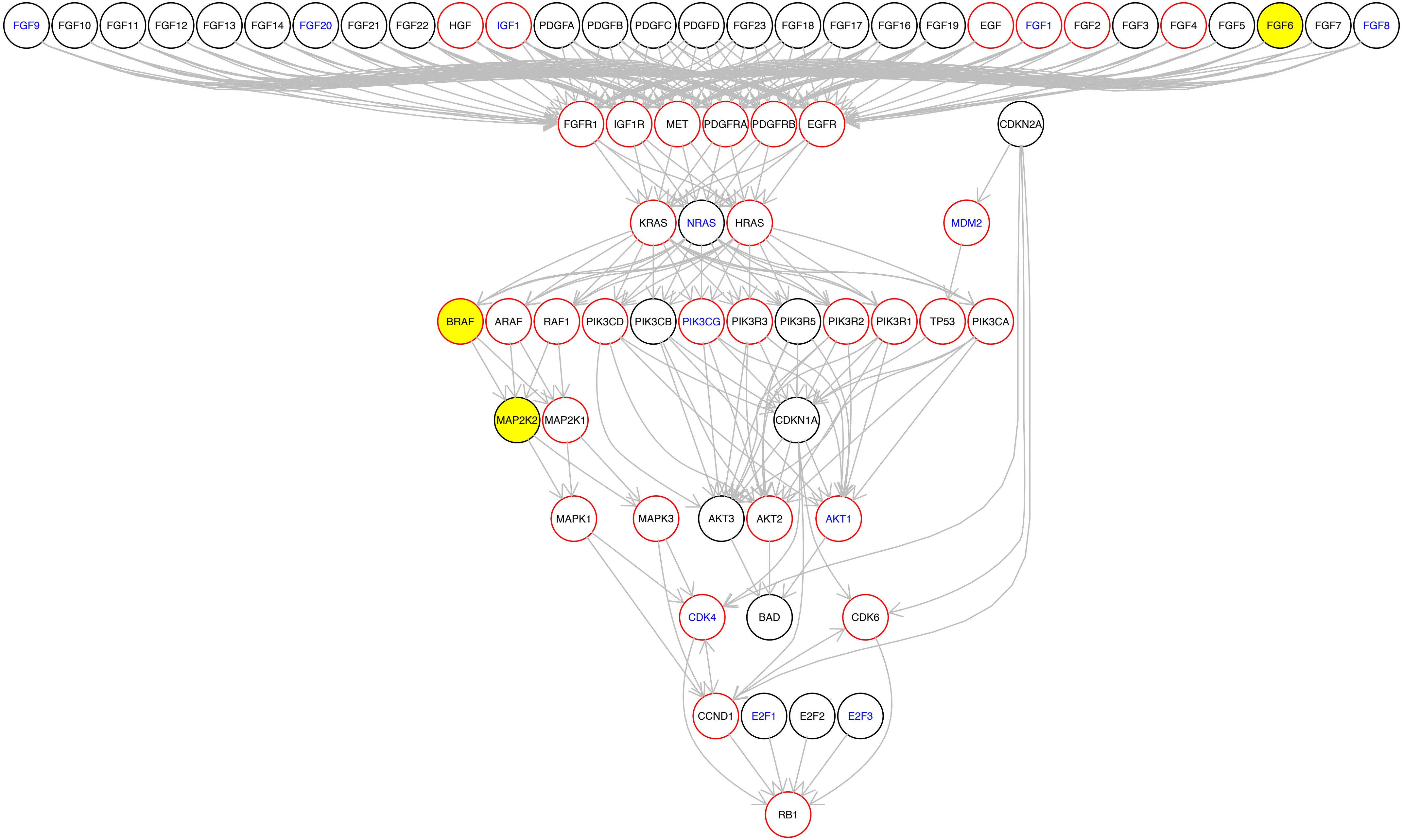

KEGG pathway = Melanoma : tumour = COLO.829 : Yellow Fill = gene variant, Blue Text = expression−survival association, Red Boarder = drug

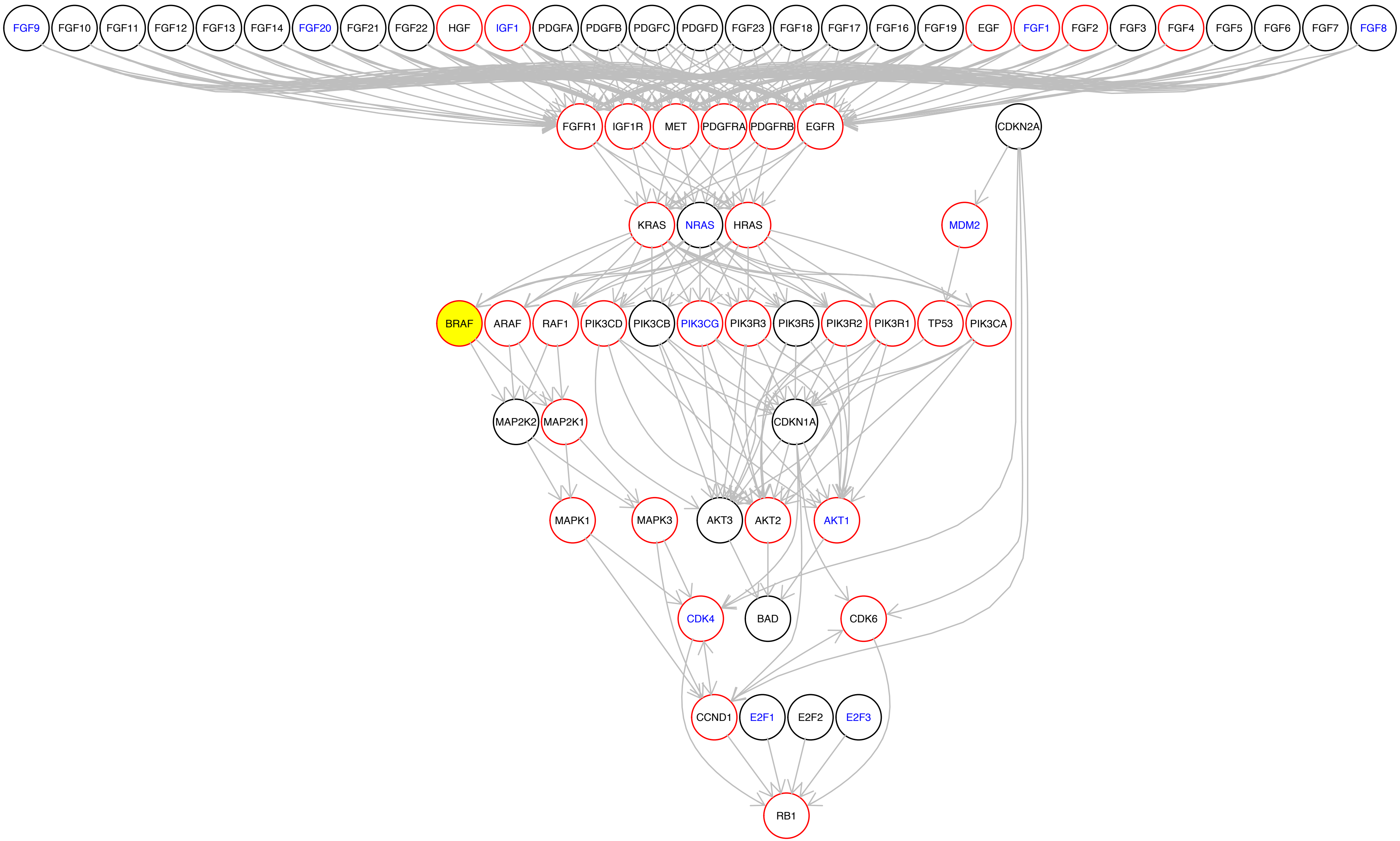

KEGG pathway = Melanoma : tumour = CP50.MEL.B : Yellow Fill = gene variant, Blue Text = expression−survival association, Red Boarder = drug

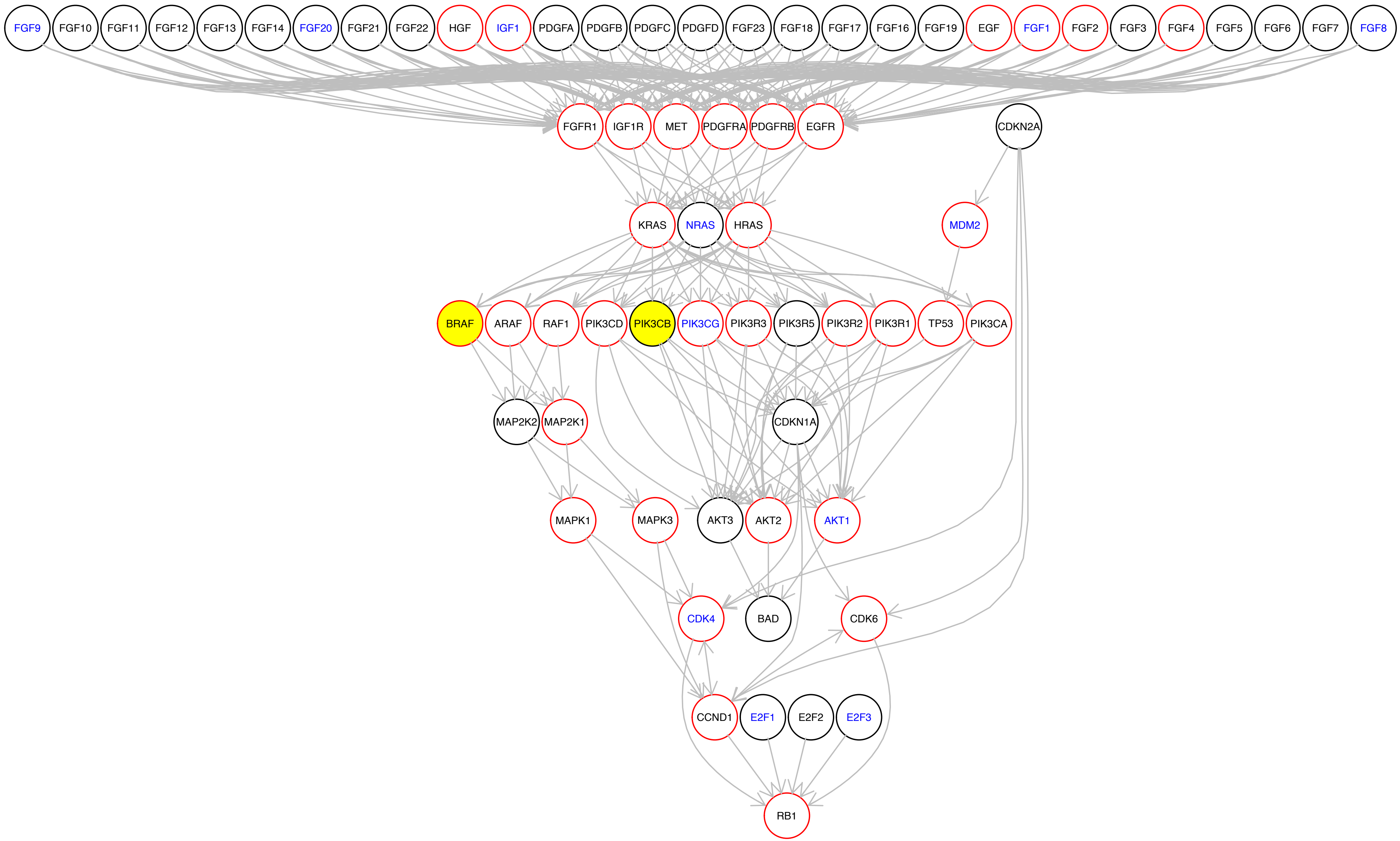

KEGG pathway = Melanoma :   tumour = CP66.MEL :   Yellow Fill = gene variant, Blue Text = expression−survival association, Red Boarder = drug

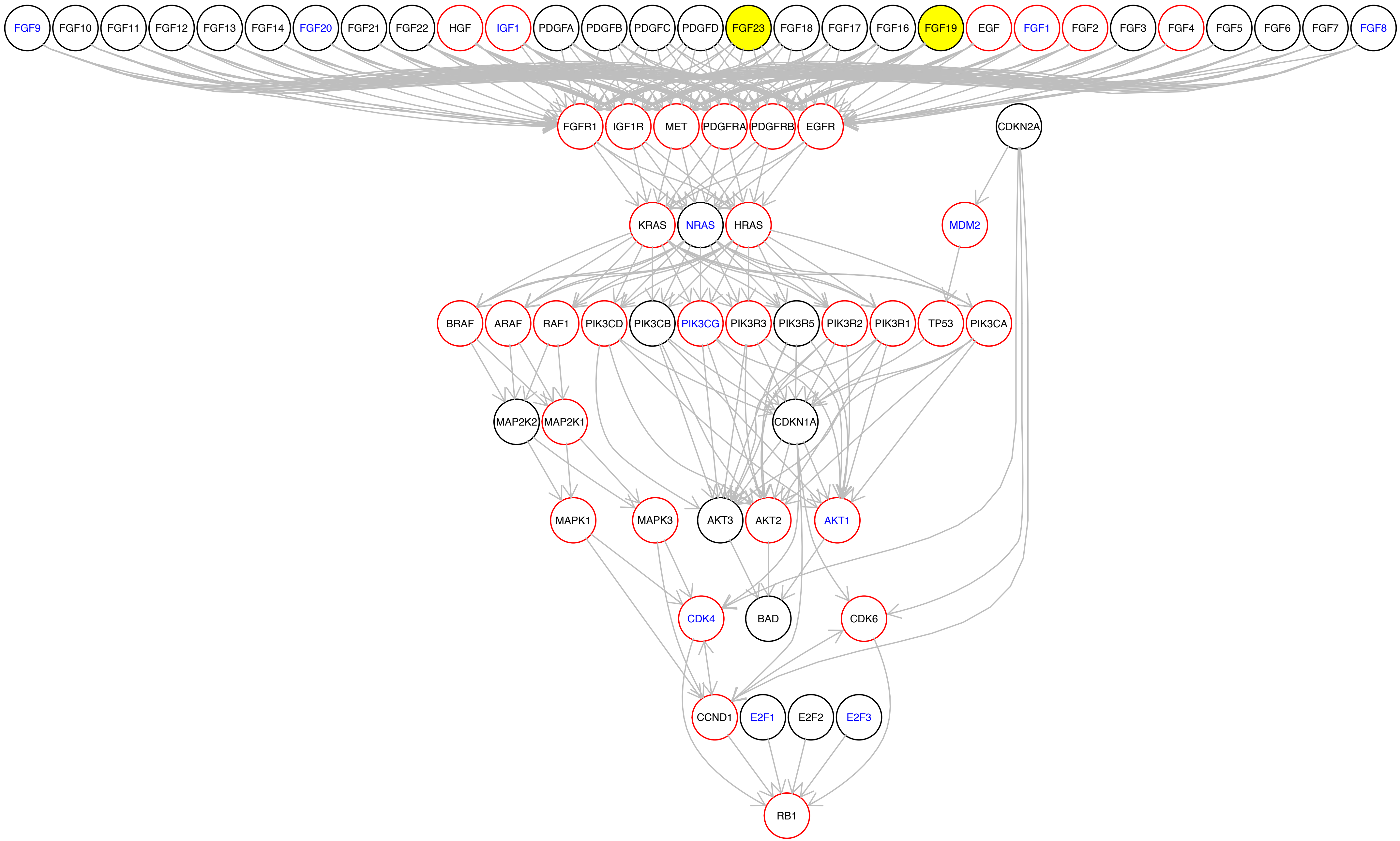

KEGG pathway = Melanoma : tumour = LB2518.MEL : Yellow Fill = gene variant, Blue Text = expression−survival association, Red Boarder = drug

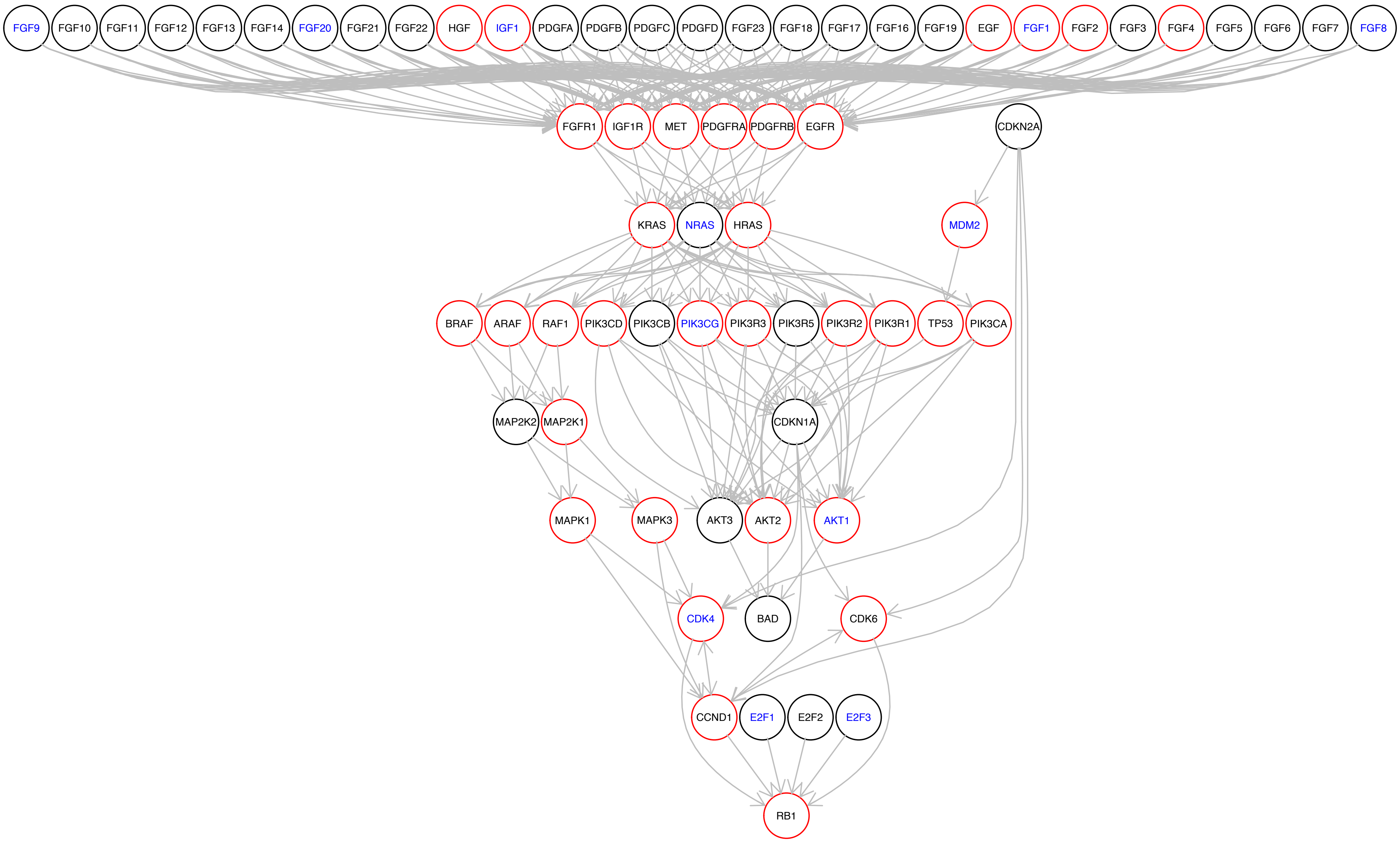

KEGG pathway = Melanoma :   tumour = LB373.MEL.D :   Yellow Fill = gene variant, Blue Text = expression−survival association, Red Boarder = drug

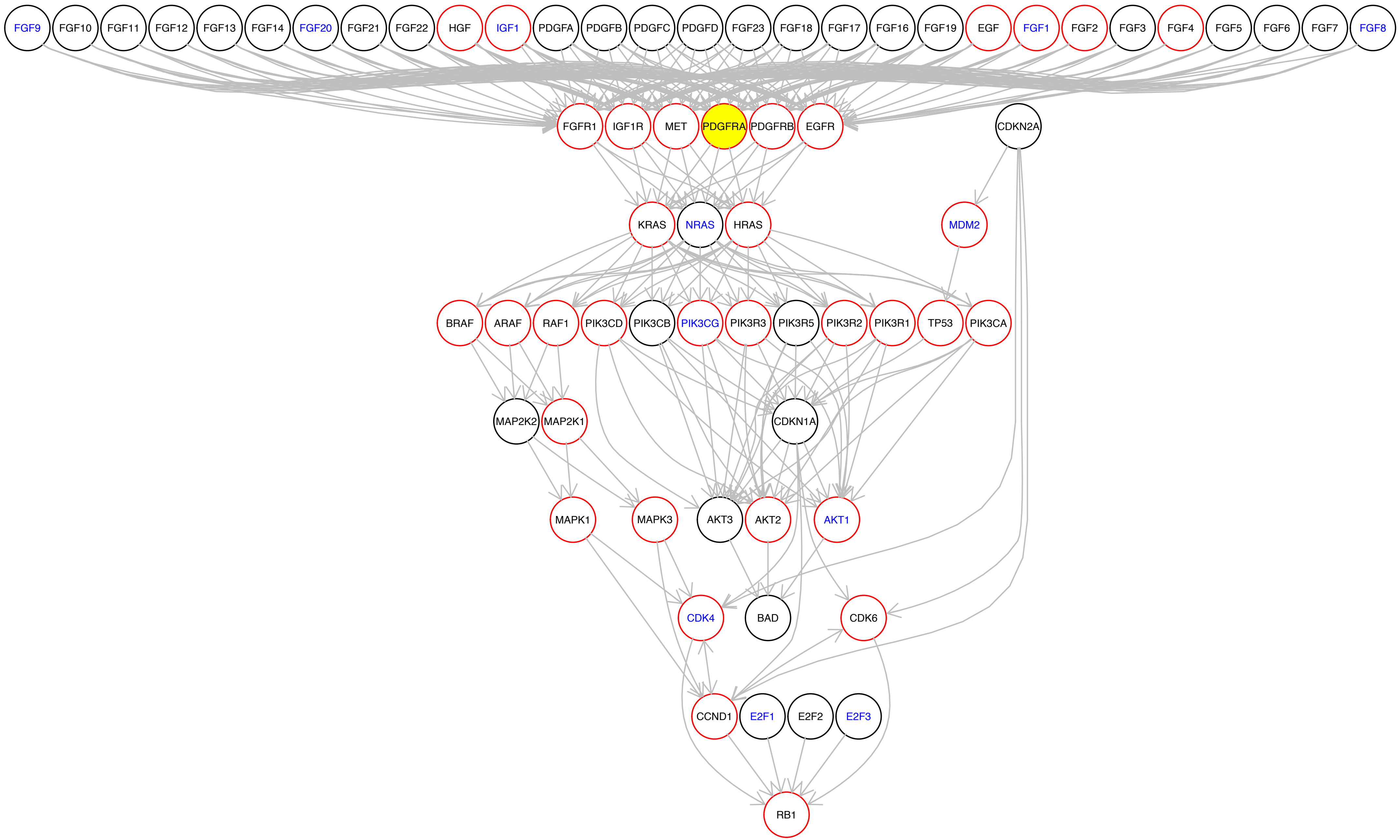

KEGG pathway = Melanoma :   tumour = MZ7.mel :   Yellow Fill = gene variant, Blue Text = expression−survival association, Red Boarder = drug

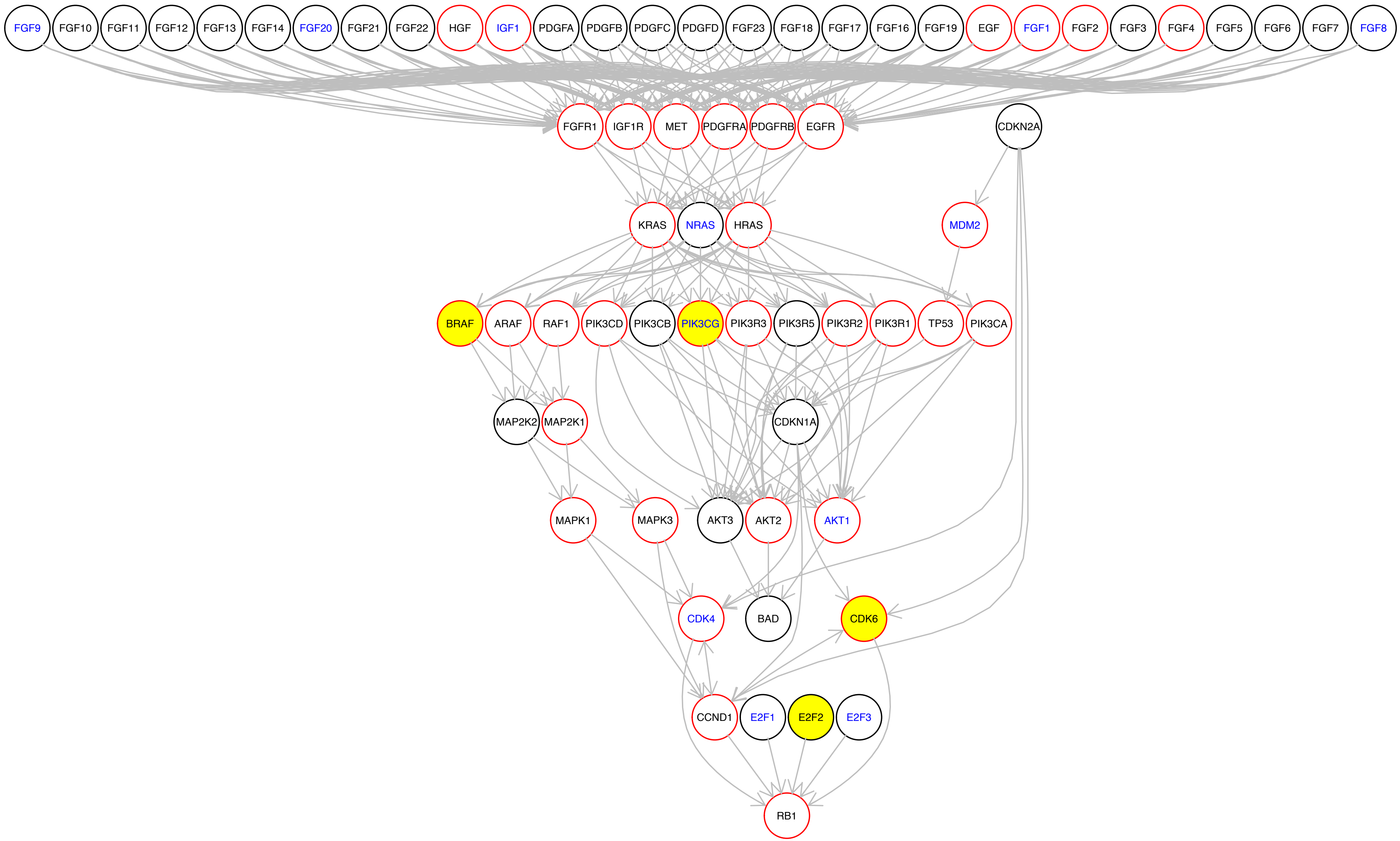

KEGG pathway = Melanoma : tumour = A02 : Yellow Fill = gene variant, Blue Text = expression−survival association, Red Boarder = drug

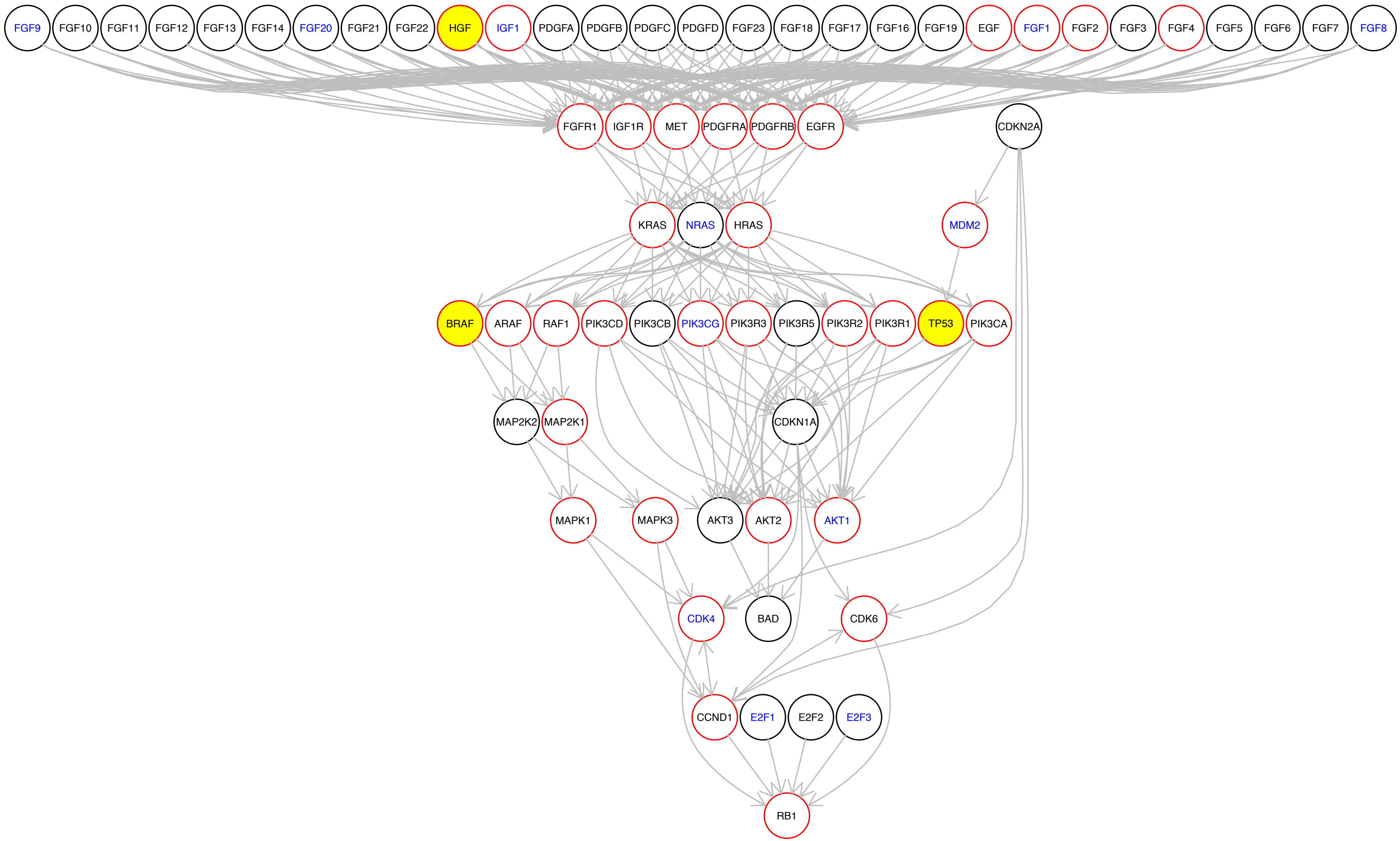

KEGG pathway = Melanoma :   tumour = A06 :   Yellow Fill = gene variant, Blue Text = expression−survival association, Red Boarder = drug

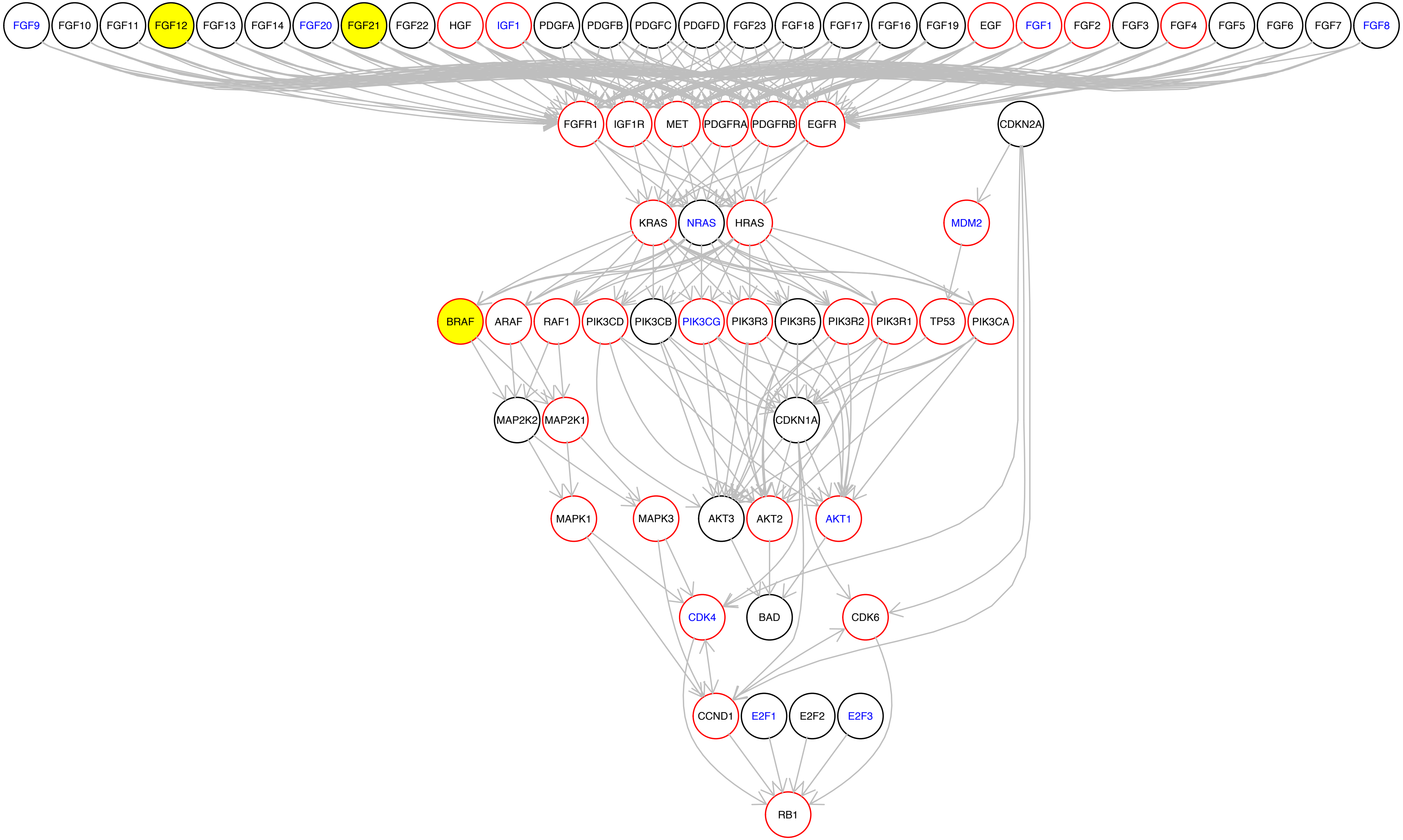

KEGG pathway = Melanoma : tumour = D05 : Yellow Fill = gene variant, Blue Text = expression−survival association, Red Boarder = drug

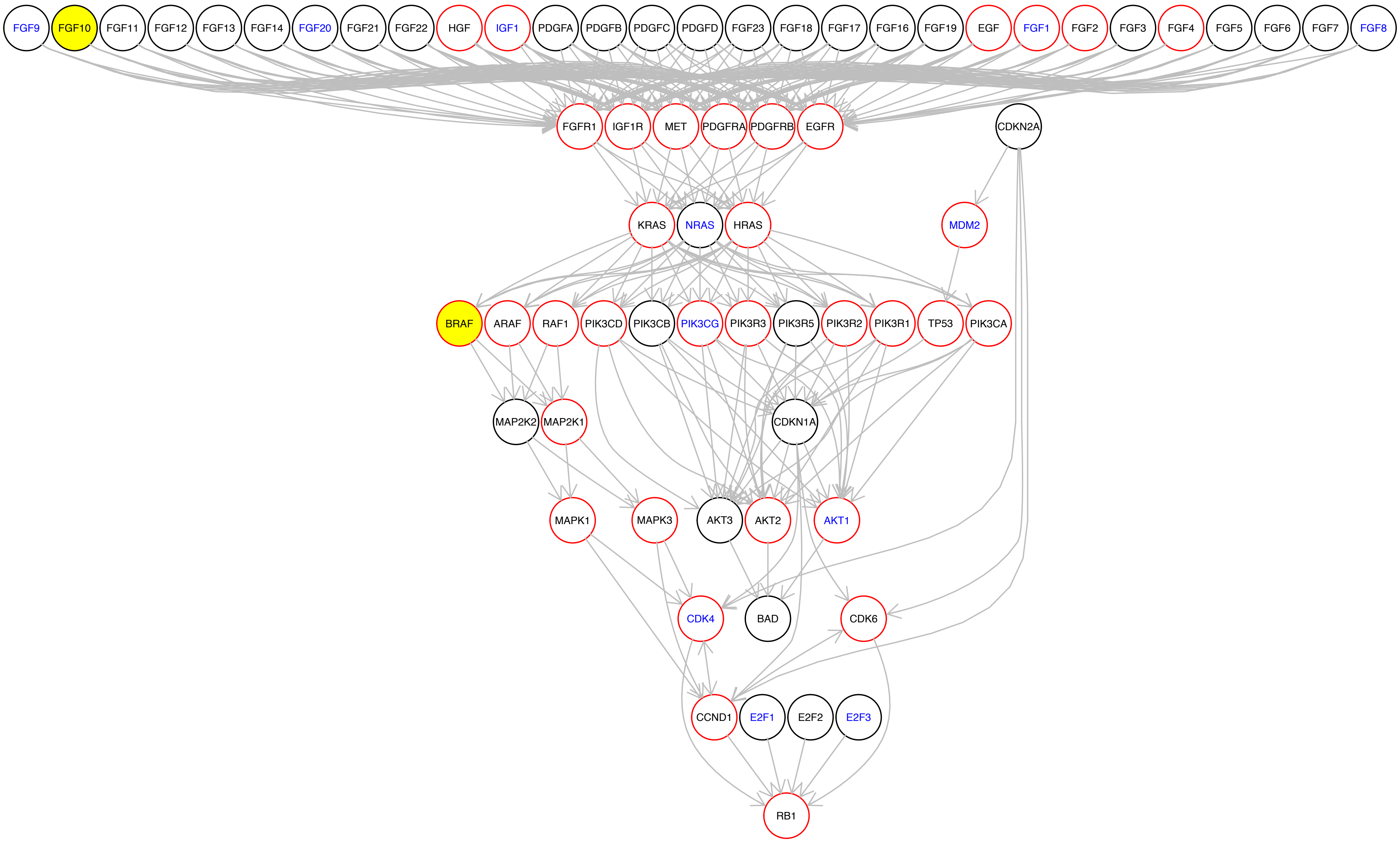

KEGG pathway = Melanoma : tumour = D14 : Yellow Fill = gene variant, Blue Text = expression−survival association, Red Boarder = drug

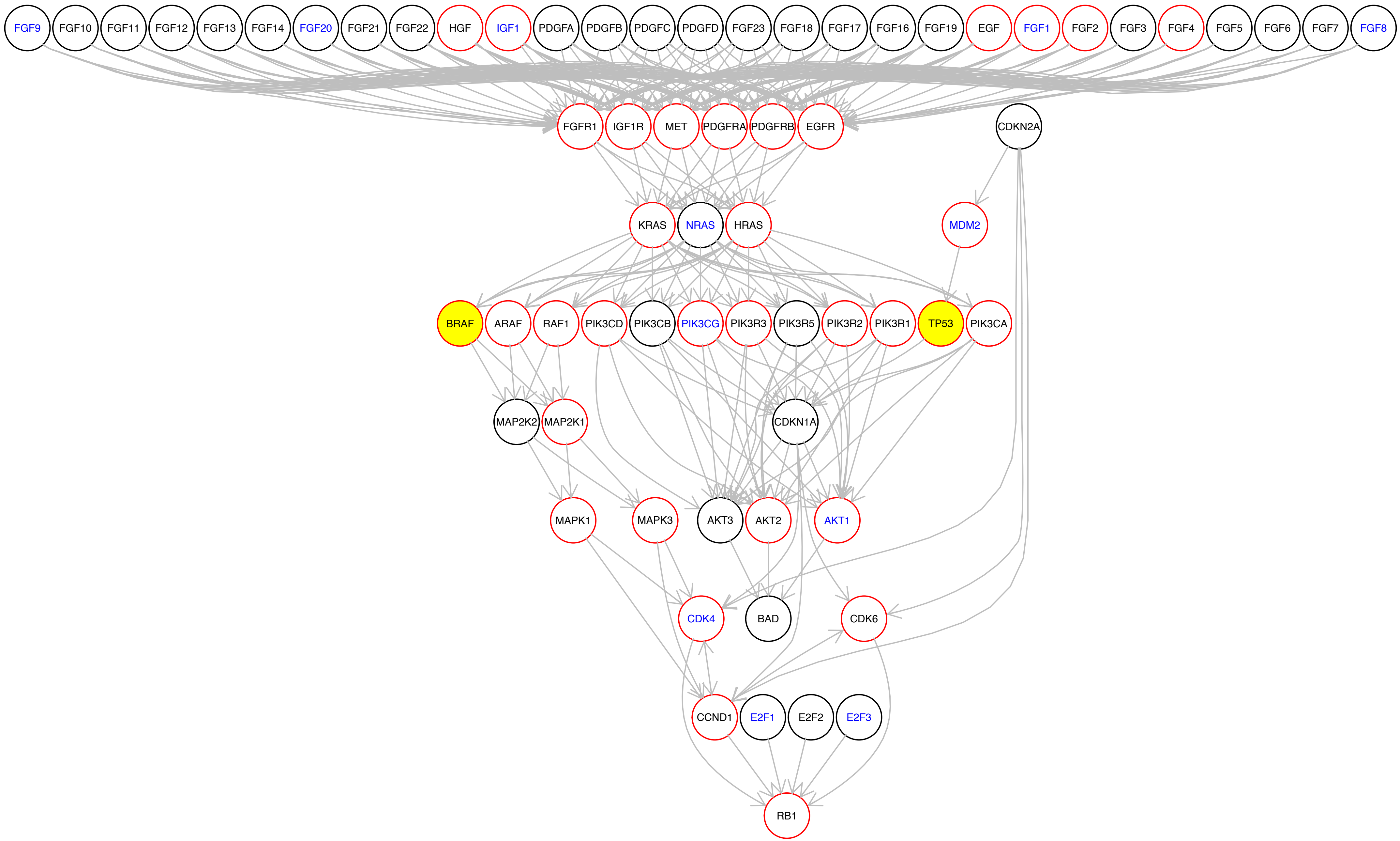

KEGG pathway = Melanoma :   tumour = D35 :   Yellow Fill = gene variant, Blue Text = expression−survival association, Red Boarder = drug

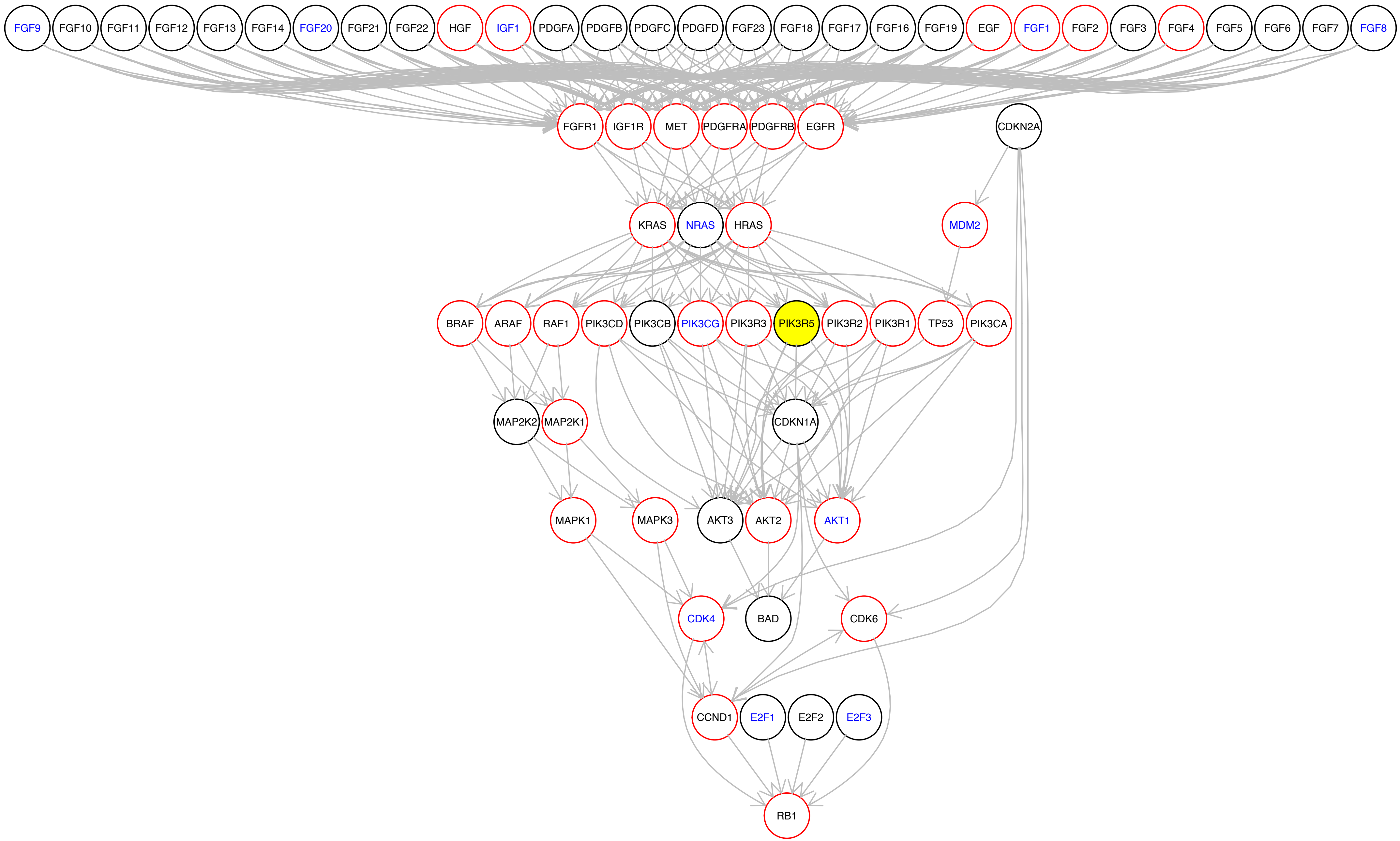

KEGG pathway = Melanoma : tumour = D36 : Yellow Fill = gene variant, Blue Text = expression−survival association, Red Boarder = drug

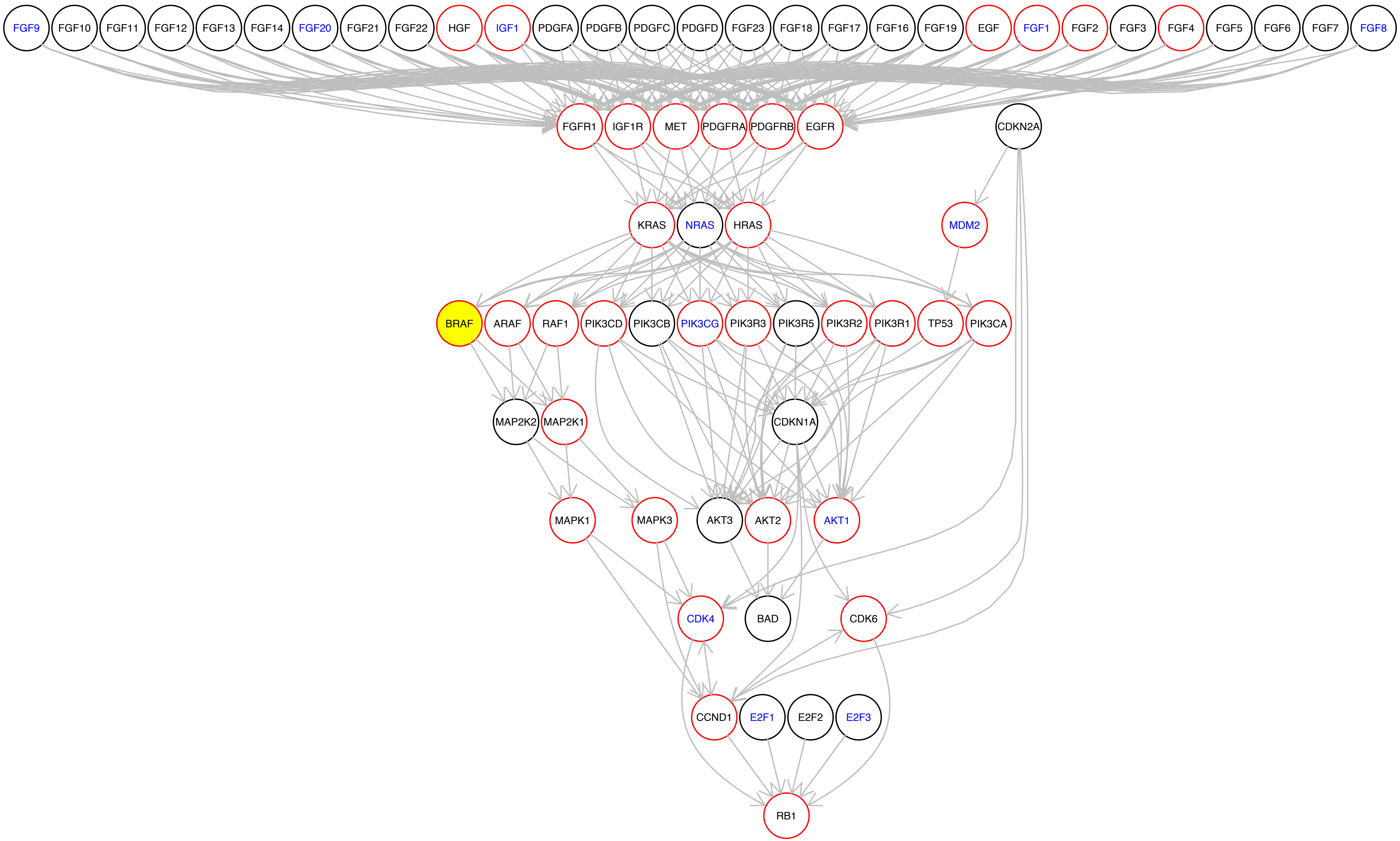

KEGG pathway = Melanoma : tumour = D41 : Yellow Fill = gene variant, Blue Text = expression–survival association, Red Boarder = drug

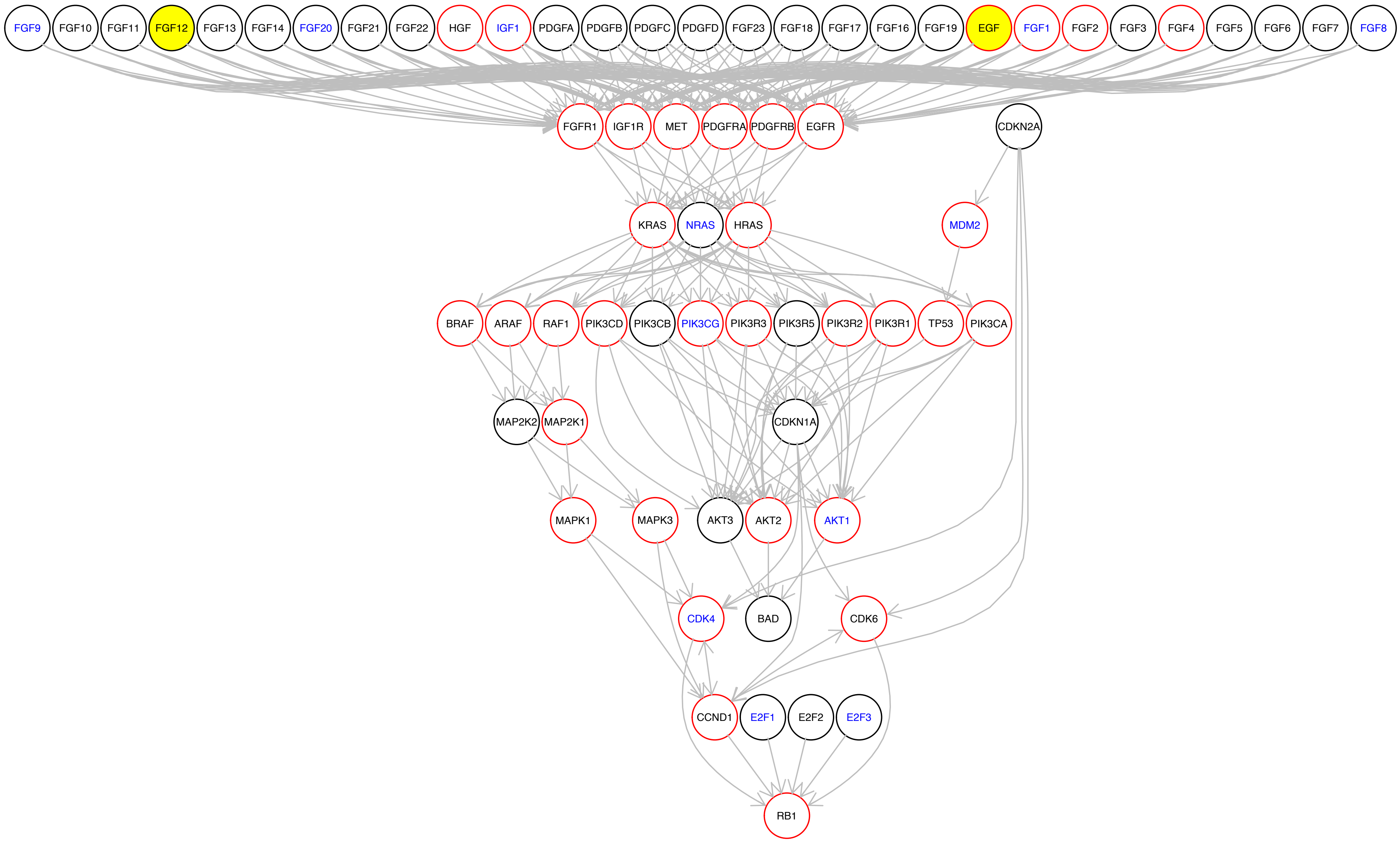

KEGG pathway = Melanoma : tumour = D49 : Yellow Fill = gene variant, Blue Text = expression−survival association, Red Boarder = drug

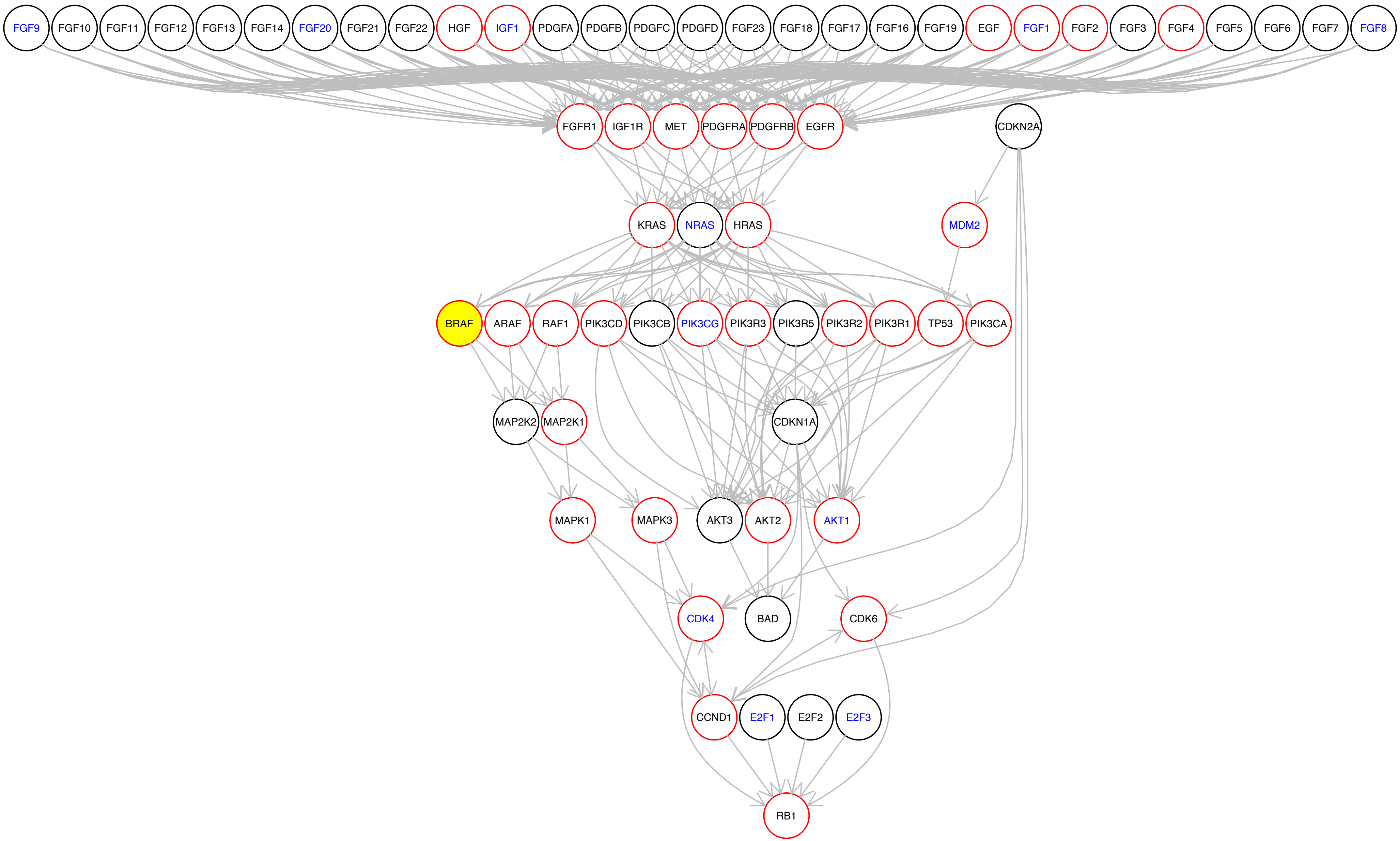

KEGG pathway = Melanoma : tumour = Turajlic : Yellow Fill = gene variant, Blue Text = expression−survival association, Red Boarder = drug

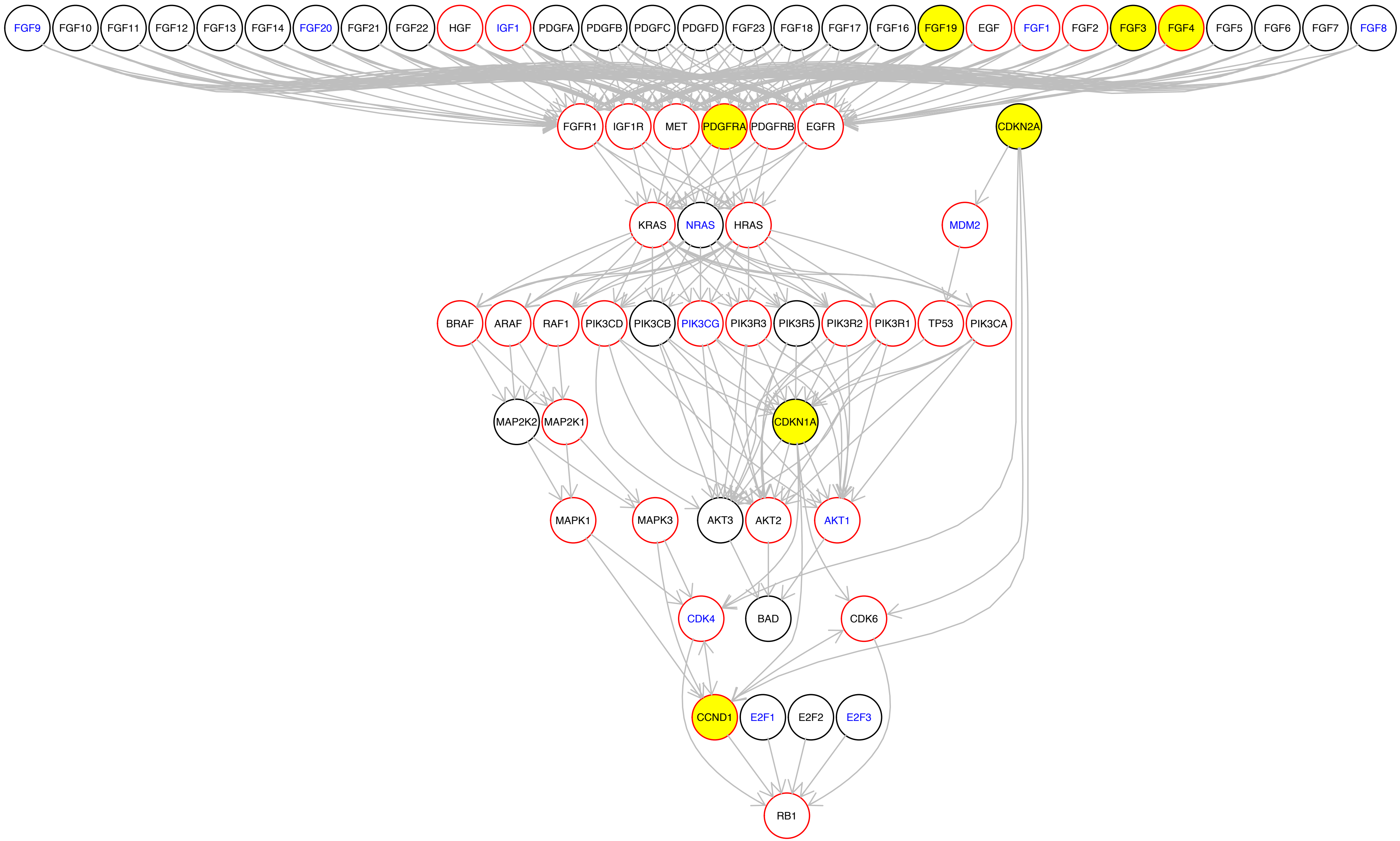

KEGG pathway = Melanoma : tumour = X01T : Yellow Fill = gene variant, Blue Text = expression−survival association, Red Boarder = drug

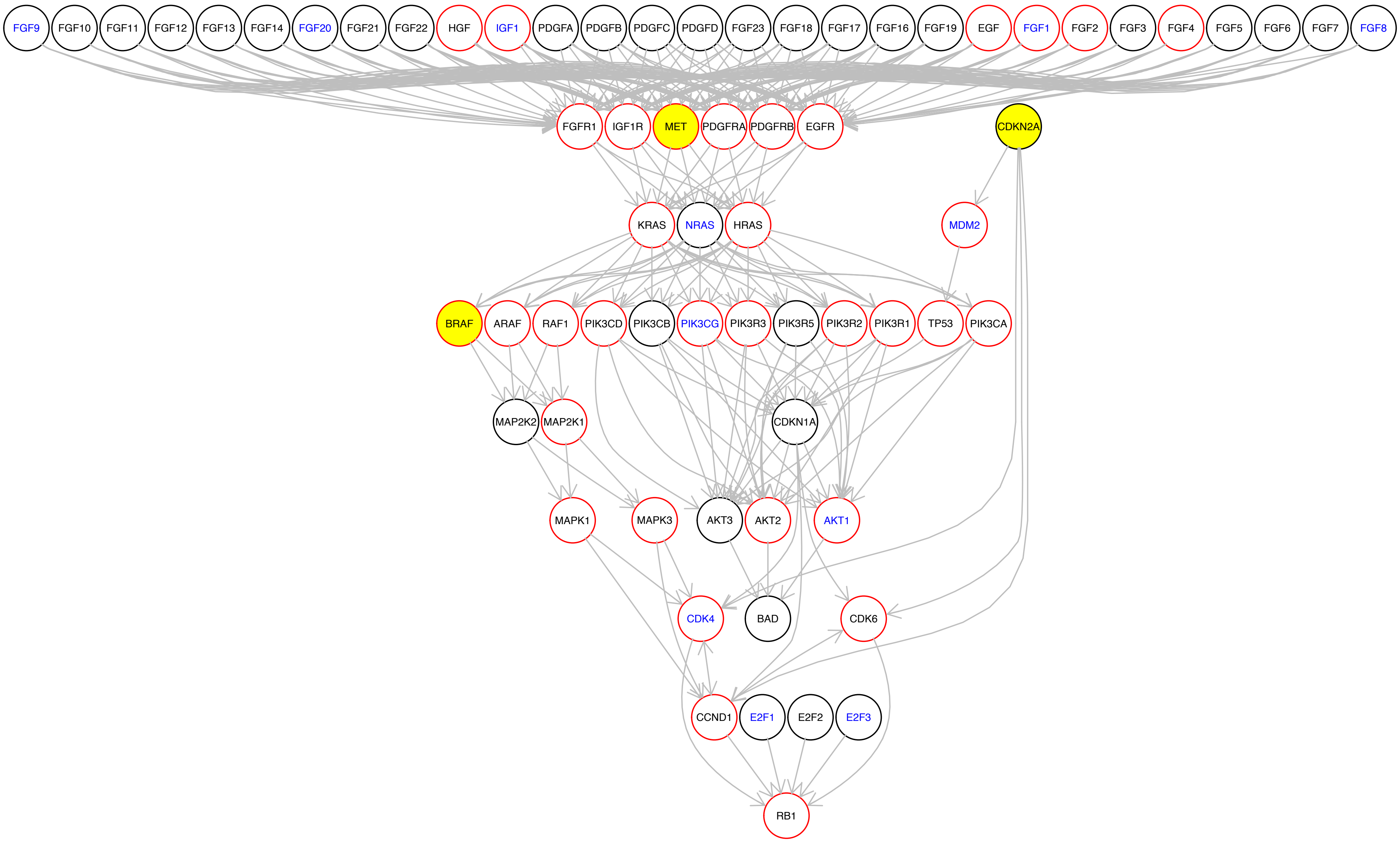

KEGG pathway = Melanoma : tumour = X05T : Yellow Fill = gene variant, Blue Text = expression−survival association, Red Boarder = drug

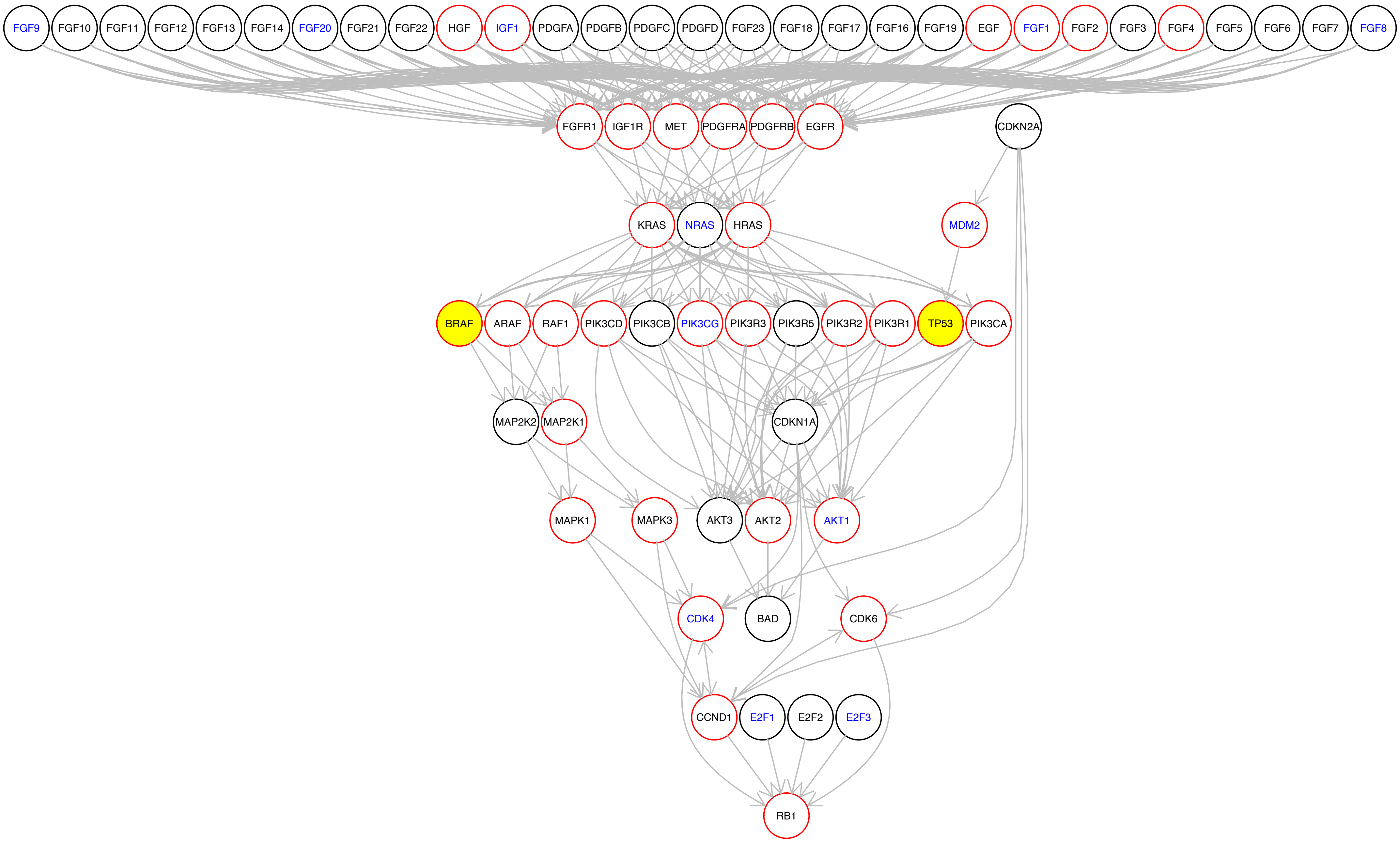

KEGG pathway = Melanoma : tumour = X09T : Yellow Fill = gene variant, Blue Text = expression–survival association, Red Boarder = drug

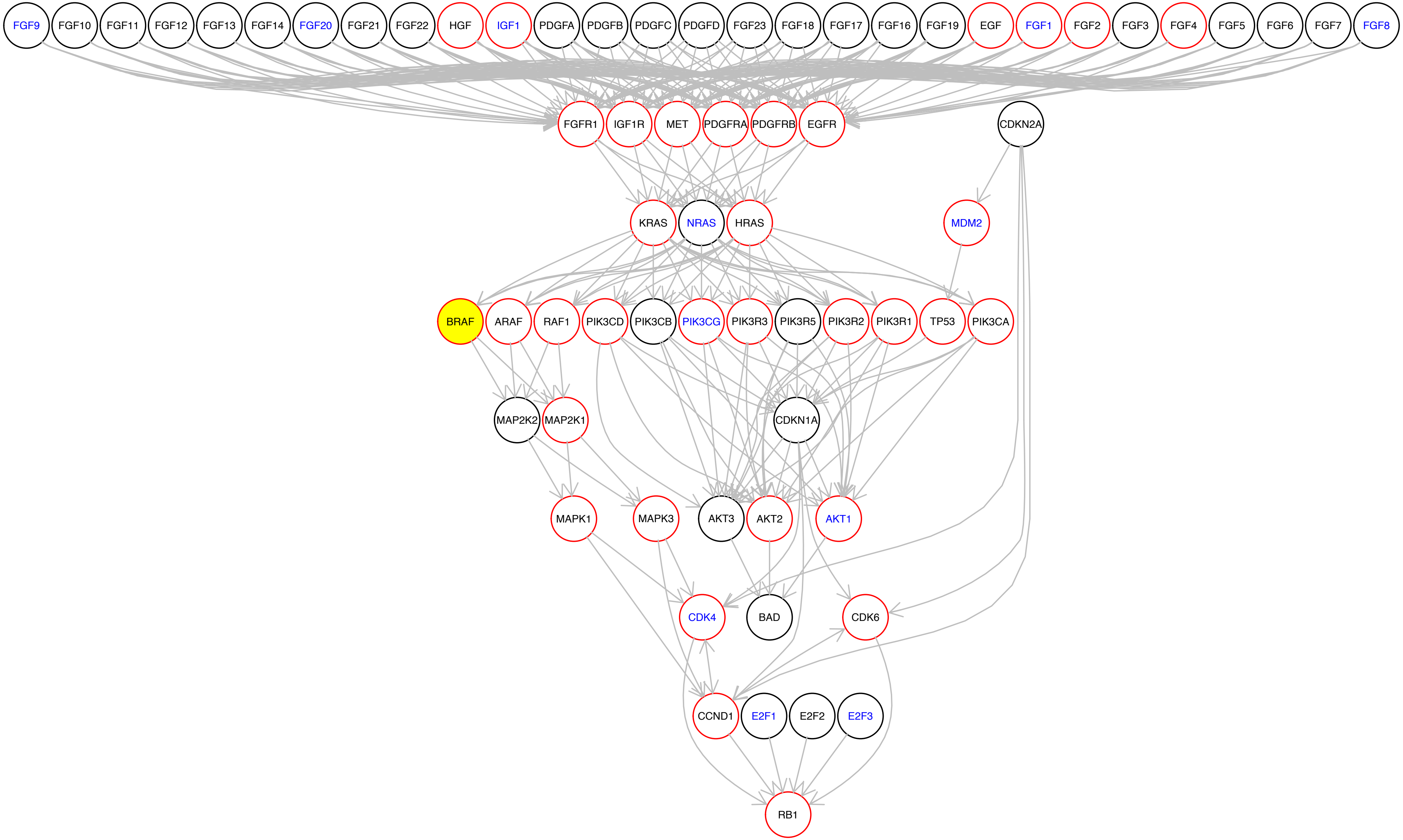

KEGG pathway = Melanoma : tumour = X12T : Yellow Fill = gene variant, Blue Text = expression−survival association, Red Boarder = drug

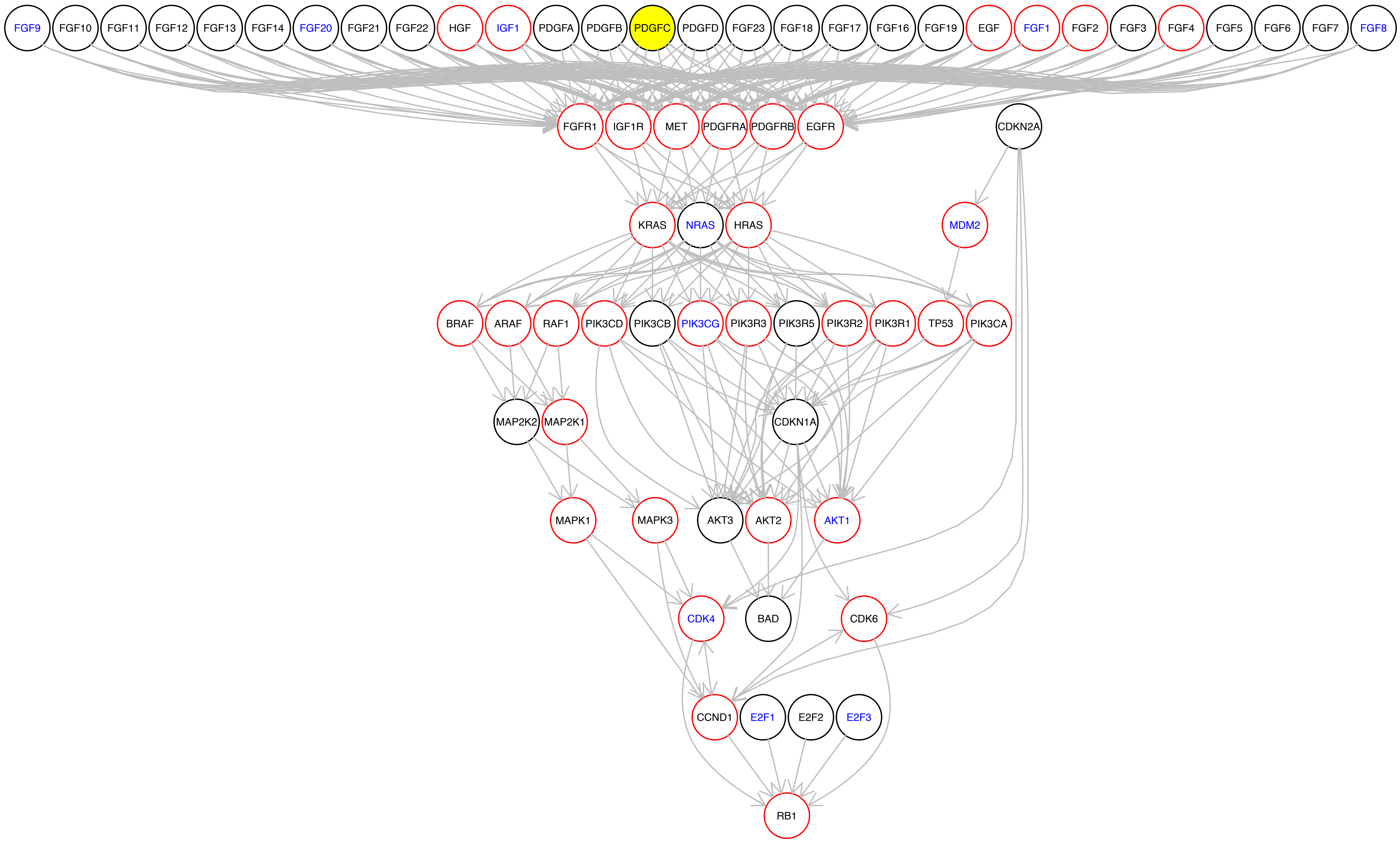

KEGG pathway = Melanoma : tumour = X18T : Yellow Fill = gene variant, Blue Text = expression−survival association, Red Boarder = drug

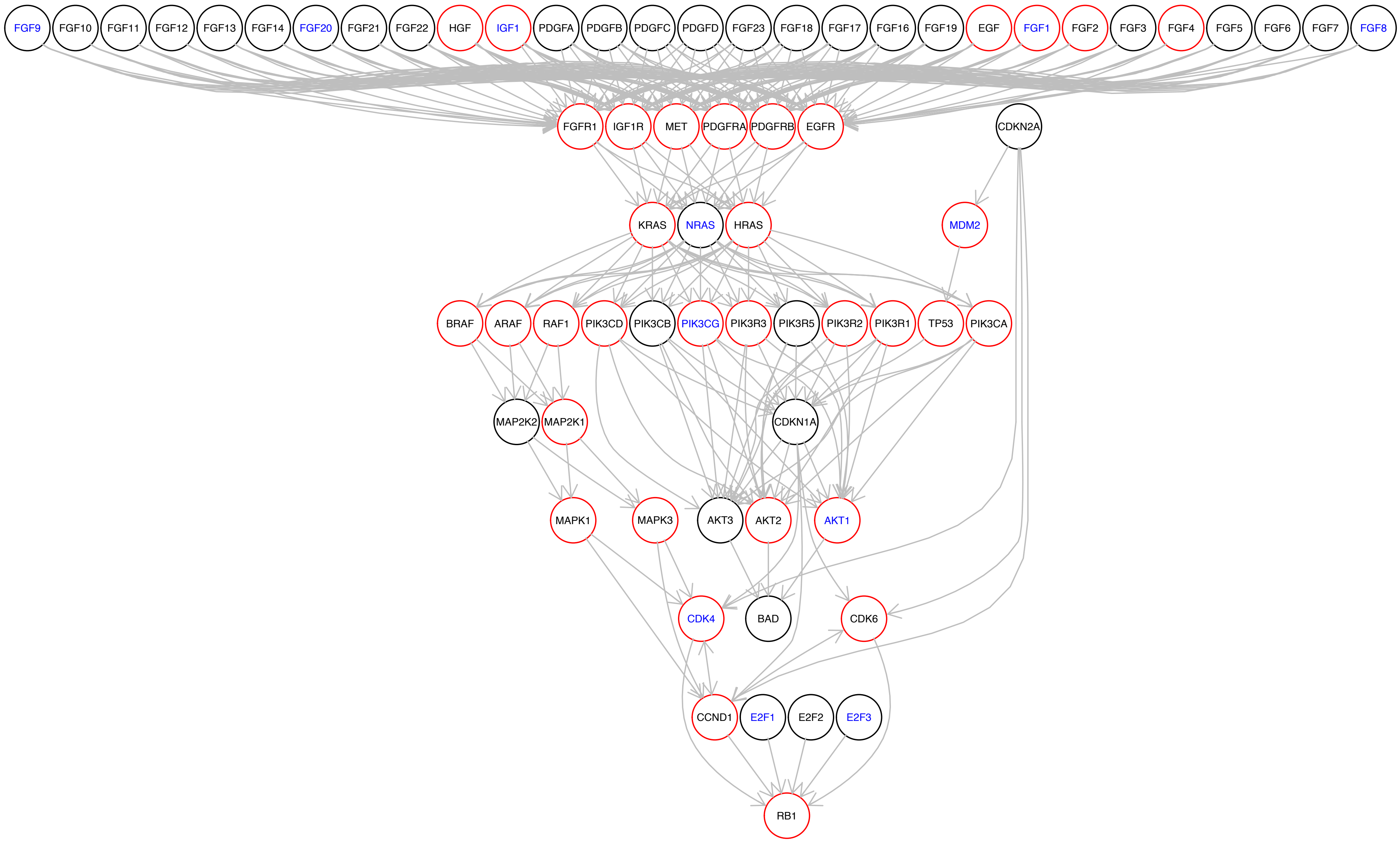

KEGG pathway = Melanoma : tumour = X22T : Yellow Fill = gene variant, Blue Text = expression−survival association, Red Boarder = drug

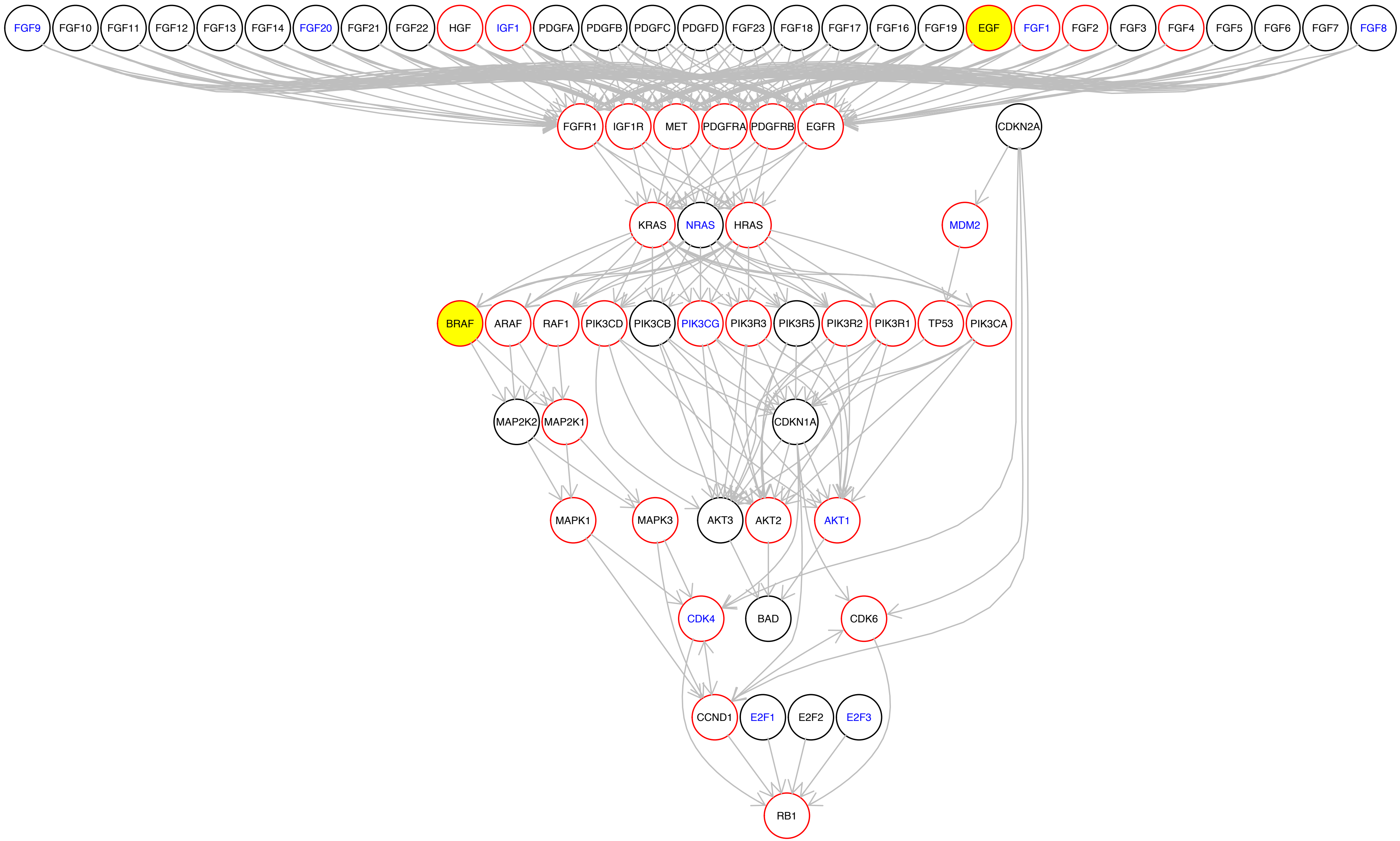

KEGG pathway = Melanoma :   tumour = X24T :   Yellow Fill = gene variant, Blue Text = expression−survival association, Red Boarder = drug

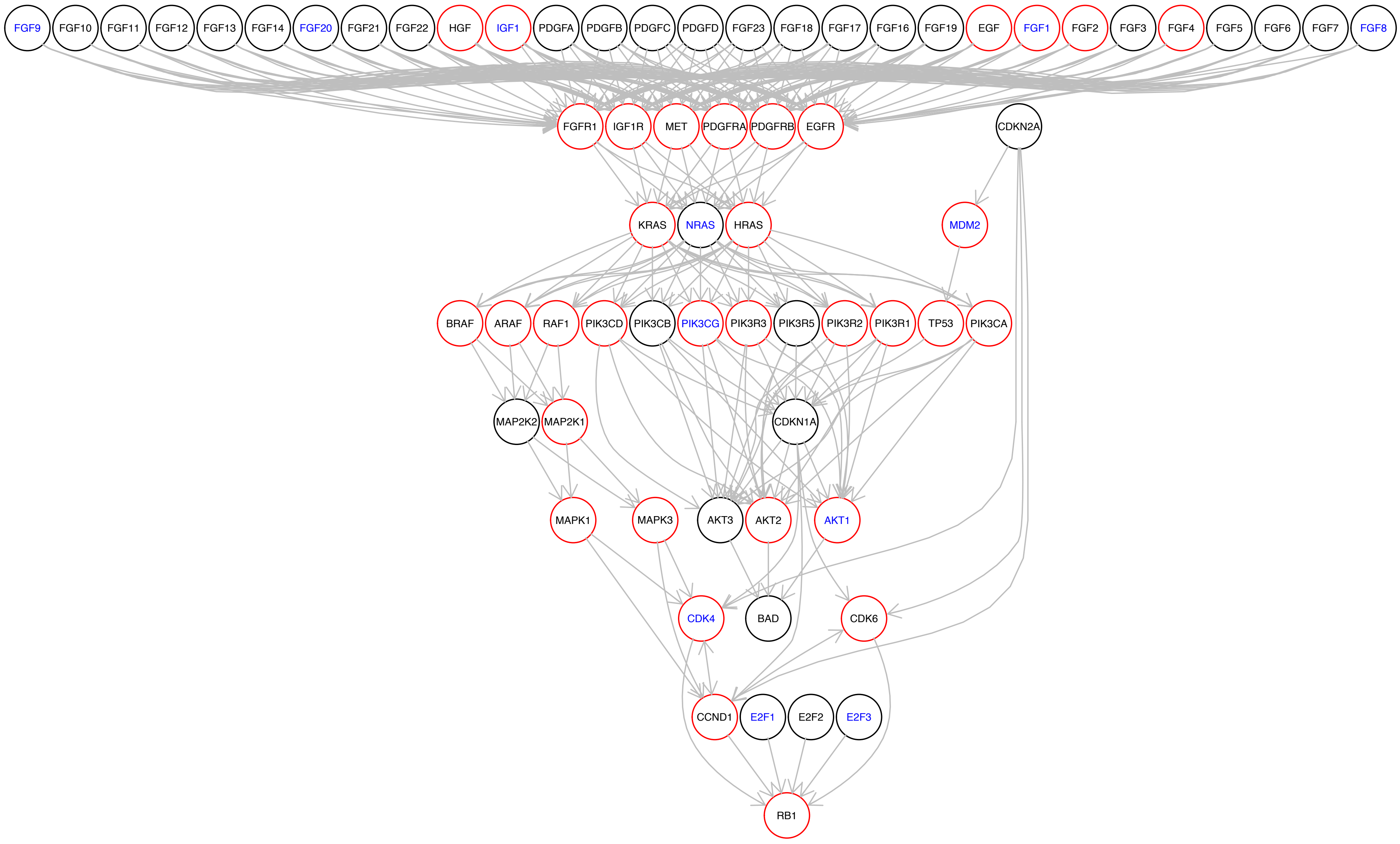

KEGG pathway = Melanoma : tumour = X35T : Yellow Fill = gene variant, Blue Text = expression−survival association, Red Boarder = drug

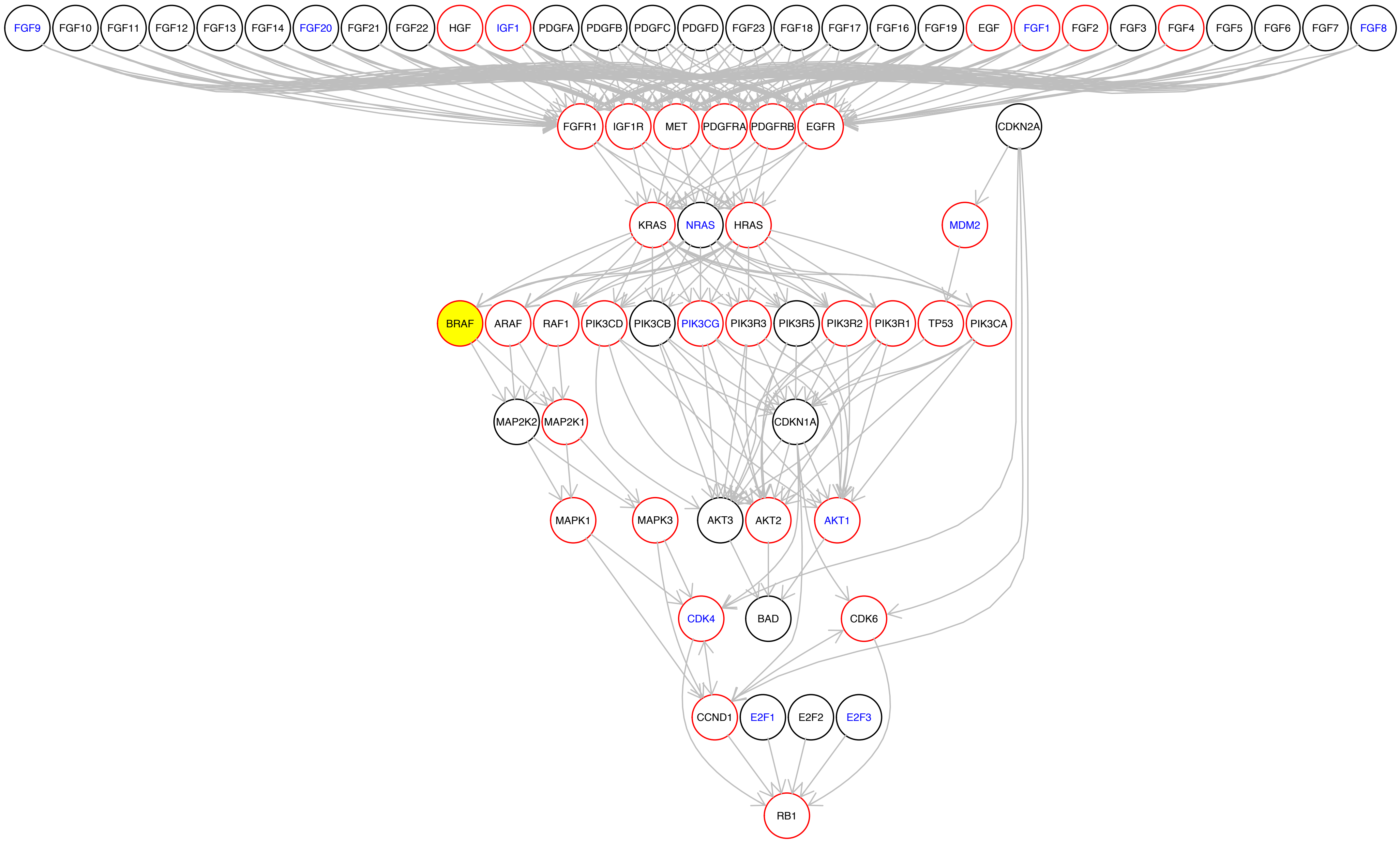

KEGG pathway = Melanoma : tumour = X43T : Yellow Fill = gene variant, Blue Text = expression−survival association, Red Boarder = drug

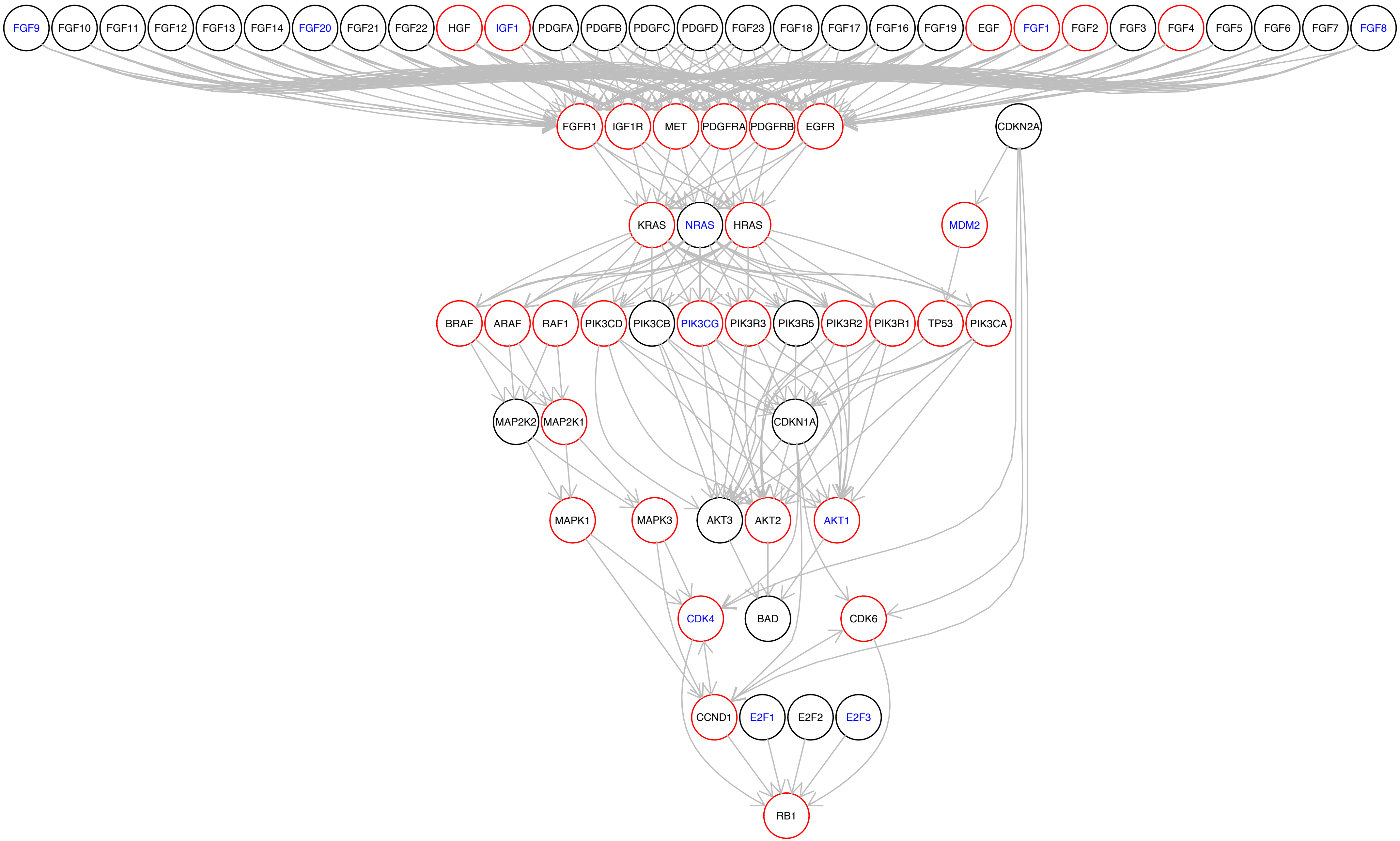

KEGG pathway = Melanoma : tumour = X51T : Yellow Fill = gene variant, Blue Text = expression−survival association, Red Boarder = drug

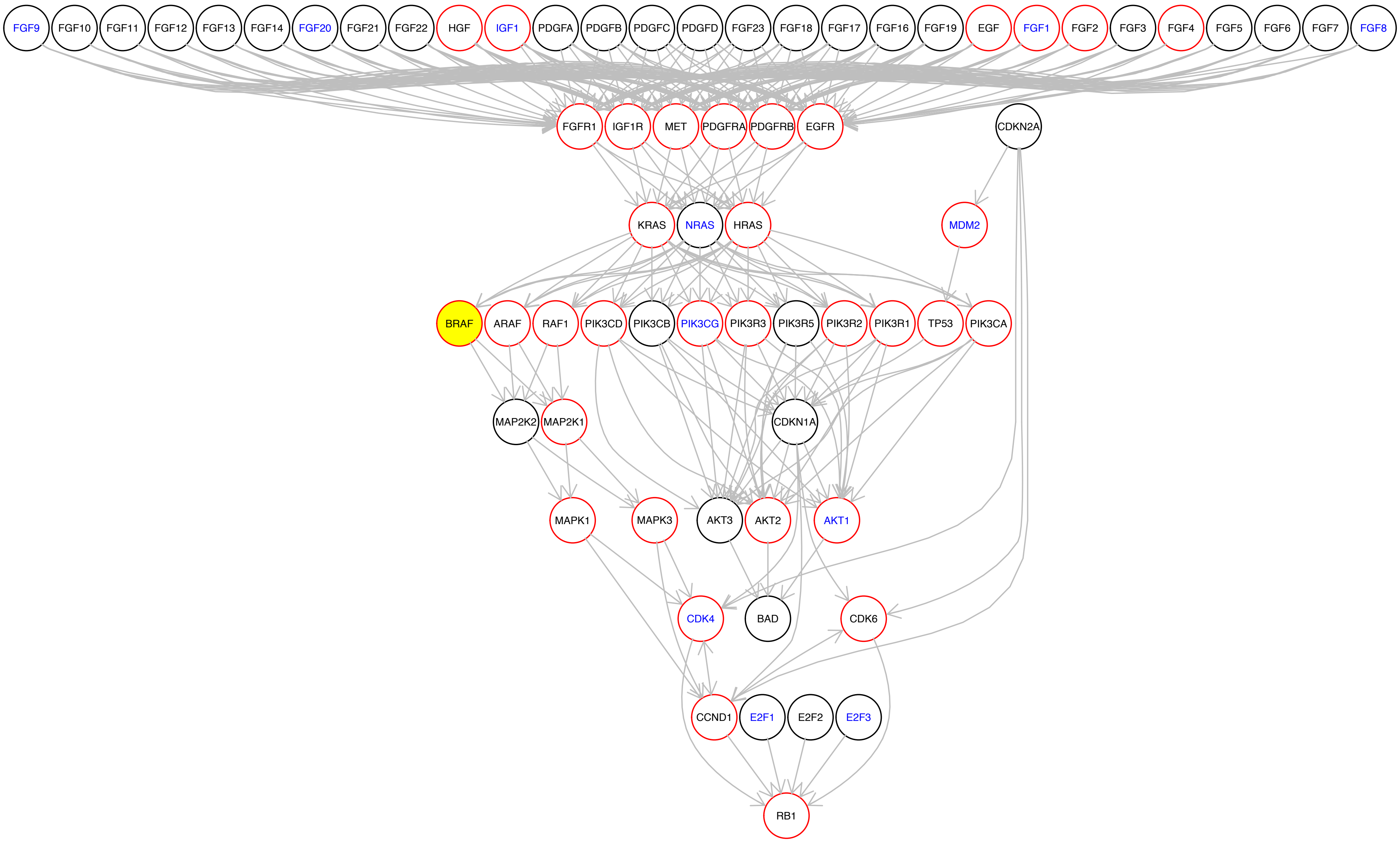

KEGG pathway = Melanoma : tumour = X60T : Yellow Fill = gene variant, Blue Text = expression−survival association, Red Boarder = drug

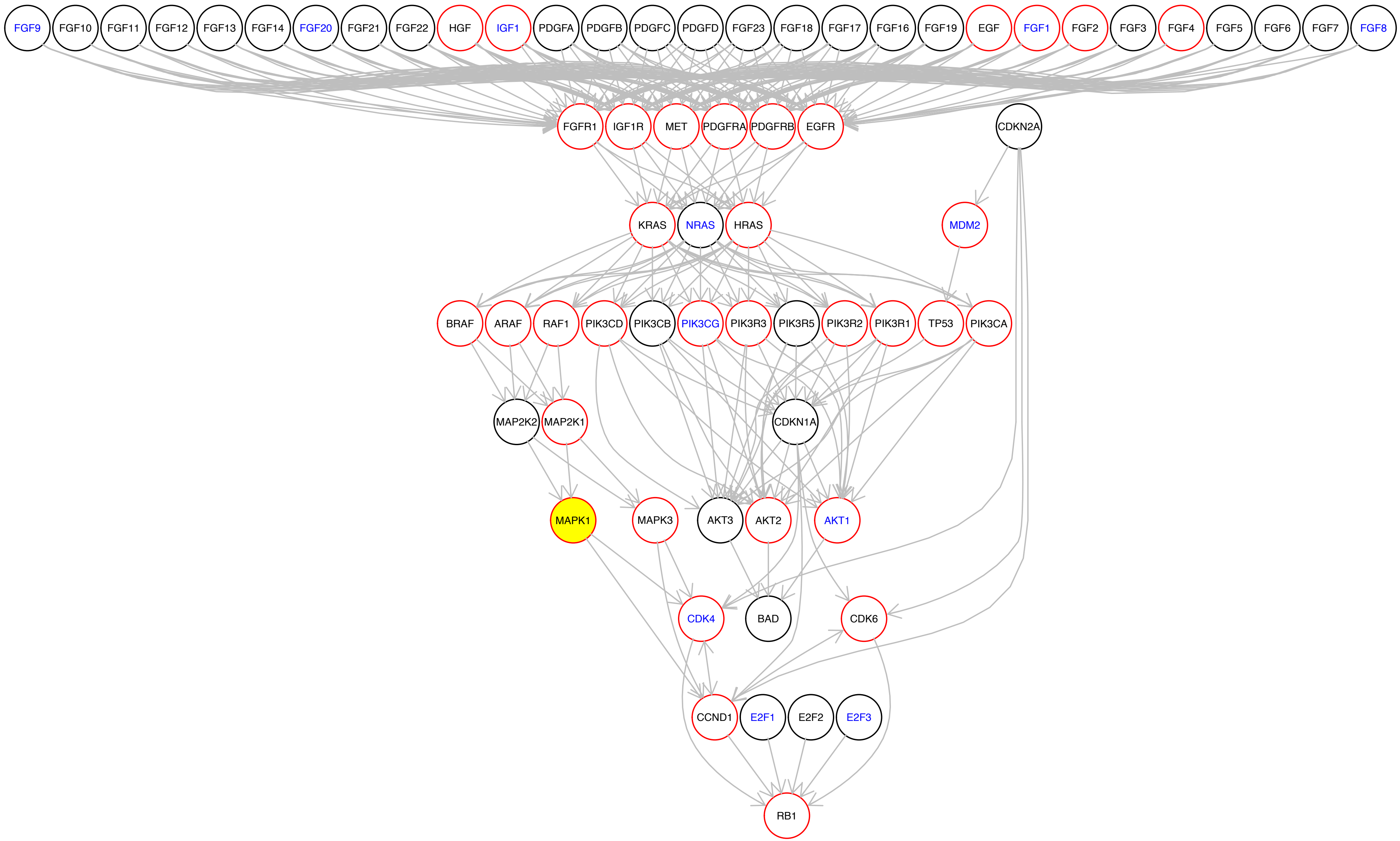

KEGG pathway = Melanoma : tumour = X91T : Yellow Fill = gene variant, Blue Text = expression−survival association, Red Boarder = drug

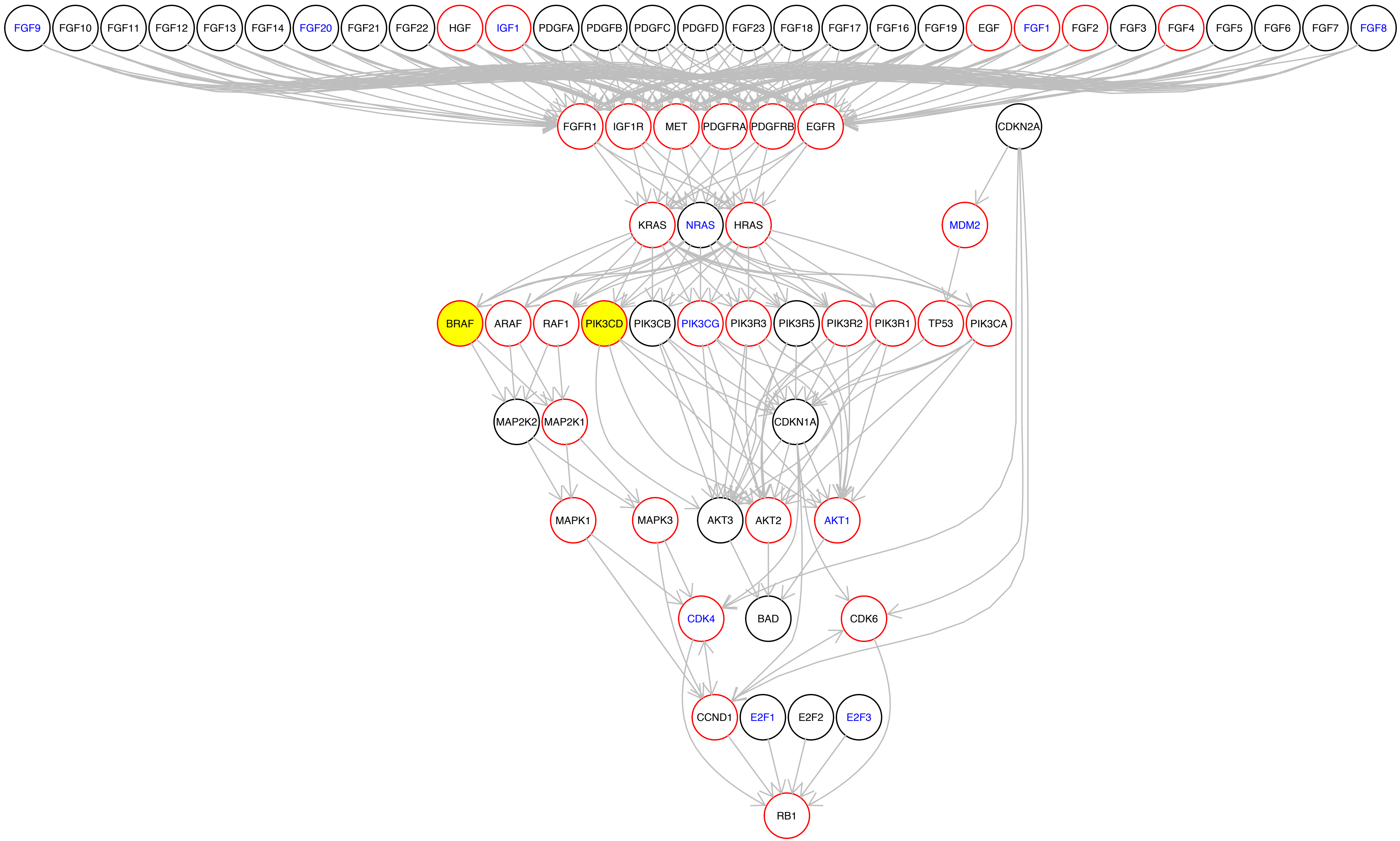

KEGG pathway = Melanoma : tumour = X93T : Yellow Fill = gene variant, Blue Text = expression-survival association, Red Boarder = drug

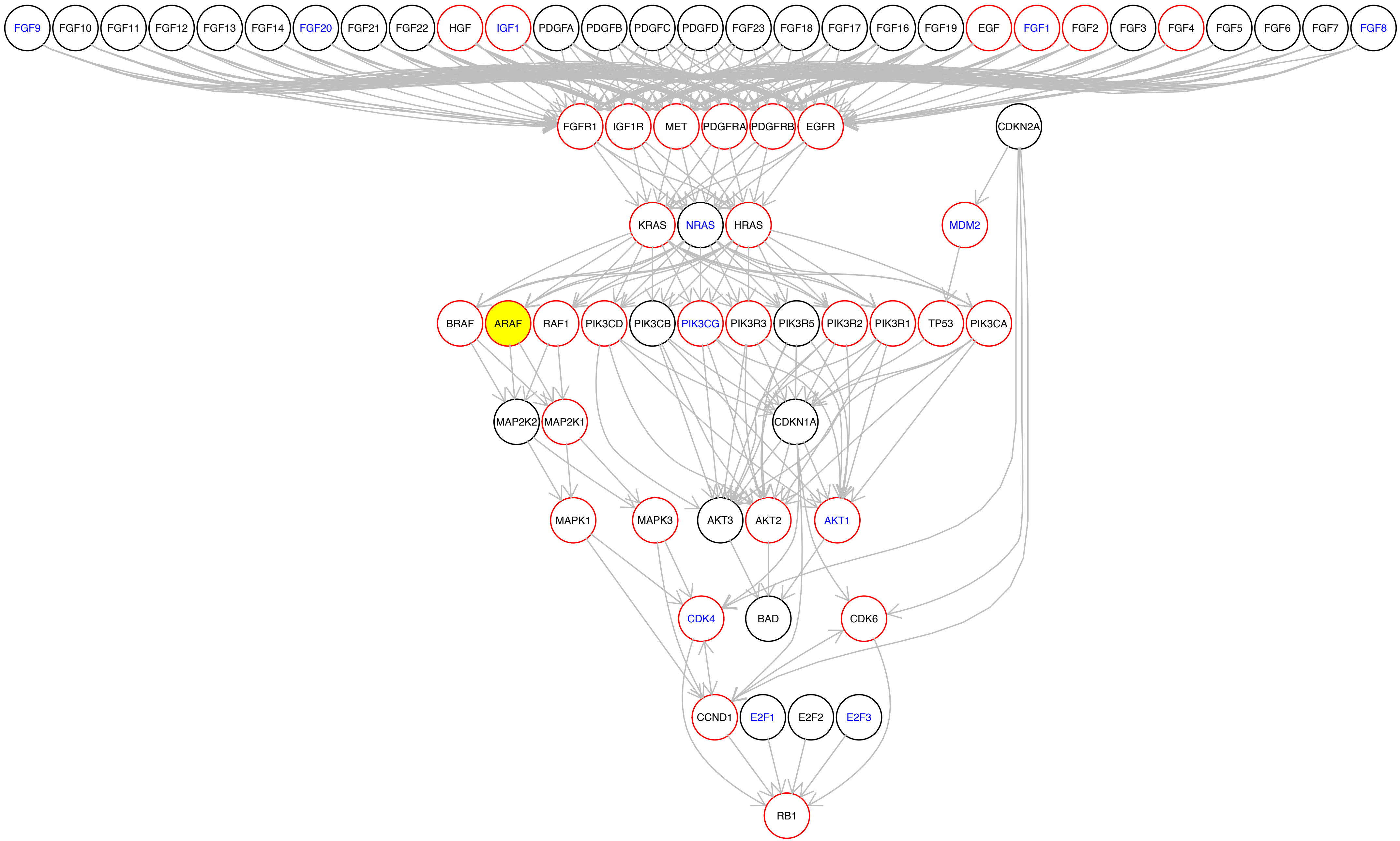

KEGG pathway = Melanoma : tumour = X96T : Yellow Fill = gene variant, Blue Text = expression–survival association, Red Boarder = drug

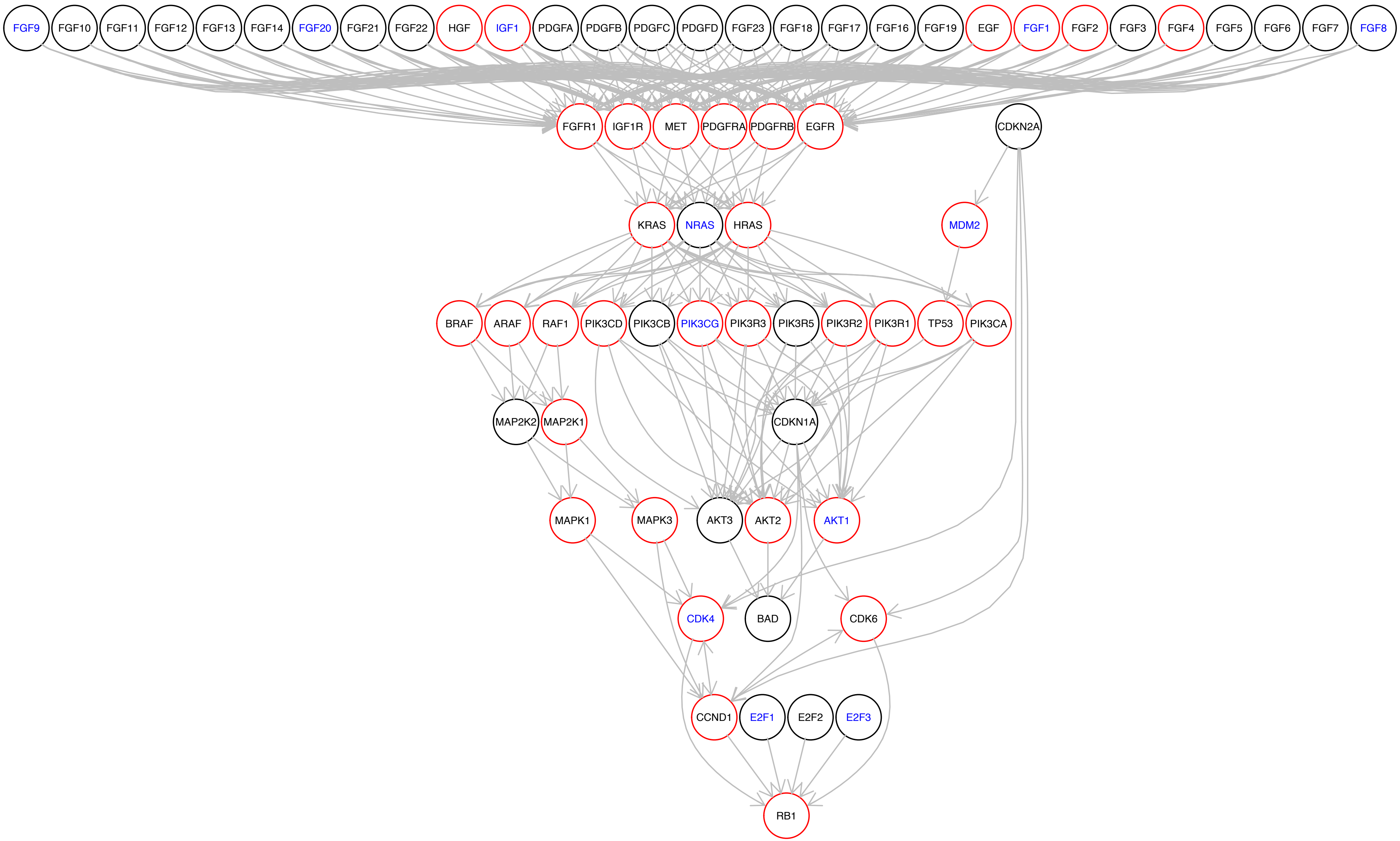

KEGG pathway = Melanoma : tumour = ME010.Tumor : Yellow Fill = gene variant, Blue Text = expression−survival association, Red Boarder = drug

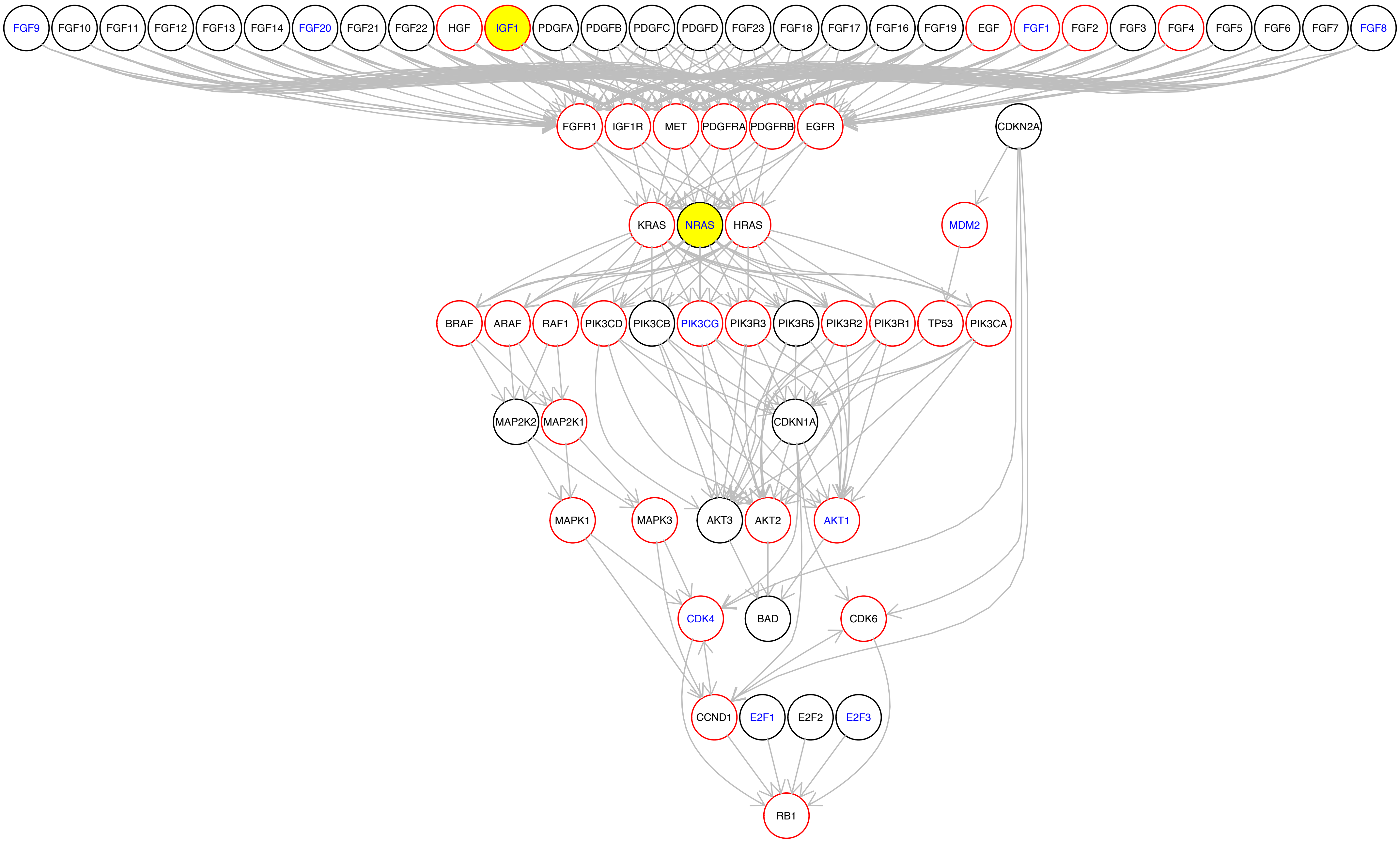

KEGG pathway = Melanoma : tumour = ME014.Tumor : Yellow Fill = gene variant, Blue Text = expression−survival association, Red Boarder = drug

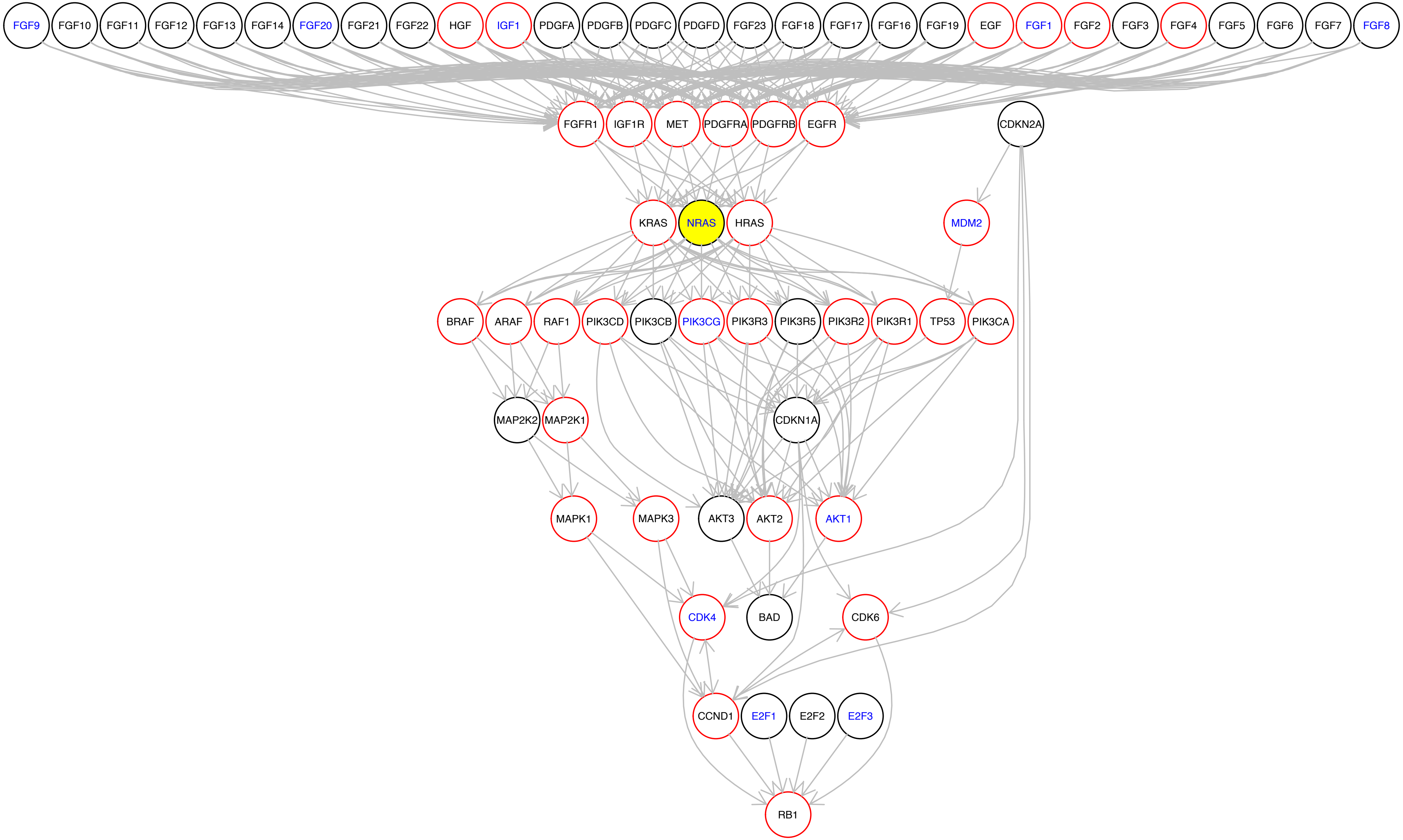

KEGG pathway = Melanoma : tumour = ME017.Tumor : Yellow Fill = gene variant, Blue Text = expression−survival association, Red Boarder = drug

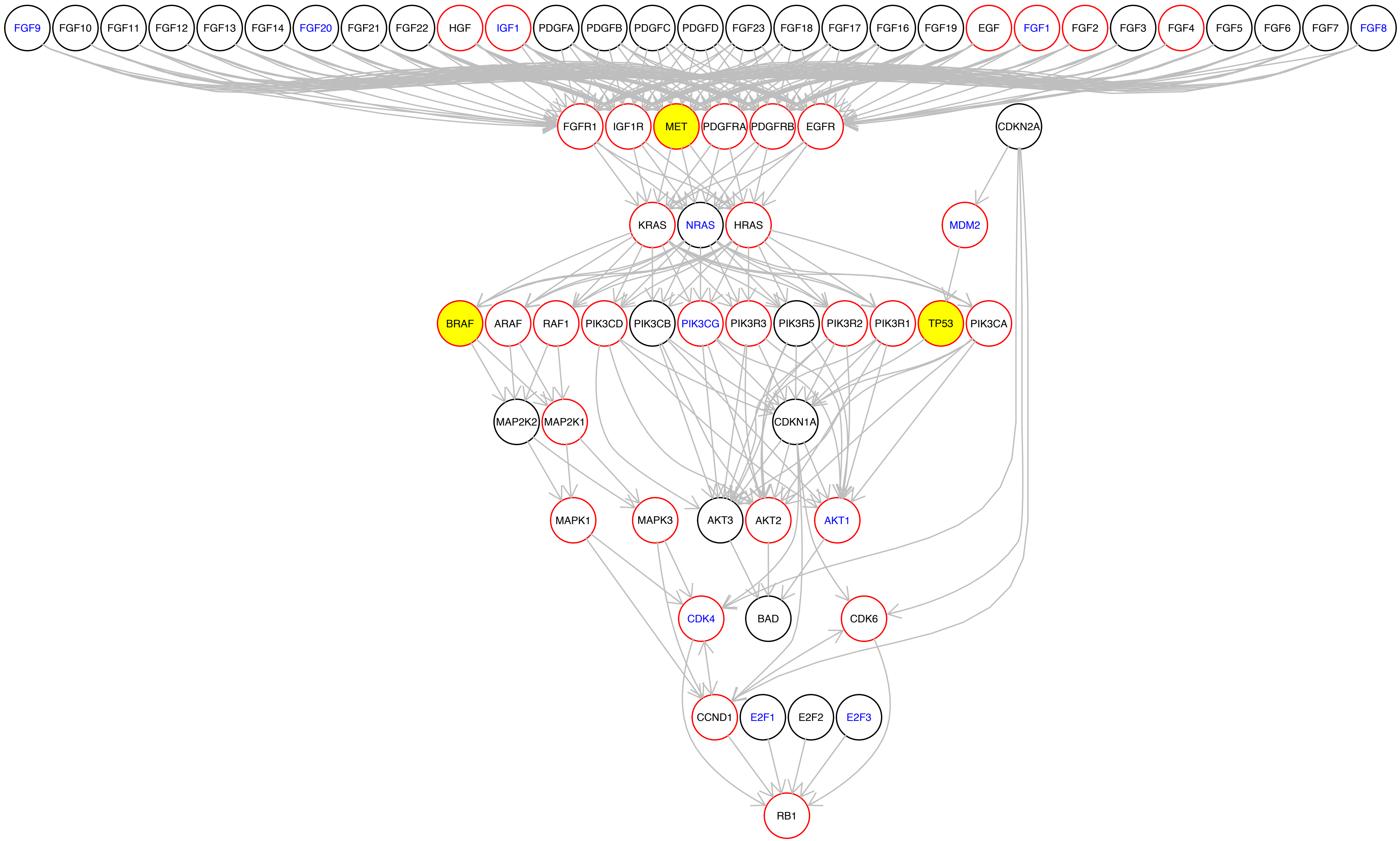

KEGG pathway = Melanoma : tumour = ME033.Tumor : Yellow Fill = gene variant, Blue Text = expression−survival association, Red Boarder = drug

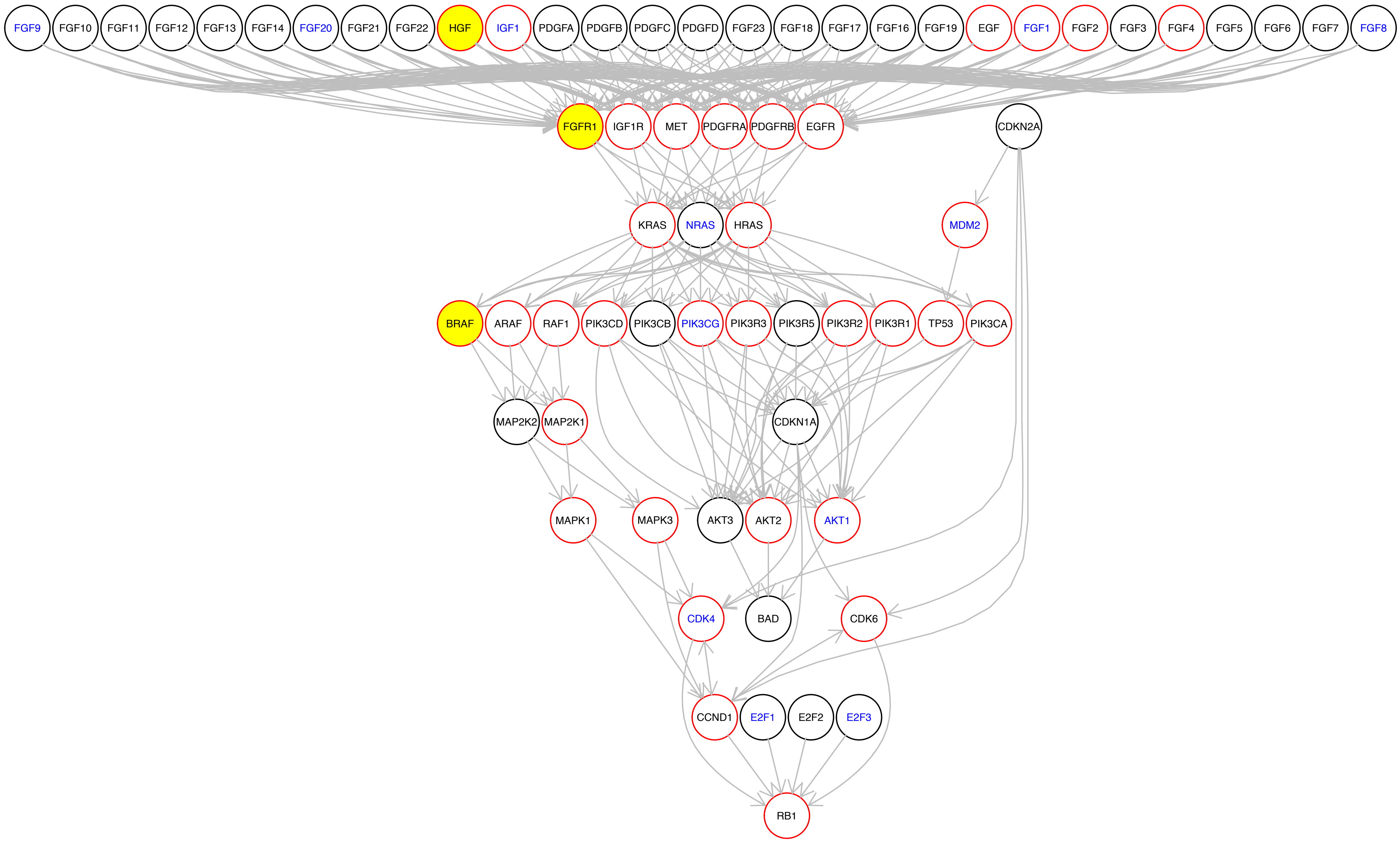

KEGG pathway = Melanoma : tumour = ME034.Tumor : Yellow Fill = gene variant, Blue Text = expression−survival association, Red Boarder = drug

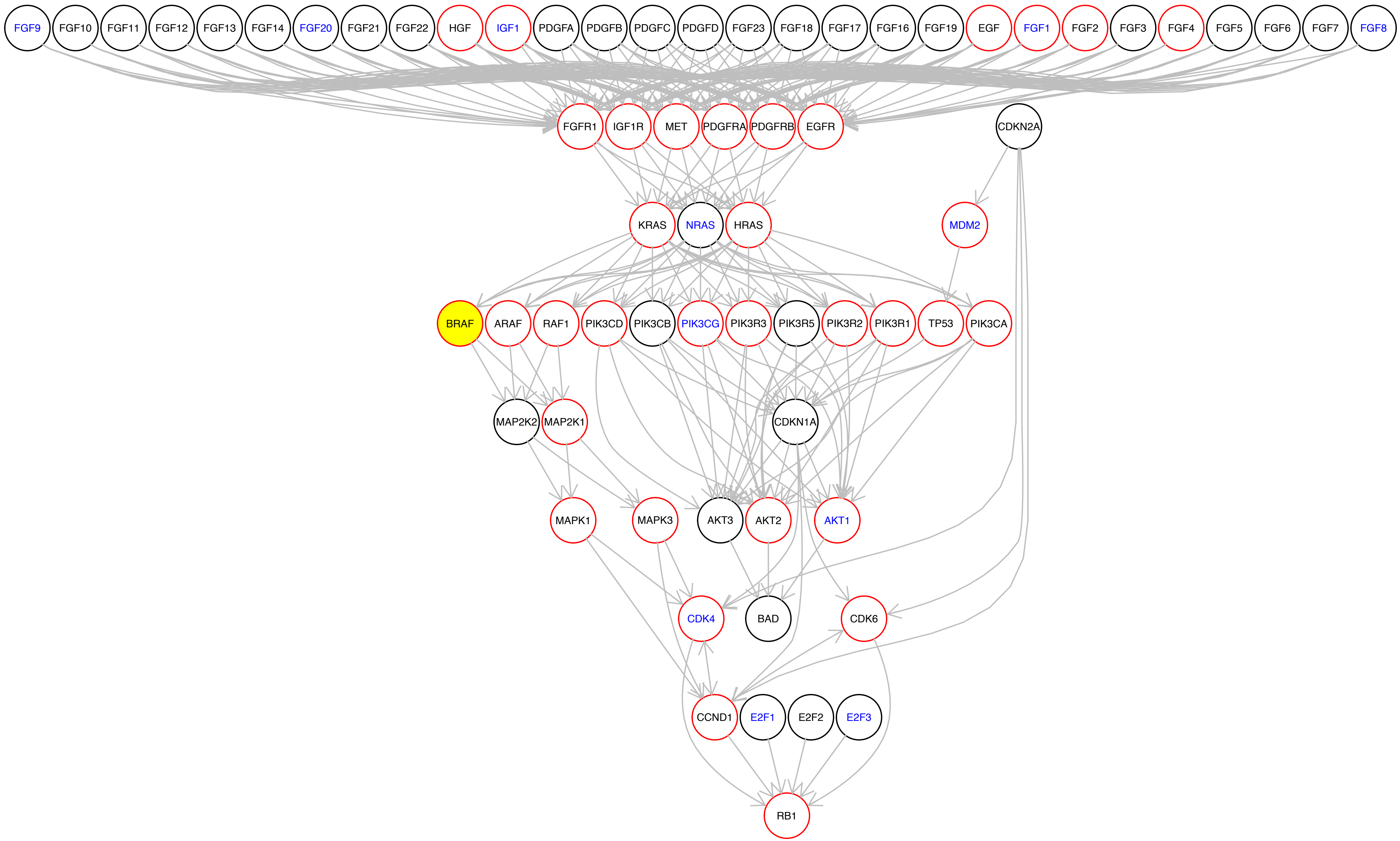

KEGG pathway = Melanoma : tumour = Mel_BRAFi_02_PRE.Tumor : Yellow Fill = gene variant, Blue Text = expression–survival association, Red Boarder = drug

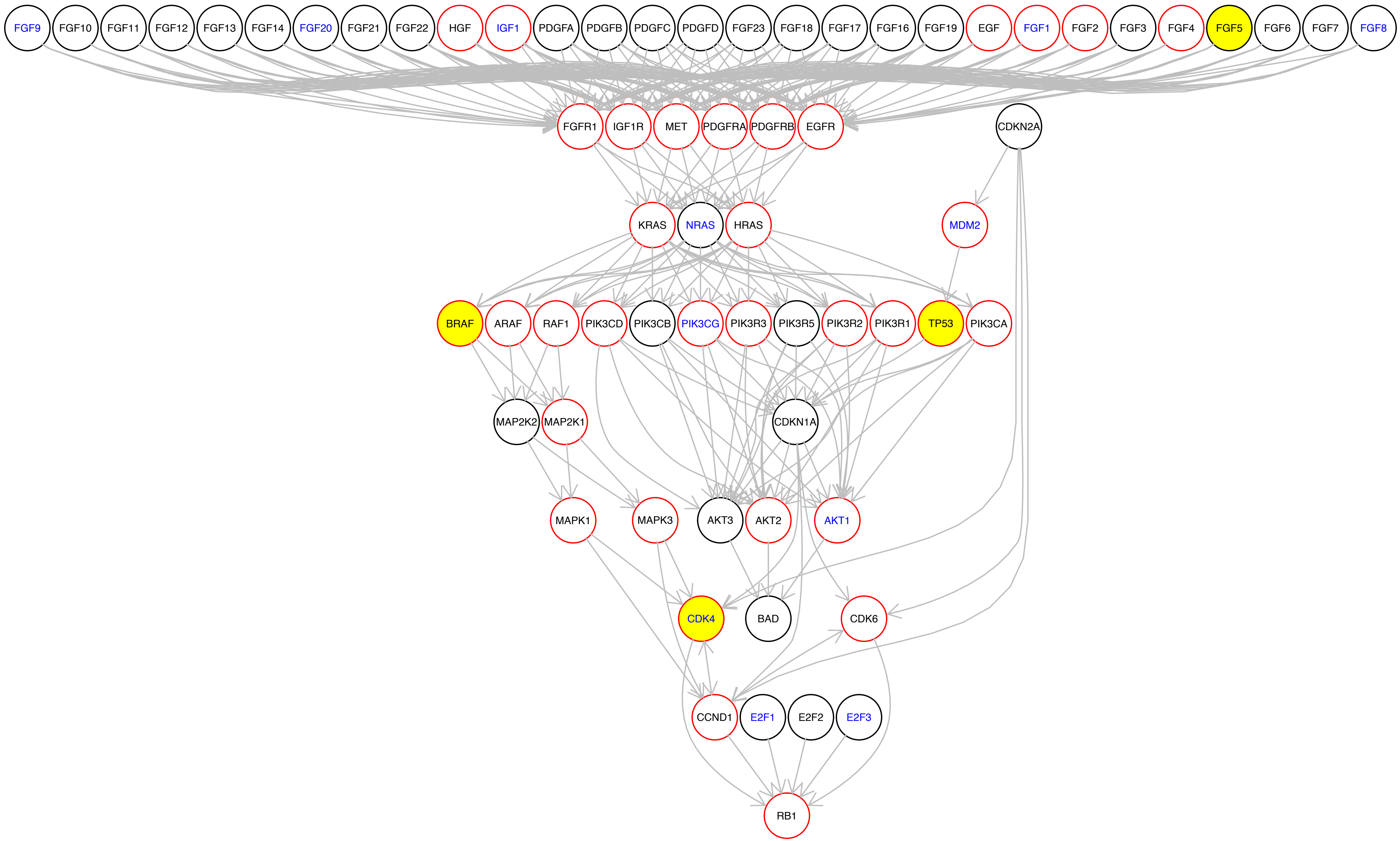

KEGG pathway = Melanoma : tumour = MEL.13447.Tumor : Yellow Fill = gene variant, Blue Text = expression−survival association, Red Boarder = drug

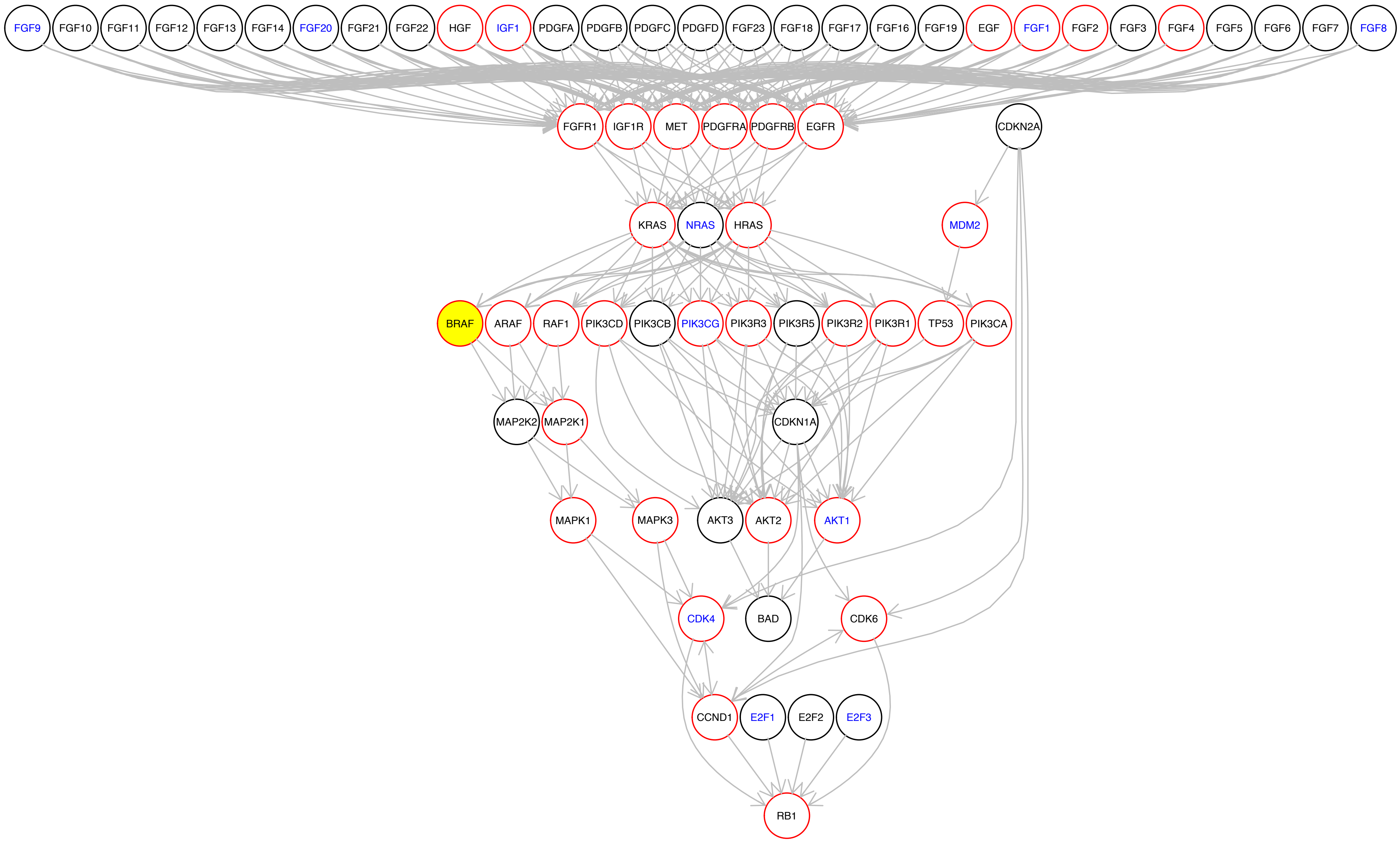

KEGG pathway = Melanoma : tumour = MEL.13456.Tumor : Yellow Fill = gene variant, Blue Text = expression−survival association, Red Boarder = drug

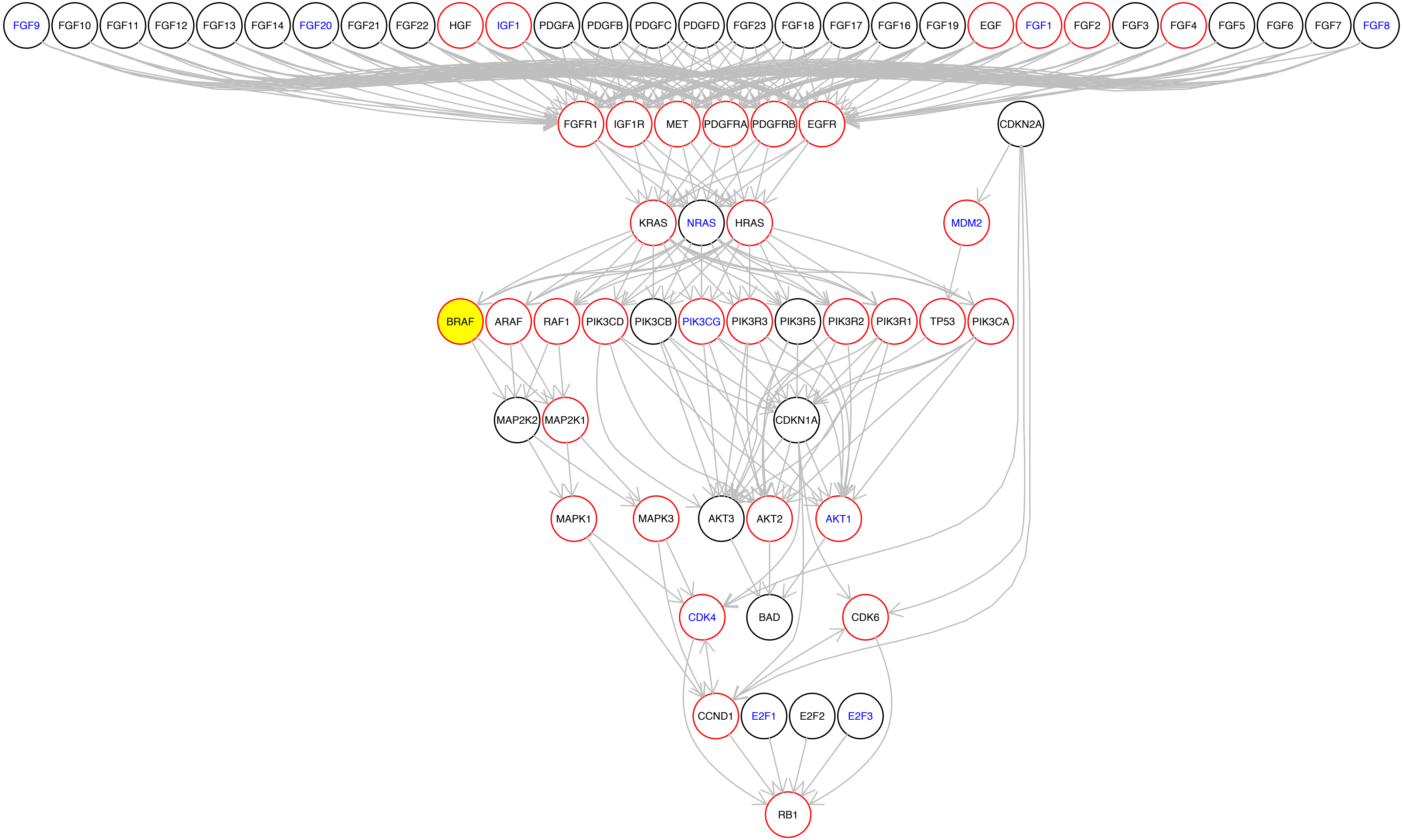

KEGG pathway = Melanoma : tumour = MEL.13463.Tumor : Yellow Fill = gene variant, Blue Text = expression−survival association, Red Boarder = drug

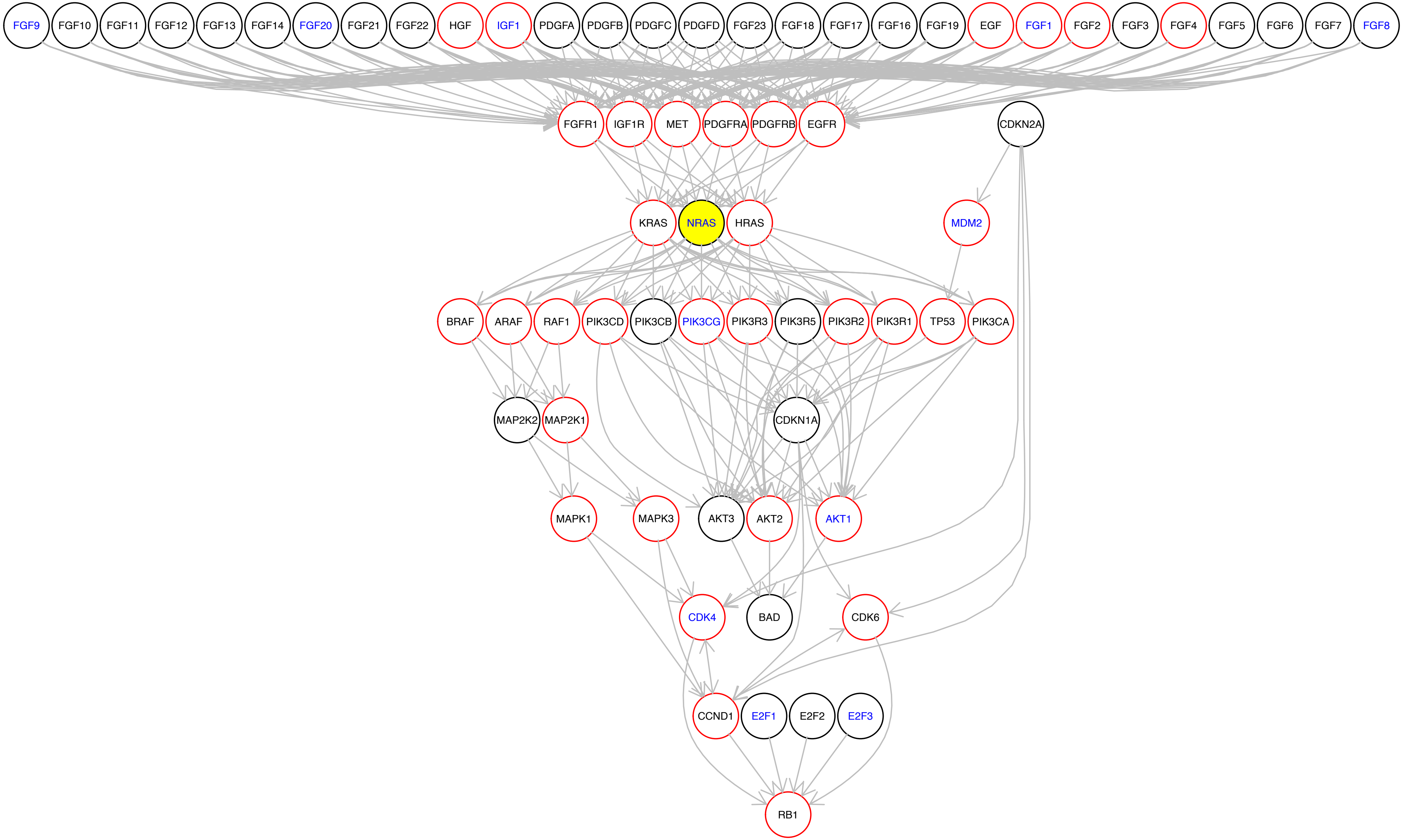

KEGG pathway = Melanoma : tumour = MEL.13468.Tumor : Yellow Fill = gene variant, Blue Text = expression−survival association, Red Boarder = drug

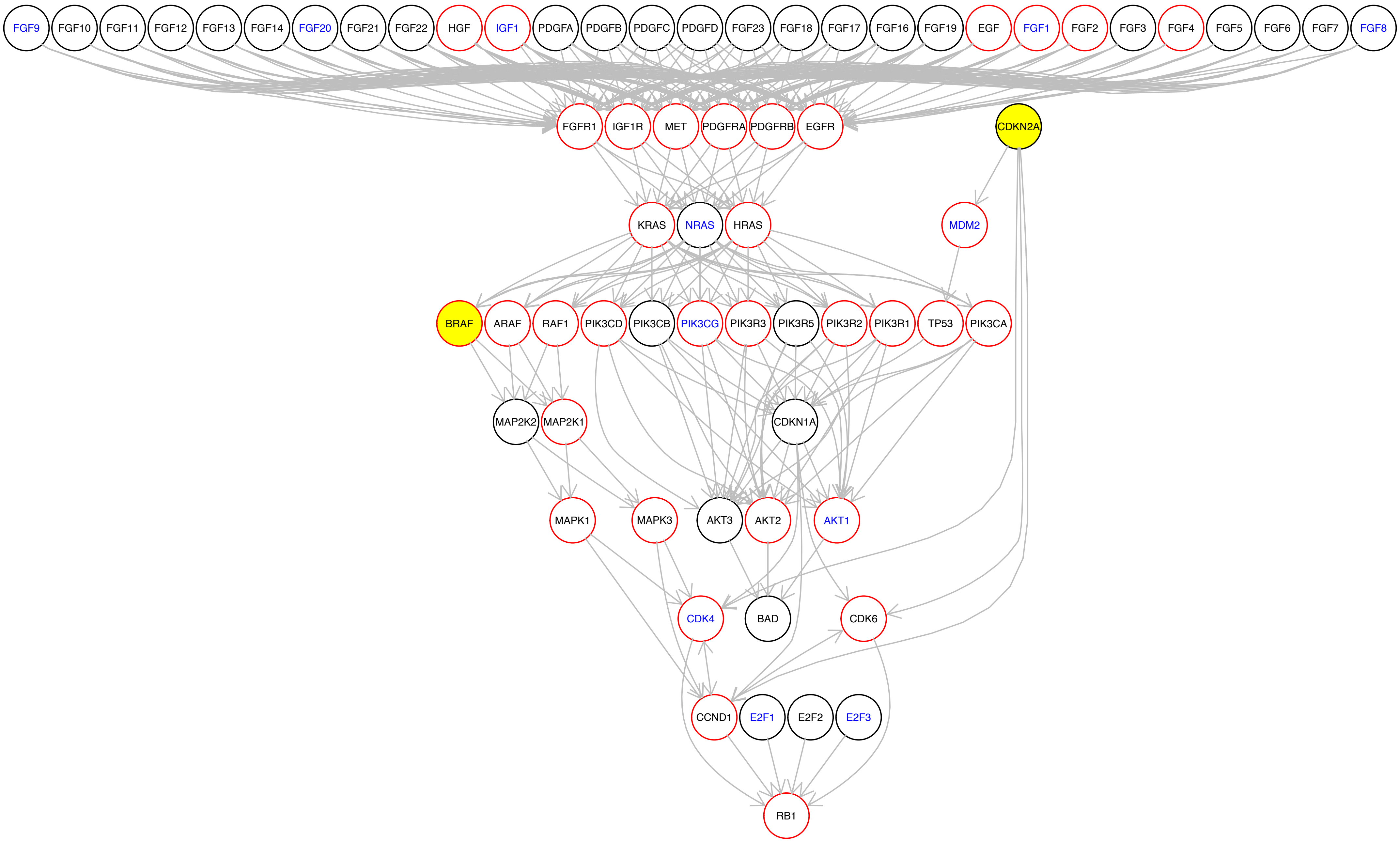

KEGG pathway = Melanoma : tumour = MEL.13473.Tumor : Yellow Fill = gene variant, Blue Text = expression−survival association, Red Boarder = drug

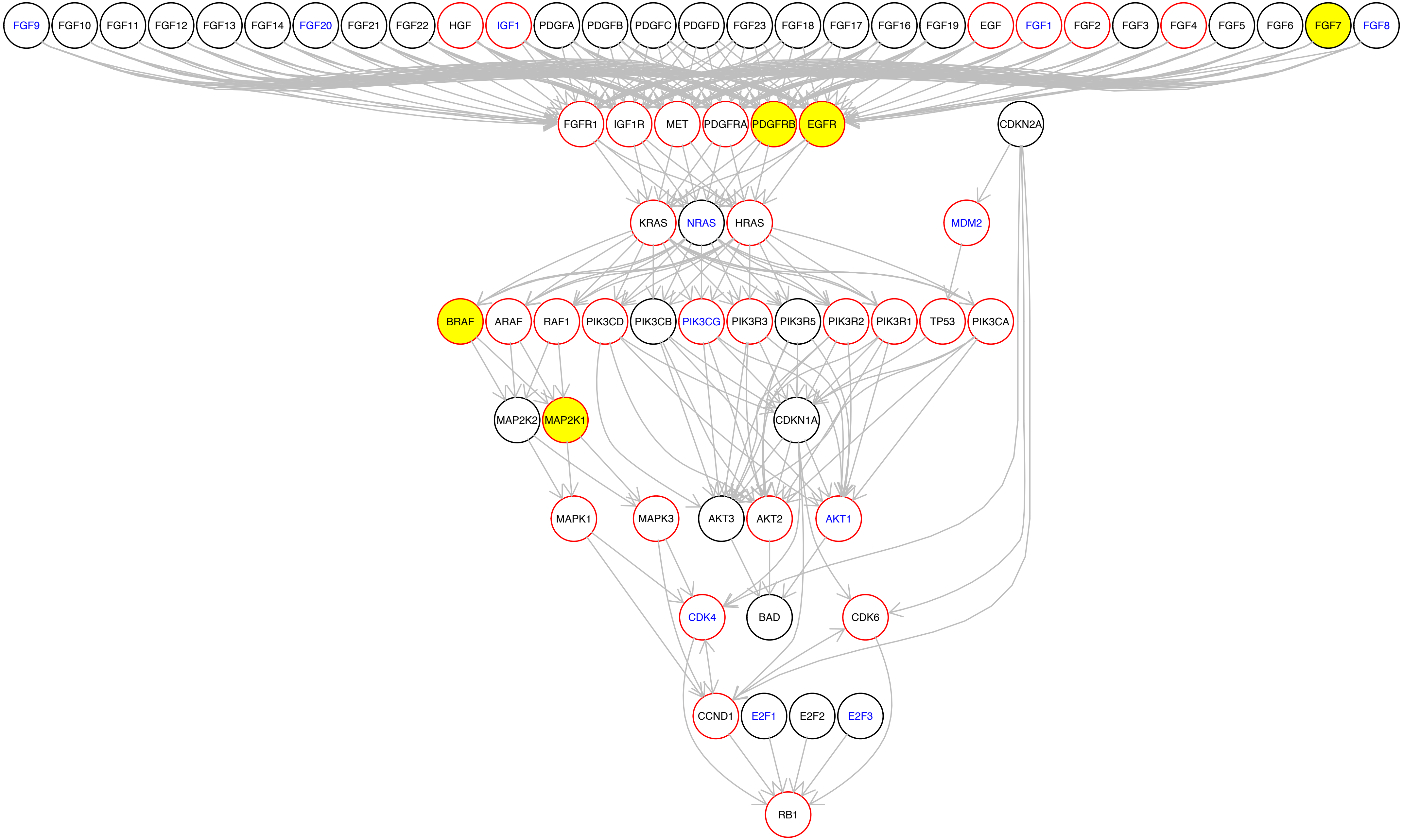

KEGG pathway = Melanoma : tumour = MEL.13531.Tumor : Yellow Fill = gene variant, Blue Text = expression−survival association, Red Boarder = drug

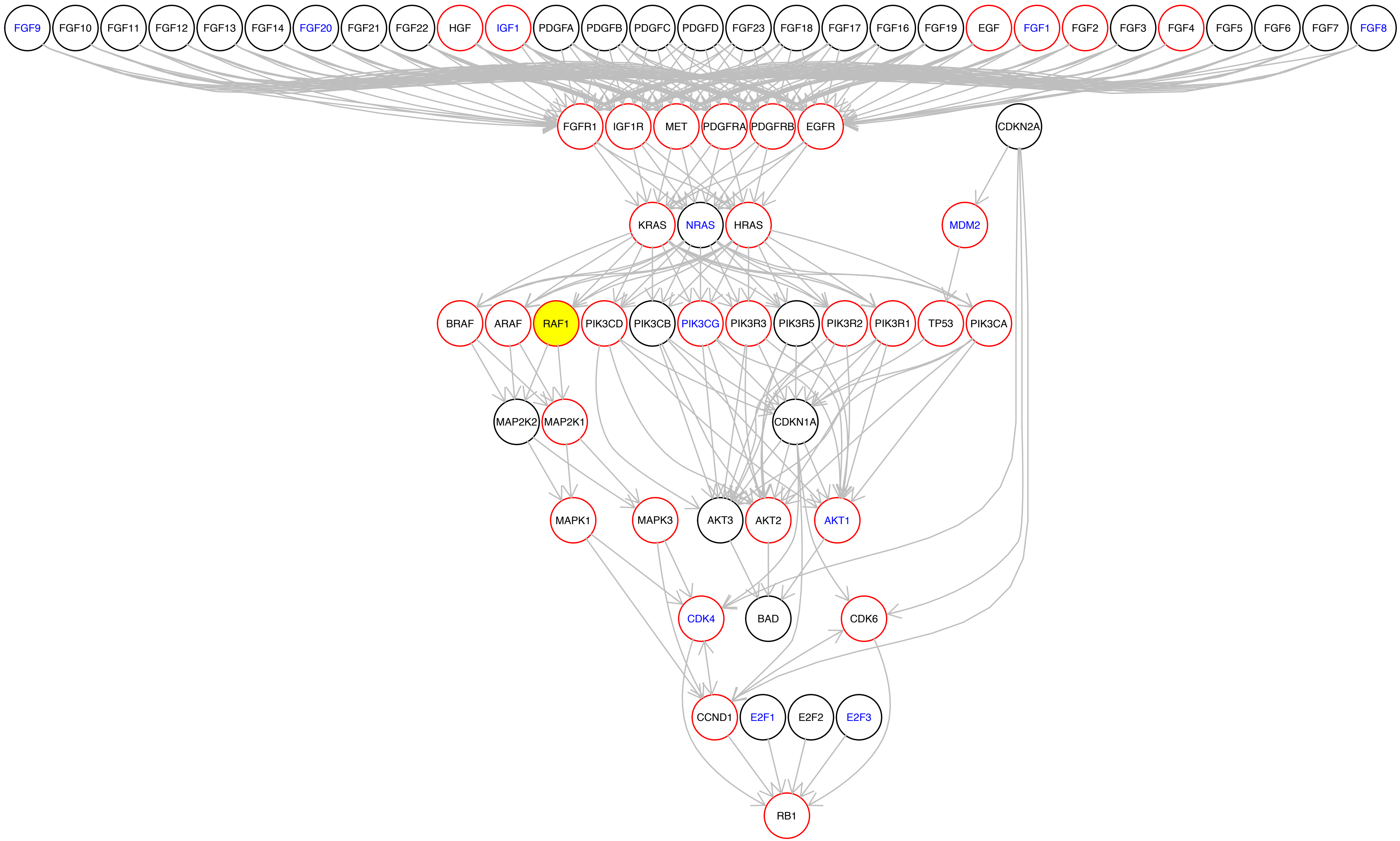

KEGG pathway = Melanoma :   tumour = MEL.13537.Tumor :   Yellow Fill = gene variant, Blue Text = expression−survival association, Red Boarder = drug

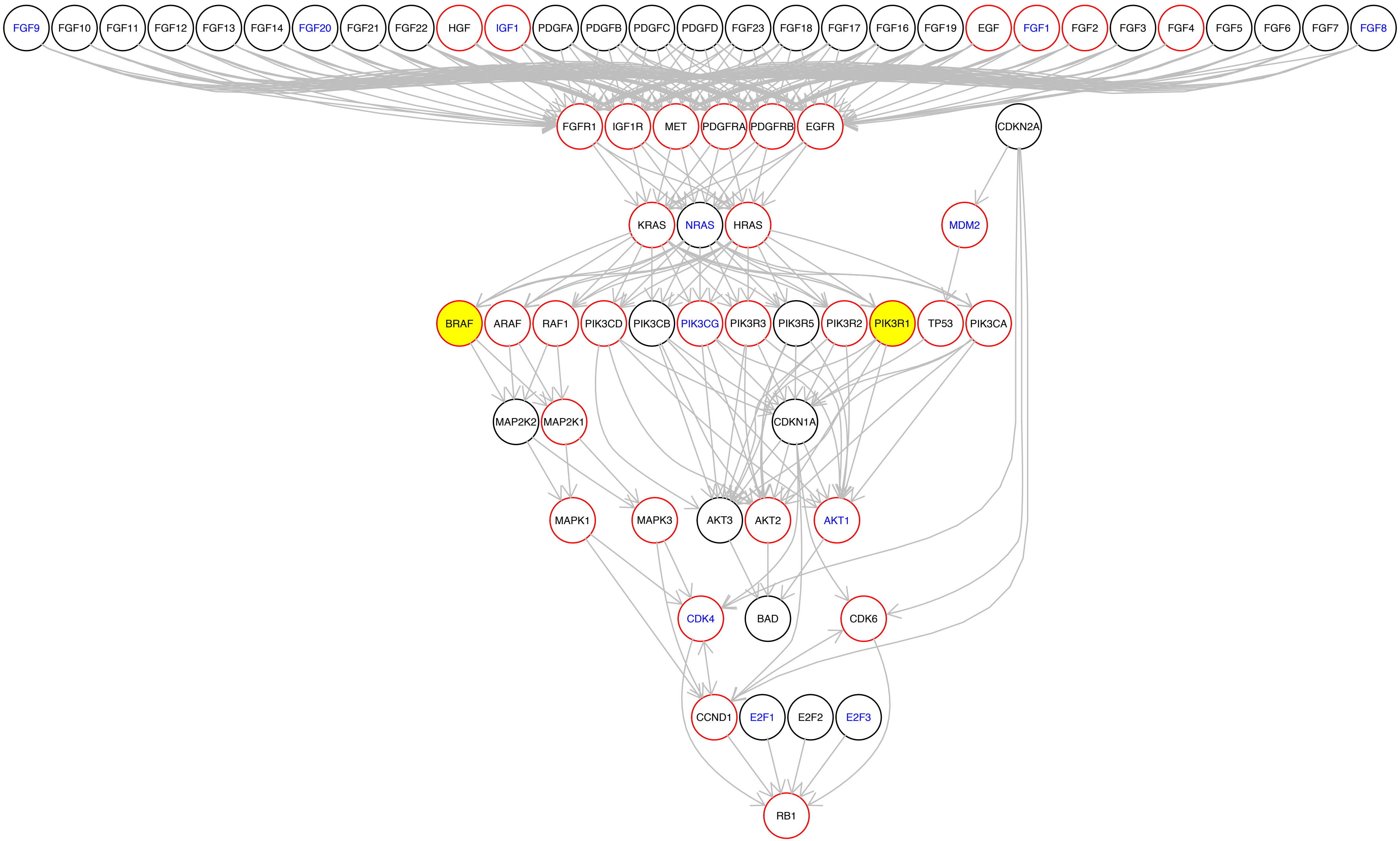

KEGG pathway = Melanoma : tumour = MEL.13543.Tumor : Yellow Fill = gene variant, Blue Text = expression−survival association, Red Boarder = drug

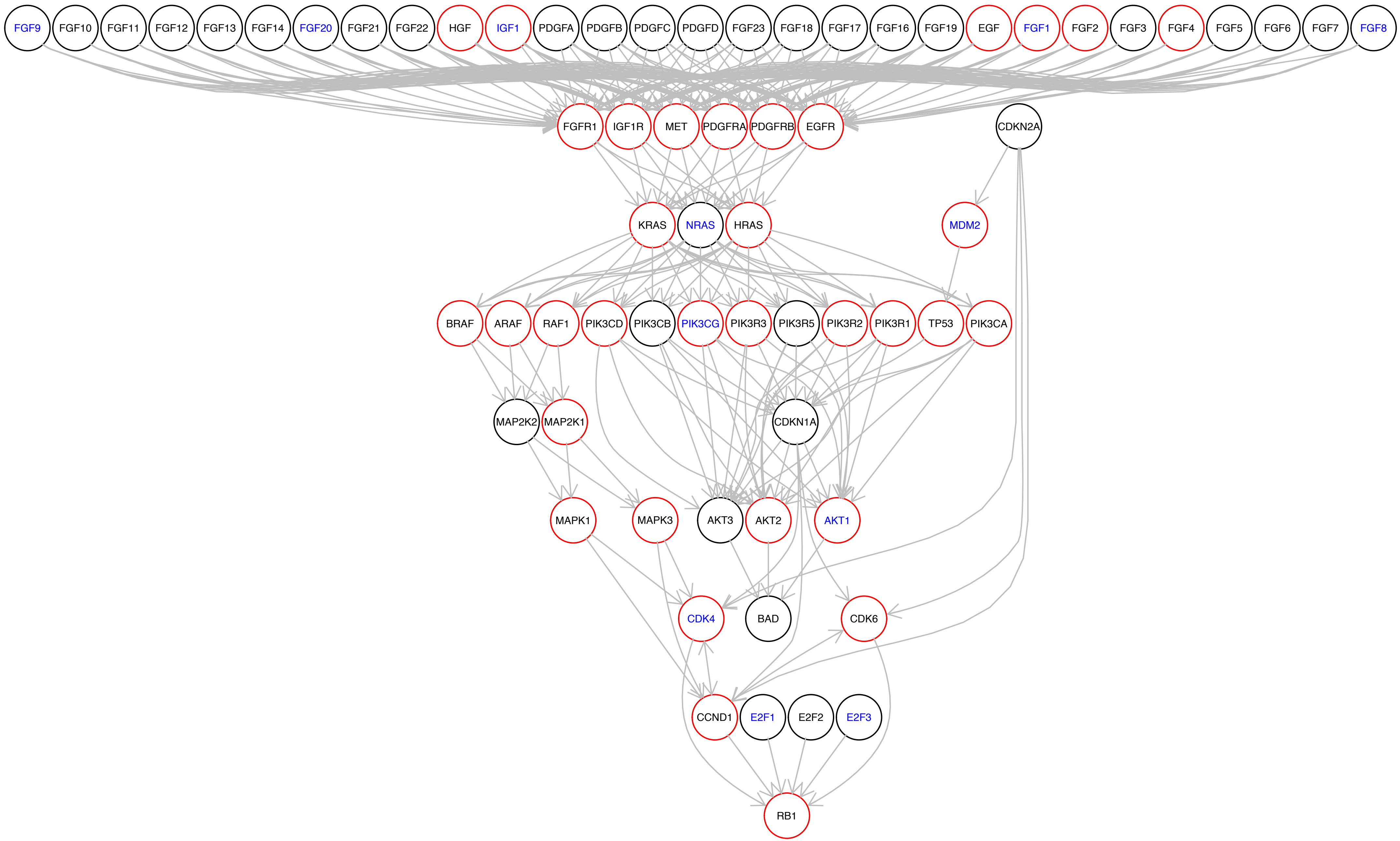

KEGG pathway = Melanoma : tumour = MEL.13549.Tumor : Yellow Fill = gene variant, Blue Text = expression−survival association, Red Boarder = drug

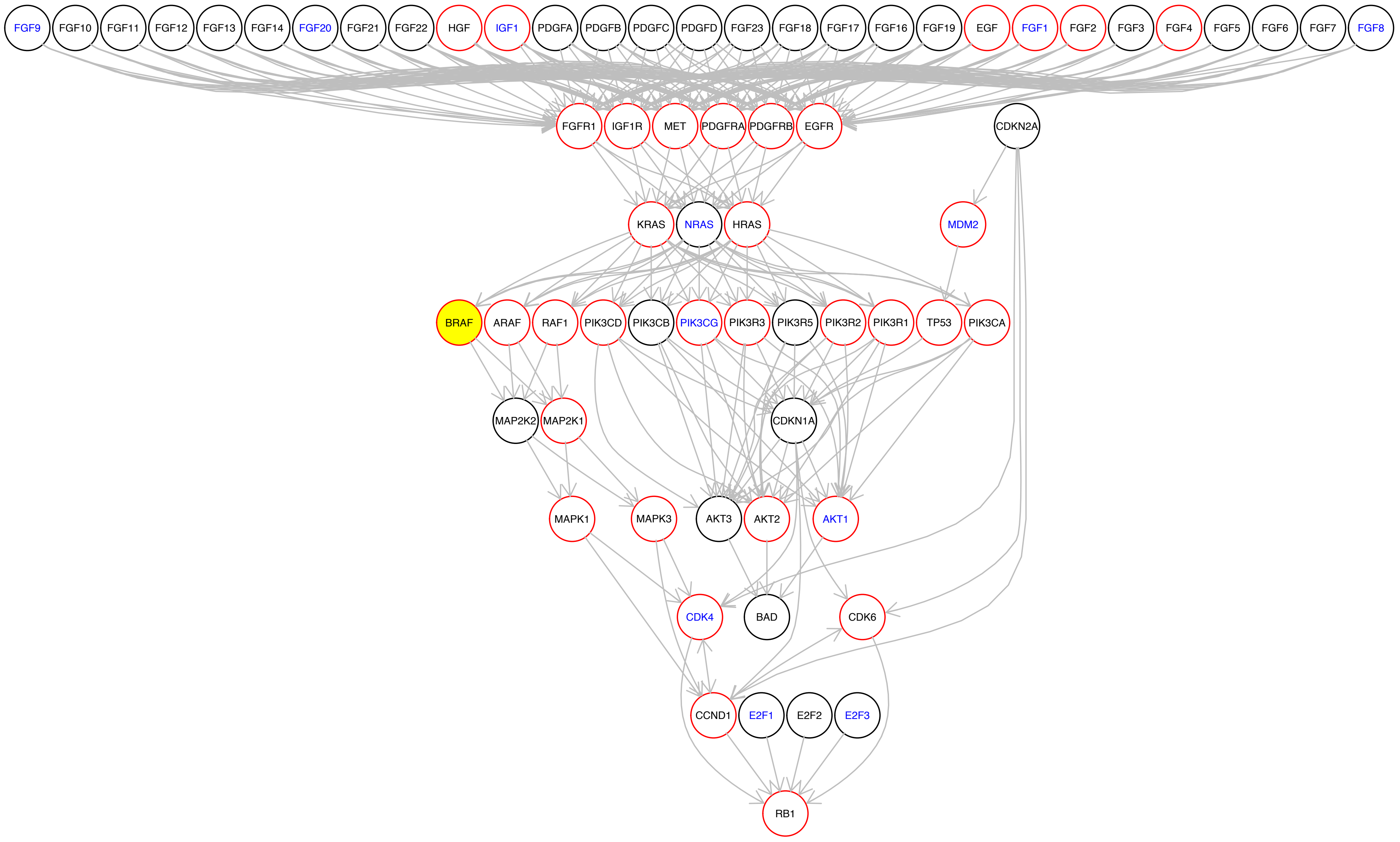

KEGG pathway = Melanoma : tumour = MEL.13560.Tumor : Yellow Fill = gene variant, Blue Text = expression−survival association, Red Boarder = drug

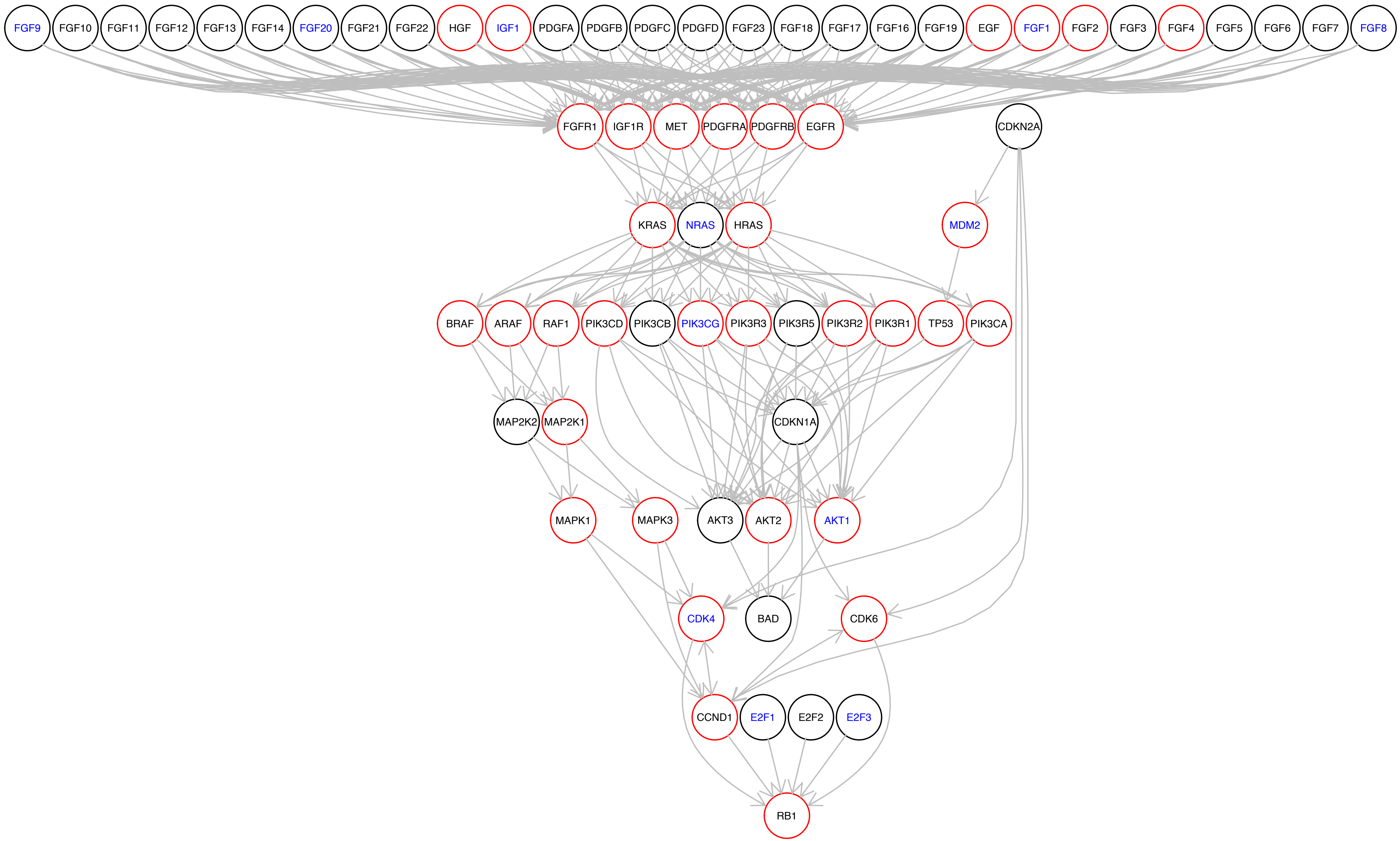

KEGG pathway = Melanoma :   tumour = MEL.13561.Tumor :   Yellow Fill = gene variant, Blue Text = expression-survival association, Red Boarder = drug

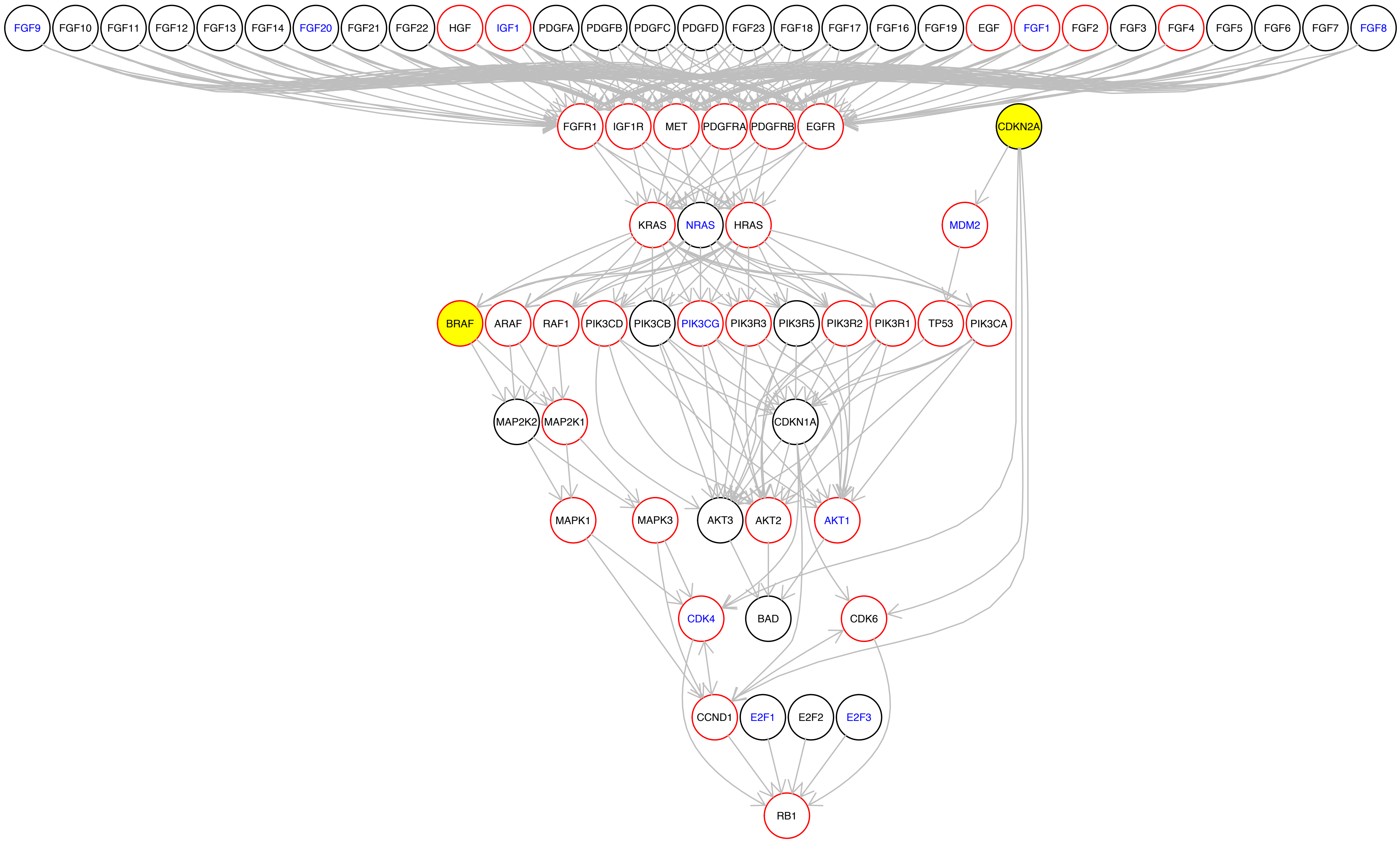

KEGG pathway = Melanoma : tumour = MEL.13567.Tumor : Yellow Fill = gene variant, Blue Text = expression−survival association, Red Boarder = drug

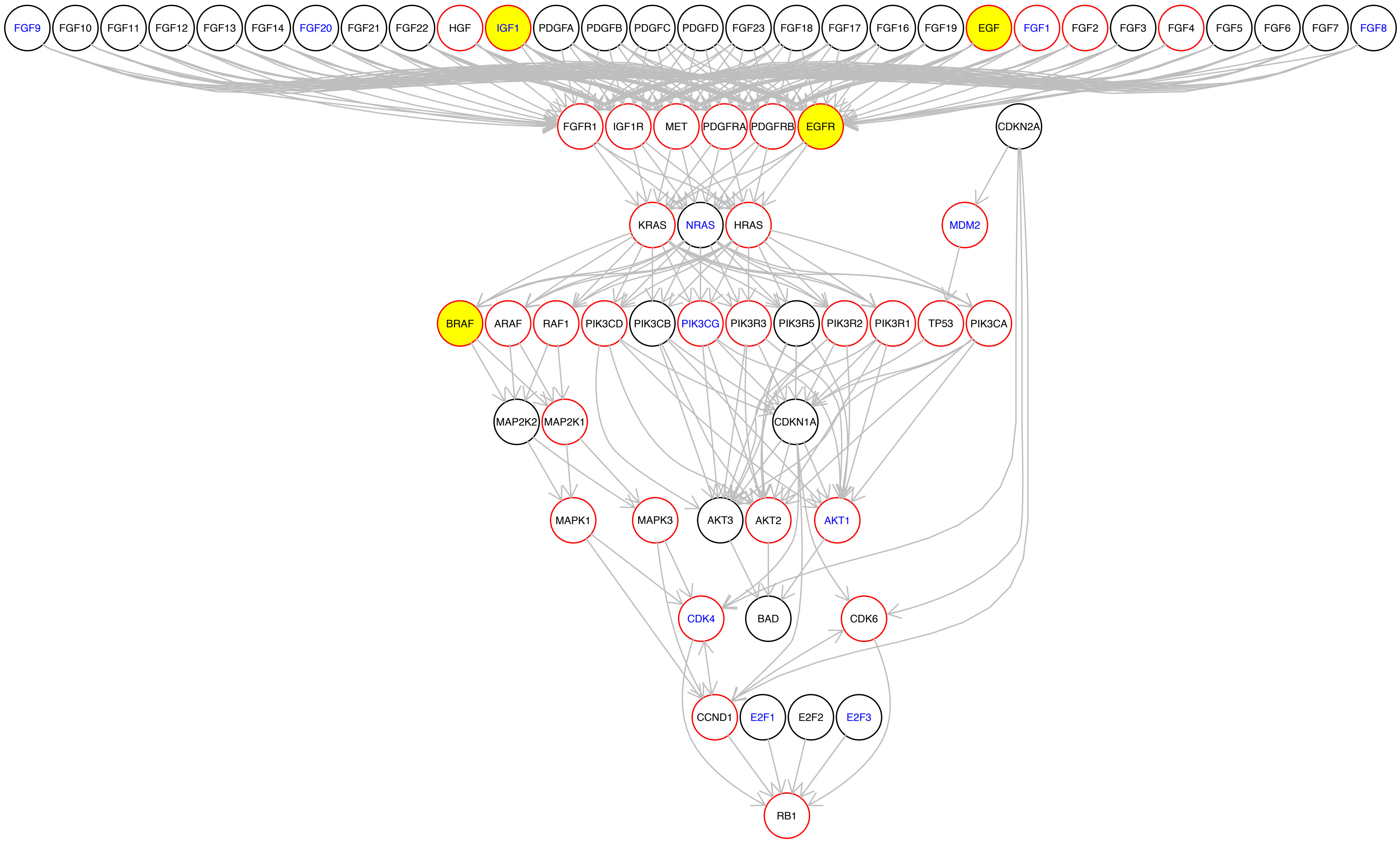

KEGG pathway = Melanoma : tumour = MEL.13575.Tumor : Yellow Fill = gene variant, Blue Text = expression–survival association, Red Boarder = drug

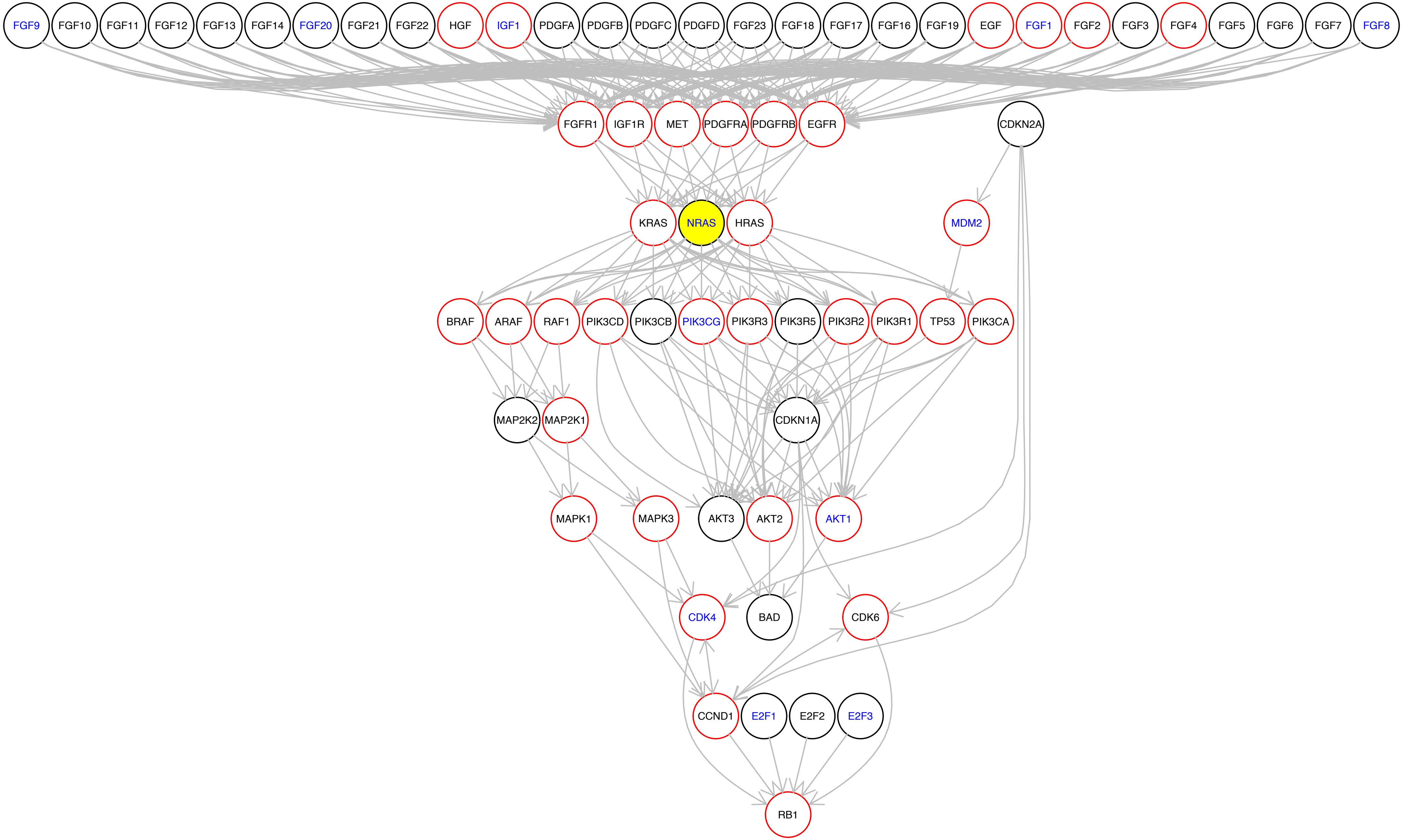

KEGG pathway = Melanoma : tumour = MEL.13591.Tumor : Yellow Fill = gene variant, Blue Text = expression−survival association, Red Boarder = drug

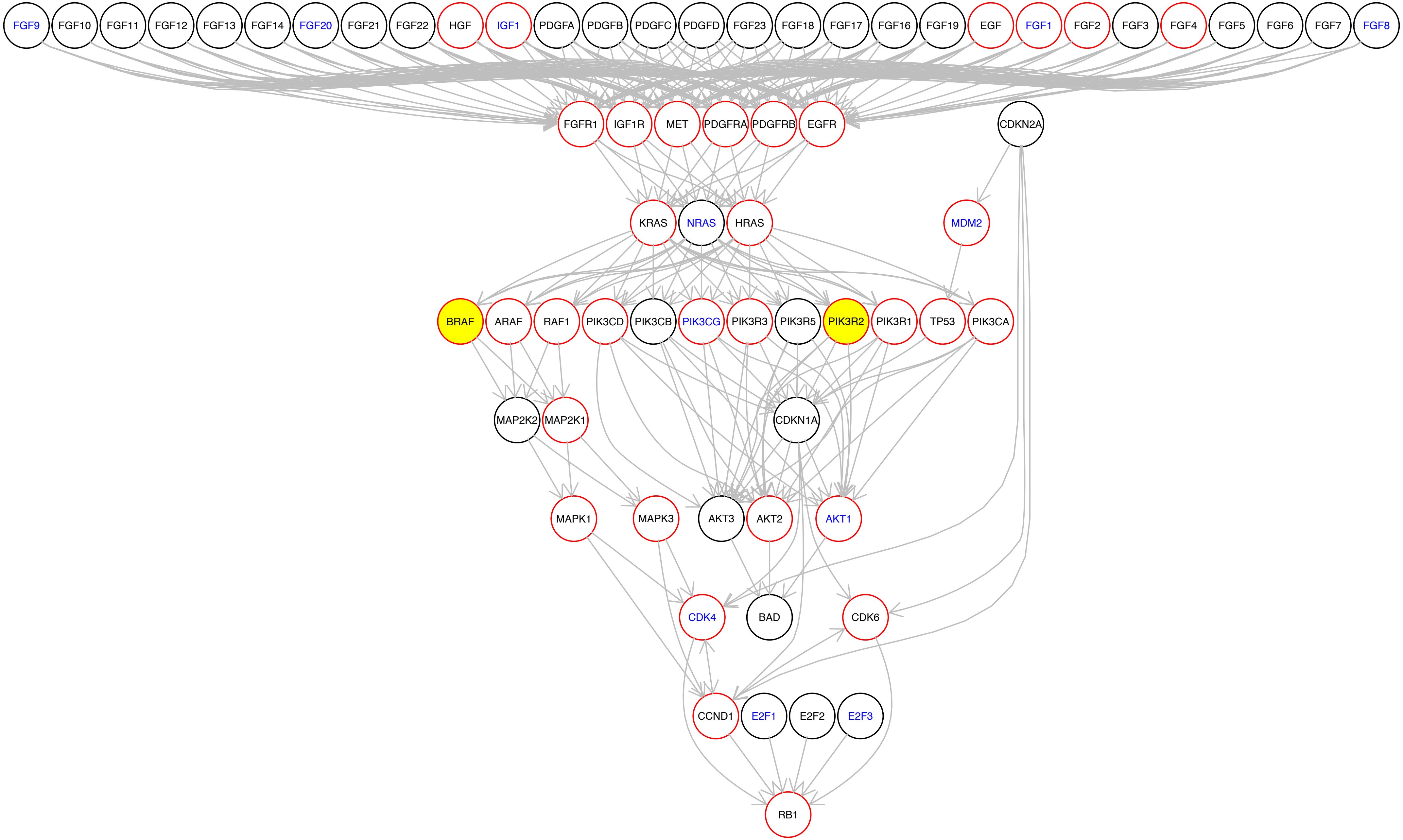

KEGG pathway = Melanoma : tumour = MEL.13600.Tumor : Yellow Fill = gene variant, Blue Text = expression−survival association, Red Boarder = drug

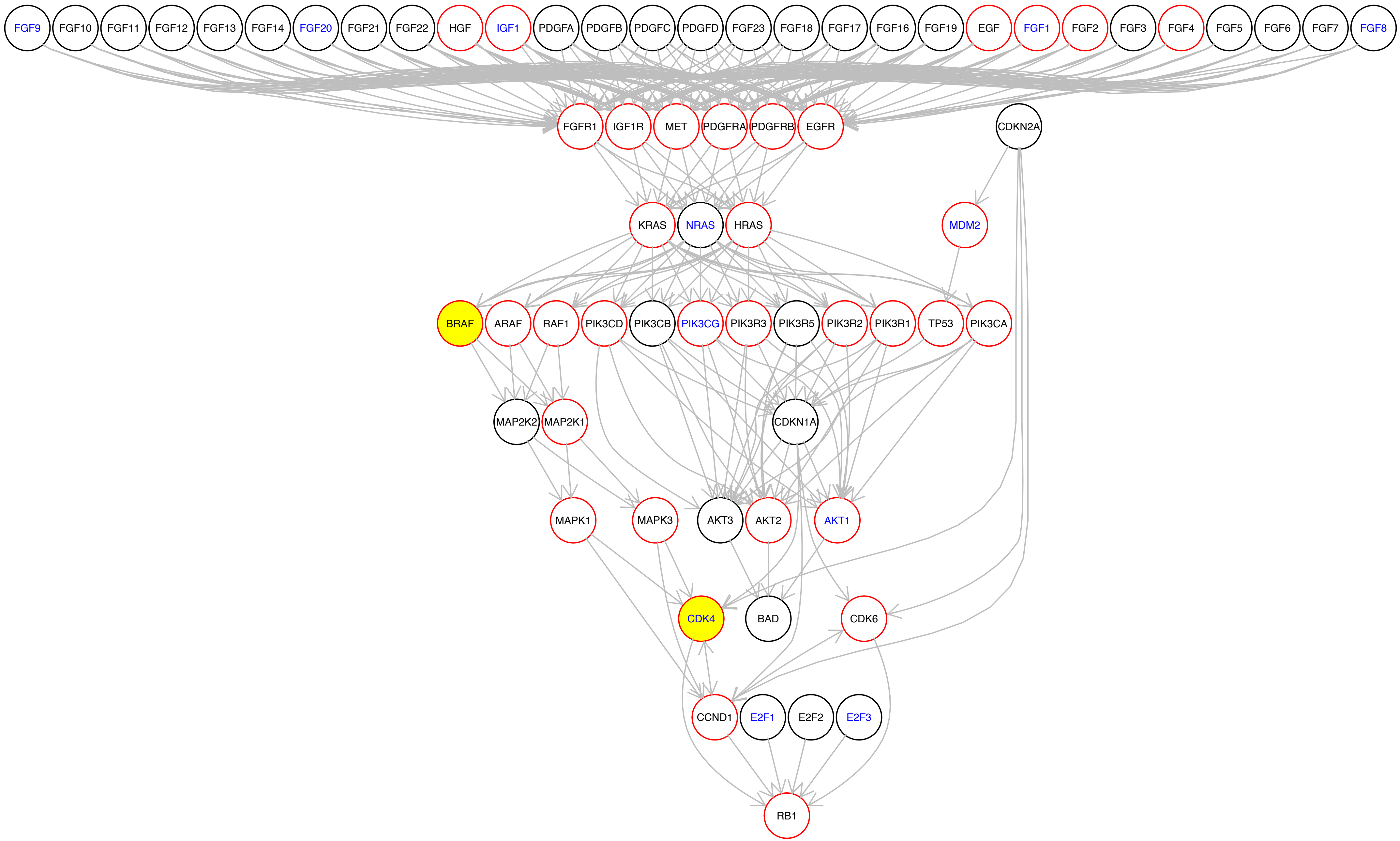

KEGG pathway = Melanoma :   tumour = Mel.BRAFi.03.Tumor :   Yellow Fill = gene variant, Blue Text = expression−survival association, Red Boarder = drug

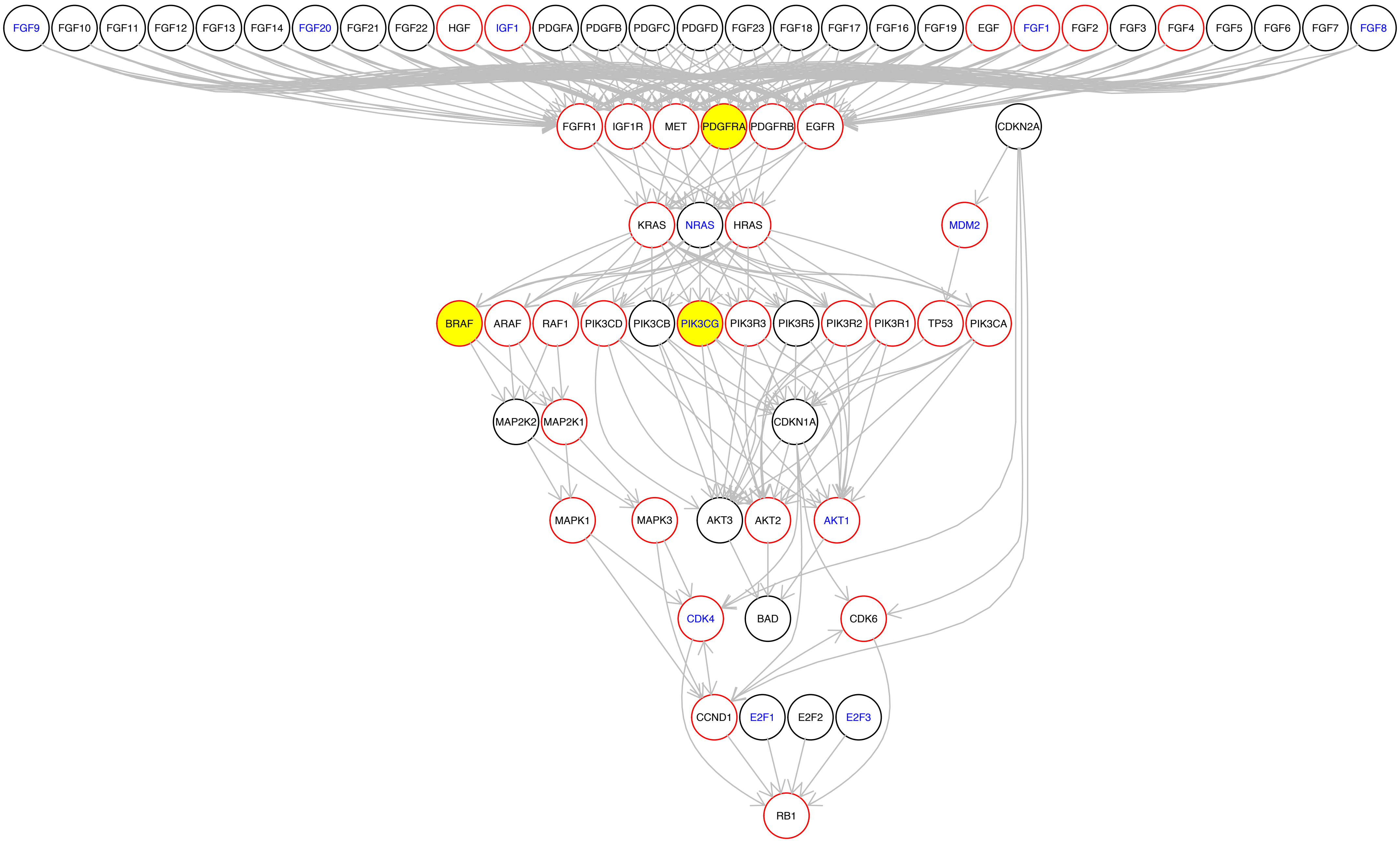

KEGG pathway = Melanoma : tumour = MEL.JWCI.14.Tumor : Yellow Fill = gene variant, Blue Text = expression–survival association, Red Boarder = drug

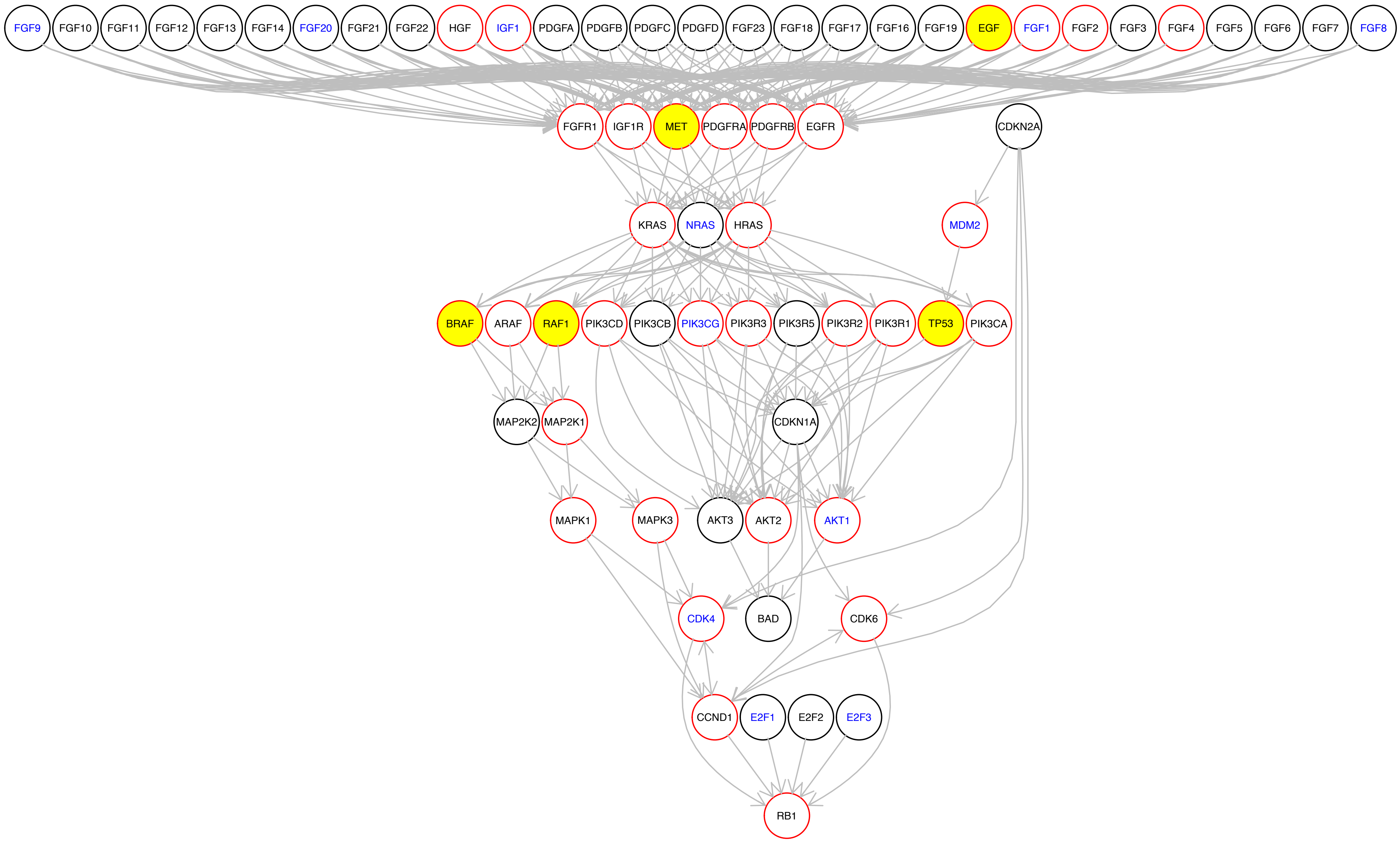

KEGG pathway = Melanoma : tumour = MEL.JWCI.27.Tumor : Yellow Fill = gene variant, Blue Text = expression−survival association, Red Boarder = drug

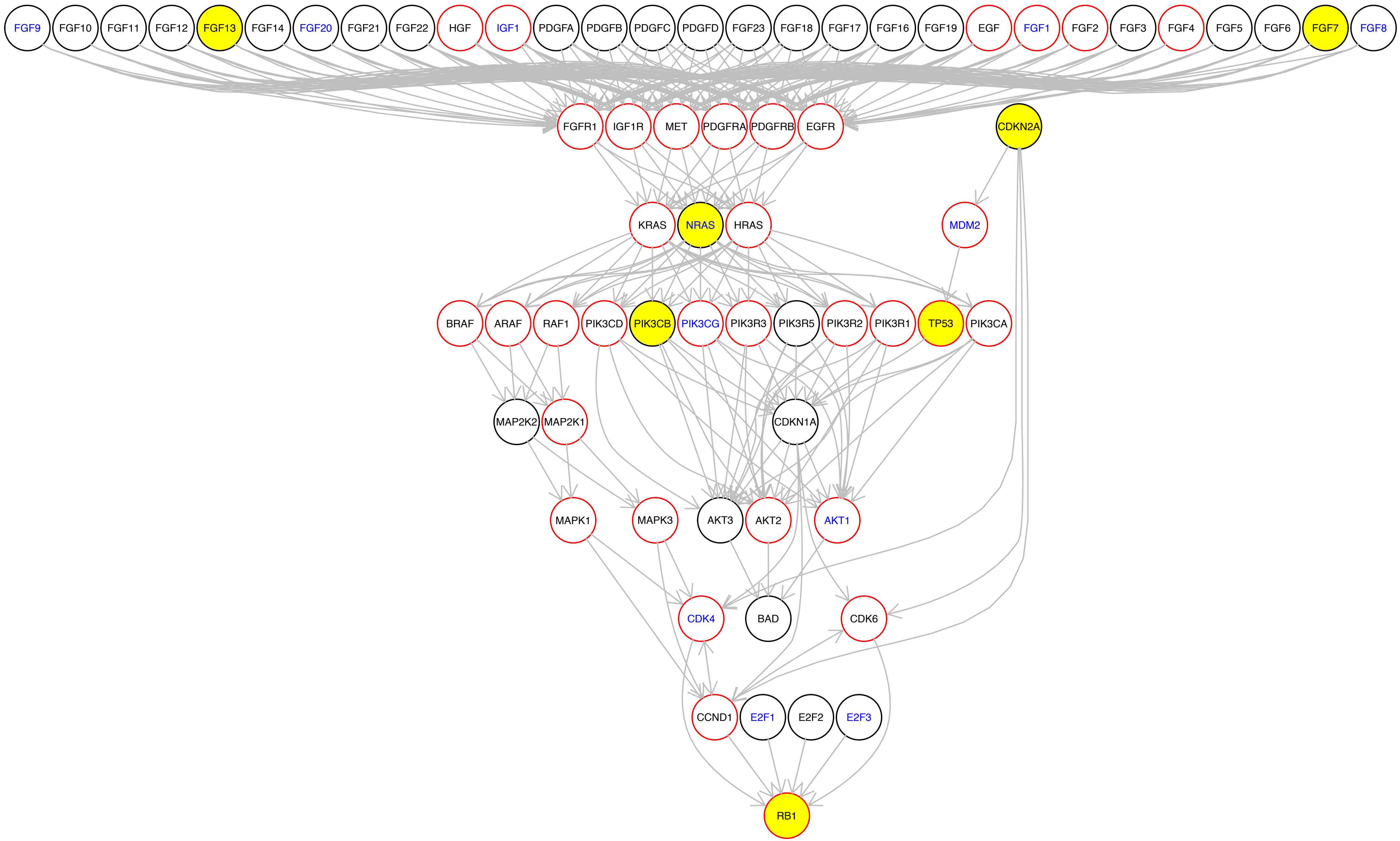

KEGG pathway = Melanoma : tumour = MEL.JWCI.WGS.11.Tumor : Yellow Fill = gene variant, Blue Text = expression−survival association, Red Boarder = drug

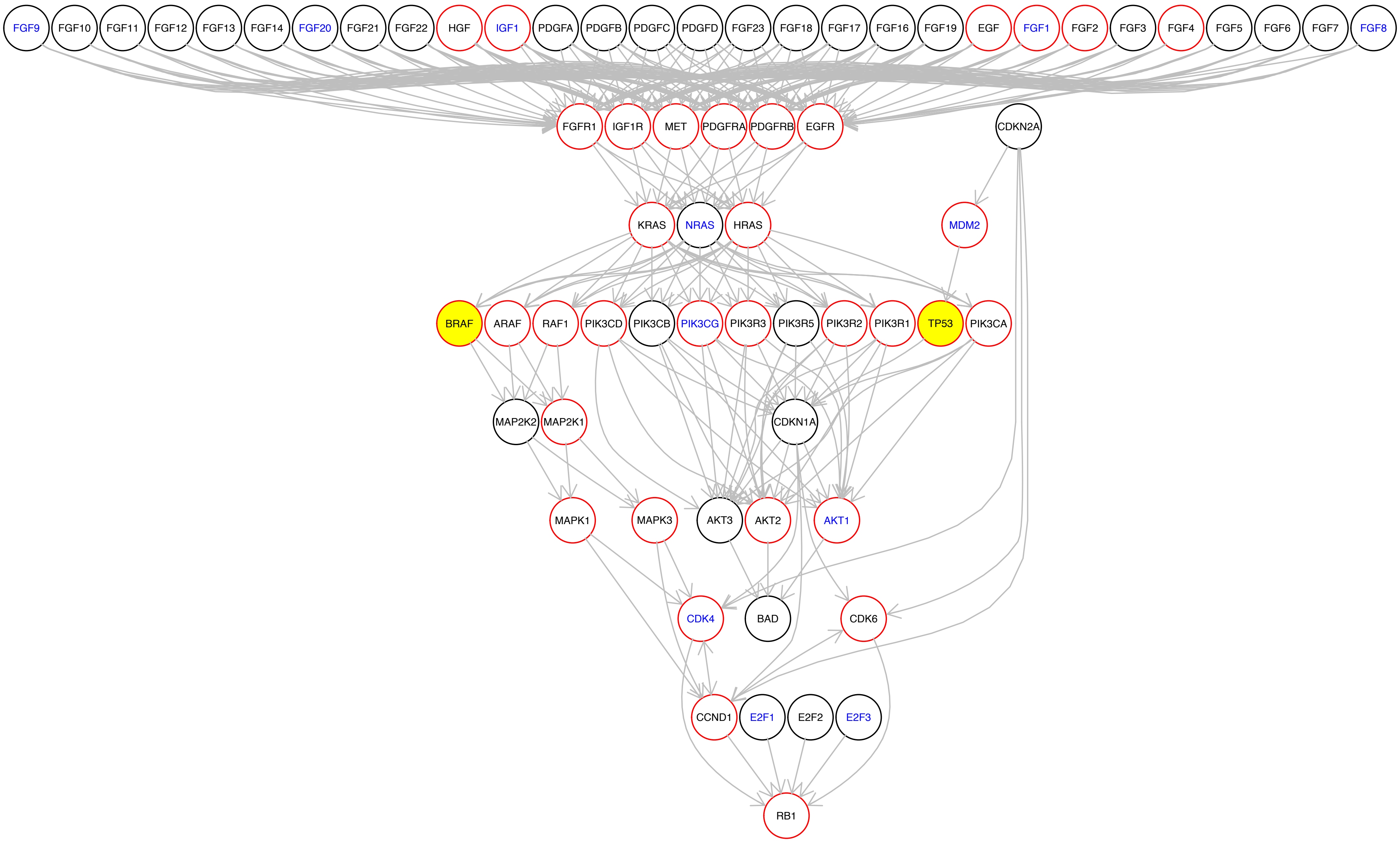

KEGG pathway = Melanoma : tumour = MEL.JWCI.WGS.12.Tumor : Yellow Fill = gene variant, Blue Text = expression−survival association, Red Boarder = drug

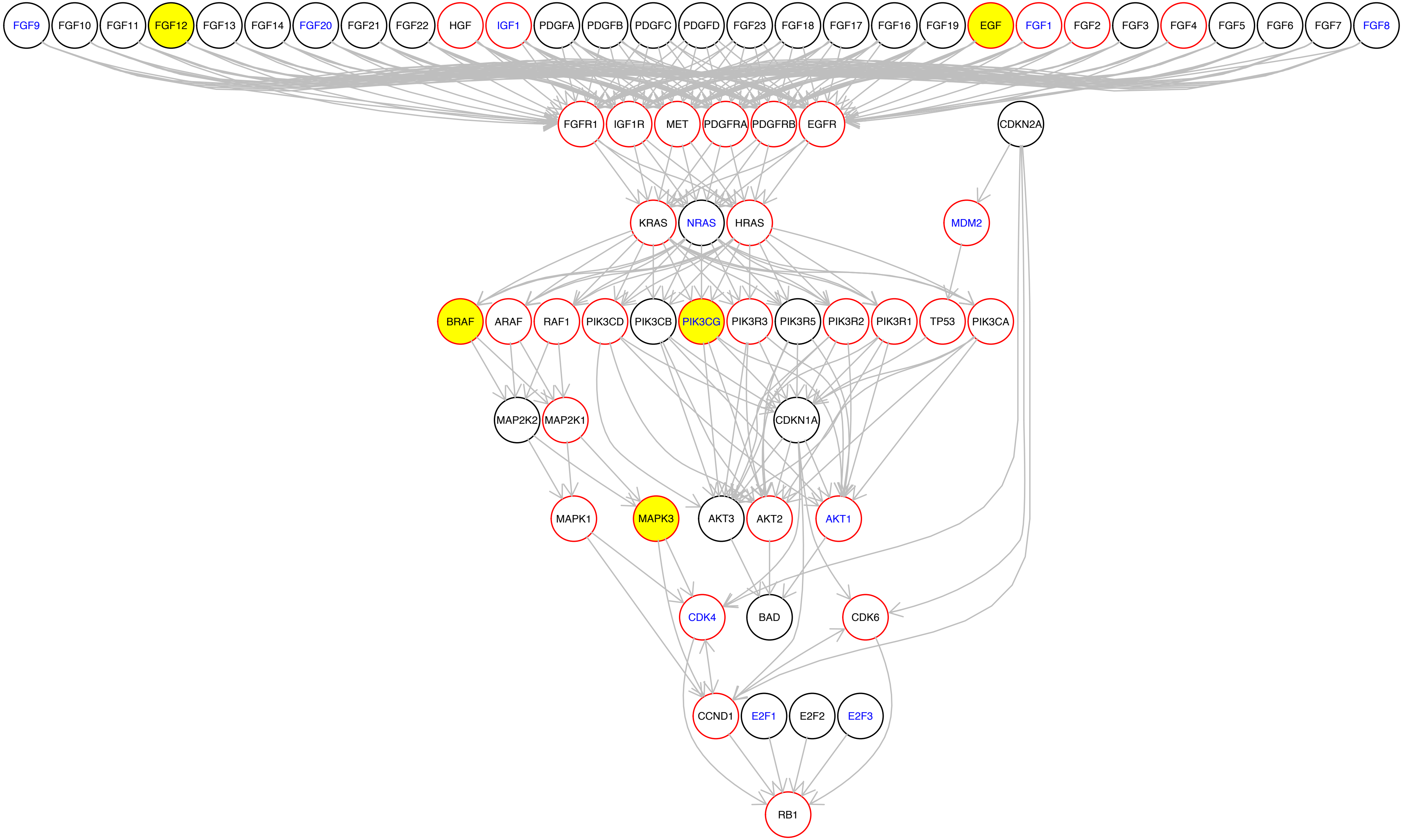

KEGG pathway = Melanoma : tumour = MEL.JWCI.WGS.13.Tumor : Yellow Fill = gene variant, Blue Text = expression−survival association, Red Boarder = drug

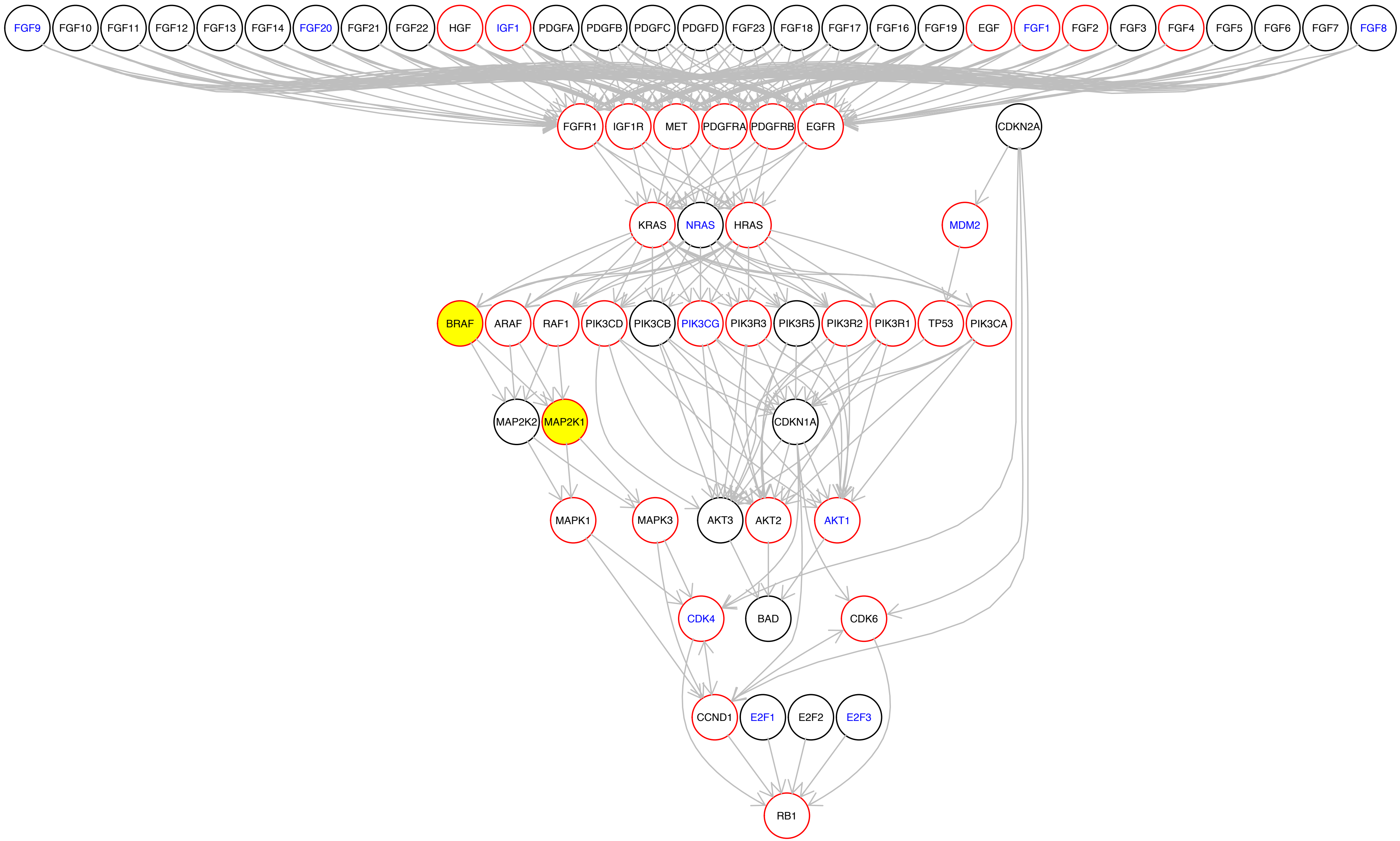

KEGG pathway = Melanoma :   tumour = MEL.JWCI.WGS.15.Tumor :   Yellow Fill = gene variant, Blue Text = expression−survival association, Red Boarder = drug

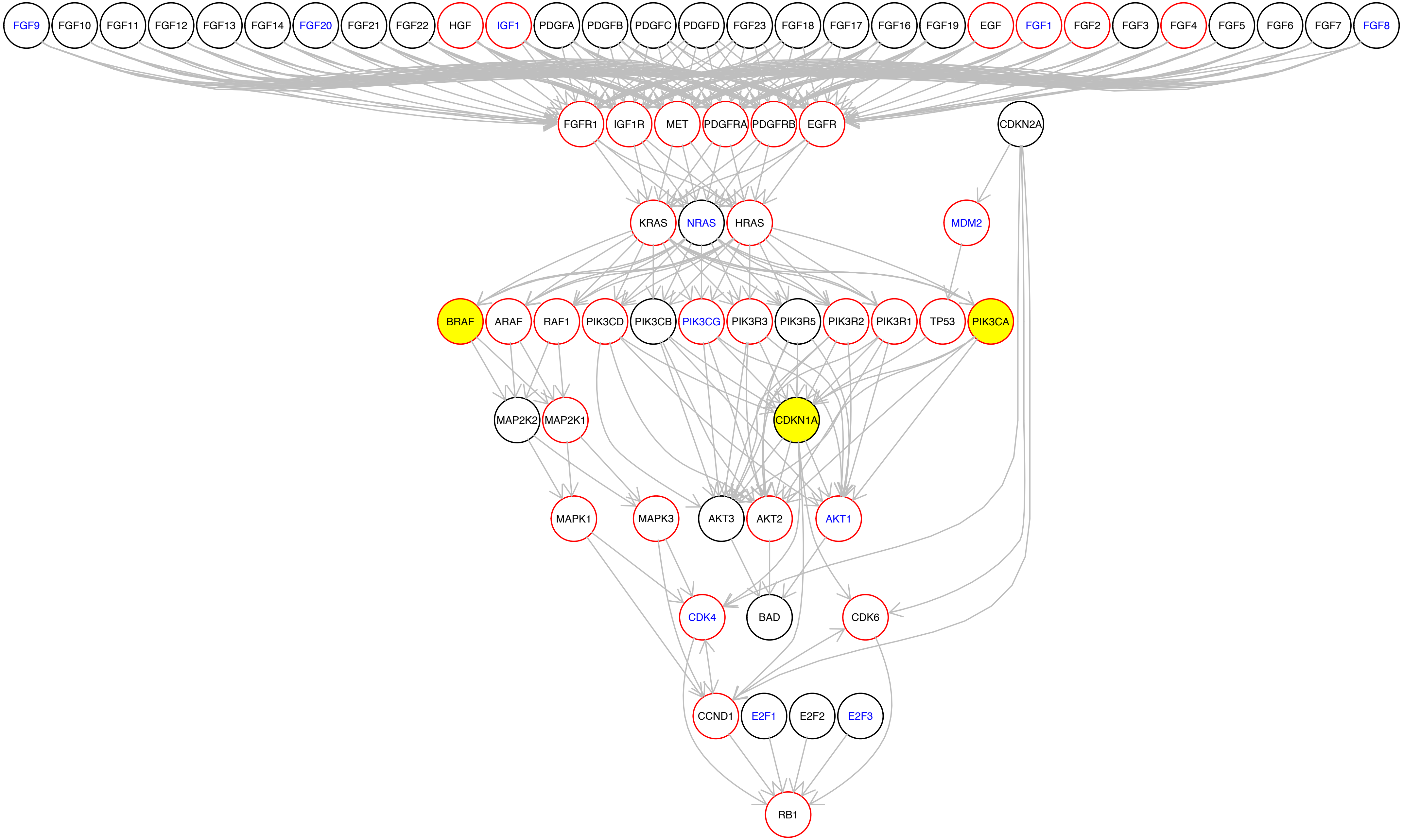

KEGG pathway = Melanoma : tumour = MEL.JWCI.WGS.18.Tumor : Yellow Fill = gene variant, Blue Text = expression−survival association, Red Boarder = drug

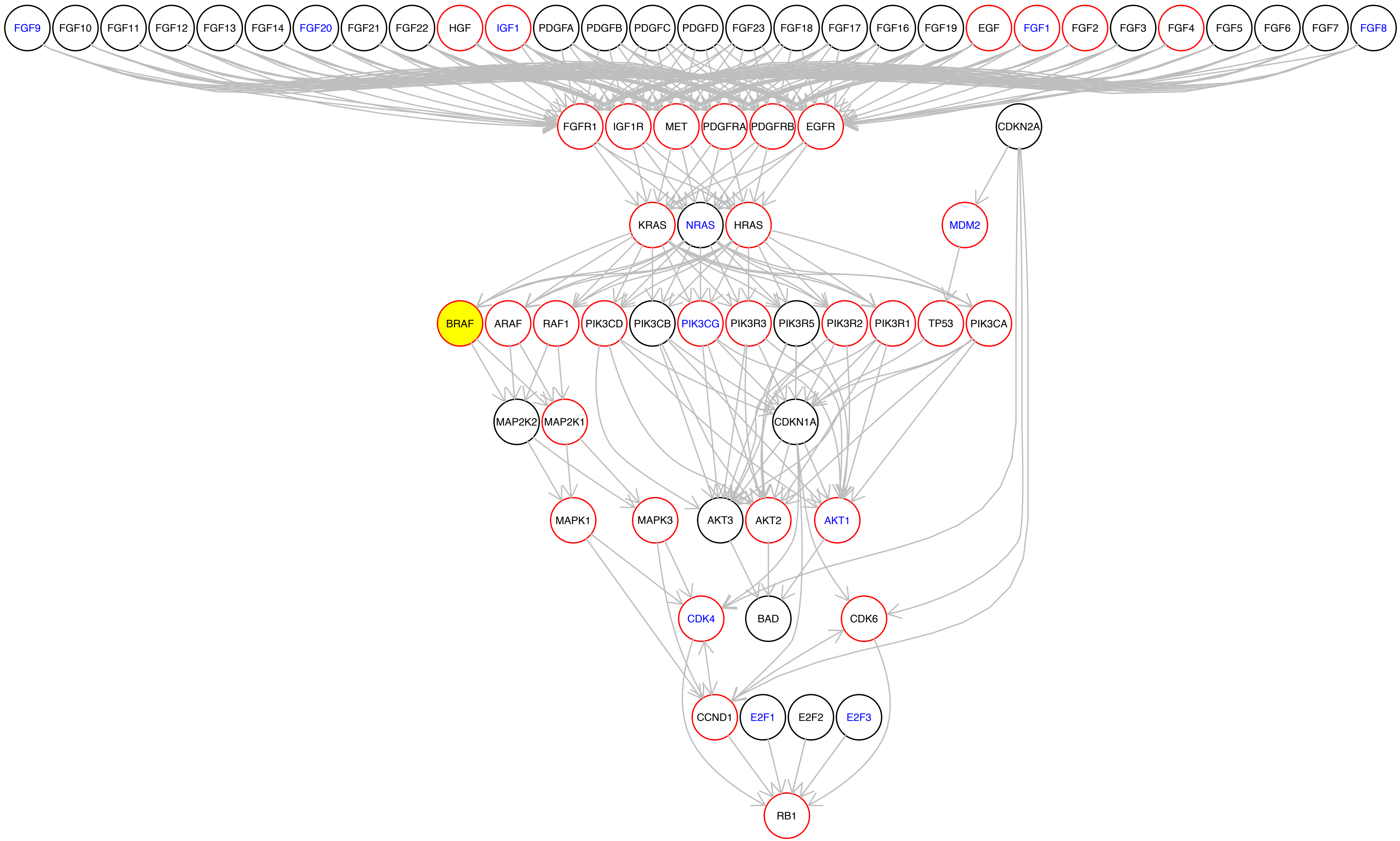

KEGG pathway = Melanoma : tumour = MEL.JWCI.WGS.19.Tumor : Yellow Fill = gene variant, Blue Text = expression−survival association, Red Boarder = drug

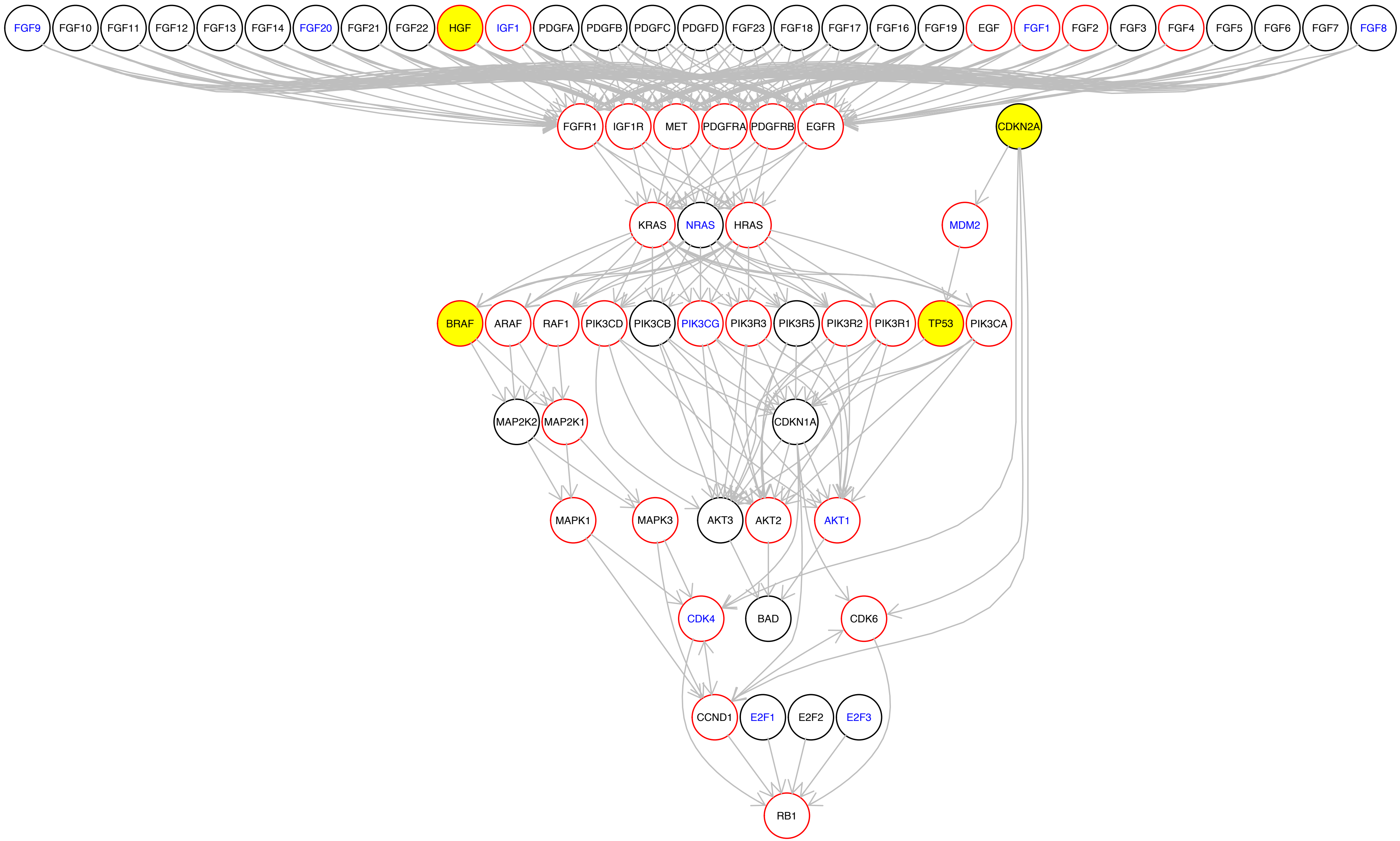

KEGG pathway = Melanoma :   tumour = MEL.JWCI.WGS.1.Tumor :   Yellow Fill = gene variant, Blue Text = expression−survival association, Red Boarder = drug

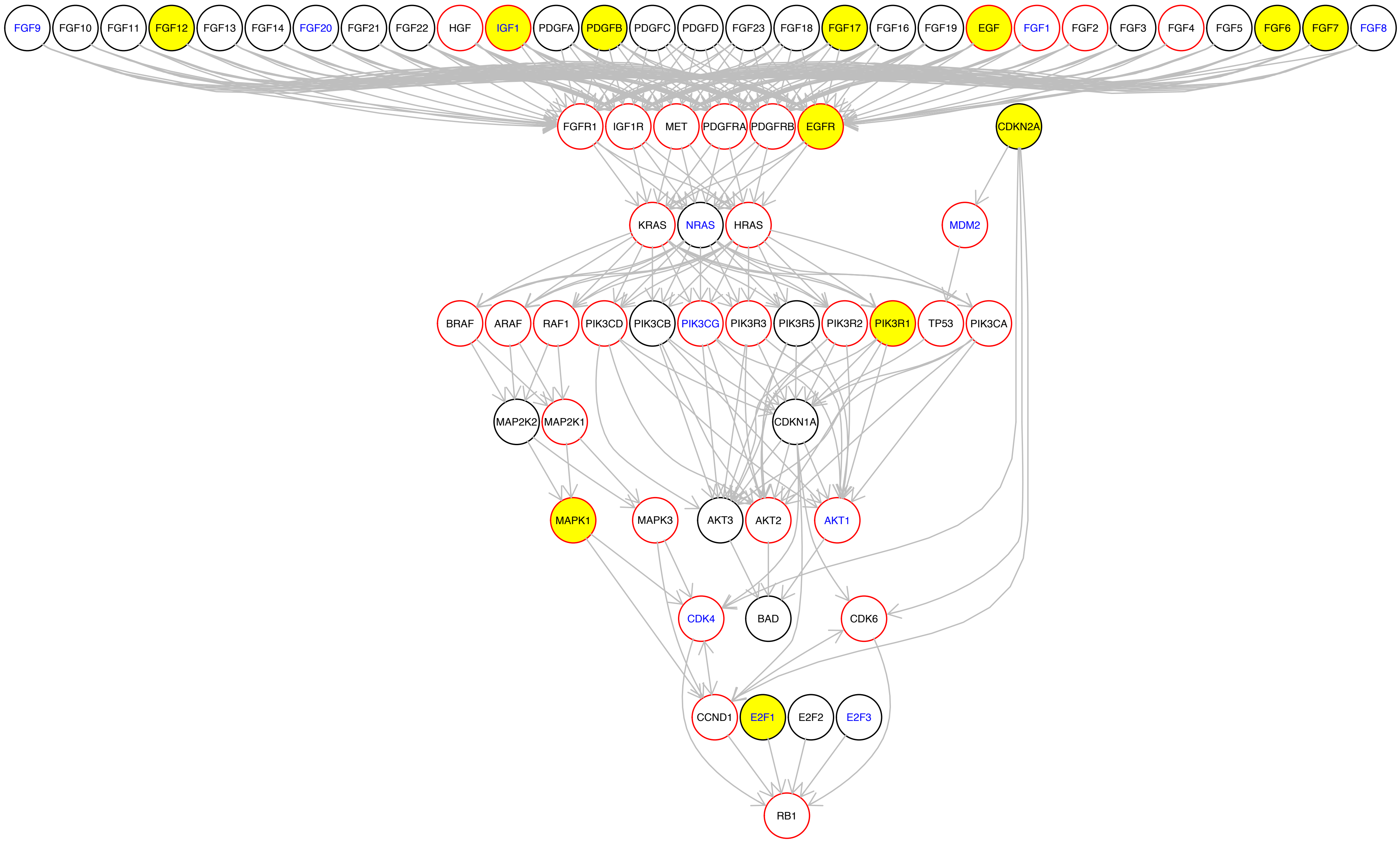

KEGG pathway = Melanoma : tumour = MEL.JWCI.WGS.20.Tumor : Yellow Fill = gene variant, Blue Text = expression−survival association, Red Boarder = drug

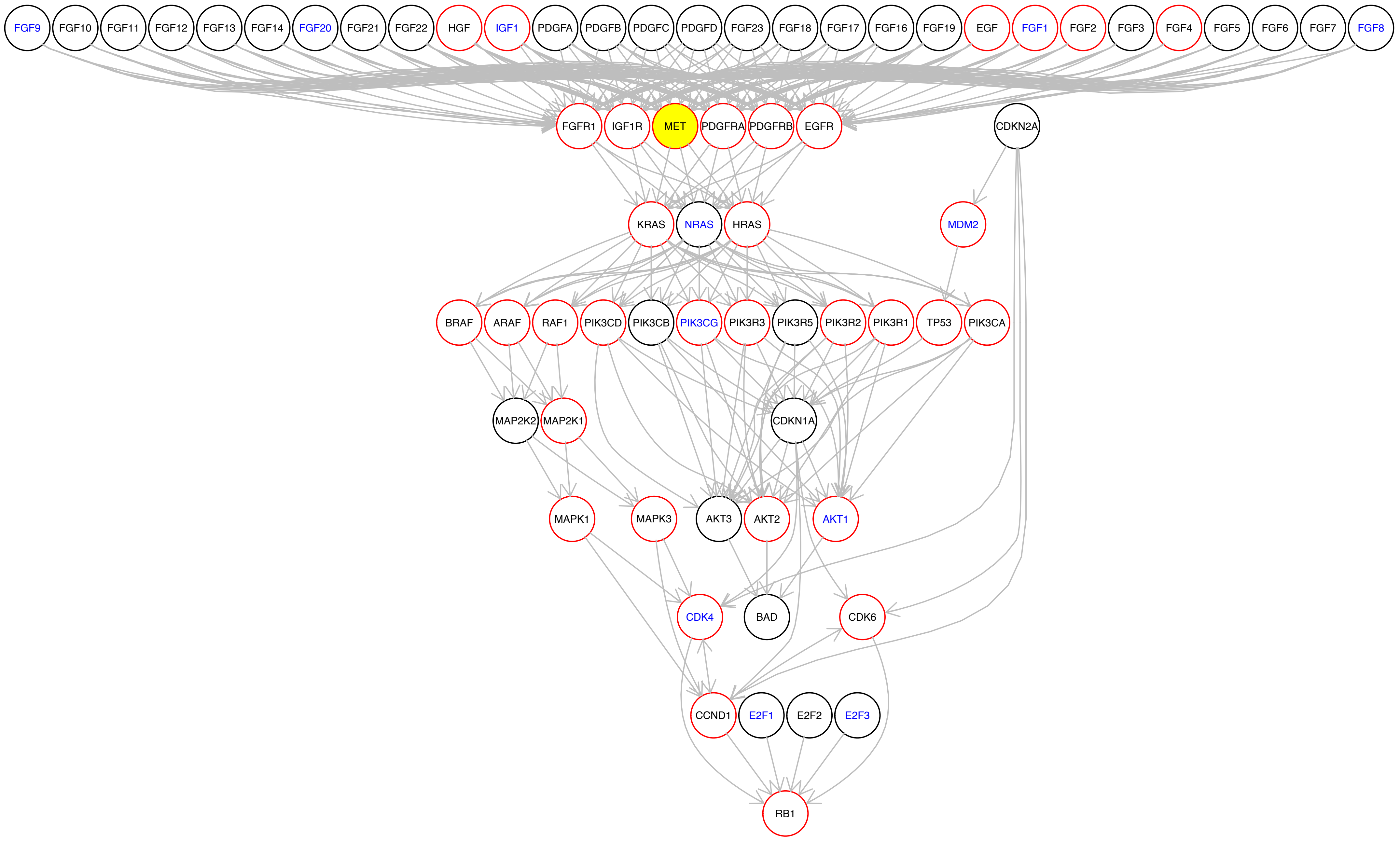

KEGG pathway = Melanoma : tumour = MEL.JWCI.WGS.21.Tumor : Yellow Fill = gene variant, Blue Text = expression−survival association, Red Boarder = drug

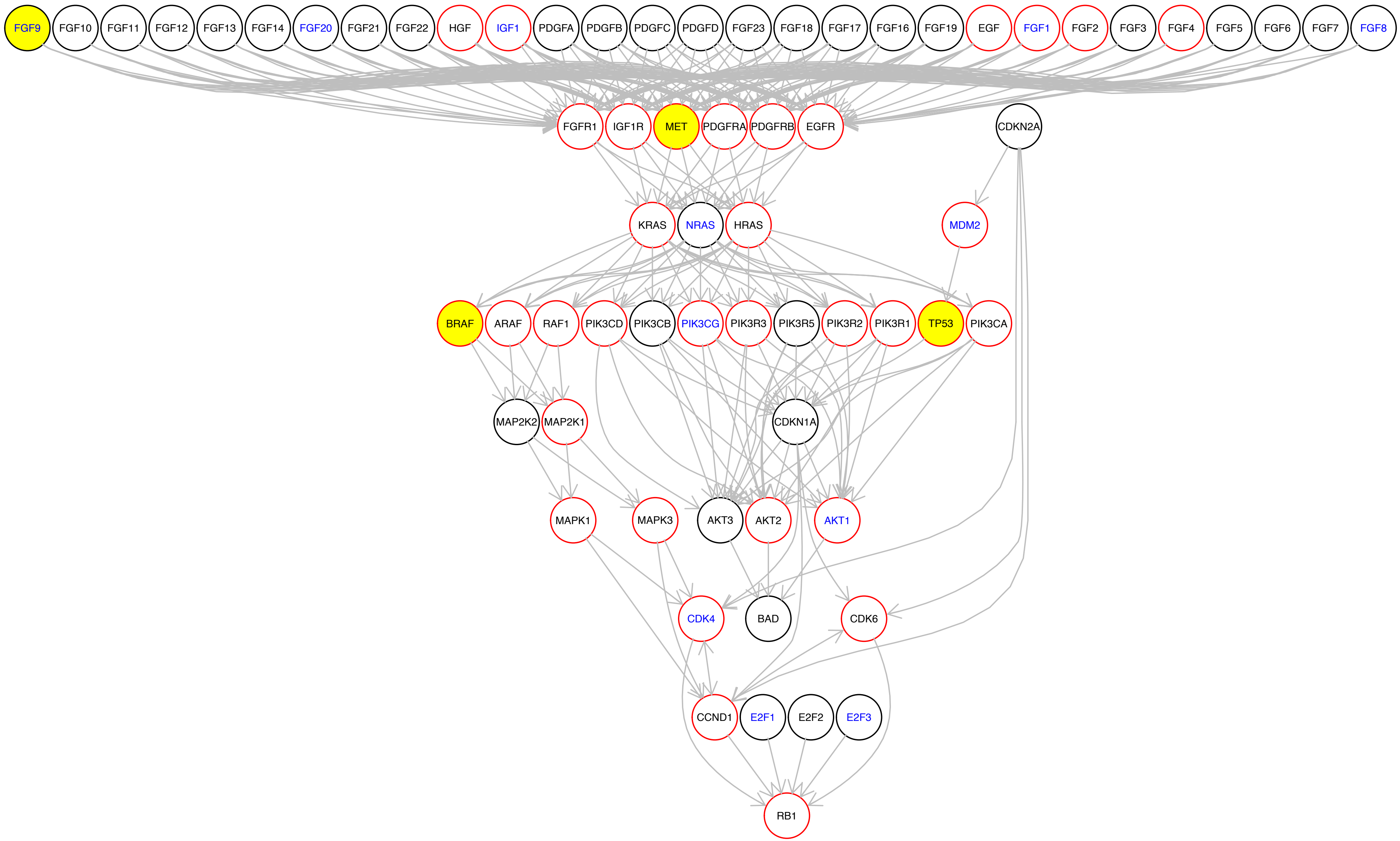

KEGG pathway = Melanoma : tumour = MEL.JWCI.WGS.22.Tumor : Yellow Fill = gene variant, Blue Text = expression–survival association, Red Boarder = drug

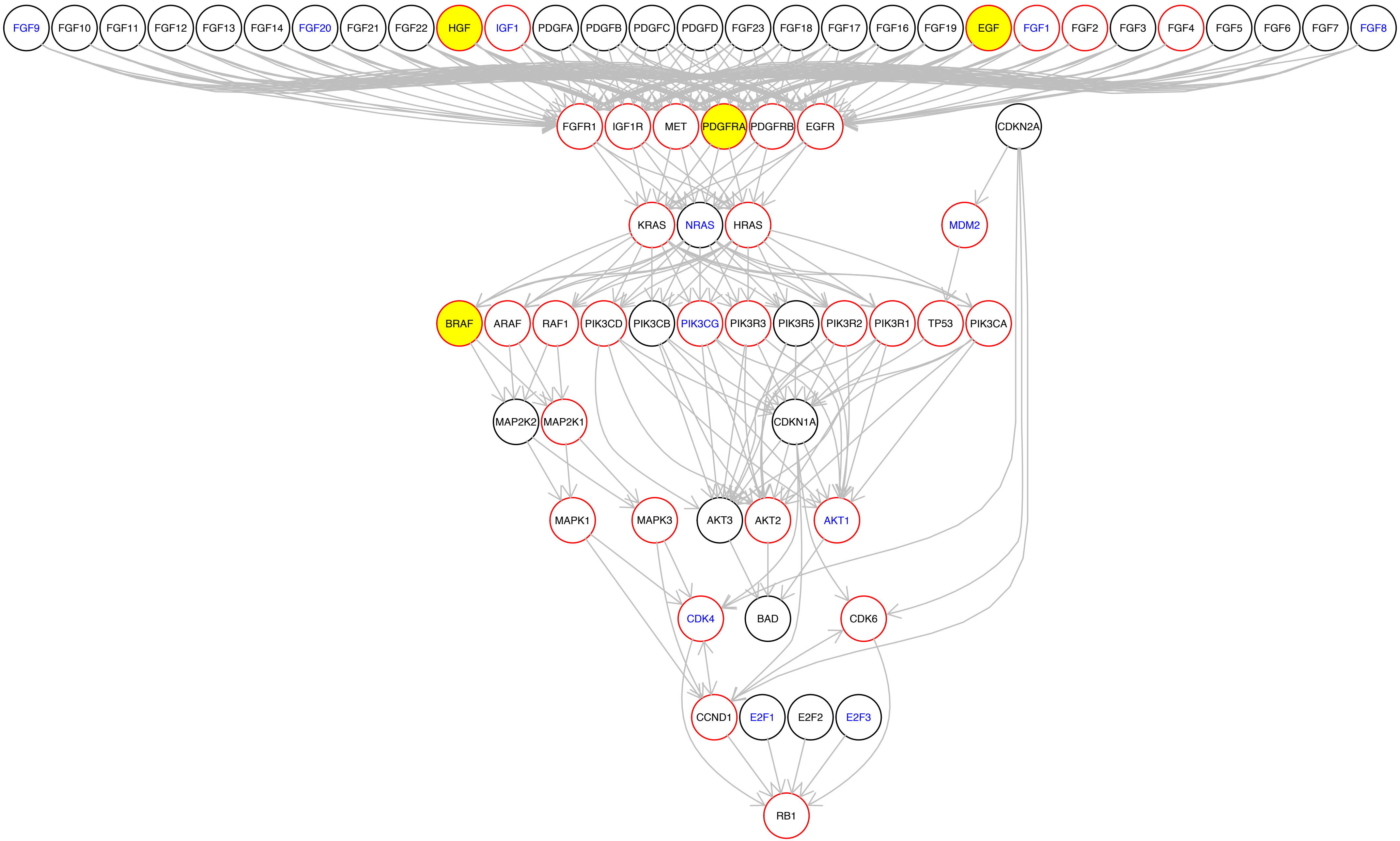

KEGG pathway = Melanoma : tumour = MEL.JWCI.WGS.23.Tumor : Yellow Fill = gene variant, Blue Text = expression−survival association, Red Boarder = drug

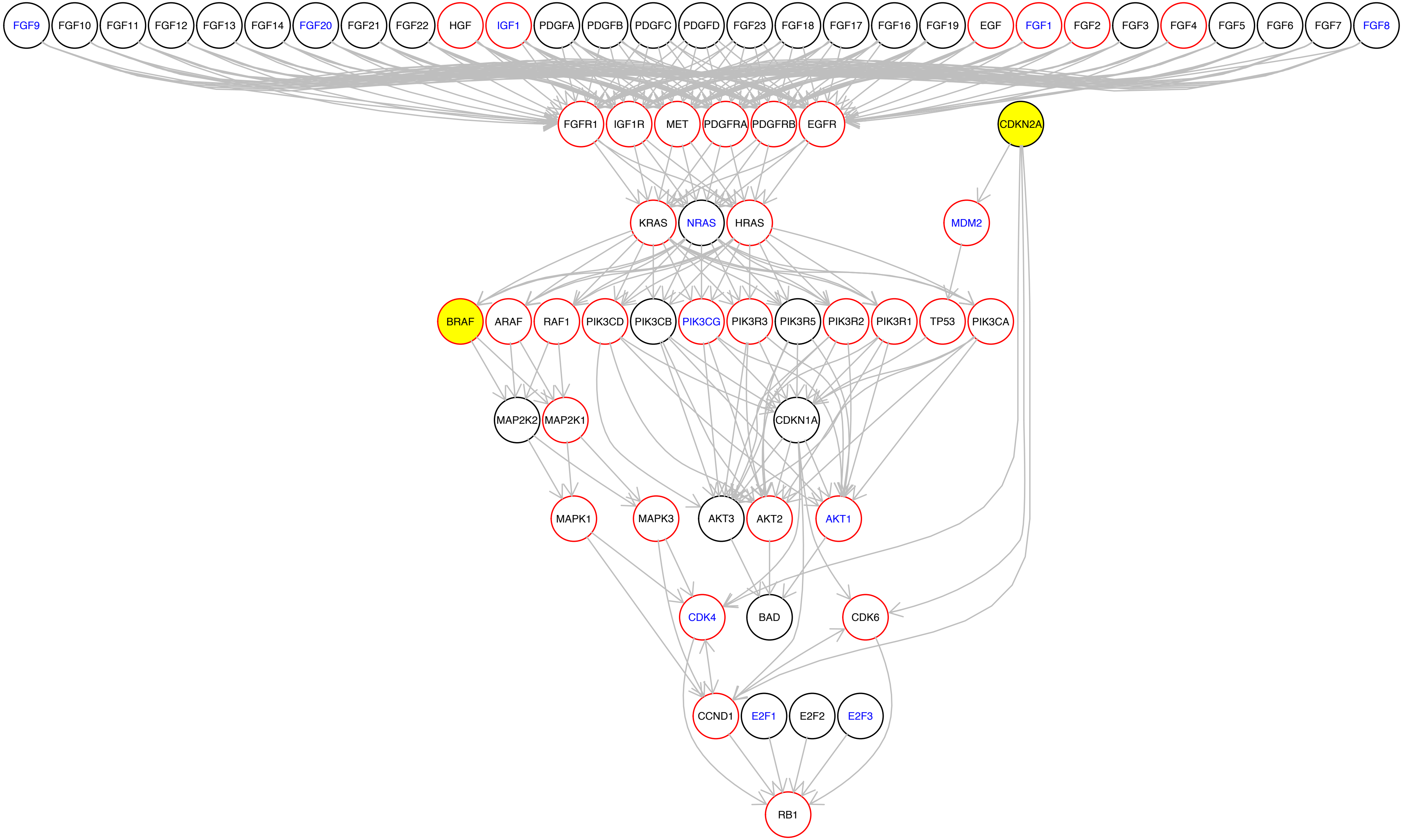

KEGG pathway = Melanoma :   tumour = MEL.JWCI.WGS.24.Tumor :   Yellow Fill = gene variant, Blue Text = expression–survival association, Red Boarder = drug

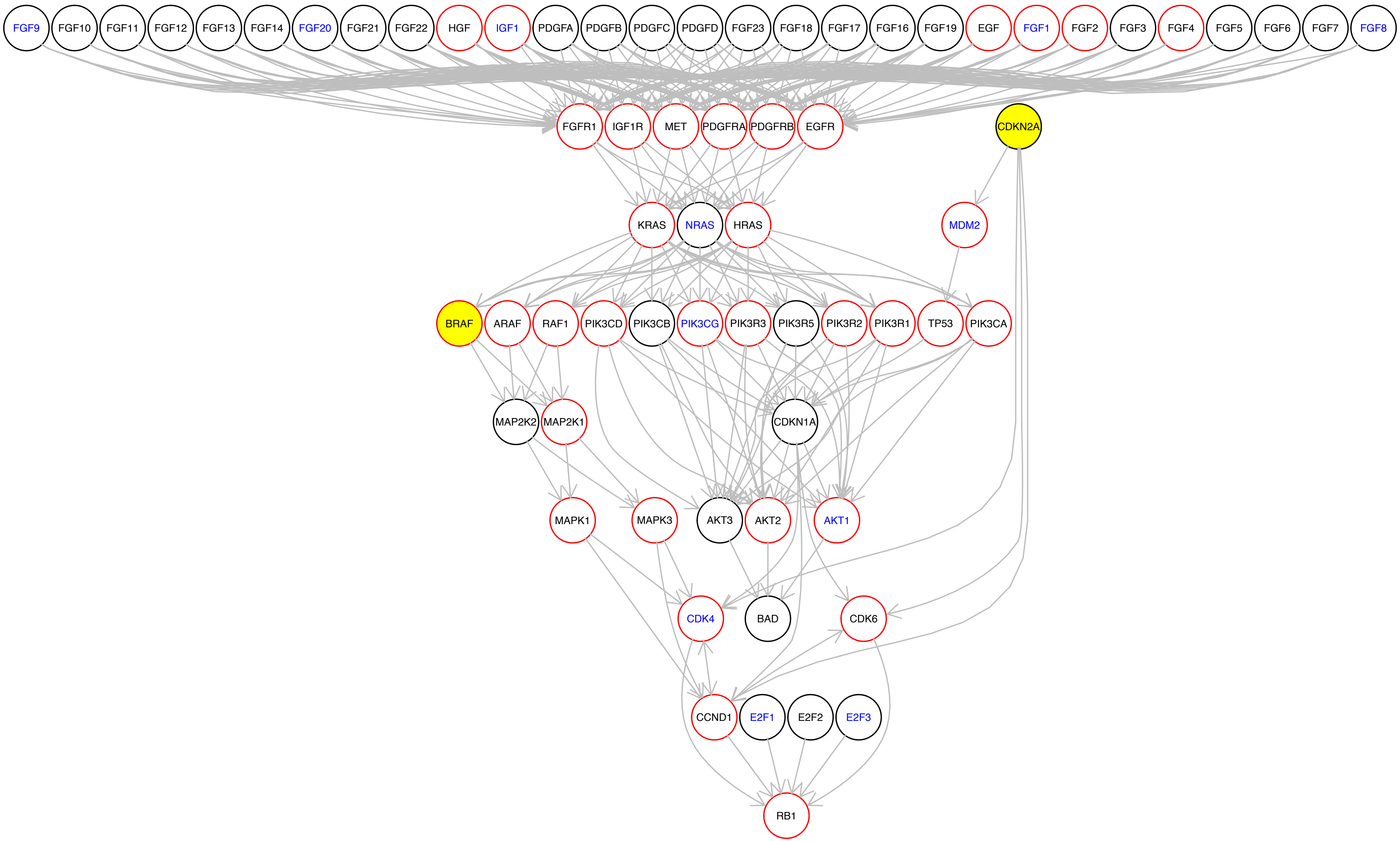

KEGG pathway = Melanoma : tumour = MEL.JWCI.WGS.25.Tumor : Yellow Fill = gene variant, Blue Text = expression−survival association, Red Boarder = drug

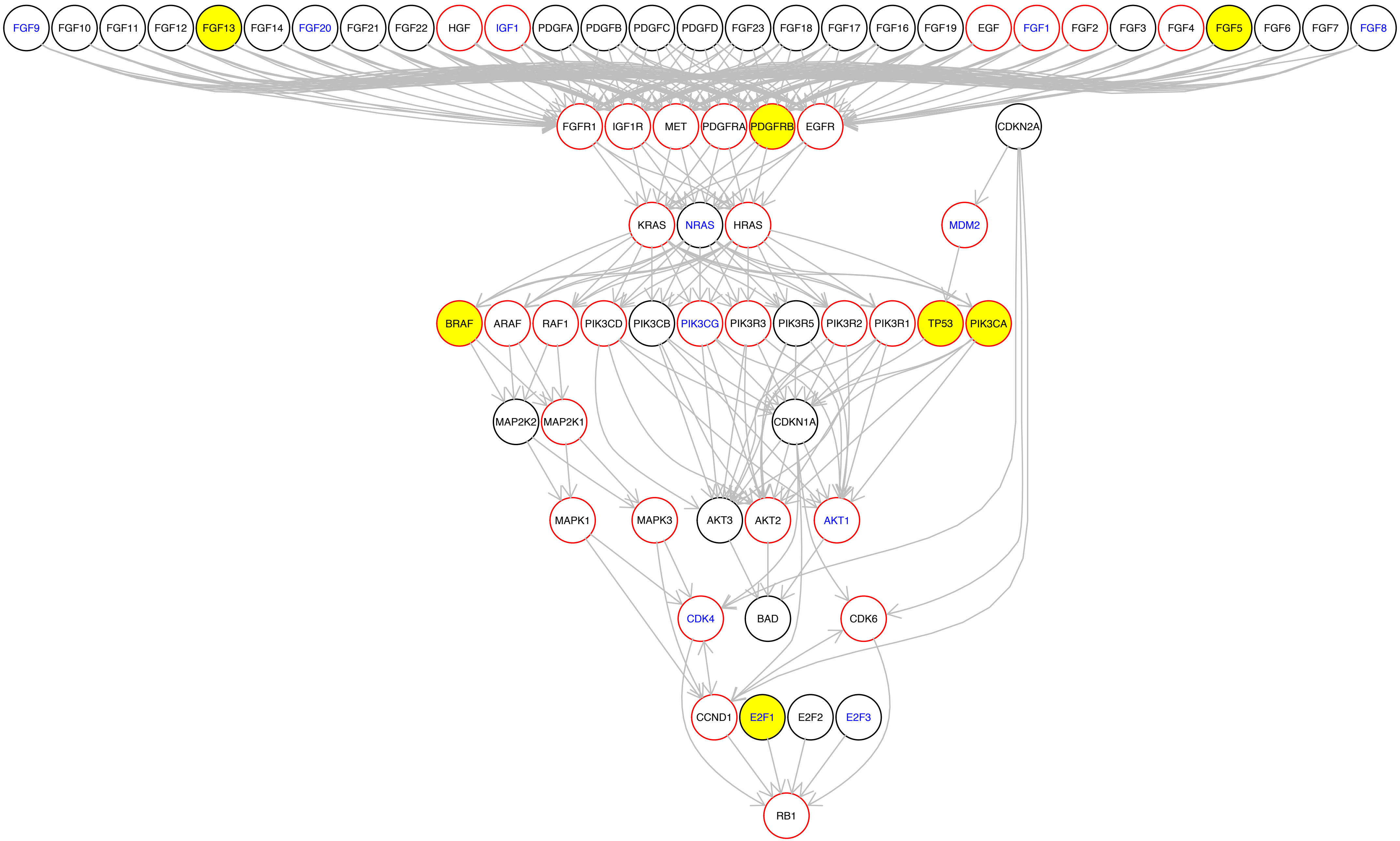

KEGG pathway = Melanoma : tumour = MEL.JWCI.WGS.26.Tumor : Yellow Fill = gene variant, Blue Text = expression−survival association, Red Boarder = drug

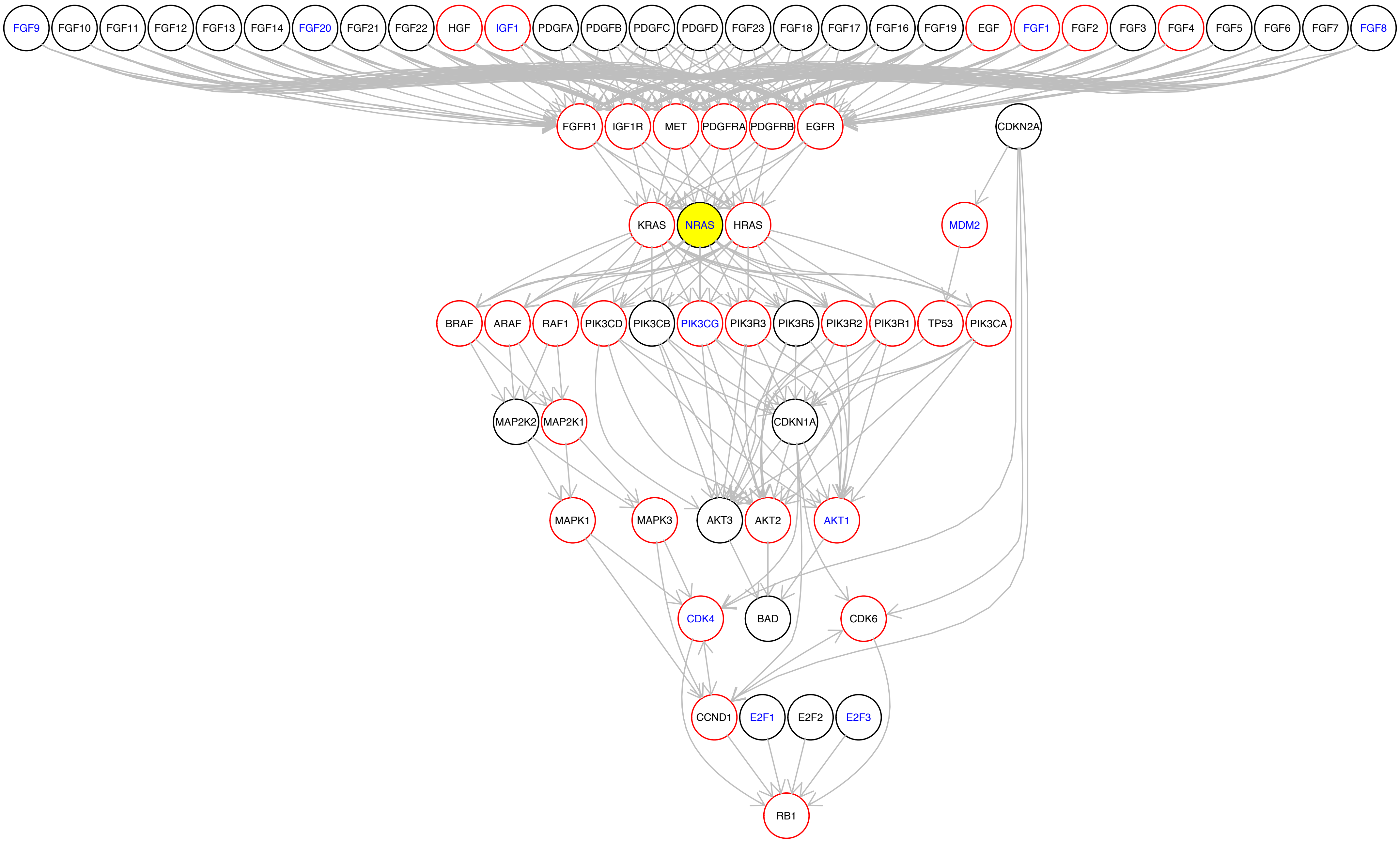

KEGG pathway = Melanoma : tumour = MEL.JWCI.WGS.29.Tumor : Yellow Fill = gene variant, Blue Text = expression−survival association, Red Boarder = drug

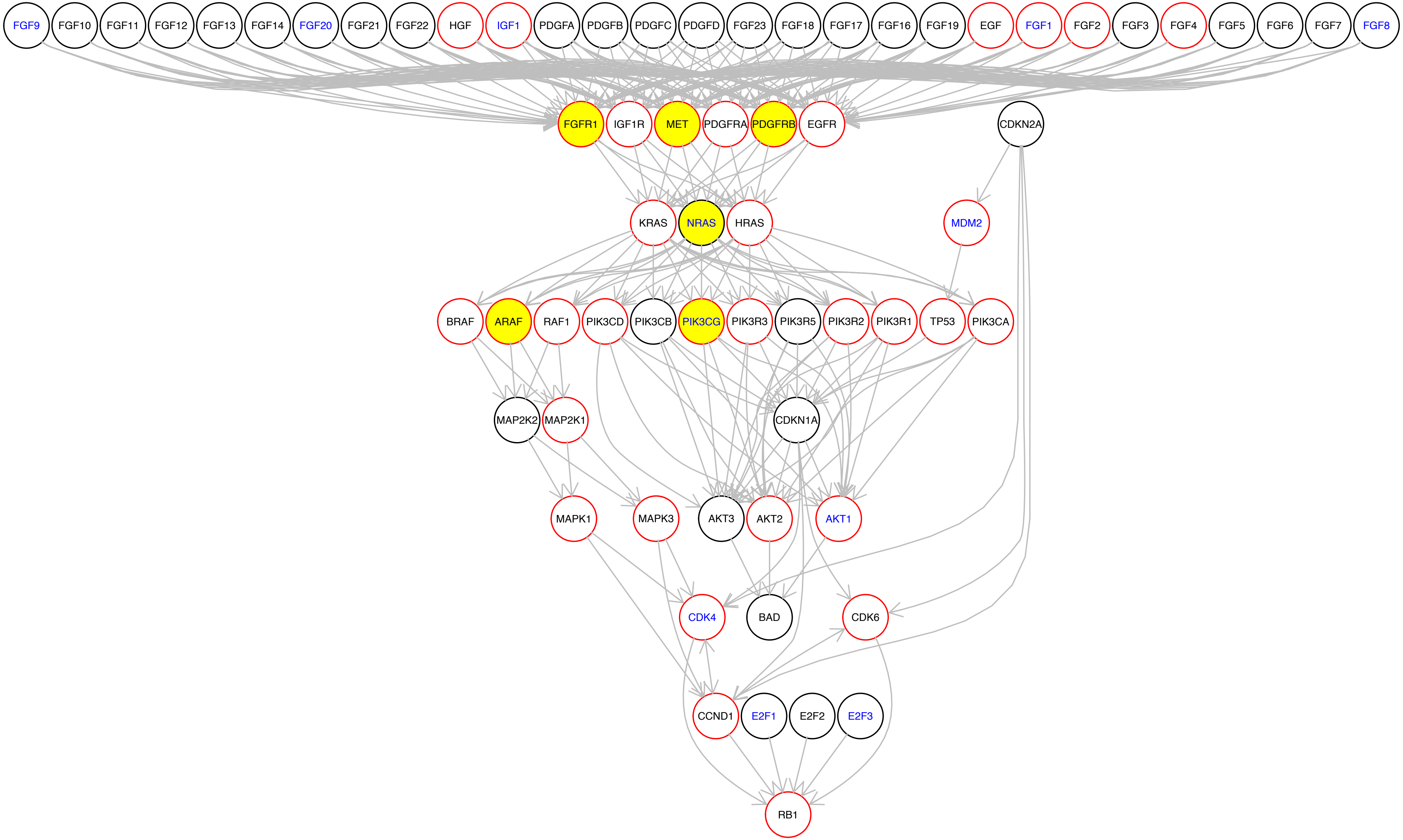

KEGG pathway = Melanoma :   tumour = MEL.JWCI.WGS.2.Tumor :   Yellow Fill = gene variant, Blue Text = expression–survival association, Red Boarder = drug

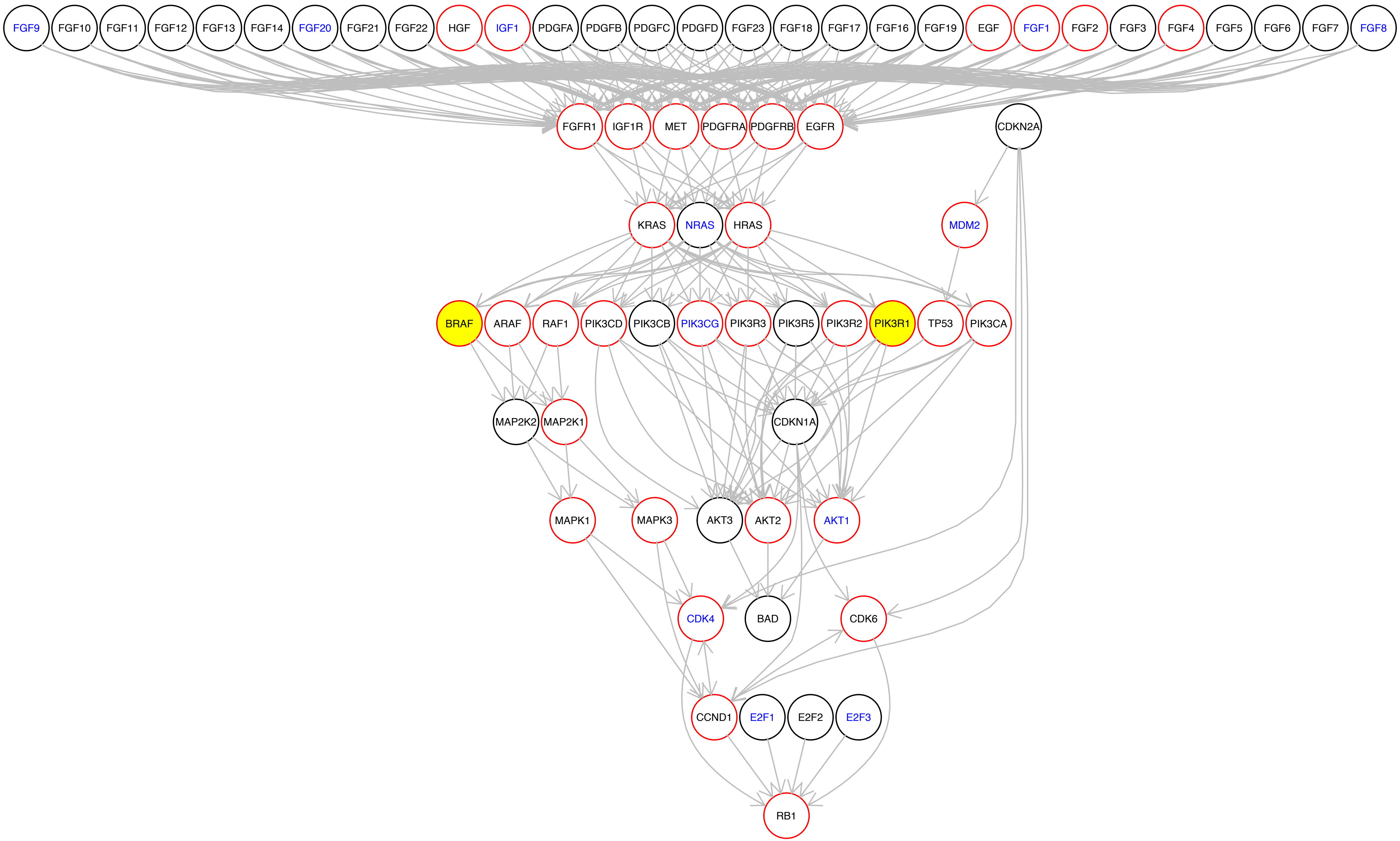

KEGG pathway = Melanoma : tumour = MEL.JWCI.WGS.31.Tumor : Yellow Fill = gene variant, Blue Text = expression–survival association, Red Boarder = drug

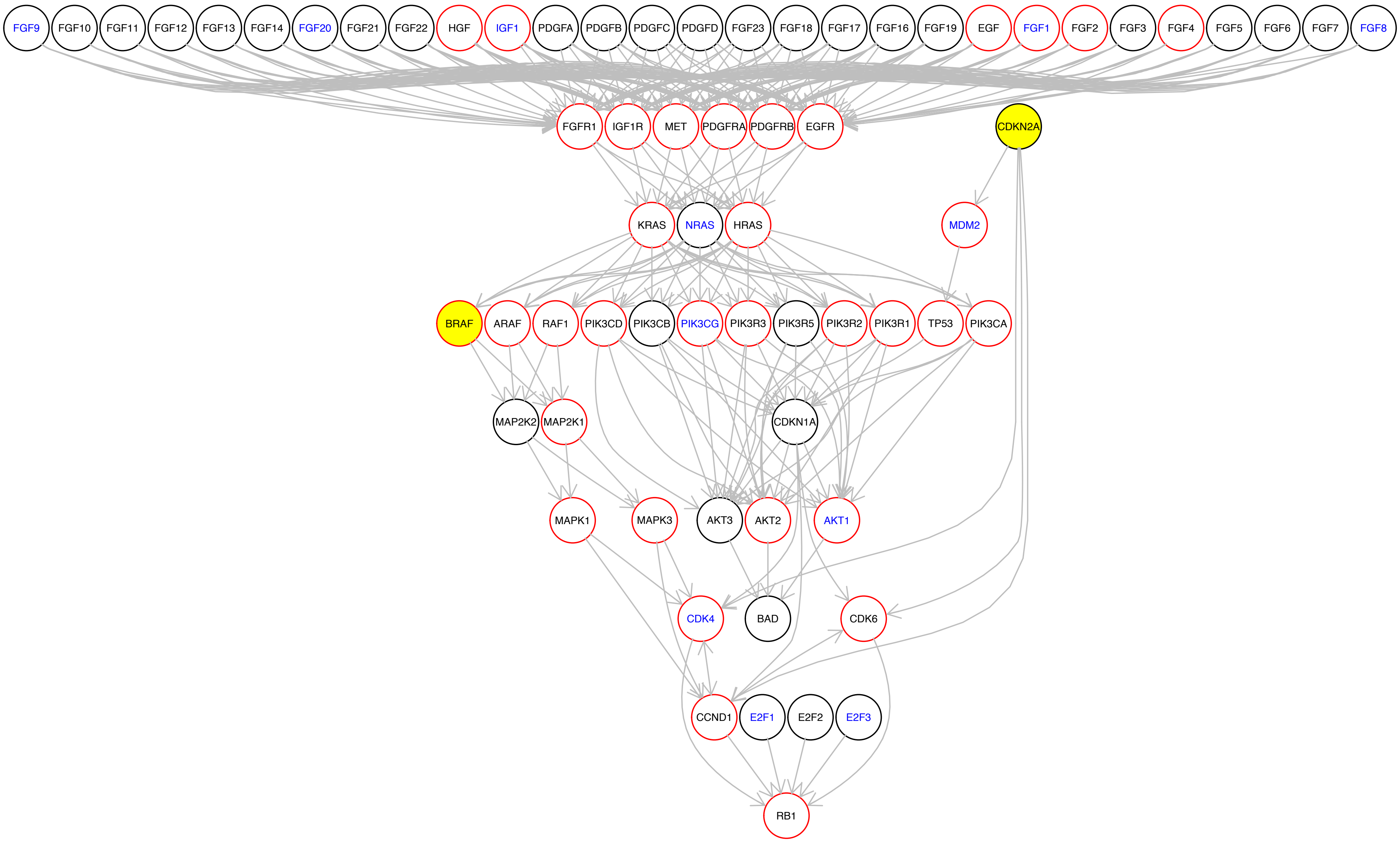

KEGG pathway = Melanoma : tumour = MEL.JWCI.WGS.32.Tumor : Yellow Fill = gene variant, Blue Text = expression–survival association, Red Boarder = drug

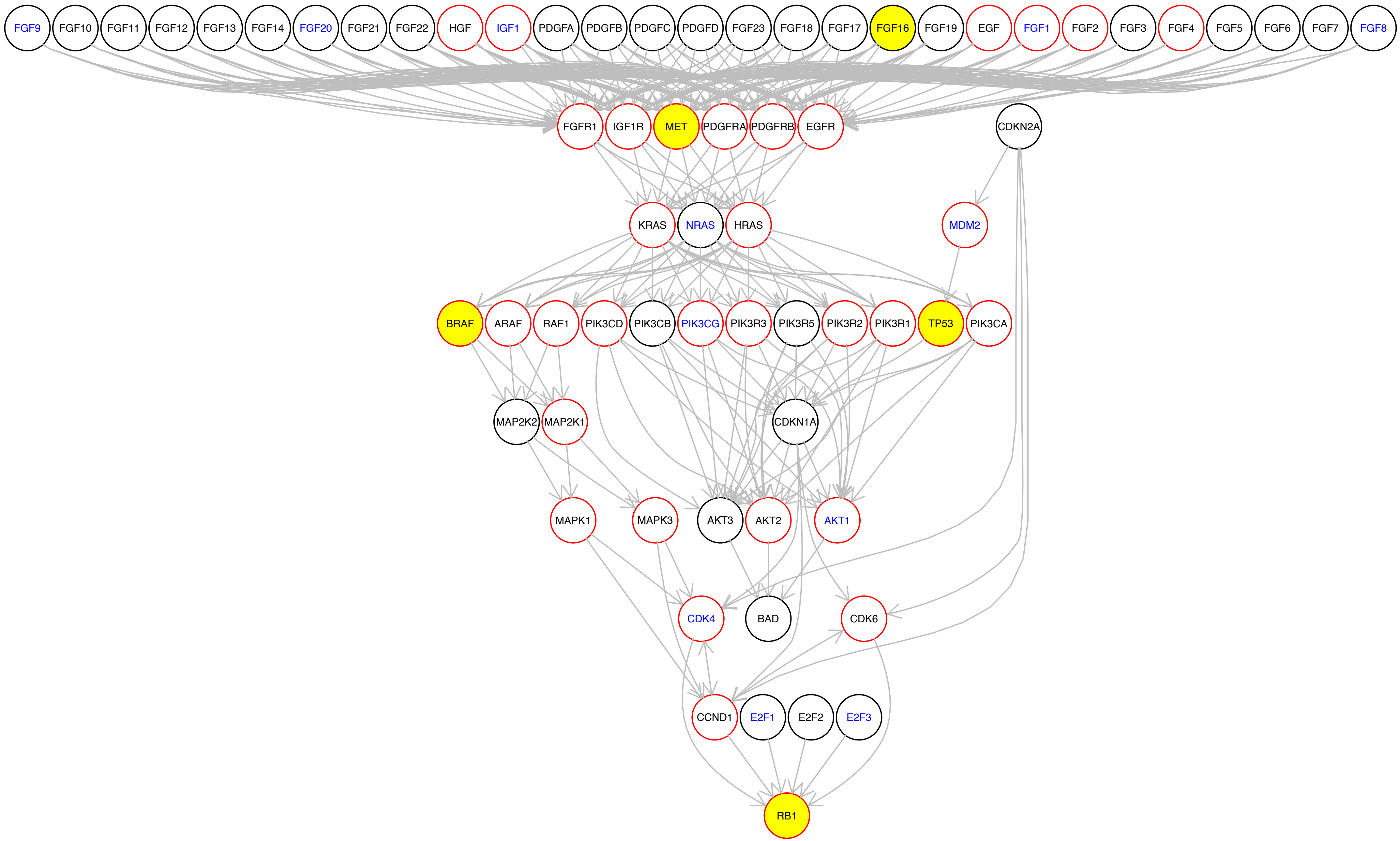

KEGG pathway = Melanoma : tumour = MEL.JWCI.WGS.33.Tumor : Yellow Fill = gene variant, Blue Text = expression–survival association, Red Boarder = drug

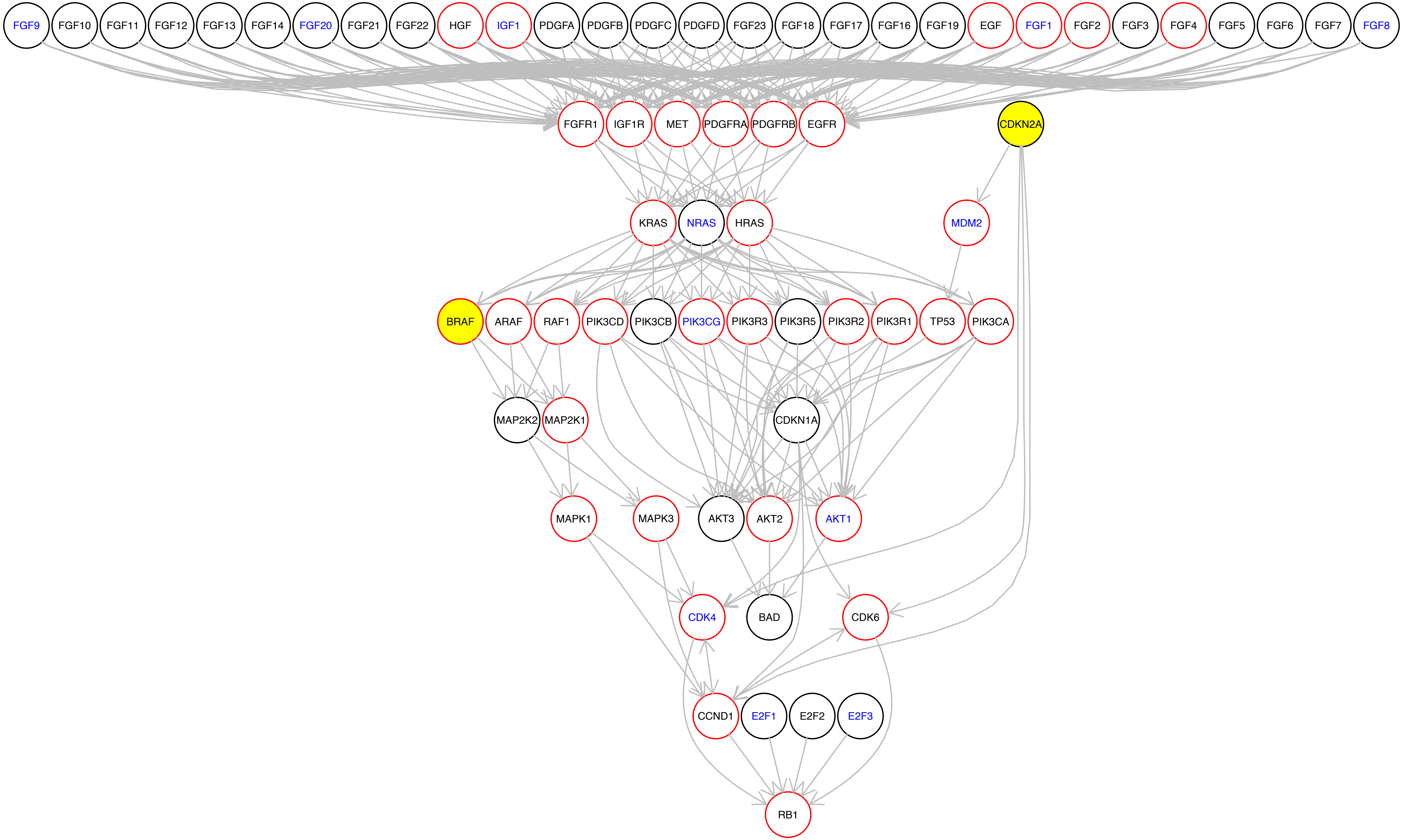

KEGG pathway = Melanoma : tumour = MEL.JWCI.WGS.34.Tumor : Yellow Fill = gene variant, Blue Text = expression−survival association, Red Boarder = drug

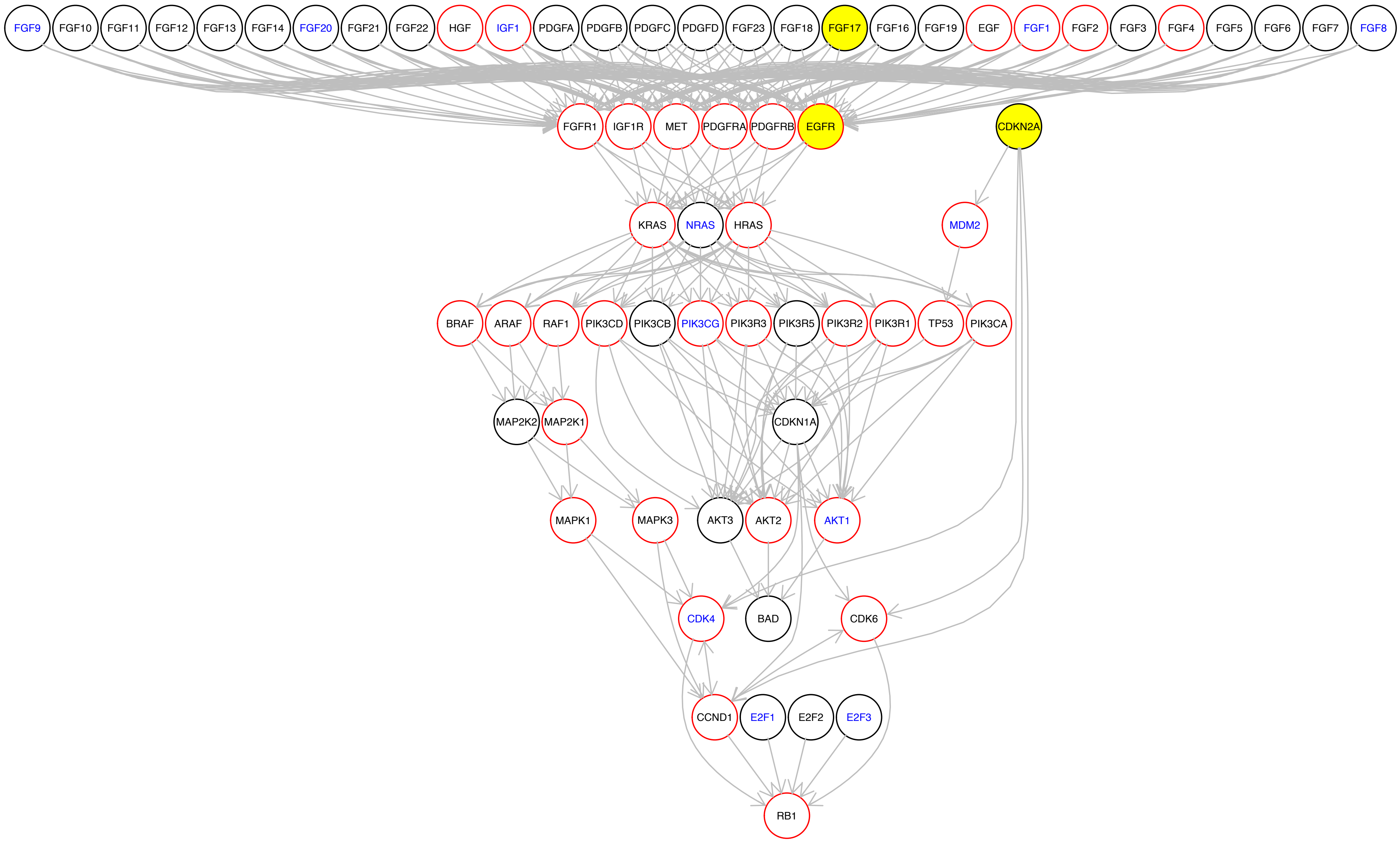

KEGG pathway = Melanoma : tumour = MEL.JWCI.WGS.35.Tumor : Yellow Fill = gene variant, Blue Text = expression−survival association, Red Boarder = drug

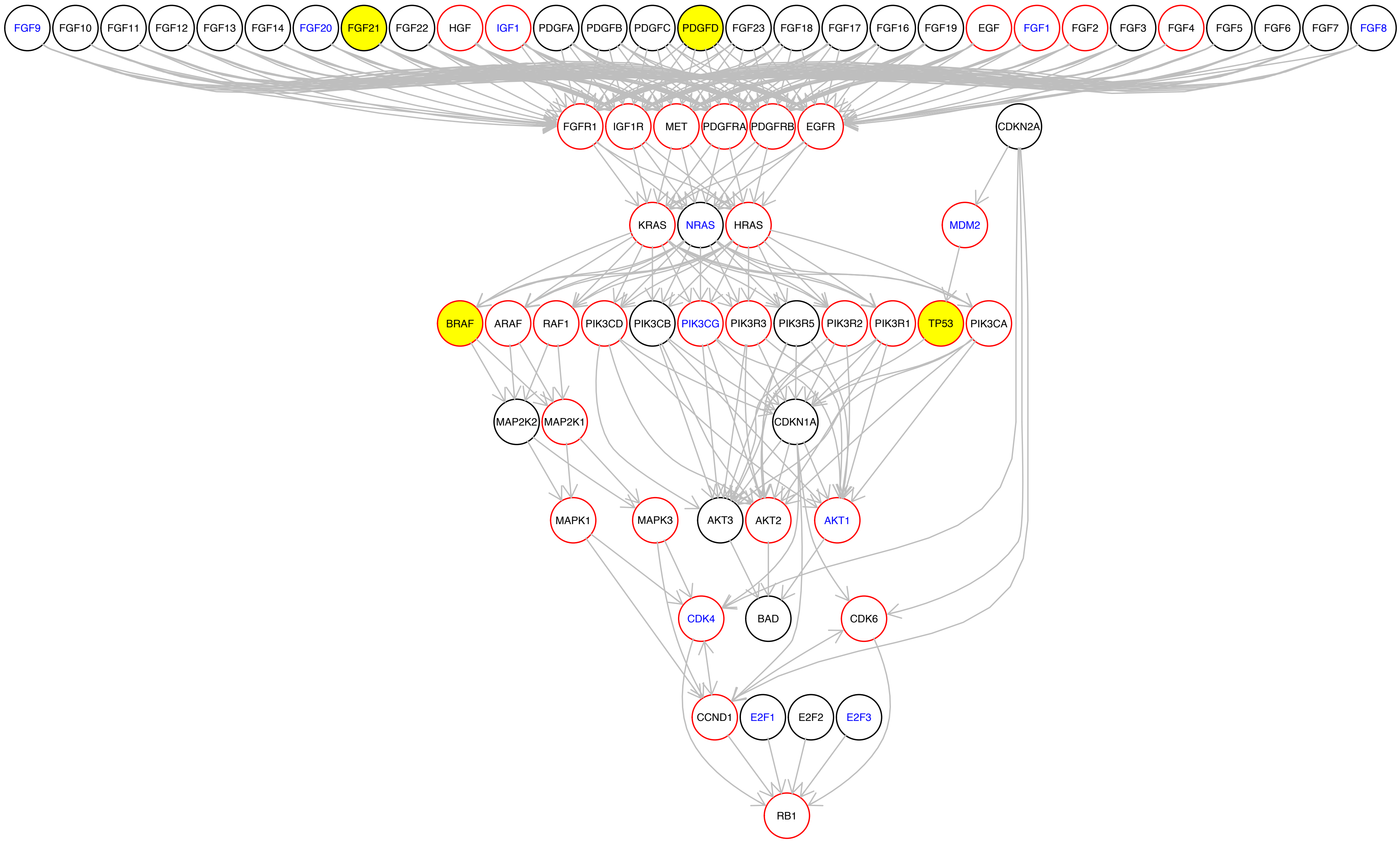

KEGG pathway = Melanoma : tumour = MEL.JWCI.WGS.36.Tumor : Yellow Fill = gene variant, Blue Text = expression−survival association, Red Boarder = drug

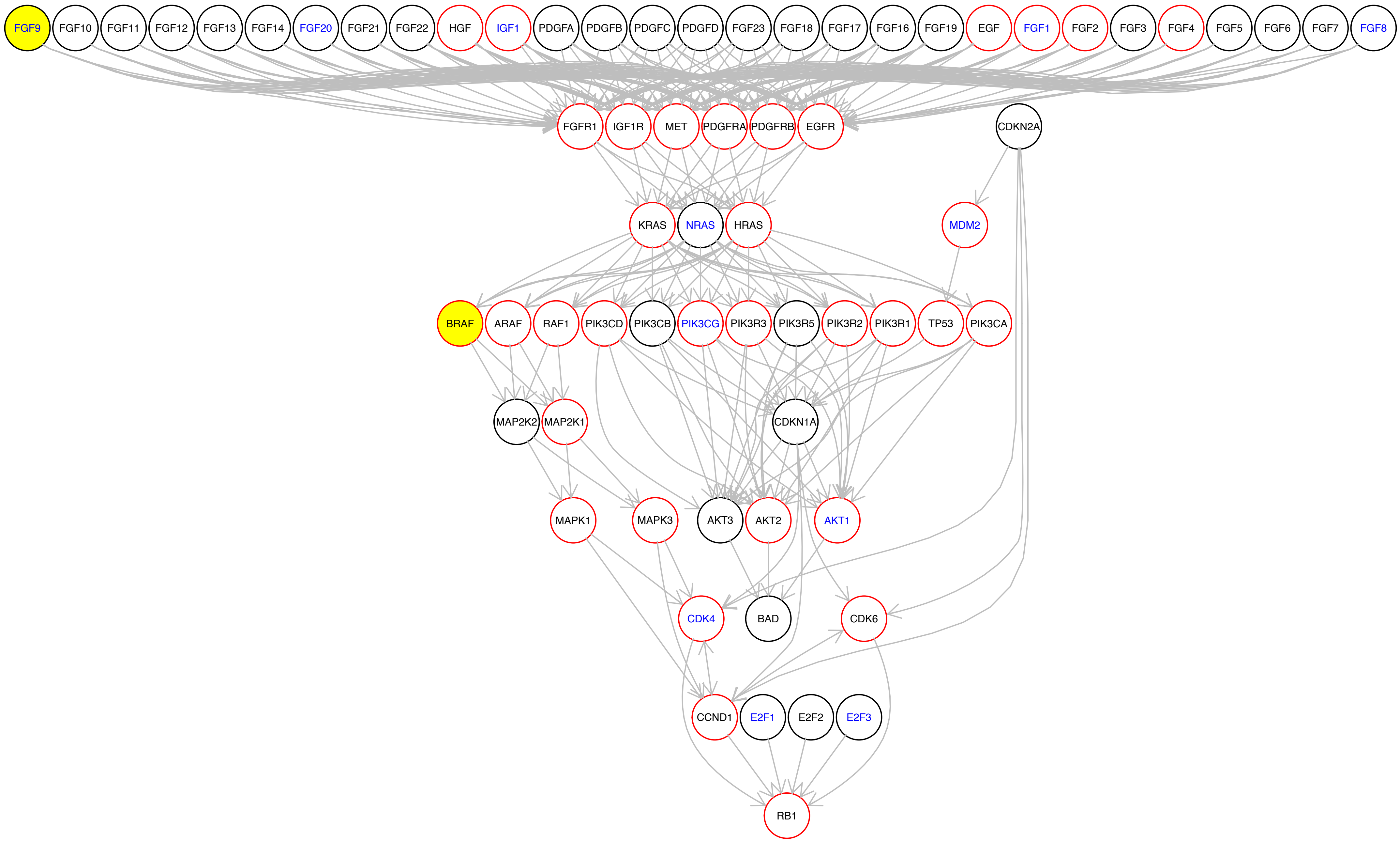

KEGG pathway = Melanoma : tumour = MEL.JWCI.WGS.37.Tumor : Yellow Fill = gene variant, Blue Text = expression−survival association, Red Boarder = drug

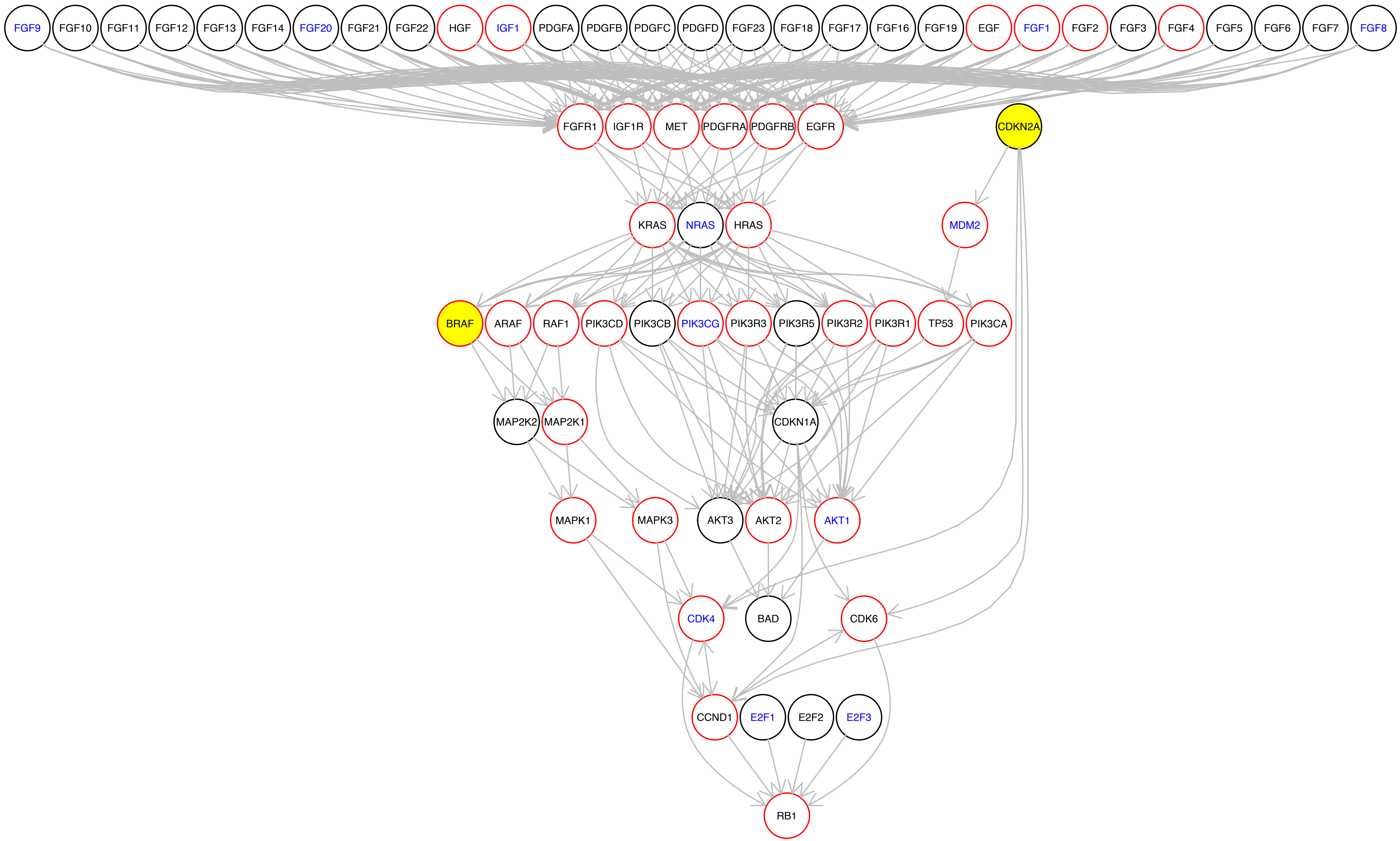

KEGG pathway = Melanoma : tumour = MEL.JWCI.WGS.38.Tumor : Yellow Fill = gene variant, Blue Text = expression−survival association, Red Boarder = drug

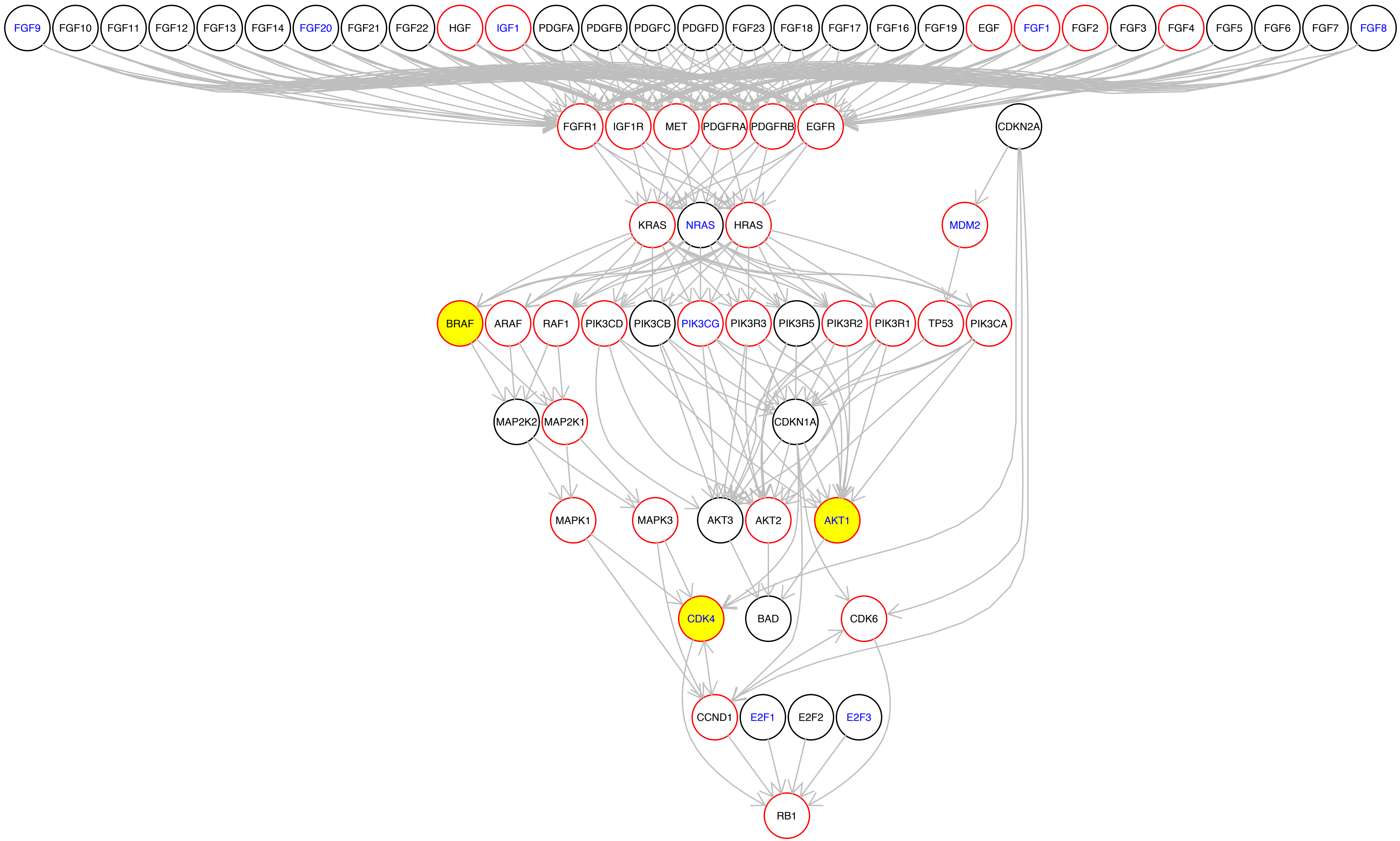

KEGG pathway = Melanoma : tumour = MEL.JWCI.WGS.39.Tumor : Yellow Fill = gene variant, Blue Text = expression−survival association, Red Boarder = drug

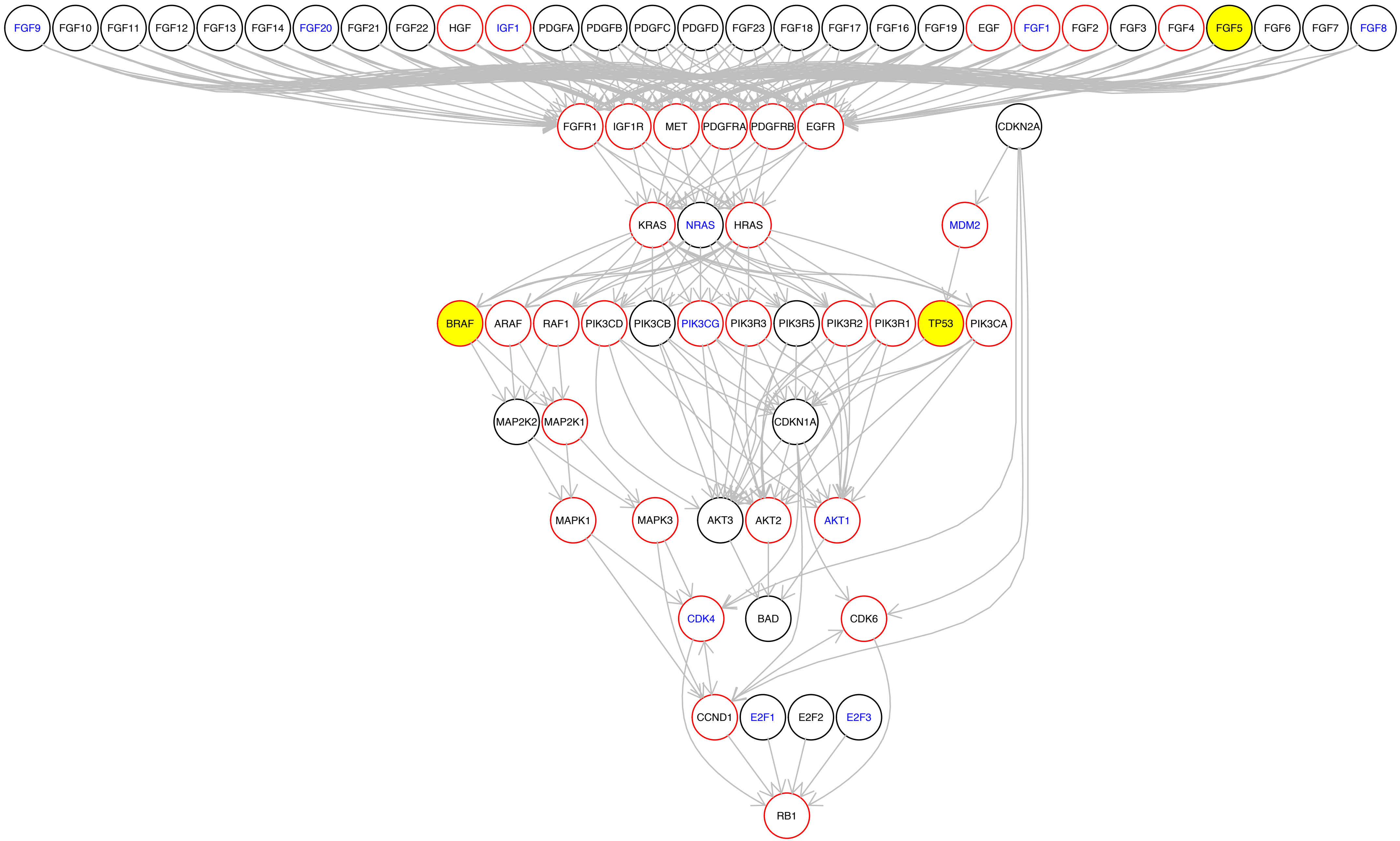

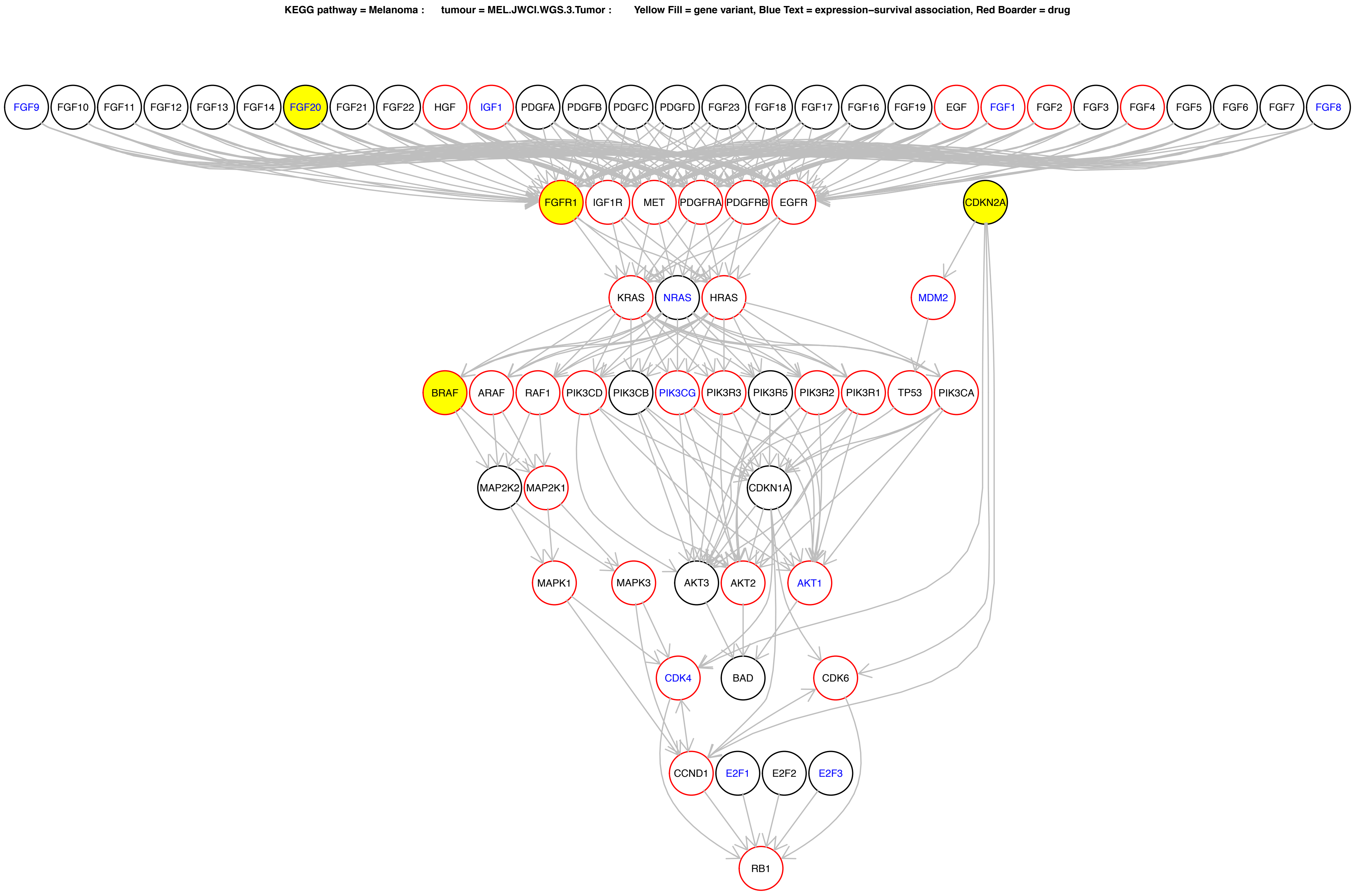
KEGG pathway = Melanoma : tumour = MEL.JWCI.WGS.3.Tumor : Yellow Fill = gene variant, Blue Text = expression−survival association, Red Boarder = drug
FGF9
FGF10
FGF11
FGF12
FGF13
FGF14
FGF20
FGF21
FGF22
HGF
IGF1
PDGFA
PDGFB
PDGFC
PDGFD
FGF23
FGF18
FGF17
FGF16
FGF19
EGF
FGF1
FGF2
FGF3
FGF4
FGF5
FGF6
FGF7
FGF8
FGFR1
IGF1R
MET
PDGFRA
PDGFRB
EGFR
CDKN2A
KRAS
NRAS
HRAS
MDM2
BRAF
ARAF
RAF1
PIK3CD
PIK3CB
PIK3CG
PIK3R3
PIK3R5
PIK3R2
PIK3R1
TP53
PIK3CA
MAP2K2
MAP2K1
CDKN1A
MAPK1
MAPK3
AKT3
AKT2
AKT1
CDK4
BAD
CDK6
CCND1
E2F1
E2F2
E2F3
RB1

KEGG pathway = Melanoma : tumour = MEL.JWCI.WGS.42.Tumor : Yellow Fill = gene variant, Blue Text = expression−survival association, Red Boarder = drug

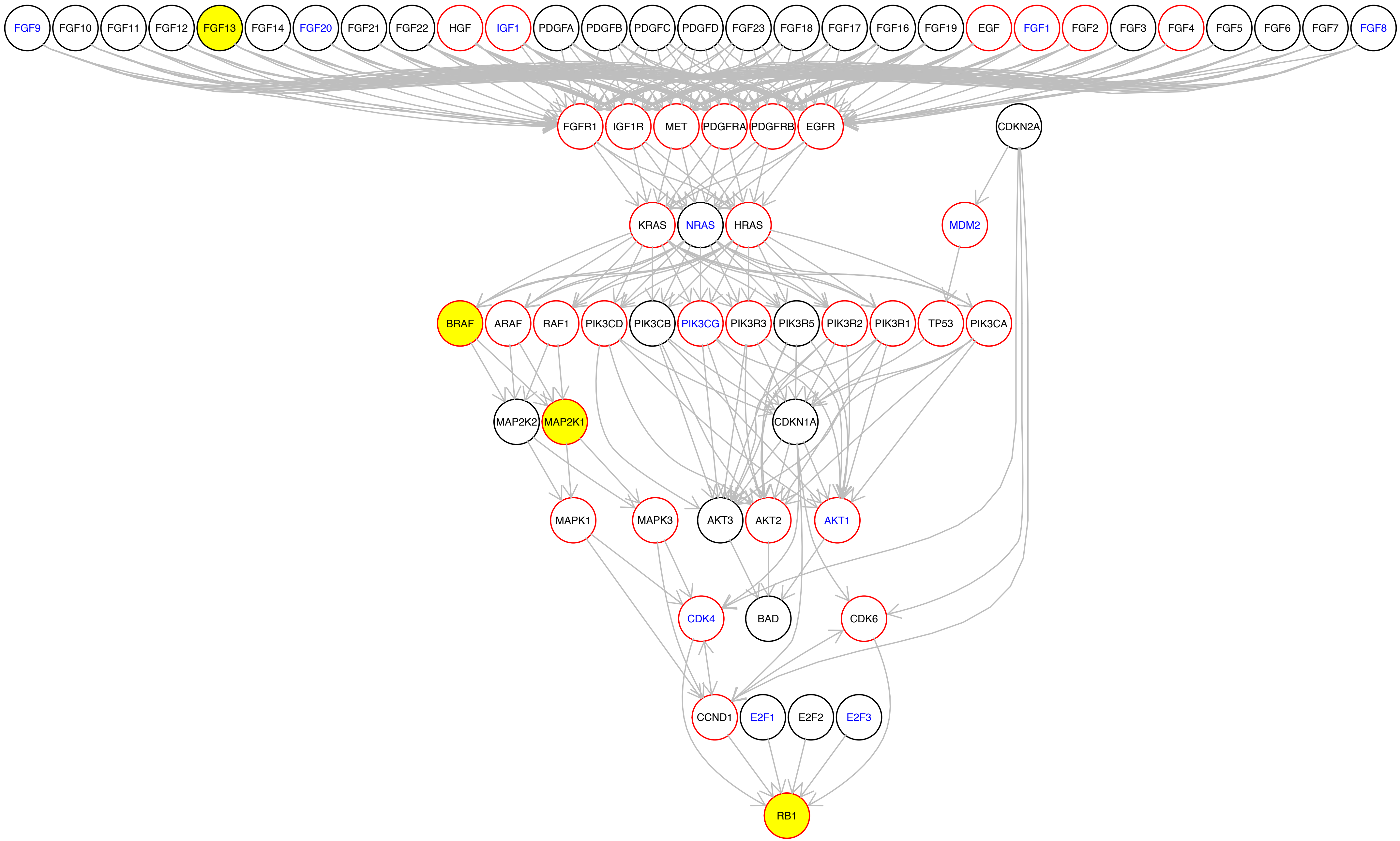

KEGG pathway = Melanoma : tumour = MEL.JWCI.WGS.43.Tumor : Yellow Fill = gene variant, Blue Text = expression−survival association, Red Boarder = drug

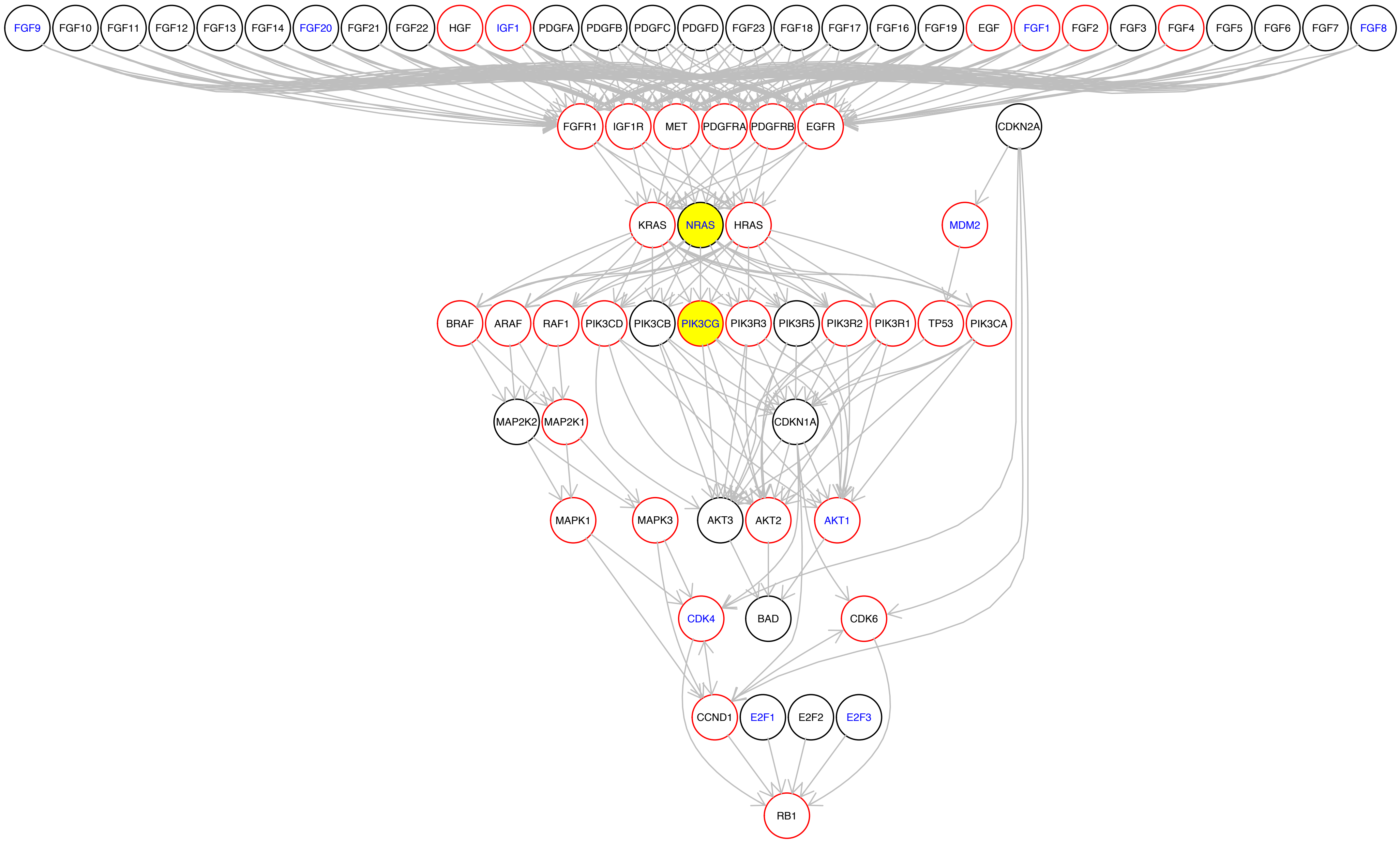

KEGG pathway = Melanoma : tumour = MEL.JWCI.WGS.4.Tumor : Yellow Fill = gene variant, Blue Text = expression–survival association, Red Boarder = drug

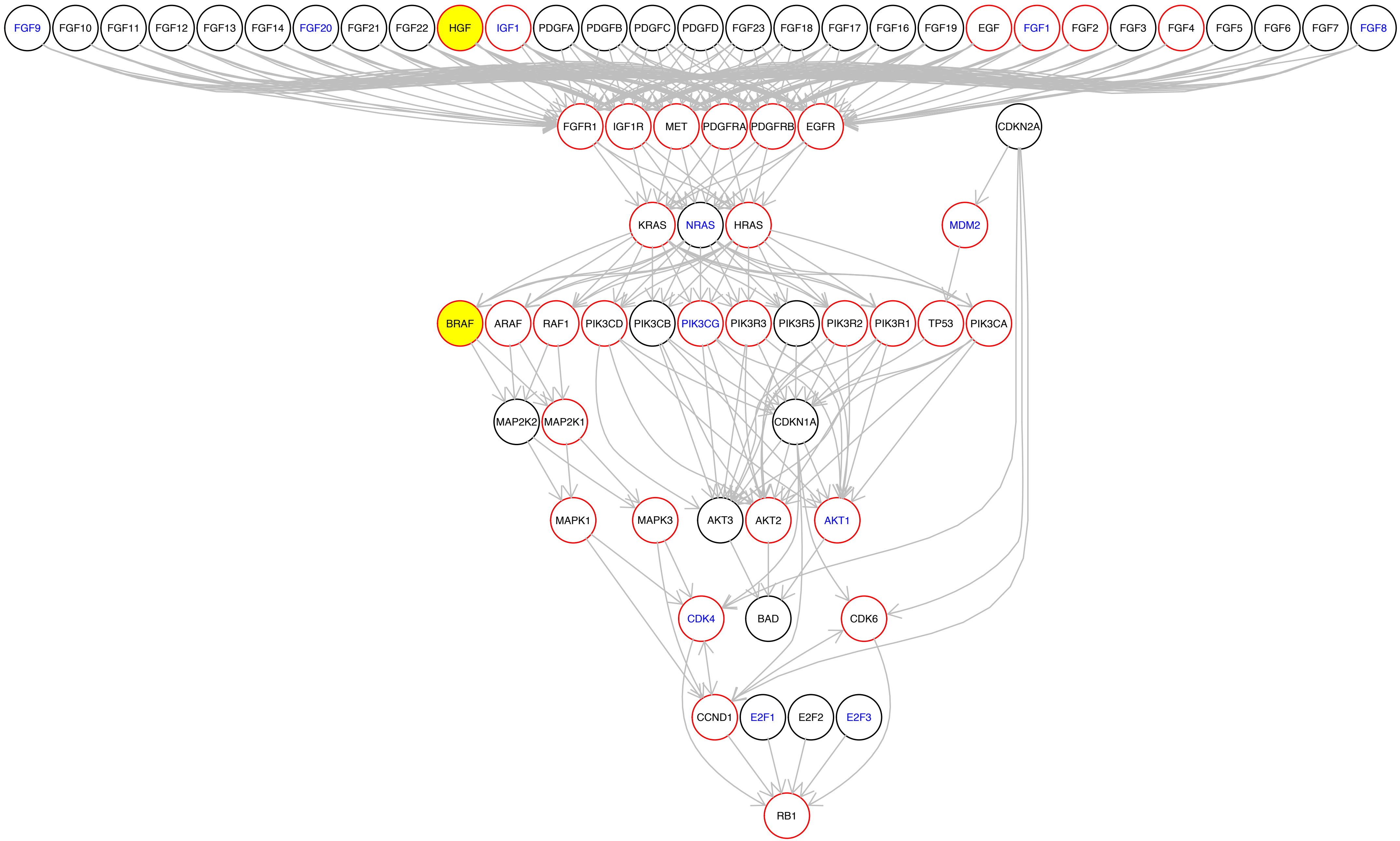

KEGG pathway = Melanoma : tumour = MEL.JWCI.WGS.5.Tumor : Yellow Fill = gene variant, Blue Text = expression–survival association, Red Boarder = drug

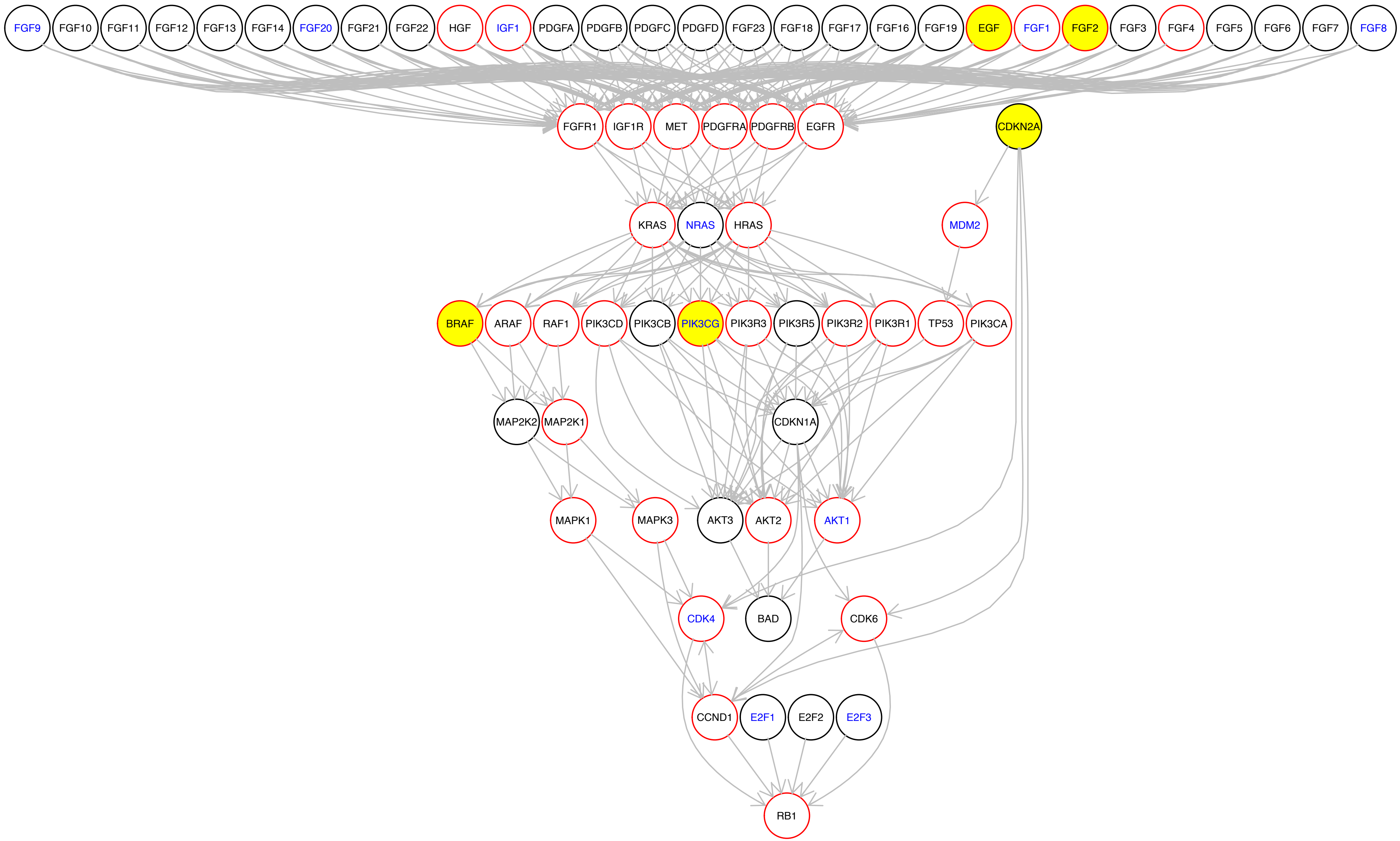

KEGG pathway = Melanoma : tumour = MEL.JWCI.WGS.6.Tumor : Yellow Fill = gene variant, Blue Text = expression−survival association, Red Boarder = drug

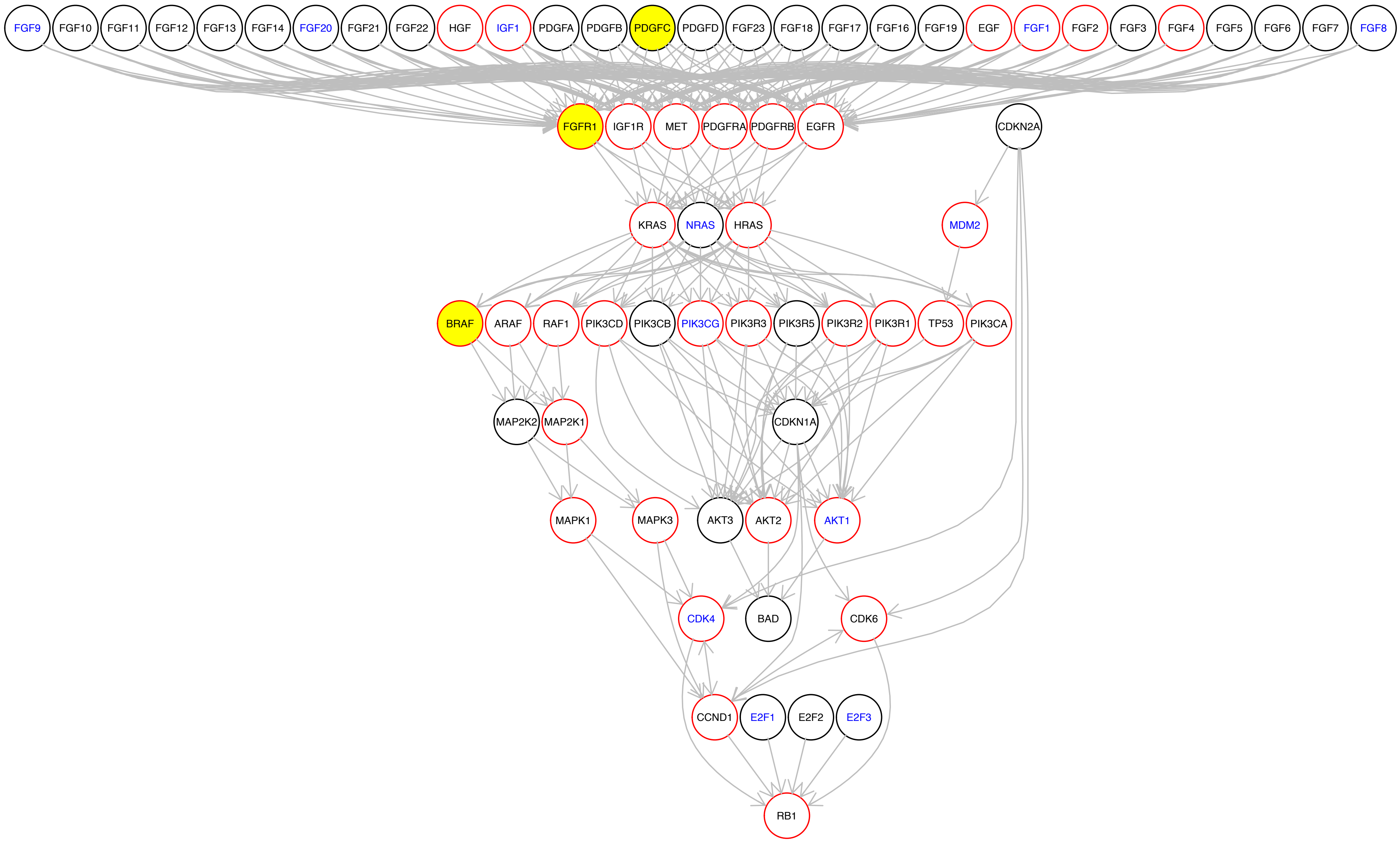

KEGG pathway = Melanoma :   tumour = MEL.JWCI.WGS.7.Tumor :   Yellow Fill = gene variant, Blue Text = expression−survival association, Red Boarder = drug

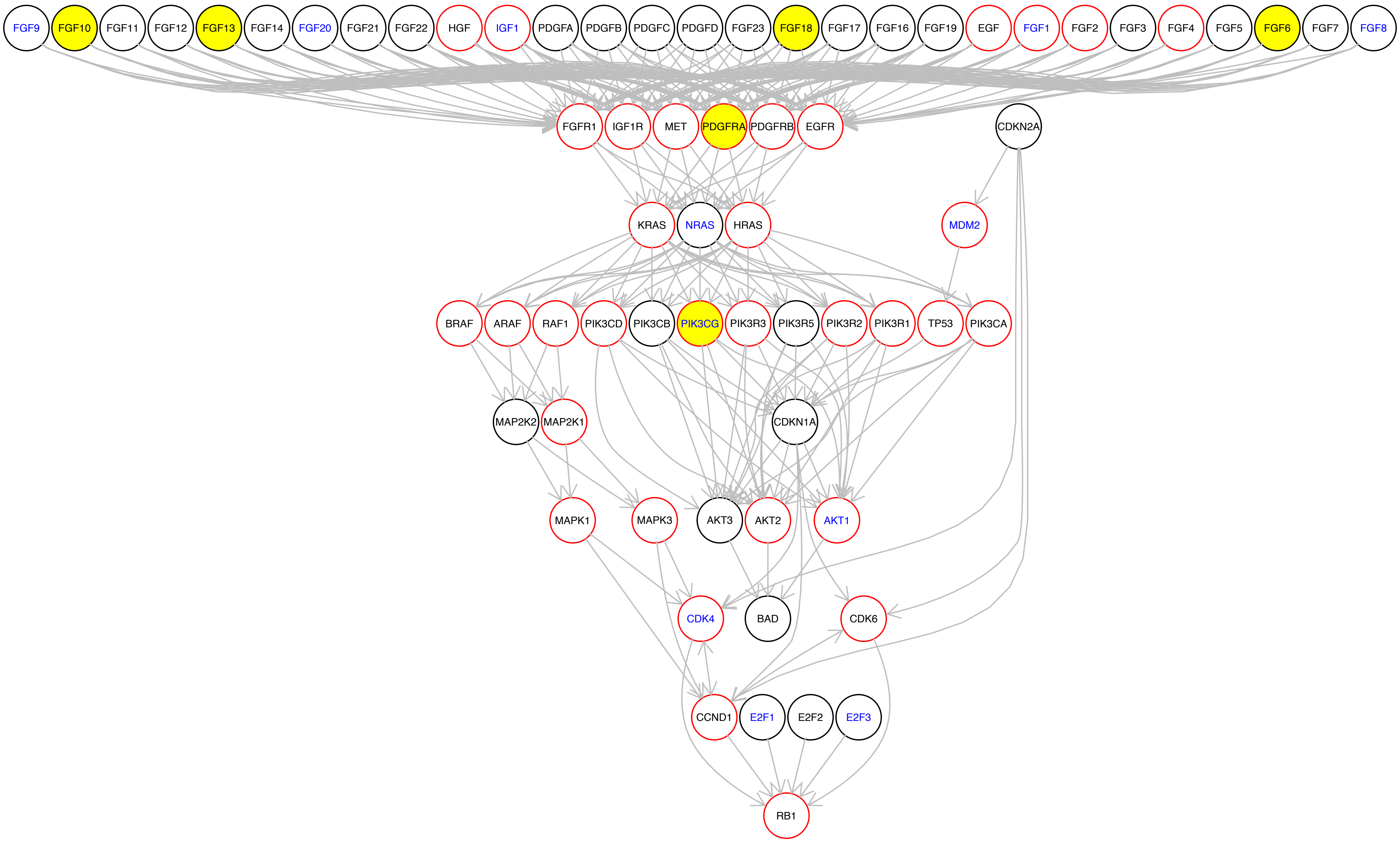

KEGG pathway = Melanoma : tumour = MEL.JWCI.WGS.8.Tumor : Yellow Fill = gene variant, Blue Text = expression−survival association, Red Boarder = drug

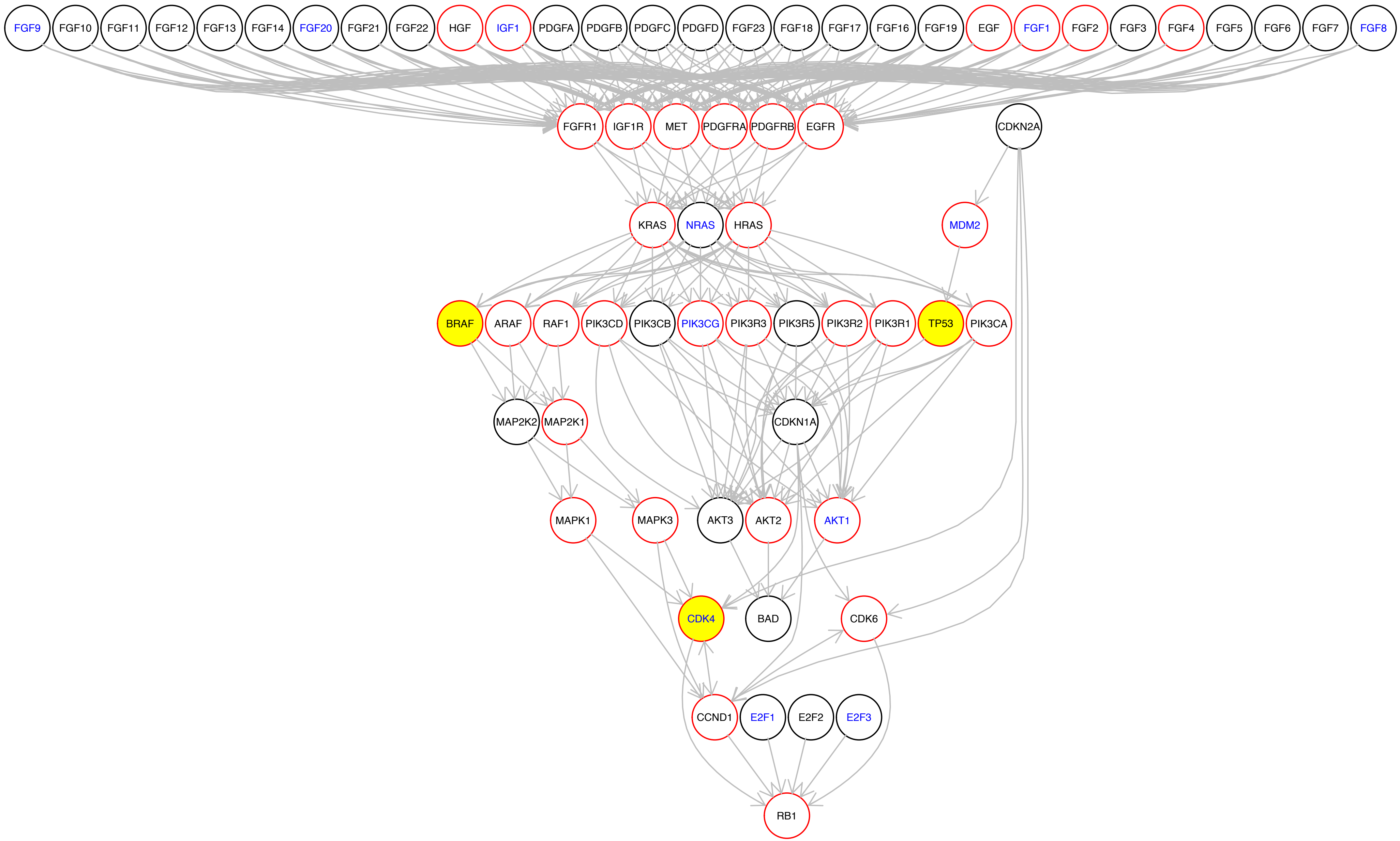

KEGG pathway = Melanoma : tumour = MEL.Ma.Mel.04.Tumor : Yellow Fill = gene variant, Blue Text = expression−survival association, Red Boarder = drug

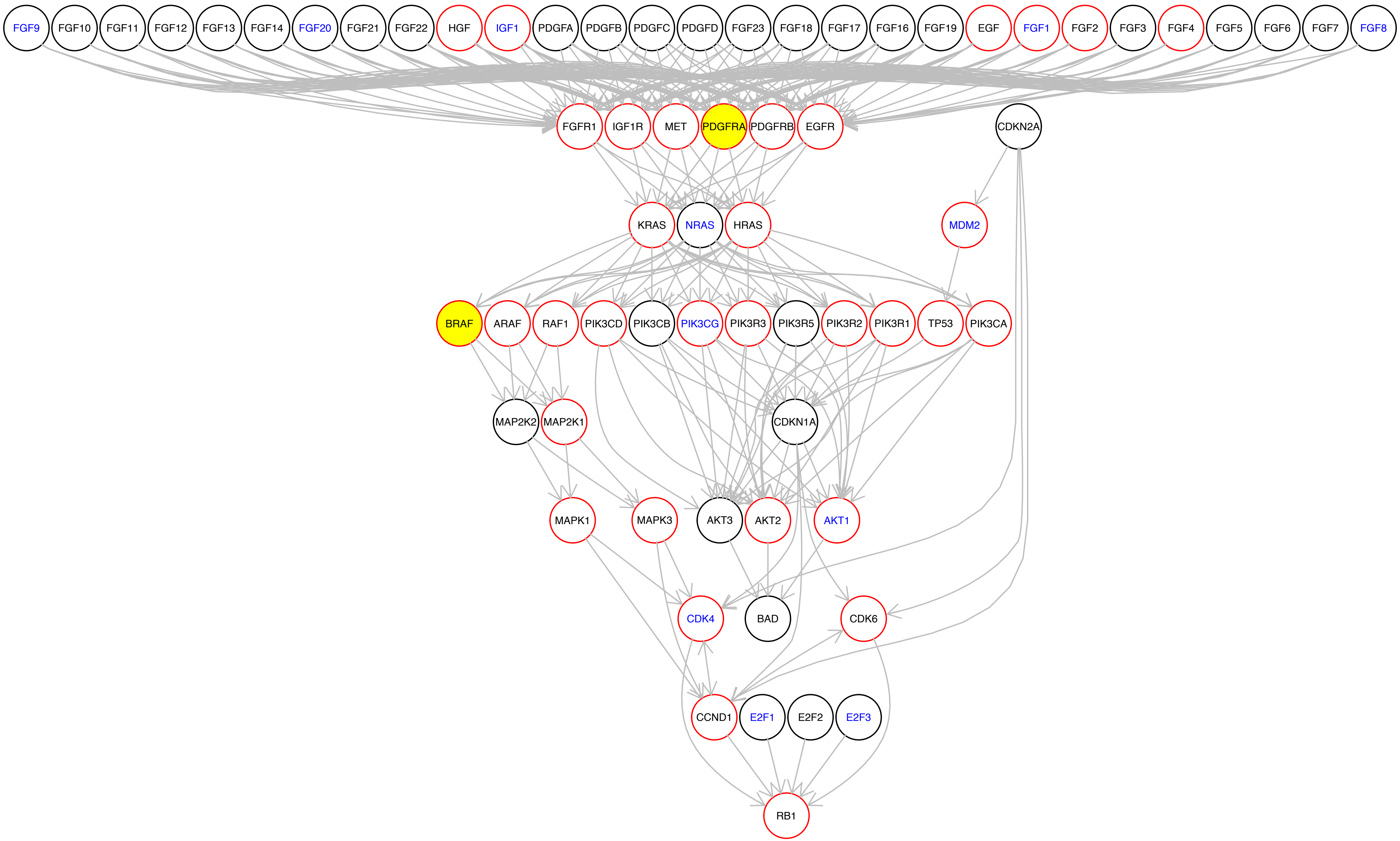

KEGG pathway = Melanoma : tumour = MEL.Ma.Mel.05.Tumor : Yellow Fill = gene variant, Blue Text = expression−survival association, Red Boarder = drug

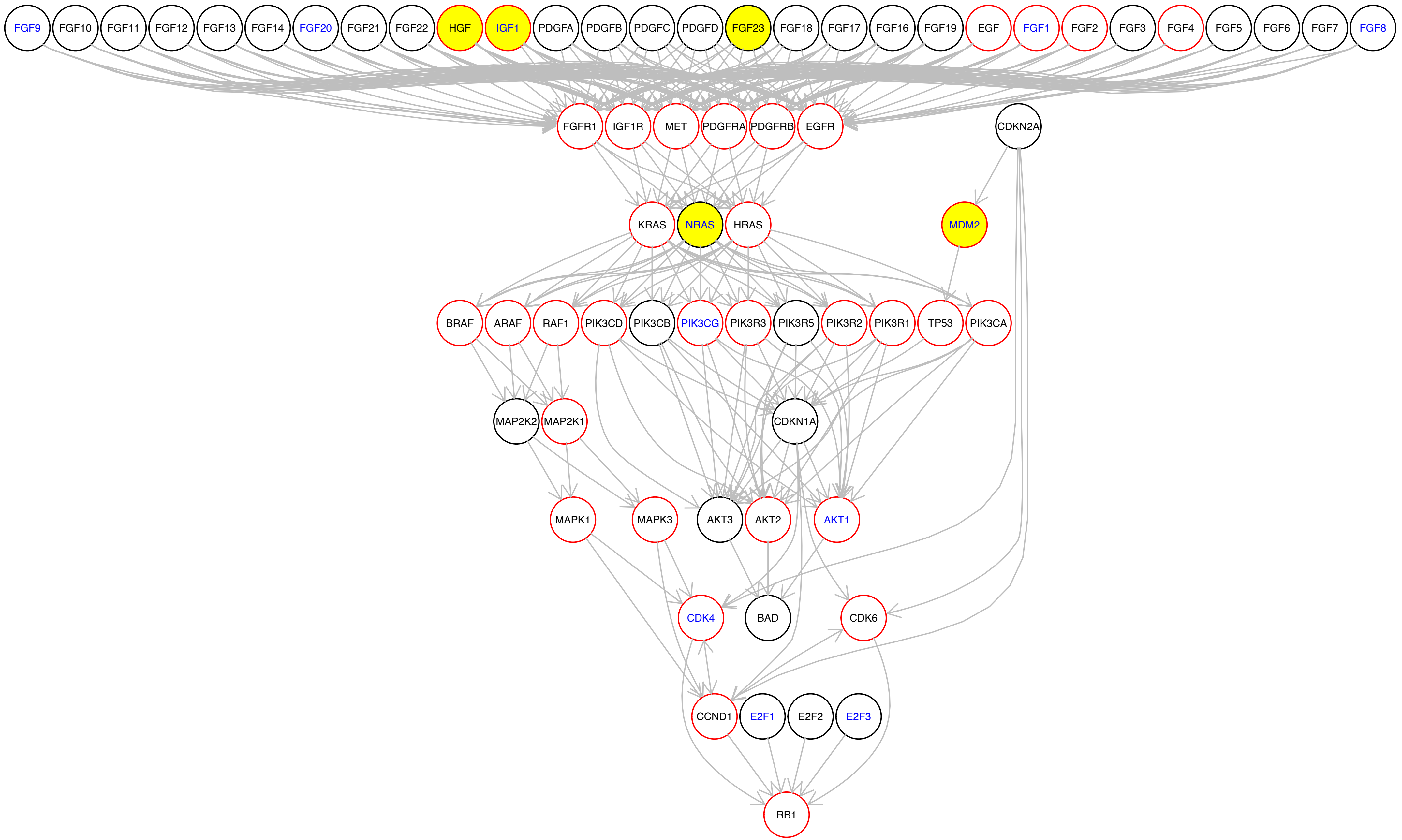

KEGG pathway = Melanoma : tumour = MEL.Ma.Mel.08a.Tumor : Yellow Fill = gene variant, Blue Text = expression−survival association, Red Boarder = drug

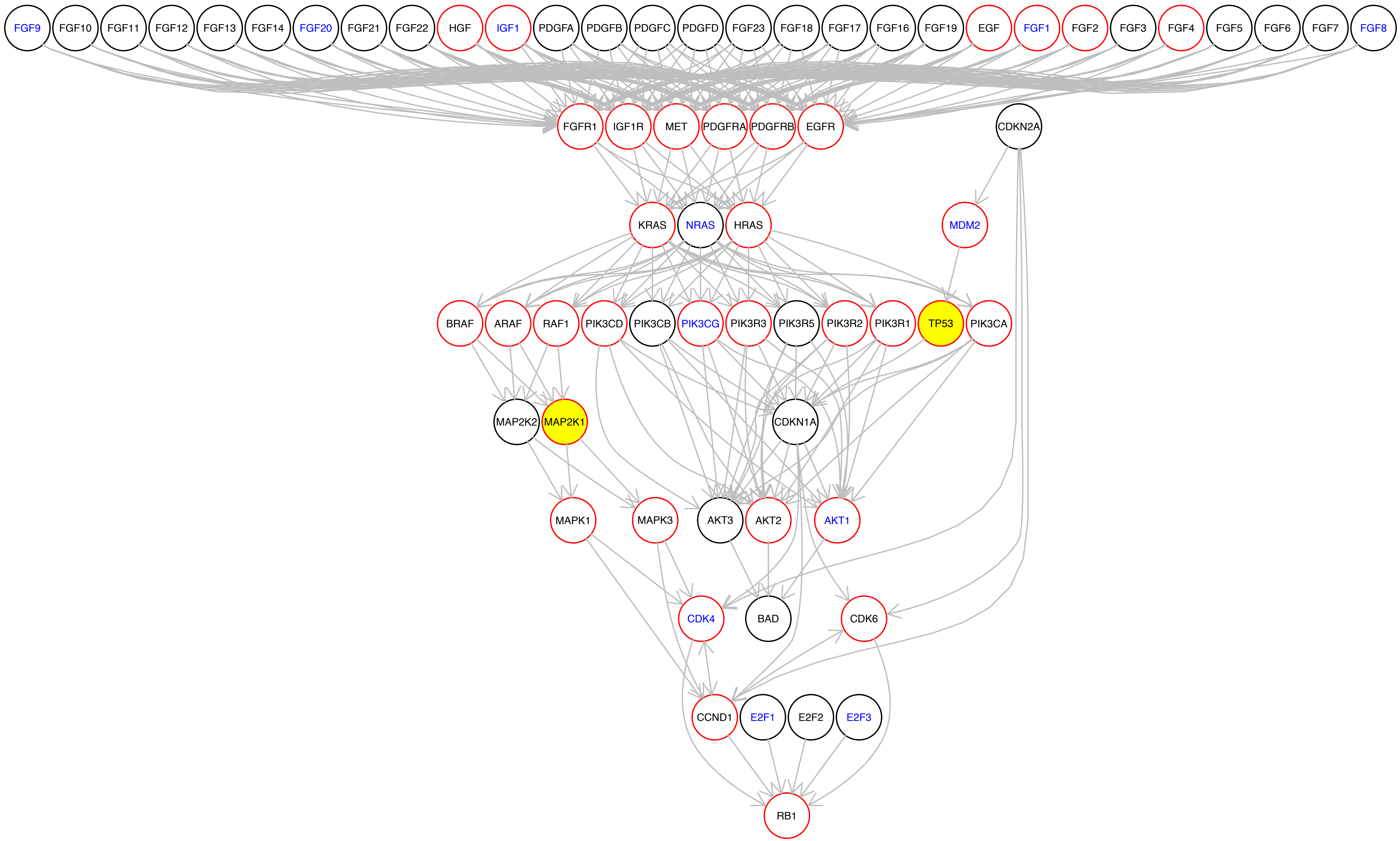

KEGG pathway = Melanoma : tumour = MEL.Ma.Mel.102.Tumor : Yellow Fill = gene variant, Blue Text = expression−survival association, Red Boarder = drug

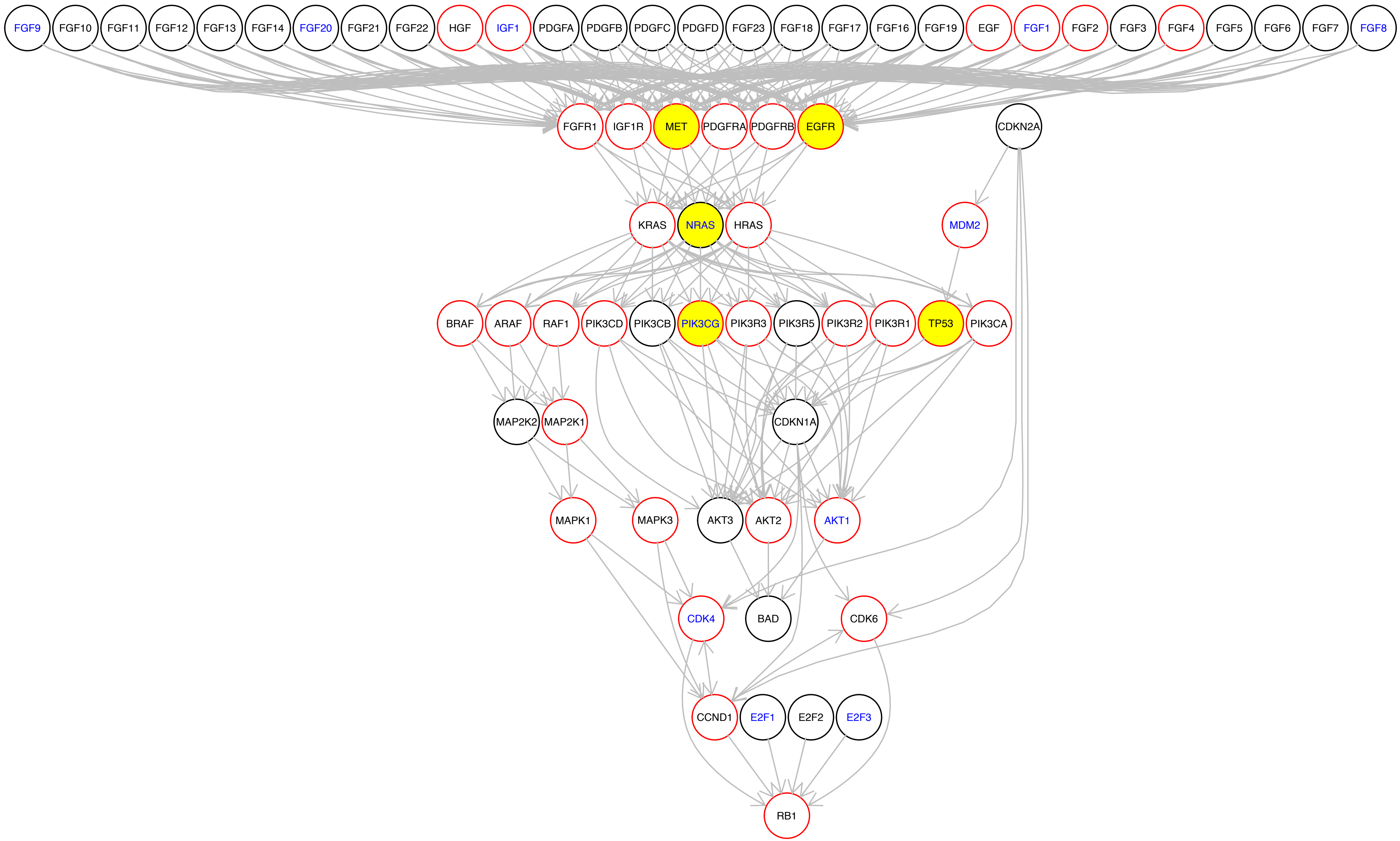

KEGG pathway = Melanoma : tumour = MEL.Ma.Mel.103b.Tumor : Yellow Fill = gene variant, Blue Text = expression−survival association, Red Boarder = drug

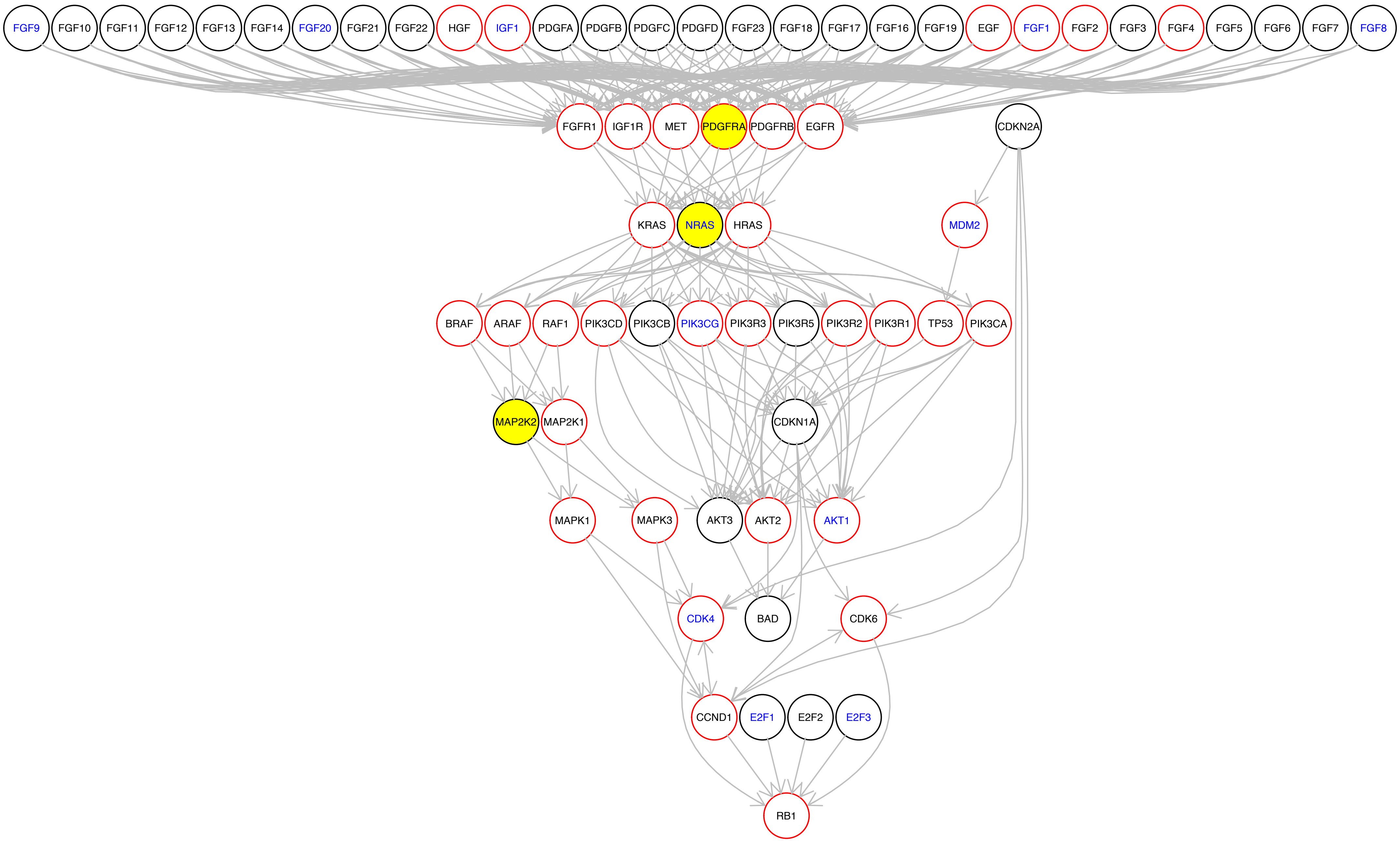

KEGG pathway = Melanoma : tumour = MEL.Ma.Mel.105.Tumor : Yellow Fill = gene variant, Blue Text = expression−survival association, Red Boarder = drug

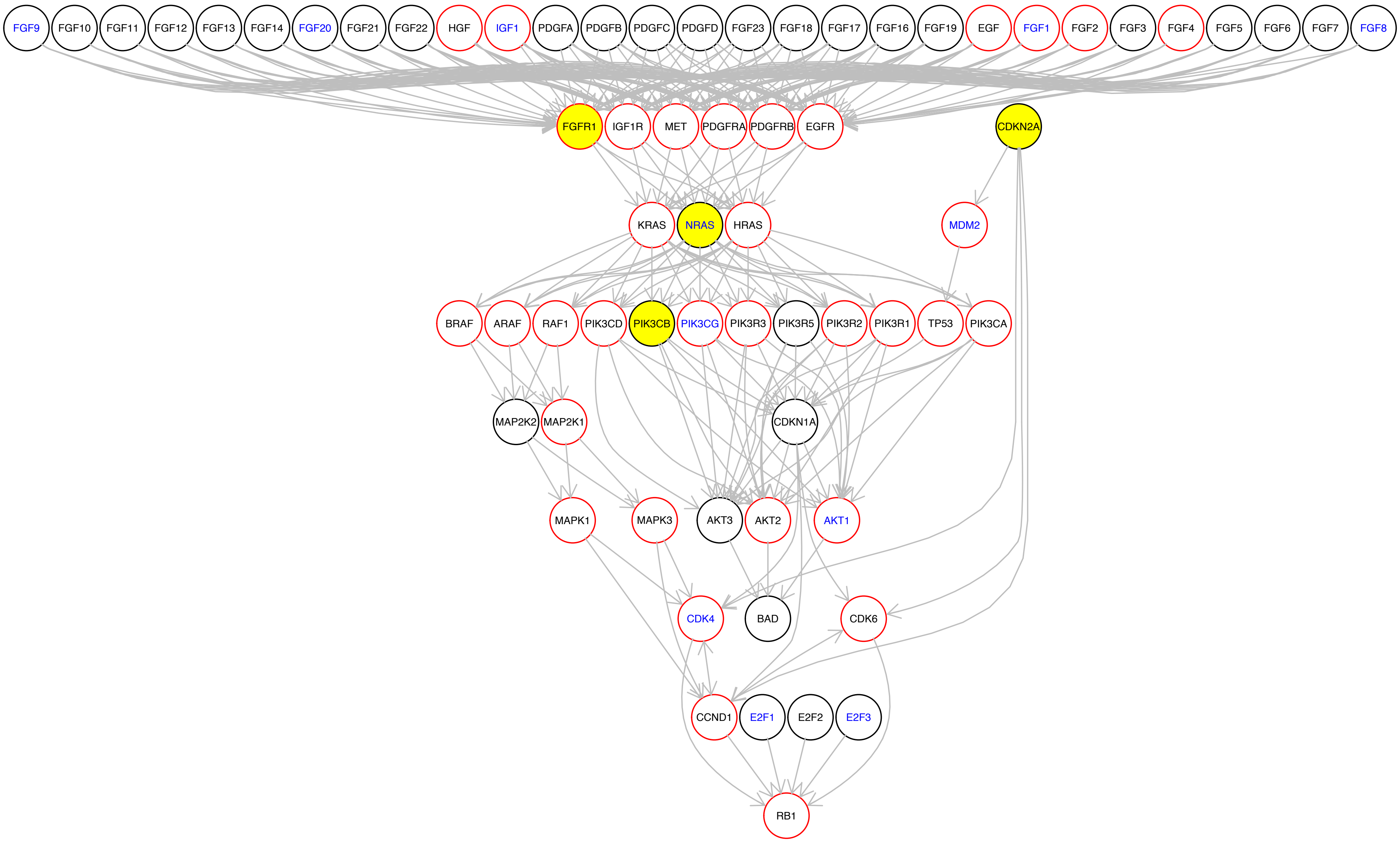

KEGG pathway = Melanoma :   tumour = MEL.Ma.Mel.107.Tumor :   Yellow Fill = gene variant, Blue Text = expression−survival association, Red Boarder = drug

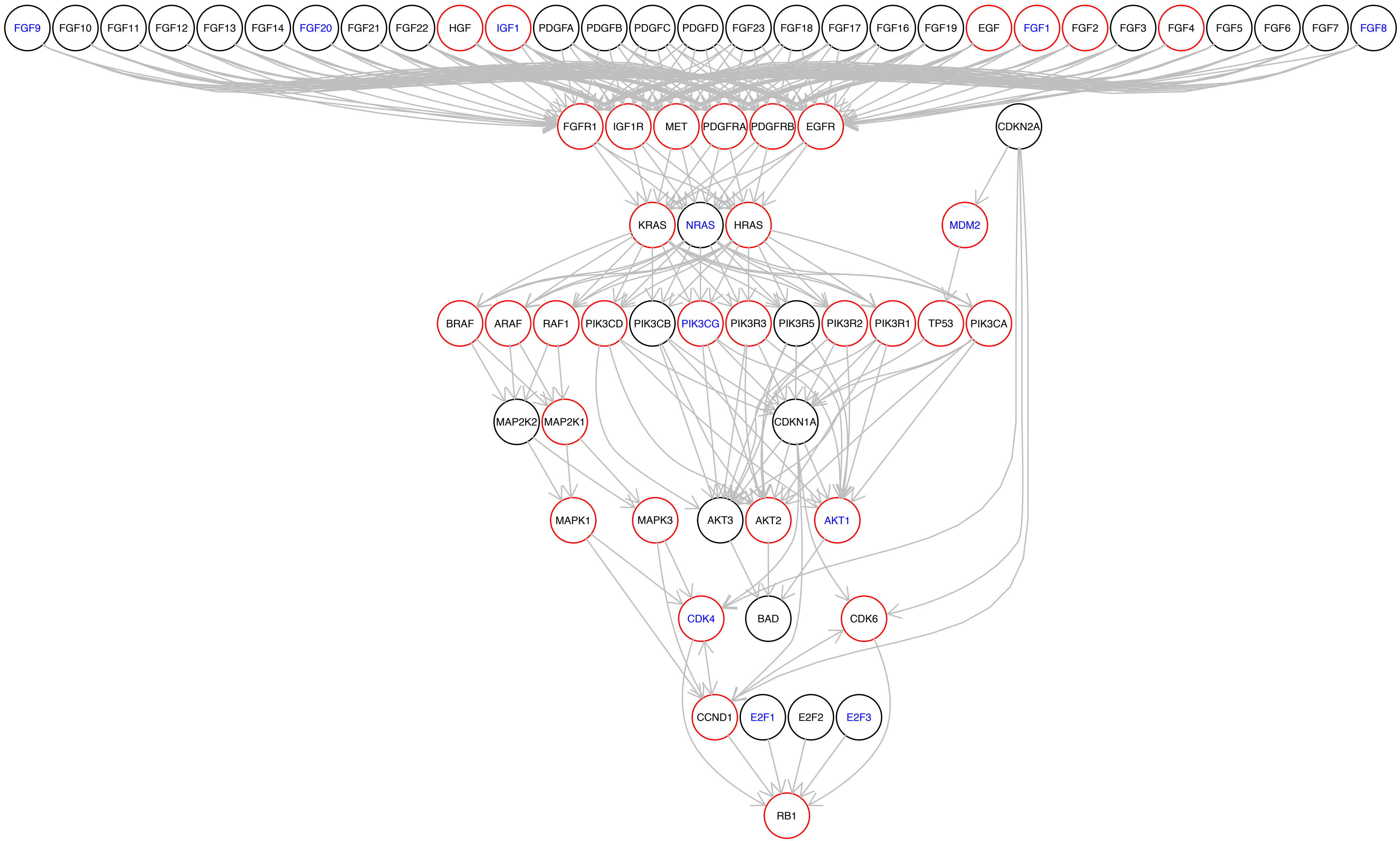

KEGG pathway = Melanoma : tumour = MEL.Ma.Mel.108.Tumor : Yellow Fill = gene variant, Blue Text = expression−survival association, Red Boarder = drug

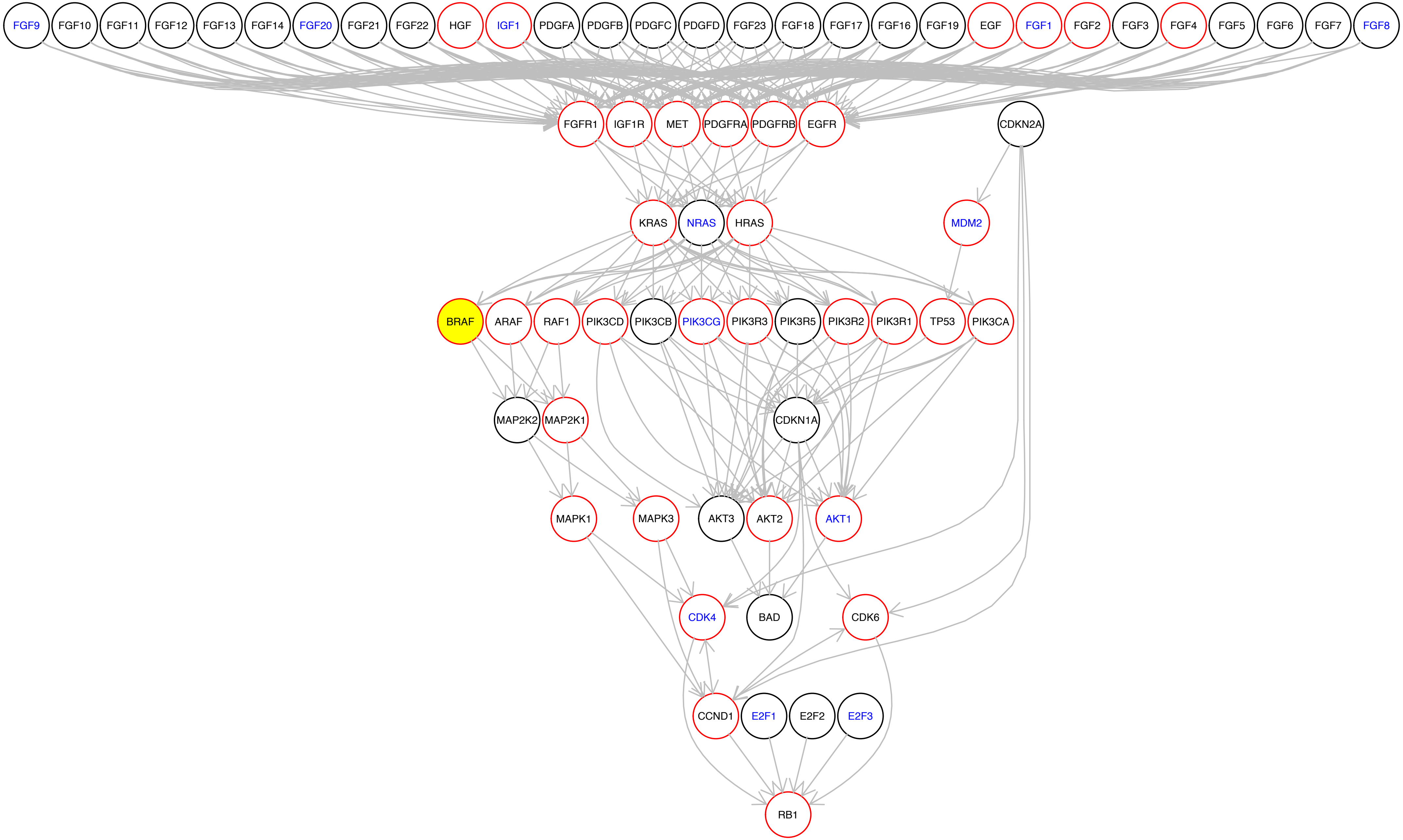

KEGG pathway = Melanoma :   tumour = MEL.Ma.Mel.114.Tumor :   Yellow Fill = gene variant, Blue Text = expression−survival association, Red Boarder = drug

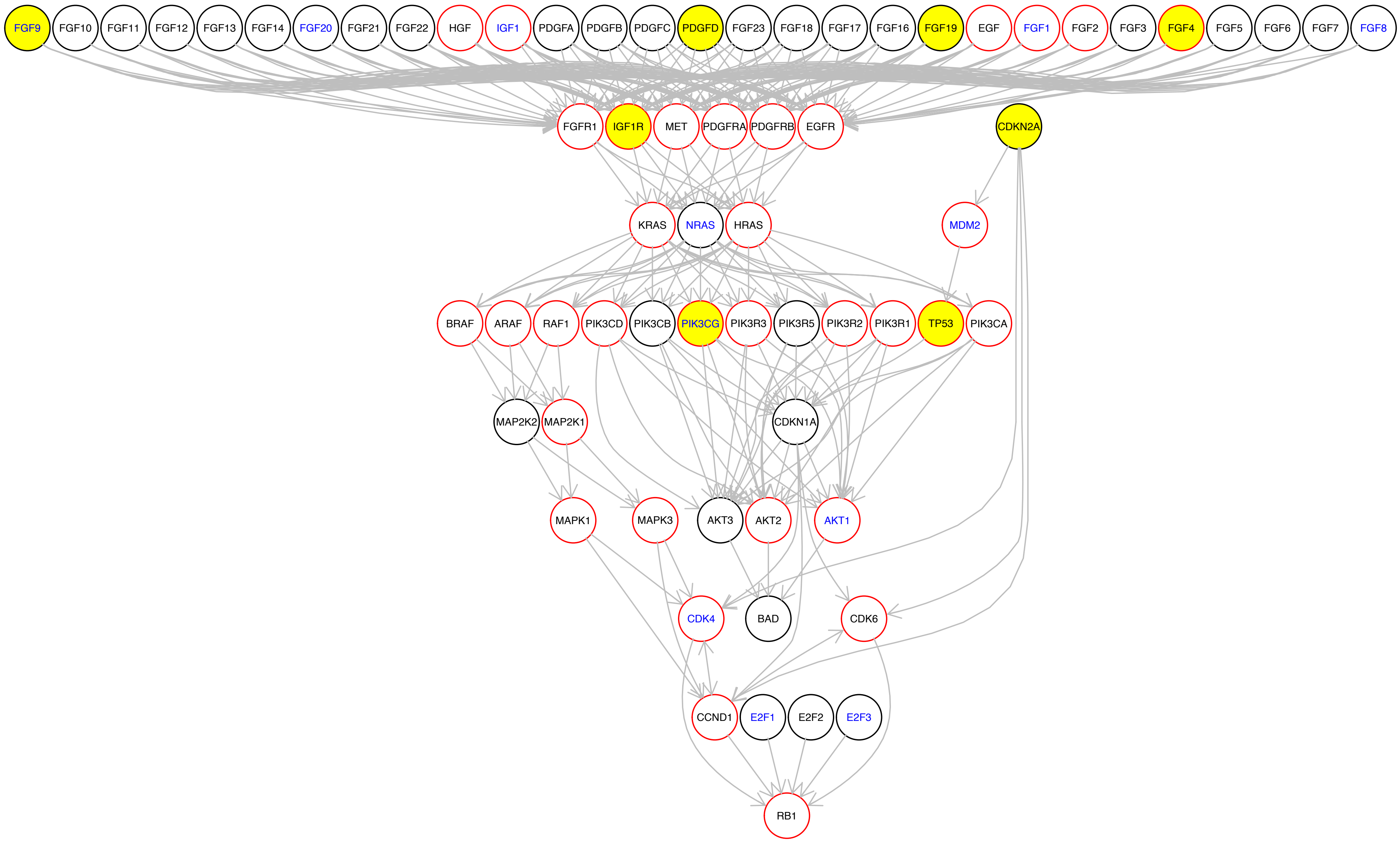

KEGG pathway = Melanoma : tumour = MEL.Ma.Mel.119.Tumor : Yellow Fill = gene variant, Blue Text = expression−survival association, Red Boarder = drug

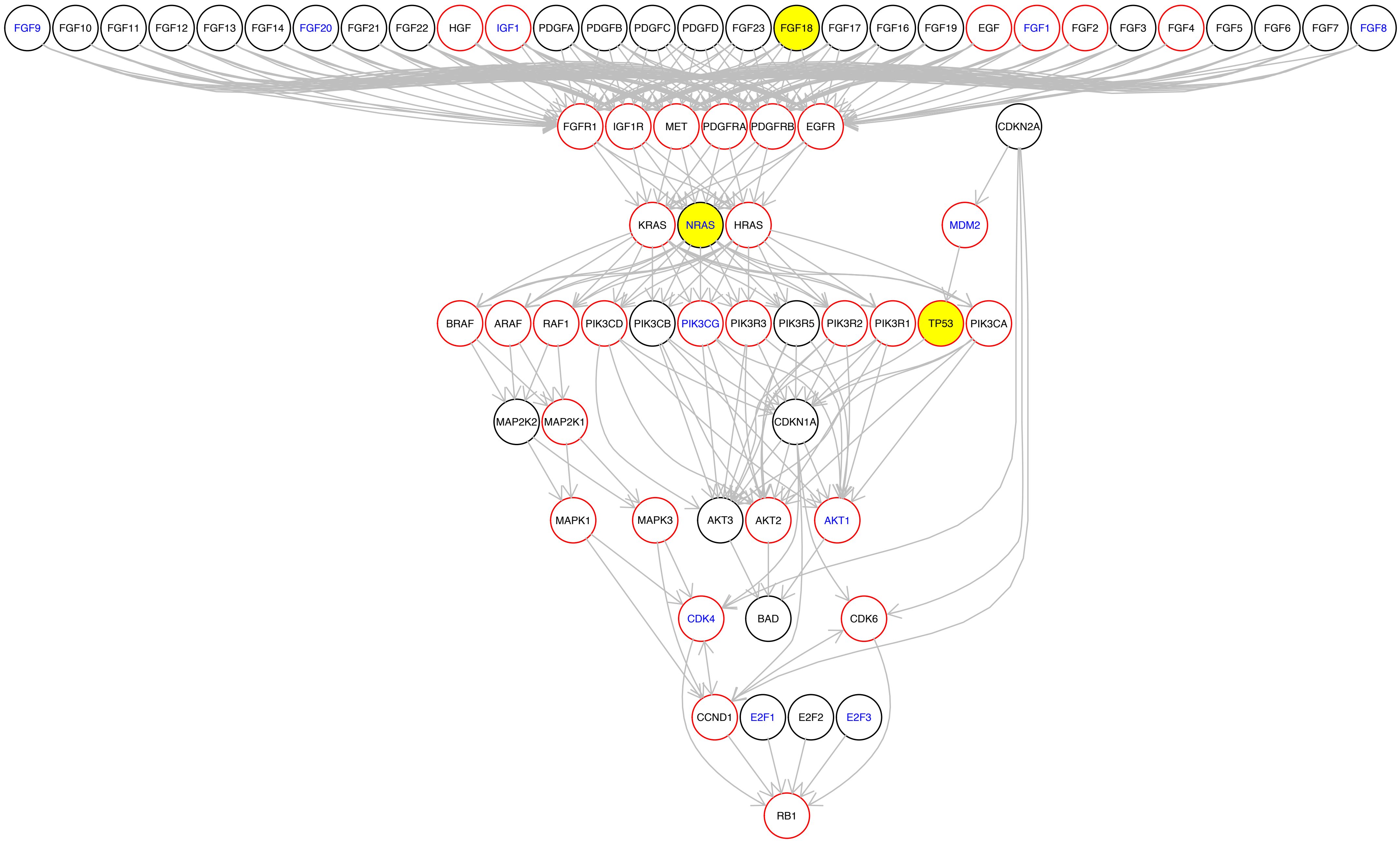

KEGG pathway = Melanoma : tumour = MEL.Ma.Mel.120.Tumor : Yellow Fill = gene variant, Blue Text = expression−survival association, Red Boarder = drug

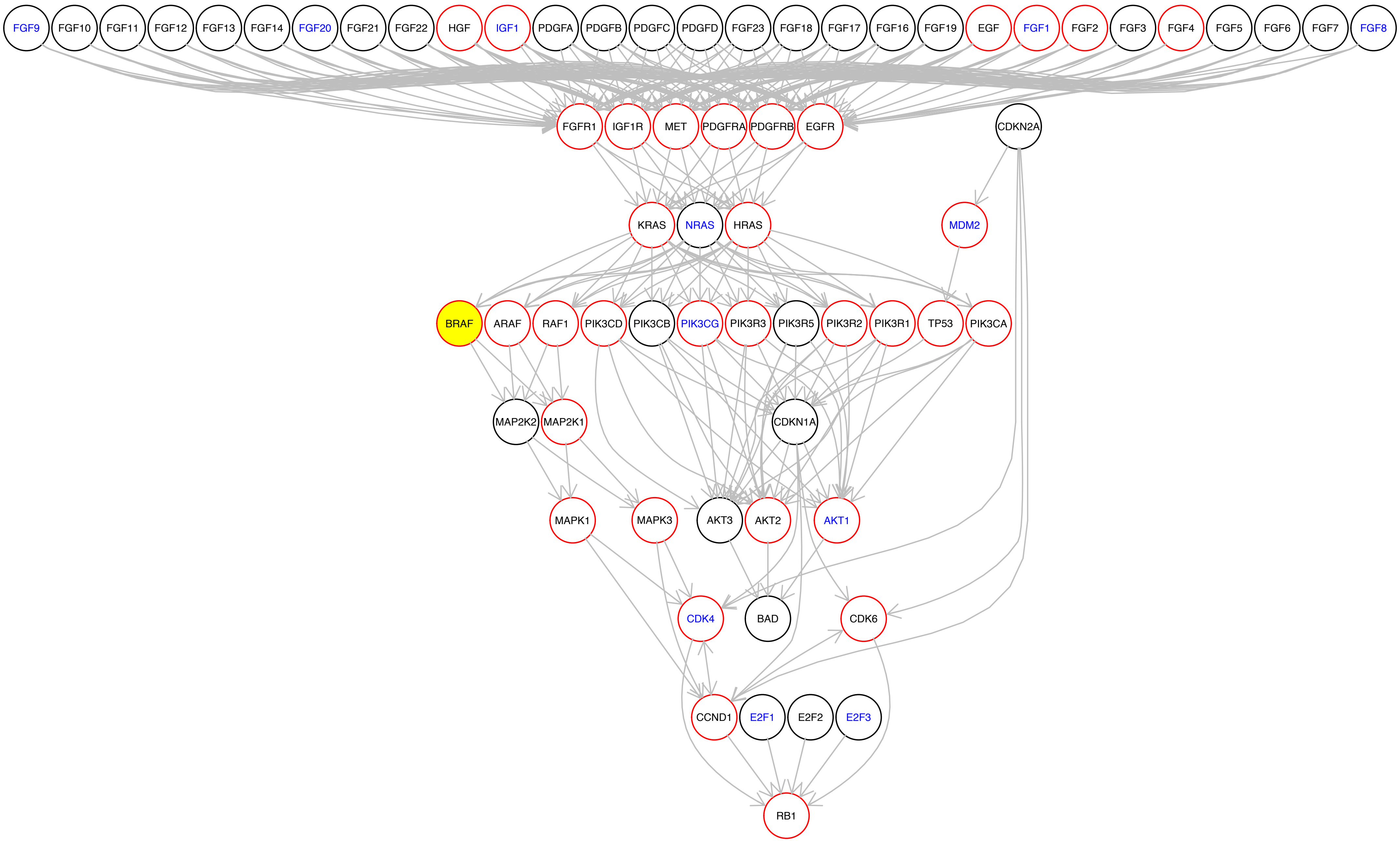

KEGG pathway = Melanoma :   tumour = MEL.Ma.Mel.122.Tumor :   Yellow Fill = gene variant, Blue Text = expression−survival association, Red Boarder = drug

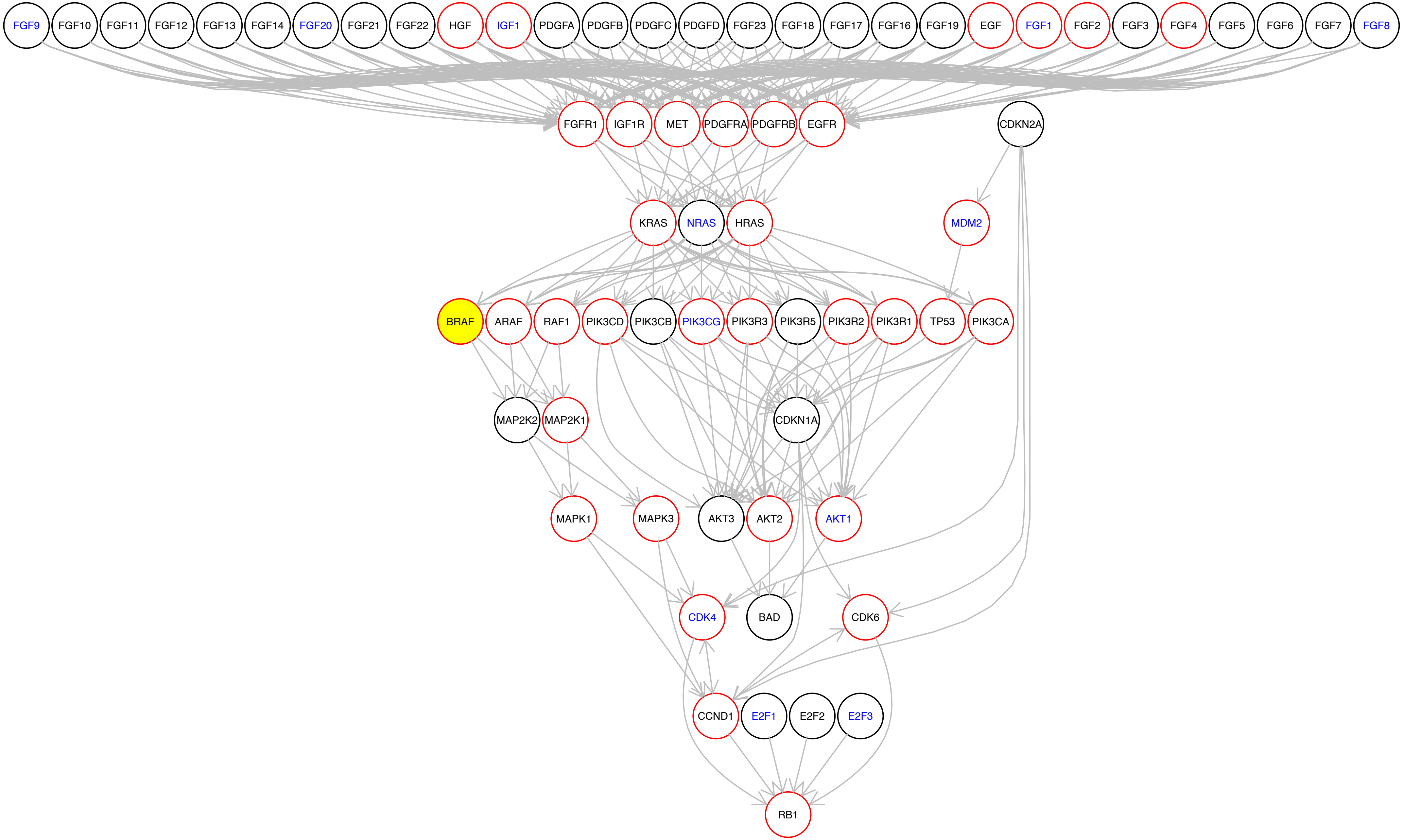

KEGG pathway = Melanoma :   tumour = MEL.Ma.Mel.123.Tumor :   Yellow Fill = gene variant, Blue Text = expression−survival association, Red Boarder = drug

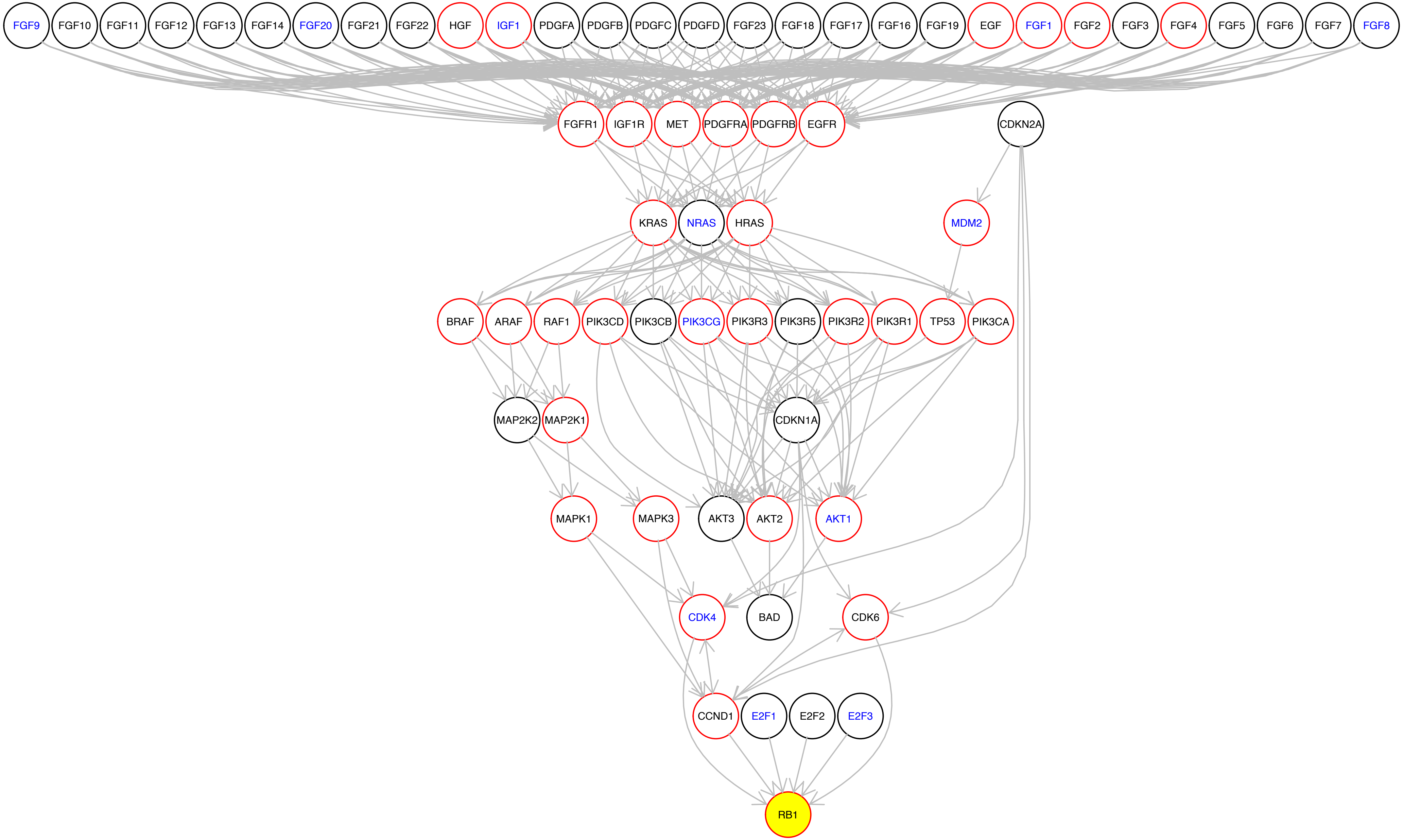

KEGG pathway = Melanoma : tumour = MEL.Ma.Mel.15.Tumor : Yellow Fill = gene variant, Blue Text = expression–survival association, Red Boarder = drug

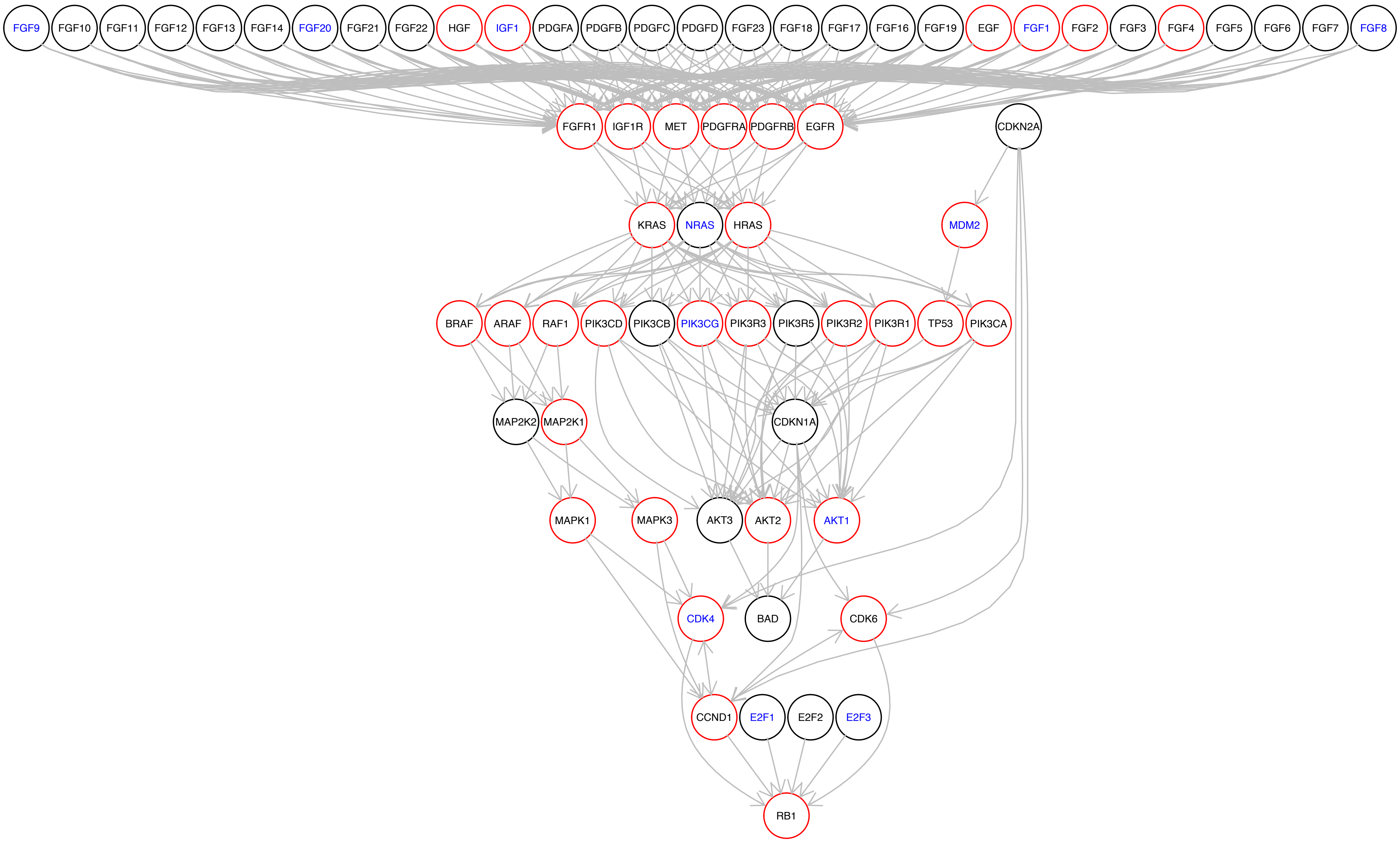

KEGG pathway = Melanoma : tumour = MEL.Ma.Mel.16.Tumor : Yellow Fill = gene variant, Blue Text = expression−survival association, Red Boarder = drug

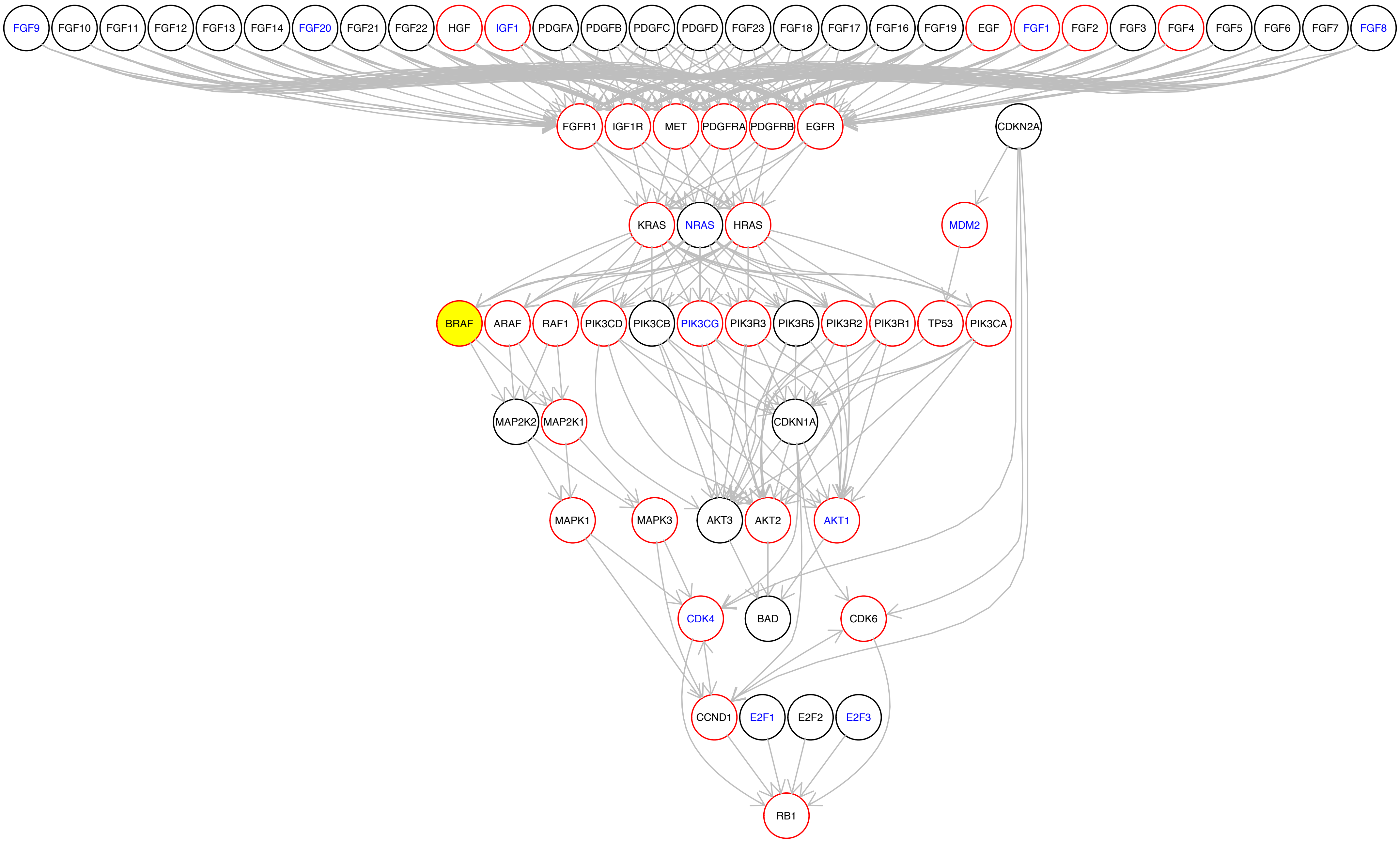

KEGG pathway = Melanoma :   tumour = MEL.Ma.Mel.19.Tumor :   Yellow Fill = gene variant, Blue Text = expression−survival association, Red Boarder = drug

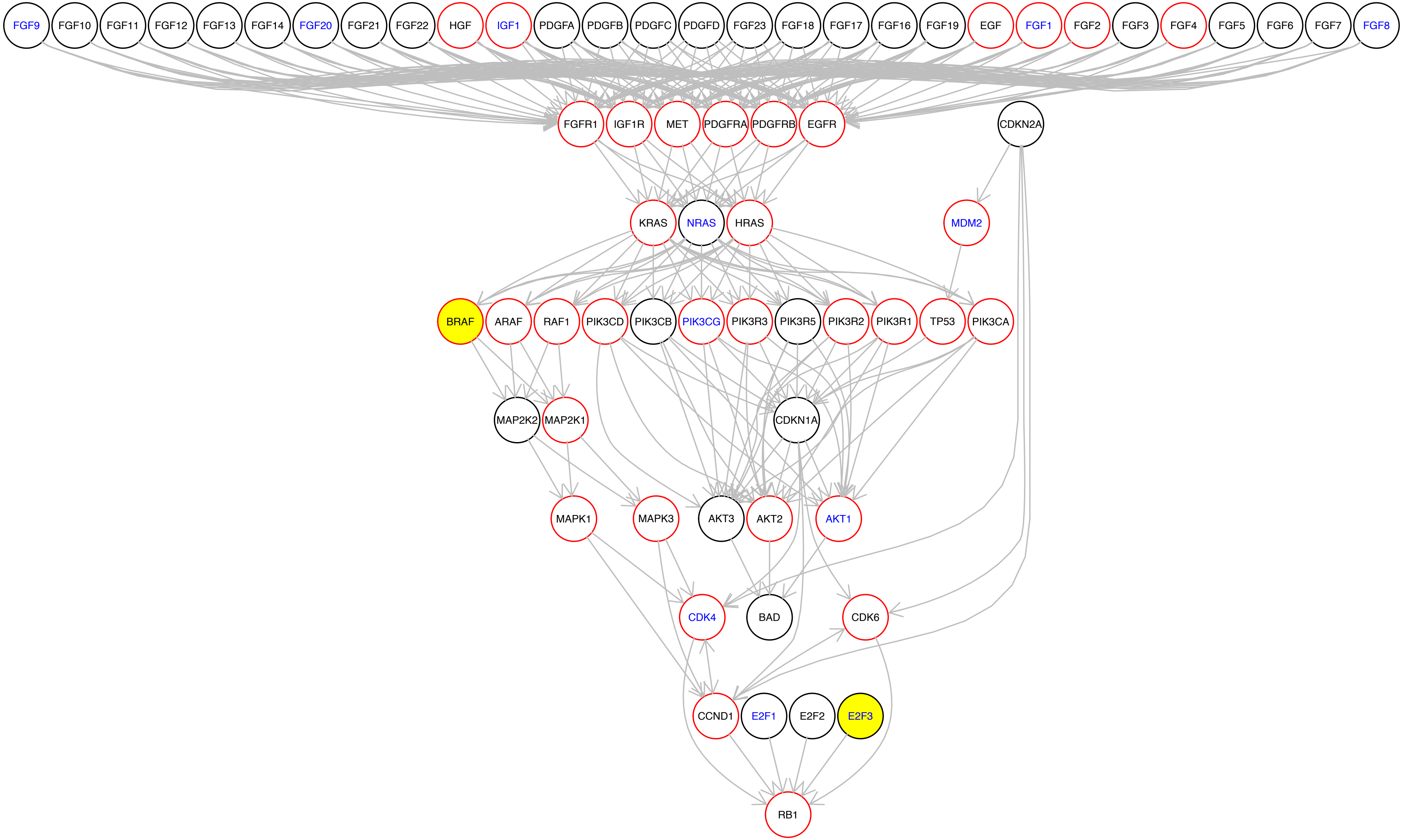

KEGG pathway = Melanoma : tumour = MEL.Ma.Mel.27.Tumor : Yellow Fill = gene variant, Blue Text = expression−survival association, Red Boarder = drug

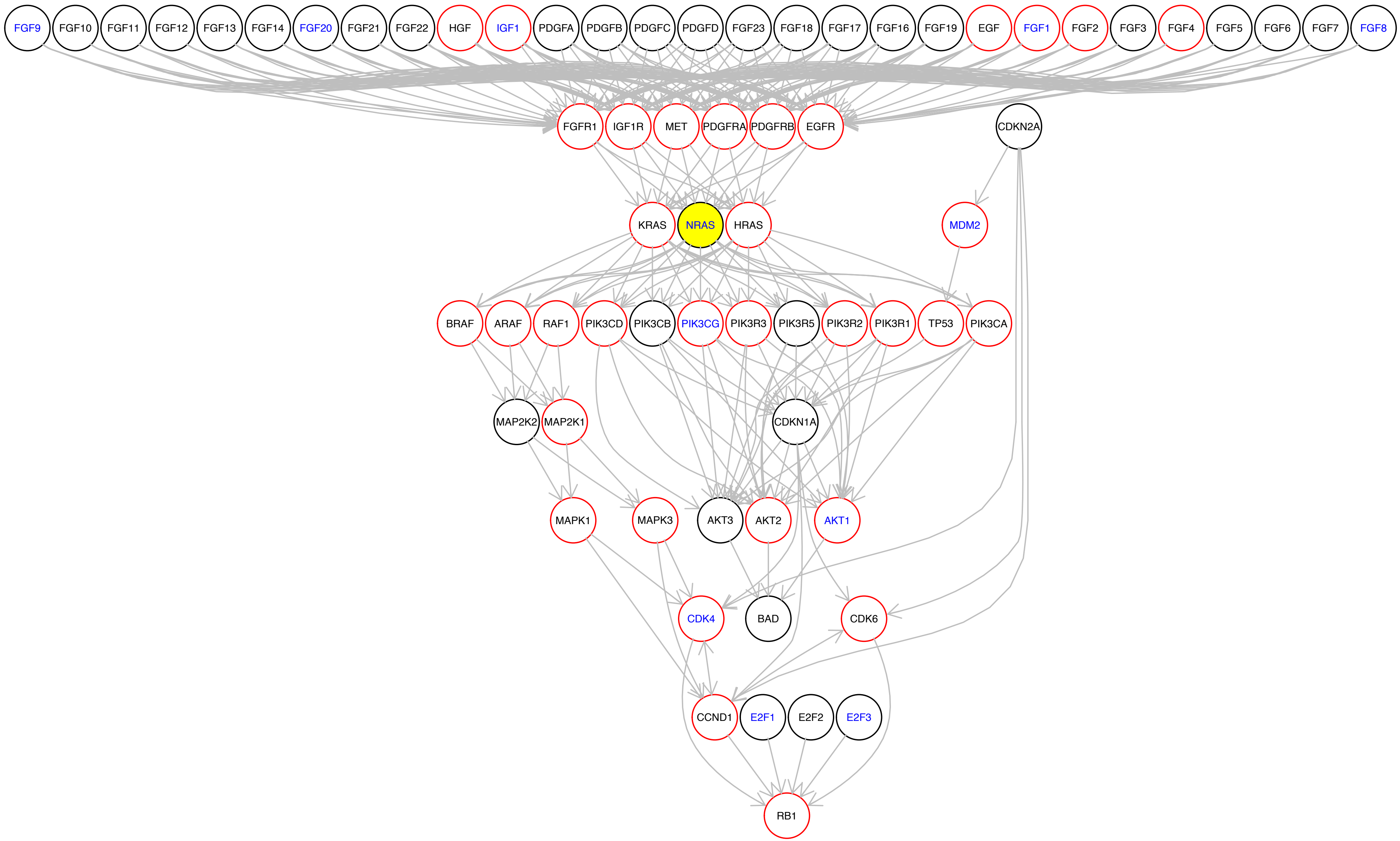

KEGG pathway = Melanoma :   tumour = MEL.Ma.Mel.28.Tumor :   Yellow Fill = gene variant, Blue Text = expression–survival association, Red Boarder = drug

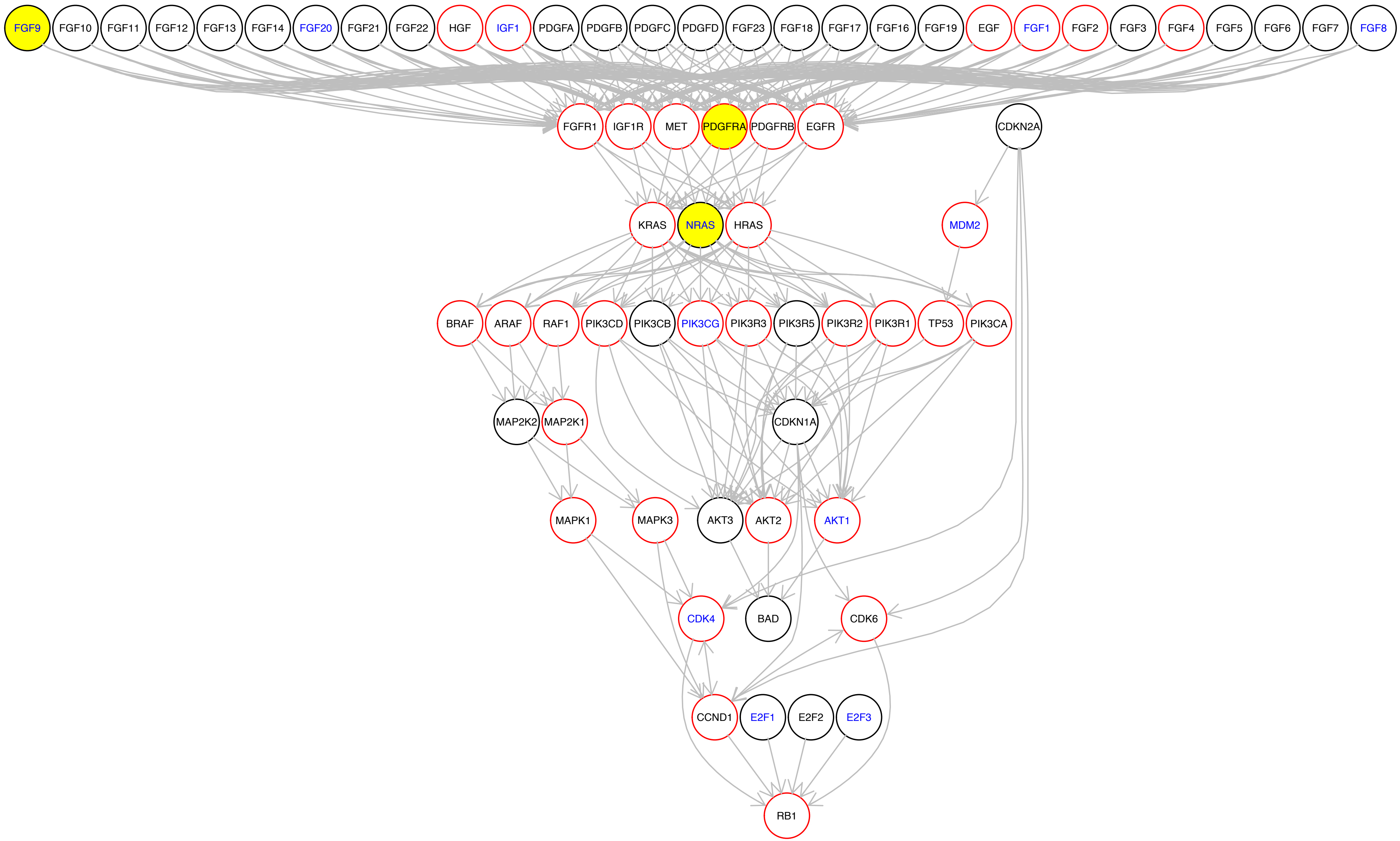

KEGG pathway = Melanoma : tumour = MEL.Ma.Mel.35.Tumor : Yellow Fill = gene variant, Blue Text = expression−survival association, Red Boarder = drug

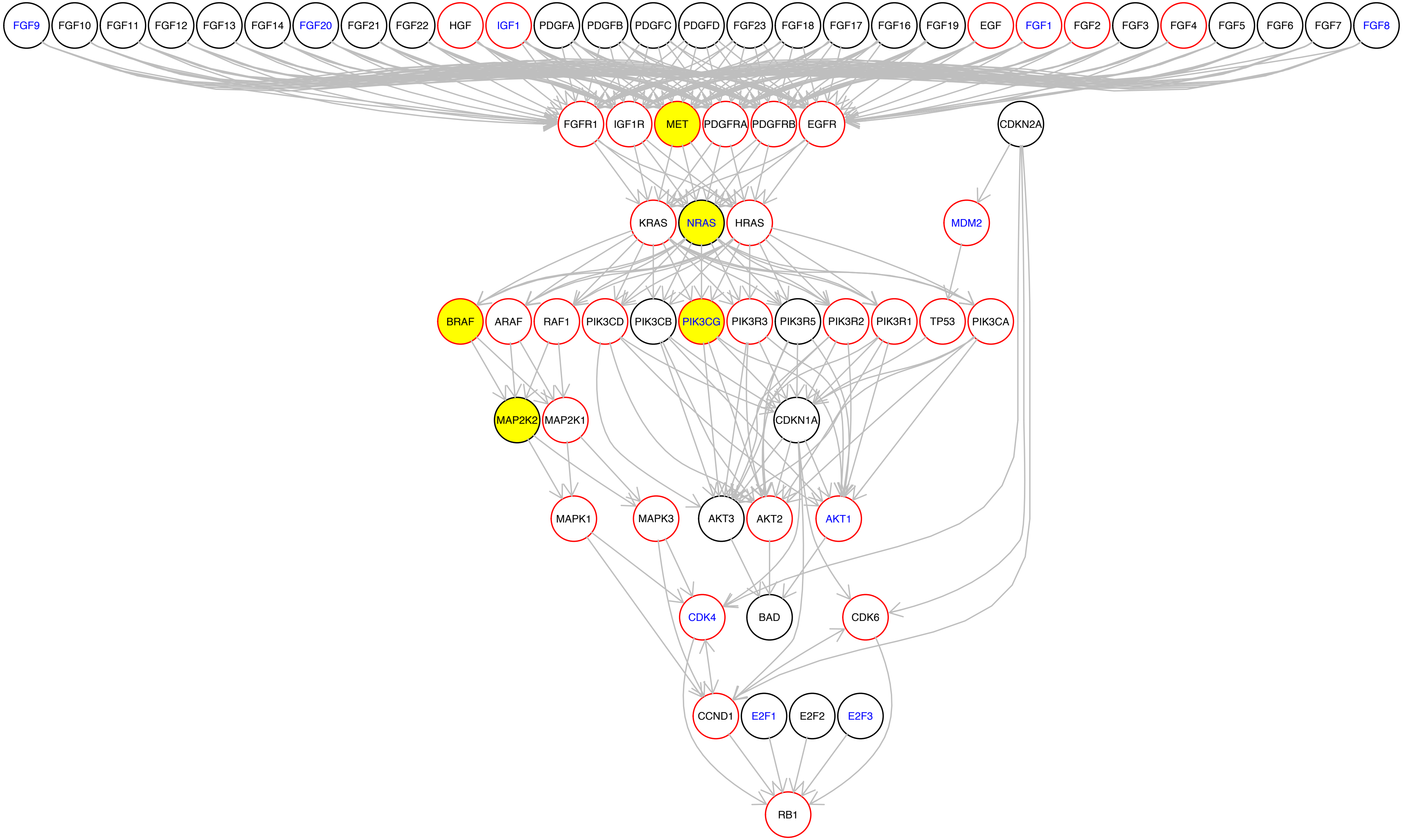

KEGG pathway = Melanoma :   tumour = MEL.Ma.Mel.36.Tumor :   Yellow Fill = gene variant, Blue Text = expression−survival association, Red Boarder = drug

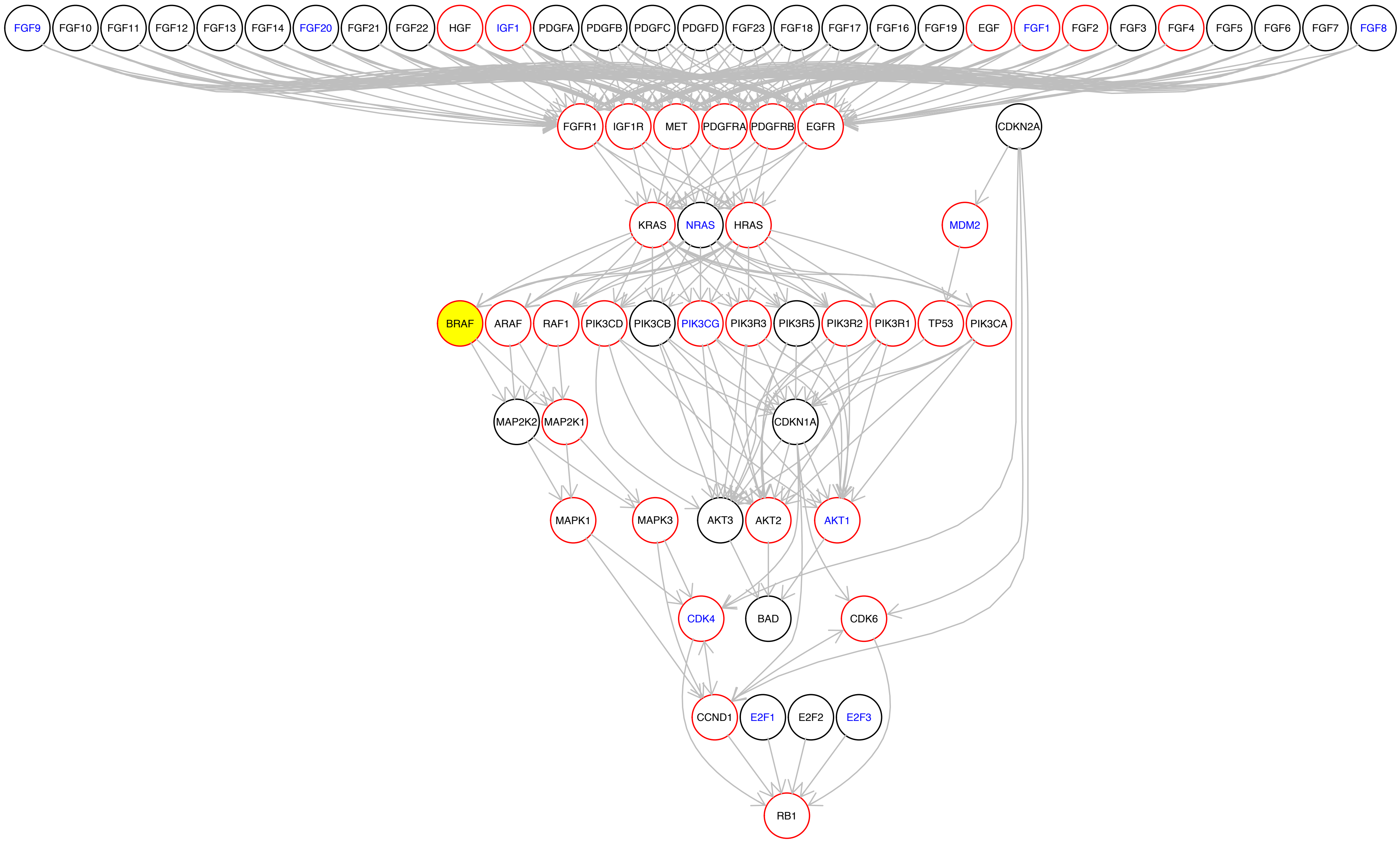

KEGG pathway = Melanoma : tumour = MEL.Ma.Mel.37.Tumor : Yellow Fill = gene variant, Blue Text = expression−survival association, Red Boarder = drug

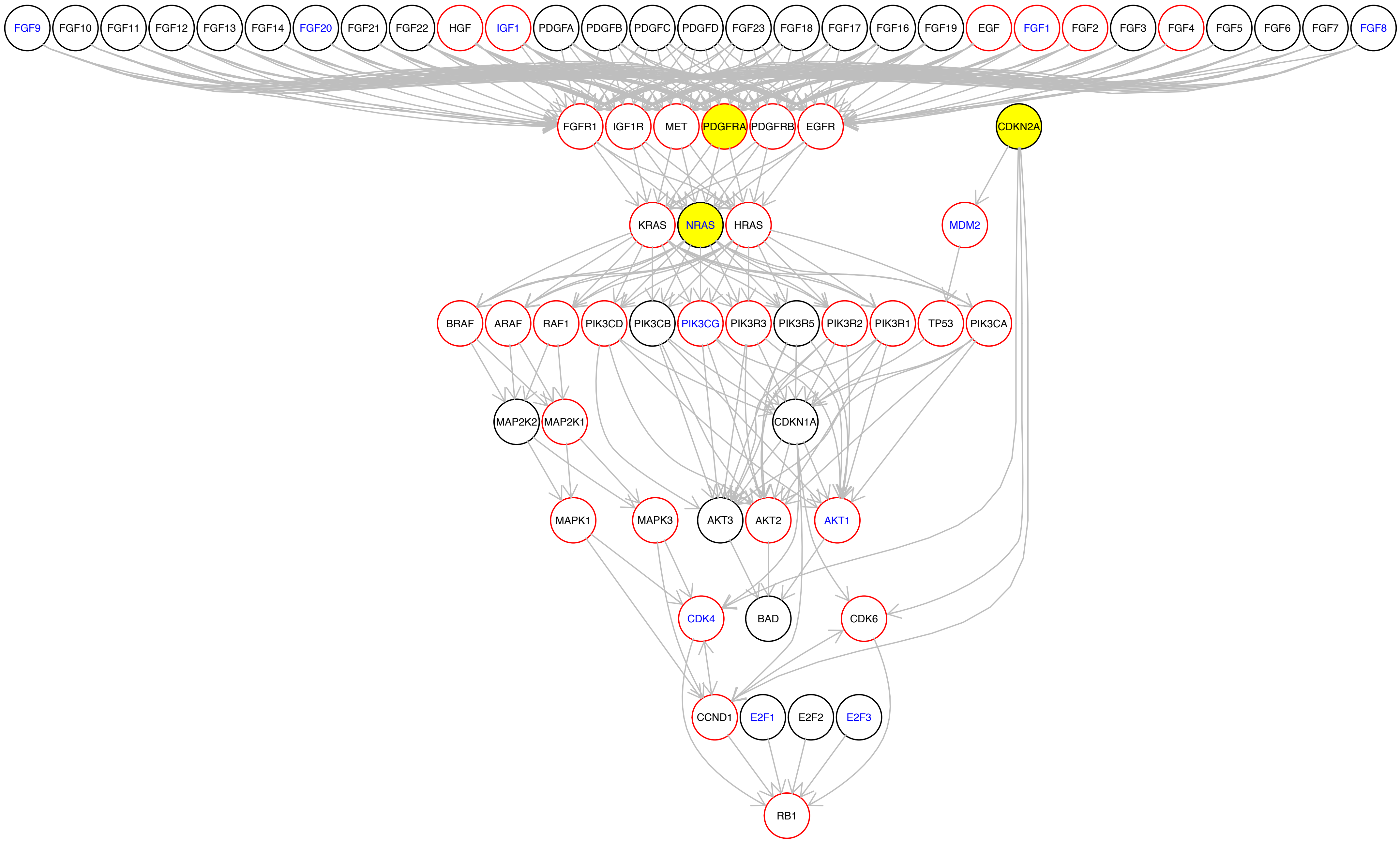

KEGG pathway = Melanoma :   tumour = MEL.Ma.Mel.48.Tumor :   Yellow Fill = gene variant, Blue Text = expression−survival association, Red Boarder = drug

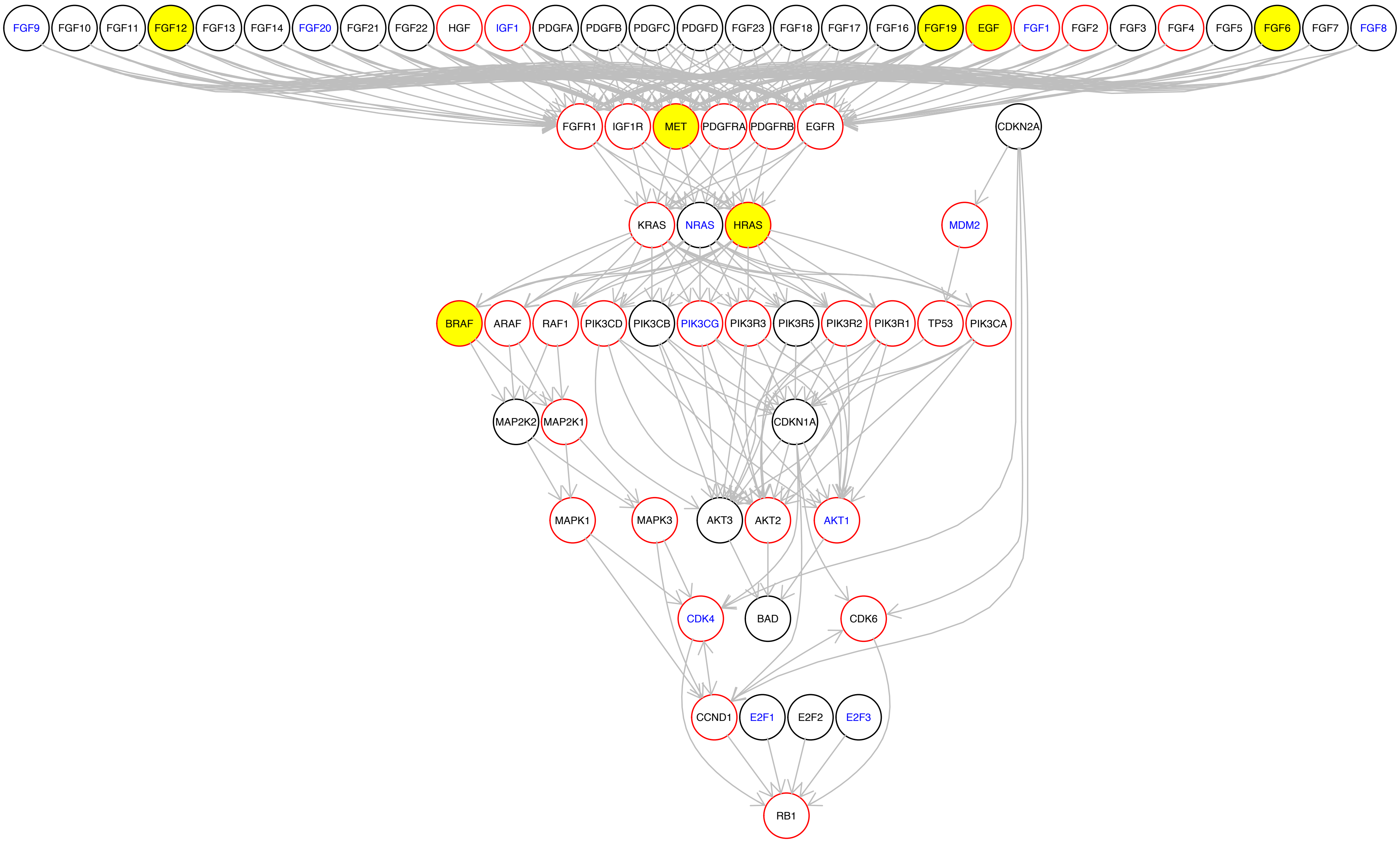

KEGG pathway = Melanoma : tumour = MEL.Ma.Mel.53.Tumor : Yellow Fill = gene variant, Blue Text = expression−survival association, Red Boarder = drug

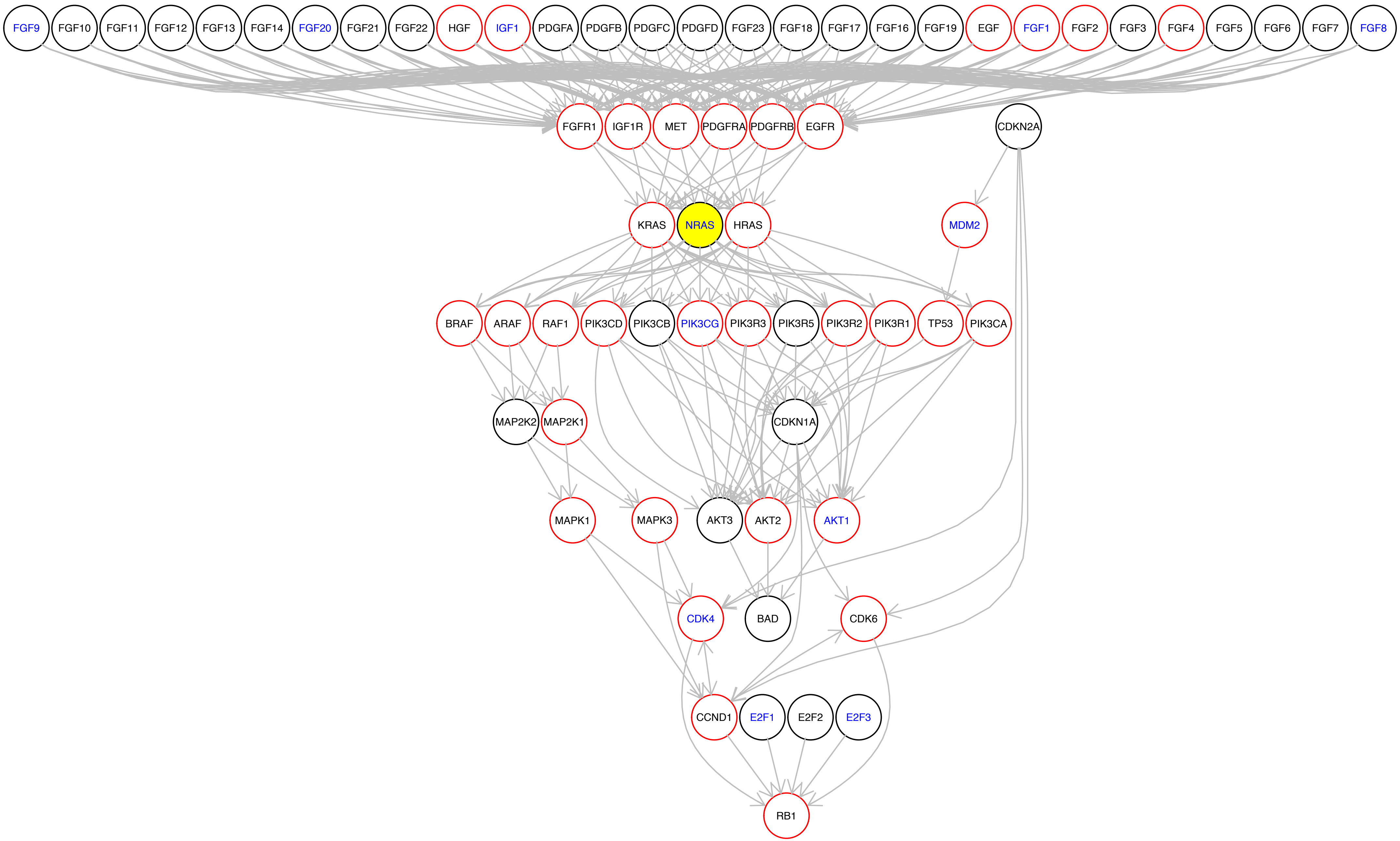

KEGG pathway = Melanoma : tumour = MEL.Ma.Mel.54a.Tumor : Yellow Fill = gene variant, Blue Text = expression–survival association, Red Boarder = drug

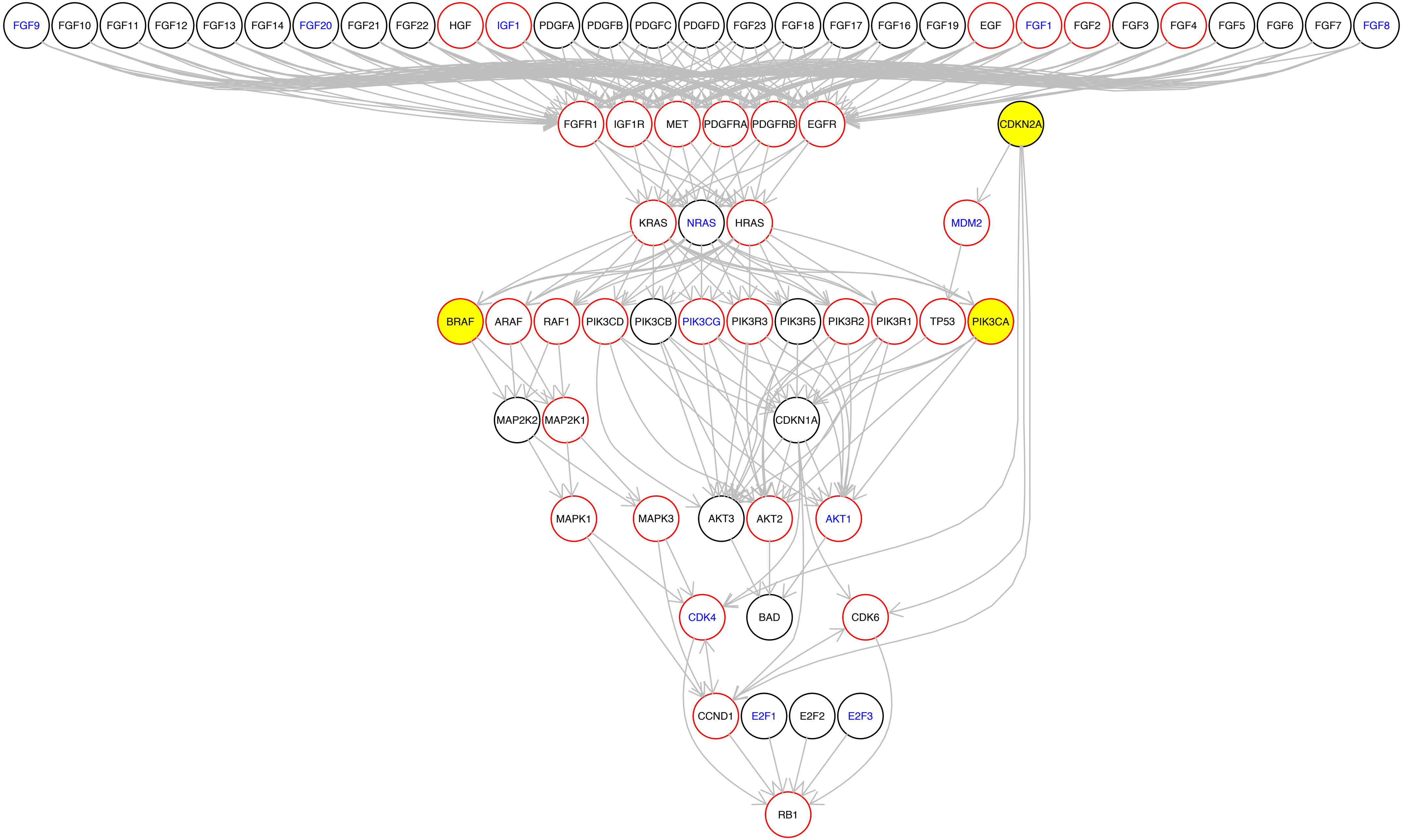

KEGG pathway = Melanoma : tumour = MEL.Ma.Mel.55.Tumor : Yellow Fill = gene variant, Blue Text = expression−survival association, Red Boarder = drug

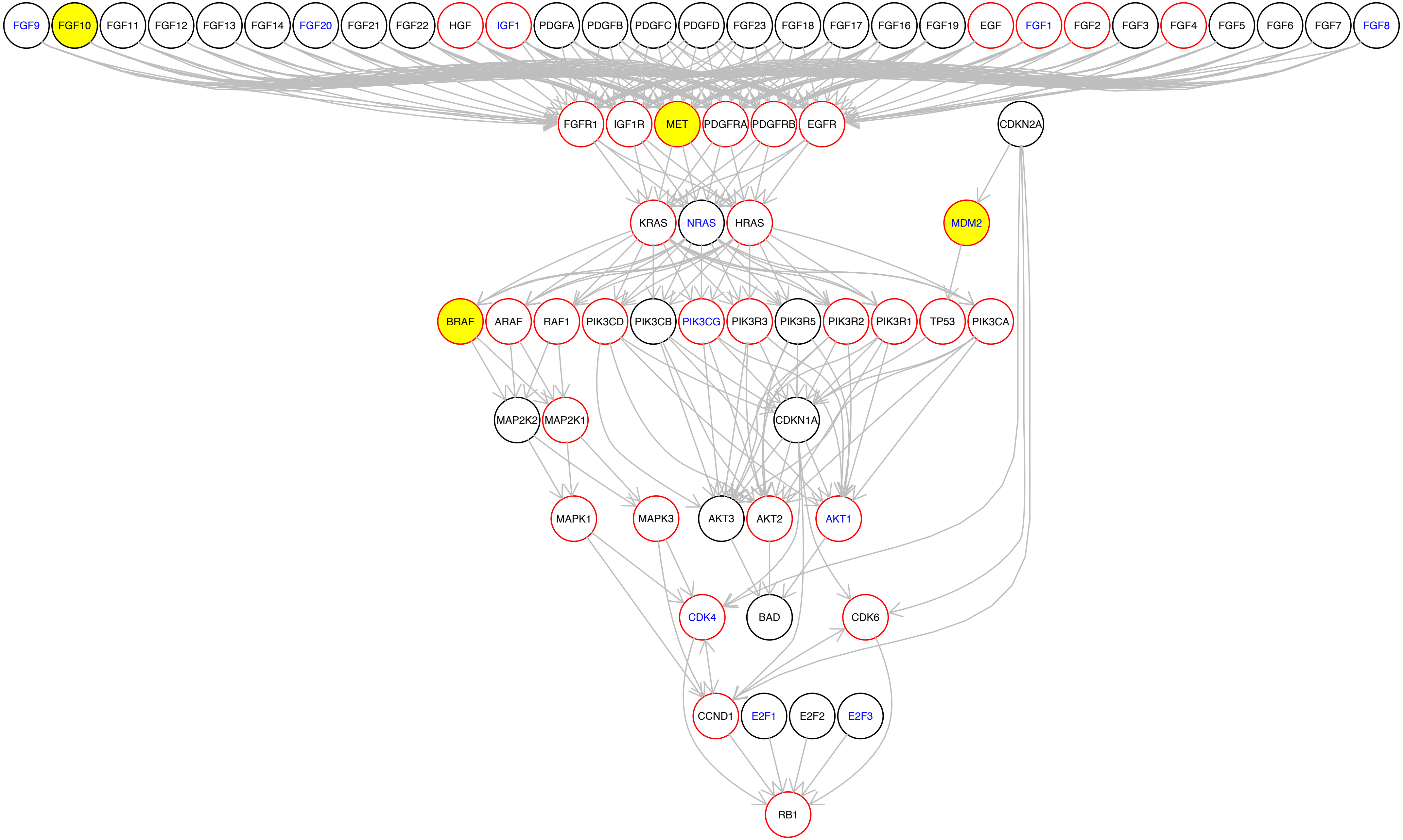

KEGG pathway = Melanoma : tumour = MEL.Ma.Mel.59.Tumor : Yellow Fill = gene variant, Blue Text = expression−survival association, Red Boarder = drug

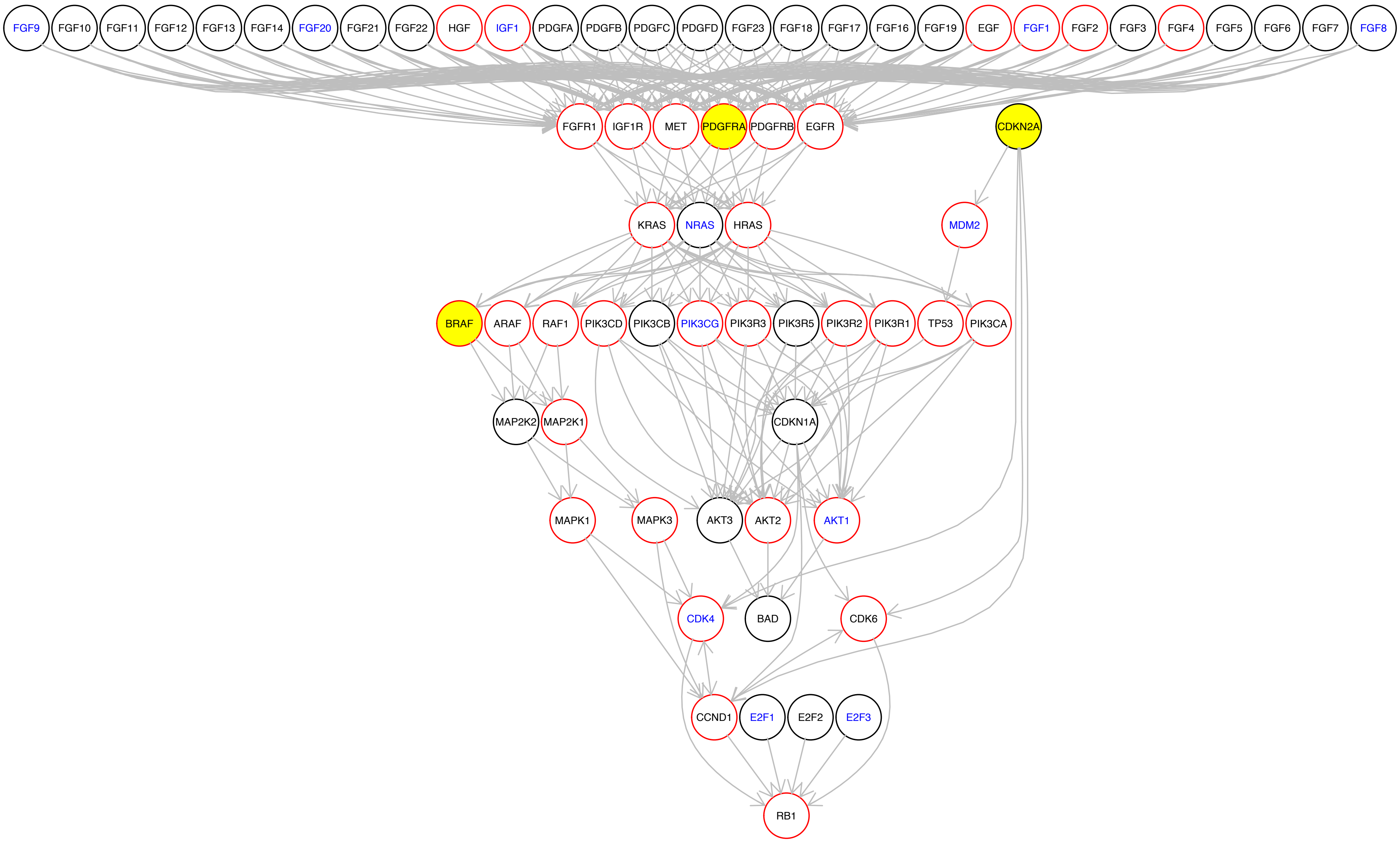

KEGG pathway = Melanoma : tumour = MEL.Ma.Mel.62.Tumor : Yellow Fill = gene variant, Blue Text = expression−survival association, Red Boarder = drug

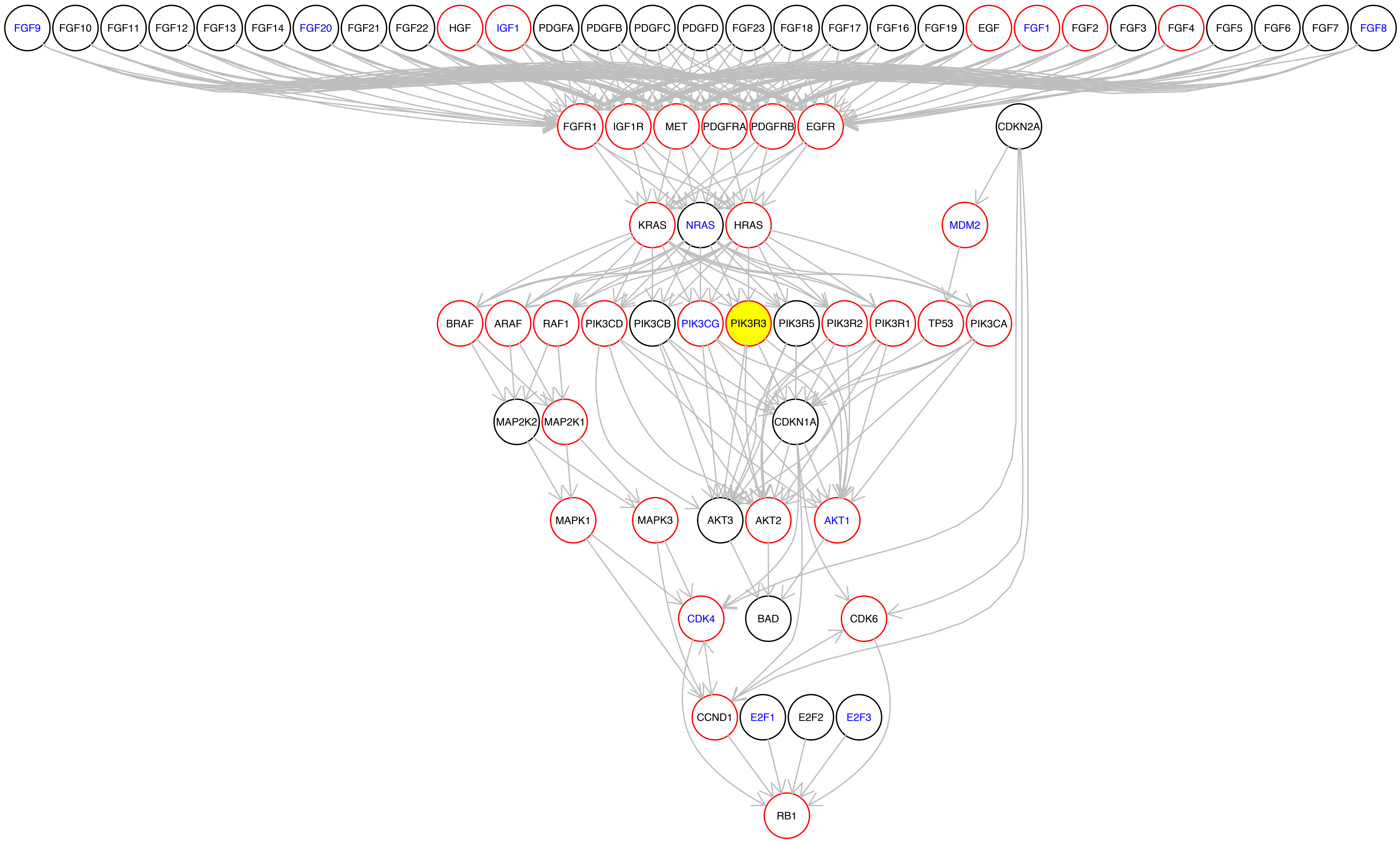

KEGG pathway = Melanoma : tumour = MEL.Ma.Mel.63.Tumor : Yellow Fill = gene variant, Blue Text = expression−survival association, Red Boarder = drug

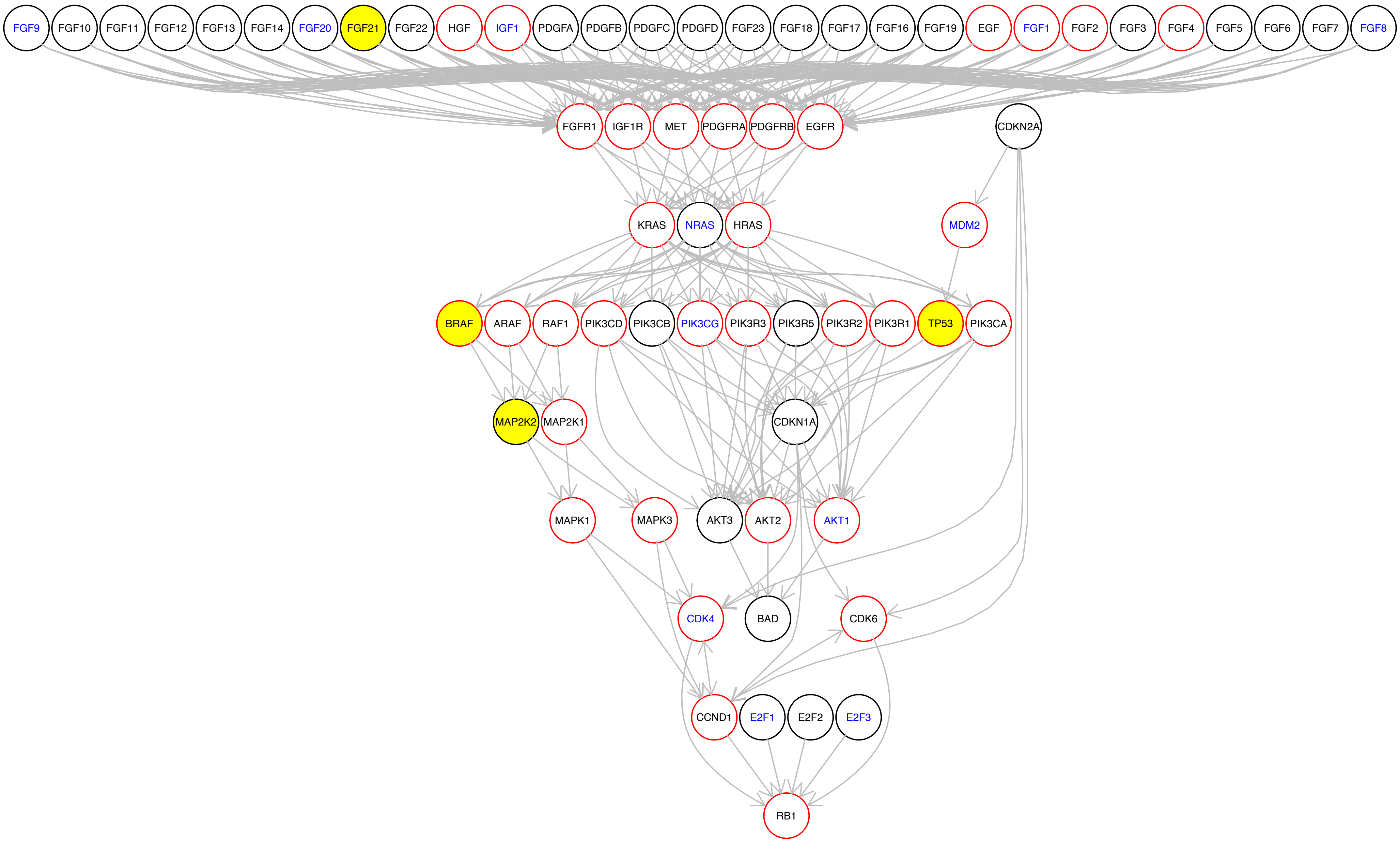

KEGG pathway = Melanoma : tumour = MEL.Ma.Mel.65.Tumor : Yellow Fill = gene variant, Blue Text = expression−survival association, Red Boarder = drug

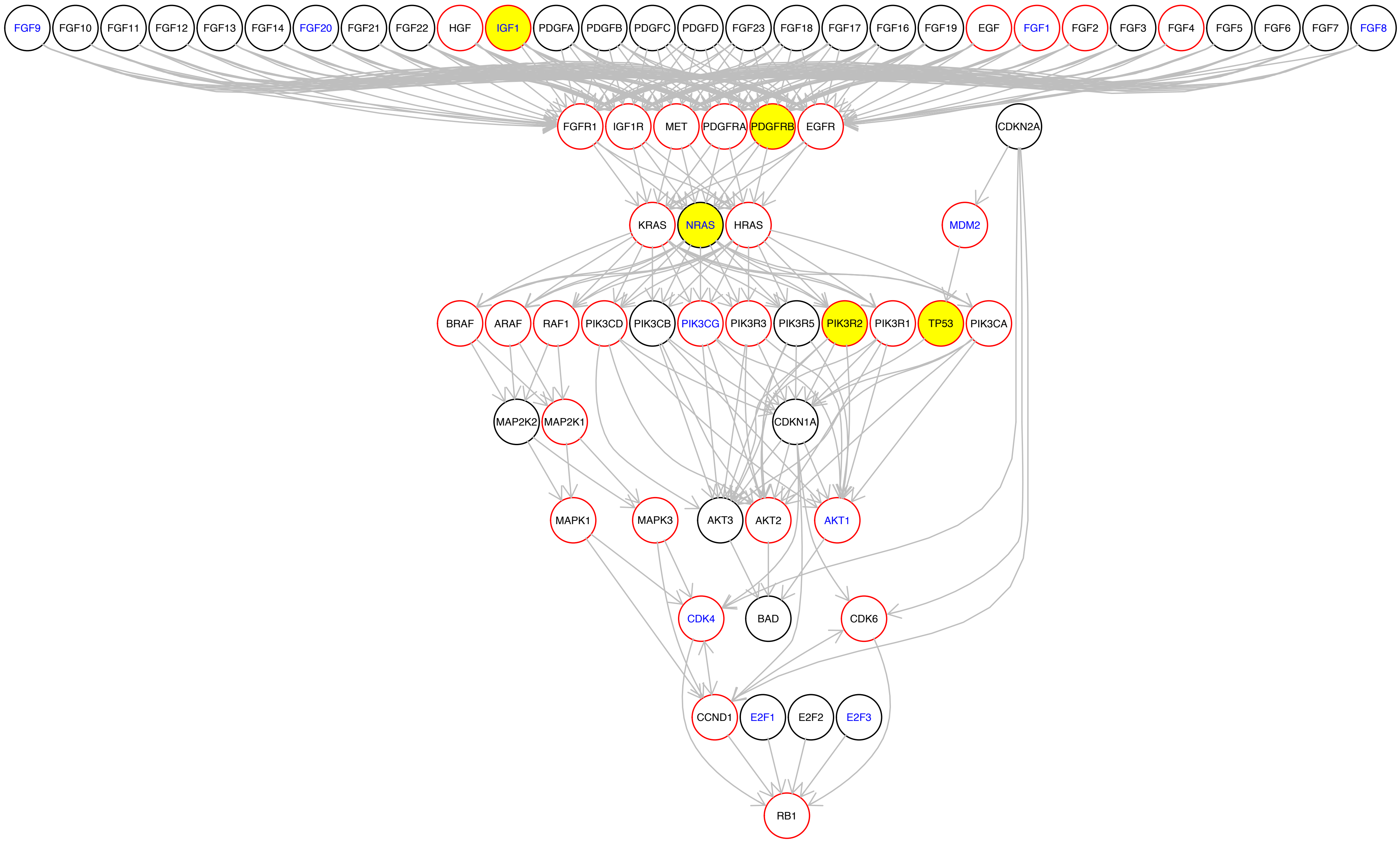

KEGG pathway = Melanoma : tumour = MEL.Ma.Mel.67.Tumor : Yellow Fill = gene variant, Blue Text = expression−survival association, Red Boarder = drug

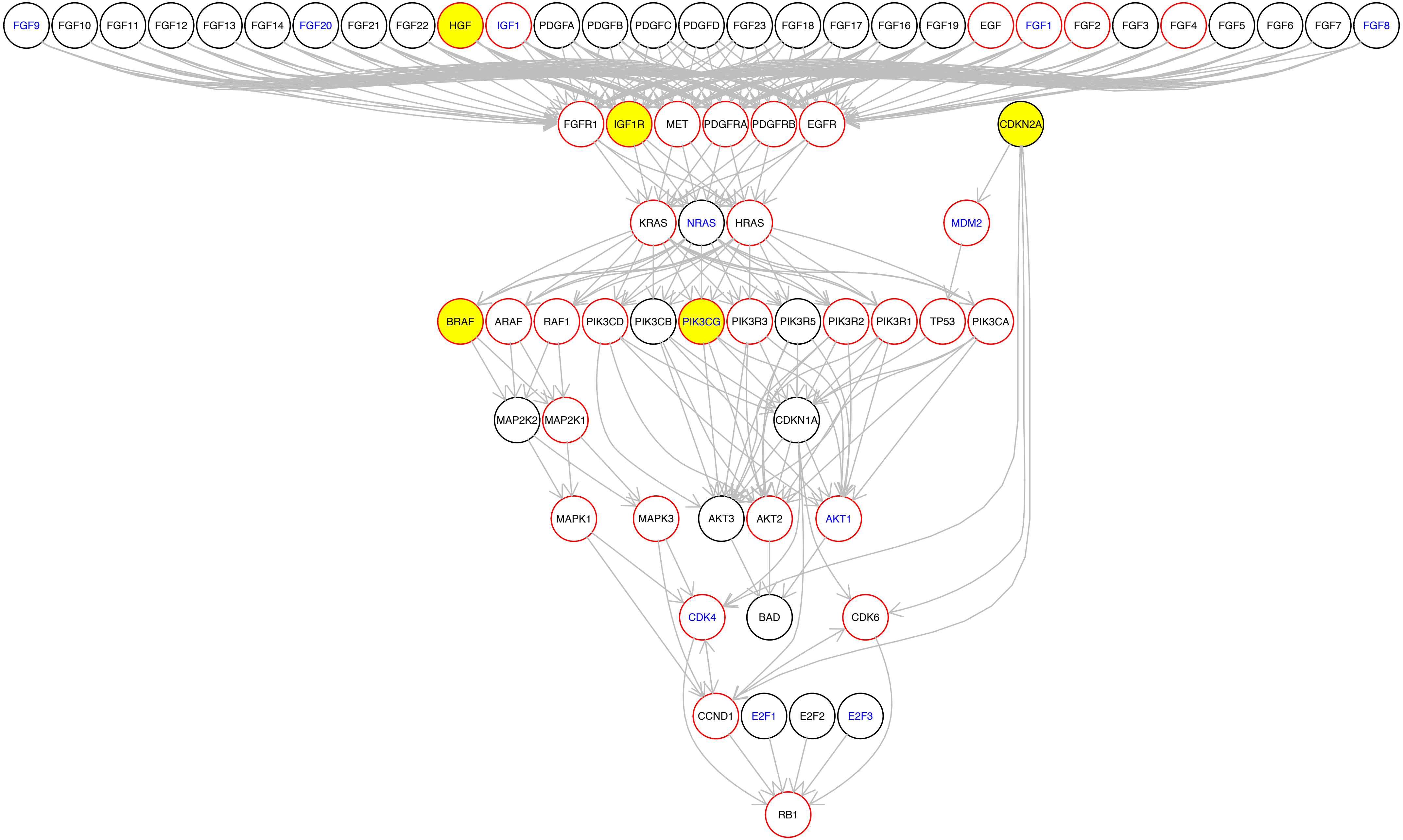

KEGG pathway = Melanoma : tumour = MEL.Ma.Mel.71.Tumor : Yellow Fill = gene variant, Blue Text = expression−survival association, Red Boarder = drug

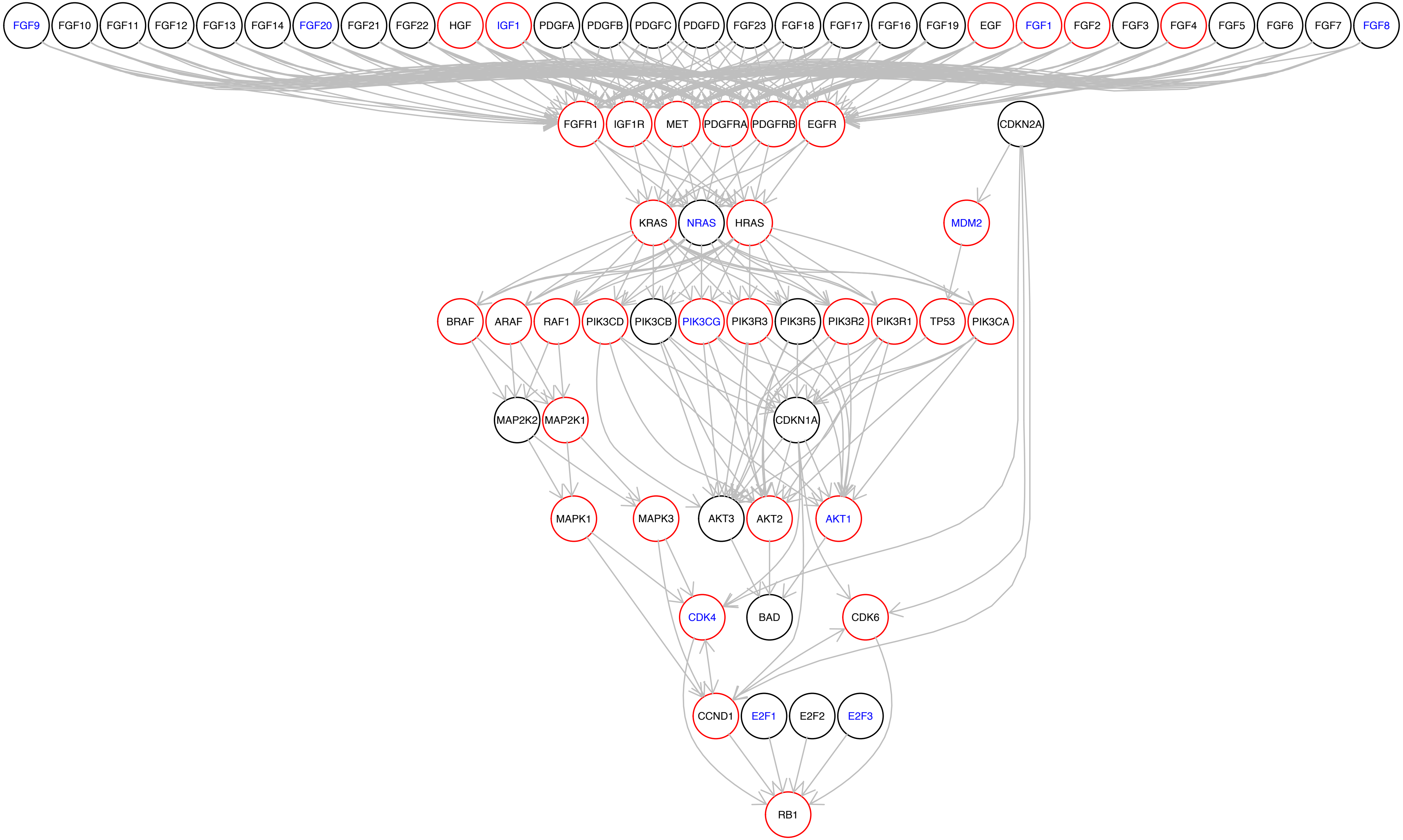

KEGG pathway = Melanoma : tumour = MEL.Ma.Mel.76.Tumor : Yellow Fill = gene variant, Blue Text = expression−survival association, Red Boarder = drug

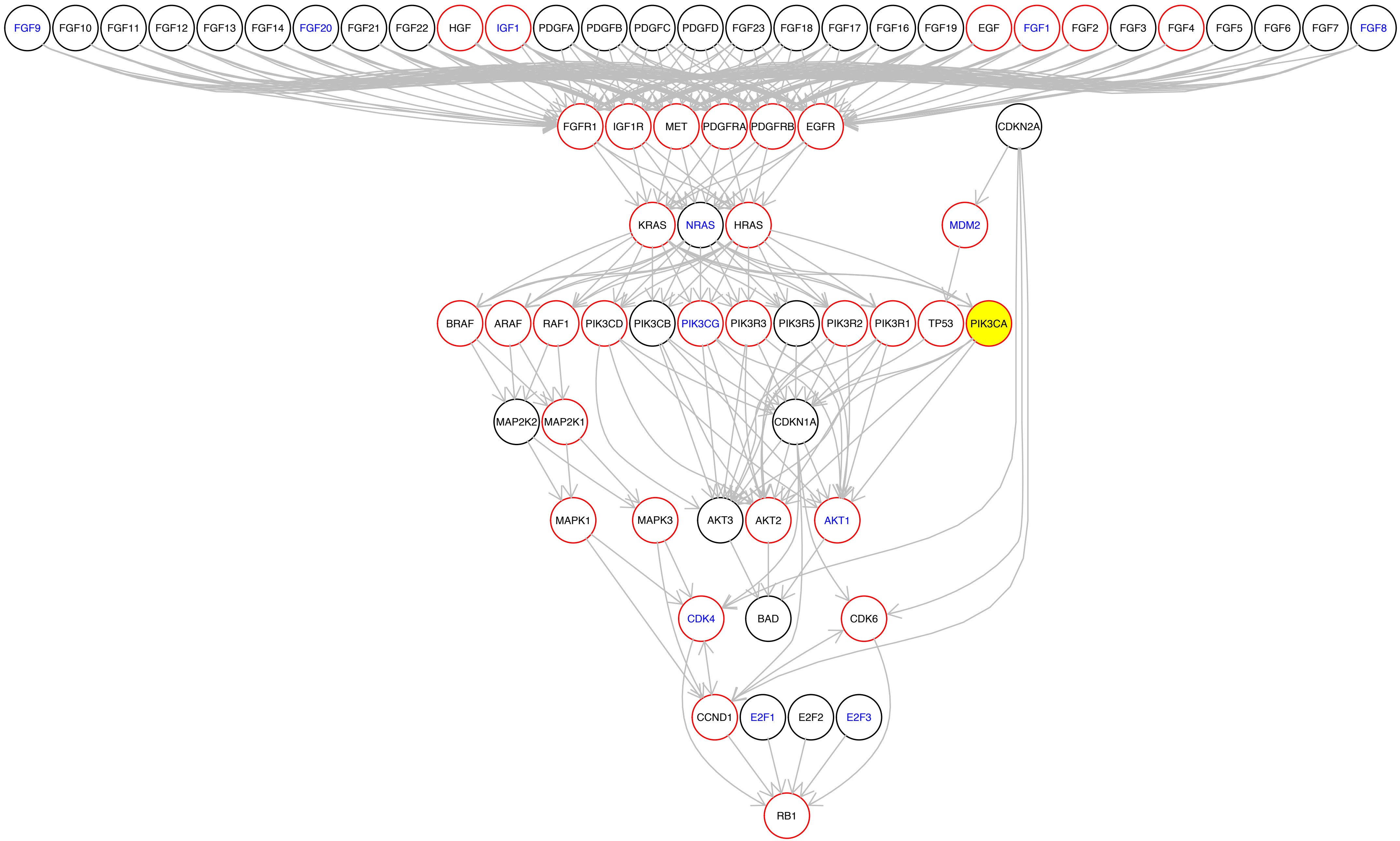

KEGG pathway = Melanoma : tumour = MEL.Ma.Mel.79.Tumor : Yellow Fill = gene variant, Blue Text = expression−survival association, Red Boarder = drug

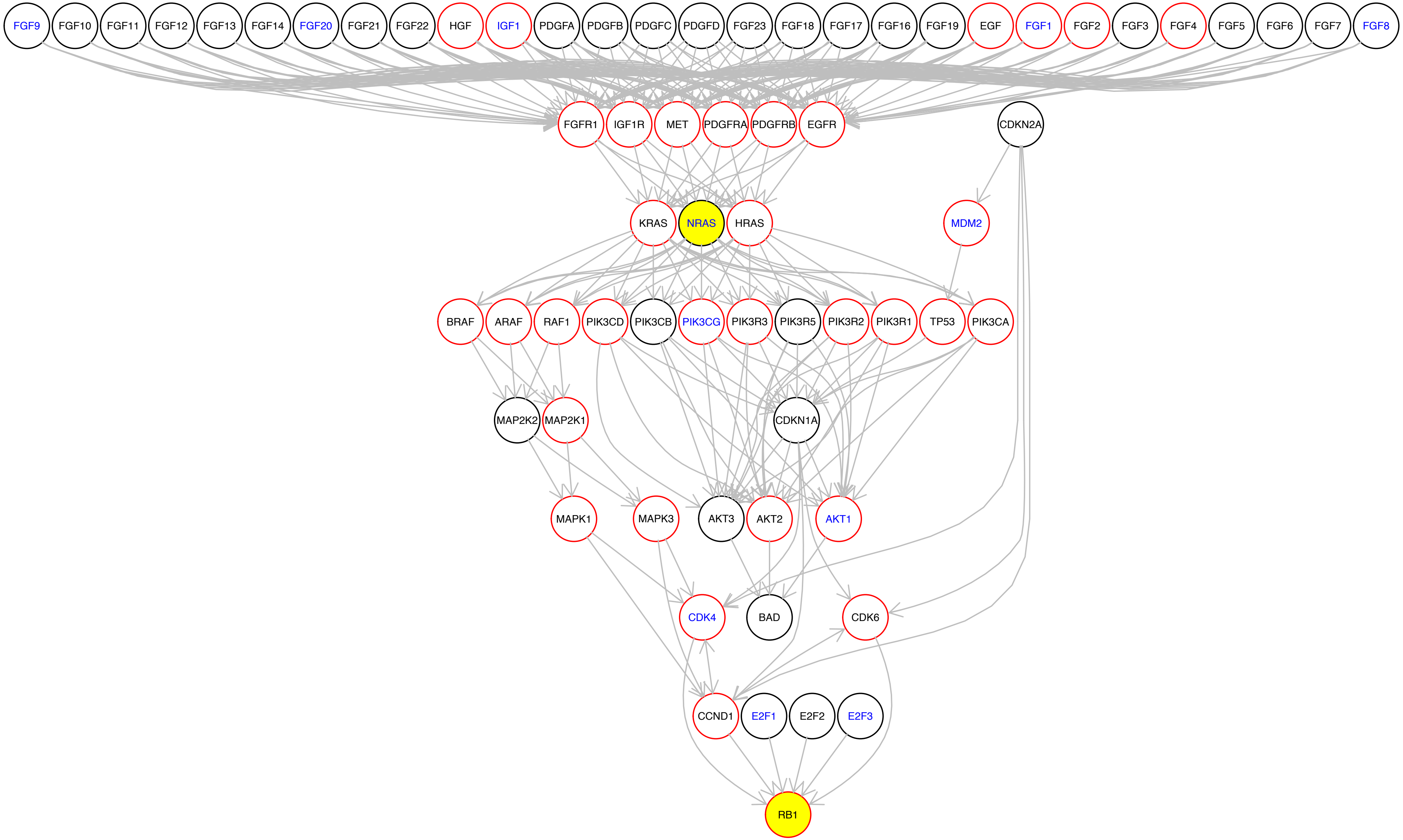

KEGG pathway = Melanoma :   tumour = MEL.Ma.Mel.85.Tumor :   Yellow Fill = gene variant, Blue Text = expression−survival association, Red Boarder = drug

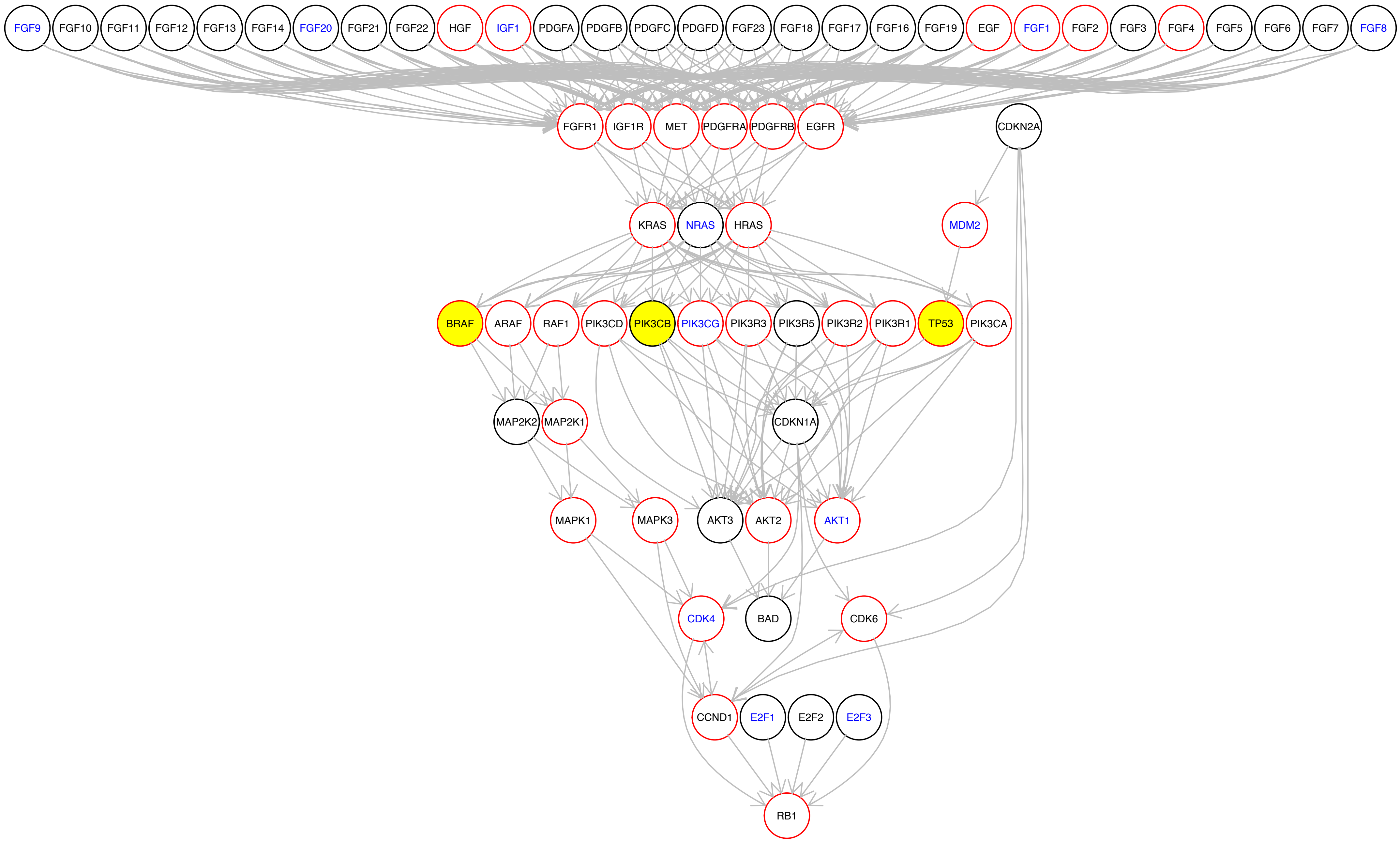

KEGG pathway = Melanoma : tumour = MEL.Ma.Mel.86.Tumor : Yellow Fill = gene variant, Blue Text = expression−survival association, Red Boarder = drug

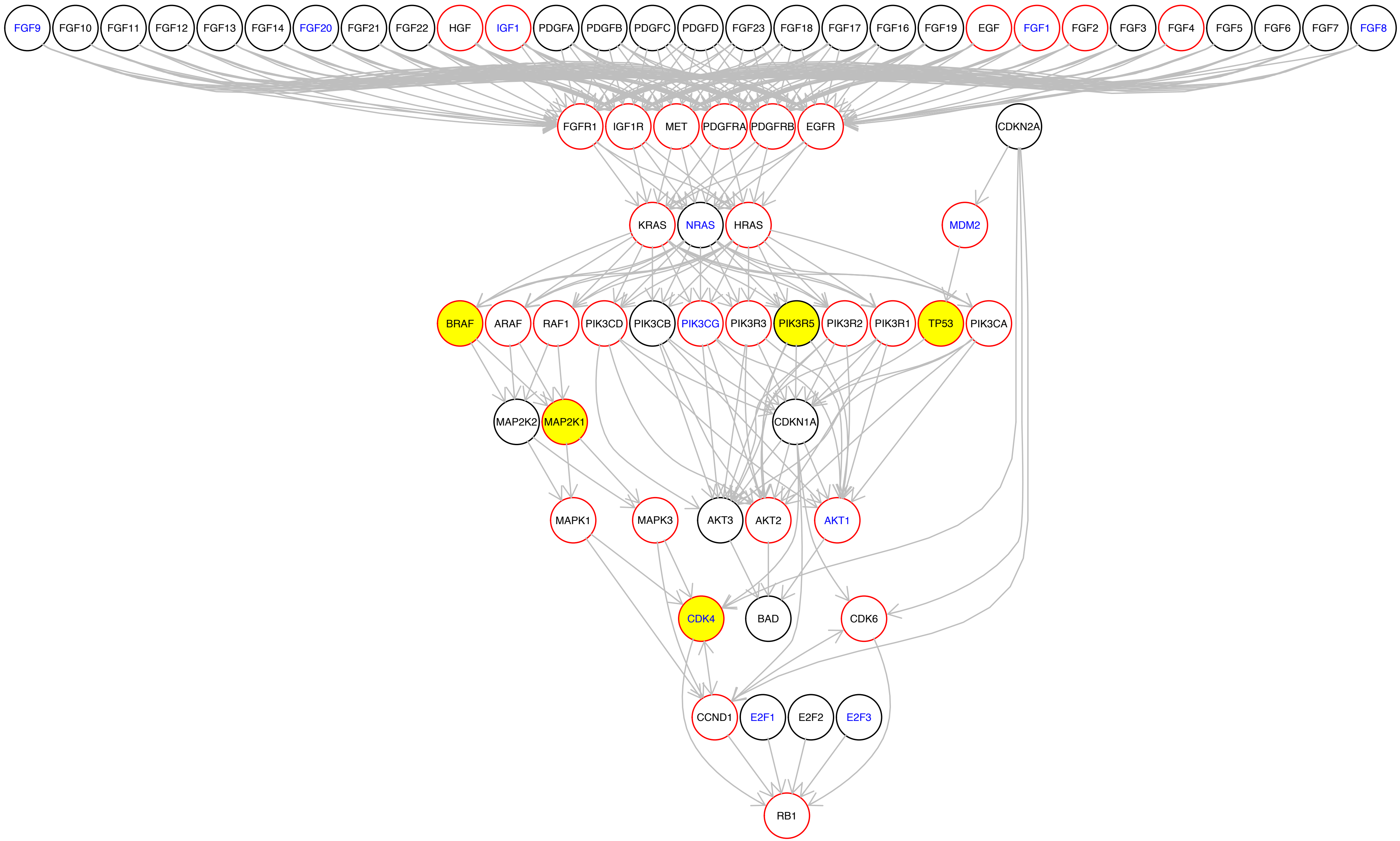

KEGG pathway = Melanoma : tumour = MEL.Ma.Mel.91.Tumor : Yellow Fill = gene variant, Blue Text = expression−survival association, Red Boarder = drug

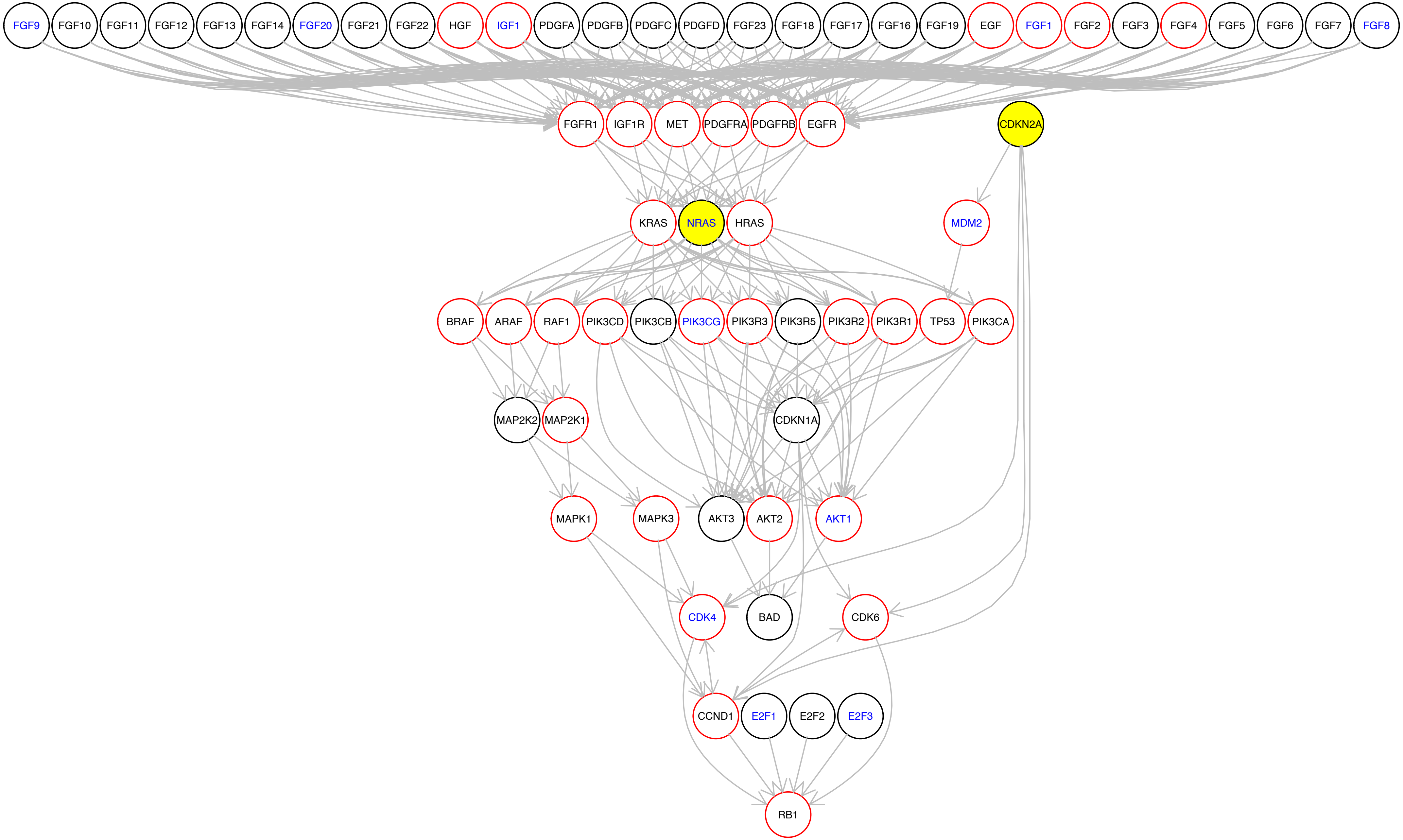

KEGG pathway = Melanoma : tumour = MEL.Ma.Mel.92.Tumor : Yellow Fill = gene variant, Blue Text = expression−survival association, Red Boarder = drug

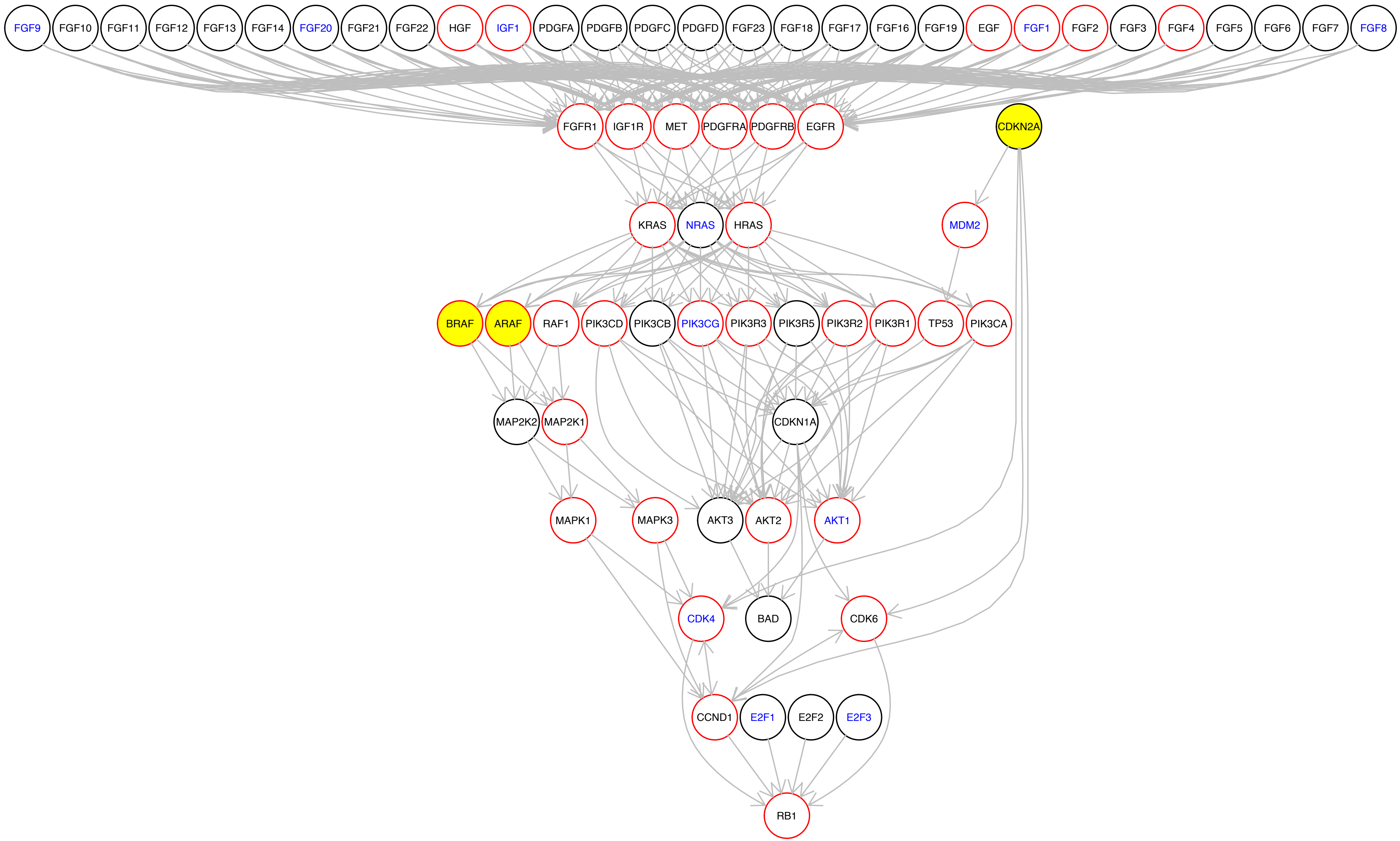

KEGG pathway = Melanoma : tumour = MEL.Ma.Mel.94.Tumor : Yellow Fill = gene variant, Blue Text = expression–survival association, Red Boarder = drug

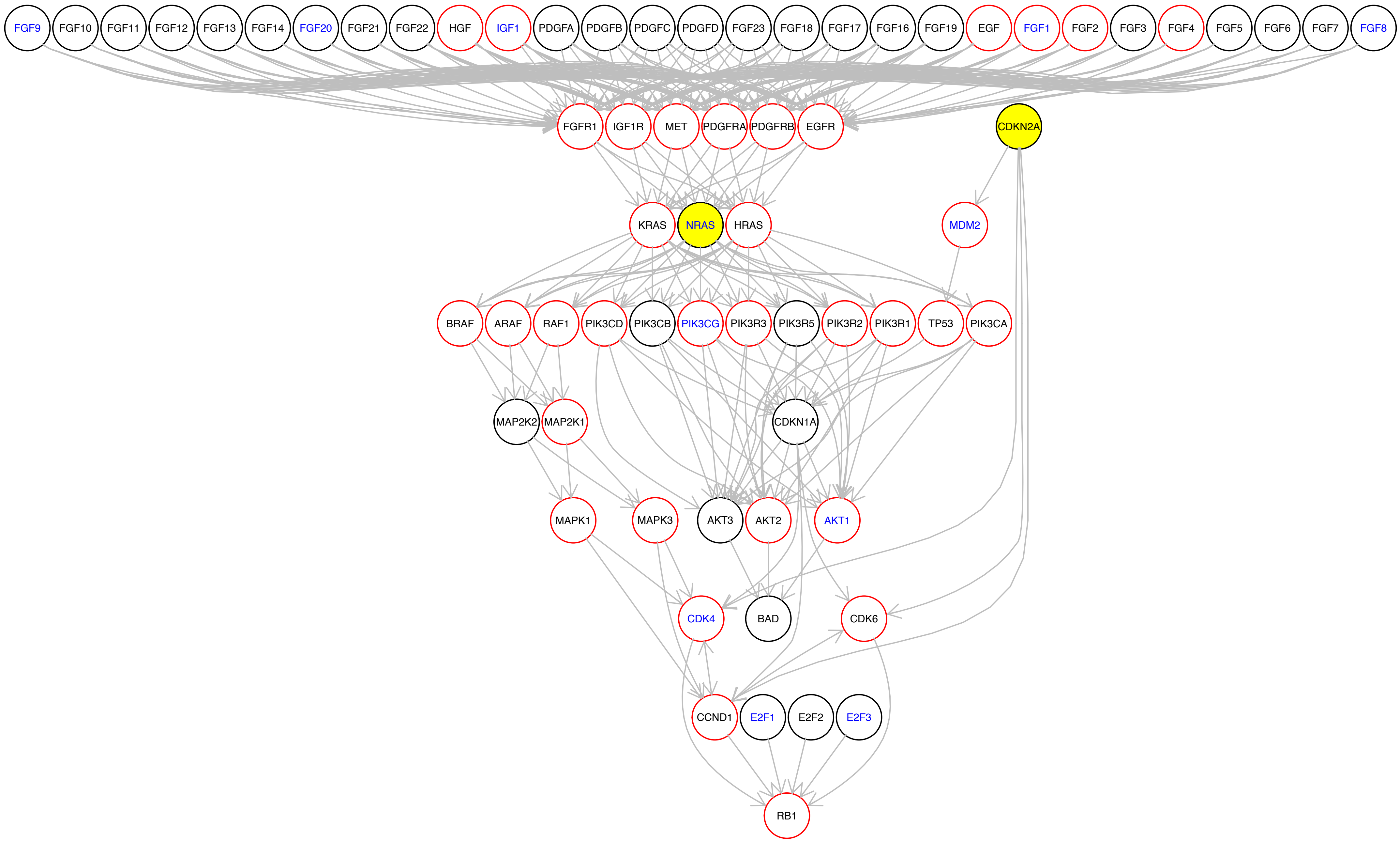

KEGG pathway = Melanoma : tumour = MEL.UKRV.Mel.20.Tumor : Yellow Fill = gene variant, Blue Text = expression−survival association, Red Boarder = drug

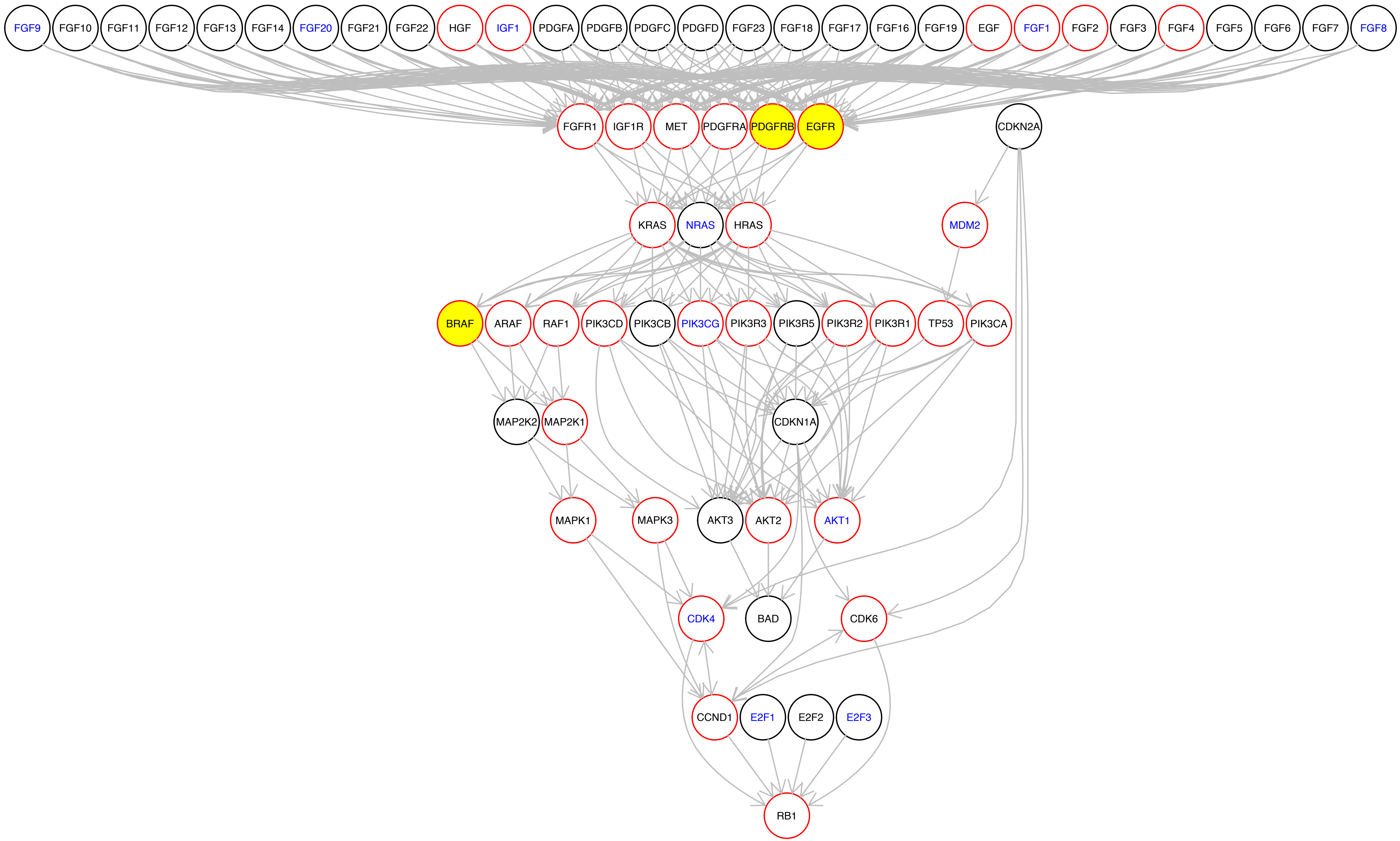

KEGG pathway = Melanoma : tumour = MEL.UKRV.Mel.24.Tumor : Yellow Fill = gene variant, Blue Text = expression−survival association, Red Boarder = drug

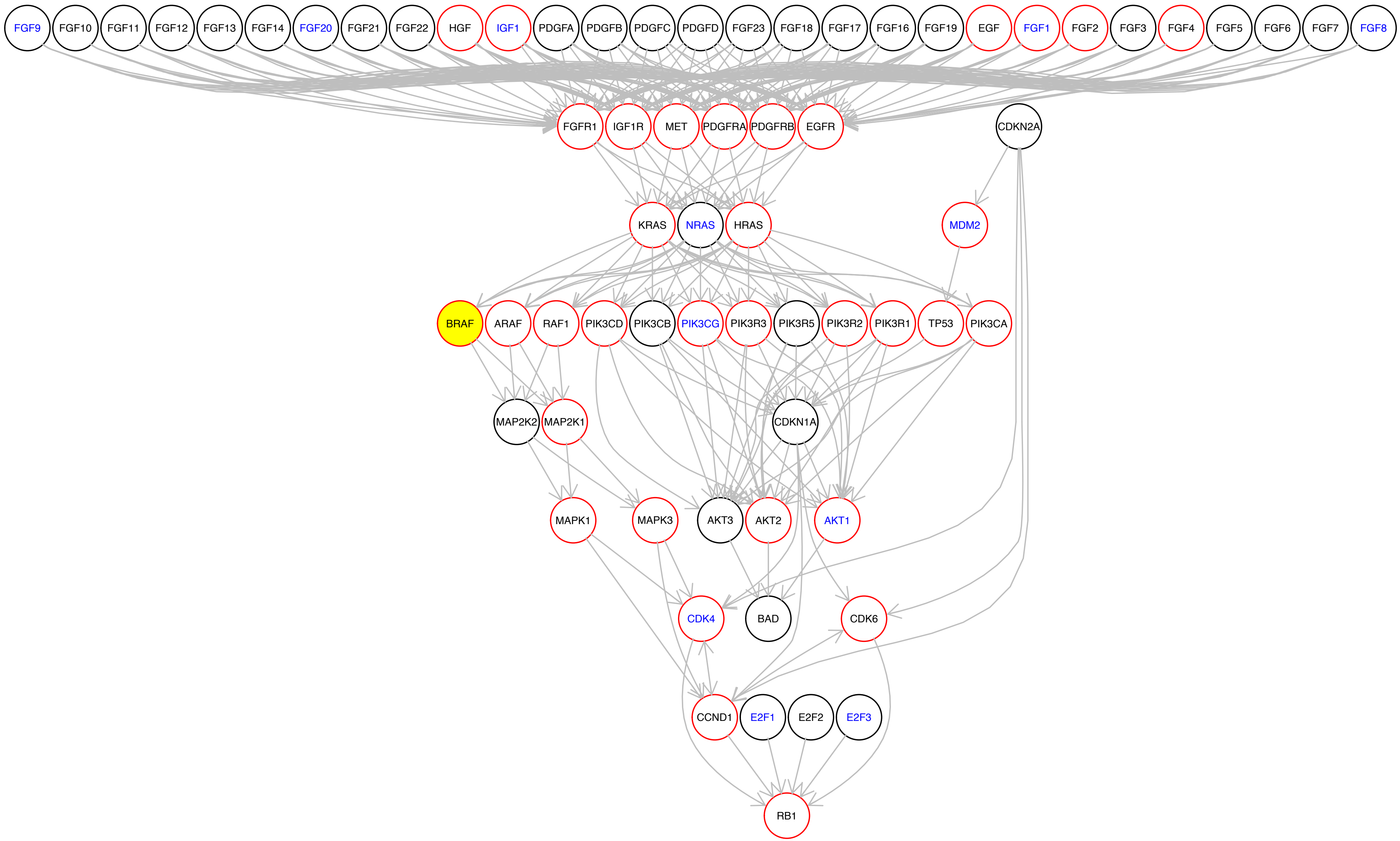

KEGG pathway = Melanoma : tumour = MEL.UKRV.Mel.6.Tumor : Yellow Fill = gene variant, Blue Text = expression−survival association, Red Boarder = drug

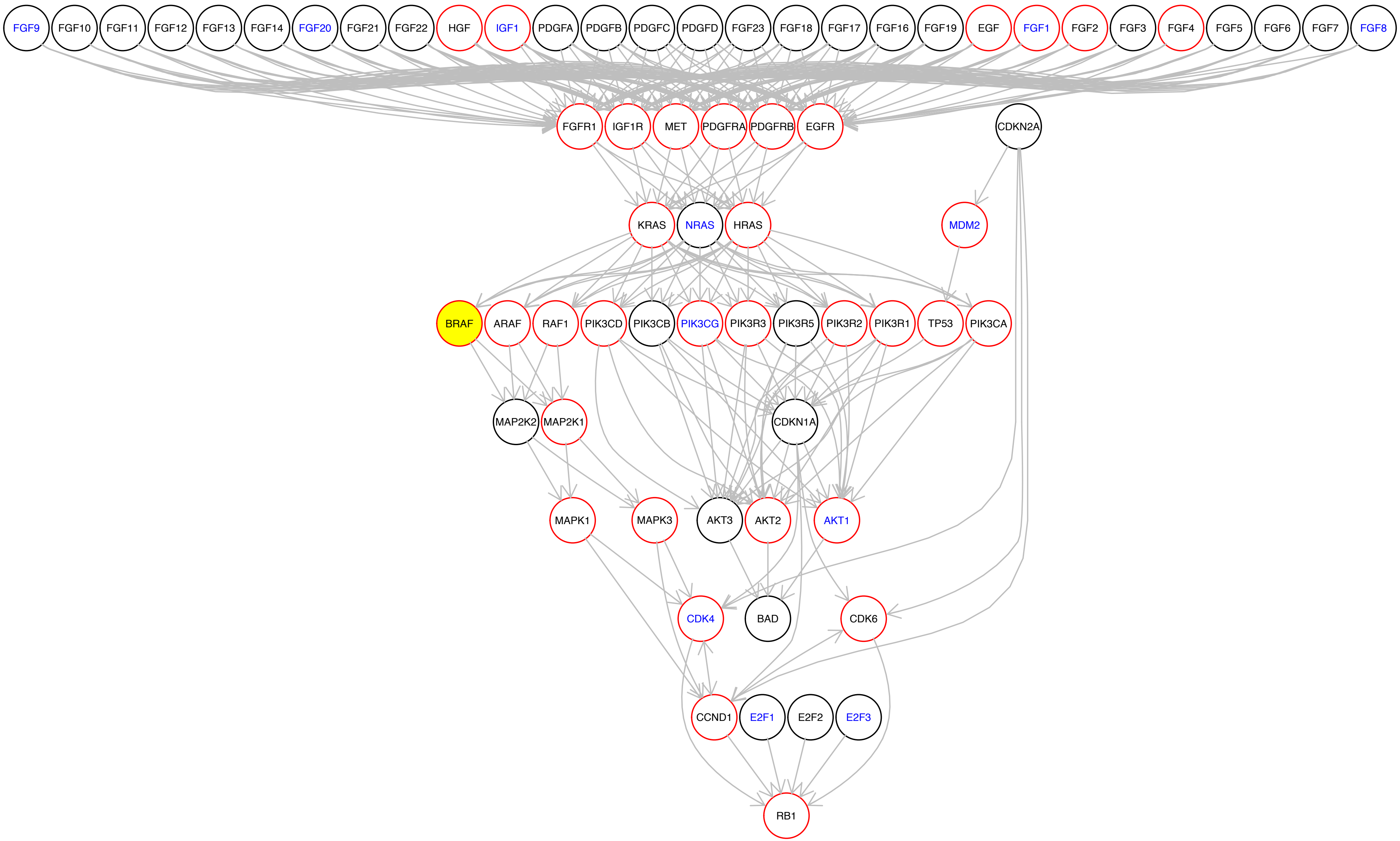

KEGG pathway = Melanoma : tumour = YUAKER : Yellow Fill = gene variant, Blue Text = expression−survival association, Red Boarder = drug

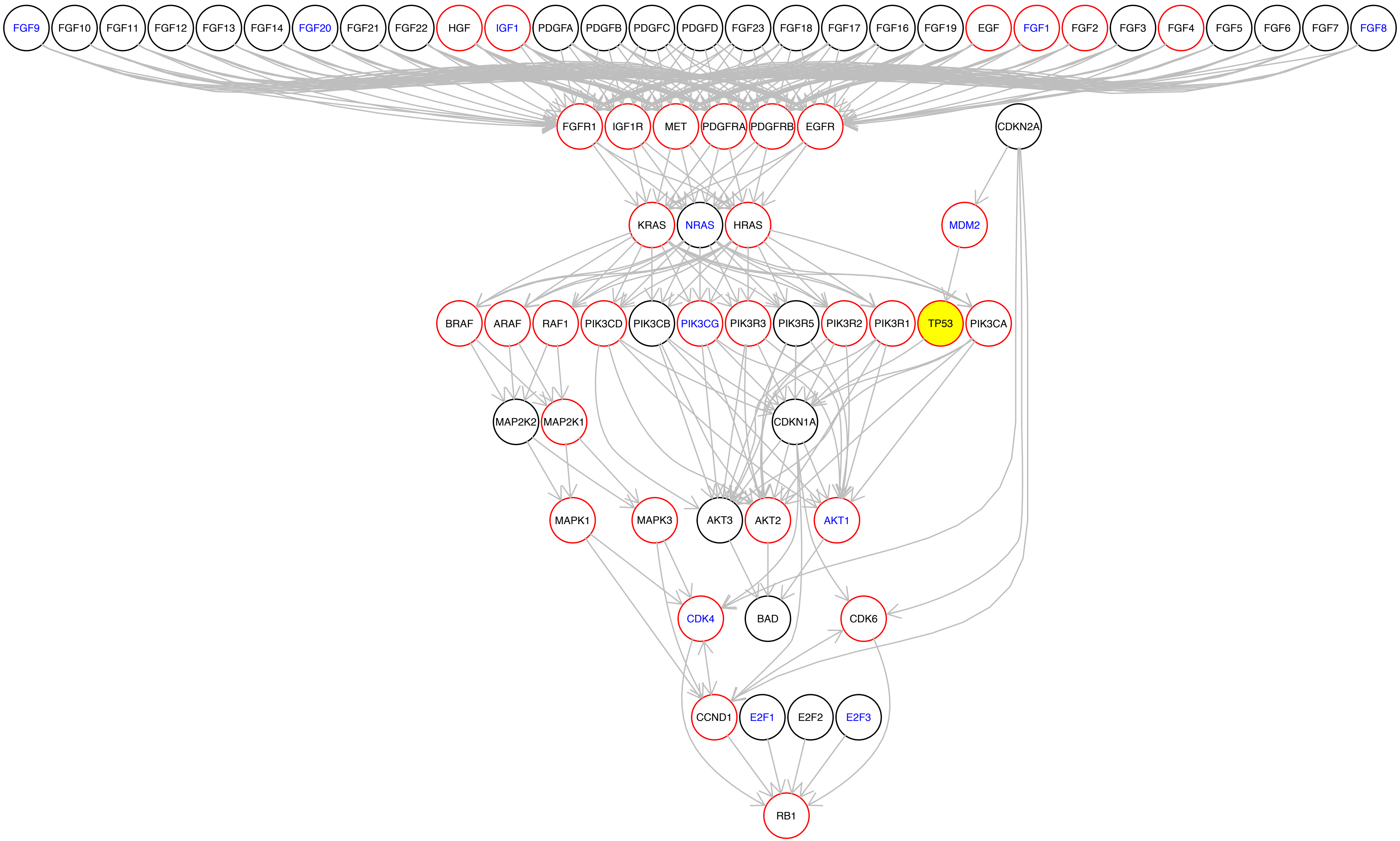

KEGG pathway = Melanoma : tumour = YUAVEY : Yellow Fill = gene variant, Blue Text = expression−survival association, Red Boarder = drug

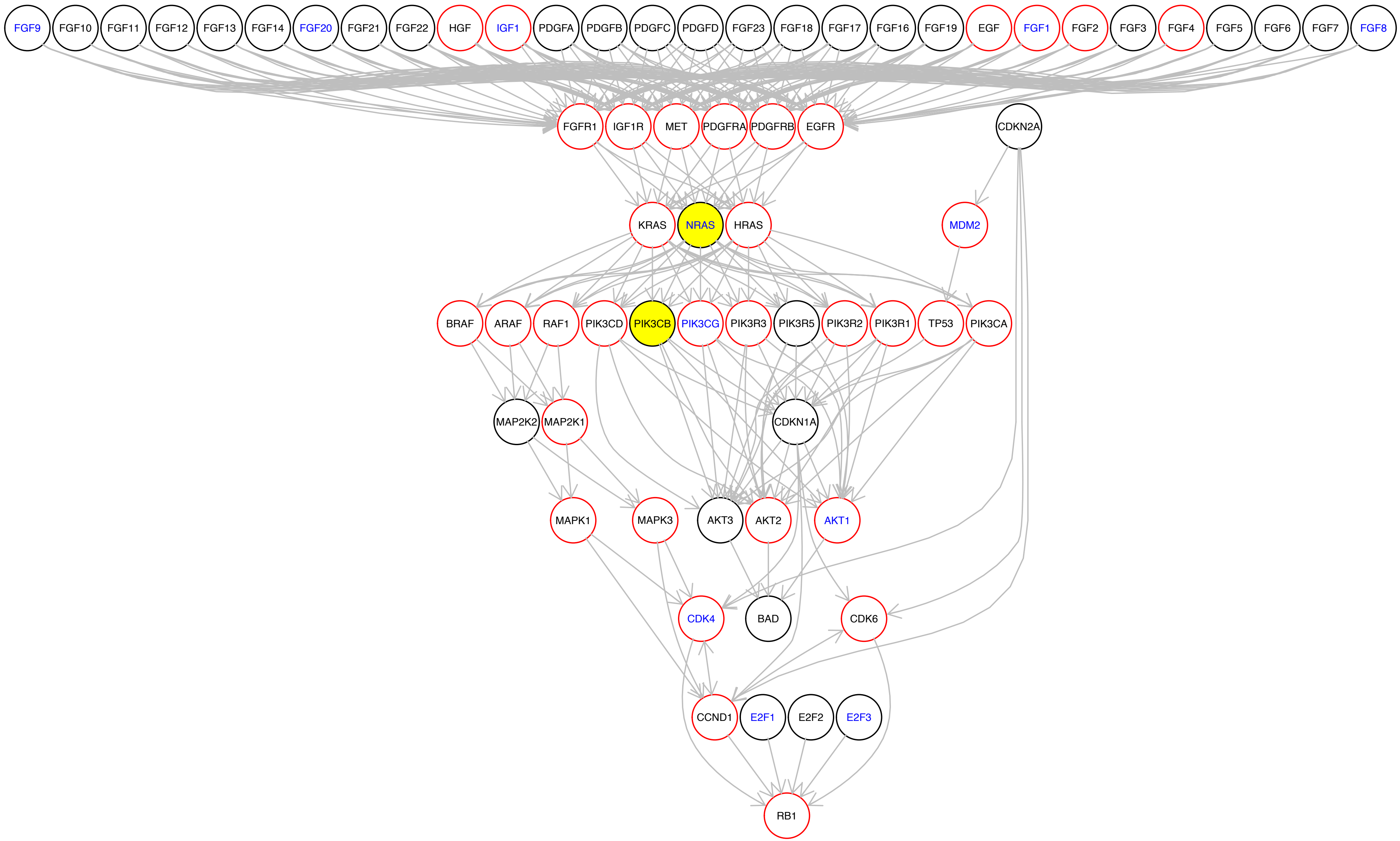

KEGG pathway = Melanoma : tumour = YUBAN : Yellow Fill = gene variant, Blue Text = expression−survival association, Red Boarder = drug

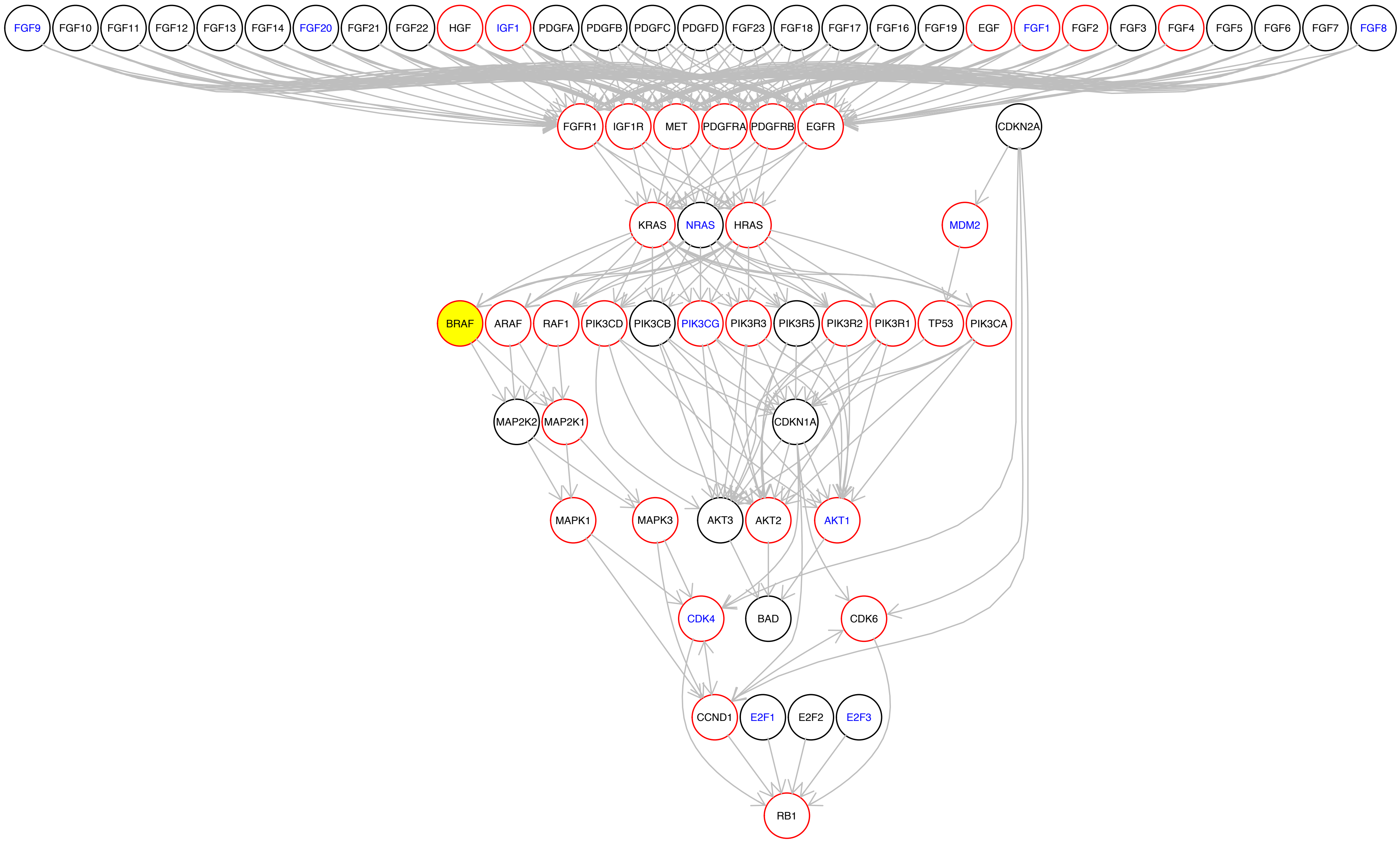

KEGG pathway = Melanoma :   tumour = YUBER :   Yellow Fill = gene variant, Blue Text = expression−survival association, Red Boarder = drug

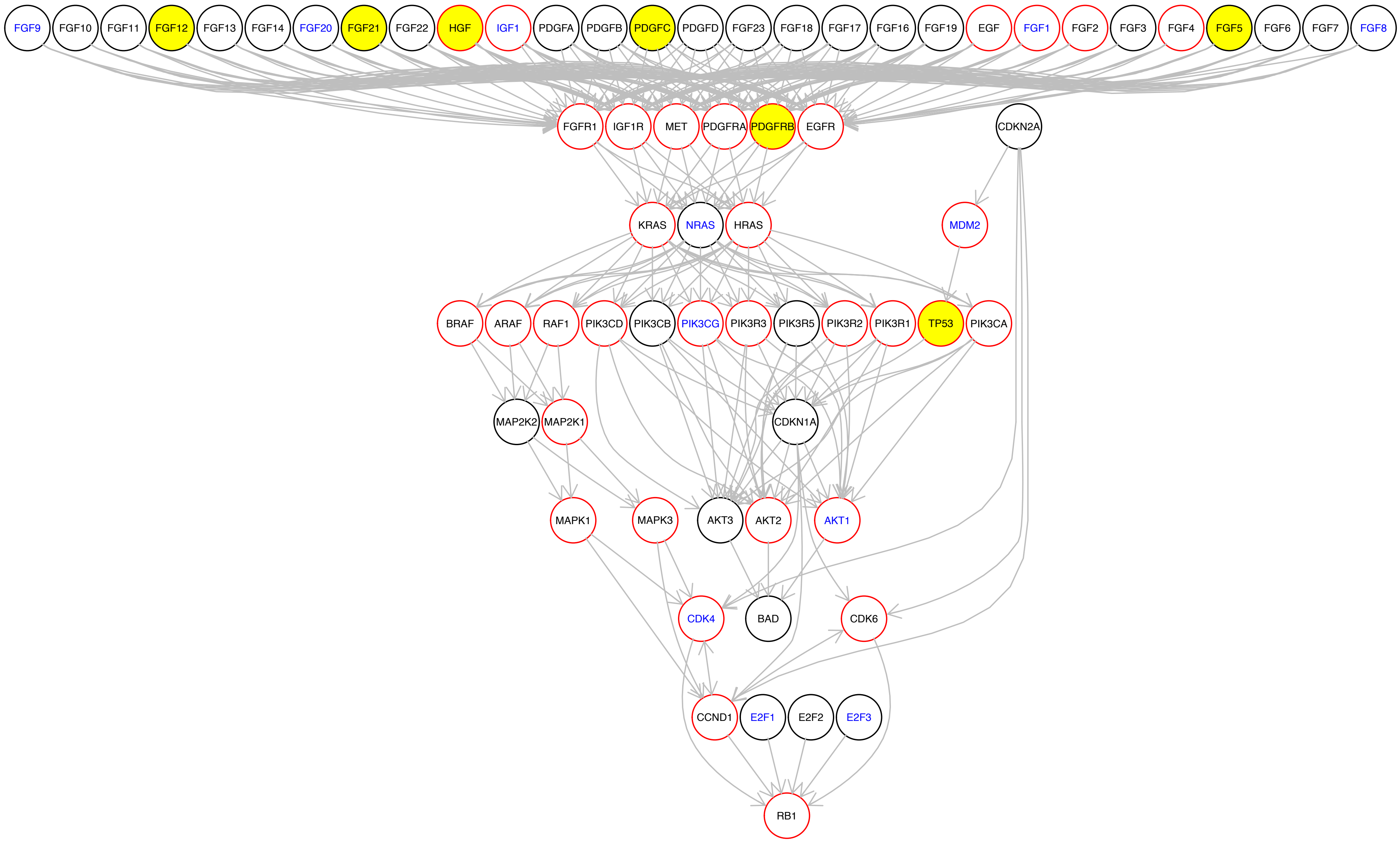

KEGG pathway = Melanoma :   tumour = YUBOO :   Yellow Fill = gene variant, Blue Text = expression−survival association, Red Boarder = drug

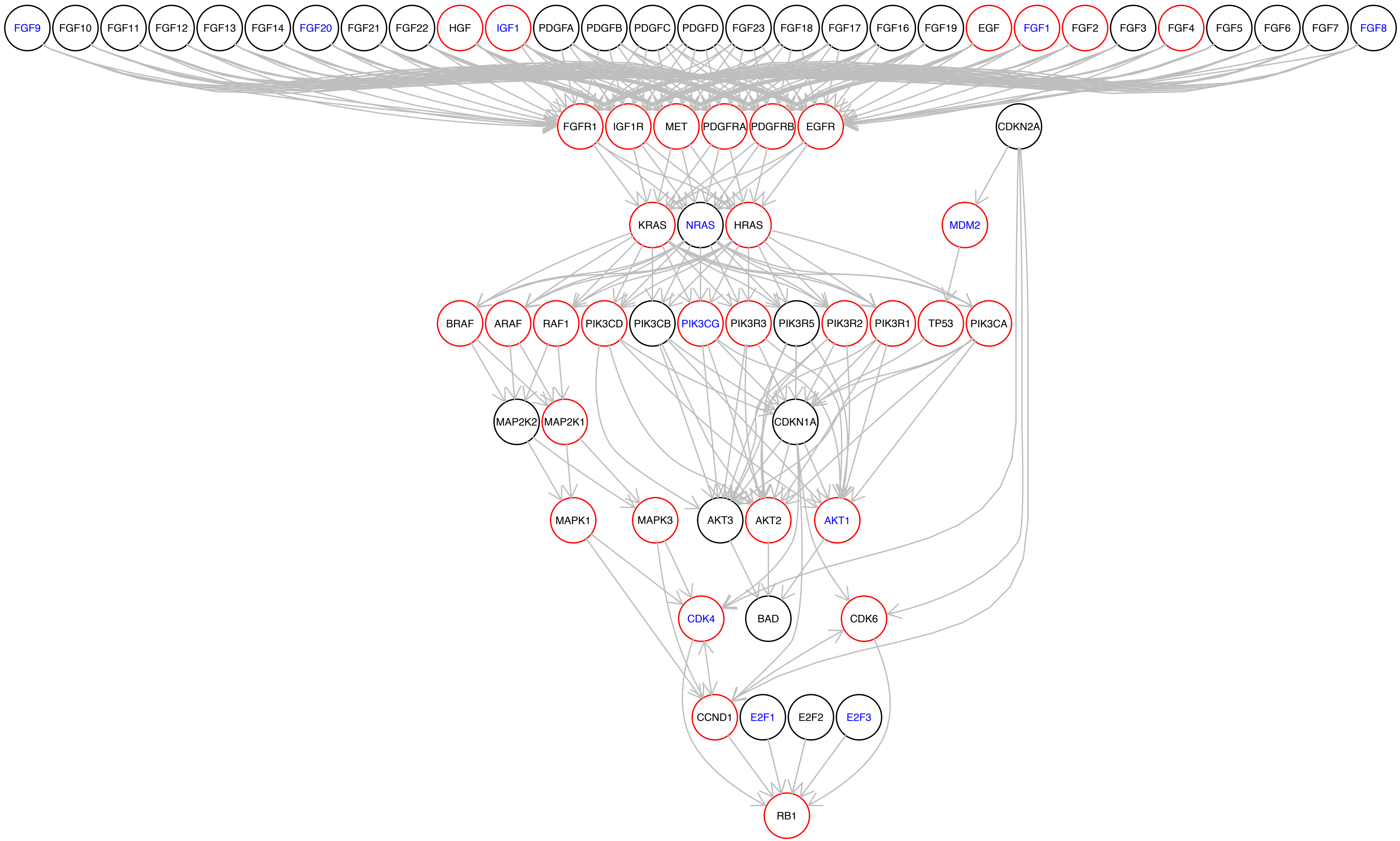

KEGG pathway = Melanoma :   tumour = YUBOT :   Yellow Fill = gene variant, Blue Text = expression−survival association, Red Boarder = drug

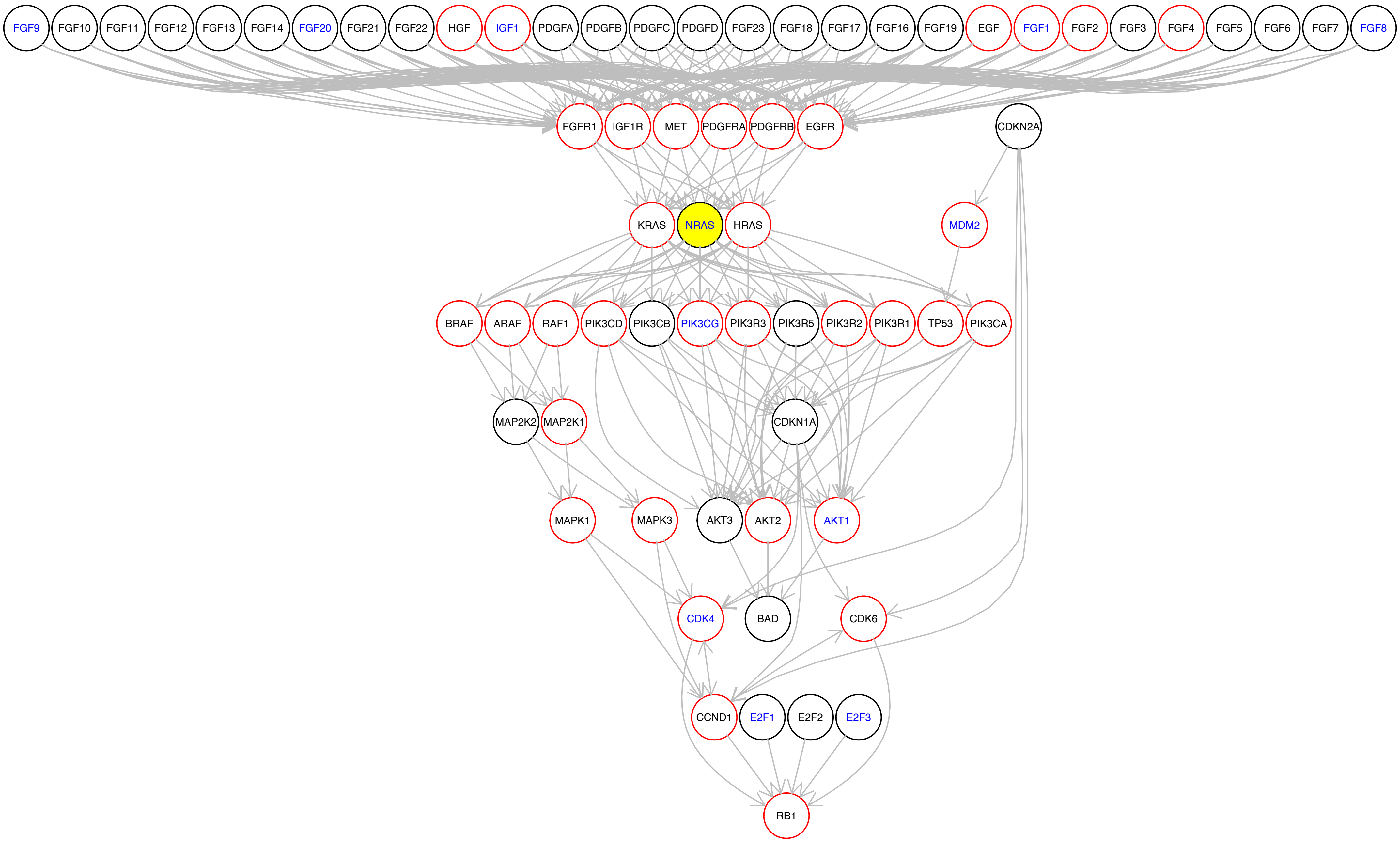

KEGG pathway = Melanoma :   tumour = YUCHIME :   Yellow Fill = gene variant, Blue Text = expression−survival association, Red Boarder = drug

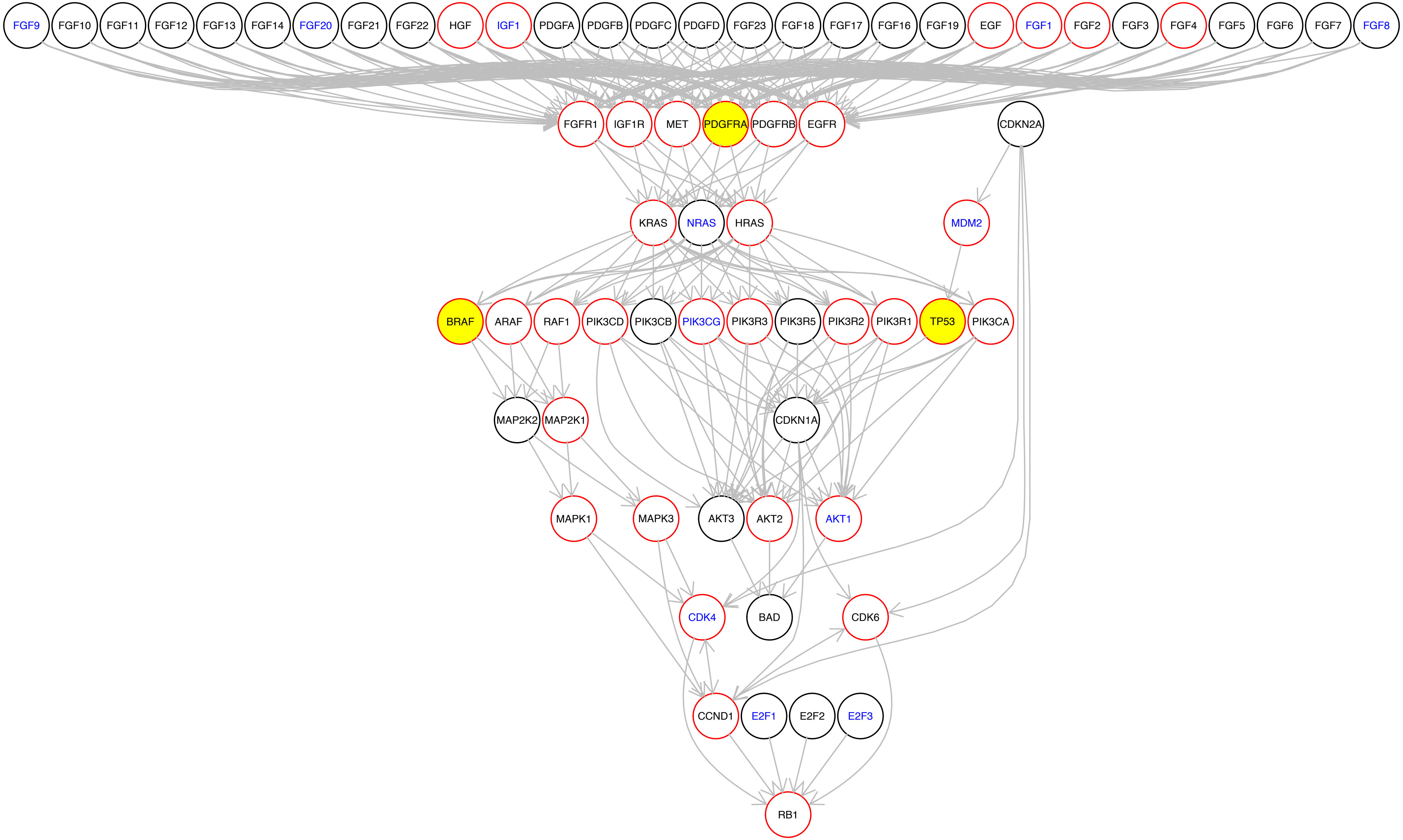

KEGG pathway = Melanoma : tumour = YUCHUFA : Yellow Fill = gene variant, Blue Text = expression–survival association, Red Boarder = drug

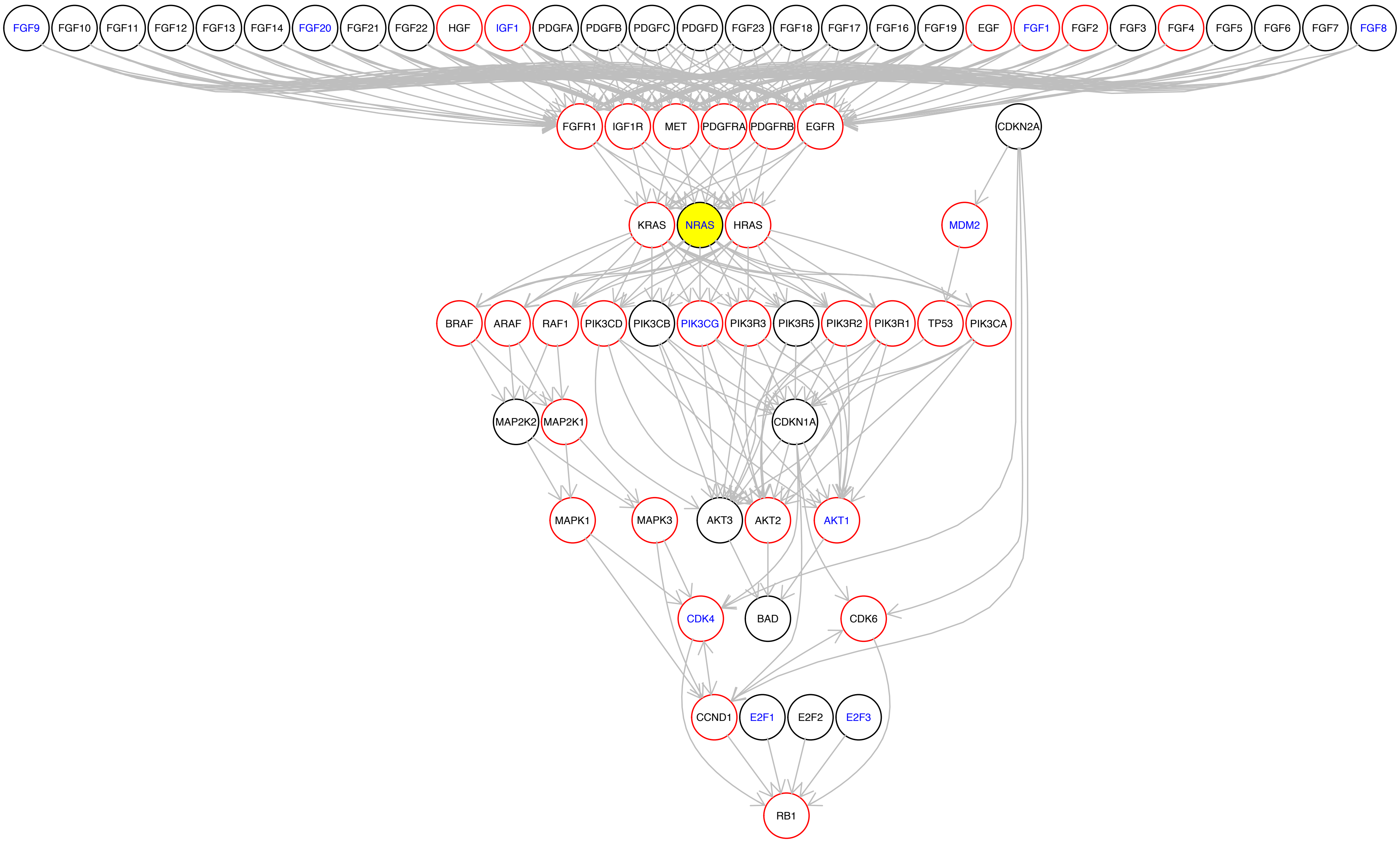

KEGG pathway = Melanoma :   tumour = YUCLAT :   Yellow Fill = gene variant, Blue Text = expression–survival association, Red Boarder = drug

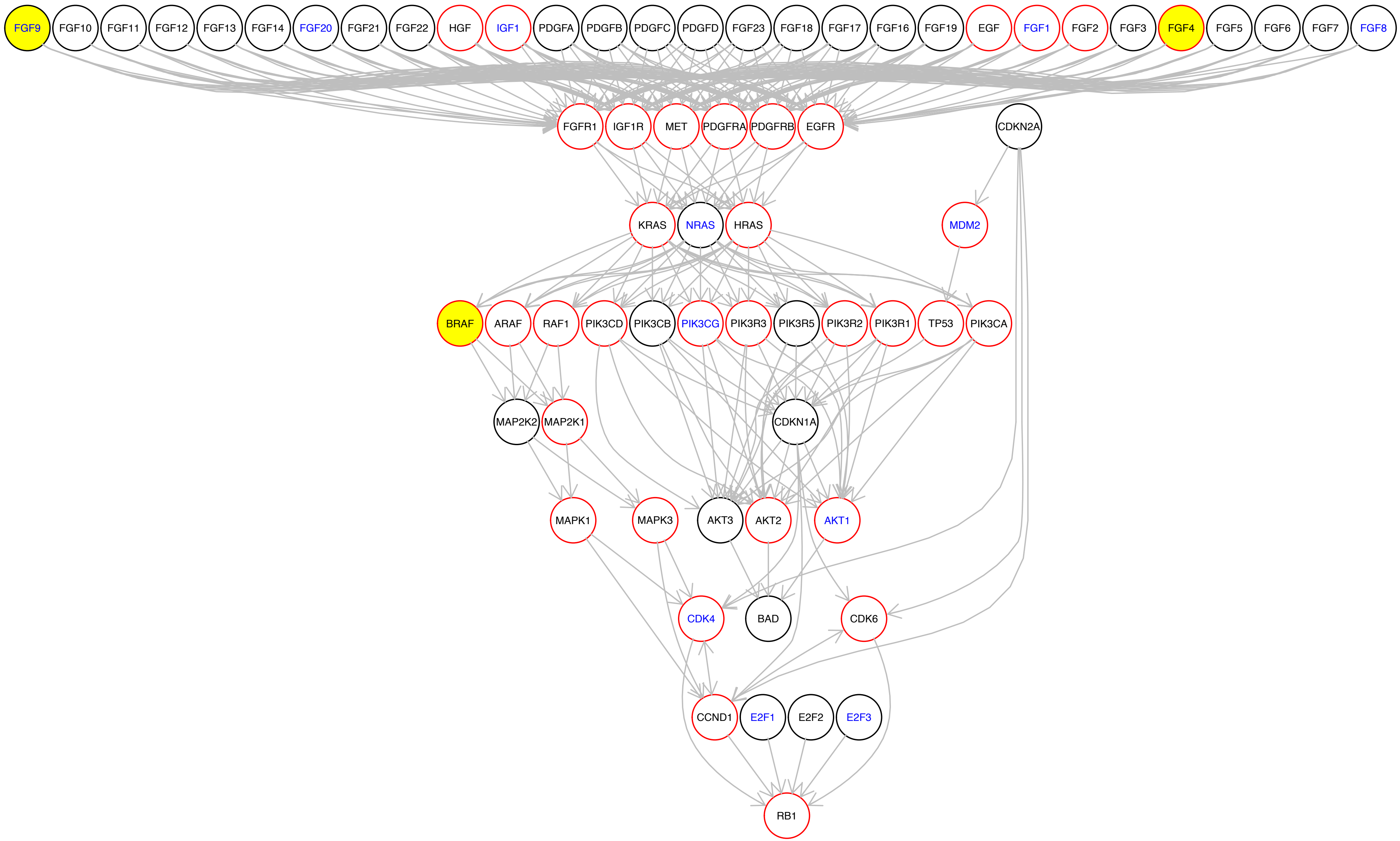

KEGG pathway = Melanoma : tumour = YUCOT : Yellow Fill = gene variant, Blue Text = expression−survival association, Red Boarder = drug

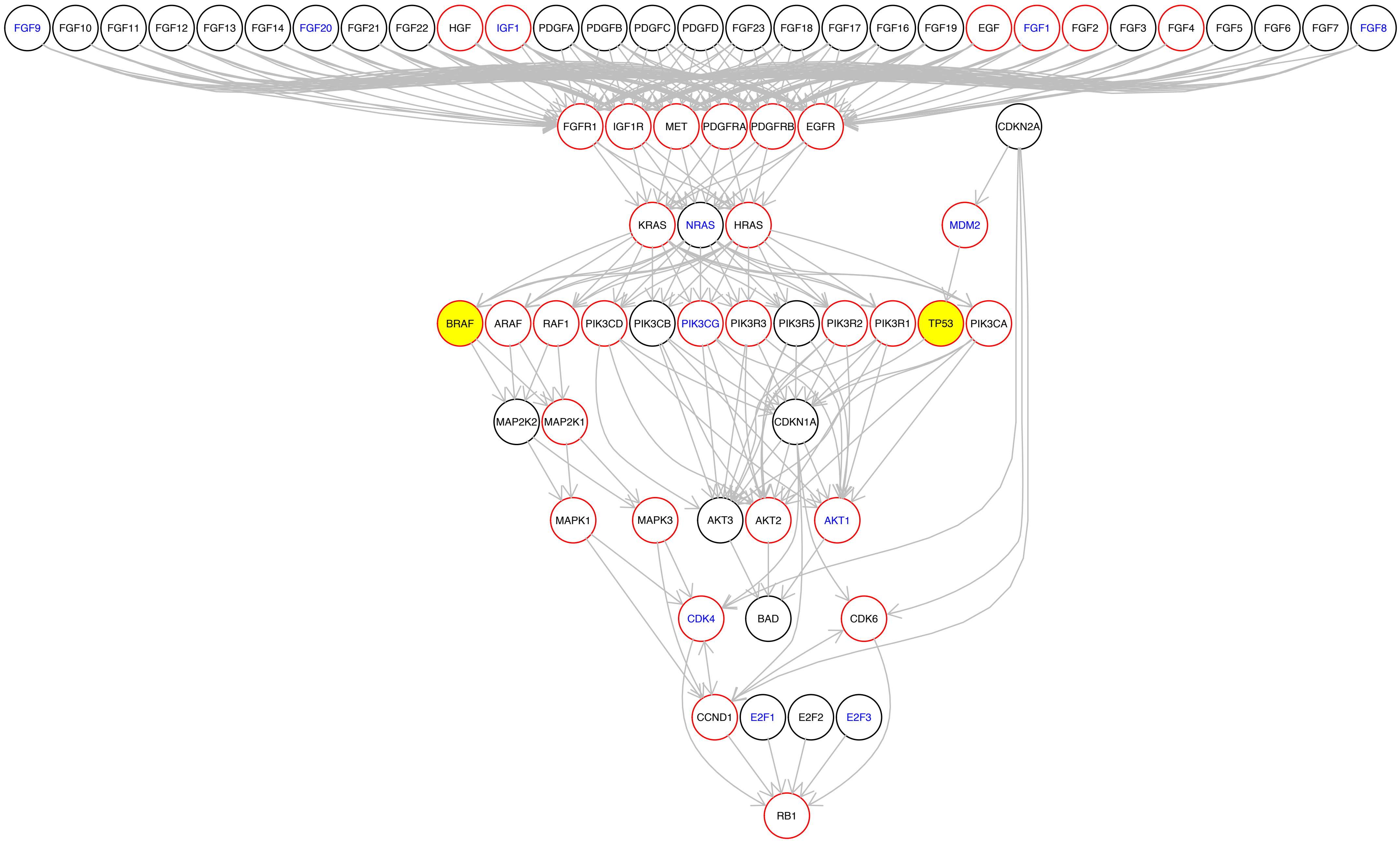

KEGG pathway = Melanoma : tumour = YUCRENA : Yellow Fill = gene variant, Blue Text = expression–survival association, Red Boarder = drug

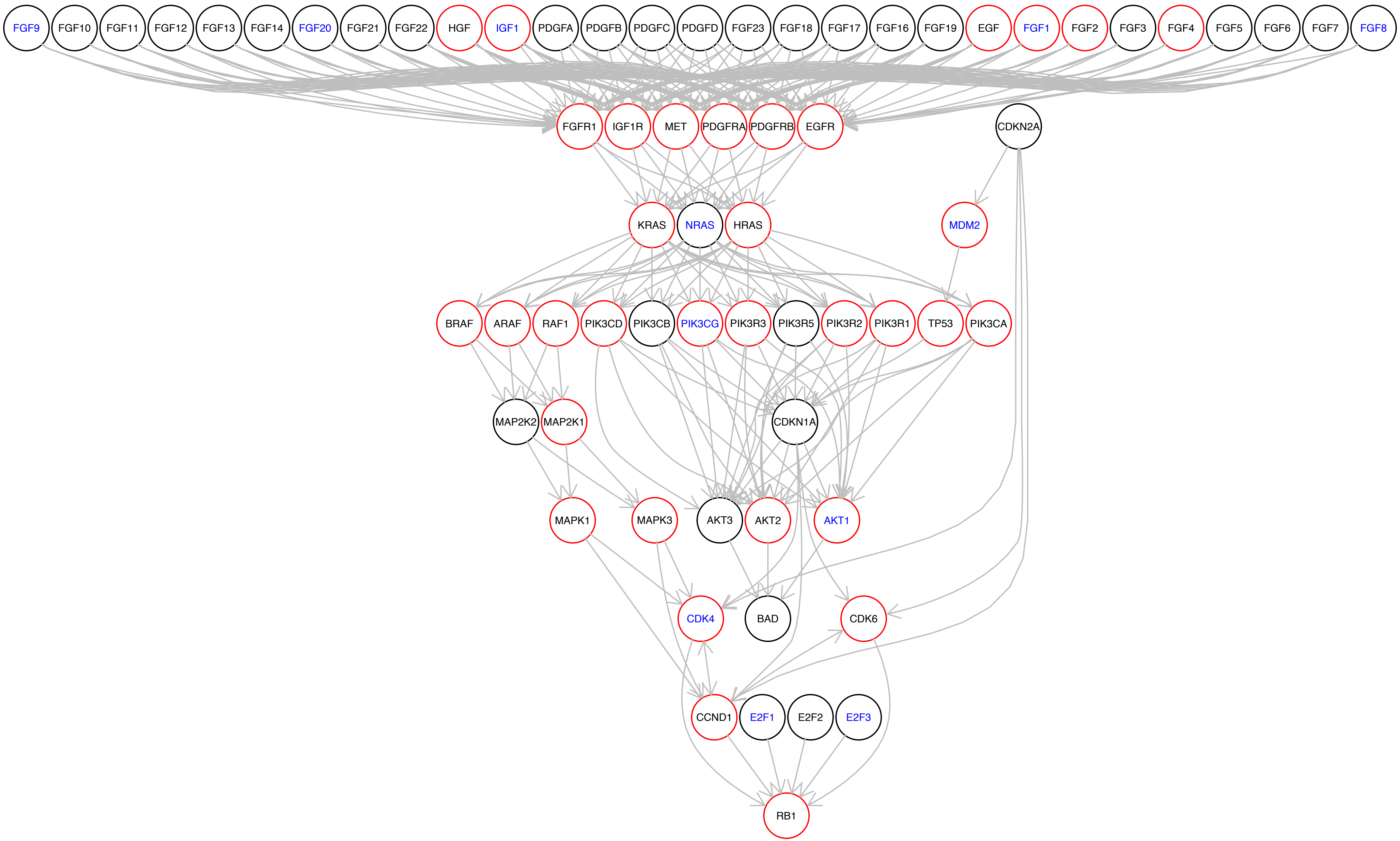

KEGG pathway = Melanoma : tumour = YUCROFT : Yellow Fill = gene variant, Blue Text = expression−survival association, Red Boarder = drug

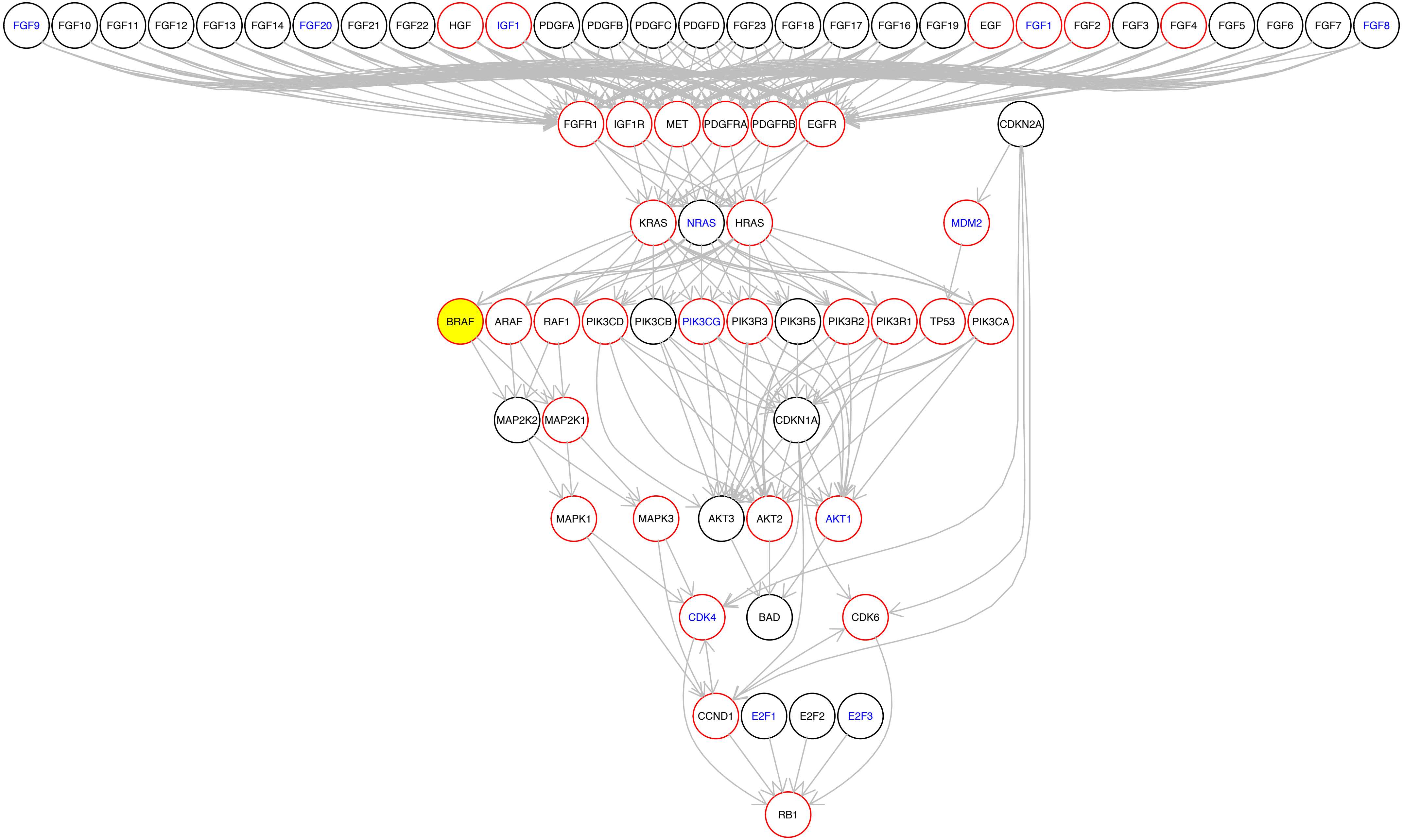

KEGG pathway = Melanoma :   tumour = YUDAB :   Yellow Fill = gene variant, Blue Text = expression−survival association, Red Boarder = drug

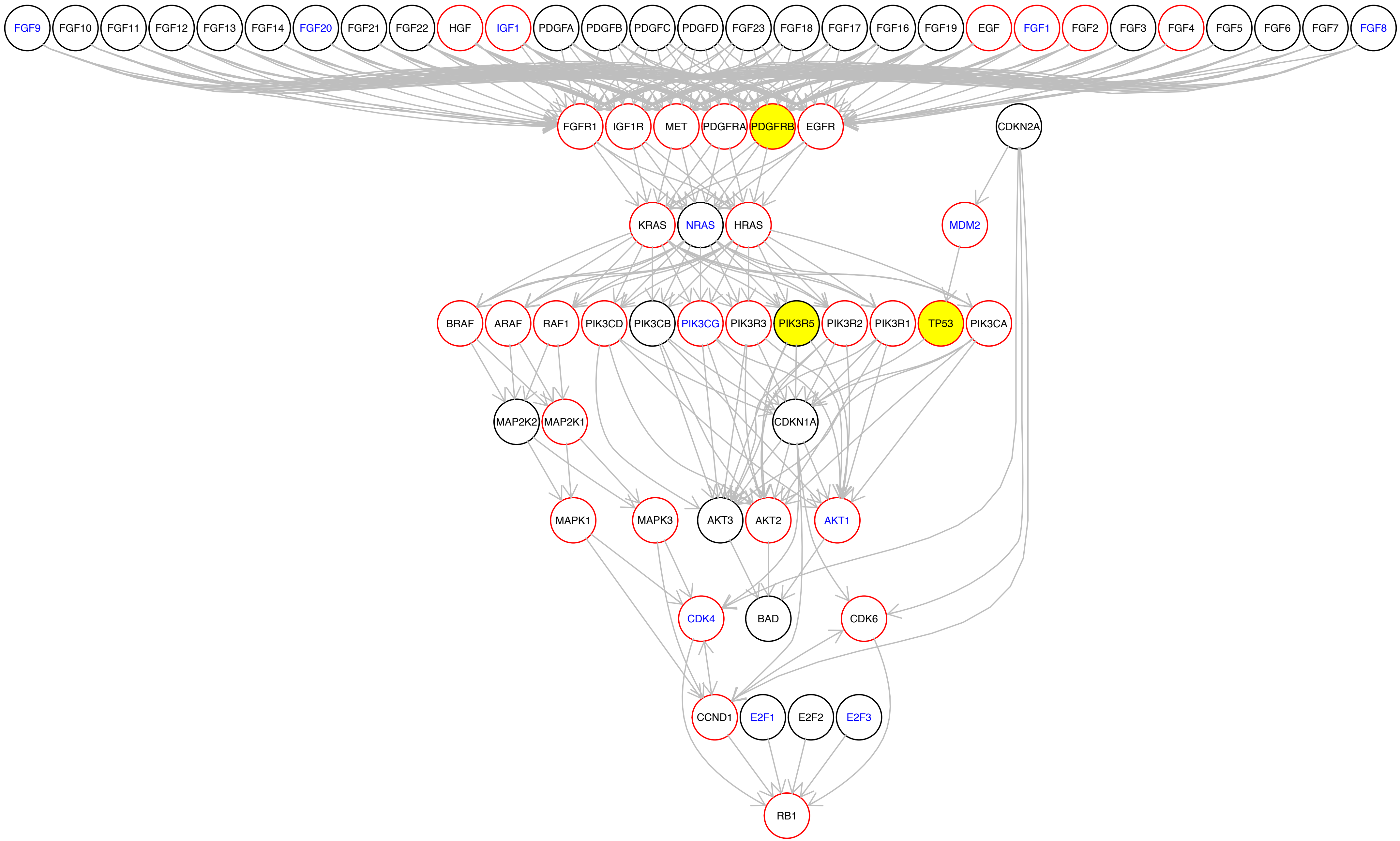

KEGG pathway = Melanoma :   tumour = YUDARE :   Yellow Fill = gene variant, Blue Text = expression−survival association, Red Boarder = drug

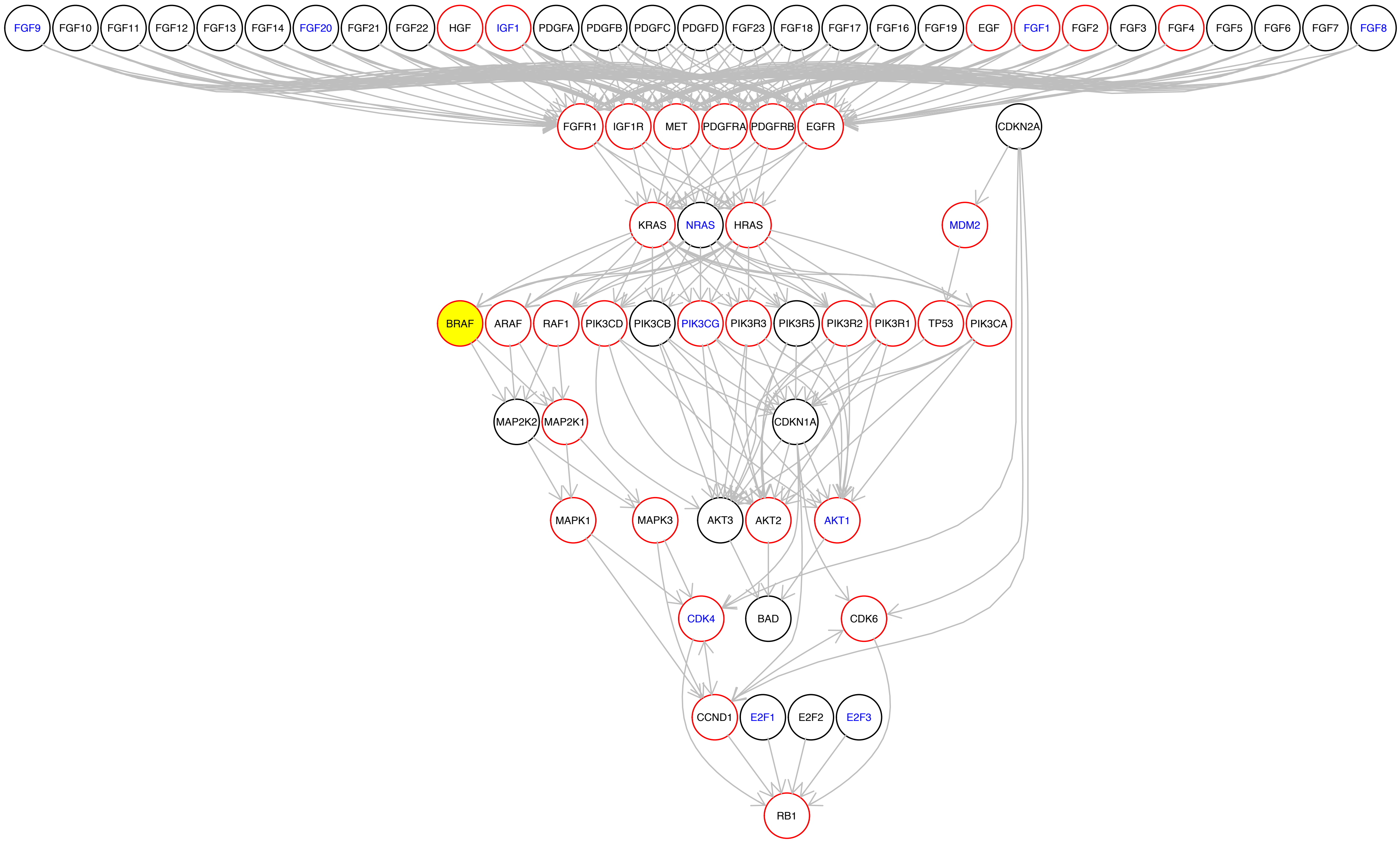

KEGG pathway = Melanoma :   tumour = YUDATE :   Yellow Fill = gene variant, Blue Text = expression−survival association, Red Boarder = drug

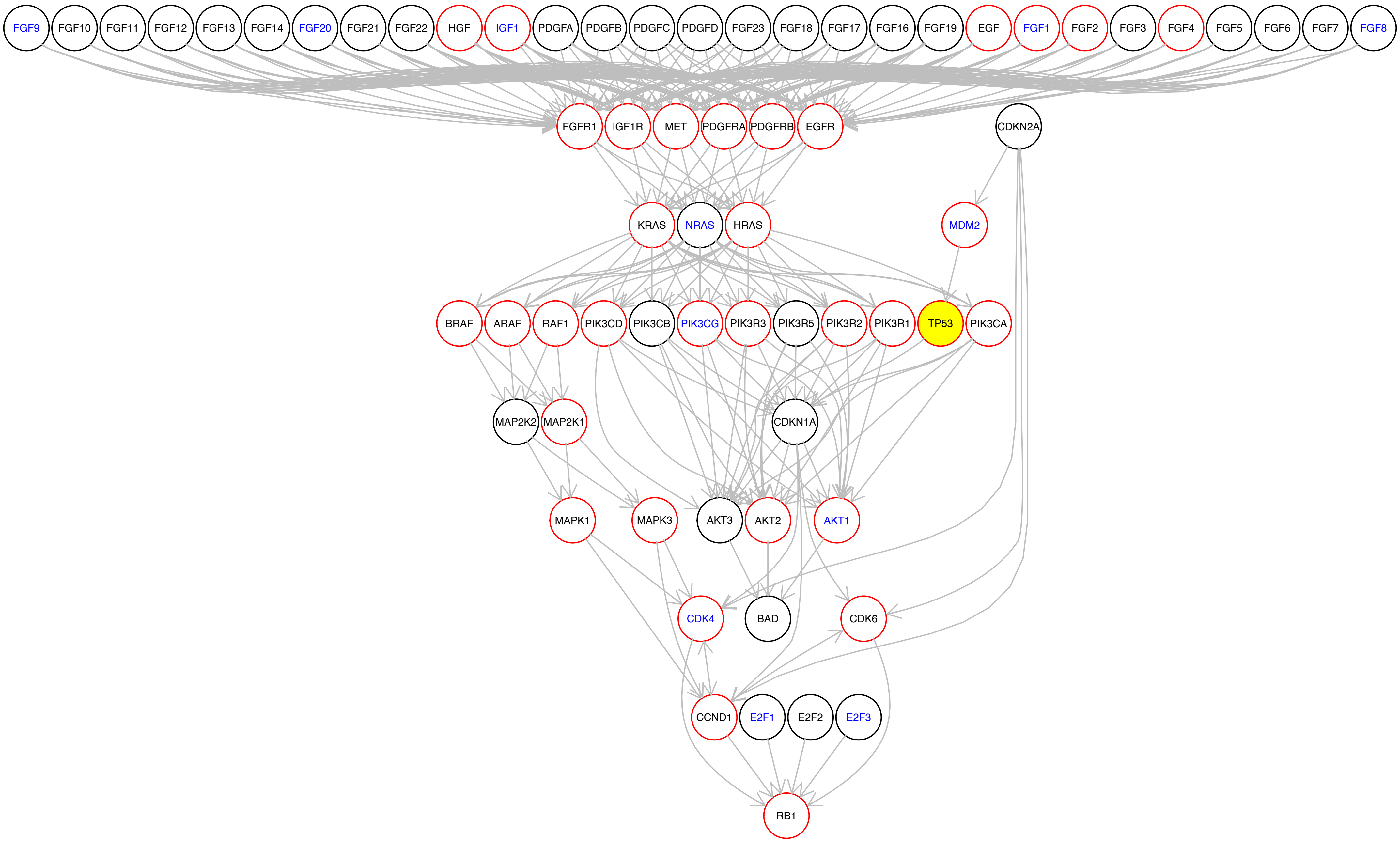

KEGG pathway = Melanoma : tumour = YUDEDE : Yellow Fill = gene variant, Blue Text = expression−survival association, Red Boarder = drug

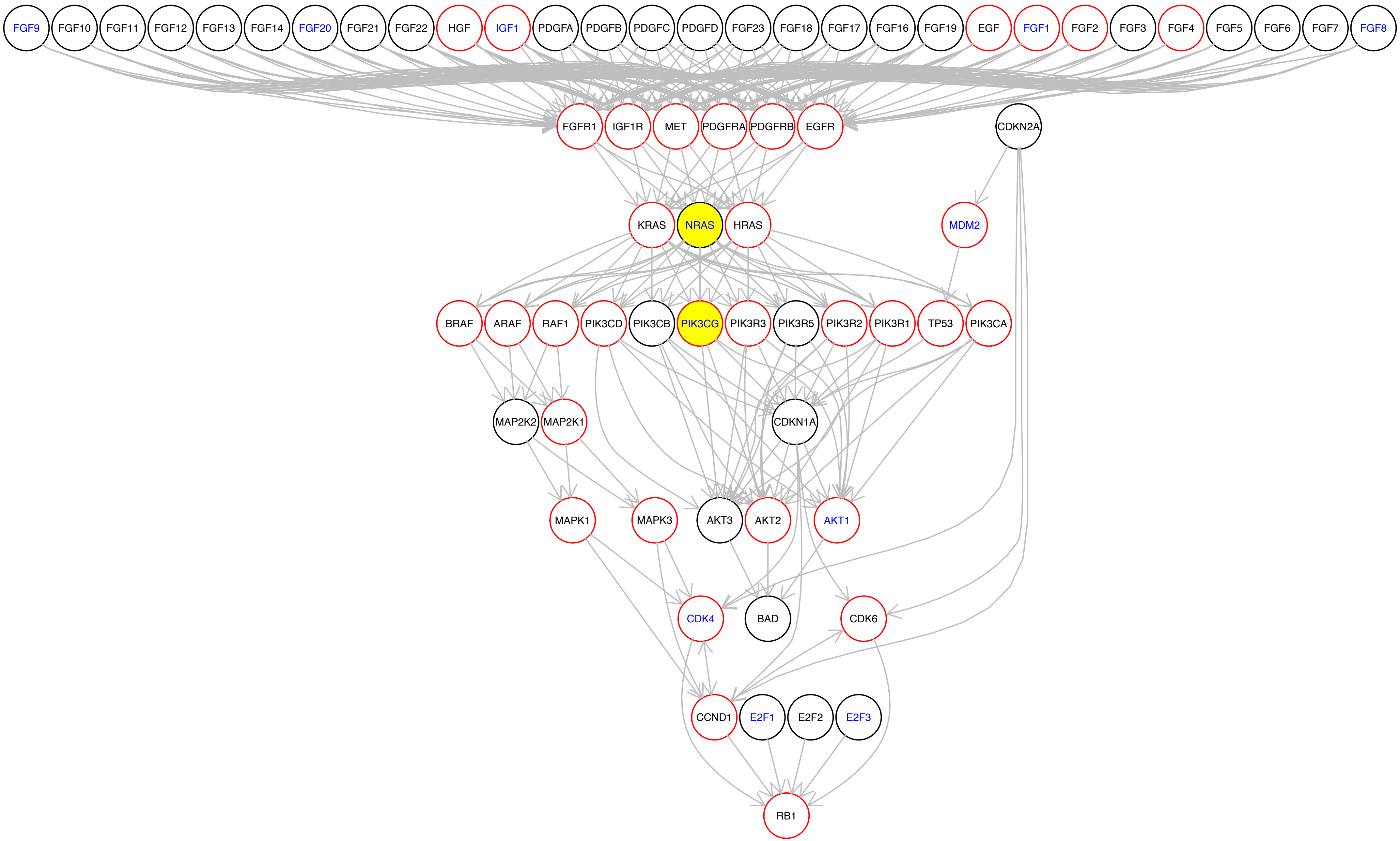

KEGG pathway = Melanoma : tumour = YUDEXA : Yellow Fill = gene variant, Blue Text = expression−survival association, Red Boarder = drug

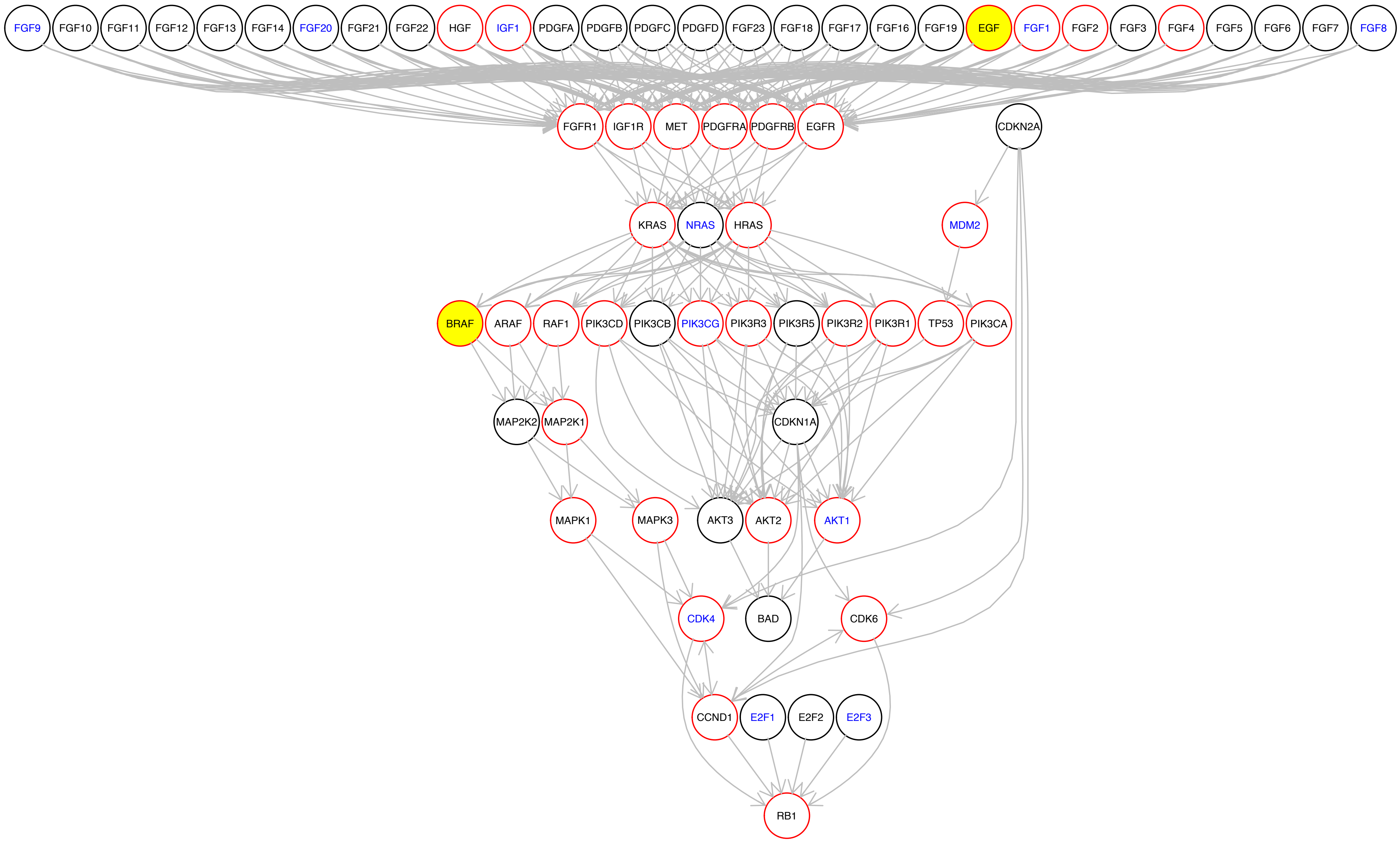

KEGG pathway = Melanoma :   tumour = YUDIALE :   Yellow Fill = gene variant, Blue Text = expression−survival association, Red Boarder = drug

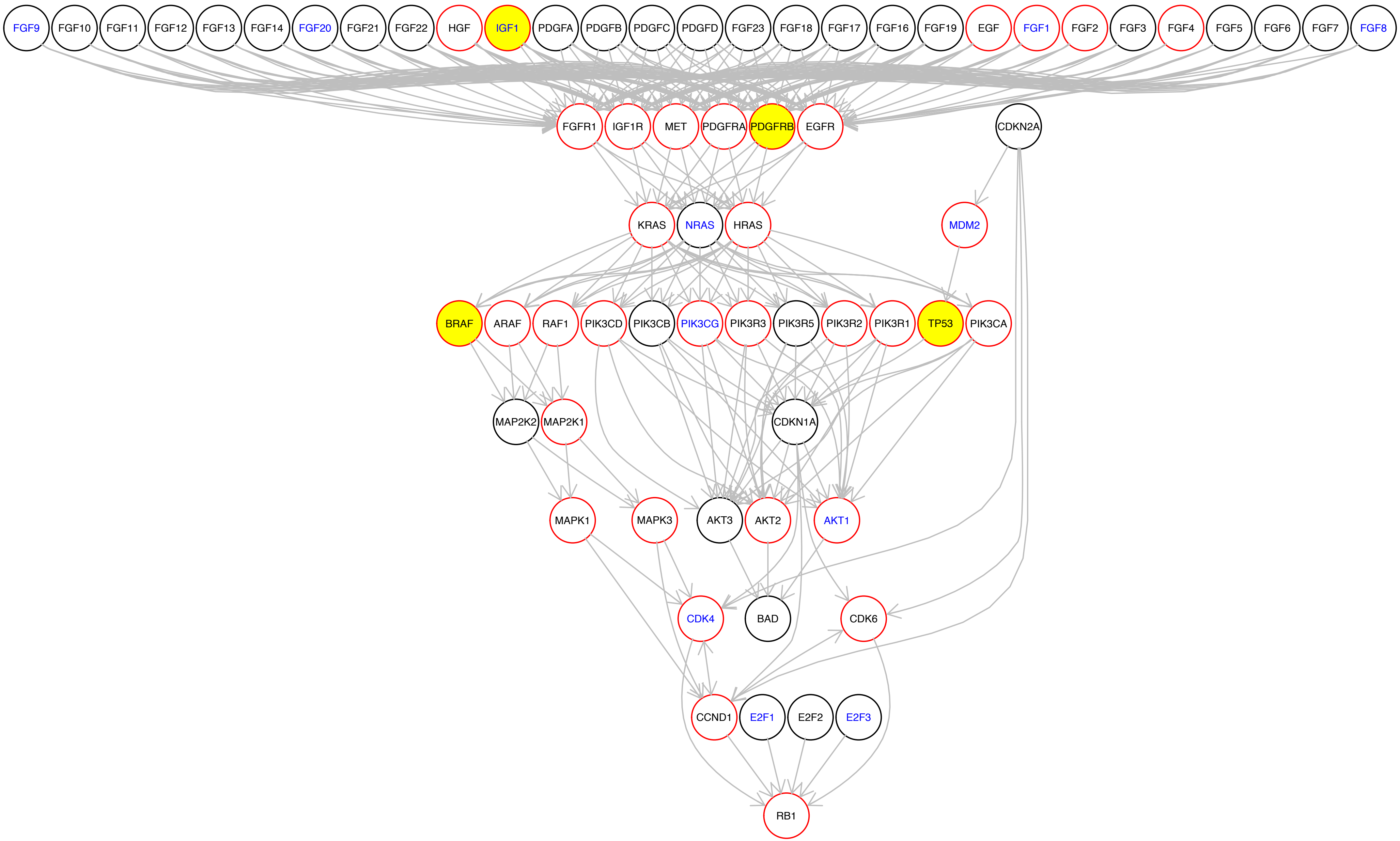

KEGG pathway = Melanoma : tumour = YUDUTY : Yellow Fill = gene variant, Blue Text = expression−survival association, Red Boarder = drug

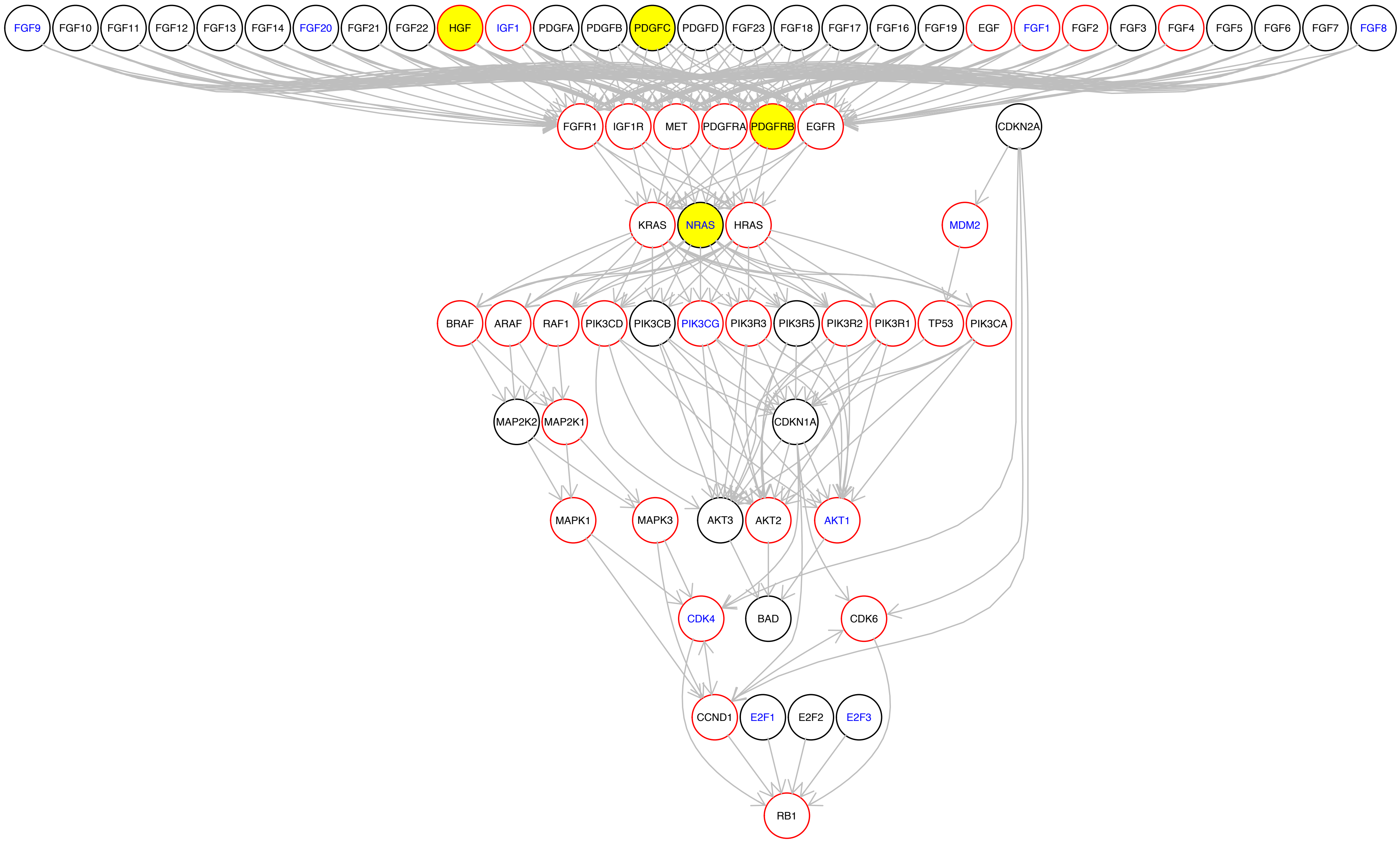

KEGG pathway = Melanoma : tumour = YUFAR : Yellow Fill = gene variant, Blue Text = expression−survival association, Red Boarder = drug

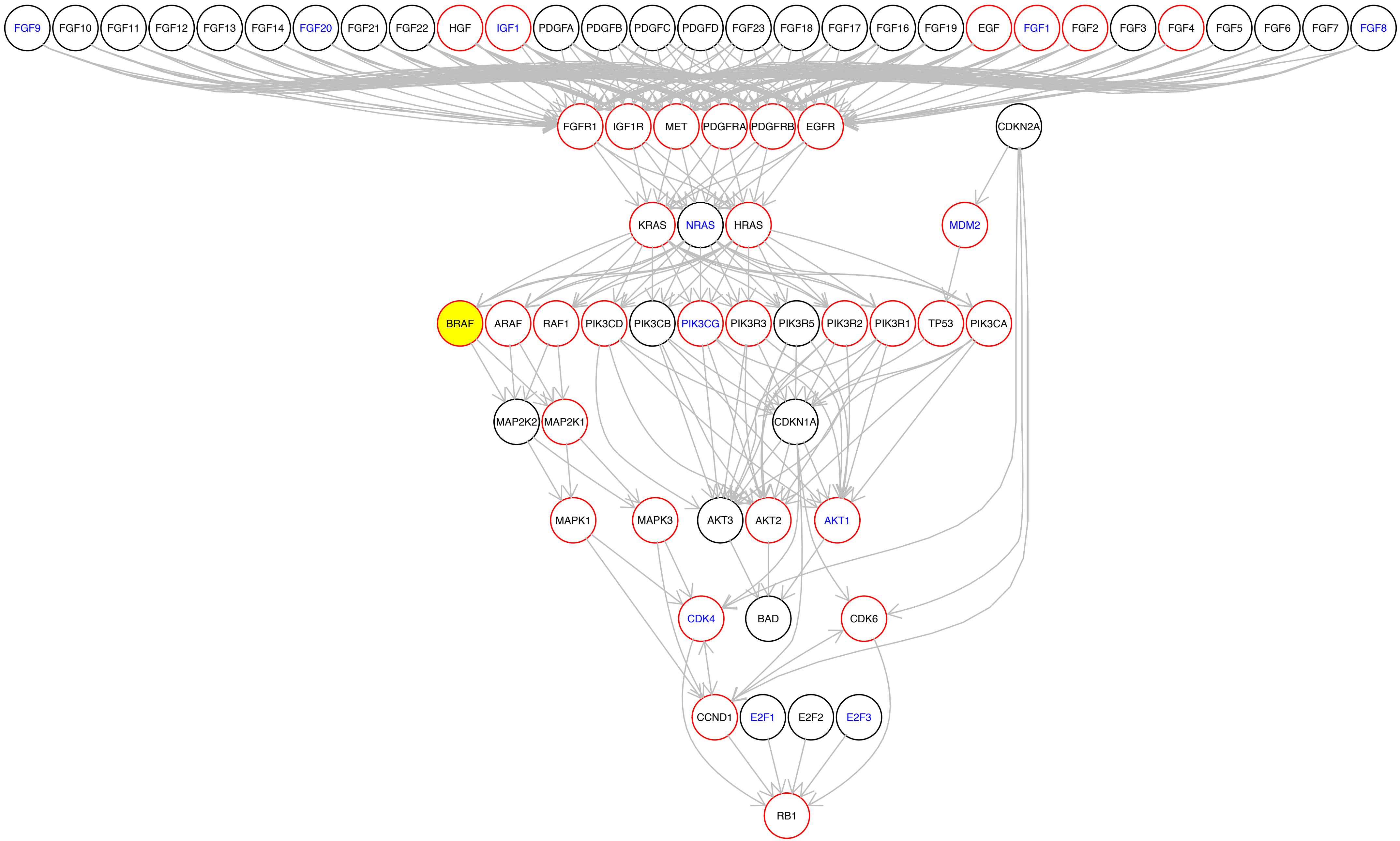

KEGG pathway = Melanoma :   tumour = YUFARCI :   Yellow Fill = gene variant, Blue Text = expression–survival association, Red Boarder = drug

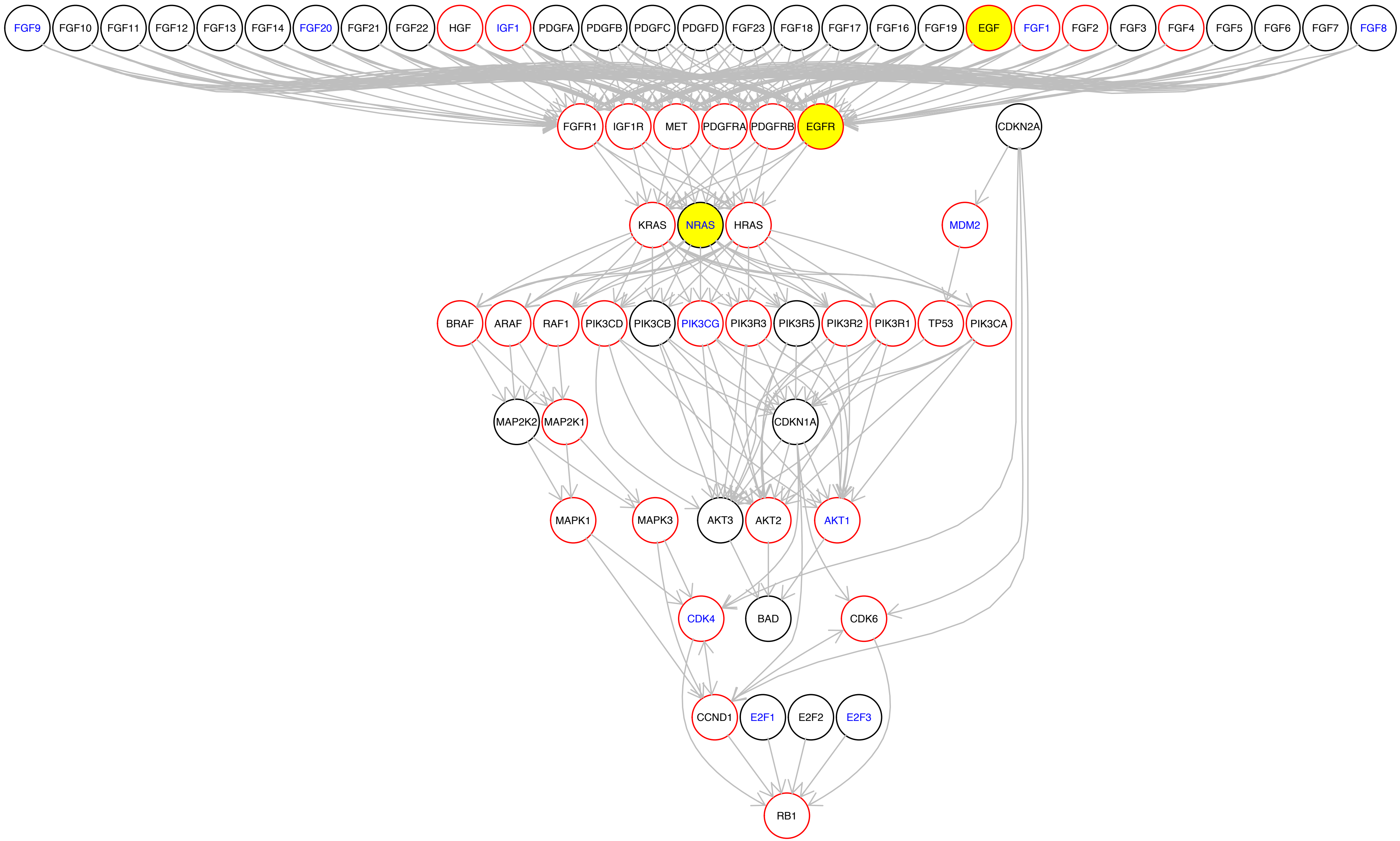

KEGG pathway = Melanoma :   tumour = YUFLA :   Yellow Fill = gene variant, Blue Text = expression−survival association, Red Boarder = drug

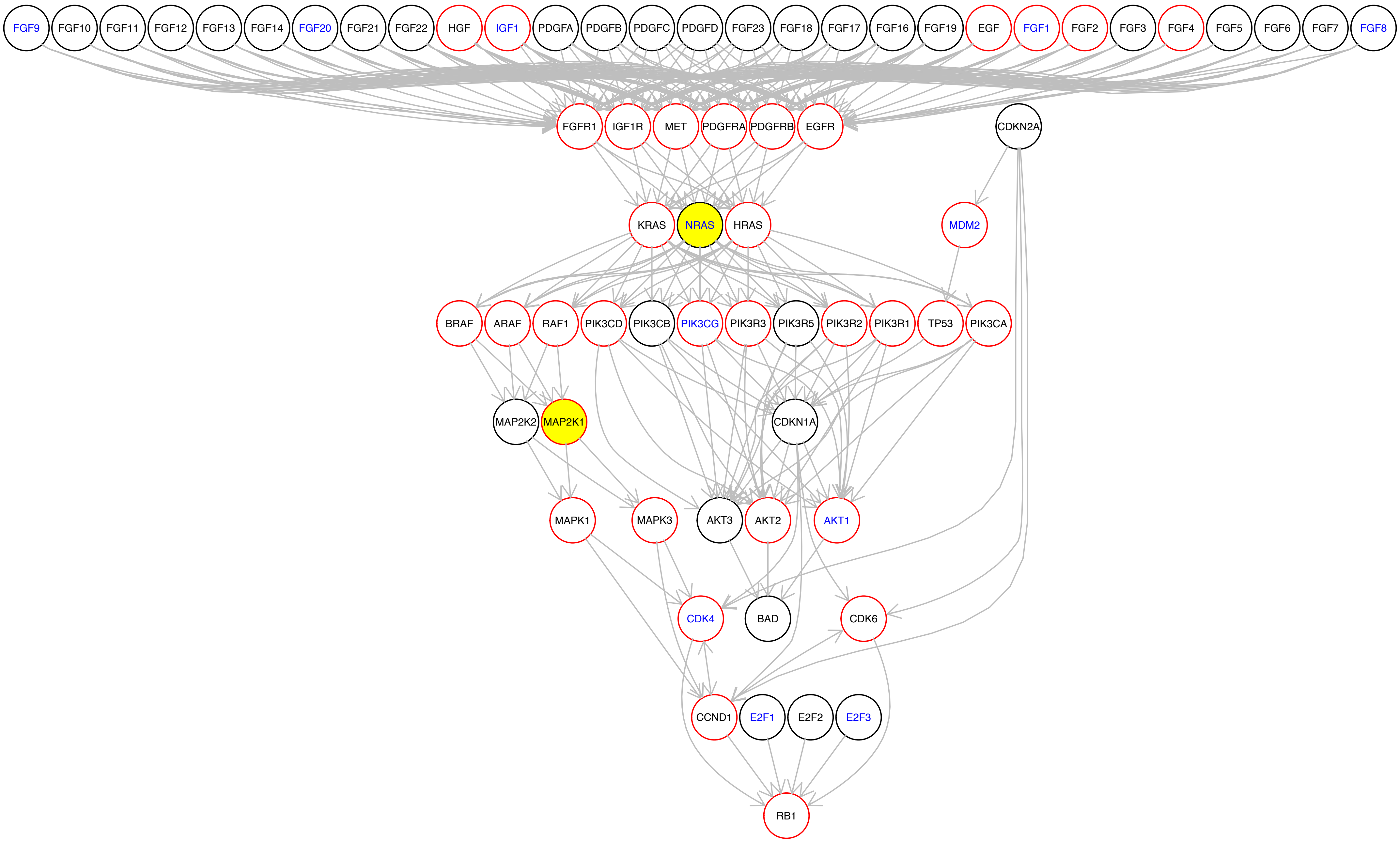

KEGG pathway = Melanoma : tumour = YUFOLD : Yellow Fill = gene variant, Blue Text = expression−survival association, Red Boarder = drug

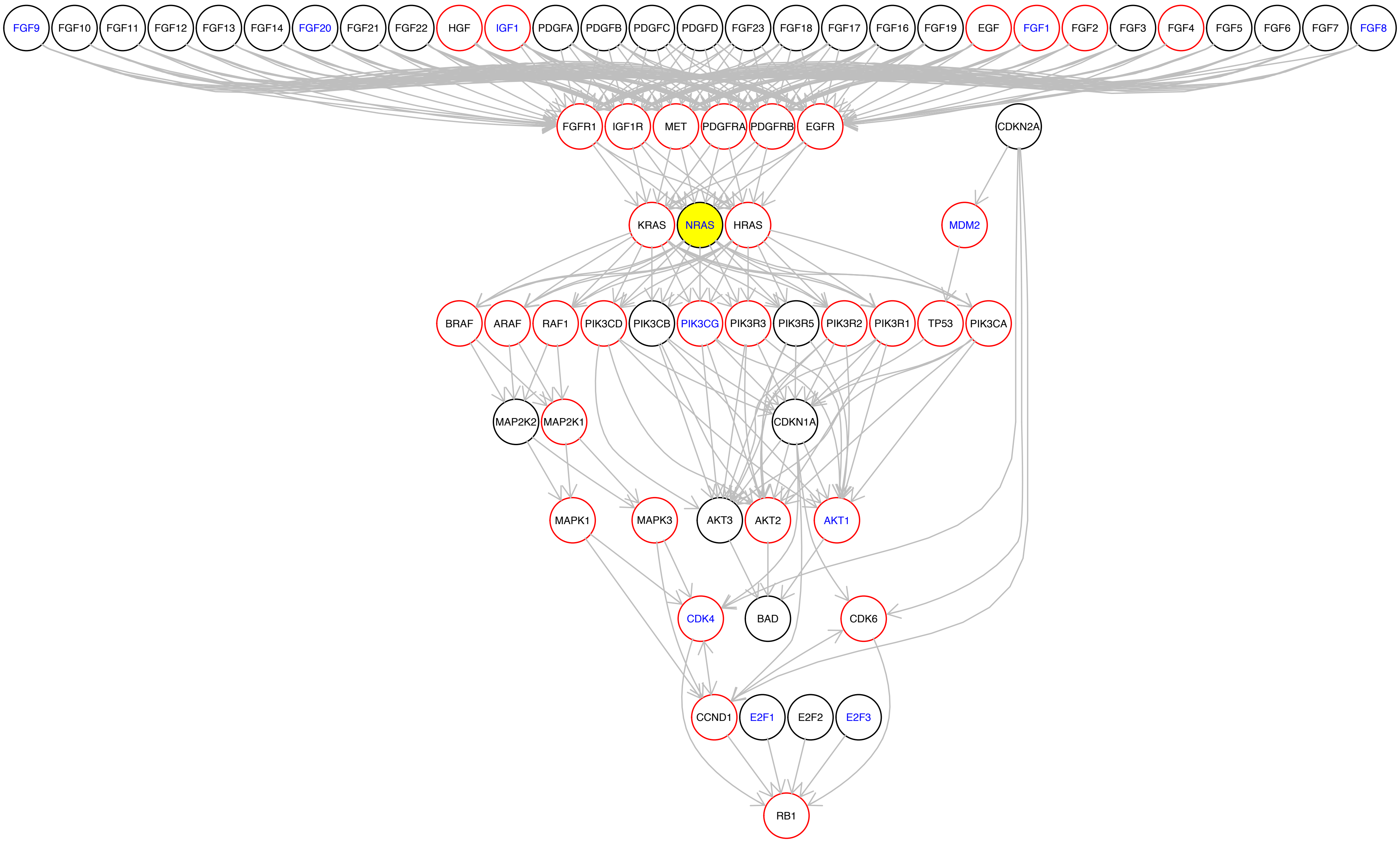

KEGG pathway = Melanoma : tumour = YUFOR : Yellow Fill = gene variant, Blue Text = expression−survival association, Red Boarder = drug

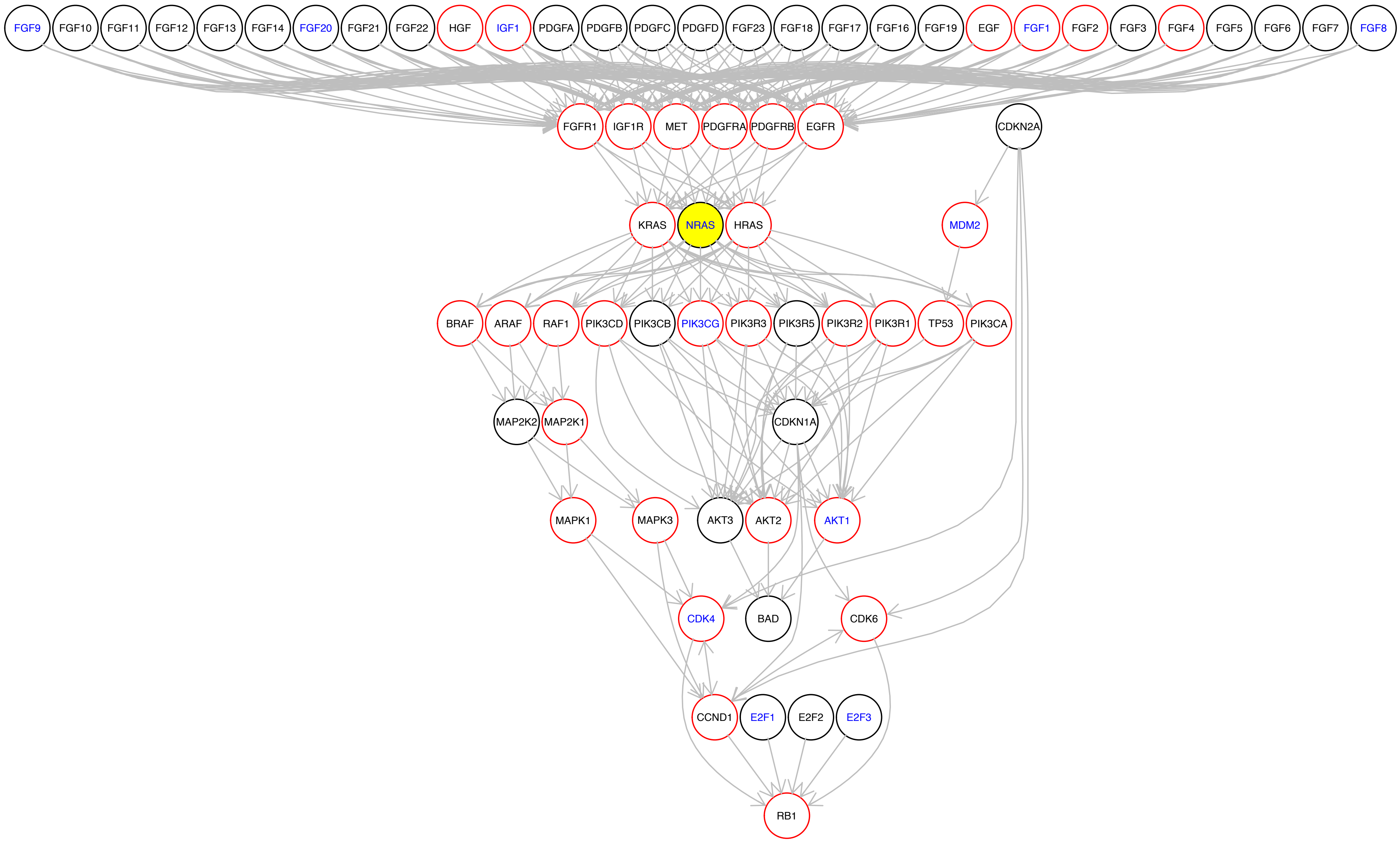

KEGG pathway = Melanoma : tumour = YUGAFFE : Yellow Fill = gene variant, Blue Text = expression-survival association, Red Boarder = drug

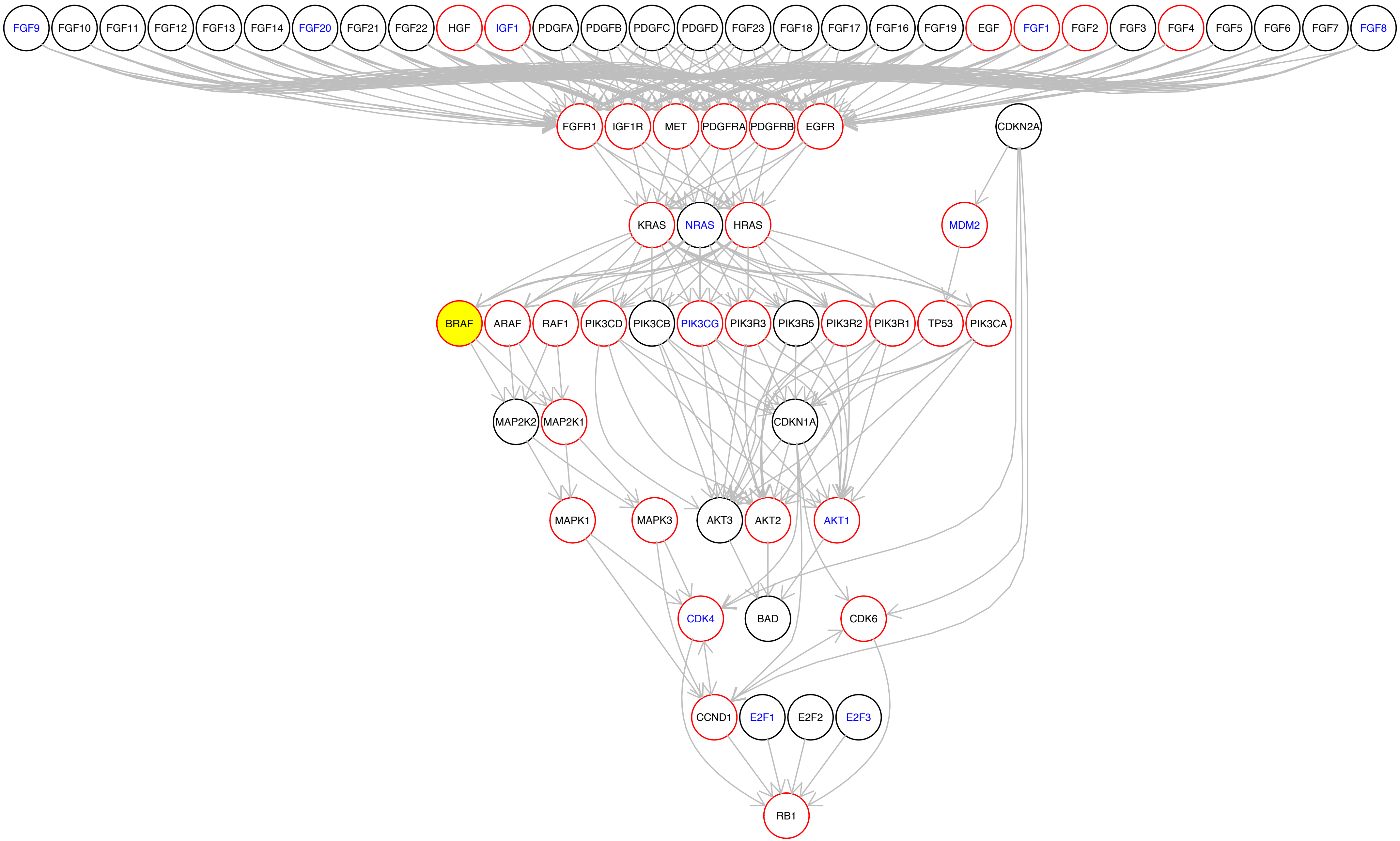

KEGG pathway = Melanoma : tumour = YUGANK : Yellow Fill = gene variant, Blue Text = expression−survival association, Red Boarder = drug

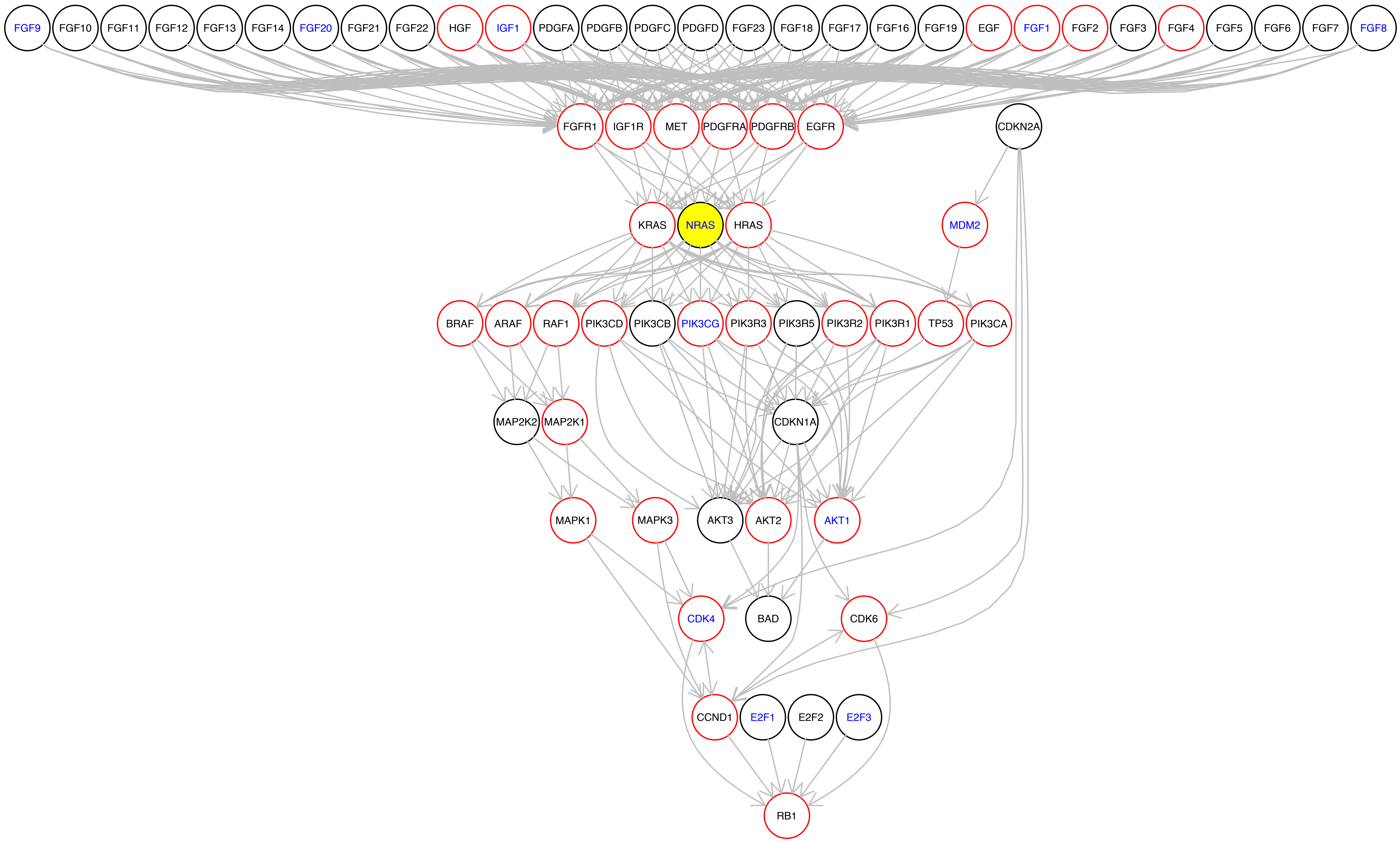

KEGG pathway = Melanoma : tumour = YUGASP : Yellow Fill = gene variant, Blue Text = expression−survival association, Red Boarder = drug

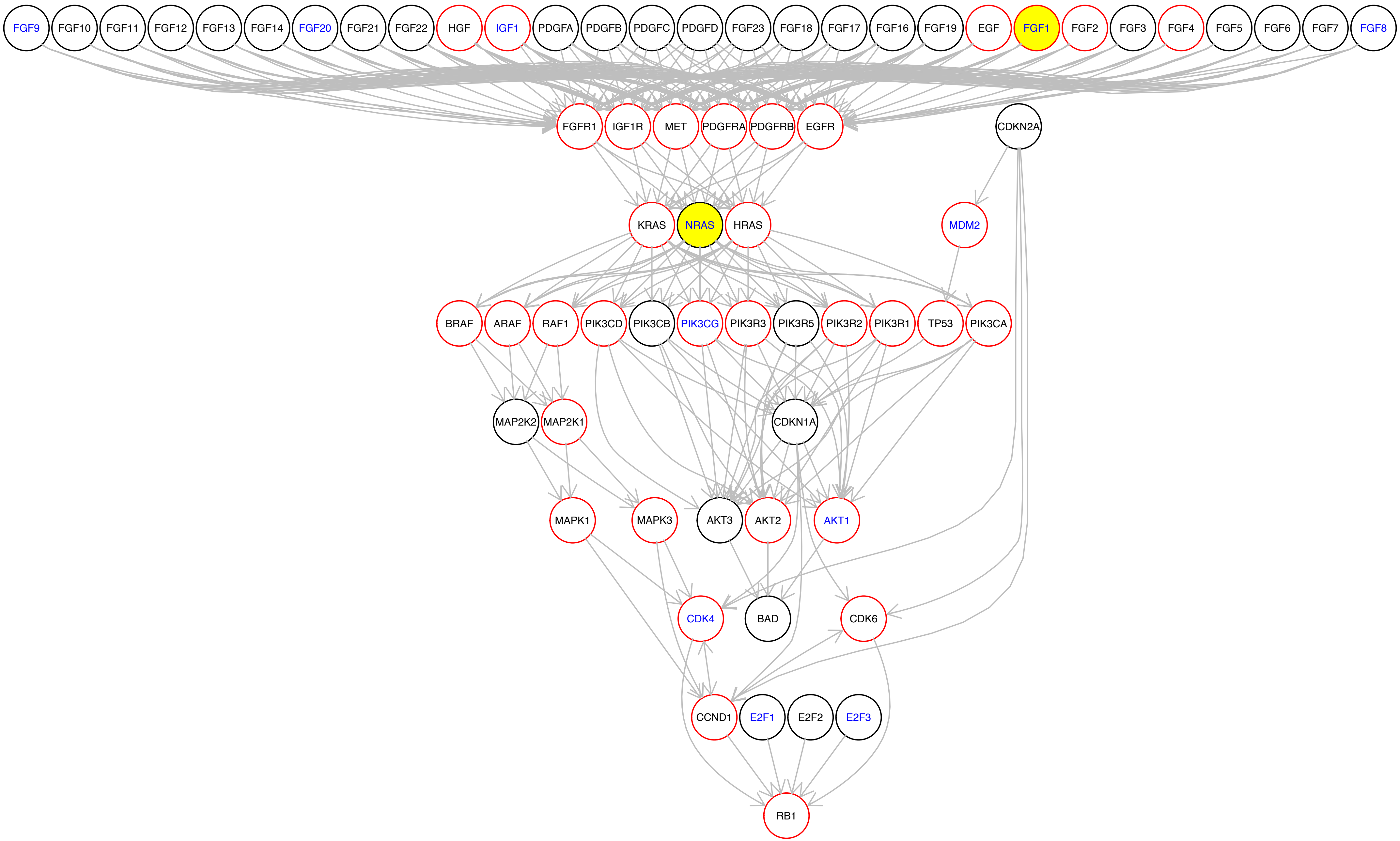

KEGG pathway = Melanoma : tumour = YUGISMO : Yellow Fill = gene variant, Blue Text = expression−survival association, Red Boarder = drug

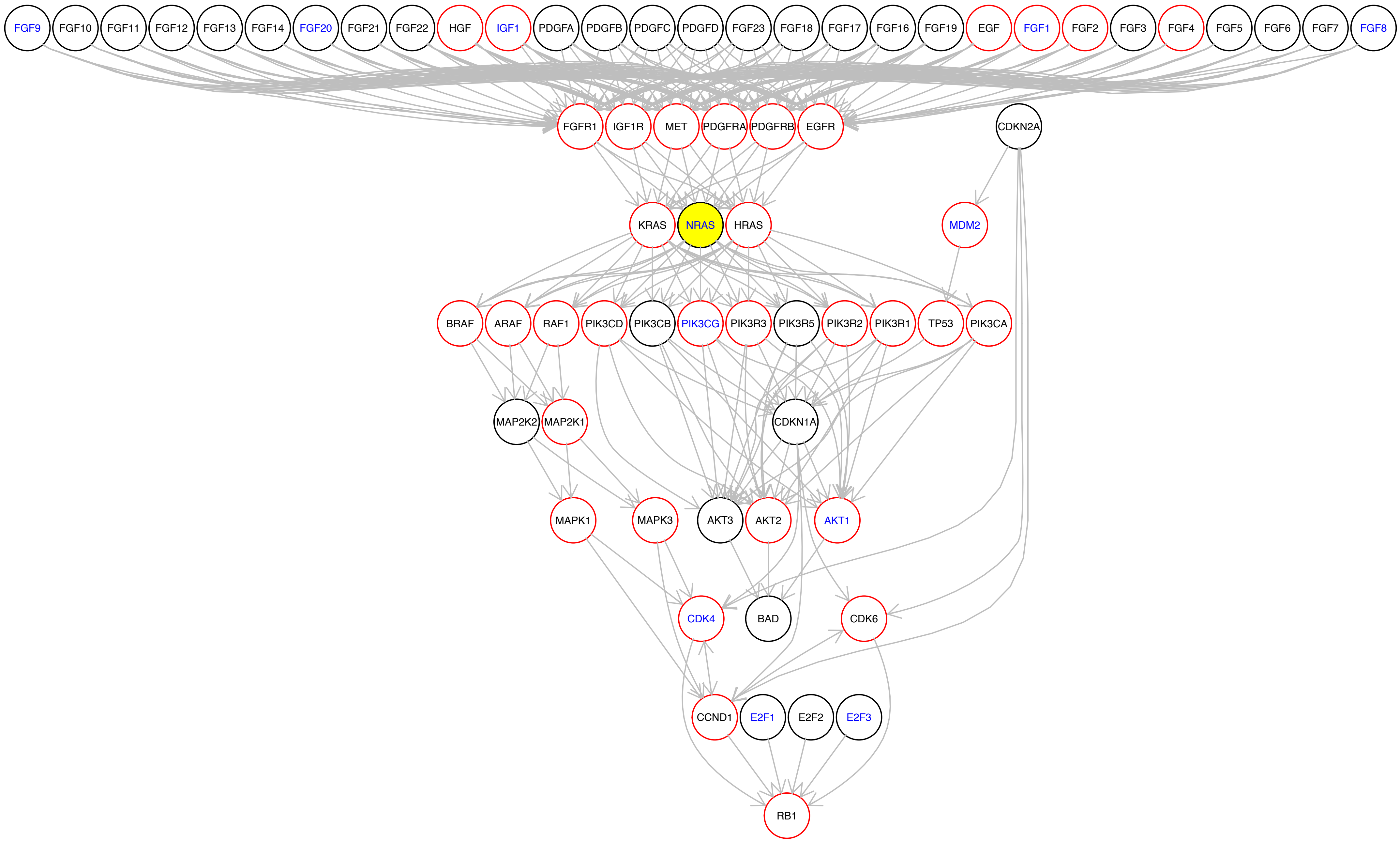

KEGG pathway = Melanoma : tumour = YUGOE : Yellow Fill = gene variant, Blue Text = expression−survival association, Red Boarder = drug

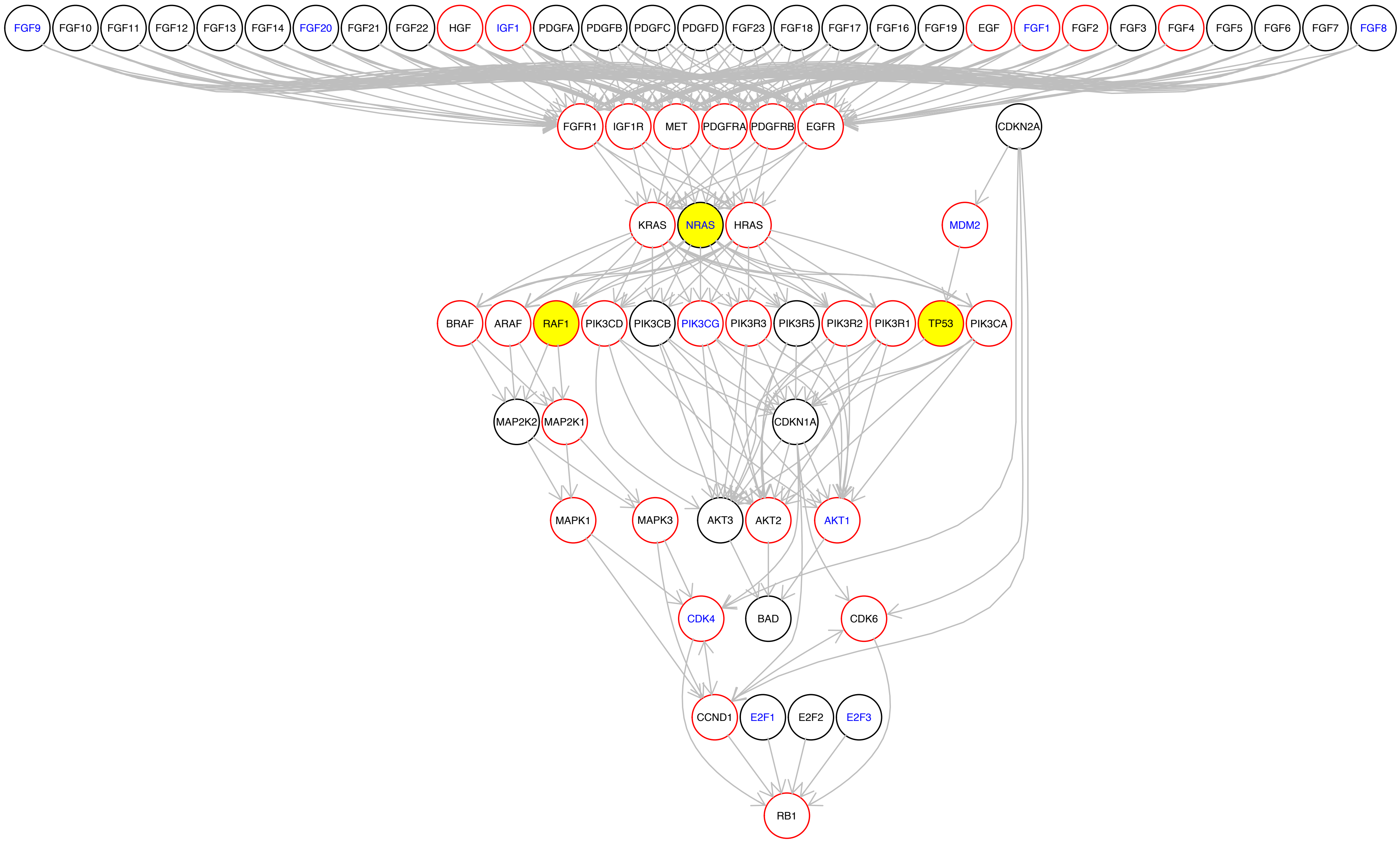

KEGG pathway = Melanoma : tumour = YUHEF : Yellow Fill = gene variant, Blue Text = expression–survival association, Red Boarder = drug

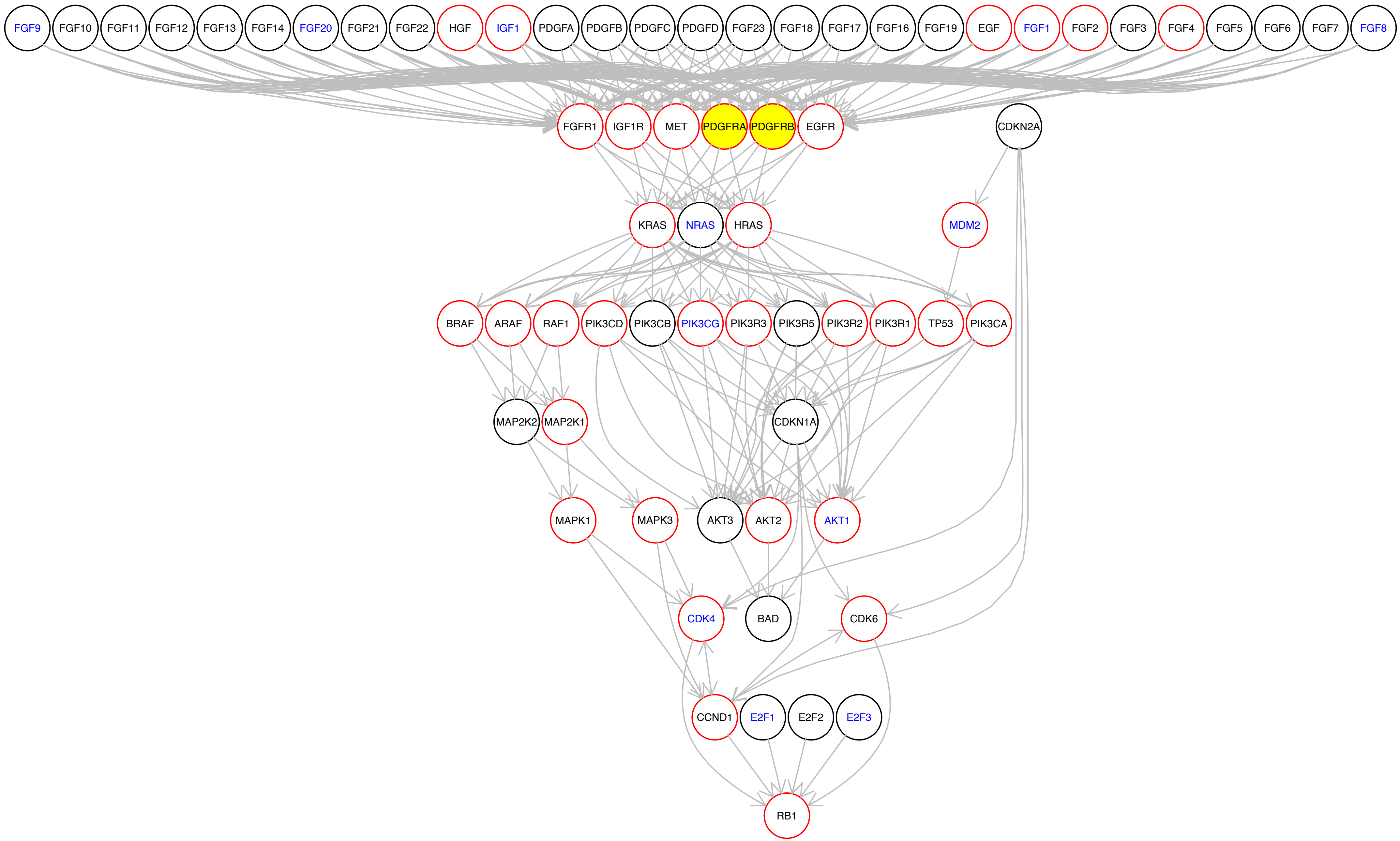

KEGG pathway = Melanoma : tumour = YUHOIN : Yellow Fill = gene variant, Blue Text = expression−survival association, Red Boarder = drug

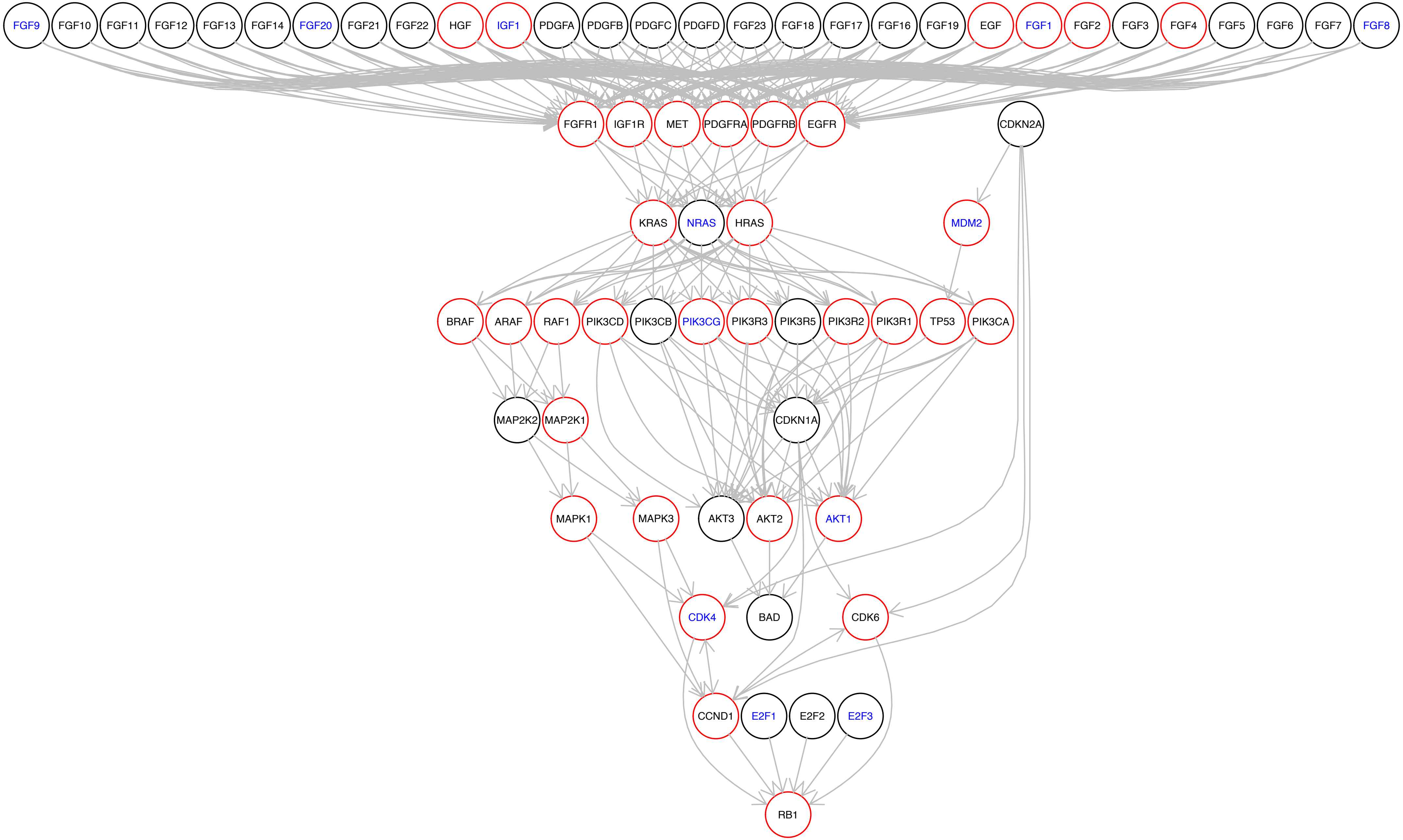

KEGG pathway = Melanoma : tumour = YUHOOD : Yellow Fill = gene variant, Blue Text = expression−survival association, Red Boarder = drug

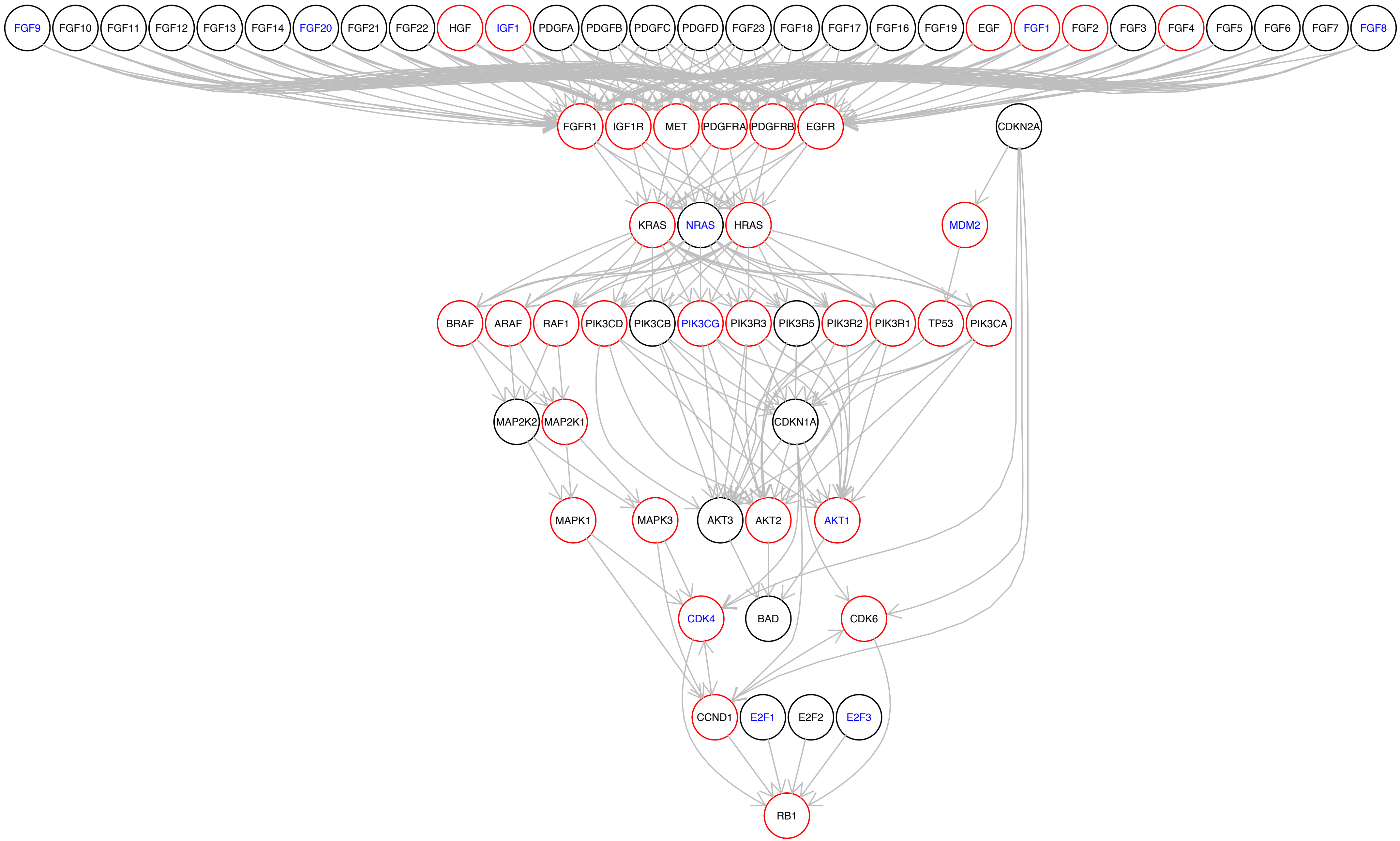

KEGG pathway = Melanoma : tumour = YUISKIA : Yellow Fill = gene variant, Blue Text = expression−survival association, Red Boarder = drug

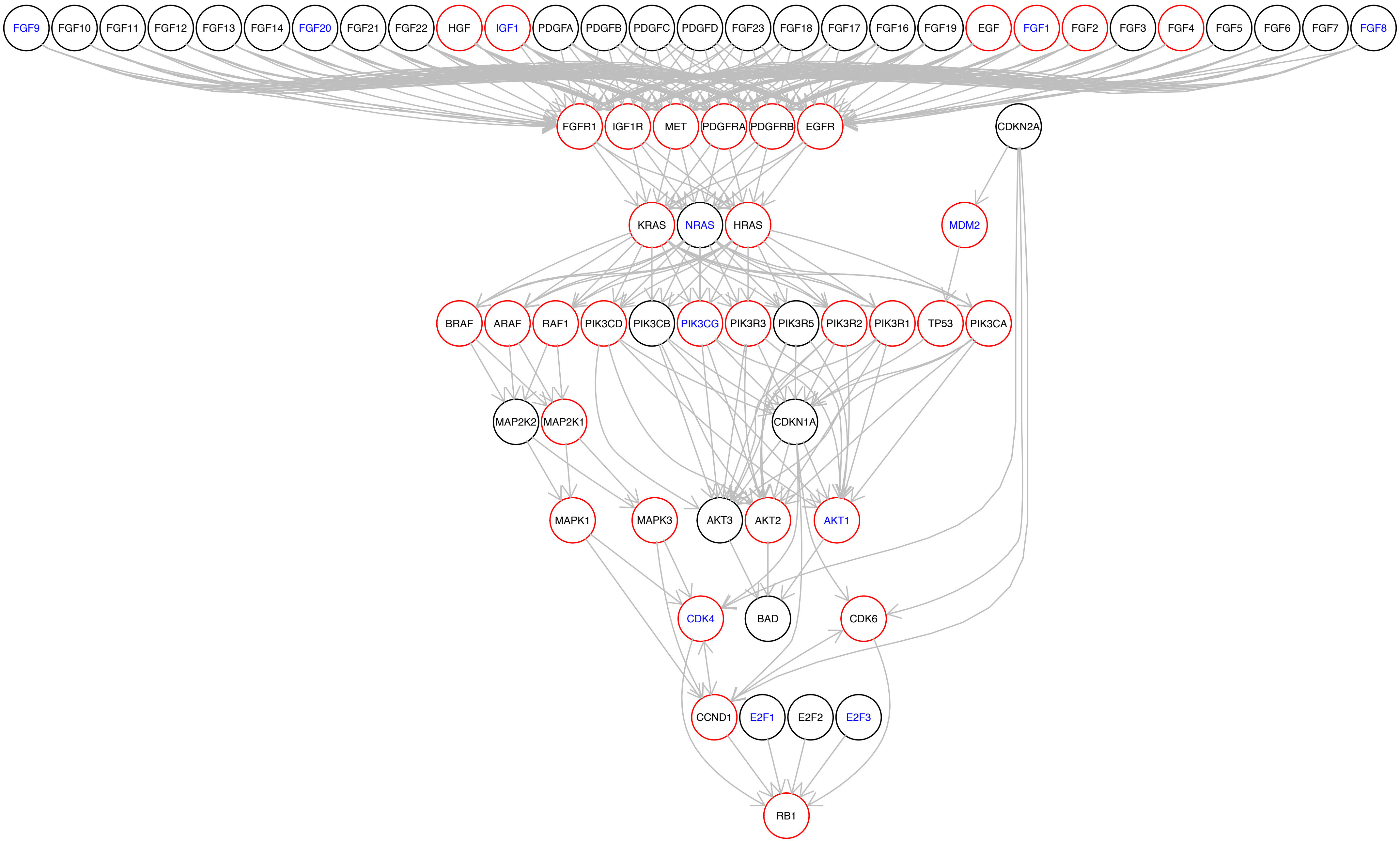

KEGG pathway = Melanoma : tumour = YUKADI : Yellow Fill = gene variant, Blue Text = expression−survival association, Red Boarder = drug

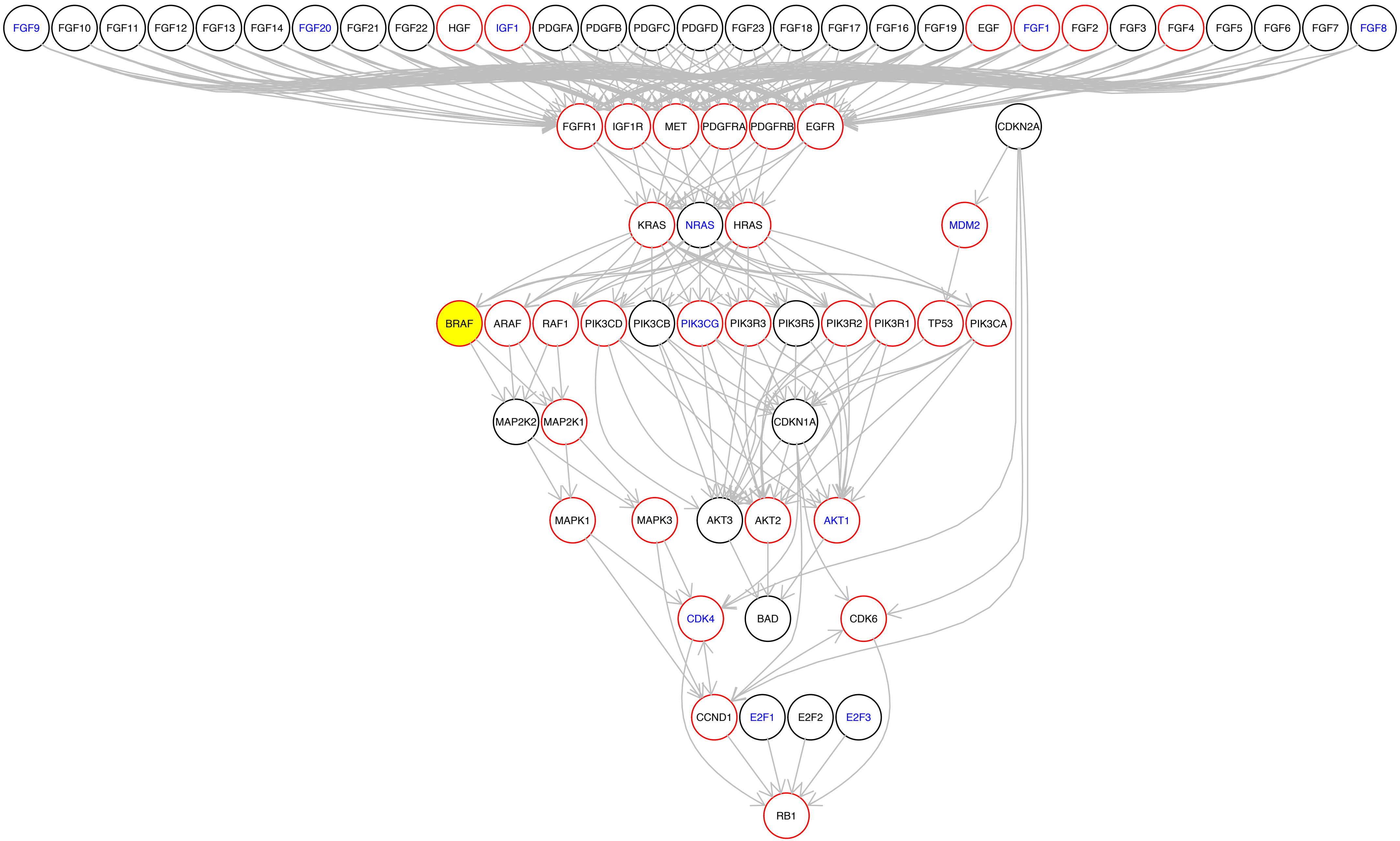

KEGG pathway = Melanoma : tumour = YUKARN : Yellow Fill = gene variant, Blue Text = expression-survival association, Red Boarder = drug

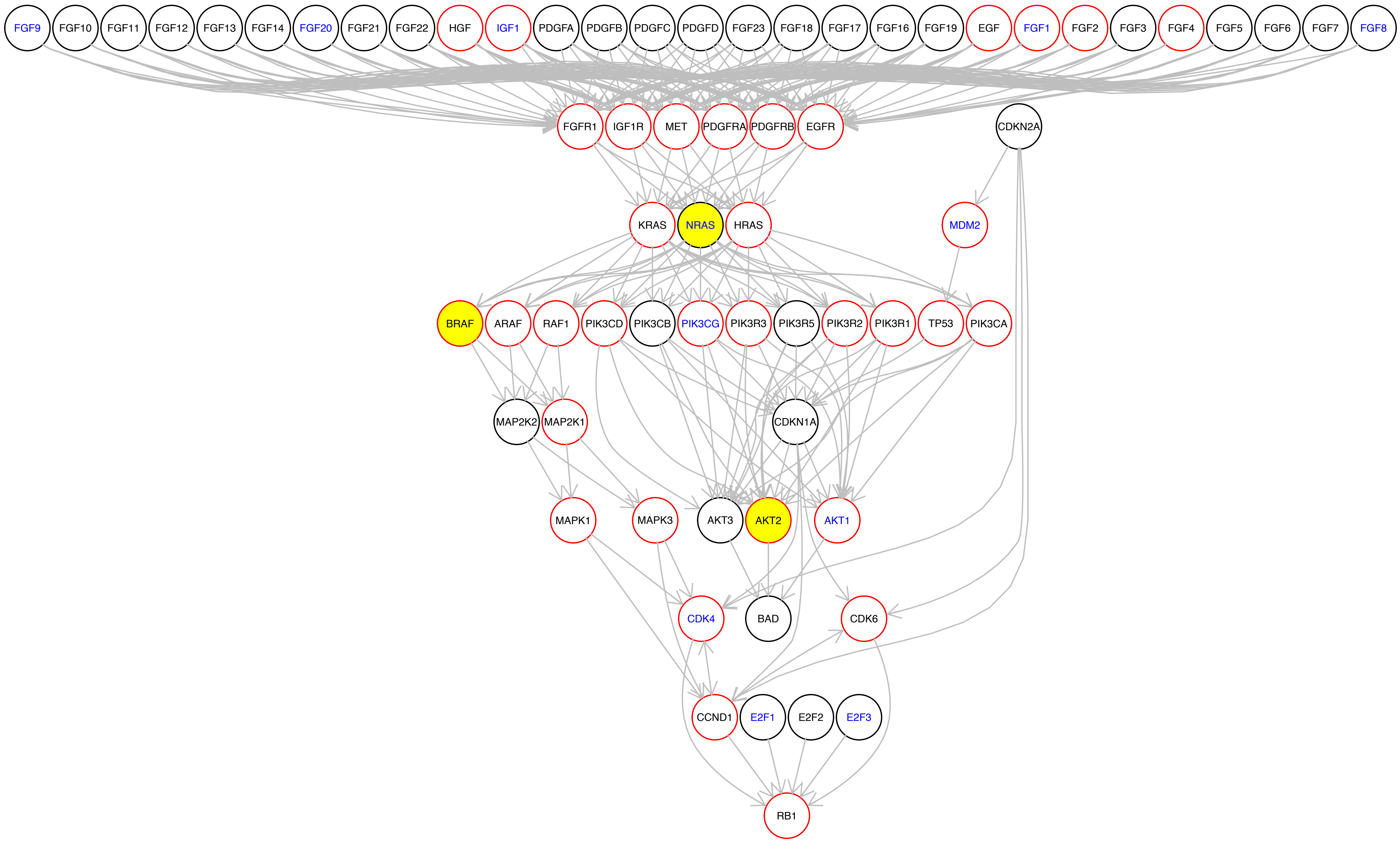

KEGG pathway = Melanoma : tumour = YUKIL : Yellow Fill = gene variant, Blue Text = expression−survival association, Red Boarder = drug

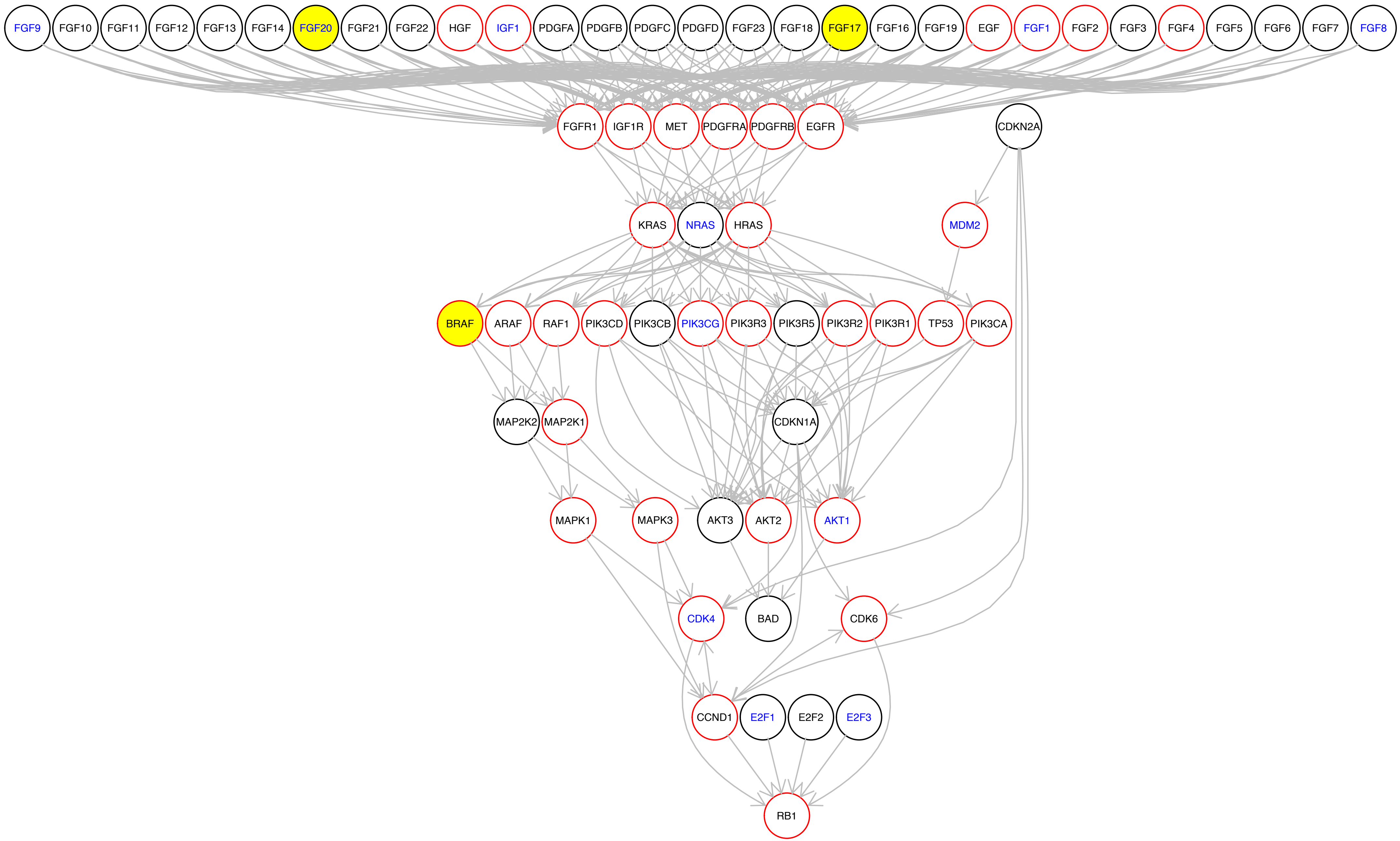

KEGG pathway = Melanoma : tumour = YUKLAB : Yellow Fill = gene variant, Blue Text = expression−survival association, Red Boarder = drug

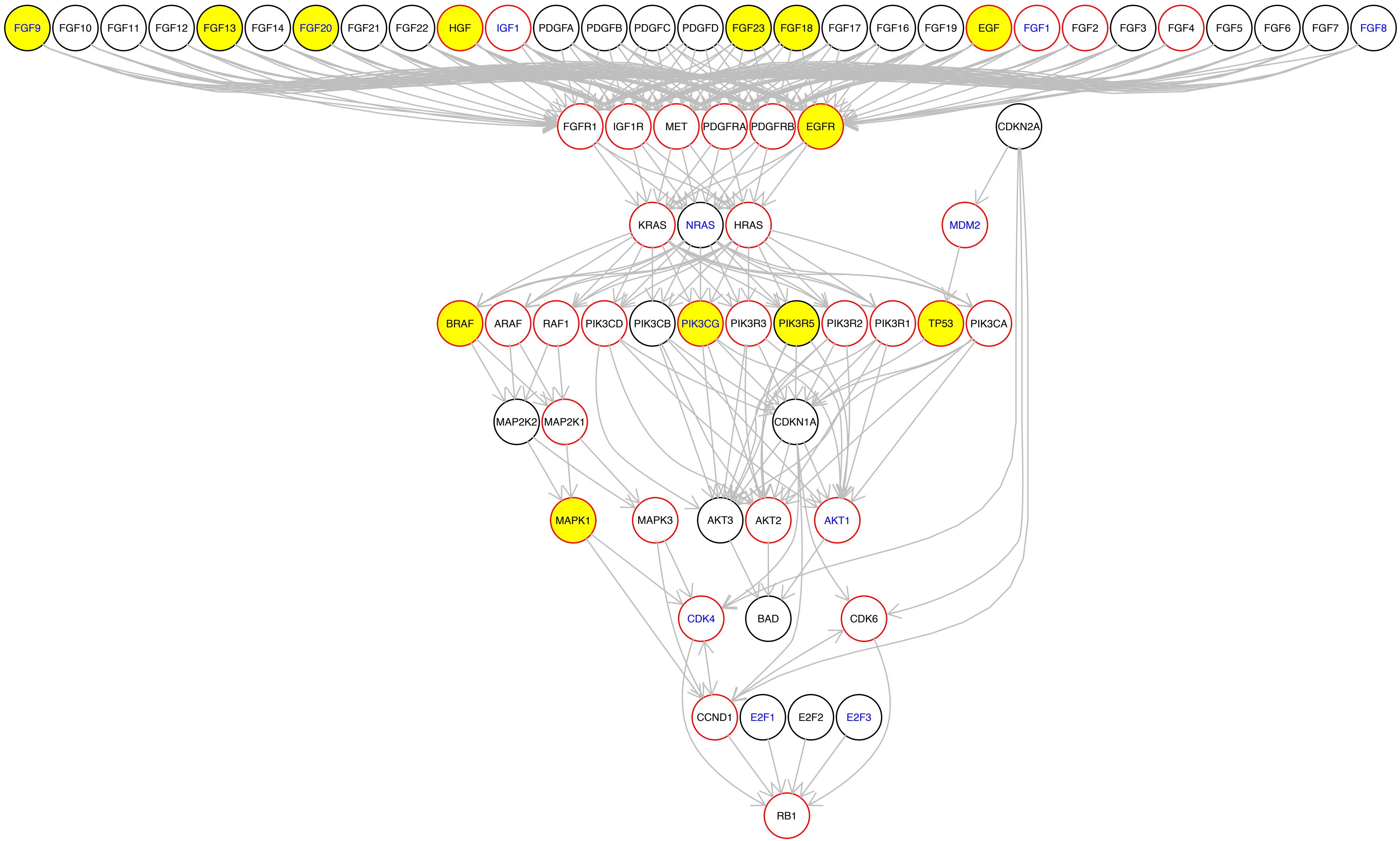

KEGG pathway = Melanoma : tumour = YUKRIN : Yellow Fill = gene variant, Blue Text = expression−survival association, Red Boarder = drug

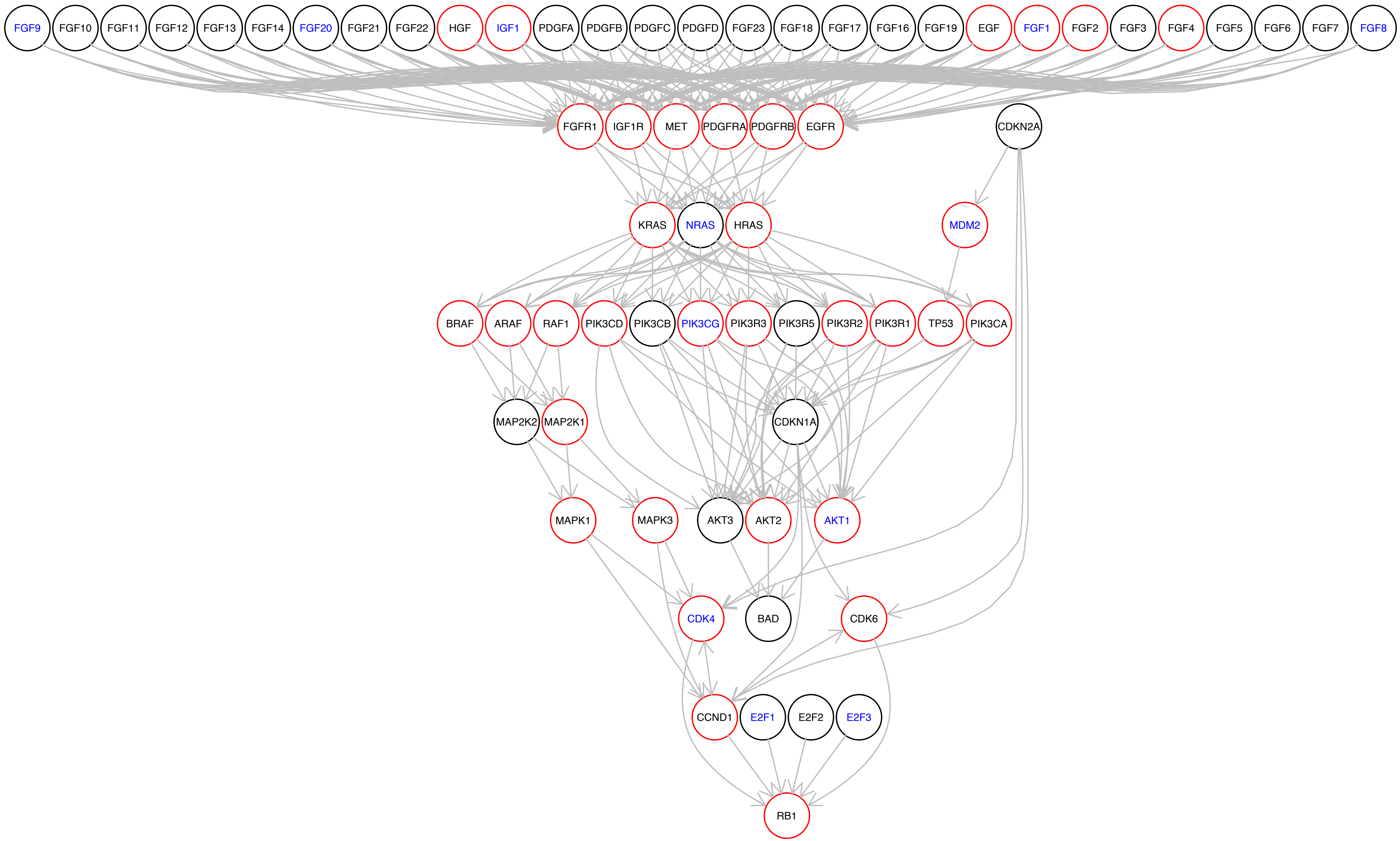

KEGG pathway = Melanoma : tumour = YULAN : Yellow Fill = gene variant, Blue Text = expression–survival association, Red Boarder = drug

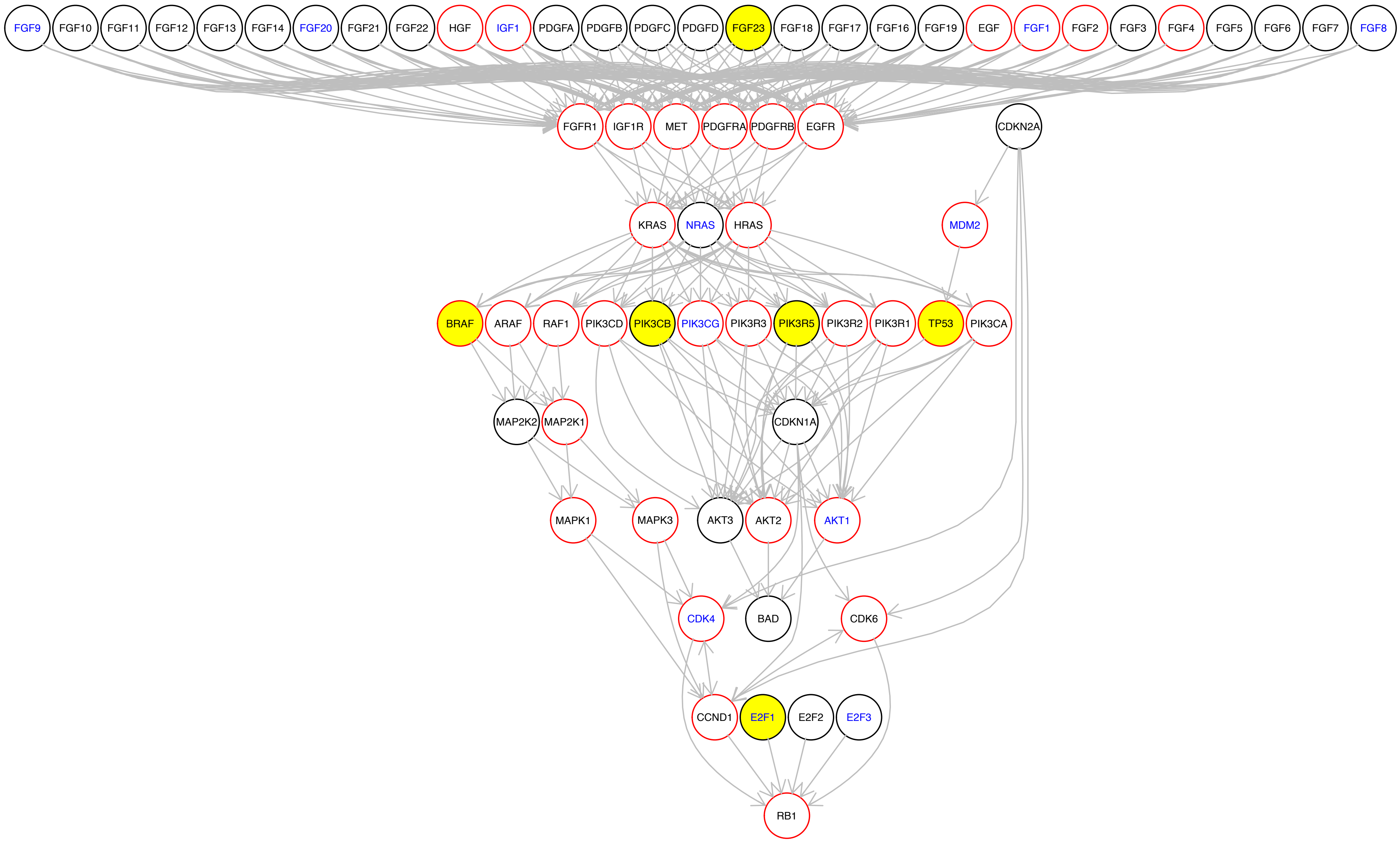

KEGG pathway = Melanoma : tumour = YULAPE : Yellow Fill = gene variant, Blue Text = expression−survival association, Red Boarder = drug

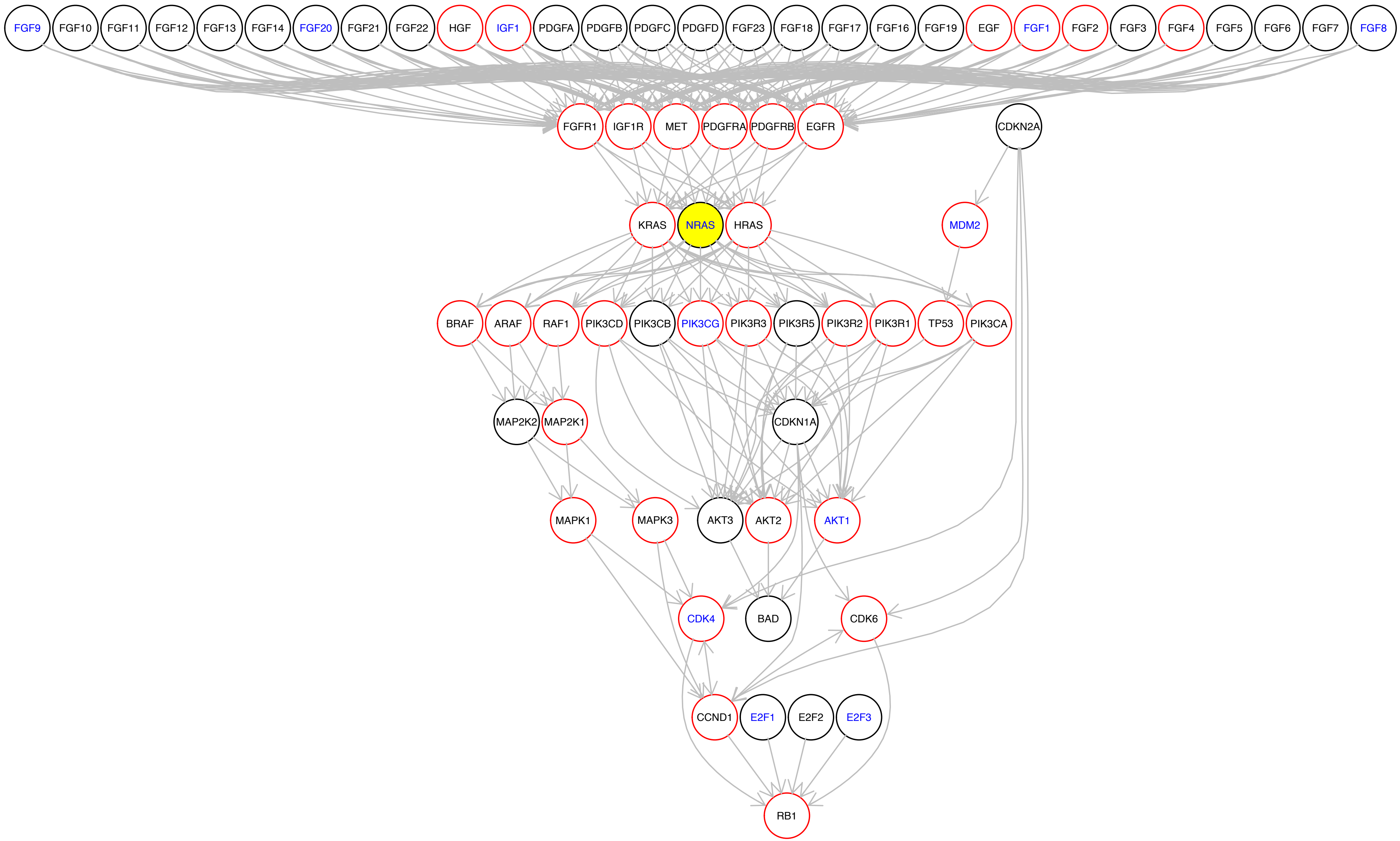

KEGG pathway = Melanoma : tumour = YULAXER : Yellow Fill = gene variant, Blue Text = expression–survival association, Red Boarder = drug

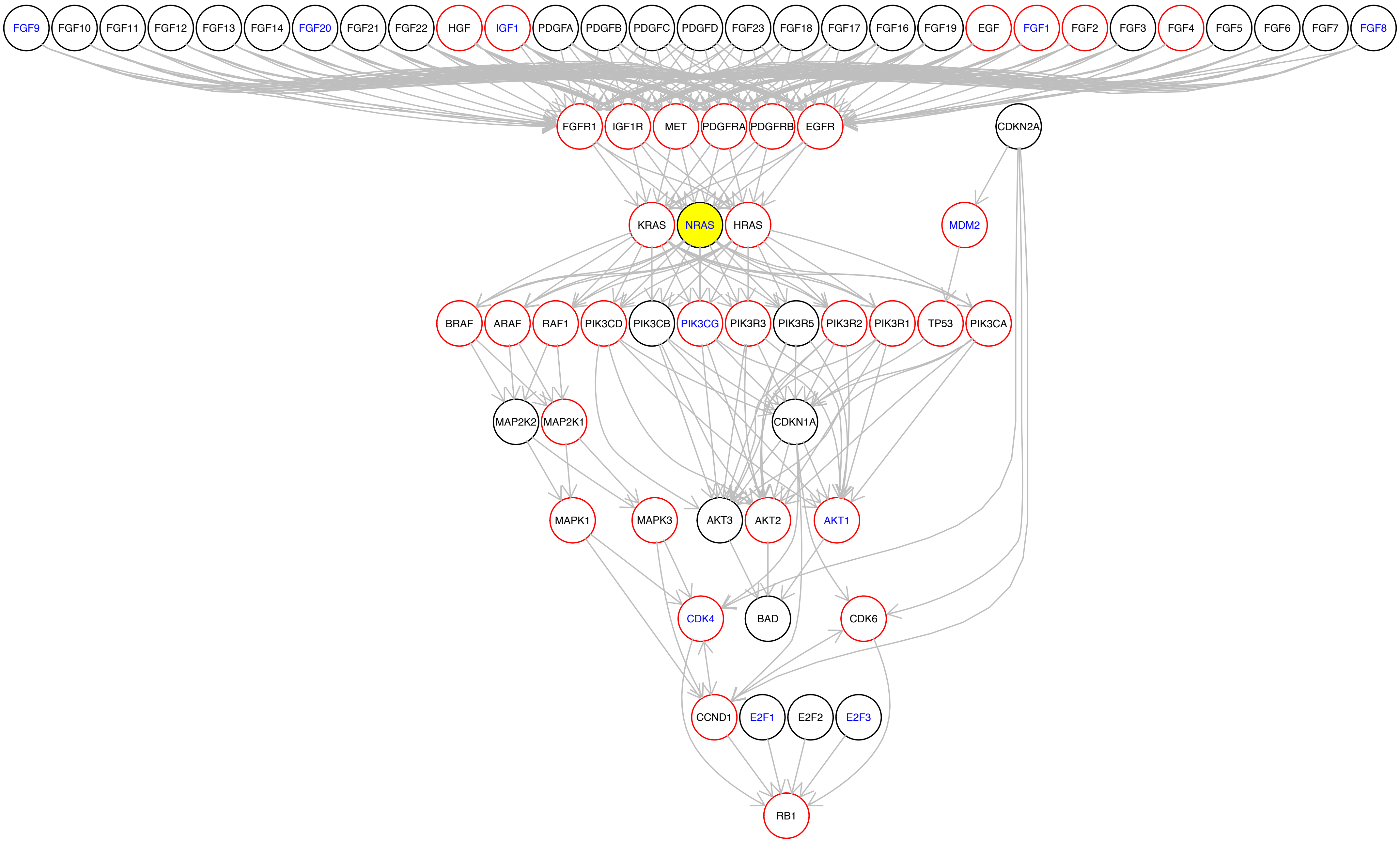

KEGG pathway = Melanoma : tumour = YULLON : Yellow Fill = gene variant, Blue Text = expression−survival association, Red Boarder = drug

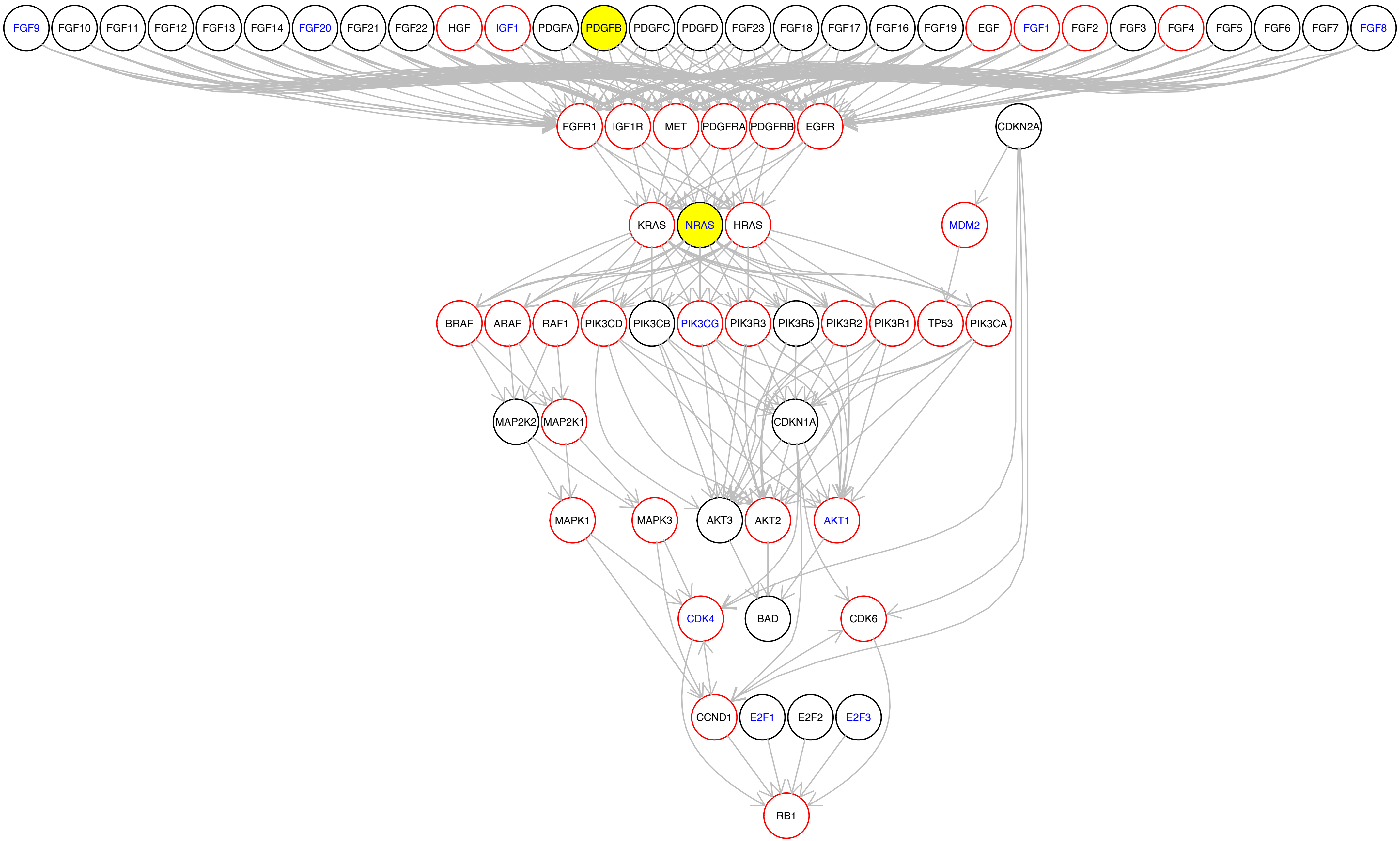

KEGG pathway = Melanoma : tumour = YULOMA : Yellow Fill = gene variant, Blue Text = expression–survival association, Red Boarder = drug

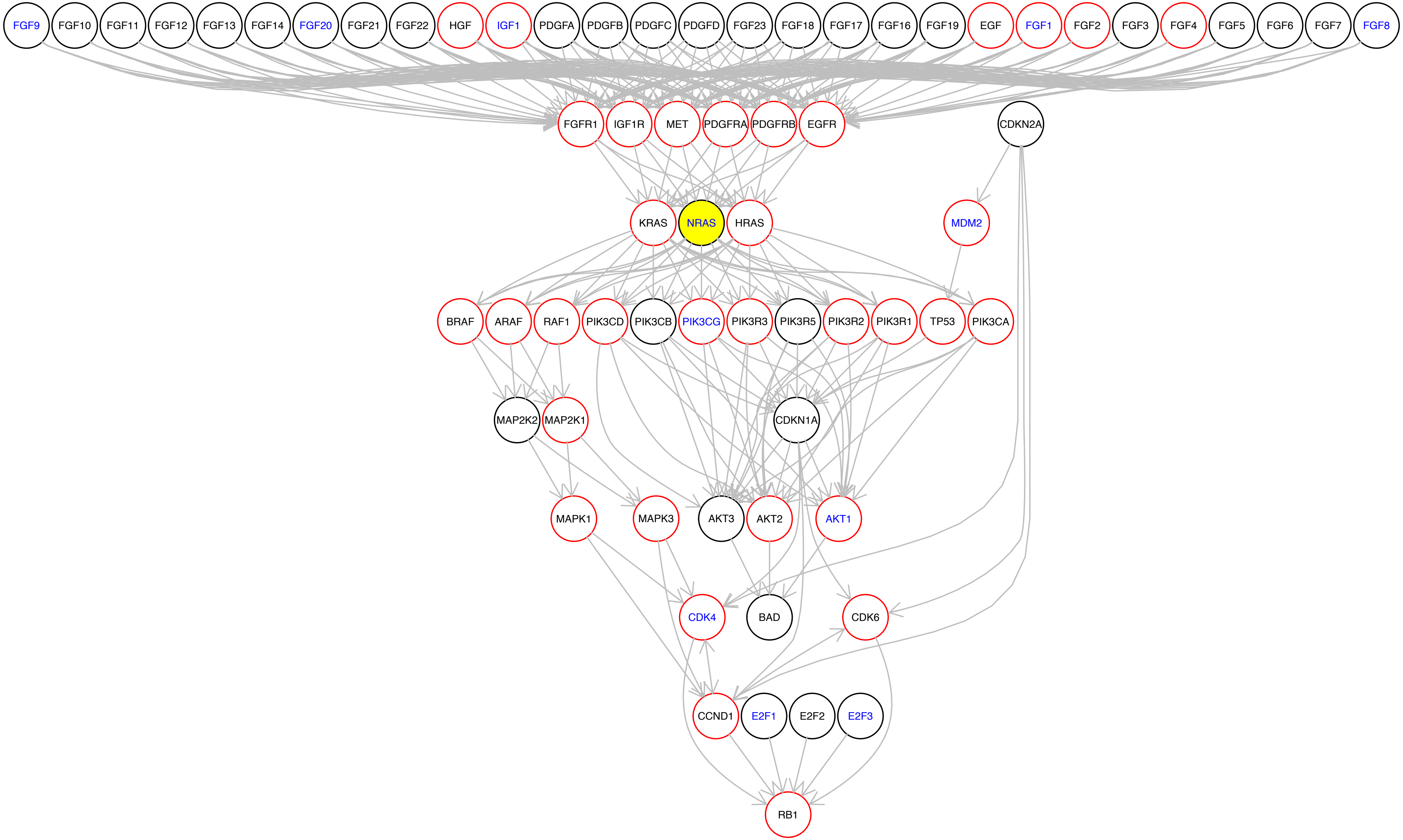

KEGG pathway = Melanoma :   tumour = YULOVY :   Yellow Fill = gene variant, Blue Text = expression−survival association, Red Boarder = drug

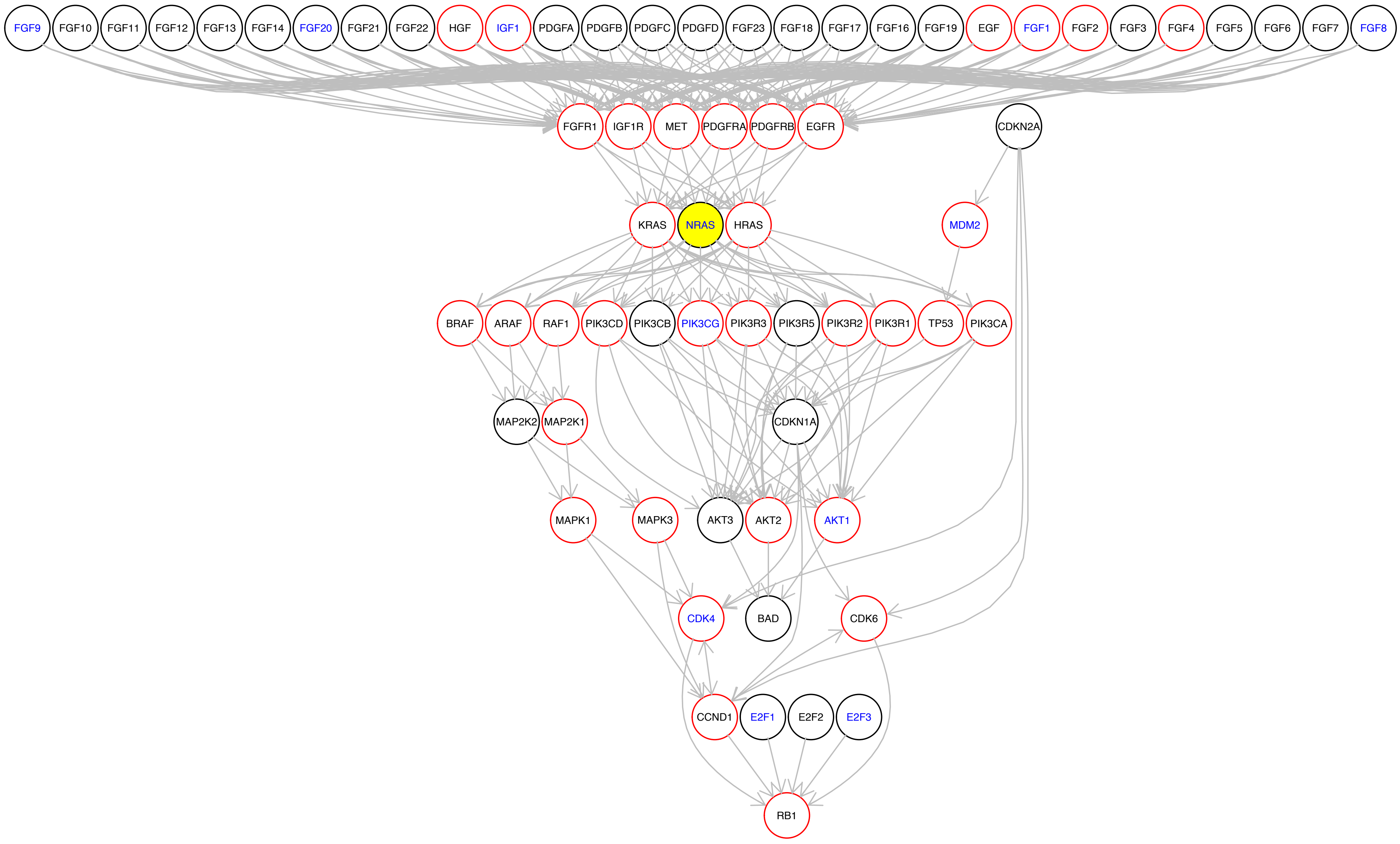

KEGG pathway = Melanoma : tumour = YUMAN : Yellow Fill = gene variant, Blue Text = expression−survival association, Red Boarder = drug

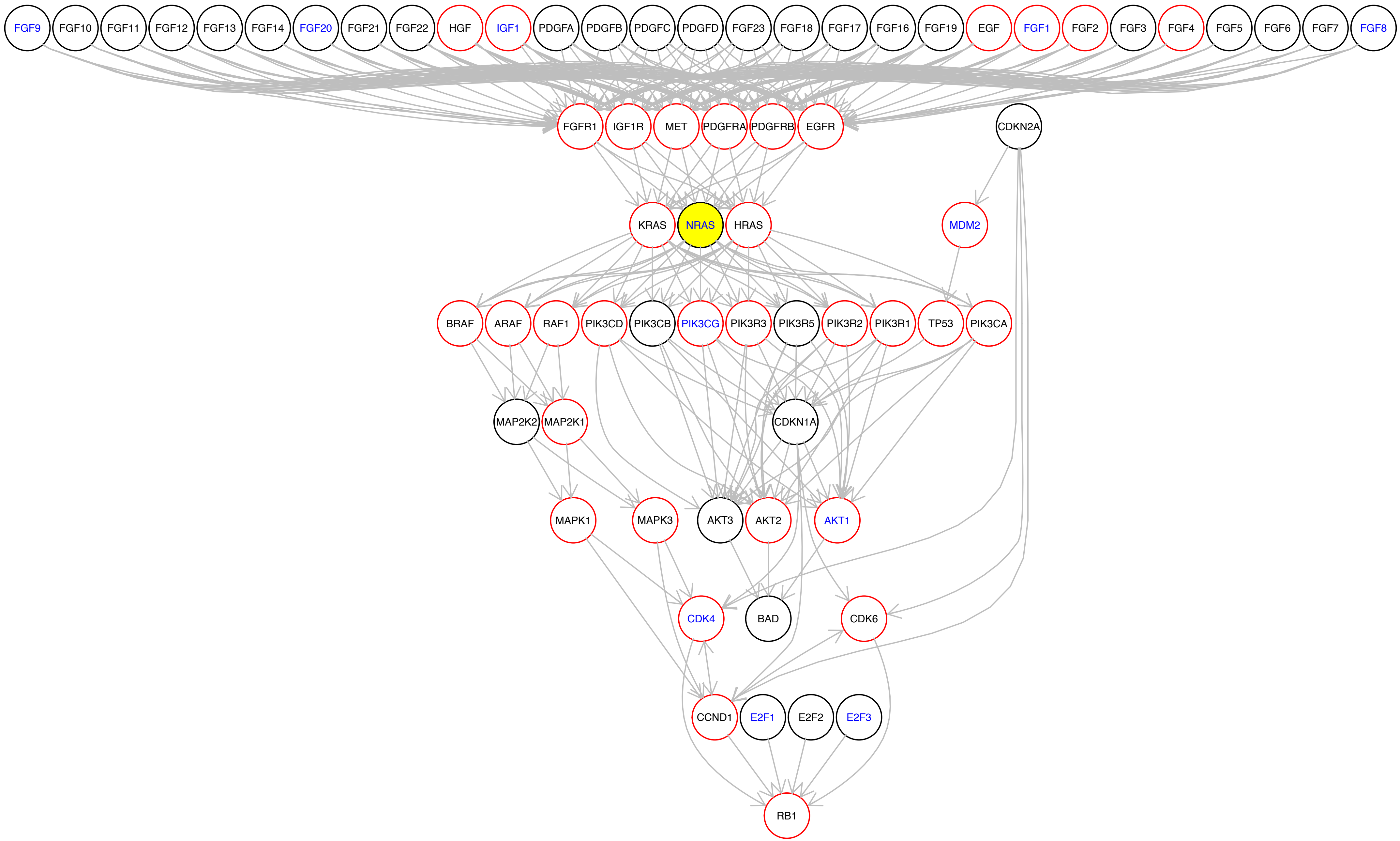

KEGG pathway = Melanoma :   tumour = YUMER :   Yellow Fill = gene variant, Blue Text = expression−survival association, Red Boarder = drug

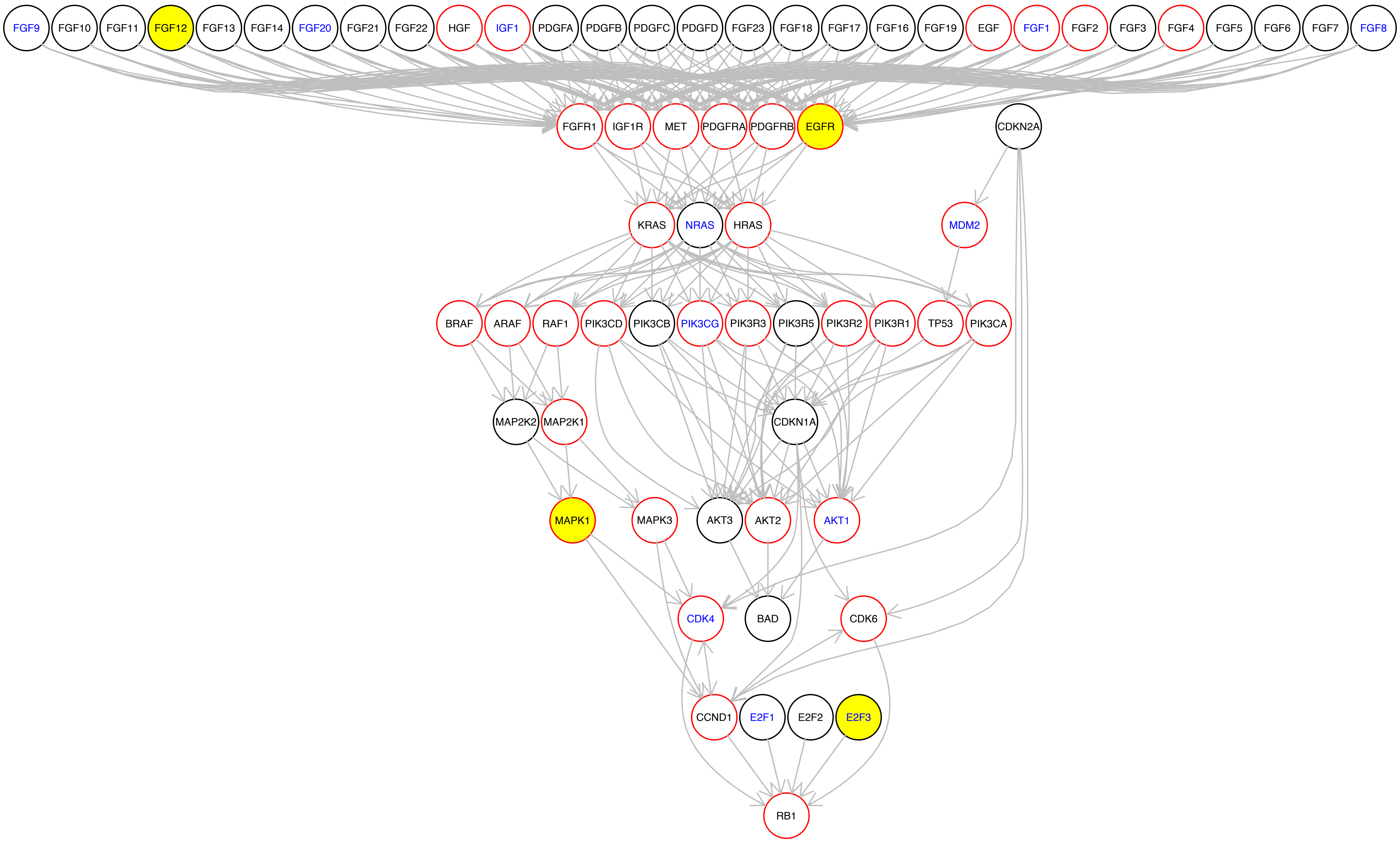

KEGG pathway = Melanoma : tumour = YUMOOK : Yellow Fill = gene variant, Blue Text = expression−survival association, Red Boarder = drug

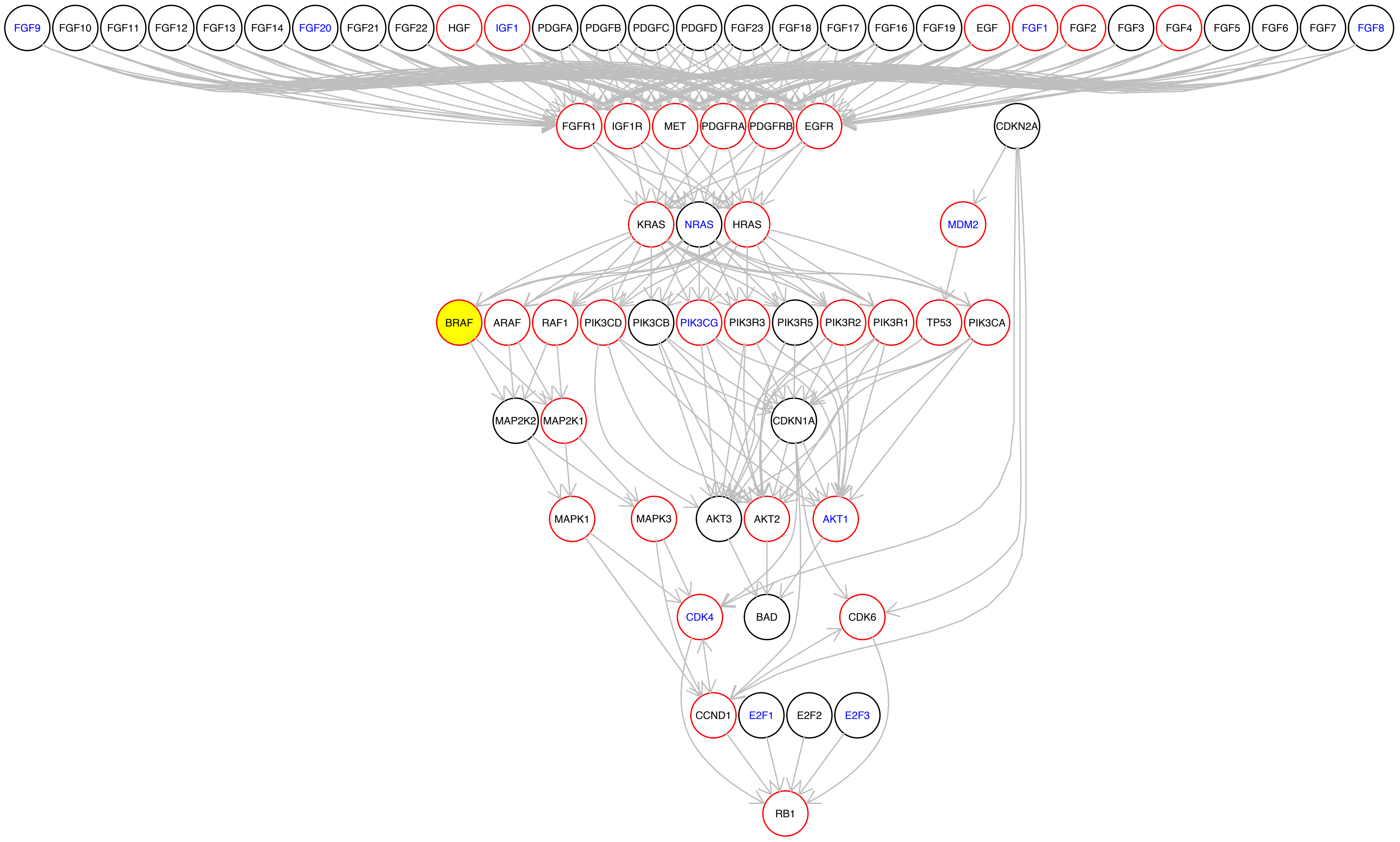

KEGG pathway = Melanoma : tumour = YUMOYA : Yellow Fill = gene variant, Blue Text = expression−survival association, Red Boarder = drug

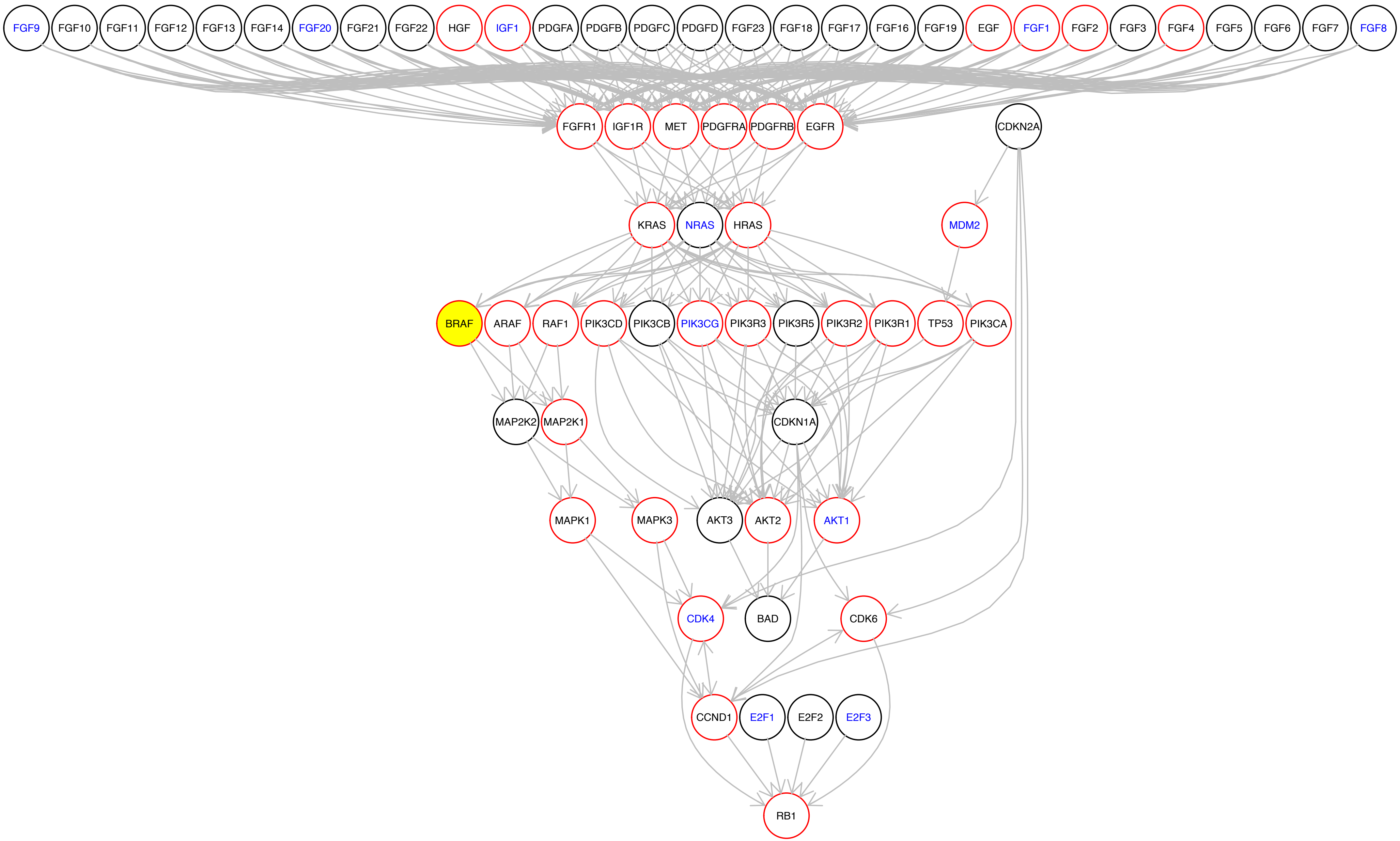

KEGG pathway = Melanoma :   tumour = YUMUT :   Yellow Fill = gene variant, Blue Text = expression−survival association, Red Boarder = drug

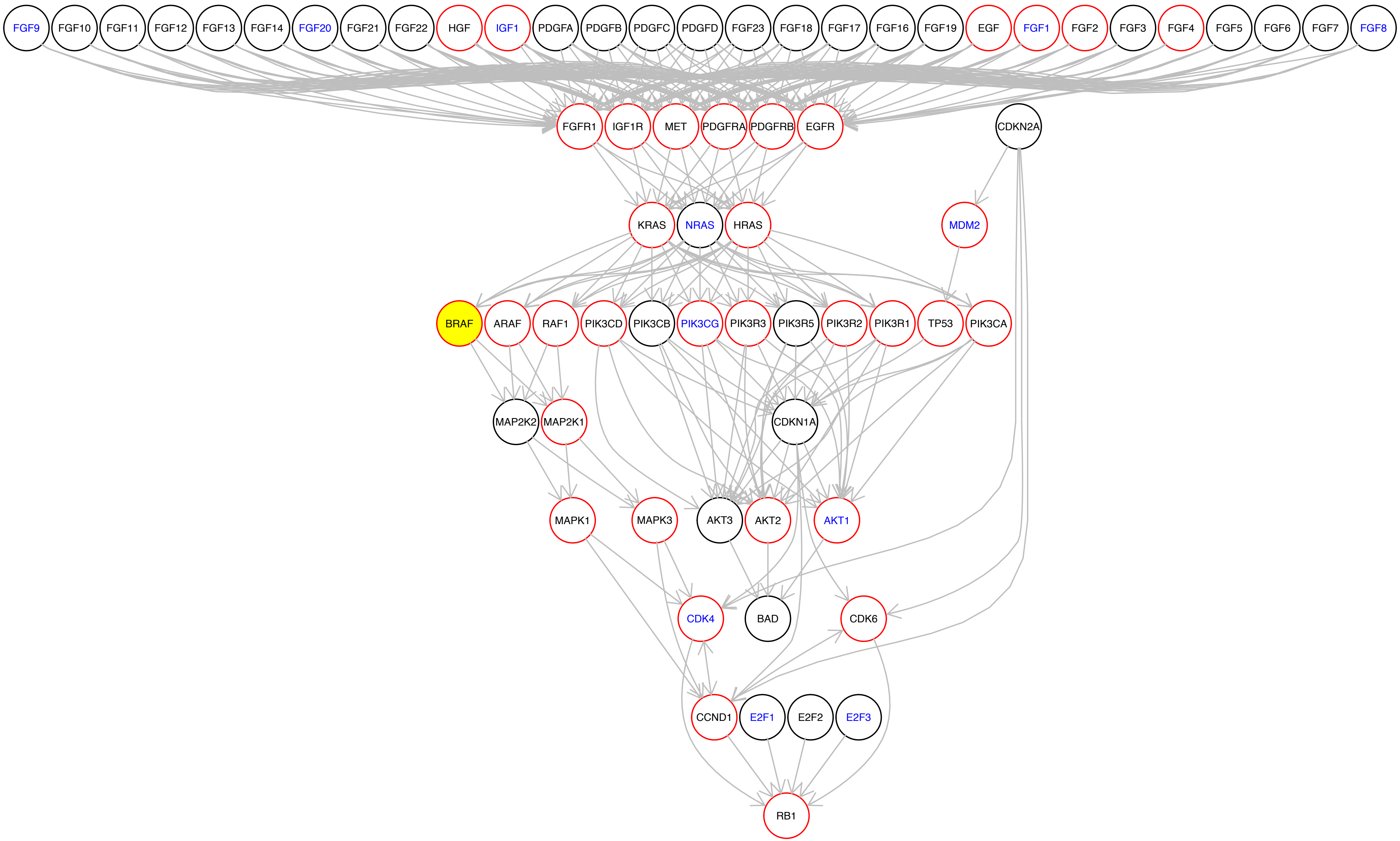

KEGG pathway = Melanoma : tumour = YUNACK : Yellow Fill = gene variant, Blue Text = expression−survival association, Red Boarder = drug

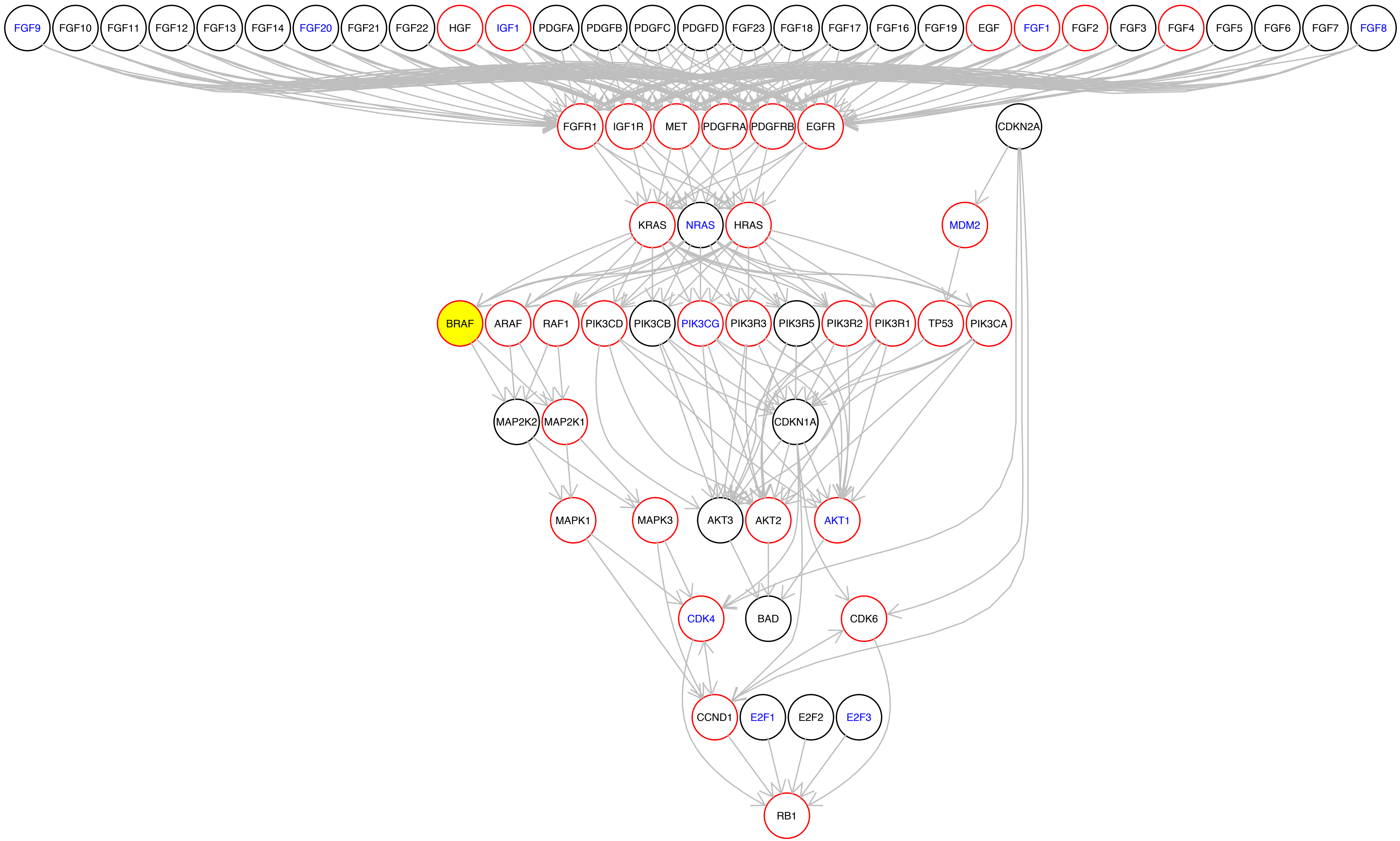

KEGG pathway = Melanoma : tumour = YUNEON : Yellow Fill = gene variant, Blue Text = expression−survival association, Red Boarder = drug

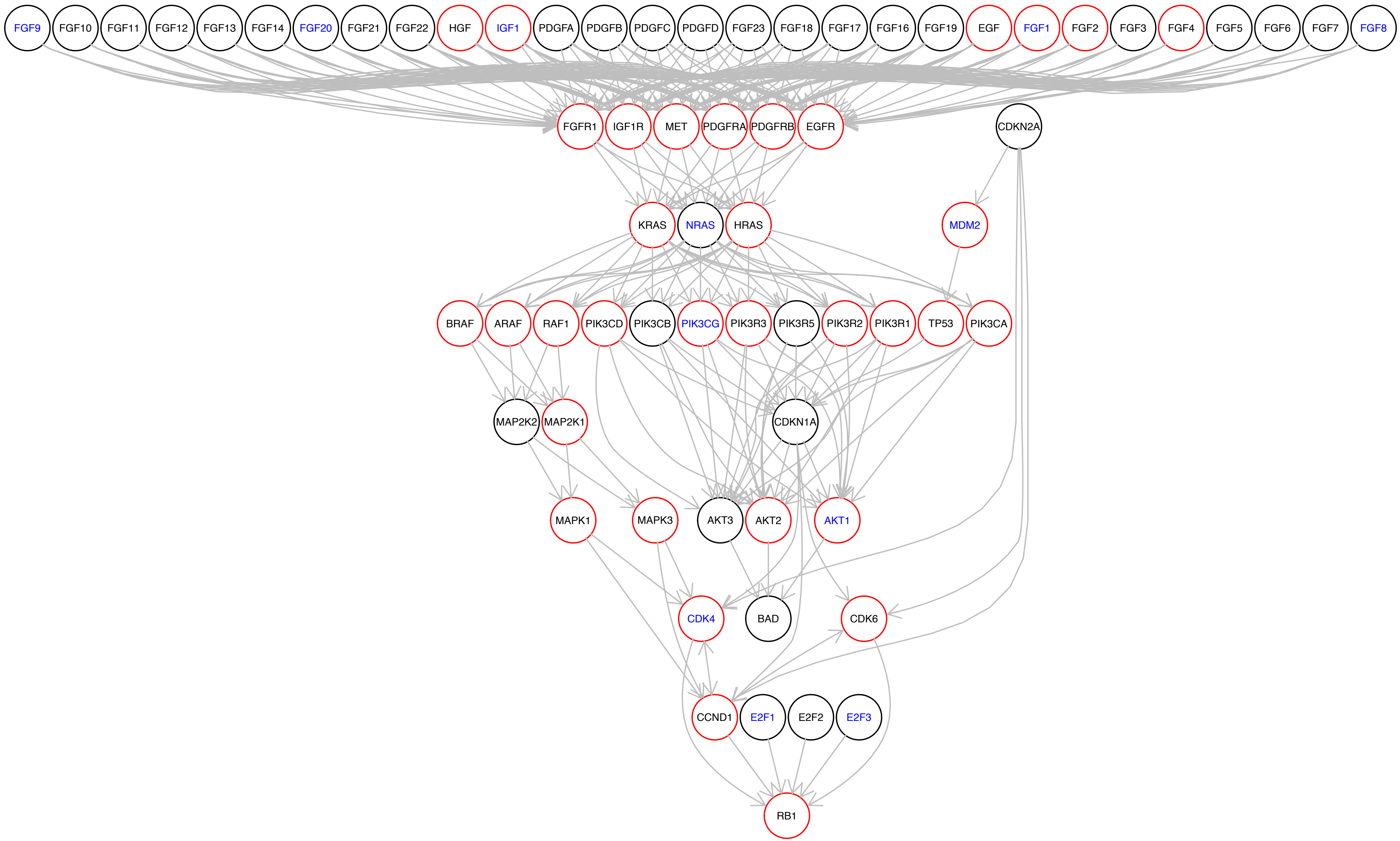

KEGG pathway = Melanoma : tumour = YUNOCA : Yellow Fill = gene variant, Blue Text = expression–survival association, Red Boarder = drug

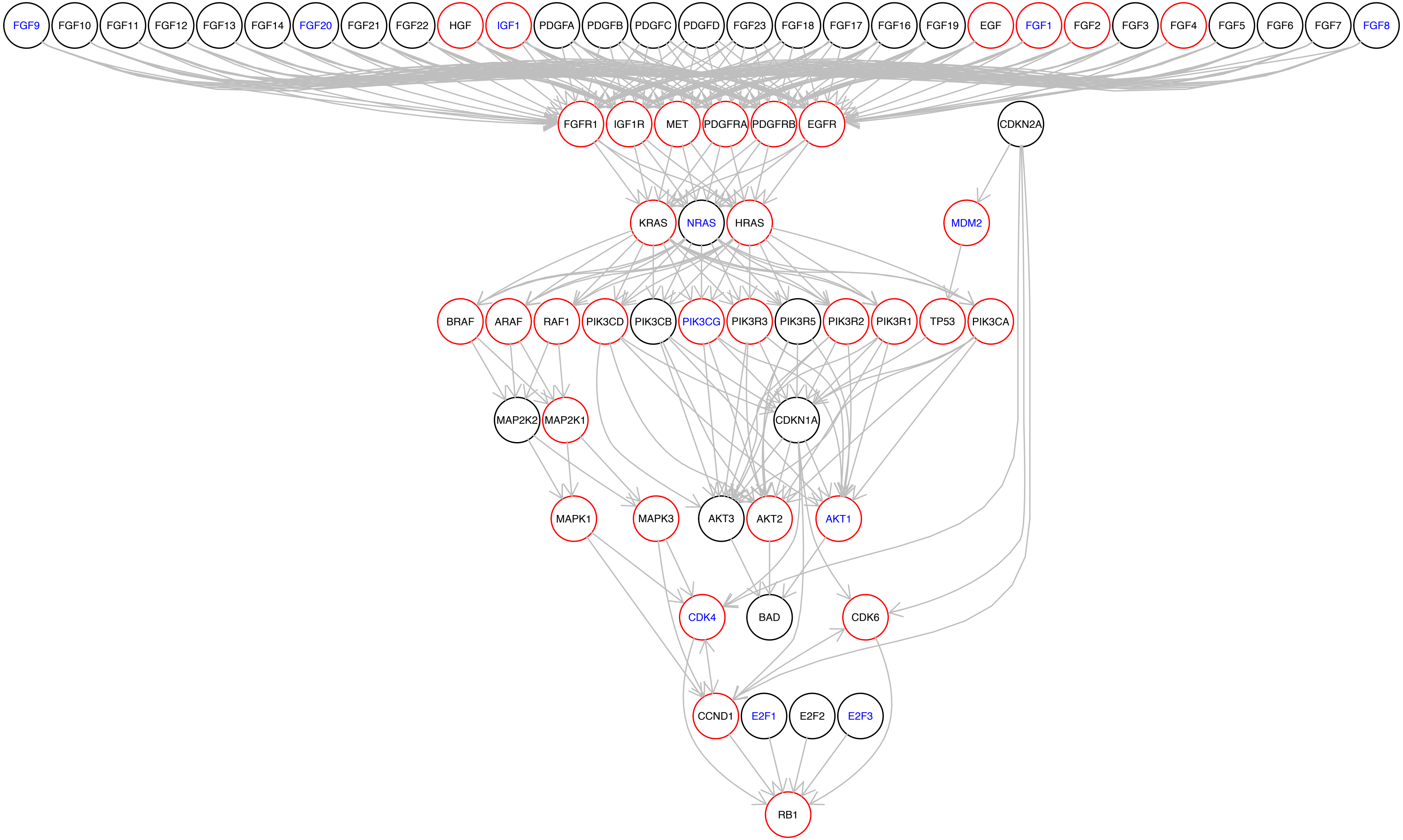

KEGG pathway = Melanoma :   tumour = YUNUFF :   Yellow Fill = gene variant, Blue Text = expression−survival association, Red Boarder = drug

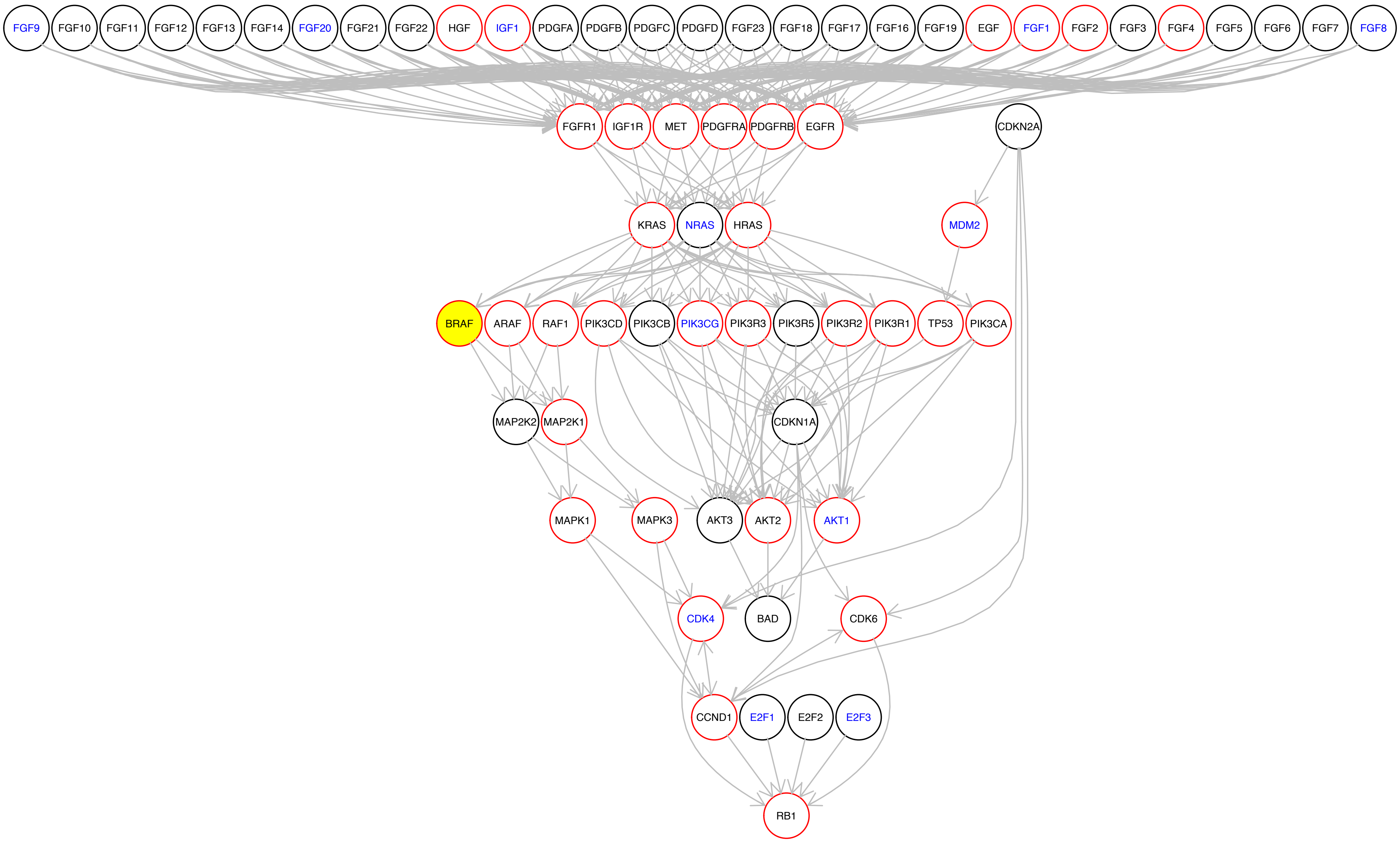

KEGG pathway = Melanoma : tumour = YUNUVO : Yellow Fill = gene variant, Blue Text = expression–survival association, Red Boarder = drug

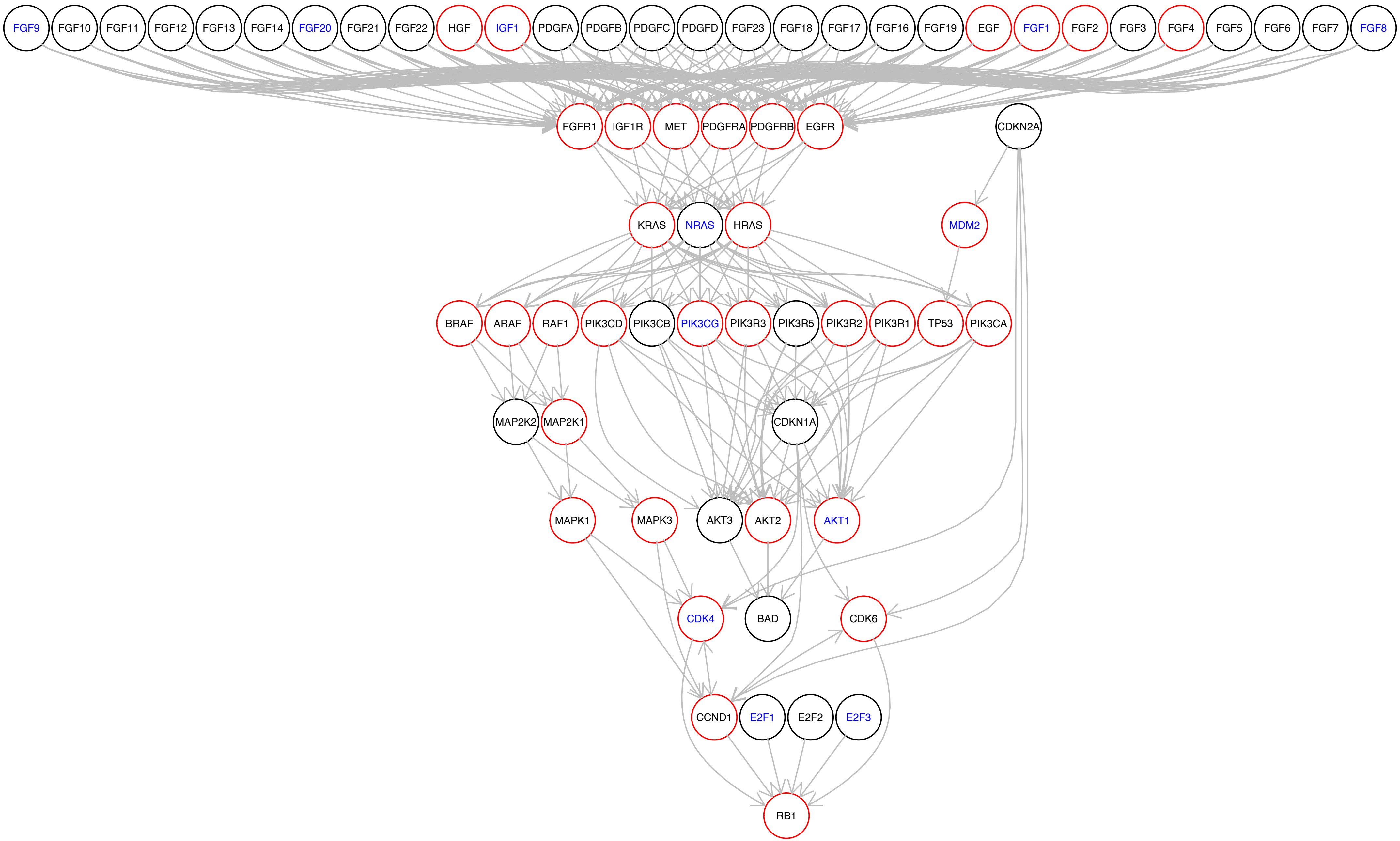

KEGG pathway = Melanoma : tumour = YUPADI : Yellow Fill = gene variant, Blue Text = expression−survival association, Red Boarder = drug

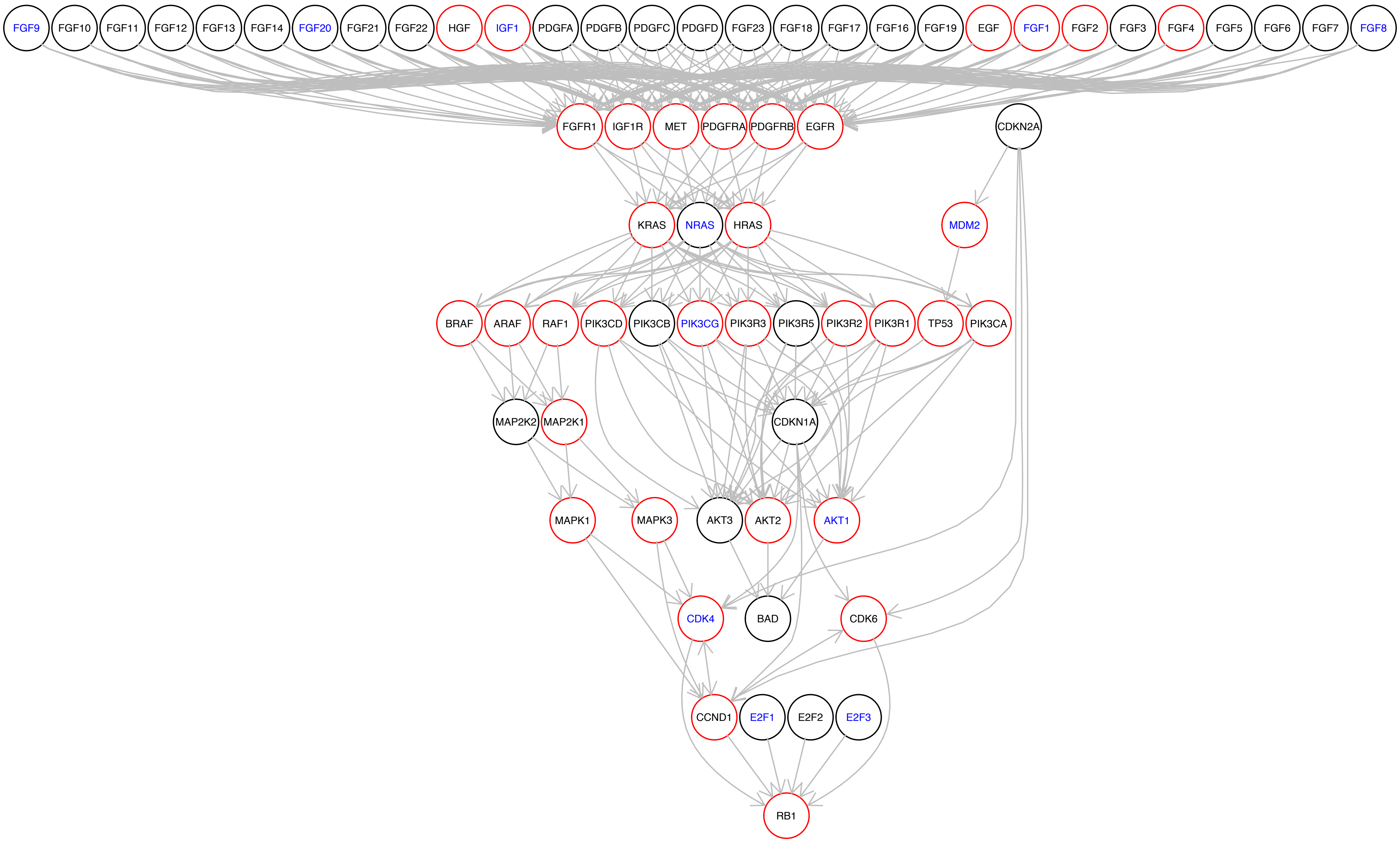

KEGG pathway = Melanoma :   tumour = YUPAF :   Yellow Fill = gene variant, Blue Text = expression−survival association, Red Boarder = drug

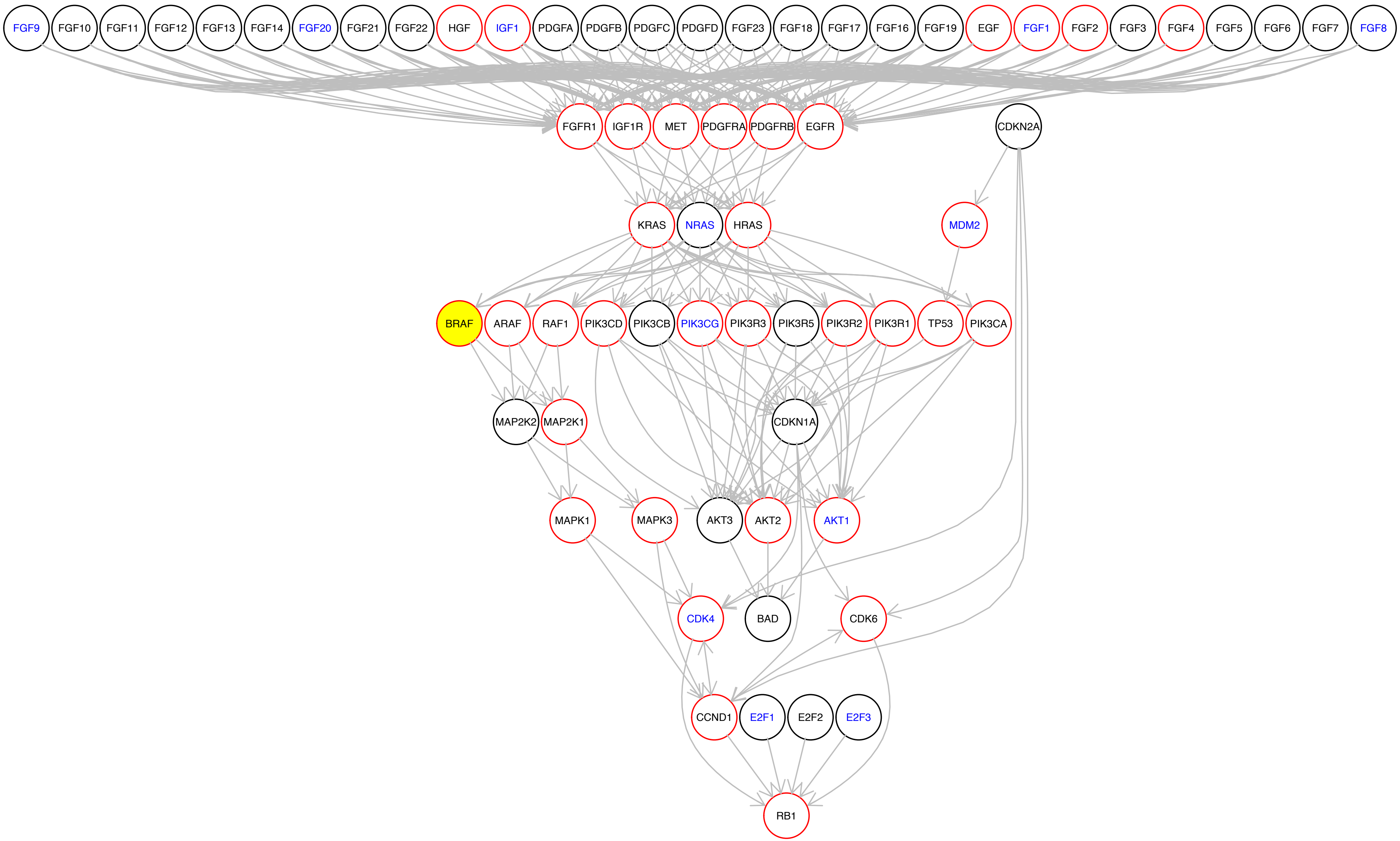

KEGG pathway = Melanoma : tumour = YUPAL : Yellow Fill = gene variant, Blue Text = expression−survival association, Red Boarder = drug

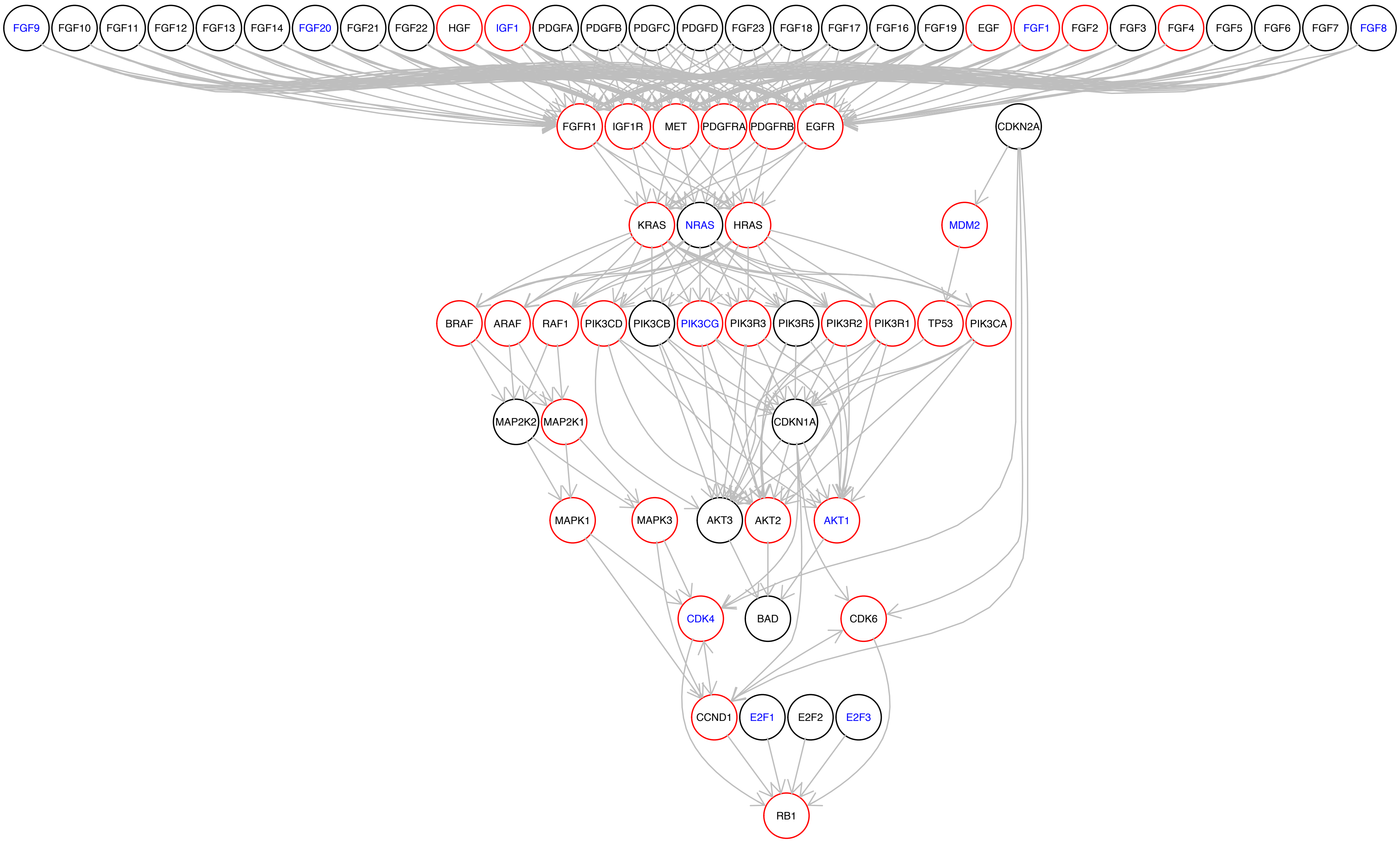

KEGG pathway = Melanoma : tumour = YUPAT : Yellow Fill = gene variant, Blue Text = expression−survival association, Red Boarder = drug

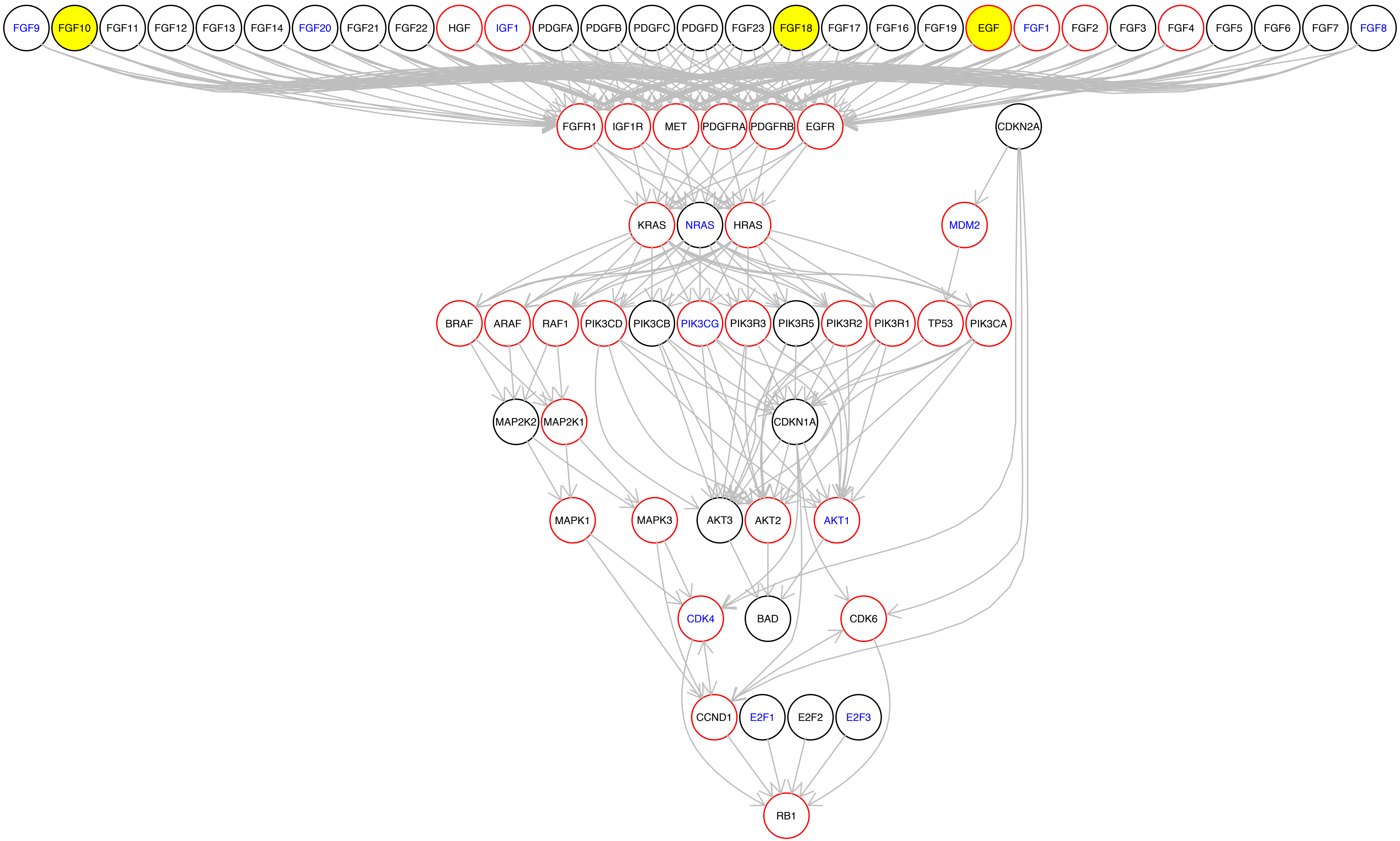

KEGG pathway = Melanoma : tumour = YUPEET : Yellow Fill = gene variant, Blue Text = expression−survival association, Red Boarder = drug

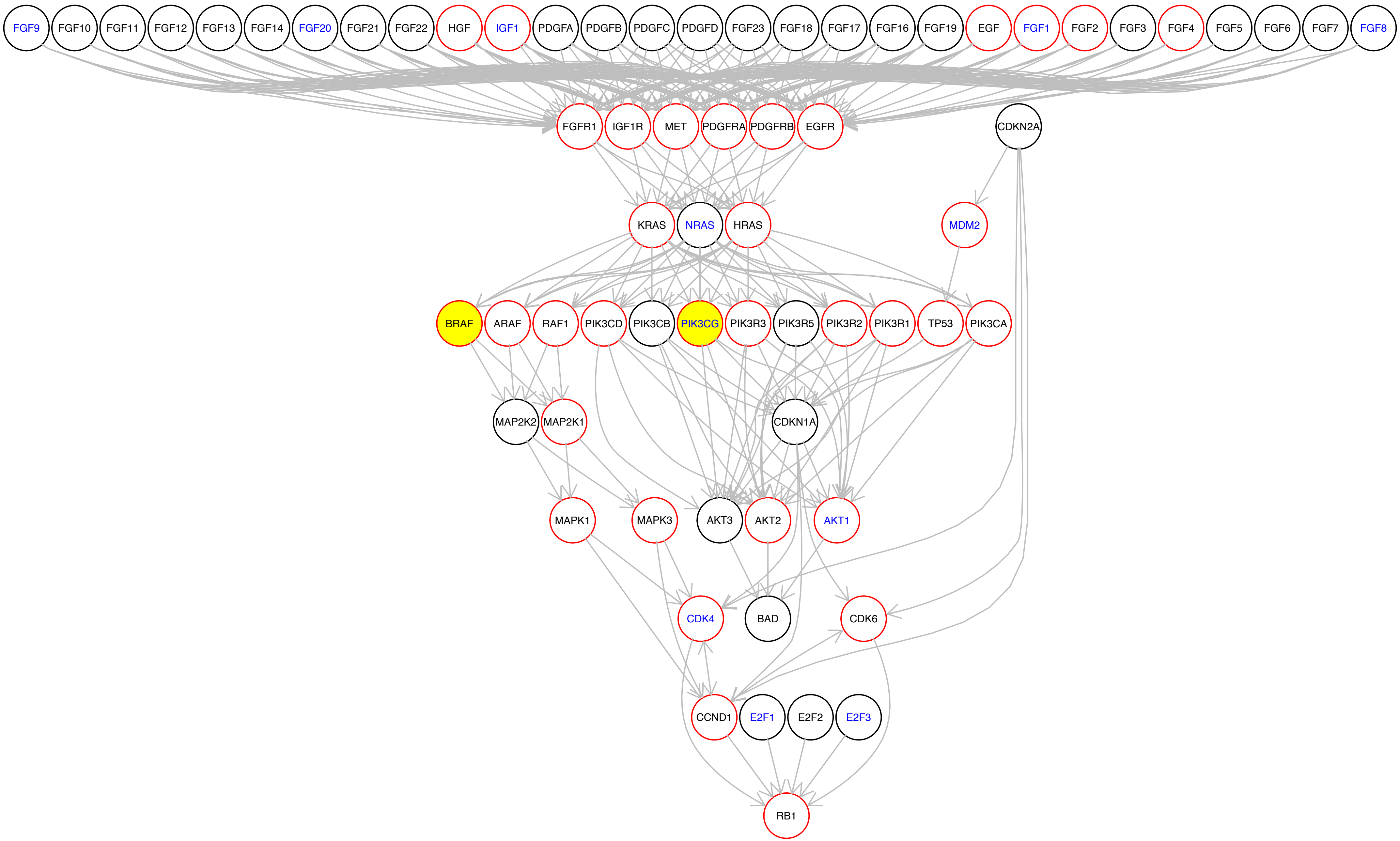

KEGG pathway = Melanoma : tumour = YUPER : Yellow Fill = gene variant, Blue Text = expression–survival association, Red Boarder = drug

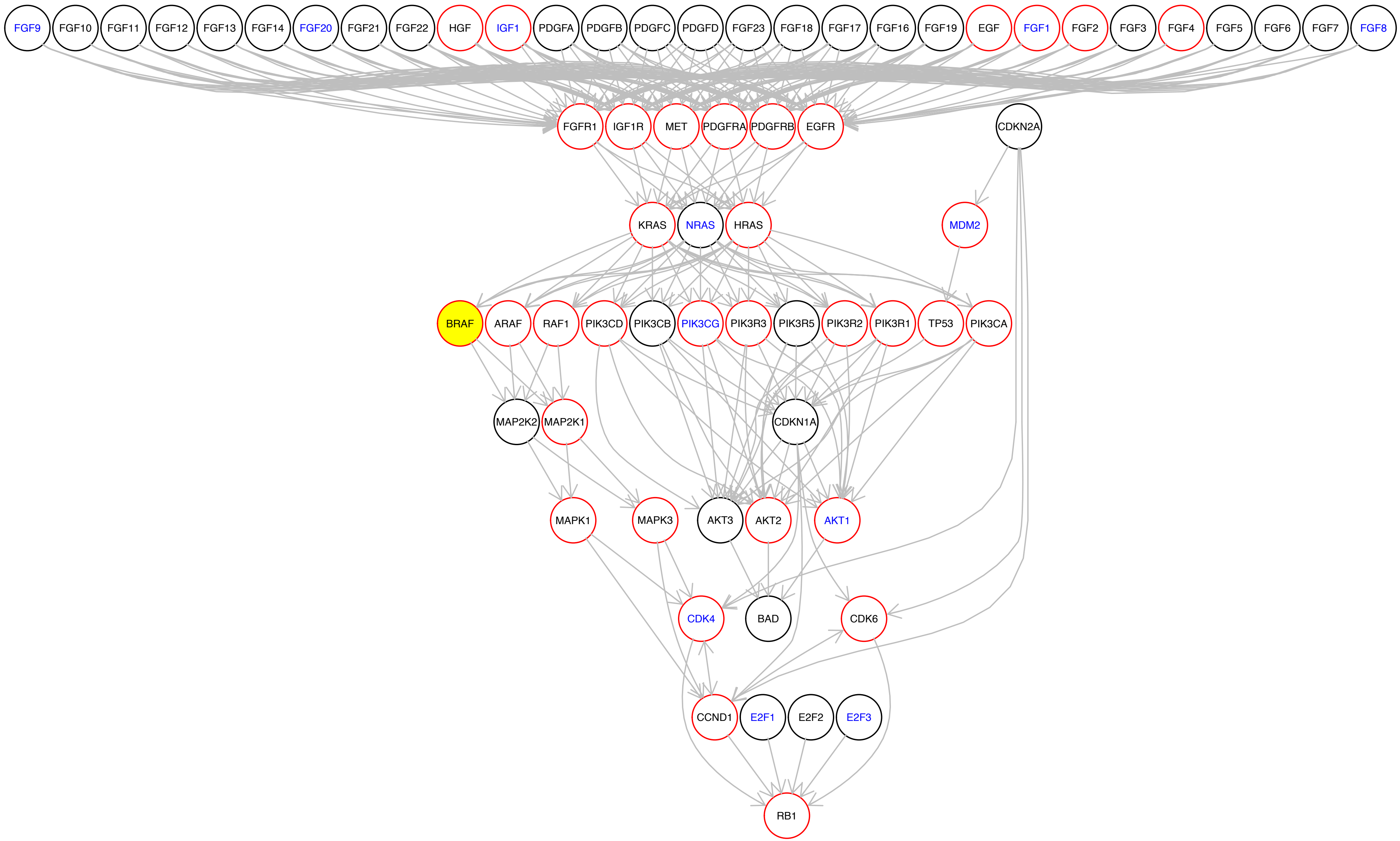

KEGG pathway = Melanoma : tumour = YUPORCH : Yellow Fill = gene variant, Blue Text = expression−survival association, Red Boarder = drug

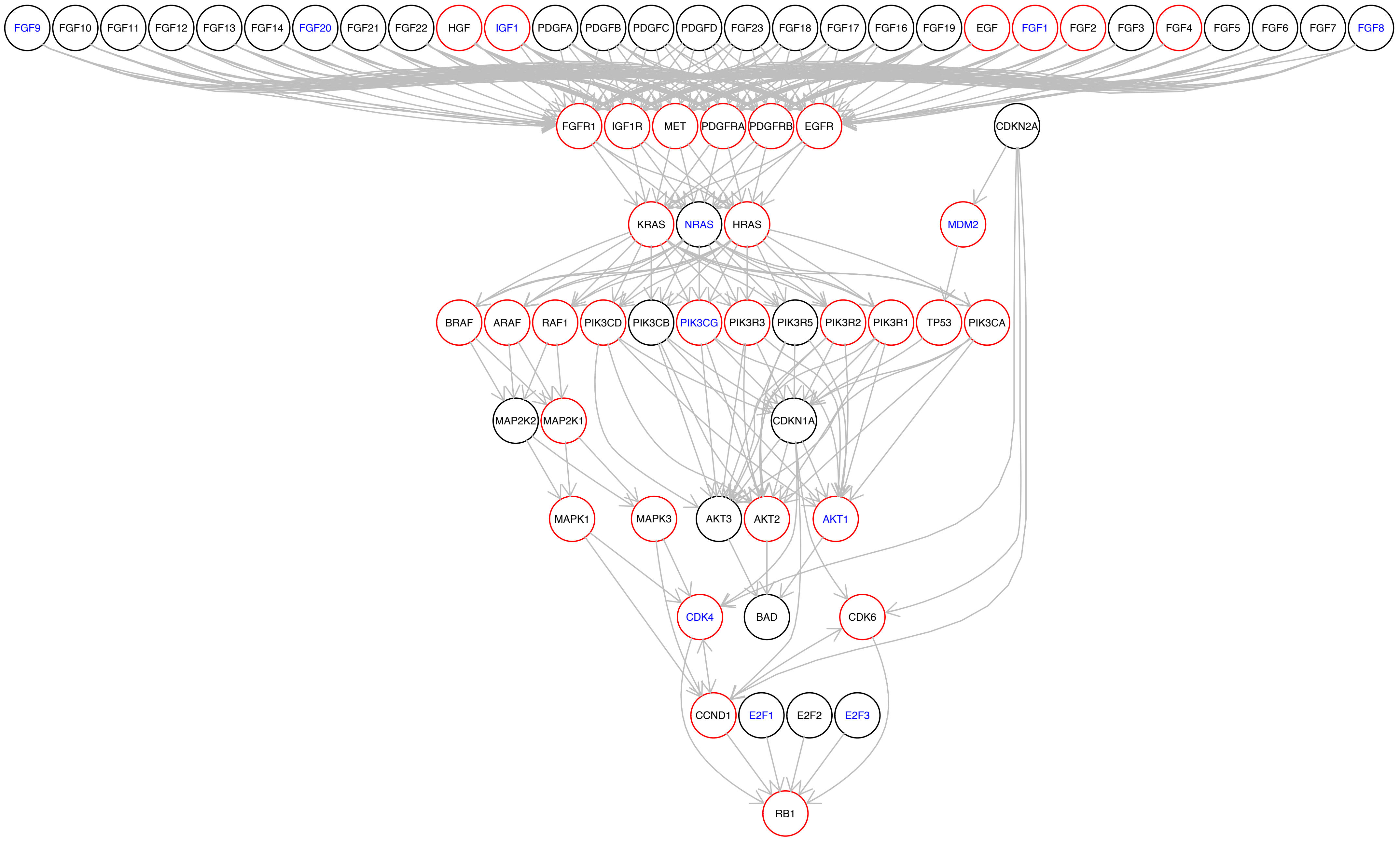

KEGG pathway = Melanoma : tumour = YUPRO : Yellow Fill = gene variant, Blue Text = expression−survival association, Red Boarder = drug

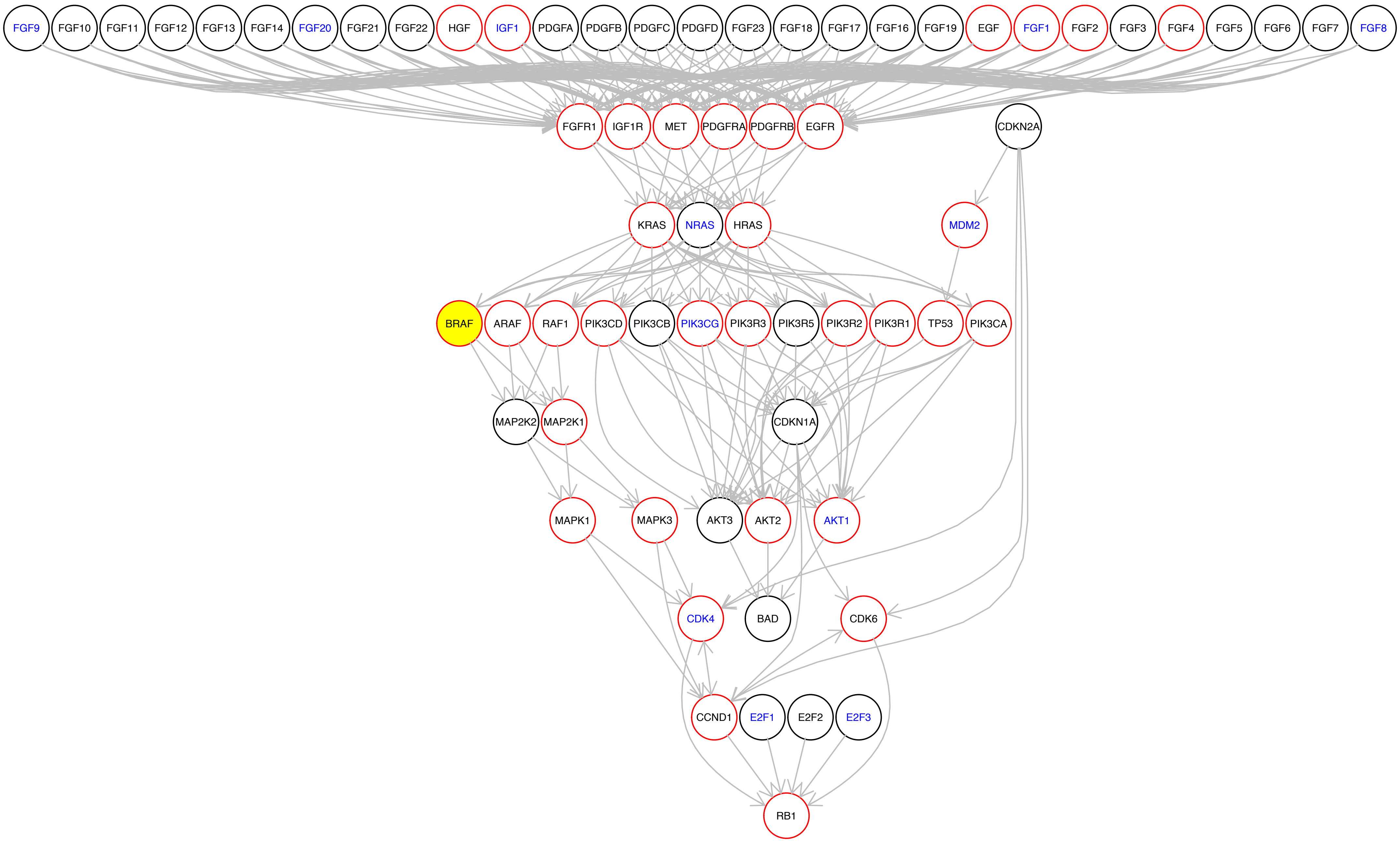

KEGG pathway = Melanoma : tumour = YUPROST : Yellow Fill = gene variant, Blue Text = expression−survival association, Red Boarder = drug

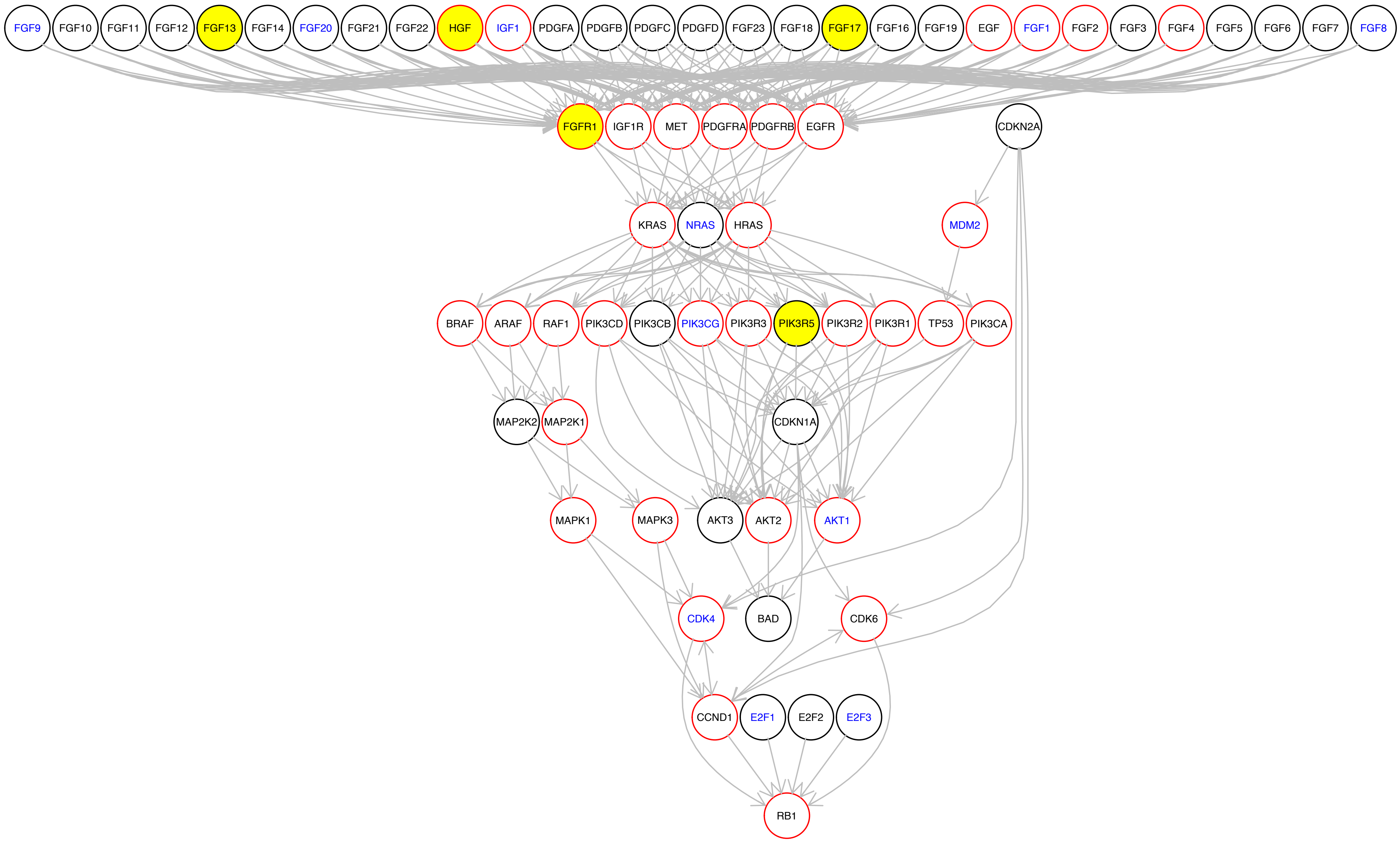

KEGG pathway = Melanoma : tumour = YURDE : Yellow Fill = gene variant, Blue Text = expression−survival association, Red Boarder = drug

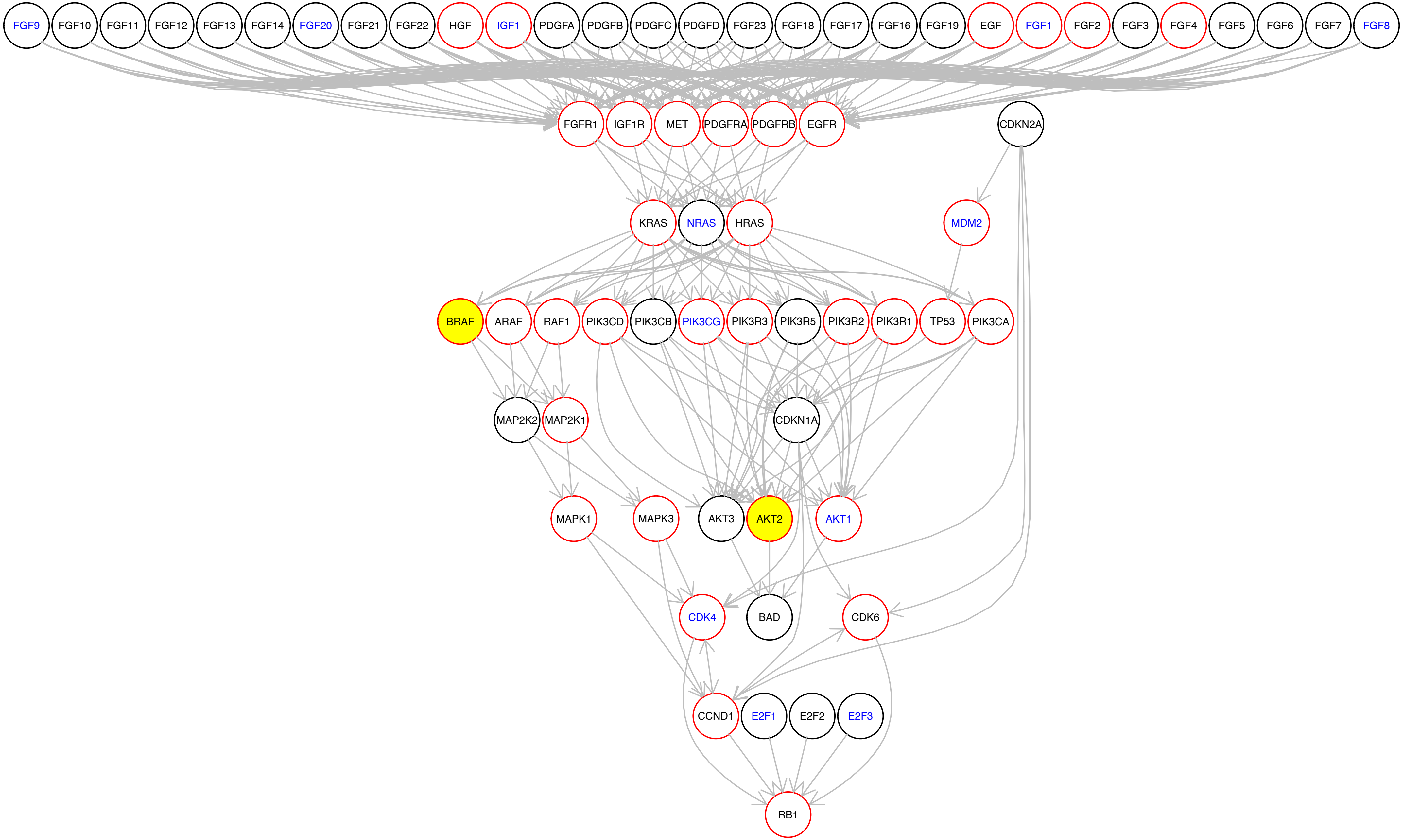

KEGG pathway = Melanoma : tumour = YURED : Yellow Fill = gene variant, Blue Text = expression−survival association, Red Boarder = drug

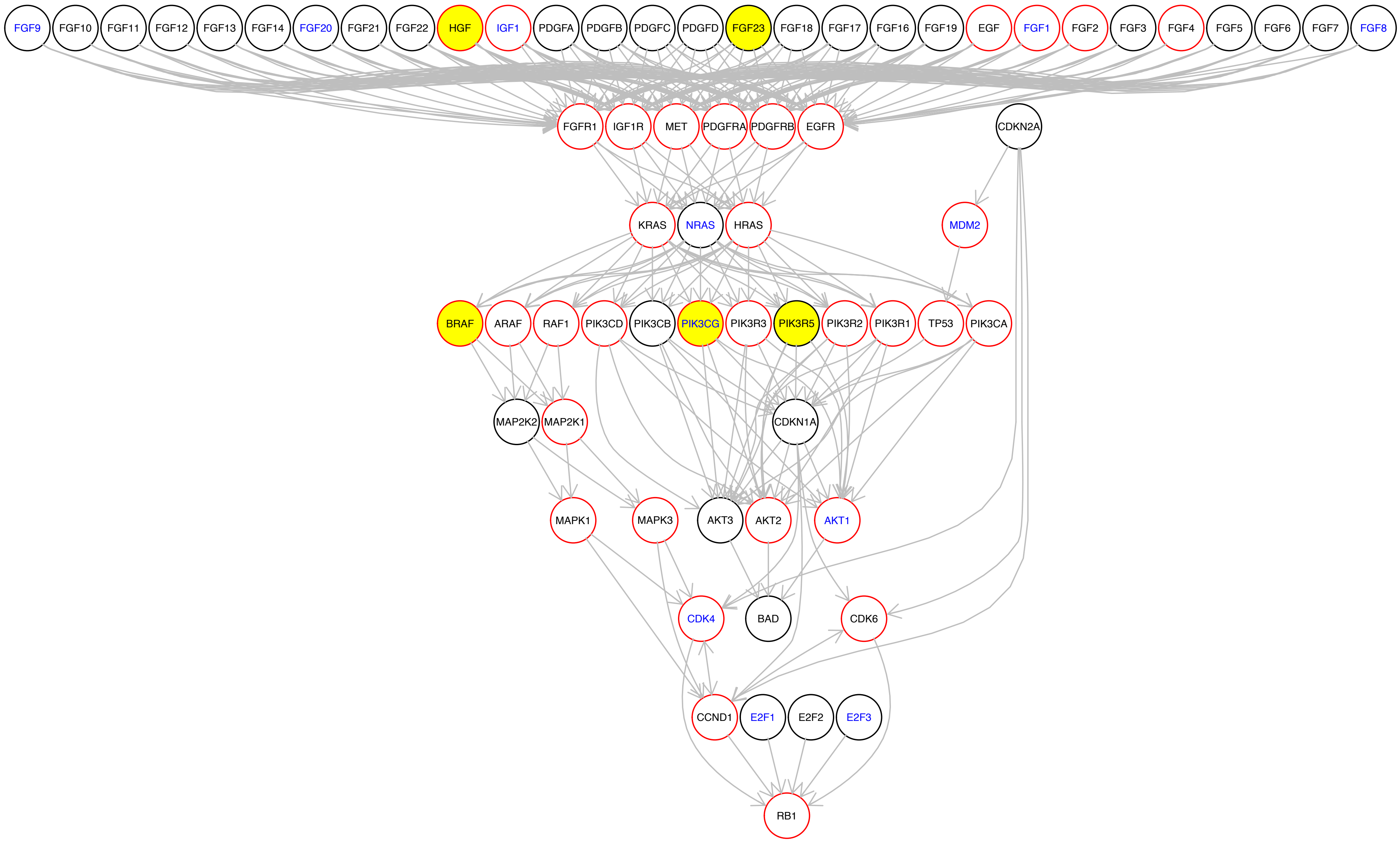

KEGG pathway = Melanoma : tumour = YURIDA : Yellow Fill = gene variant, Blue Text = expression−survival association, Red Boarder = drug

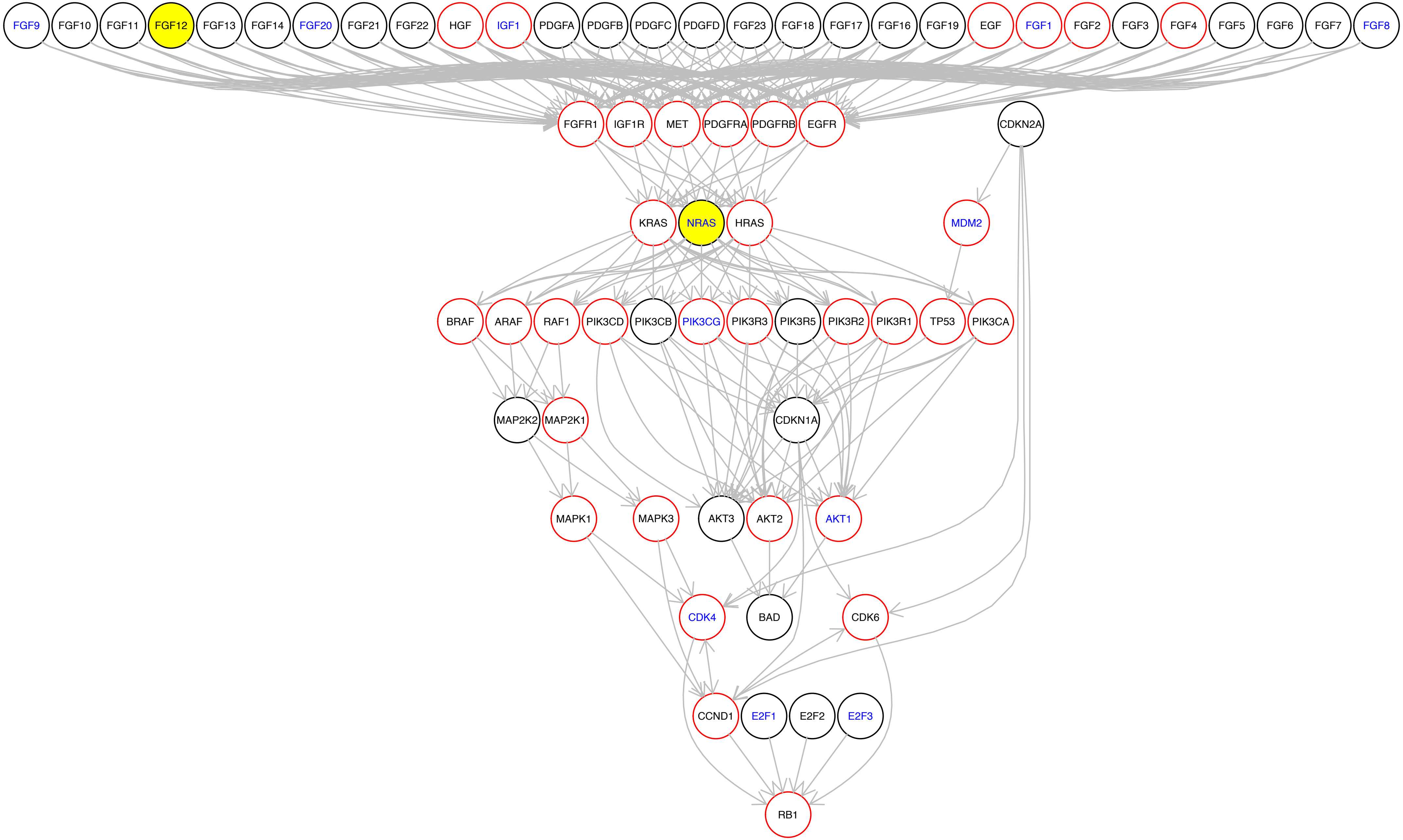

KEGG pathway = Melanoma : tumour = YURIF : Yellow Fill = gene variant, Blue Text = expression−survival association, Red Boarder = drug

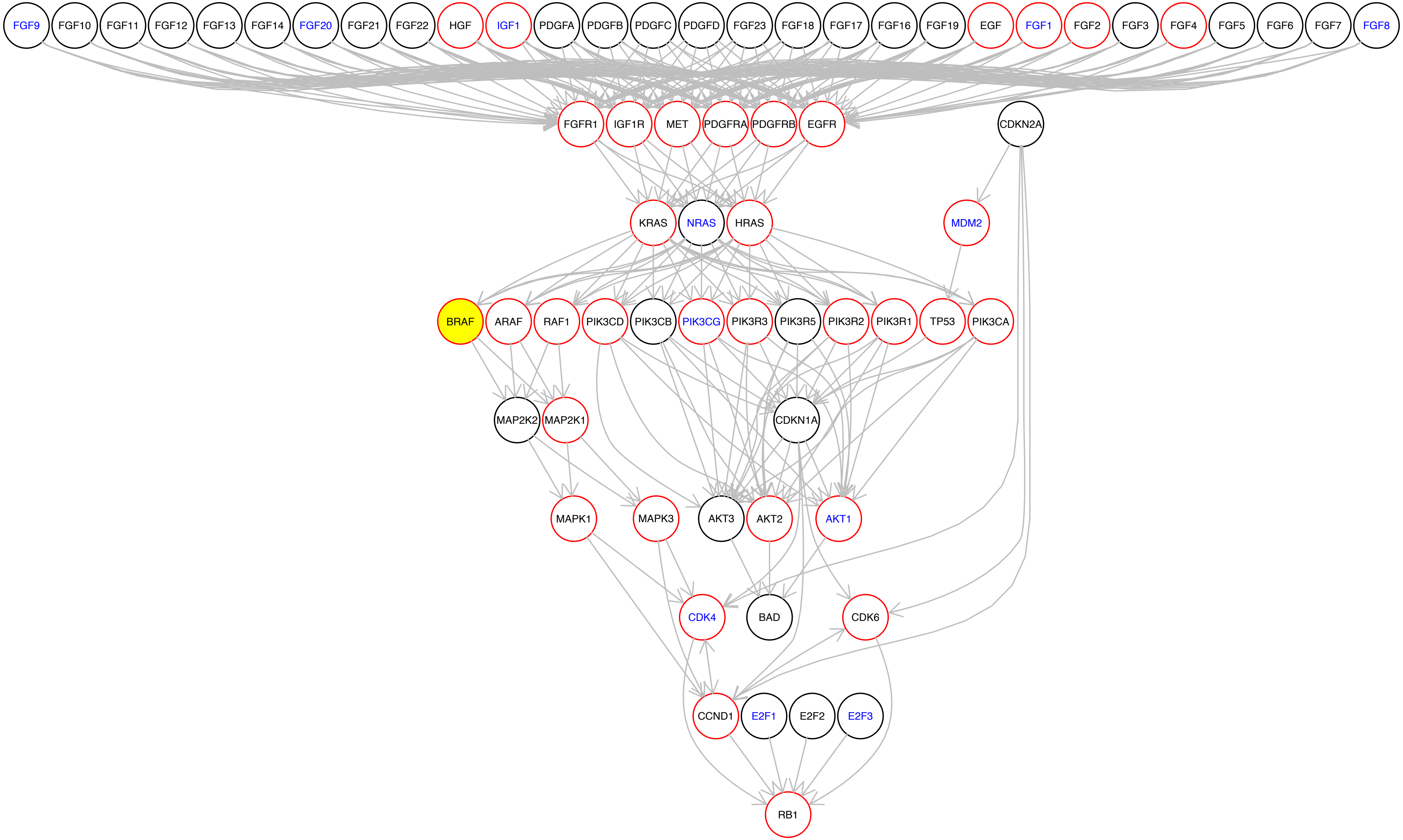

KEGG pathway = Melanoma : tumour = YURKEN : Yellow Fill = gene variant, Blue Text = expression−survival association, Red Boarder = drug

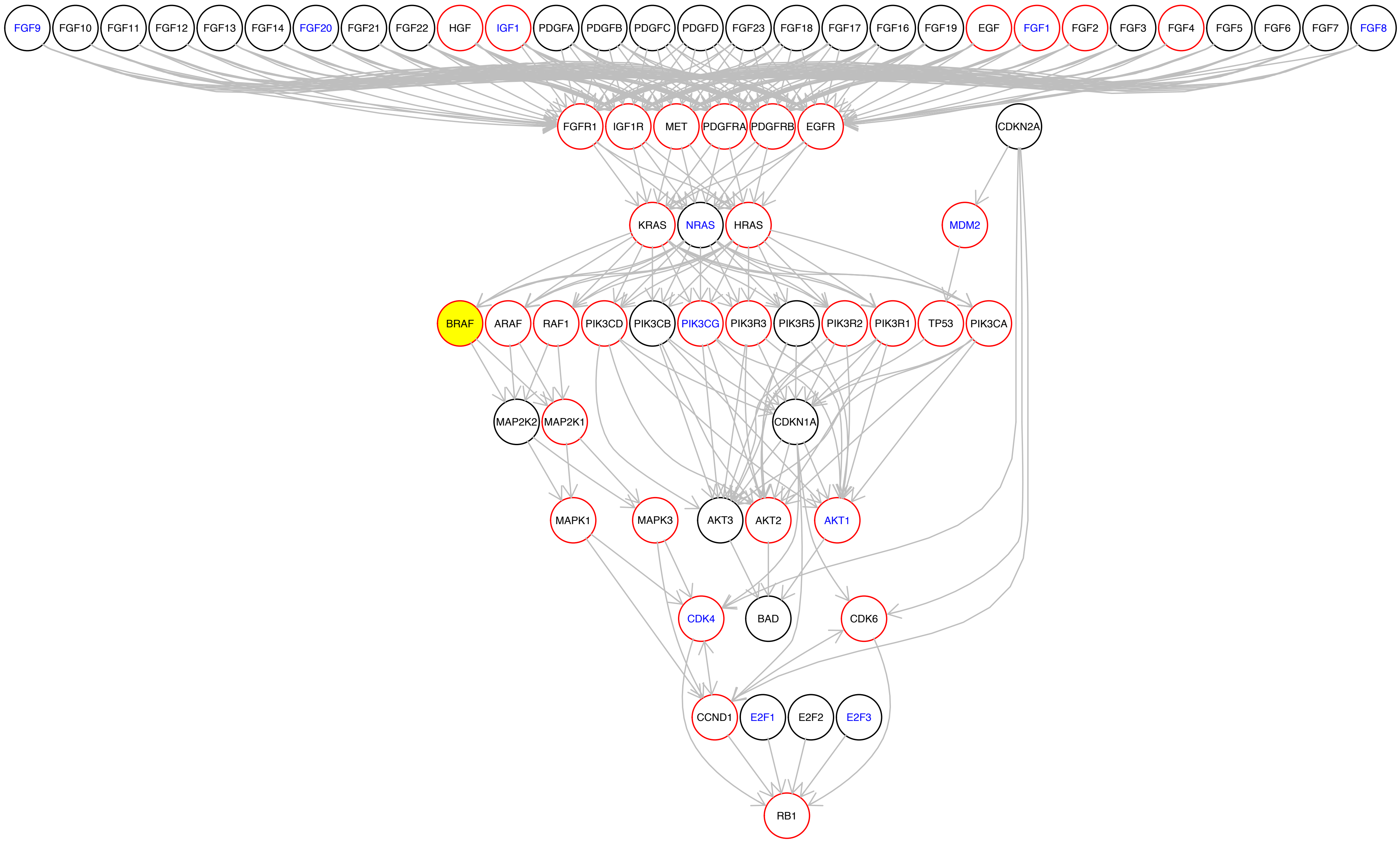

KEGG pathway = Melanoma : tumour = YUROO : Yellow Fill = gene variant, Blue Text = expression−survival association, Red Boarder = drug

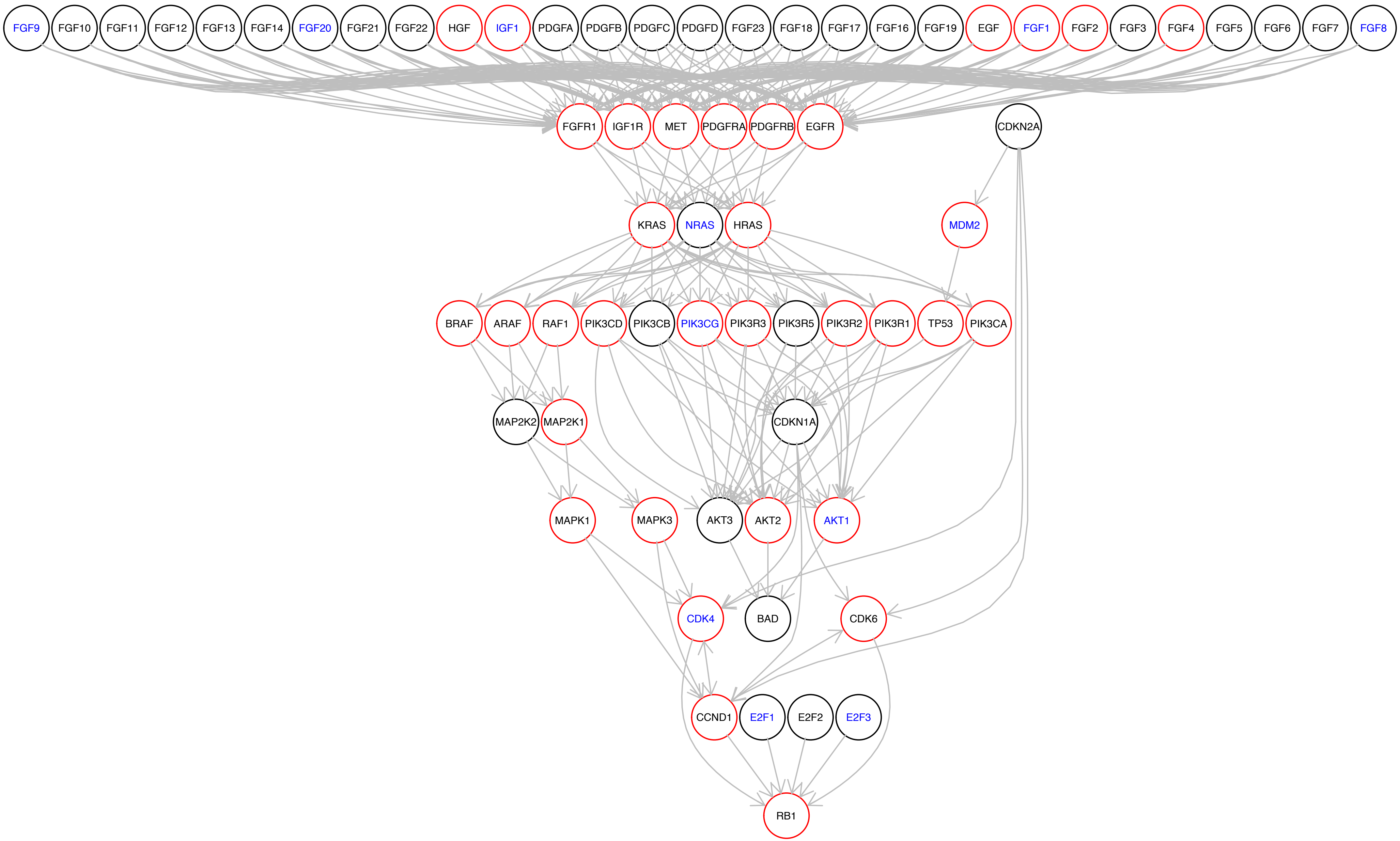

KEGG pathway = Melanoma : tumour = YURTHE : Yellow Fill = gene variant, Blue Text = expression−survival association, Red Boarder = drug

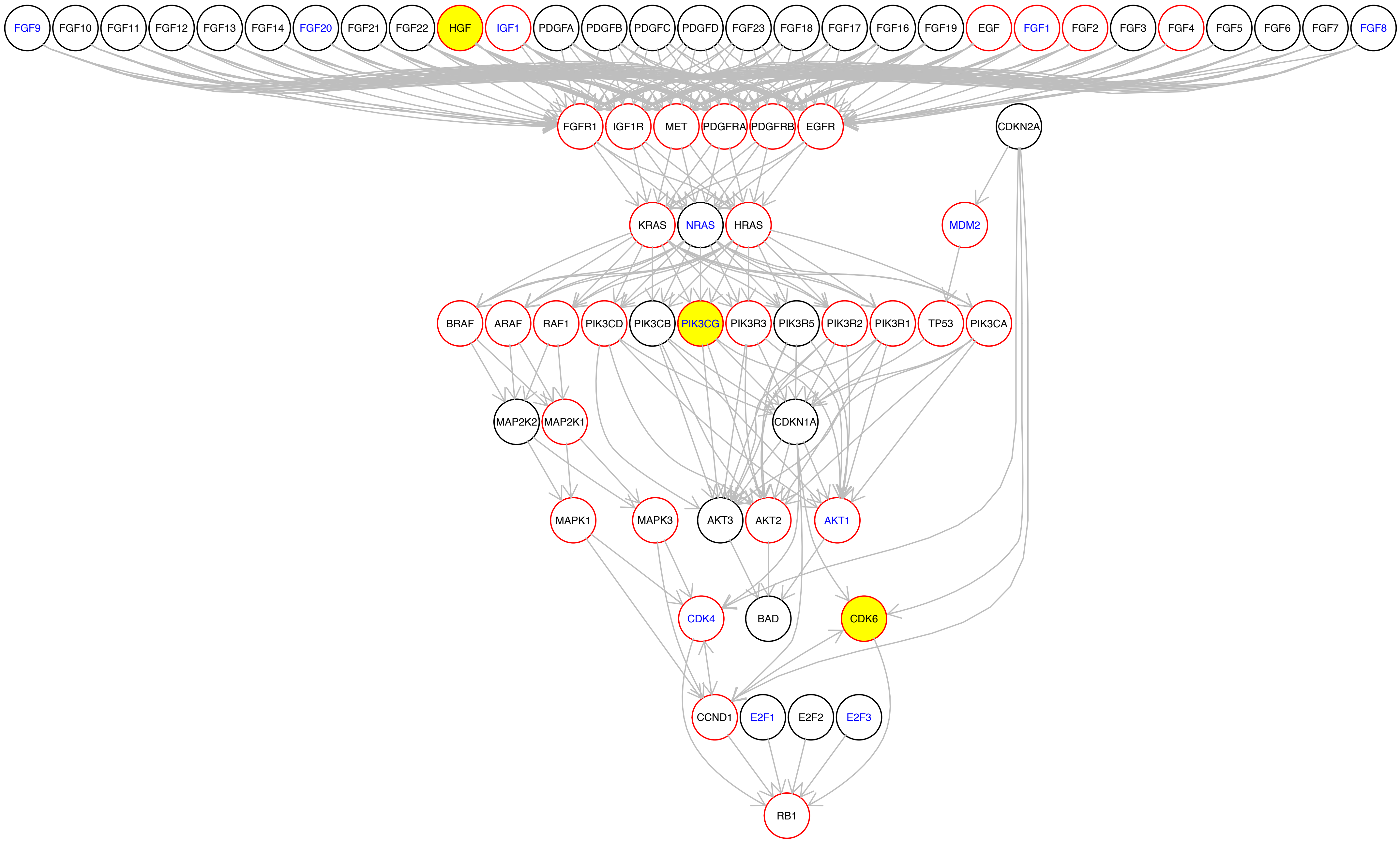

KEGG pathway = Melanoma : tumour = YURUB : Yellow Fill = gene variant, Blue Text = expression−survival association, Red Boarder = drug

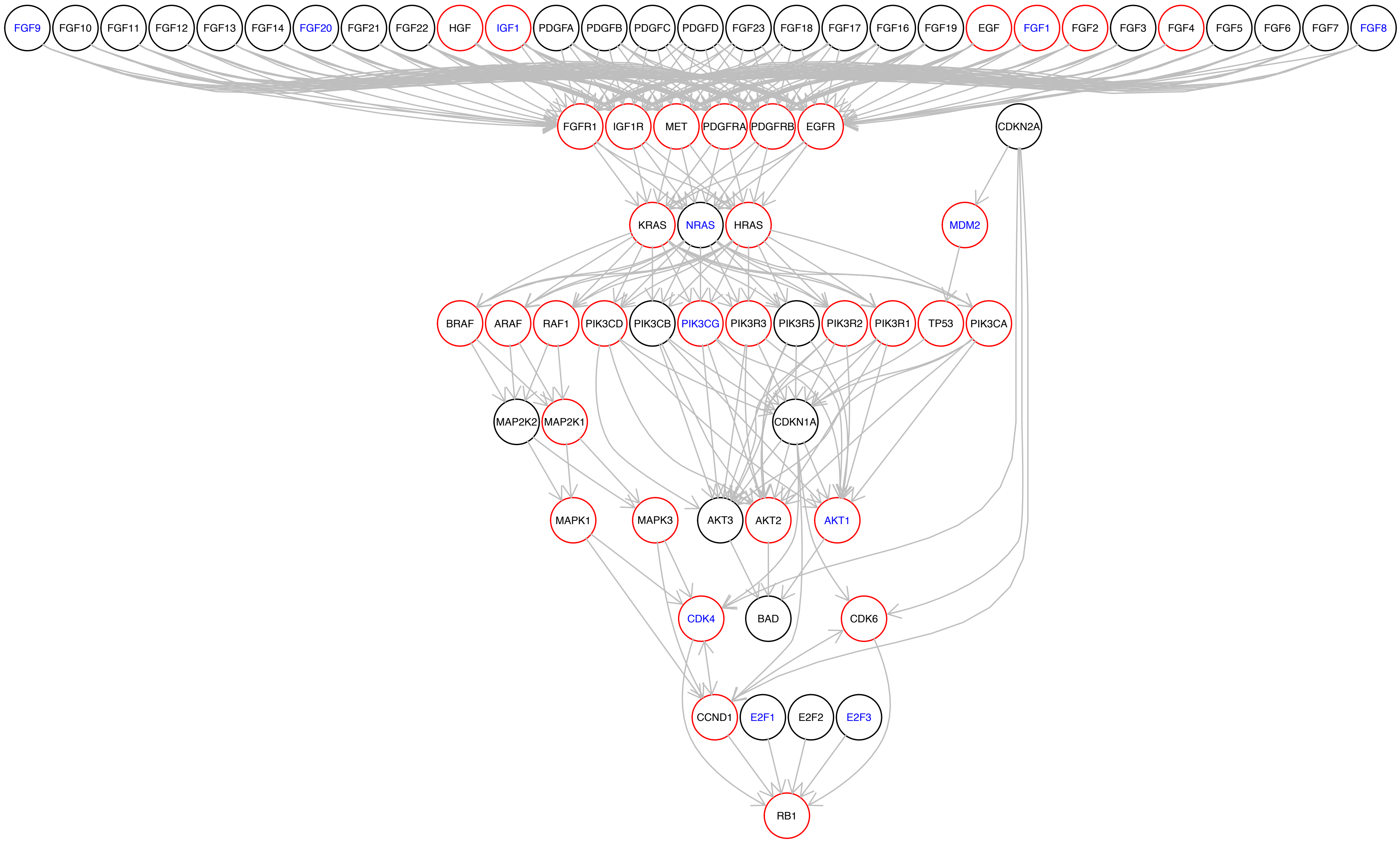

KEGG pathway = Melanoma : tumour = YURUS : Yellow Fill = gene variant, Blue Text = expression−survival association, Red Boarder = drug

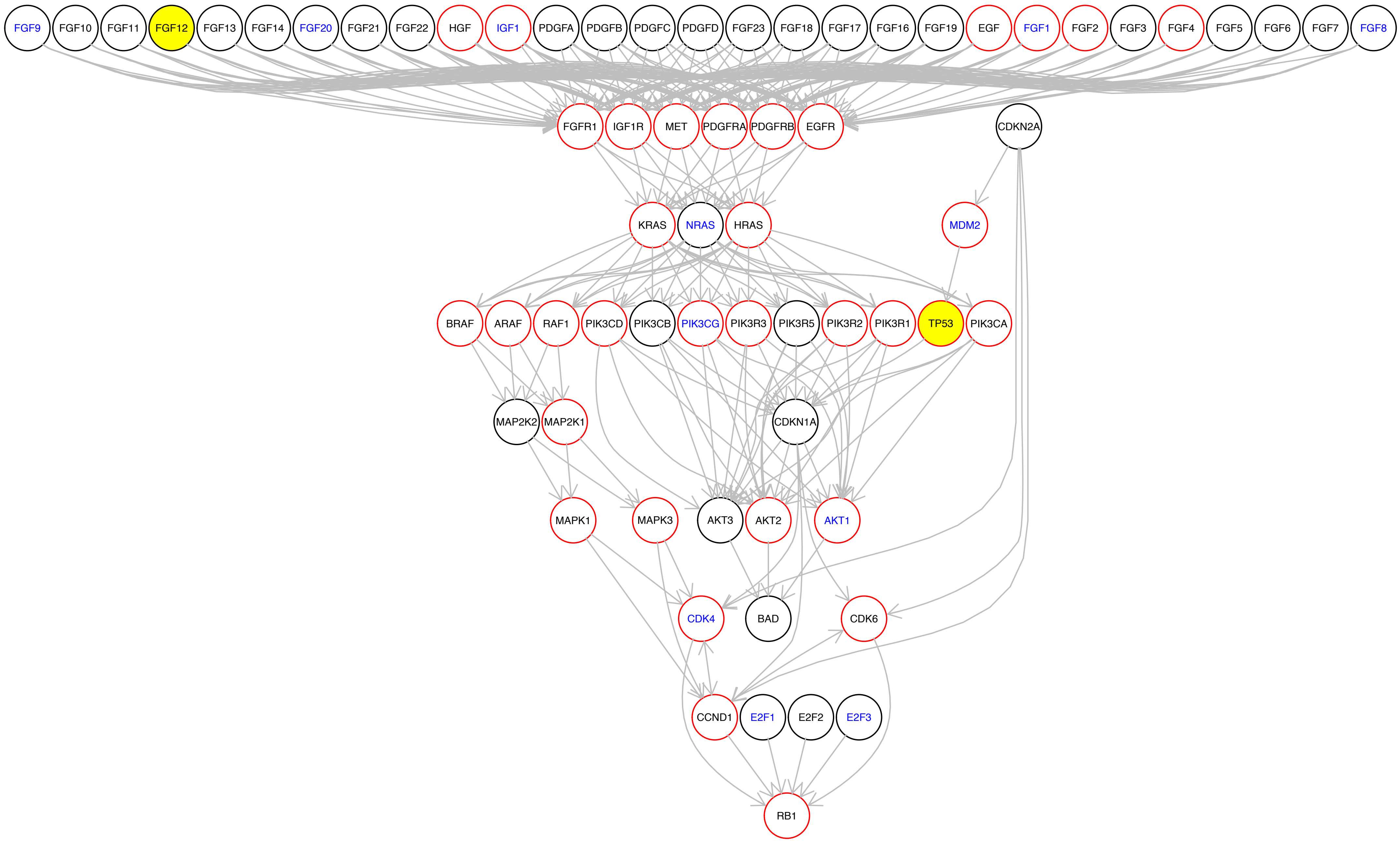

KEGG pathway = Melanoma :   tumour = YUSAG :   Yellow Fill = gene variant, Blue Text = expression−survival association, Red Boarder = drug

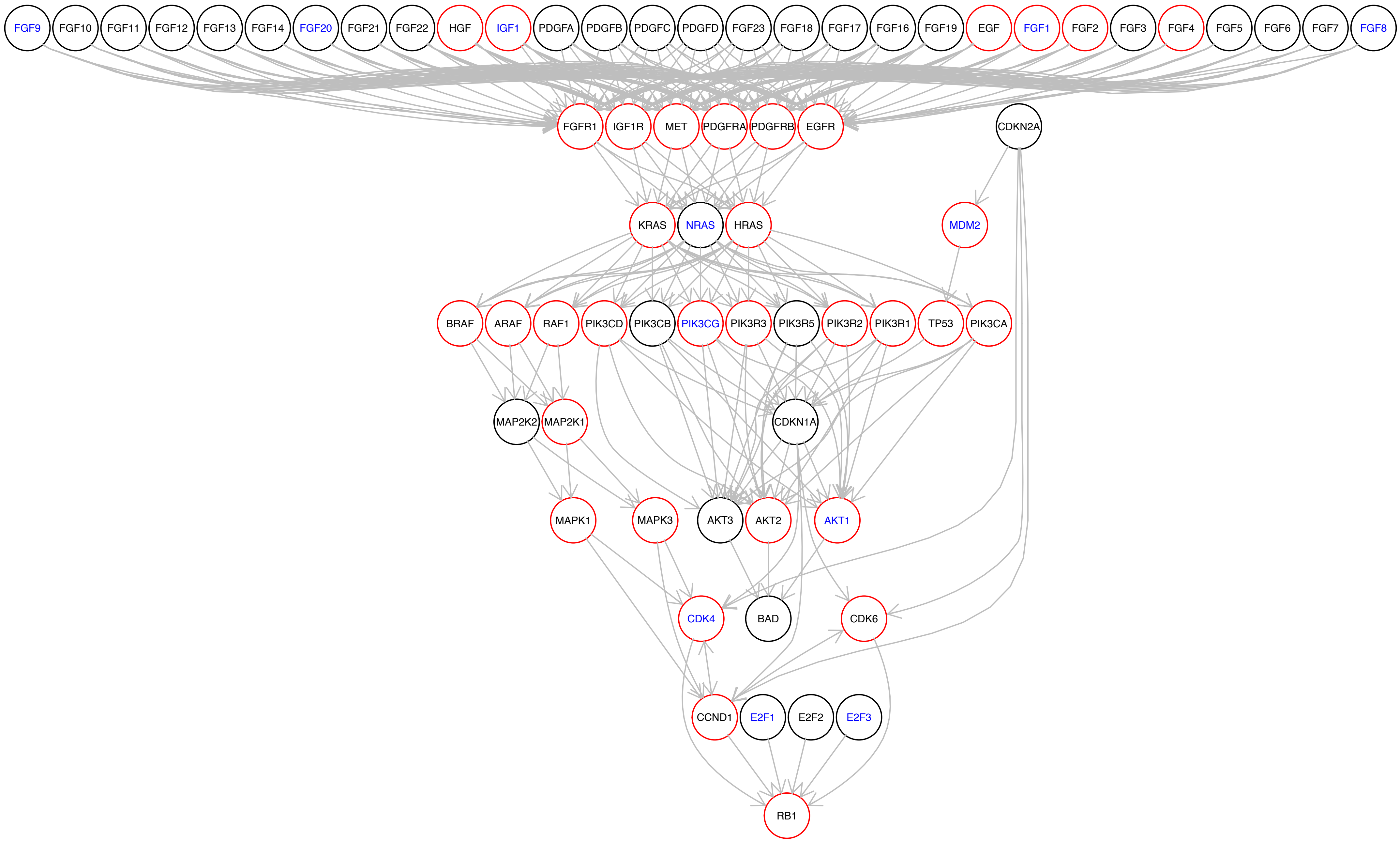

KEGG pathway = Melanoma : tumour = YUSAN : Yellow Fill = gene variant, Blue Text = expression–survival association, Red Boarder = drug

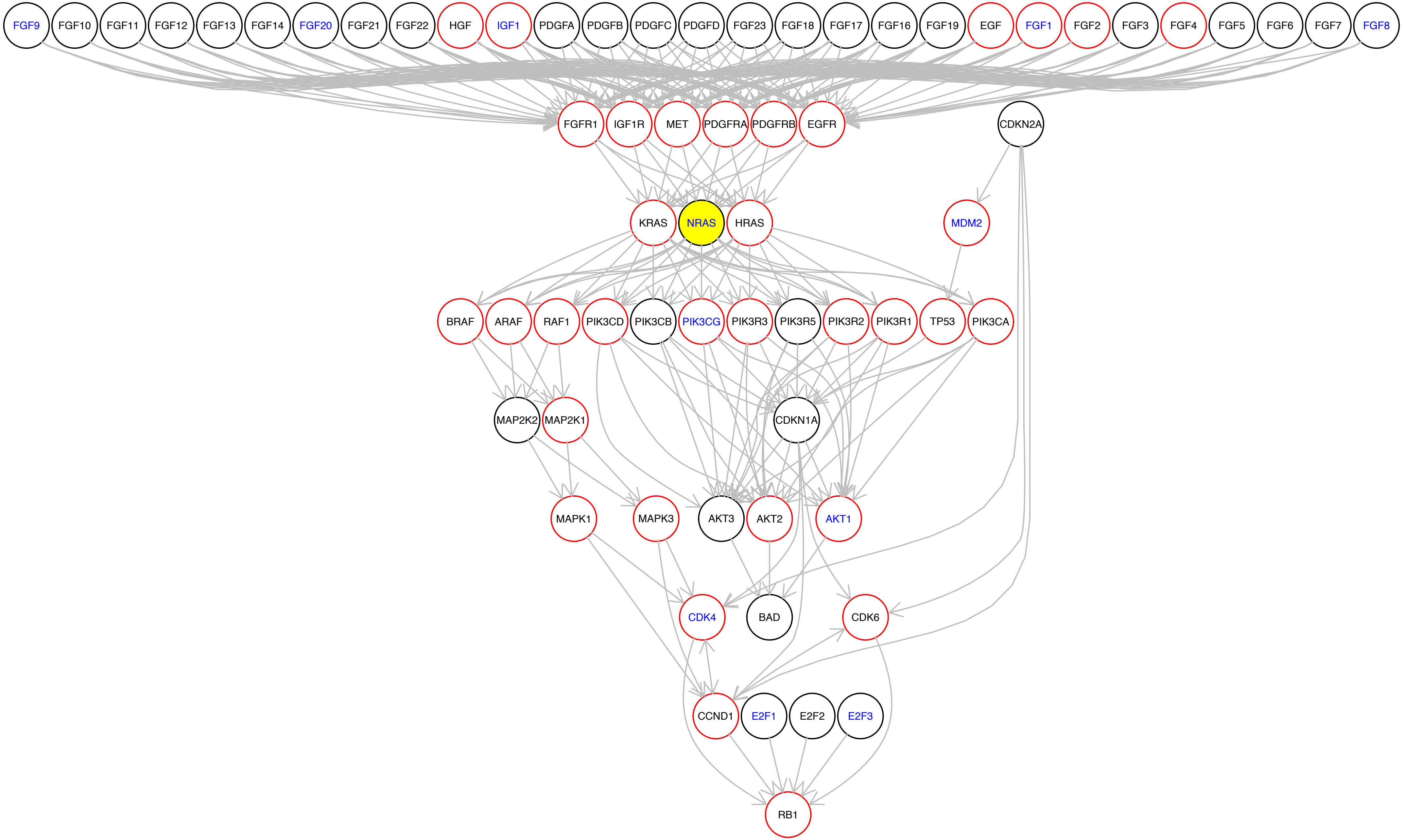

KEGG pathway = Melanoma : tumour = YUSARI : Yellow Fill = gene variant, Blue Text = expression−survival association, Red Boarder = drug

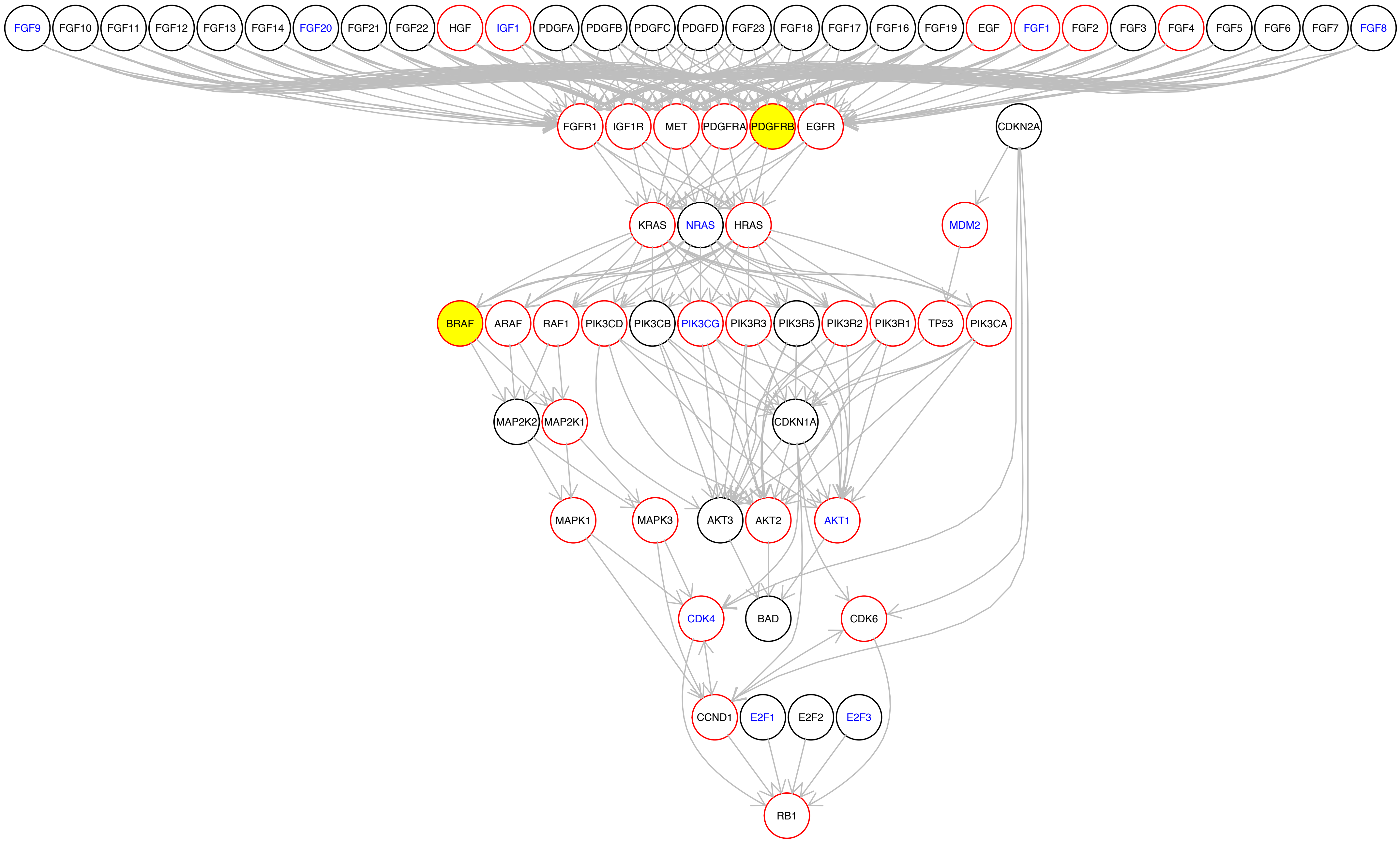

KEGG pathway = Melanoma : tumour = YUSCO : Yellow Fill = gene variant, Blue Text = expression−survival association, Red Boarder = drug

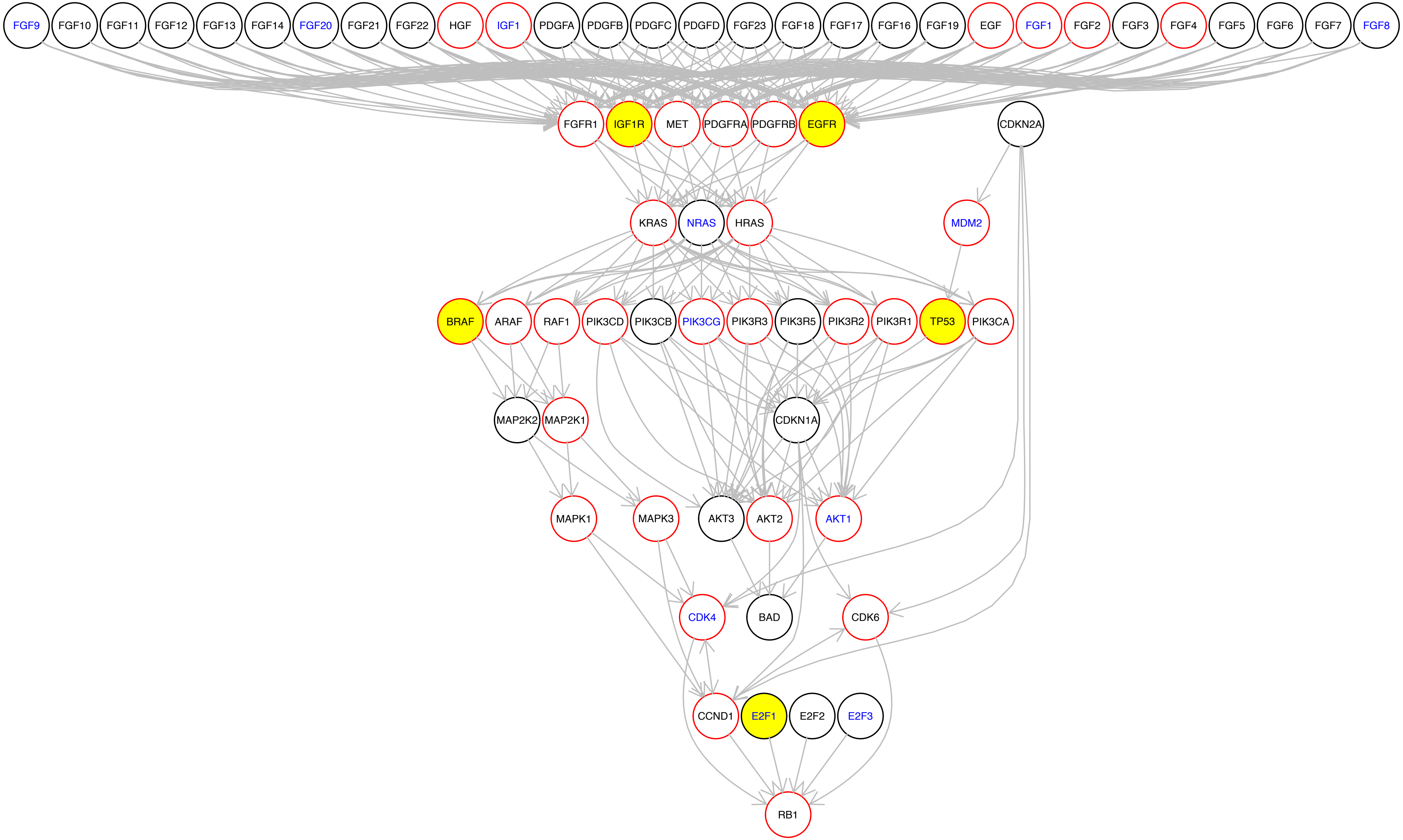

KEGG pathway = Melanoma :   tumour = YUSEL :   Yellow Fill = gene variant, Blue Text = expression−survival association, Red Boarder = drug

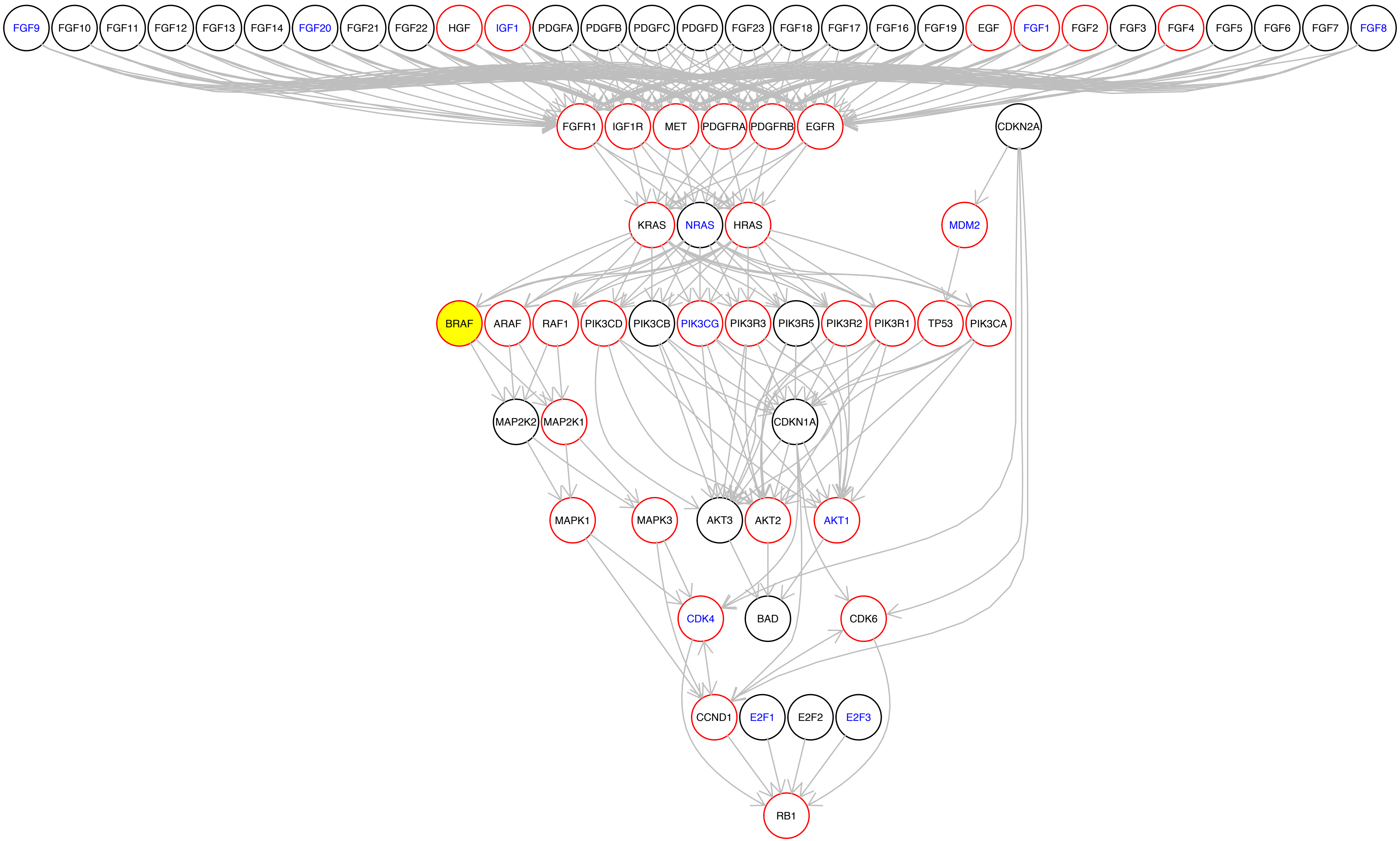

KEGG pathway = Melanoma : tumour = YUSUBA : Yellow Fill = gene variant, Blue Text = expression−survival association, Red Boarder = drug

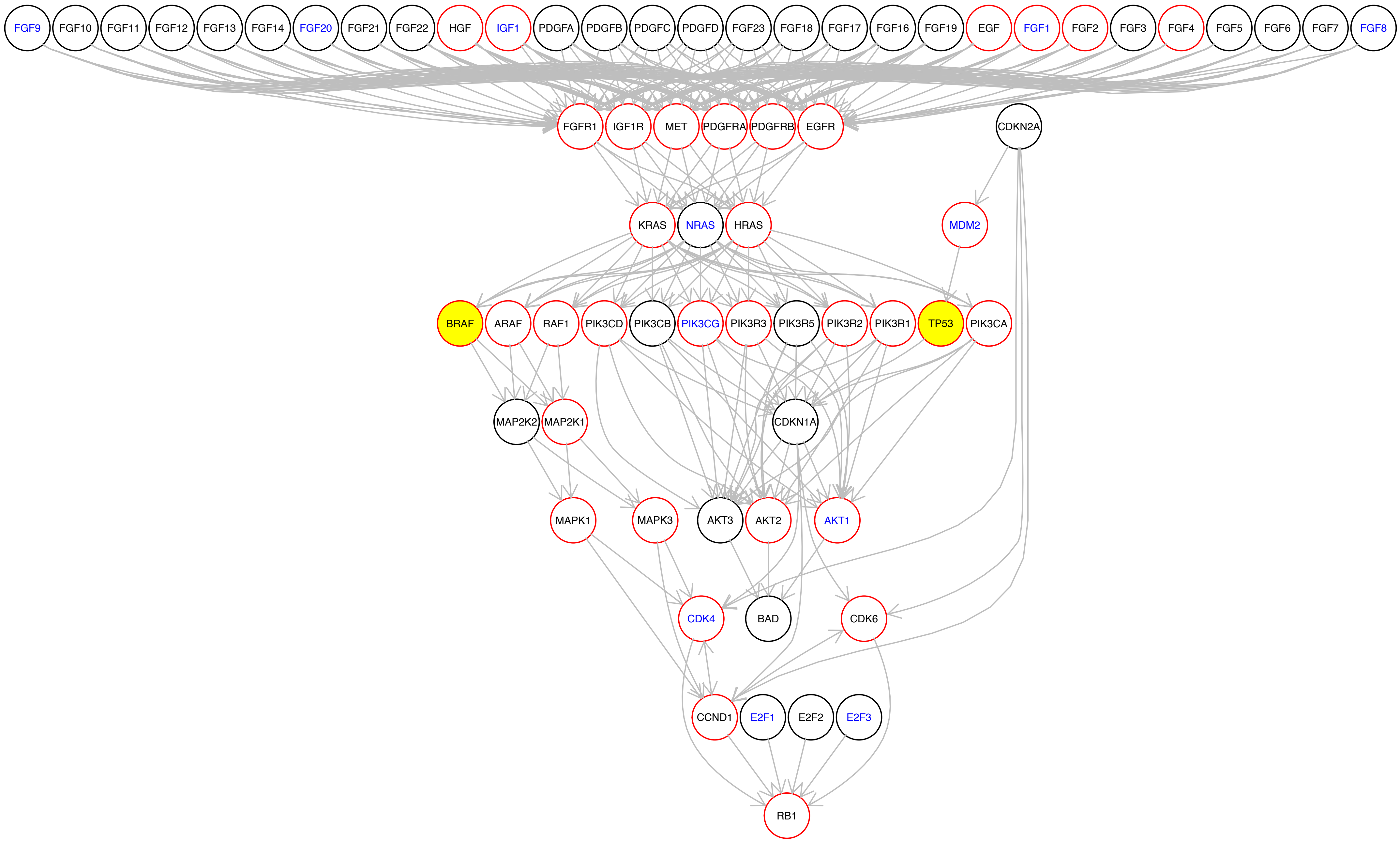

KEGG pathway = Melanoma : tumour = YUSWI : Yellow Fill = gene variant, Blue Text = expression−survival association, Red Boarder = drug

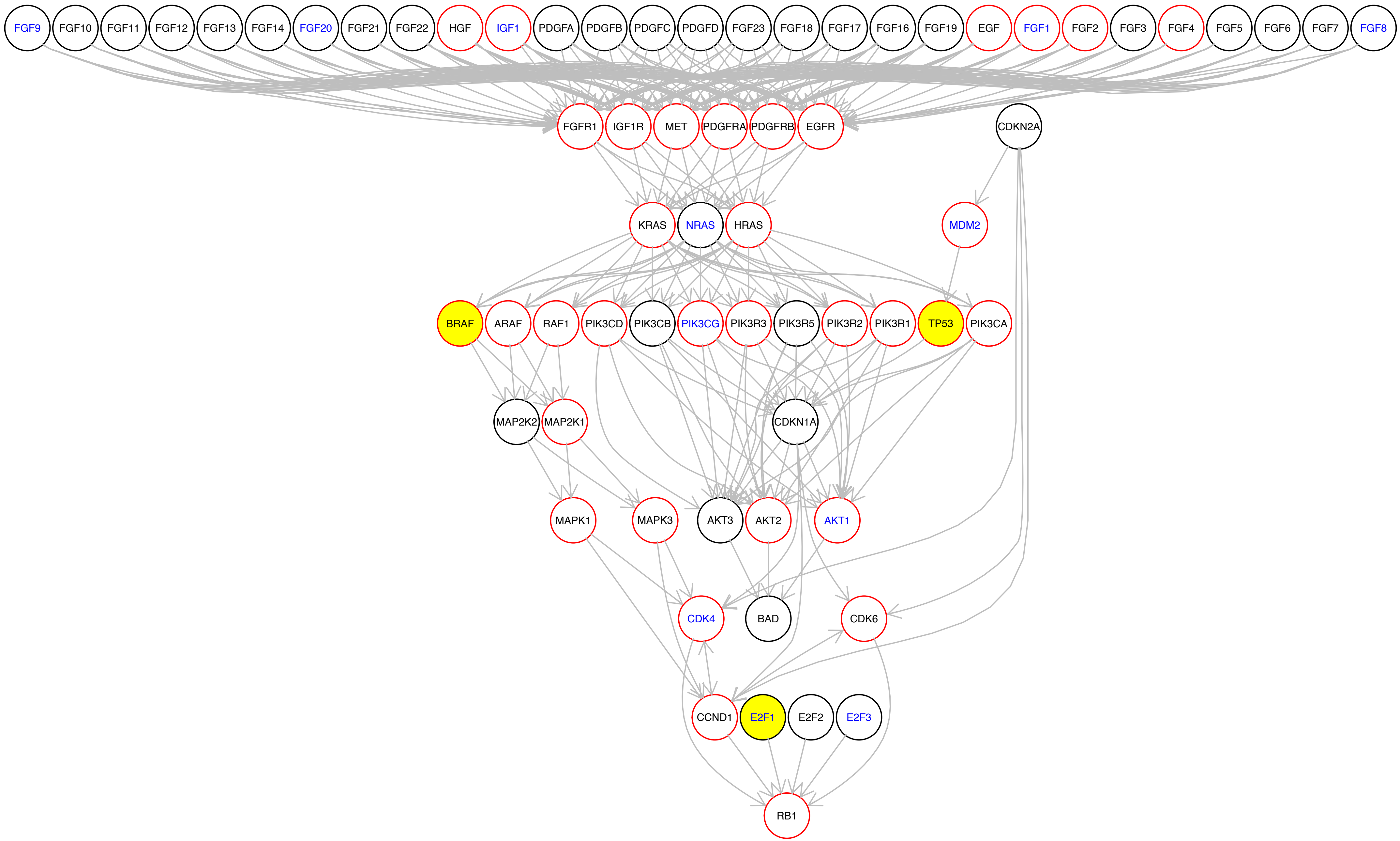

KEGG pathway = Melanoma : tumour = YUTEPA : Yellow Fill = gene variant, Blue Text = expression−survival association, Red Boarder = drug

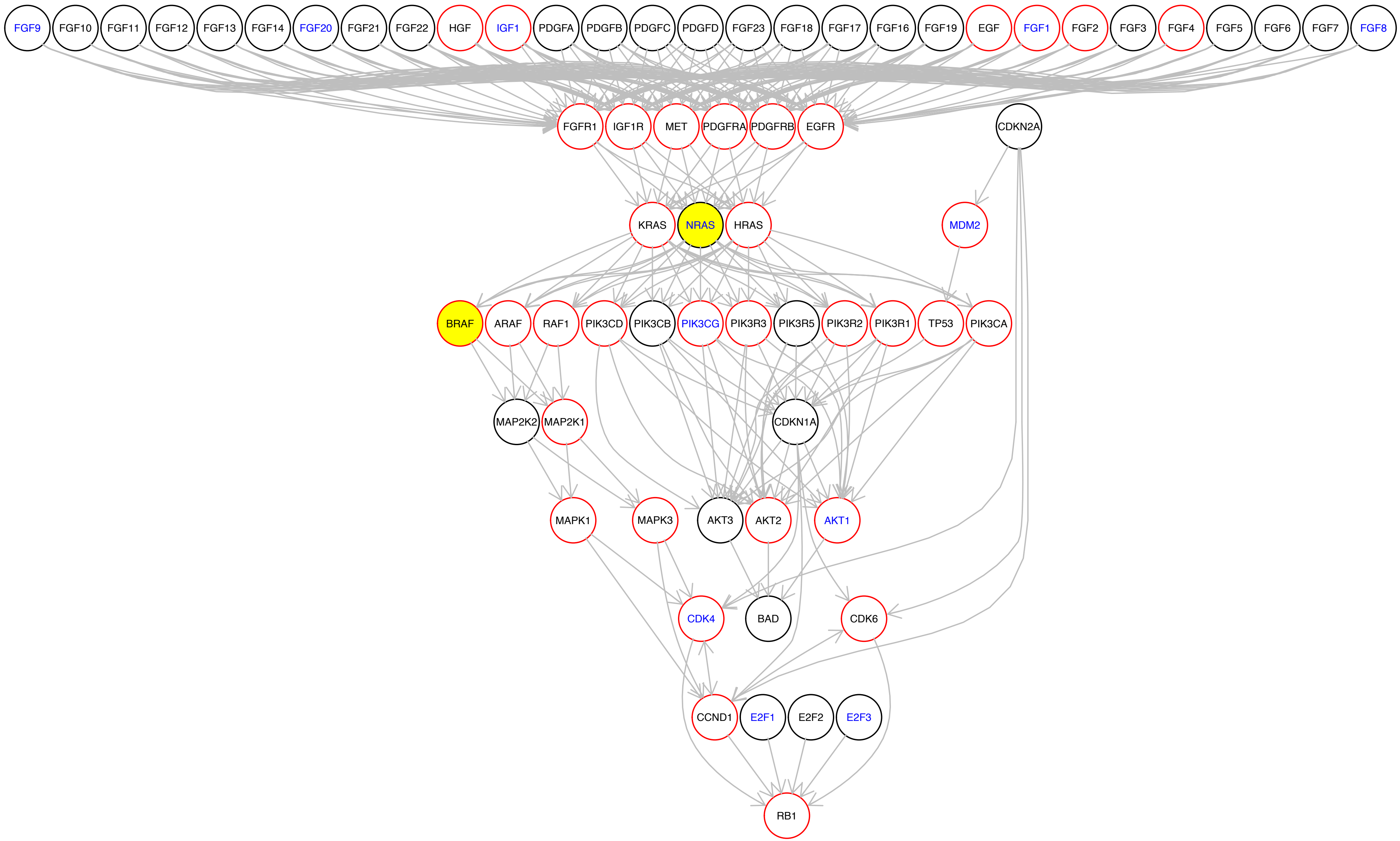

KEGG pathway = Melanoma : tumour = YUTER : Yellow Fill = gene variant, Blue Text = expression−survival association, Red Boarder = drug

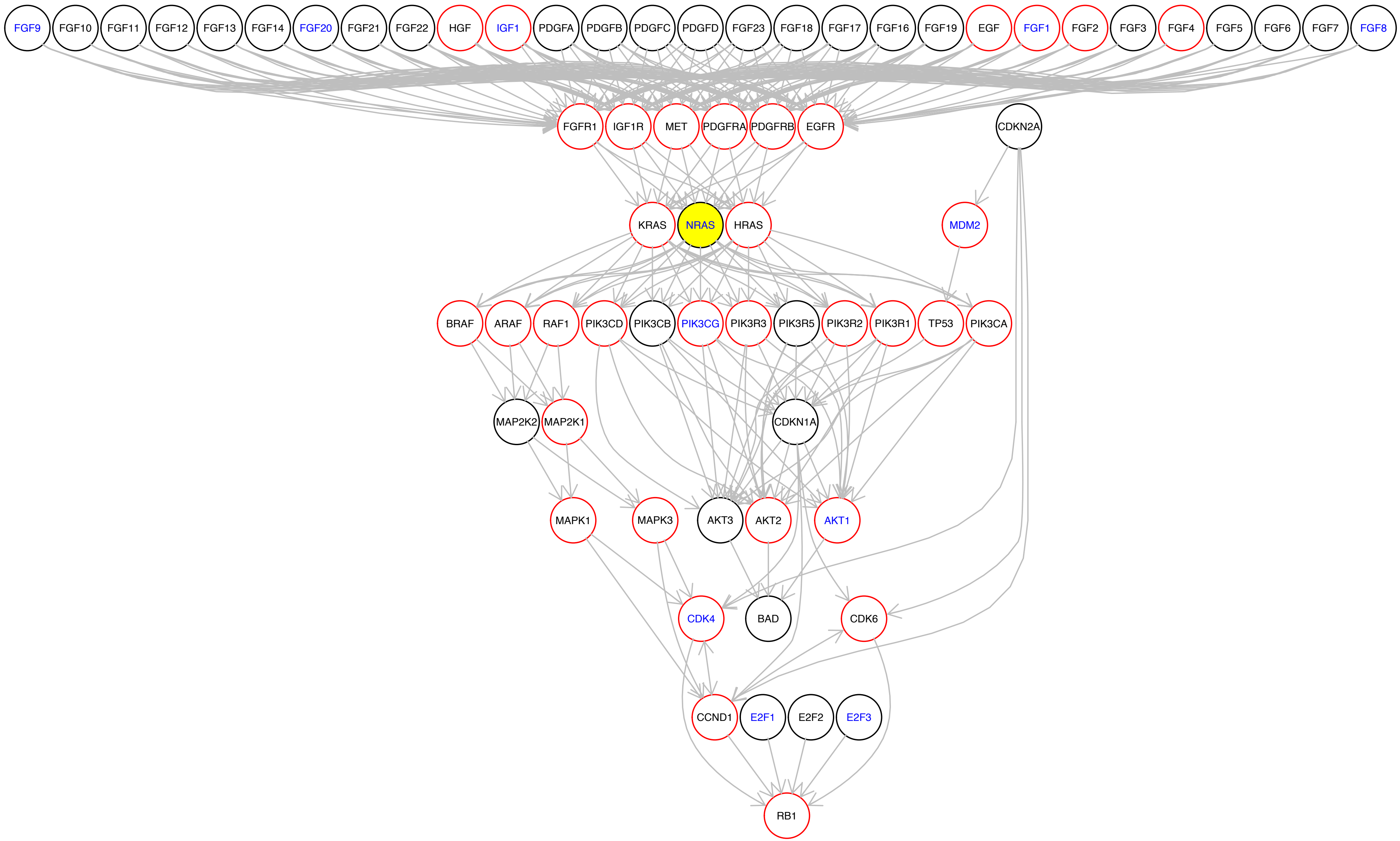

KEGG pathway = Melanoma : tumour = YUTOGS : Yellow Fill = gene variant, Blue Text = expression−survival association, Red Boarder = drug

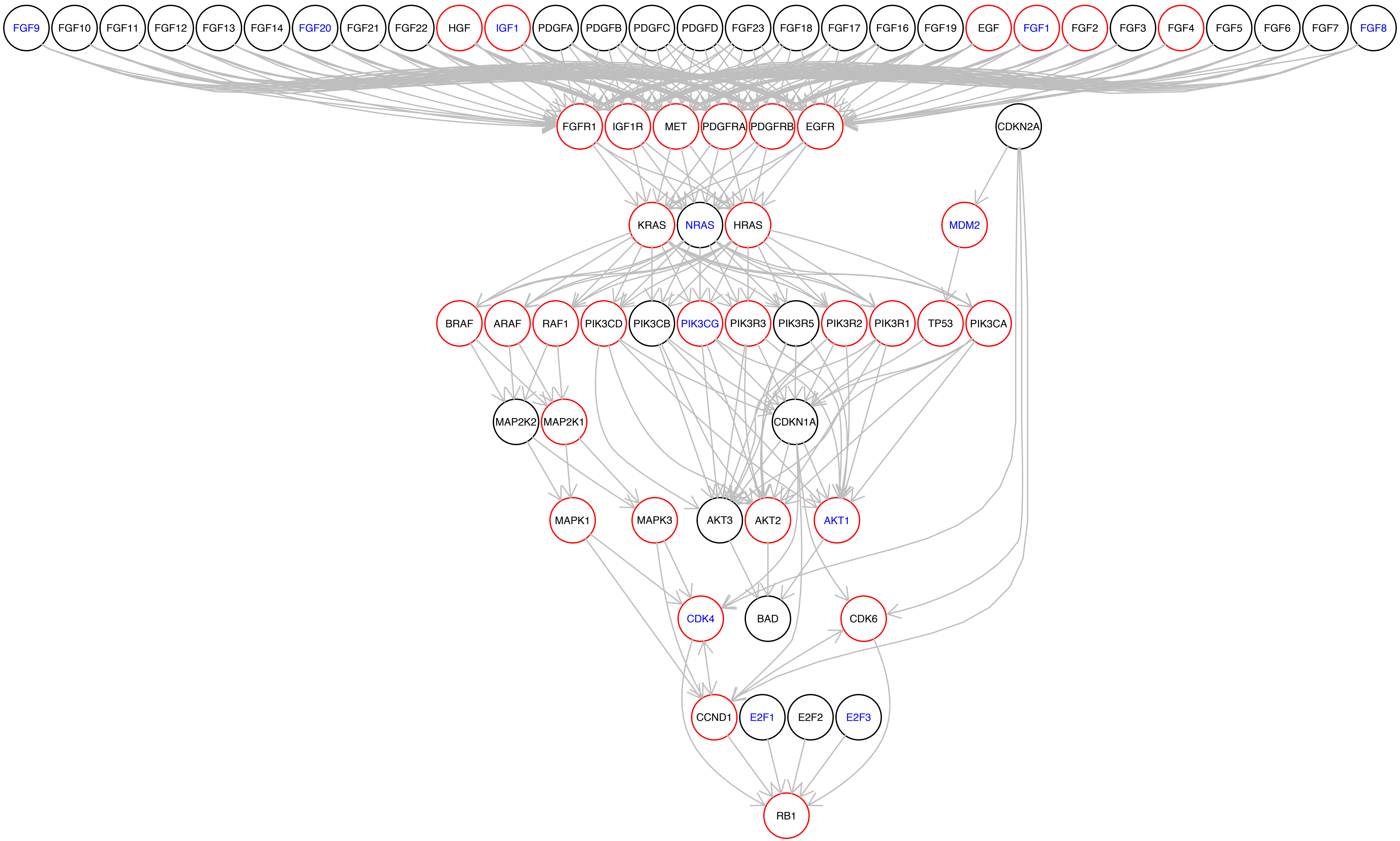

KEGG pathway = Melanoma : tumour = YUTOLL : Yellow Fill = gene variant, Blue Text = expression−survival association, Red Boarder = drug

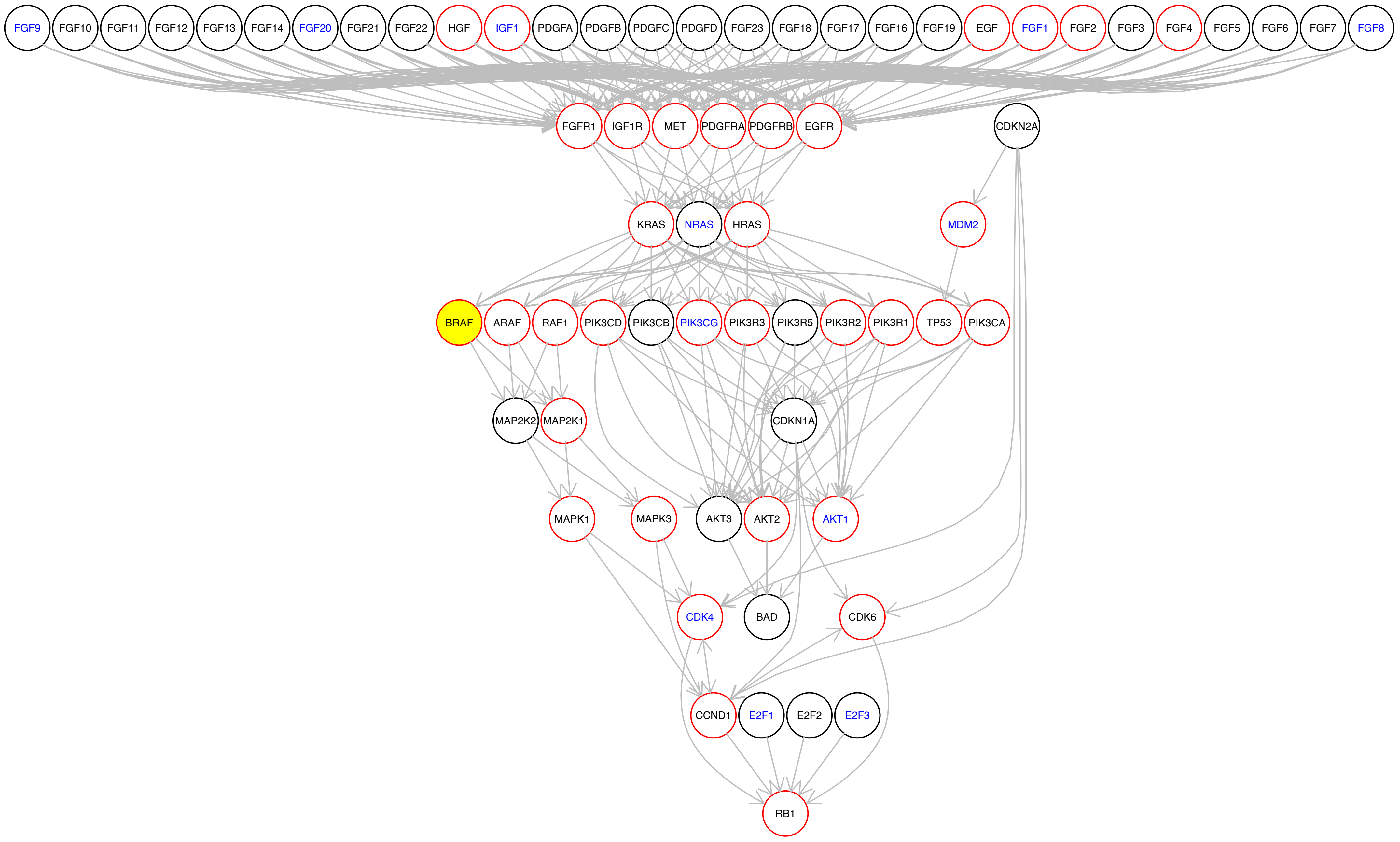

KEGG pathway = Melanoma : tumour = YUVAIL : Yellow Fill = gene variant, Blue Text = expression−survival association, Red Boarder = drug

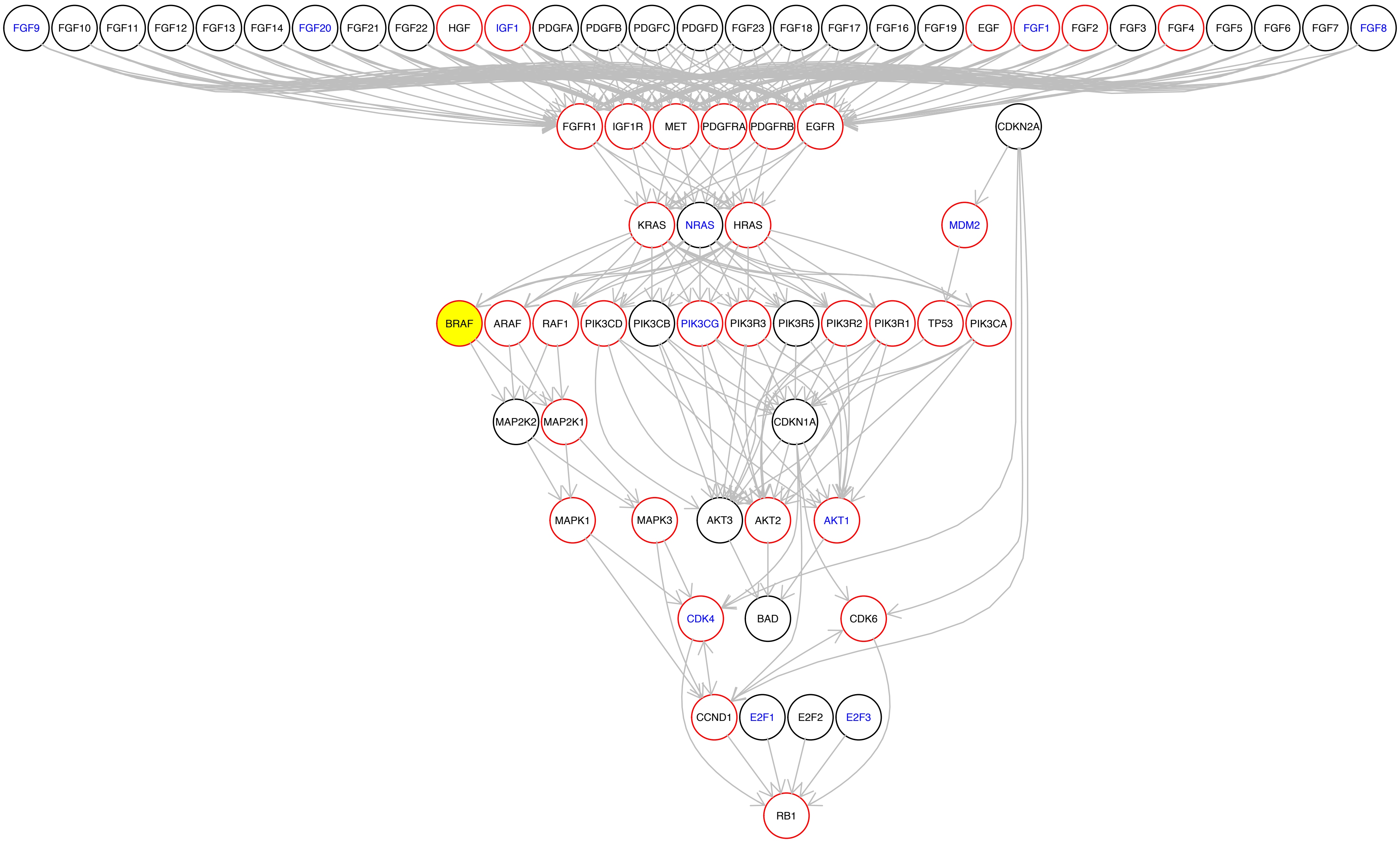

KEGG pathway = Melanoma : tumour = YUWAGE : Yellow Fill = gene variant, Blue Text = expression−survival association, Red Boarder = drug

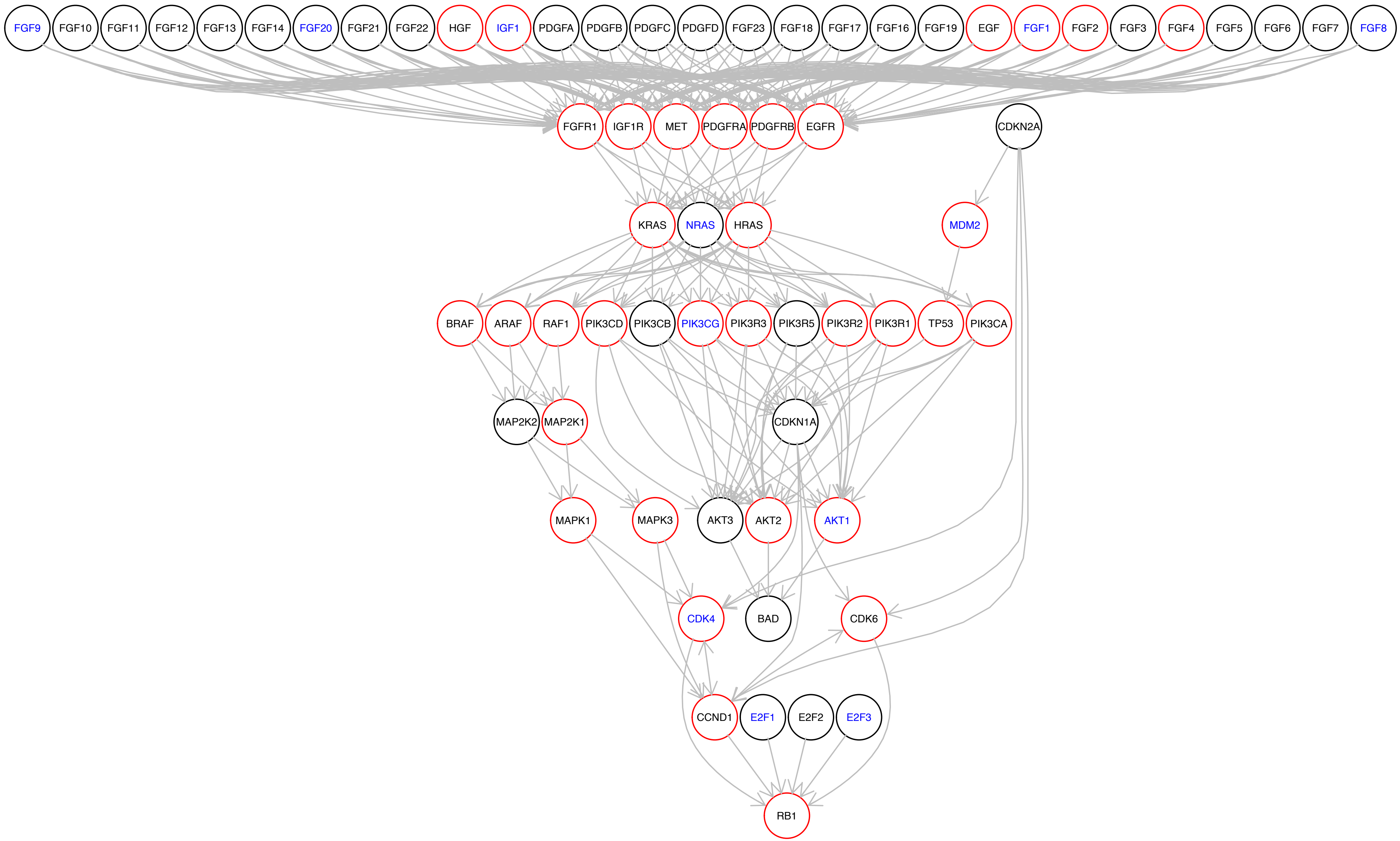

KEGG pathway = Melanoma : tumour = YUWALI : Yellow Fill = gene variant, Blue Text = expression−survival association, Red Boarder = drug

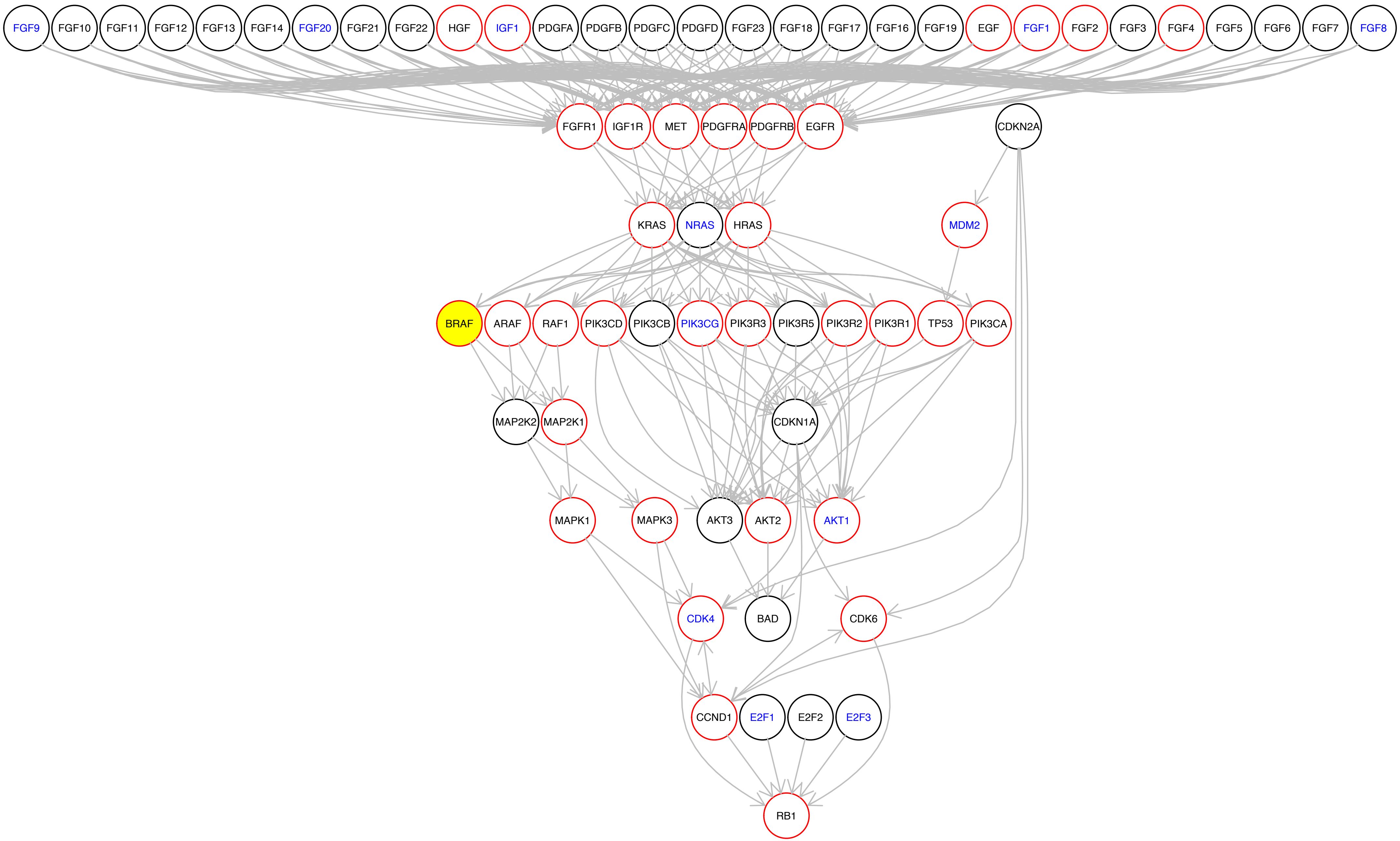

KEGG pathway = Melanoma : tumour = YUWAND : Yellow Fill = gene variant, Blue Text = expression–survival association, Red Boarder = drug

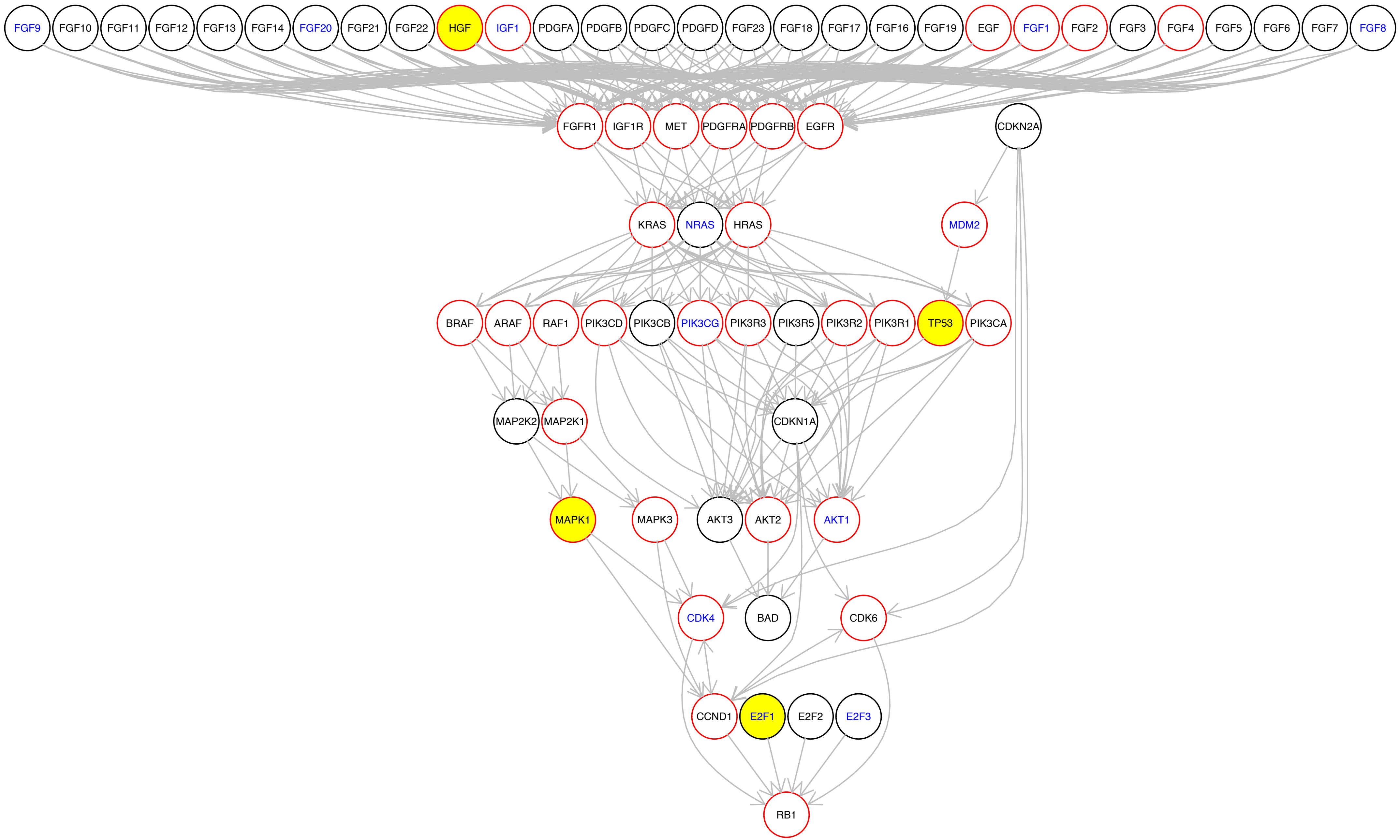

KEGG pathway = Melanoma : tumour = YUWHIM : Yellow Fill = gene variant, Blue Text = expression−survival association, Red Boarder = drug

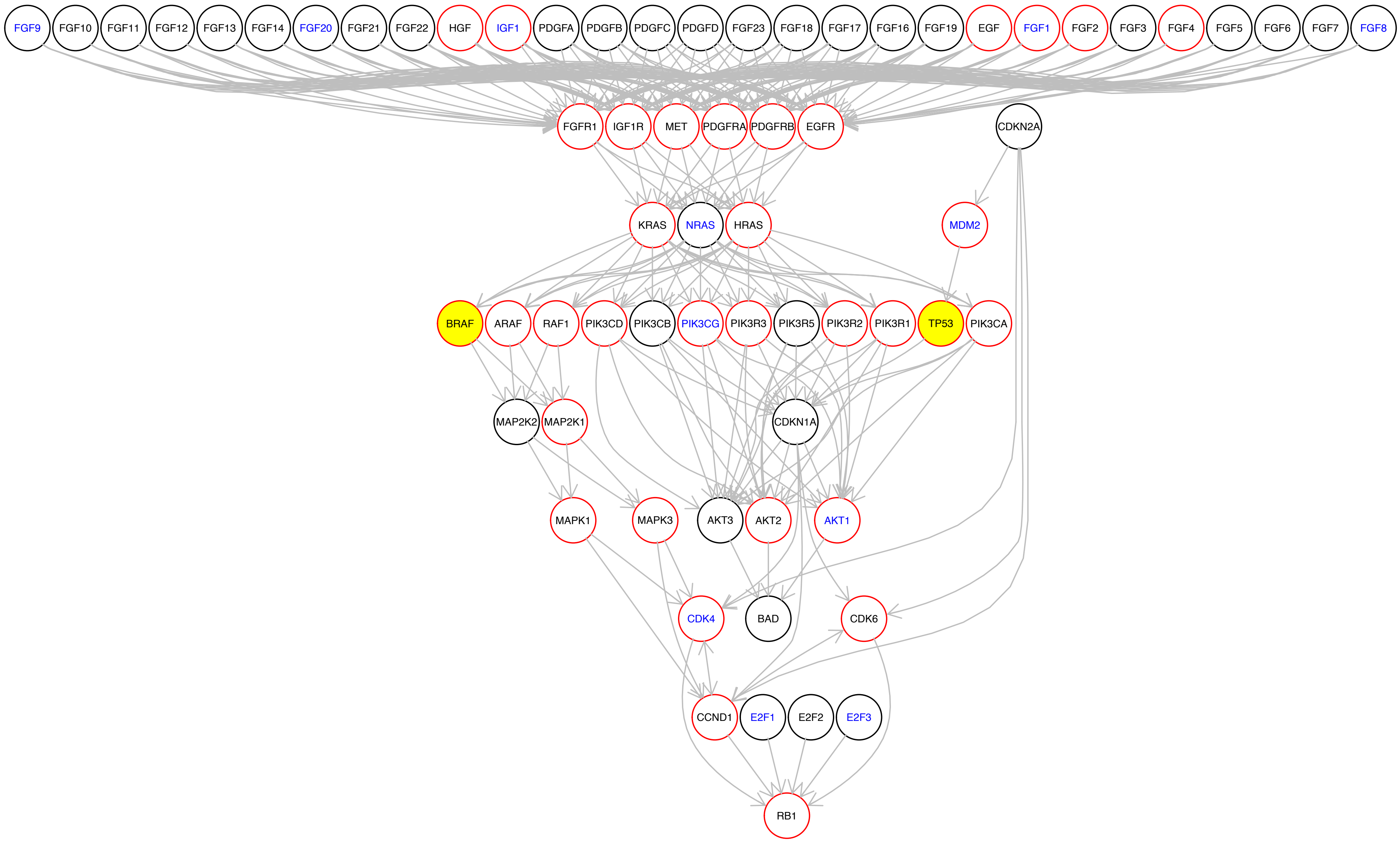

KEGG pathway = Melanoma : tumour = YUZEAL : Yellow Fill = gene variant, Blue Text = expression−survival association, Red Boarder = drug

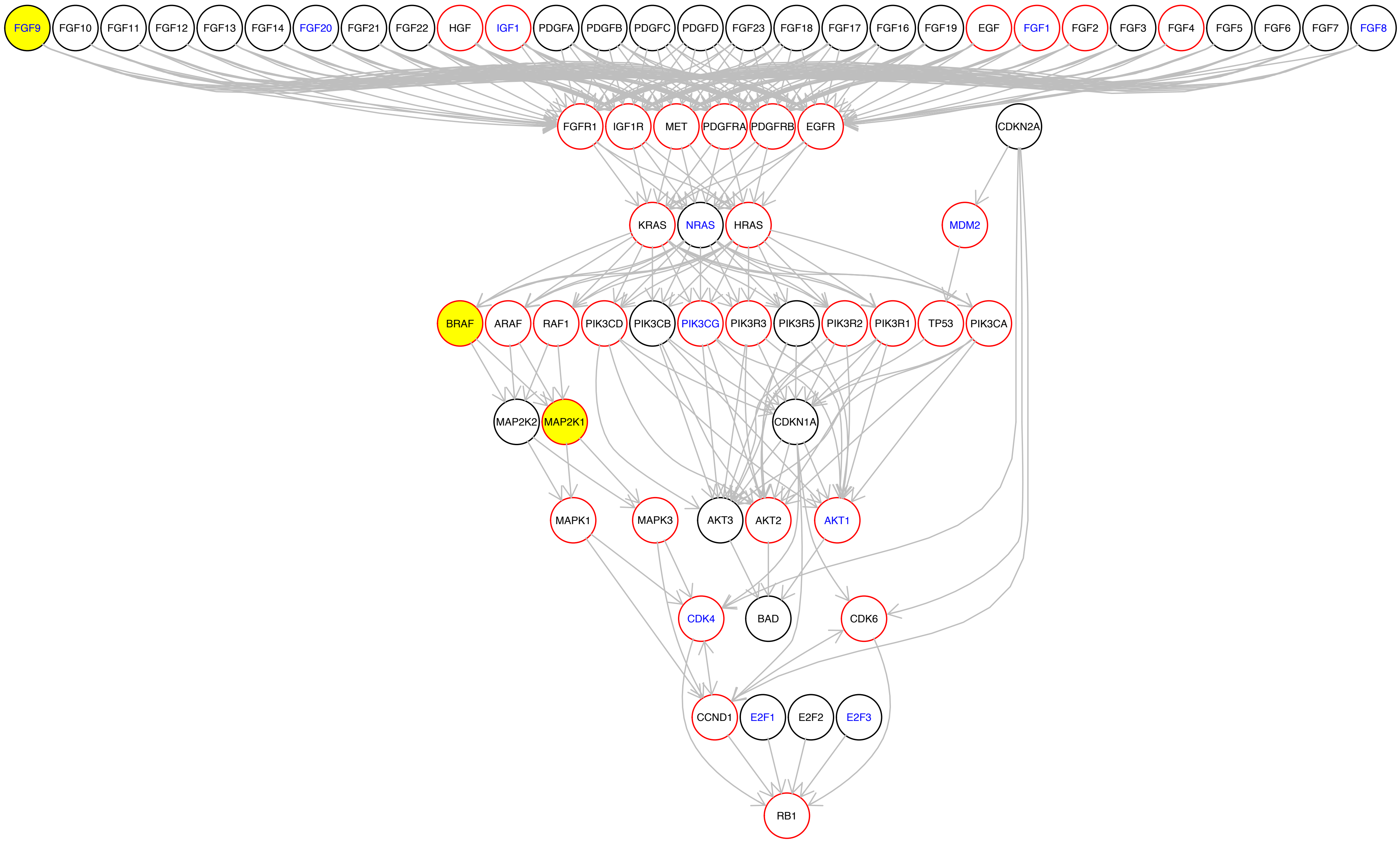

KEGG pathway = Melanoma : tumour = YUZEST : Yellow Fill = gene variant, Blue Text = expression–survival association, Red Boarder = drug

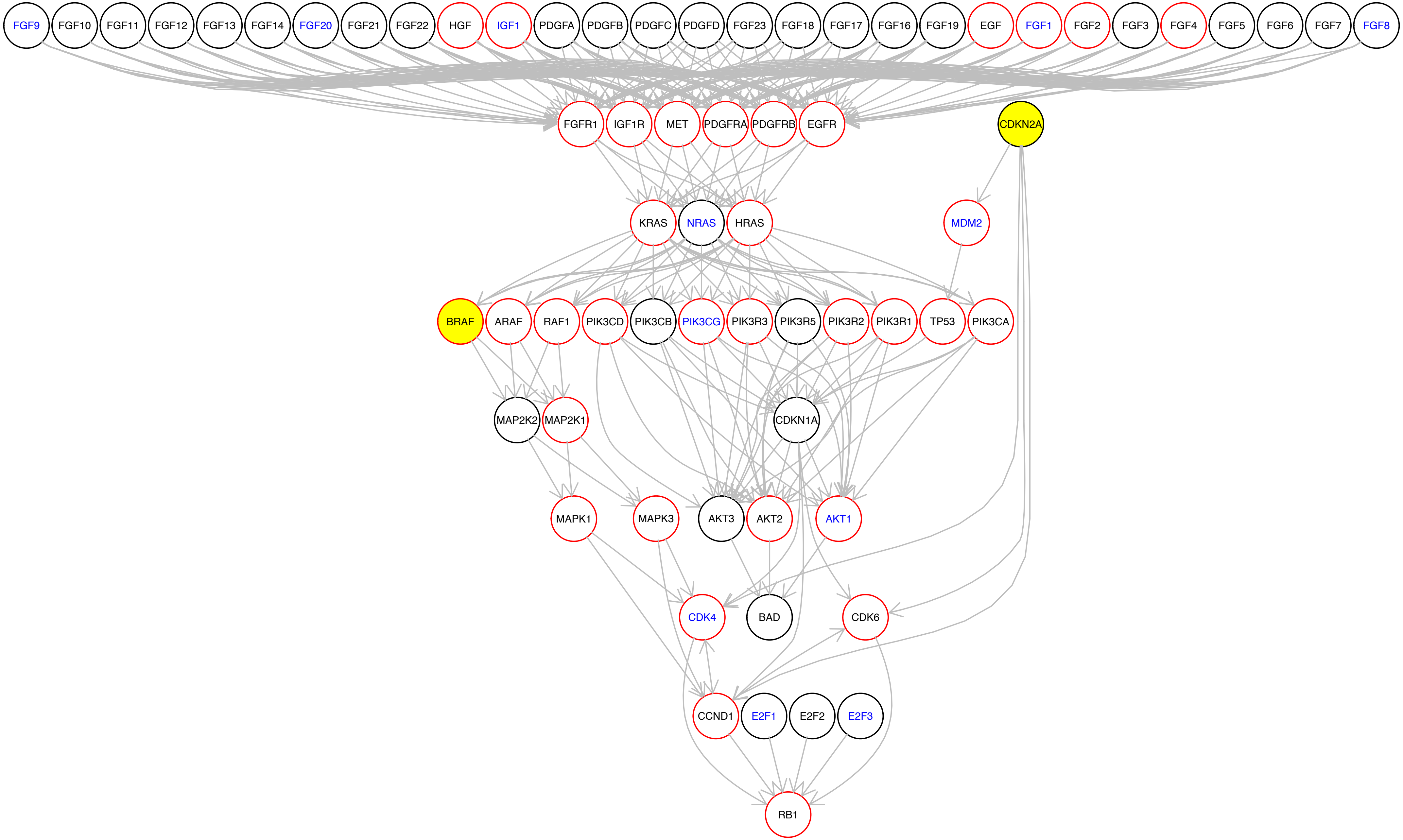

KEGG pathway = Melanoma : tumour = YUZINO : Yellow Fill = gene variant, Blue Text = expression−survival association, Red Boarder = drug

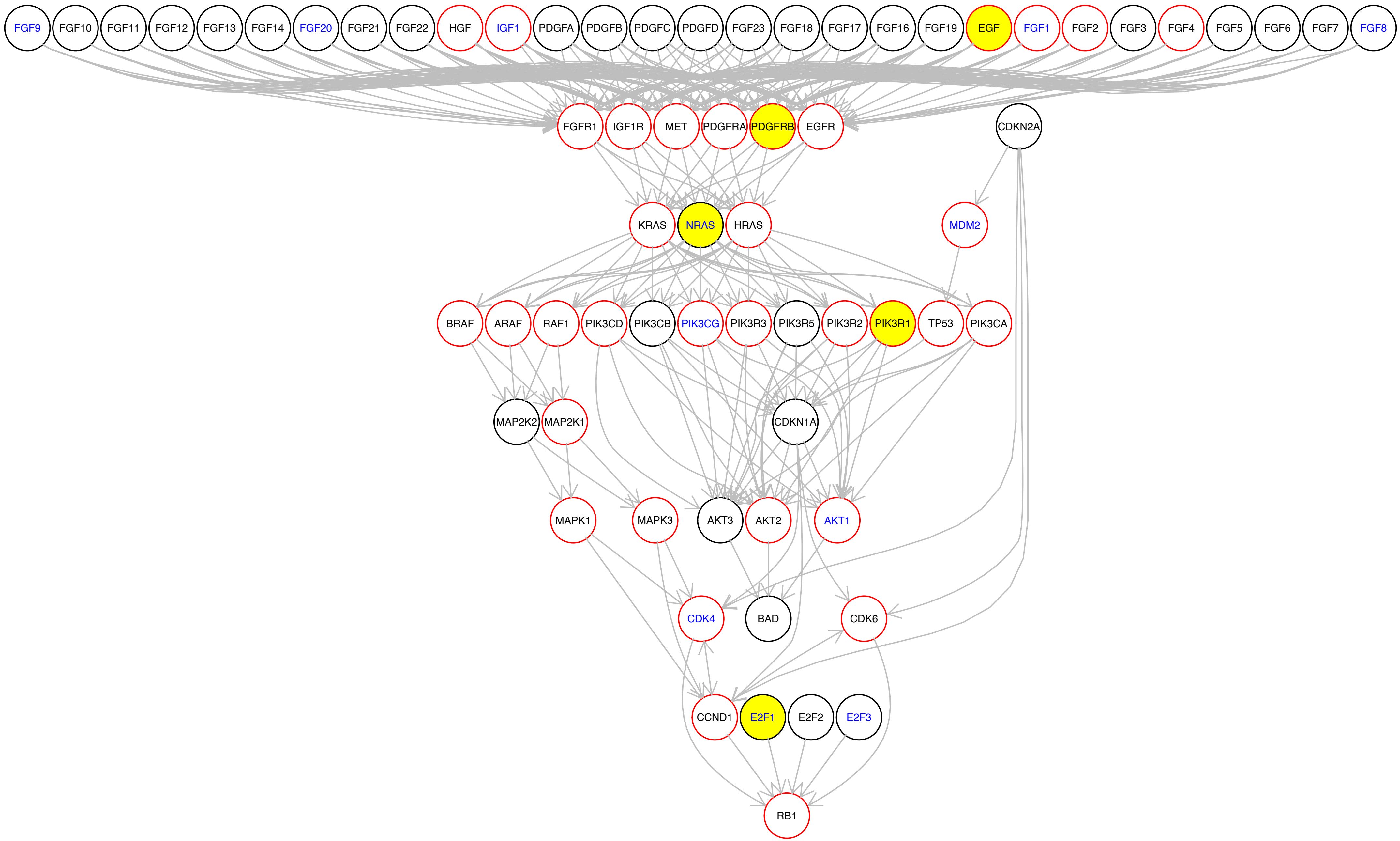

Supplement: Supplementary file 1 [file 51894_Print_Presentation1.PDF]
