# Supplementary material for: MelanomaDB: A Web Tool for Integrative Analysis of Melanoma Genomic Information to Identify Disease-Associated Molecular Pathways
Source: Front Oncol. 2013 Jul 16;3:184. doi: 10.3389/fonc.2013.00184 (PMC3712543; doi:10.3389/fonc.2013.00184)

The two pages that follows are: (i) a clustered heatmap and (ii) a reverse waterfall plot for genes encoding proteins of the KEGG "Neuroactive ligand-receptor interaction" signalling pathway. Gene names are on the horizontal axis, individual melanoma tumour names are on the vertical axis. Blue blocks at the intersection of a gene and a tumour indicates the presence of a protein-altering somatic variant in that gene in that tumour. Clustering of genes and tumours using single linkage clustering with binary distance was performed based on this variant information. The clustered figure was then annotated with additional information above the heatmap. in the first row above the heatmap red blocks mark genes encoding known drug targets according to version 3 of the DrugBank database. In the second row yellow blocks mark genes encoding potentially druggable proteins, as indicated by the MelanomaDB gene set "Druggability: Sophic ENSEMBL list"[31]. In the third orange and red blocks indicate genes mutated in ≥1% or ≥ 5% of the 310 melanomas in our database, respectively. In the fourth row blue blocks mark genes that encode RNAs with a significant association between expression and patient survival (p≤ 0.05 no multiple testing correction applied, Cox proportion hazards model, Bogunovic et al data[48]). In the fifth row brown blocks indicate genes that are members of the Wellcome Trust Cosmic "Cancer Gene Census" gene set, as on 1st March 2013 (http://cancer.sanger.ac.uk/cancergenome/projects/census/). In the sixth row, purple blocks mark genes thought to be melanoma drivers when mutated (MelanomaDB gene set "Melanomagenesis Drivers" [76]).the "Melanoma" KEGG pathway in an individual tumour.

KEGG pathway = Neuroactive ligand−receptor interaction

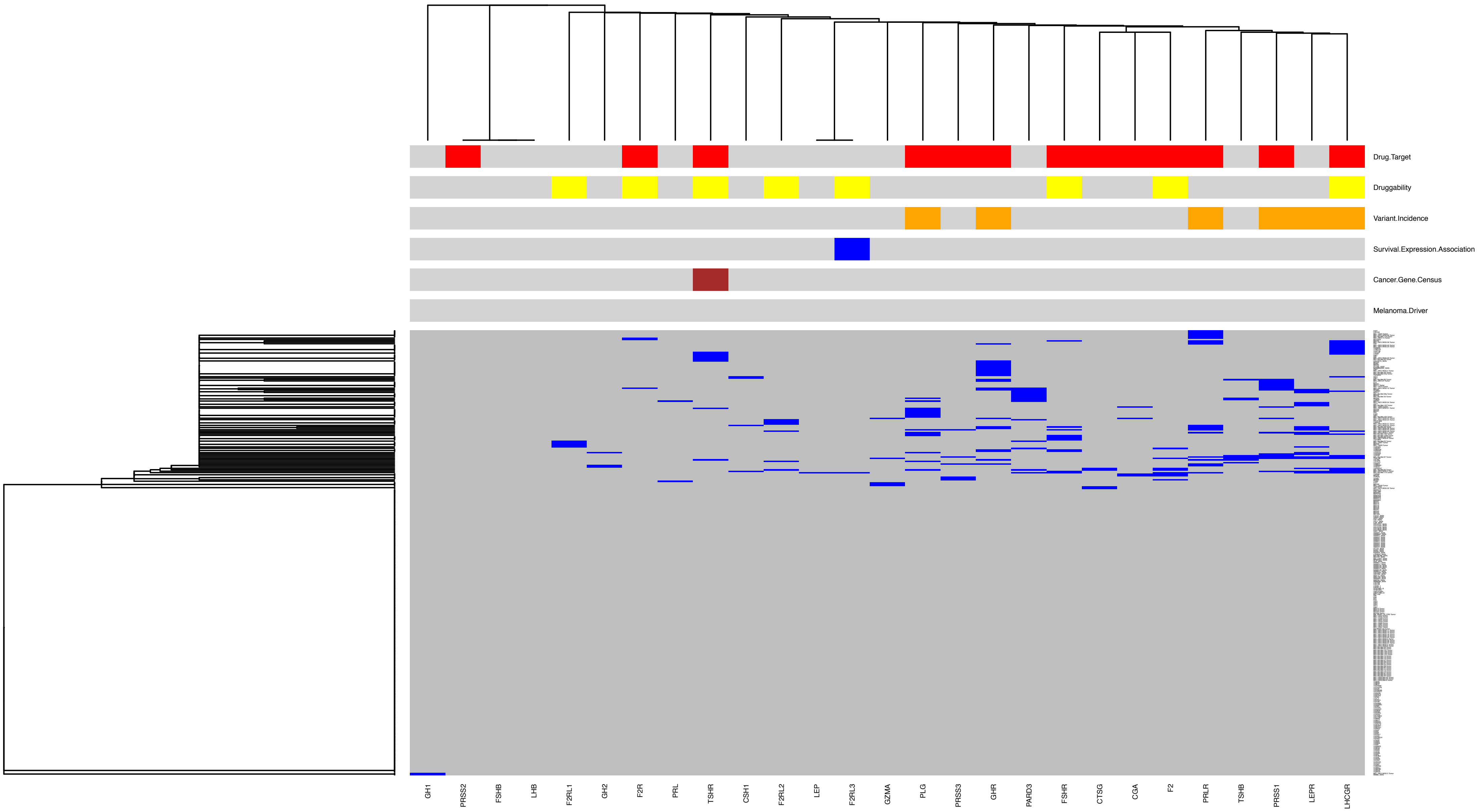

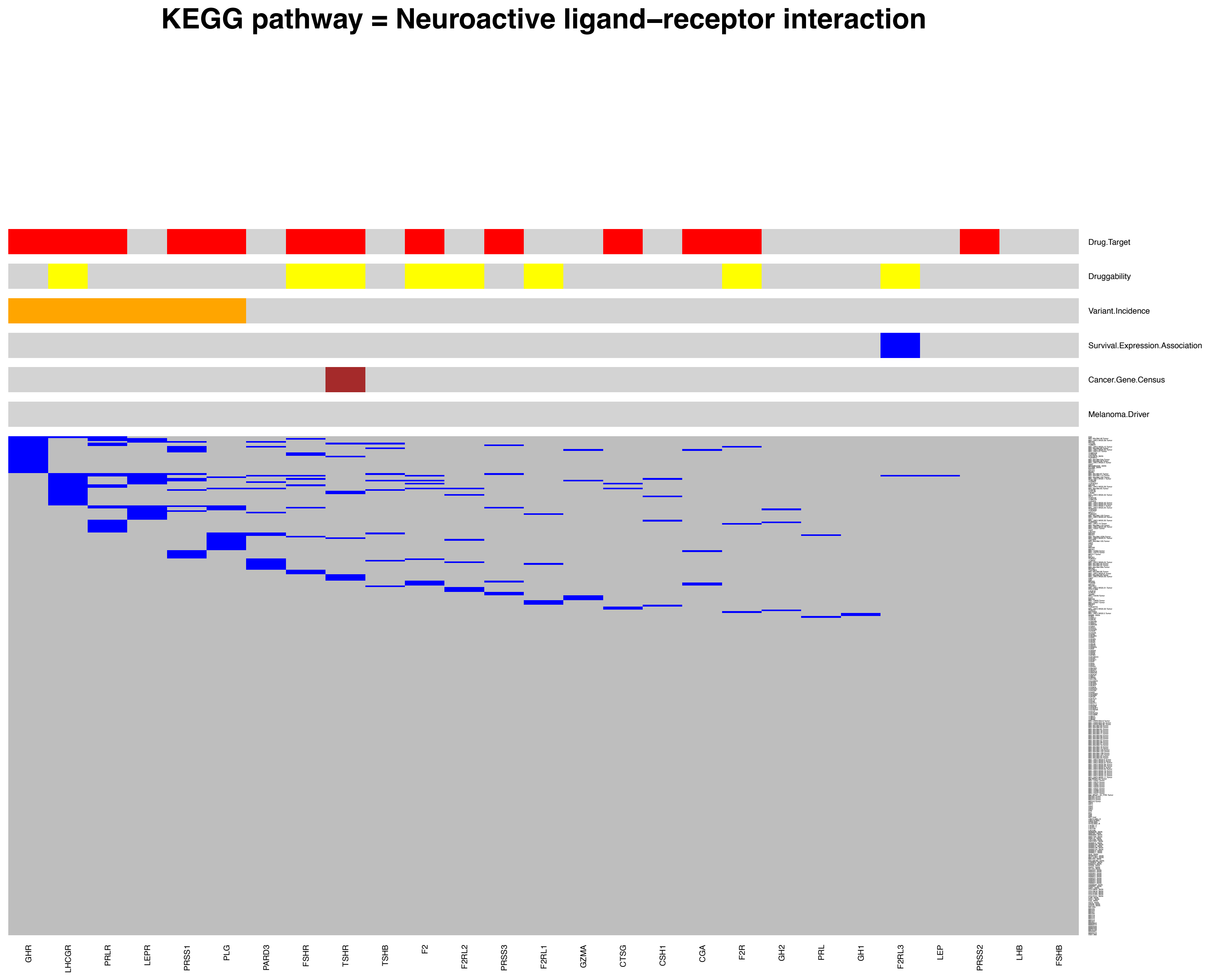
KEGG pathway = Neuroactive ligand–receptor interaction
Drug.Target
Druggability
Variant.Incidence
Survival.Expression.Association
Cancer.Gene.Census
Melanoma.Driver
GHR
LHCGR
PRLR
LEPR
PRSS1
PLG
PARD3
FSHR
TSHR
TSHB
F2
F2RL2
PRSS3
F2RL1
GZMA
CTSG
CSH1
CGA
F2R
GH2
PRL
GH1
F2RL3
LEP
PRSS2
LHB
FSHB

Each page that follows represents the “Neuroactive ligand-receptor interaction” KEGG pathway in an individual tumour.

Yellow fill in nodes indicates that the gene has a somatic variation in that tumour.

Red border of nodes indicates that there is a drug available to target this gene’s encoded protein.

Blue text in nodes indicate genes that encode RNAs with a significant association between expression and patient survival ($p \leq 0.05$ no multiple testing correction applied, Cox proportion hazards model, Bogunovic et al data [48])

KEGG pathway = Neuroactive ligand−receptor interaction : tumour = X501.Mel : Yellow Fill = gene variant, Blue Text = expression−survival association, Red Boarder = drug

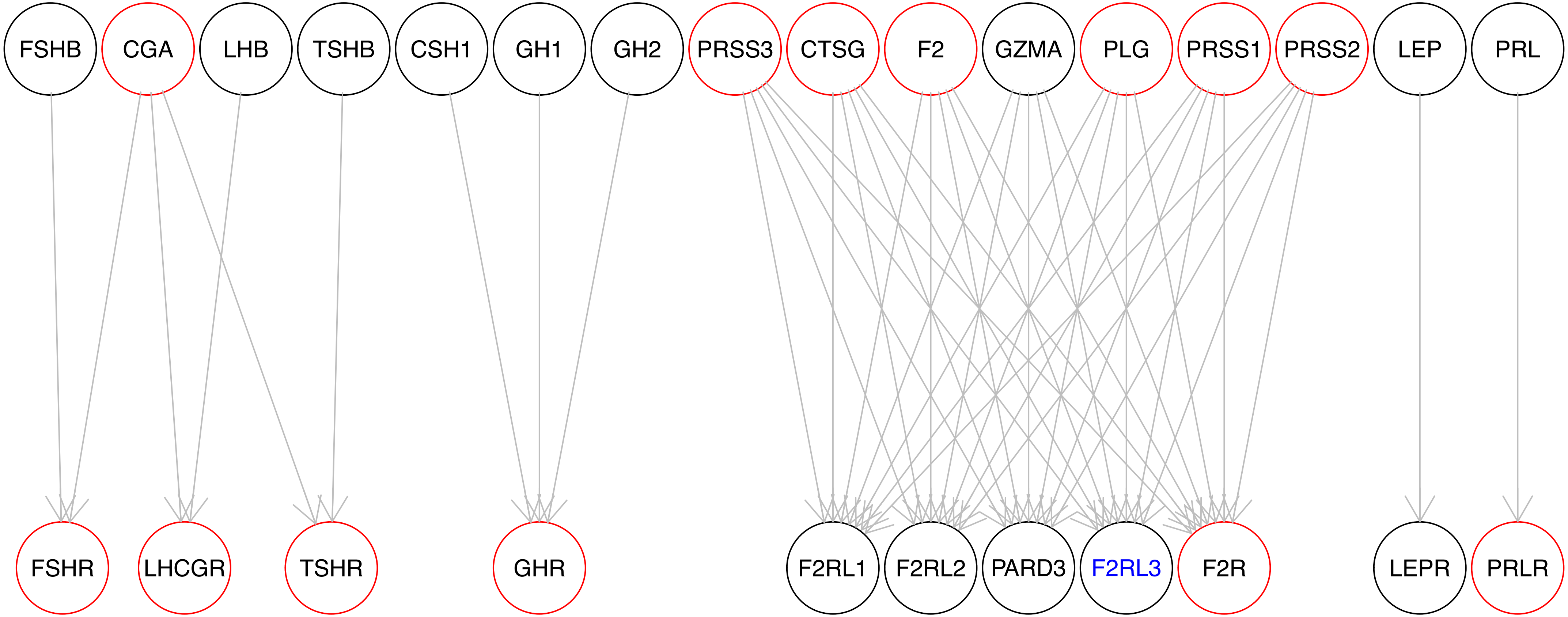

KEGG pathway = Neuroactive ligand−receptor interaction :   tumour = M000216 :   Yellow Fill = gene variant, Blue Text = expression−survival association, Red Boarder = drug

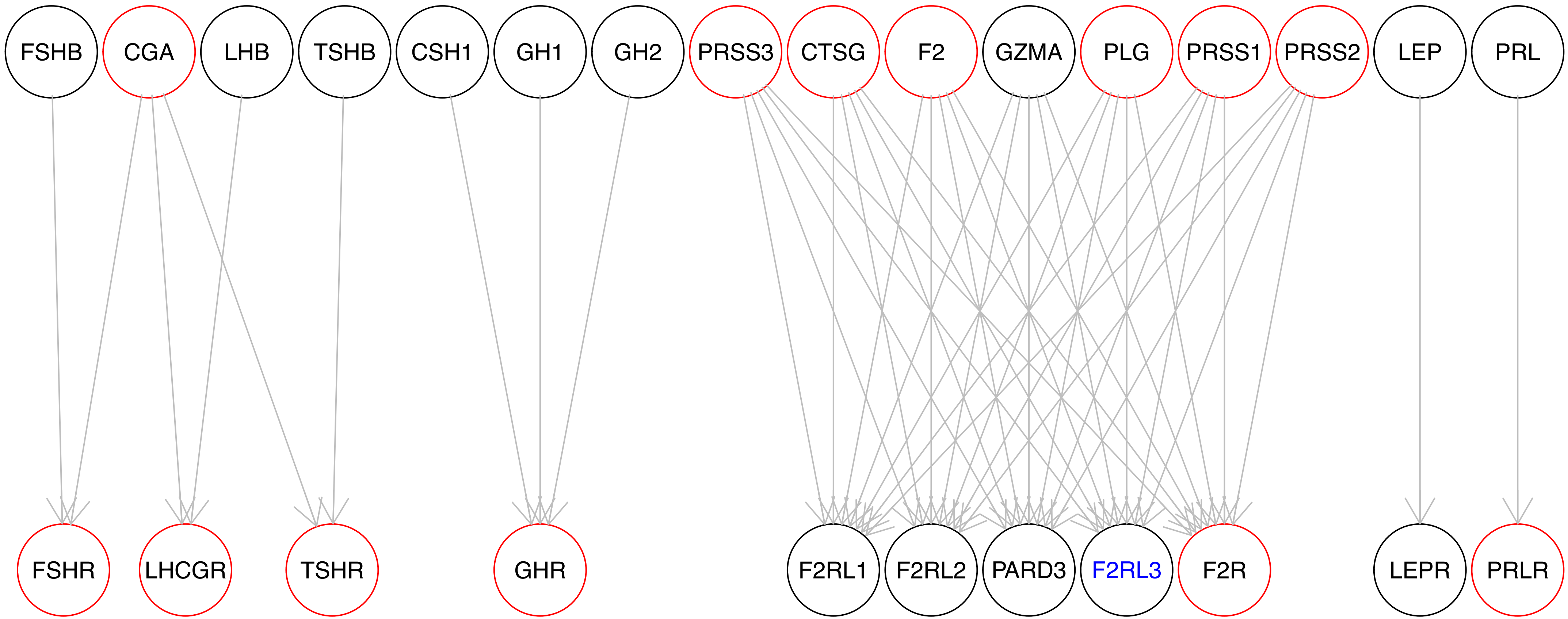

KEGG pathway = Neuroactive ligand−receptor interaction : tumour = M000921 : Yellow Fill = gene variant, Blue Text = expression−survival association, Red Boarder = drug

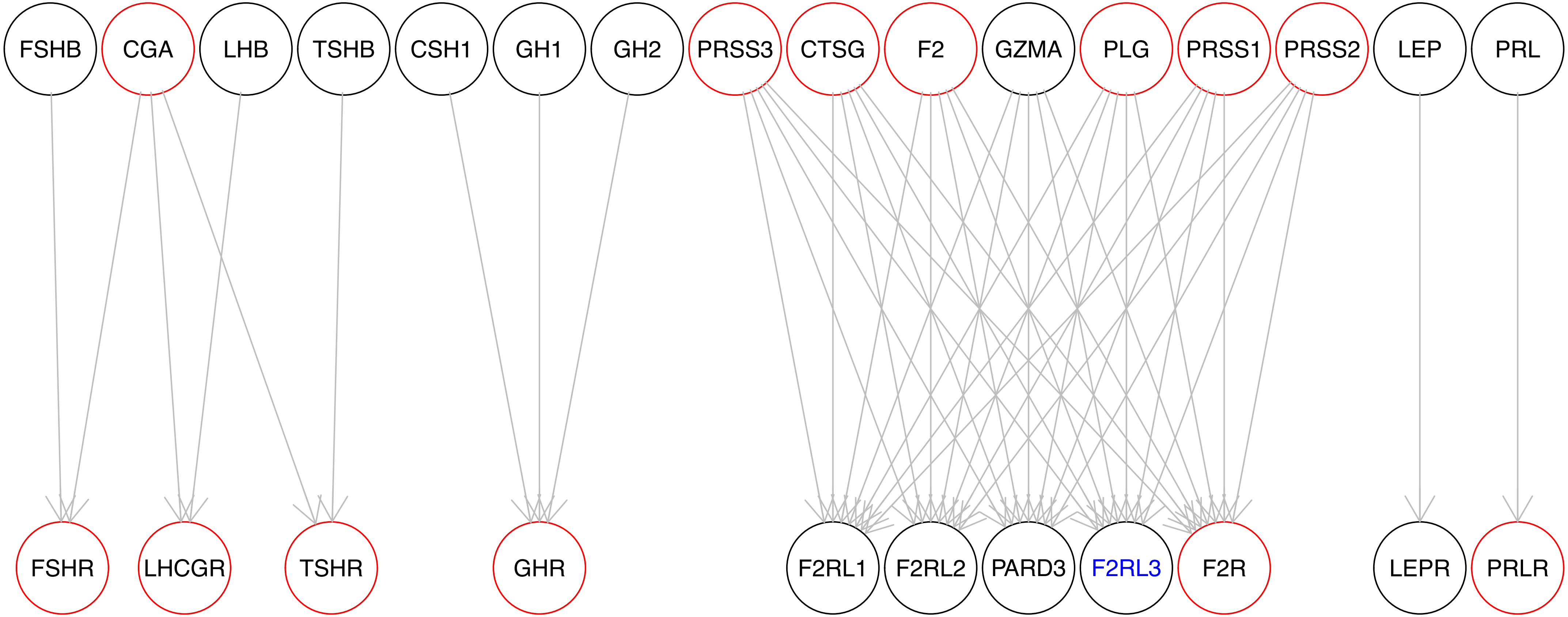

KEGG pathway = Neuroactive ligand−receptor interaction : tumour = M010403 : Yellow Fill = gene variant, Blue Text = expression−survival association, Red Boarder = drug

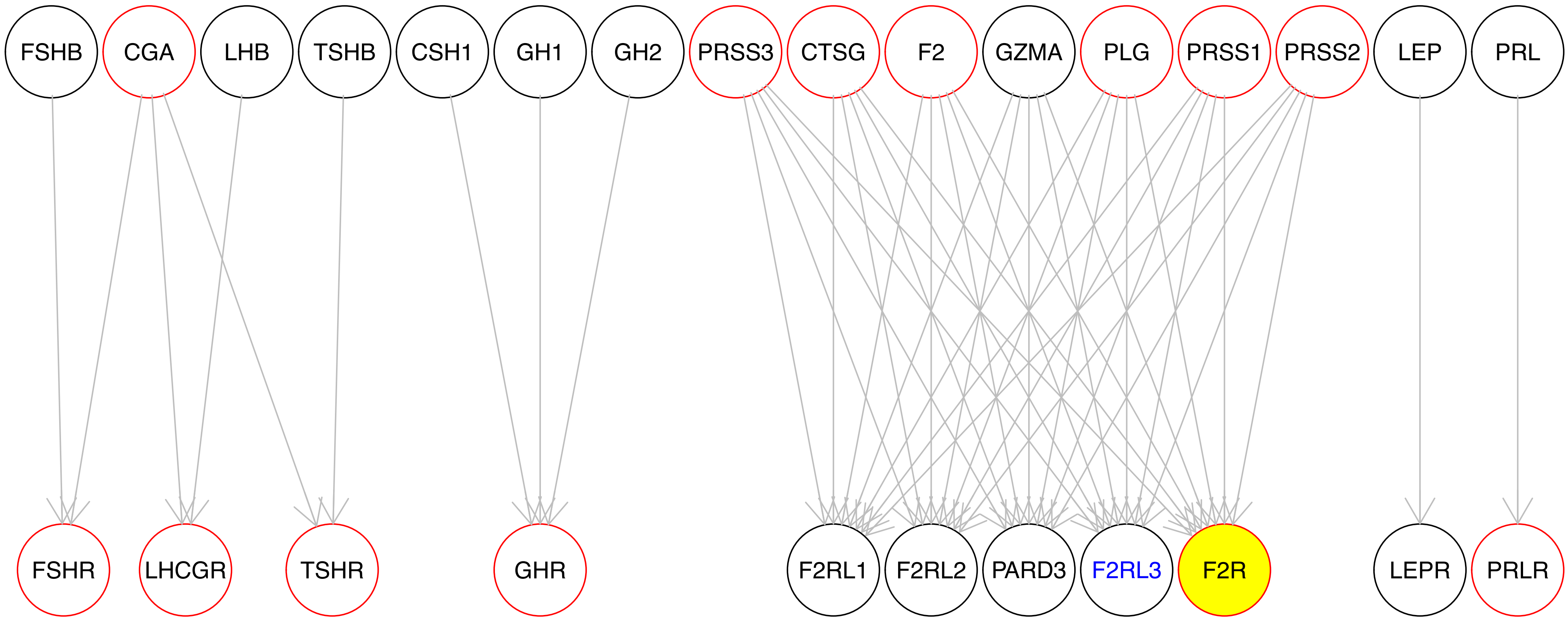

KEGG pathway = Neuroactive ligand−receptor interaction :   tumour = M970109 :   Yellow Fill = gene variant, Blue Text = expression−survival association, Red Boarder = drug

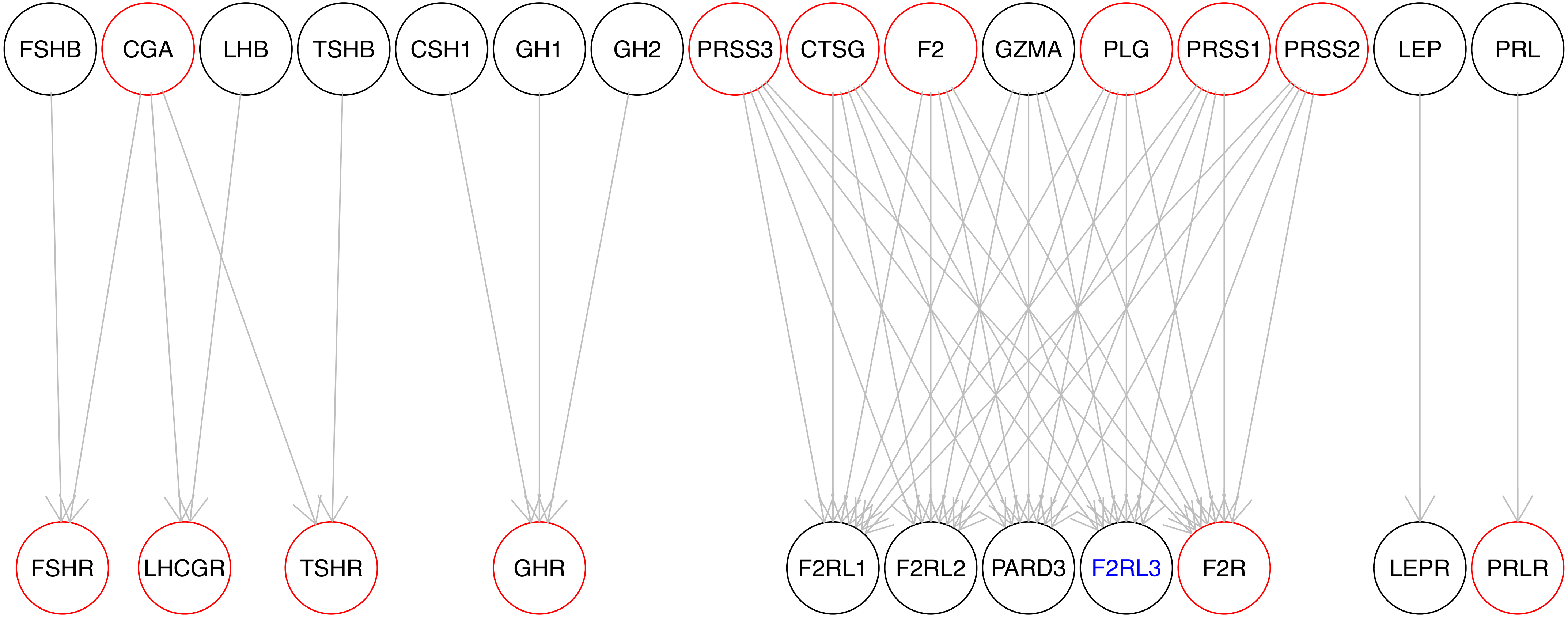

KEGG pathway = Neuroactive ligand−receptor interaction : tumour = M980409 : Yellow Fill = gene variant, Blue Text = expression−survival association, Red Boarder = drug

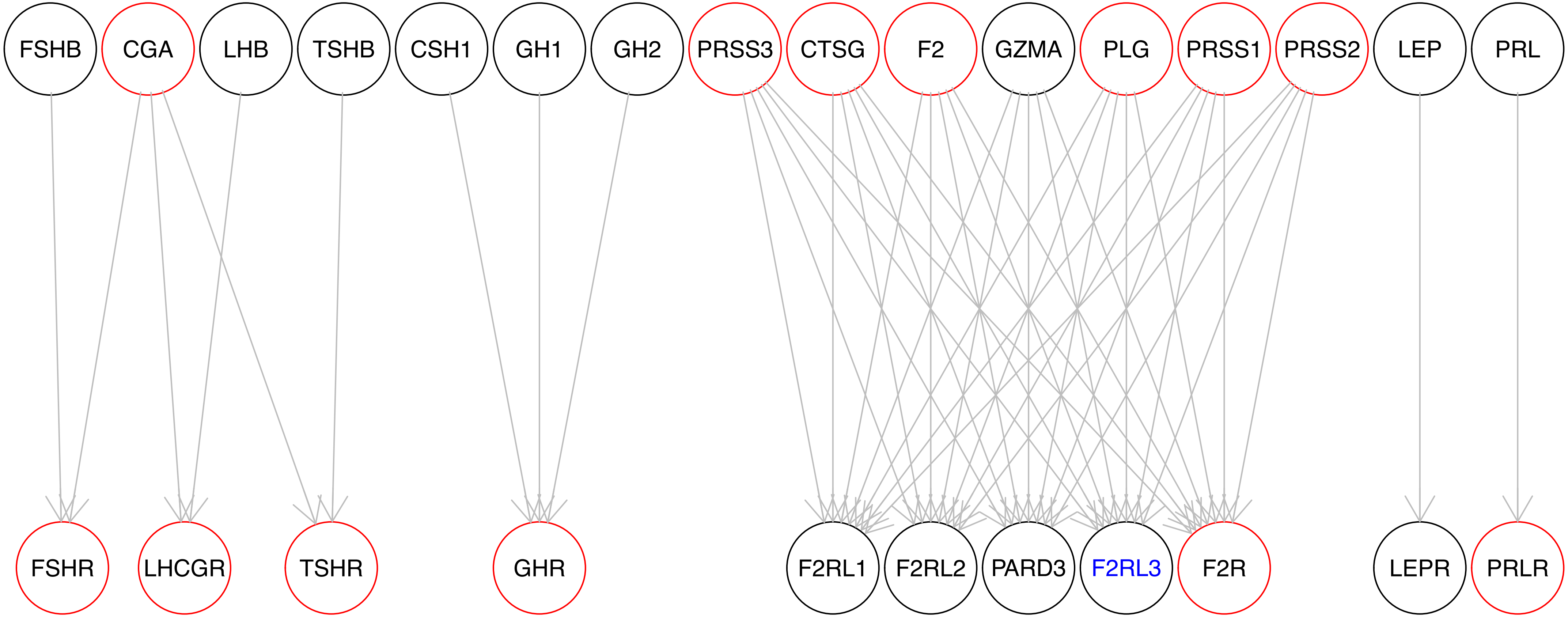

KEGG pathway = Neuroactive ligand−receptor interaction : tumour = M980928 : Yellow Fill = gene variant, Blue Text = expression−survival association, Red Boarder = drug

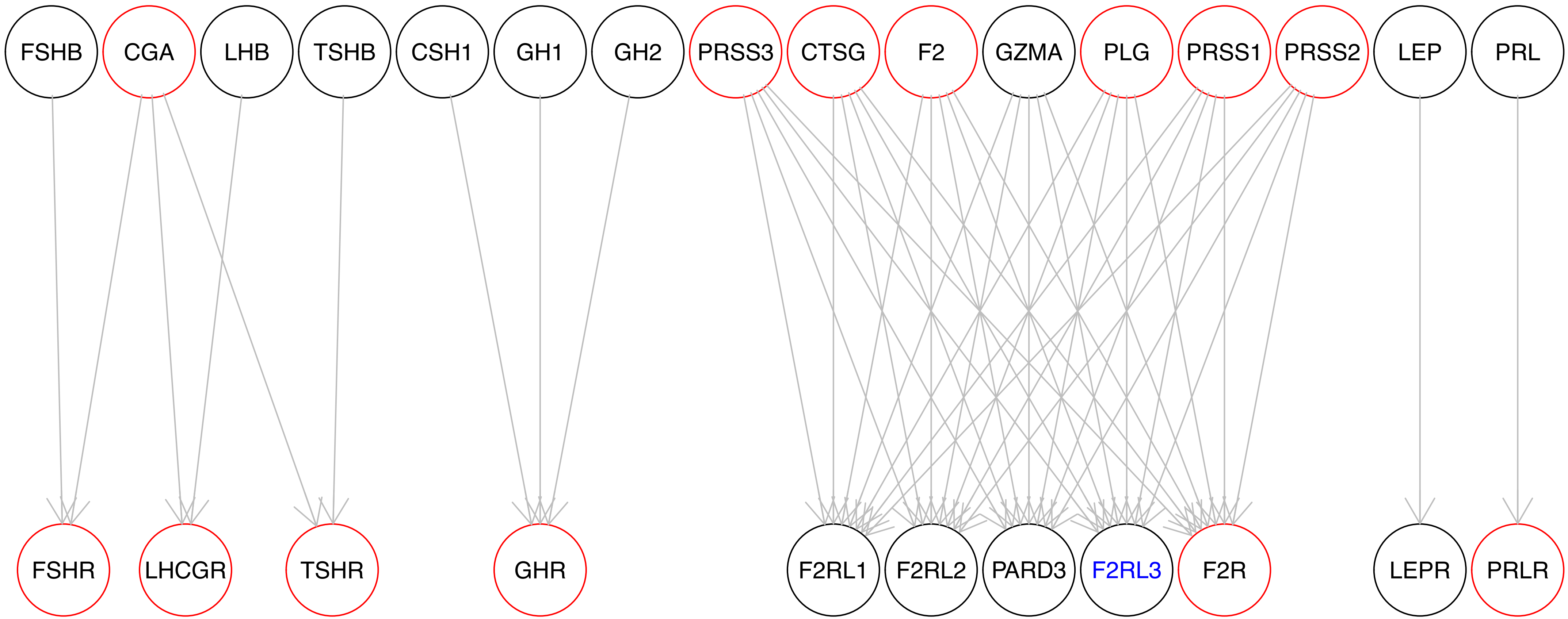

KEGG pathway = Neuroactive ligand−receptor interaction :   tumour = M990514 :   Yellow Fill = gene variant, Blue Text = expression−survival association, Red Boarder = drug

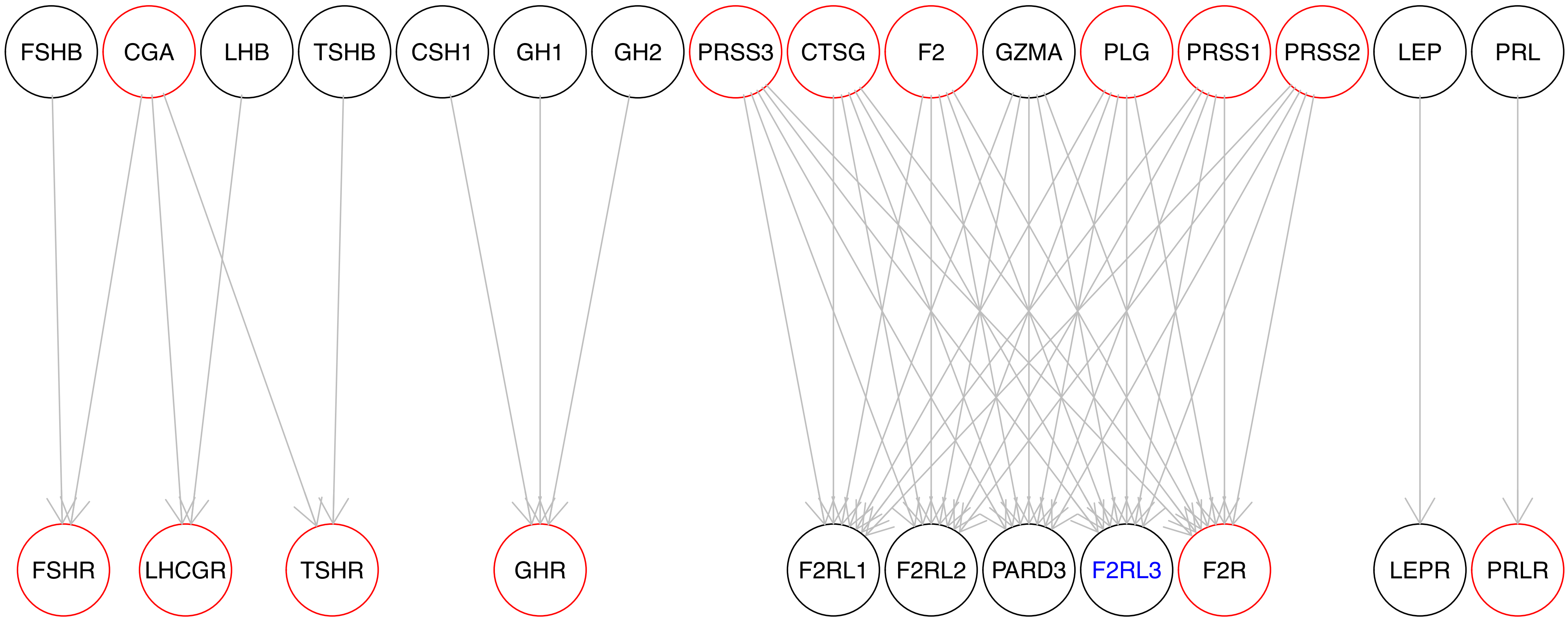

KEGG pathway = Neuroactive ligand−receptor interaction : tumour = M990802 : Yellow Fill = gene variant, Blue Text = expression−survival association, Red Boarder = drug

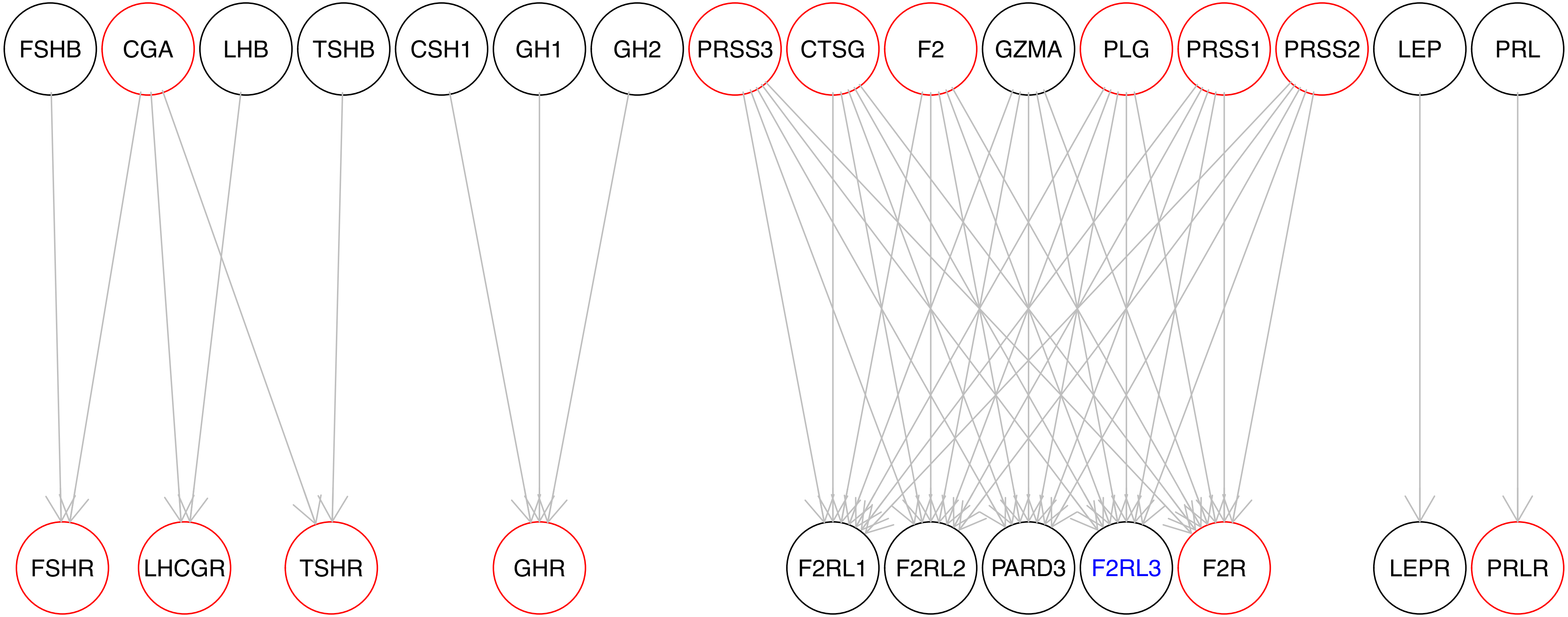

KEGG pathway = Neuroactive ligand−receptor interaction :   tumour = MeWo :   Yellow Fill = gene variant, Blue Text = expression−survival association, Red Boarder = drug

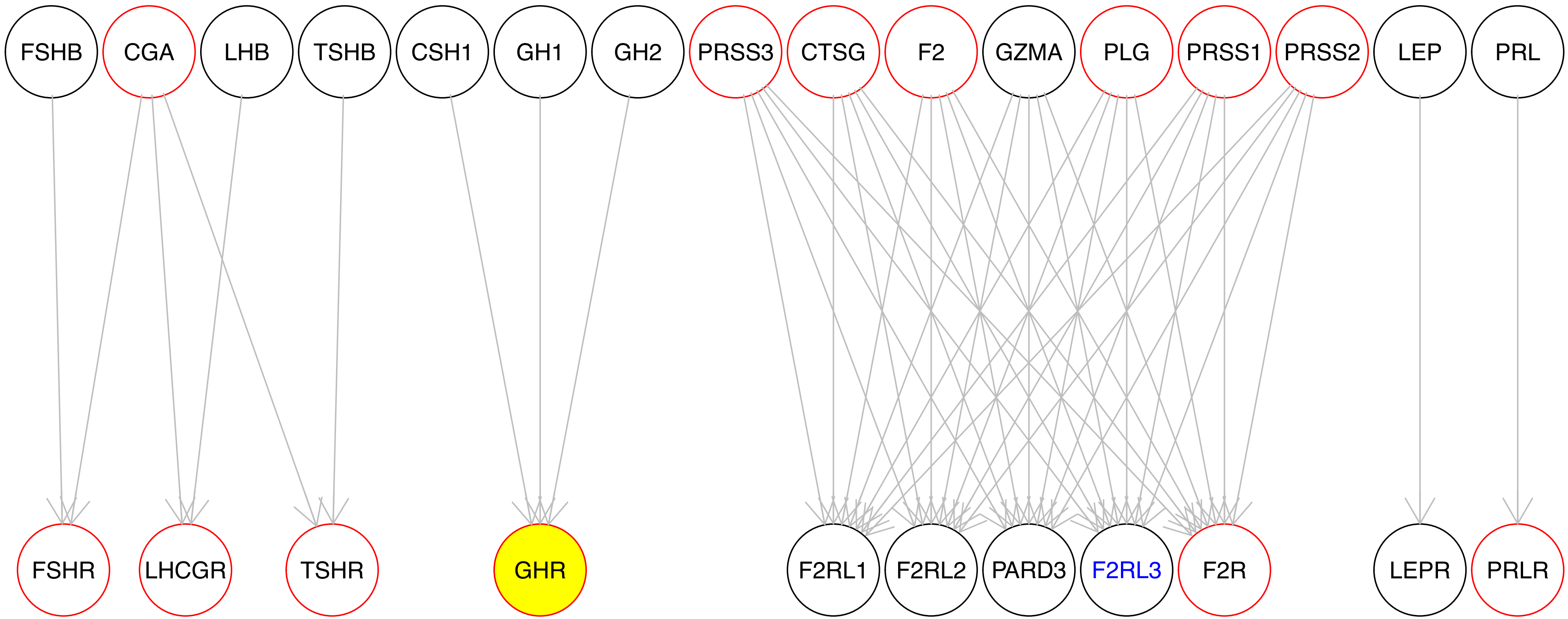

KEGG pathway = Neuroactive ligand−receptor interaction : tumour = ME001 : Yellow Fill = gene variant, Blue Text = expression−survival association, Red Boarder = drug

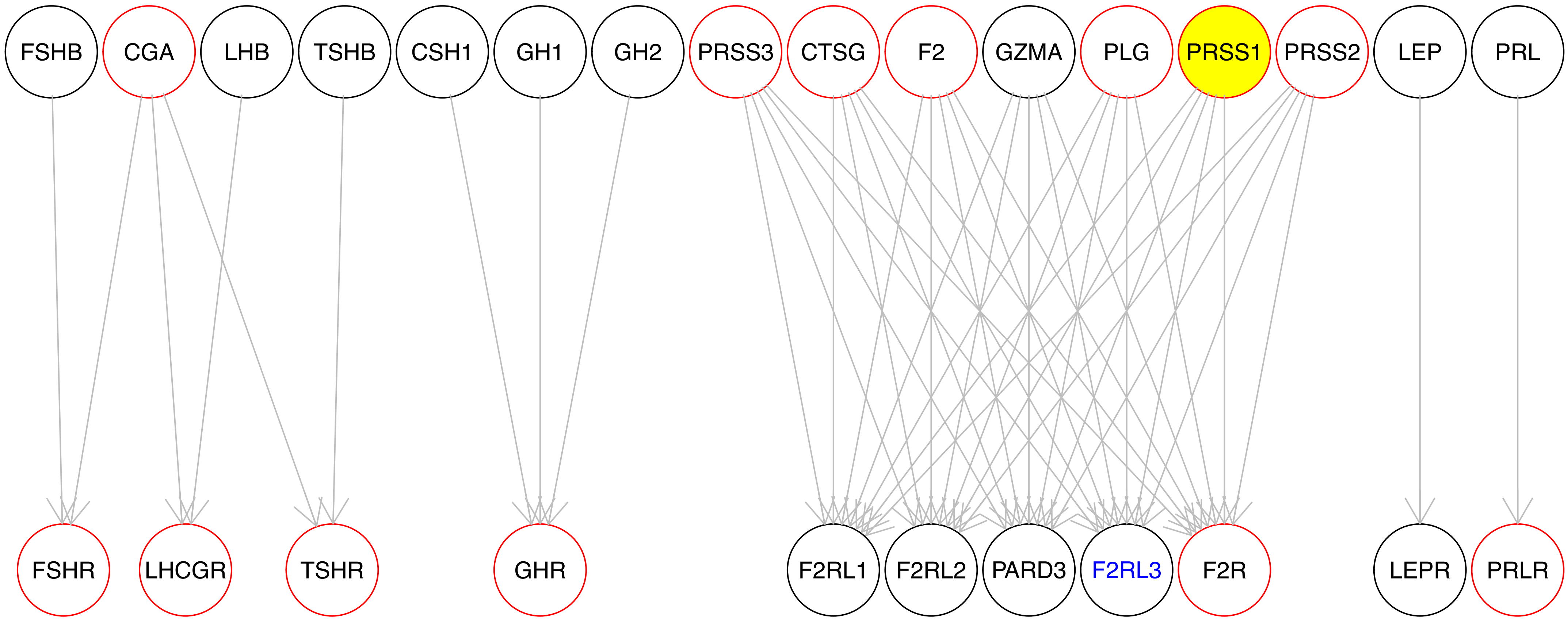

KEGG pathway = Neuroactive ligand−receptor interaction :   tumour = ME002 :   Yellow Fill = gene variant, Blue Text = expression−survival association, Red Boarder = drug

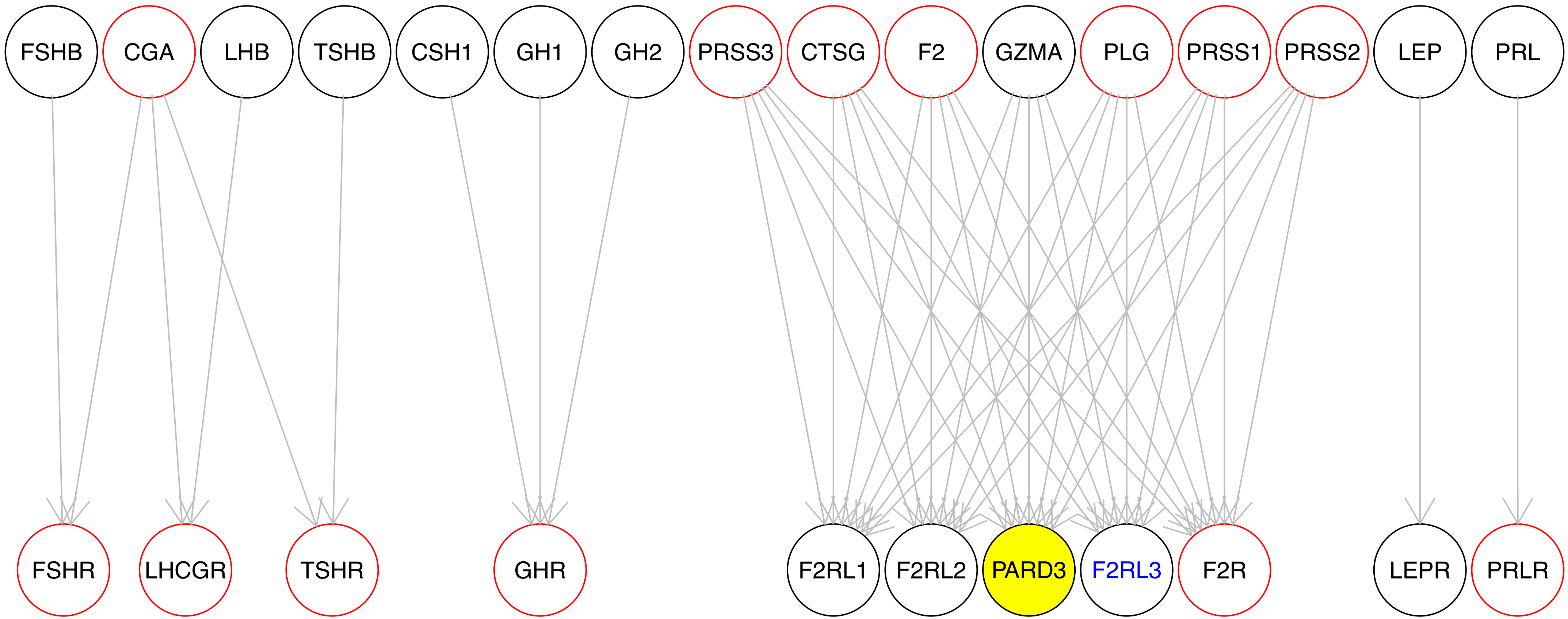

KEGG pathway = Neuroactive ligand−receptor interaction : tumour = ME007 : Yellow Fill = gene variant, Blue Text = expression−survival association, Red Boarder = drug

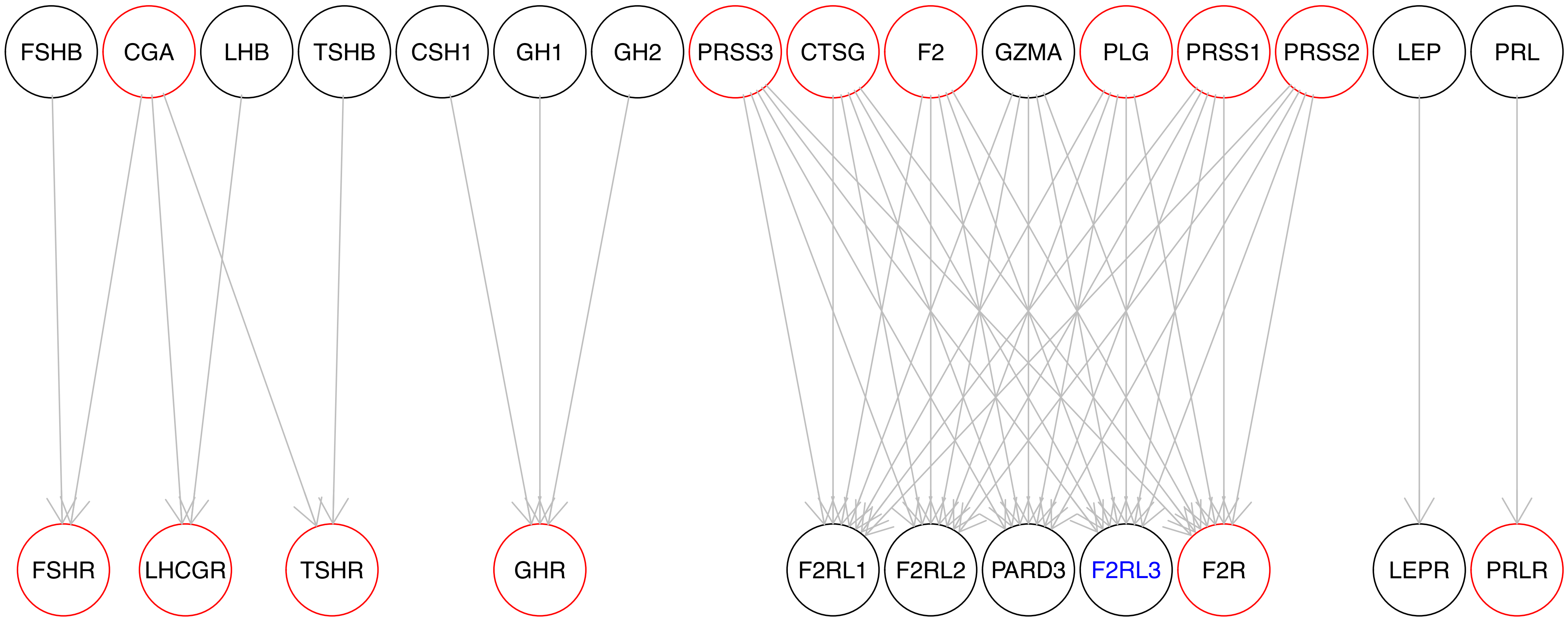

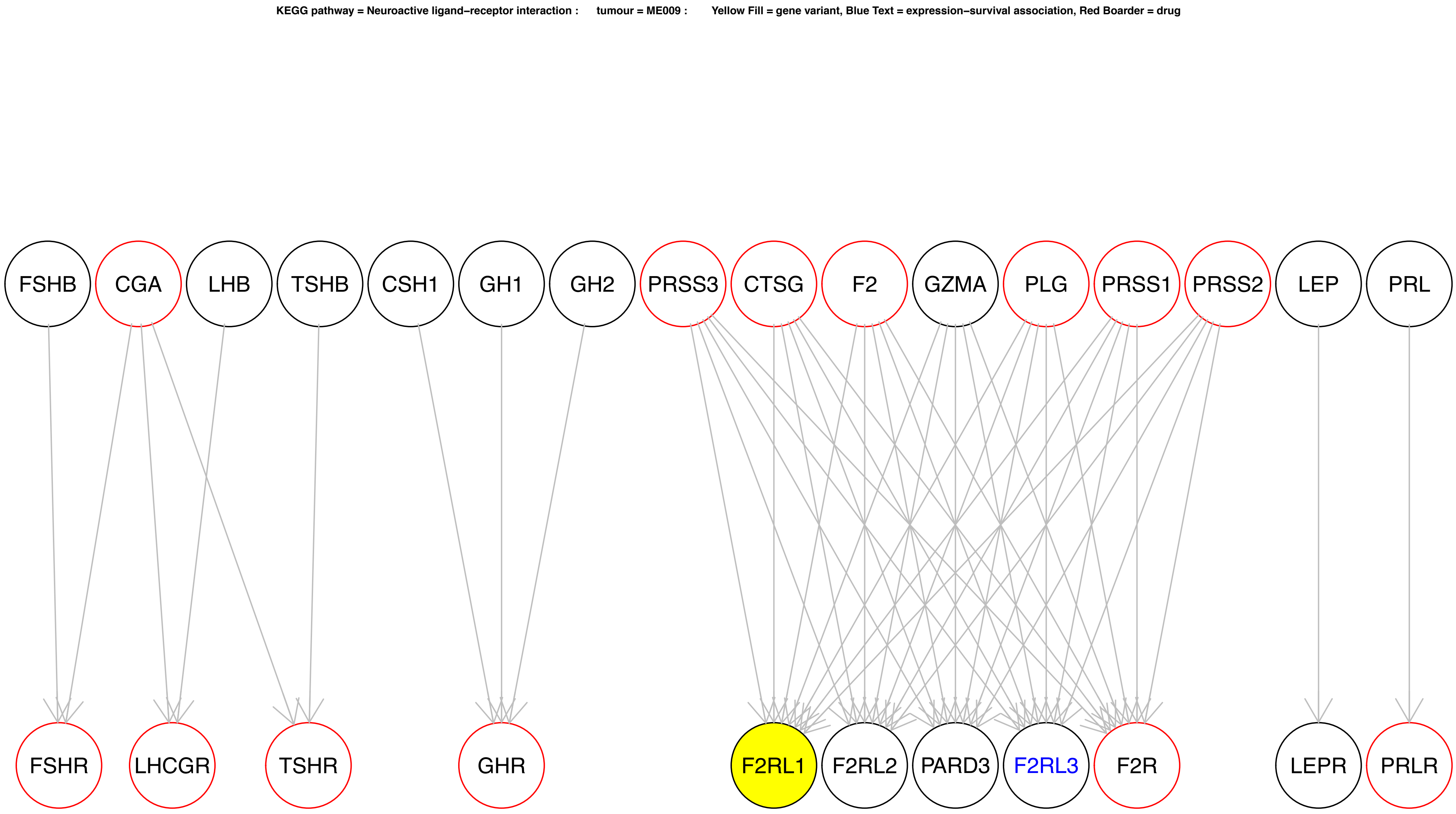
KEGG pathway = Neuroactive ligand−receptor interaction : tumour = ME009 : Yellow Fill = gene variant, Blue Text = expression−survival association, Red Boarder = drug
FSHB
CGA
LHB
TSHB
CSH1
GH1
GH2
PRSS3
CTSG
F2
GZMA
PLG
PRSS1
PRSS2
LEP
PRL
FSHR
LHCGR
TSHR
GHR
F2RL1
F2RL2
PARD3
F2RL3
F2R
LEPR
PRLR

KEGG pathway = Neuroactive ligand−receptor interaction :   tumour = ME011 :   Yellow Fill = gene variant, Blue Text = expression−survival association, Red Boarder = drug

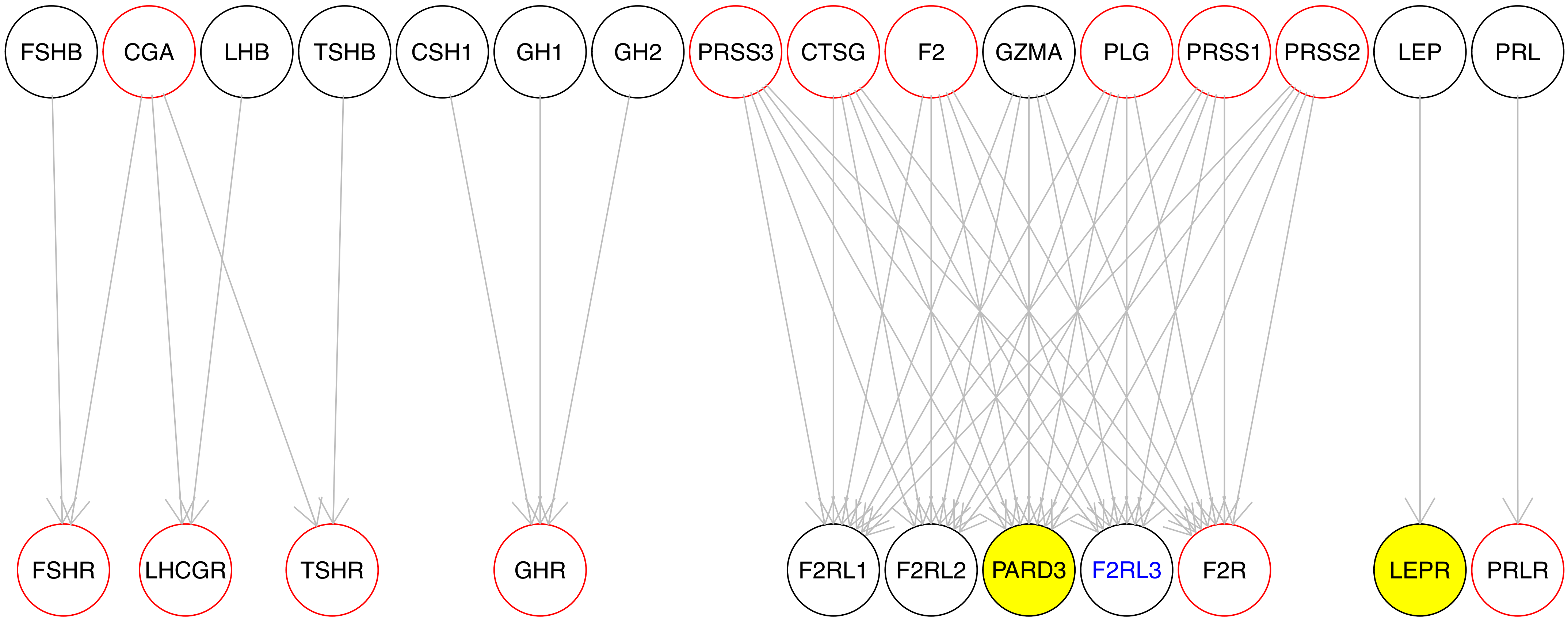

KEGG pathway = Neuroactive ligand−receptor interaction :   tumour = ME012 :   Yellow Fill = gene variant, Blue Text = expression−survival association, Red Boarder = drug

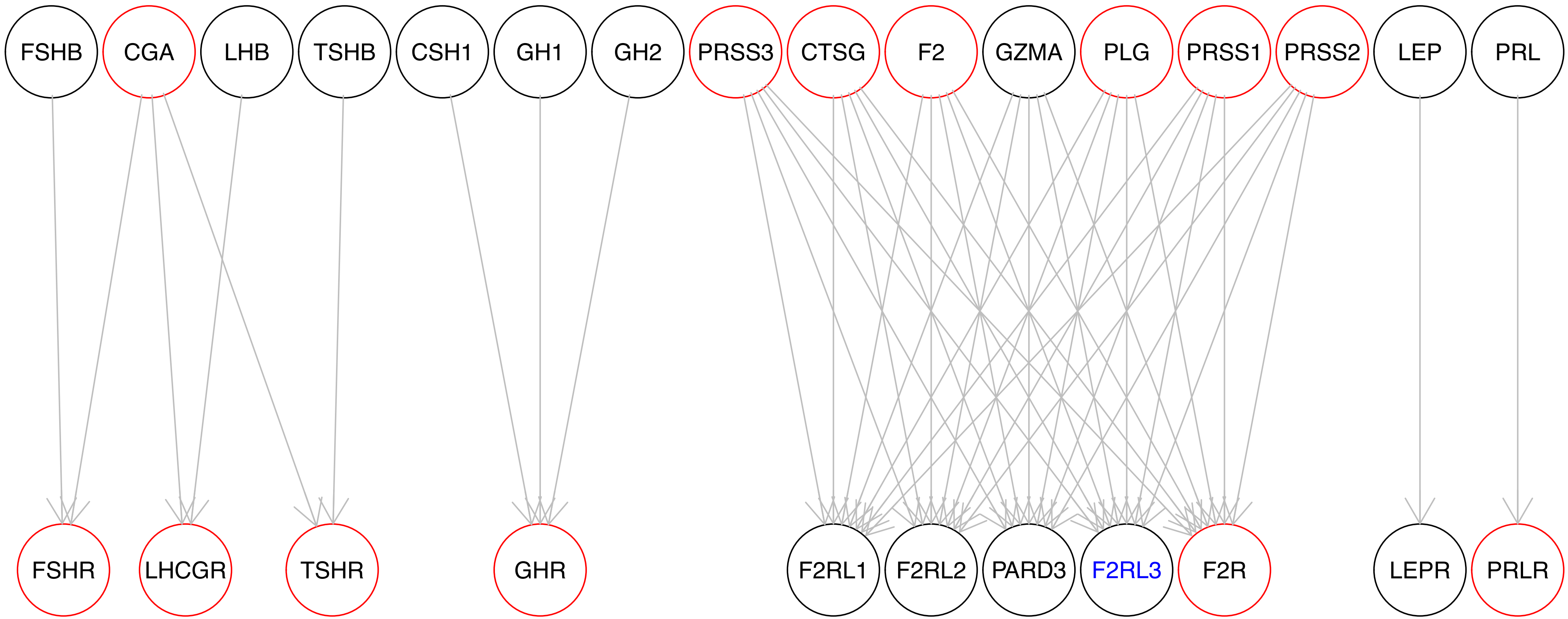

KEGG pathway = Neuroactive ligand−receptor interaction :   tumour = ME015 :   Yellow Fill = gene variant, Blue Text = expression−survival association, Red Boarder = drug

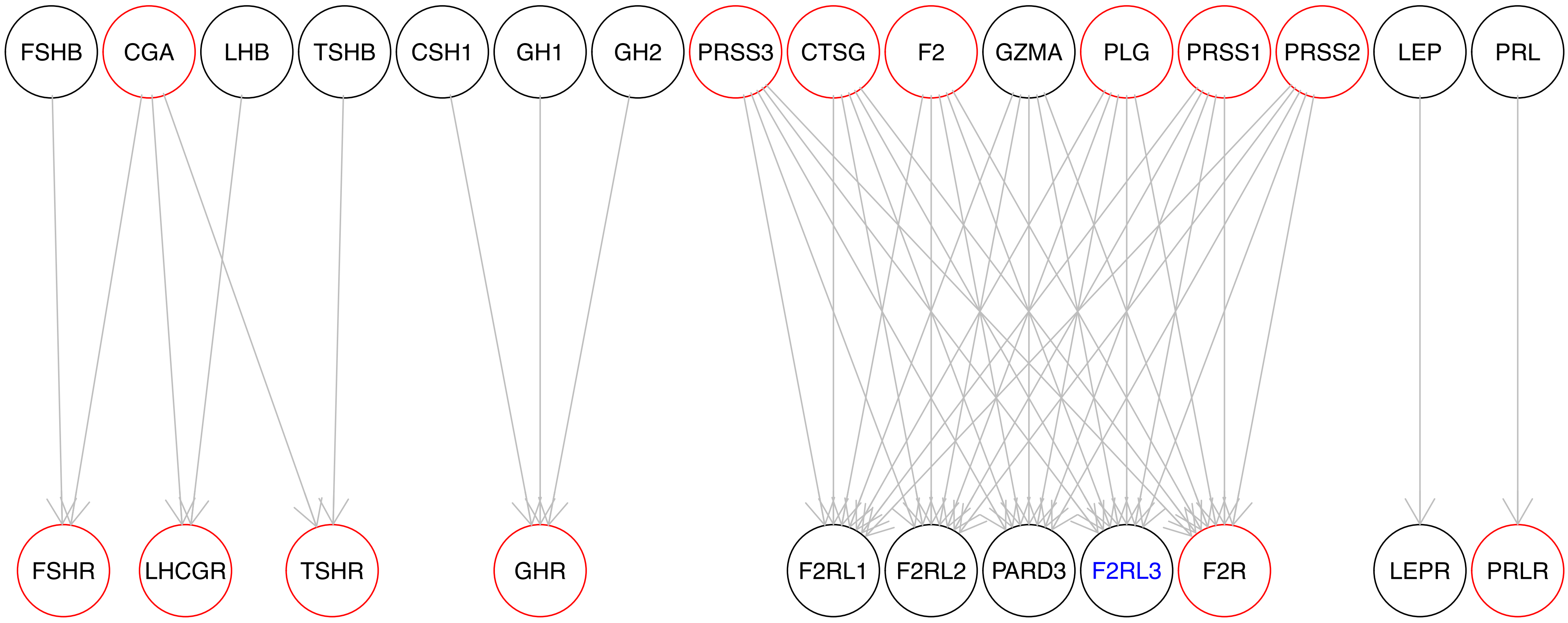

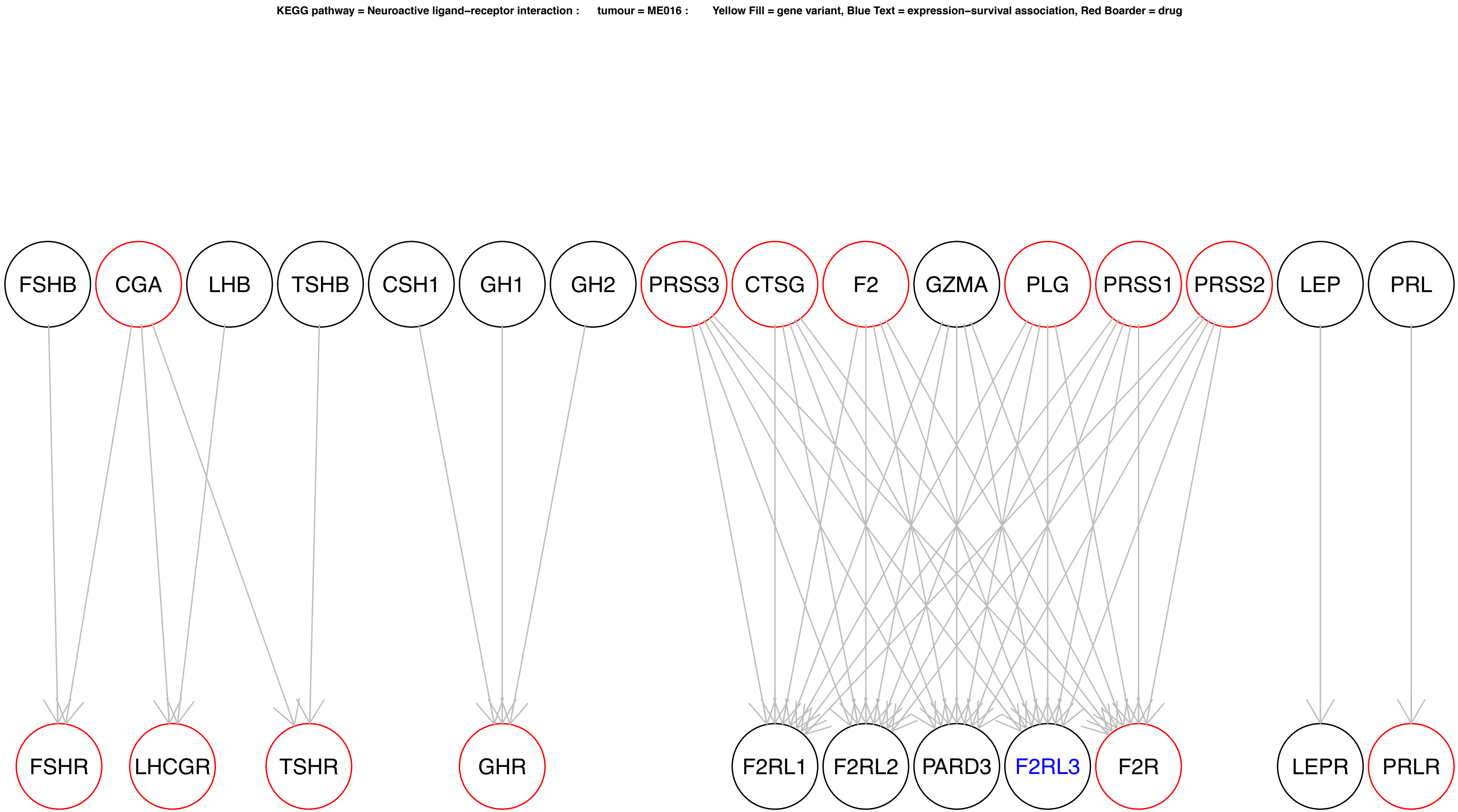
KEGG pathway = Neuroactive ligand−receptor interaction : tumour = ME016 : Yellow Fill = gene variant, Blue Text = expression−survival association, Red Boarder = drug
FSHB
CGA
LHB
TSHB
CSH1
GH1
GH2
PRSS3
CTSG
F2
GZMA
PLG
PRSS1
PRSS2
LEP
PRL
FSHR
LHCGR
TSHR
GHR
F2RL1
F2RL2
PARD3
F2RL3
F2R
LEPR
PRLR

KEGG pathway = Neuroactive ligand−receptor interaction : tumour = ME018 : Yellow Fill = gene variant, Blue Text = expression−survival association, Red Boarder = drug

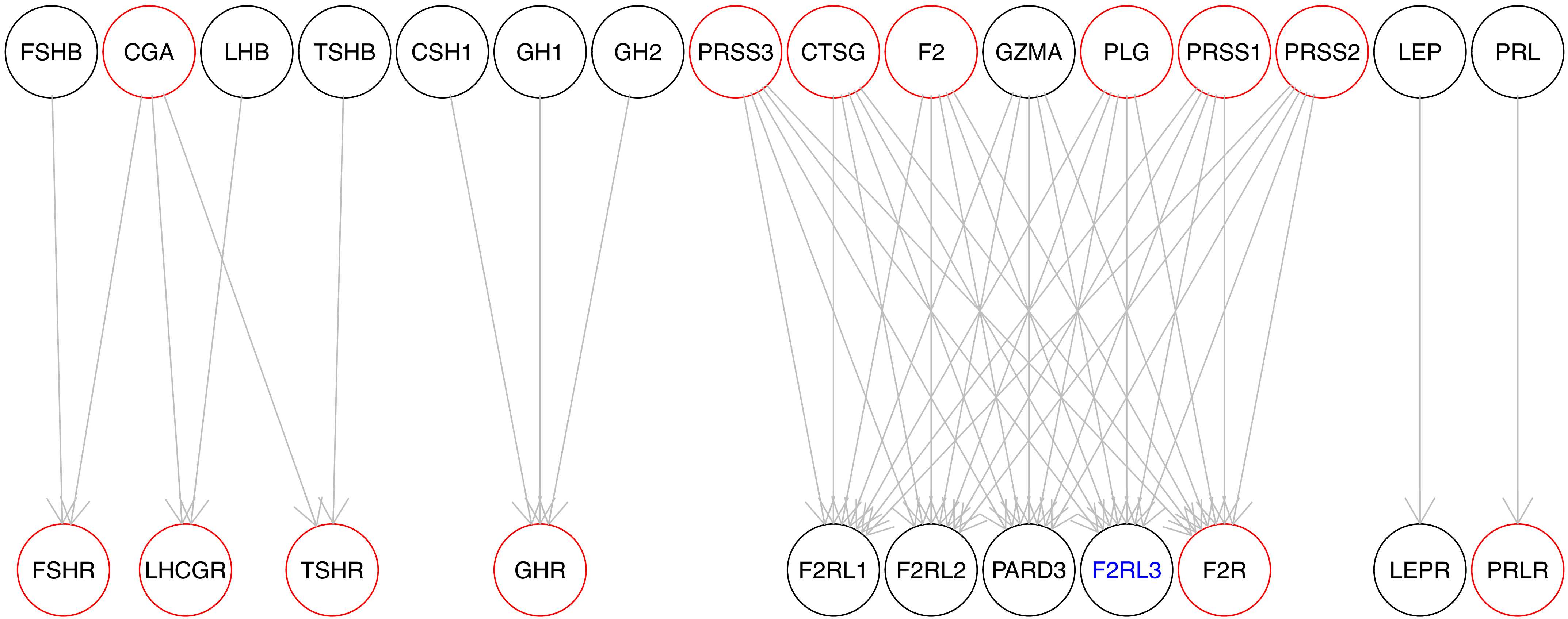

KEGG pathway = Neuroactive ligand−receptor interaction :   tumour = ME020 :   Yellow Fill = gene variant, Blue Text = expression−survival association, Red Boarder = drug

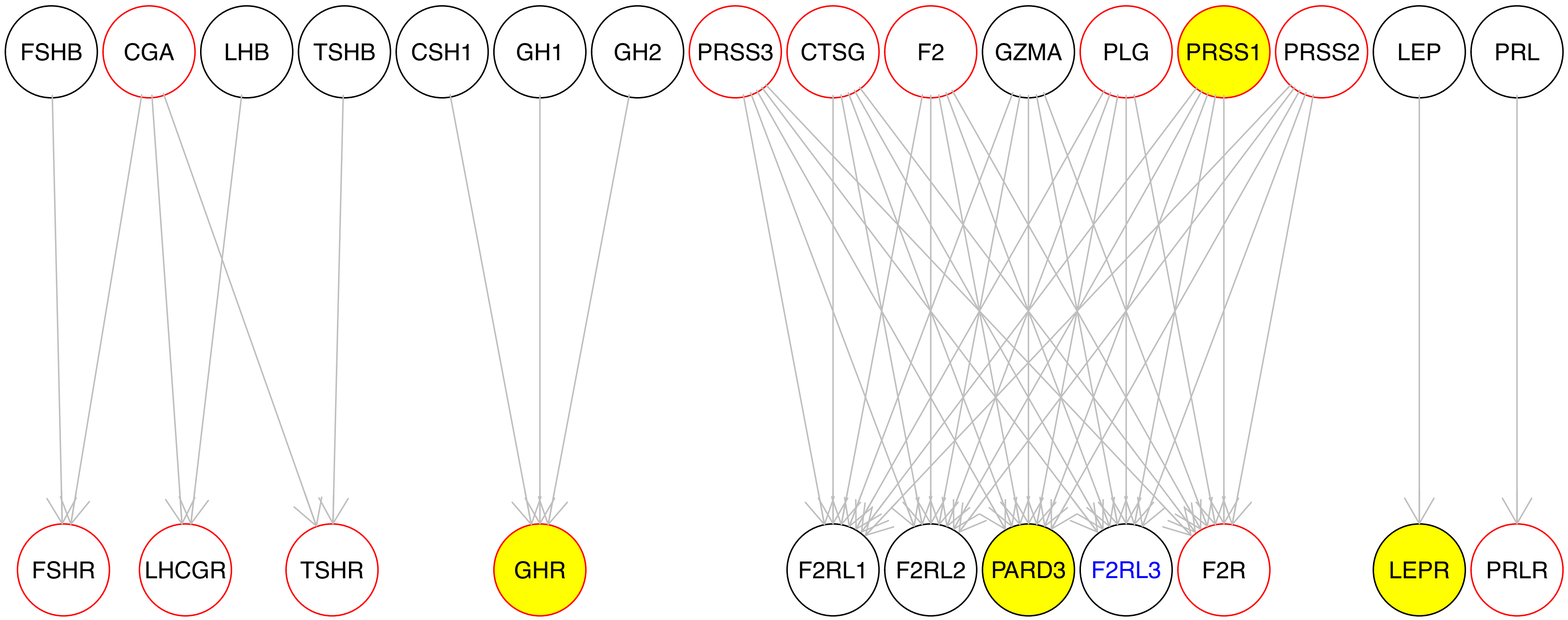

KEGG pathway = Neuroactive ligand−receptor interaction :   tumour = ME021 :   Yellow Fill = gene variant, Blue Text = expression−survival association, Red Boarder = drug

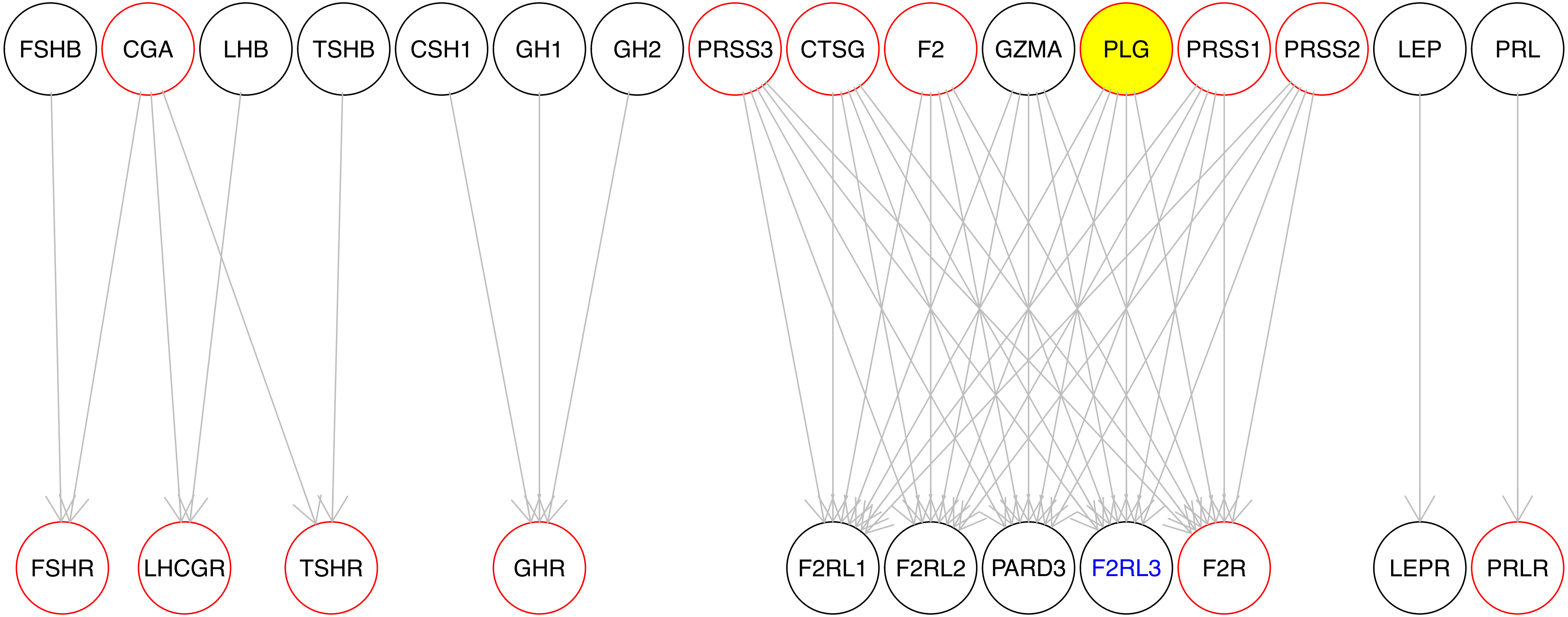

KEGG pathway = Neuroactive ligand−receptor interaction :   tumour = ME024 :   Yellow Fill = gene variant, Blue Text = expression−survival association, Red Boarder = drug

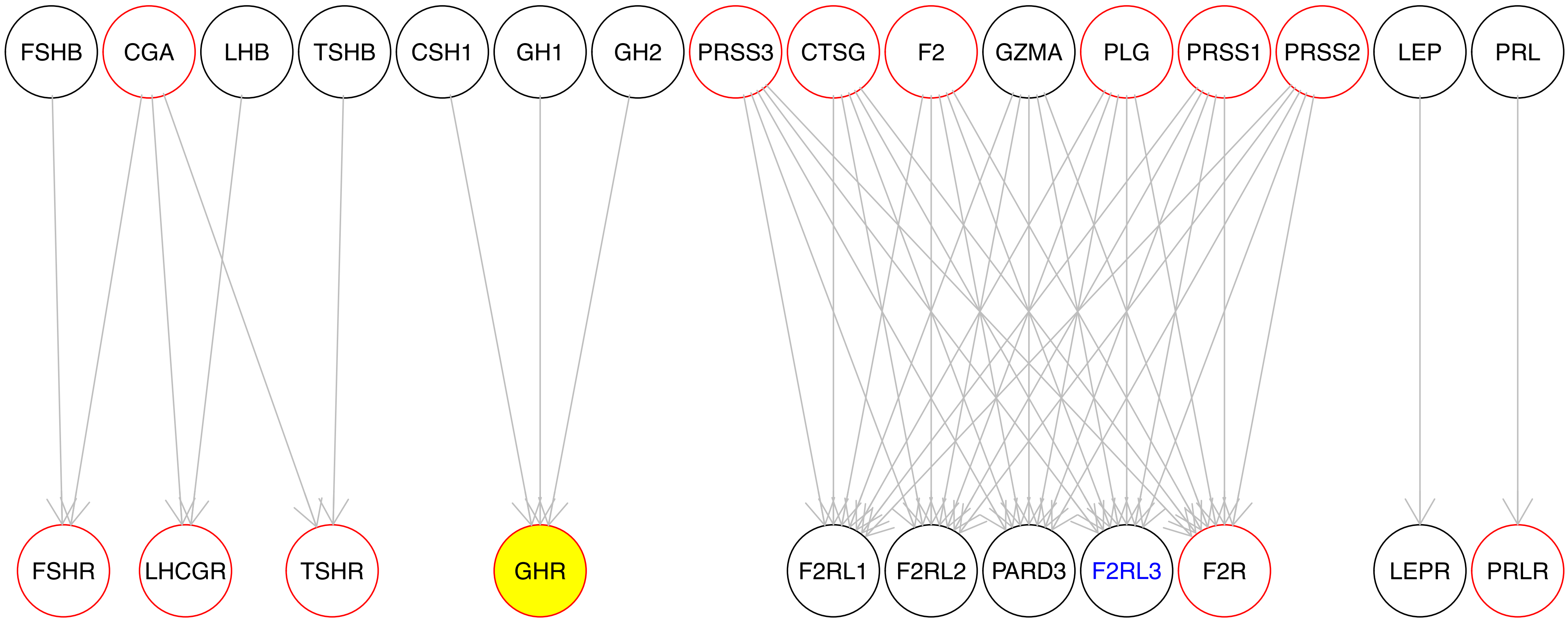

KEGG pathway = Neuroactive ligand−receptor interaction : tumour = ME029 : Yellow Fill = gene variant, Blue Text = expression−survival association, Red Boarder = drug

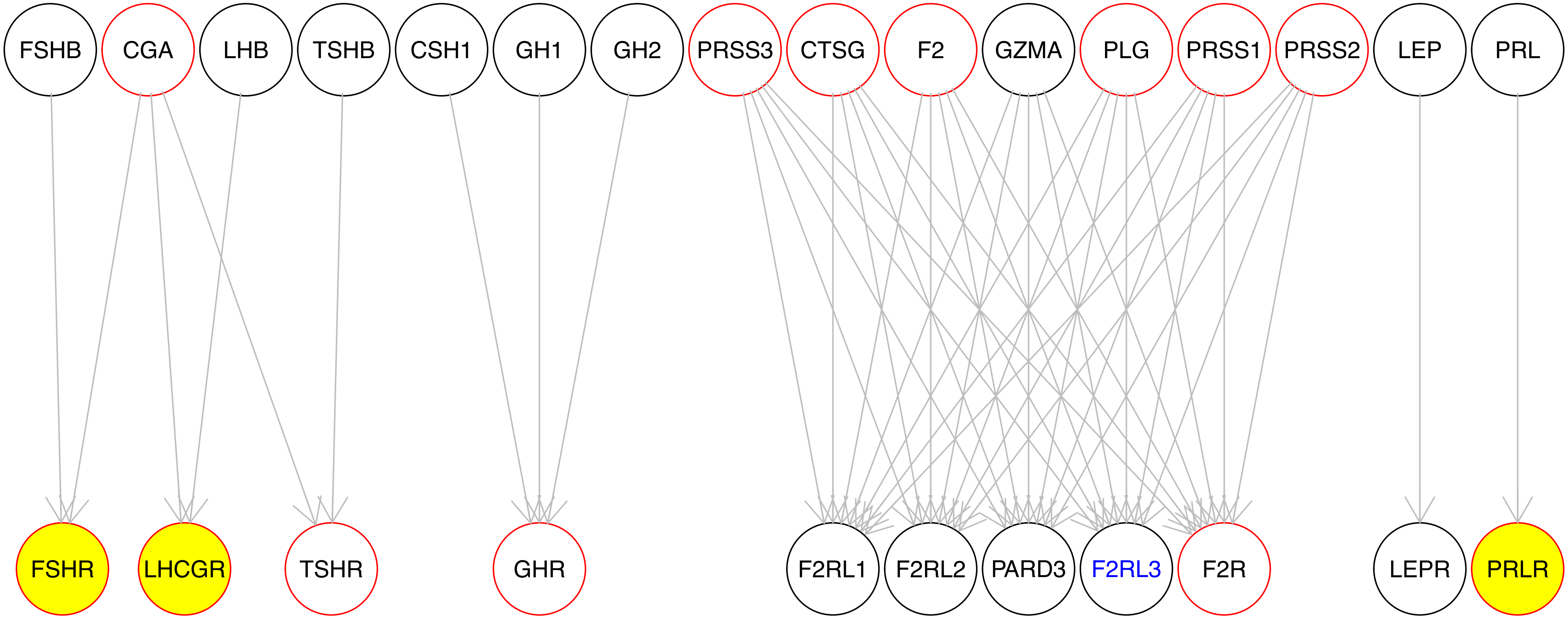

KEGG pathway = Neuroactive ligand−receptor interaction : tumour = ME030 : Yellow Fill = gene variant, Blue Text = expression−survival association, Red Boarder = drug

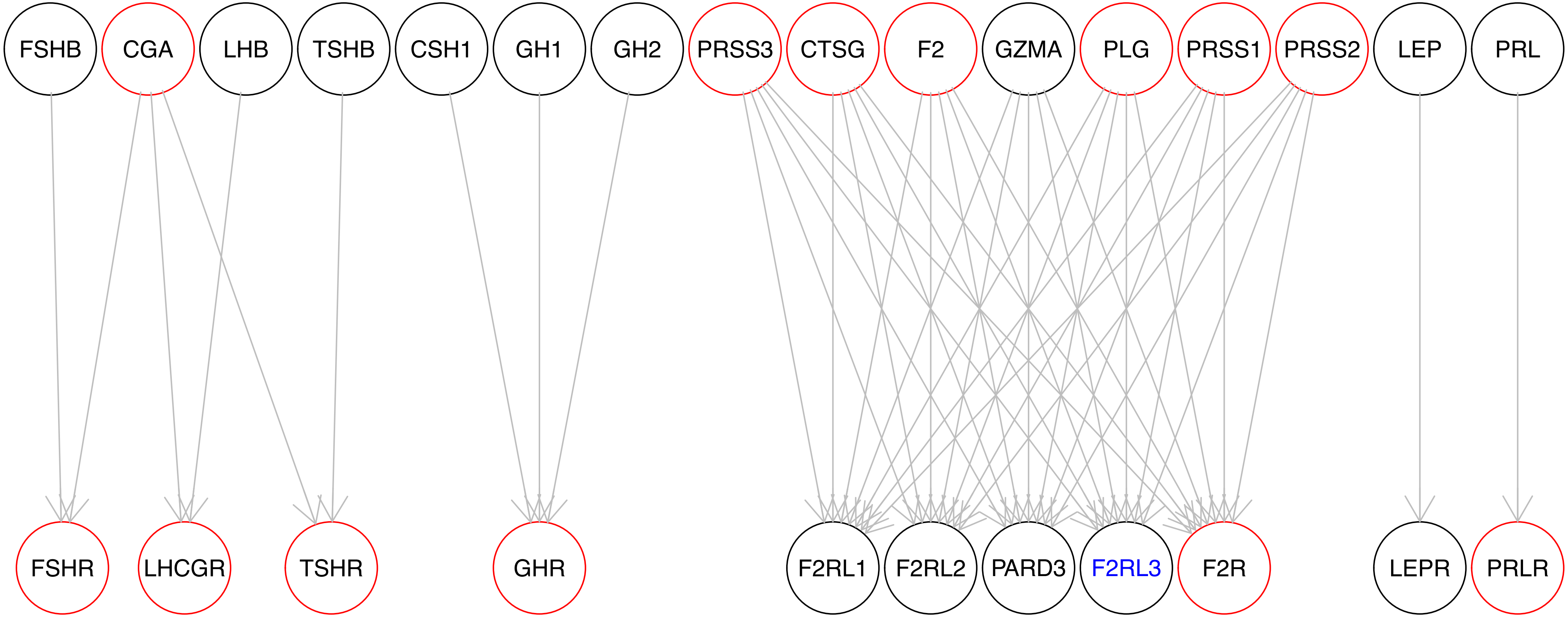

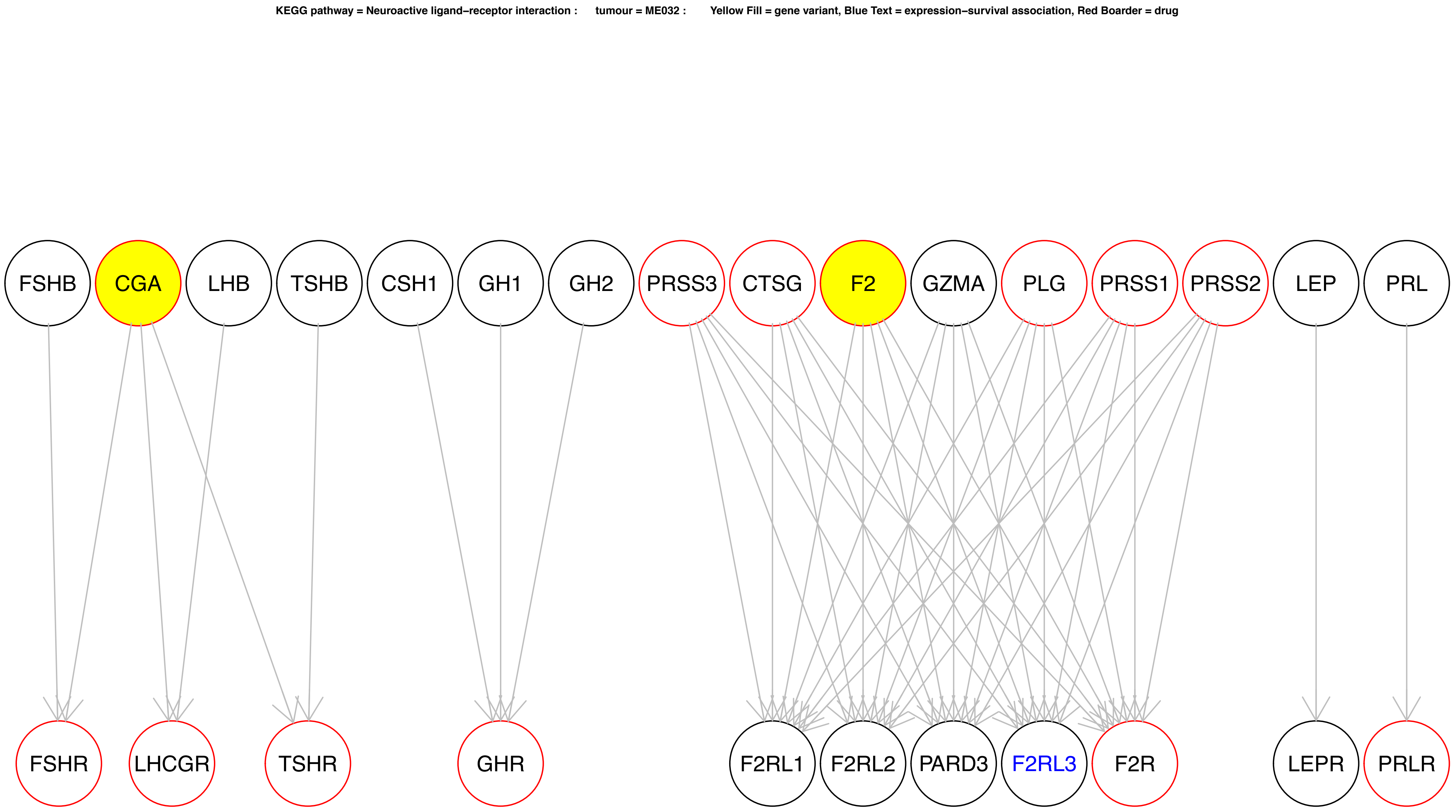
KEGG pathway = Neuroactive ligand−receptor interaction :   tumour = ME032 :   Yellow Fill = gene variant, Blue Text = expression−survival association, Red Boarder = drug
FSHB
CGA
LHB
TSHB
CSH1
GH1
GH2
PRSS3
CTSG
F2
GZMA
PLG
PRSS1
PRSS2
LEP
PRL
FSHR
LHCGR
TSHR
GHR
F2RL1
F2RL2
PARD3
F2RL3
F2R
LEPR
PRLR

KEGG pathway = Neuroactive ligand−receptor interaction :   tumour = ME035 :   Yellow Fill = gene variant, Blue Text = expression−survival association, Red Boarder = drug

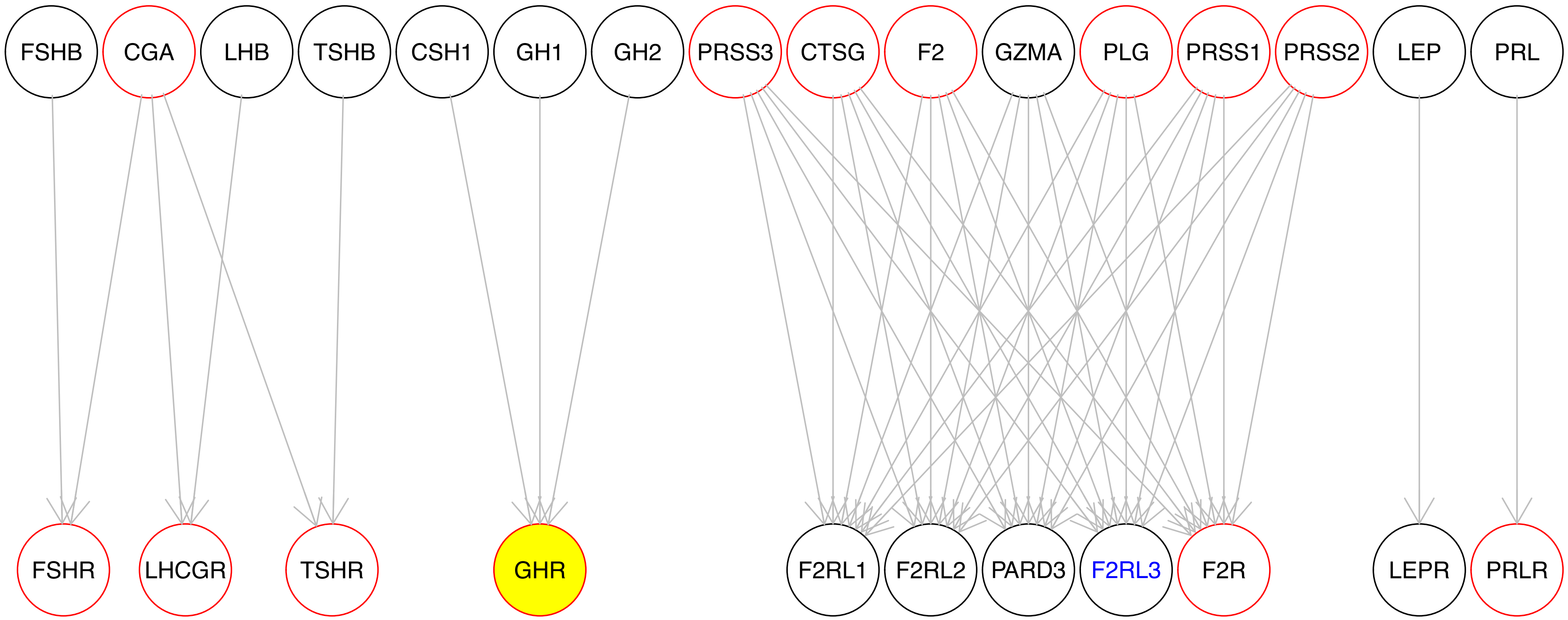

KEGG pathway = Neuroactive ligand−receptor interaction :   tumour = ME037 :   Yellow Fill = gene variant, Blue Text = expression−survival association, Red Boarder = drug

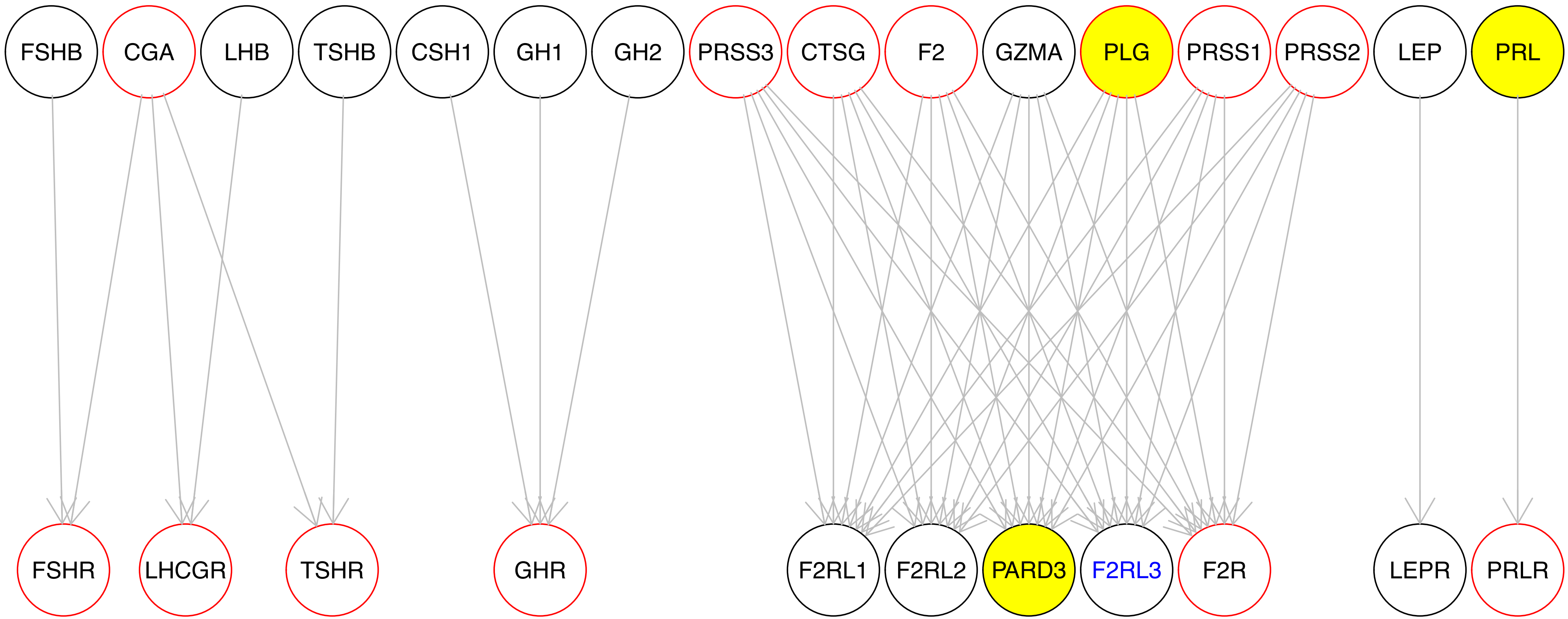

KEGG pathway = Neuroactive ligand−receptor interaction : tumour = ME041 : Yellow Fill = gene variant, Blue Text = expression−survival association, Red Boarder = drug

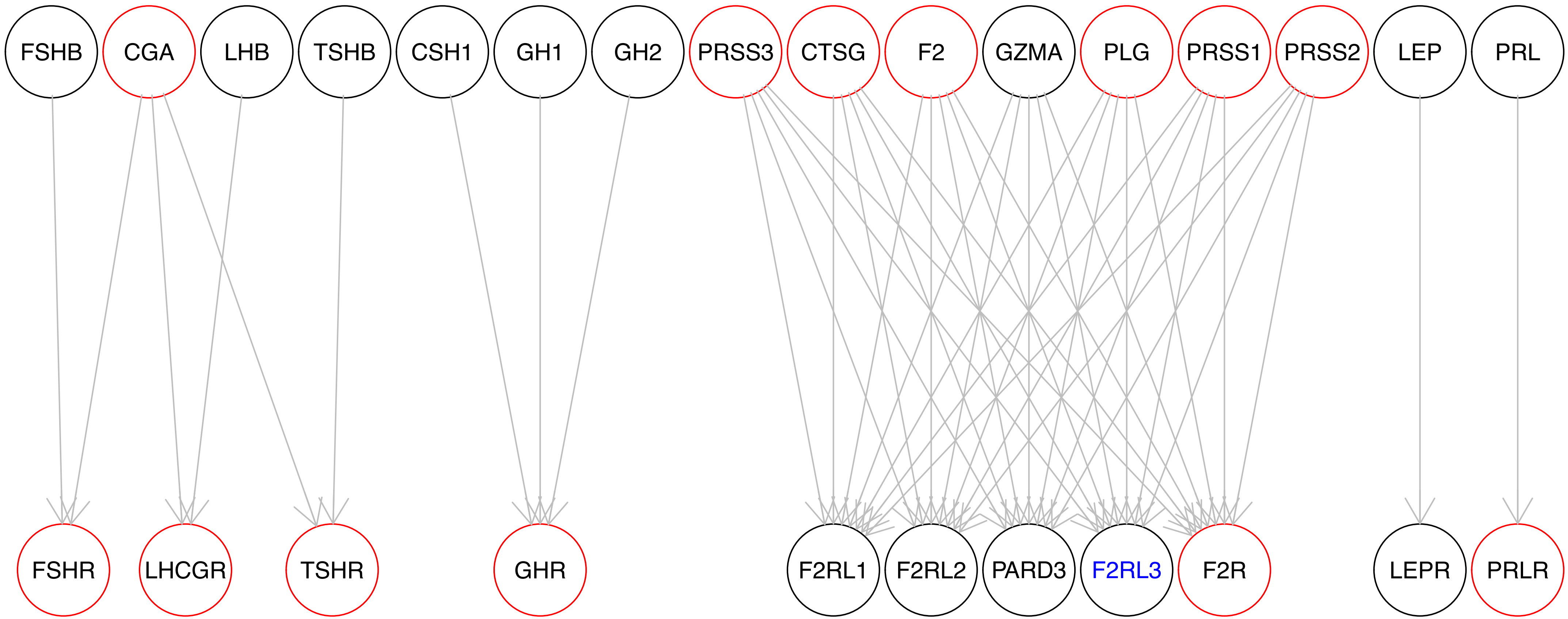

KEGG pathway = Neuroactive ligand−receptor interaction :   tumour = ME043 :   Yellow Fill = gene variant, Blue Text = expression−survival association, Red Boarder = drug

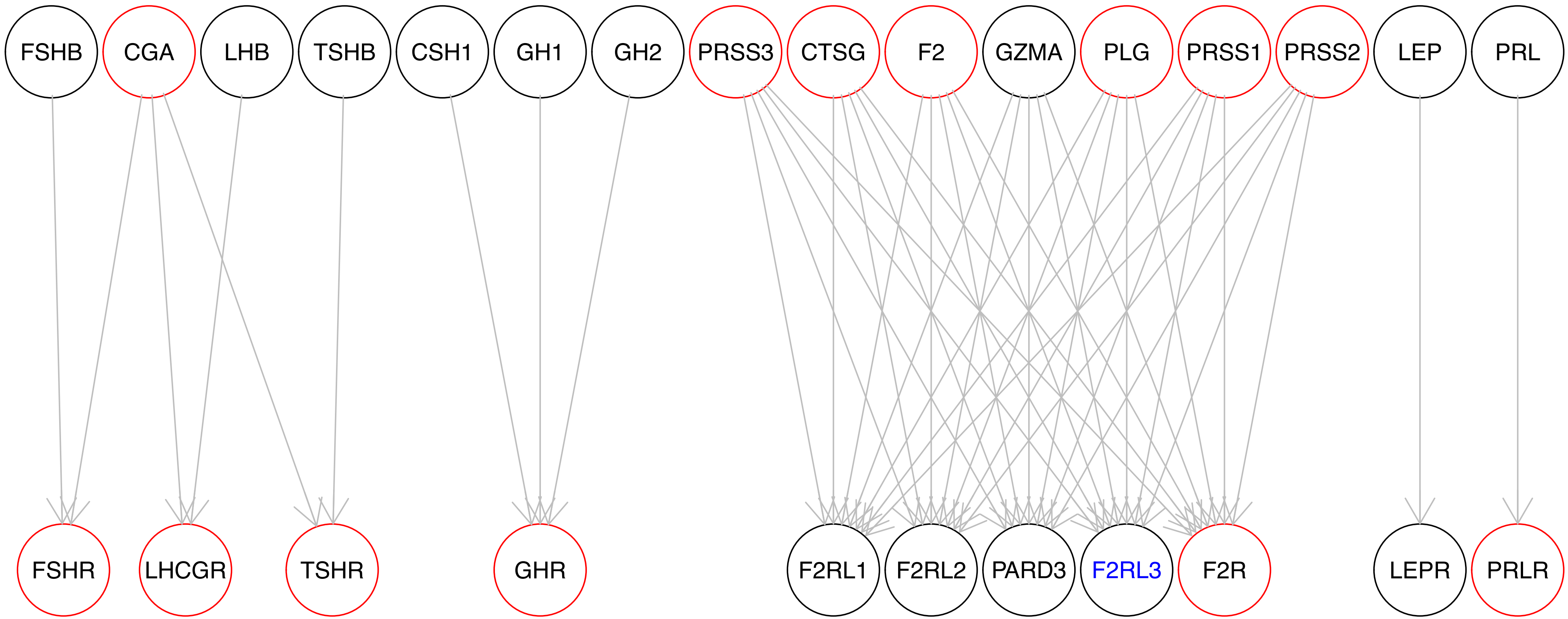

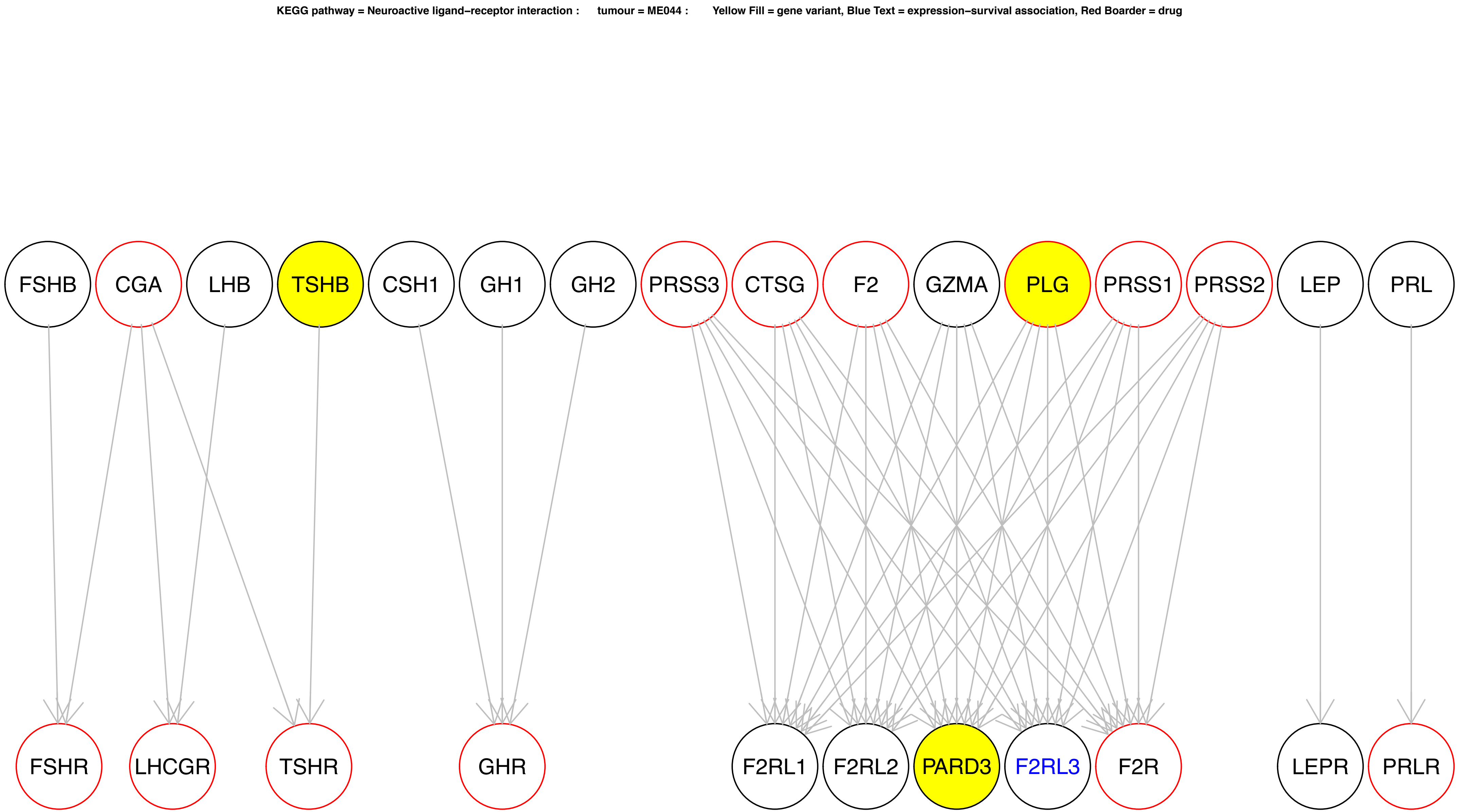

KEGG pathway = Neuroactive ligand−receptor interaction : tumour = ME044 : Yellow Fill = gene variant, Blue Text = expression−survival association, Red Boarder = drug
FSHB
CGA
LHB
TSHB
CSH1
GH1
GH2
PRSS3
CTSG
F2
GZMA
PLG
PRSS1
PRSS2
LEP
PRL
FSHR
LHCGR
TSHR
GHR
F2RL1
F2RL2
PARD3
F2RL3
F2R
LEPR
PRLR

KEGG pathway = Neuroactive ligand−receptor interaction : tumour = ME045 : Yellow Fill = gene variant, Blue Text = expression−survival association, Red Boarder = drug

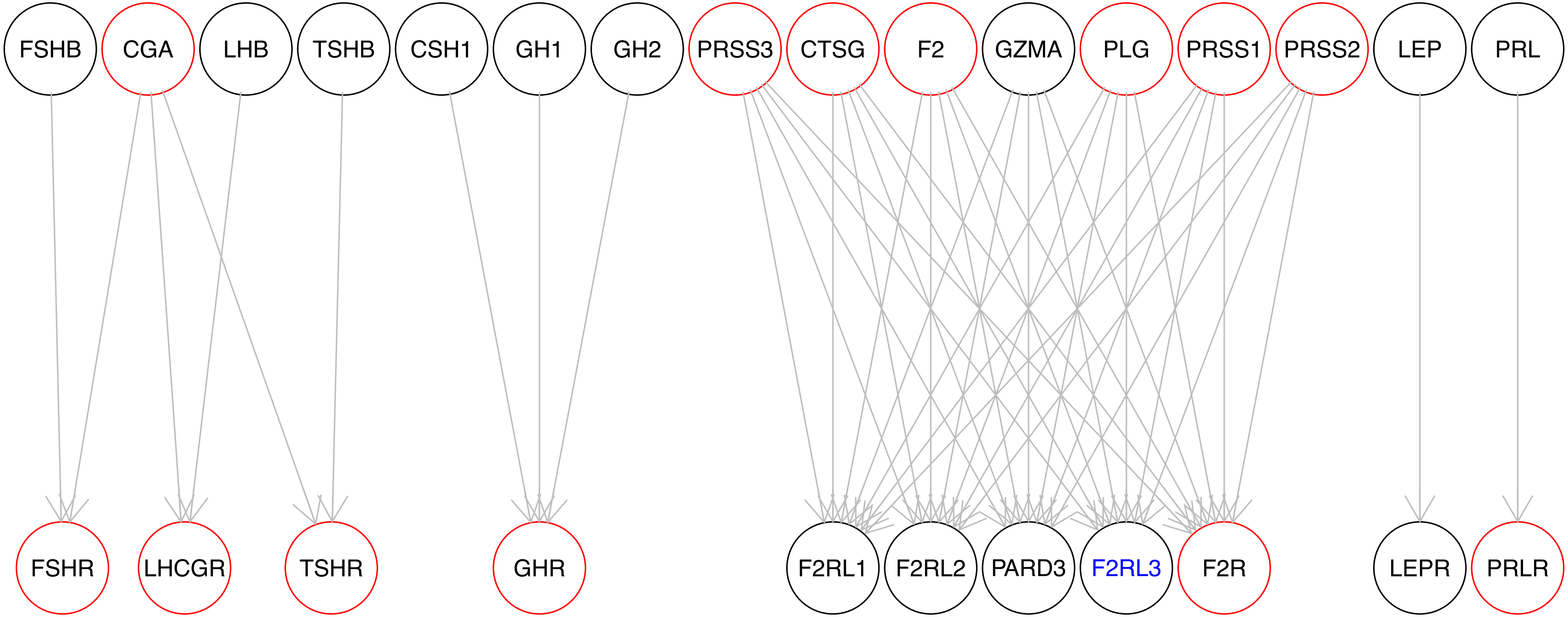

KEGG pathway = Neuroactive ligand−receptor interaction : tumour = ME048 : Yellow Fill = gene variant, Blue Text = expression−survival association, Red Boarder = drug

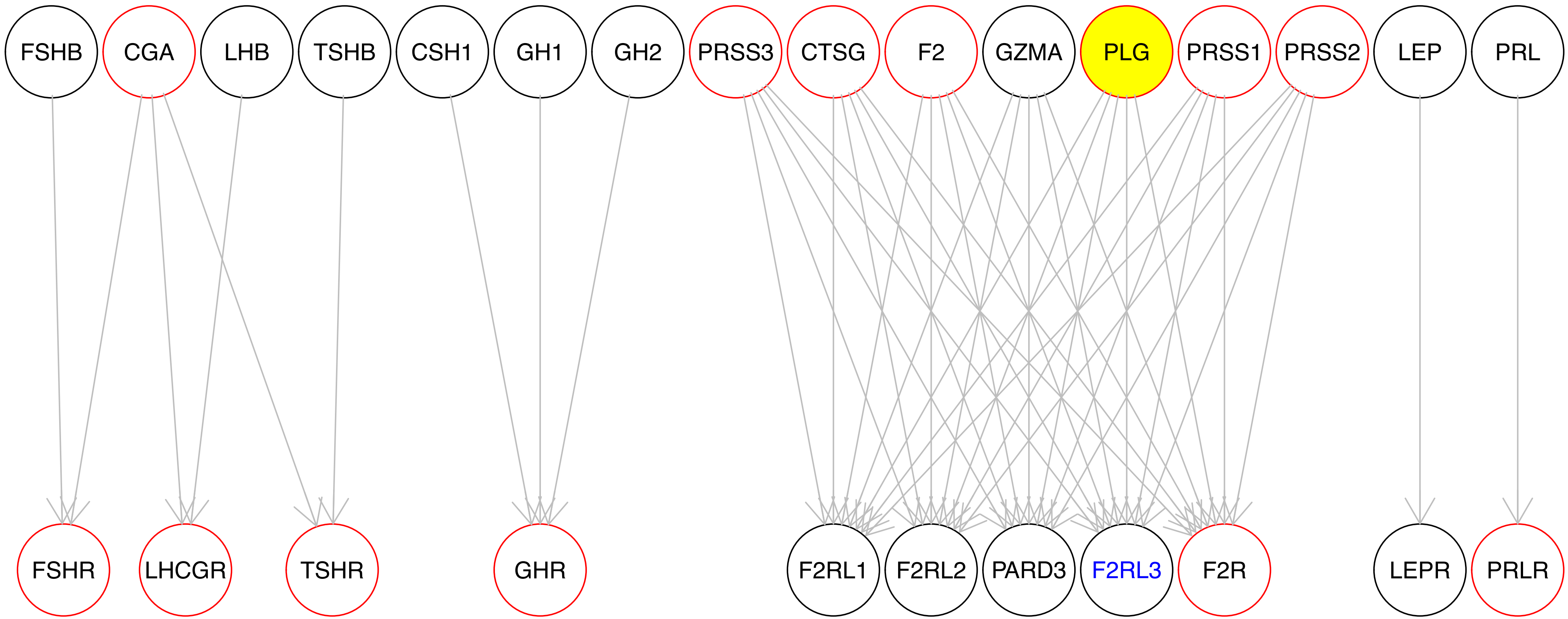

KEGG pathway = Neuroactive ligand−receptor interaction :   tumour = ME049 :   Yellow Fill = gene variant, Blue Text = expression−survival association, Red Boarder = drug

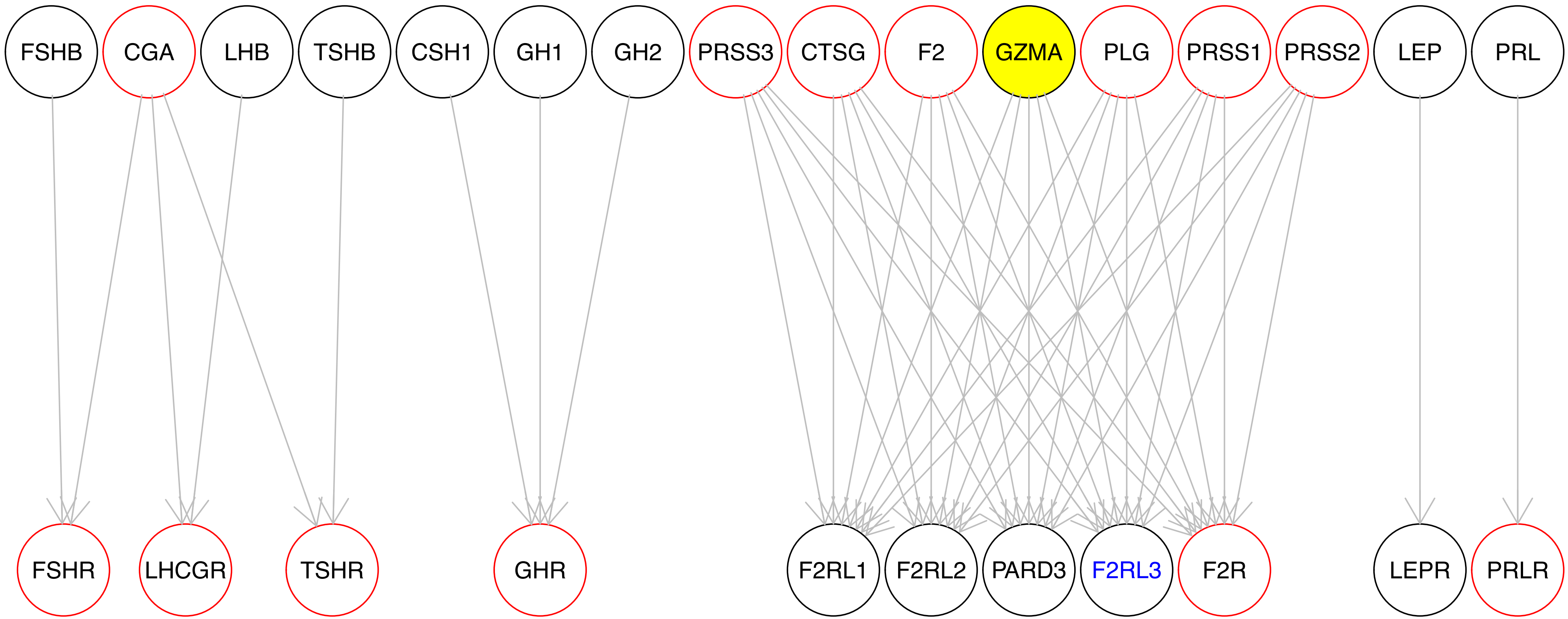

KEGG pathway = Neuroactive ligand−receptor interaction :   tumour = ME050 :   Yellow Fill = gene variant, Blue Text = expression−survival association, Red Boarder = drug

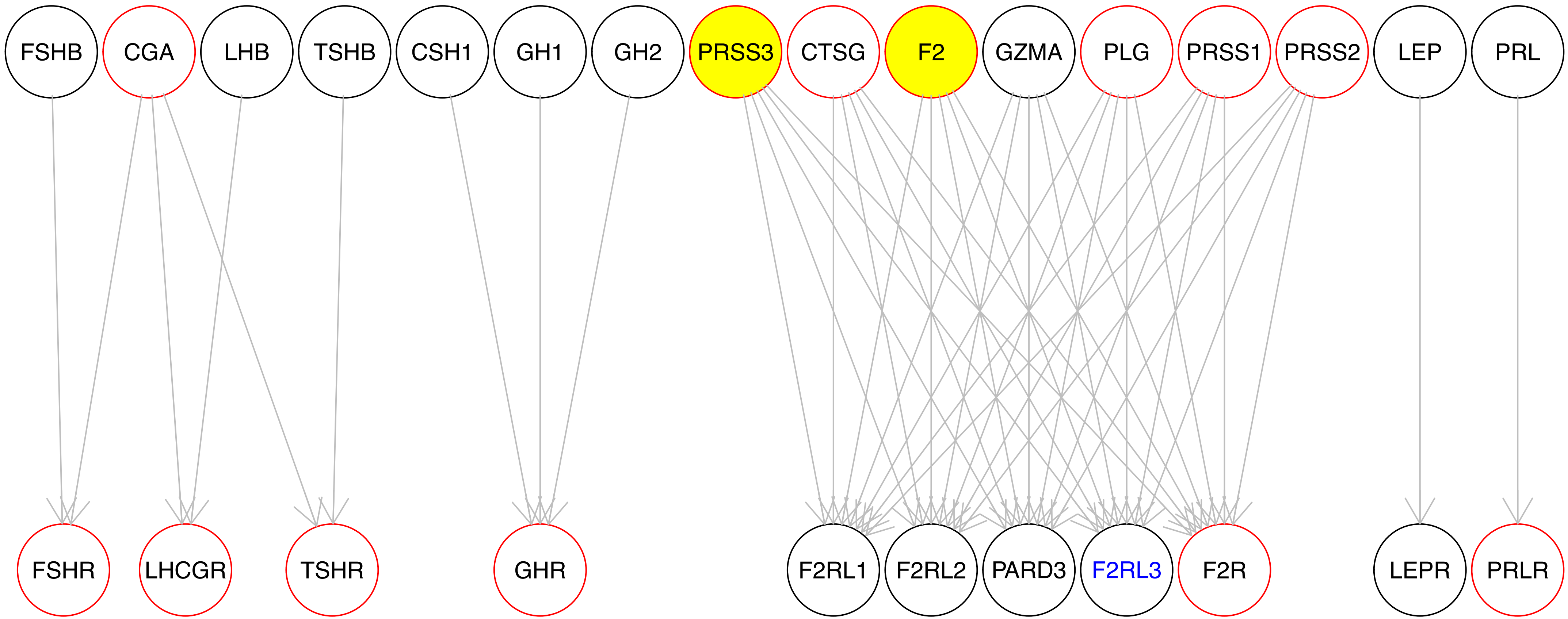

KEGG pathway = Neuroactive ligand−receptor interaction : tumour = ME100L : Yellow Fill = gene variant, Blue Text = expression−survival association, Red Boarder = drug

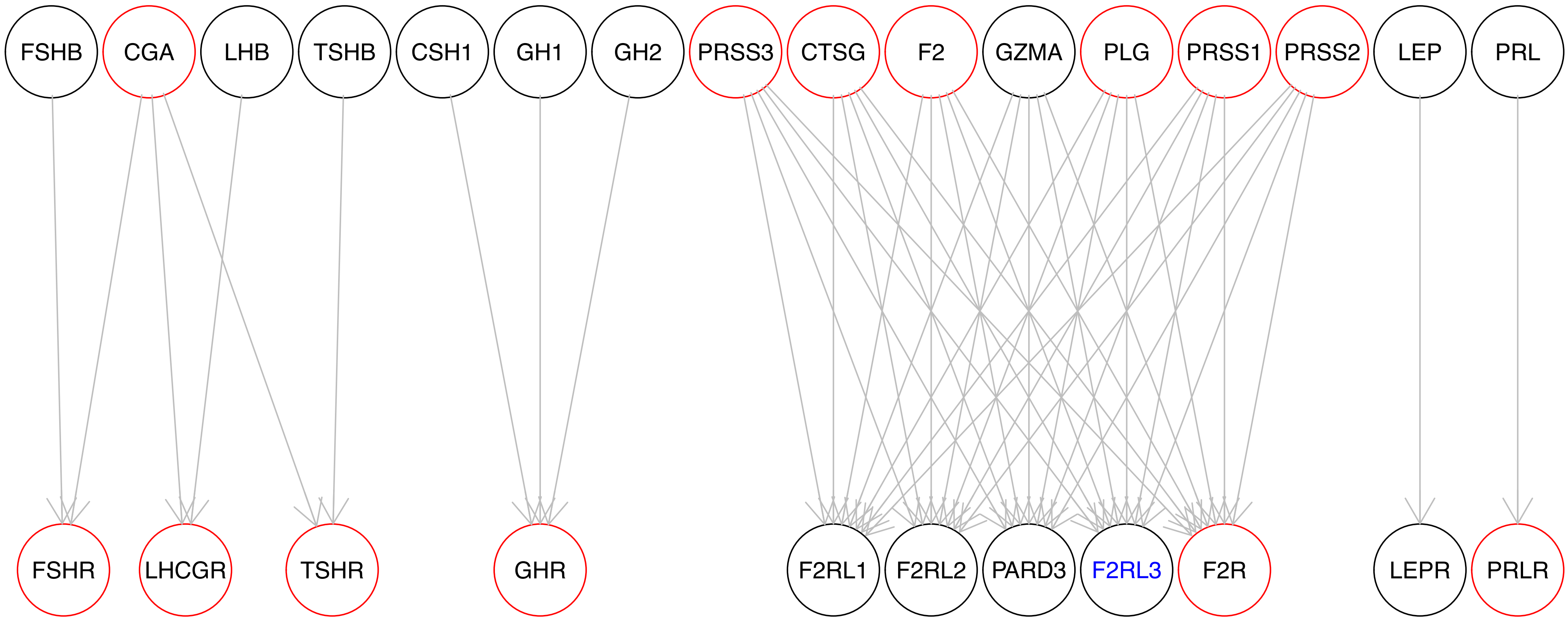

KEGG pathway = Neuroactive ligand−receptor interaction :   tumour = A101D_SKIN :   Yellow Fill = gene variant, Blue Text = expression−survival association, Red Boarder = drug

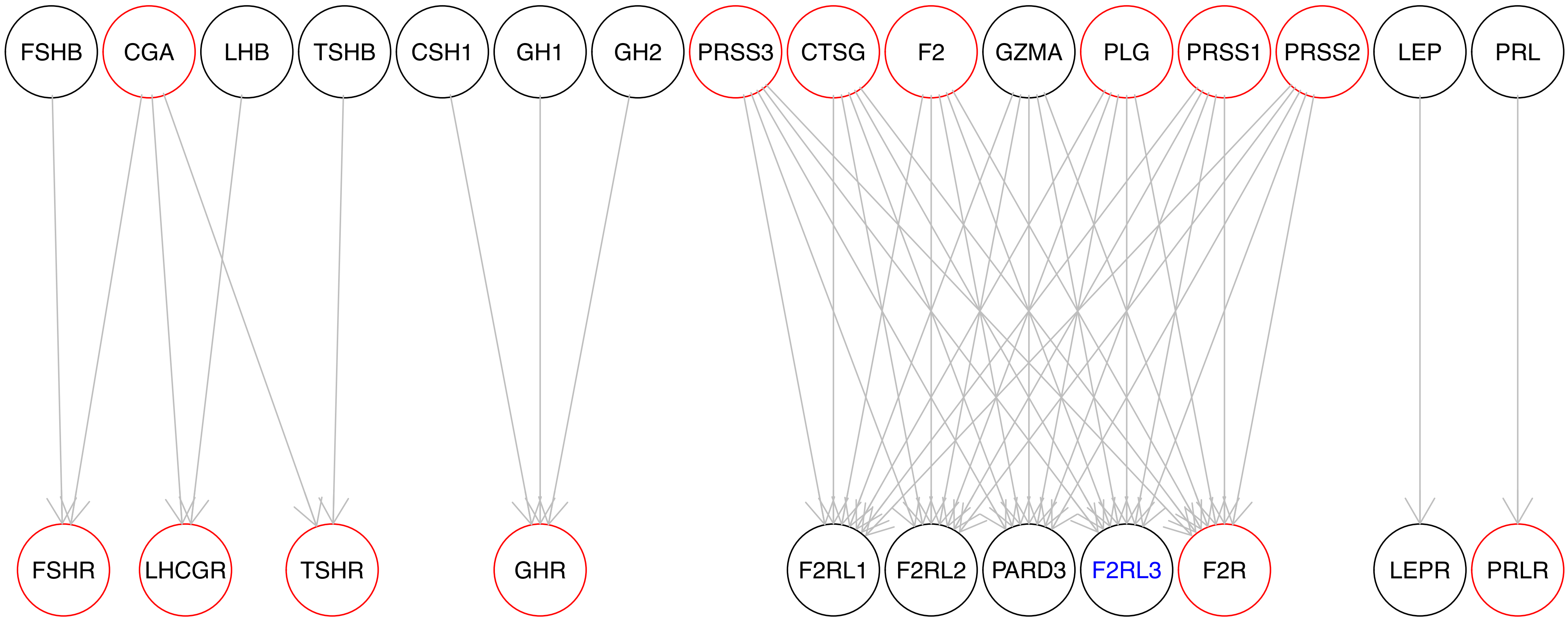

KEGG pathway = Neuroactive ligand−receptor interaction : tumour = A2058_SKIN : Yellow Fill = gene variant, Blue Text = expression−survival association, Red Boarder = drug

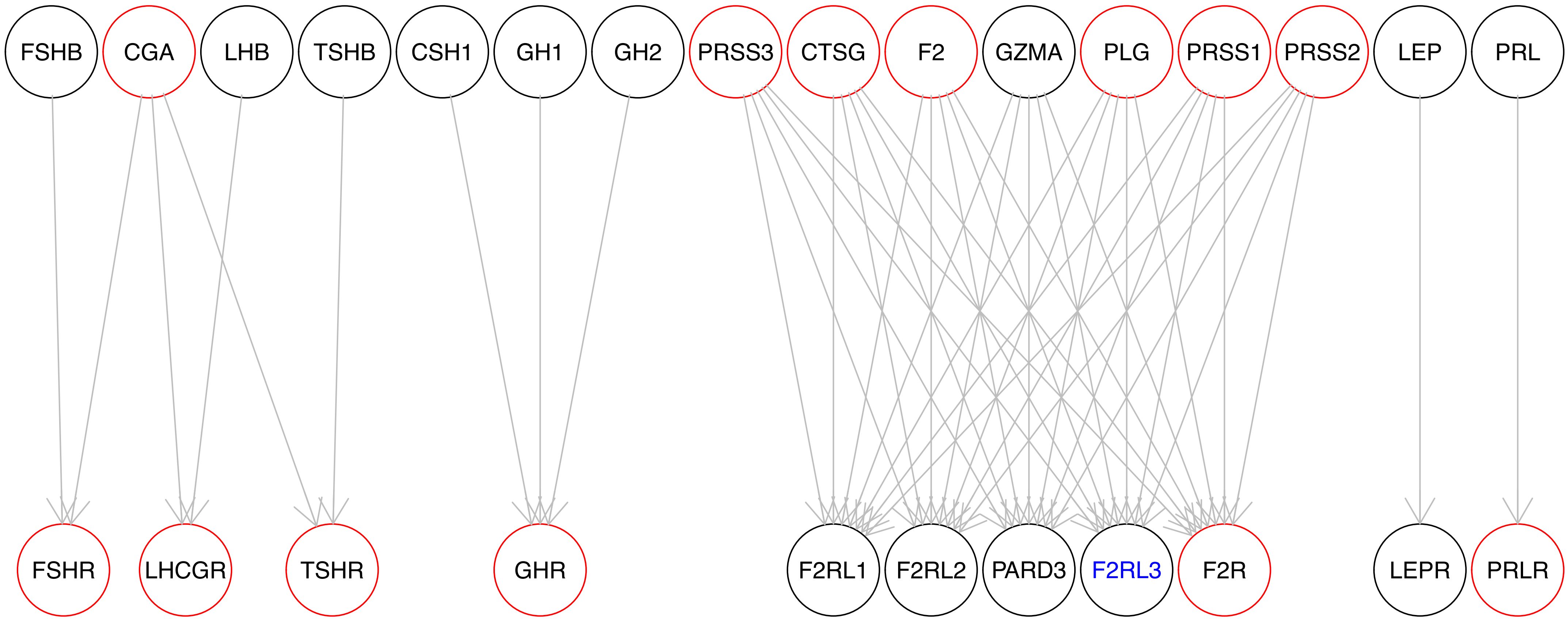

KEGG pathway = Neuroactive ligand−receptor interaction : tumour = A375_SKIN : Yellow Fill = gene variant, Blue Text = expression−survival association, Red Boarder = drug

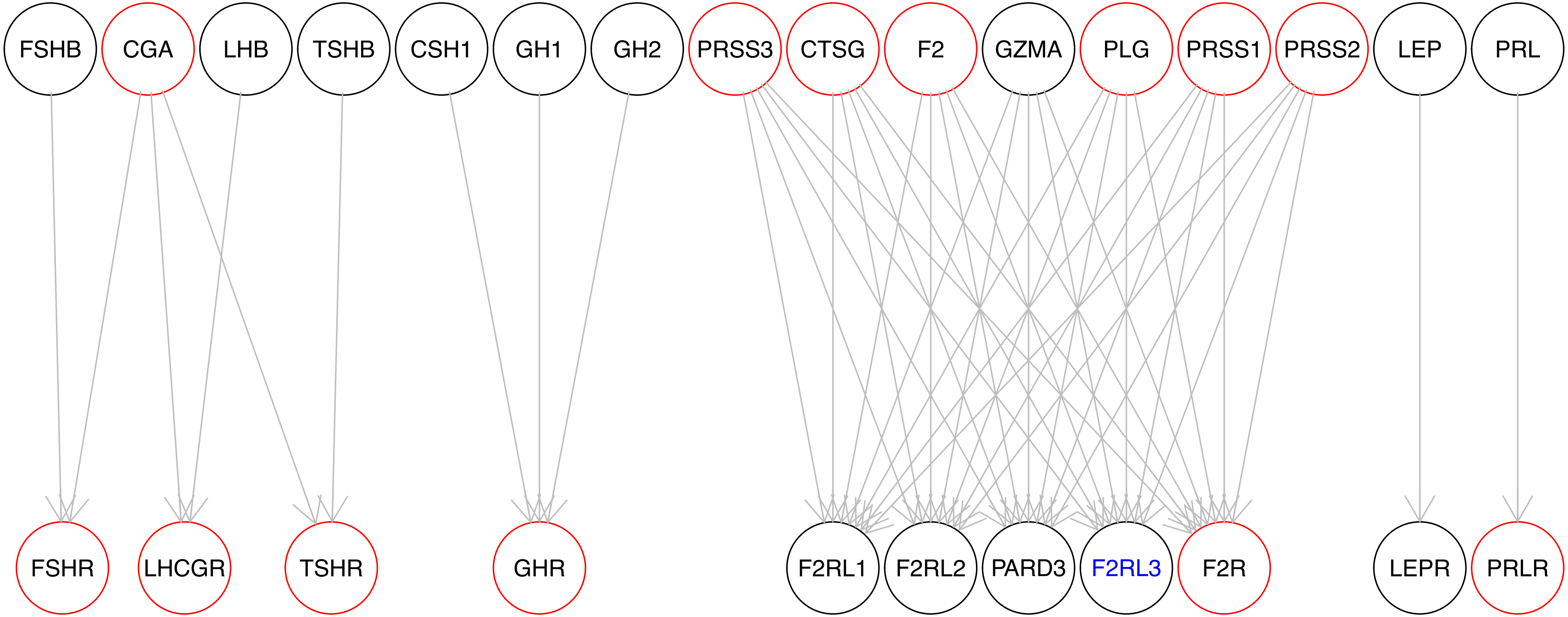

KEGG pathway = Neuroactive ligand−receptor interaction : tumour = C32_SKIN : Yellow Fill = gene variant, Blue Text = expression−survival association, Red Boarder = drug

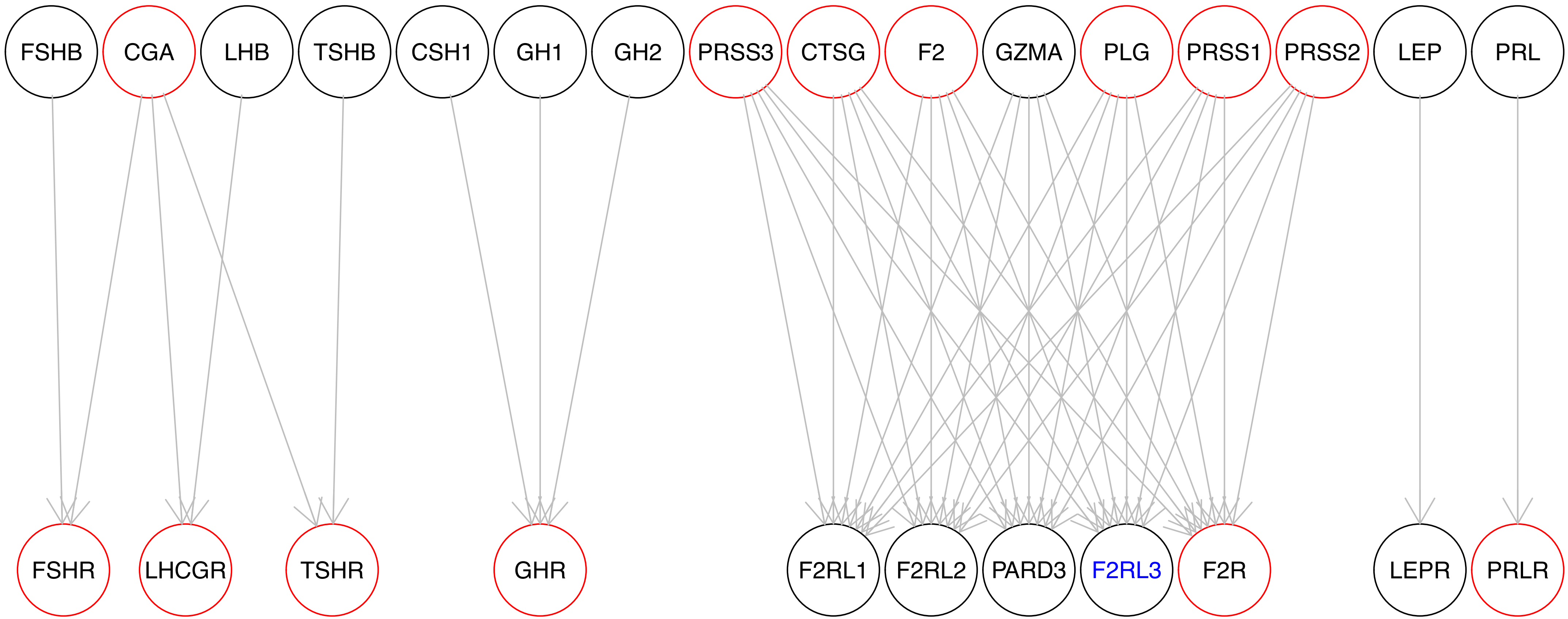

KEGG pathway = Neuroactive ligand–receptor interaction : tumour = CHL1_SKIN : Yellow Fill = gene variant, Blue Text = expression–survival association, Red Boarder = drug

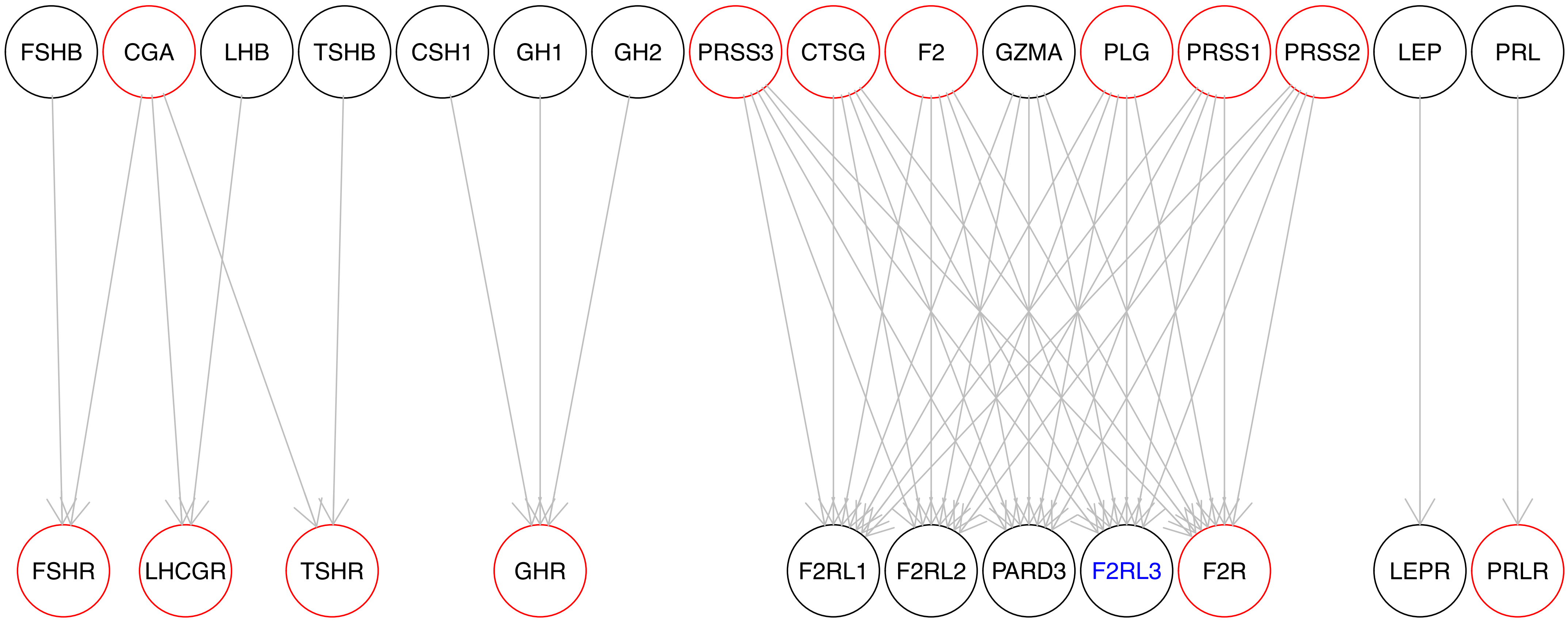

KEGG pathway = Neuroactive ligand−receptor interaction : tumour = CJM_SKIN : Yellow Fill = gene variant, Blue Text = expression−survival association, Red Boarder = drug

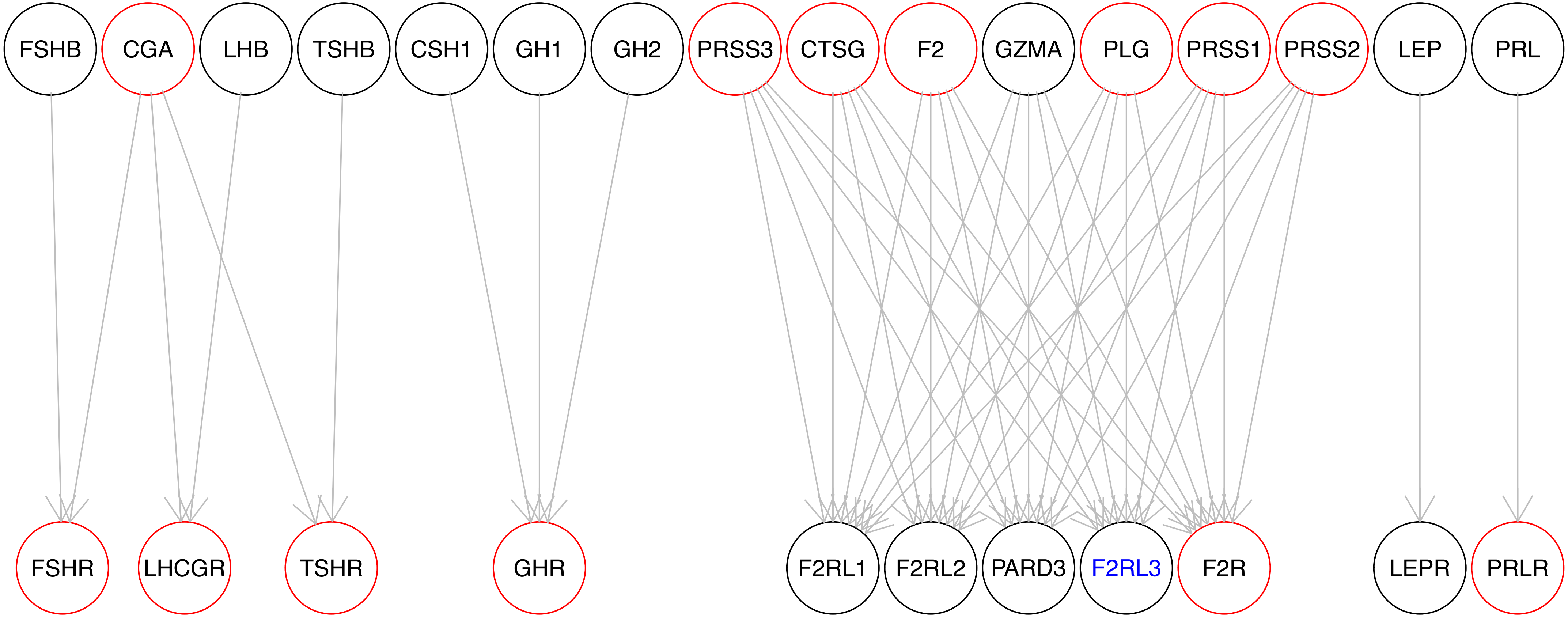

KEGG pathway = Neuroactive ligand−receptor interaction : tumour = COLO679_SKIN : Yellow Fill = gene variant, Blue Text = expression−survival association, Red Boarder = drug

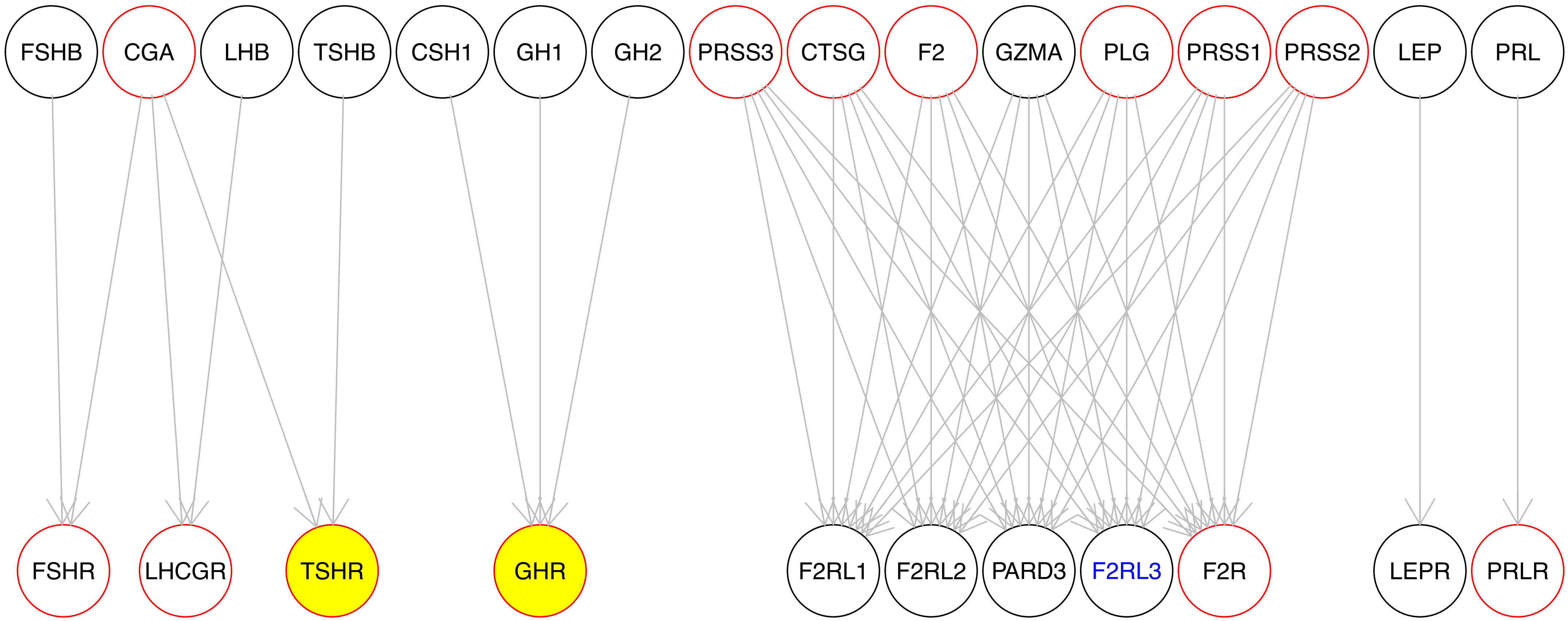

KEGG pathway = Neuroactive ligand−receptor interaction : tumour = COLO741_SKIN : Yellow Fill = gene variant, Blue Text = expression−survival association, Red Boarder = drug

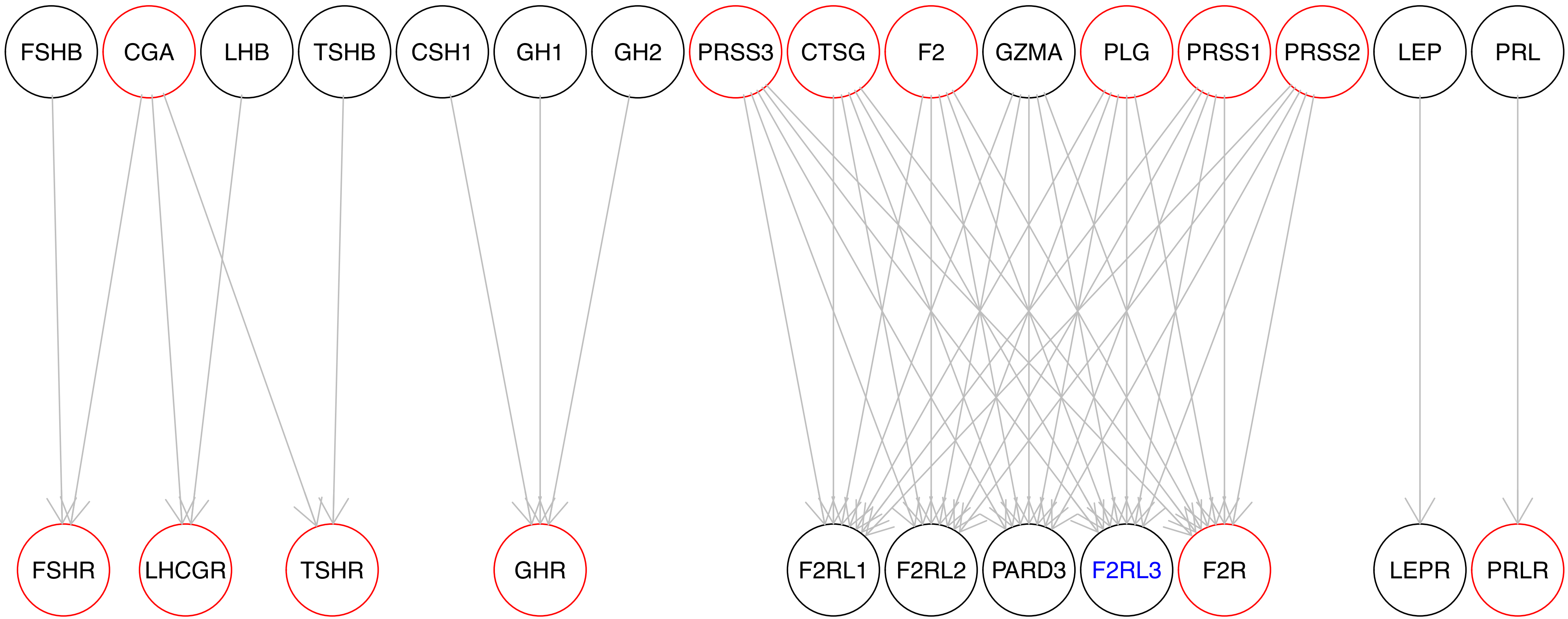

KEGG pathway = Neuroactive ligand−receptor interaction : tumour = COLO783_SKIN : Yellow Fill = gene variant, Blue Text = expression−survival association, Red Boarder = drug

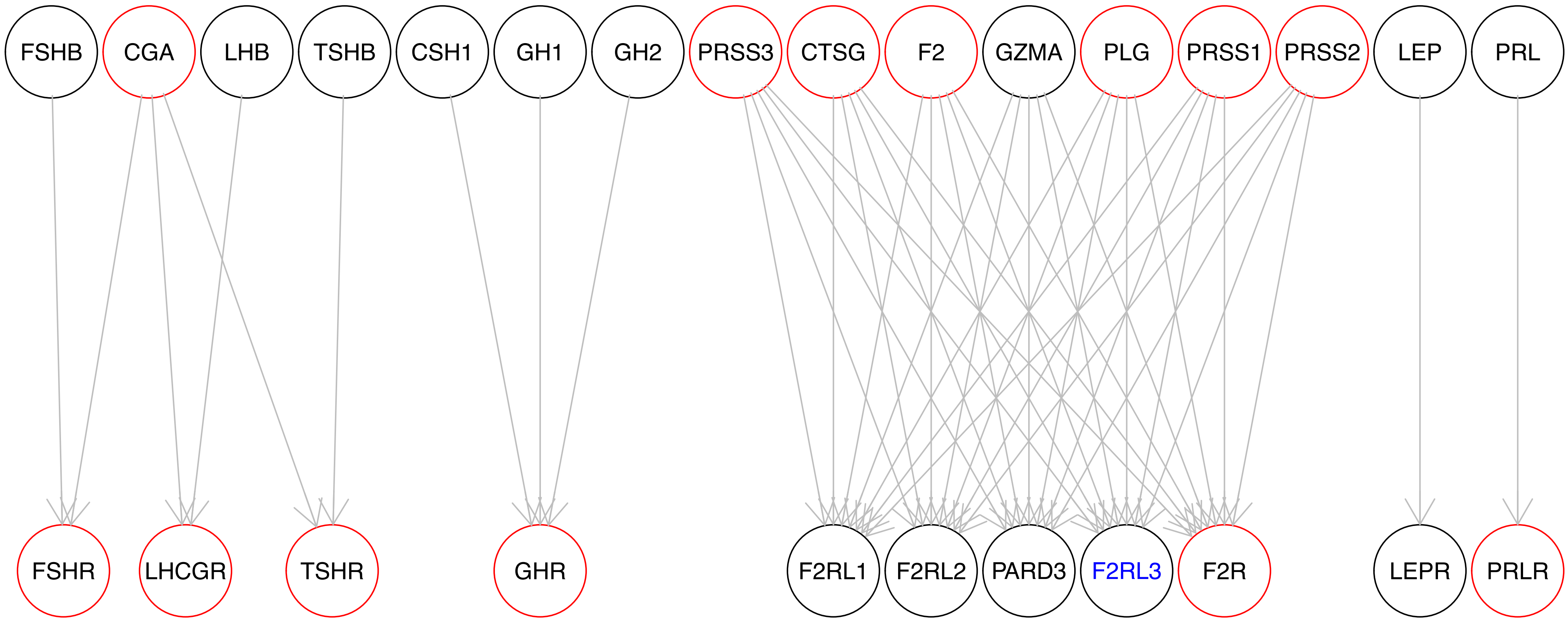

KEGG pathway = Neuroactive ligand−receptor interaction :   tumour = COLO792_SKIN :   Yellow Fill = gene variant, Blue Text = expression−survival association, Red Boarder = drug

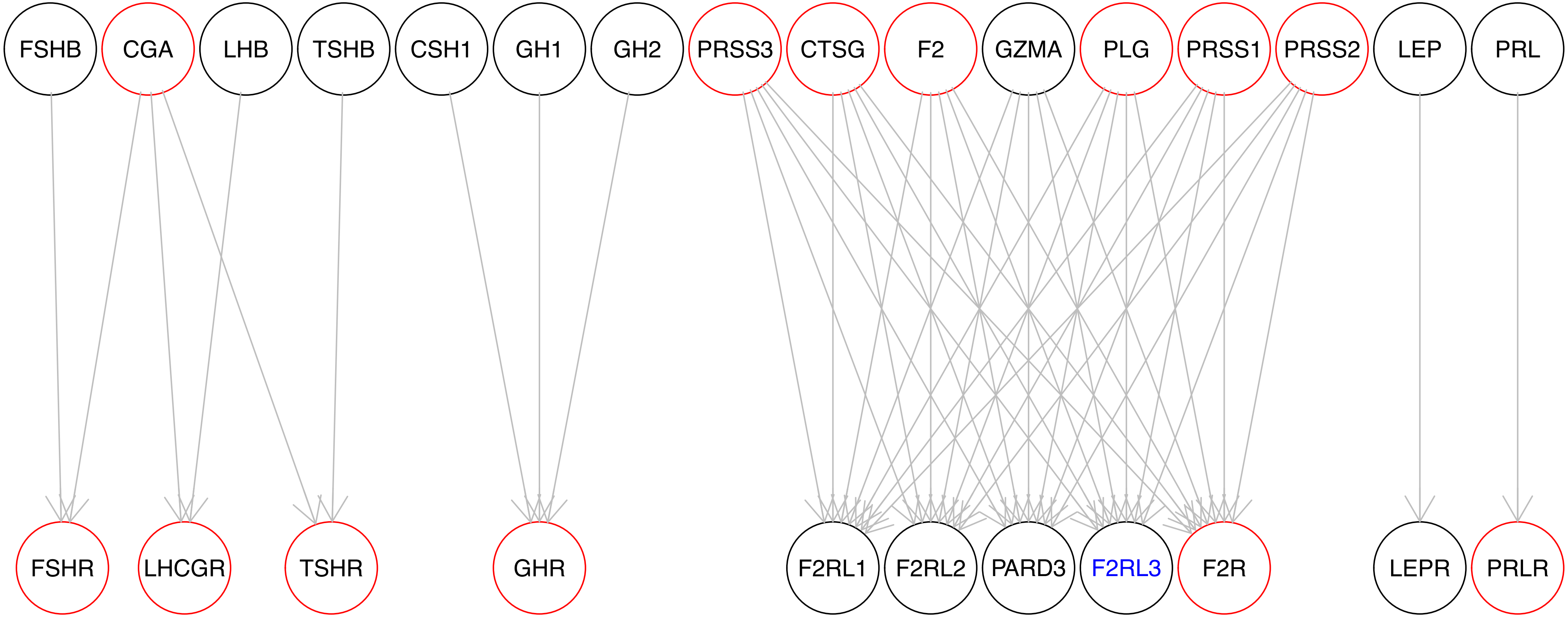

KEGG pathway = Neuroactive ligand−receptor interaction : tumour = COLO818_SKIN : Yellow Fill = gene variant, Blue Text = expression−survival association, Red Boarder = drug

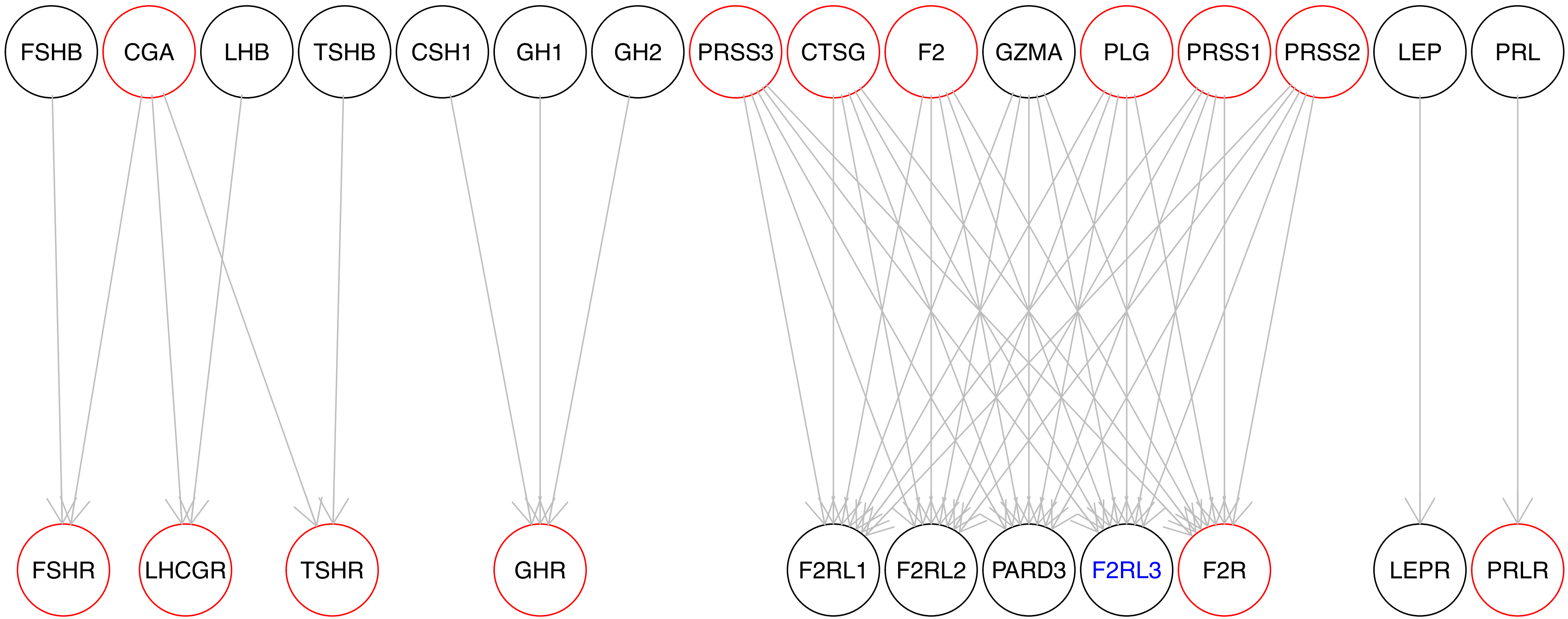

KEGG pathway = Neuroactive ligand−receptor interaction : tumour = COLO829_SKIN : Yellow Fill = gene variant, Blue Text = expression−survival association, Red Boarder = drug

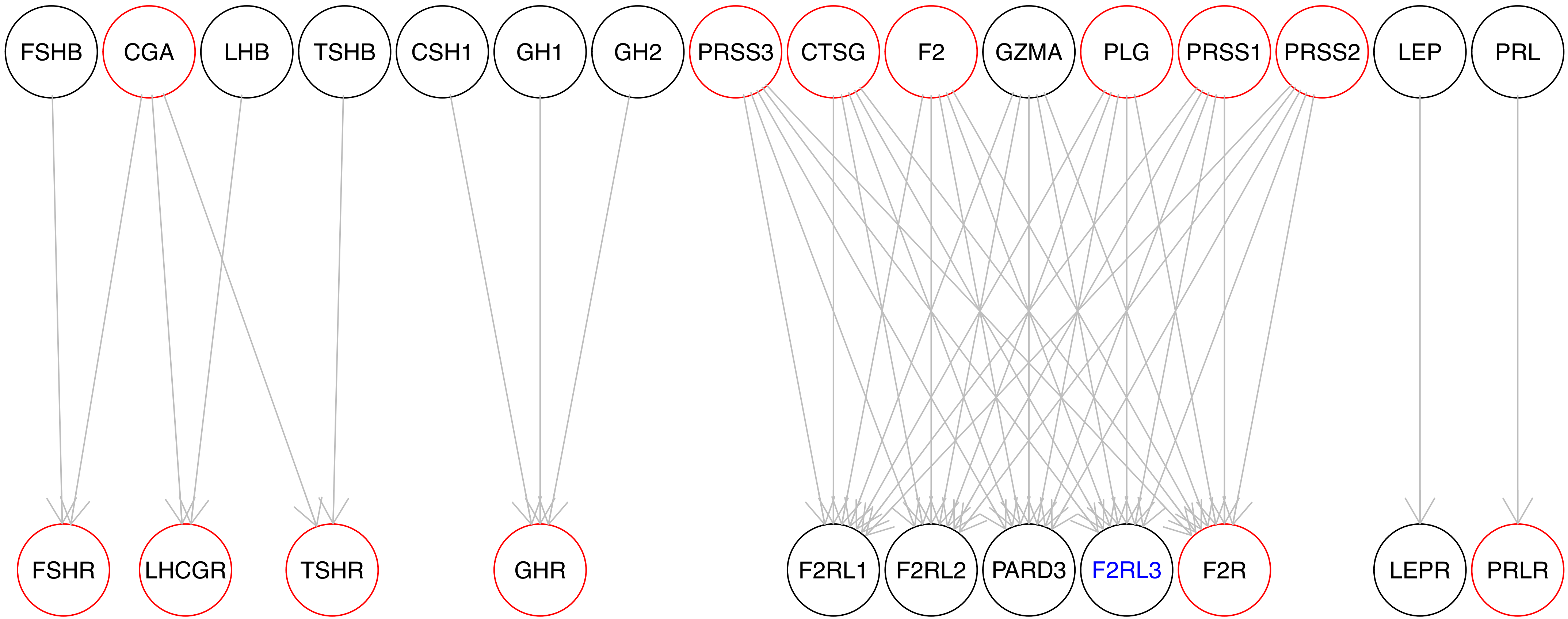

KEGG pathway = Neuroactive ligand−receptor interaction : tumour = G361_SKIN : Yellow Fill = gene variant, Blue Text = expression−survival association, Red Boarder = drug

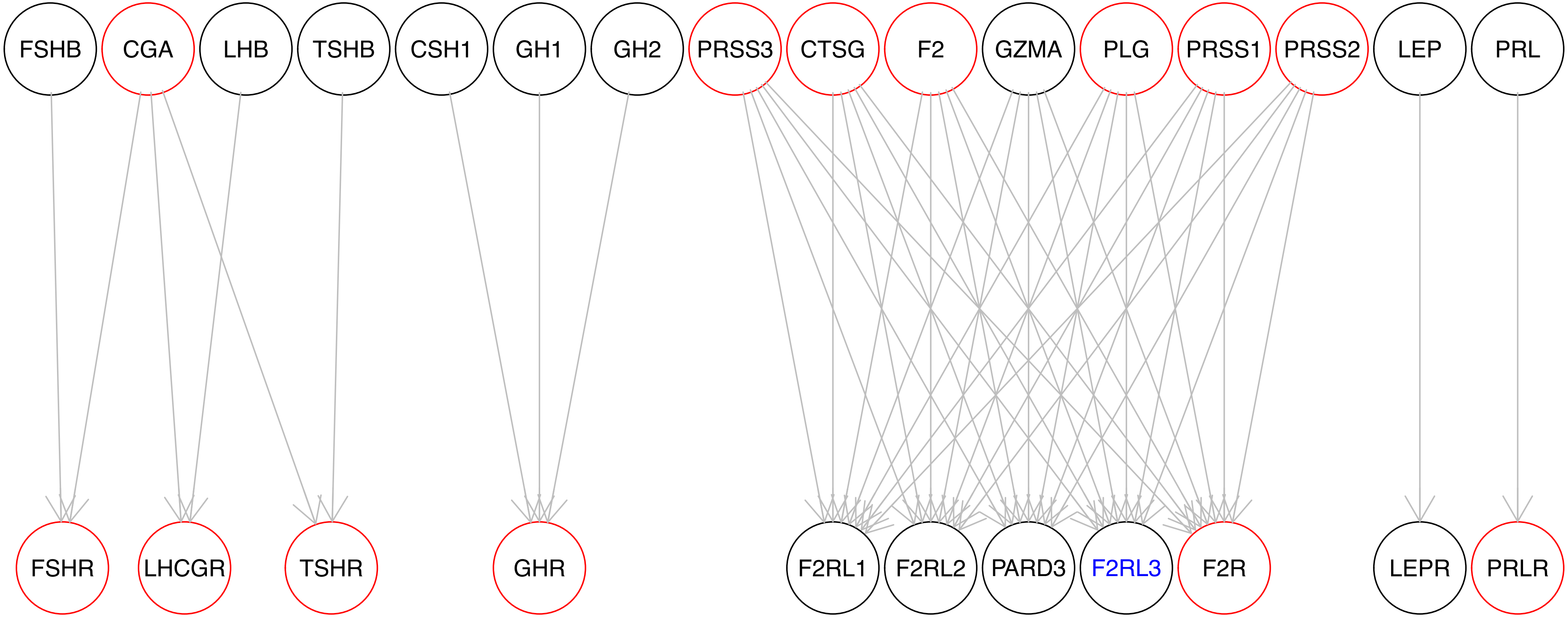

KEGG pathway = Neuroactive ligand−receptor interaction : tumour = HS600T_SKIN : Yellow Fill = gene variant, Blue Text = expression−survival association, Red Boarder = drug

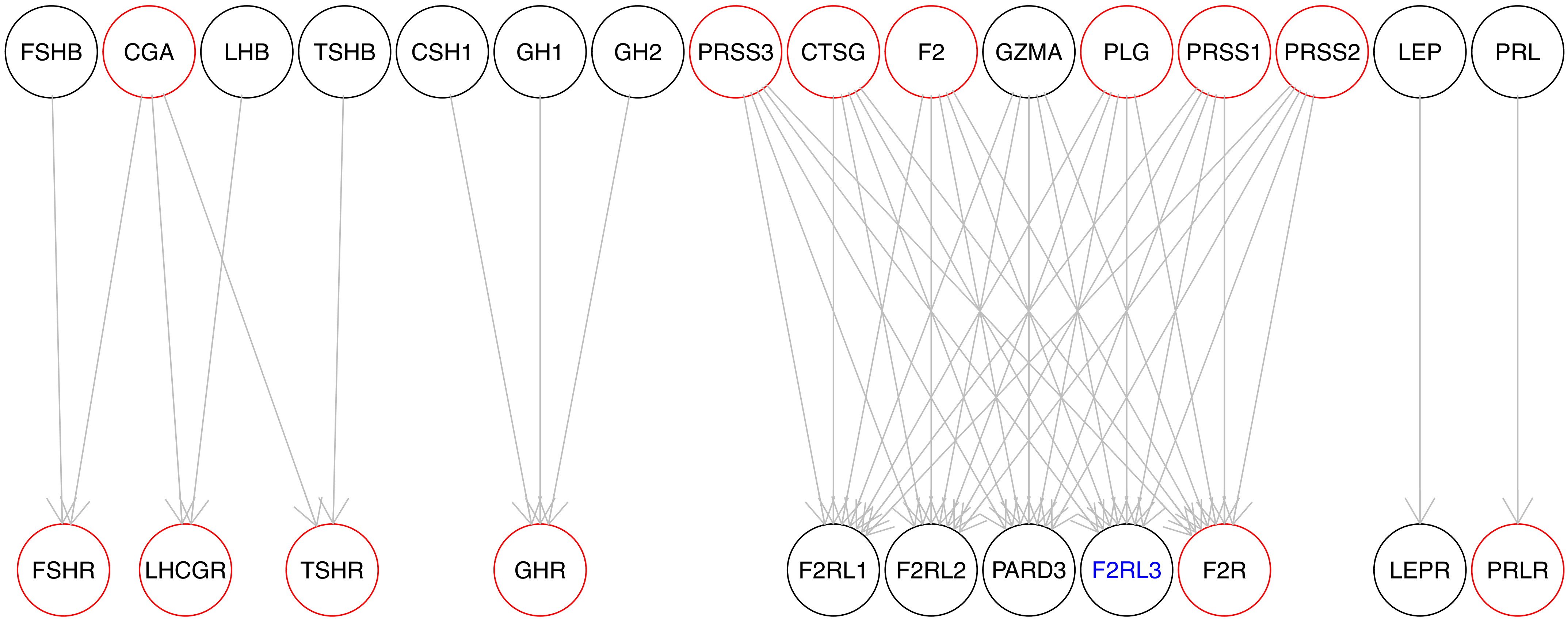

KEGG pathway = Neuroactive ligand−receptor interaction :   tumour = HS688AT_SKIN :   Yellow Fill = gene variant, Blue Text = expression−survival association, Red Boarder = drug

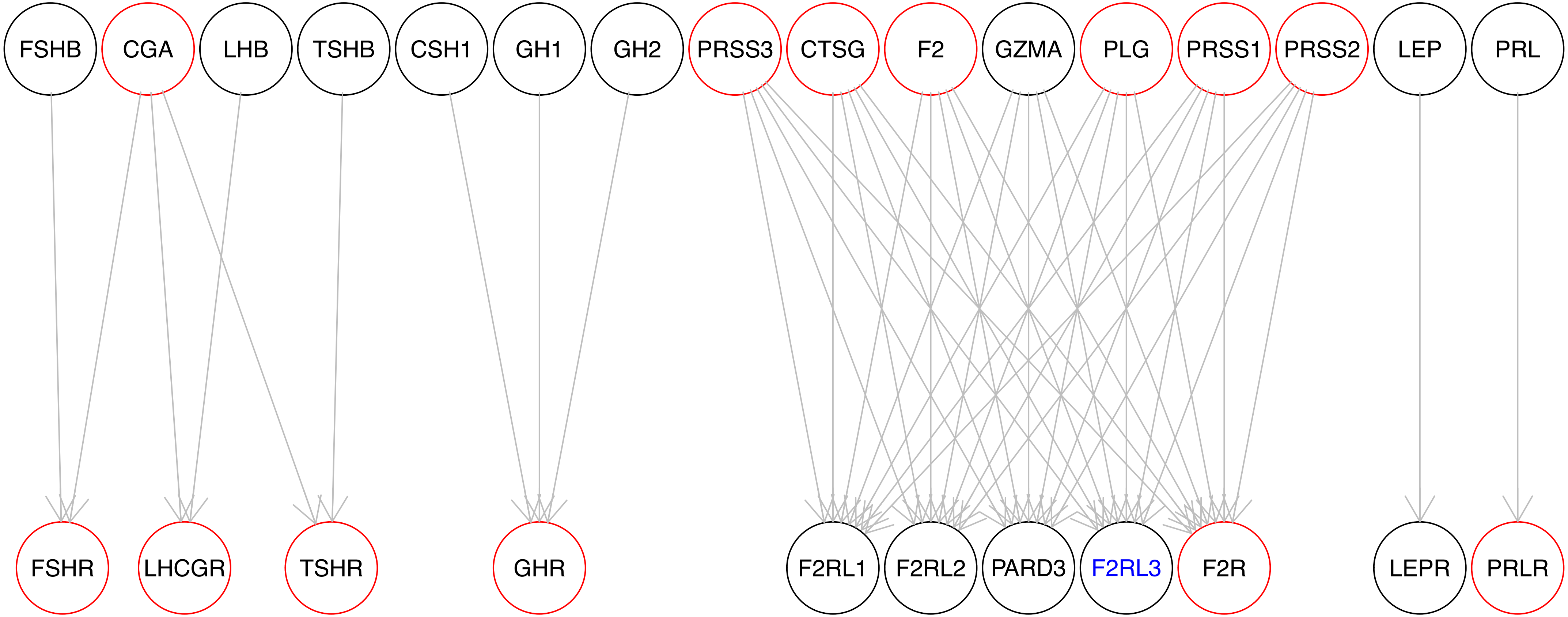

KEGG pathway = Neuroactive ligand−receptor interaction :   tumour = HS695T_SKIN :   Yellow Fill = gene variant, Blue Text = expression−survival association, Red Boarder = drug

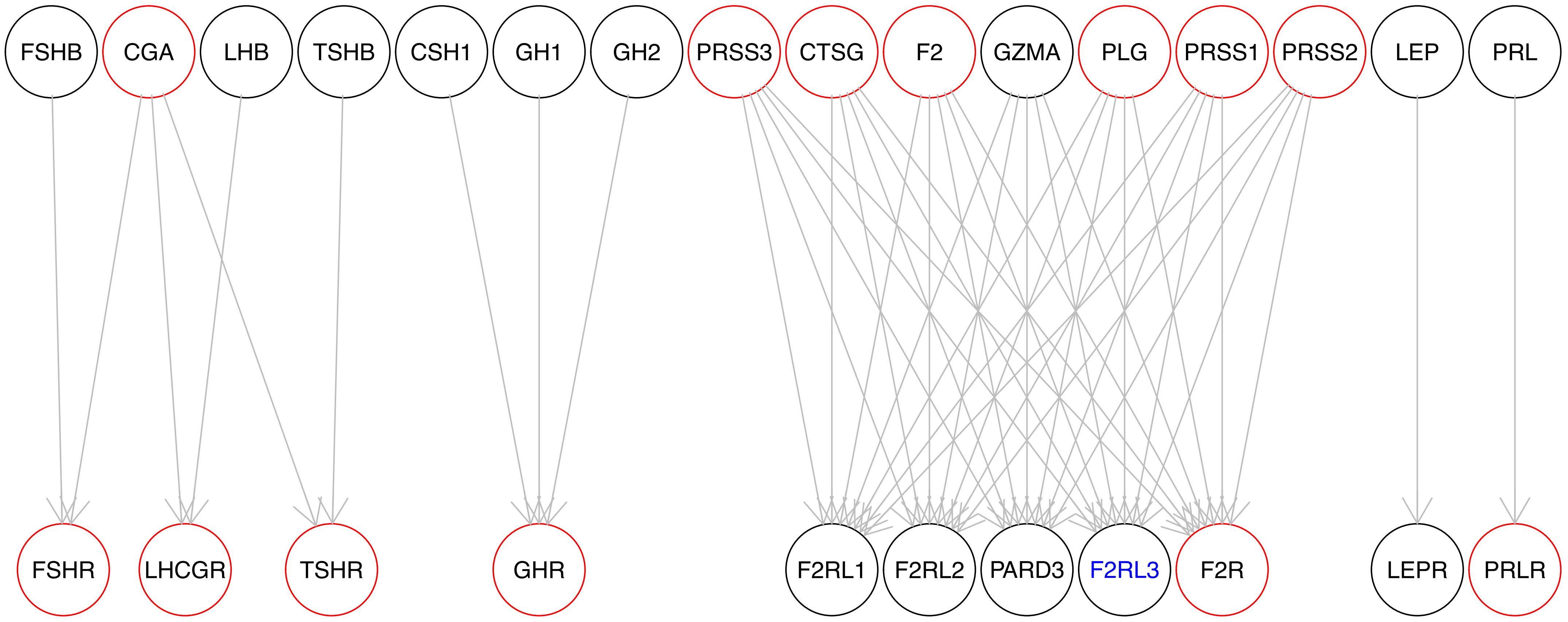

KEGG pathway = Neuroactive ligand−receptor interaction :   tumour = HS834T_SKIN :   Yellow Fill = gene variant, Blue Text = expression−survival association, Red Boarder = drug

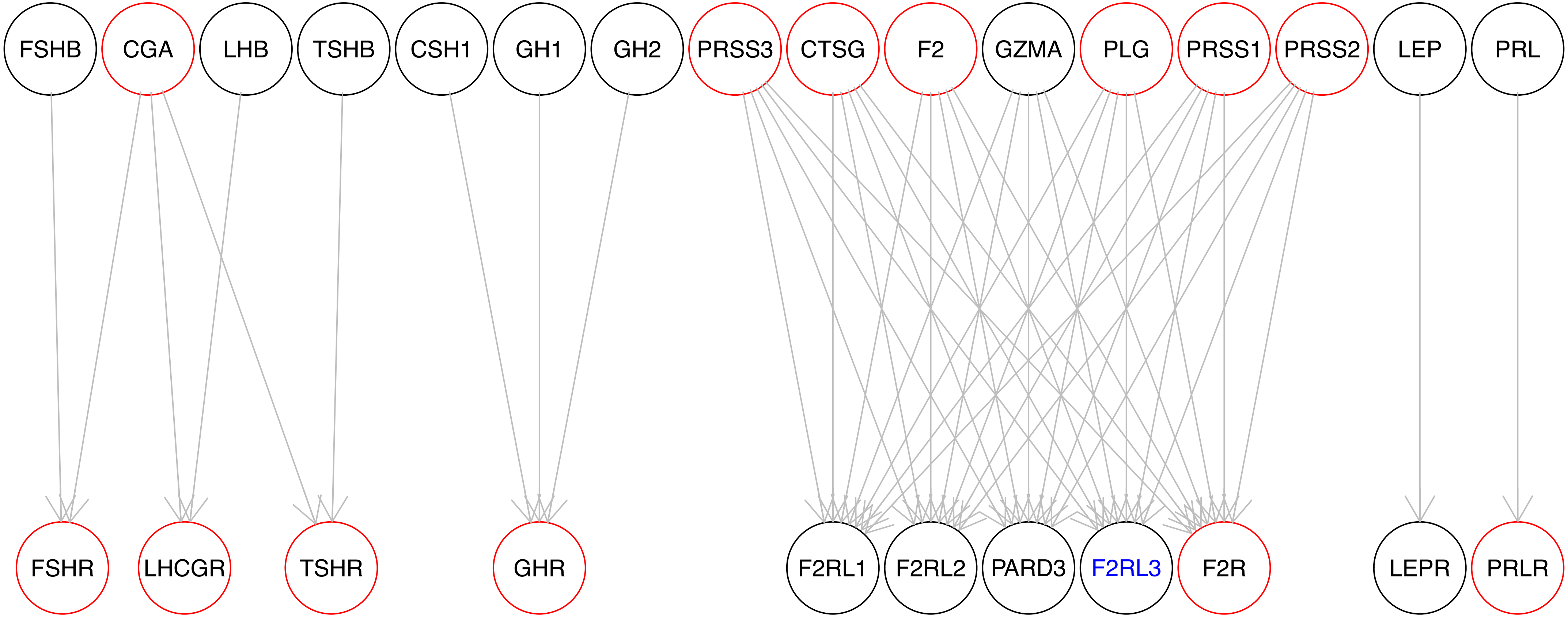

KEGG pathway = Neuroactive ligand−receptor interaction :   tumour = HS839T_SKIN :   Yellow Fill = gene variant, Blue Text = expression−survival association, Red Boarder = drug

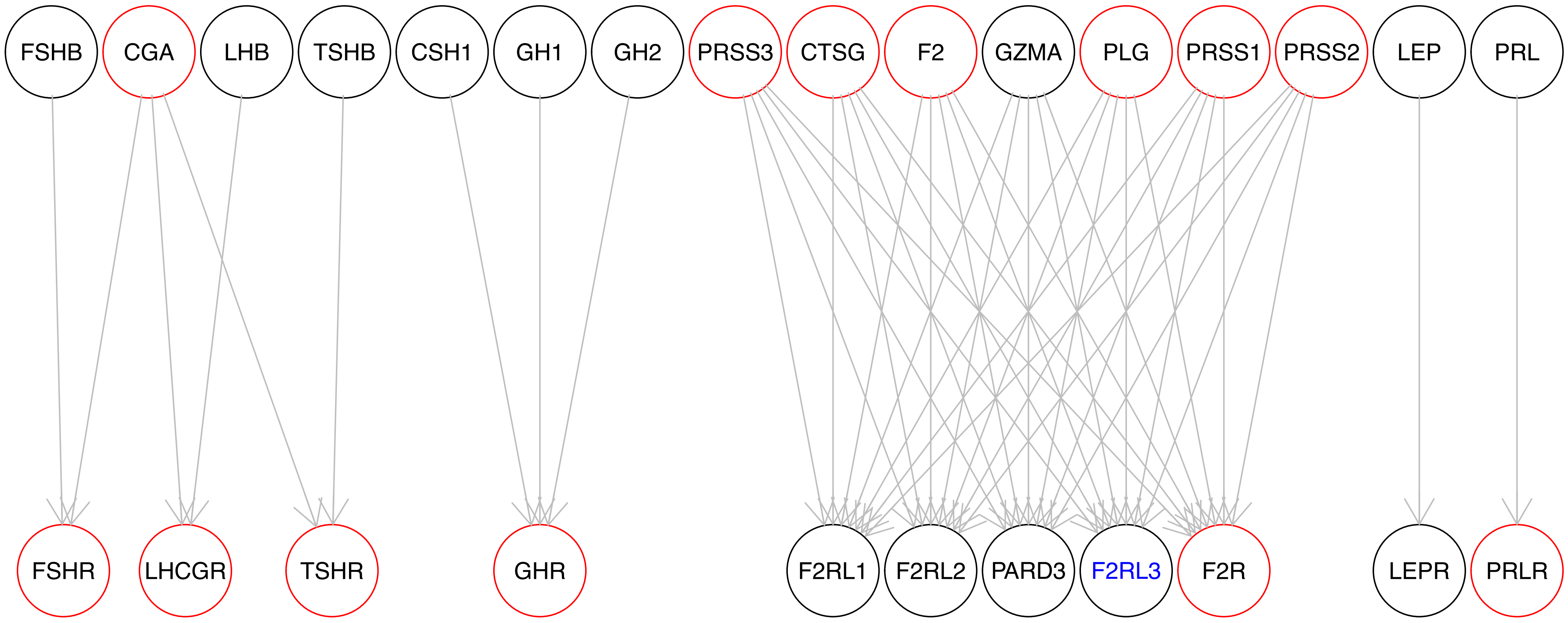

KEGG pathway = Neuroactive ligand−receptor interaction : tumour = HS852T_SKIN : Yellow Fill = gene variant, Blue Text = expression−survival association, Red Boarder = drug

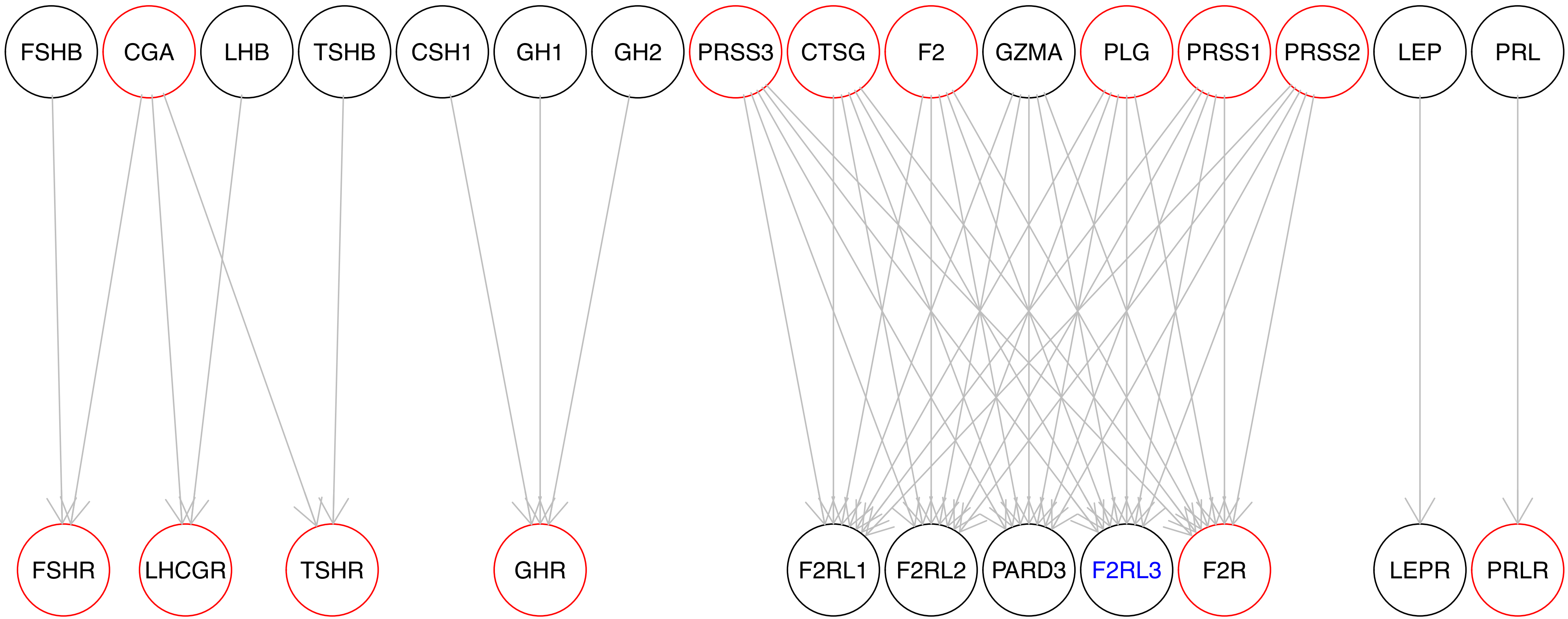

KEGG pathway = Neuroactive ligand−receptor interaction :   tumour = HS895T_SKIN :   Yellow Fill = gene variant, Blue Text = expression−survival association, Red Boarder = drug

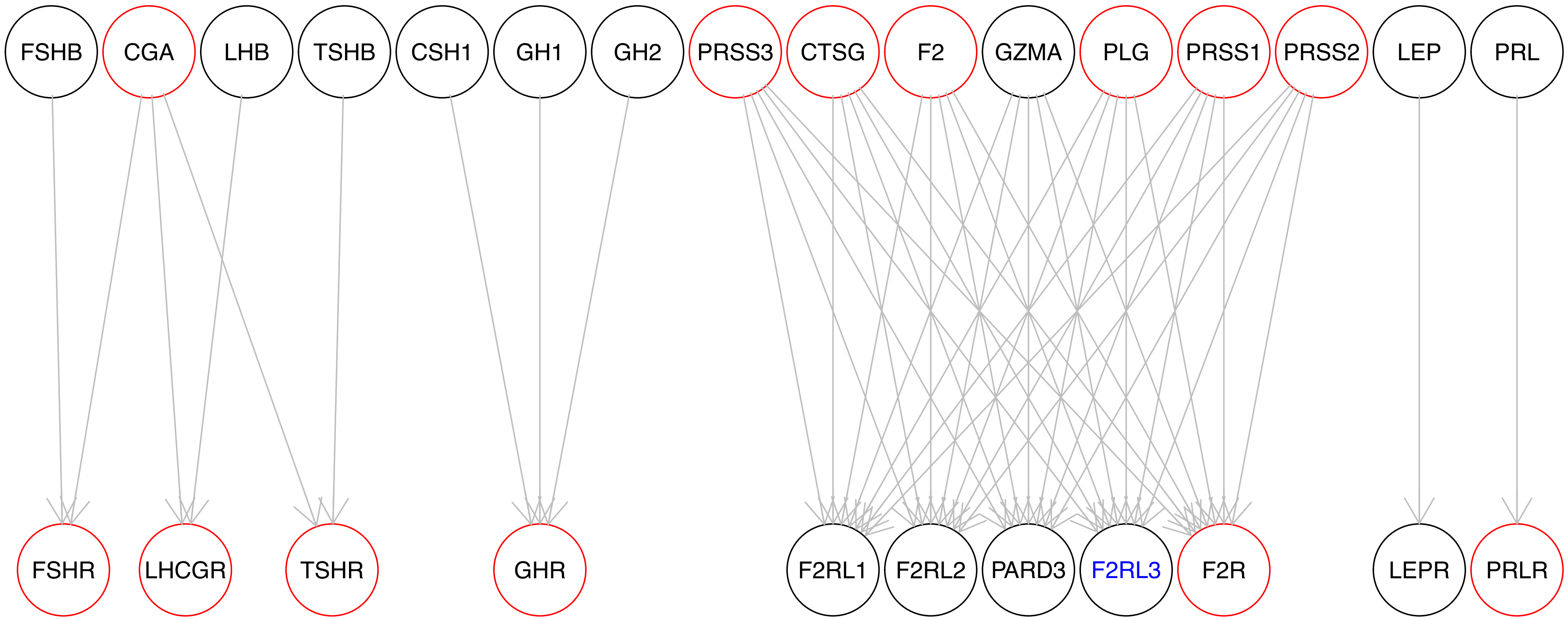

KEGG pathway = Neuroactive ligand−receptor interaction : tumour = HS934T_SKIN : Yellow Fill = gene variant, Blue Text = expression−survival association, Red Boarder = drug

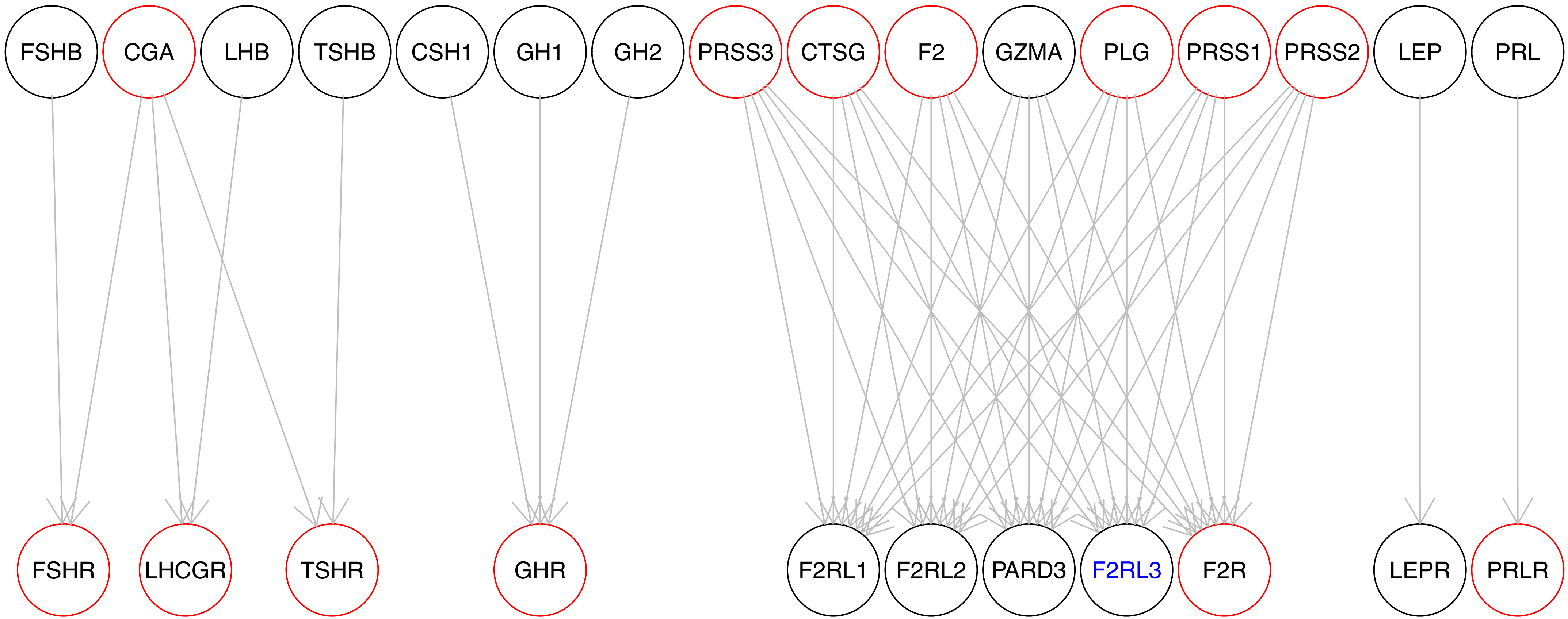

KEGG pathway = Neuroactive ligand−receptor interaction :   tumour = HS936T_SKIN :   Yellow Fill = gene variant, Blue Text = expression−survival association, Red Boarder = drug

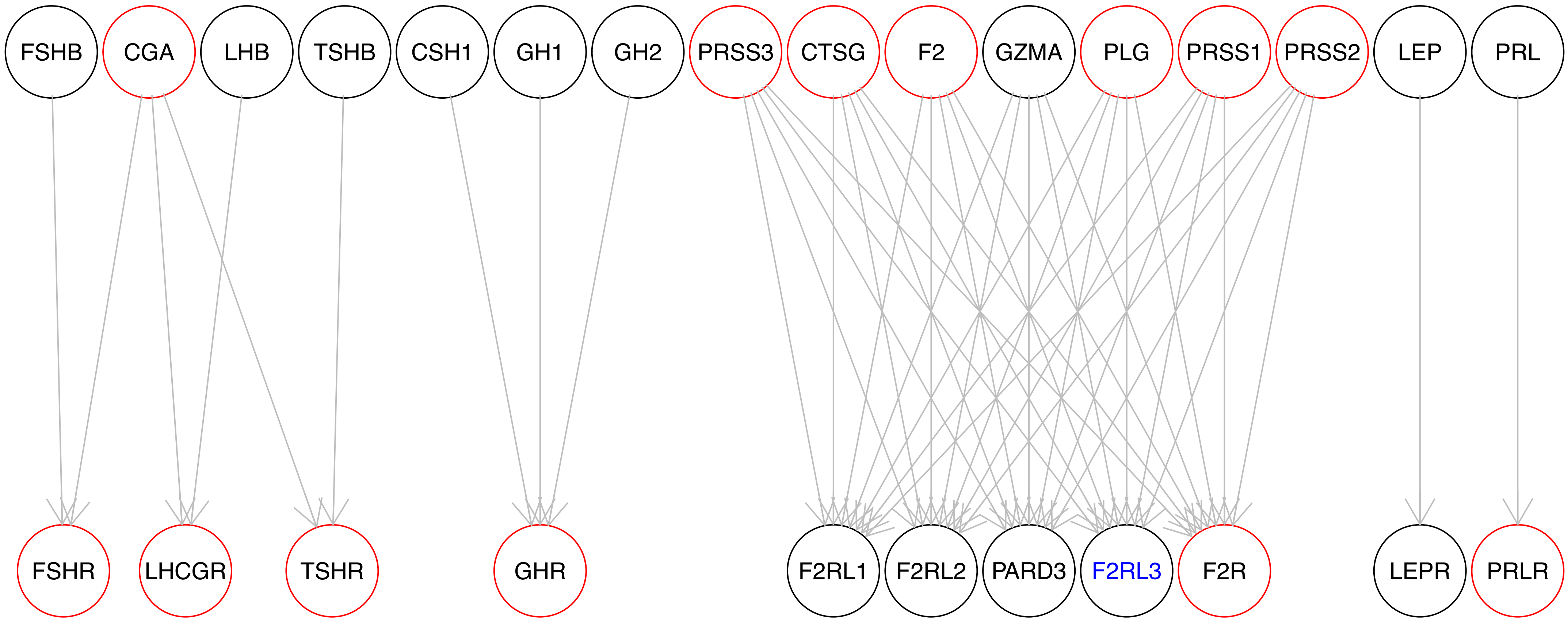

KEGG pathway = Neuroactive ligand−receptor interaction :   tumour = HS940T_SKIN :   Yellow Fill = gene variant, Blue Text = expression−survival association, Red Boarder = drug

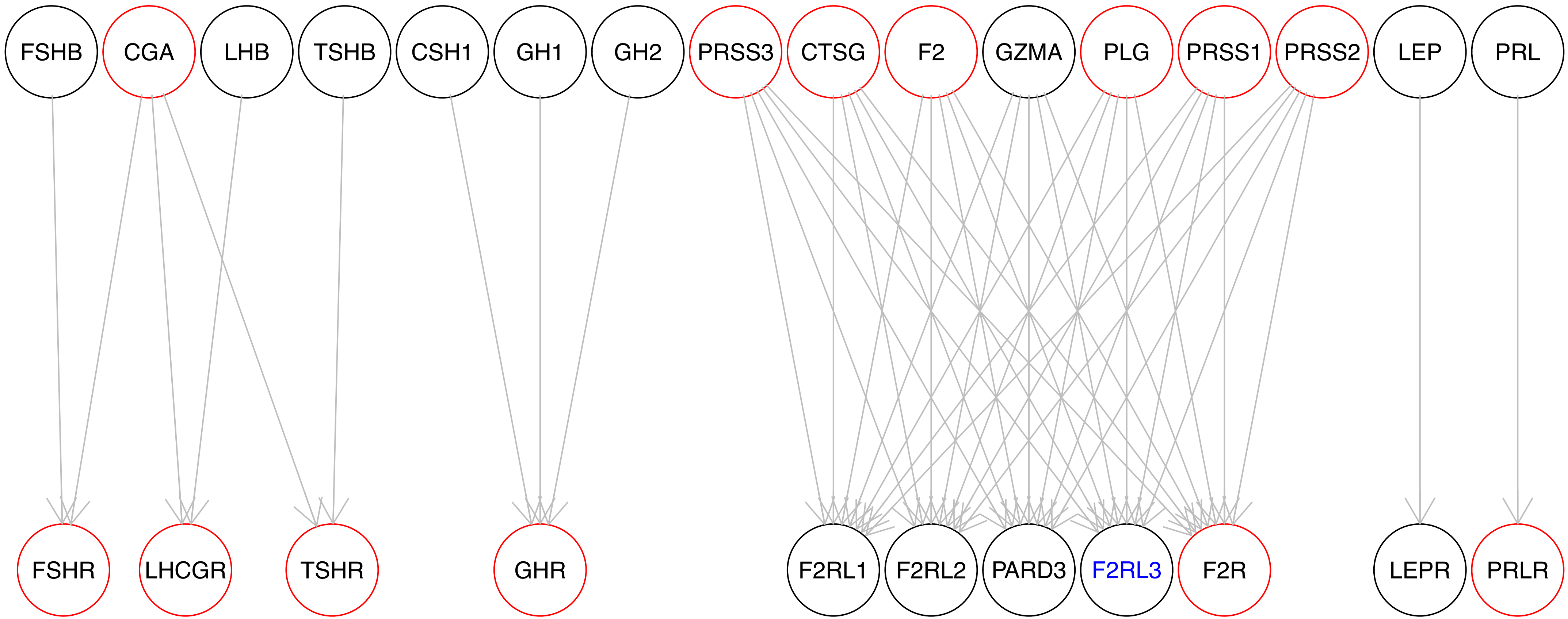

KEGG pathway = Neuroactive ligand−receptor interaction :   tumour = HS944T_SKIN :   Yellow Fill = gene variant, Blue Text = expression−survival association, Red Boarder = drug

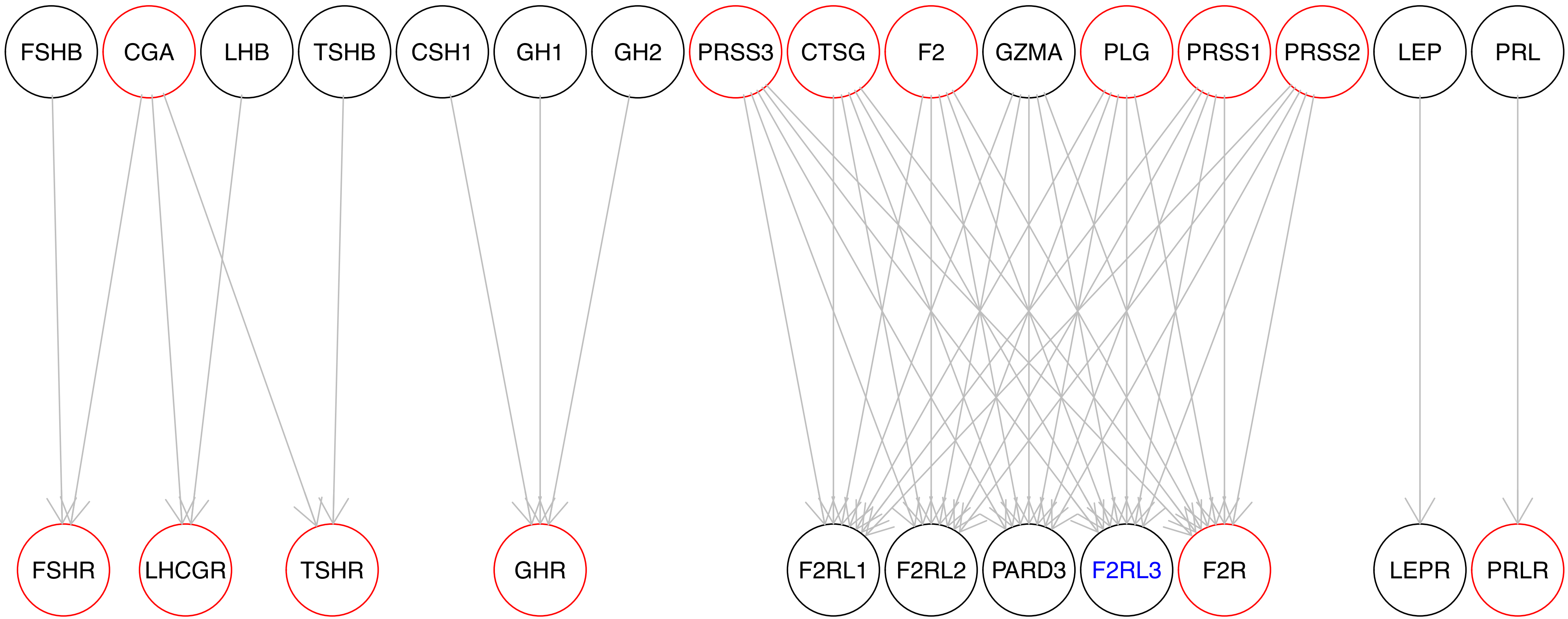

KEGG pathway = Neuroactive ligand−receptor interaction : tumour = HT144_SKIN : Yellow Fill = gene variant, Blue Text = expression−survival association, Red Boarder = drug

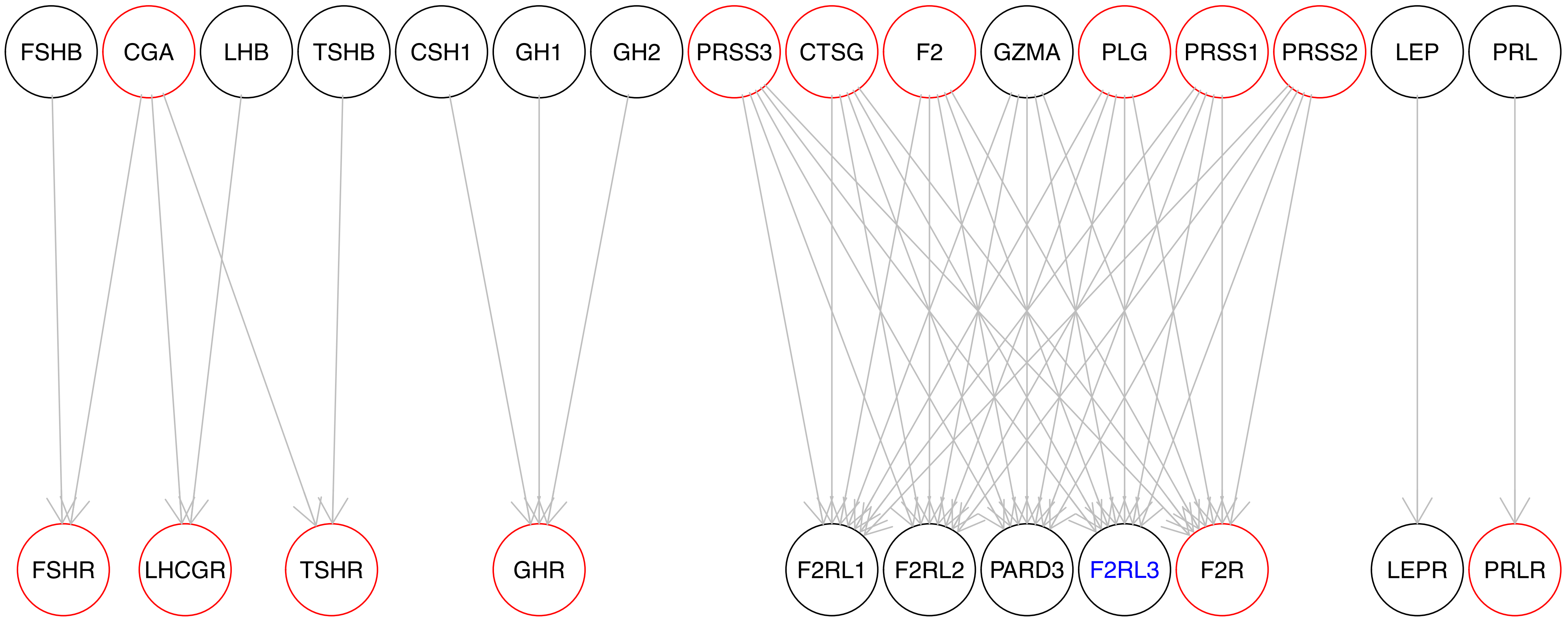

KEGG pathway = Neuroactive ligand−receptor interaction : tumour = IGR37_SKIN : Yellow Fill = gene variant, Blue Text = expression−survival association, Red Boarder = drug

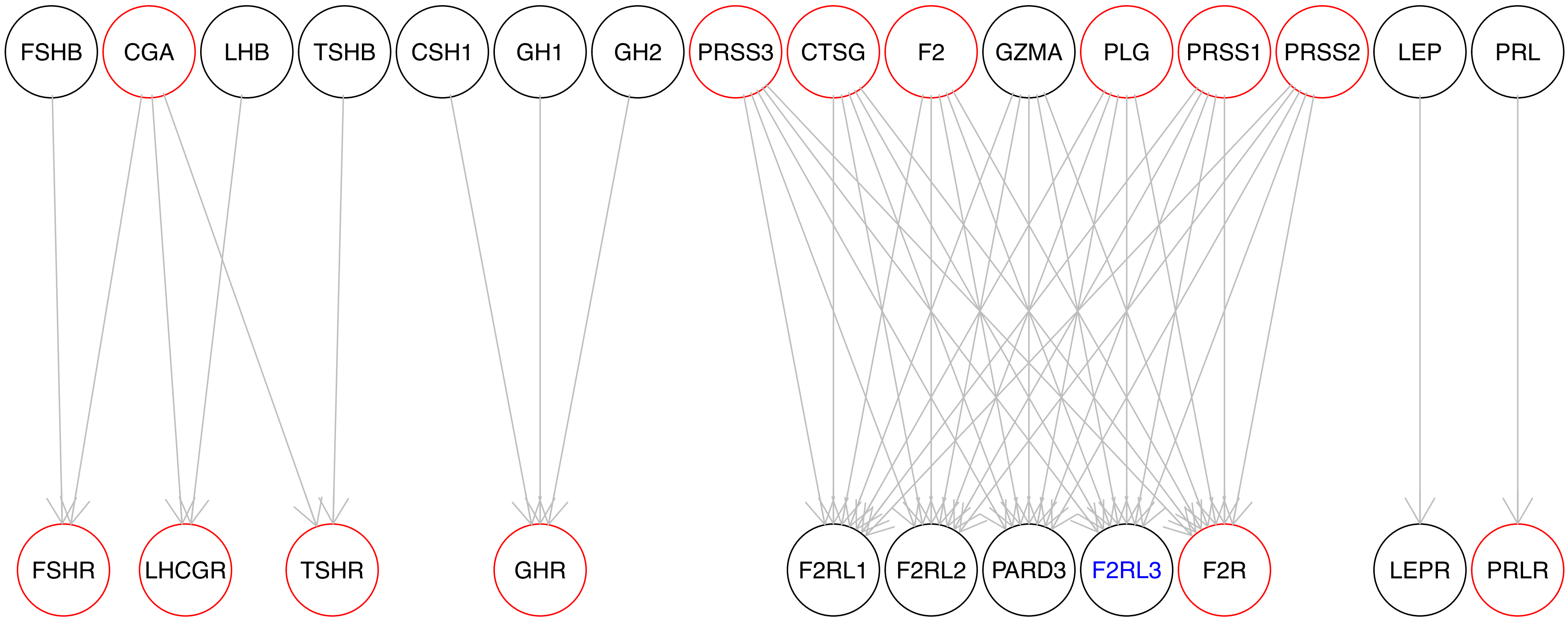

KEGG pathway = Neuroactive ligand−receptor interaction : tumour = IGR39_SKIN : Yellow Fill = gene variant, Blue Text = expression−survival association, Red Boarder = drug

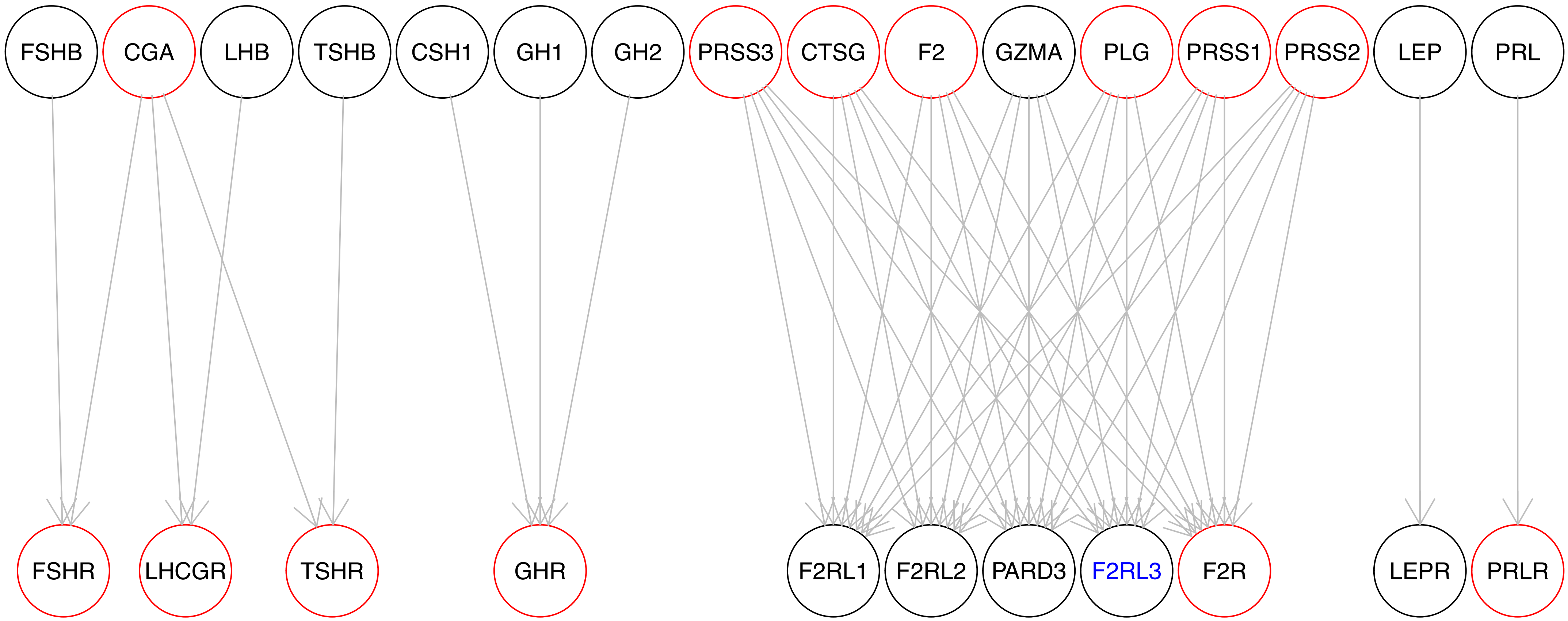

KEGG pathway = Neuroactive ligand−receptor interaction :   tumour = IPC298_SKIN :   Yellow Fill = gene variant, Blue Text = expression−survival association, Red Boarder = drug

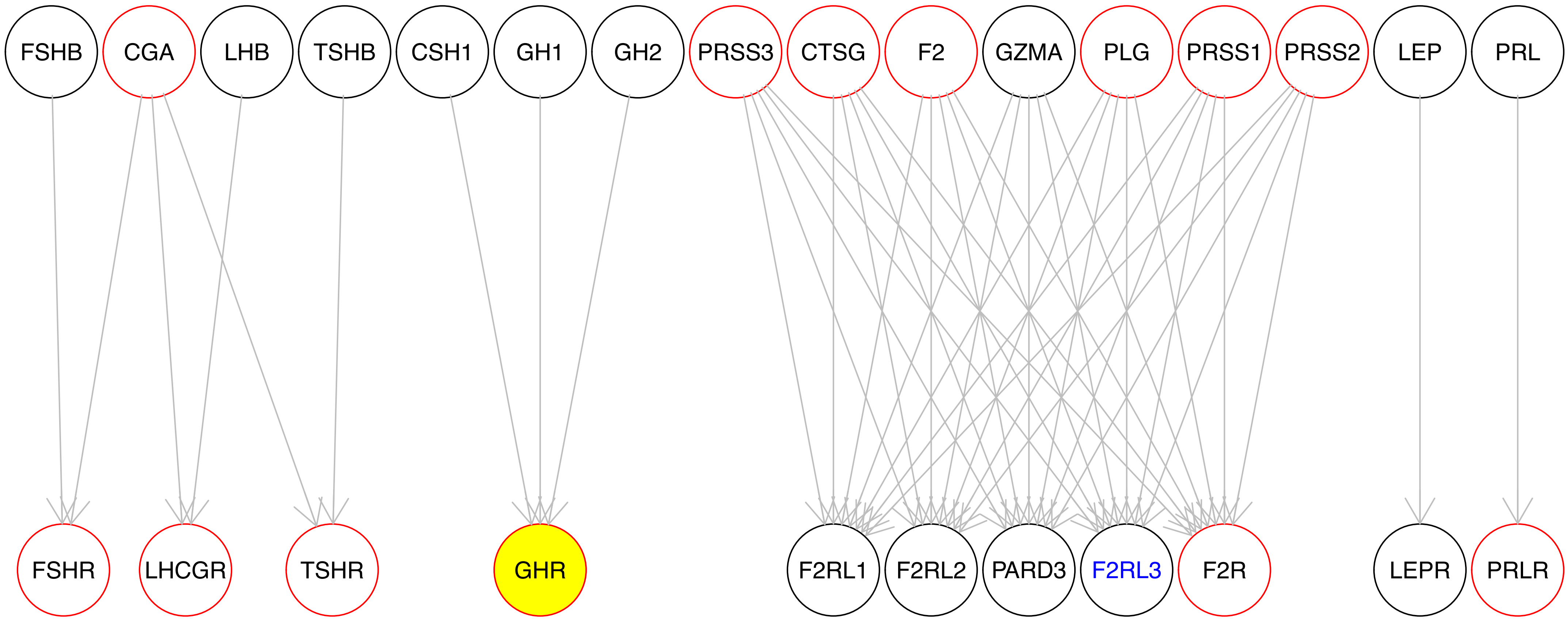

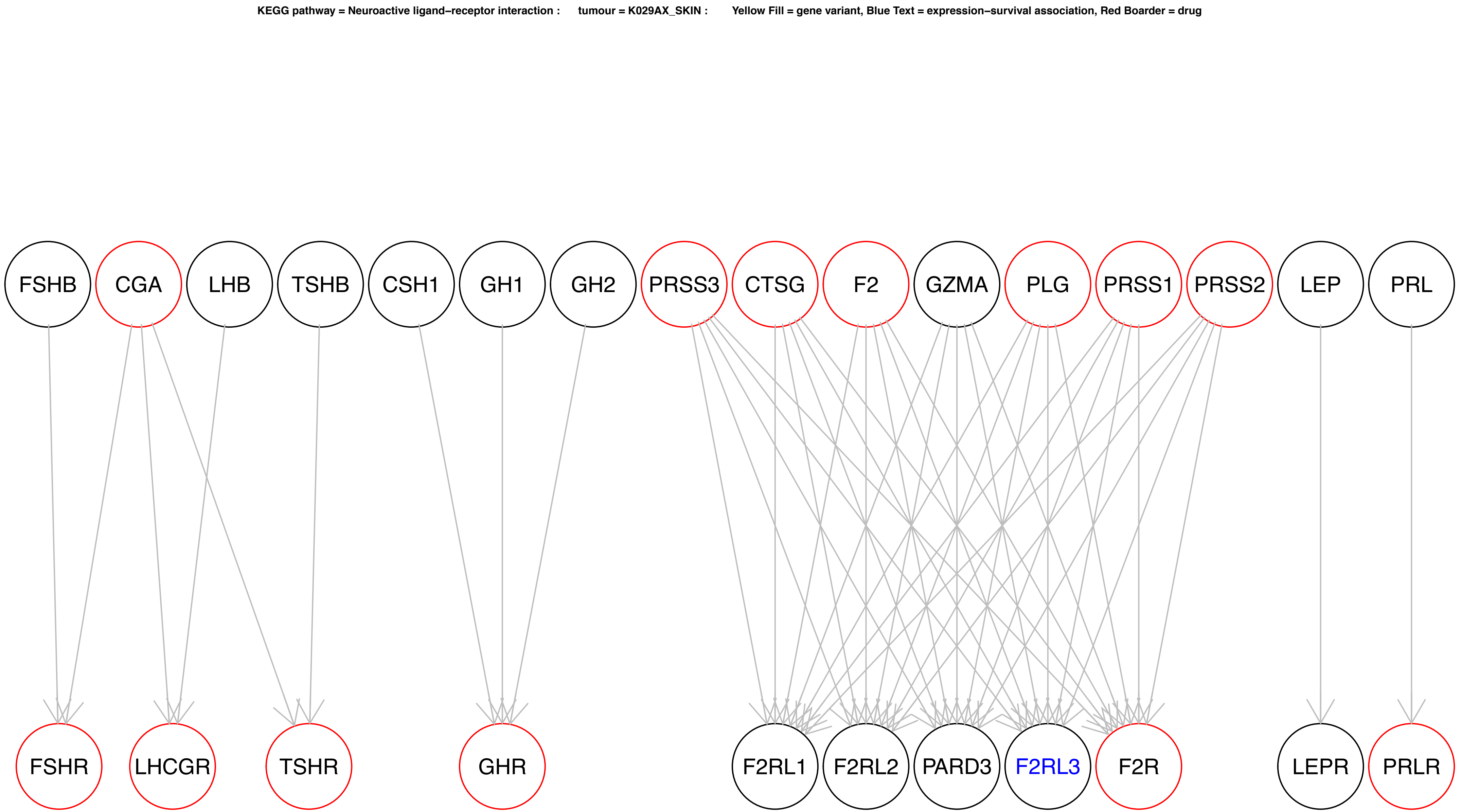
KEGG pathway = Neuroactive ligand−receptor interaction :   tumour = K029AX_SKIN :   Yellow Fill = gene variant, Blue Text = expression−survival association, Red Boarder = drug
FSHB
CGA
LHB
TSHB
CSH1
GH1
GH2
PRSS3
CTSG
F2
GZMA
PLG
PRSS1
PRSS2
LEP
PRL
FSHR
LHCGR
TSHR
GHR
F2RL1
F2RL2
PARD3
F2RL3
F2R
LEPR
PRLR

KEGG pathway = Neuroactive ligand−receptor interaction :   tumour = LOXIMVI_SKIN :   Yellow Fill = gene variant, Blue Text = expression−survival association, Red Boarder = drug

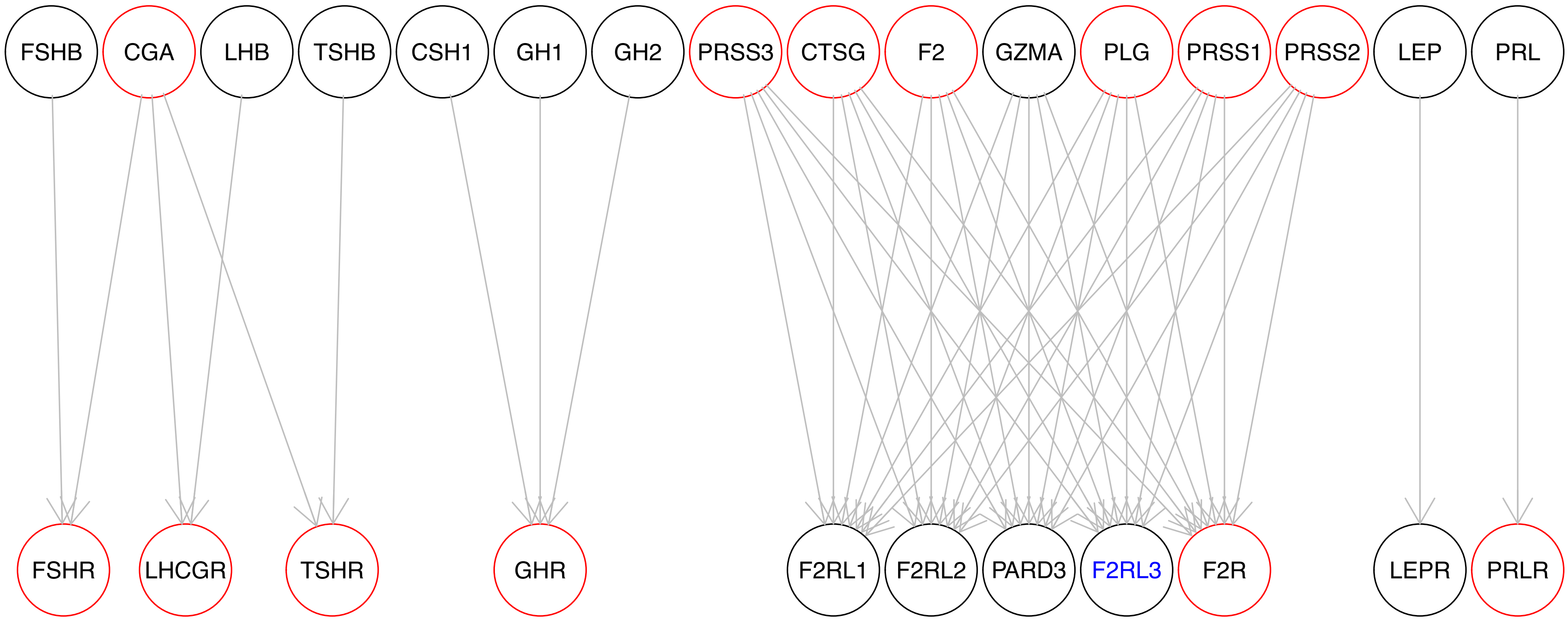

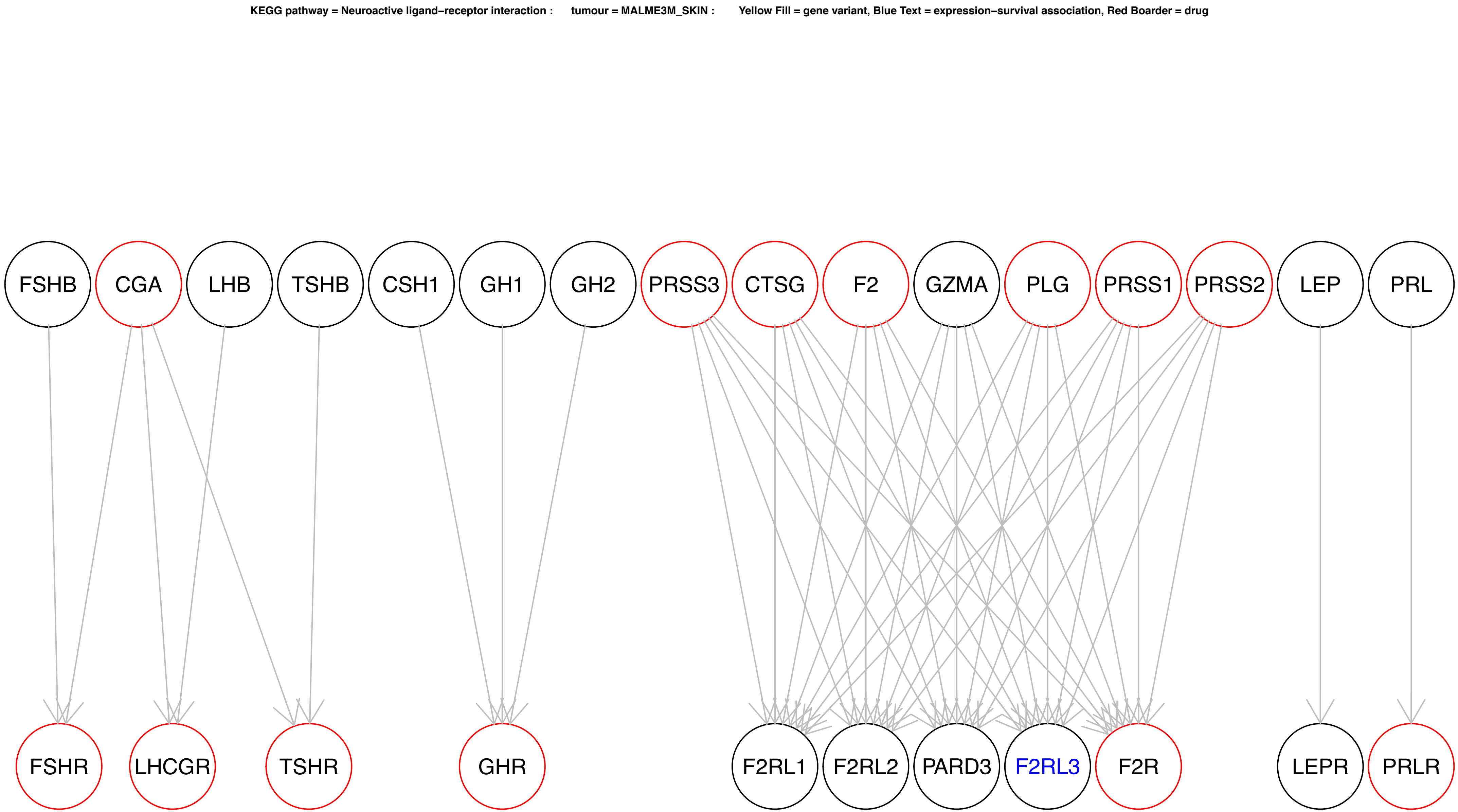
KEGG pathway = Neuroactive ligand−receptor interaction :   tumour = MALME3M_SKIN :   Yellow Fill = gene variant, Blue Text = expression−survival association, Red Boarder = drug
FSHB
CGA
LHB
TSHB
CSH1
GH1
GH2
PRSS3
CTSG
F2
GZMA
PLG
PRSS1
PRSS2
LEP
PRL
FSHR
LHCGR
TSHR
GHR
F2RL1
F2RL2
PARD3
F2RL3
F2R
LEPR
PRLR

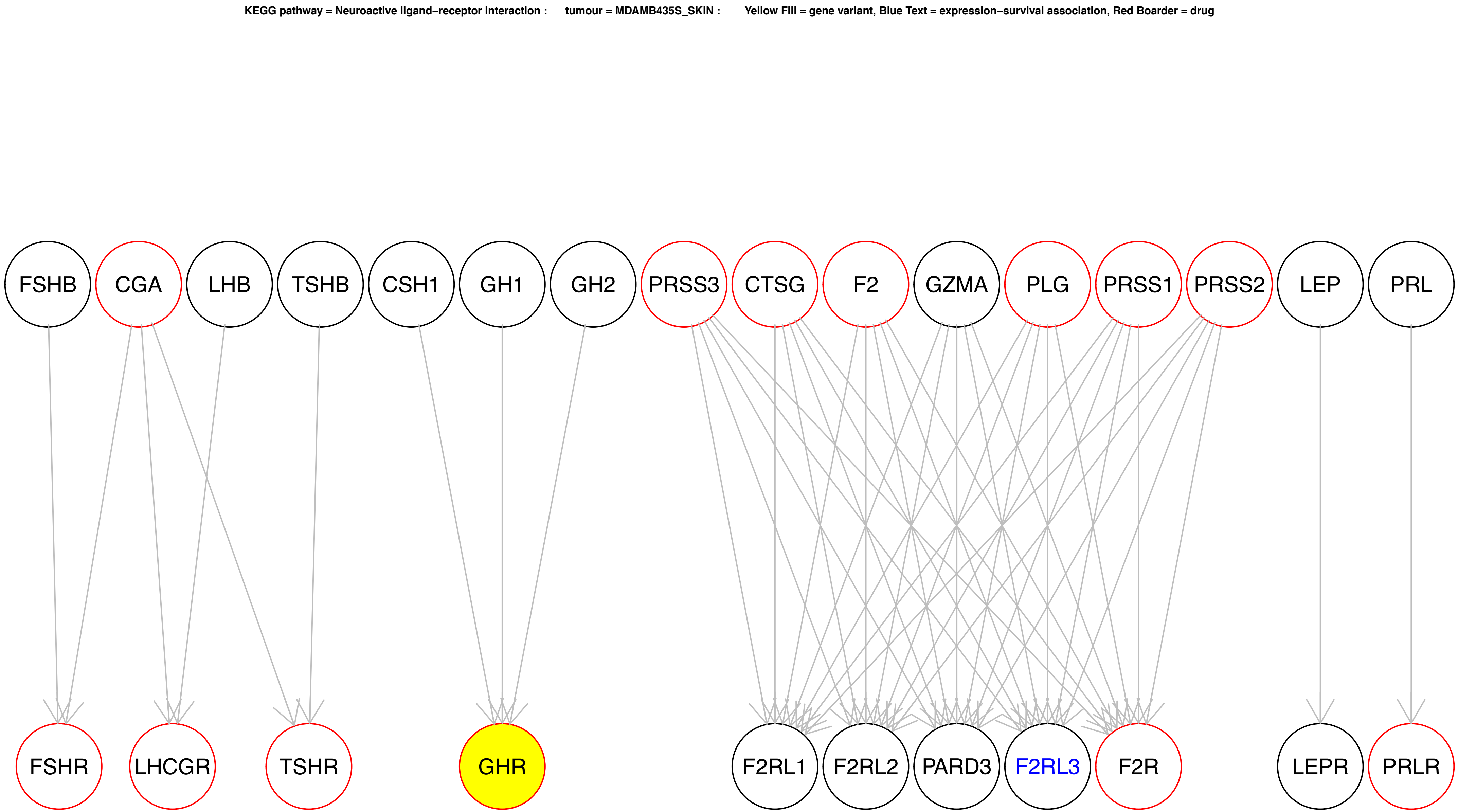
KEGG pathway = Neuroactive ligand−receptor interaction : tumour = MDAMB435S_SKIN : Yellow Fill = gene variant, Blue Text = expression−survival association, Red Boarder = drug
FSHB
CGA
LHB
TSHB
CSH1
GH1
GH2
PRSS3
CTSG
F2
GZMA
PLG
PRSS1
PRSS2
LEP
PRL
FSHR
LHCGR
TSHR
GHR
F2RL1
F2RL2
PARD3
F2RL3
F2R
LEPR
PRLR

KEGG pathway = Neuroactive ligand−receptor interaction :   tumour = MELHO_SKIN :   Yellow Fill = gene variant, Blue Text = expression−survival association, Red Boarder = drug

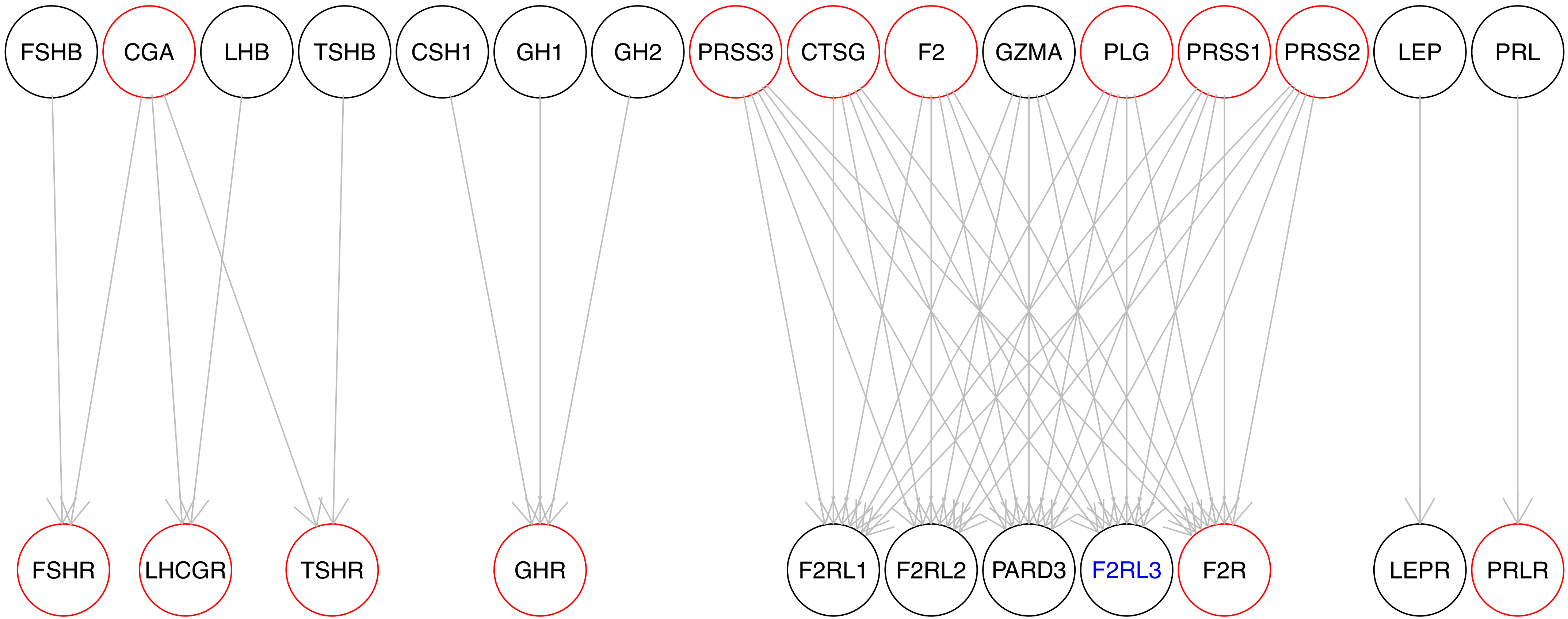

KEGG pathway = Neuroactive ligand−receptor interaction :   tumour = MELJUSO_SKIN :   Yellow Fill = gene variant, Blue Text = expression−survival association, Red Boarder = drug

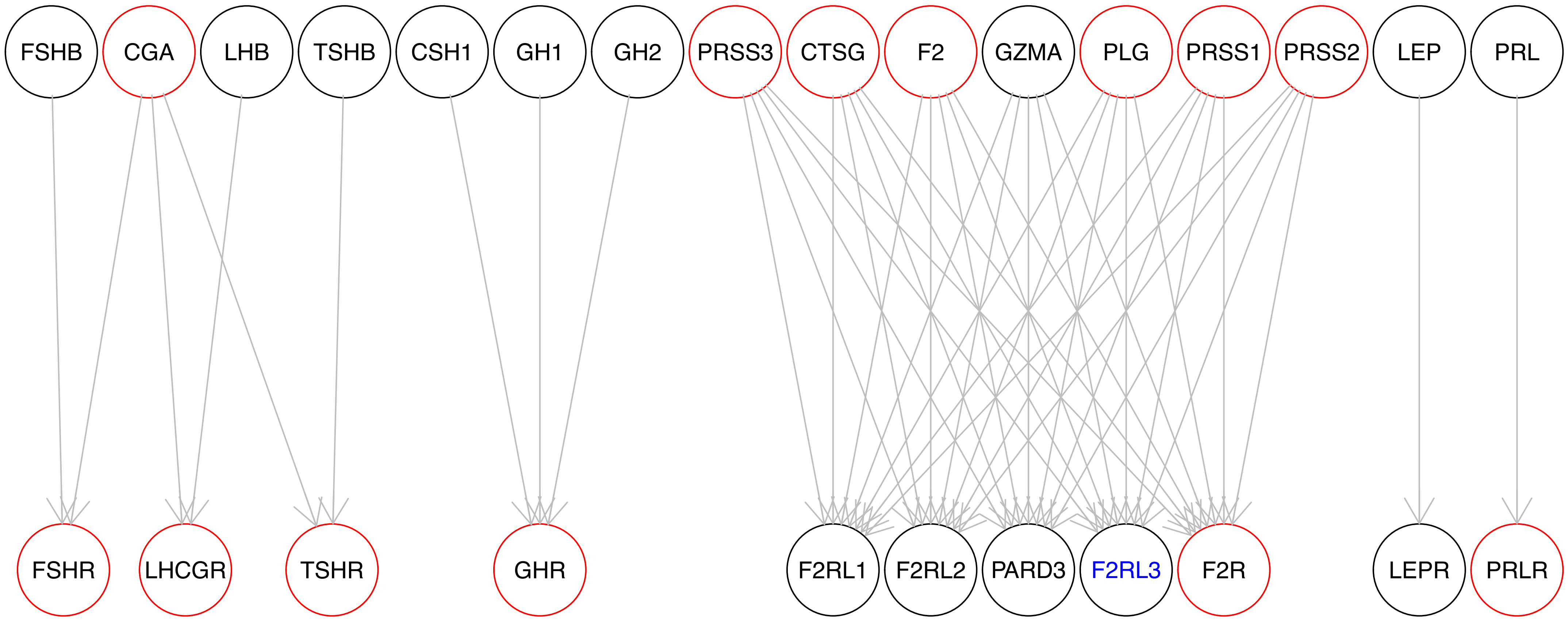

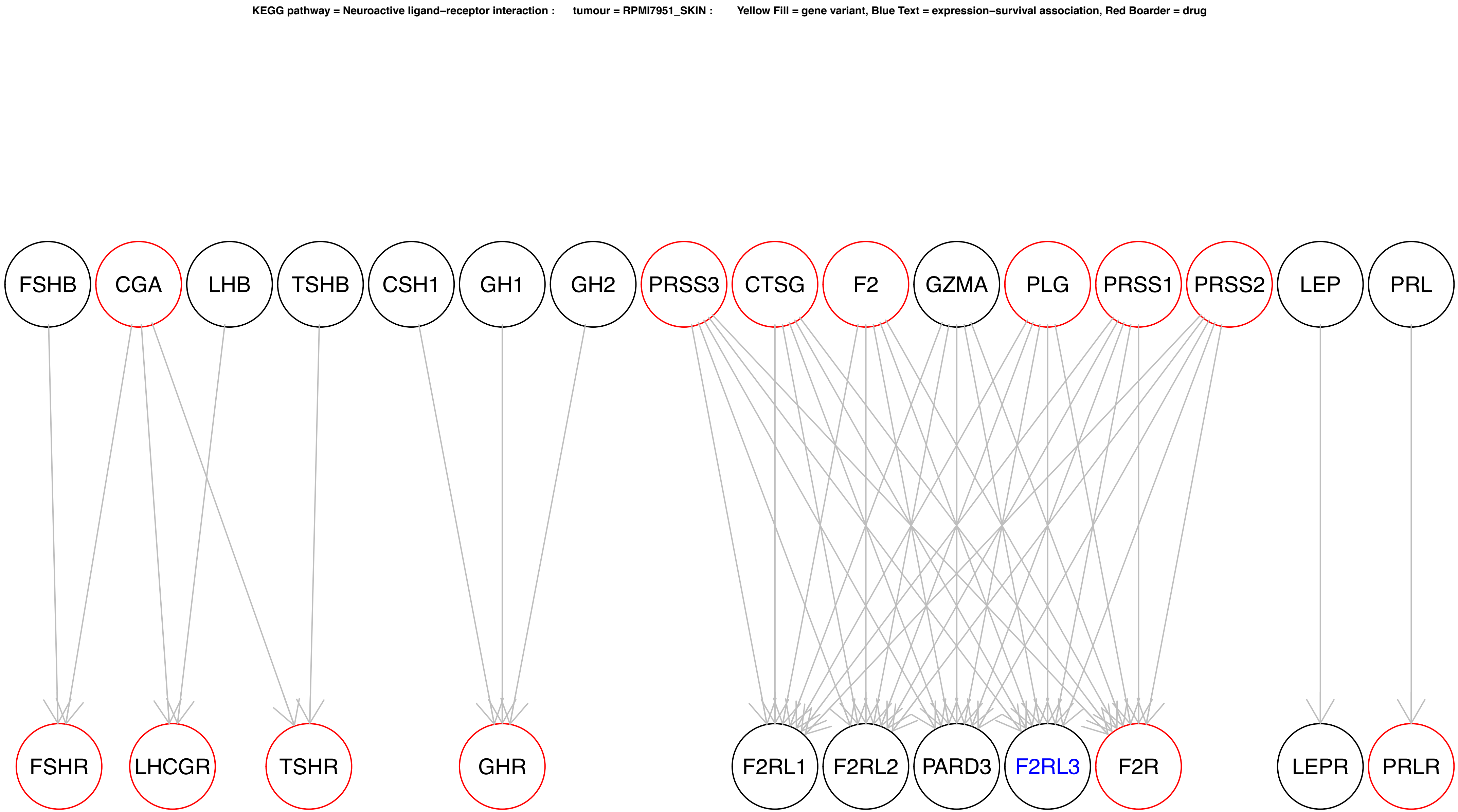
KEGG pathway = Neuroactive ligand–receptor interaction : tumour = RPMI7951_SKIN : Yellow Fill = gene variant, Blue Text = expression–survival association, Red Boarder = drug
FSHB
CGA
LHB
TSHB
CSH1
GH1
GH2
PRSS3
CTSG
F2
GZMA
PLG
PRSS1
PRSS2
LEP
PRL
FSHR
LHCGR
TSHR
GHR
F2RL1
F2RL2
PARD3
F2RL3
F2R
LEPR
PRLR

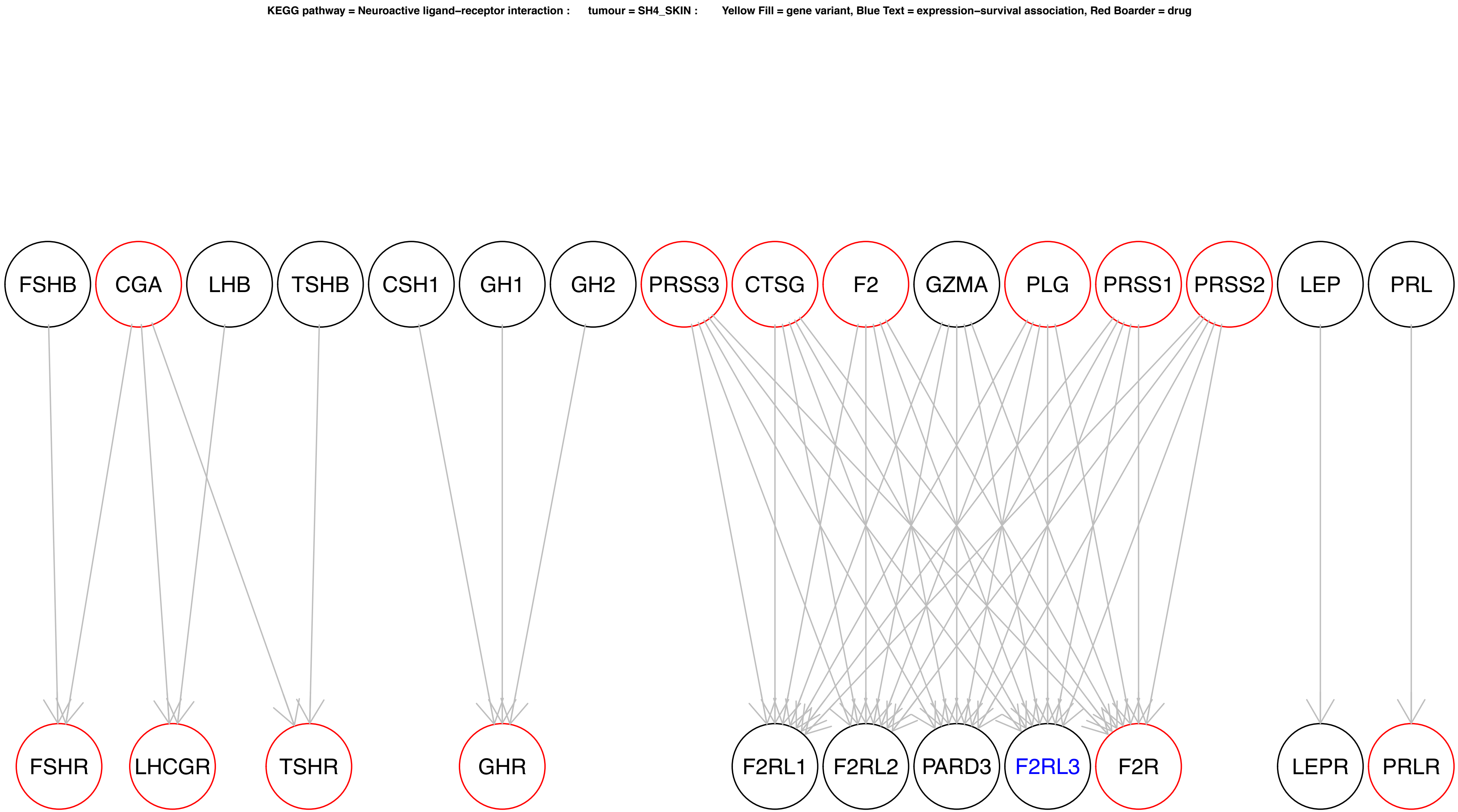

KEGG pathway = Neuroactive ligand−receptor interaction : tumour = SH4_SKIN : Yellow Fill = gene variant, Blue Text = expression−survival association, Red Boarder = drug
FSHB
CGA
LHB
TSHB
CSH1
GH1
GH2
PRSS3
CTSG
F2
GZMA
PLG
PRSS1
PRSS2
LEP
PRL
FSHR
LHCGR
TSHR
GHR
F2RL1
F2RL2
PARD3
F2RL3
F2R
LEPR
PRLR

KEGG pathway = Neuroactive ligand−receptor interaction : tumour = SKMEL1_SKIN : Yellow Fill = gene variant, Blue Text = expression−survival association, Red Boarder = drug

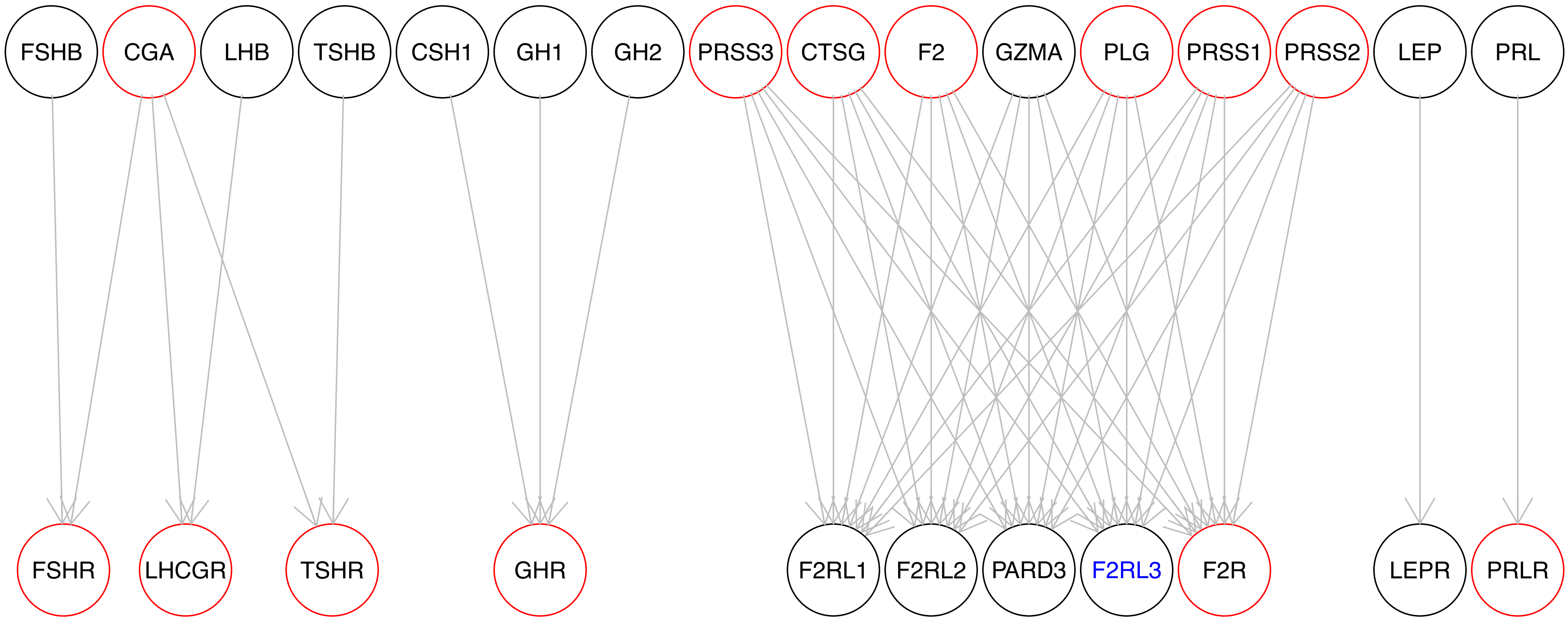

KEGG pathway = Neuroactive ligand−receptor interaction : tumour = SKMEL2_SKIN : Yellow Fill = gene variant, Blue Text = expression−survival association, Red Boarder = drug

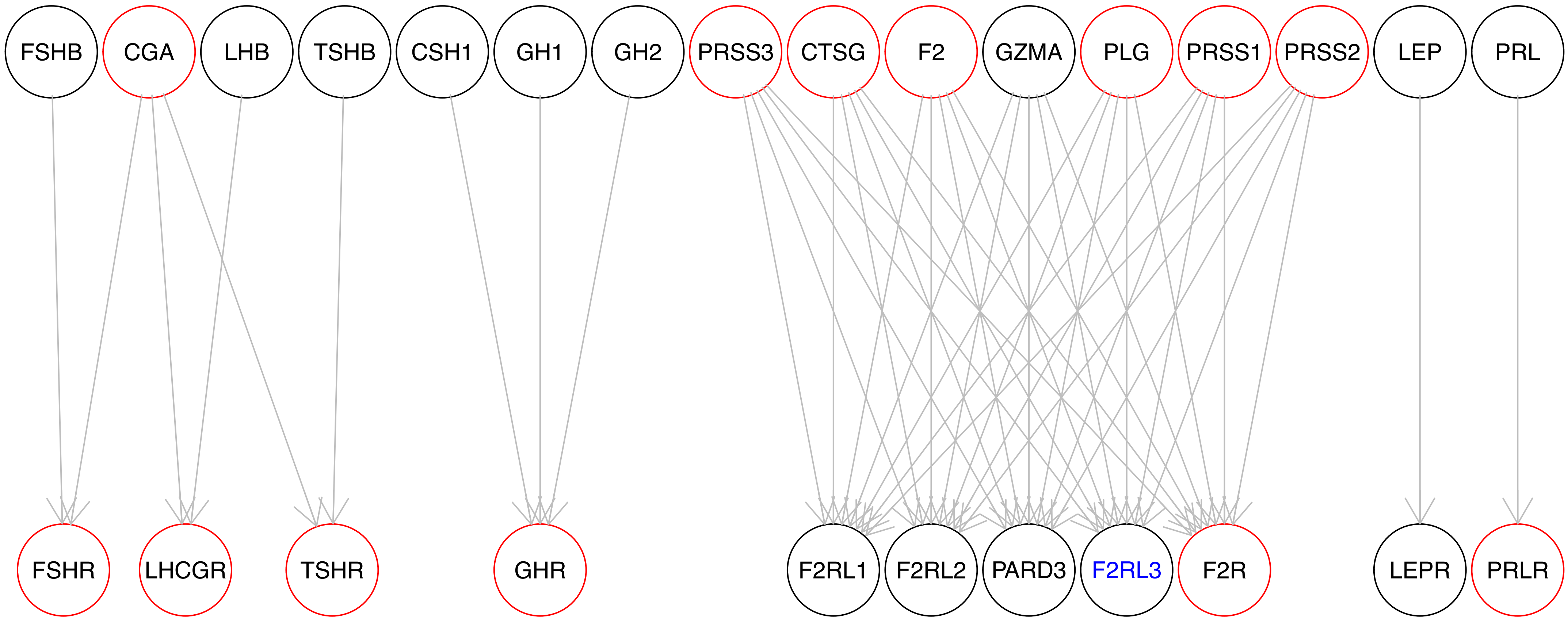

KEGG pathway = Neuroactive ligand−receptor interaction : tumour = SKMEL24_SKIN : Yellow Fill = gene variant, Blue Text = expression−survival association, Red Boarder = drug

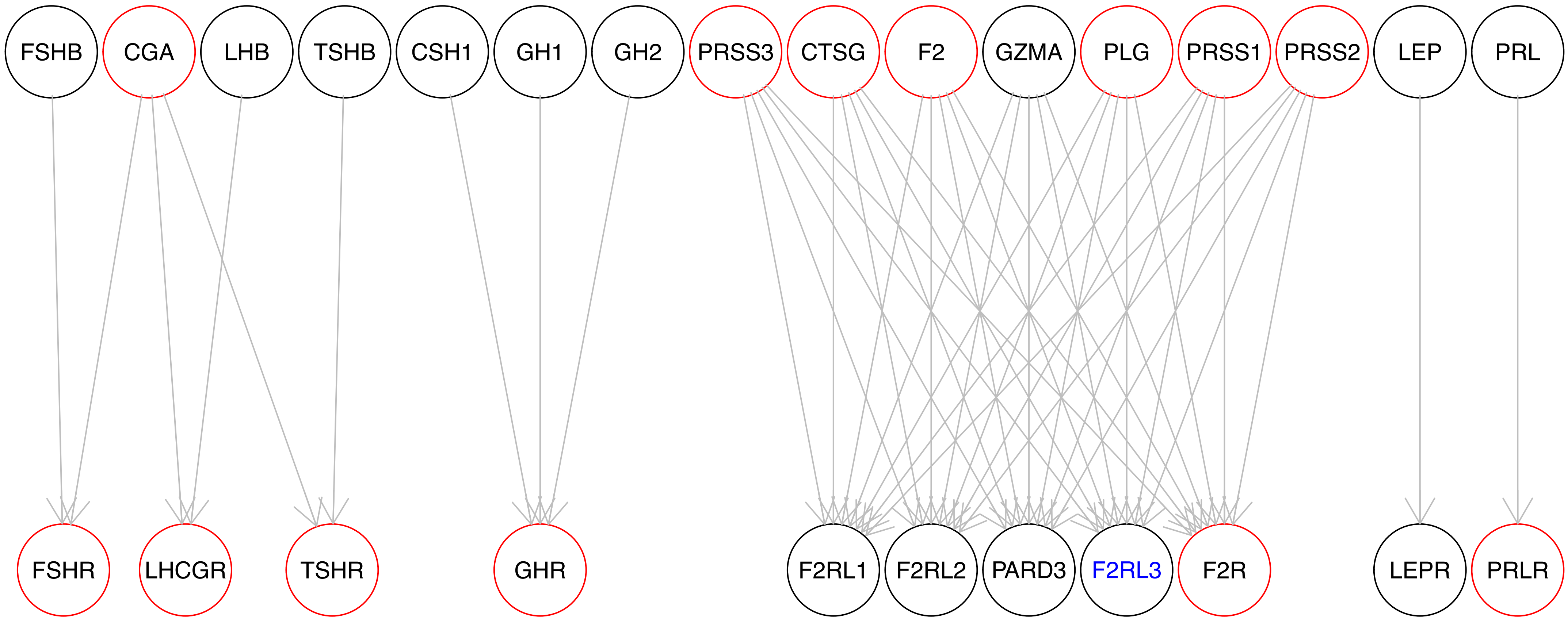

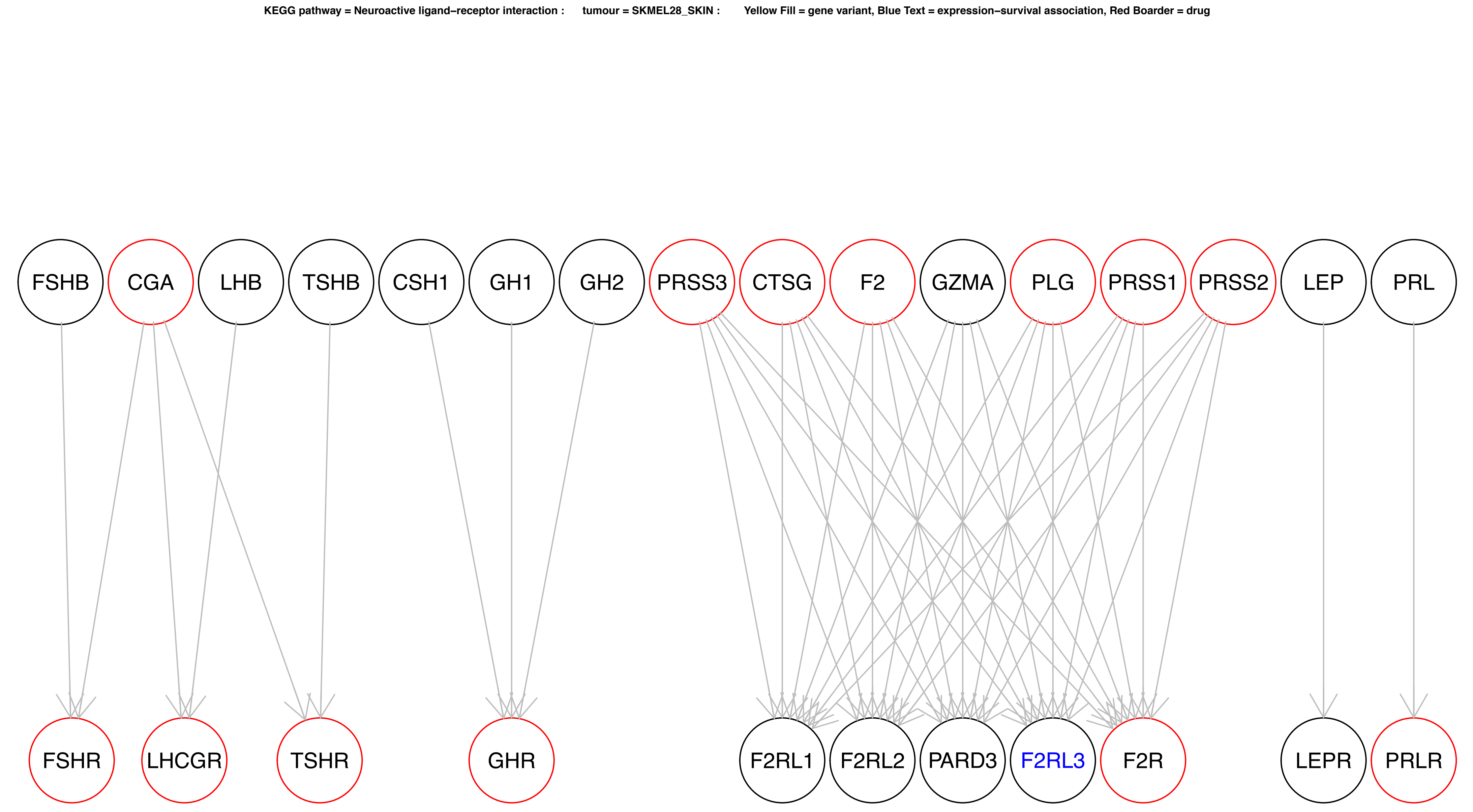
KEGG pathway = Neuroactive ligand−receptor interaction : tumour = SKMEL28_SKIN : Yellow Fill = gene variant, Blue Text = expression−survival association, Red Boarder = drug
FSHB
CGA
LHB
TSHB
CSH1
GH1
GH2
PRSS3
CTSG
F2
GZMA
PLG
PRSS1
PRSS2
LEP
PRL
FSHR
LHCGR
TSHR
GHR
F2RL1
F2RL2
PARD3
F2RL3
F2R
LEPR
PRLR

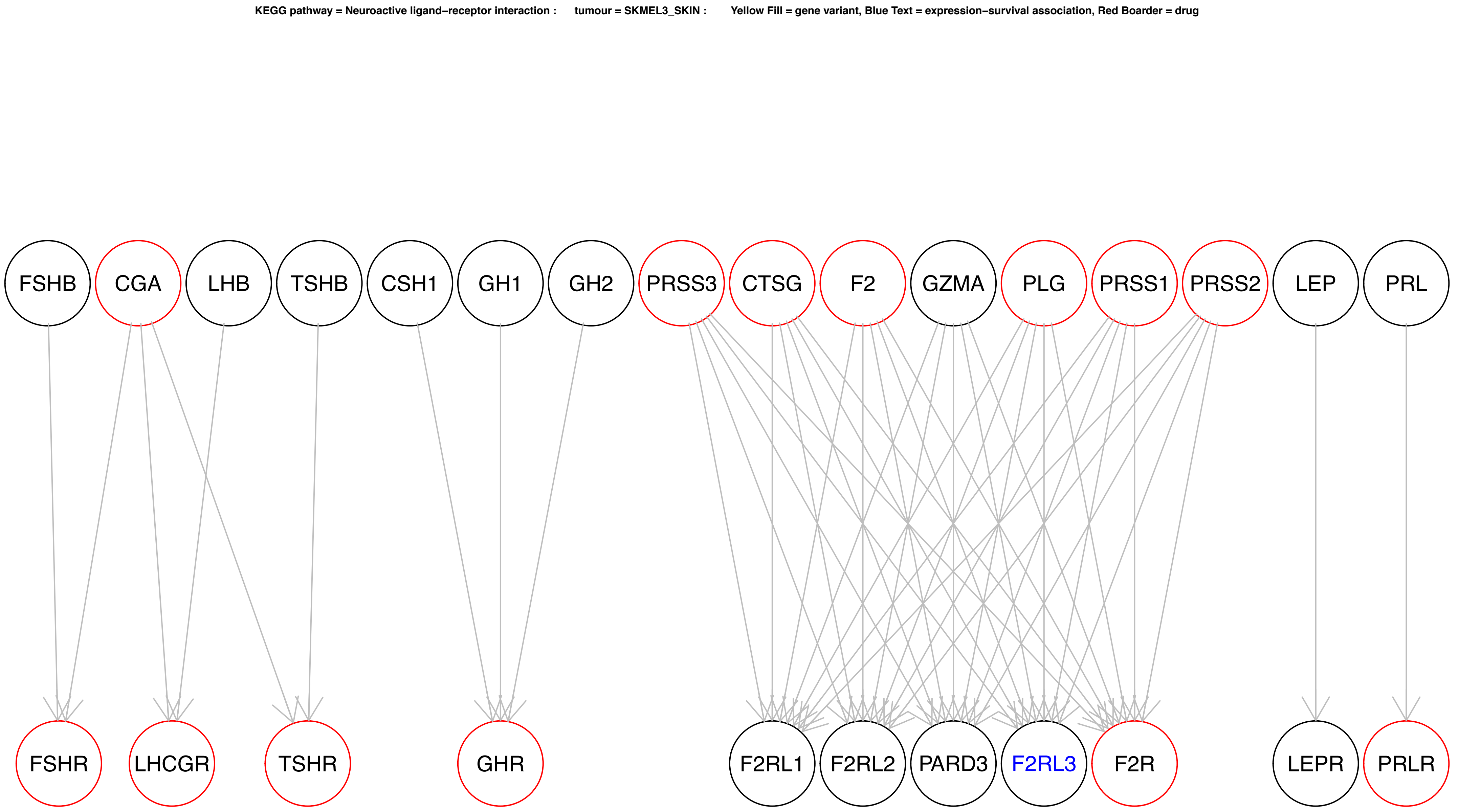
KEGG pathway = Neuroactive ligand−receptor interaction : tumour = SKMEL3_SKIN : Yellow Fill = gene variant, Blue Text = expression−survival association, Red Boarder = drug
FSHB
CGA
LHB
TSHB
CSH1
GH1
GH2
PRSS3
CTSG
F2
GZMA
PLG
PRSS1
PRSS2
LEP
PRL
FSHR
LHCGR
TSHR
GHR
F2RL1
F2RL2
PARD3
F2RL3
F2R
LEPR
PRLR

KEGG pathway = Neuroactive ligand−receptor interaction : tumour = SKMEL30_SKIN : Yellow Fill = gene variant, Blue Text = expression−survival association, Red Boarder = drug

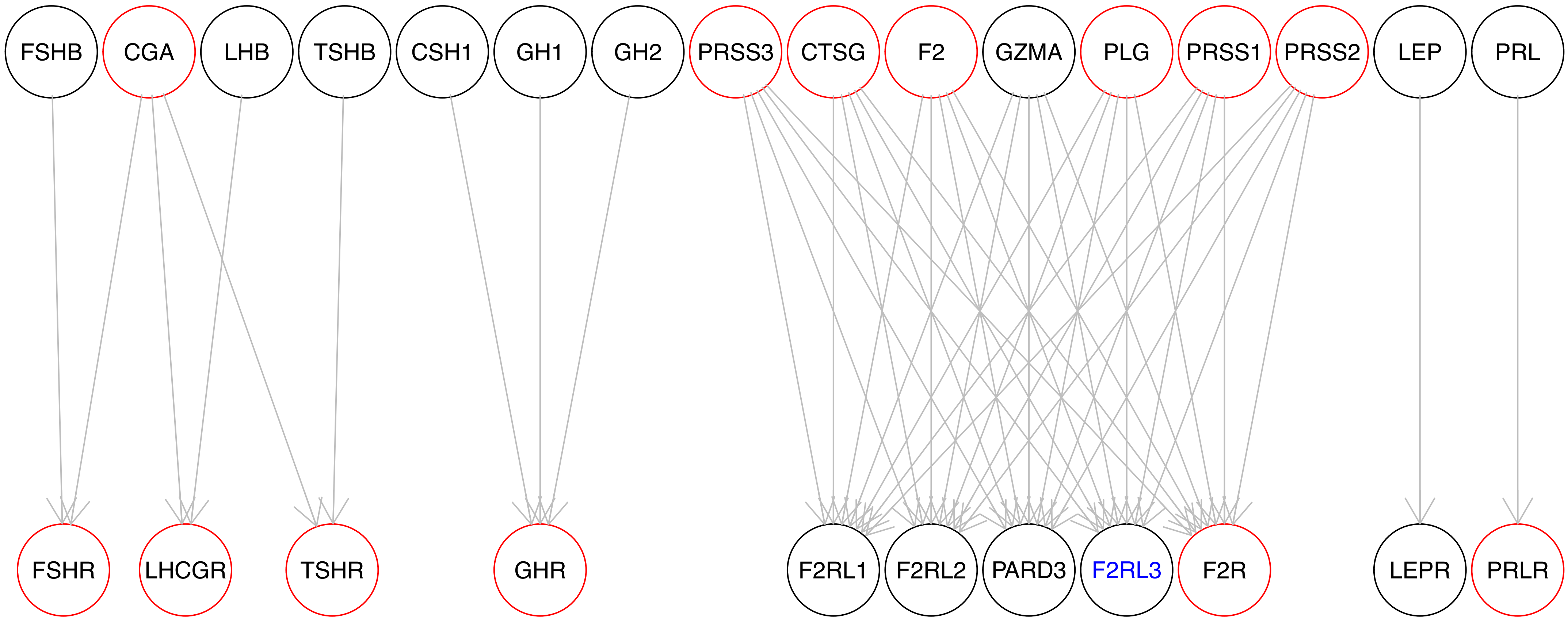

KEGG pathway = Neuroactive ligand–receptor interaction : tumour = SKMEL5_SKIN : Yellow Fill = gene variant, Blue Text = expression–survival association, Red Boarder = drug

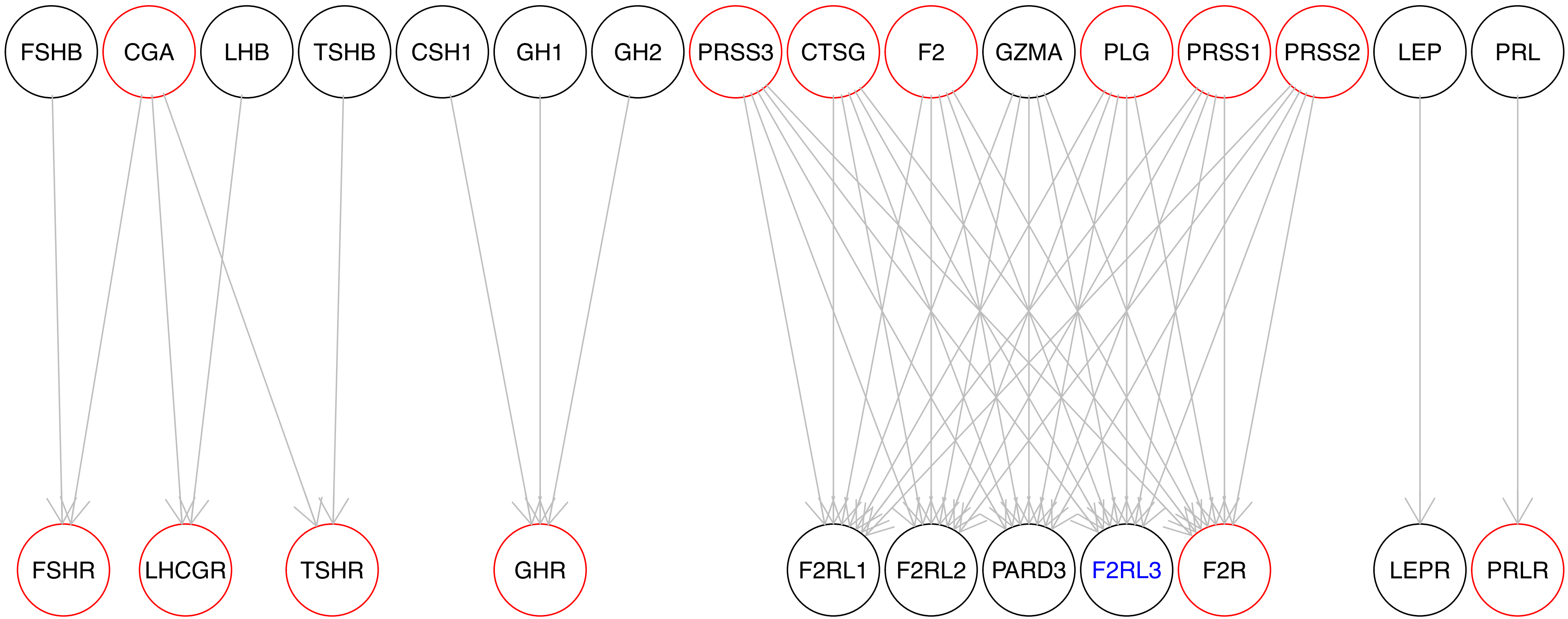

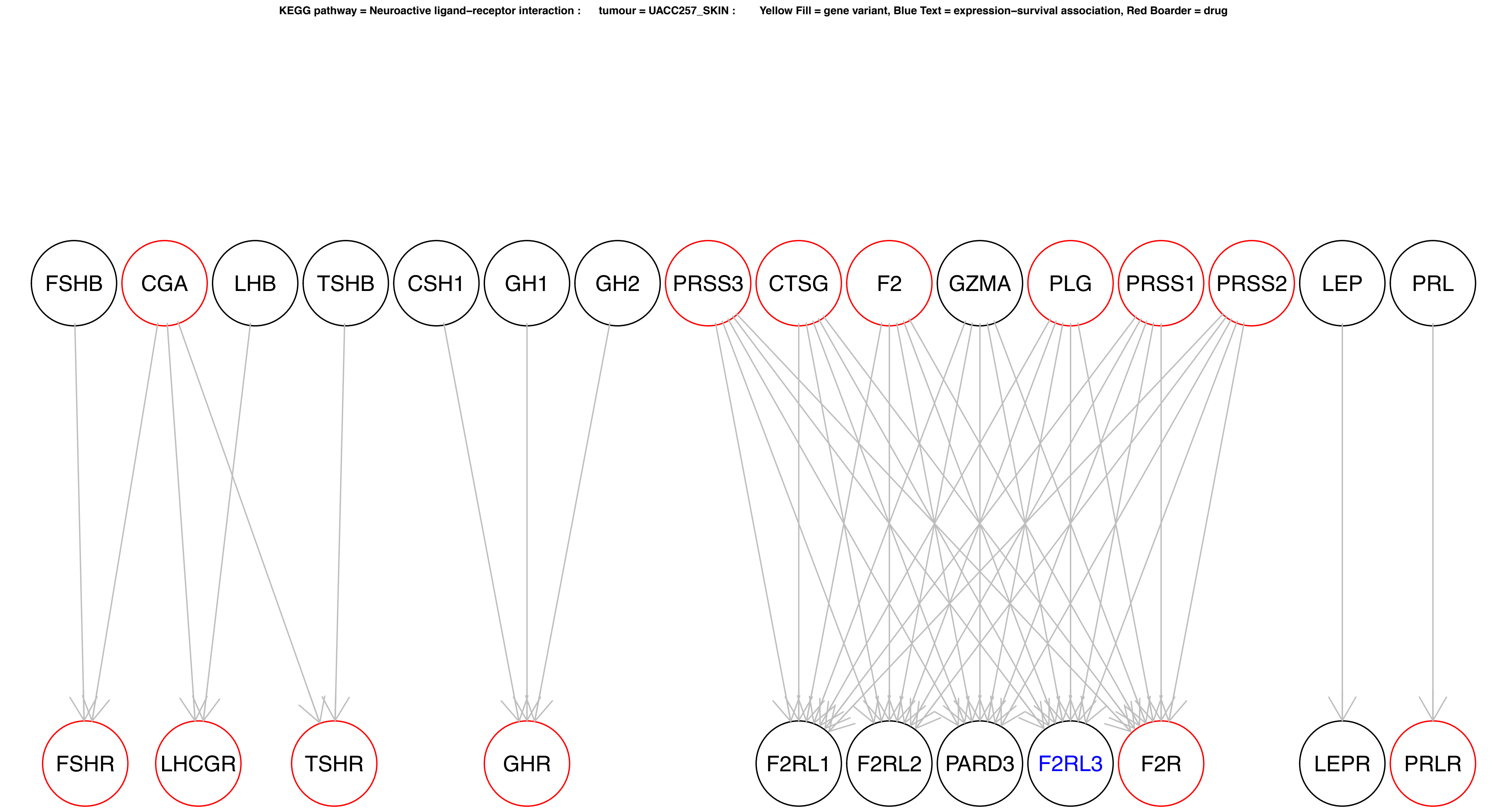
KEGG pathway = Neuroactive ligand−receptor interaction : tumour = UACC257_SKIN : Yellow Fill = gene variant, Blue Text = expression−survival association, Red Boarder = drug
FSHB
CGA
LHB
TSHB
CSH1
GH1
GH2
PRSS3
CTSG
F2
GZMA
PLG
PRSS1
PRSS2
LEP
PRL
FSHR
LHCGR
TSHR
GHR
F2RL1
F2RL2
PARD3
F2RL3
F2R
LEPR
PRLR

KEGG pathway = Neuroactive ligand−receptor interaction : tumour = UACC62_SKIN : Yellow Fill = gene variant, Blue Text = expression−survival association, Red Boarder = drug

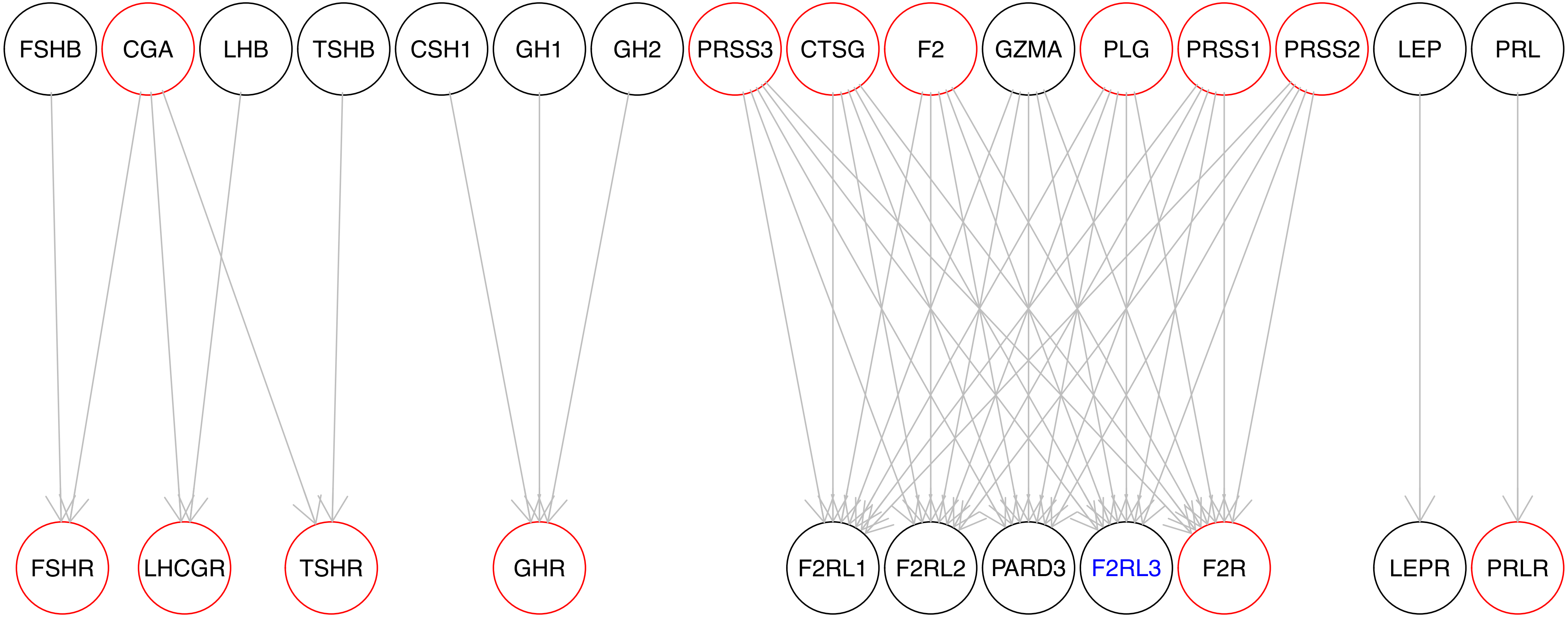

KEGG pathway = Neuroactive ligand−receptor interaction : tumour = WM115_SKIN : Yellow Fill = gene variant, Blue Text = expression−survival association, Red Boarder = drug

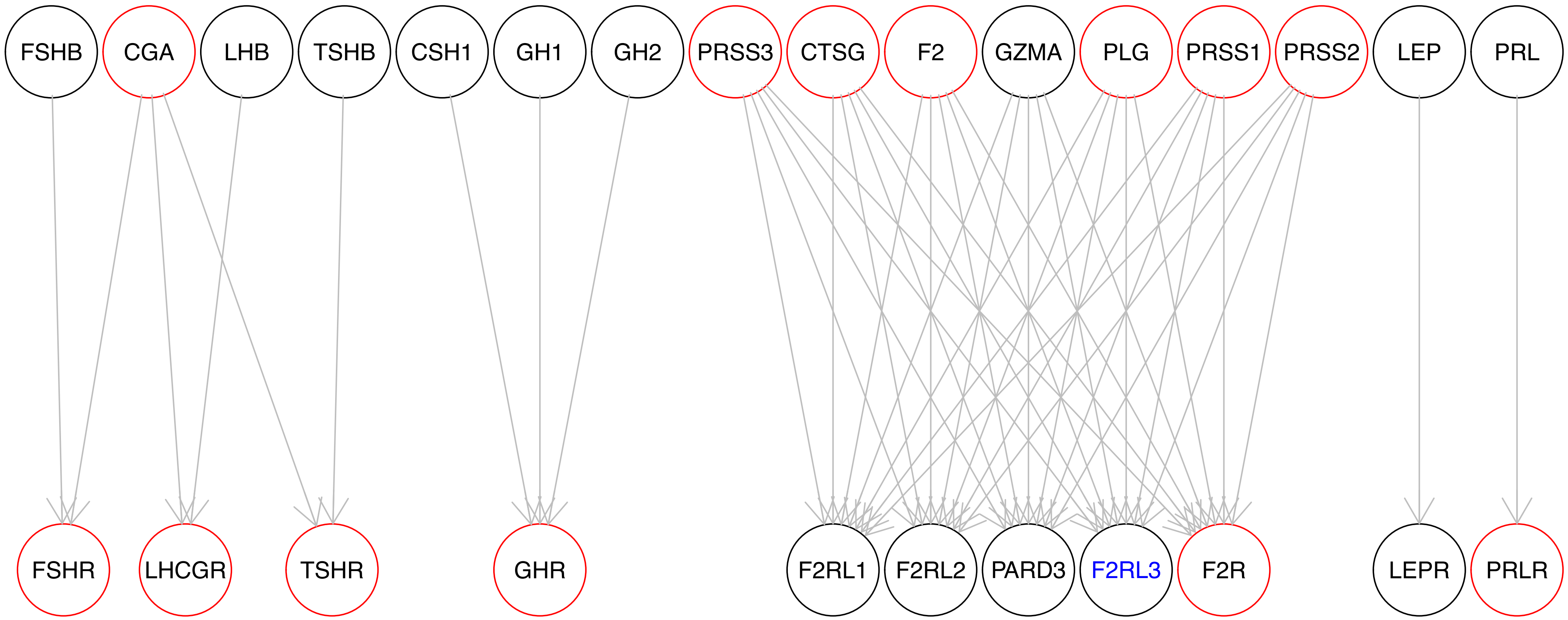

KEGG pathway = Neuroactive ligand−receptor interaction :   tumour = WM1799_SKIN :   Yellow Fill = gene variant, Blue Text = expression−survival association, Red Boarder = drug

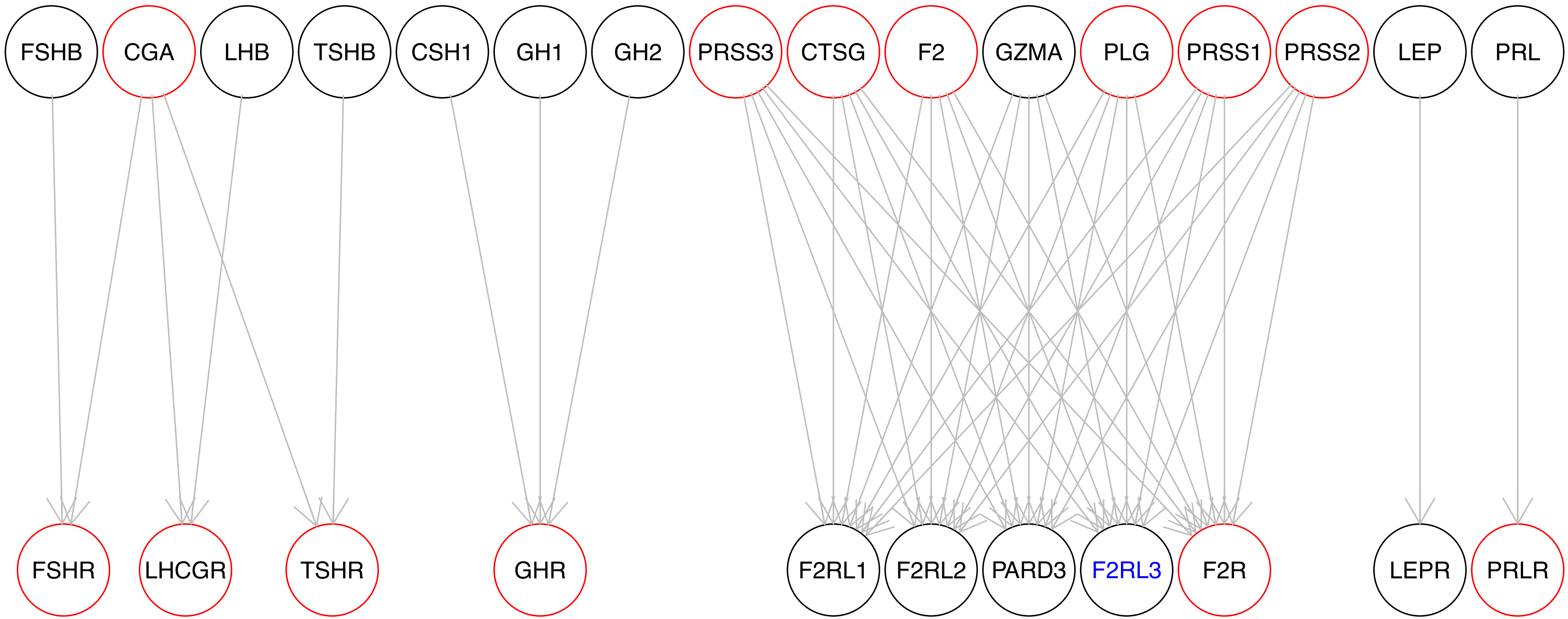

KEGG pathway = Neuroactive ligand−receptor interaction :   tumour = WM2664_SKIN :   Yellow Fill = gene variant, Blue Text = expression−survival association, Red Boarder = drug

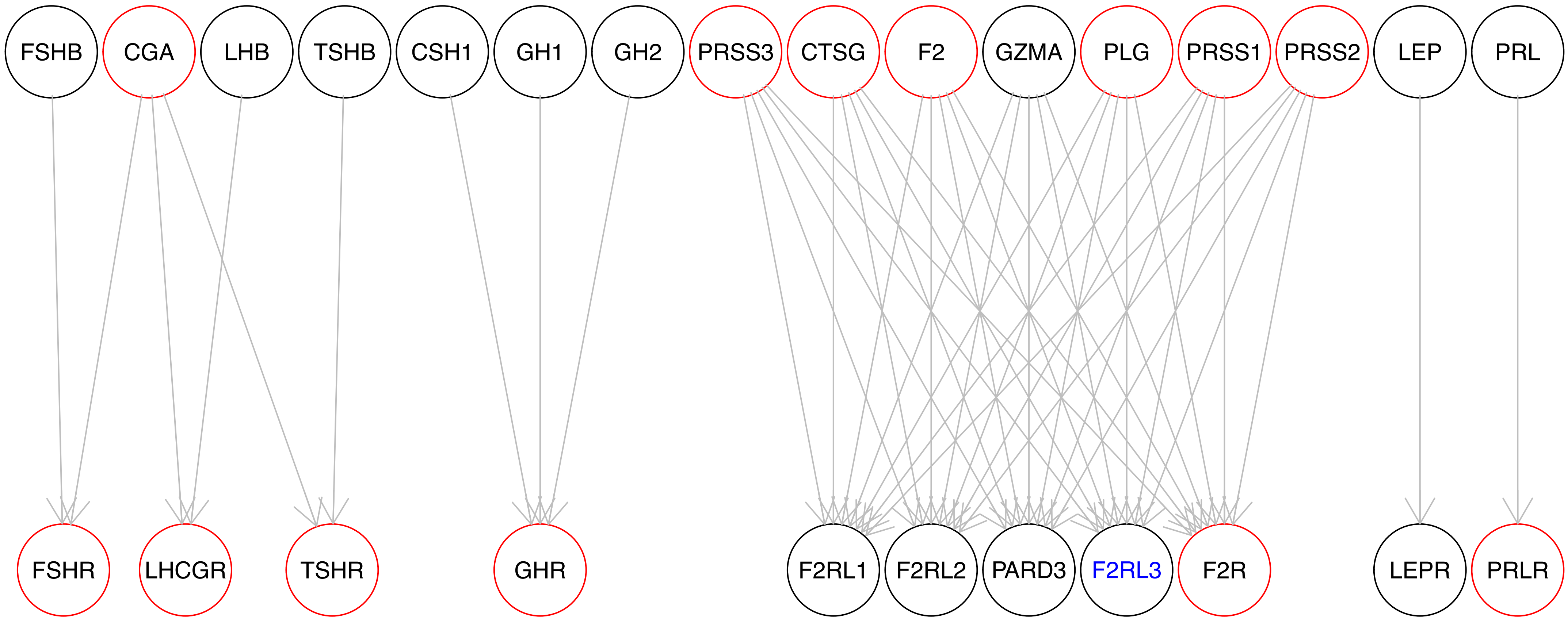

KEGG pathway = Neuroactive ligand−receptor interaction : tumour = WM793_SKIN : Yellow Fill = gene variant, Blue Text = expression−survival association, Red Boarder = drug

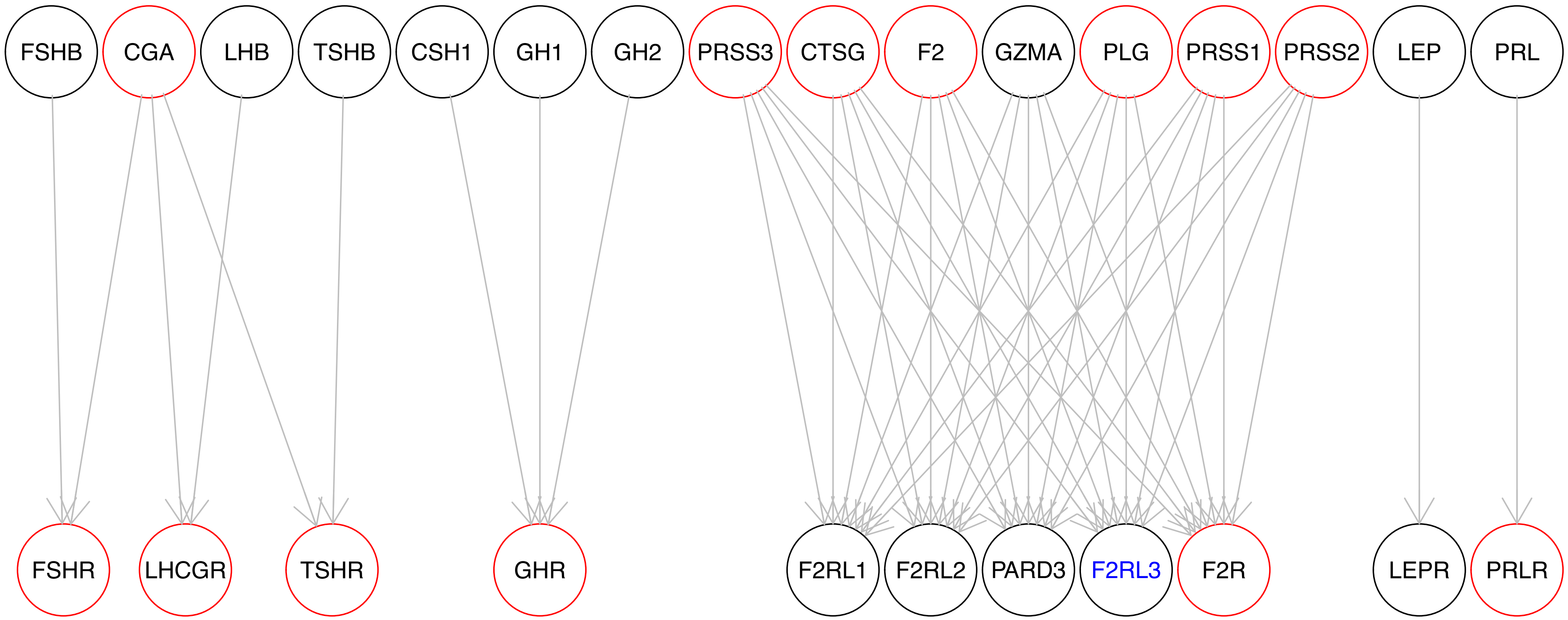

KEGG pathway = Neuroactive ligand−receptor interaction : tumour = WM88_SKIN : Yellow Fill = gene variant, Blue Text = expression−survival association, Red Boarder = drug

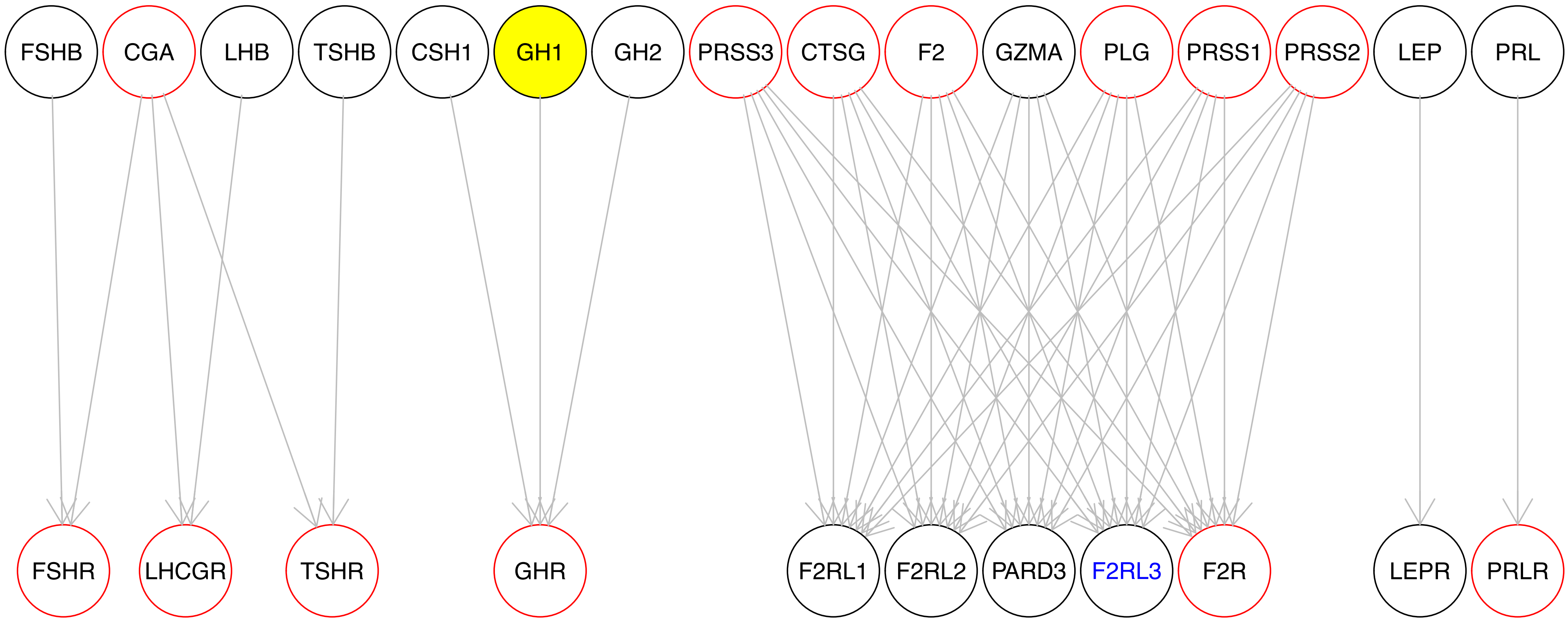

KEGG pathway = Neuroactive ligand−receptor interaction : tumour = WM983B_SKIN : Yellow Fill = gene variant, Blue Text = expression−survival association, Red Boarder = drug

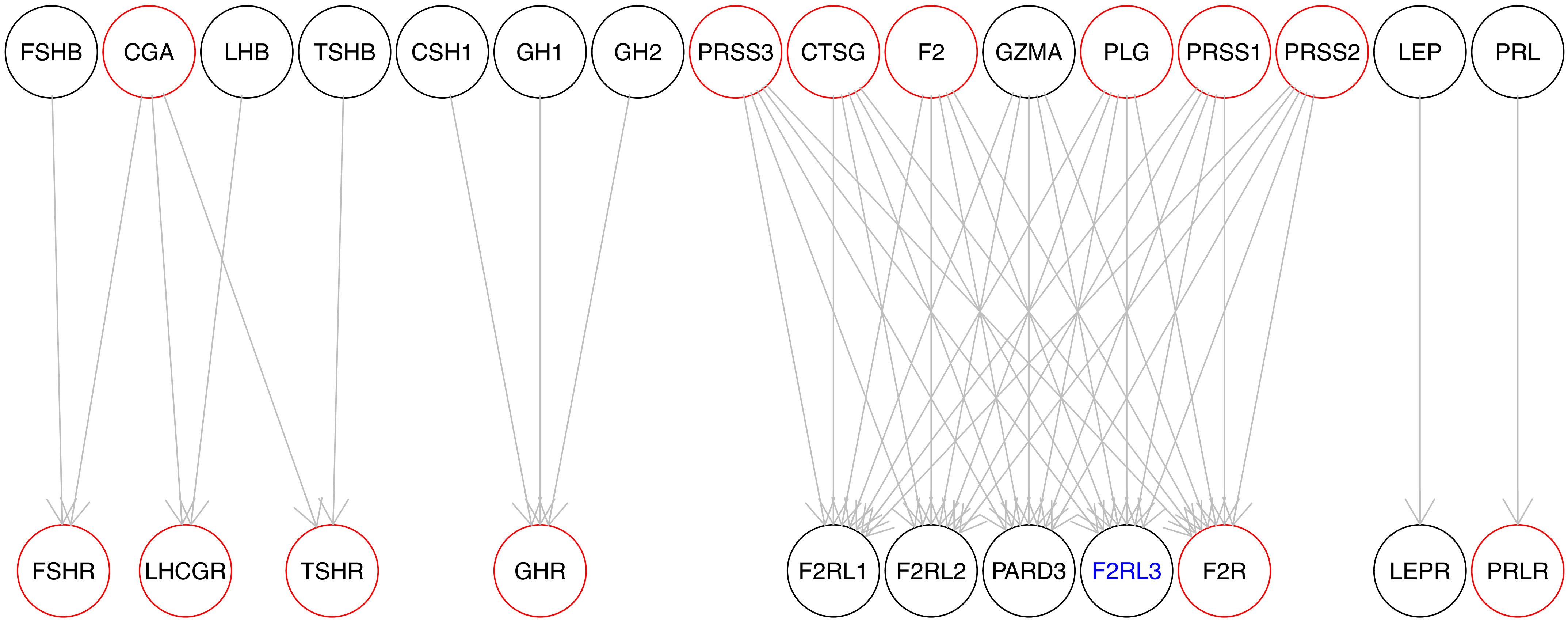

KEGG pathway = Neuroactive ligand−receptor interaction :   tumour = LAU108 :   Yellow Fill = gene variant, Blue Text = expression−survival association, Red Boarder = drug

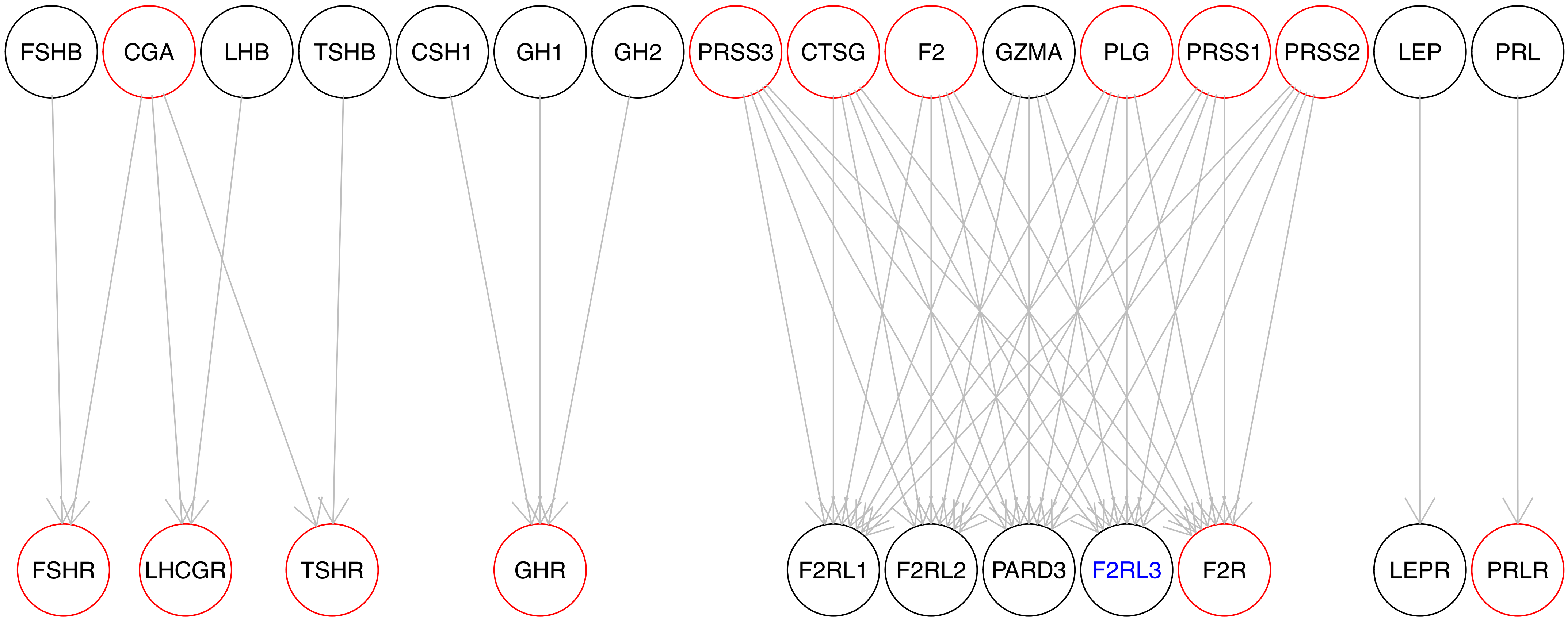

KEGG pathway = Neuroactive ligand−receptor interaction : tumour = LAU149 : Yellow Fill = gene variant, Blue Text = expression−survival association, Red Boarder = drug

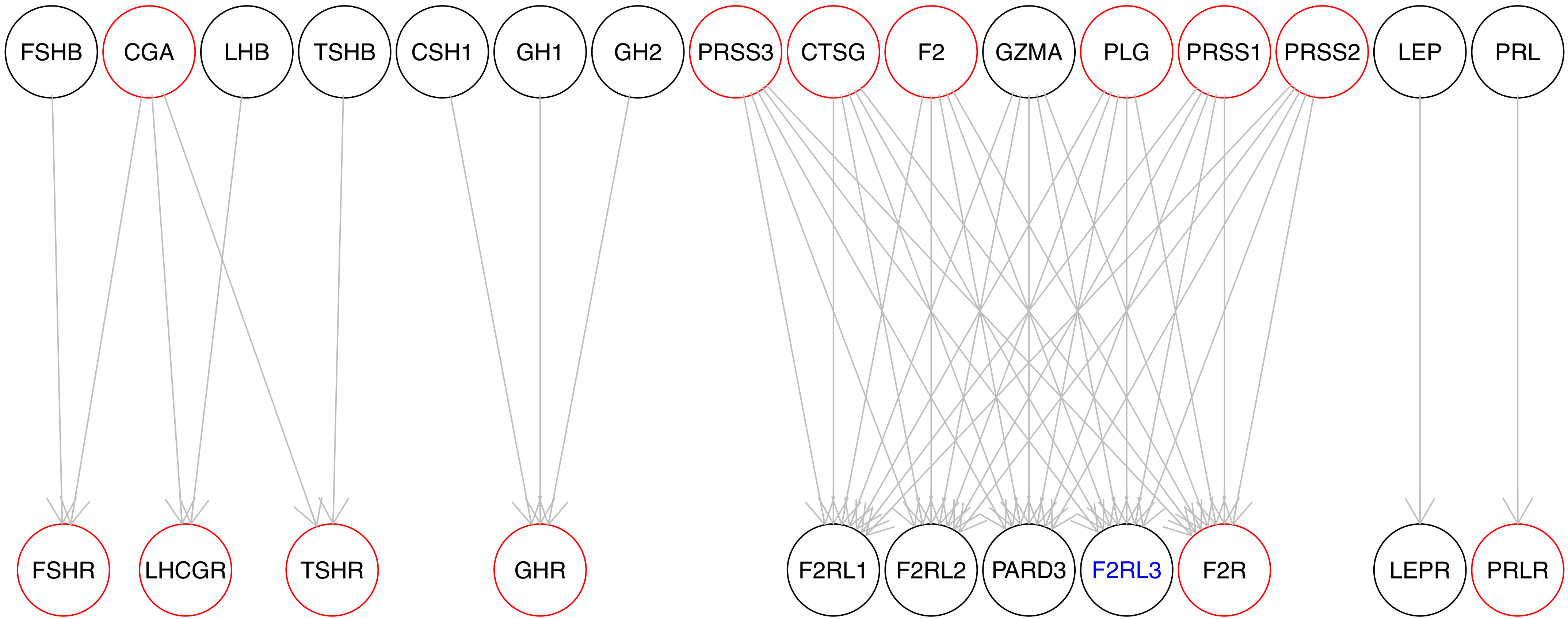

KEGG pathway = Neuroactive ligand−receptor interaction : tumour = LAU165 : Yellow Fill = gene variant, Blue Text = expression−survival association, Red Boarder = drug

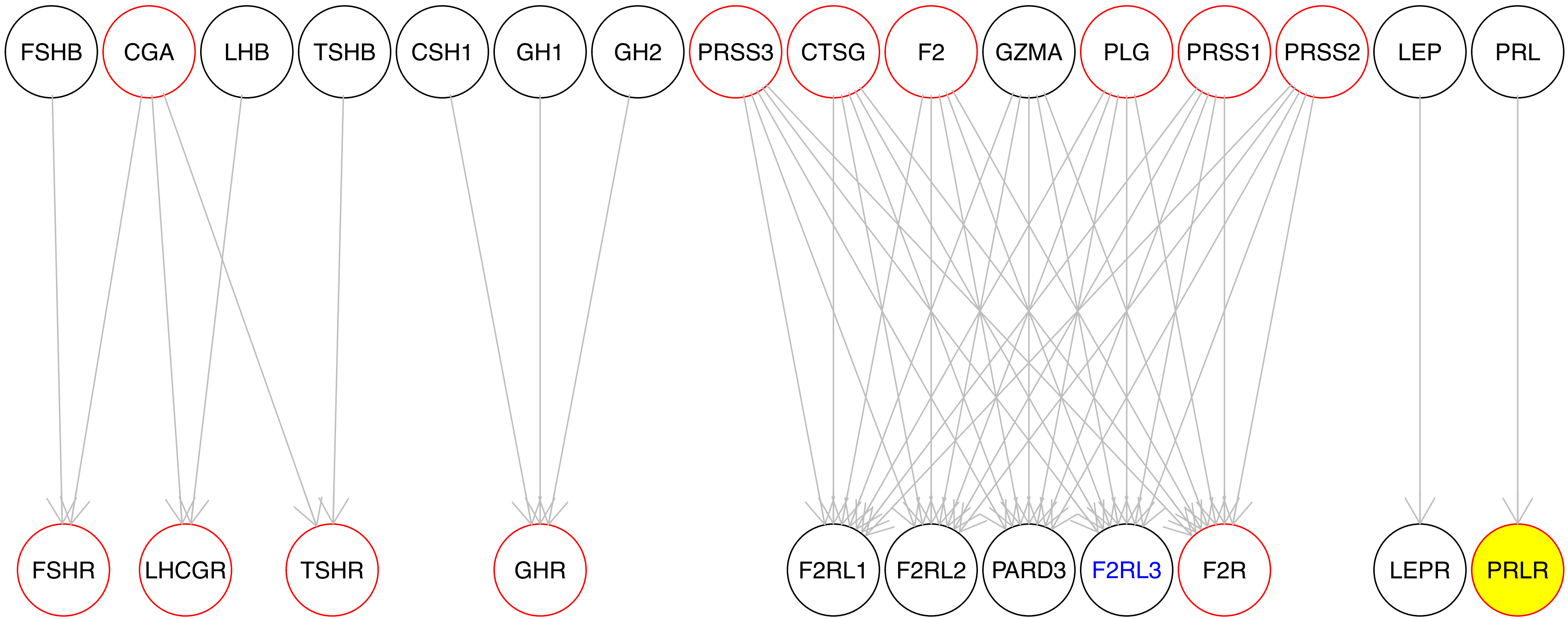

KEGG pathway = Neuroactive ligand−receptor interaction :   tumour = LAU50_1 :   Yellow Fill = gene variant, Blue Text = expression−survival association, Red Boarder = drug

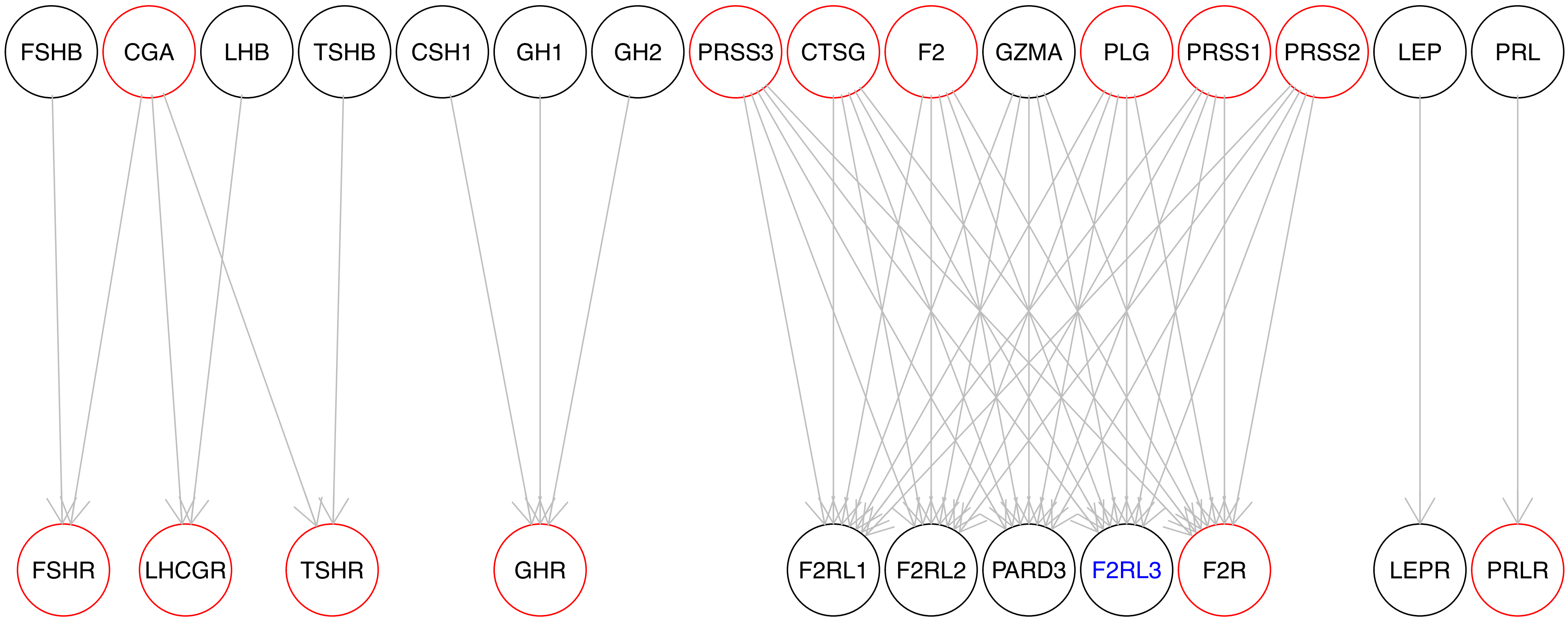

KEGG pathway = Neuroactive ligand−receptor interaction : tumour = LAU50_2 : Yellow Fill = gene variant, Blue Text = expression−survival association, Red Boarder = drug

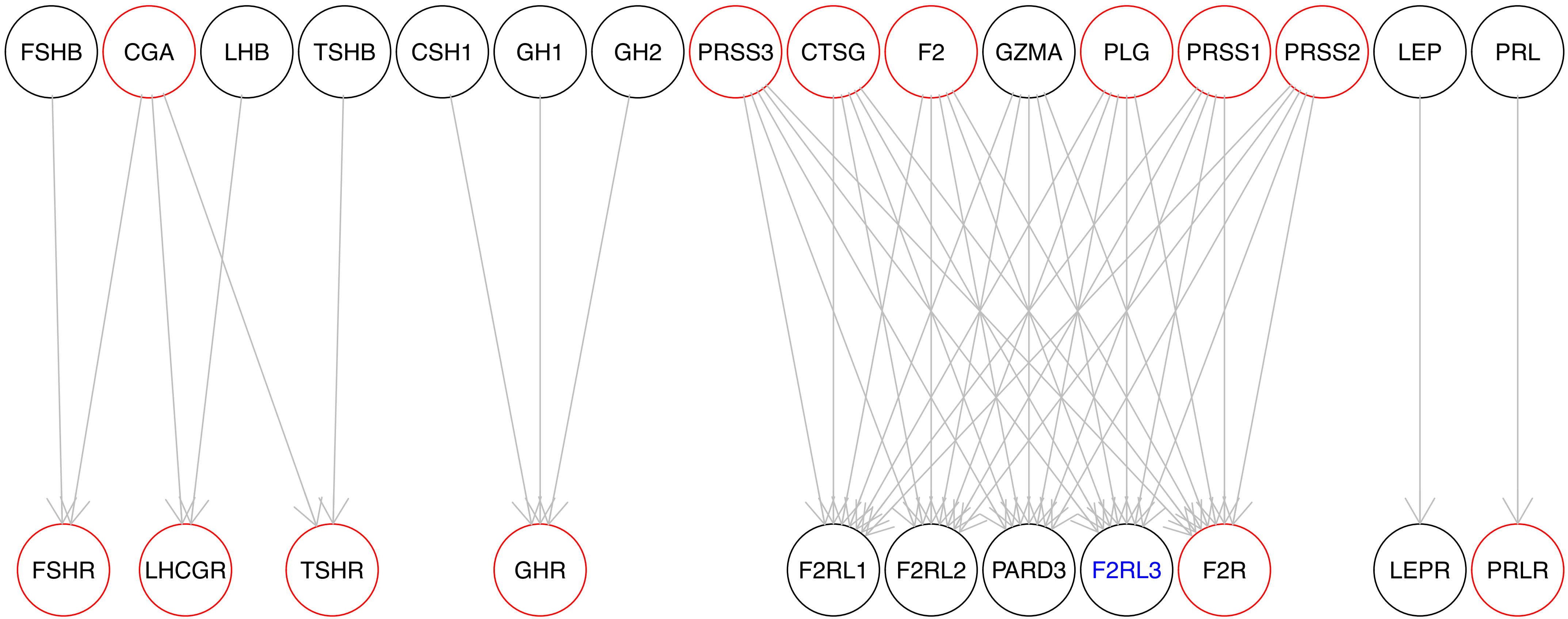

KEGG pathway = Neuroactive ligand−receptor interaction :   tumour = LAU618 :   Yellow Fill = gene variant, Blue Text = expression−survival association, Red Boarder = drug

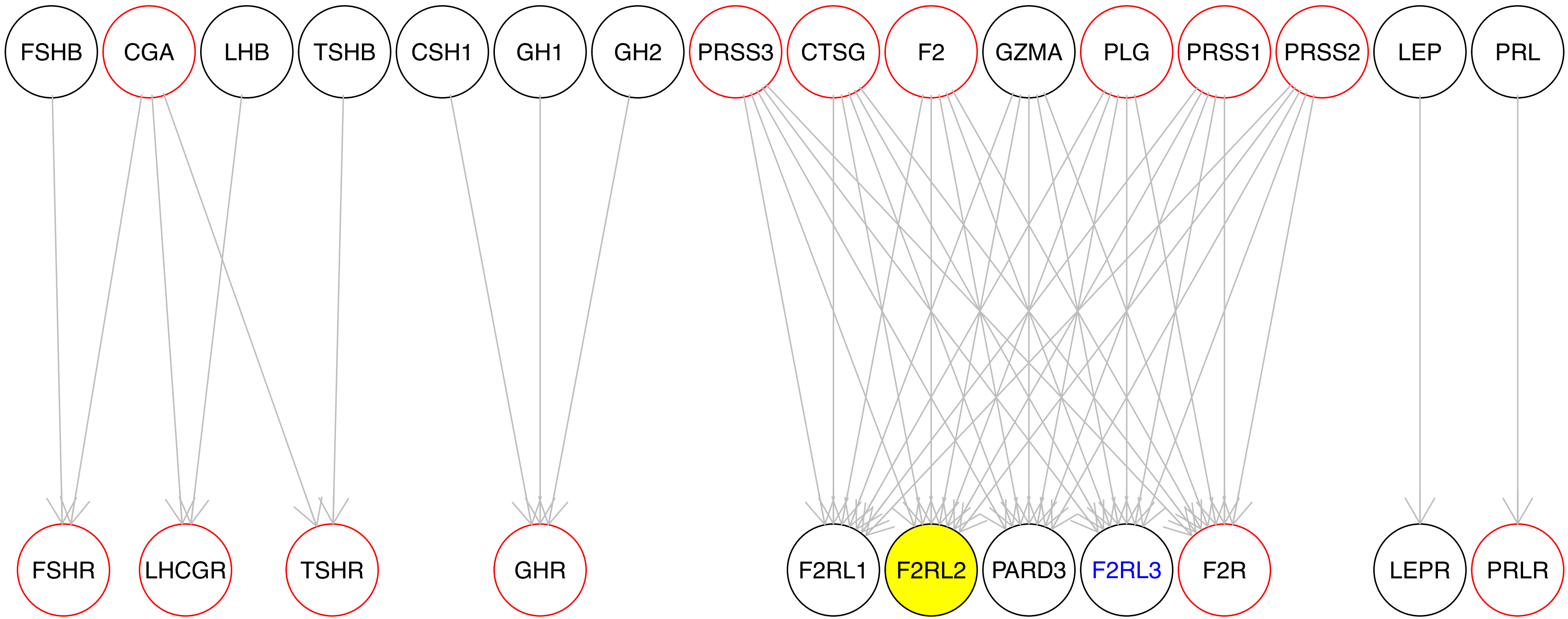

KEGG pathway = Neuroactive ligand−receptor interaction :   tumour = LAU63 :   Yellow Fill = gene variant, Blue Text = expression−survival association, Red Boarder = drug

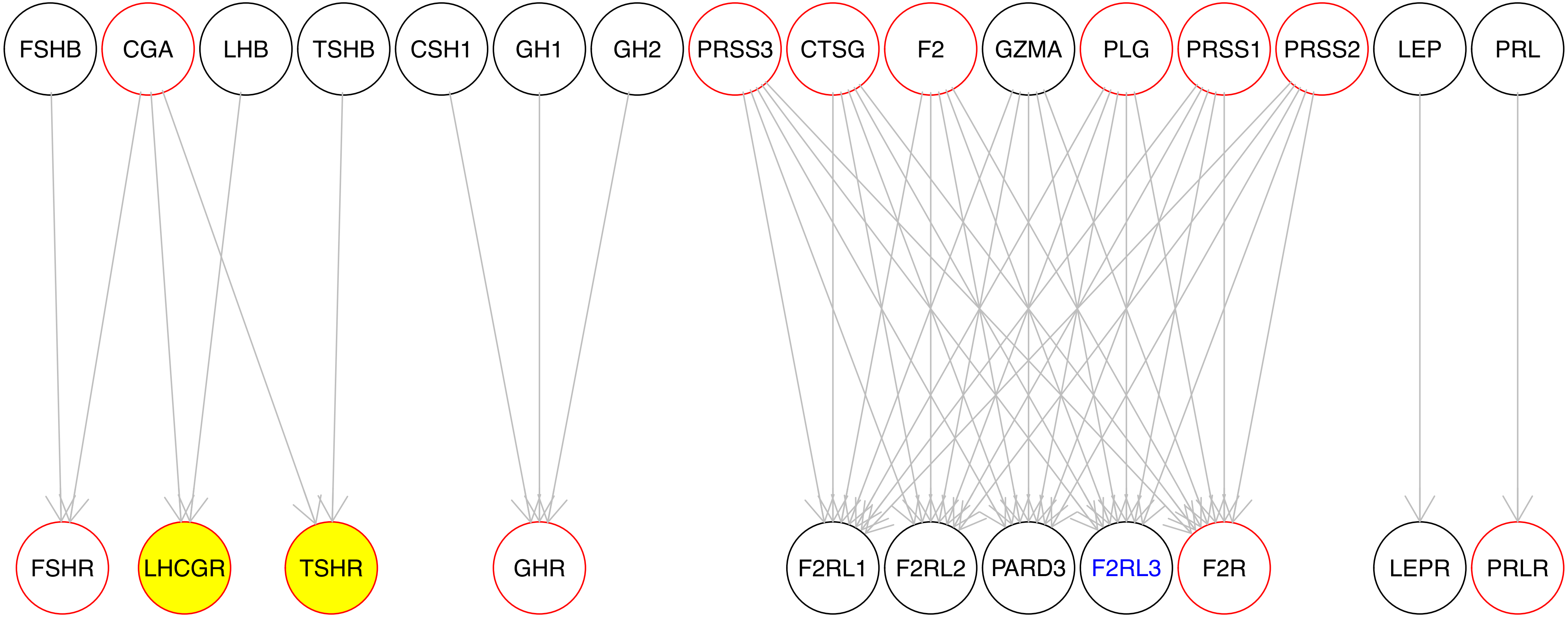

KEGG pathway = Neuroactive ligand−receptor interaction :   tumour = COLO.829 :   Yellow Fill = gene variant, Blue Text = expression−survival association, Red Boarder = drug

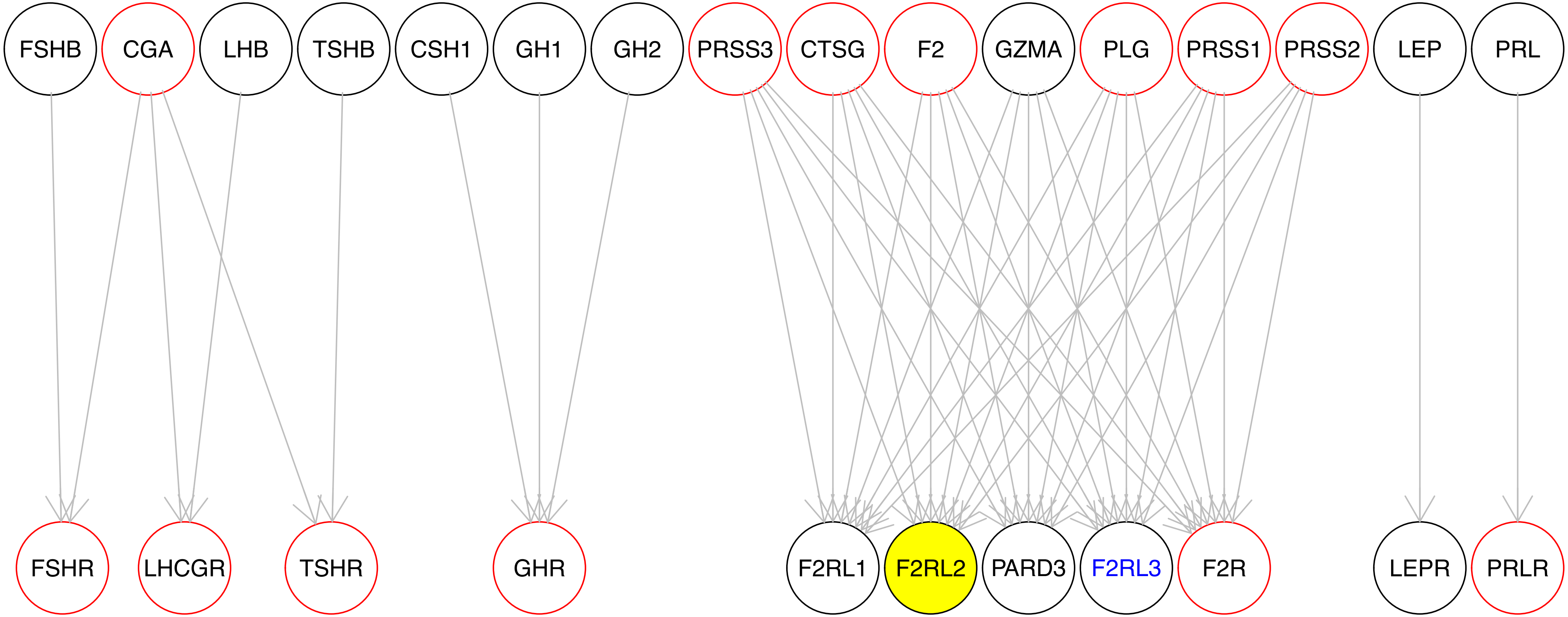

KEGG pathway = Neuroactive ligand−receptor interaction :   tumour = CP50.MEL.B :   Yellow Fill = gene variant, Blue Text = expression−survival association, Red Boarder = drug

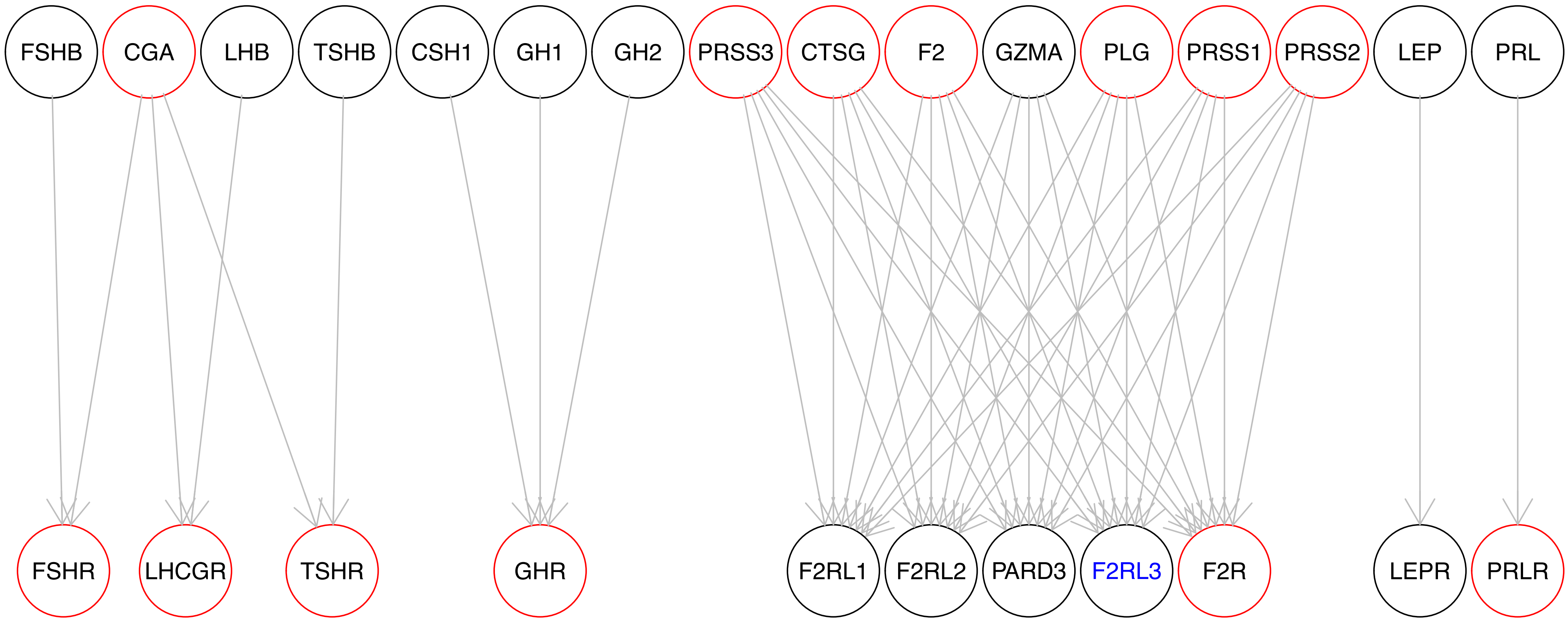

KEGG pathway = Neuroactive ligand−receptor interaction : tumour = CP66.MEL : Yellow Fill = gene variant, Blue Text = expression−survival association, Red Boarder = drug

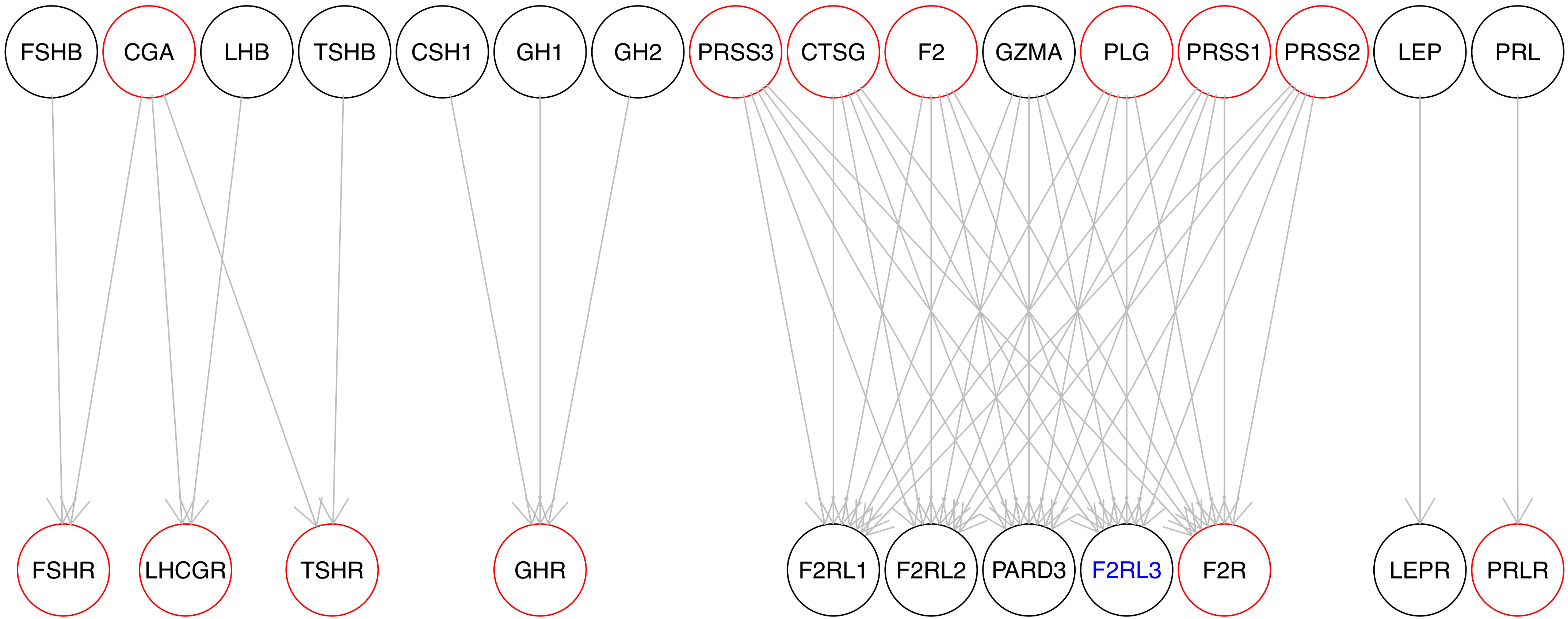

KEGG pathway = Neuroactive ligand−receptor interaction : tumour = LB2518.MEL : Yellow Fill = gene variant, Blue Text = expression−survival association, Red Boarder = drug

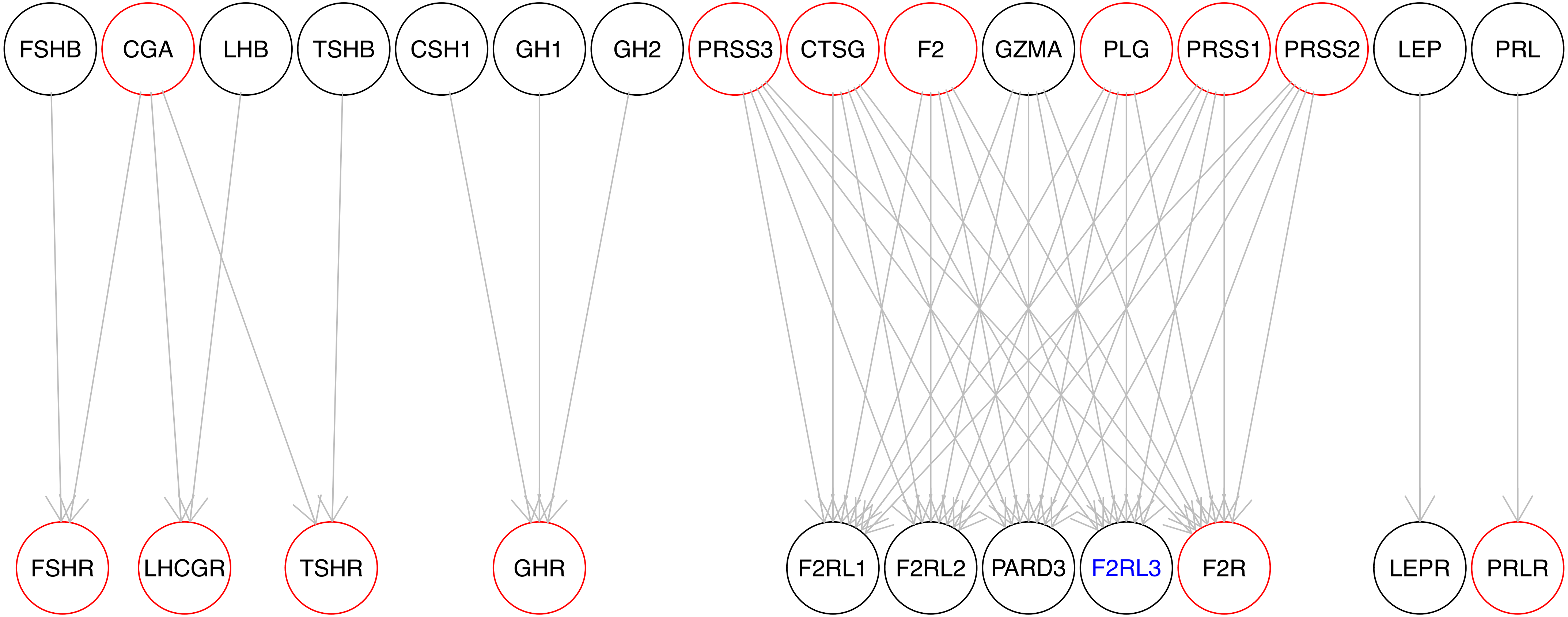

KEGG pathway = Neuroactive ligand−receptor interaction :   tumour = LB373.MEL.D :   Yellow Fill = gene variant, Blue Text = expression−survival association, Red Boarder = drug

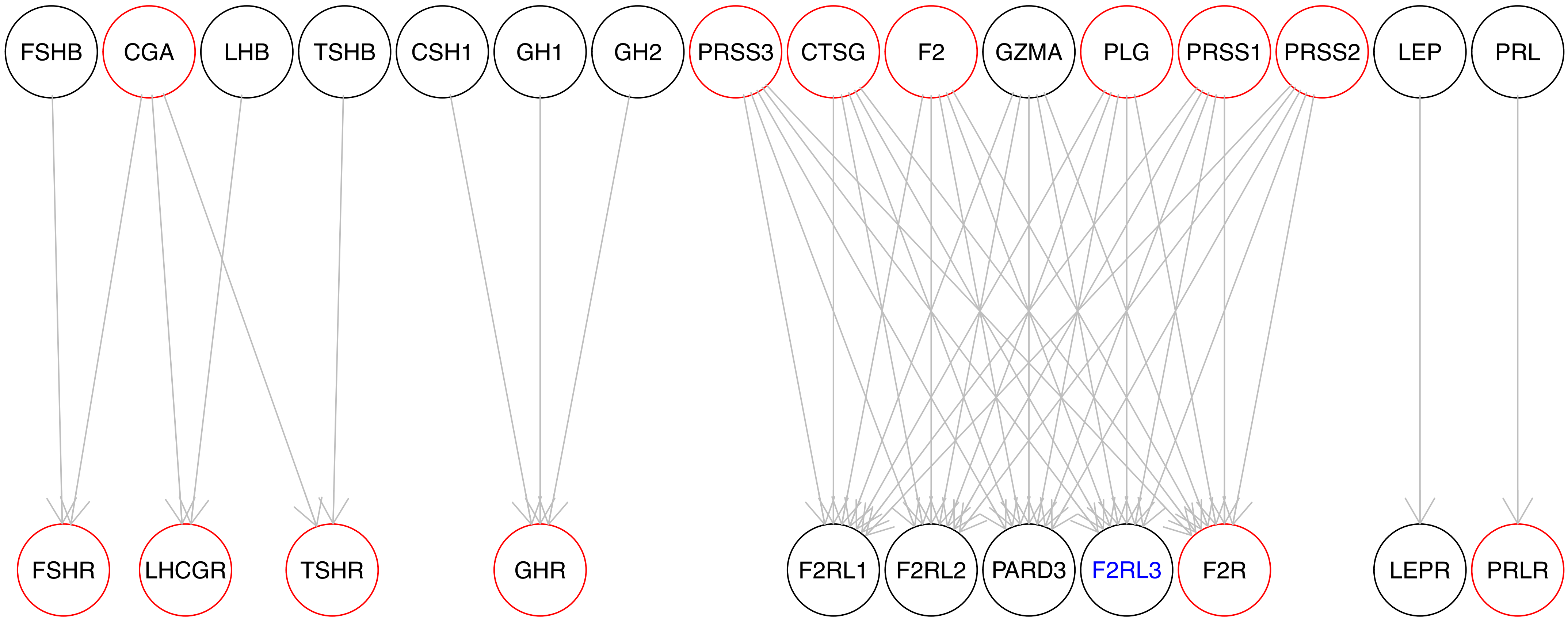

KEGG pathway = Neuroactive ligand−receptor interaction :   tumour = MZ7.mel :   Yellow Fill = gene variant, Blue Text = expression−survival association, Red Boarder = drug

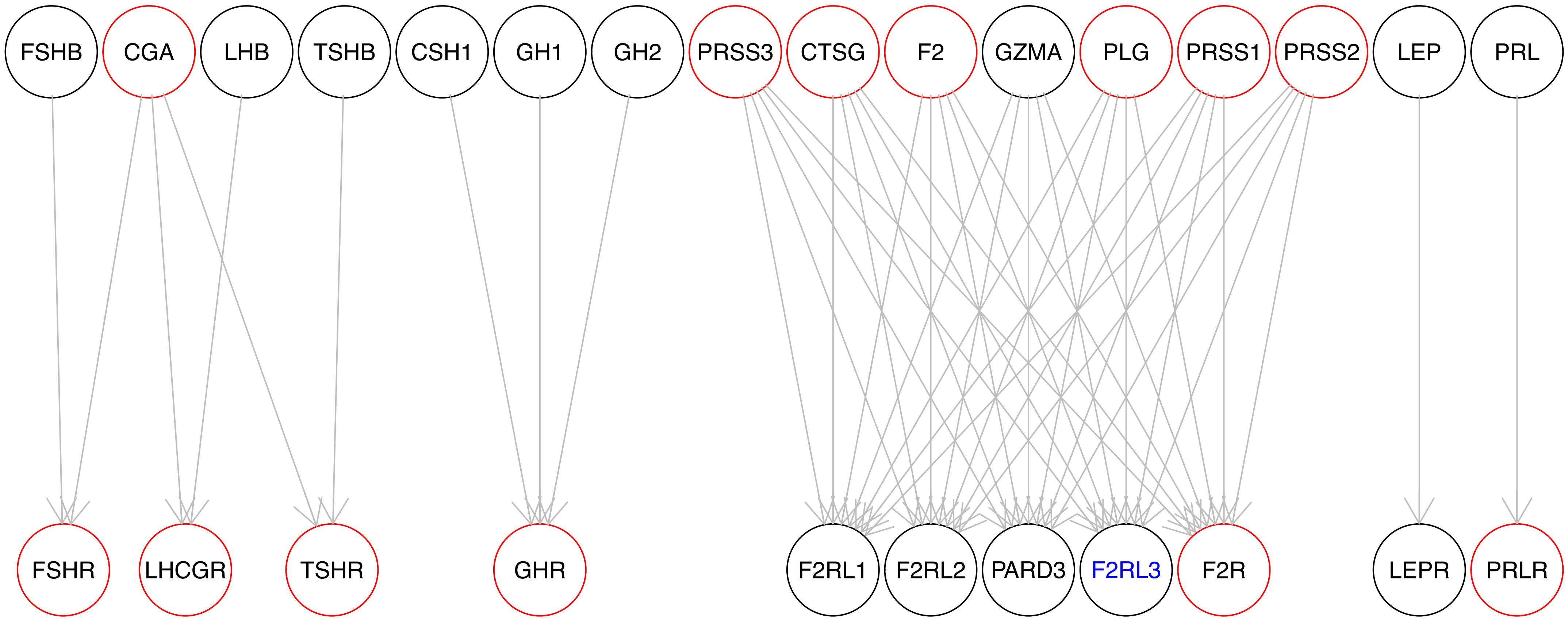

KEGG pathway = Neuroactive ligand−receptor interaction :   tumour = A02 :   Yellow Fill = gene variant, Blue Text = expression−survival association, Red Boarder = drug

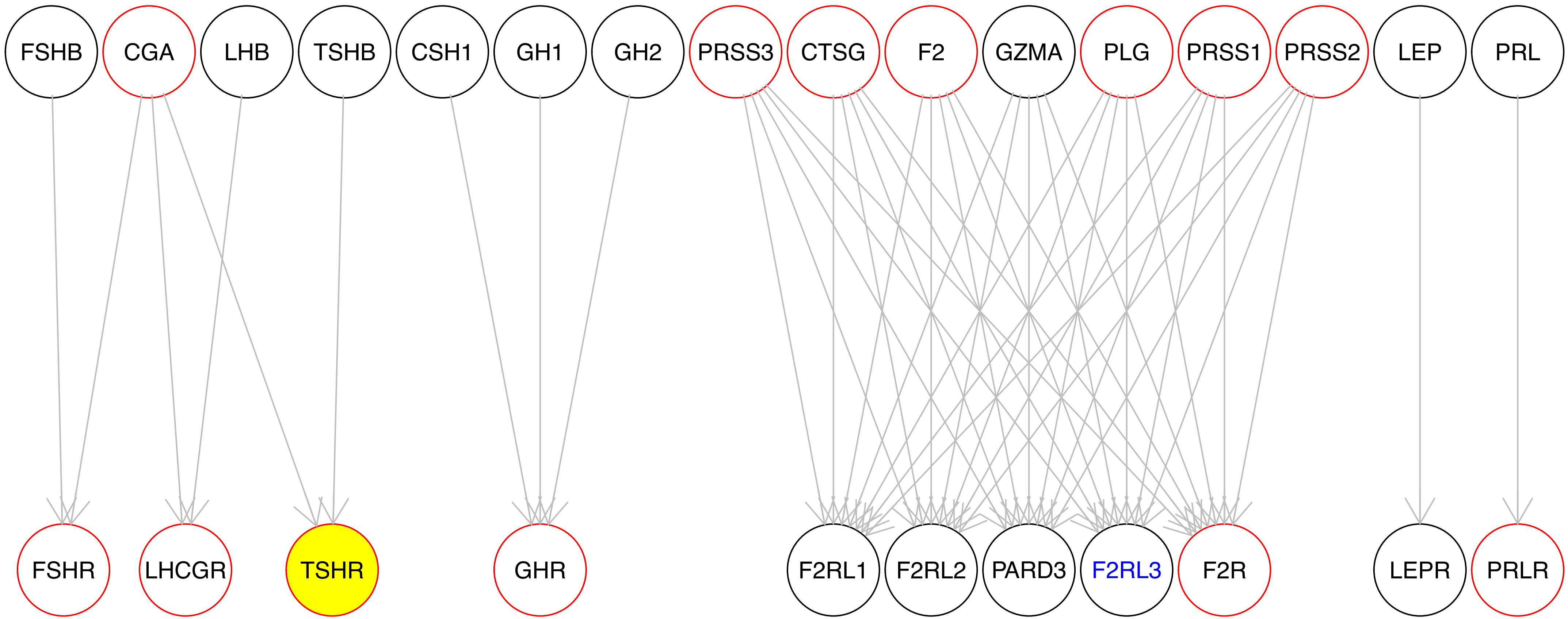

KEGG pathway = Neuroactive ligand−receptor interaction : tumour = A06 : Yellow Fill = gene variant, Blue Text = expression−survival association, Red Boarder = drug

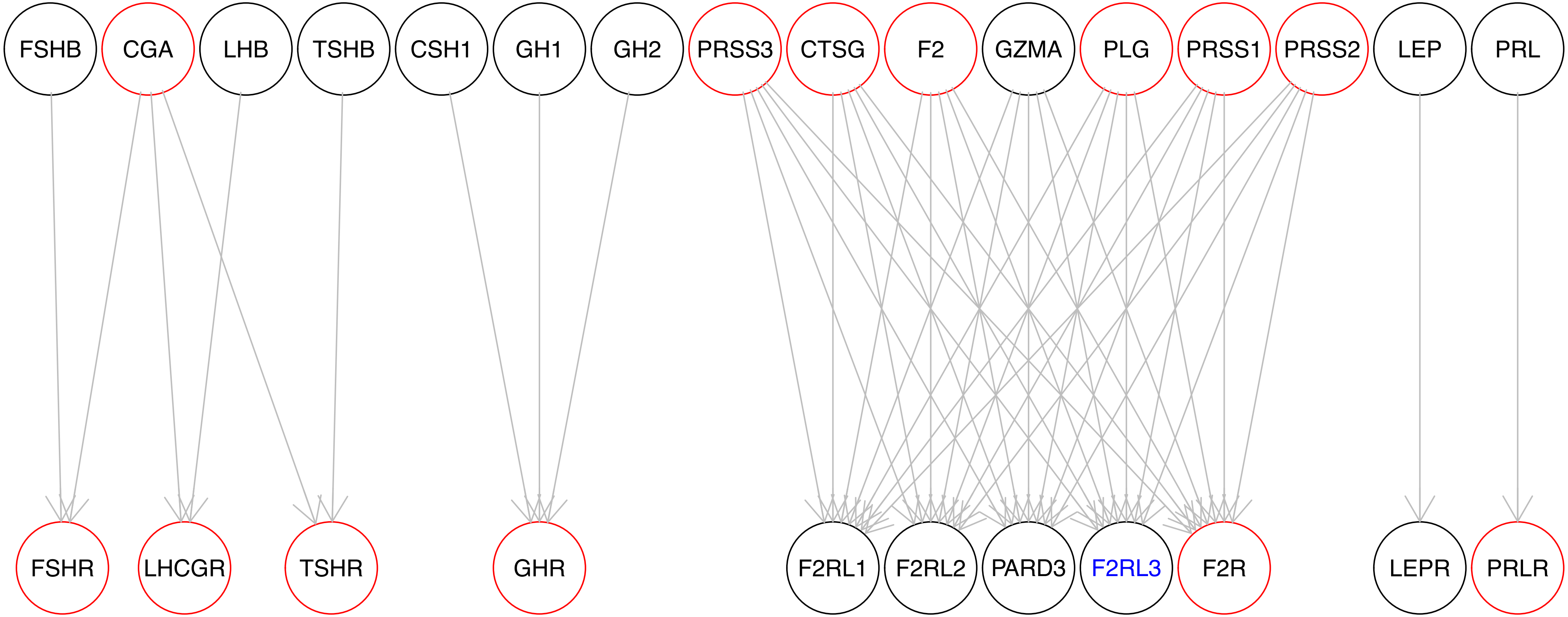

KEGG pathway = Neuroactive ligand−receptor interaction : tumour = D05 : Yellow Fill = gene variant, Blue Text = expression−survival association, Red Boarder = drug

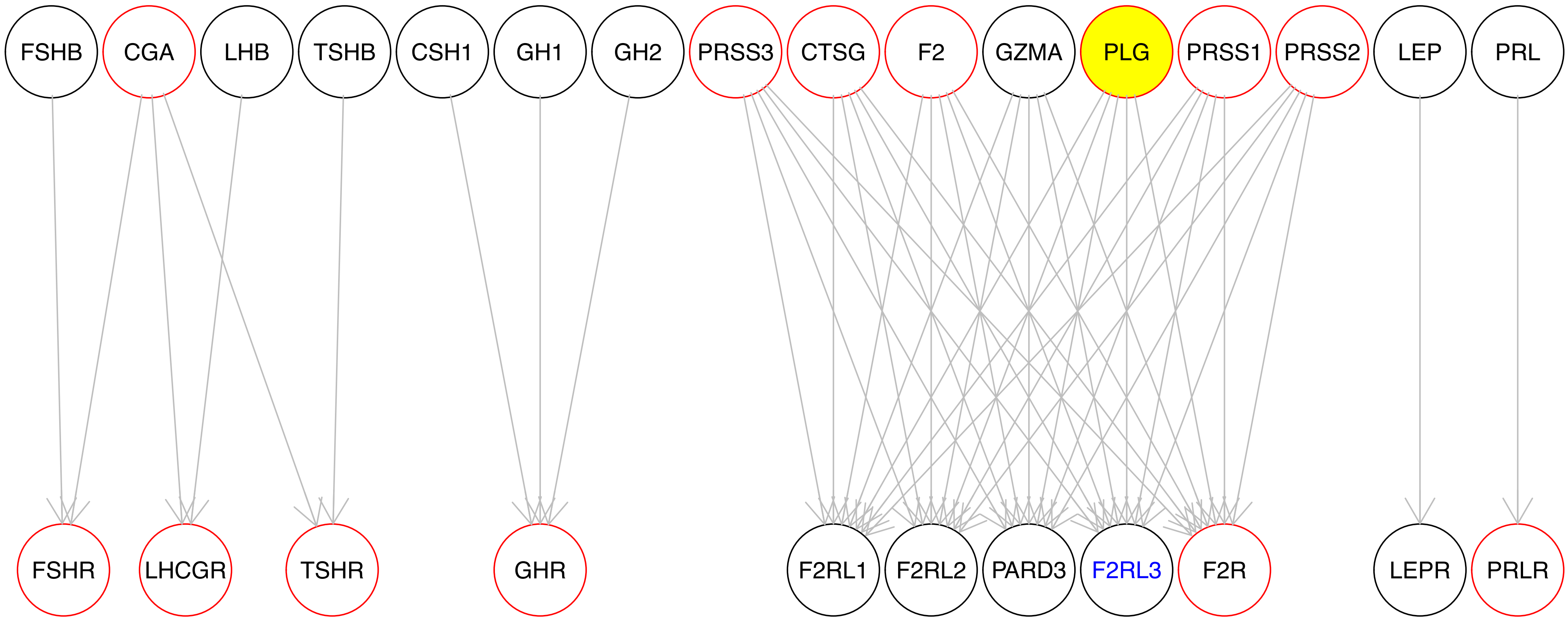

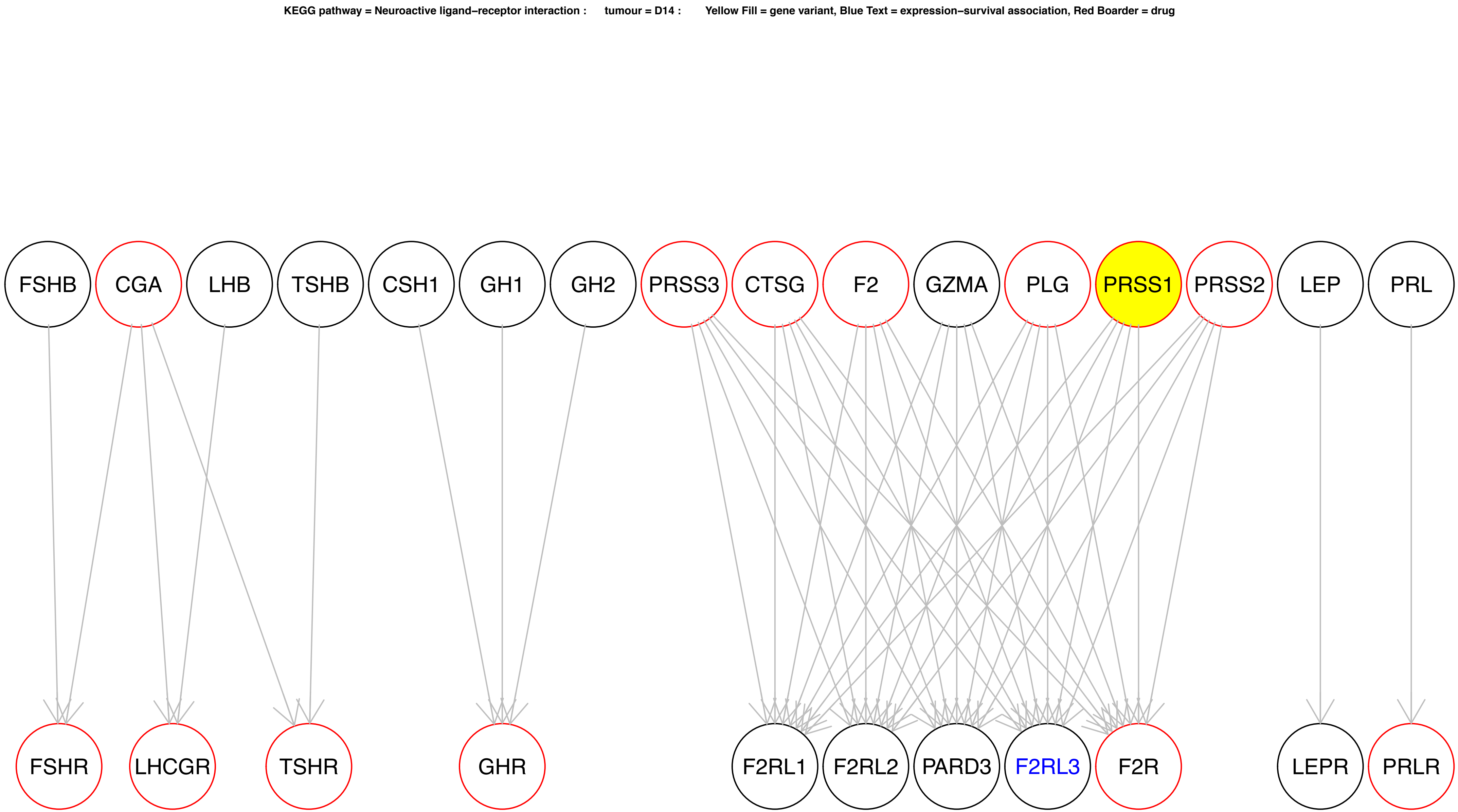

KEGG pathway = Neuroactive ligand−receptor interaction : tumour = D14 : Yellow Fill = gene variant, Blue Text = expression−survival association, Red Boarder = drug
FSHB
CGA
LHB
TSHB
CSH1
GH1
GH2
PRSS3
CTSG
F2
GZMA
PLG
PRSS1
PRSS2
LEP
PRL
FSHR
LHCGR
TSHR
GHR
F2RL1
F2RL2
PARD3
F2RL3
F2R
LEPR
PRLR

KEGG pathway = Neuroactive ligand−receptor interaction : tumour = D35 : Yellow Fill = gene variant, Blue Text = expression−survival association, Red Boarder = drug

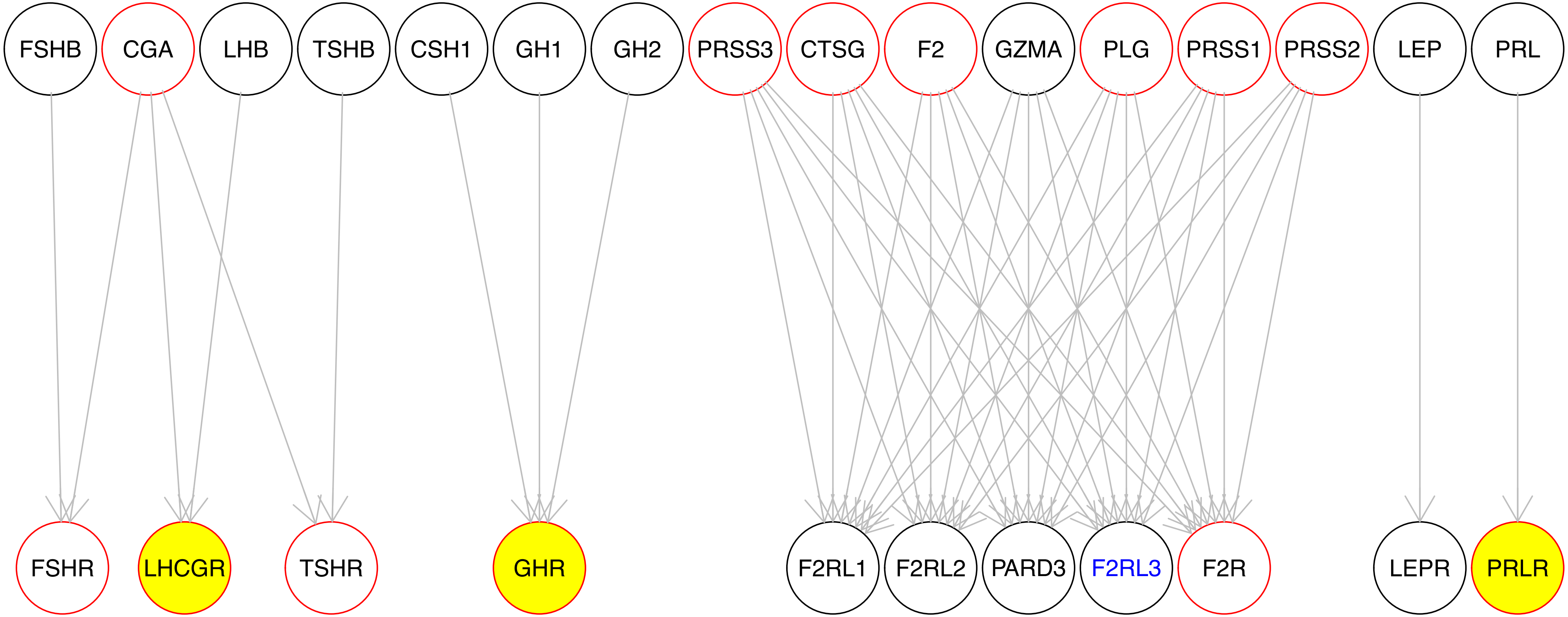

KEGG pathway = Neuroactive ligand−receptor interaction : tumour = D36 : Yellow Fill = gene variant, Blue Text = expression−survival association, Red Boarder = drug

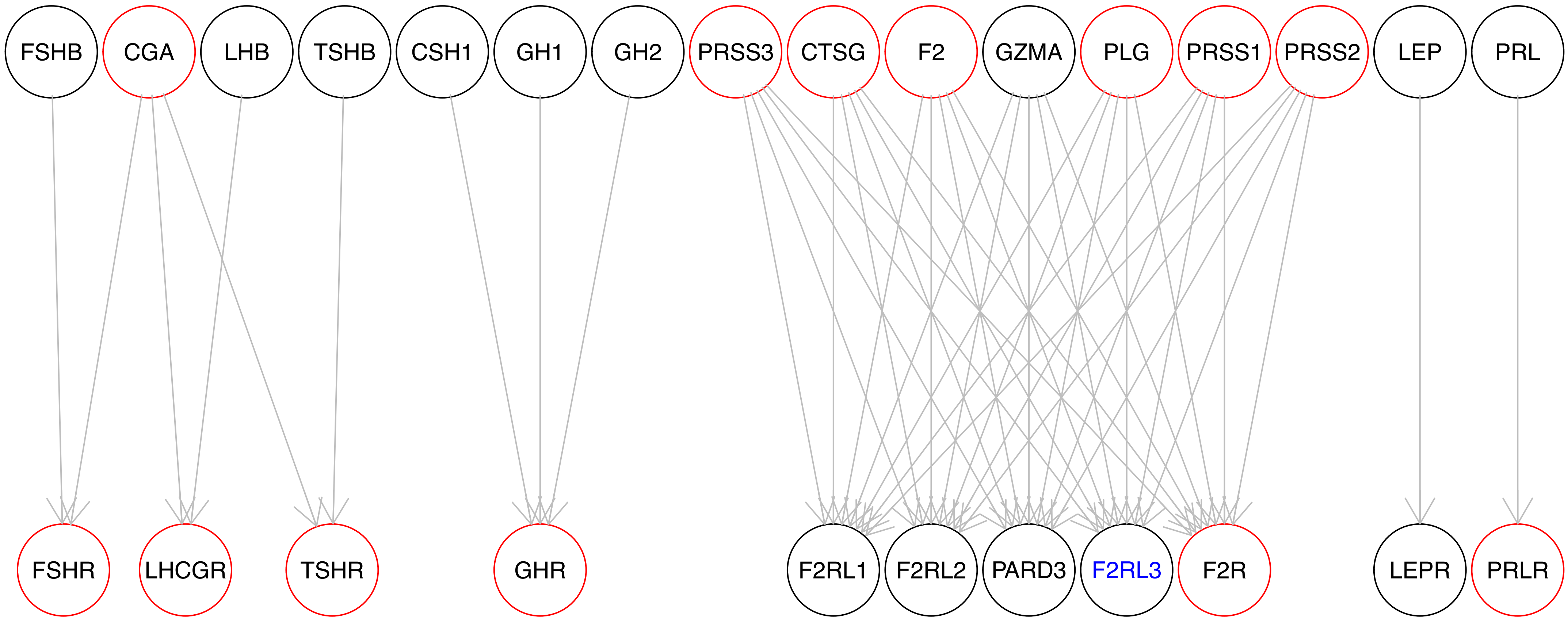

KEGG pathway = Neuroactive ligand−receptor interaction : tumour = D41 : Yellow Fill = gene variant, Blue Text = expression−survival association, Red Boarder = drug

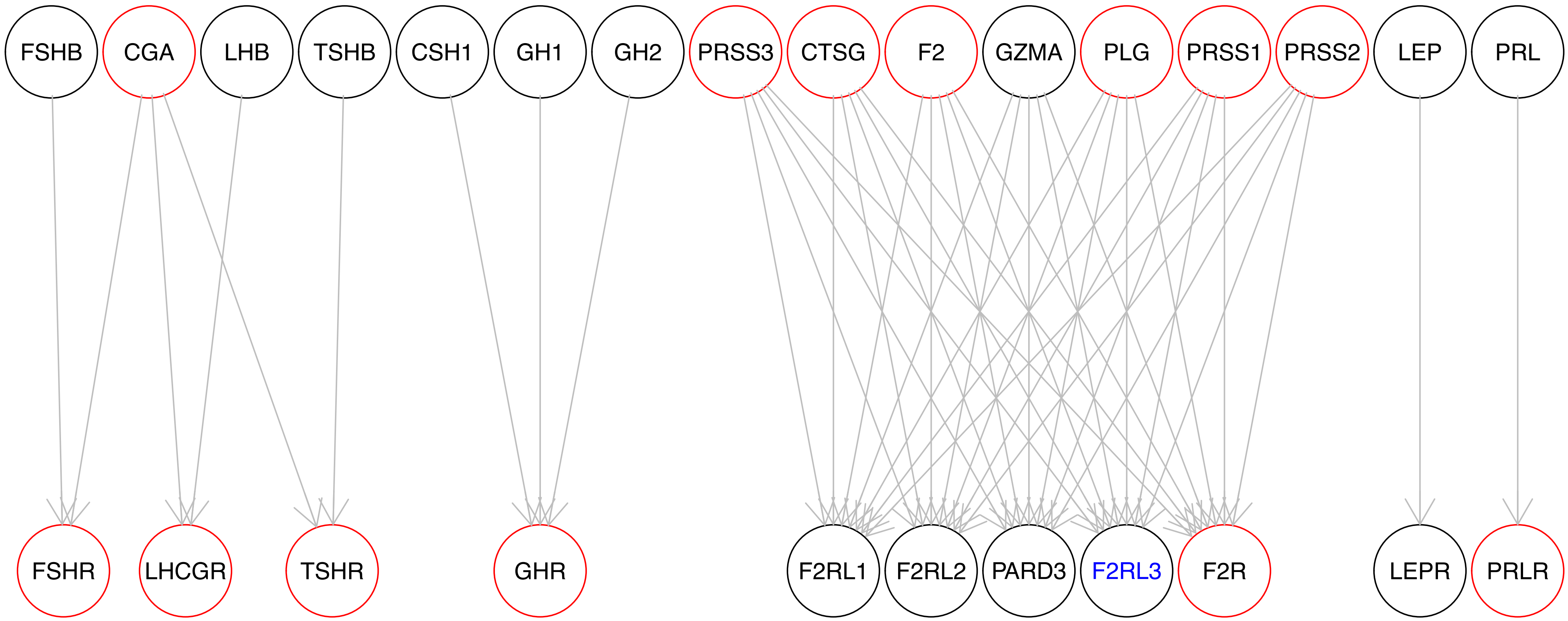

KEGG pathway = Neuroactive ligand−receptor interaction : tumour = D49 : Yellow Fill = gene variant, Blue Text = expression−survival association, Red Boarder = drug

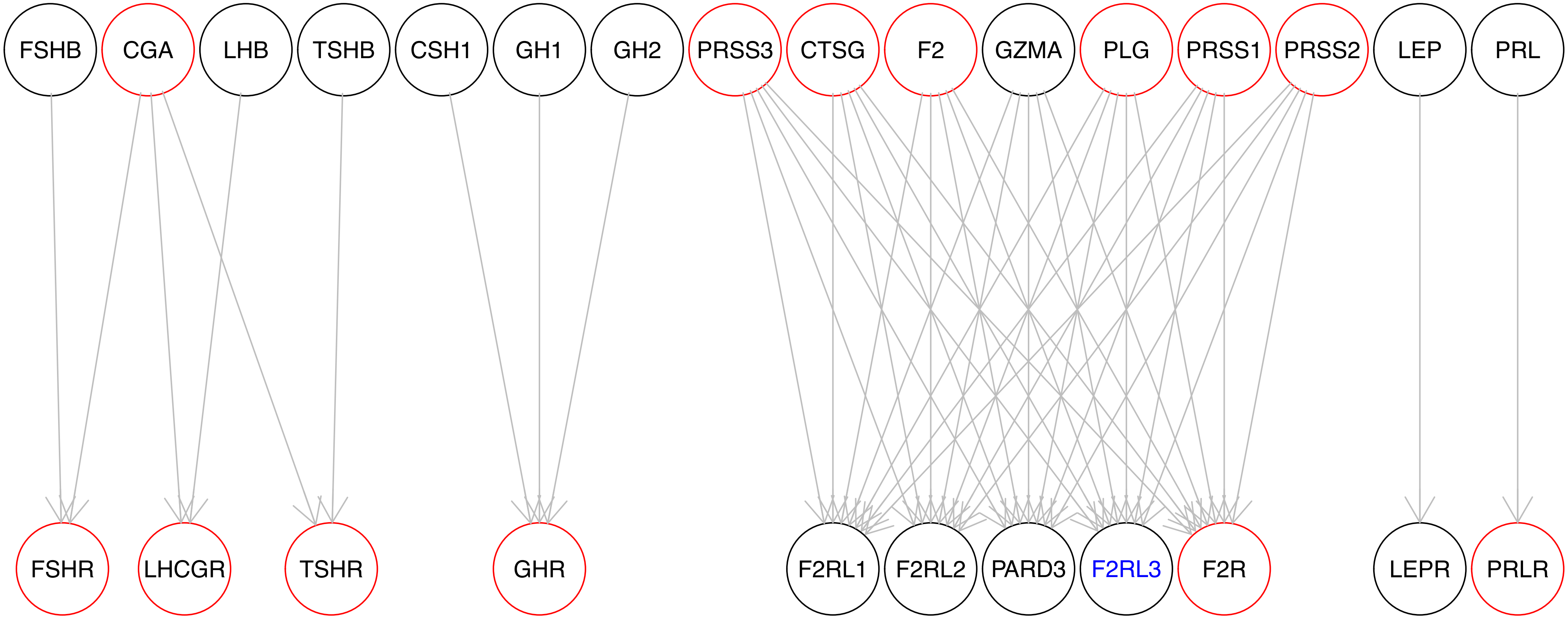

KEGG pathway = Neuroactive ligand−receptor interaction : tumour = Turajlic : Yellow Fill = gene variant, Blue Text = expression−survival association, Red Boarder = drug

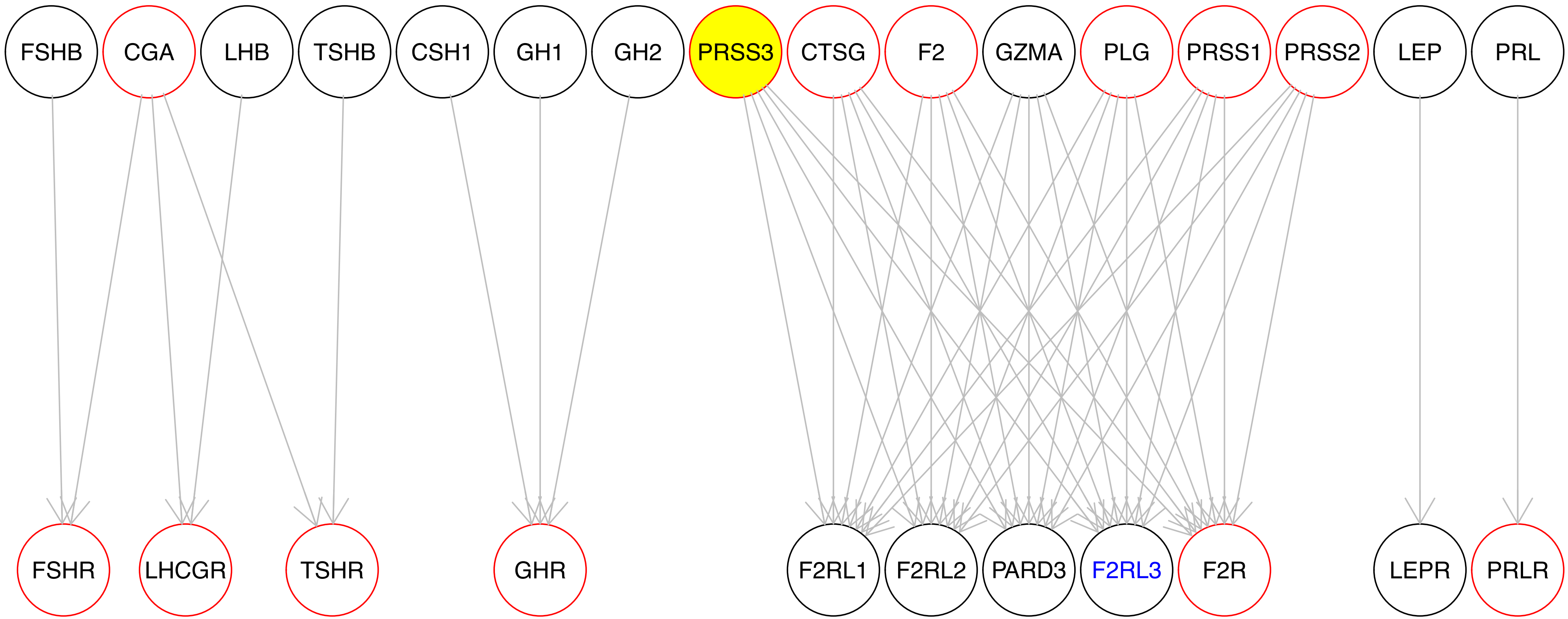

KEGG pathway = Neuroactive ligand−receptor interaction : tumour = X01T : Yellow Fill = gene variant, Blue Text = expression−survival association, Red Boarder = drug

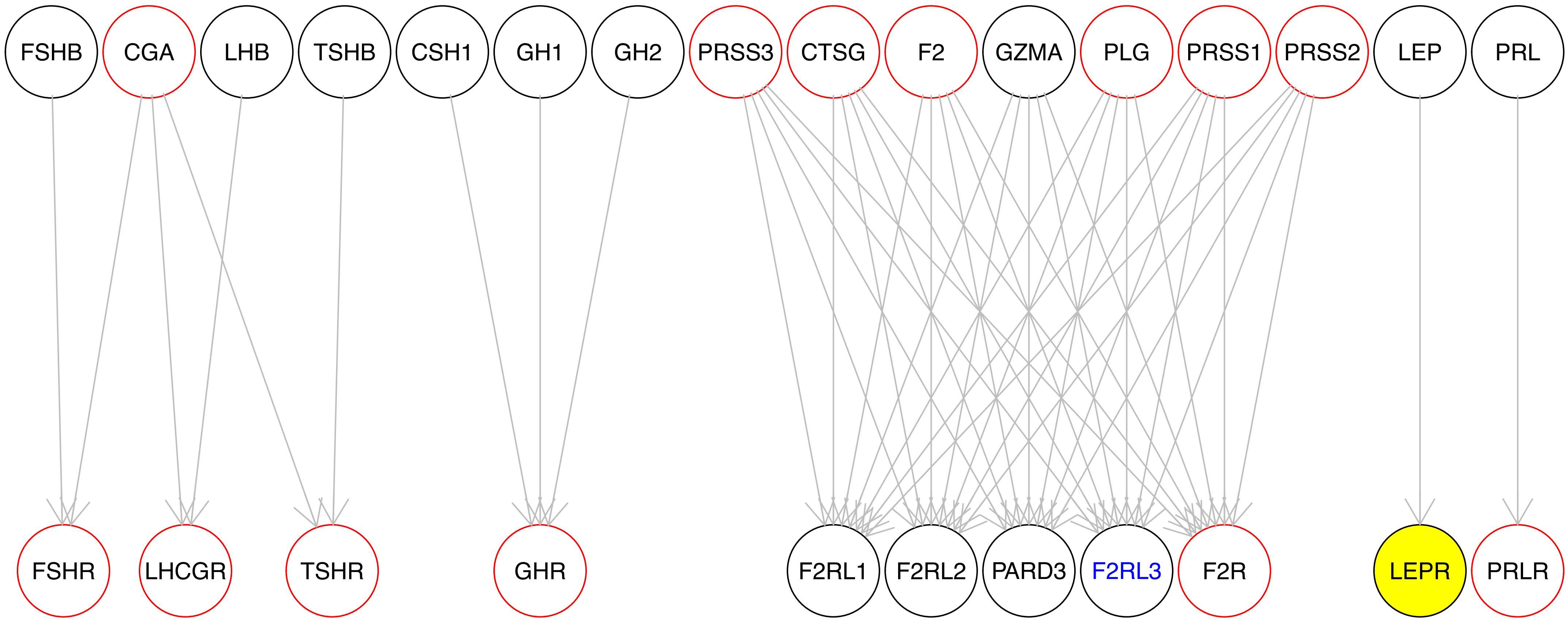

KEGG pathway = Neuroactive ligand−receptor interaction :   tumour = X05T :   Yellow Fill = gene variant, Blue Text = expression−survival association, Red Boarder = drug

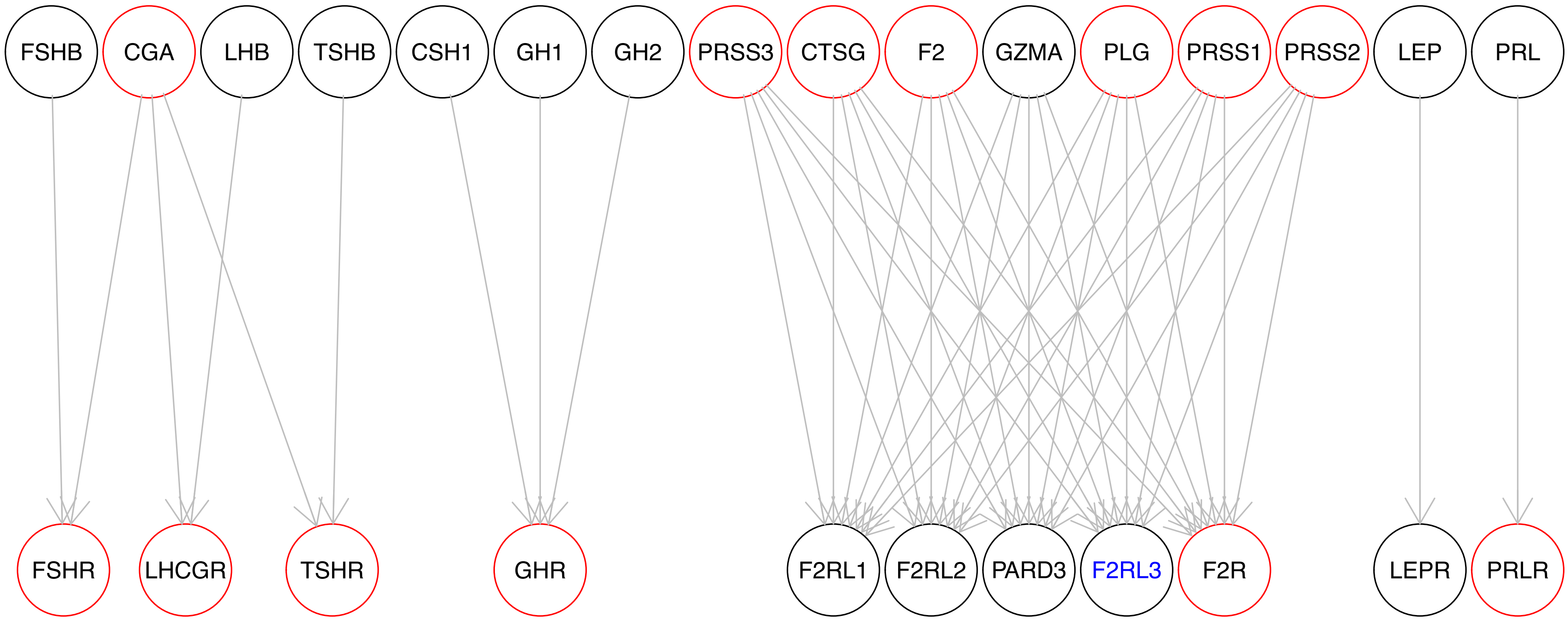

KEGG pathway = Neuroactive ligand−receptor interaction :   tumour = X09T :   Yellow Fill = gene variant, Blue Text = expression−survival association, Red Boarder = drug

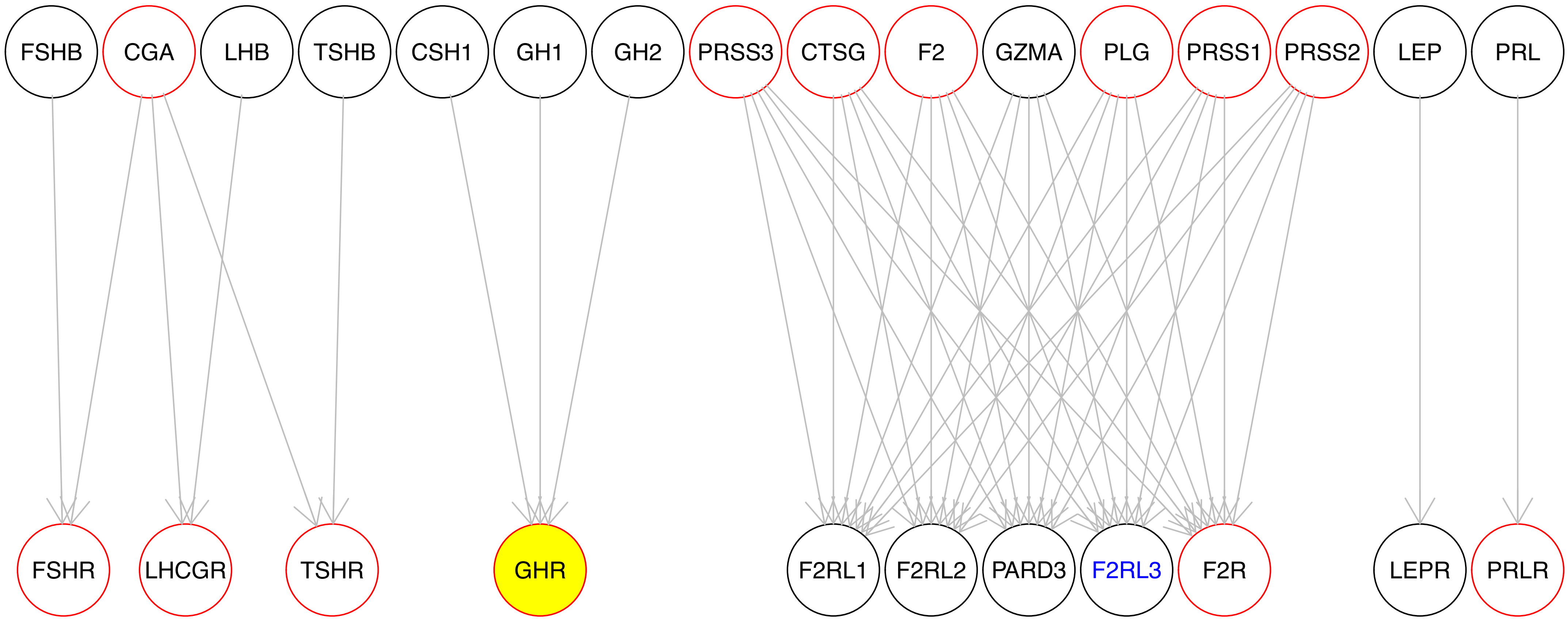

KEGG pathway = Neuroactive ligand−receptor interaction : tumour = X12T : Yellow Fill = gene variant, Blue Text = expression−survival association, Red Boarder = drug

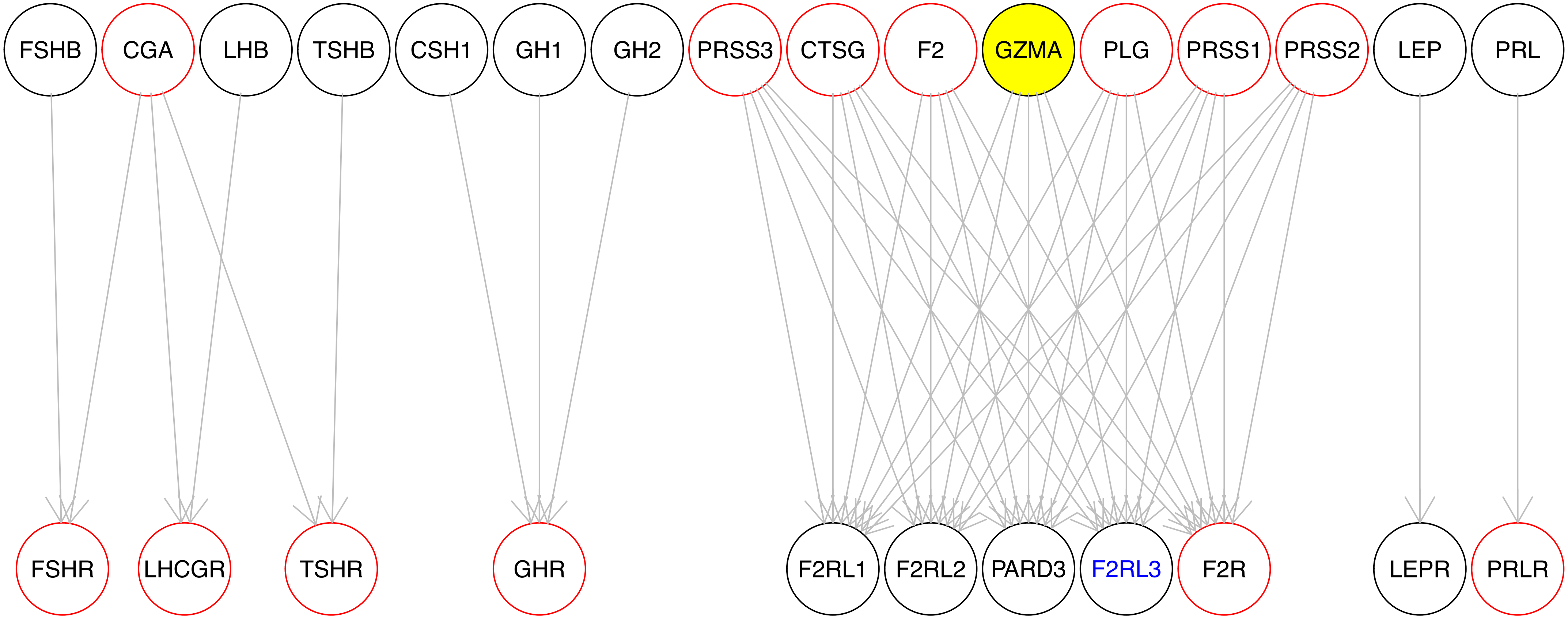

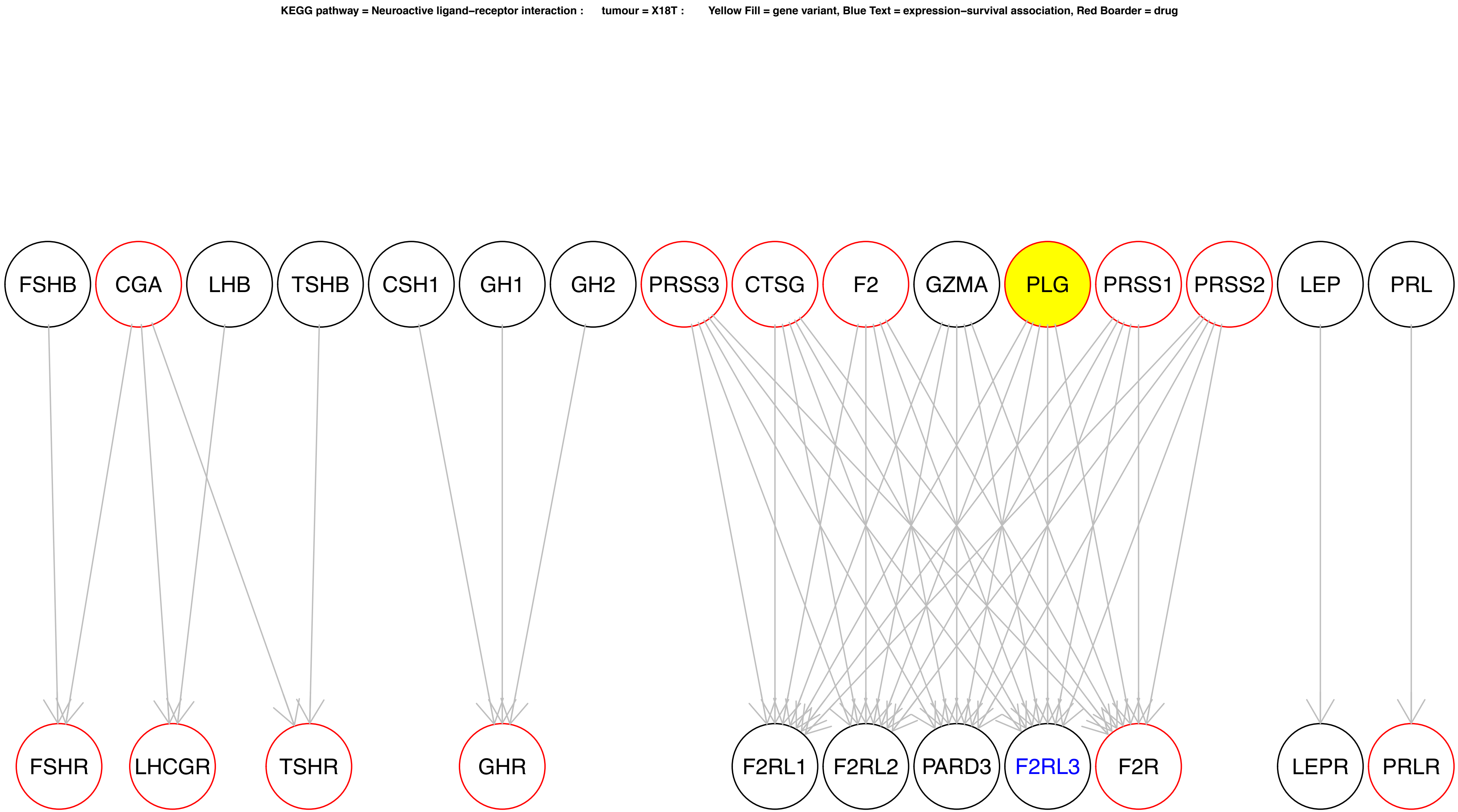
KEGG pathway = Neuroactive ligand−receptor interaction : tumour = X18T : Yellow Fill = gene variant, Blue Text = expression−survival association, Red Boarder = drug
FSHB
CGA
LHB
TSHB
CSH1
GH1
GH2
PRSS3
CTSG
F2
GZMA
PLG
PRSS1
PRSS2
LEP
PRL
FSHR
LHCGR
TSHR
GHR
F2RL1
F2RL2
PARD3
F2RL3
F2R
LEPR
PRLR

KEGG pathway = Neuroactive ligand−receptor interaction :   tumour = X22T :   Yellow Fill = gene variant, Blue Text = expression−survival association, Red Boarder = drug

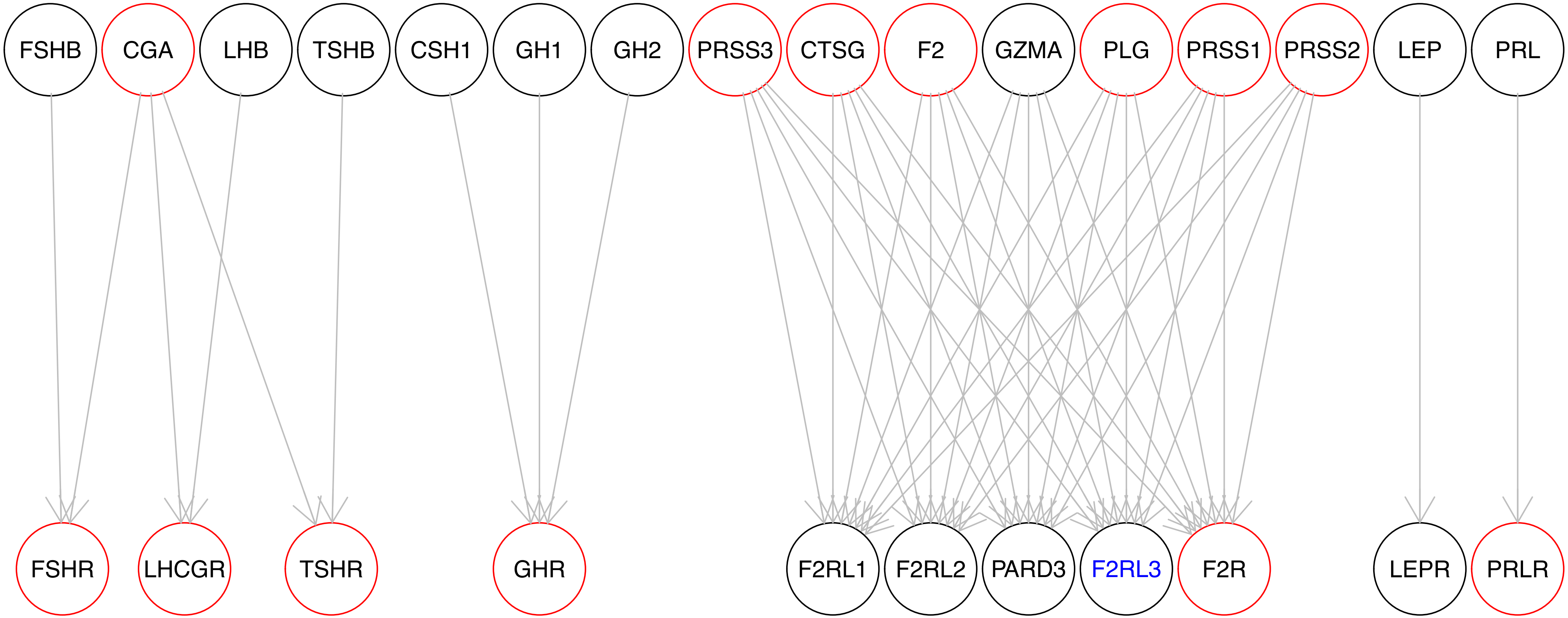

KEGG pathway = Neuroactive ligand−receptor interaction : tumour = X24T : Yellow Fill = gene variant, Blue Text = expression−survival association, Red Boarder = drug

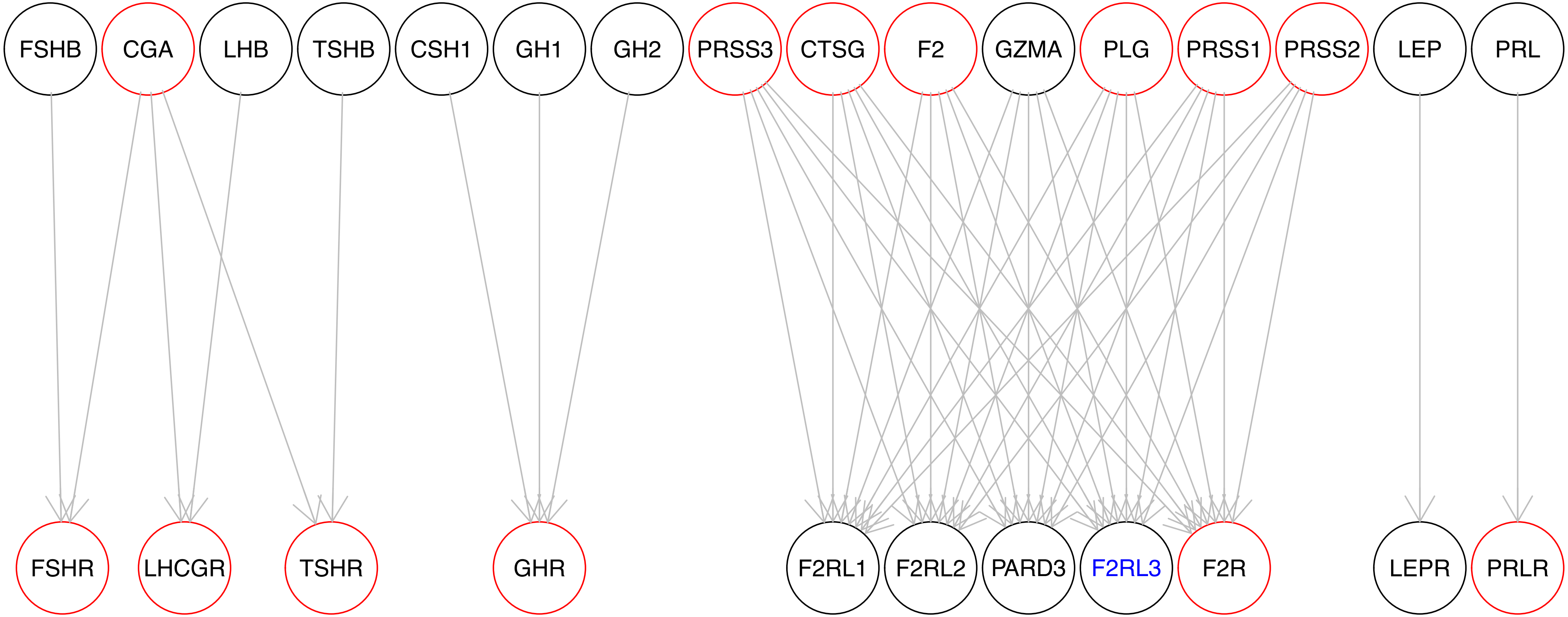

KEGG pathway = Neuroactive ligand−receptor interaction : tumour = X35T : Yellow Fill = gene variant, Blue Text = expression−survival association, Red Boarder = drug

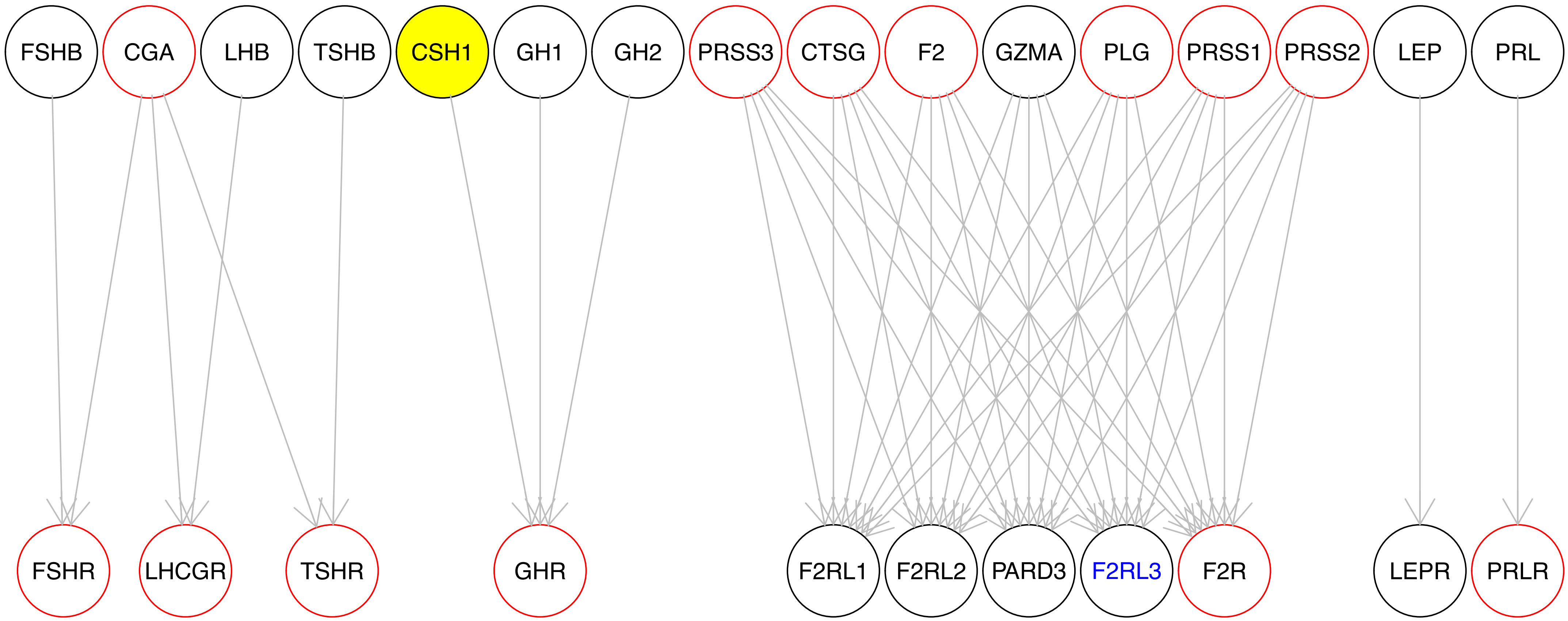

KEGG pathway = Neuroactive ligand−receptor interaction :   tumour = X43T :   Yellow Fill = gene variant, Blue Text = expression−survival association, Red Boarder = drug

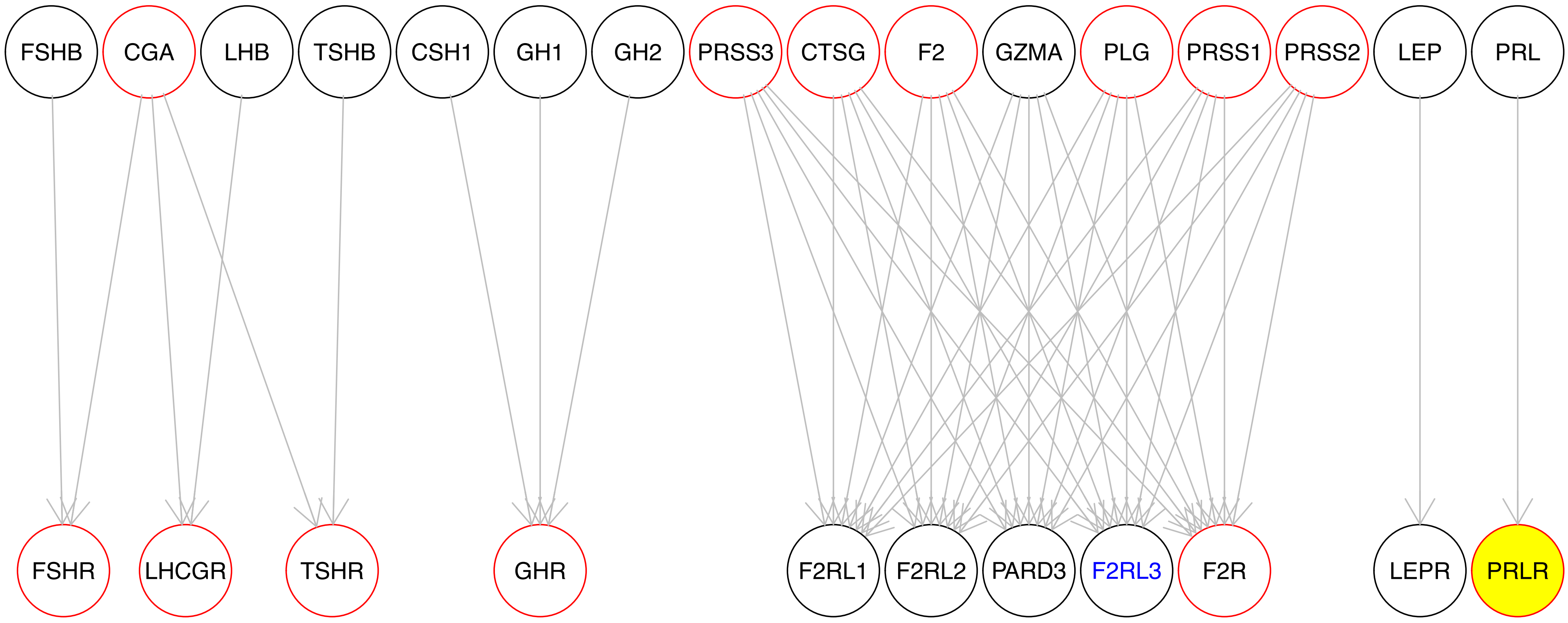

KEGG pathway = Neuroactive ligand−receptor interaction : tumour = X51T : Yellow Fill = gene variant, Blue Text = expression−survival association, Red Boarder = drug

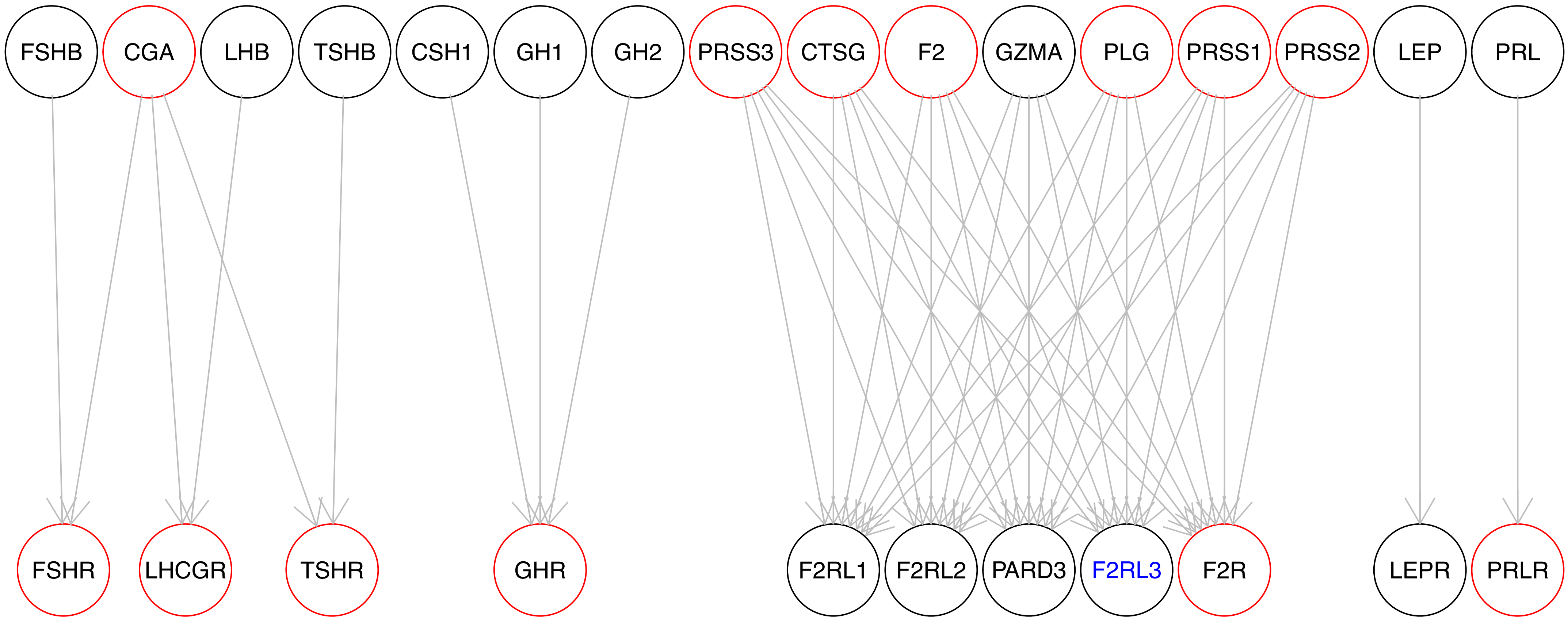

KEGG pathway = Neuroactive ligand−receptor interaction : tumour = X60T : Yellow Fill = gene variant, Blue Text = expression−survival association, Red Boarder = drug

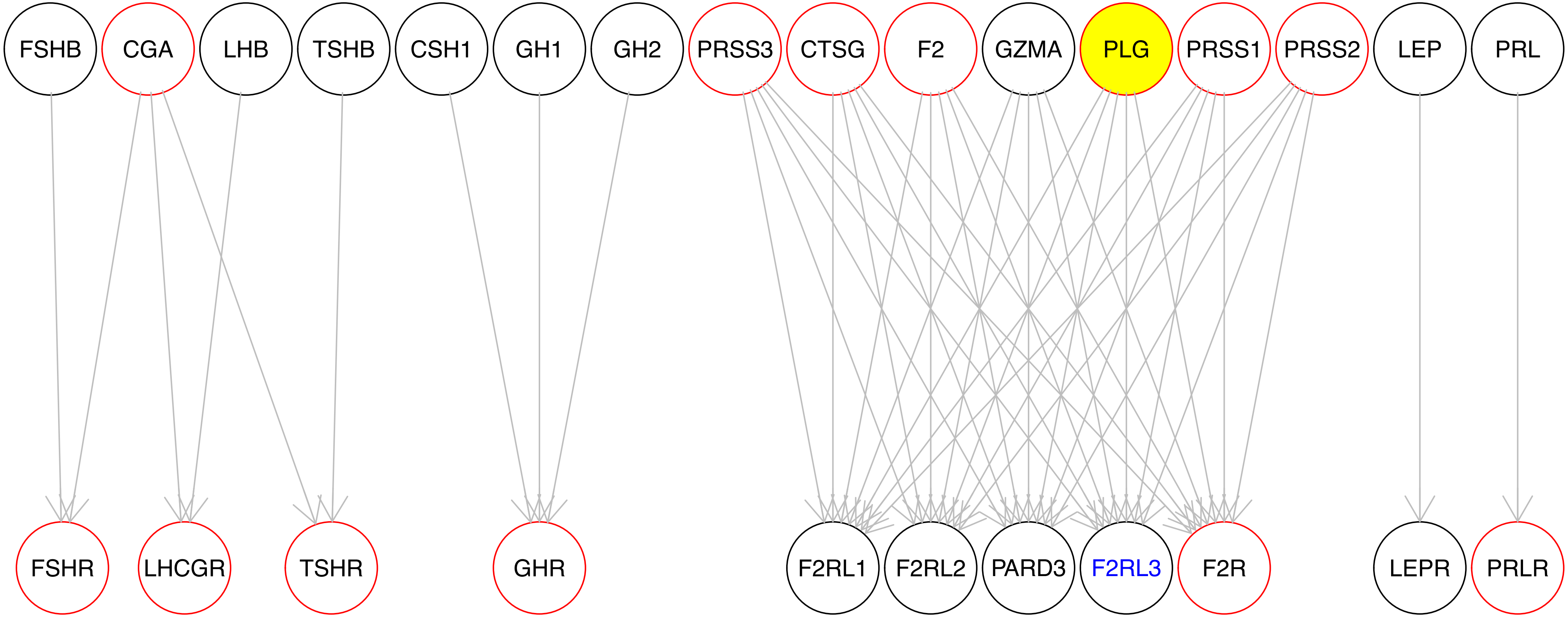

KEGG pathway = Neuroactive ligand−receptor interaction : tumour = X91T : Yellow Fill = gene variant, Blue Text = expression−survival association, Red Boarder = drug

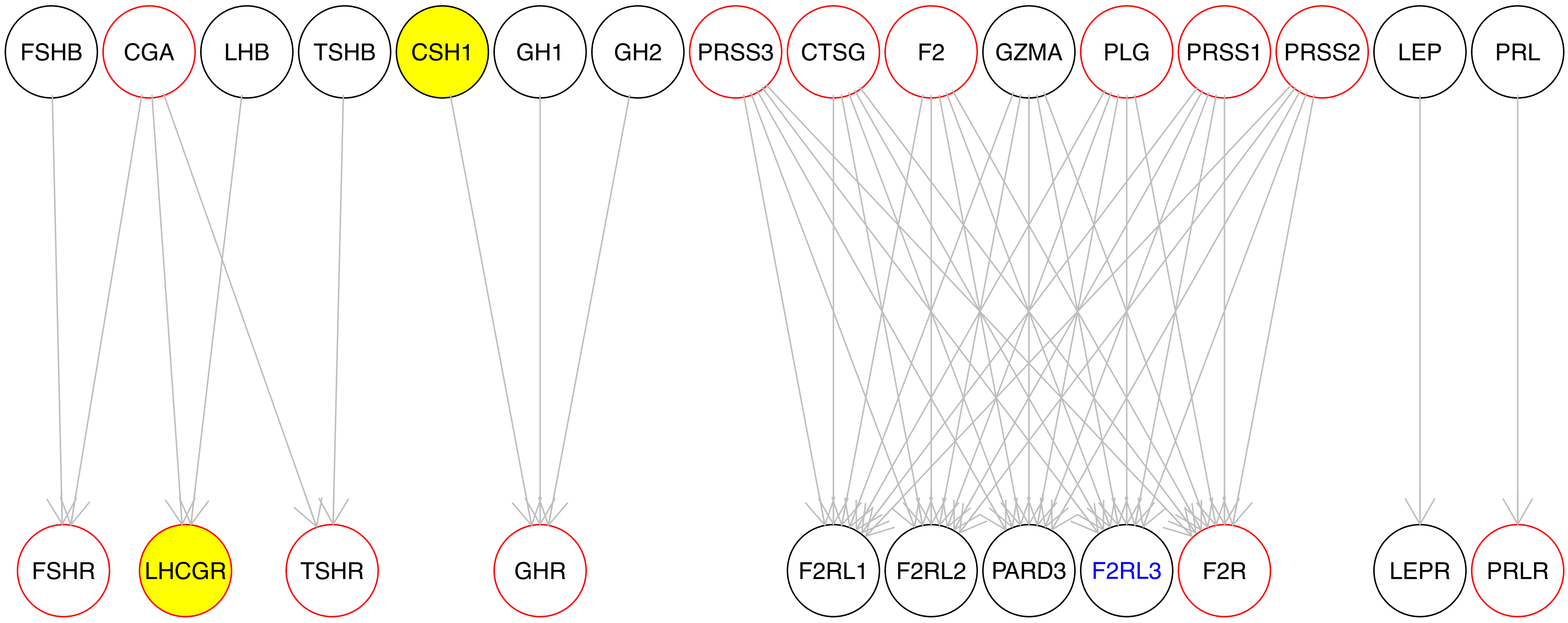

KEGG pathway = Neuroactive ligand−receptor interaction :   tumour = X93T :   Yellow Fill = gene variant, Blue Text = expression−survival association, Red Boarder = drug

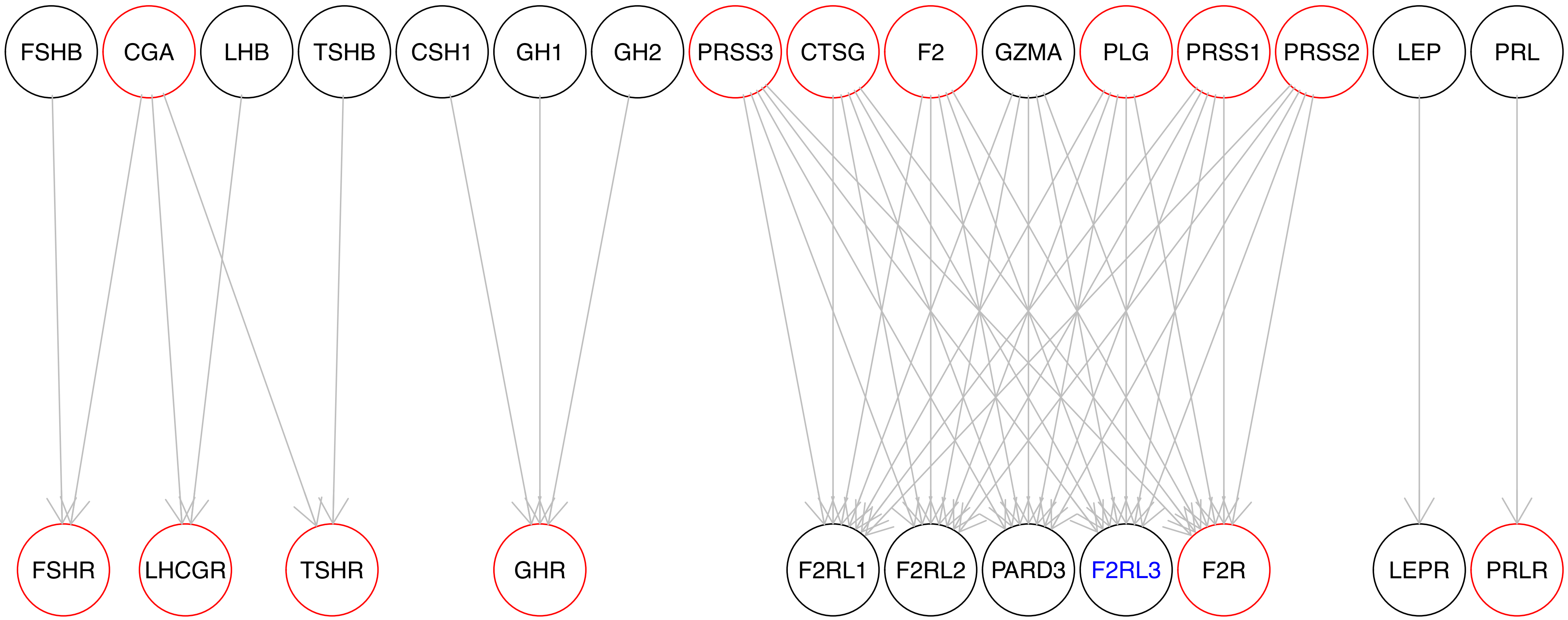

KEGG pathway = Neuroactive ligand−receptor interaction : tumour = X96T : Yellow Fill = gene variant, Blue Text = expression−survival association, Red Boarder = drug

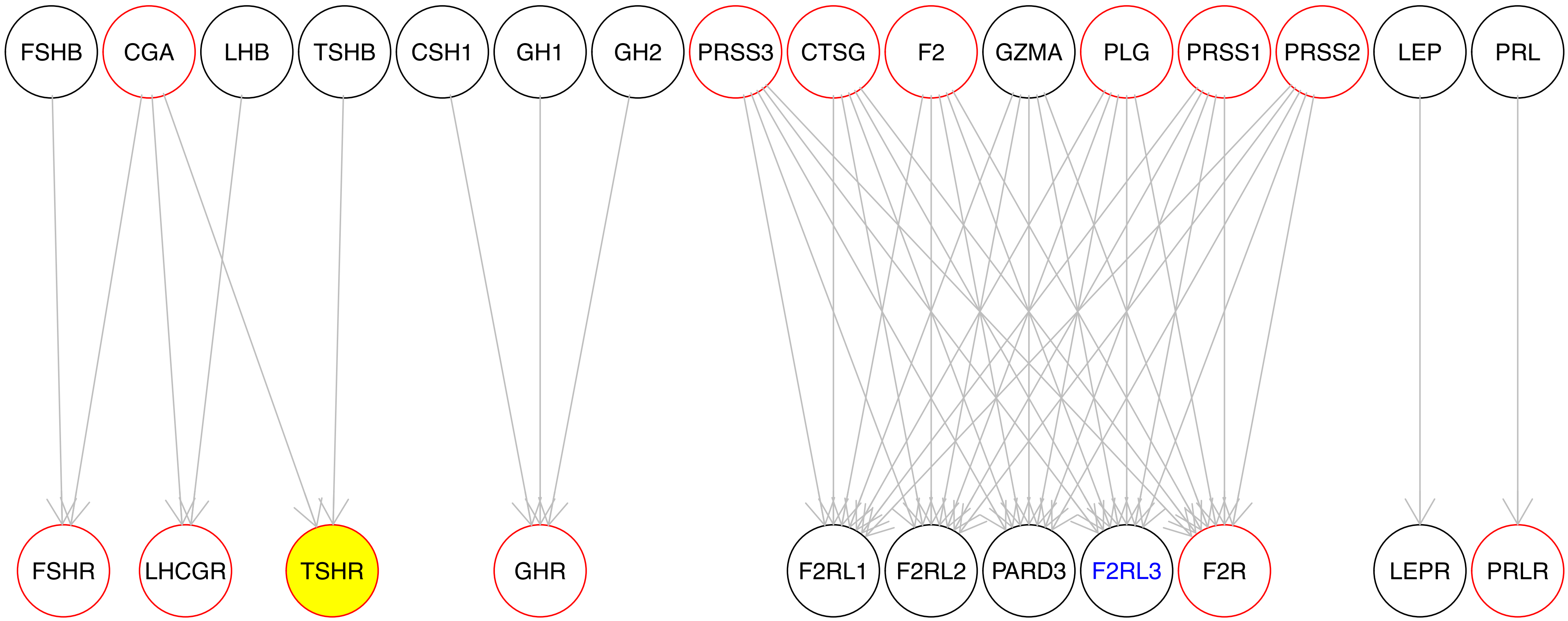

KEGG pathway = Neuroactive ligand−receptor interaction :   tumour = ME010.Tumor :   Yellow Fill = gene variant, Blue Text = expression−survival association, Red Boarder = drug

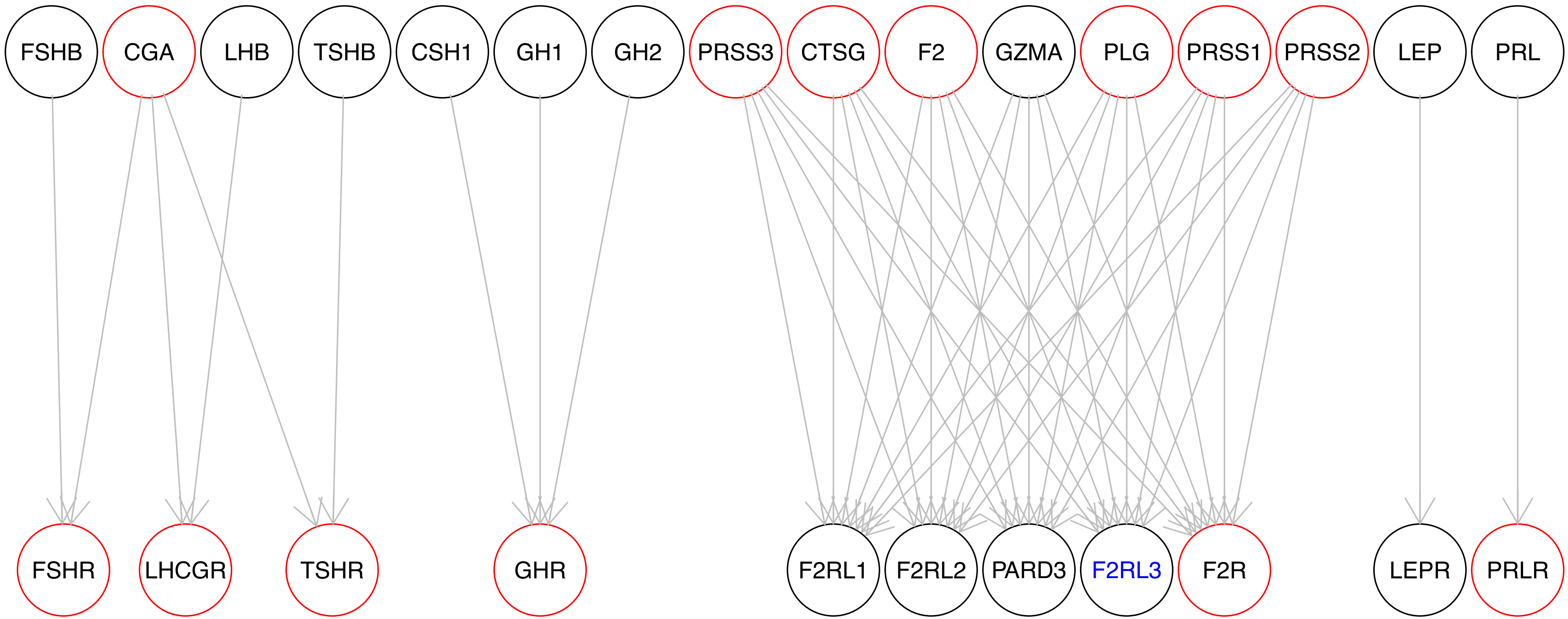

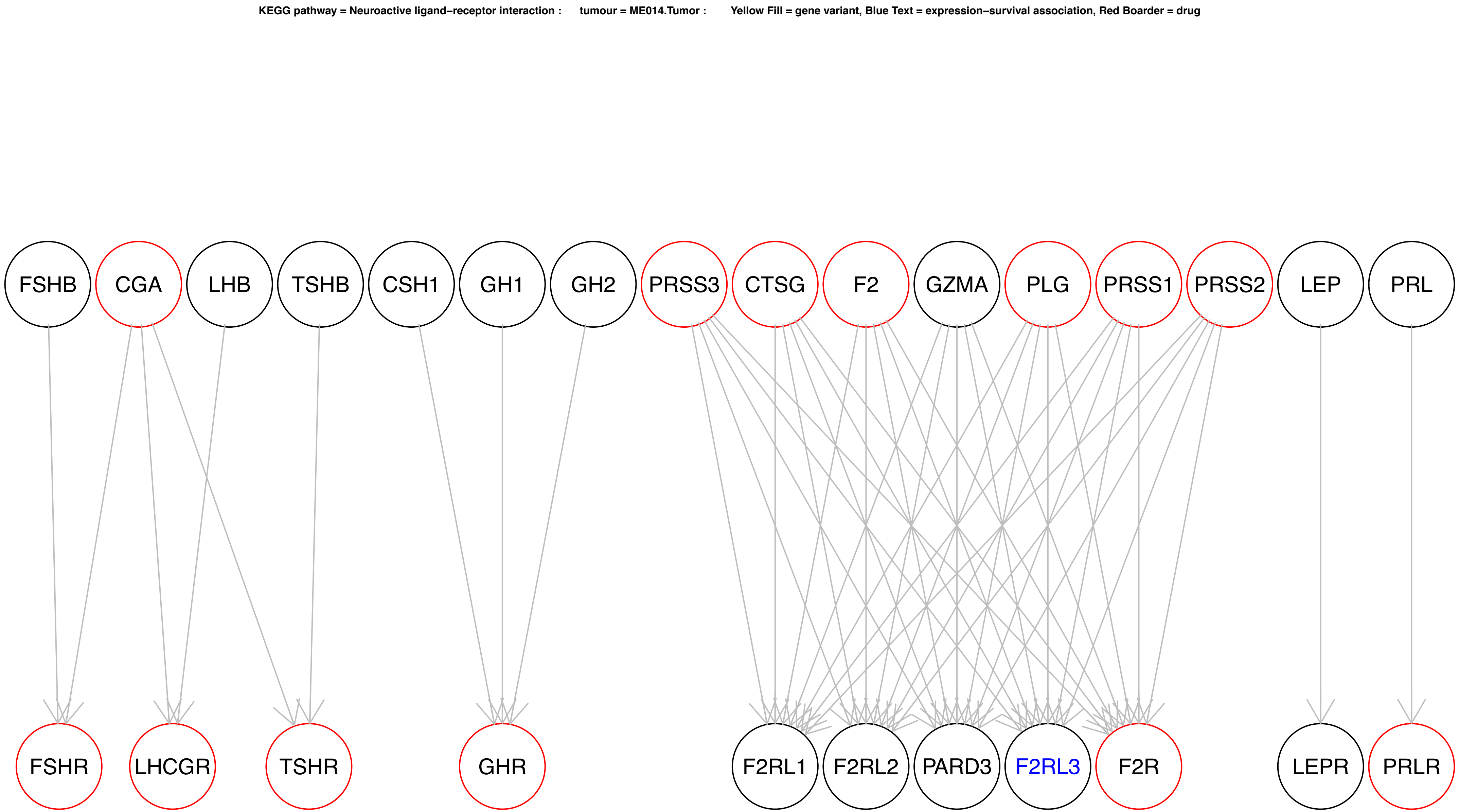
KEGG pathway = Neuroactive ligand−receptor interaction : tumour = ME014.Tumor : Yellow Fill = gene variant, Blue Text = expression−survival association, Red Boarder = drug
FSHB
CGA
LHB
TSHB
CSH1
GH1
GH2
PRSS3
CTSG
F2
GZMA
PLG
PRSS1
PRSS2
LEP
PRL
FSHR
LHCGR
TSHR
GHR
F2RL1
F2RL2
PARD3
F2RL3
F2R
LEPR
PRLR

KEGG pathway = Neuroactive ligand−receptor interaction : tumour = ME017.Tumor : Yellow Fill = gene variant, Blue Text = expression−survival association, Red Boarder = drug

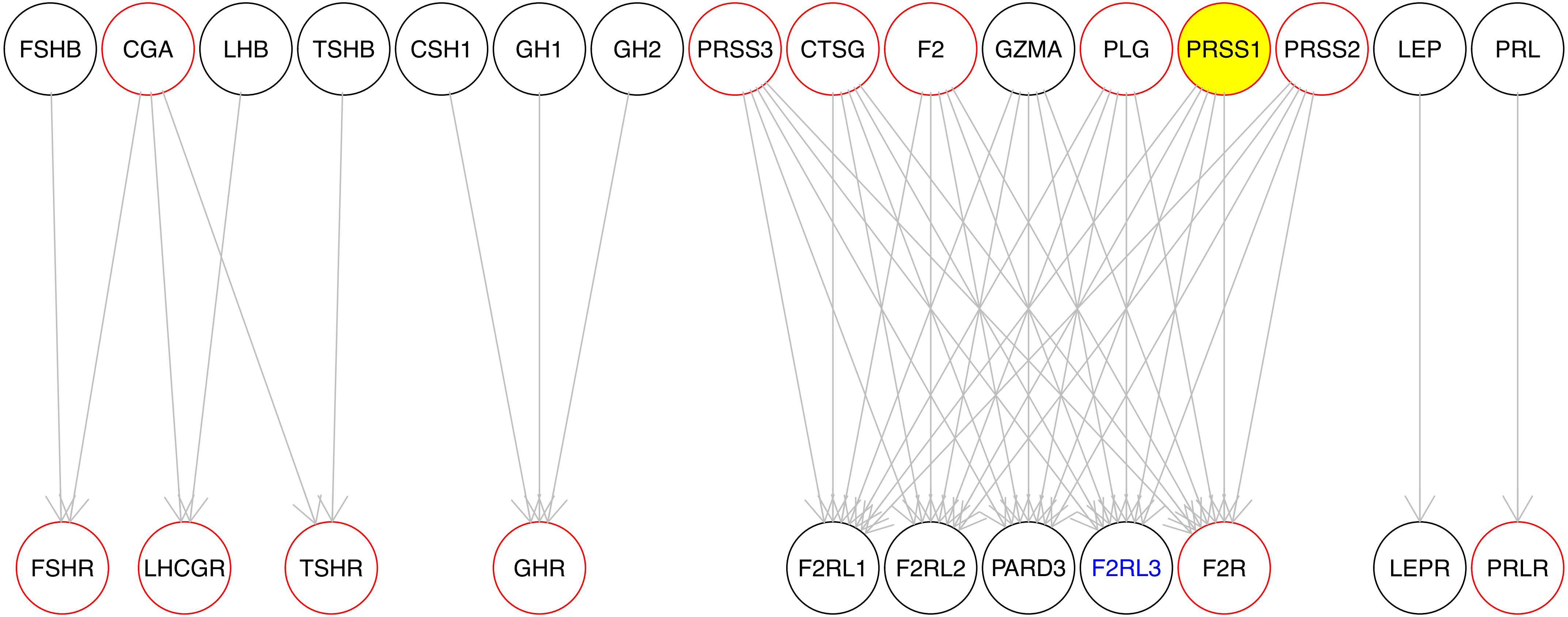

KEGG pathway = Neuroactive ligand−receptor interaction : tumour = ME033.Tumor : Yellow Fill = gene variant, Blue Text = expression−survival association, Red Boarder = drug

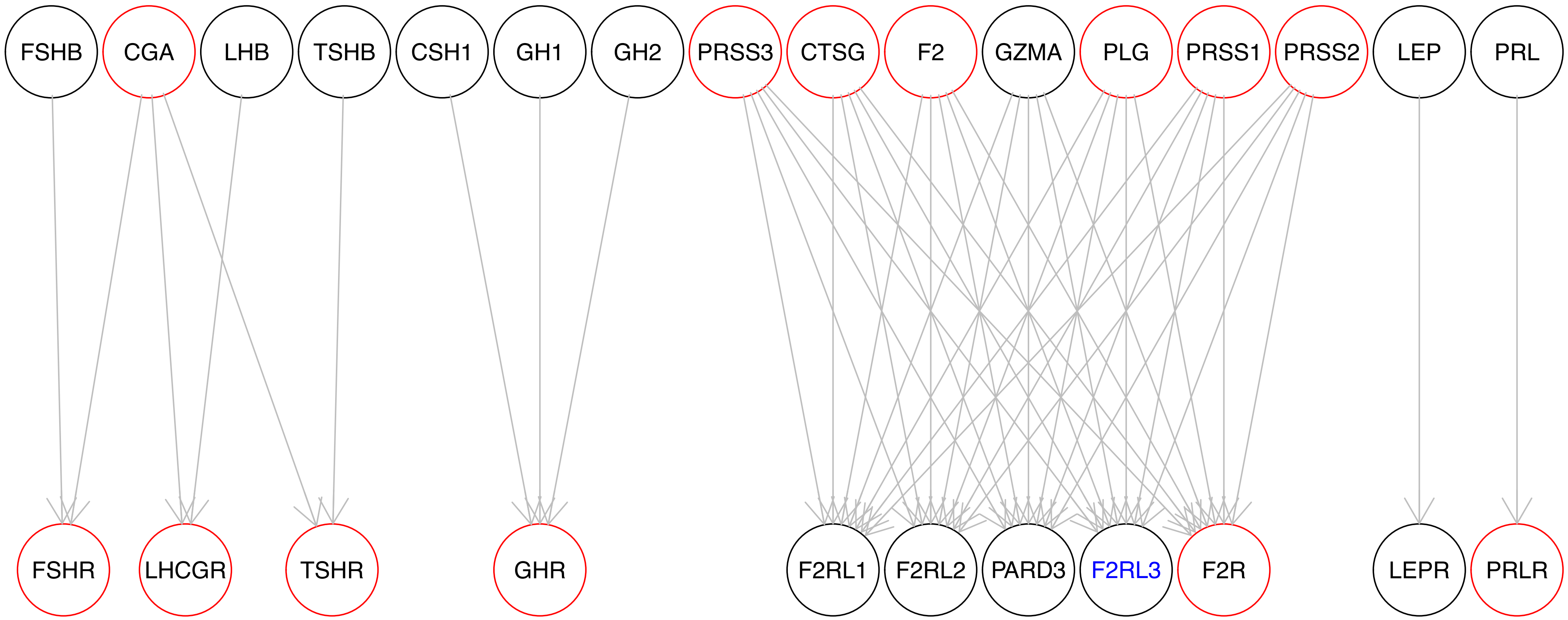

KEGG pathway = Neuroactive ligand−receptor interaction : tumour = ME034.Tumor : Yellow Fill = gene variant, Blue Text = expression−survival association, Red Boarder = drug

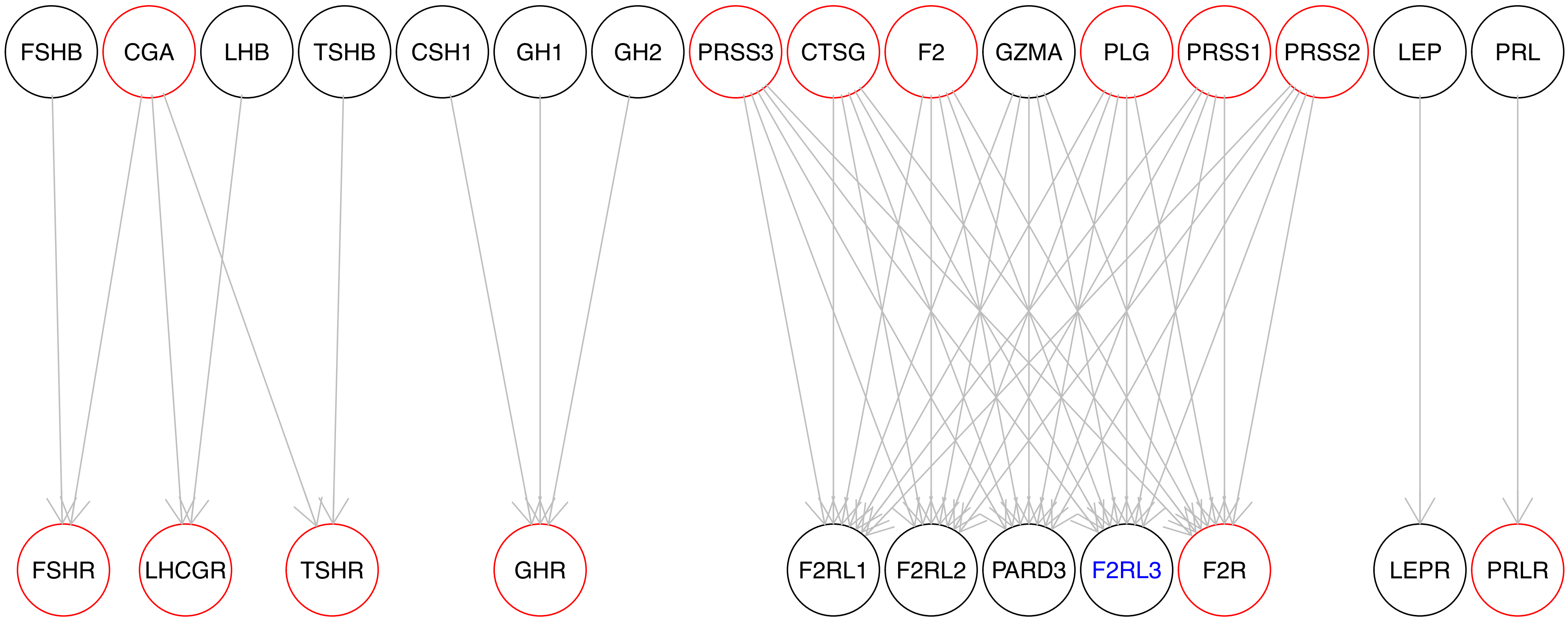

KEGG pathway = Neuroactive ligand−receptor interaction :   tumour = Mel_BRAFi_02_PRE.Tumor :   Yellow Fill = gene variant, Blue Text = expression−survival association, Red Boarder = drug

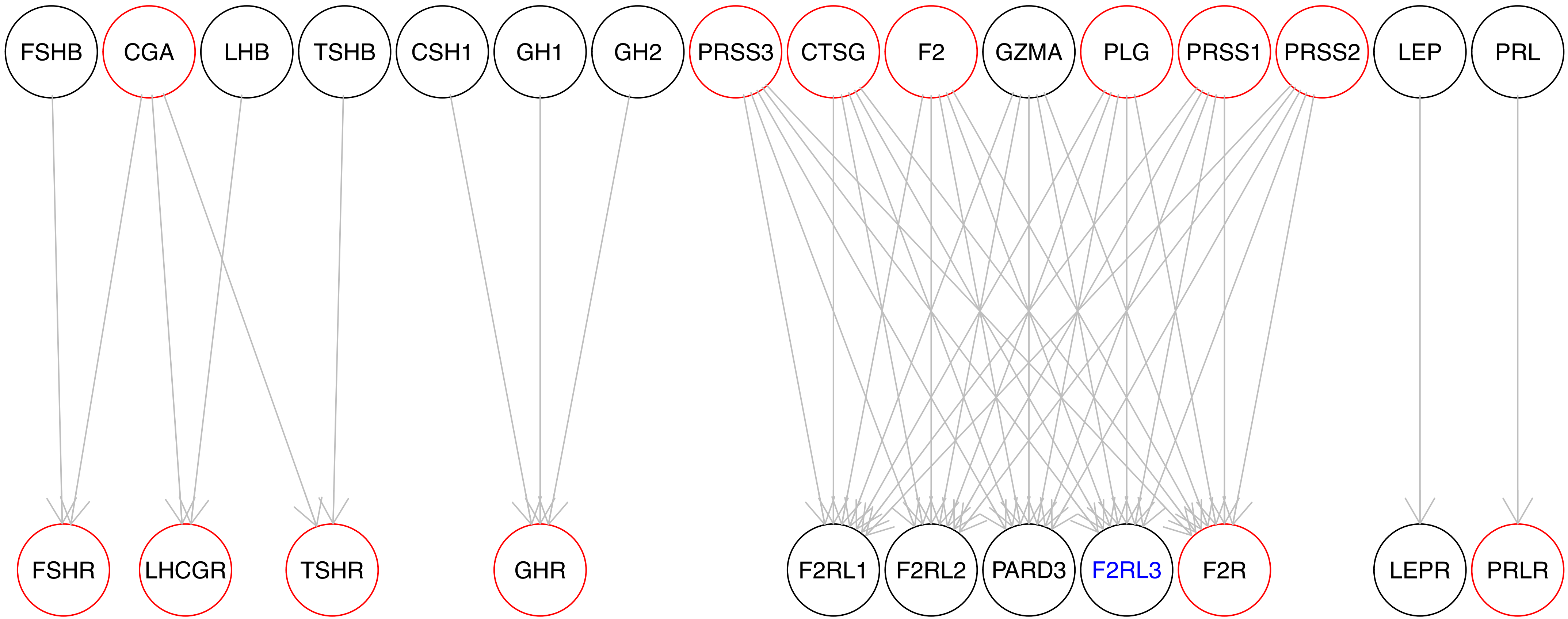

KEGG pathway = Neuroactive ligand−receptor interaction :   tumour = MEL.13447.Tumor :   Yellow Fill = gene variant, Blue Text = expression−survival association, Red Boarder = drug

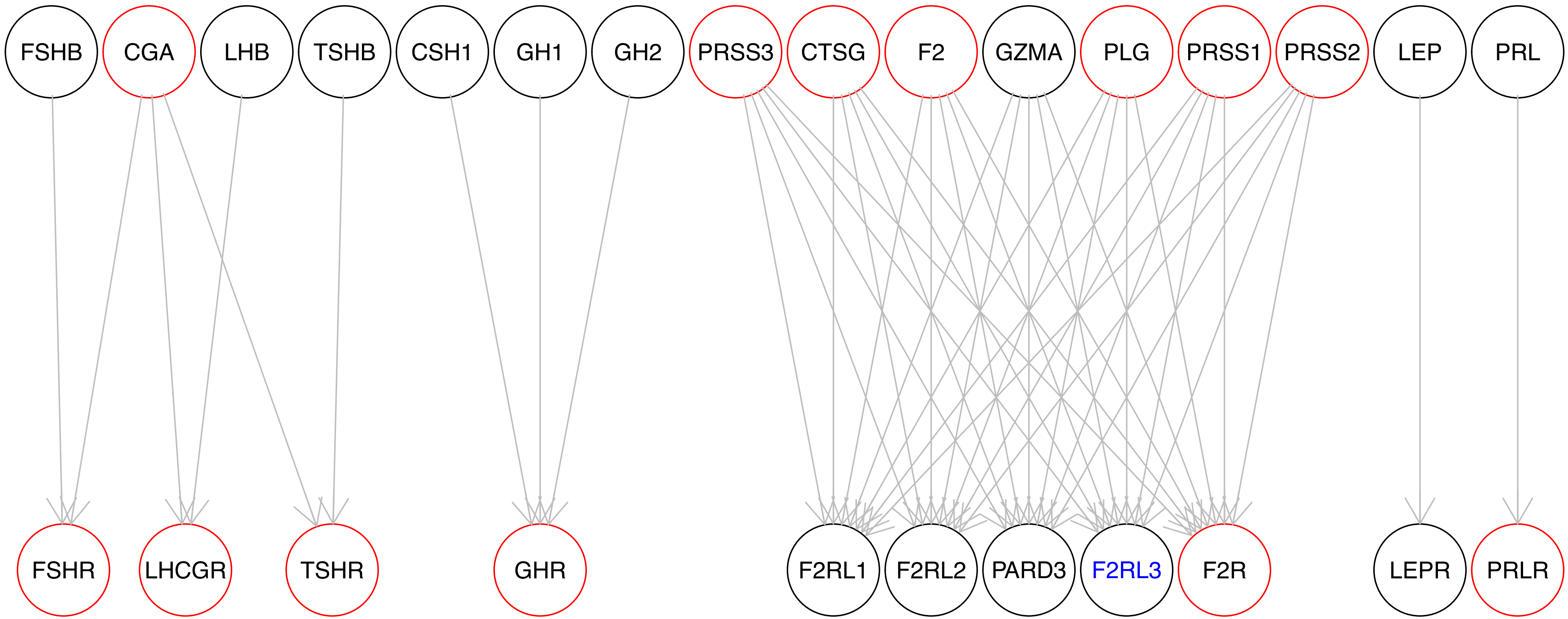

KEGG pathway = Neuroactive ligand−receptor interaction :   tumour = MEL.13456.Tumor :   Yellow Fill = gene variant, Blue Text = expression−survival association, Red Boarder = drug

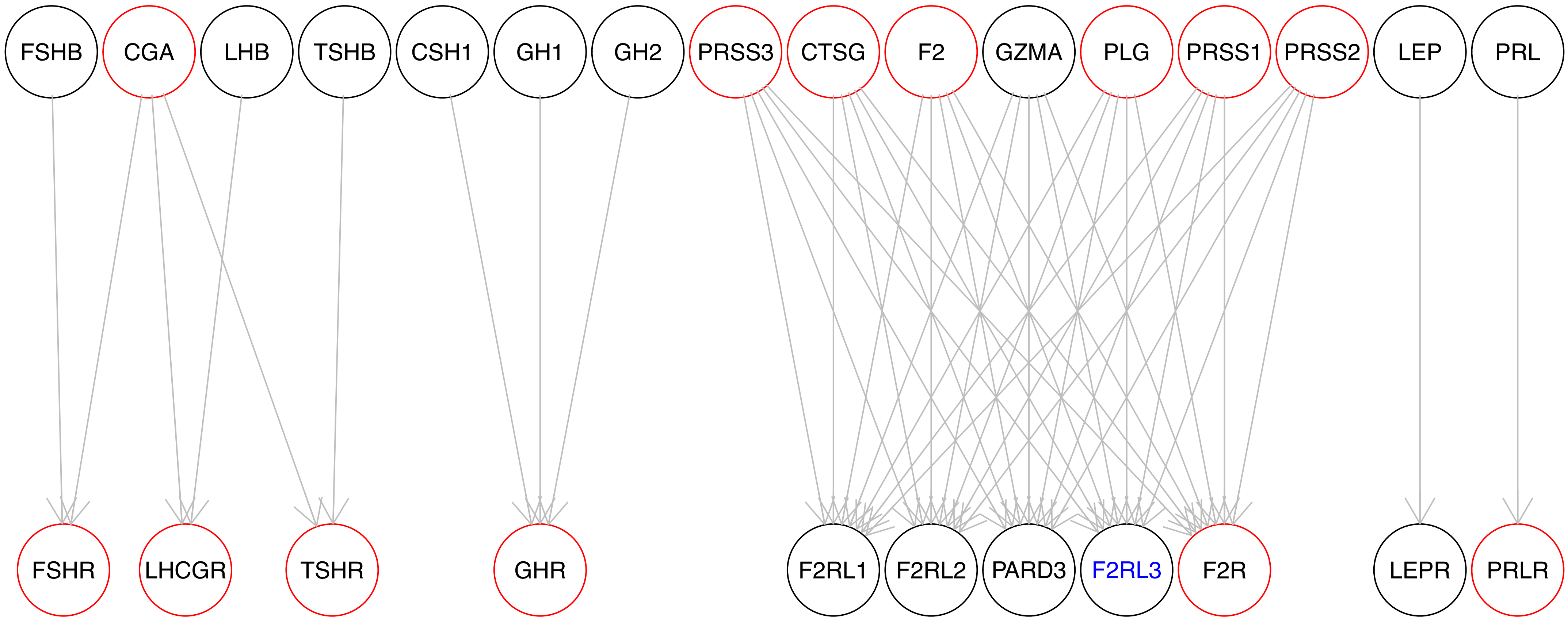

KEGG pathway = Neuroactive ligand−receptor interaction : tumour = MEL.13463.Tumor : Yellow Fill = gene variant, Blue Text = expression−survival association, Red Boarder = drug

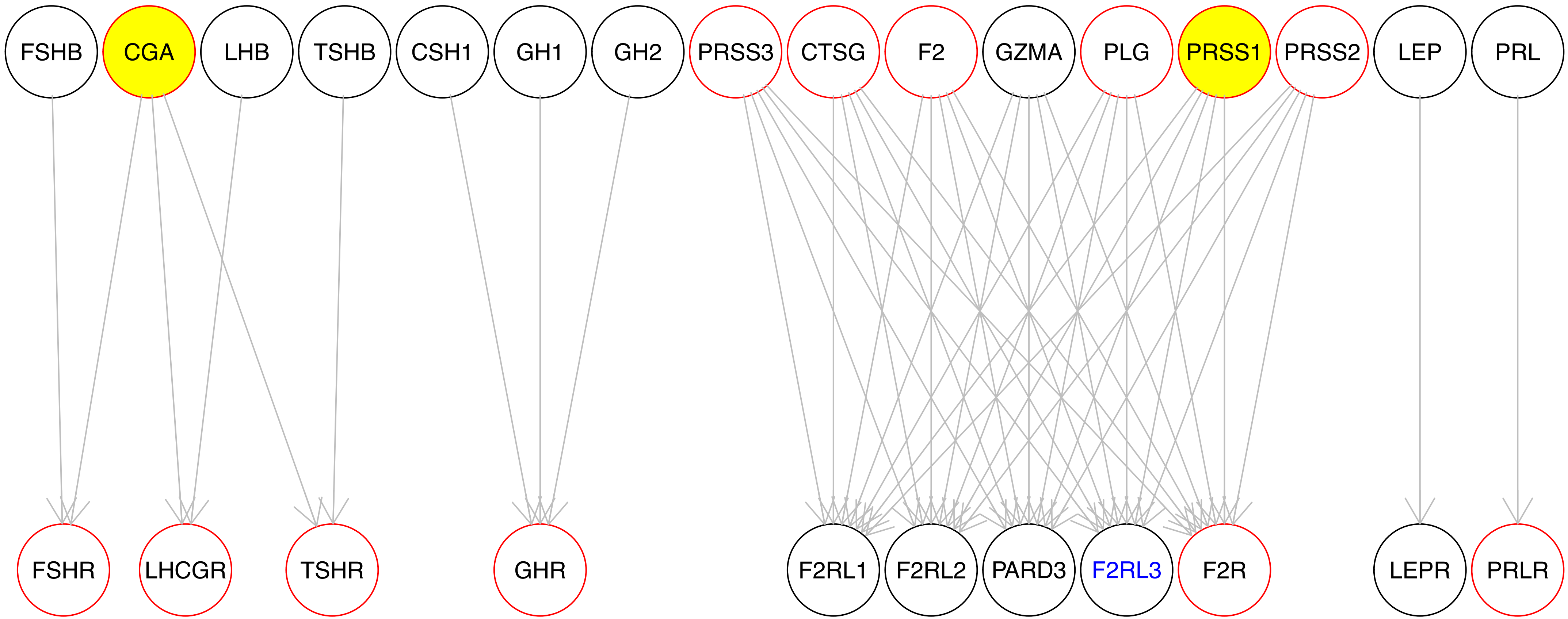

KEGG pathway = Neuroactive ligand−receptor interaction : tumour = MEL.13468.Tumor : Yellow Fill = gene variant, Blue Text = expression−survival association, Red Boarder = drug

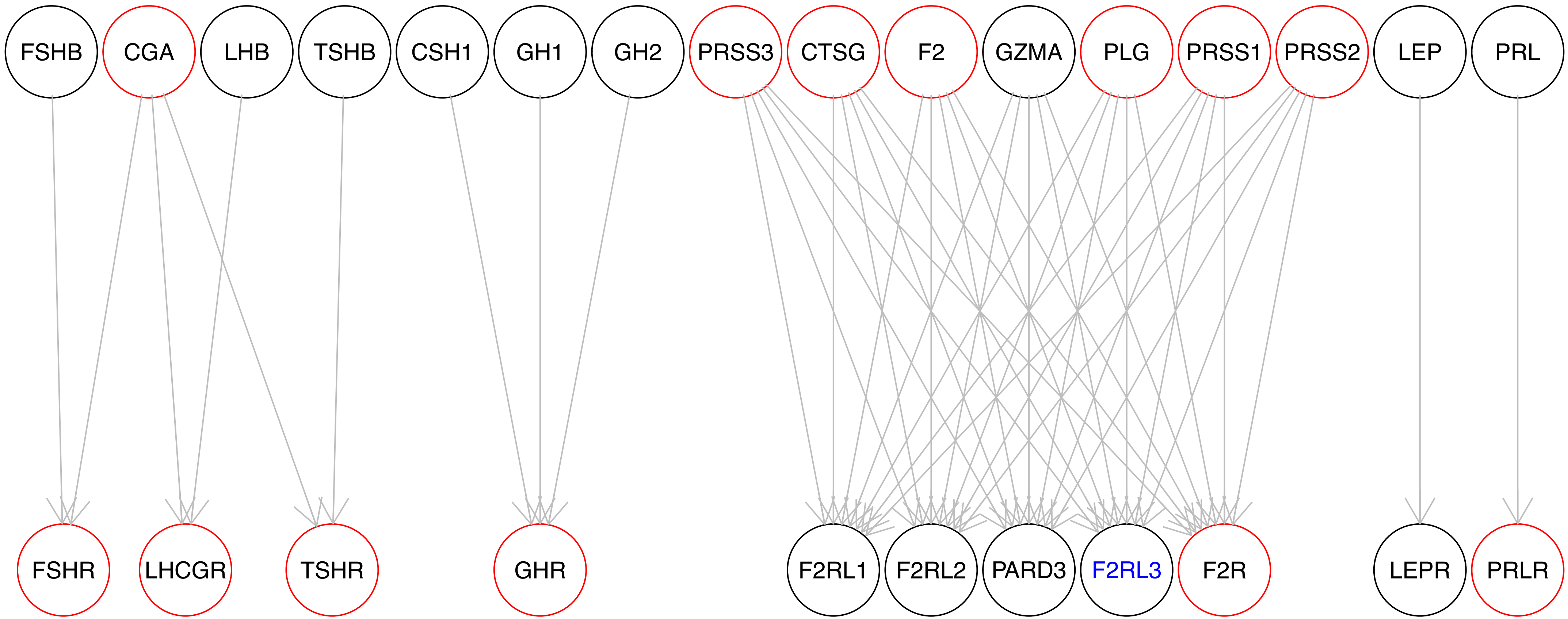

KEGG pathway = Neuroactive ligand−receptor interaction : tumour = MEL.13473.Tumor : Yellow Fill = gene variant, Blue Text = expression−survival association, Red Boarder = drug

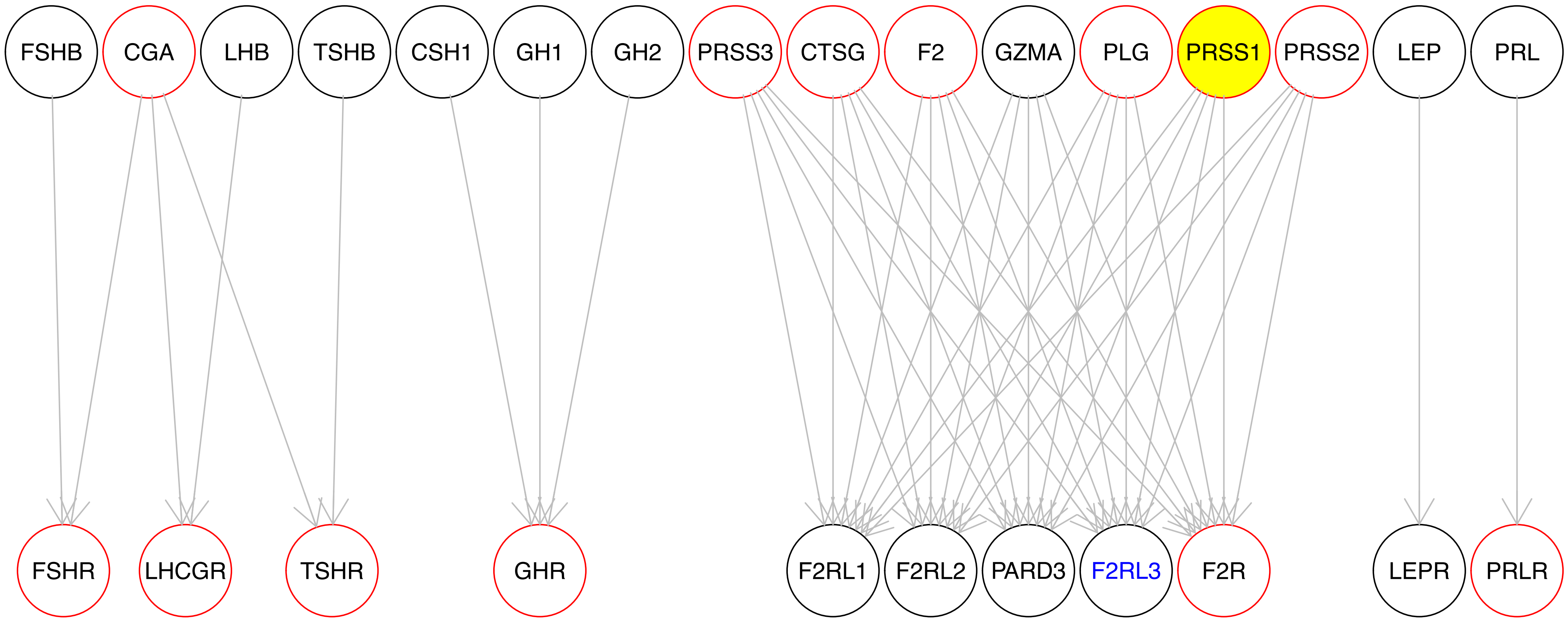

KEGG pathway = Neuroactive ligand−receptor interaction : tumour = MEL.13531.Tumor : Yellow Fill = gene variant, Blue Text = expression−survival association, Red Boarder = drug

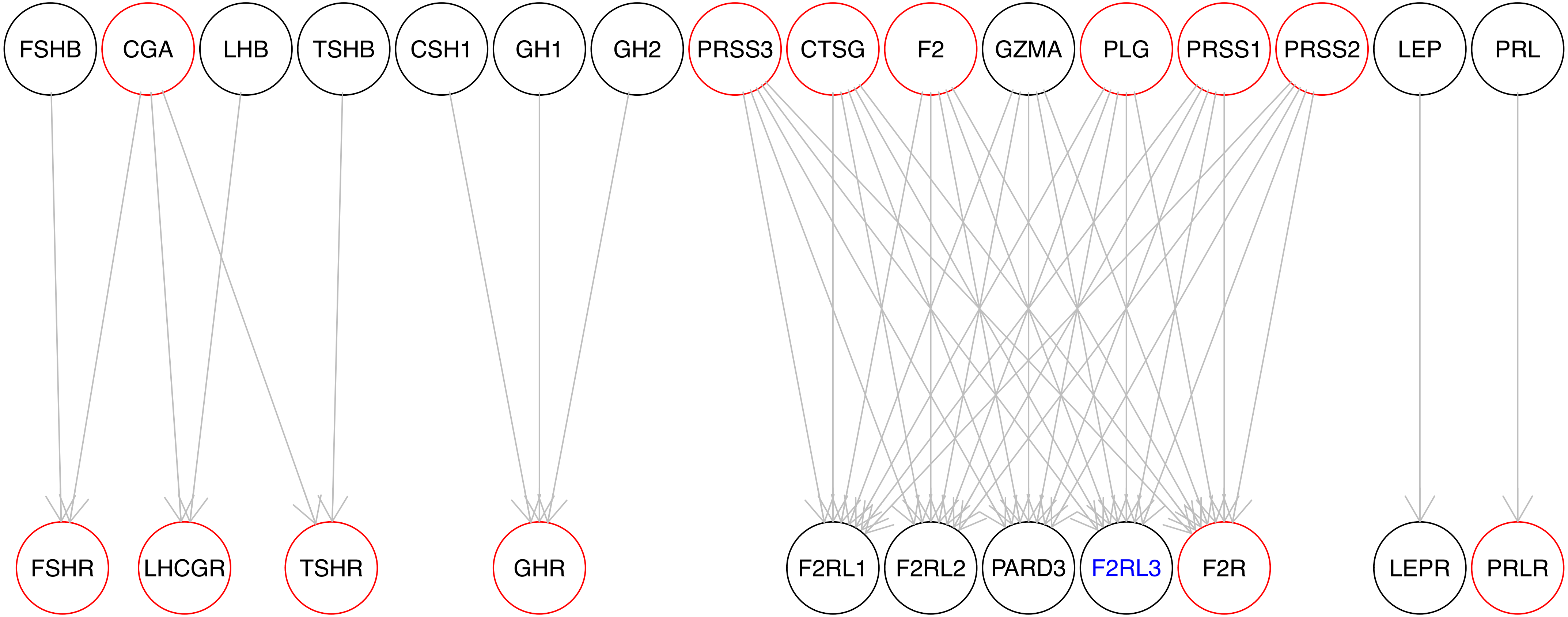

KEGG pathway = Neuroactive ligand−receptor interaction : tumour = MEL.13537.Tumor : Yellow Fill = gene variant, Blue Text = expression−survival association, Red Boarder = drug

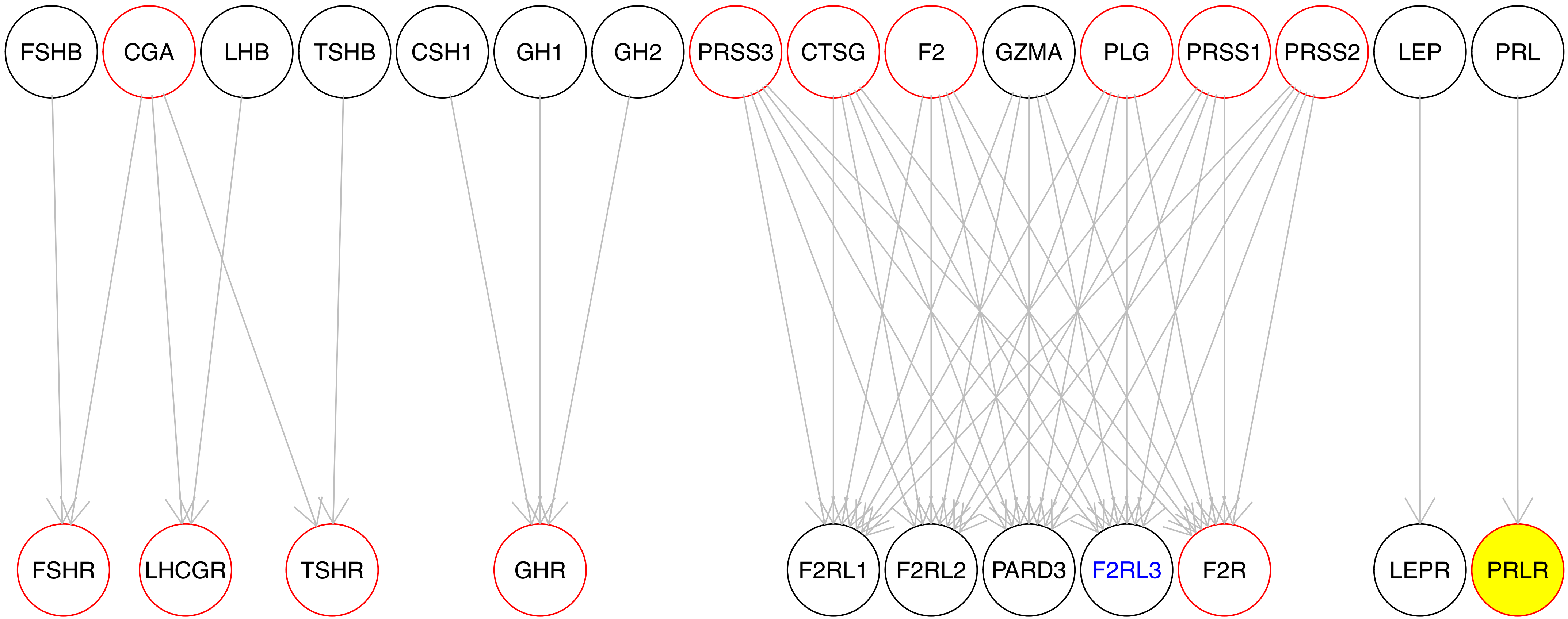

KEGG pathway = Neuroactive ligand−receptor interaction : tumour = MEL.13543.Tumor : Yellow Fill = gene variant, Blue Text = expression−survival association, Red Boarder = drug

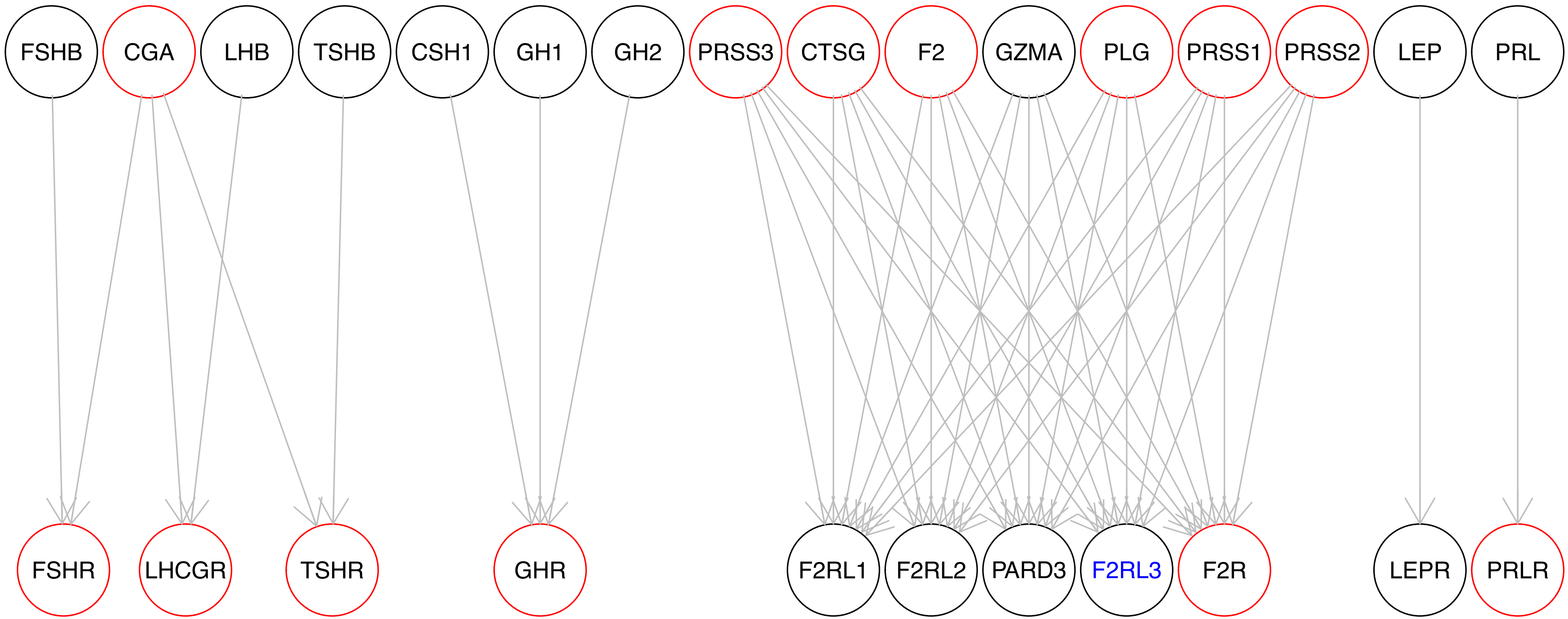

KEGG pathway = Neuroactive ligand−receptor interaction :   tumour = MEL.13549.Tumor :   Yellow Fill = gene variant, Blue Text = expression−survival association, Red Boarder = drug

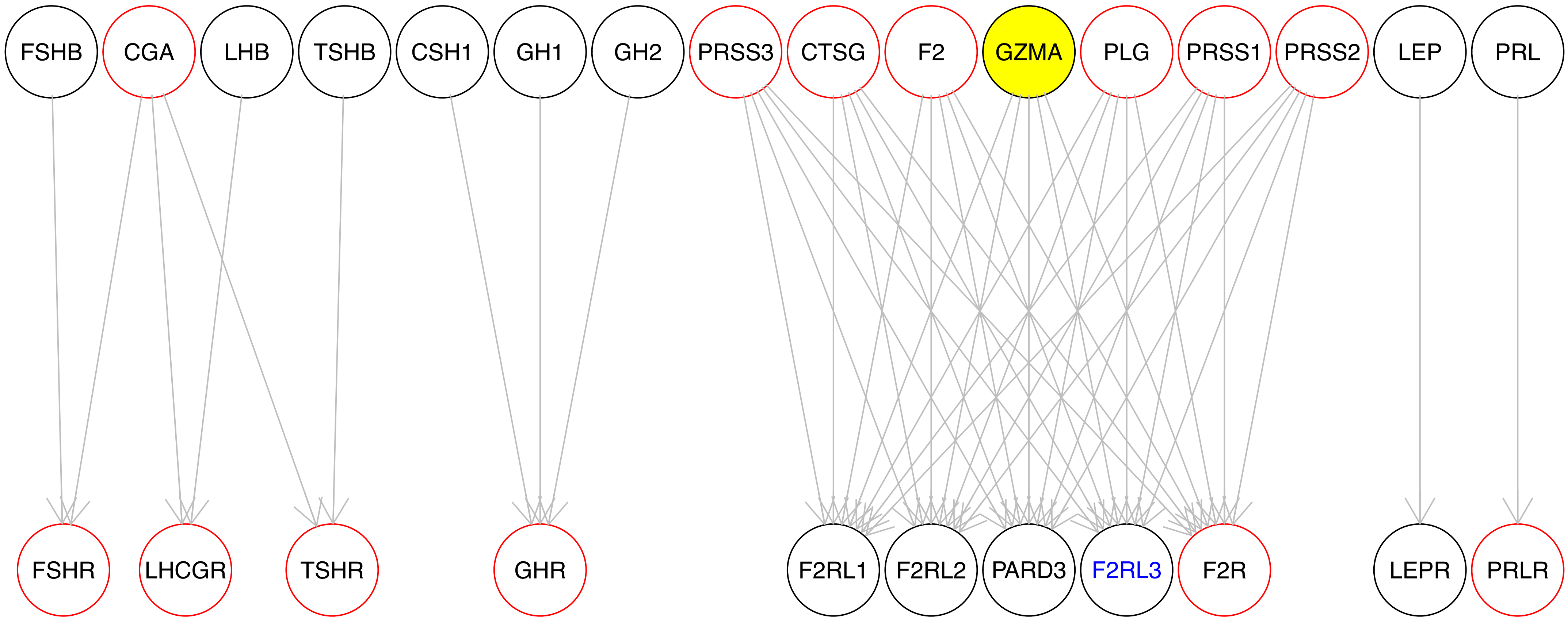

KEGG pathway = Neuroactive ligand−receptor interaction : tumour = MEL.13560.Tumor : Yellow Fill = gene variant, Blue Text = expression−survival association, Red Boarder = drug

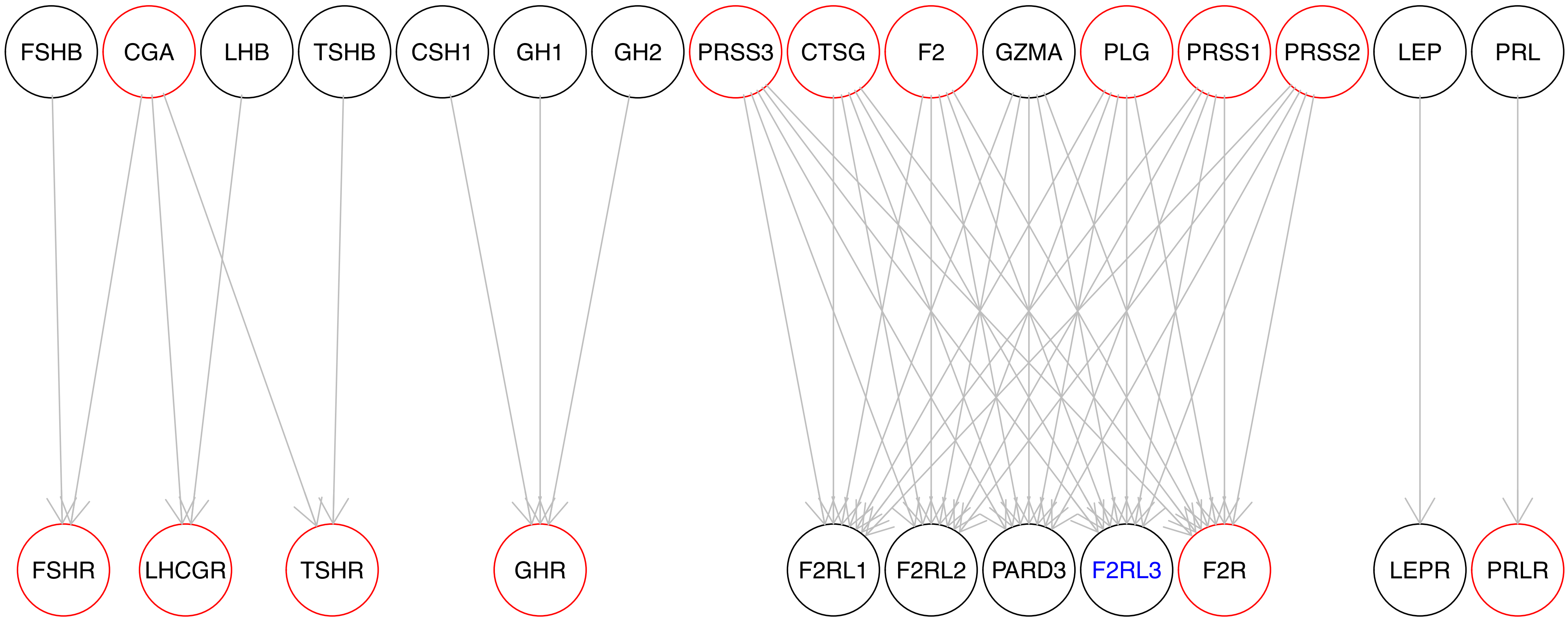

KEGG pathway = Neuroactive ligand−receptor interaction : tumour = MEL.13561.Tumor : Yellow Fill = gene variant, Blue Text = expression−survival association, Red Boarder = drug

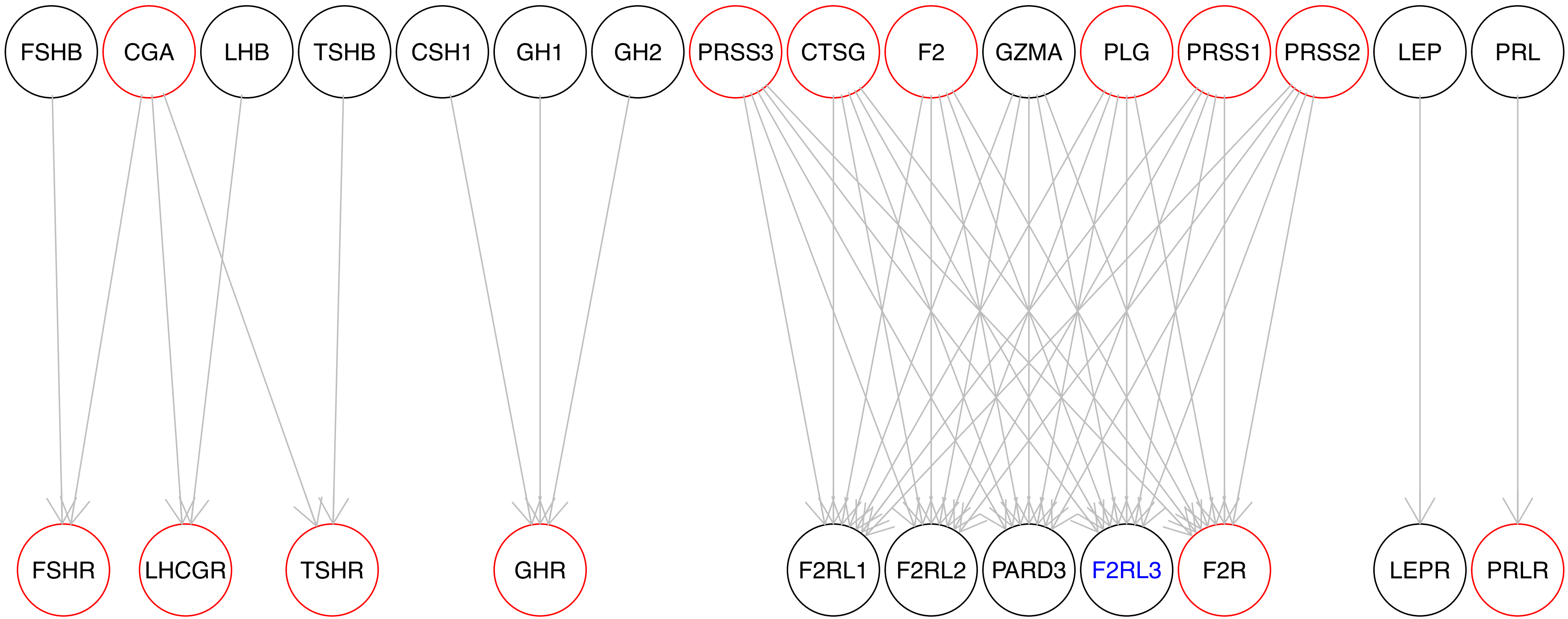

KEGG pathway = Neuroactive ligand−receptor interaction : tumour = MEL.13567.Tumor : Yellow Fill = gene variant, Blue Text = expression−survival association, Red Boarder = drug

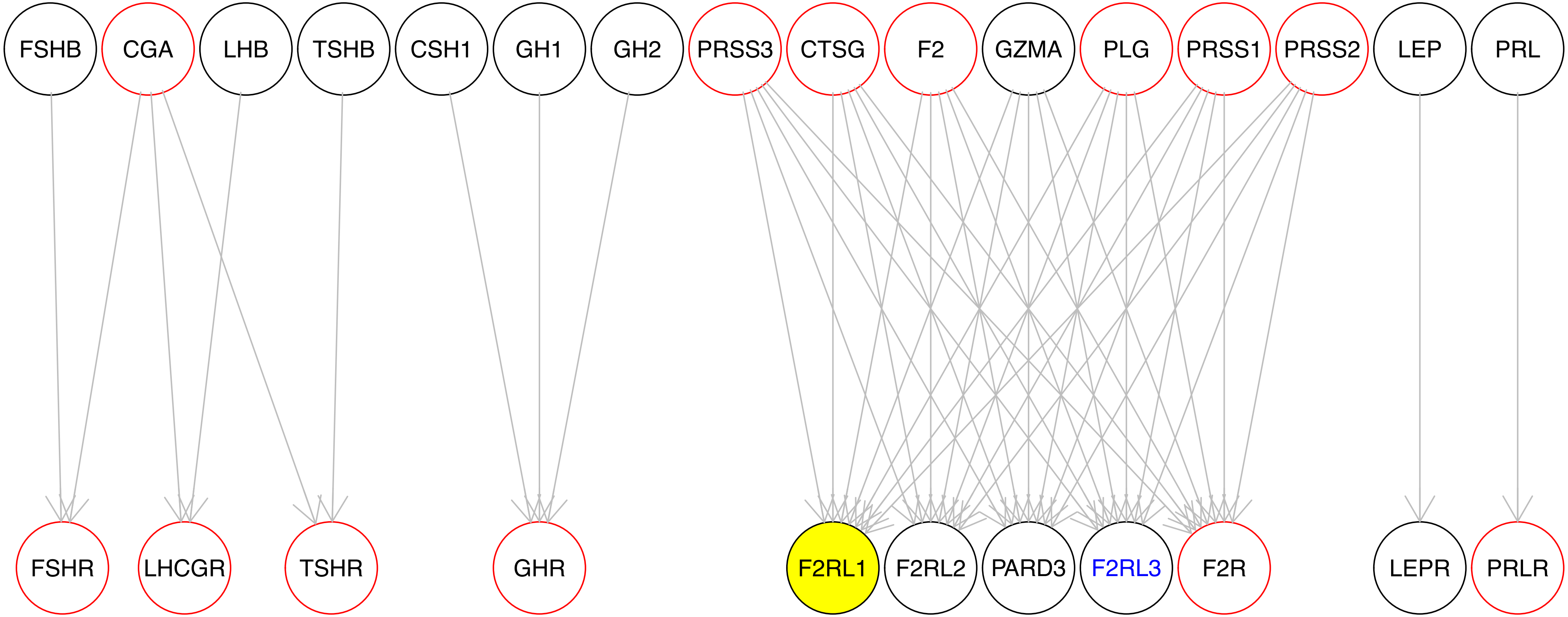

KEGG pathway = Neuroactive ligand−receptor interaction : tumour = MEL.13575.Tumor : Yellow Fill = gene variant, Blue Text = expression−survival association, Red Boarder = drug

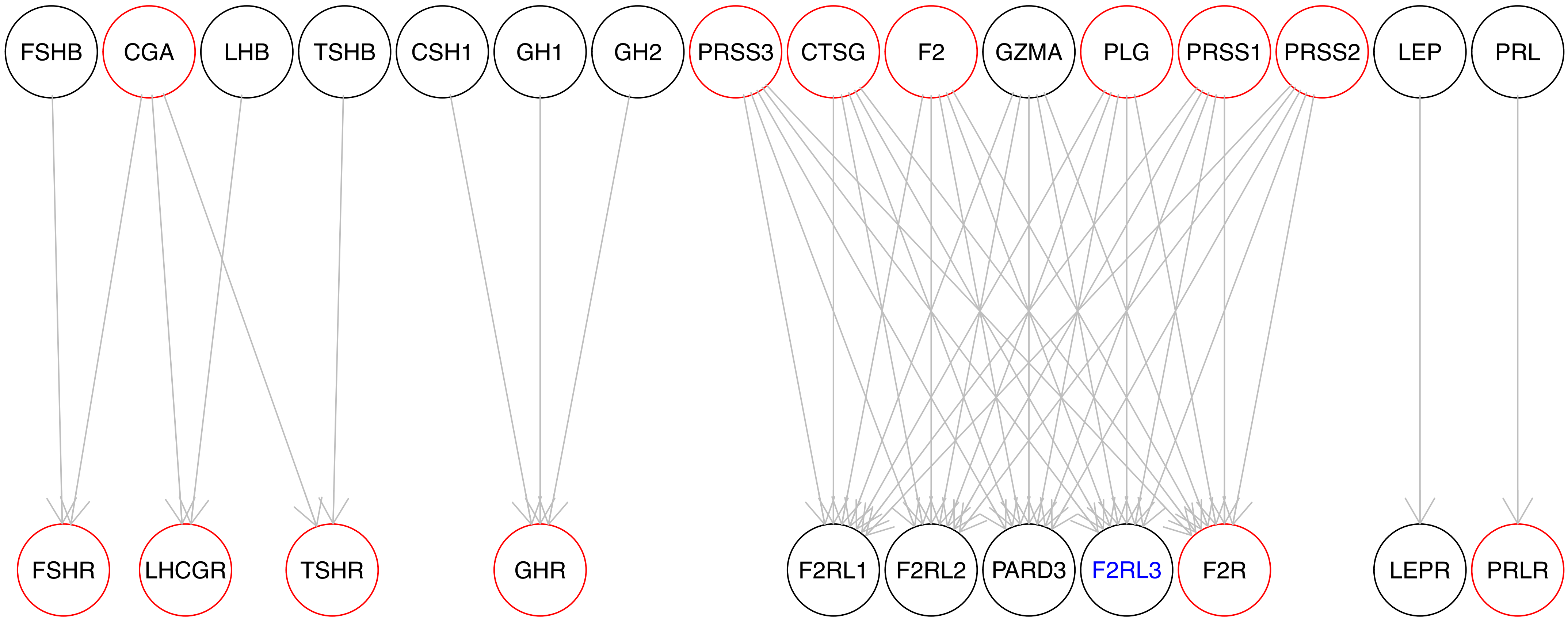

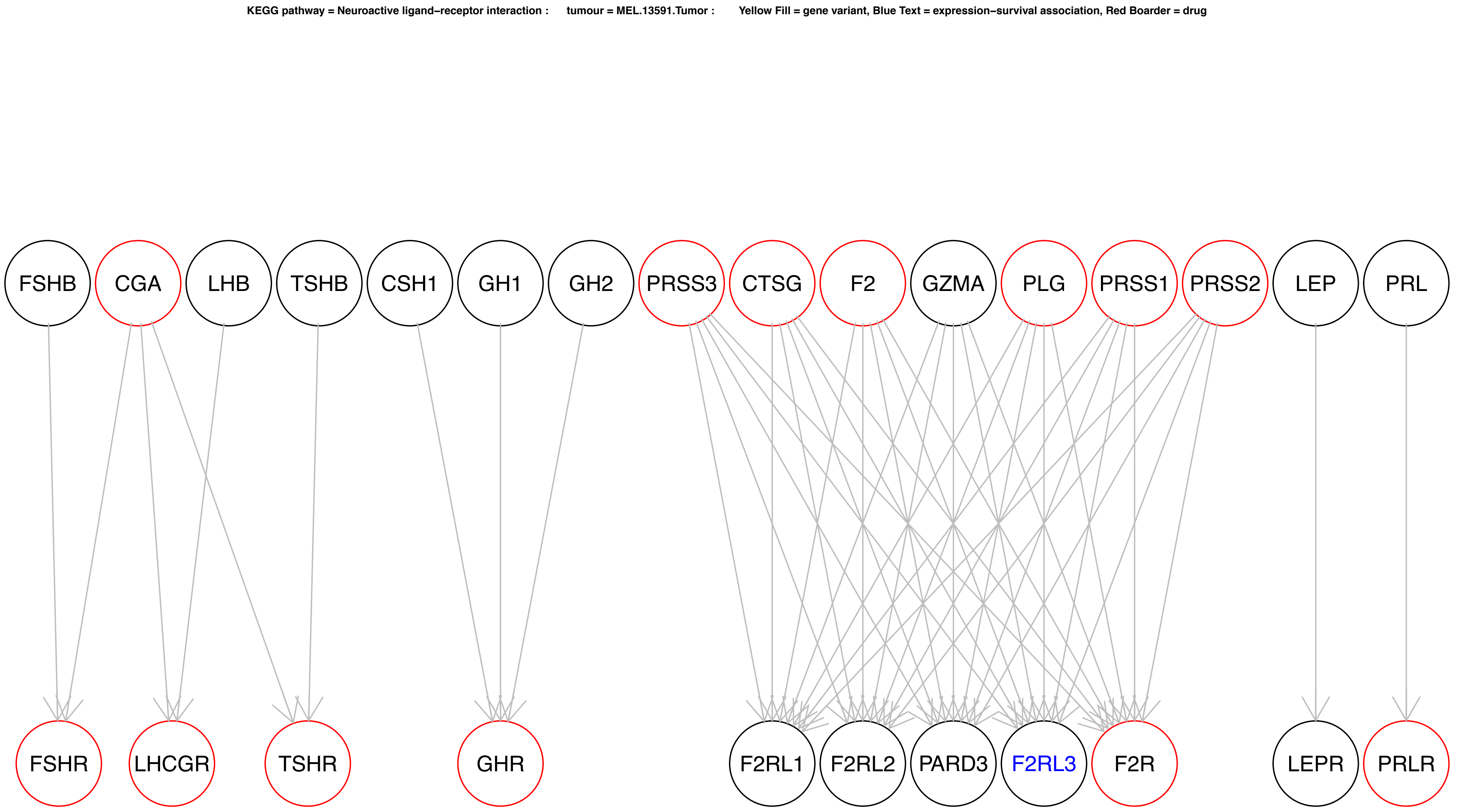
KEGG pathway = Neuroactive ligand−receptor interaction : tumour = MEL.13591.Tumor : Yellow Fill = gene variant, Blue Text = expression−survival association, Red Boarder = drug
FSHB
CGA
LHB
TSHB
CSH1
GH1
GH2
PRSS3
CTSG
F2
GZMA
PLG
PRSS1
PRSS2
LEP
PRL
FSHR
LHCGR
TSHR
GHR
F2RL1
F2RL2
PARD3
F2RL3
F2R
LEPR
PRLR

KEGG pathway = Neuroactive ligand−receptor interaction : tumour = MEL.13600.Tumor : Yellow Fill = gene variant, Blue Text = expression−survival association, Red Boarder = drug

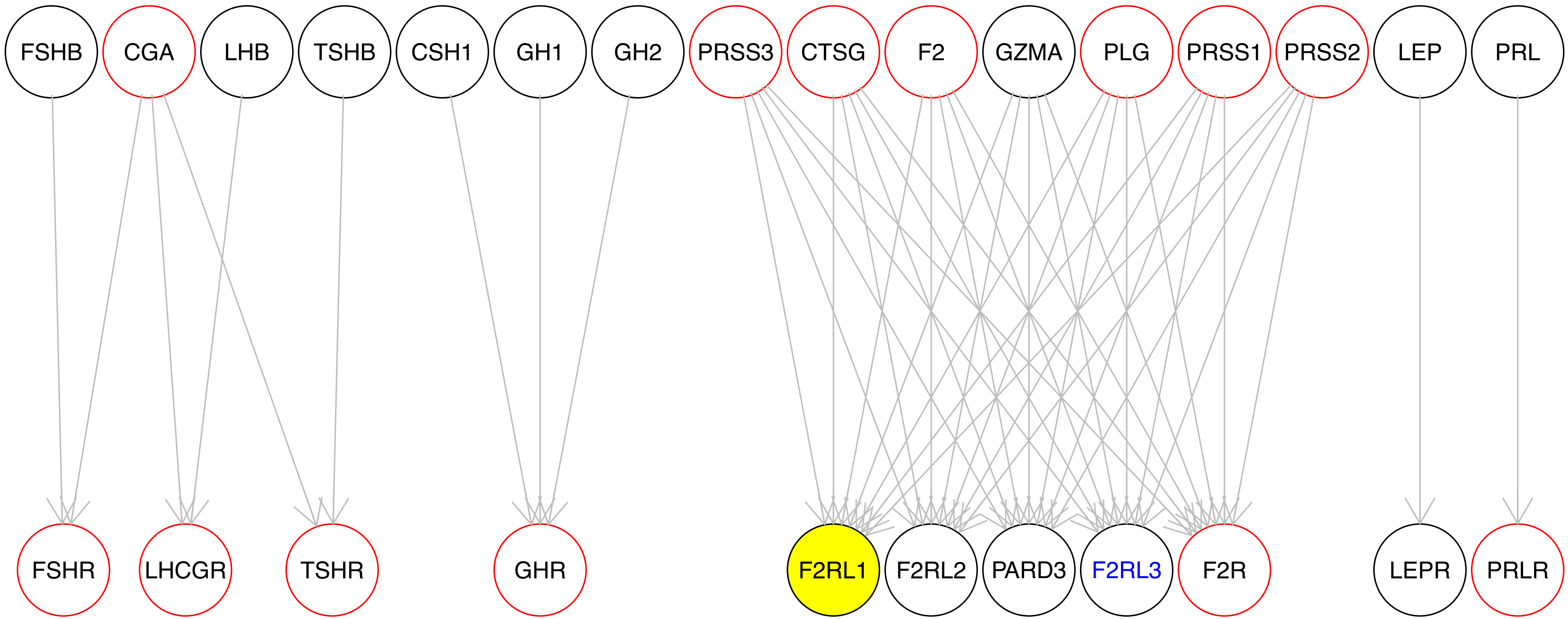

KEGG pathway = Neuroactive ligand−receptor interaction :   tumour = Mel.BRAFi.03.Tumor :   Yellow Fill = gene variant, Blue Text = expression−survival association, Red Boarder = drug

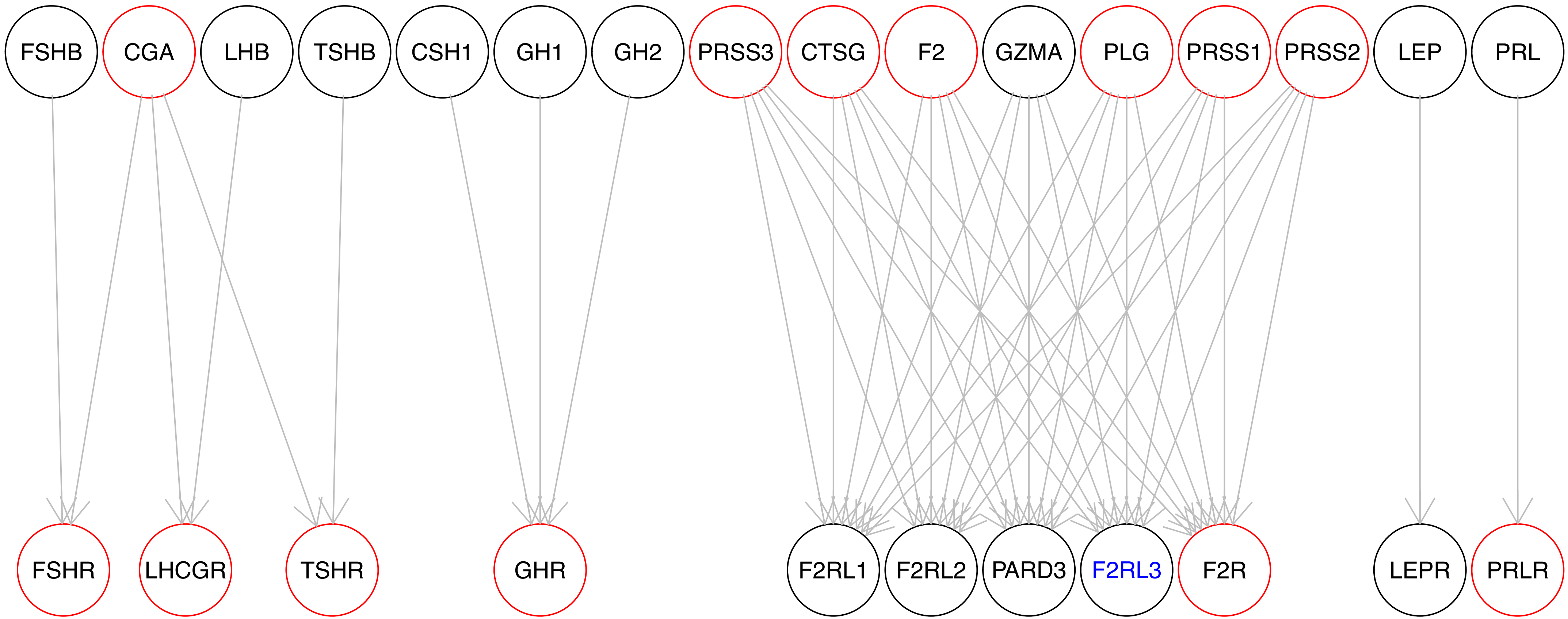

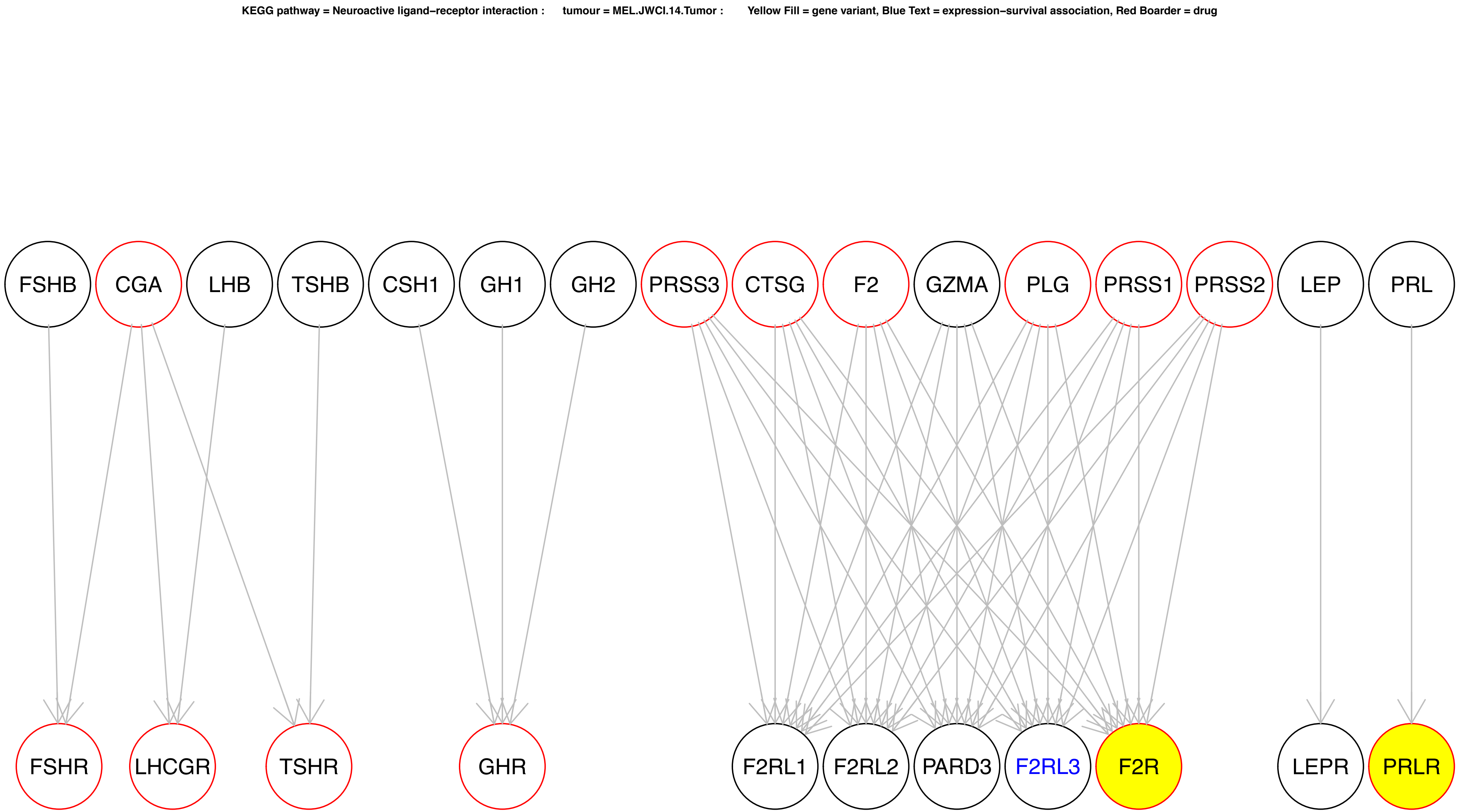

KEGG pathway = Neuroactive ligand−receptor interaction : tumour = MEL.JWCI.14.Tumor : Yellow Fill = gene variant, Blue Text = expression−survival association, Red Boarder = drug
FSHB
CGA
LHB
TSHB
CSH1
GH1
GH2
PRSS3
CTSG
F2
GZMA
PLG
PRSS1
PRSS2
LEP
PRL
FSHR
LHCGR
TSHR
GHR
F2RL1
F2RL2
PARD3
F2RL3
F2R
LEPR
PRLR

KEGG pathway = Neuroactive ligand−receptor interaction : tumour = MEL.JWCI.27.Tumor : Yellow Fill = gene variant, Blue Text = expression−survival association, Red Boarder = drug

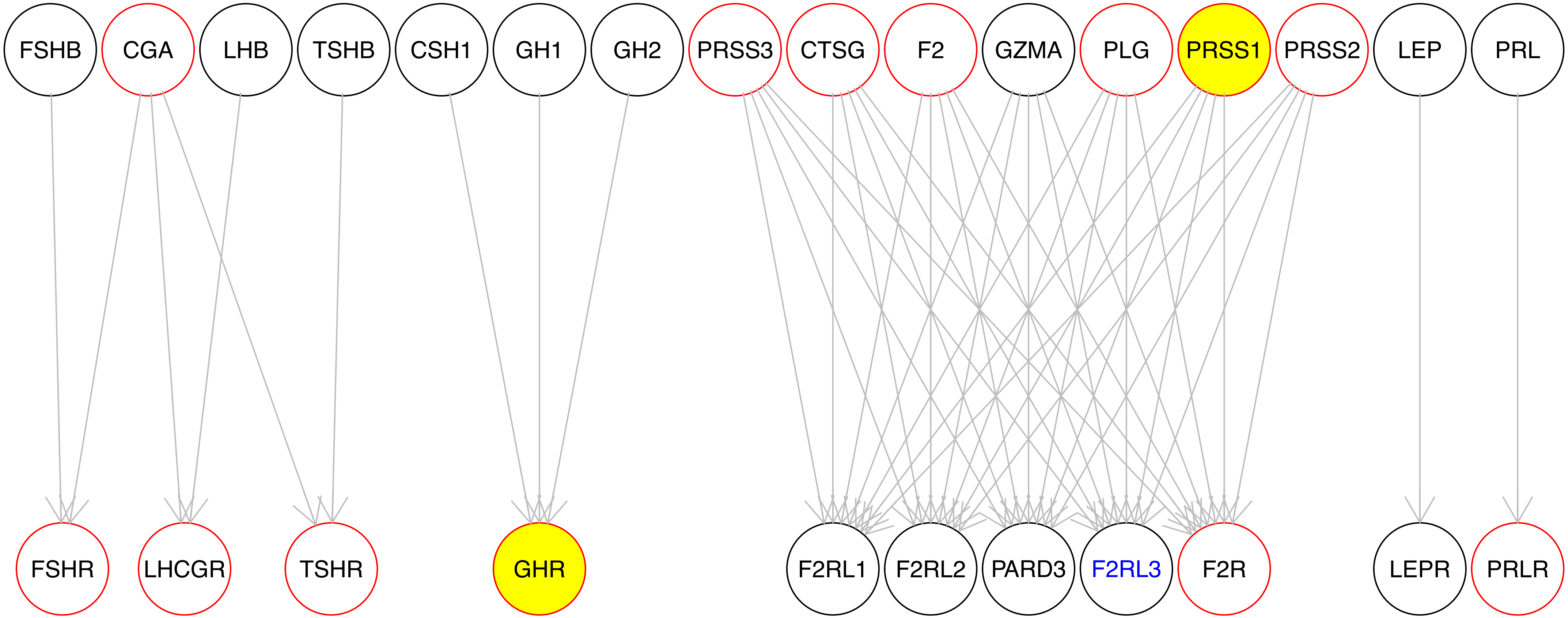

KEGG pathway = Neuroactive ligand−receptor interaction : tumour = MEL.JWCI.WGS.11.Tumor : Yellow Fill = gene variant, Blue Text = expression−survival association, Red Boarder = drug

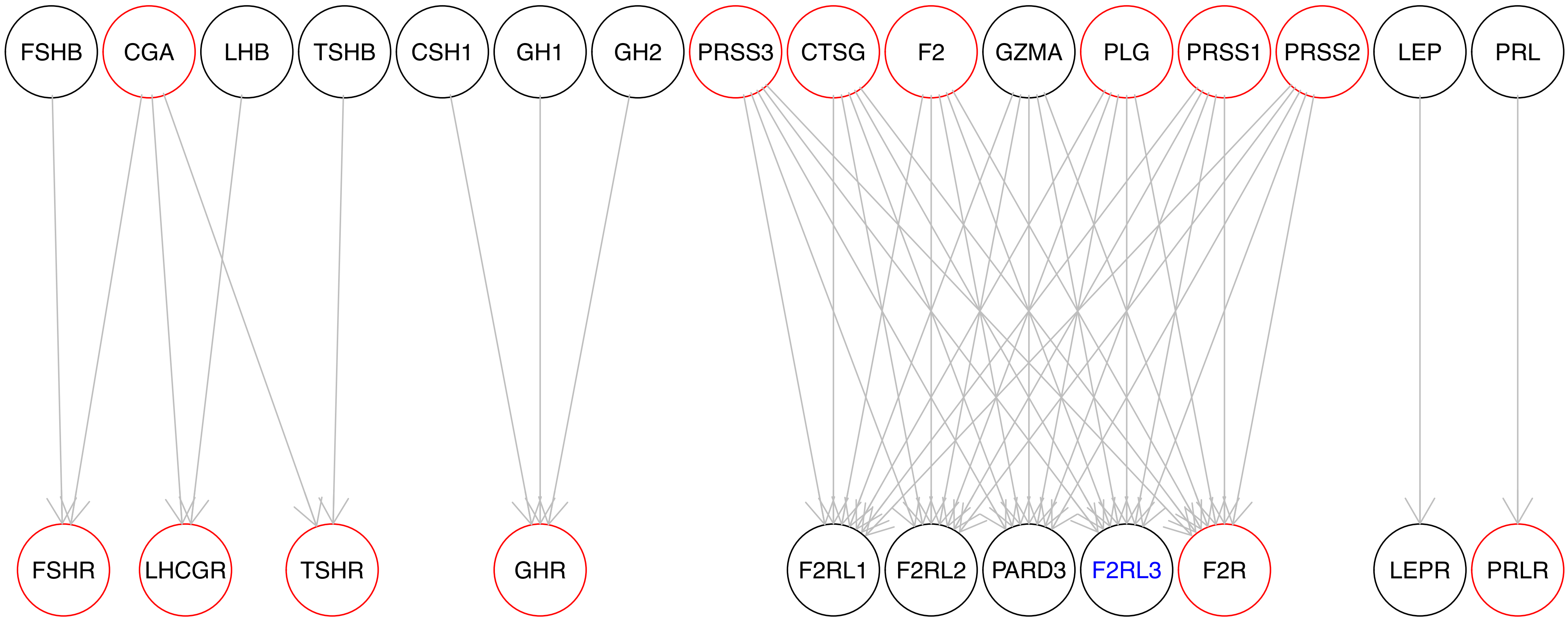

KEGG pathway = Neuroactive ligand−receptor interaction :   tumour = MEL.JWCI.WGS.12.Tumor :   Yellow Fill = gene variant, Blue Text = expression−survival association, Red Boarder = drug

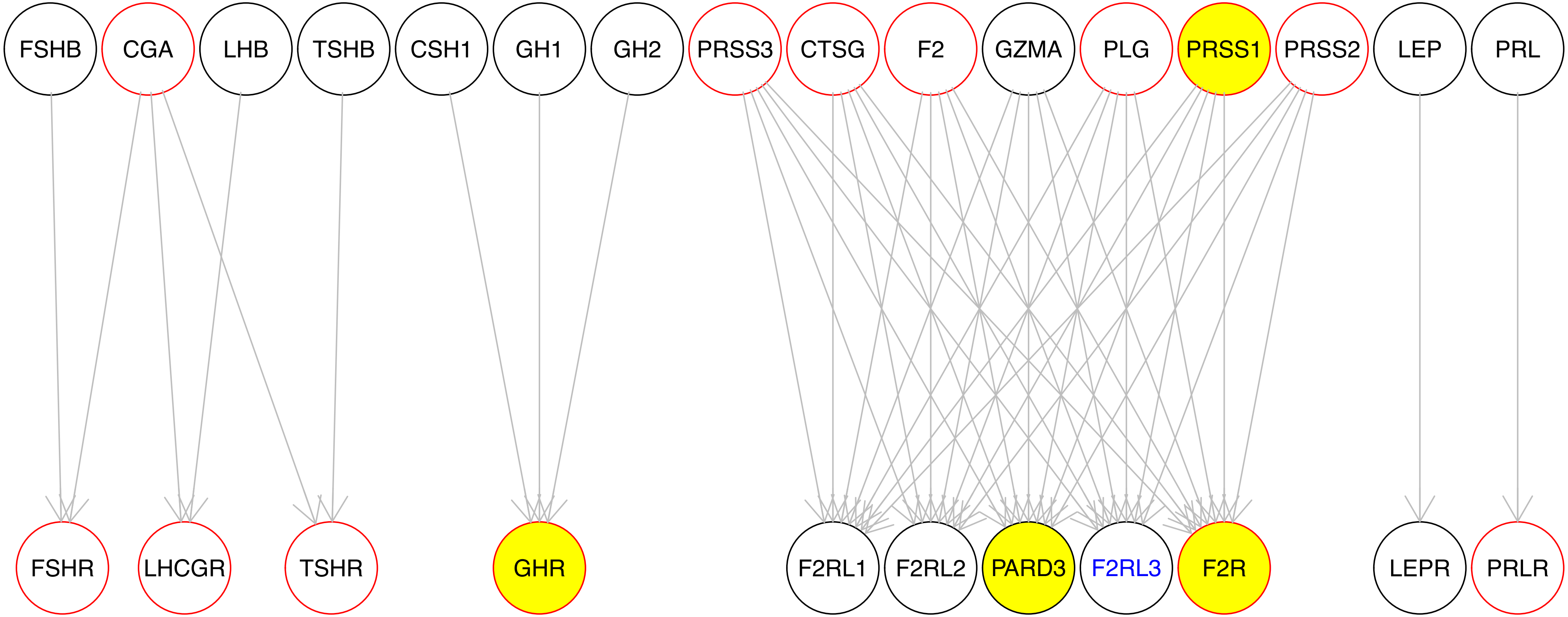

KEGG pathway = Neuroactive ligand−receptor interaction :   tumour = MEL.JWCI.WGS.13.Tumor :   Yellow Fill = gene variant, Blue Text = expression−survival association, Red Boarder = drug

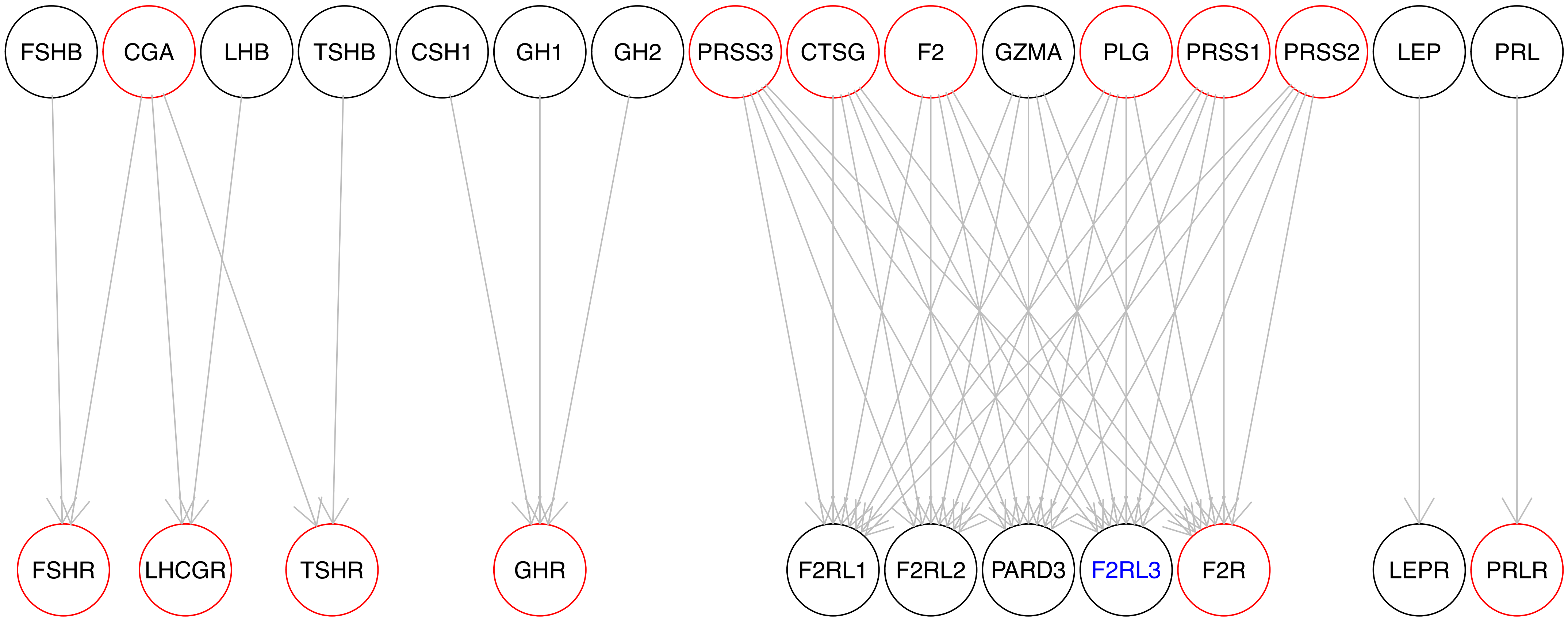

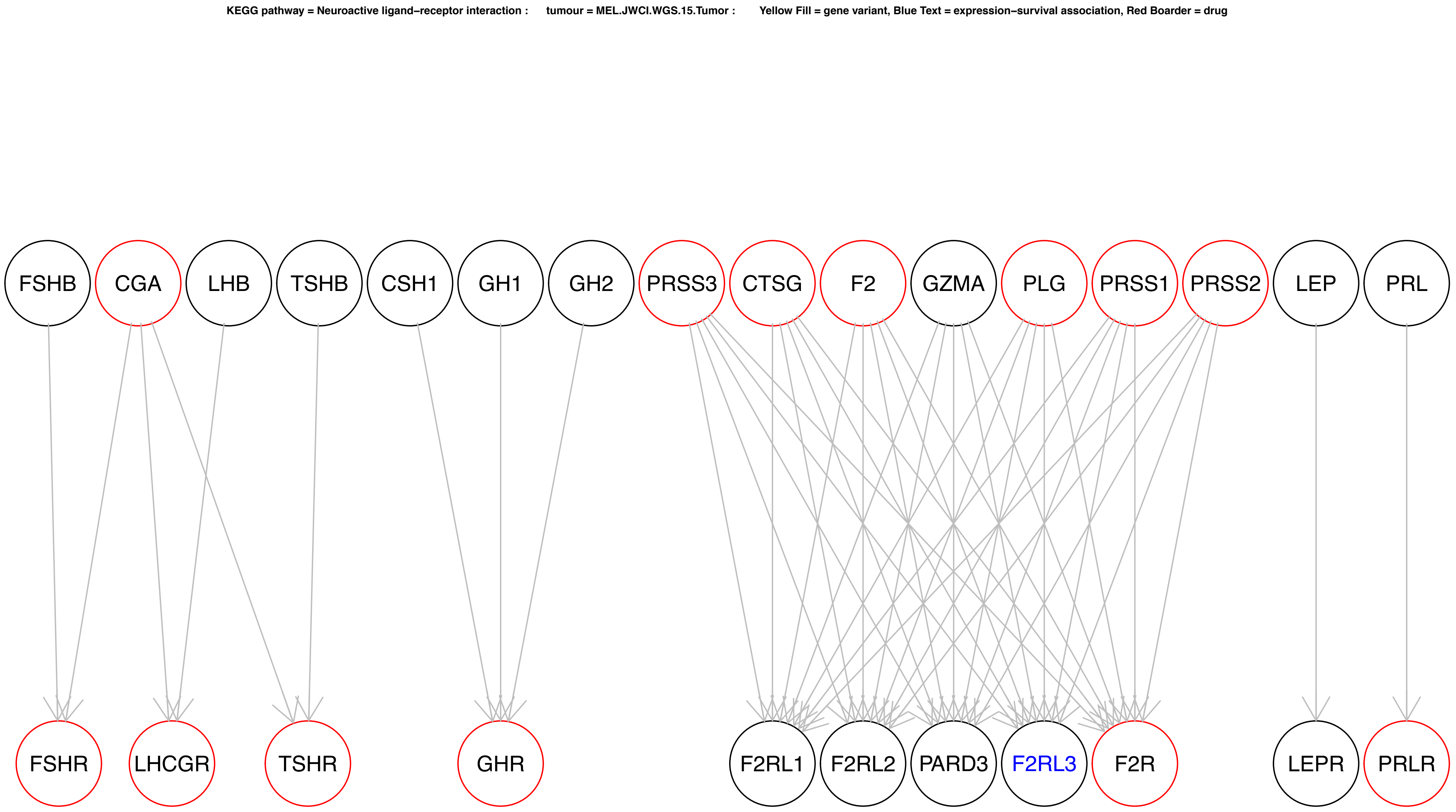
KEGG pathway = Neuroactive ligand−receptor interaction :   tumour = MEL.JWCI.WGS.15.Tumor :   Yellow Fill = gene variant, Blue Text = expression−survival association, Red Boarder = drug
FSHB
CGA
LHB
TSHB
CSH1
GH1
GH2
PRSS3
CTSG
F2
GZMA
PLG
PRSS1
PRSS2
LEP
PRL
FSHR
LHCGR
TSHR
GHR
F2RL1
F2RL2
PARD3
F2RL3
F2R
LEPR
PRLR

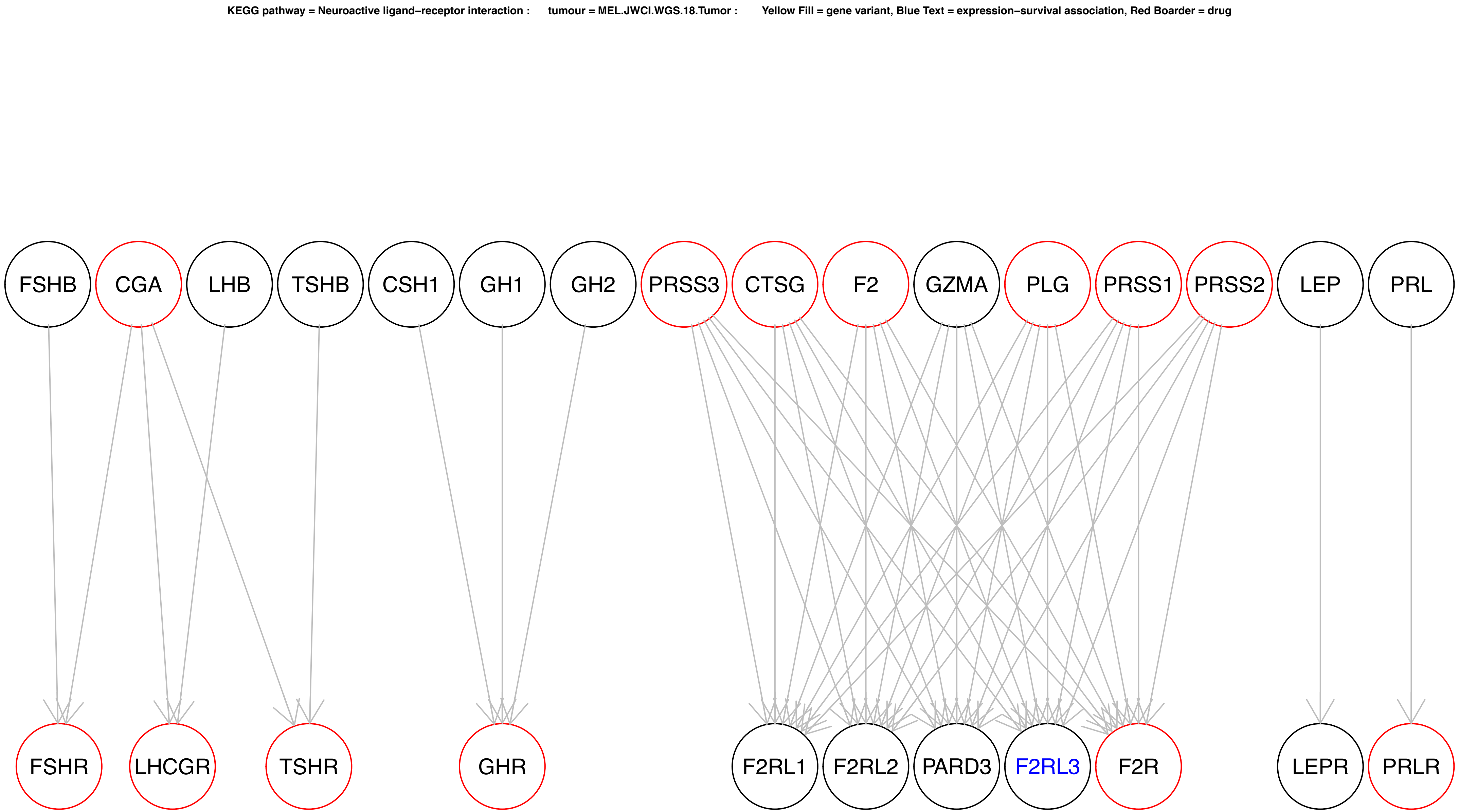
KEGG pathway = Neuroactive ligand−receptor interaction : tumour = MEL.JWCI.WGS.18.Tumor : Yellow Fill = gene variant, Blue Text = expression−survival association, Red Boarder = drug
FSHB
CGA
LHB
TSHB
CSH1
GH1
GH2
PRSS3
CTSG
F2
GZMA
PLG
PRSS1
PRSS2
LEP
PRL
FSHR
LHCGR
TSHR
GHR
F2RL1
F2RL2
PARD3
F2RL3
F2R
LEPR
PRLR

KEGG pathway = Neuroactive ligand−receptor interaction : tumour = MEL.JWCI.WGS.19.Tumor : Yellow Fill = gene variant, Blue Text = expression−survival association, Red Boarder = drug

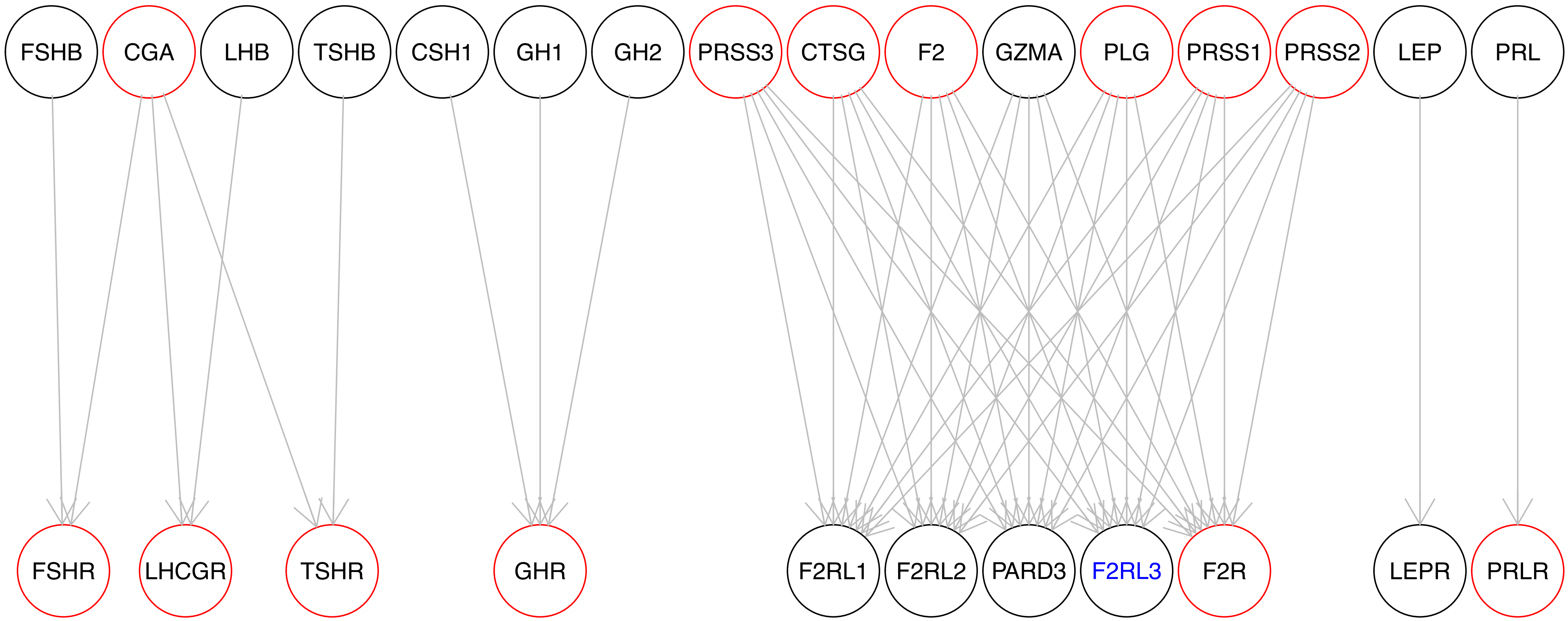

KEGG pathway = Neuroactive ligand−receptor interaction : tumour = MEL.JWCI.WGS.1.Tumor : Yellow Fill = gene variant, Blue Text = expression−survival association, Red Boarder = drug

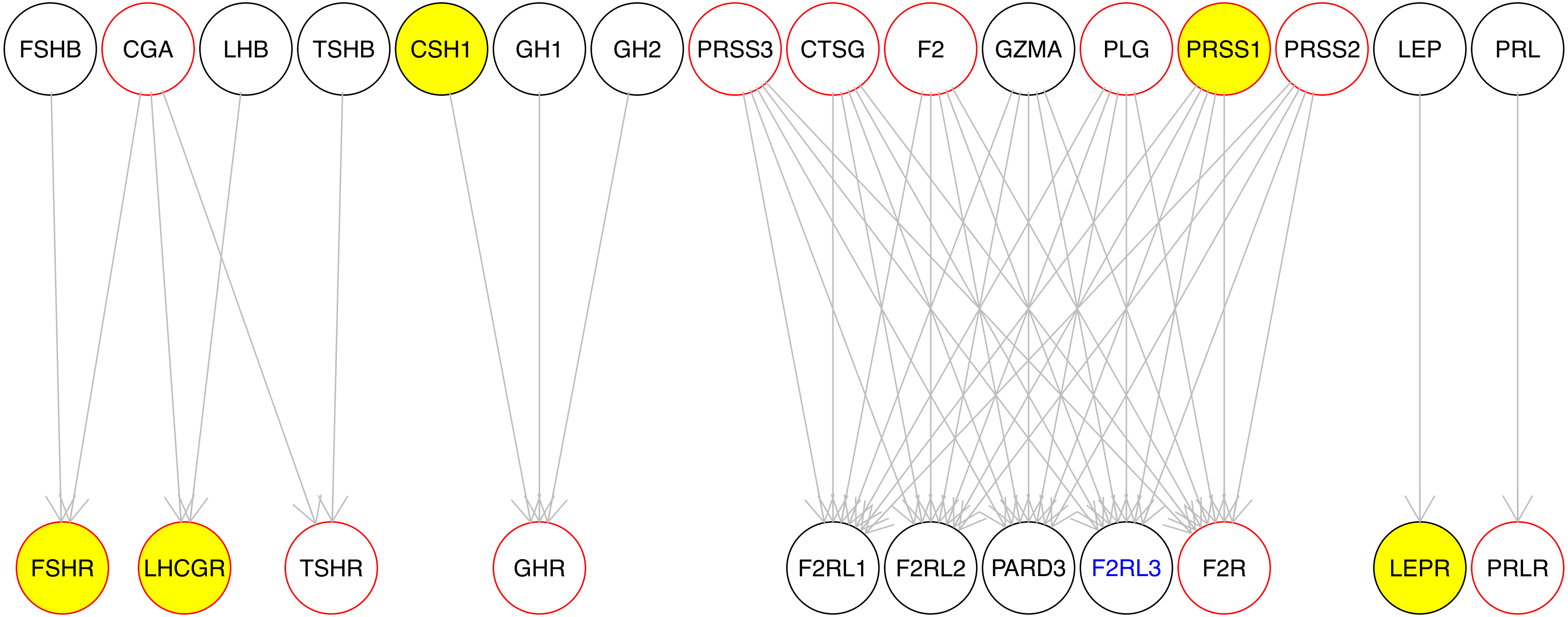

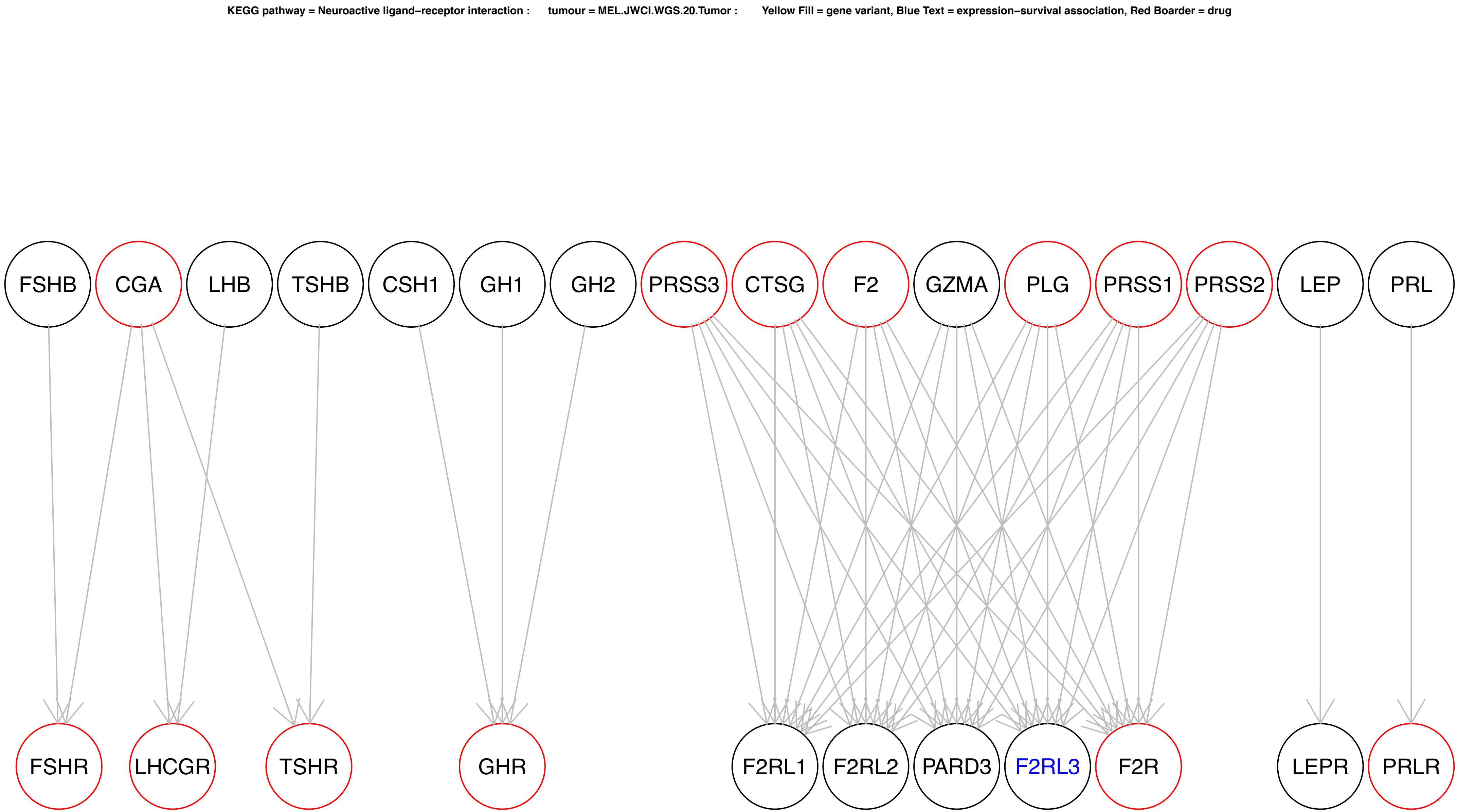
KEGG pathway = Neuroactive ligand−receptor interaction :
tumour = MEL.JWCI.WGS.20.Tumor :
Yellow Fill = gene variant, Blue Text = expression−survival association, Red Boarder = drug
FSHB
CGA
LHB
TSHB
CSH1
GH1
GH2
PRSS3
CTSG
F2
GZMA
PLG
PRSS1
PRSS2
LEP
PRL
FSHR
LHCGR
TSHR
GHR
F2RL1
F2RL2
PARD3
F2RL3
F2R
LEPR
PRLR

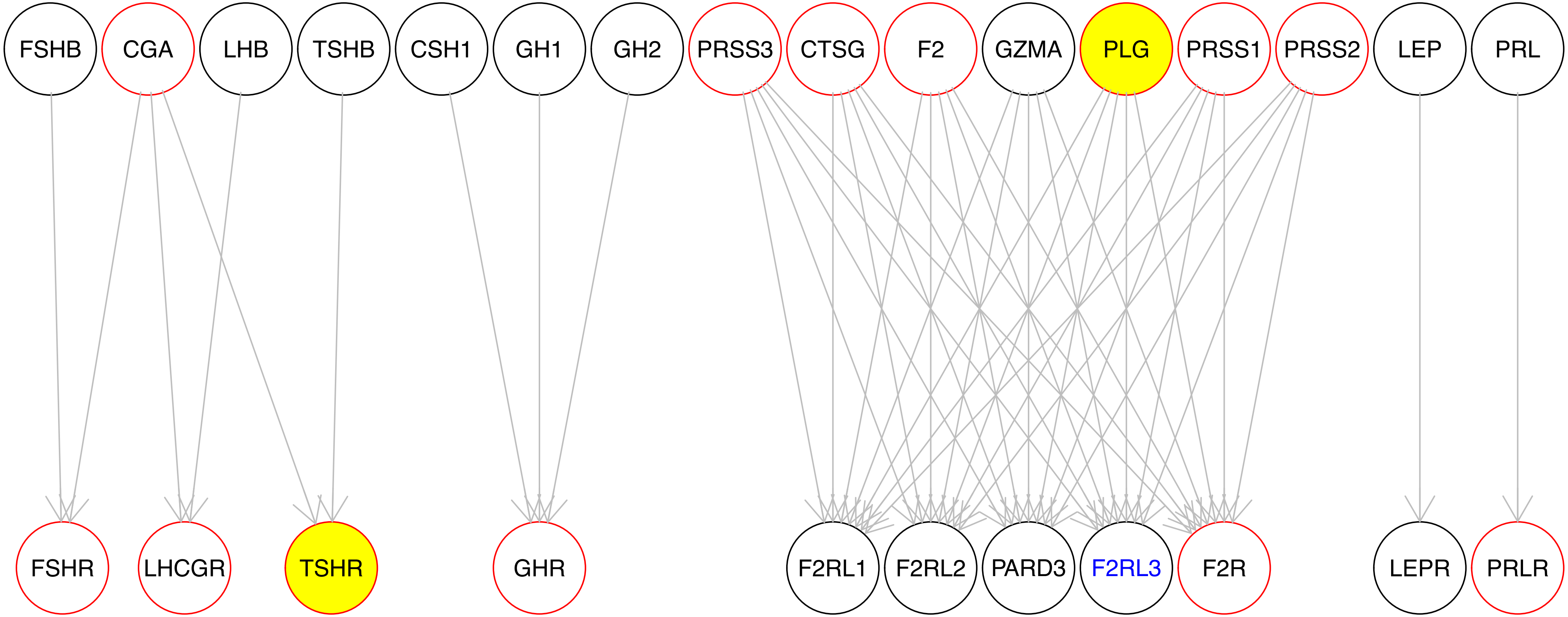
FSHB
CGA
LHB
TSHB
CSH1
GH1
GH2
PRSS3
CTSG
F2
GZMA
PLG
PRSS1
PRSS2
LEP
PRL
FSHR
LHCGR
TSHR
GHR
F2RL1
F2RL2
PARD3
F2RL3
F2R
LEPR
PRLR

KEGG pathway = Neuroactive ligand−receptor interaction : tumour = MEL.JWCI.WGS.22.Tumor : Yellow Fill = gene variant, Blue Text = expression−survival association, Red Boarder = drug

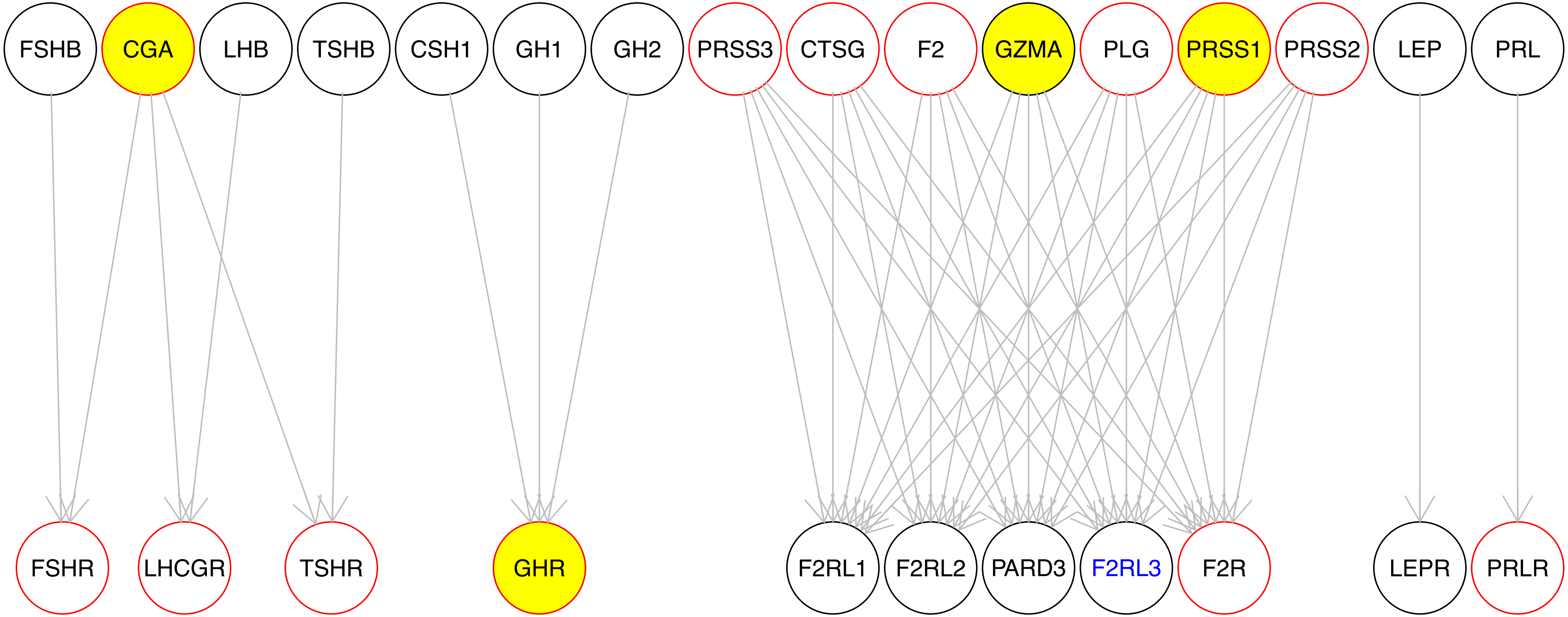

KEGG pathway = Neuroactive ligand−receptor interaction :   tumour = MEL.JWCI.WGS.23.Tumor :   Yellow Fill = gene variant, Blue Text = expression−survival association, Red Boarder = drug

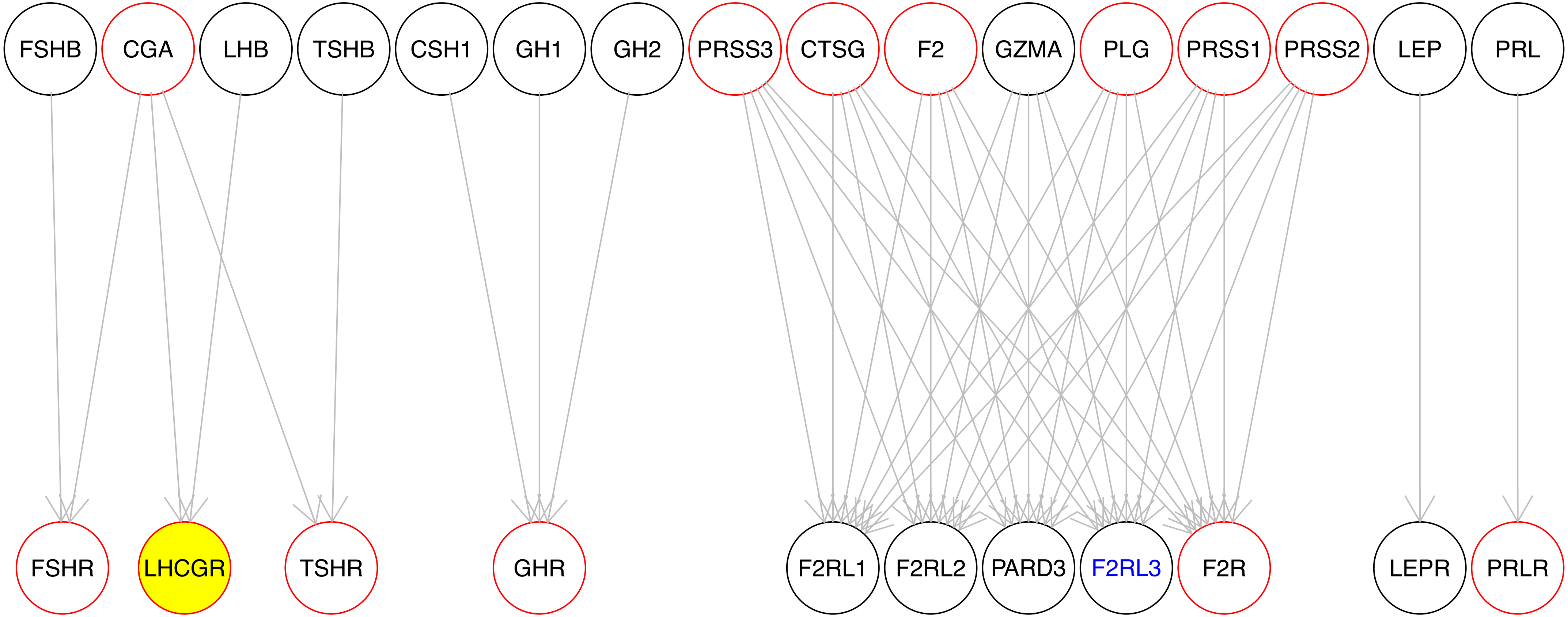

KEGG pathway = Neuroactive ligand−receptor interaction : tumour = MEL.JWCI.WGS.24.Tumor : Yellow Fill = gene variant, Blue Text = expression−survival association, Red Boarder = drug

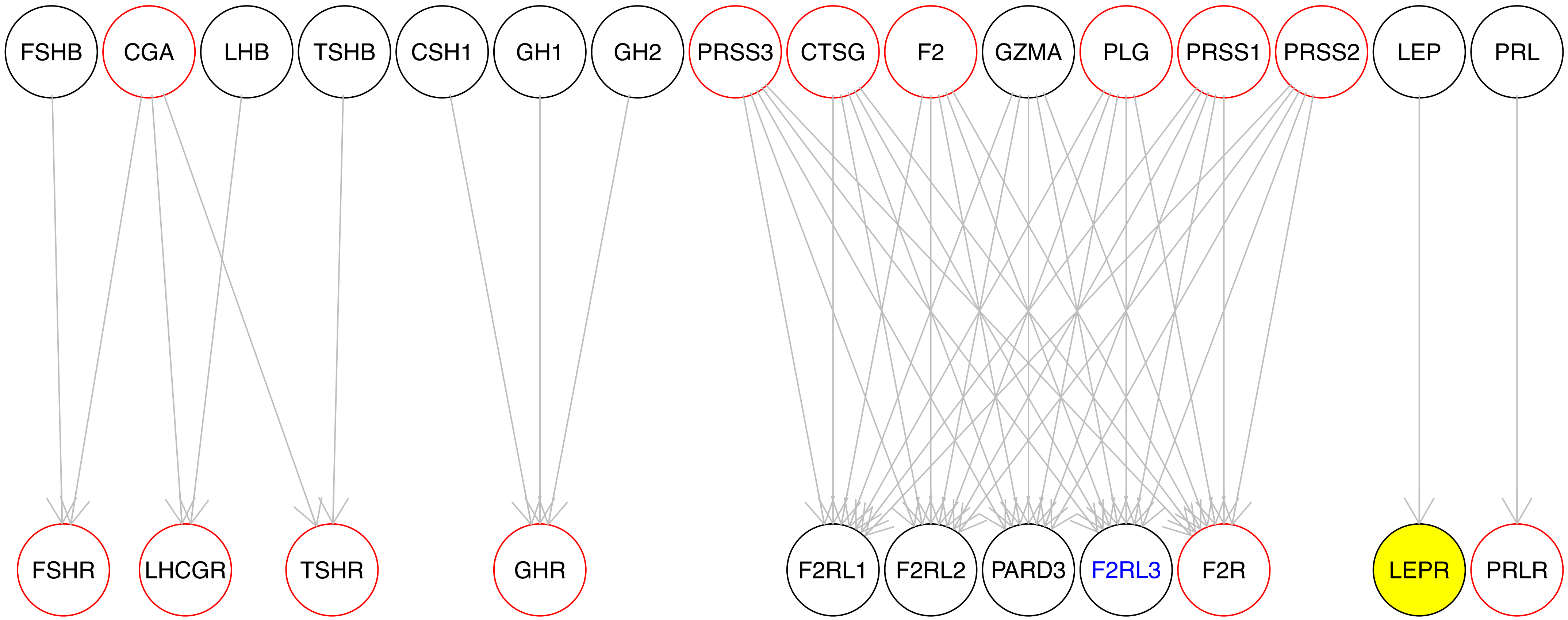

KEGG pathway = Neuroactive ligand–receptor interaction :   tumour = MEL.JWCI.WGS.25.Tumor :   Yellow Fill = gene variant, Blue Text = expression–survival association, Red Boarder = drug

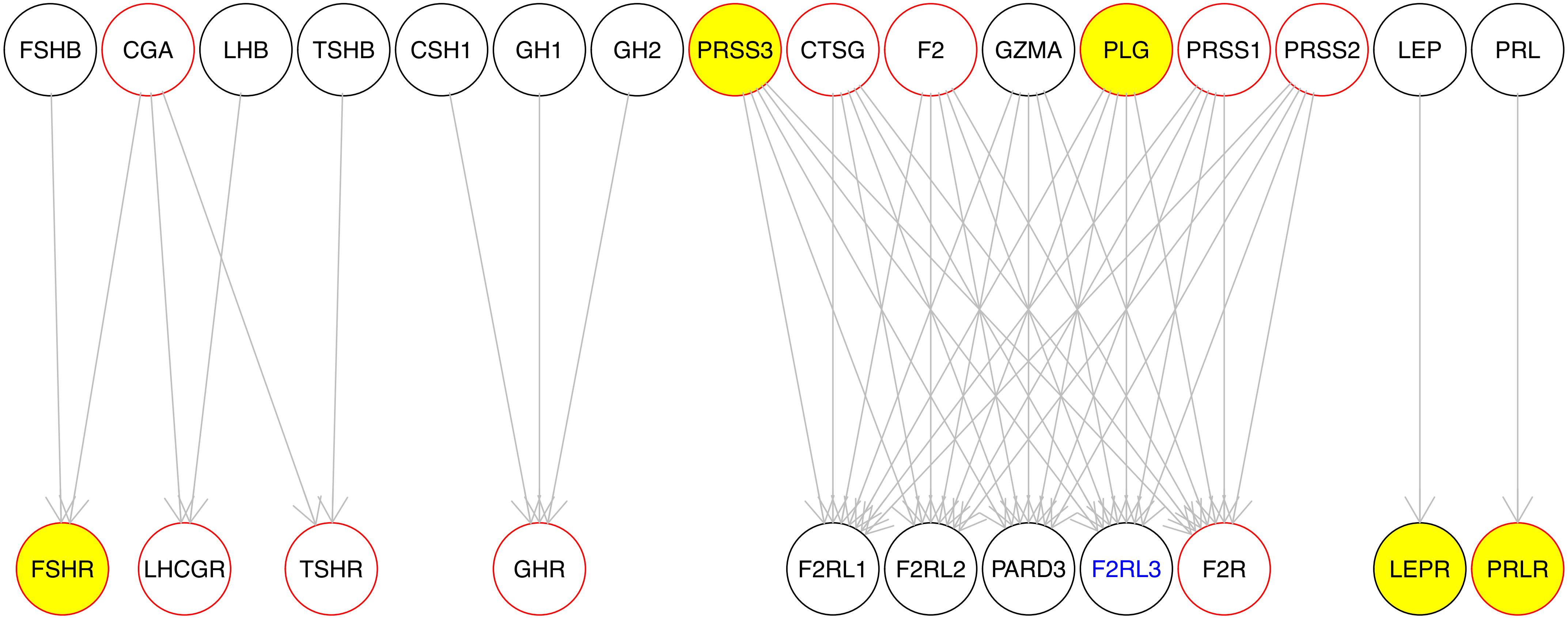

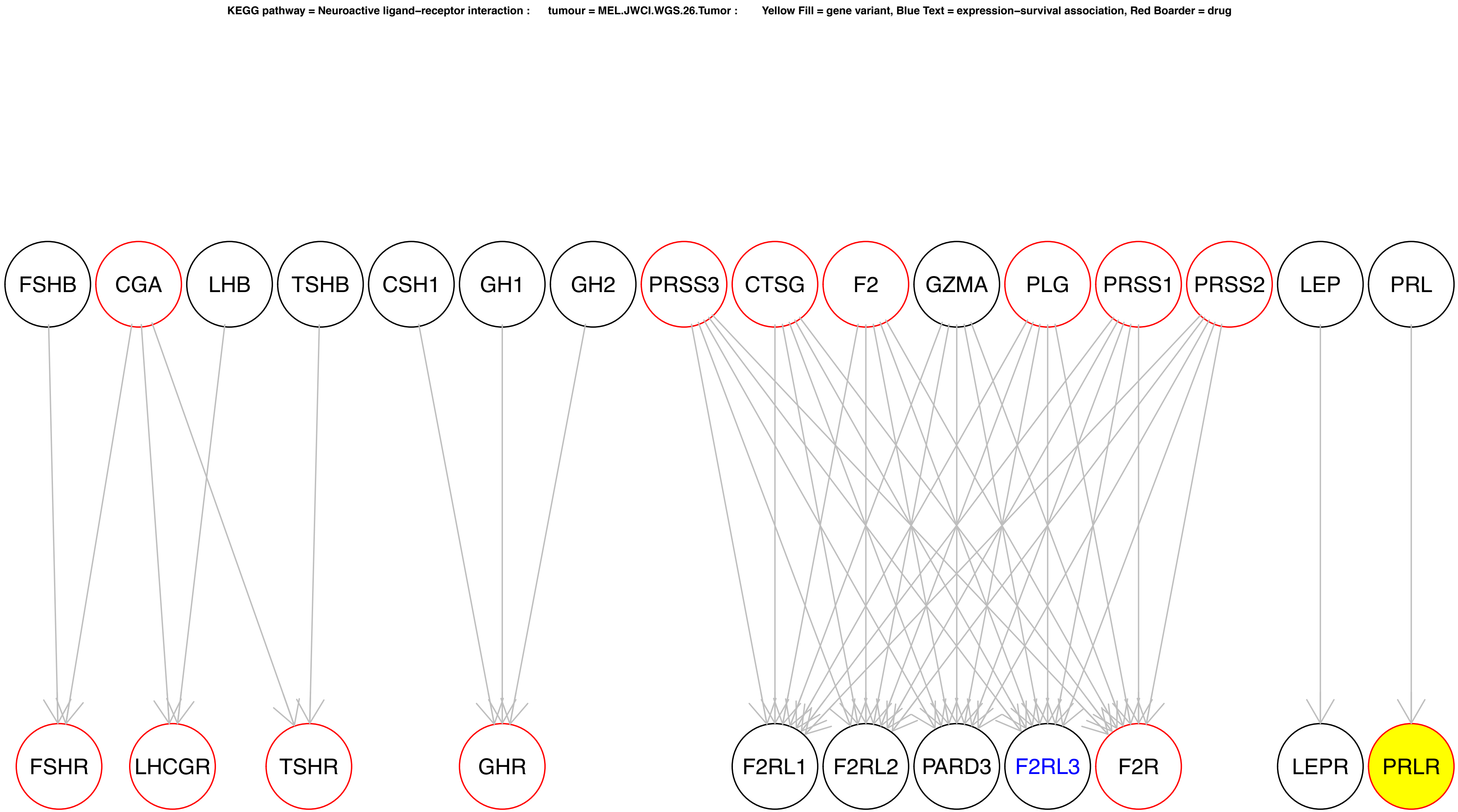
KEGG pathway = Neuroactive ligand−receptor interaction :   tumour = MEL.JWCI.WGS.26.Tumor :   Yellow Fill = gene variant, Blue Text = expression−survival association, Red Boarder = drug
FSHB
CGA
LHB
TSHB
CSH1
GH1
GH2
PRSS3
CTSG
F2
GZMA
PLG
PRSS1
PRSS2
LEP
PRL
FSHR
LHCGR
TSHR
GHR
F2RL1
F2RL2
PARD3
F2RL3
F2R
LEPR
PRLR

KEGG pathway = Neuroactive ligand−receptor interaction :   tumour = MEL.JWCI.WGS.29.Tumor :   Yellow Fill = gene variant, Blue Text = expression−survival association, Red Boarder = drug

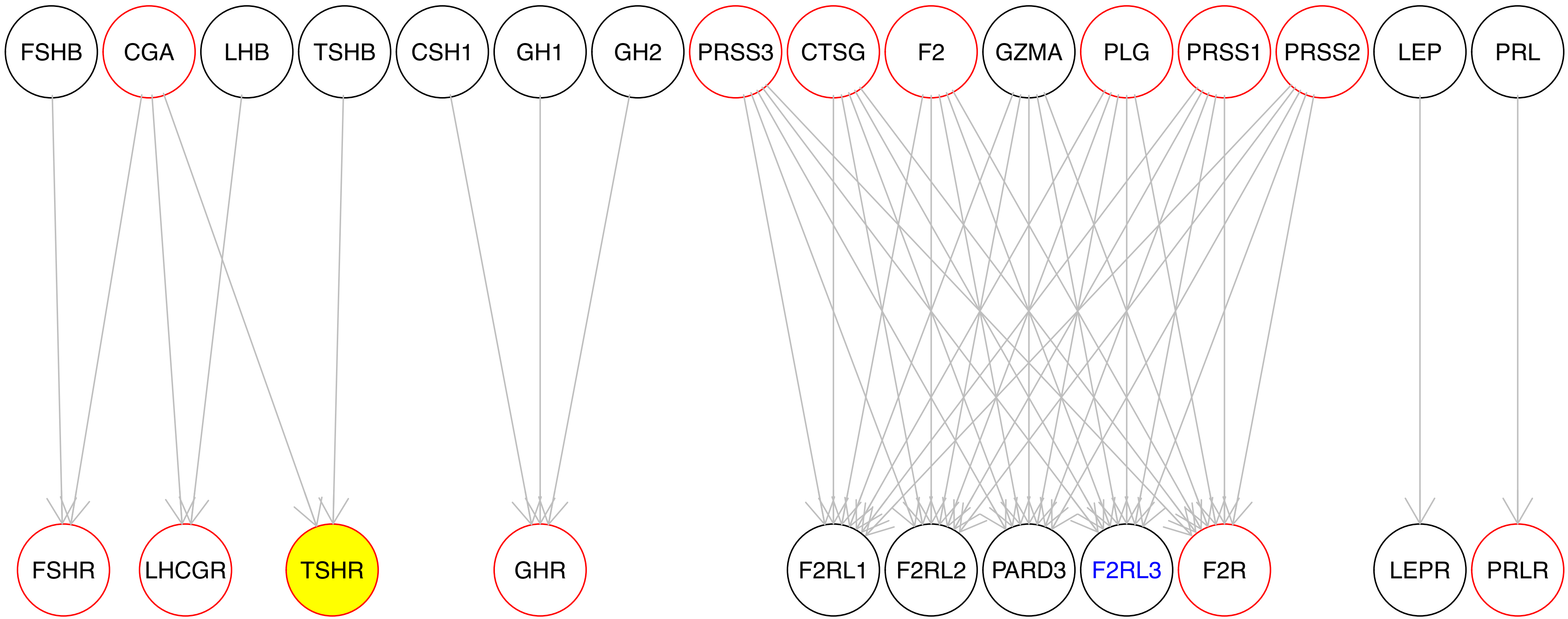

KEGG pathway = Neuroactive ligand−receptor interaction : tumour = MEL.JWCI.WGS.2.Tumor : Yellow Fill = gene variant, Blue Text = expression−survival association, Red Boarder = drug

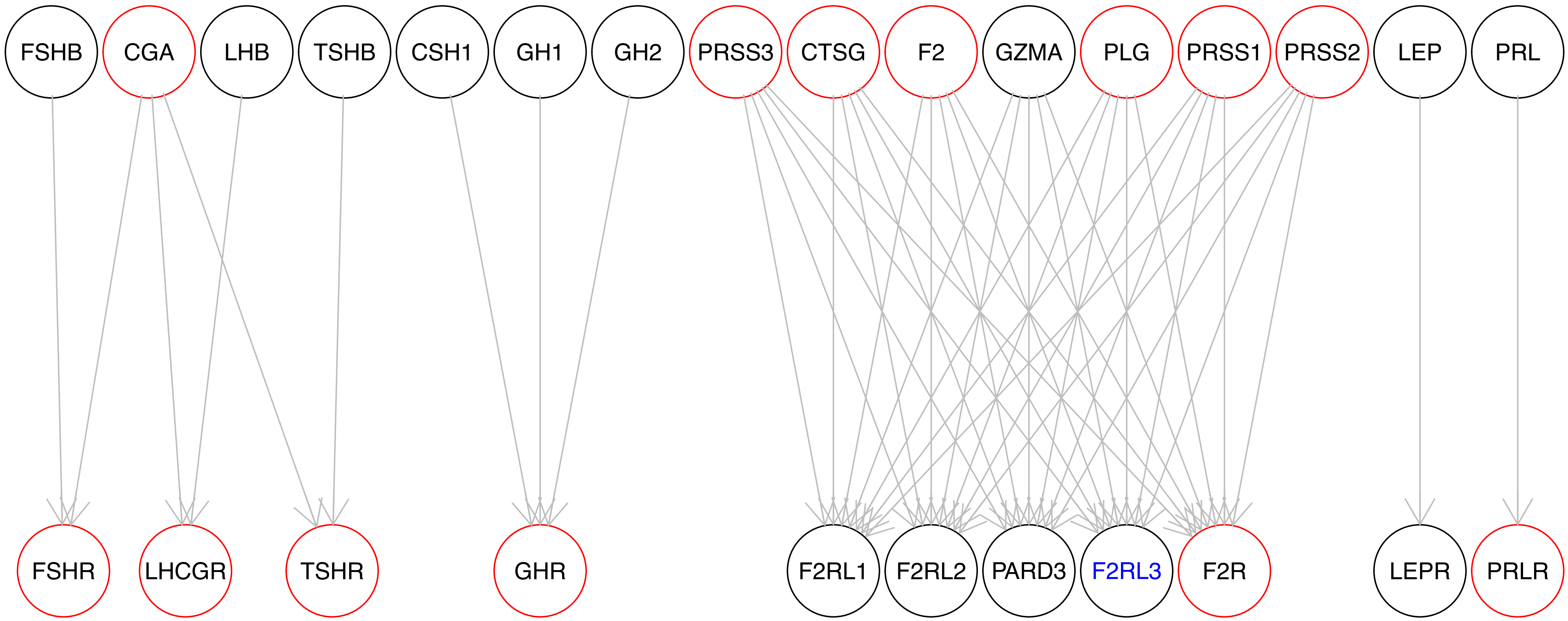

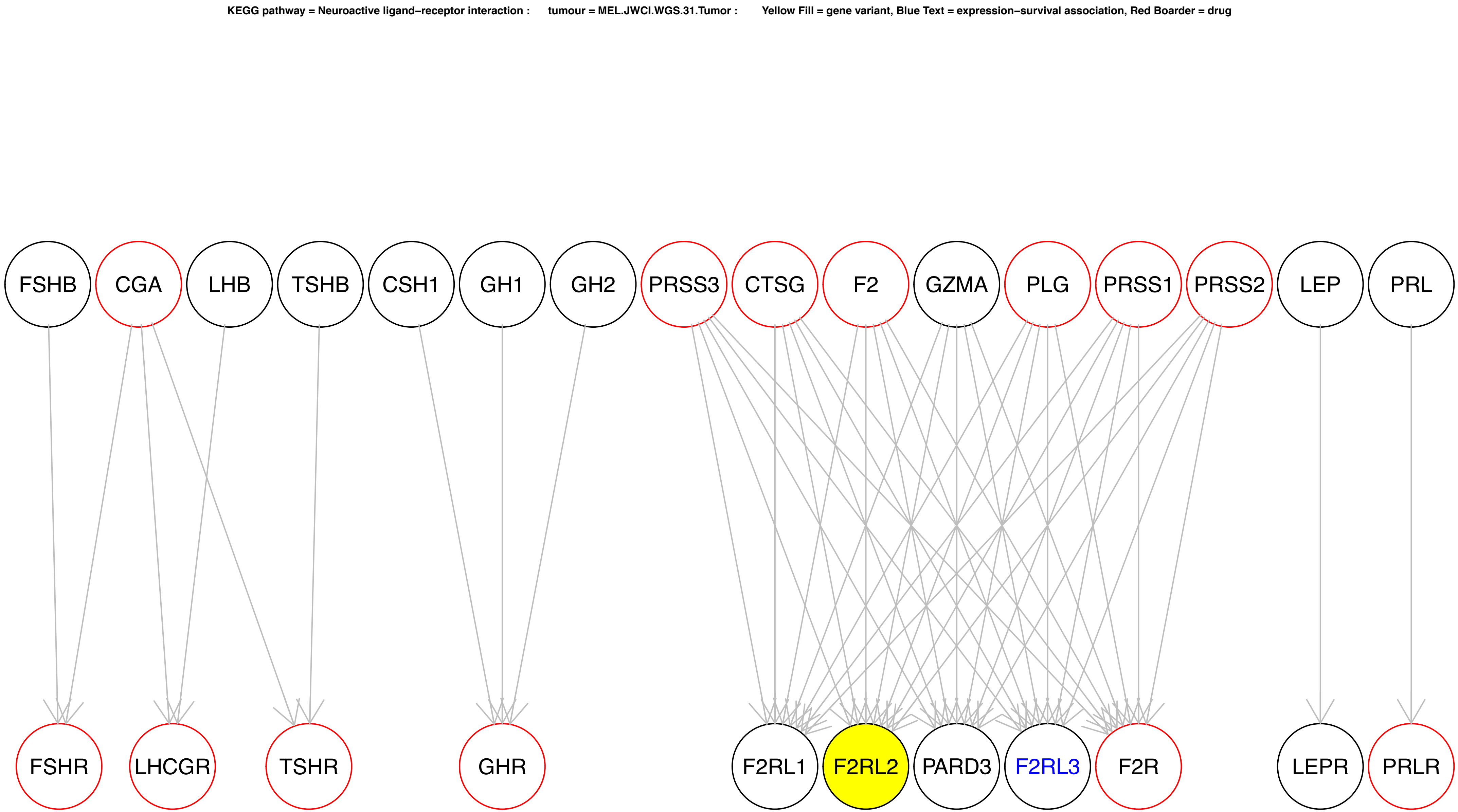
KEGG pathway = Neuroactive ligand−receptor interaction : tumour = MEL.JWCI.WGS.31.Tumor : Yellow Fill = gene variant, Blue Text = expression−survival association, Red Boarder = drug
FSHB
CGA
LHB
TSHB
CSH1
GH1
GH2
PRSS3
CTSG
F2
GZMA
PLG
PRSS1
PRSS2
LEP
PRL
FSHR
LHCGR
TSHR
GHR
F2RL1
F2RL2
PARD3
F2RL3
F2R
LEPR
PRLR

KEGG pathway = Neuroactive ligand−receptor interaction : tumour = MEL.JWCI.WGS.32.Tumor : Yellow Fill = gene variant, Blue Text = expression−survival association, Red Boarder = drug

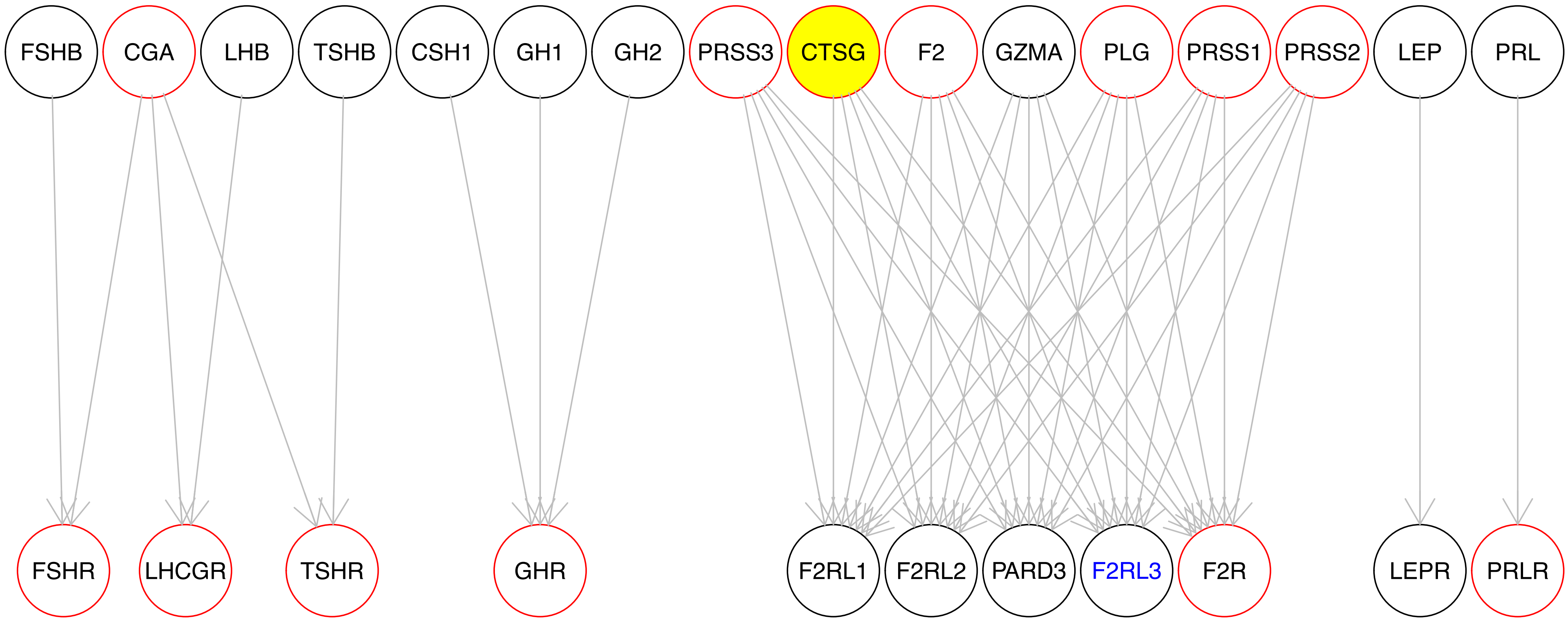

KEGG pathway = Neuroactive ligand−receptor interaction : tumour = MEL.JWCI.WGS.33.Tumor : Yellow Fill = gene variant, Blue Text = expression−survival association, Red Boarder = drug

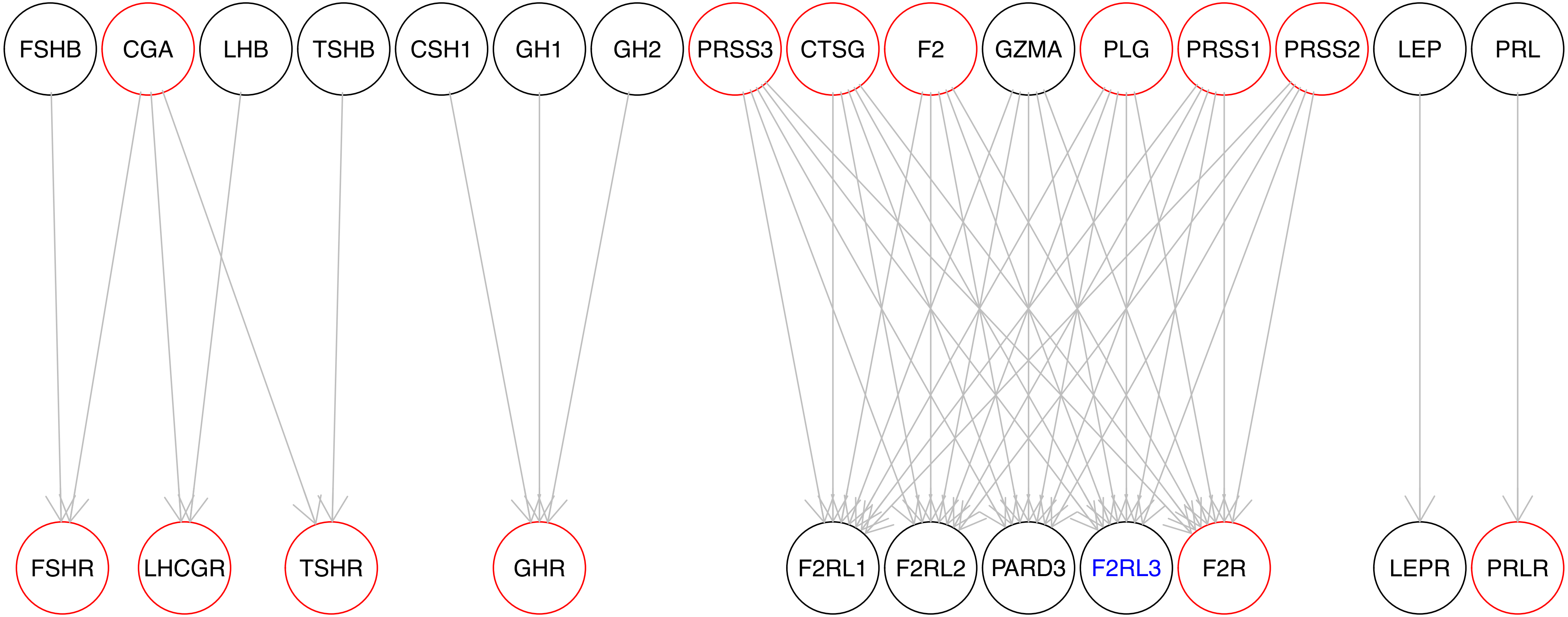

KEGG pathway = Neuroactive ligand−receptor interaction :   tumour = MEL.JWCI.WGS.34.Tumor :   Yellow Fill = gene variant, Blue Text = expression−survival association, Red Boarder = drug

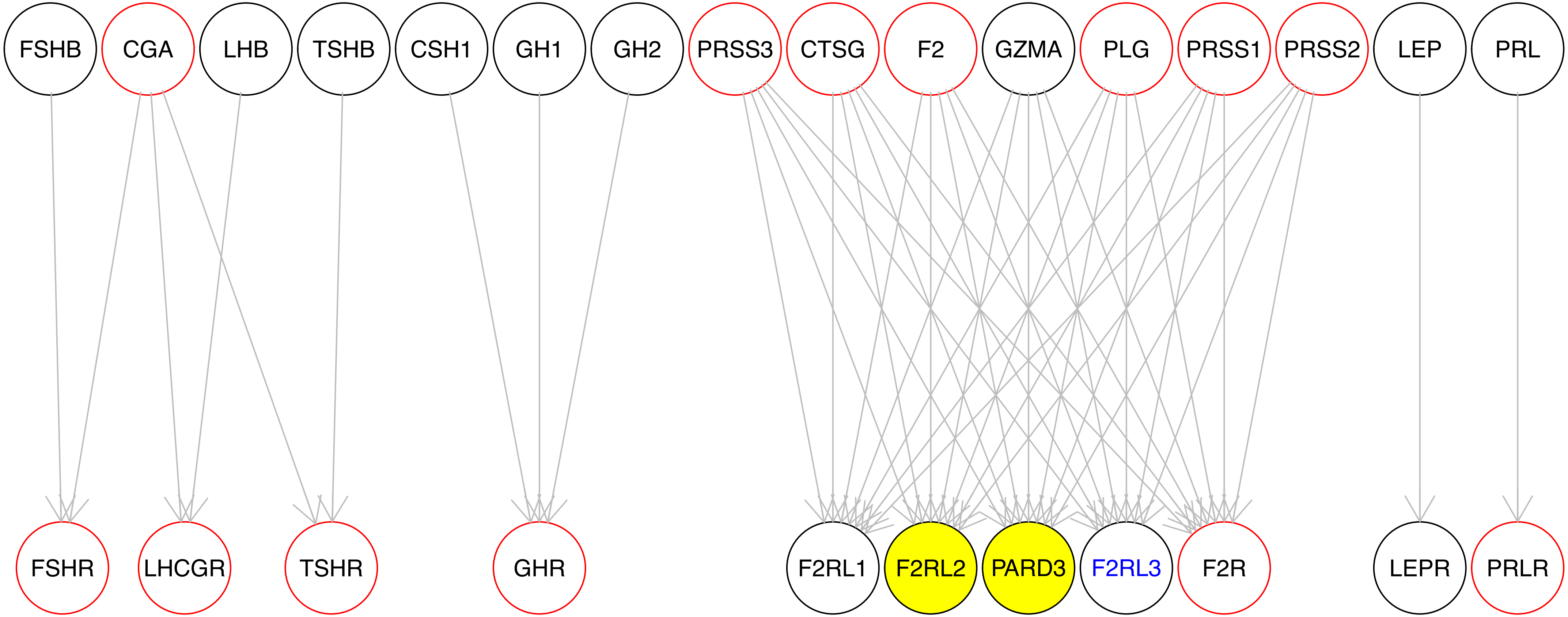

KEGG pathway = Neuroactive ligand−receptor interaction :   tumour = MEL.JWCI.WGS.35.Tumor :   Yellow Fill = gene variant, Blue Text = expression−survival association, Red Boarder = drug

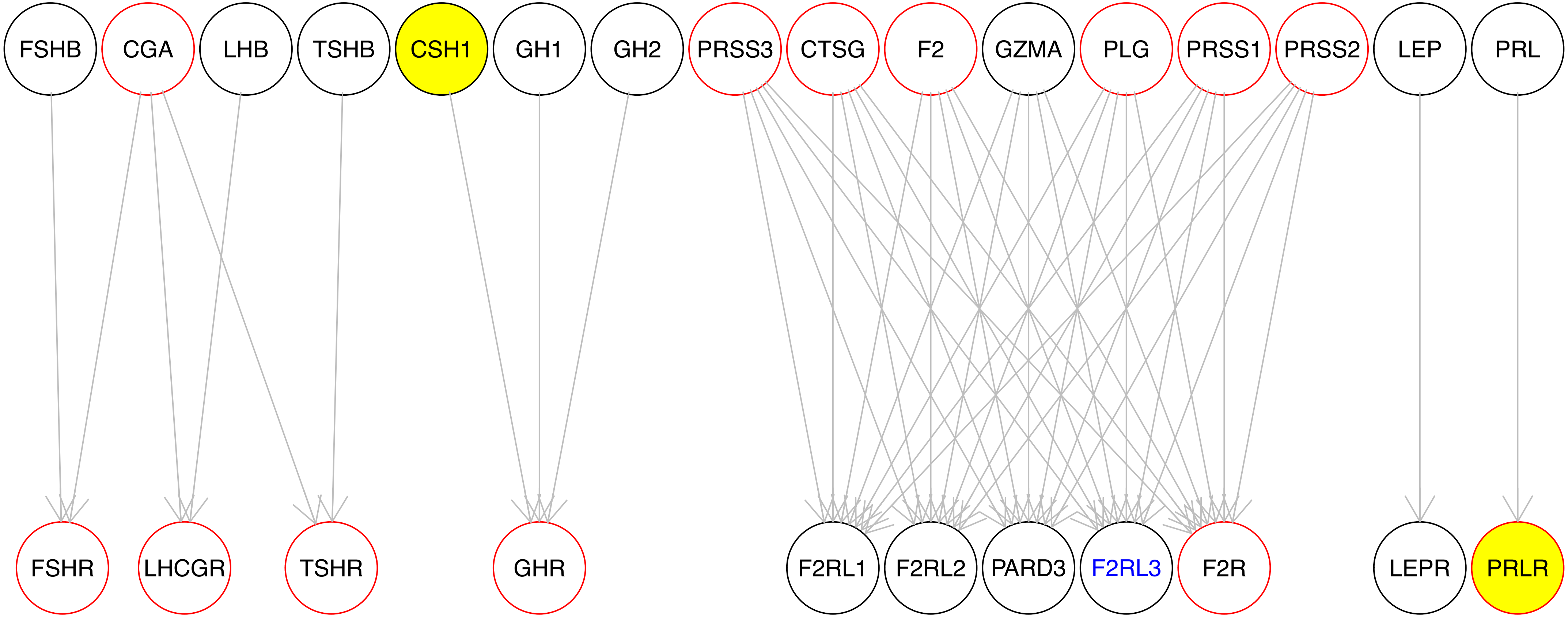

KEGG pathway = Neuroactive ligand–receptor interaction : tumour = MEL.JWCI.WGS.36.Tumor : Yellow Fill = gene variant, Blue Text = expression–survival association, Red Boarder = drug

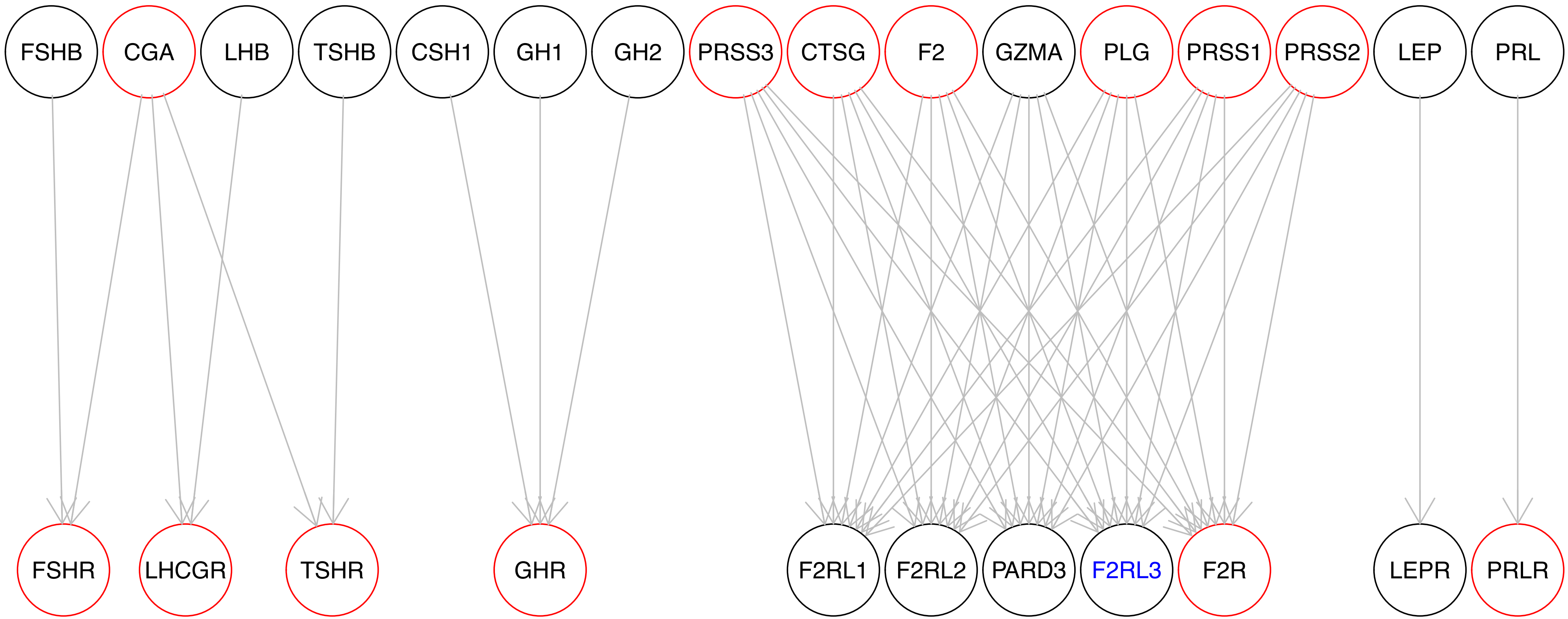

KEGG pathway = Neuroactive ligand–receptor interaction : tumour = MEL.JWCI.WGS.37.Tumor : Yellow Fill = gene variant, Blue Text = expression–survival association, Red Boarder = drug

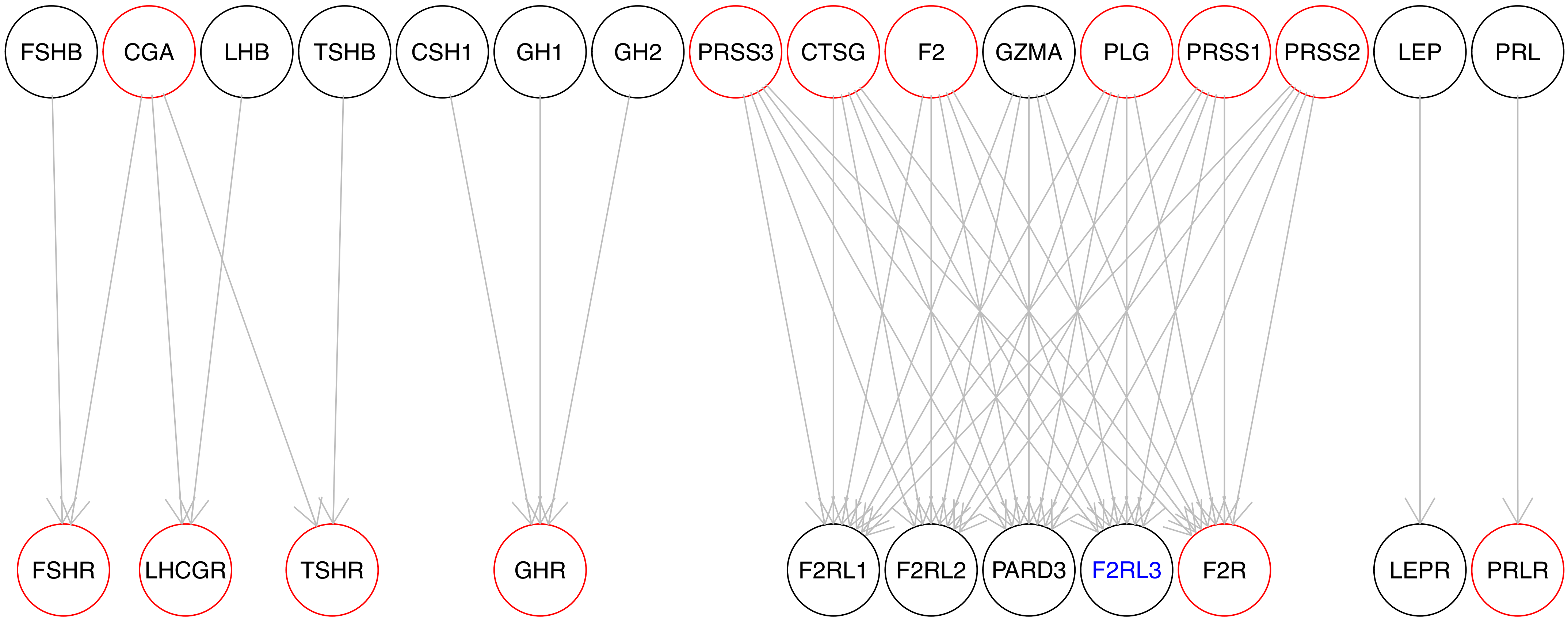

KEGG pathway = Neuroactive ligand–receptor interaction : tumour = MEL.JWCI.WGS.38.Tumor : Yellow Fill = gene variant, Blue Text = expression–survival association, Red Boarder = drug

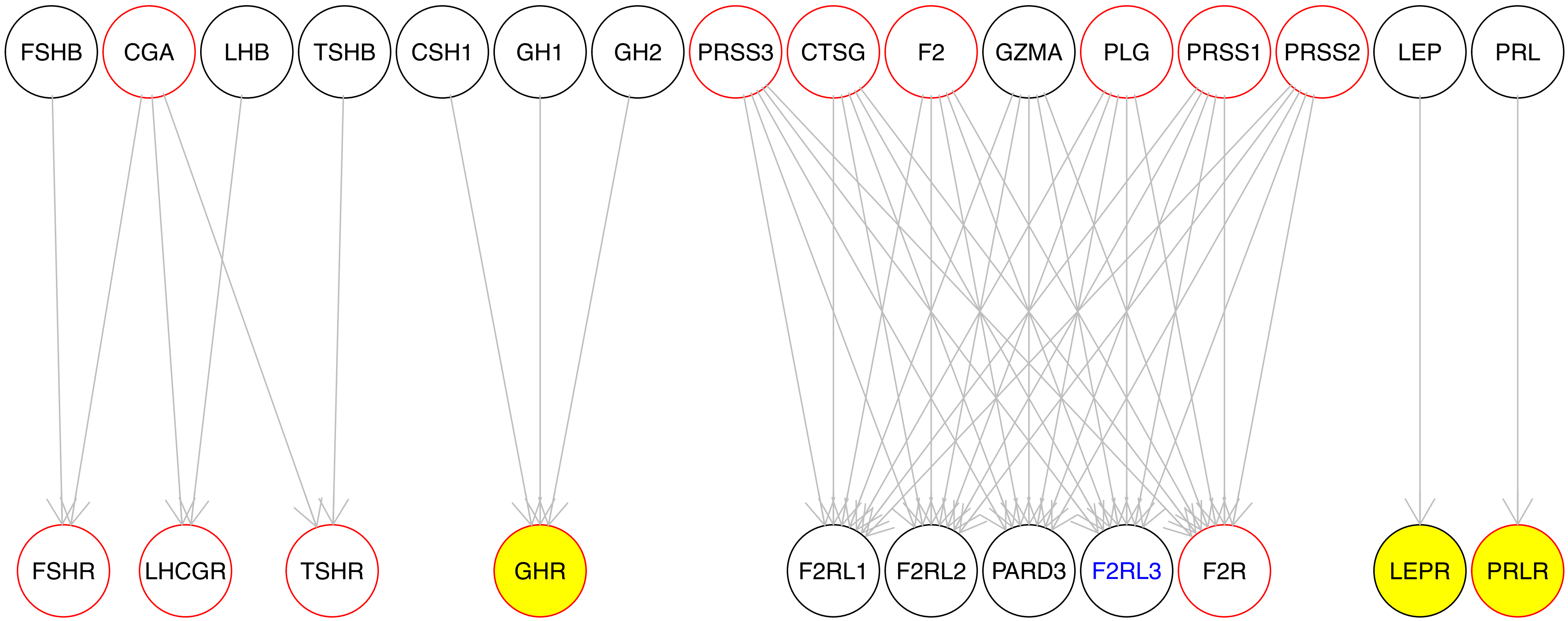

KEGG pathway = Neuroactive ligand−receptor interaction : tumour = MEL.JWCI.WGS.39.Tumor : Yellow Fill = gene variant, Blue Text = expression−survival association, Red Boarder = drug

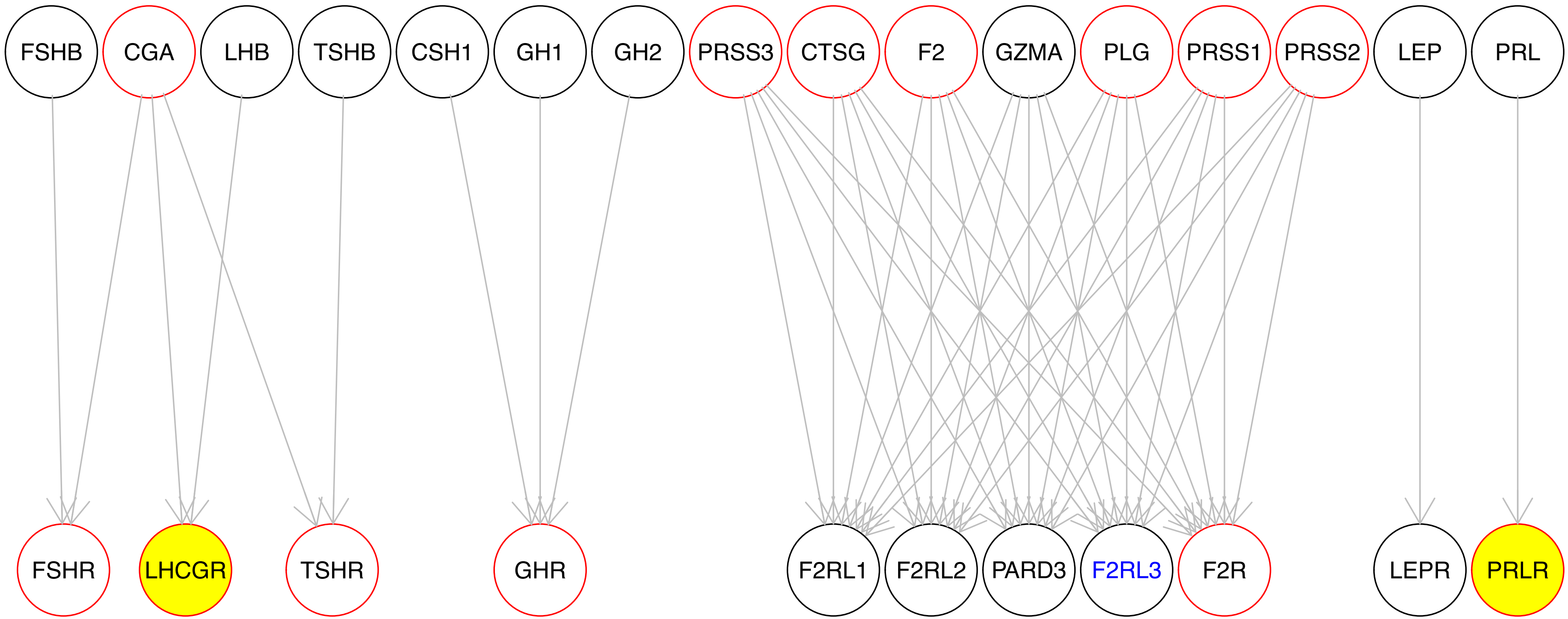

KEGG pathway = Neuroactive ligand−receptor interaction : tumour = MEL.JWCI.WGS.3.Tumor : Yellow Fill = gene variant, Blue Text = expression−survival association, Red Boarder = drug

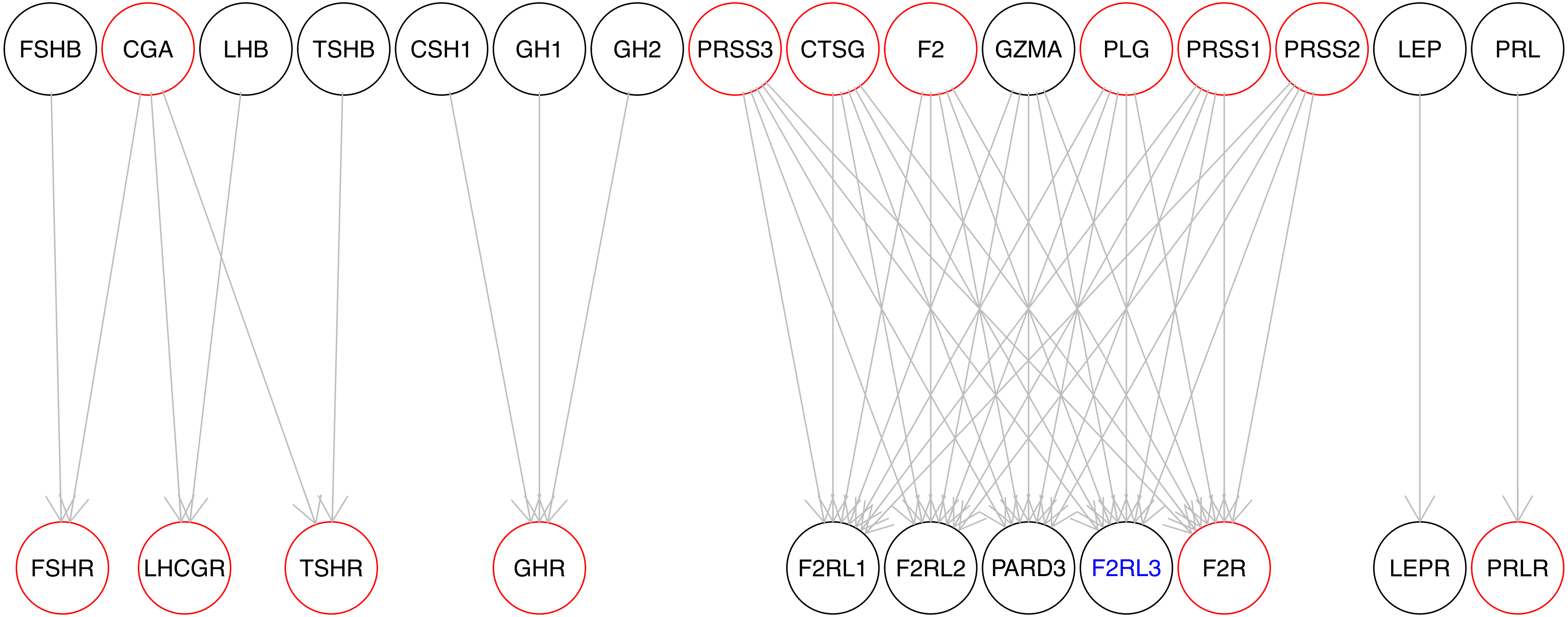

KEGG pathway = Neuroactive ligand−receptor interaction : tumour = MEL.JWCI.WGS.42.Tumor : Yellow Fill = gene variant, Blue Text = expression−survival association, Red Boarder = drug

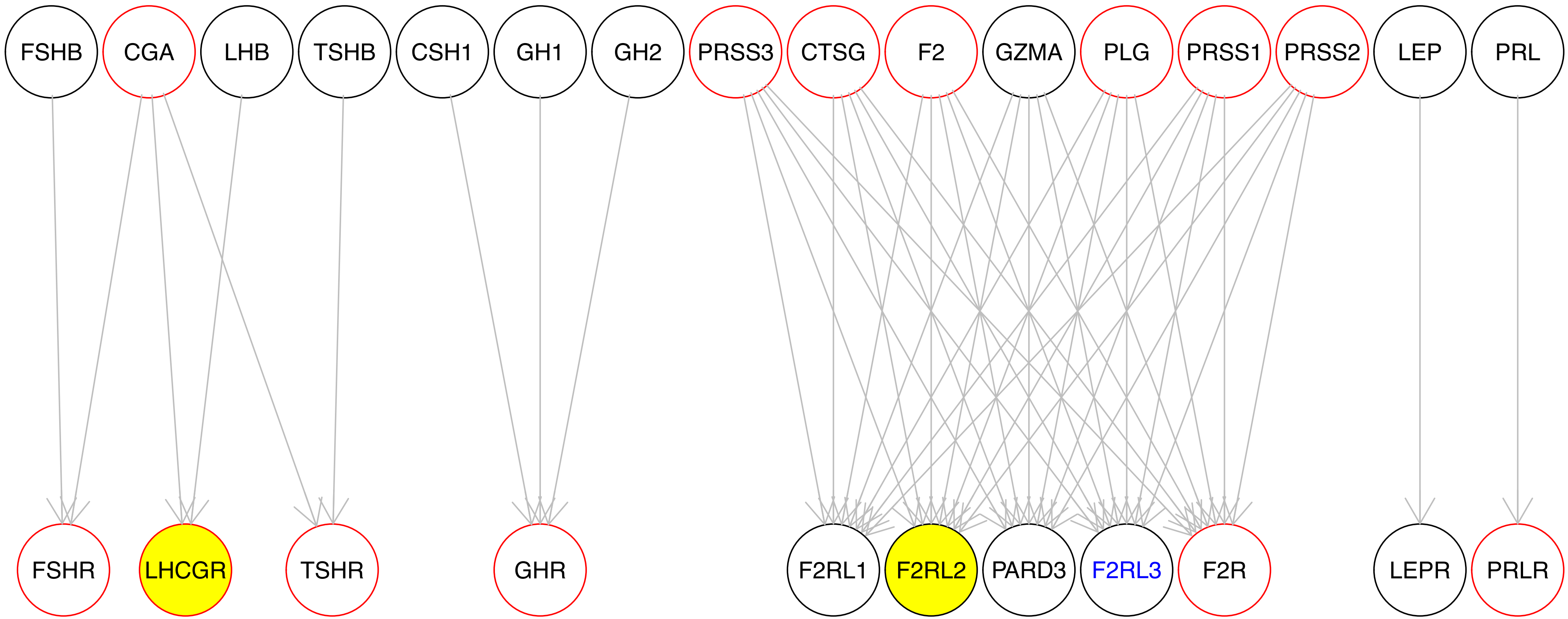

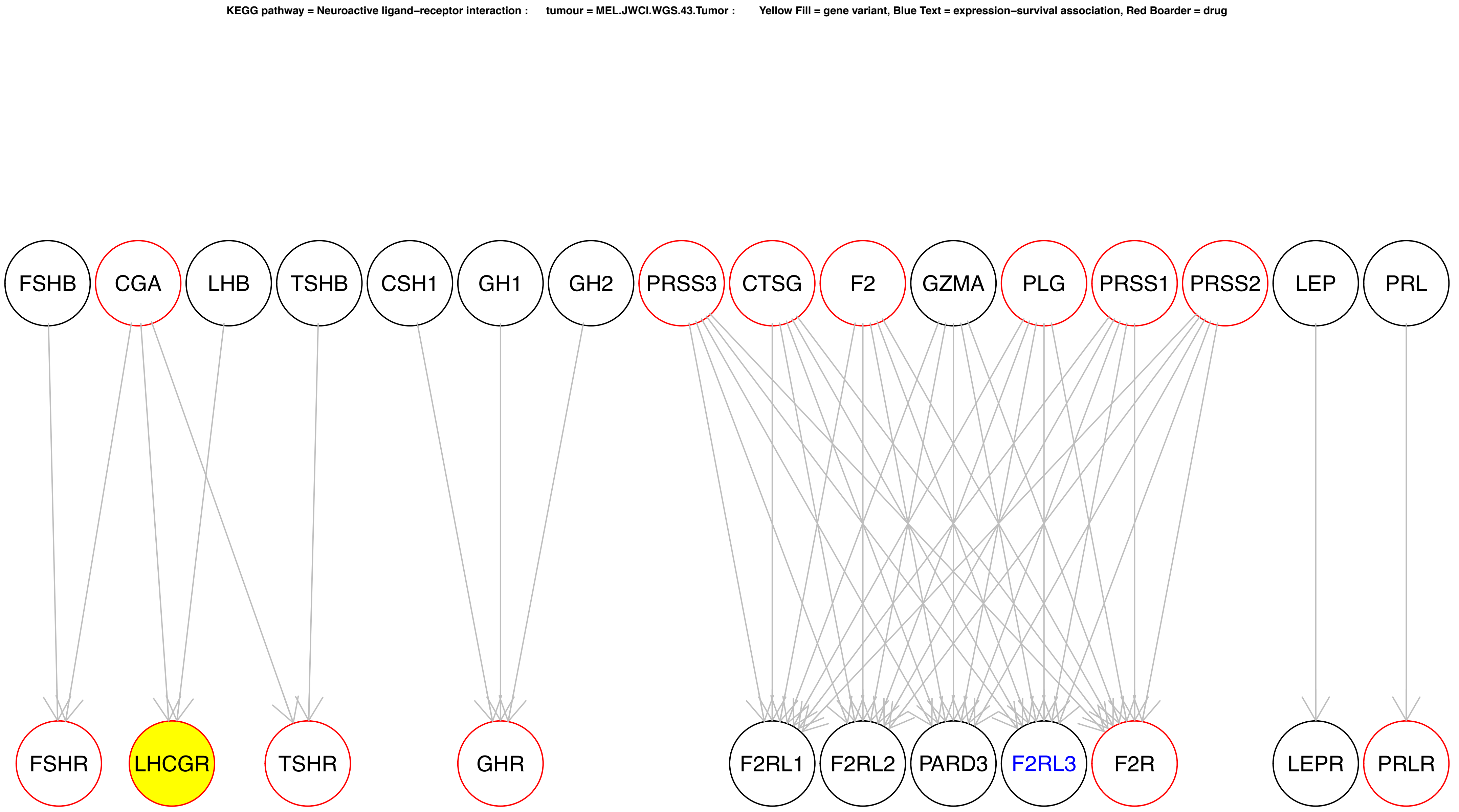
KEGG pathway = Neuroactive ligand−receptor interaction : tumour = MEL.JWCI.WGS.43.Tumor : Yellow Fill = gene variant, Blue Text = expression−survival association, Red Boarder = drug
FSHB
CGA
LHB
TSHB
CSH1
GH1
GH2
PRSS3
CTSG
F2
GZMA
PLG
PRSS1
PRSS2
LEP
PRL
FSHR
LHCGR
TSHR
GHR
F2RL1
F2RL2
PARD3
F2RL3
F2R
LEPR
PRLR

KEGG pathway = Neuroactive ligand−receptor interaction : tumour = MEL.JWCI.WGS.4.Tumor : Yellow Fill = gene variant, Blue Text = expression−survival association, Red Boarder = drug

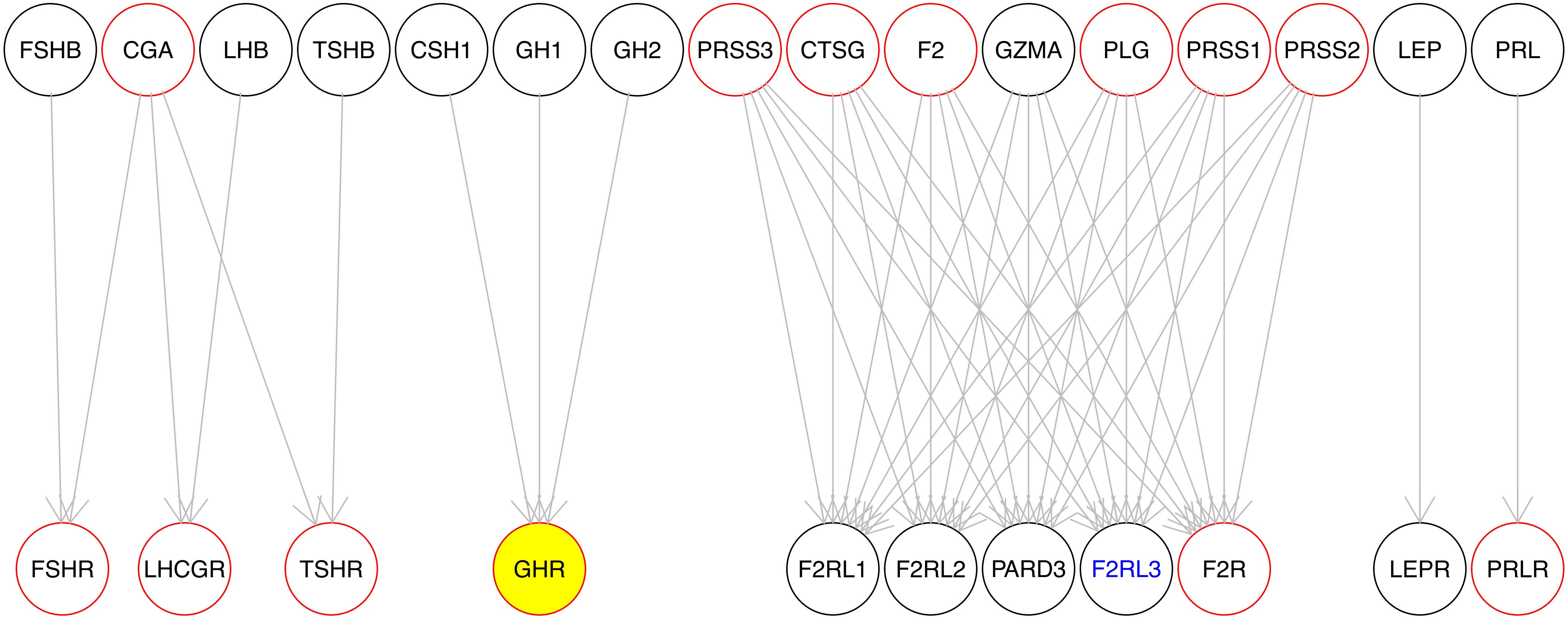

KEGG pathway = Neuroactive ligand−receptor interaction : tumour = MEL.JWCI.WGS.5.Tumor : Yellow Fill = gene variant, Blue Text = expression−survival association, Red Boarder = drug

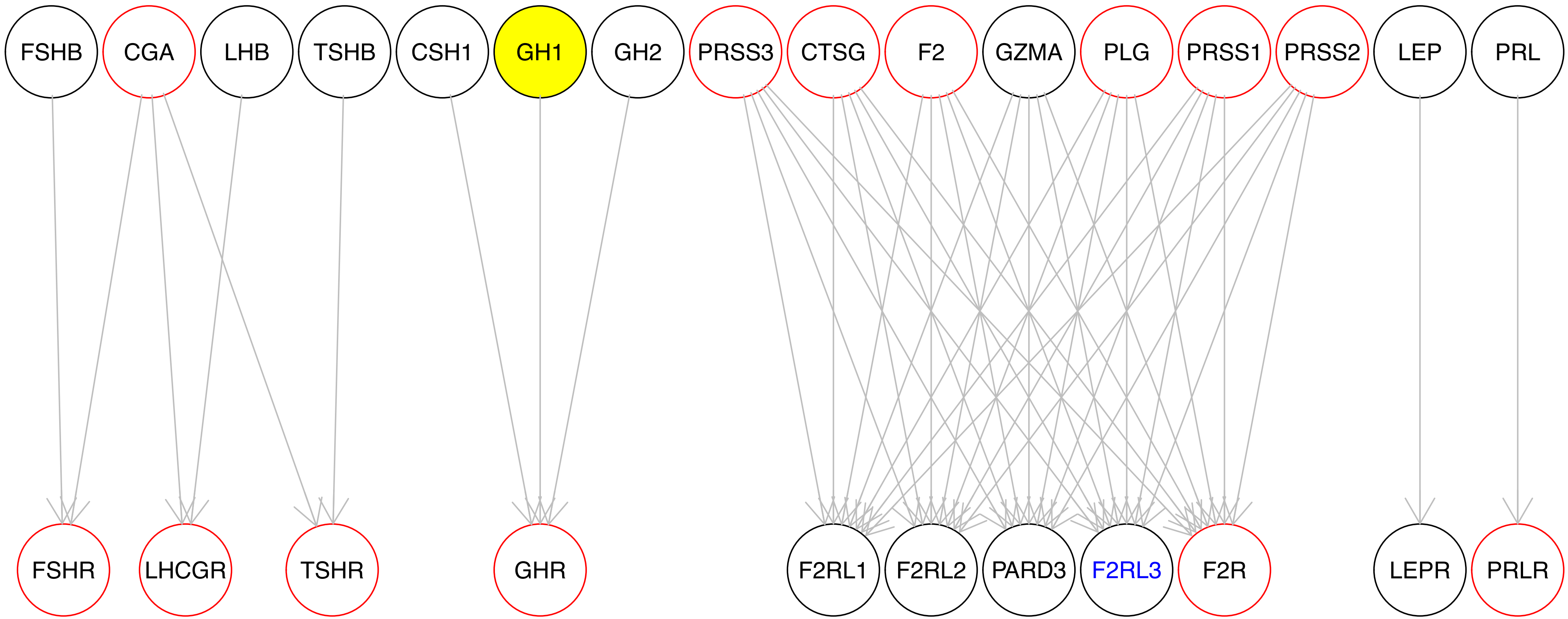

KEGG pathway = Neuroactive ligand−receptor interaction :   tumour = MEL.JWCI.WGS.6.Tumor :   Yellow Fill = gene variant, Blue Text = expression−survival association, Red Boarder = drug

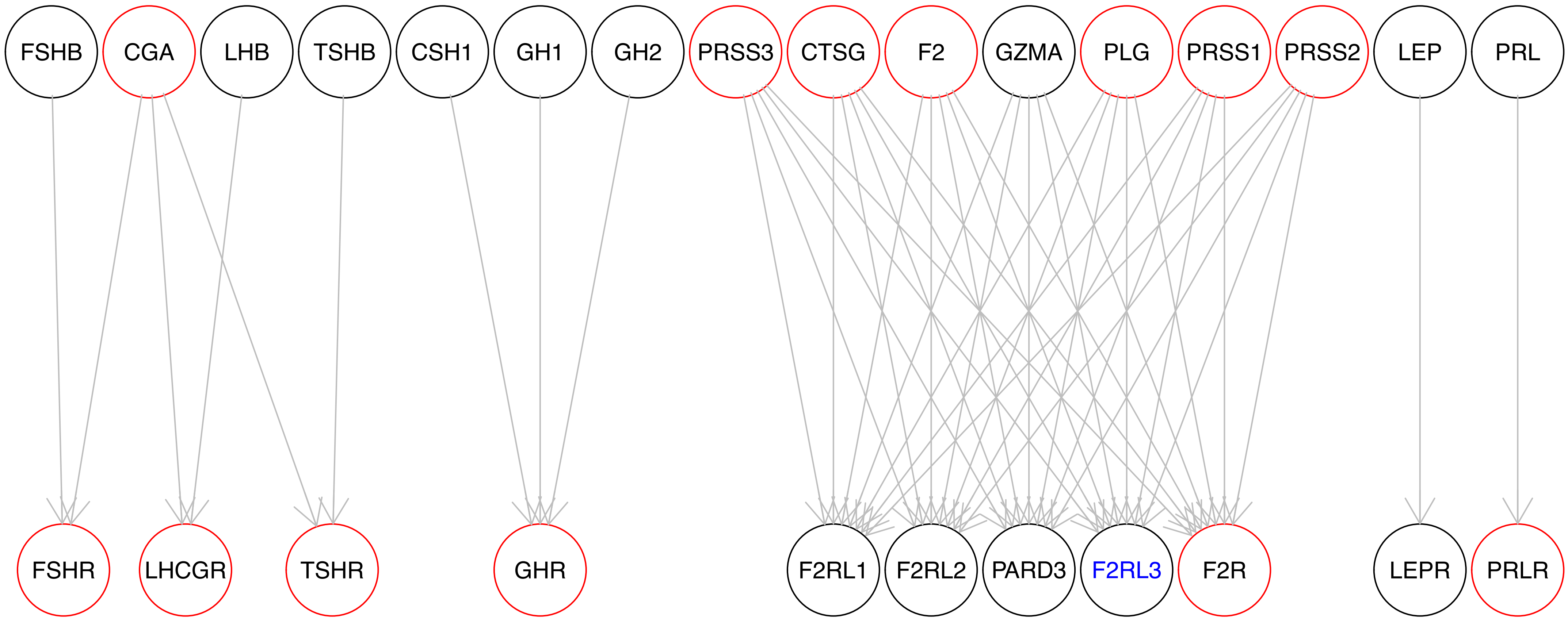

KEGG pathway = Neuroactive ligand−receptor interaction : tumour = MEL.JWCI.WGS.7.Tumor : Yellow Fill = gene variant, Blue Text = expression−survival association, Red Boarder = drug

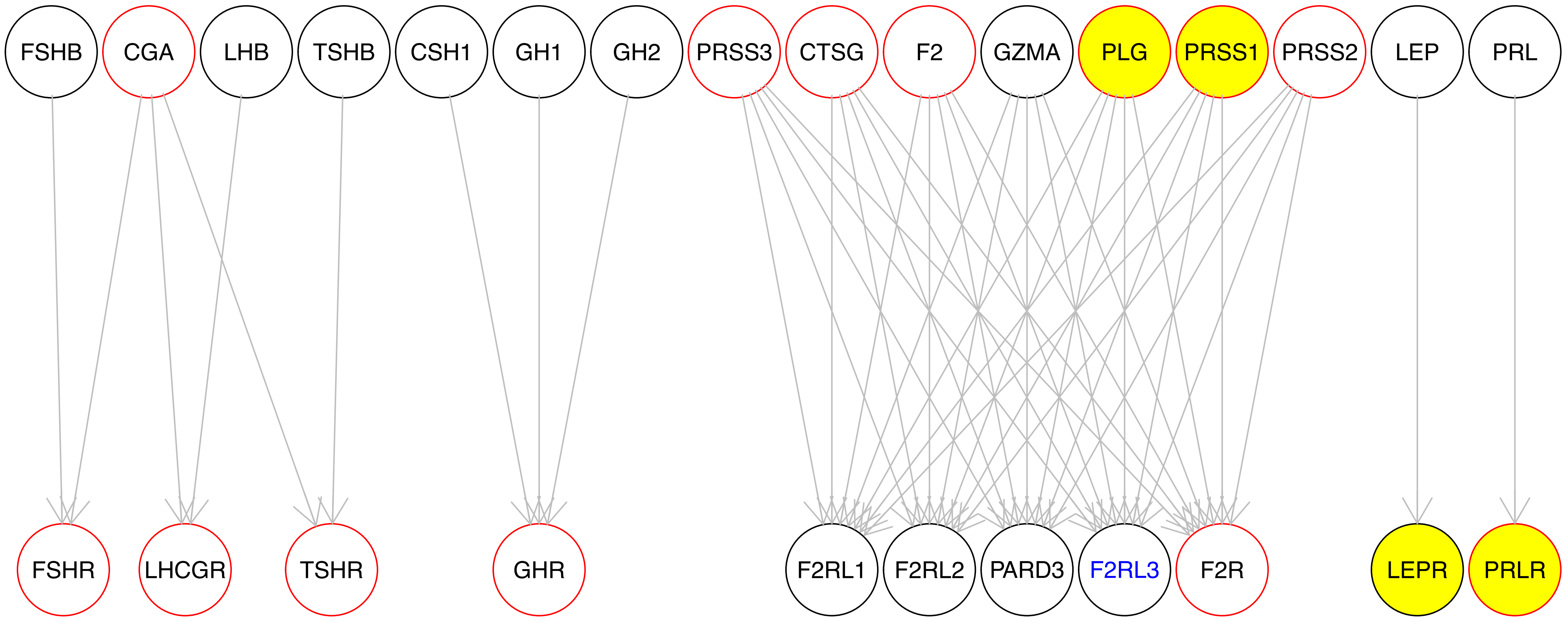

KEGG pathway = Neuroactive ligand−receptor interaction : tumour = MEL.JWCI.WGS.8.Tumor : Yellow Fill = gene variant, Blue Text = expression−survival association, Red Boarder = drug

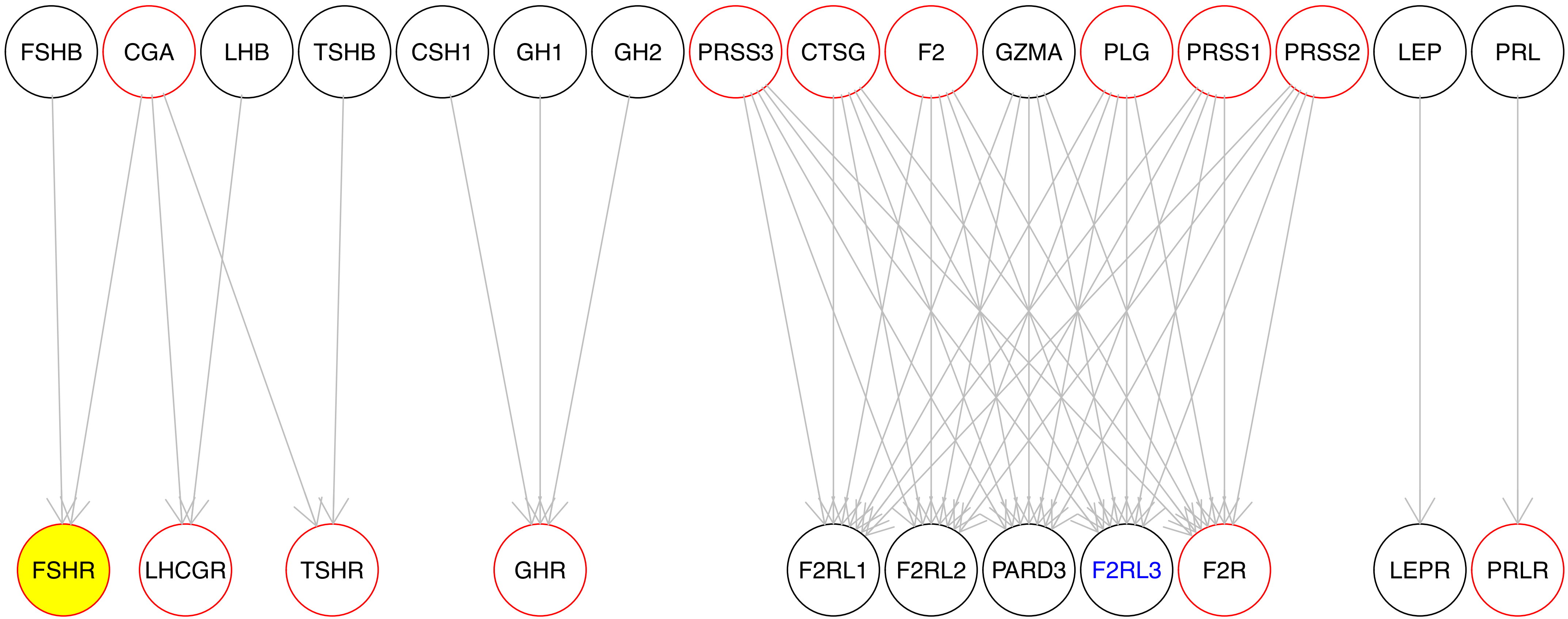

KEGG pathway = Neuroactive ligand−receptor interaction : tumour = MEL.Ma.Mel.04.Tumor : Yellow Fill = gene variant, Blue Text = expression−survival association, Red Boarder = drug

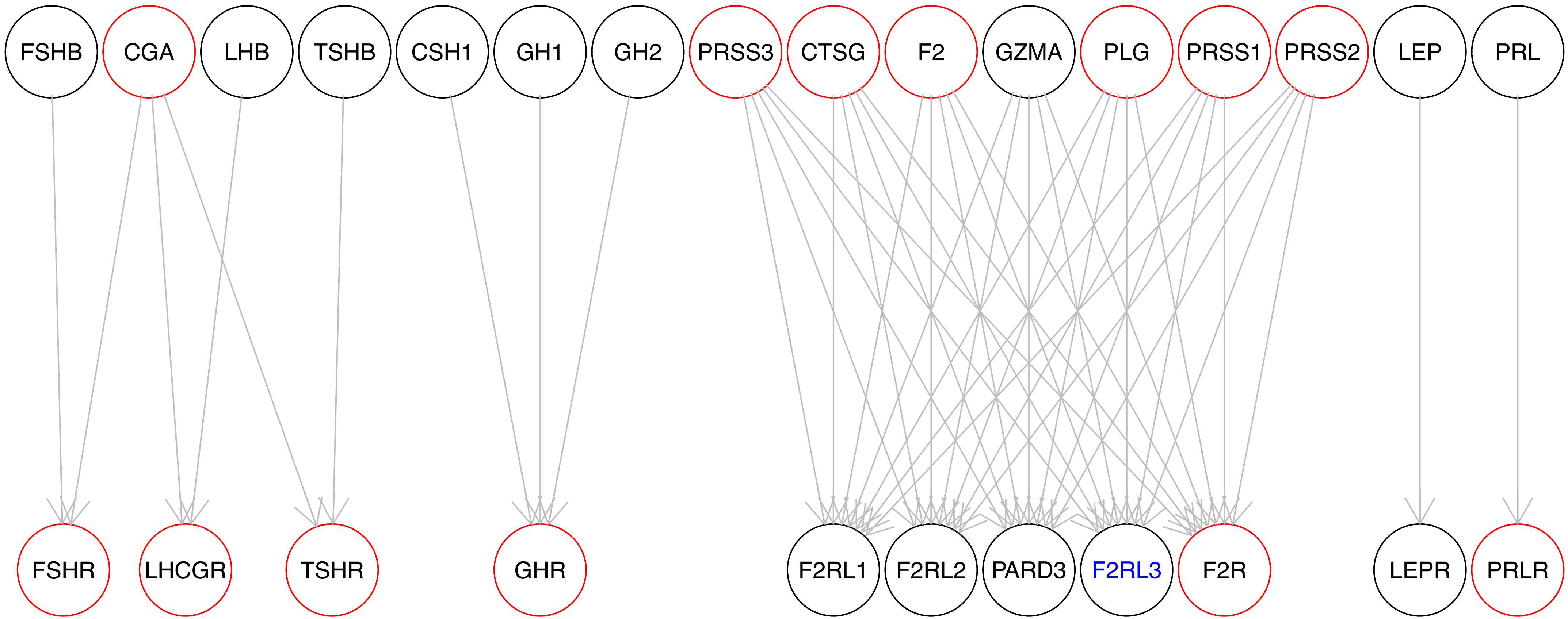

KEGG pathway = Neuroactive ligand−receptor interaction :   tumour = MEL.Ma.Mel.05.Tumor :   Yellow Fill = gene variant, Blue Text = expression−survival association, Red Boarder = drug

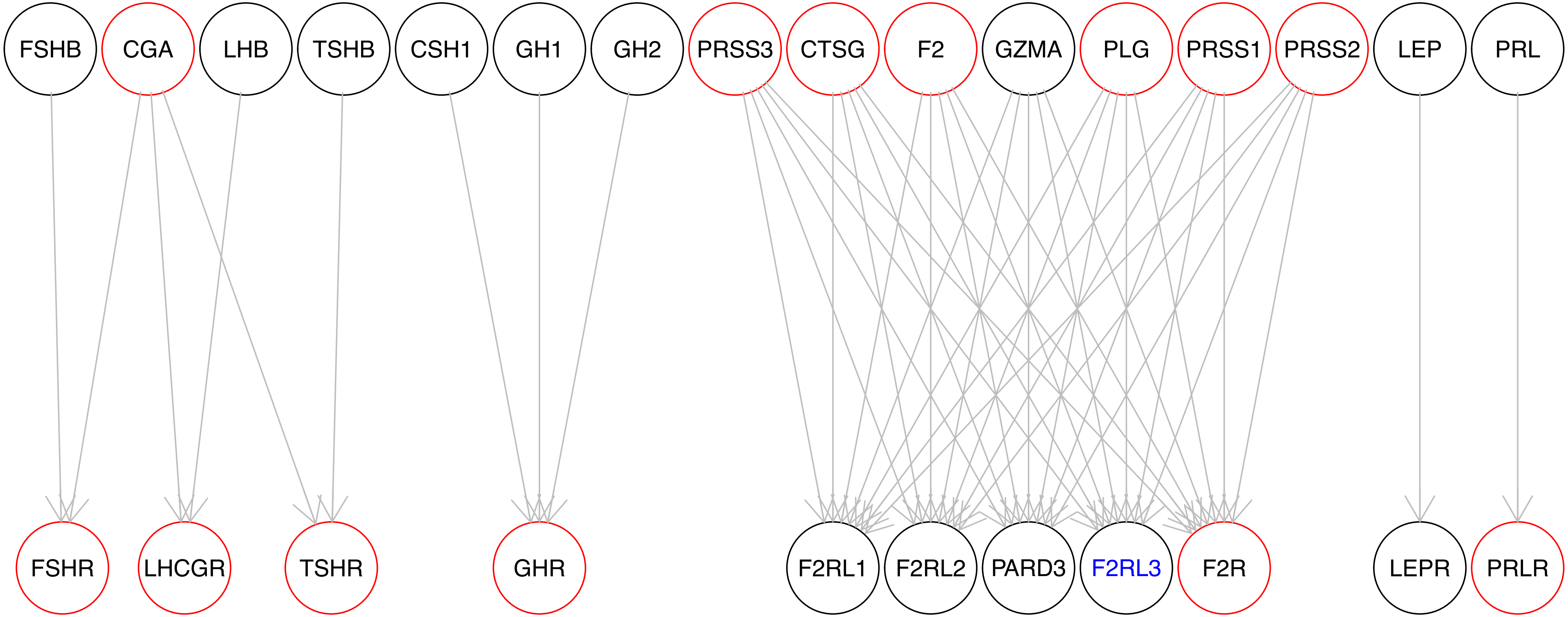

KEGG pathway = Neuroactive ligand−receptor interaction : tumour = MEL.Ma.Mel.08a.Tumor : Yellow Fill = gene variant, Blue Text = expression−survival association, Red Boarder = drug

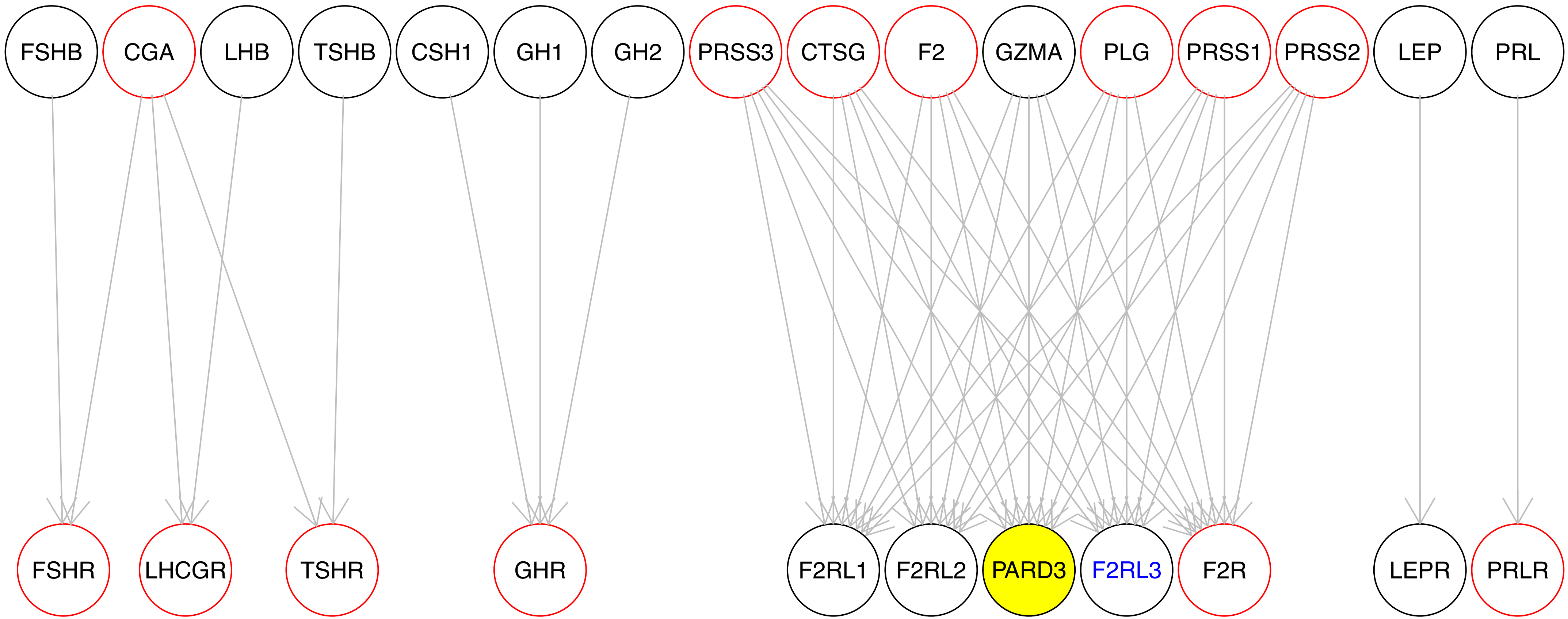

KEGG pathway = Neuroactive ligand−receptor interaction : tumour = MEL.Ma.Mel.102.Tumor : Yellow Fill = gene variant, Blue Text = expression−survival association, Red Boarder = drug

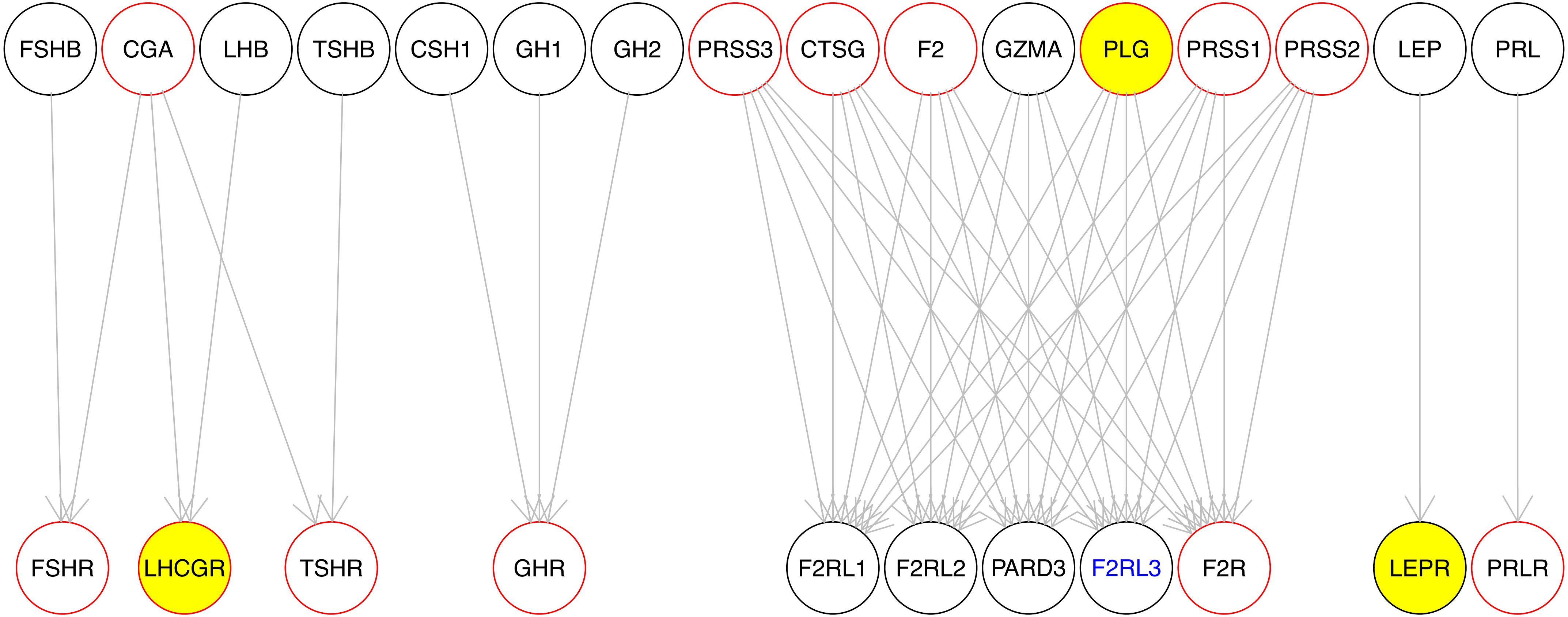

KEGG pathway = Neuroactive ligand−receptor interaction :   tumour = MEL.Ma.Mel.103b.Tumor :   Yellow Fill = gene variant, Blue Text = expression−survival association, Red Boarder = drug

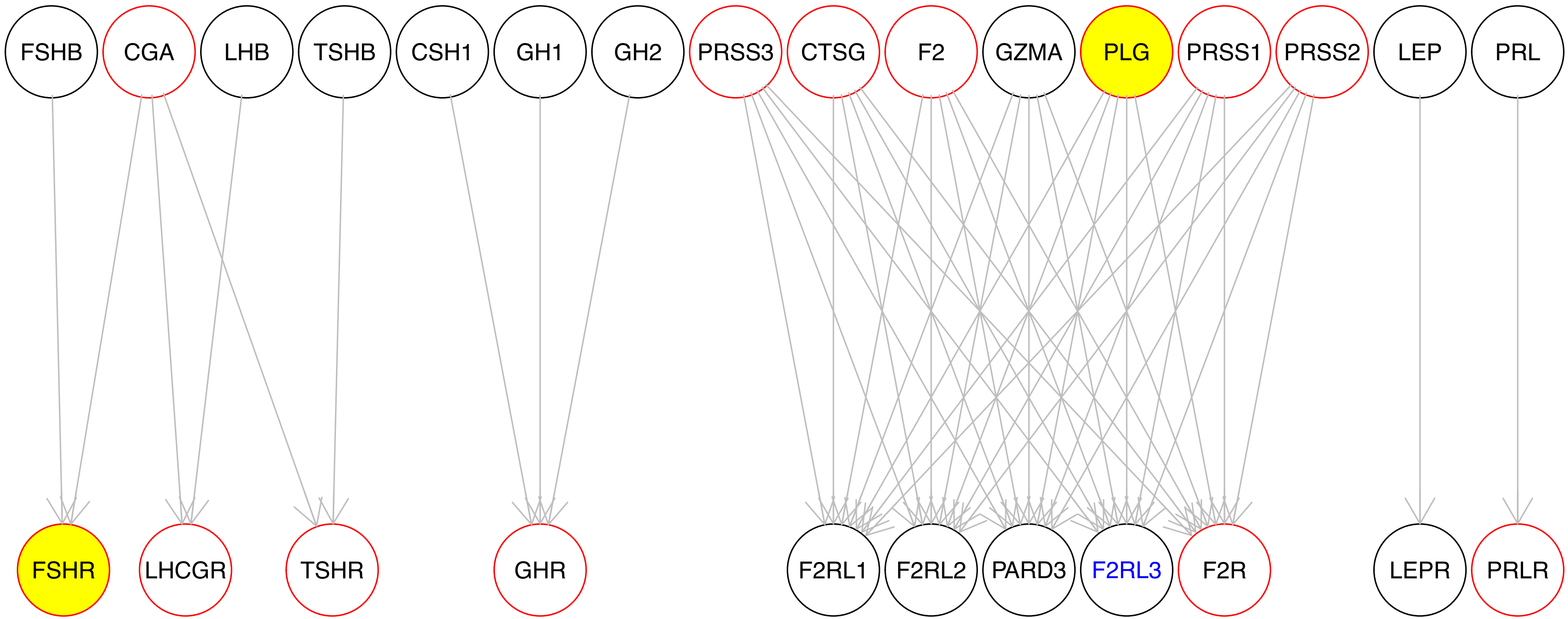

KEGG pathway = Neuroactive ligand−receptor interaction : tumour = MEL.Ma.Mel.105.Tumor : Yellow Fill = gene variant, Blue Text = expression−survival association, Red Boarder = drug

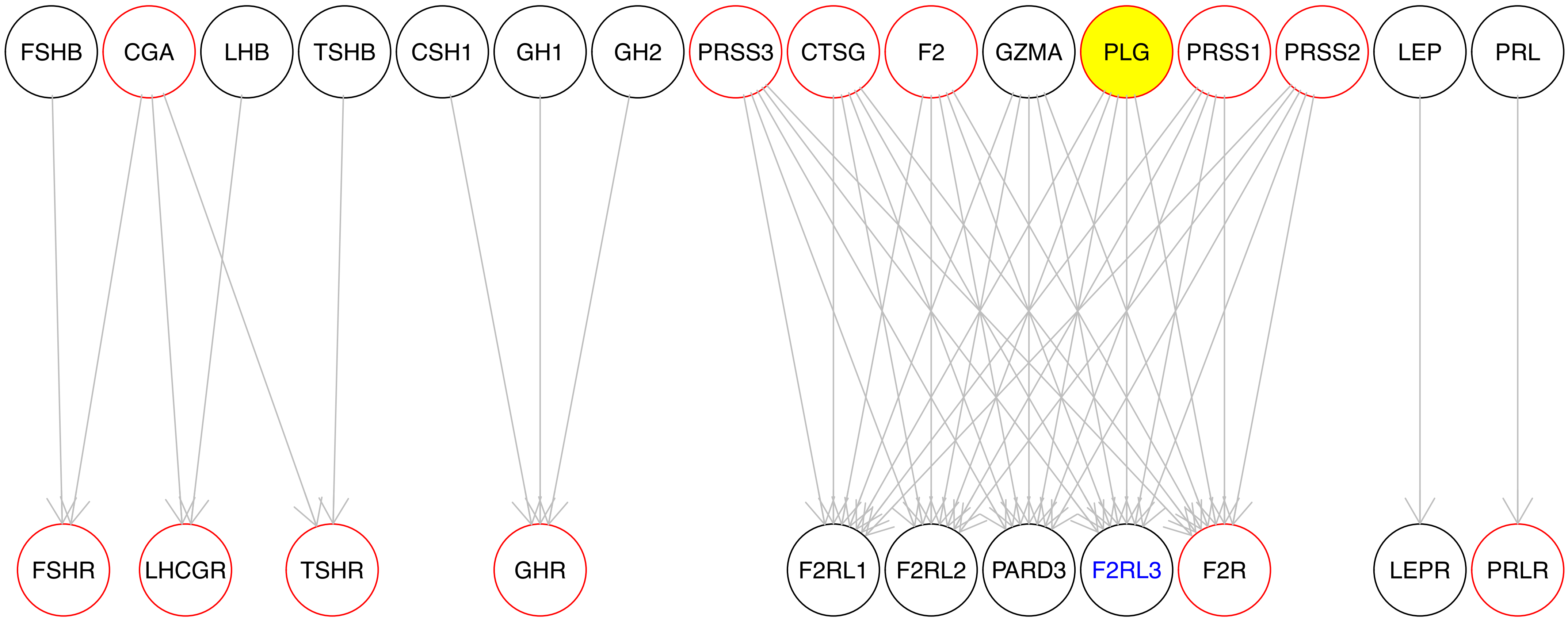

KEGG pathway = Neuroactive ligand−receptor interaction :   tumour = MEL.Ma.Mel.107.Tumor :   Yellow Fill = gene variant, Blue Text = expression−survival association, Red Boarder = drug

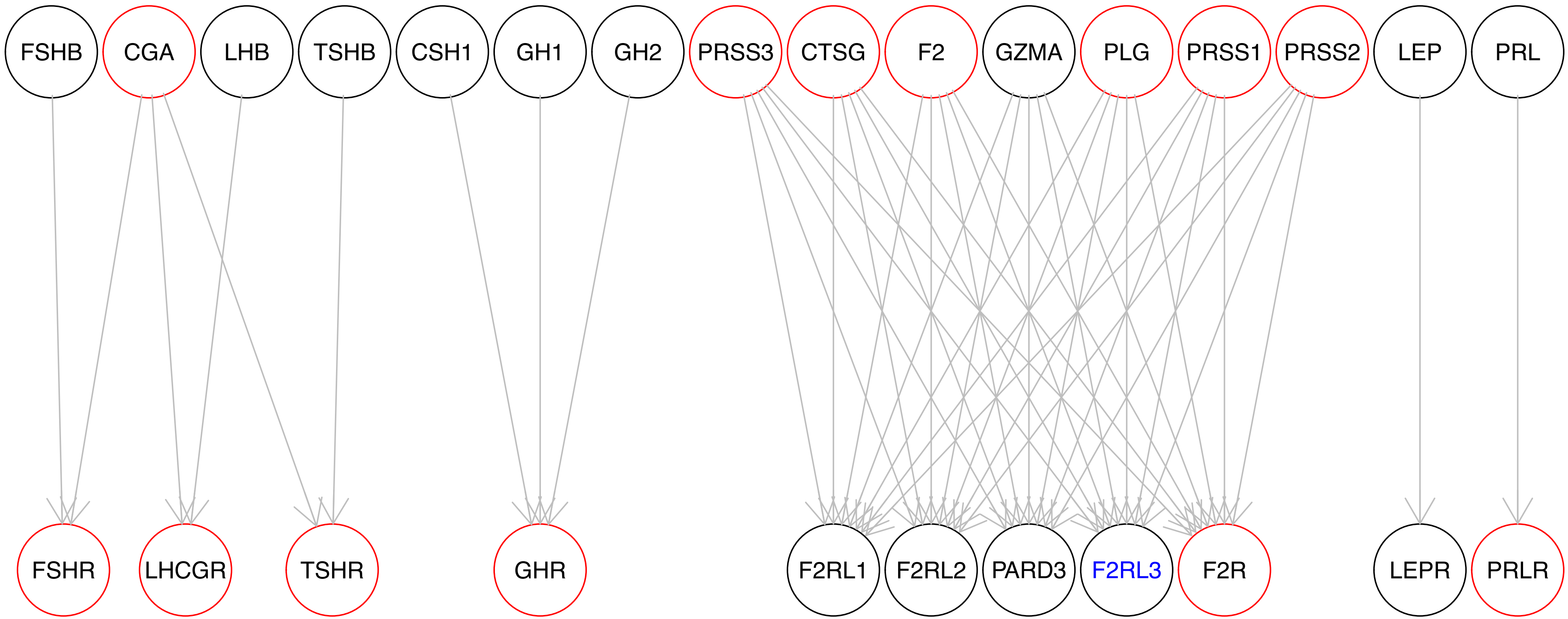

KEGG pathway = Neuroactive ligand−receptor interaction : tumour = MEL.Ma.Mel.108.Tumor : Yellow Fill = gene variant, Blue Text = expression−survival association, Red Boarder = drug

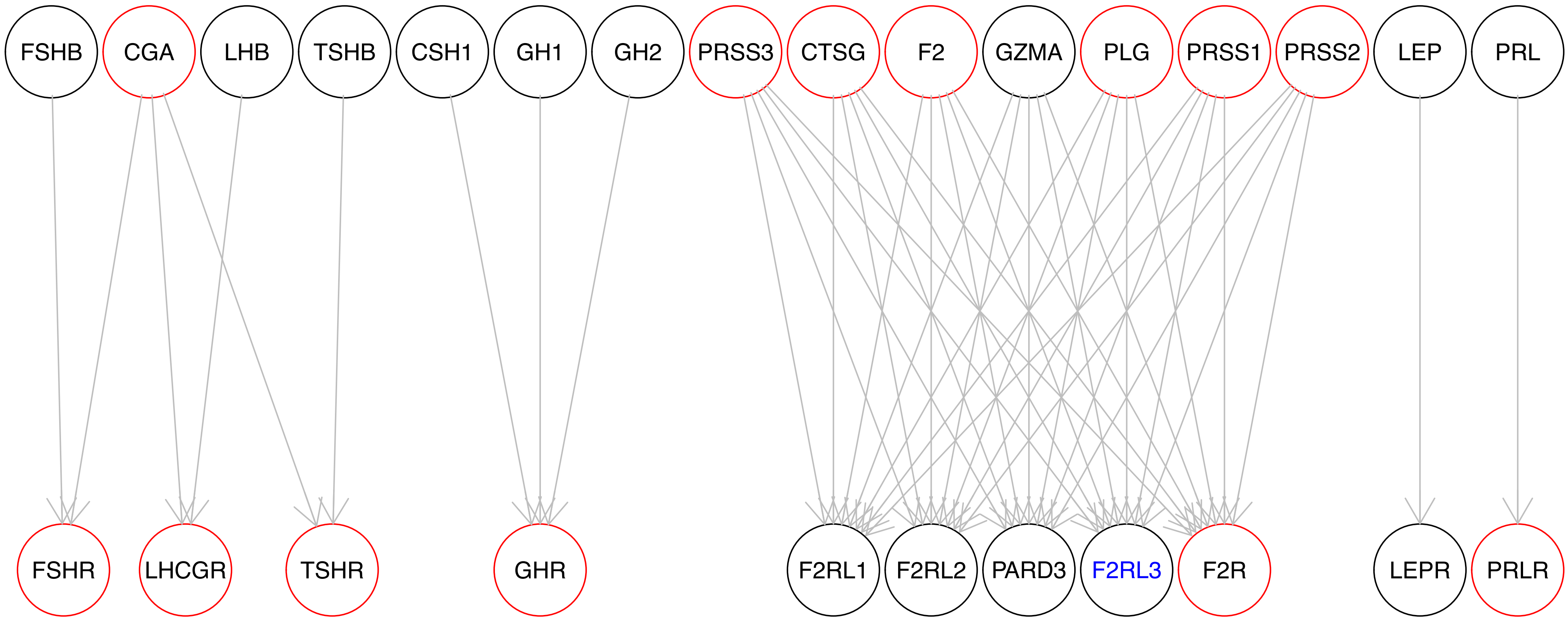

KEGG pathway = Neuroactive ligand−receptor interaction : tumour = MEL.Ma.Mel.114.Tumor : Yellow Fill = gene variant, Blue Text = expression−survival association, Red Boarder = drug

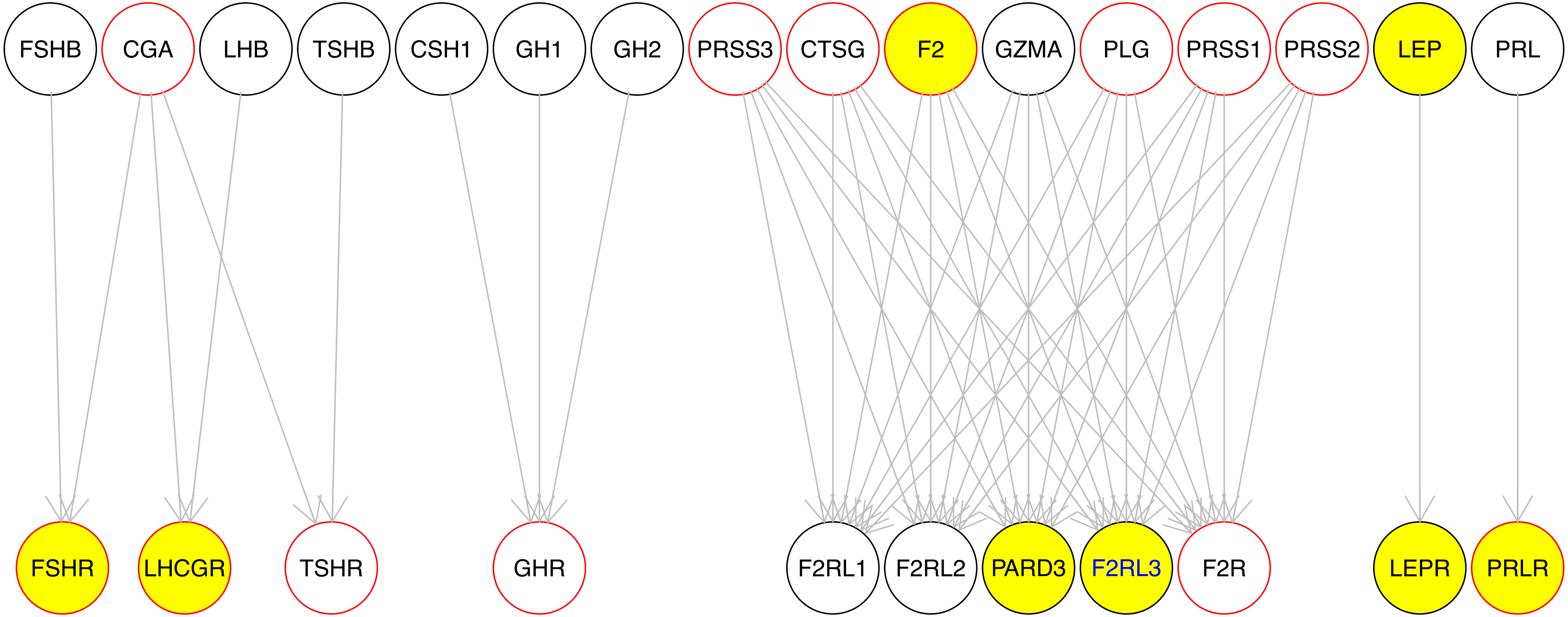

KEGG pathway = Neuroactive ligand−receptor interaction : tumour = MEL.Ma.Mel.119.Tumor : Yellow Fill = gene variant, Blue Text = expression−survival association, Red Boarder = drug

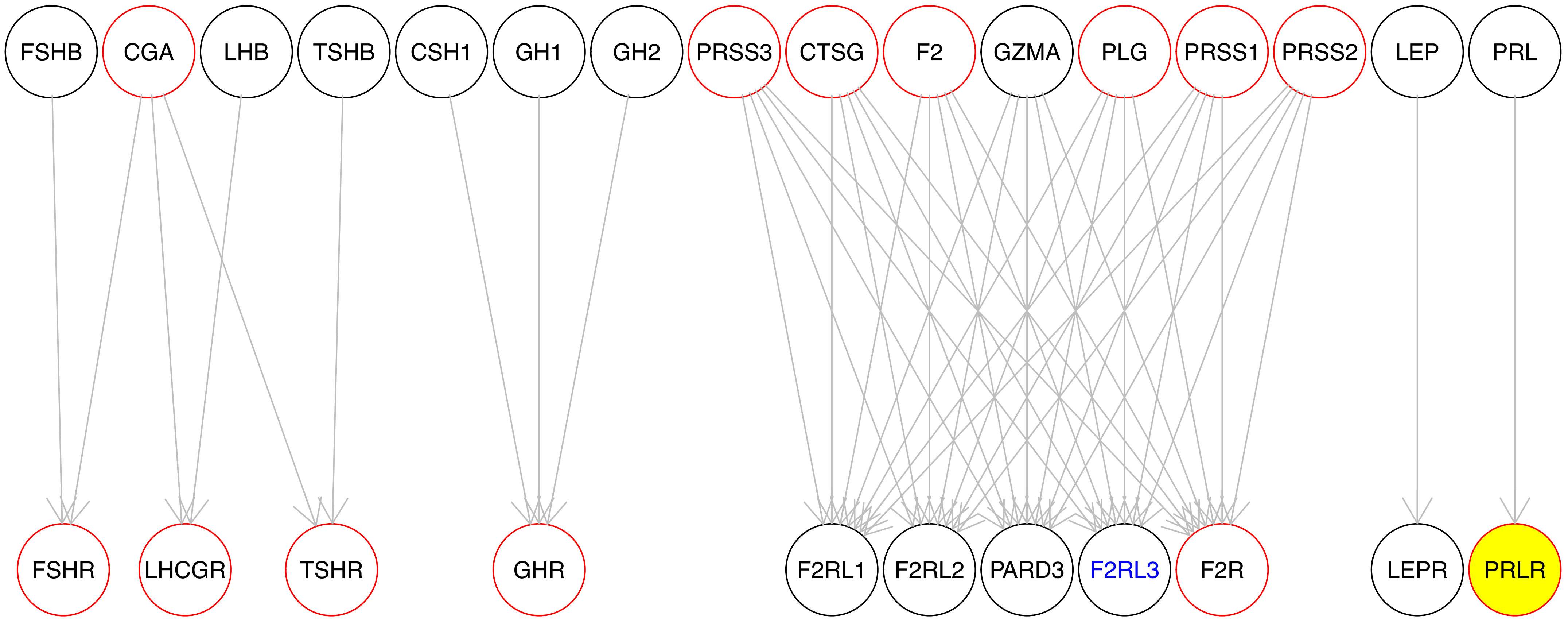

KEGG pathway = Neuroactive ligand−receptor interaction : tumour = MEL.Ma.Mel.120.Tumor : Yellow Fill = gene variant, Blue Text = expression−survival association, Red Boarder = drug

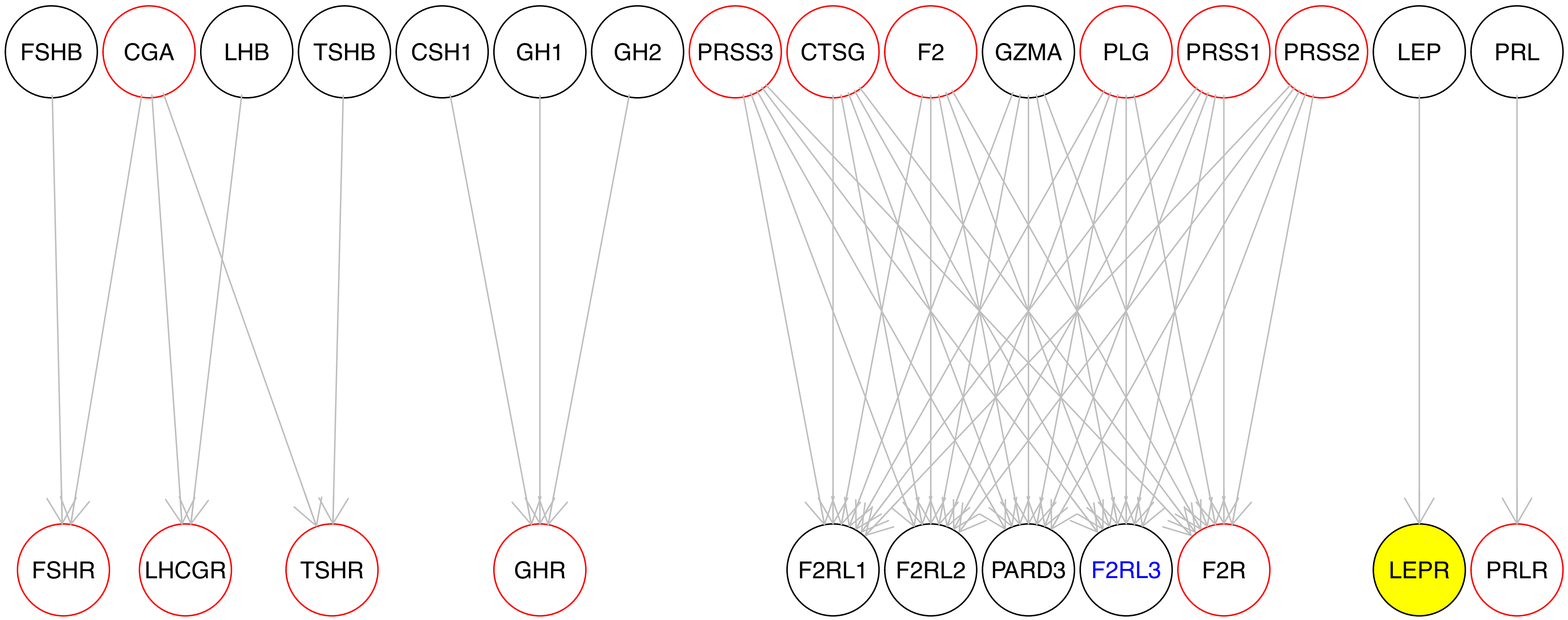

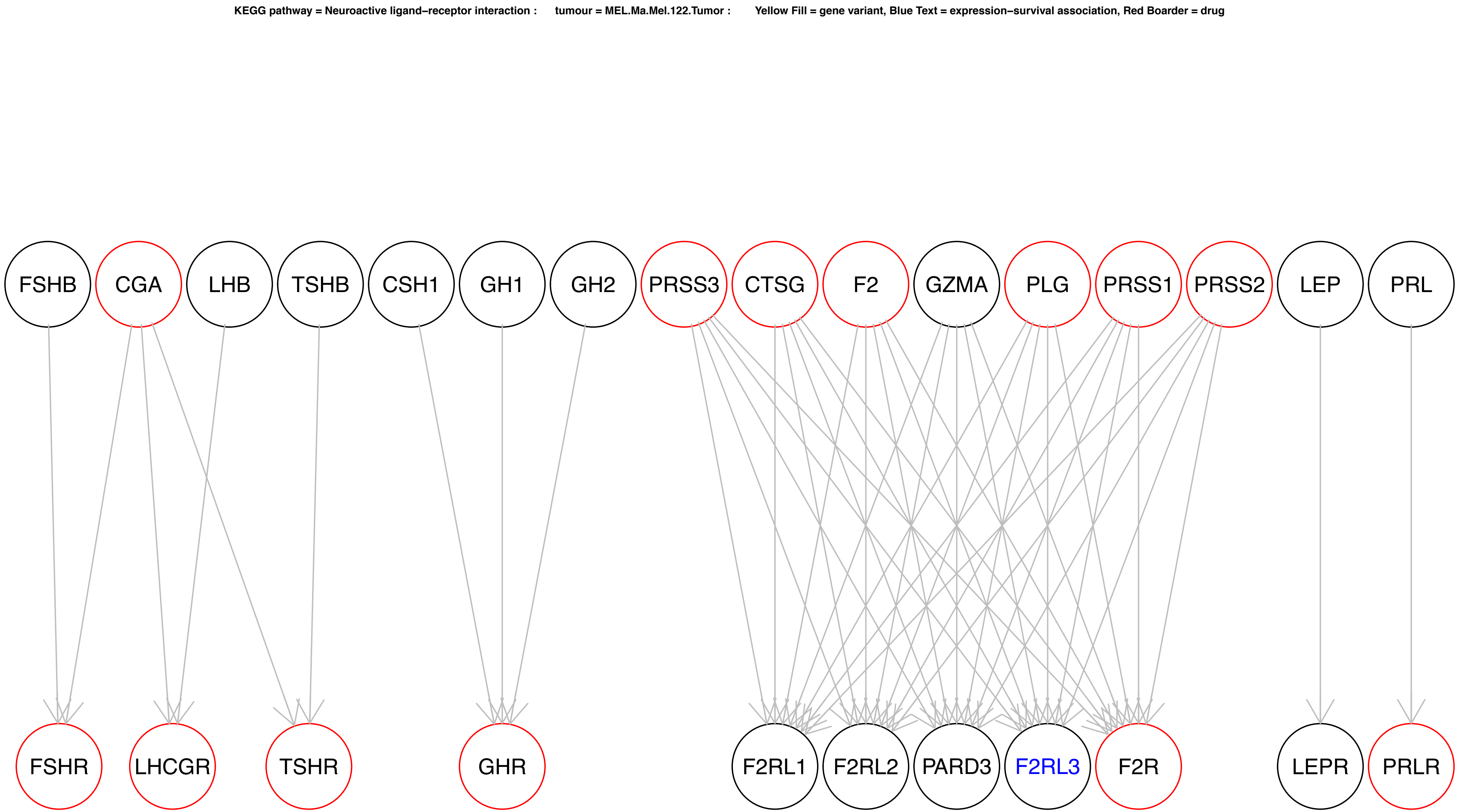
KEGG pathway = Neuroactive ligand−receptor interaction : tumour = MEL.Ma.Mel.122.Tumor : Yellow Fill = gene variant, Blue Text = expression−survival association, Red Boarder = drug
FSHB
CGA
LHB
TSHB
CSH1
GH1
GH2
PRSS3
CTSG
F2
GZMA
PLG
PRSS1
PRSS2
LEP
PRL
FSHR
LHCGR
TSHR
GHR
F2RL1
F2RL2
PARD3
F2RL3
F2R
LEPR
PRLR

KEGG pathway = Neuroactive ligand−receptor interaction : tumour = MEL.Ma.Mel.123.Tumor : Yellow Fill = gene variant, Blue Text = expression−survival association, Red Boarder = drug

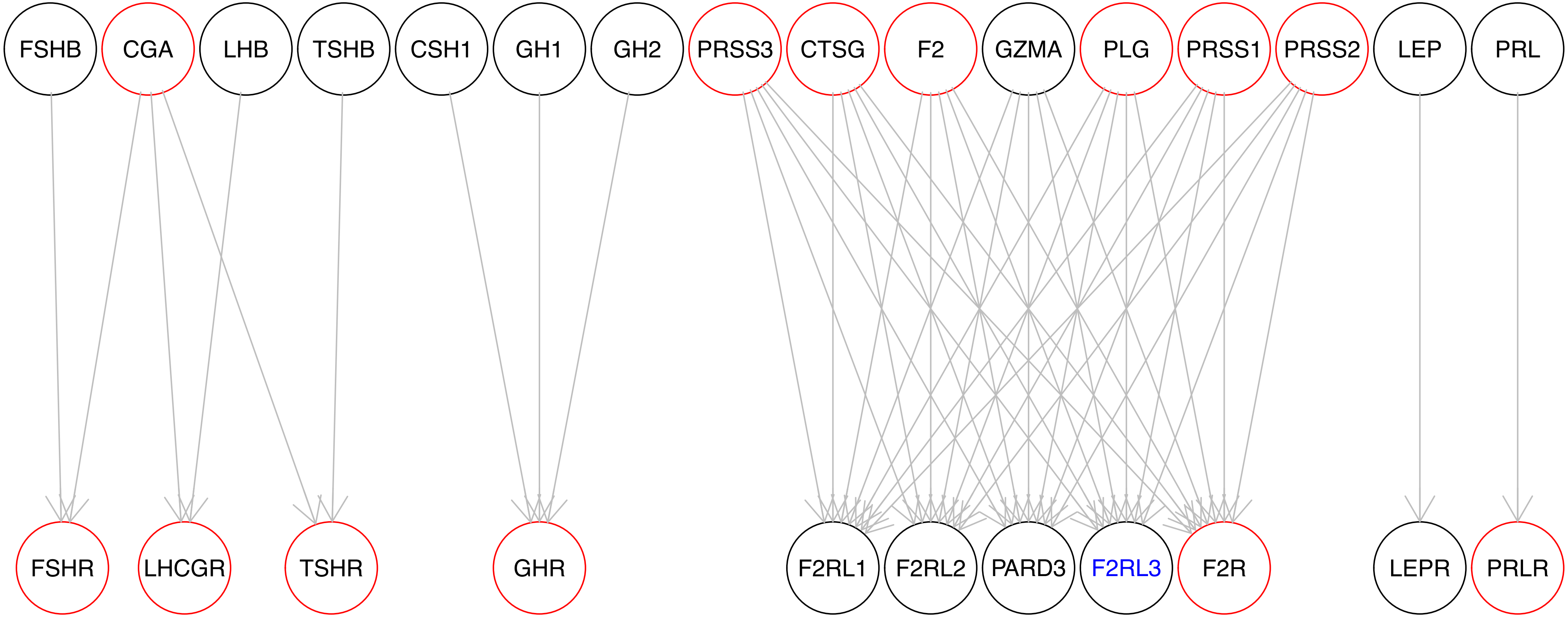

KEGG pathway = Neuroactive ligand−receptor interaction : tumour = MEL.Ma.Mel.15.Tumor : Yellow Fill = gene variant, Blue Text = expression−survival association, Red Boarder = drug

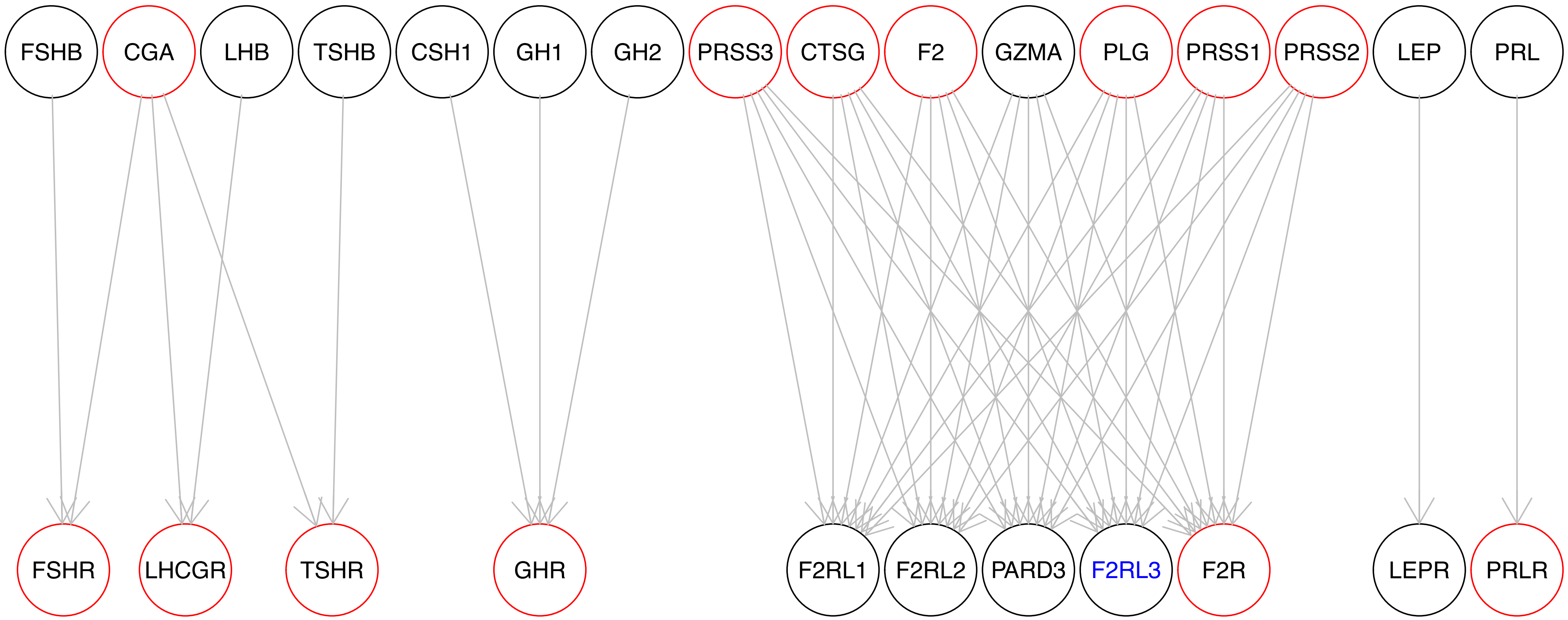

KEGG pathway = Neuroactive ligand−receptor interaction :   tumour = MEL.Ma.Mel.16.Tumor :   Yellow Fill = gene variant, Blue Text = expression−survival association, Red Boarder = drug

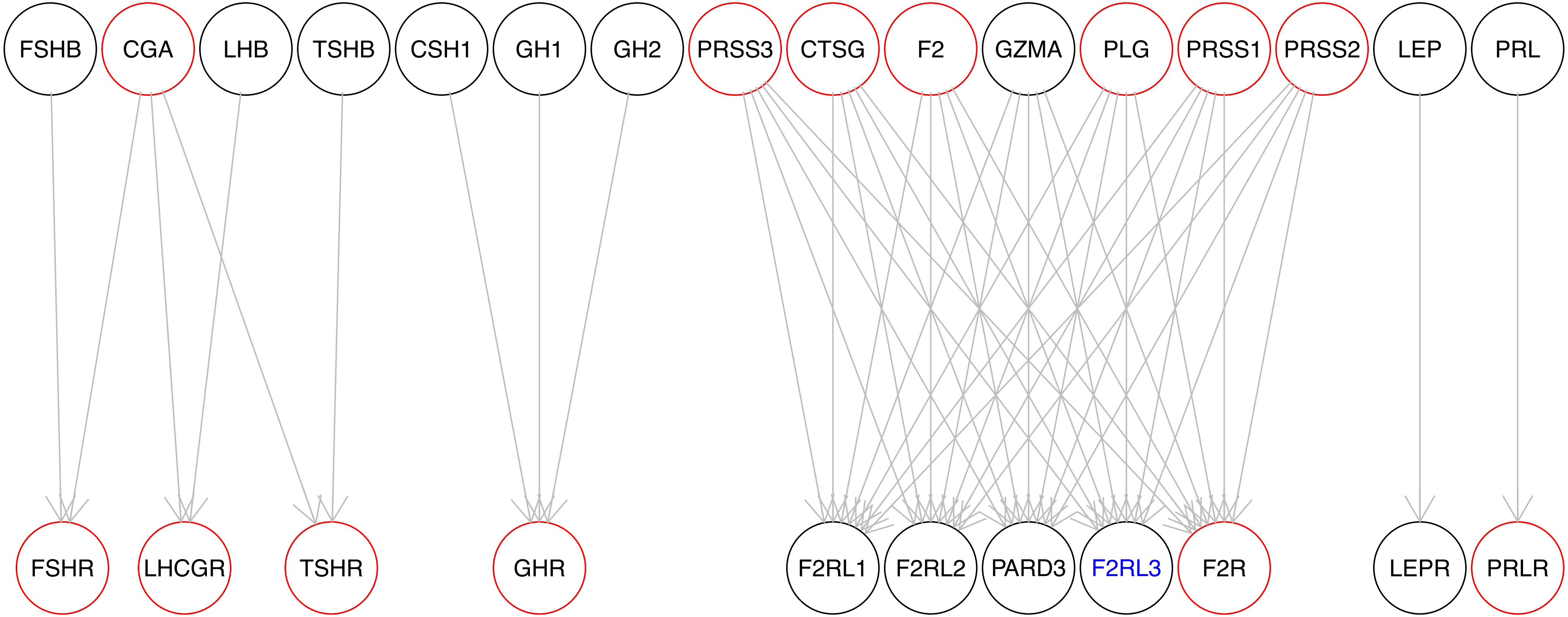

KEGG pathway = Neuroactive ligand−receptor interaction : tumour = MEL.Ma.Mel.19.Tumor : Yellow Fill = gene variant, Blue Text = expression−survival association, Red Boarder = drug

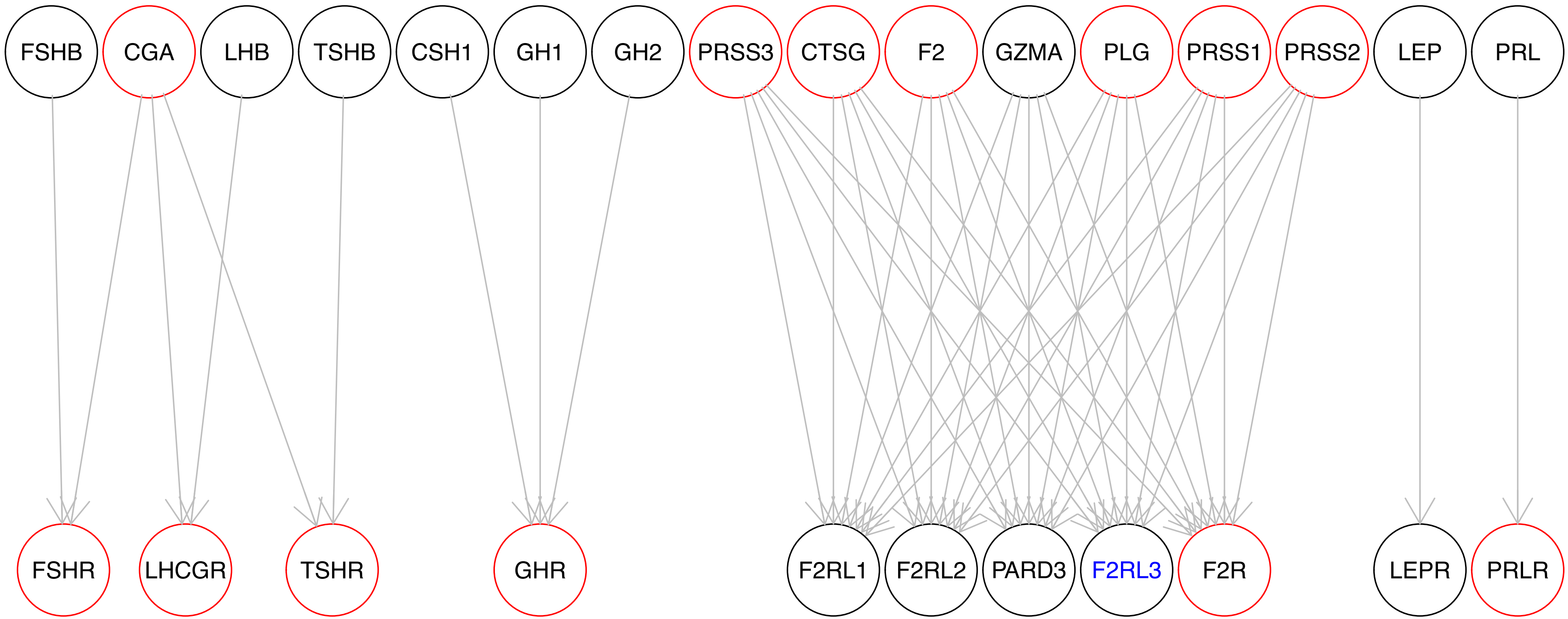

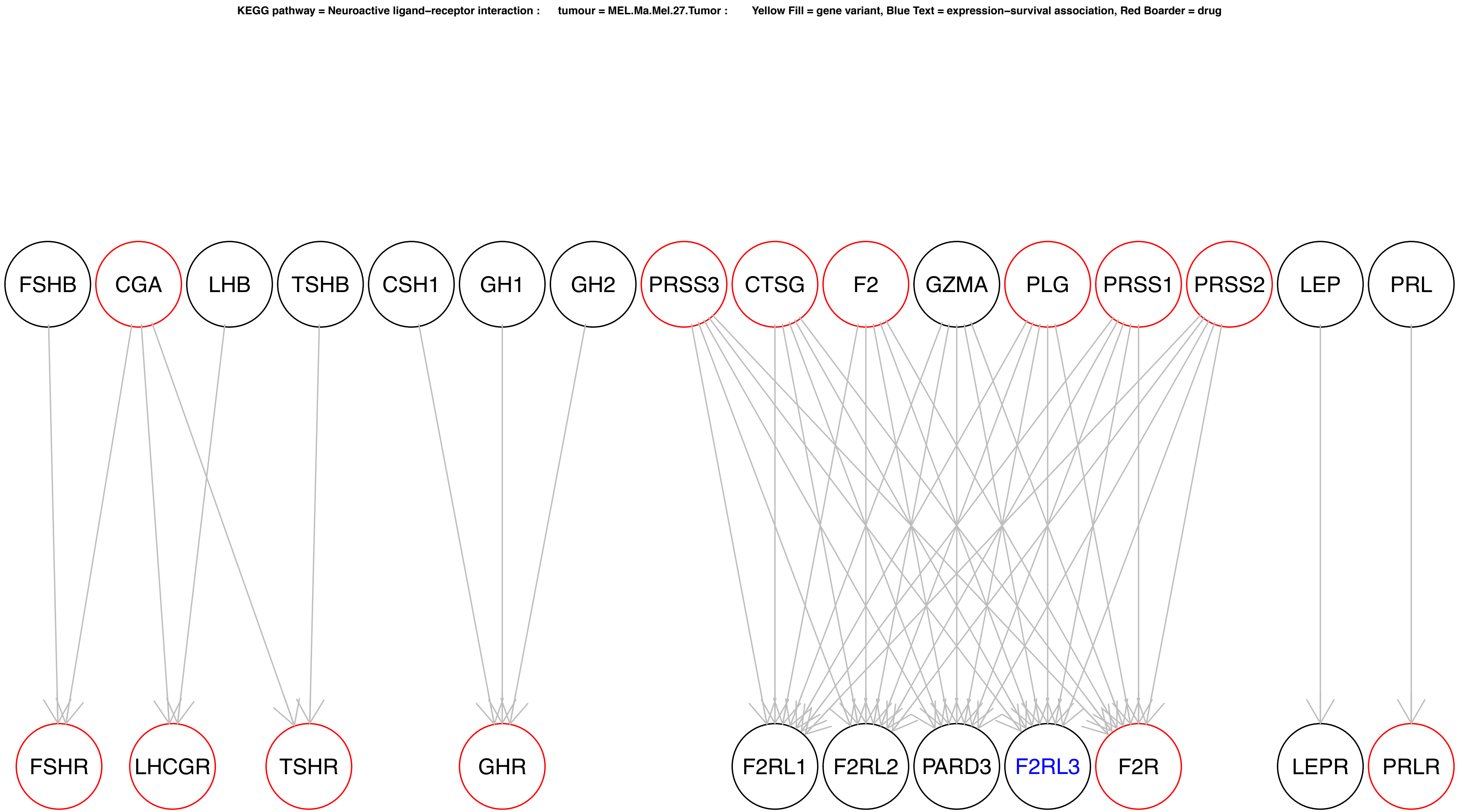
KEGG pathway = Neuroactive ligand−receptor interaction :   tumour = MEL.Ma.Mel.27.Tumor :   Yellow Fill = gene variant, Blue Text = expression−survival association, Red Boarder = drug
FSHB
CGA
LHB
TSHB
CSH1
GH1
GH2
PRSS3
CTSG
F2
GZMA
PLG
PRSS1
PRSS2
LEP
PRL
FSHR
LHCGR
TSHR
GHR
F2RL1
F2RL2
PARD3
F2RL3
F2R
LEPR
PRLR

KEGG pathway = Neuroactive ligand−receptor interaction :   tumour = MEL.Ma.Mel.28.Tumor :   Yellow Fill = gene variant, Blue Text = expression−survival association, Red Boarder = drug

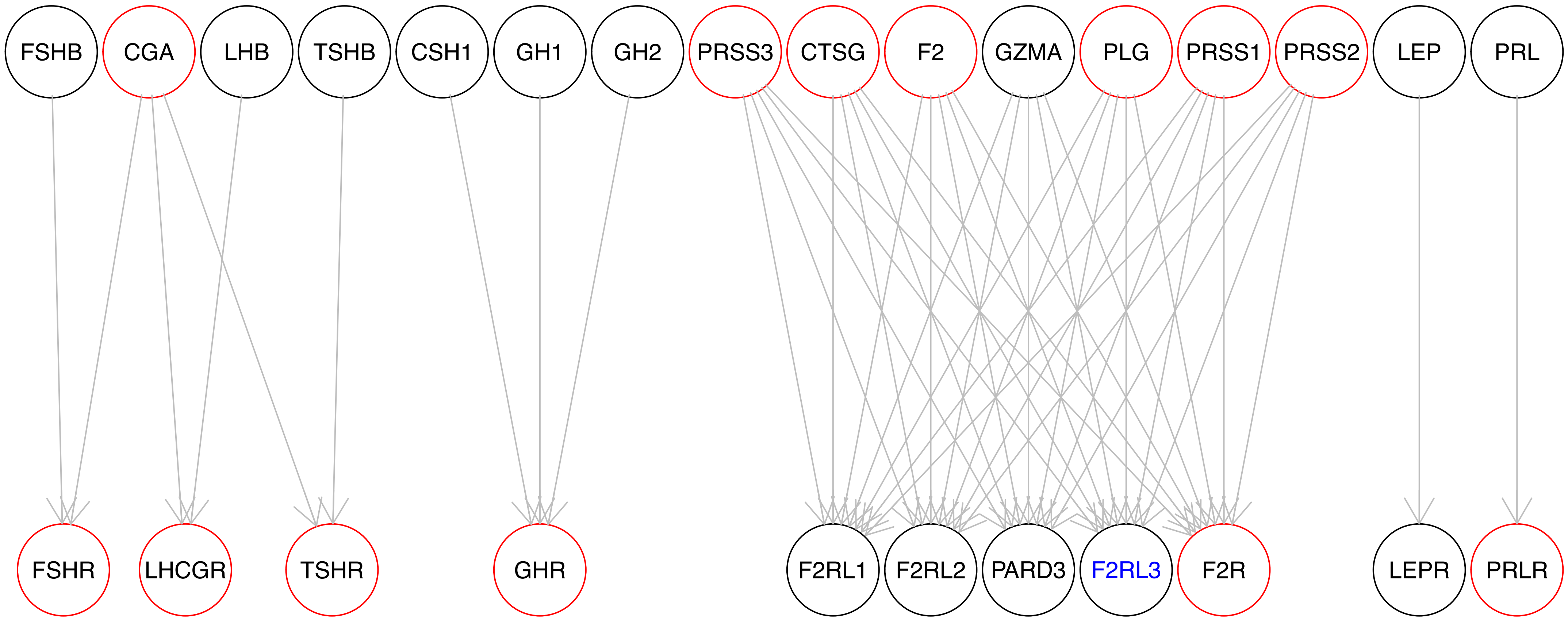

KEGG pathway = Neuroactive ligand−receptor interaction : tumour = MEL.Ma.Mel.35.Tumor : Yellow Fill = gene variant, Blue Text = expression−survival association, Red Boarder = drug

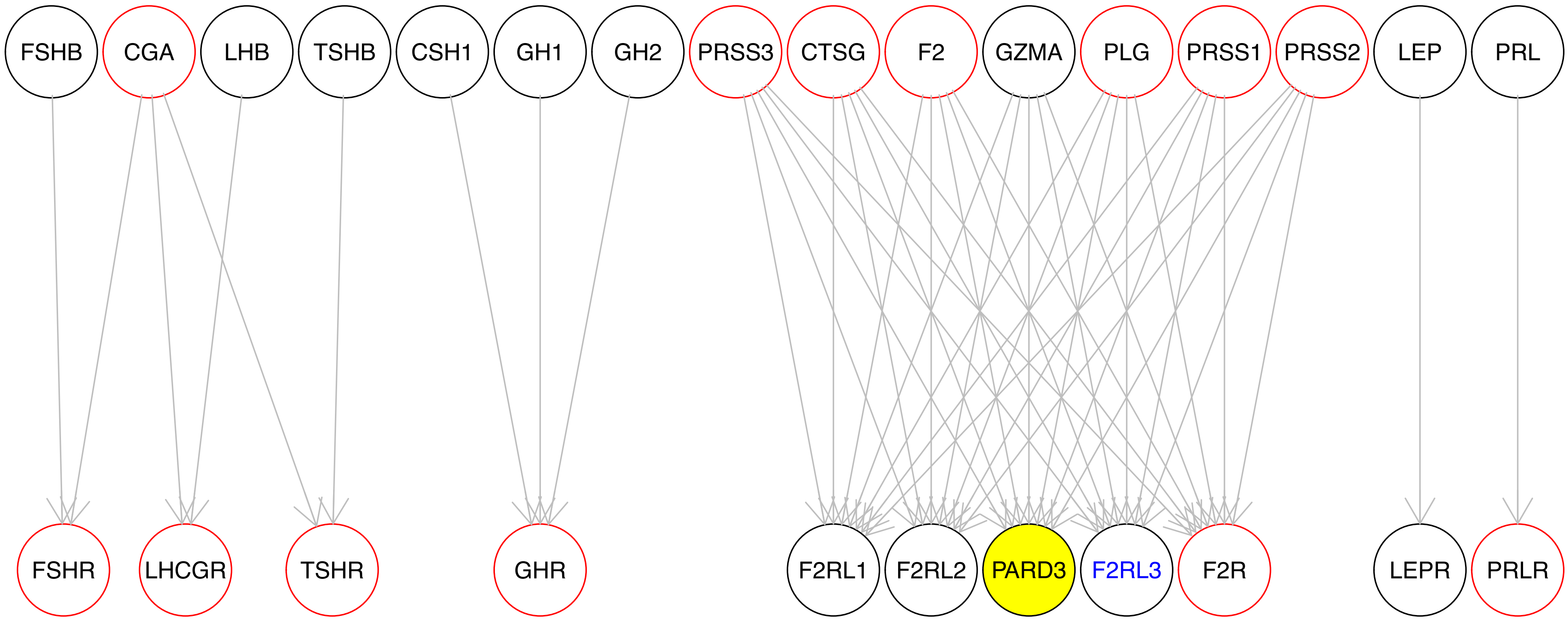

KEGG pathway = Neuroactive ligand−receptor interaction :   tumour = MEL.Ma.Mel.36.Tumor :   Yellow Fill = gene variant, Blue Text = expression−survival association, Red Boarder = drug

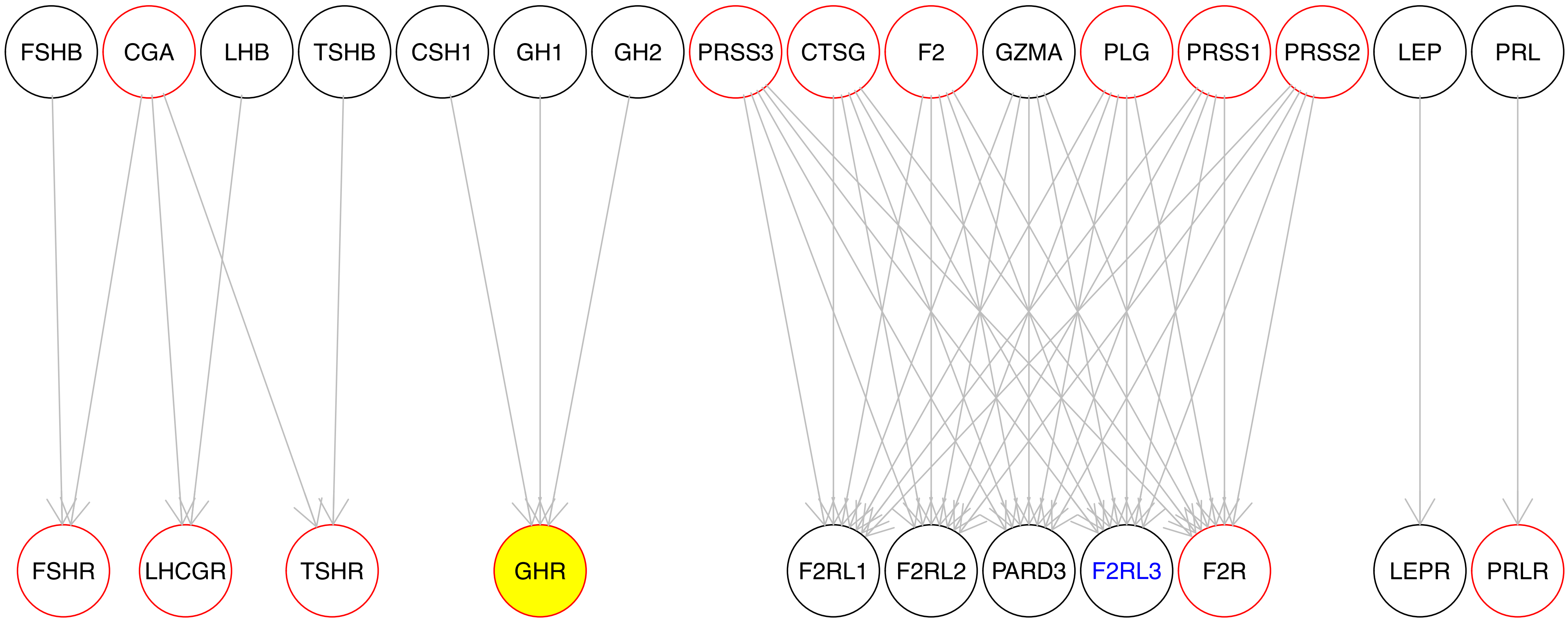

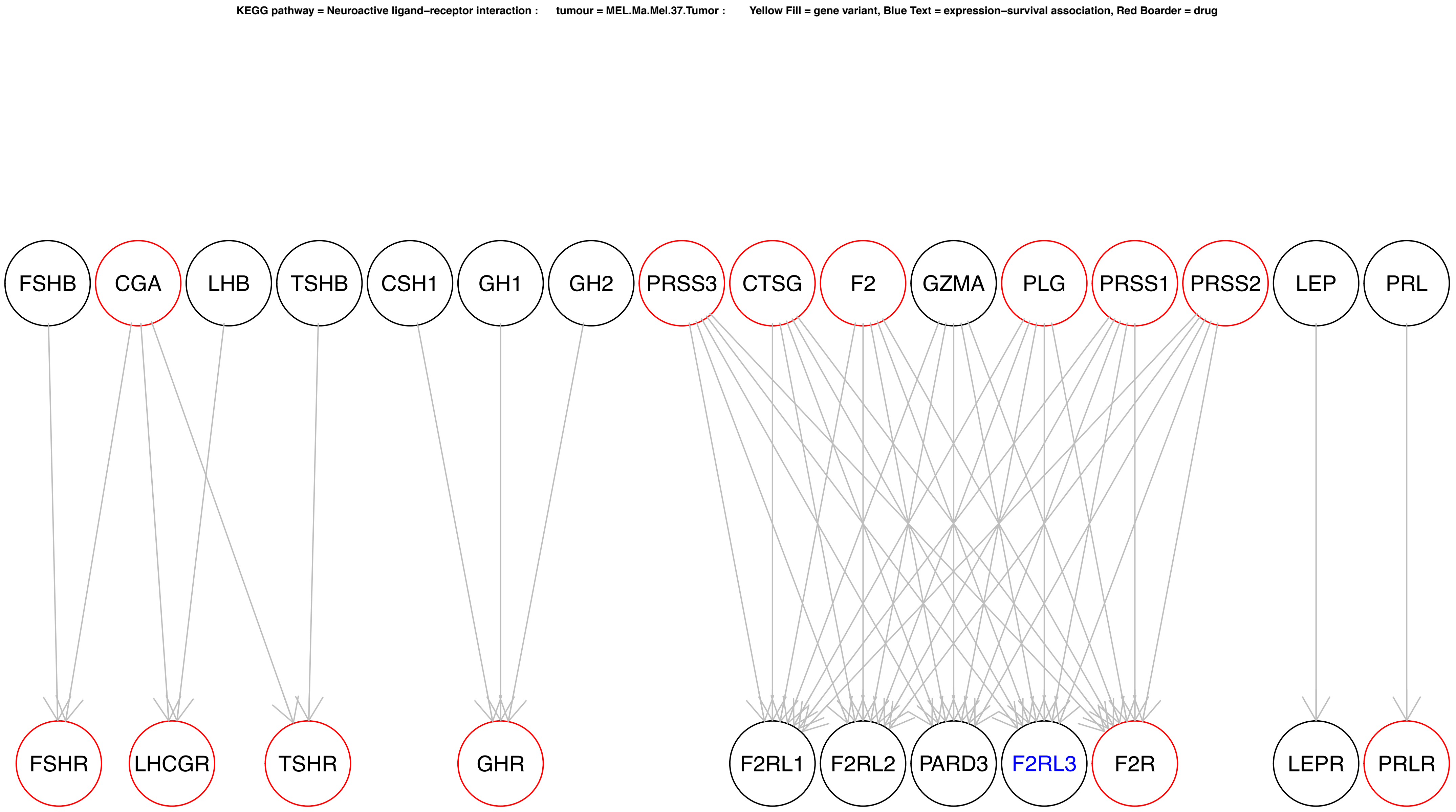
KEGG pathway = Neuroactive ligand−receptor interaction :
tumour = MEL.Ma.Mel.37.Tumor :
Yellow Fill = gene variant, Blue Text = expression−survival association, Red Boarder = drug
FSHB
CGA
LHB
TSHB
CSH1
GH1
GH2
PRSS3
CTSG
F2
GZMA
PLG
PRSS1
PRSS2
LEP
PRL
FSHR
LHCGR
TSHR
GHR
F2RL1
F2RL2
PARD3
F2RL3
F2R
LEPR
PRLR

KEGG pathway = Neuroactive ligand–receptor interaction : tumour = MEL.Ma.Mel.48.Tumor : Yellow Fill = gene variant, Blue Text = expression–survival association, Red Boarder = drug

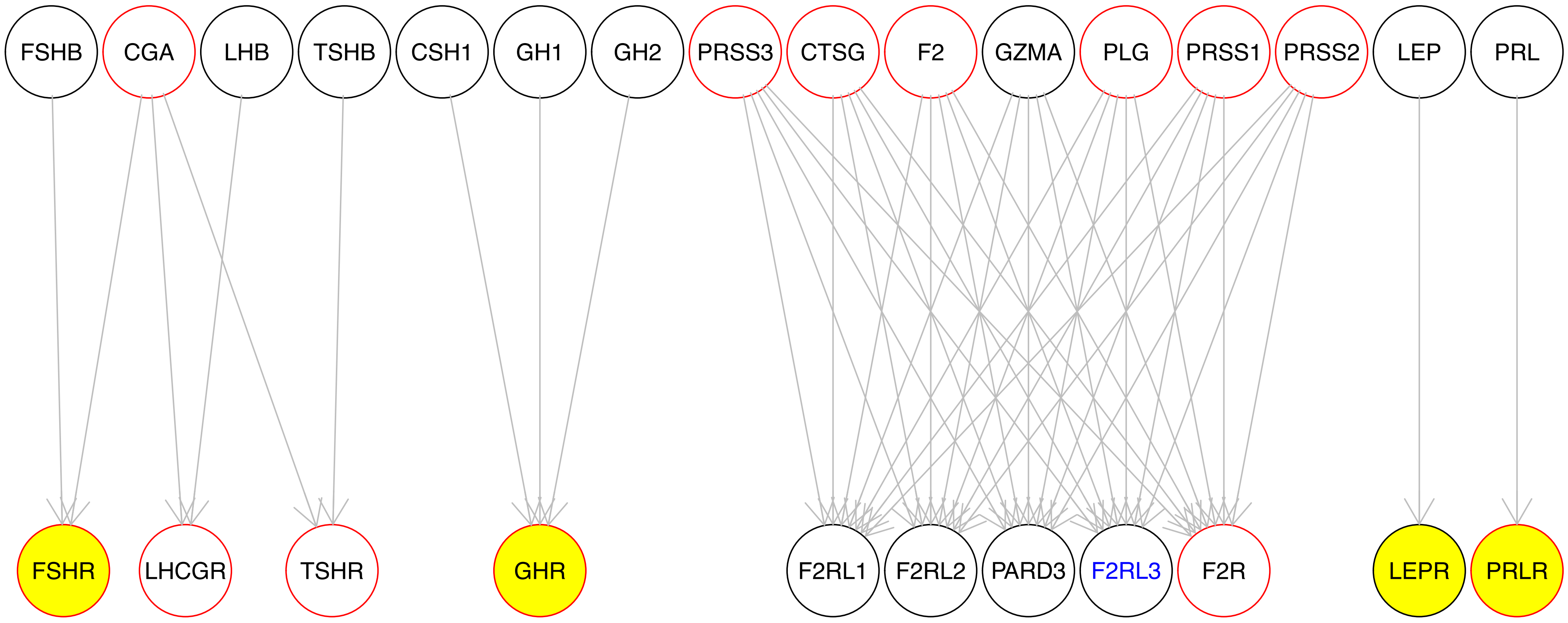

KEGG pathway = Neuroactive ligand−receptor interaction : tumour = MEL.Ma.Mel.53.Tumor : Yellow Fill = gene variant, Blue Text = expression−survival association, Red Boarder = drug

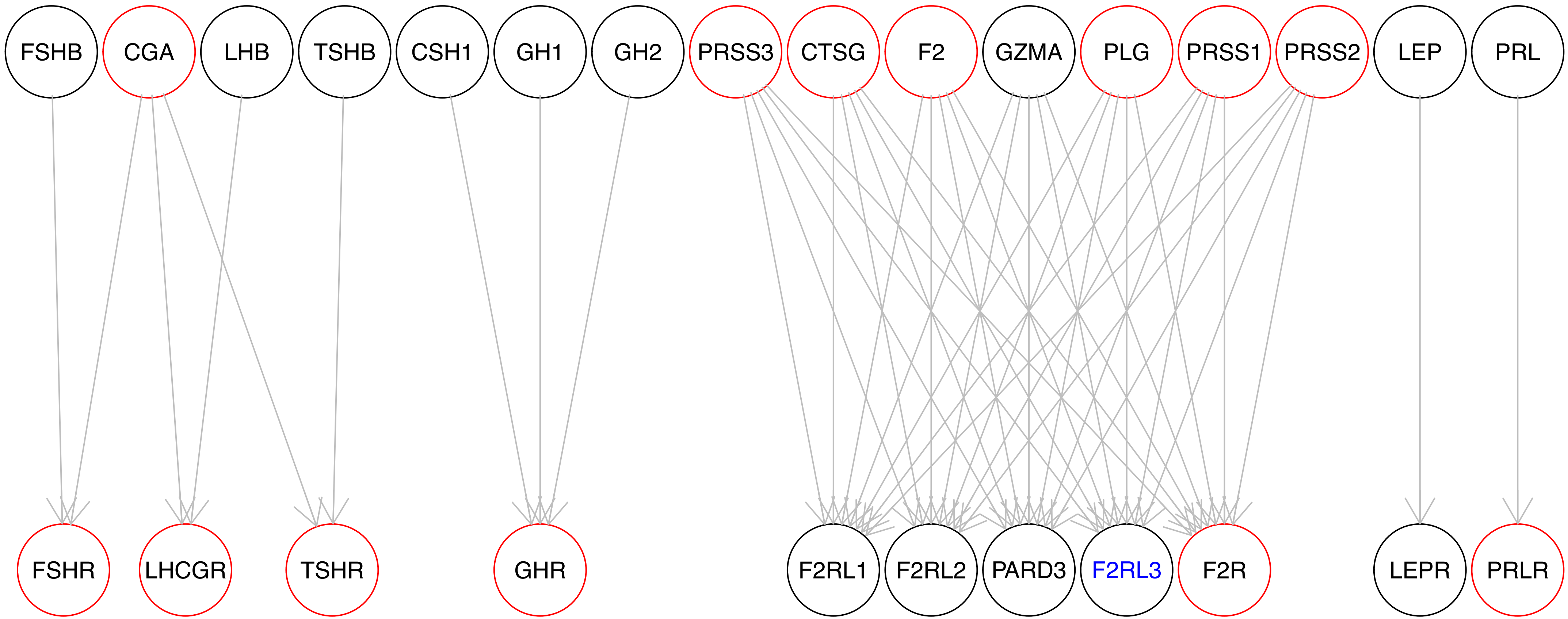

KEGG pathway = Neuroactive ligand–receptor interaction : tumour = MEL.Ma.Mel.54a.Tumor : Yellow Fill = gene variant, Blue Text = expression–survival association, Red Boarder = drug

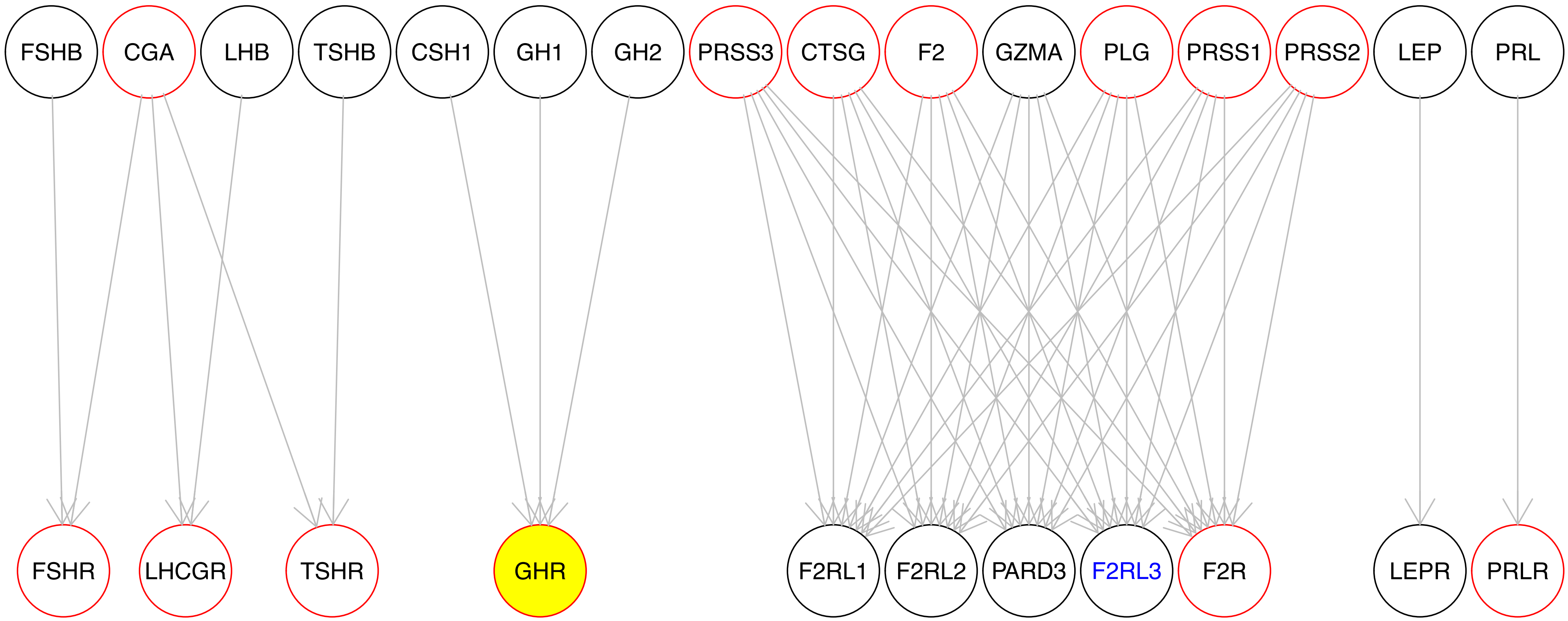

KEGG pathway = Neuroactive ligand−receptor interaction : tumour = MEL.Ma.Mel.55.Tumor : Yellow Fill = gene variant, Blue Text = expression−survival association, Red Boarder = drug

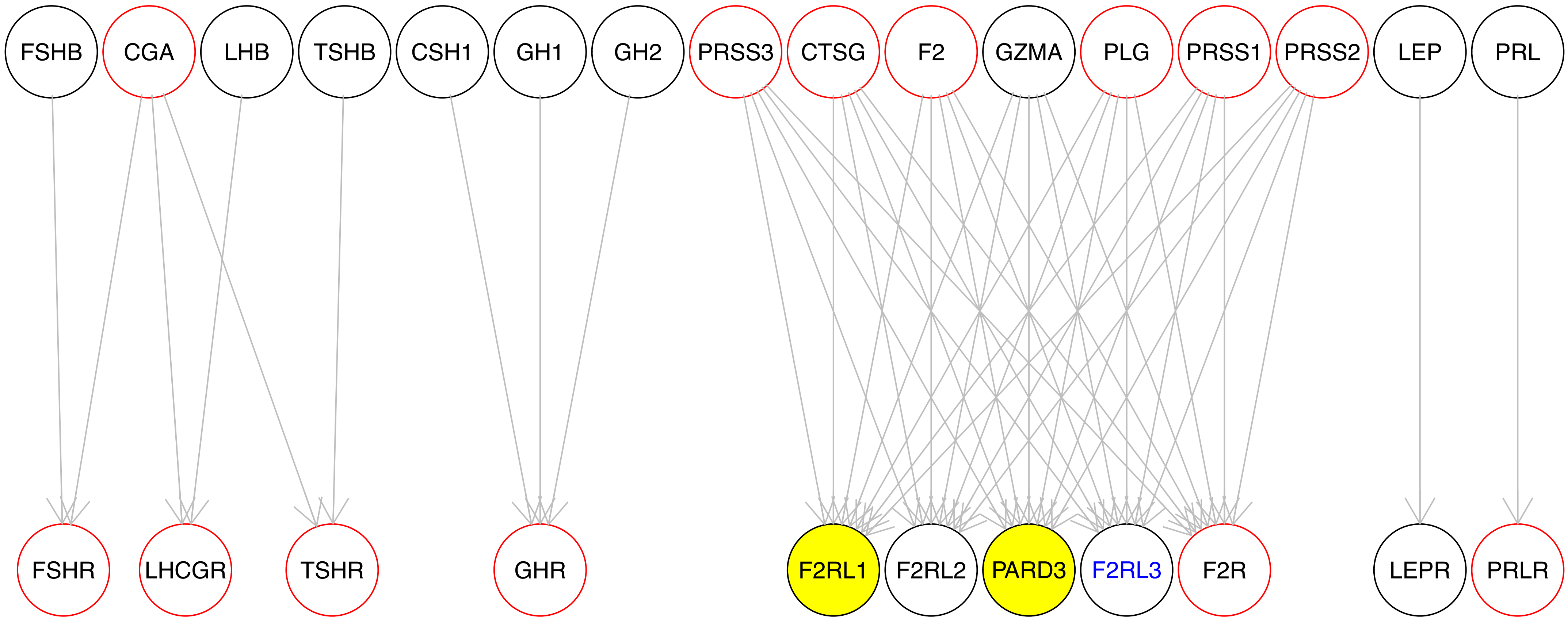

KEGG pathway = Neuroactive ligand−receptor interaction : tumour = MEL.Ma.Mel.59.Tumor : Yellow Fill = gene variant, Blue Text = expression−survival association, Red Boarder = drug

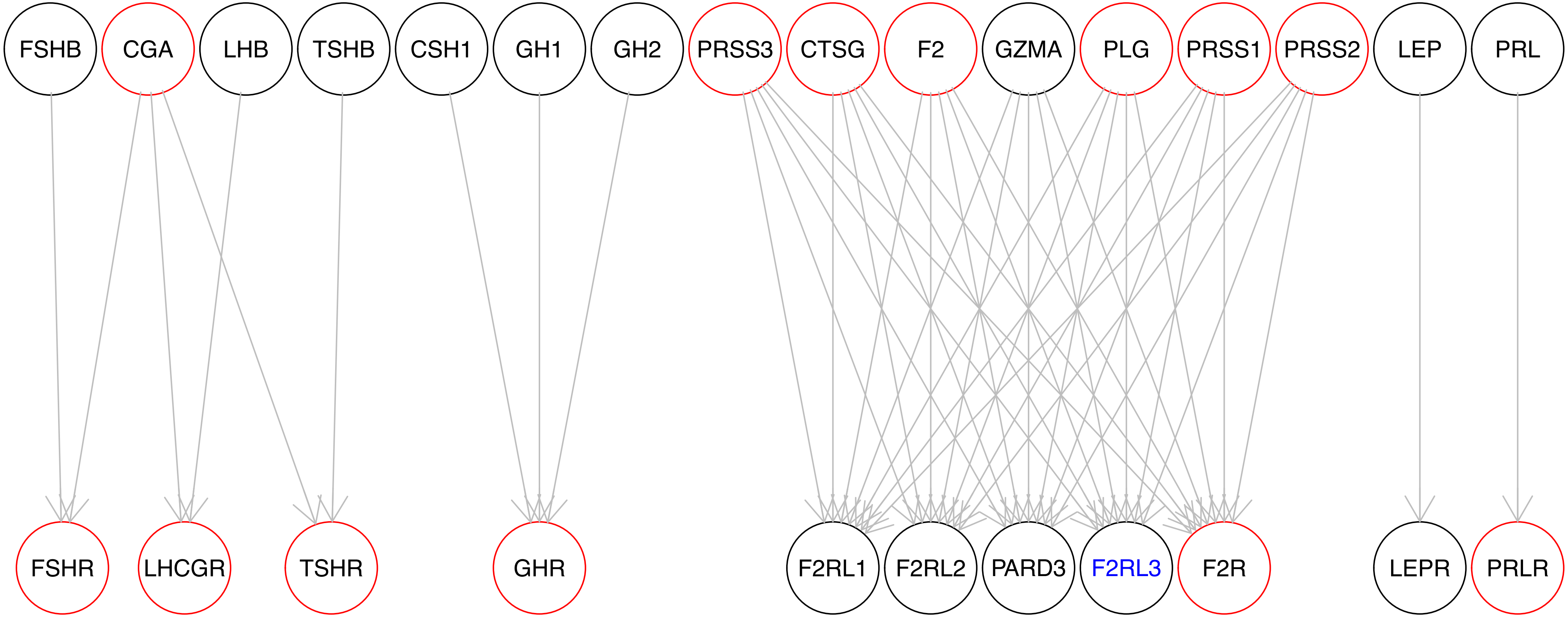

KEGG pathway = Neuroactive ligand−receptor interaction : tumour = MEL.Ma.Mel.62.Tumor : Yellow Fill = gene variant, Blue Text = expression−survival association, Red Boarder = drug

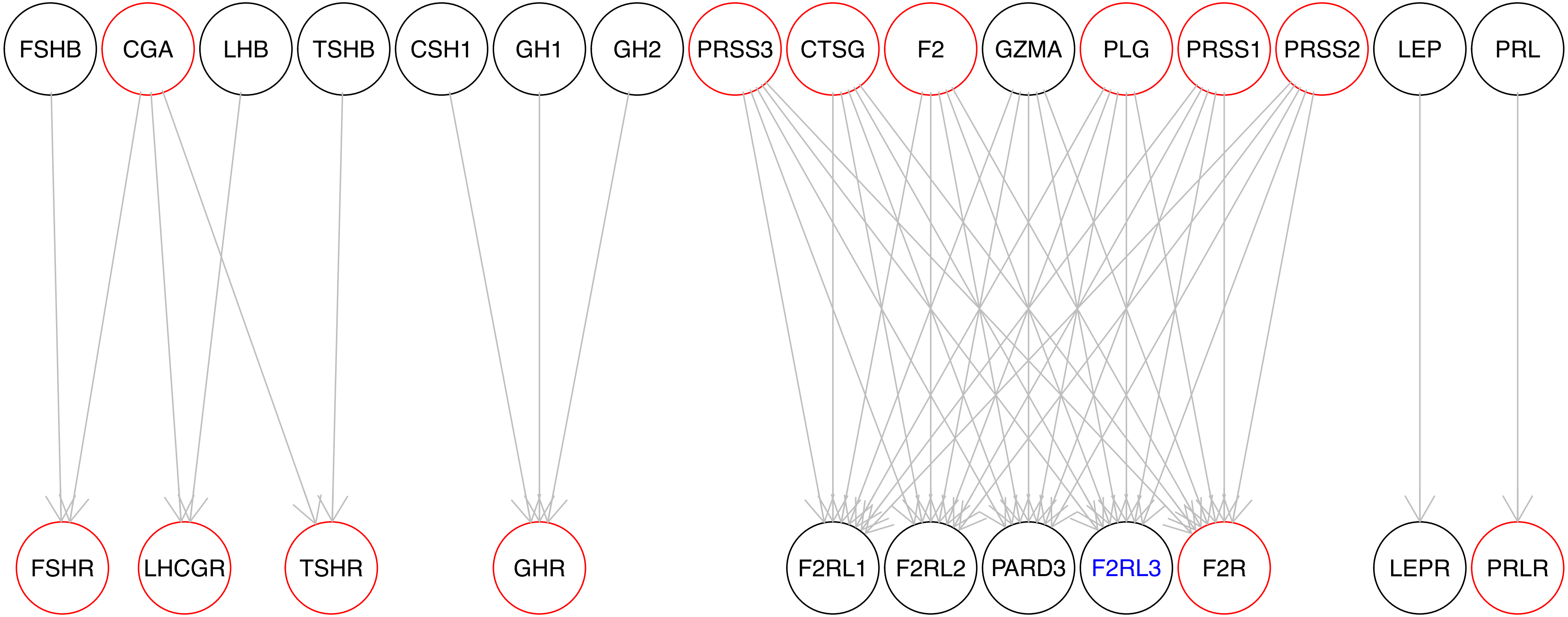

KEGG pathway = Neuroactive ligand−receptor interaction : tumour = MEL.Ma.Mel.63.Tumor : Yellow Fill = gene variant, Blue Text = expression−survival association, Red Boarder = drug

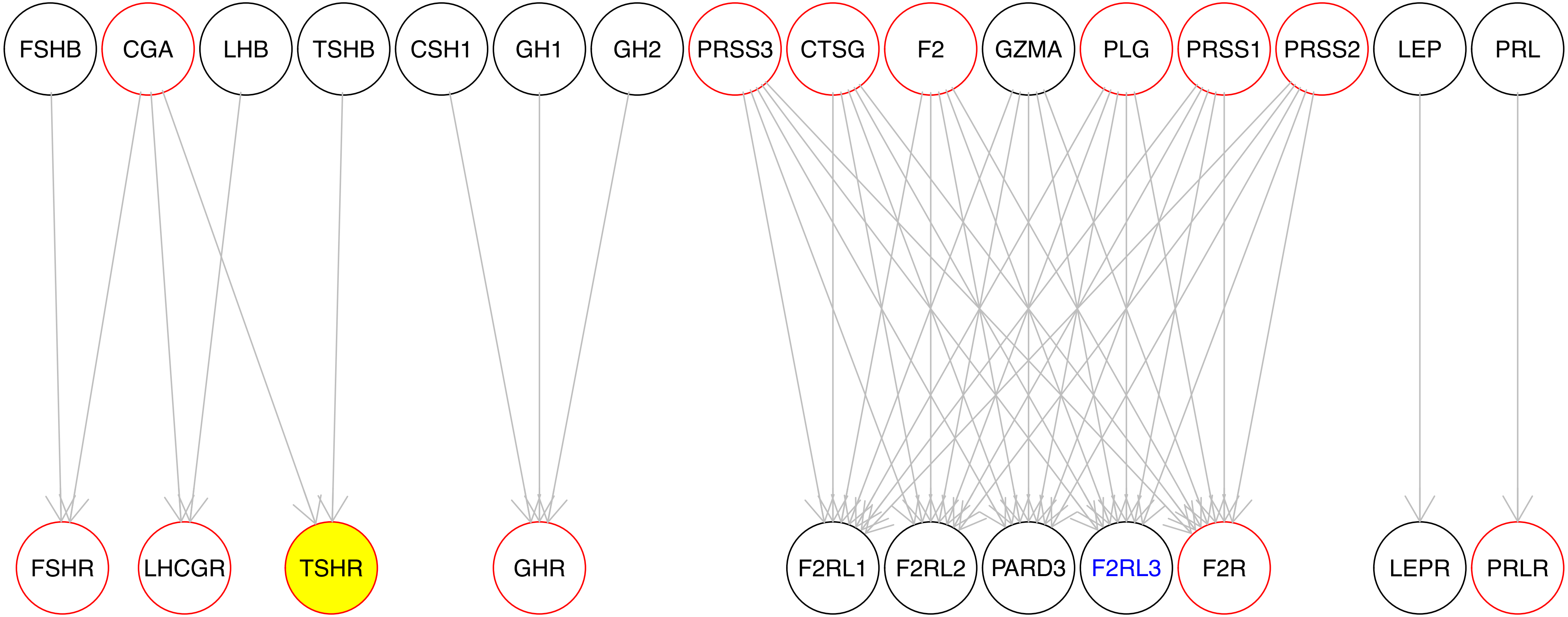

KEGG pathway = Neuroactive ligand−receptor interaction : tumour = MEL.Ma.Mel.65.Tumor : Yellow Fill = gene variant, Blue Text = expression−survival association, Red Boarder = drug

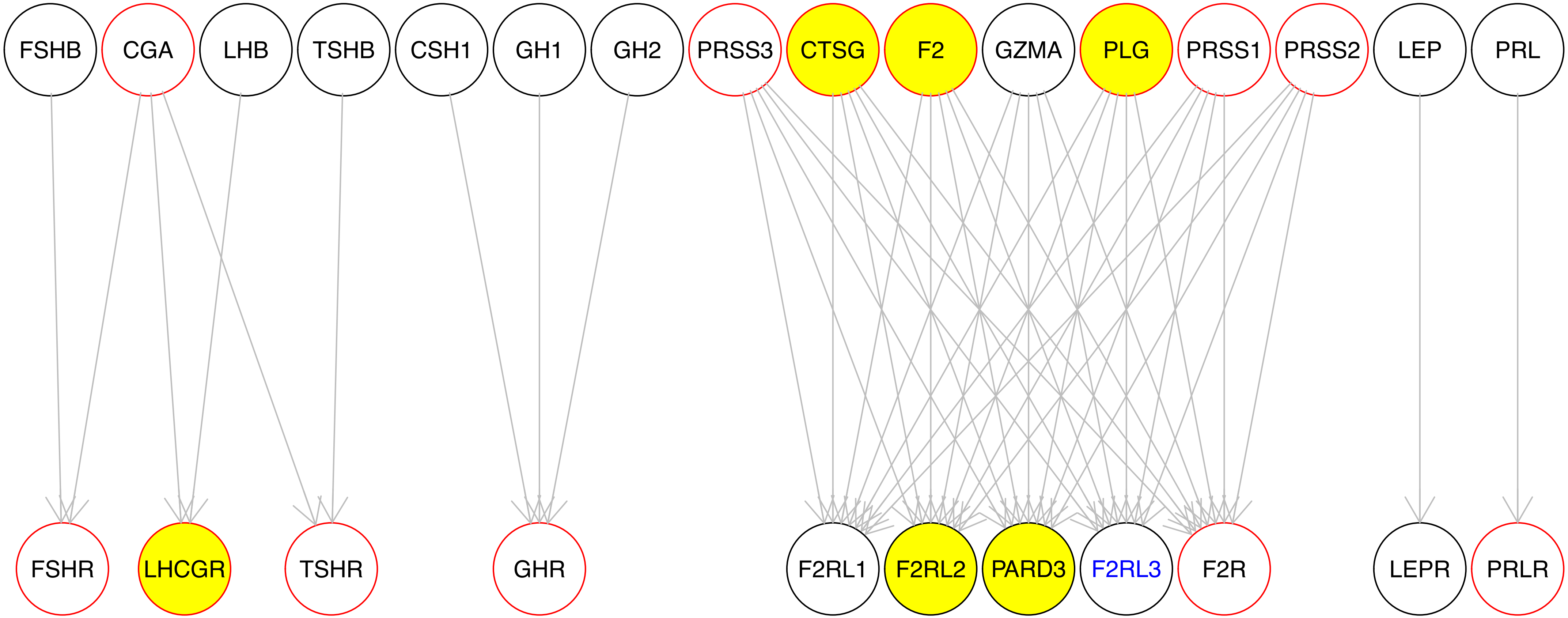

KEGG pathway = Neuroactive ligand−receptor interaction : tumour = MEL.Ma.Mel.67.Tumor : Yellow Fill = gene variant, Blue Text = expression−survival association, Red Boarder = drug

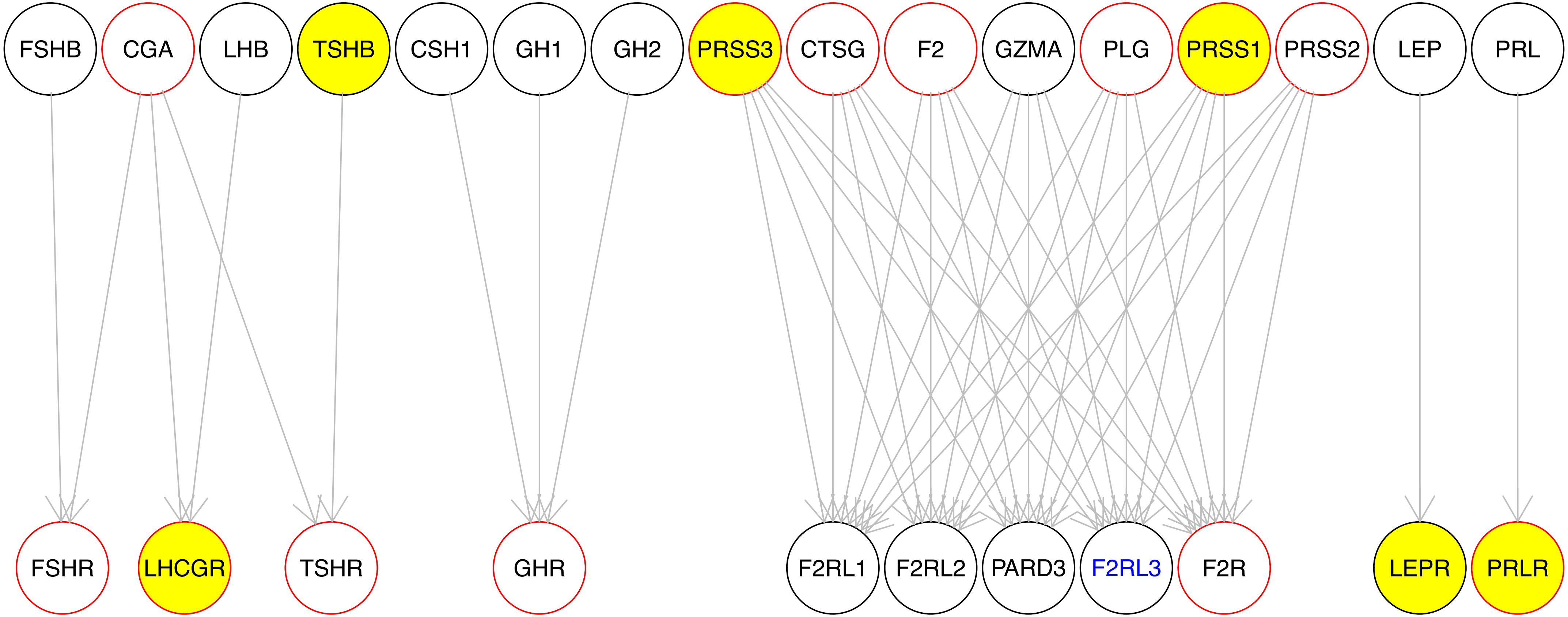

KEGG pathway = Neuroactive ligand−receptor interaction : tumour = MEL.Ma.Mel.71.Tumor : Yellow Fill = gene variant, Blue Text = expression−survival association, Red Boarder = drug

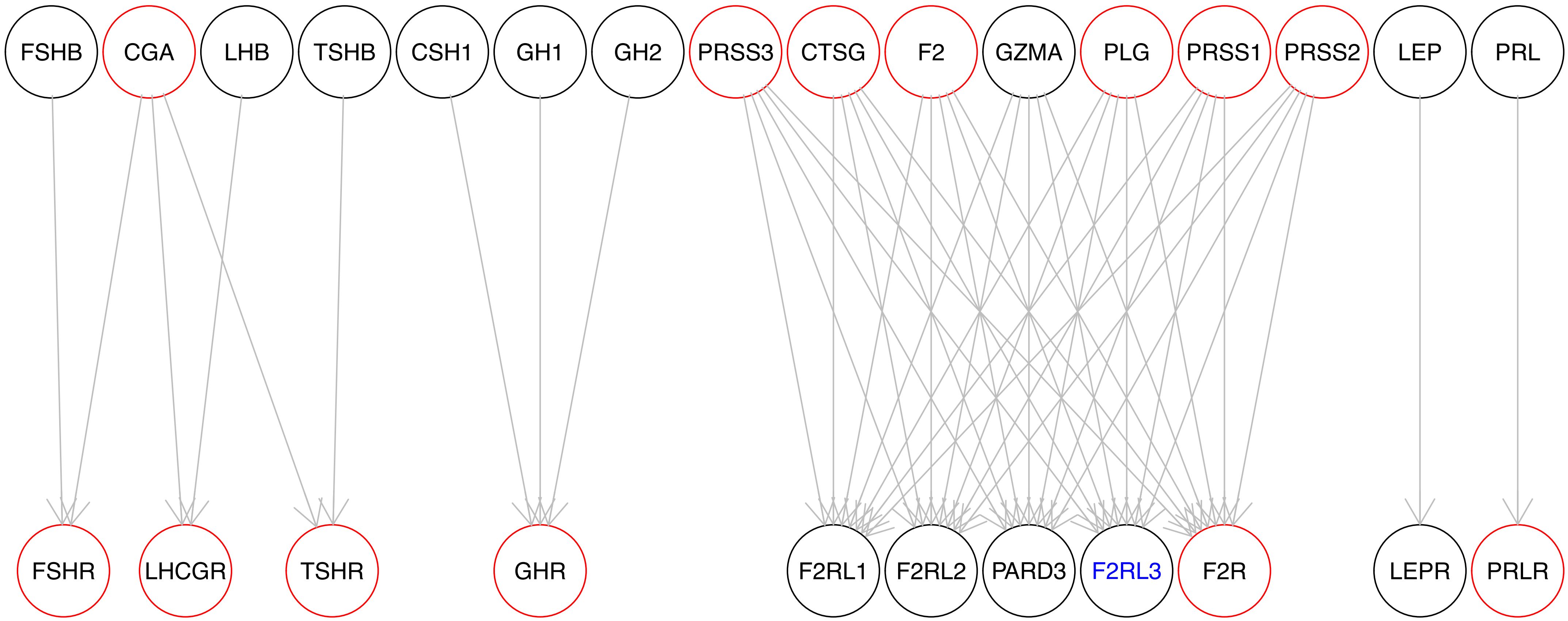

KEGG pathway = Neuroactive ligand−receptor interaction :   tumour = MEL.Ma.Mel.76.Tumor :   Yellow Fill = gene variant, Blue Text = expression−survival association, Red Boarder = drug

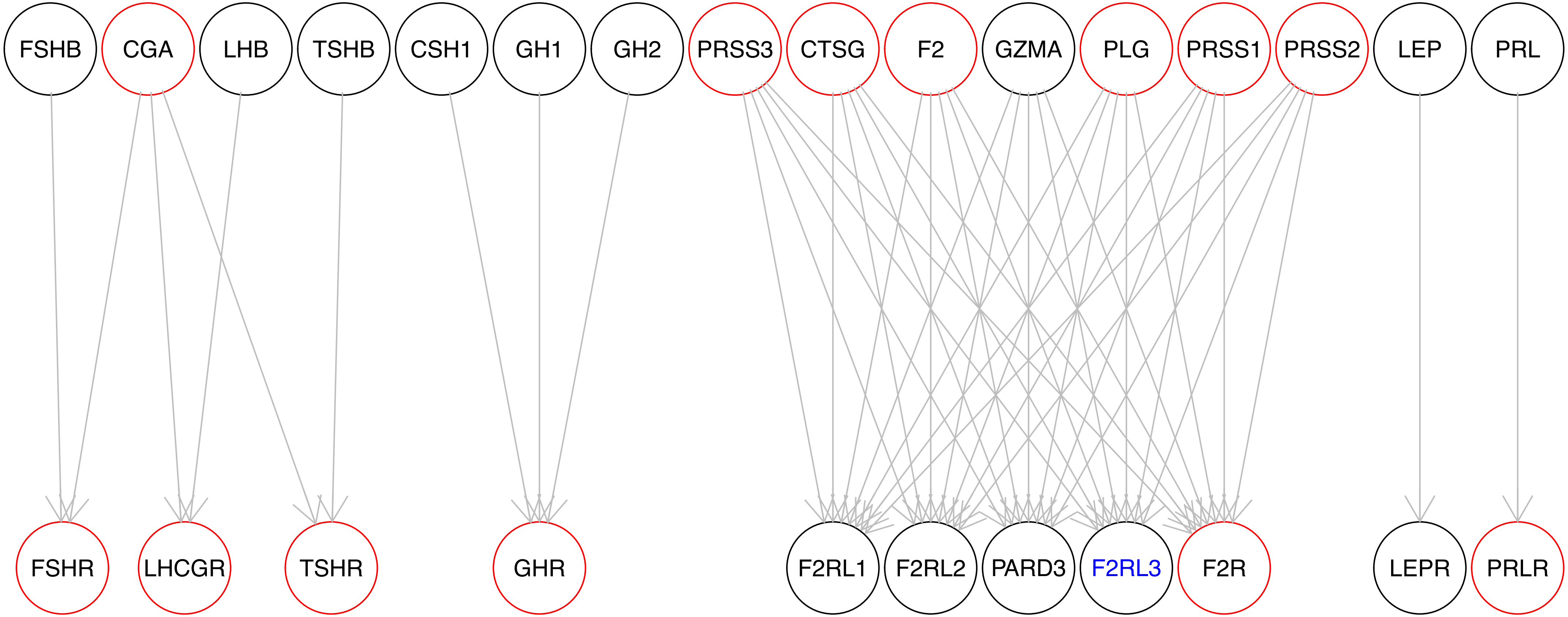

KEGG pathway = Neuroactive ligand−receptor interaction : tumour = MEL.Ma.Mel.79.Tumor : Yellow Fill = gene variant, Blue Text = expression−survival association, Red Boarder = drug

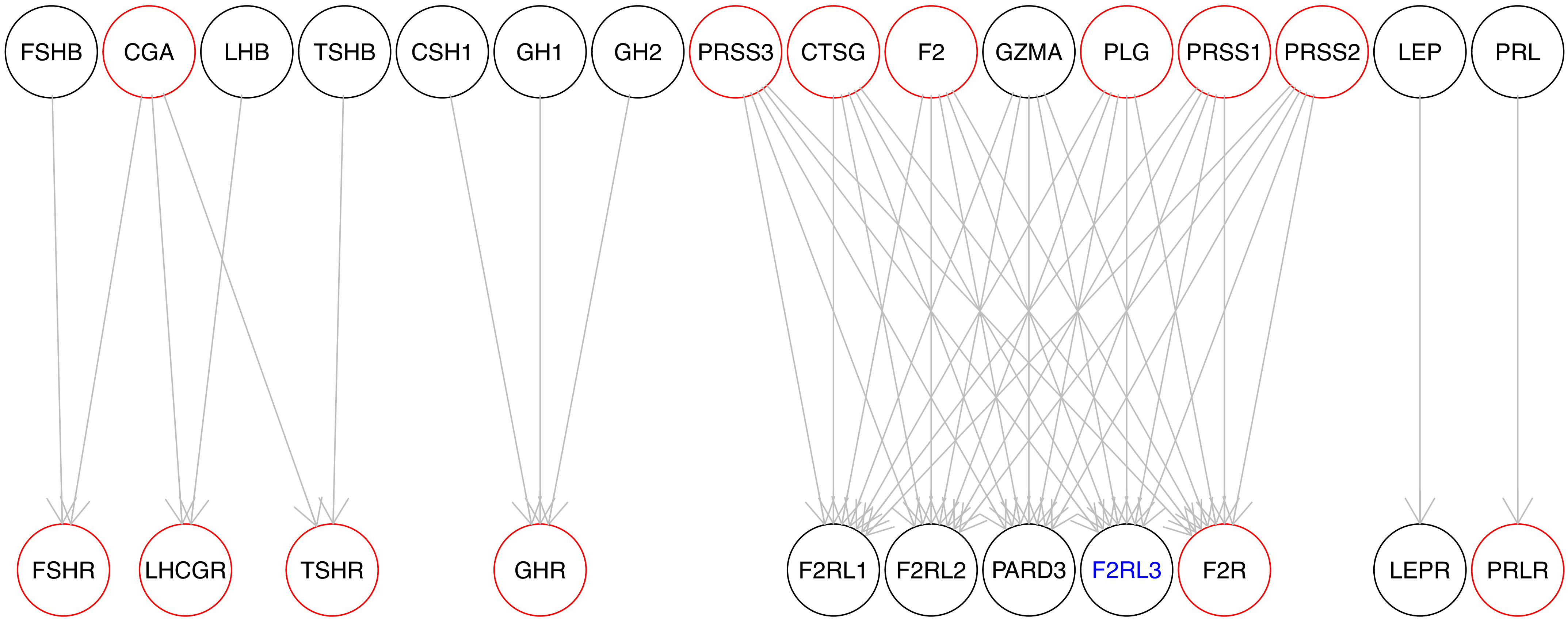

KEGG pathway = Neuroactive ligand−receptor interaction : tumour = MEL.Ma.Mel.85.Tumor : Yellow Fill = gene variant, Blue Text = expression−survival association, Red Boarder = drug

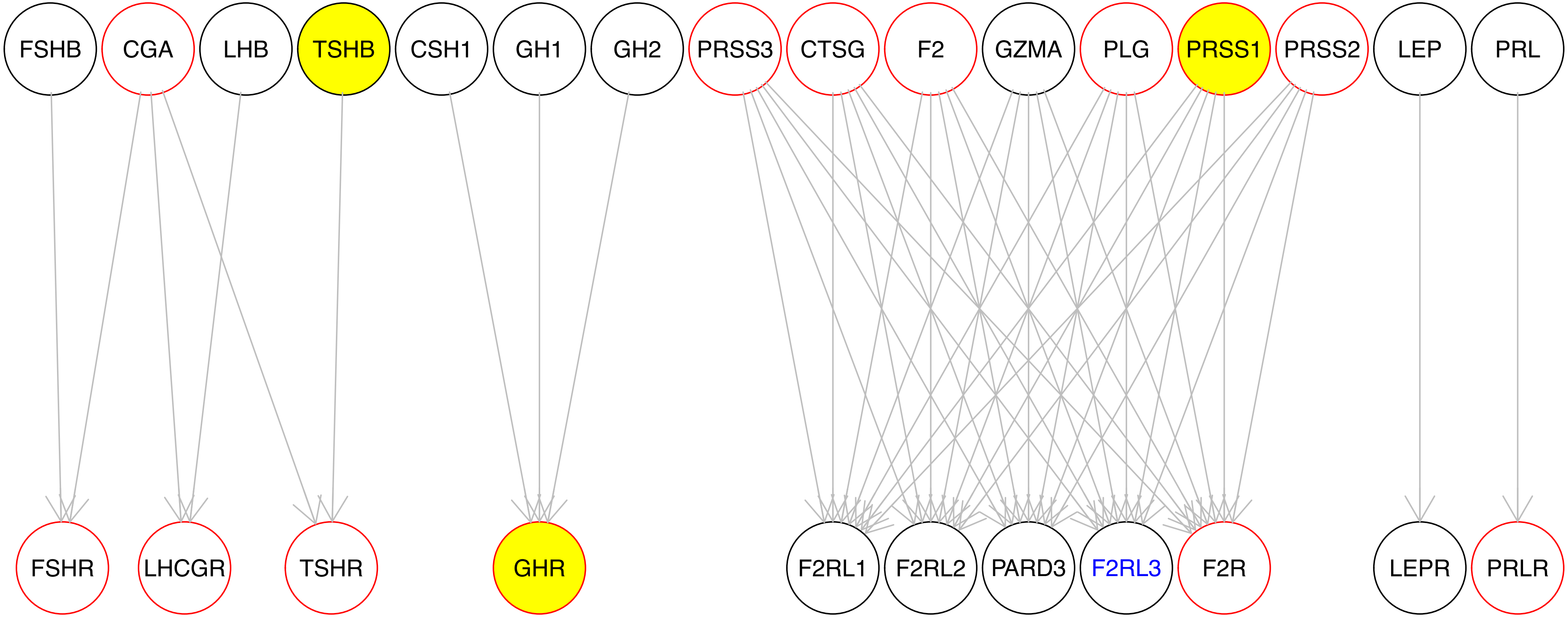

KEGG pathway = Neuroactive ligand−receptor interaction : tumour = MEL.Ma.Mel.86.Tumor : Yellow Fill = gene variant, Blue Text = expression−survival association, Red Boarder = drug

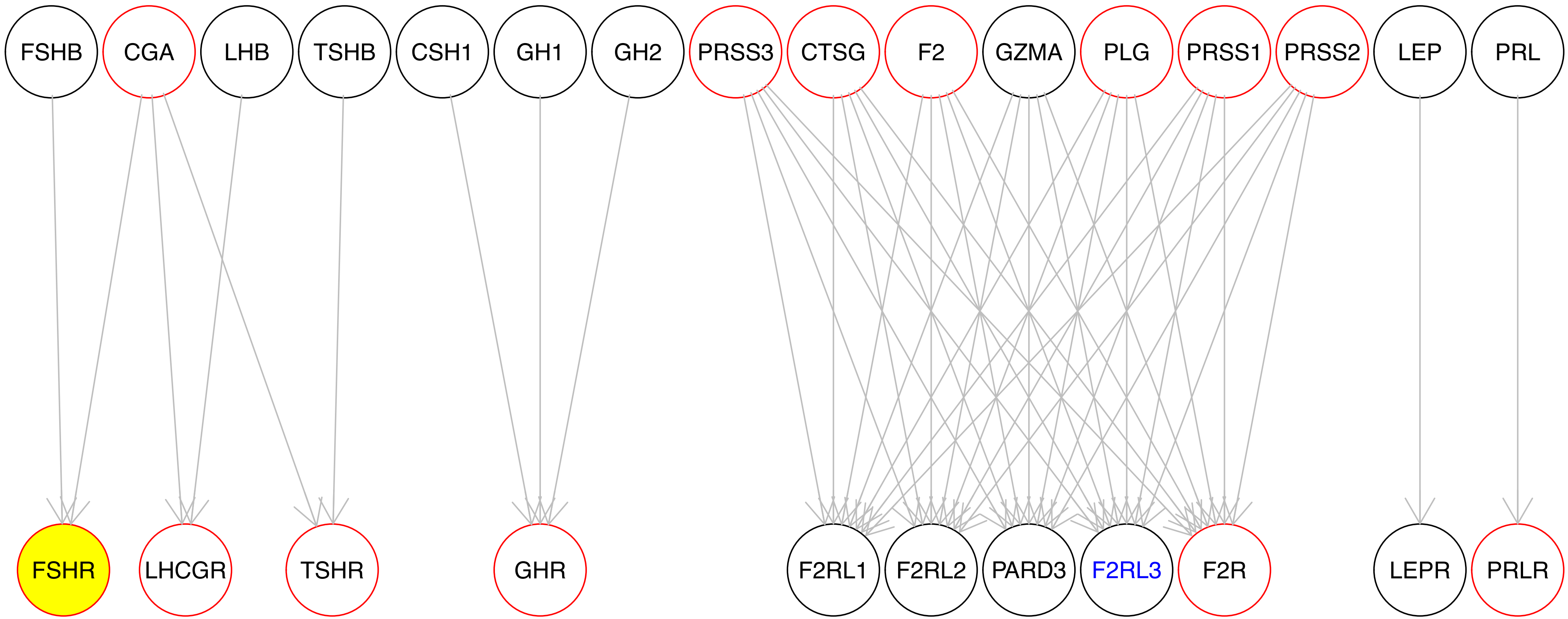

KEGG pathway = Neuroactive ligand−receptor interaction :   tumour = MEL.Ma.Mel.91.Tumor :   Yellow Fill = gene variant, Blue Text = expression−survival association, Red Boarder = drug

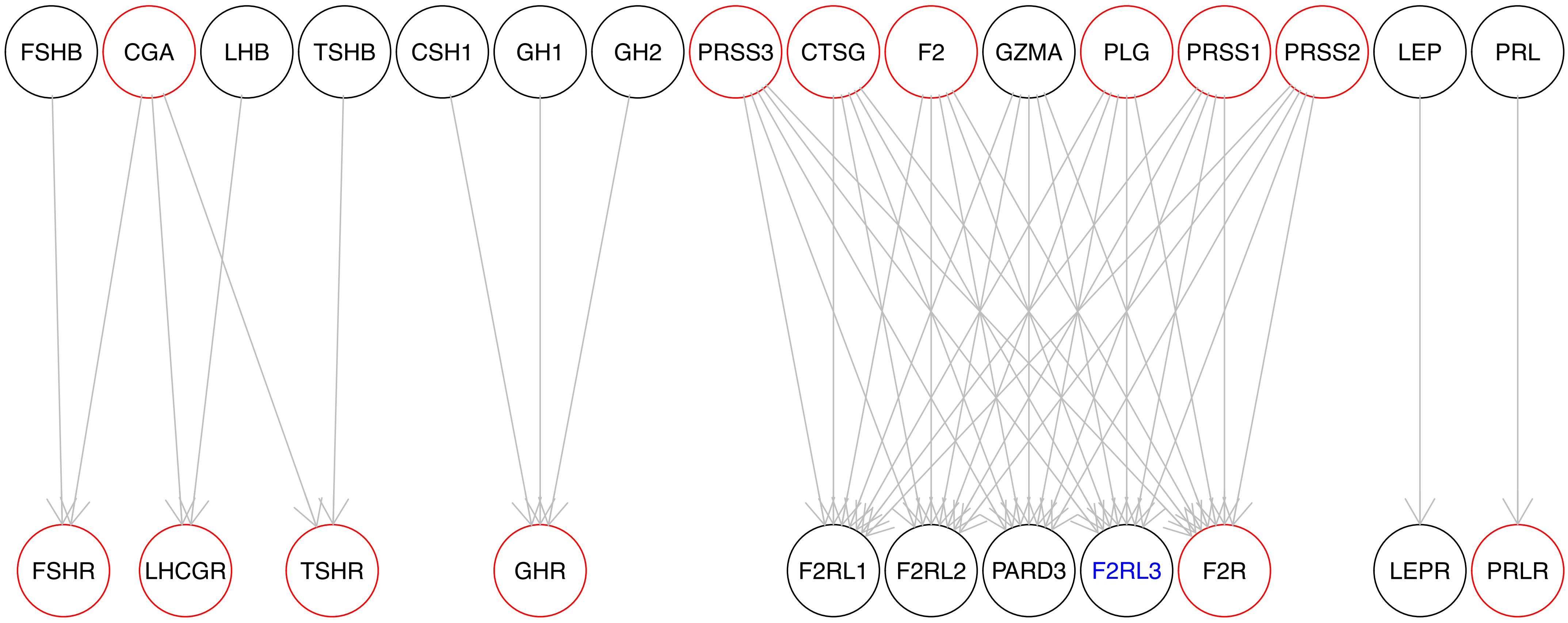

KEGG pathway = Neuroactive ligand–receptor interaction :   tumour = MEL.Ma.Mel.92.Tumor :   Yellow Fill = gene variant, Blue Text = expression–survival association, Red Boarder = drug

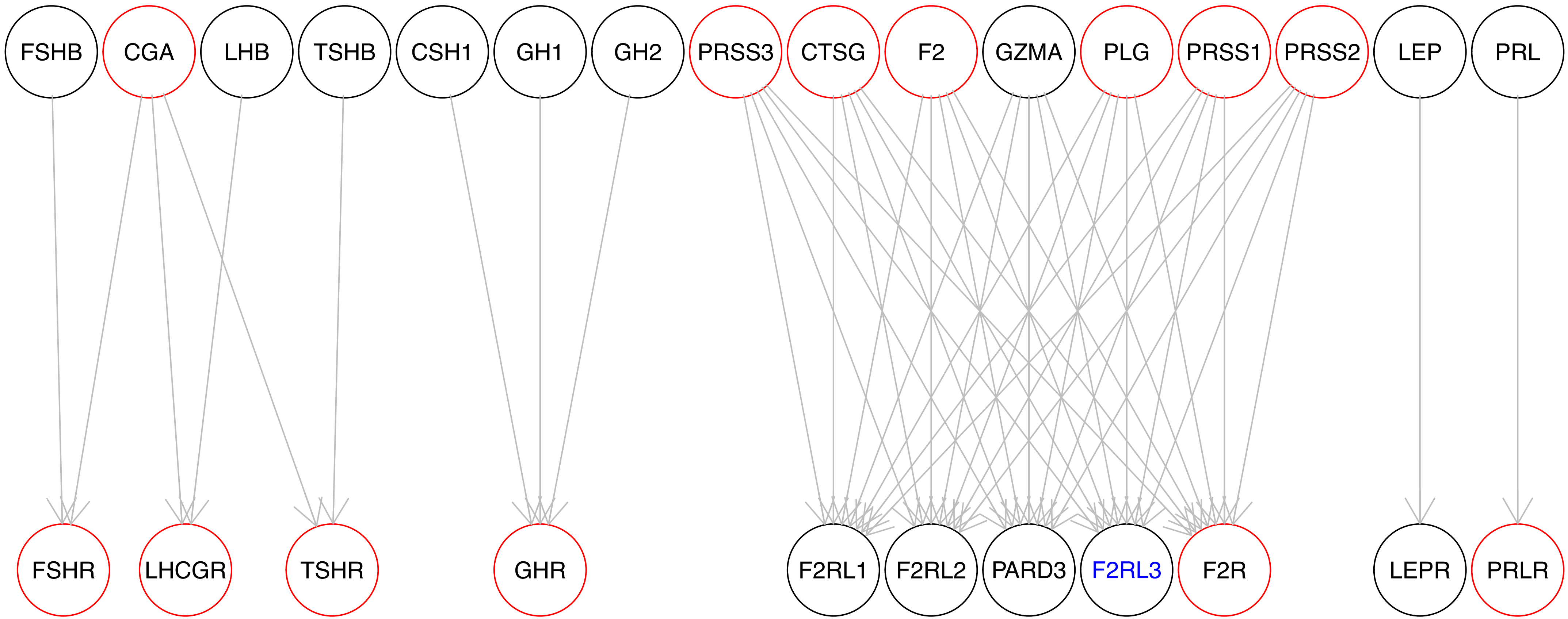

KEGG pathway = Neuroactive ligand−receptor interaction :   tumour = MEL.Ma.Mel.94.Tumor :   Yellow Fill = gene variant, Blue Text = expression−survival association, Red Boarder = drug

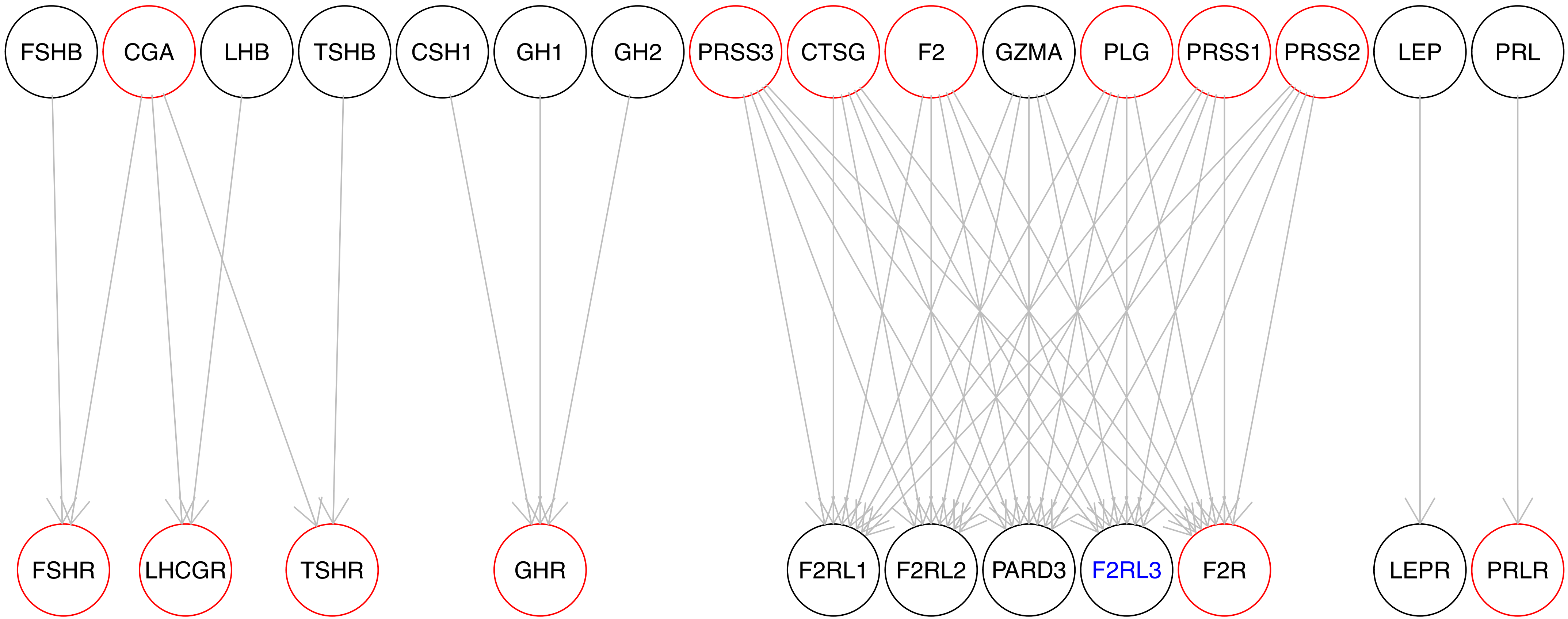

KEGG pathway = Neuroactive ligand−receptor interaction : tumour = MEL.UKRV.Mel.20.Tumor : Yellow Fill = gene variant, Blue Text = expression−survival association, Red Boarder = drug

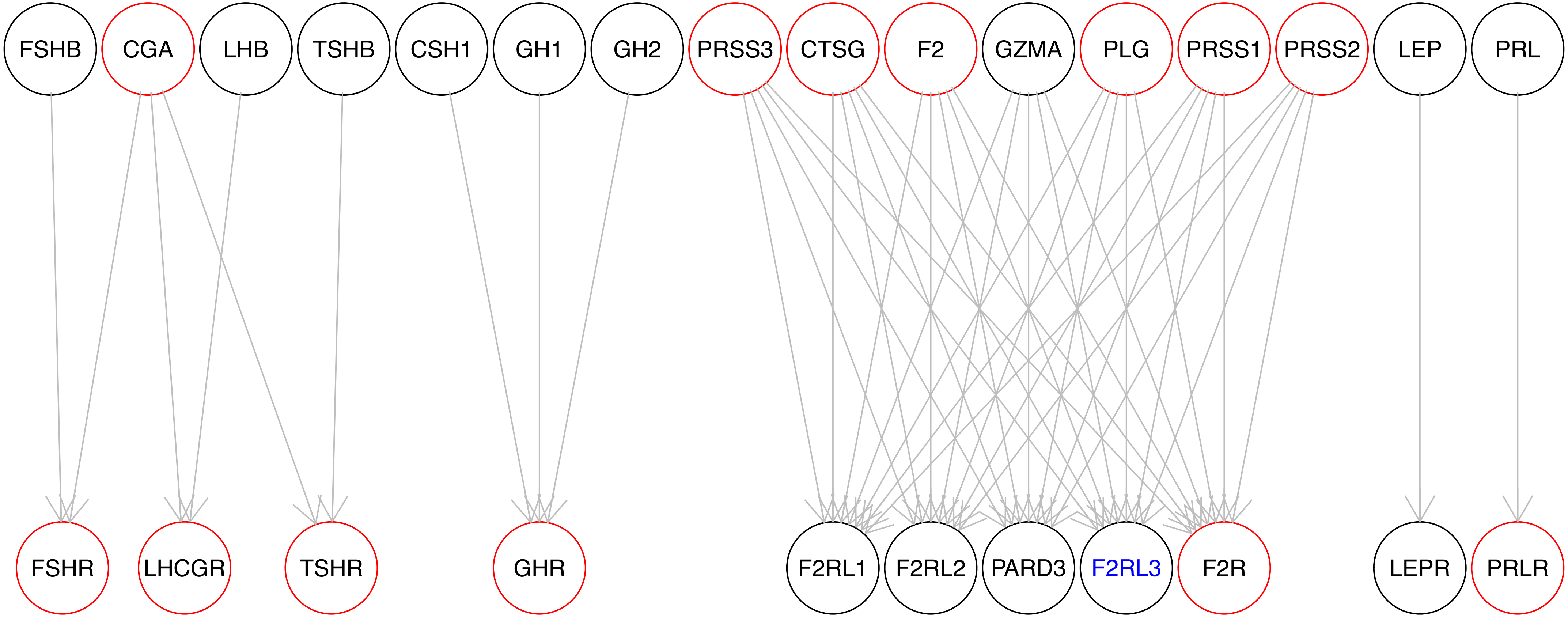

KEGG pathway = Neuroactive ligand−receptor interaction : tumour = MEL.UKRV.Mel.24.Tumor : Yellow Fill = gene variant, Blue Text = expression−survival association, Red Boarder = drug

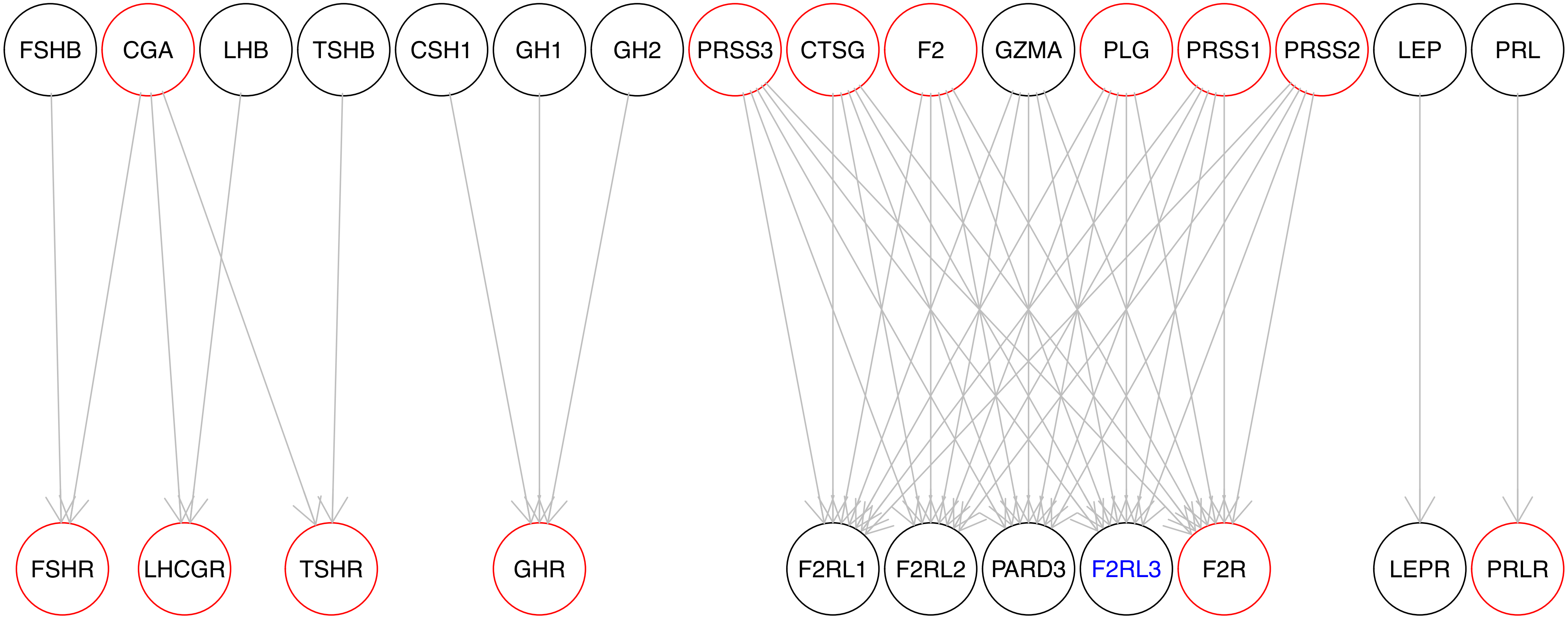

KEGG pathway = Neuroactive ligand−receptor interaction : tumour = MEL.UKRV.Mel.6.Tumor : Yellow Fill = gene variant, Blue Text = expression−survival association, Red Boarder = drug

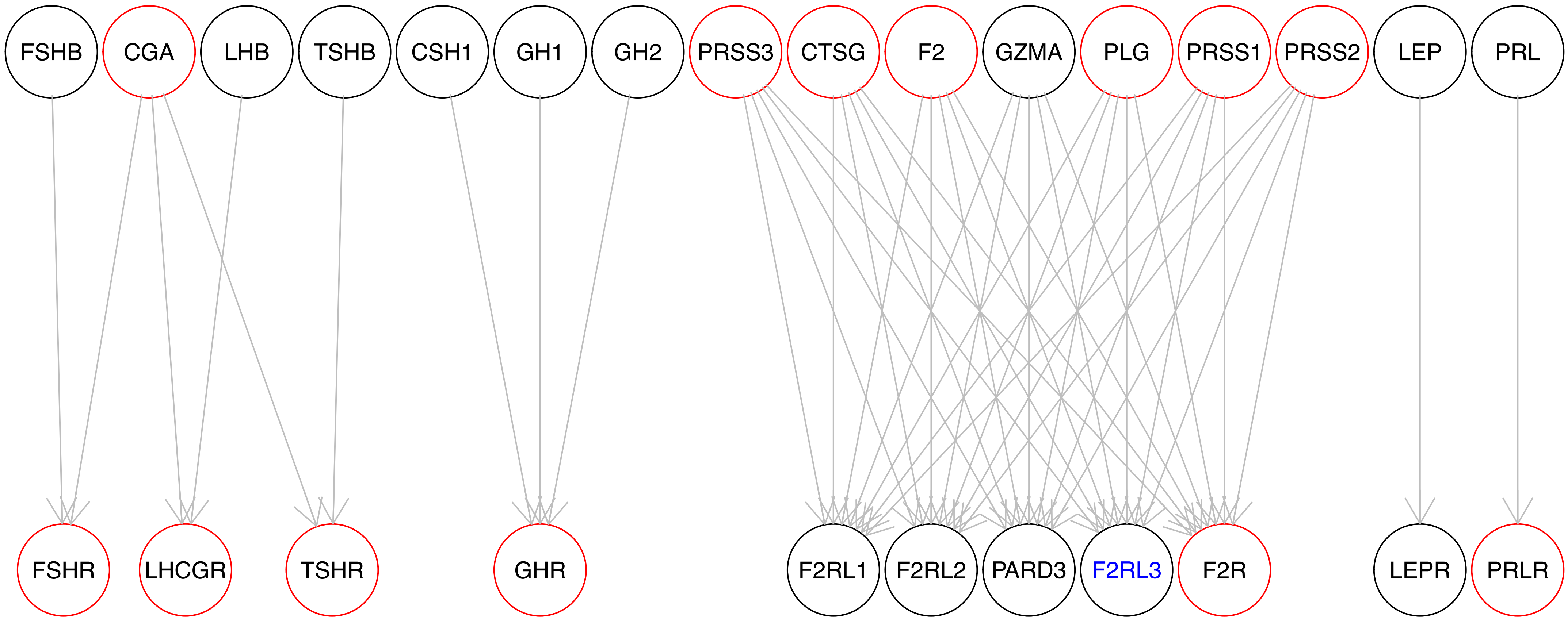

KEGG pathway = Neuroactive ligand−receptor interaction :   tumour = YUAKER :   Yellow Fill = gene variant, Blue Text = expression−survival association, Red Boarder = drug

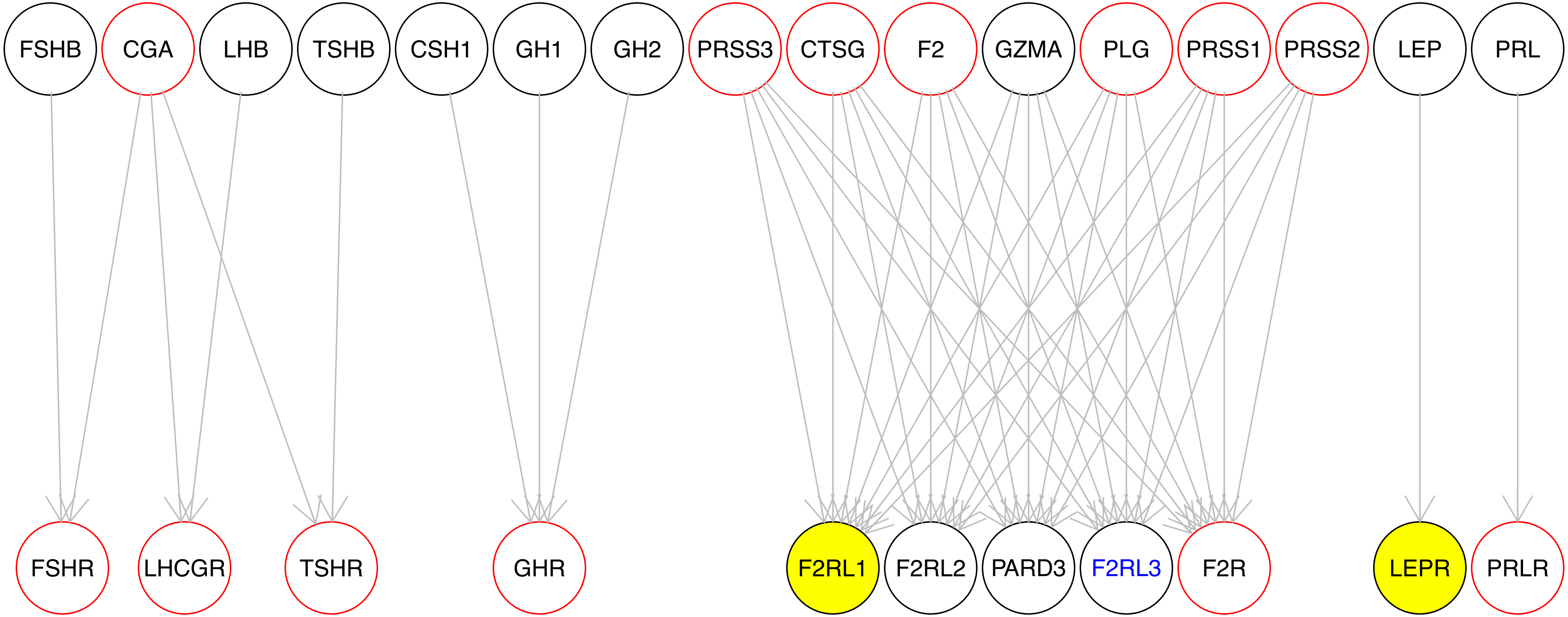

KEGG pathway = Neuroactive ligand−receptor interaction : tumour = YUAVEY : Yellow Fill = gene variant, Blue Text = expression−survival association, Red Boarder = drug

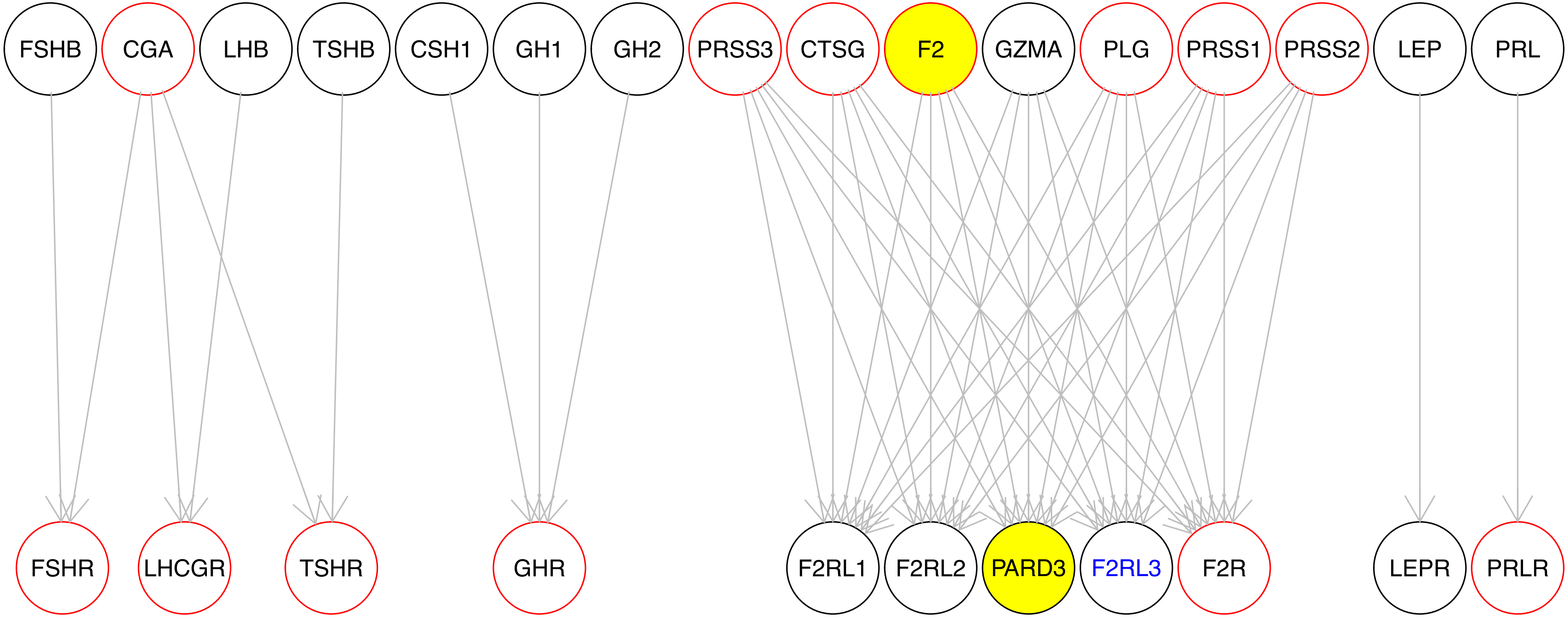

KEGG pathway = Neuroactive ligand−receptor interaction :   tumour = YUBAN :   Yellow Fill = gene variant, Blue Text = expression−survival association, Red Boarder = drug

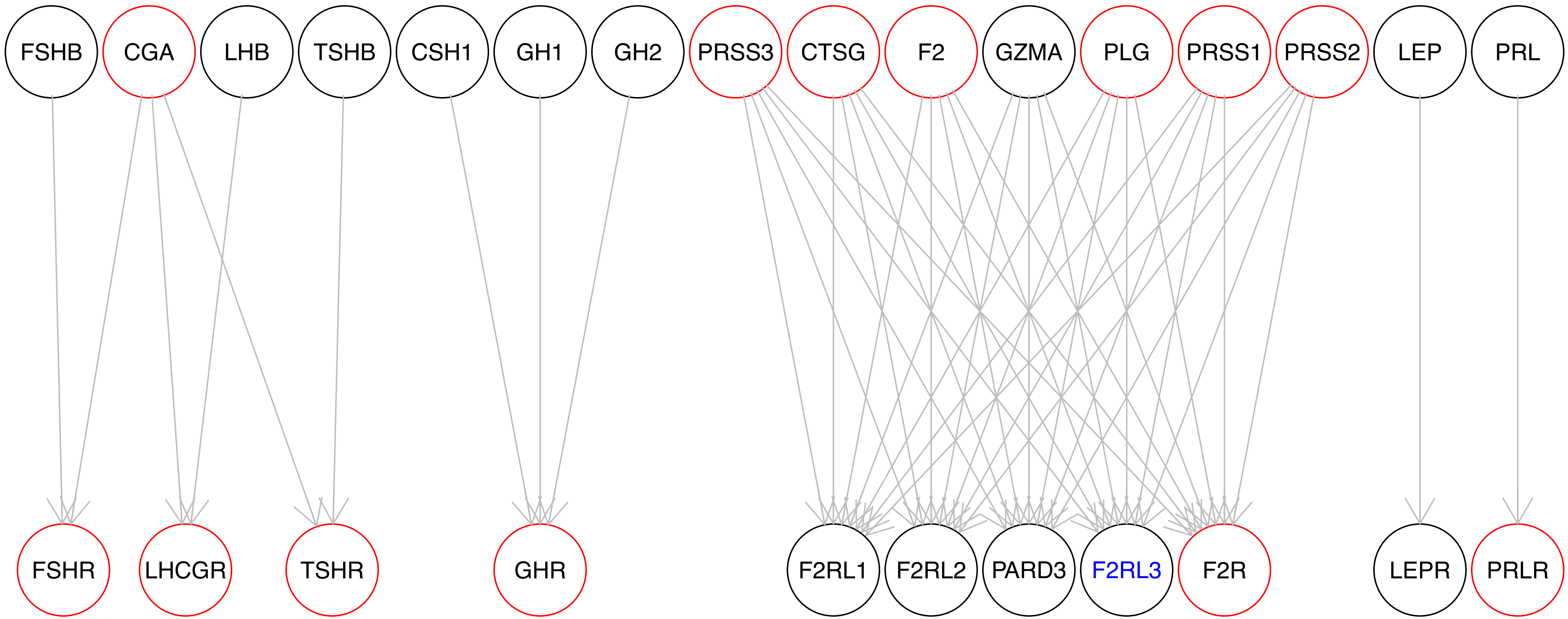

KEGG pathway = Neuroactive ligand−receptor interaction :   tumour = YUBER :   Yellow Fill = gene variant, Blue Text = expression−survival association, Red Boarder = drug

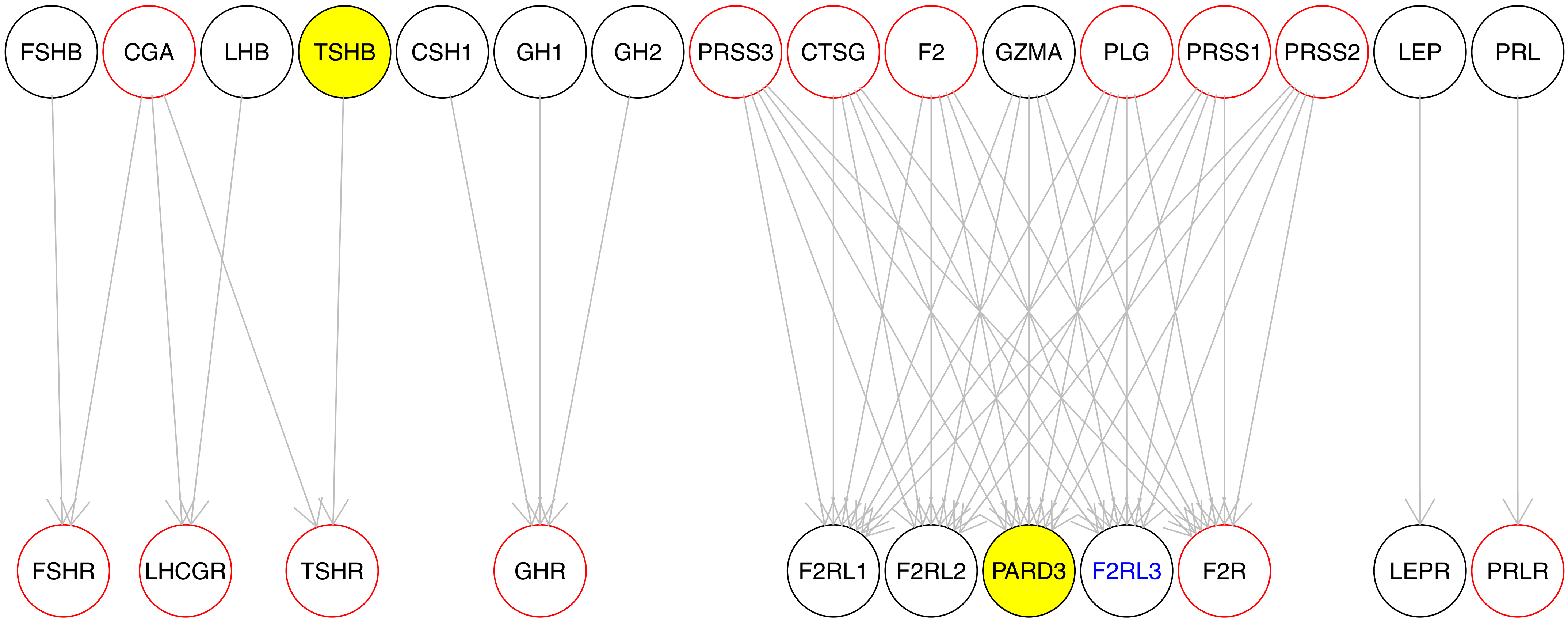

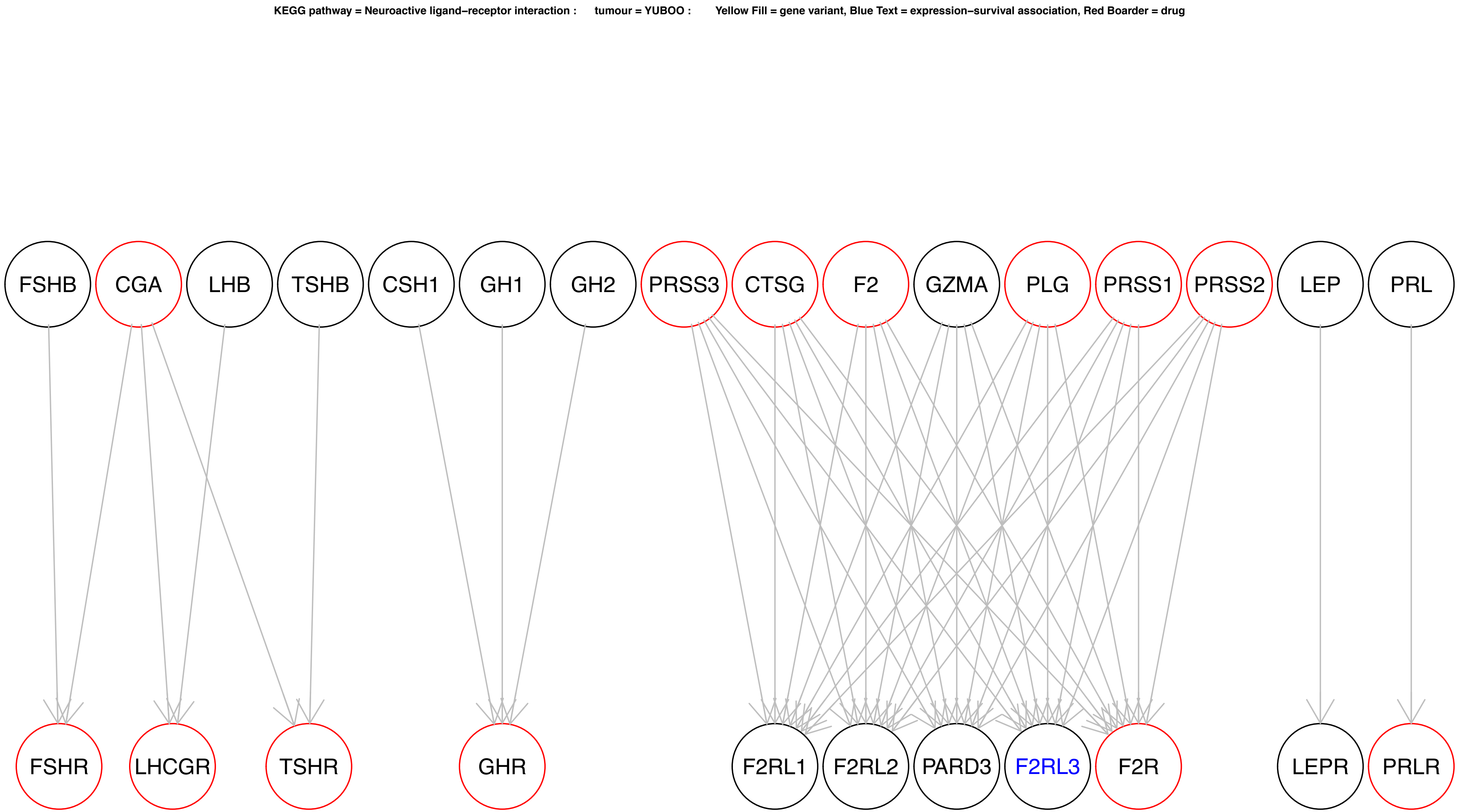
KEGG pathway = Neuroactive ligand−receptor interaction : tumour = YUBOO : Yellow Fill = gene variant, Blue Text = expression−survival association, Red Boarder = drug
FSHB
CGA
LHB
TSHB
CSH1
GH1
GH2
PRSS3
CTSG
F2
GZMA
PLG
PRSS1
PRSS2
LEP
PRL
FSHR
LHCGR
TSHR
GHR
F2RL1
F2RL2
PARD3
F2RL3
F2R
LEPR
PRLR

KEGG pathway = Neuroactive ligand−receptor interaction :   tumour = YUBOT :   Yellow Fill = gene variant, Blue Text = expression−survival association, Red Boarder = drug

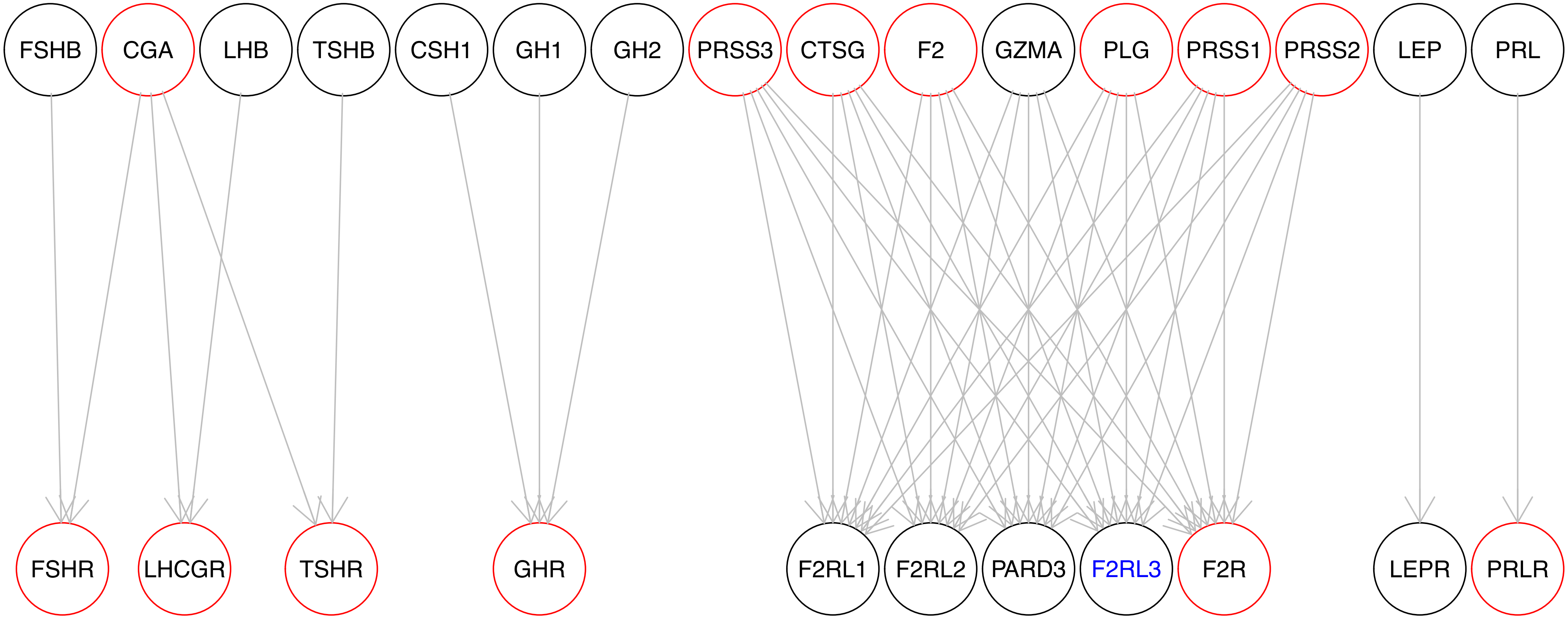

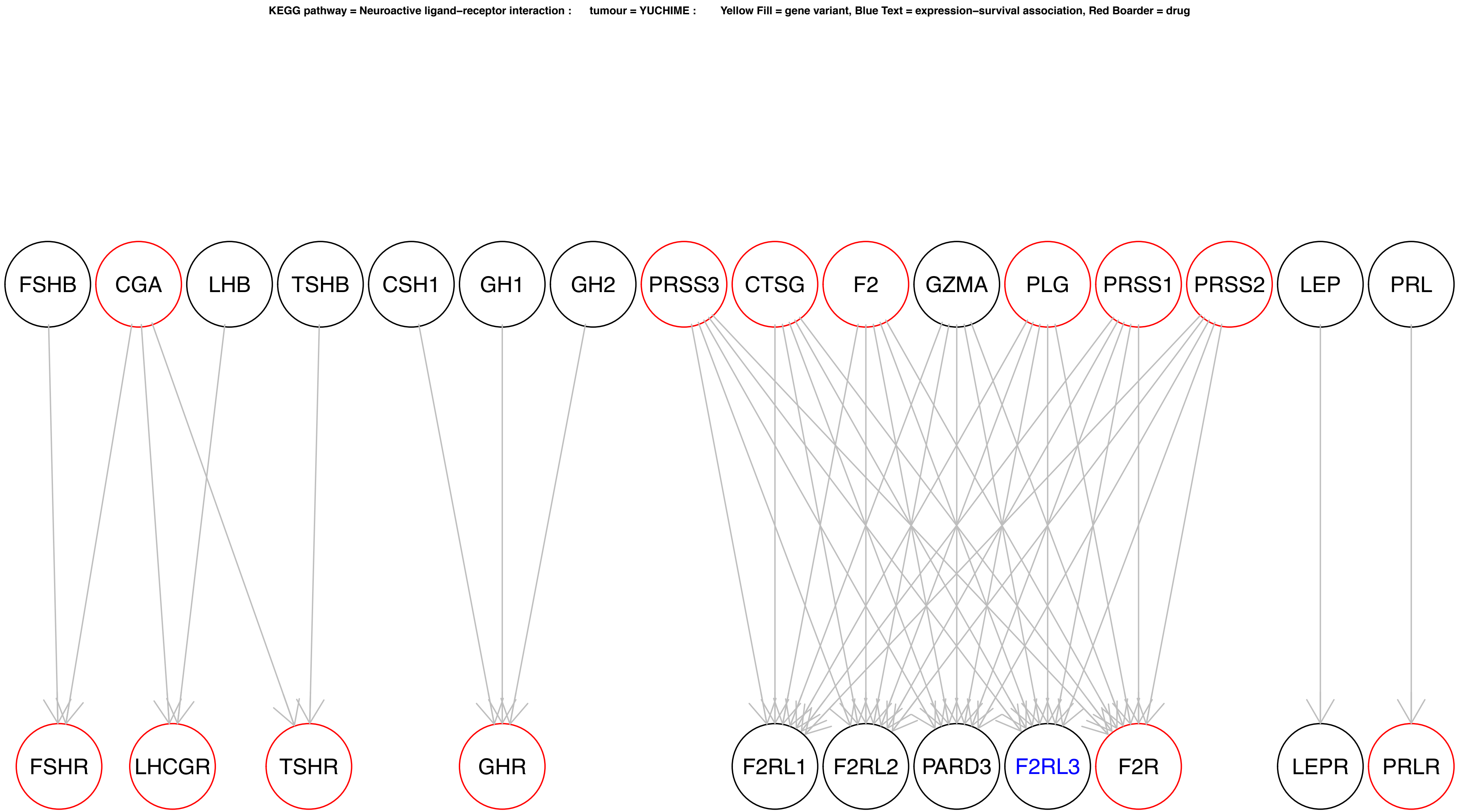
KEGG pathway = Neuroactive ligand−receptor interaction : tumour = YUCHIME : Yellow Fill = gene variant, Blue Text = expression−survival association, Red Boarder = drug
FSHB
CGA
LHB
TSHB
CSH1
GH1
GH2
PRSS3
CTSG
F2
GZMA
PLG
PRSS1
PRSS2
LEP
PRL
FSHR
LHCGR
TSHR
GHR
F2RL1
F2RL2
PARD3
F2RL3
F2R
LEPR
PRLR

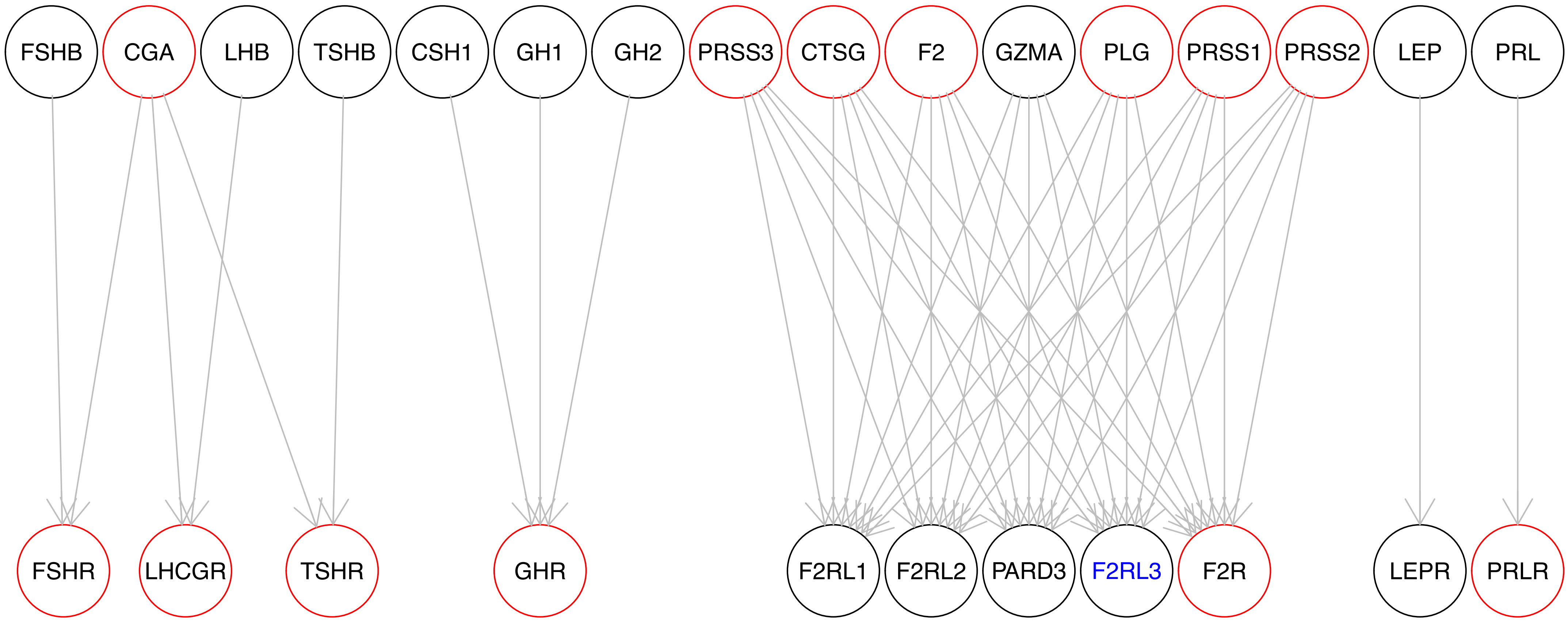
FSHB
CGA
LHB
TSHB
CSH1
GH1
GH2
PRSS3
CTSG
F2
GZMA
PLG
PRSS1
PRSS2
LEP
PRL
FSHR
LHCGR
TSHR
GHR
F2RL1
F2RL2
PARD3
F2RL3
F2R
LEPR
PRLR

KEGG pathway = Neuroactive ligand−receptor interaction : tumour = YUCLAT : Yellow Fill = gene variant, Blue Text = expression−survival association, Red Boarder = drug

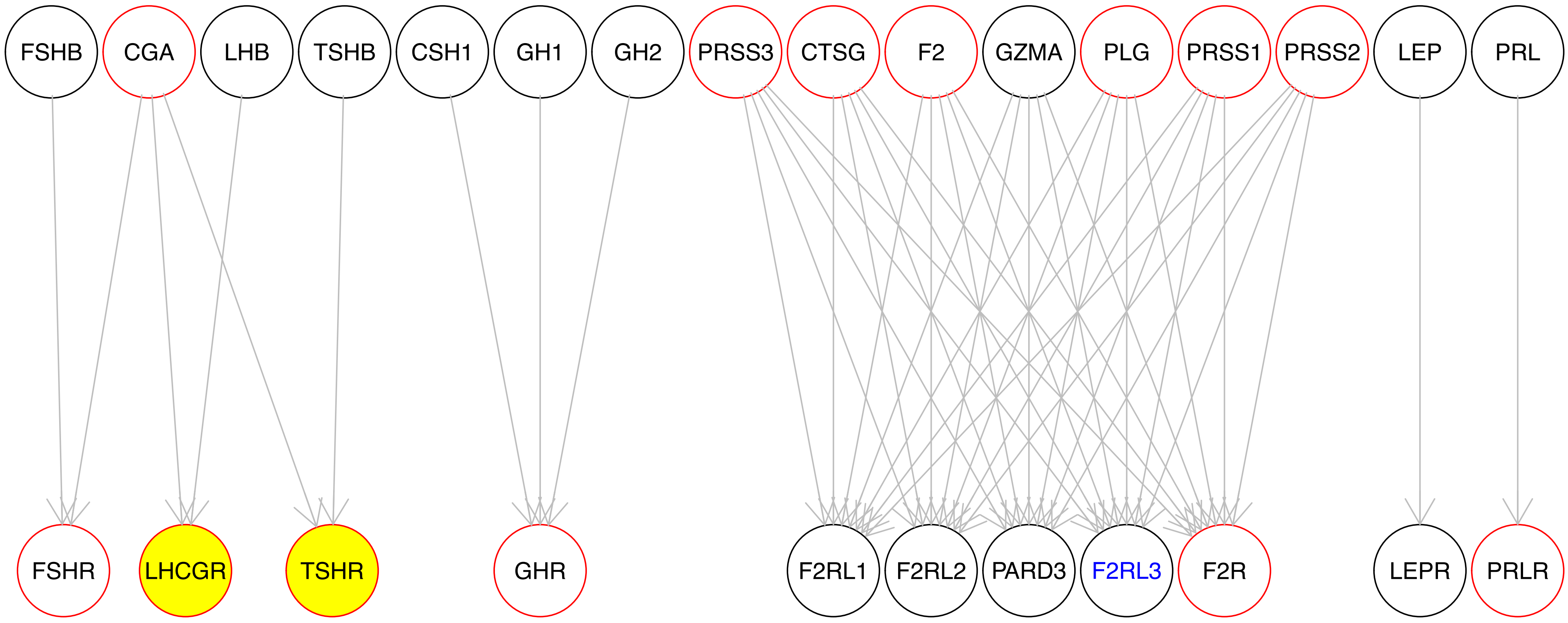

KEGG pathway = Neuroactive ligand−receptor interaction :   tumour = YUCOT :   Yellow Fill = gene variant, Blue Text = expression−survival association, Red Boarder = drug

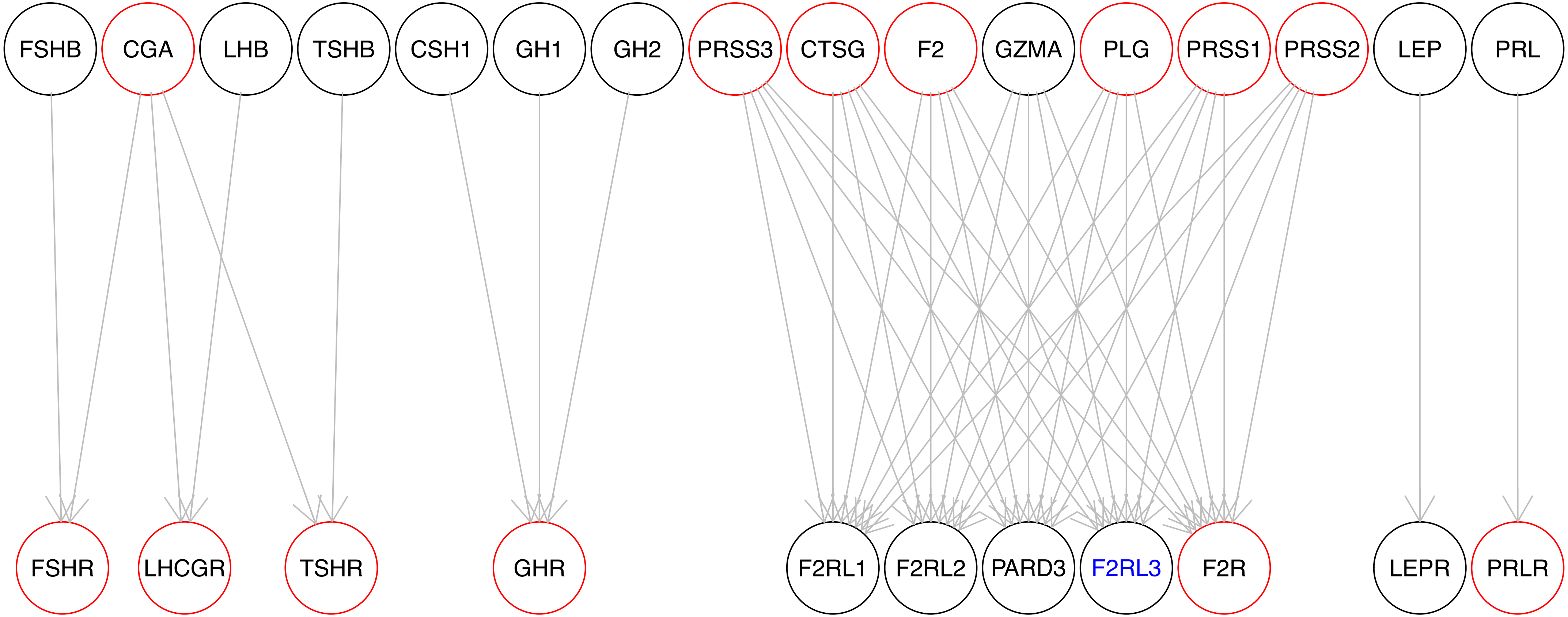

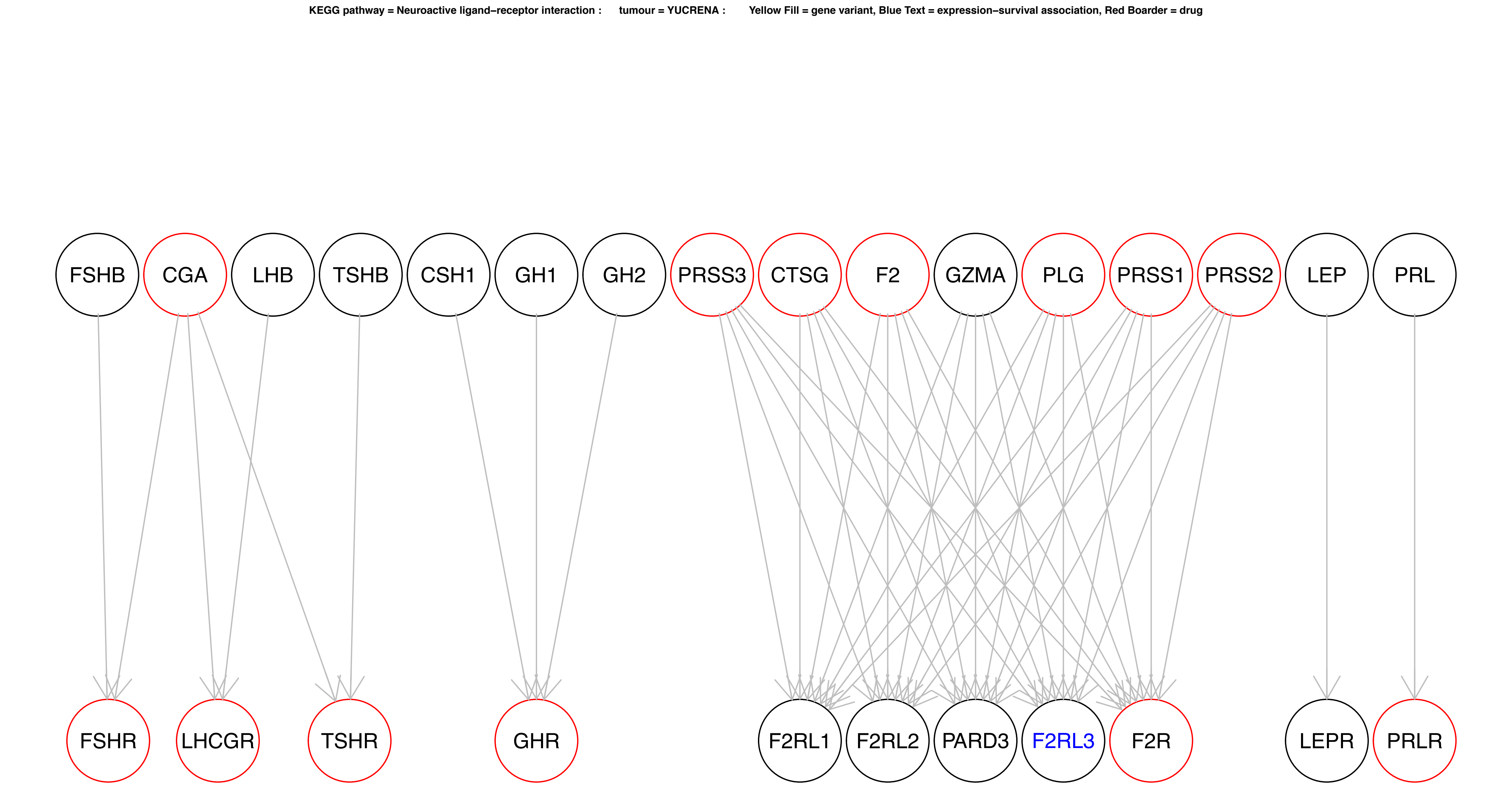
KEGG pathway = Neuroactive ligand−receptor interaction :   tumour = YUCRENA :   Yellow Fill = gene variant, Blue Text = expression−survival association, Red Boarder = drug
FSHB
CGA
LHB
TSHB
CSH1
GH1
GH2
PRSS3
CTSG
F2
GZMA
PLG
PRSS1
PRSS2
LEP
PRL
FSHR
LHCGR
TSHR
GHR
F2RL1
F2RL2
PARD3
F2RL3
F2R
LEPR
PRLR

KEGG pathway = Neuroactive ligand−receptor interaction : tumour = YUCROFT : Yellow Fill = gene variant, Blue Text = expression−survival association, Red Boarder = drug

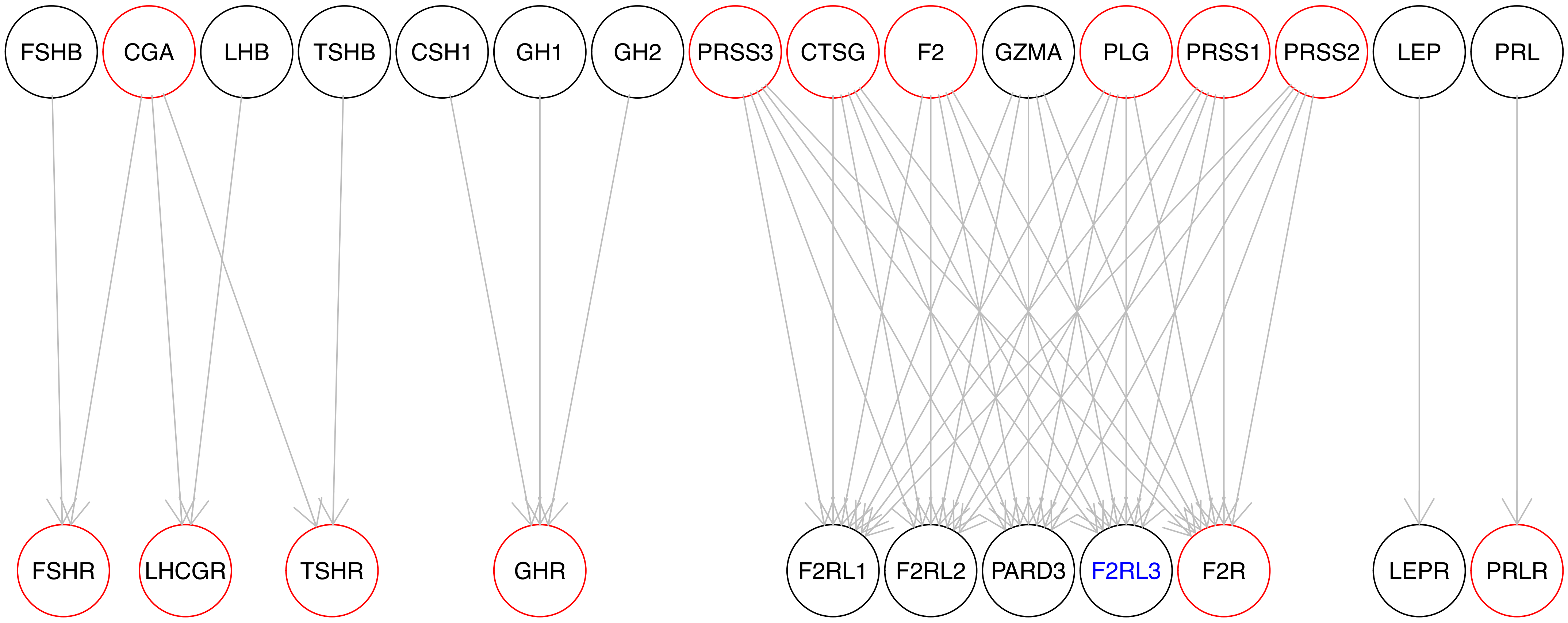

KEGG pathway = Neuroactive ligand−receptor interaction : tumour = YUDAB : Yellow Fill = gene variant, Blue Text = expression−survival association, Red Boarder = drug

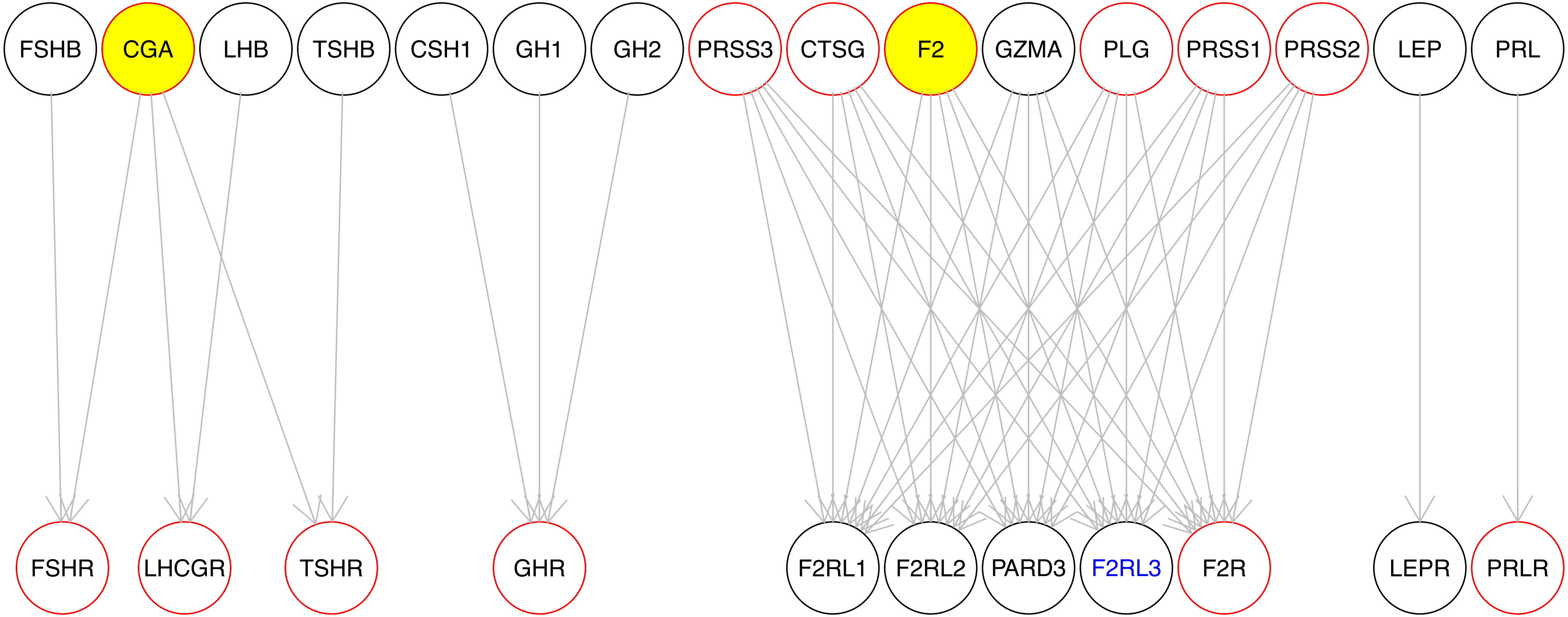

KEGG pathway = Neuroactive ligand−receptor interaction : tumour = YUDARE : Yellow Fill = gene variant, Blue Text = expression−survival association, Red Boarder = drug

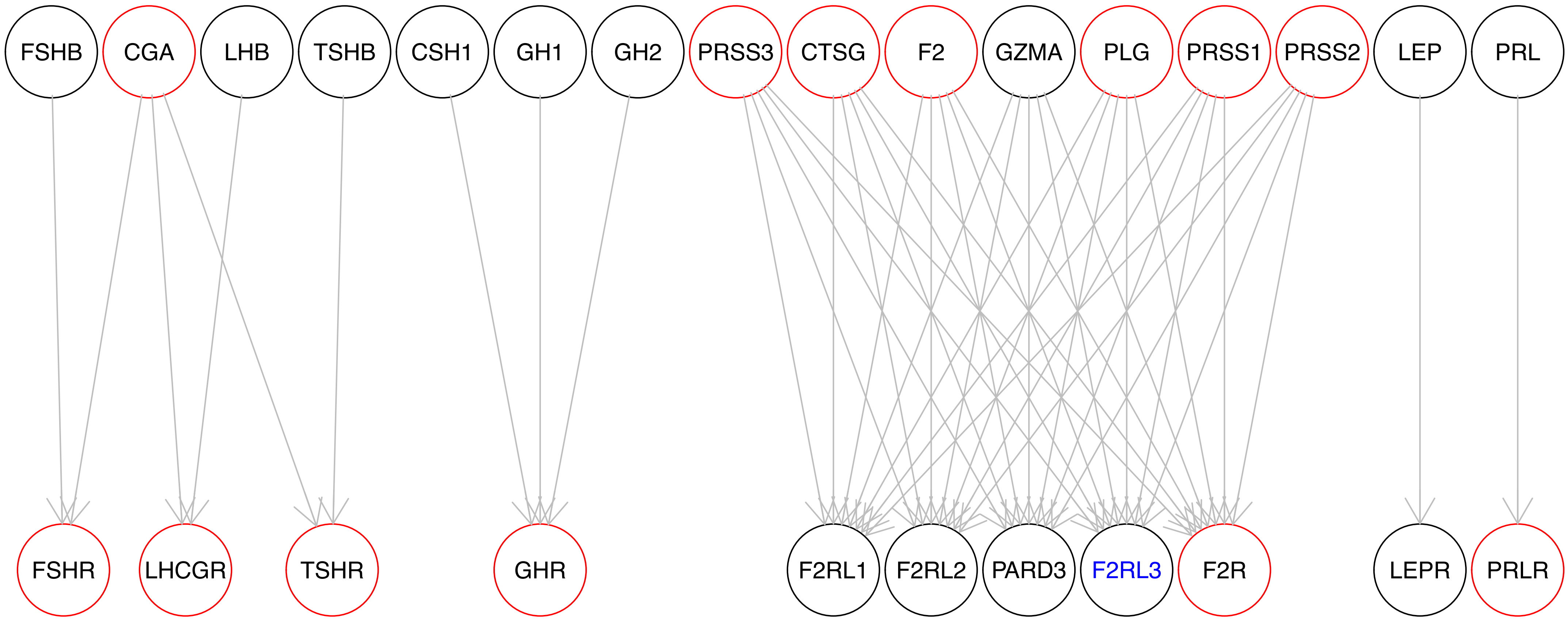

KEGG pathway = Neuroactive ligand−receptor interaction : tumour = YUDATE : Yellow Fill = gene variant, Blue Text = expression−survival association, Red Boarder = drug

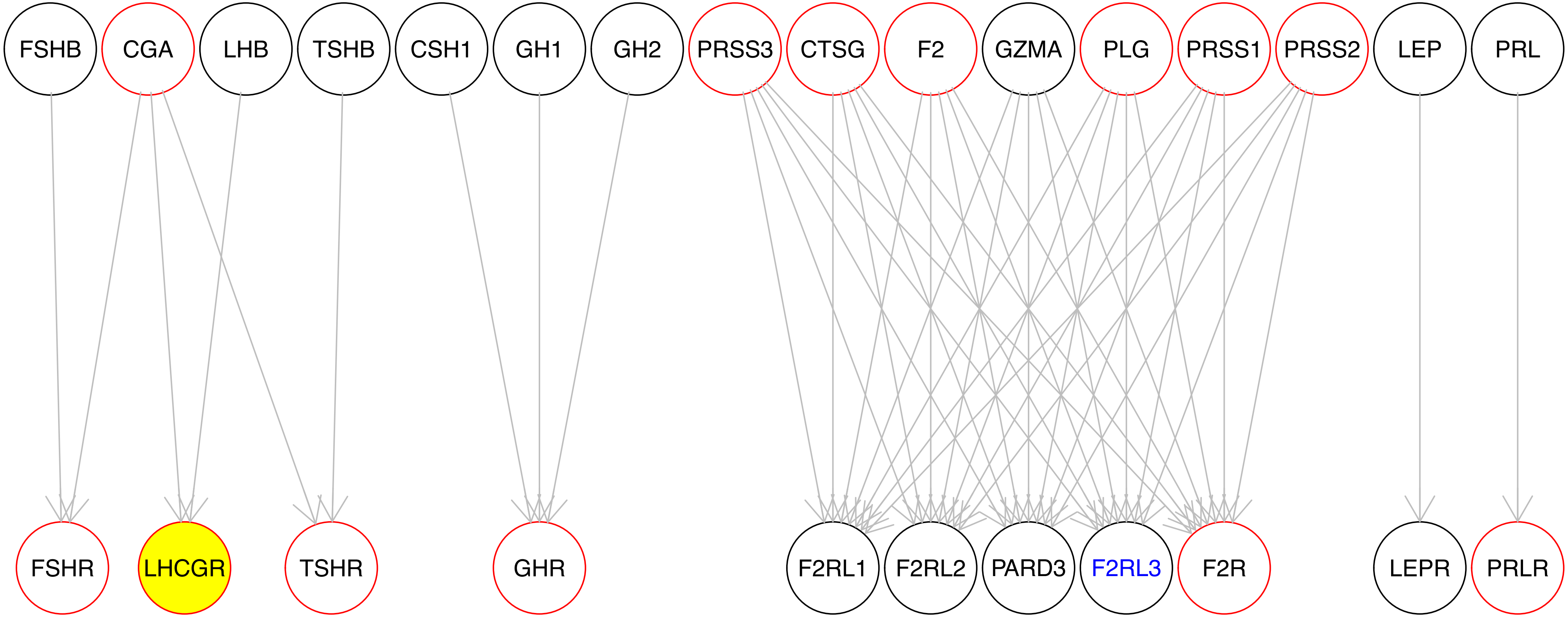

KEGG pathway = Neuroactive ligand−receptor interaction : tumour = YUDEDE : Yellow Fill = gene variant, Blue Text = expression−survival association, Red Boarder = drug

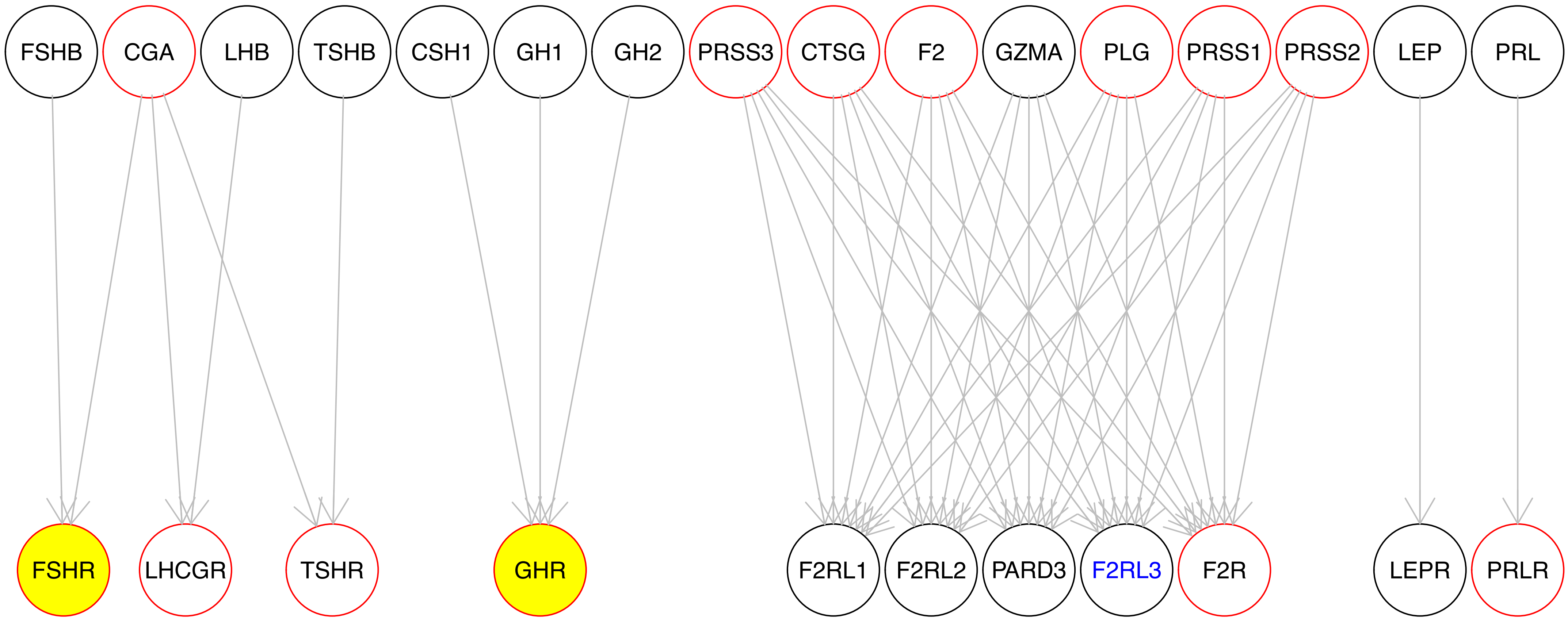

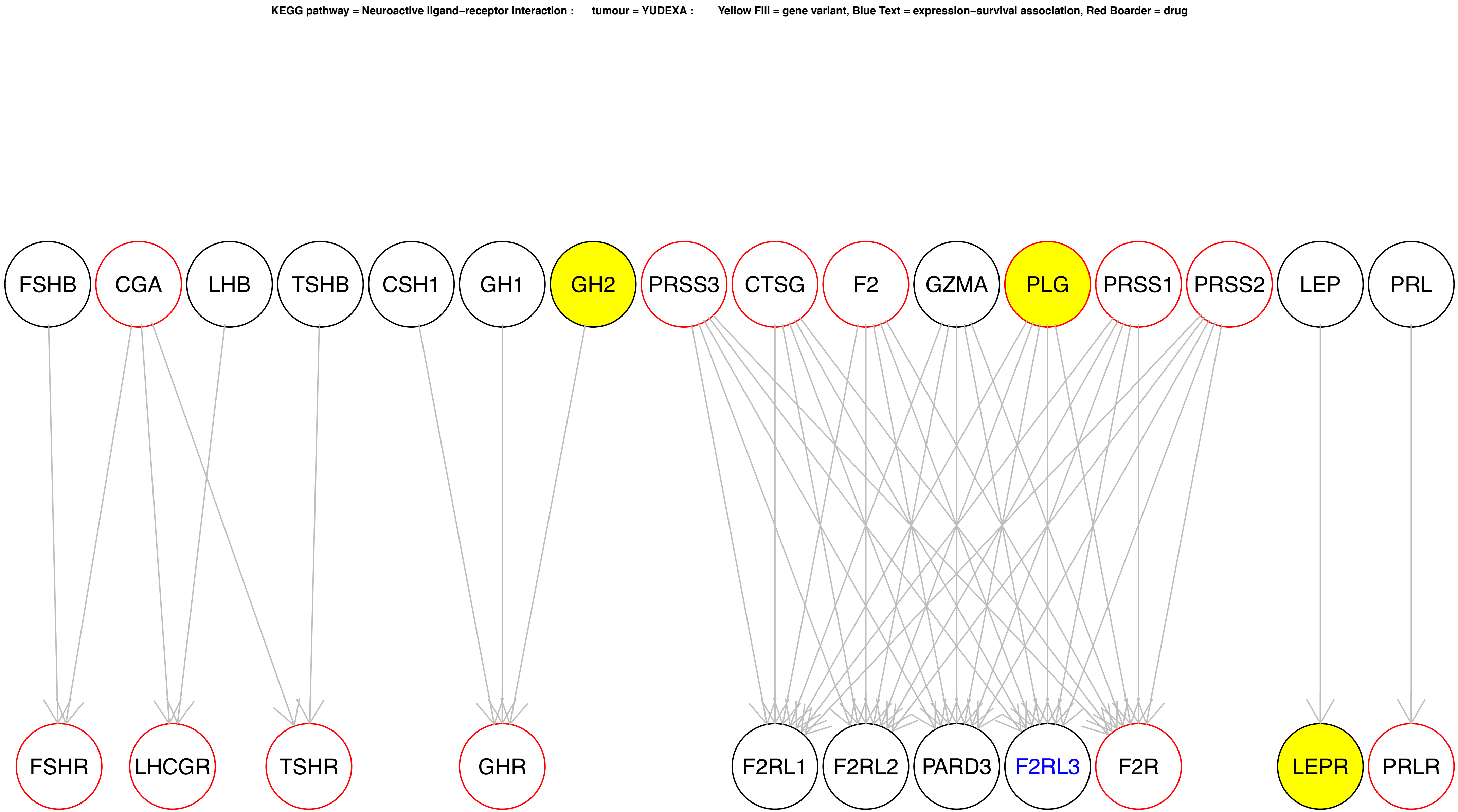
KEGG pathway = Neuroactive ligand−receptor interaction : tumour = YUDEXA : Yellow Fill = gene variant, Blue Text = expression−survival association, Red Boarder = drug
FSHB
CGA
LHB
TSHB
CSH1
GH1
GH2
PRSS3
CTSG
F2
GZMA
PLG
PRSS1
PRSS2
LEP
PRL
FSHR
LHCGR
TSHR
GHR
F2RL1
F2RL2
PARD3
F2RL3
F2R
LEPR
PRLR

KEGG pathway = Neuroactive ligand–receptor interaction : tumour = YUDIALE : Yellow Fill = gene variant, Blue Text = expression–survival association, Red Boarder = drug

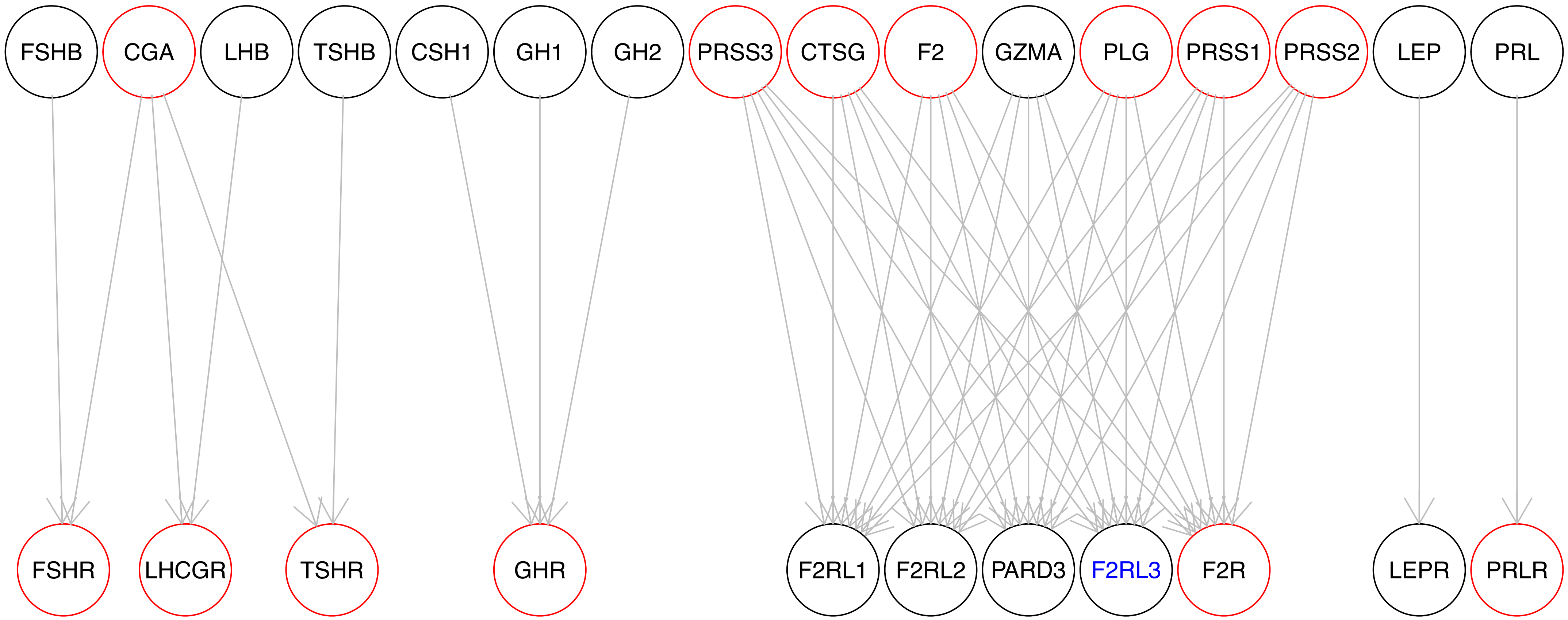

KEGG pathway = Neuroactive ligand−receptor interaction : tumour = YUDUTY : Yellow Fill = gene variant, Blue Text = expression−survival association, Red Boarder = drug

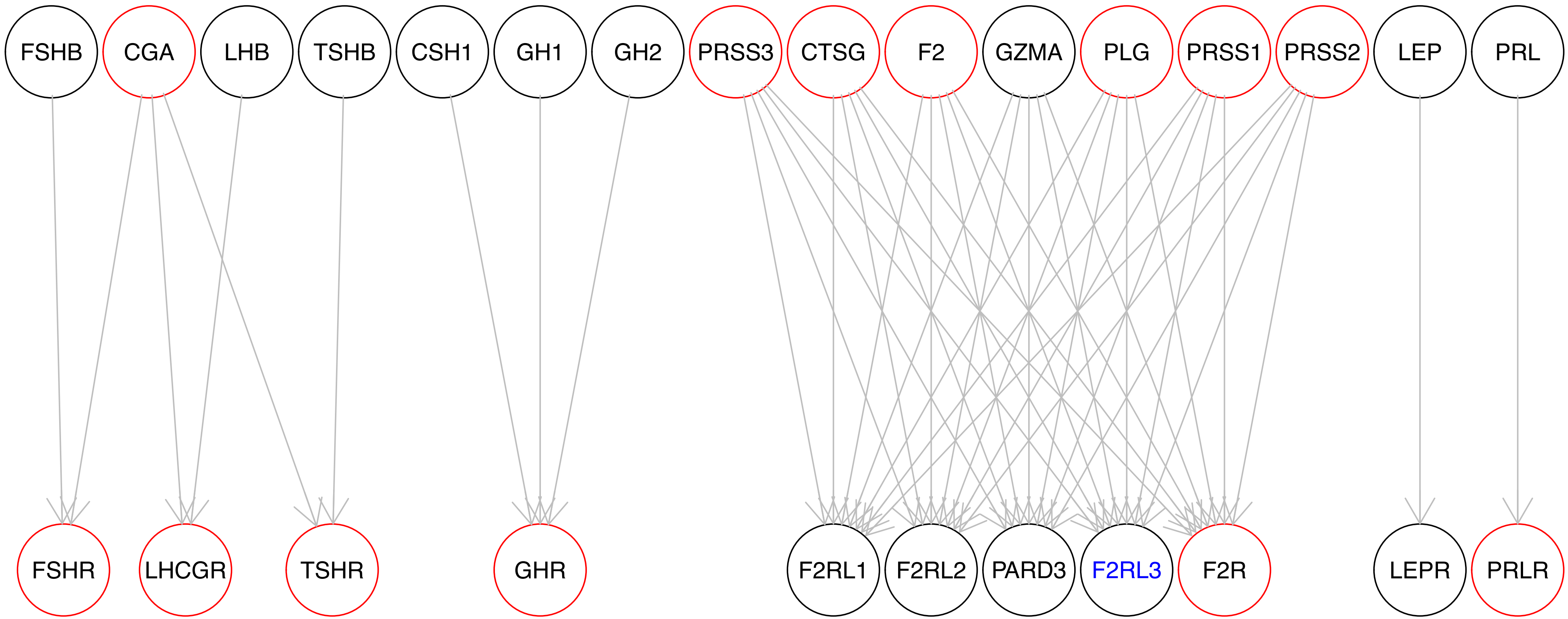

KEGG pathway = Neuroactive ligand−receptor interaction :   tumour = YUFAR :   Yellow Fill = gene variant, Blue Text = expression−survival association, Red Boarder = drug

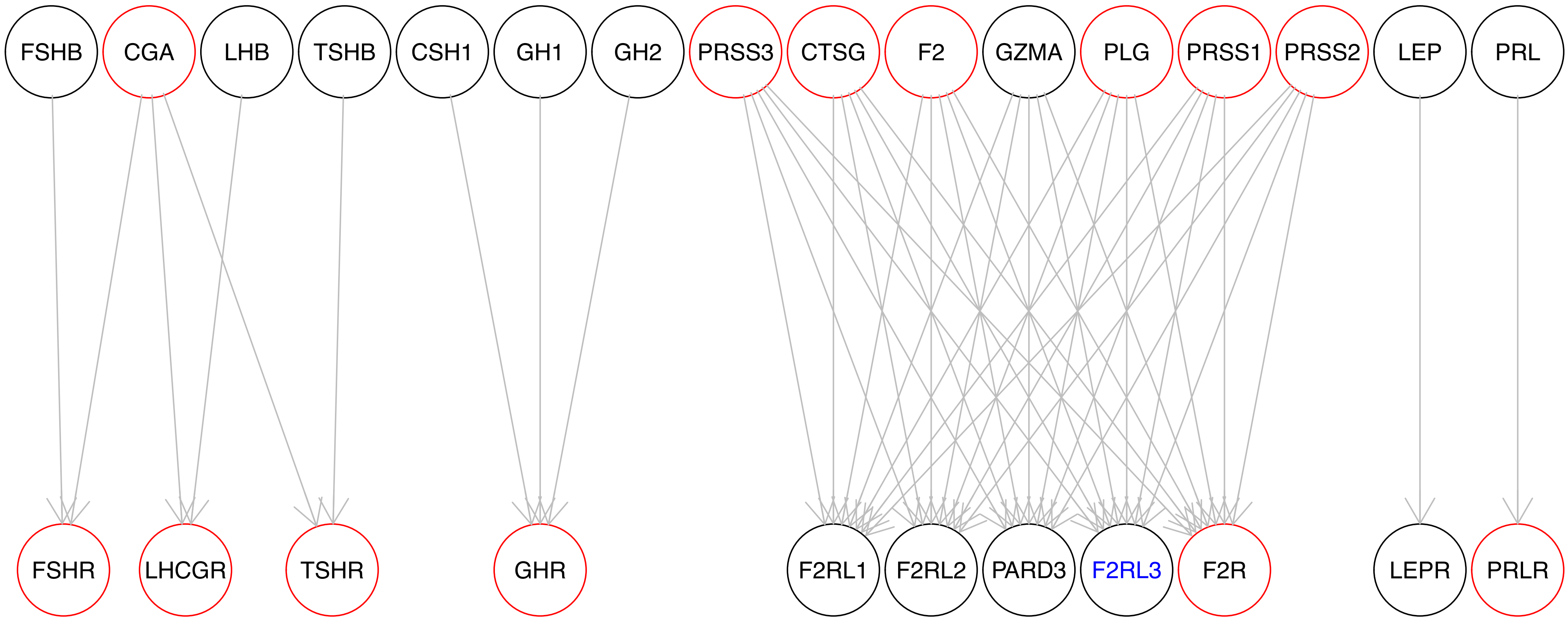

KEGG pathway = Neuroactive ligand−receptor interaction : tumour = YUFARCI : Yellow Fill = gene variant, Blue Text = expression−survival association, Red Boarder = drug

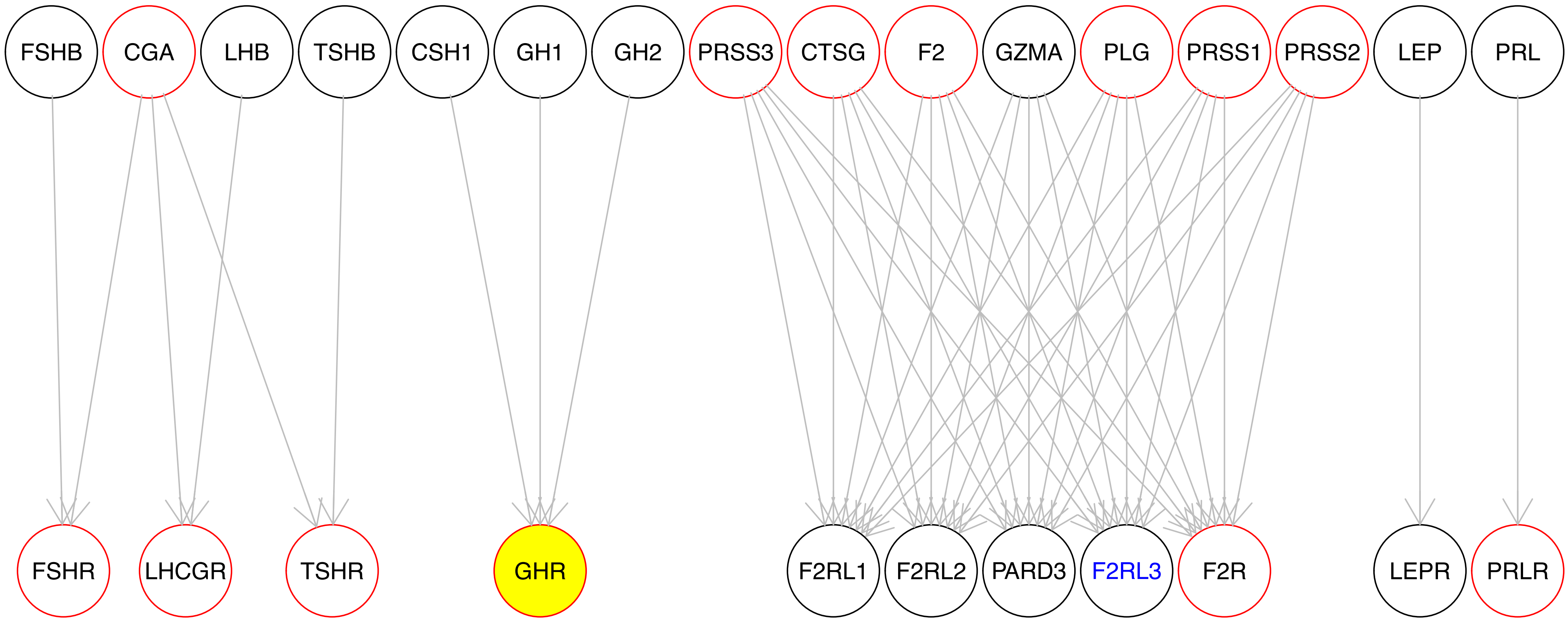

KEGG pathway = Neuroactive ligand−receptor interaction :   tumour = YUFLA :   Yellow Fill = gene variant, Blue Text = expression−survival association, Red Boarder = drug

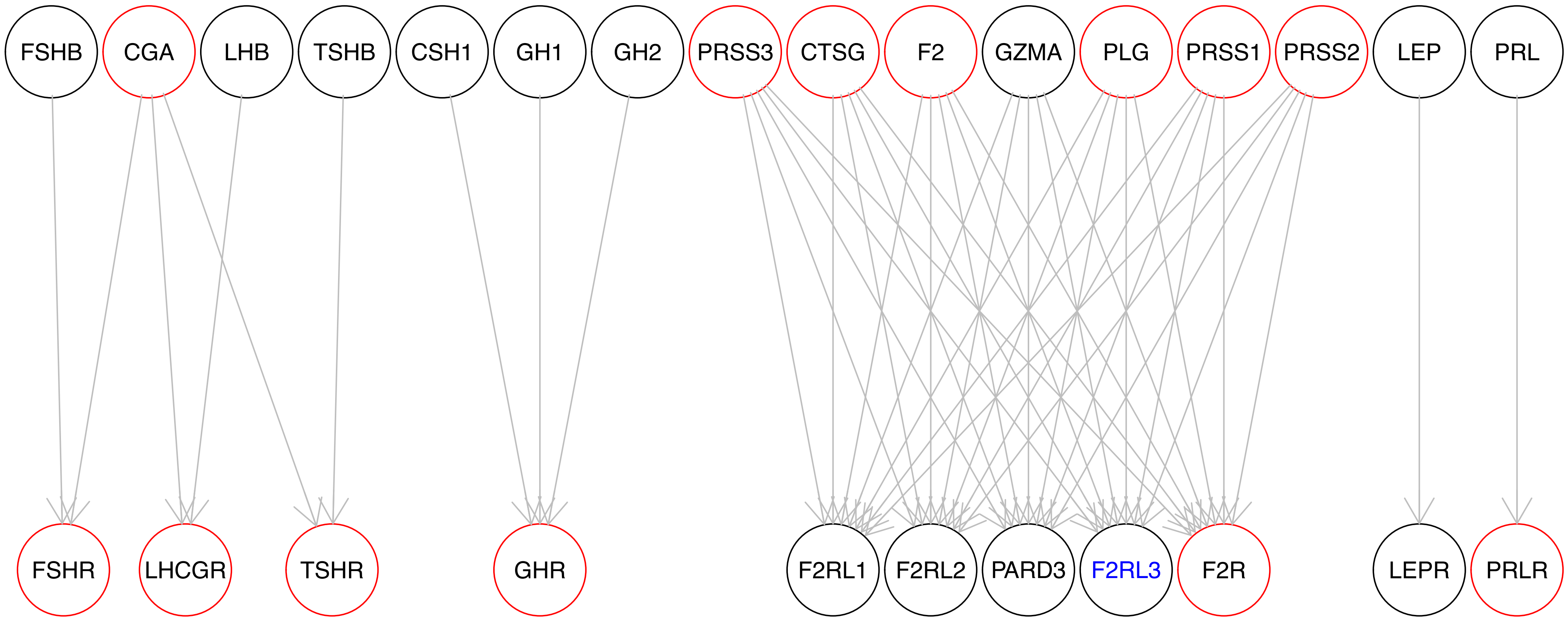

KEGG pathway = Neuroactive ligand−receptor interaction : tumour = YUFOLD : Yellow Fill = gene variant, Blue Text = expression−survival association, Red Boarder = drug

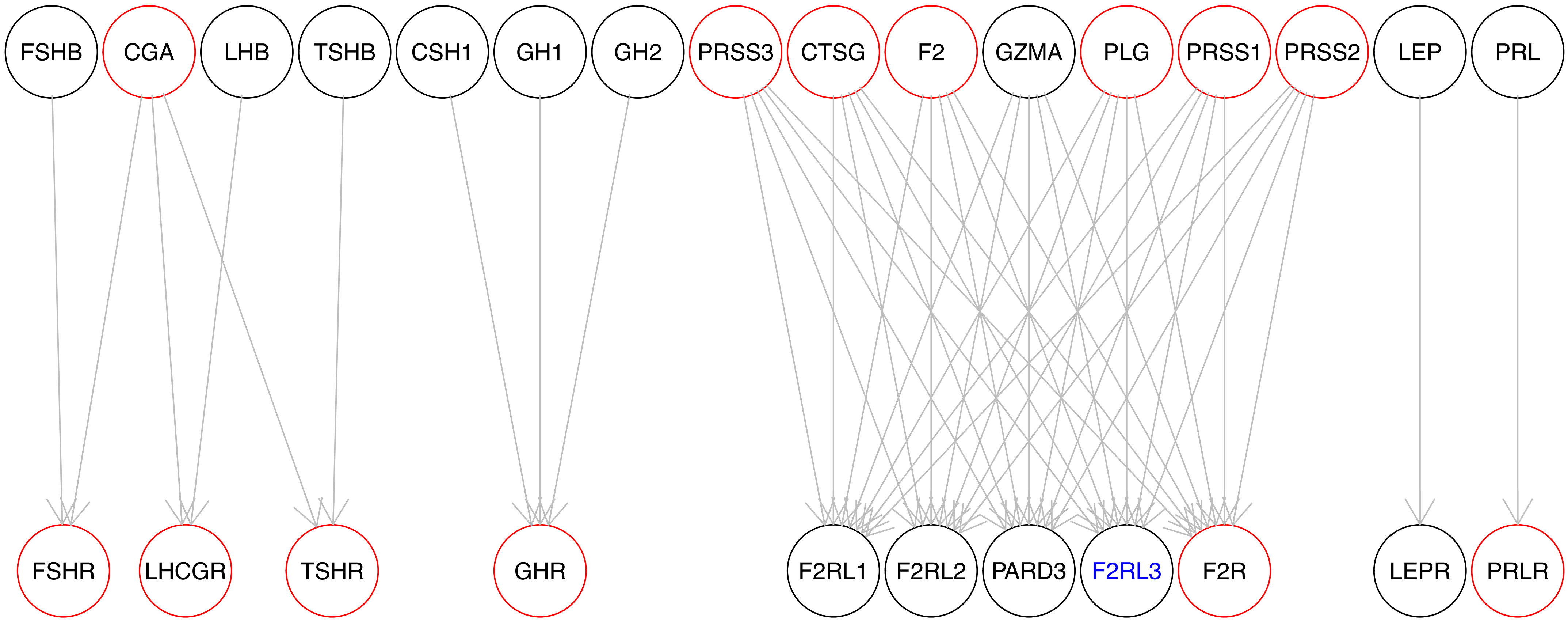

KEGG pathway = Neuroactive ligand−receptor interaction :   tumour = YUFOR :   Yellow Fill = gene variant, Blue Text = expression−survival association, Red Boarder = drug

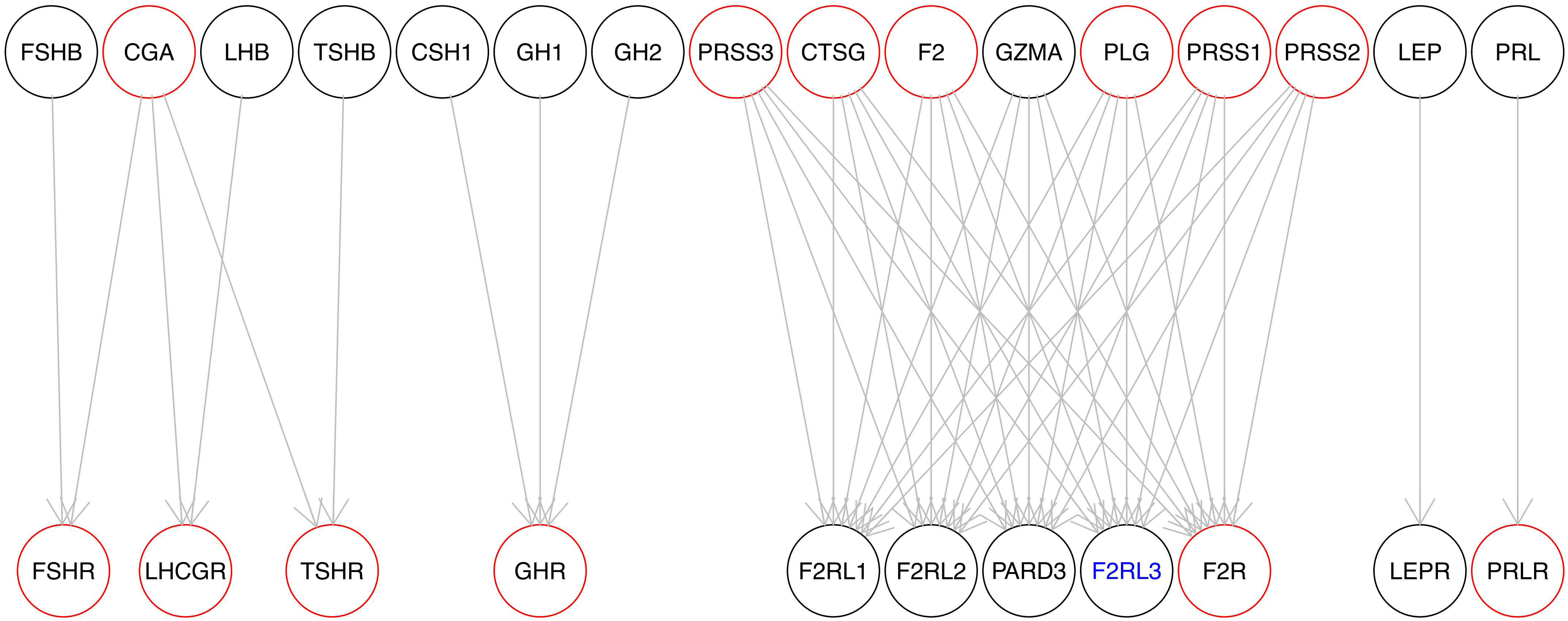

KEGG pathway = Neuroactive ligand−receptor interaction : tumour = YUGAFFE : Yellow Fill = gene variant, Blue Text = expression−survival association, Red Boarder = drug

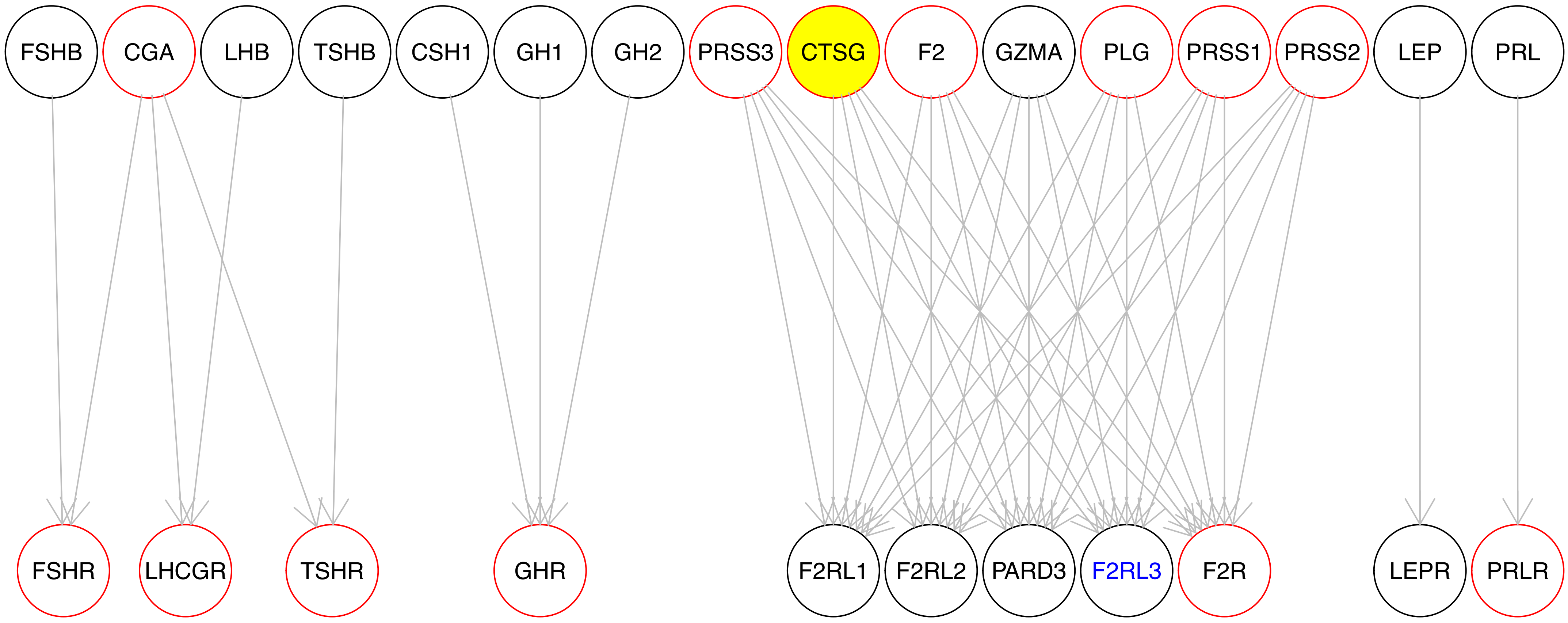

KEGG pathway = Neuroactive ligand−receptor interaction :   tumour = YUGANK :   Yellow Fill = gene variant, Blue Text = expression−survival association, Red Boarder = drug

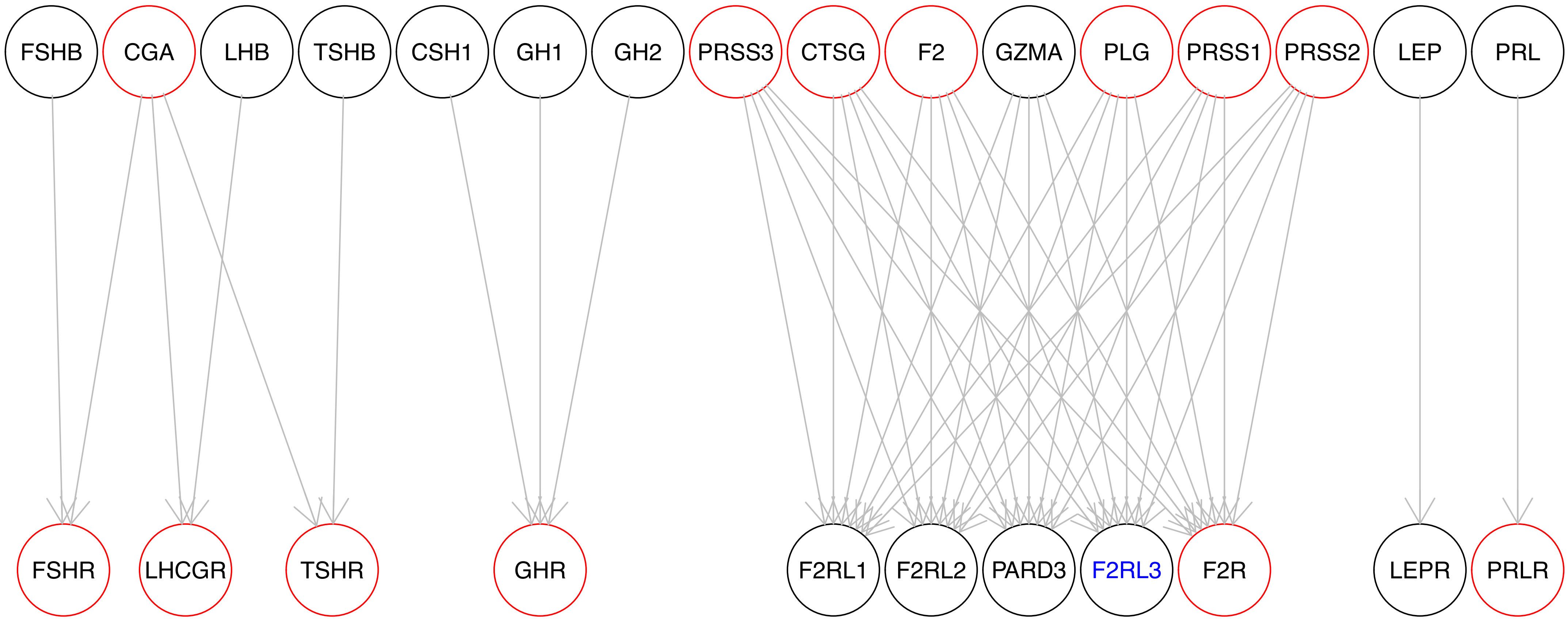

KEGG pathway = Neuroactive ligand−receptor interaction : tumour = YUGASP : Yellow Fill = gene variant, Blue Text = expression−survival association, Red Boarder = drug

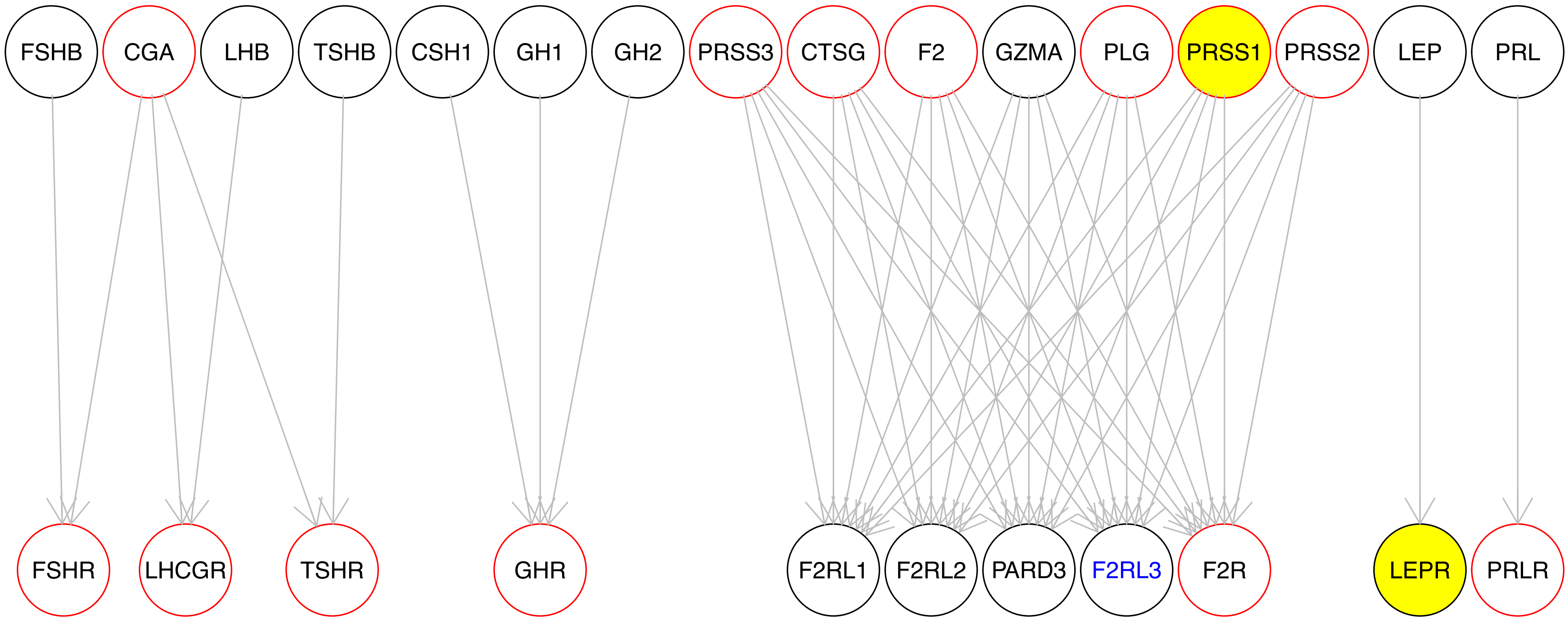

KEGG pathway = Neuroactive ligand−receptor interaction : tumour = YUGISMO : Yellow Fill = gene variant, Blue Text = expression−survival association, Red Boarder = drug

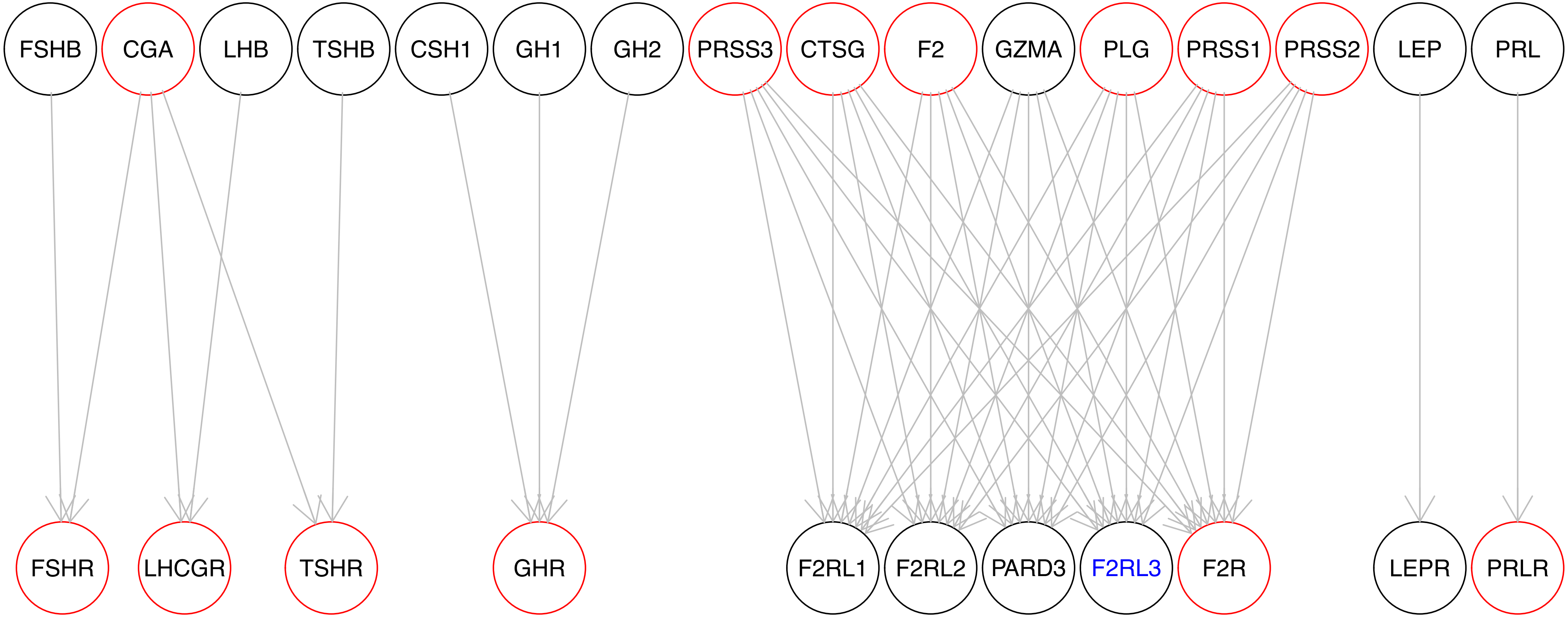

KEGG pathway = Neuroactive ligand−receptor interaction : tumour = YUGOE : Yellow Fill = gene variant, Blue Text = expression−survival association, Red Boarder = drug

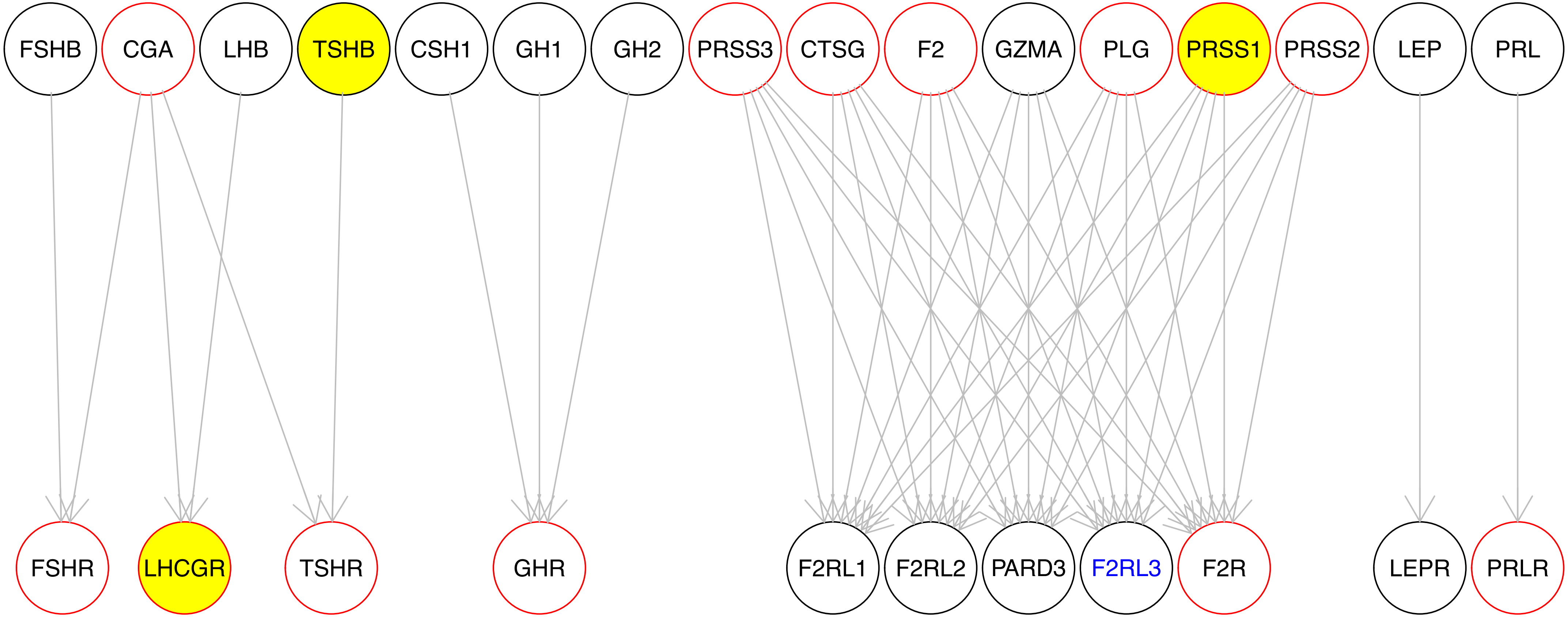

KEGG pathway = Neuroactive ligand−receptor interaction : tumour = YUHEF : Yellow Fill = gene variant, Blue Text = expression−survival association, Red Boarder = drug

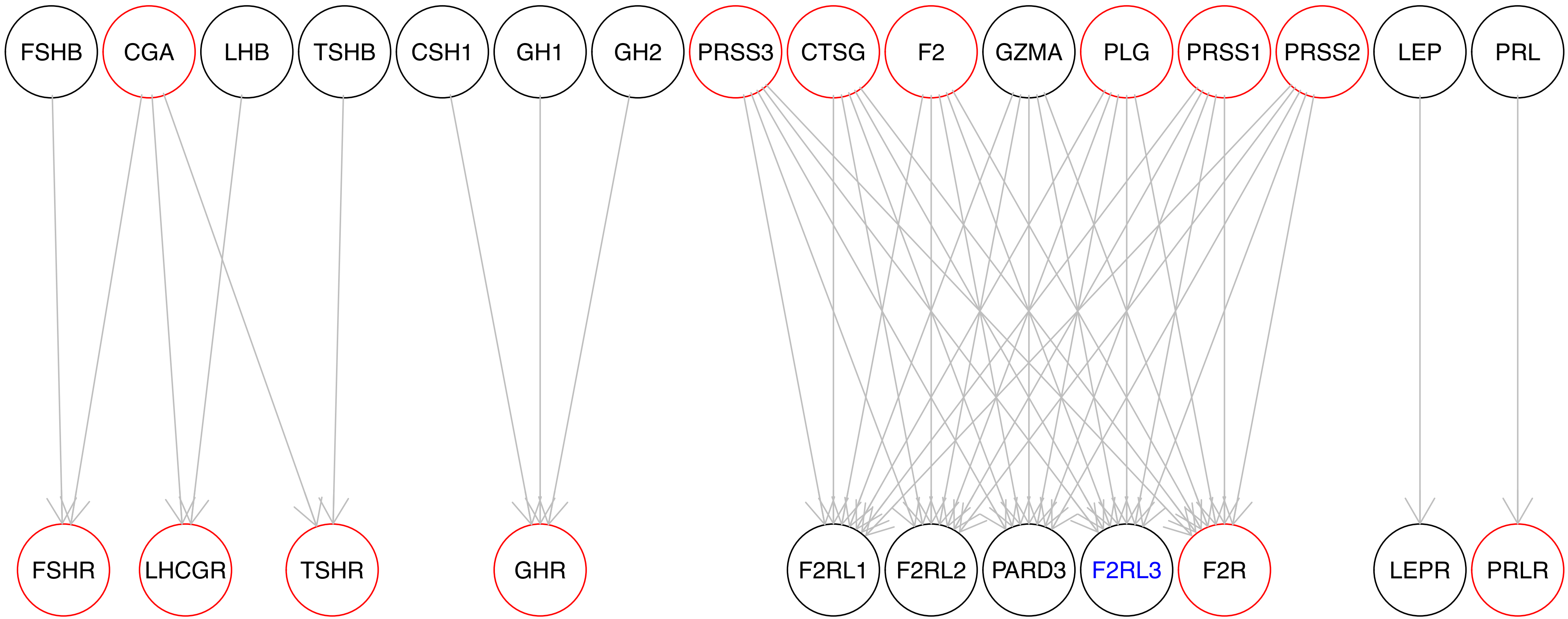

KEGG pathway = Neuroactive ligand−receptor interaction :   tumour = YUHOIN :   Yellow Fill = gene variant, Blue Text = expression−survival association, Red Boarder = drug

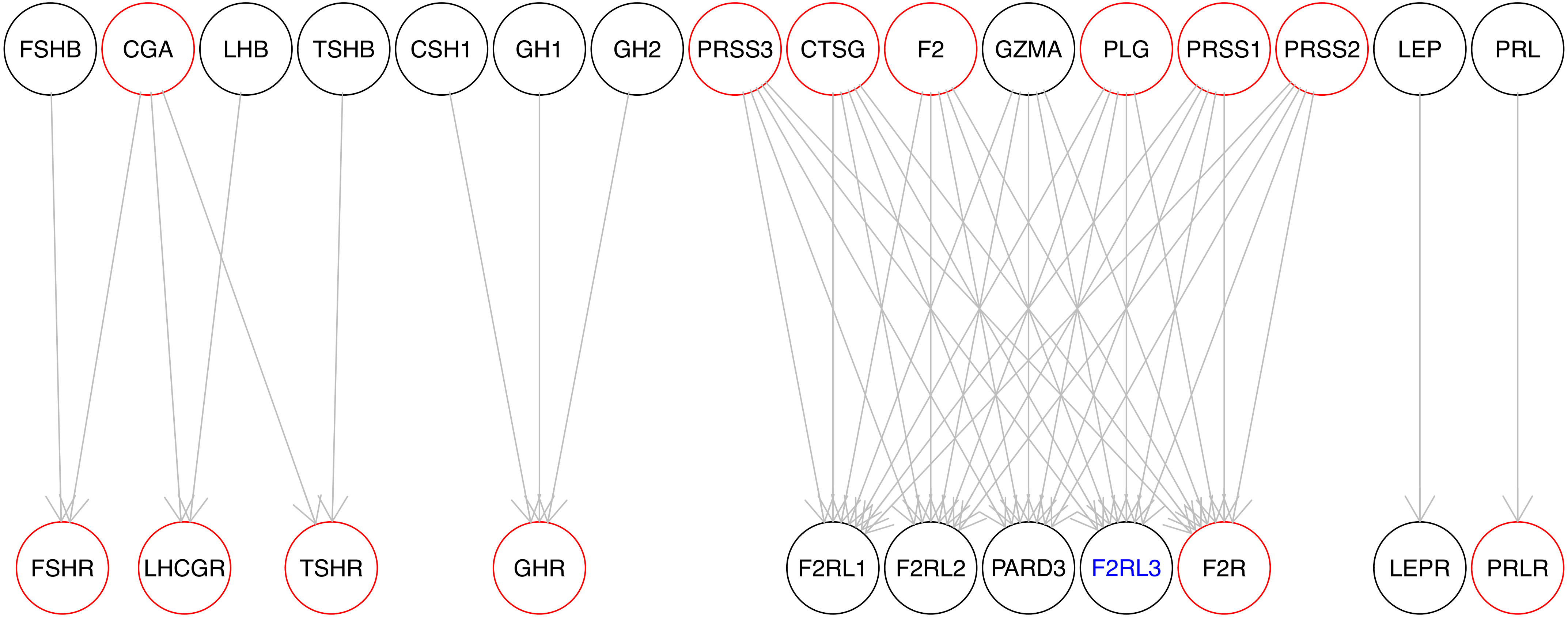

KEGG pathway = Neuroactive ligand−receptor interaction : tumour = YUHOOD : Yellow Fill = gene variant, Blue Text = expression−survival association, Red Boarder = drug

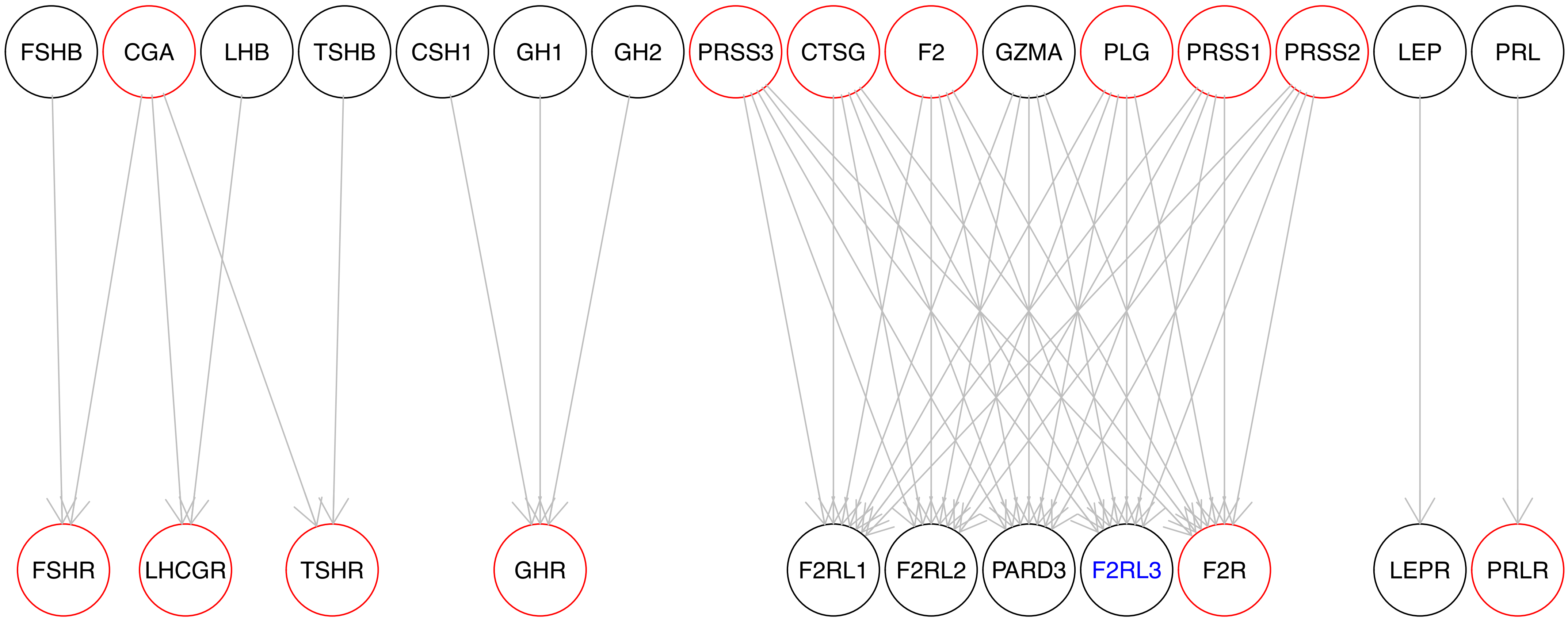

KEGG pathway = Neuroactive ligand−receptor interaction :   tumour = YUISKIA :   Yellow Fill = gene variant, Blue Text = expression−survival association, Red Boarder = drug

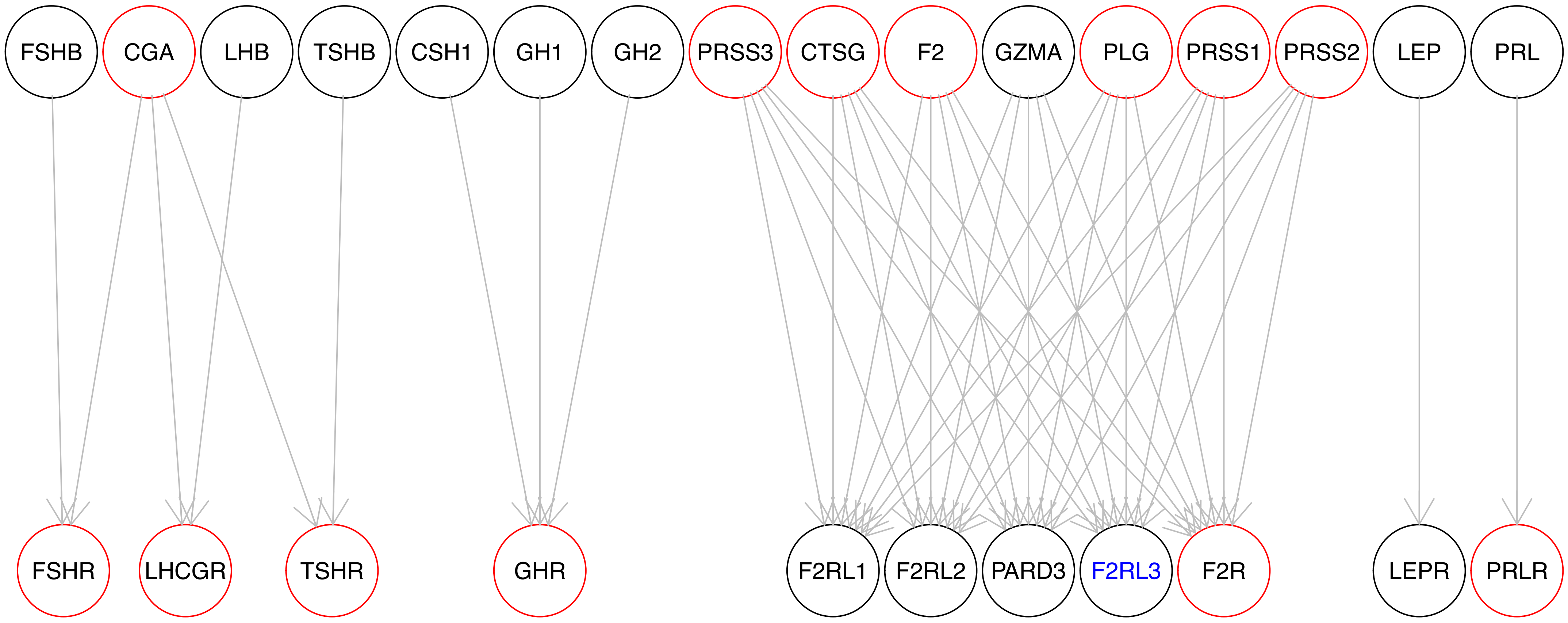

KEGG pathway = Neuroactive ligand−receptor interaction : tumour = YUKADI : Yellow Fill = gene variant, Blue Text = expression−survival association, Red Boarder = drug

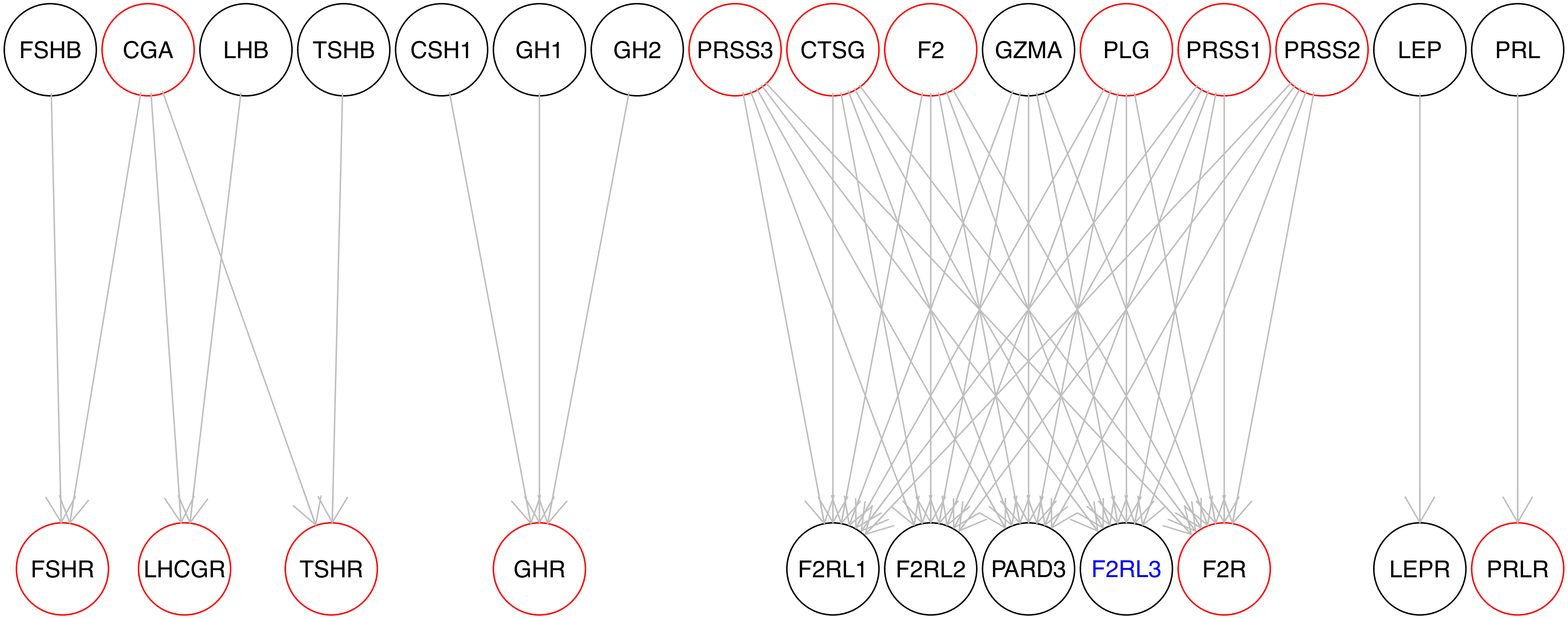

KEGG pathway = Neuroactive ligand−receptor interaction : tumour = YUKARN : Yellow Fill = gene variant, Blue Text = expression−survival association, Red Boarder = drug

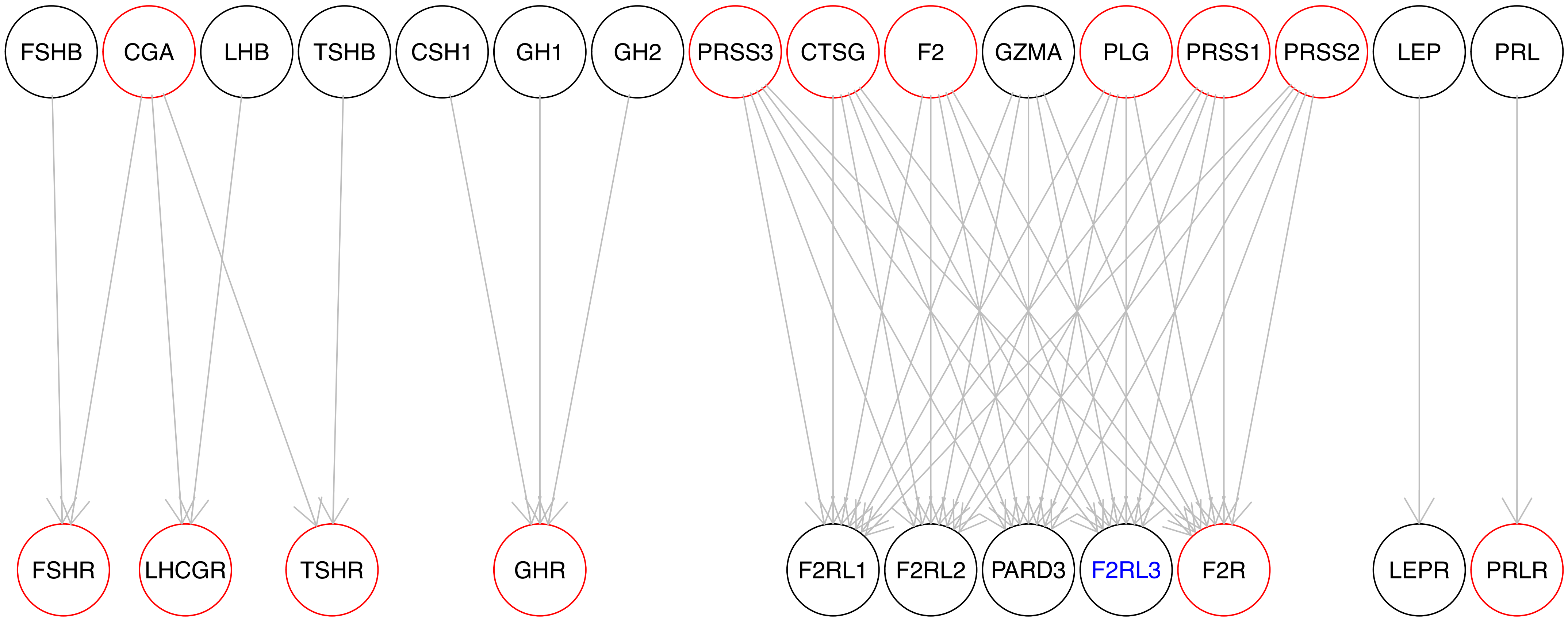

KEGG pathway = Neuroactive ligand−receptor interaction : tumour = YUKIL : Yellow Fill = gene variant, Blue Text = expression−survival association, Red Boarder = drug

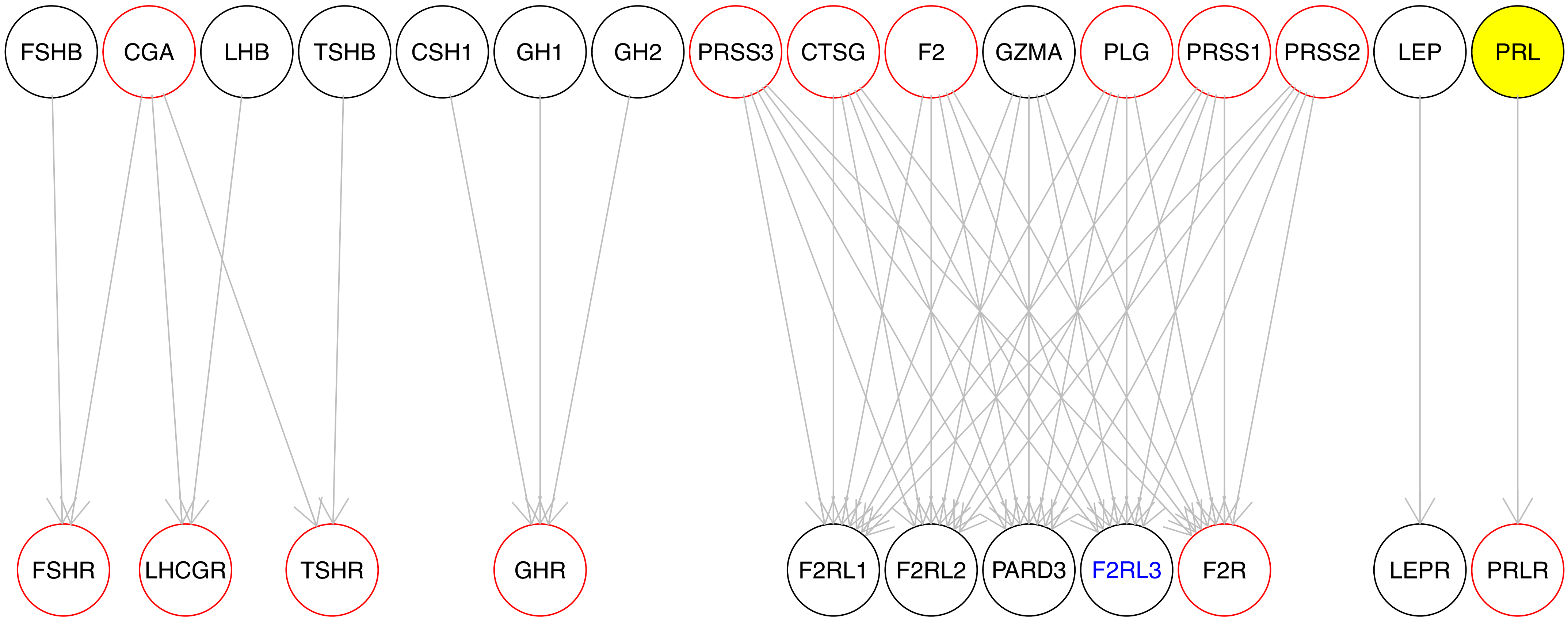

KEGG pathway = Neuroactive ligand−receptor interaction : tumour = YUKLAB : Yellow Fill = gene variant, Blue Text = expression−survival association, Red Boarder = drug

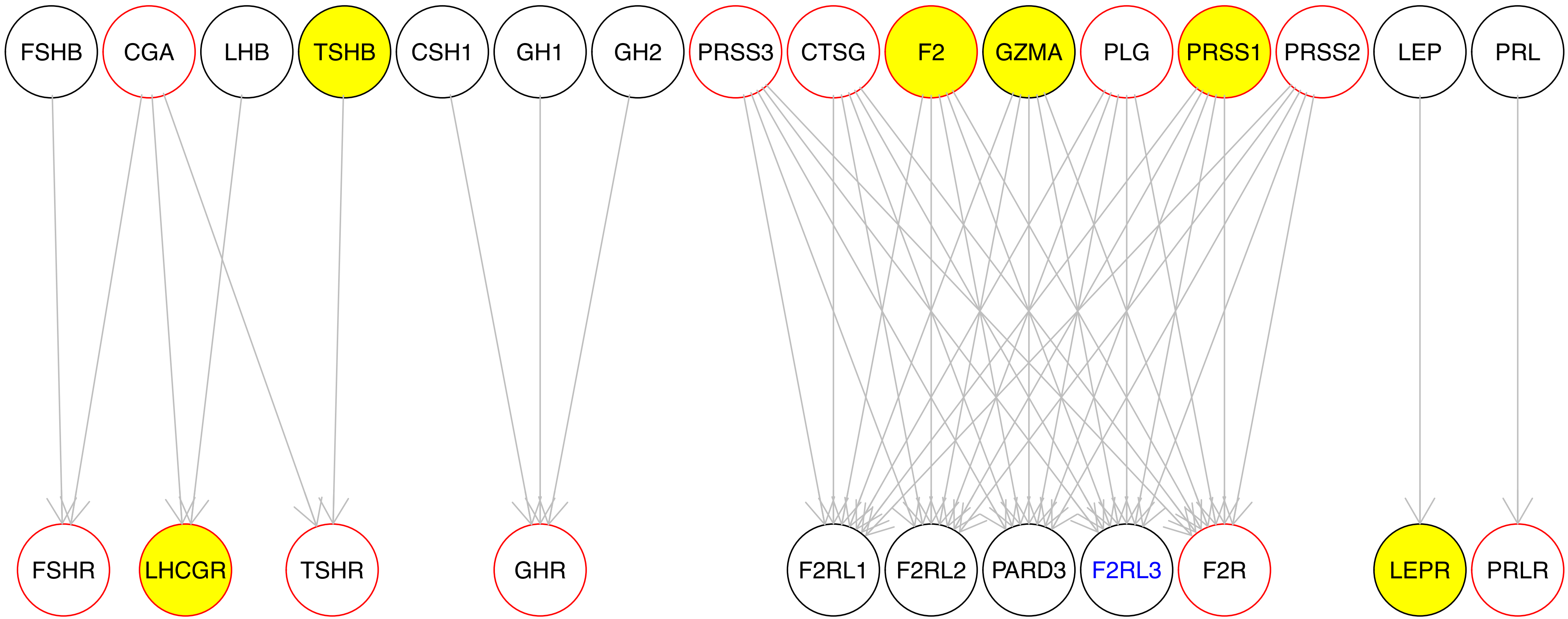

KEGG pathway = Neuroactive ligand−receptor interaction : tumour = YUKRIN : Yellow Fill = gene variant, Blue Text = expression−survival association, Red Boarder = drug

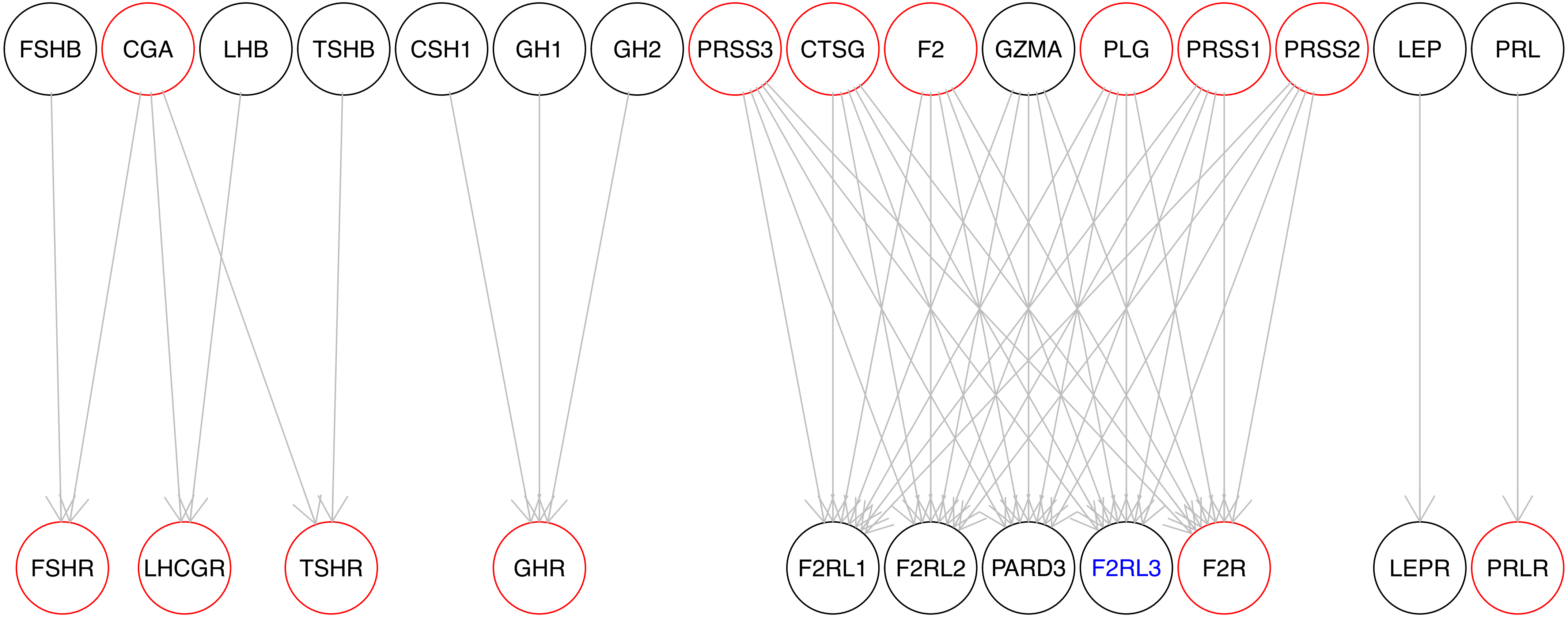

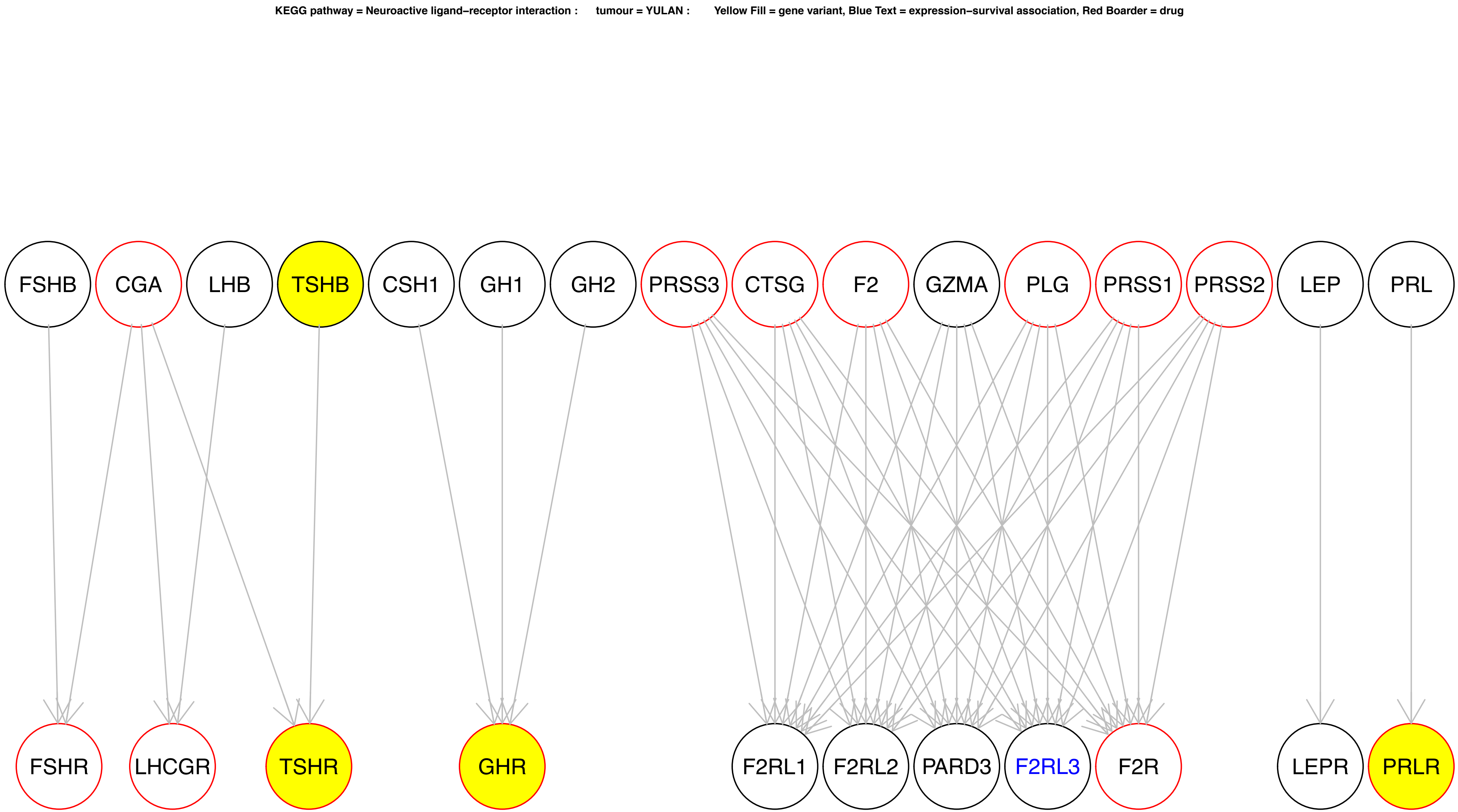
KEGG pathway = Neuroactive ligand−receptor interaction : tumour = YULAN : Yellow Fill = gene variant, Blue Text = expression−survival association, Red Boarder = drug
FSHB
CGA
LHB
TSHB
CSH1
GH1
GH2
PRSS3
CTSG
F2
GZMA
PLG
PRSS1
PRSS2
LEP
PRL
FSHR
LHCGR
TSHR
GHR
F2RL1
F2RL2
PARD3
F2RL3
F2R
LEPR
PRLR

KEGG pathway = Neuroactive ligand−receptor interaction :   tumour = YULAPE :   Yellow Fill = gene variant, Blue Text = expression−survival association, Red Boarder = drug

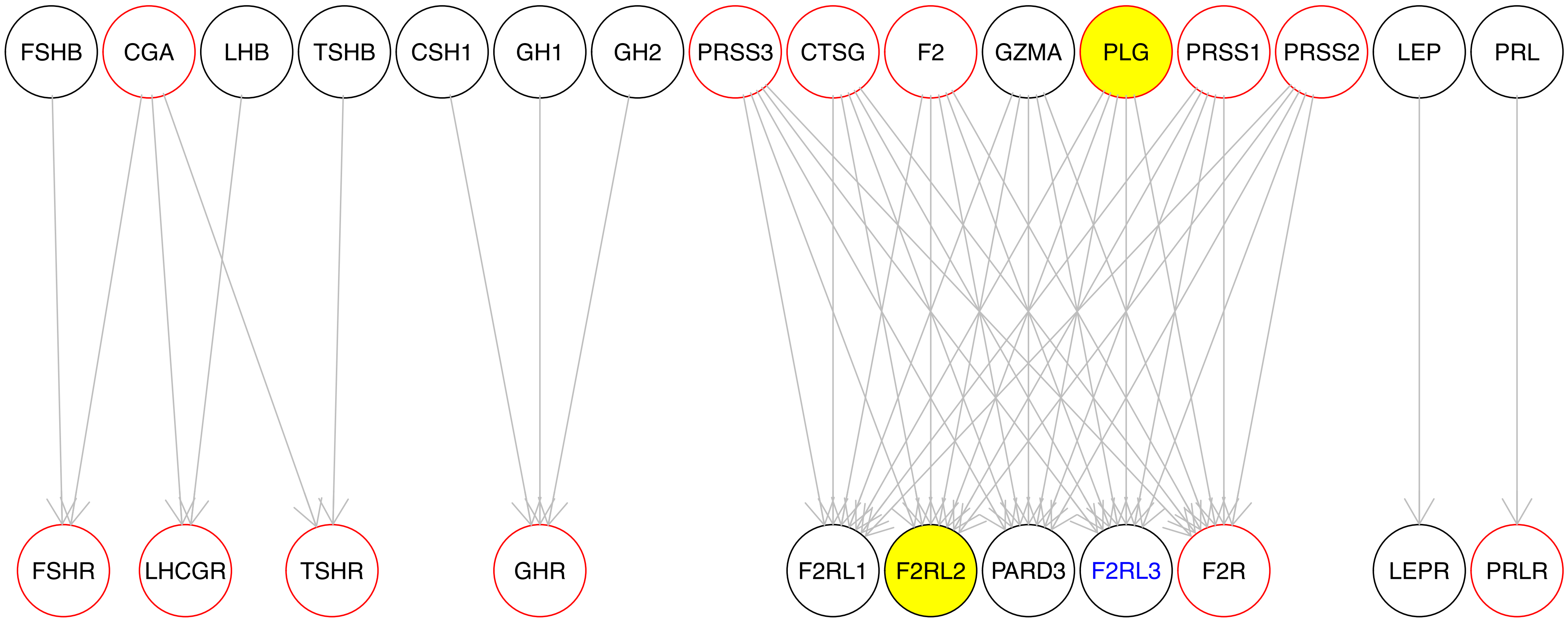

KEGG pathway = Neuroactive ligand−receptor interaction : tumour = YULAXER : Yellow Fill = gene variant, Blue Text = expression−survival association, Red Boarder = drug

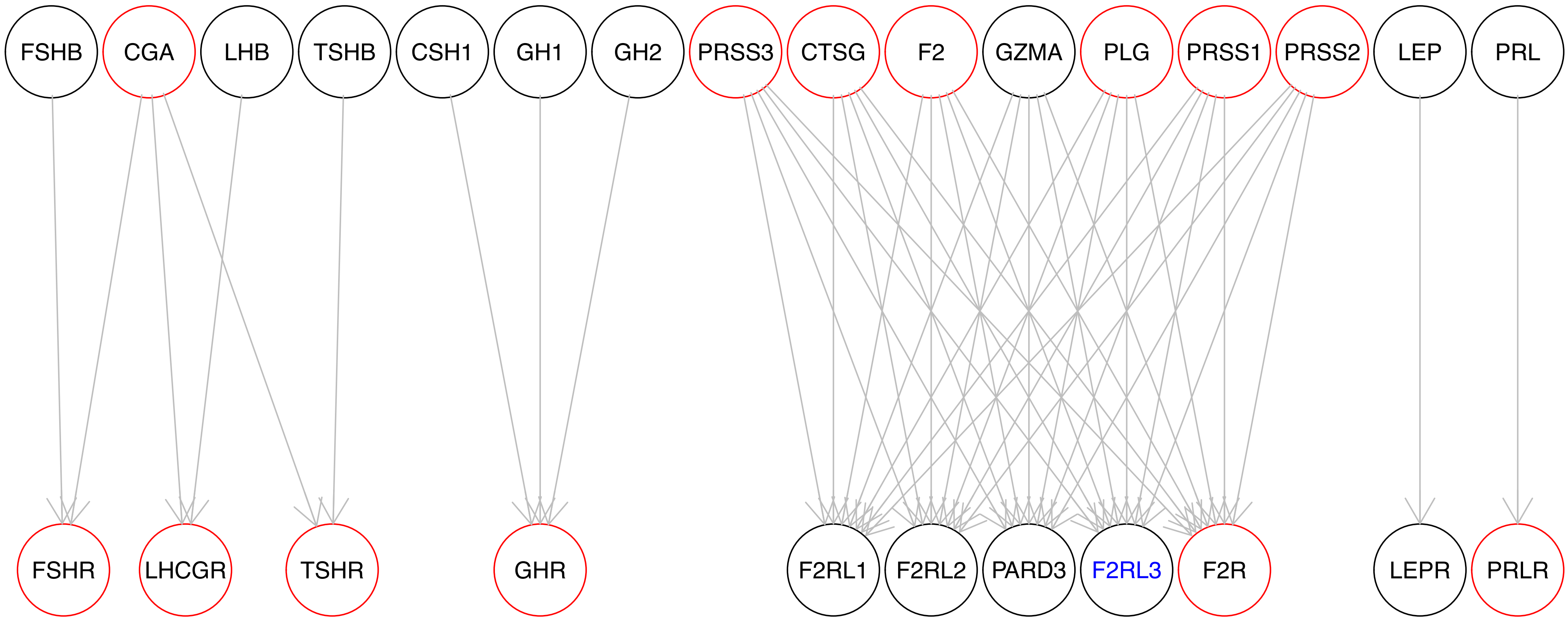

KEGG pathway = Neuroactive ligand–receptor interaction : tumour = YULLON : Yellow Fill = gene variant, Blue Text = expression–survival association, Red Boarder = drug

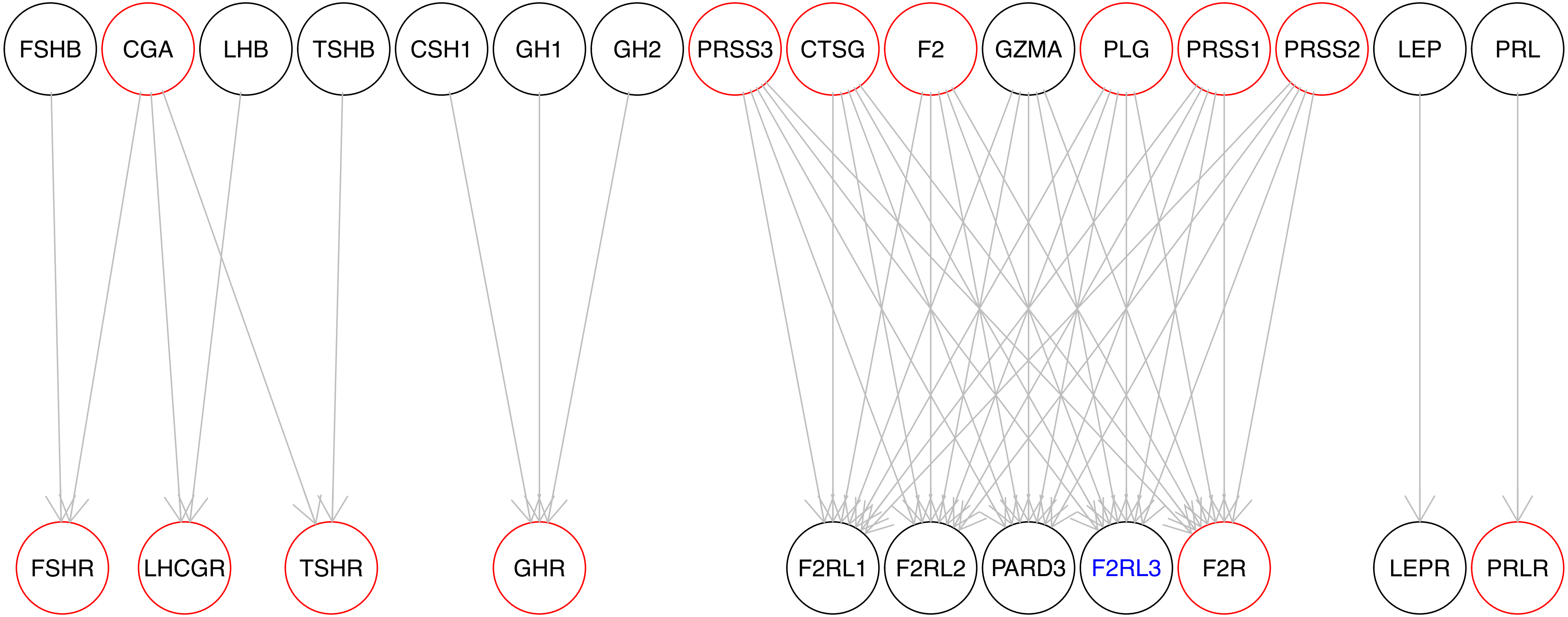

KEGG pathway = Neuroactive ligand−receptor interaction :   tumour = YULOMA :   Yellow Fill = gene variant, Blue Text = expression−survival association, Red Boarder = drug

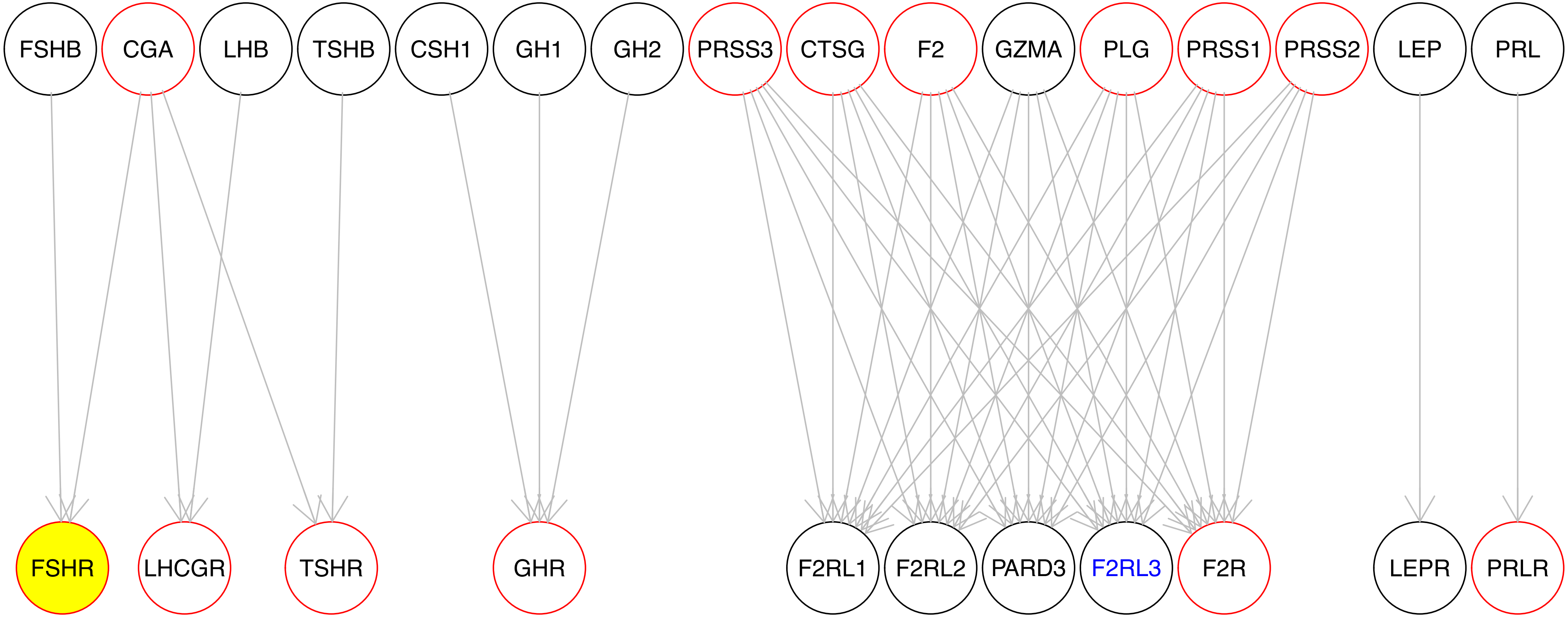

KEGG pathway = Neuroactive ligand−receptor interaction :   tumour = YULOVY :   Yellow Fill = gene variant, Blue Text = expression−survival association, Red Boarder = drug

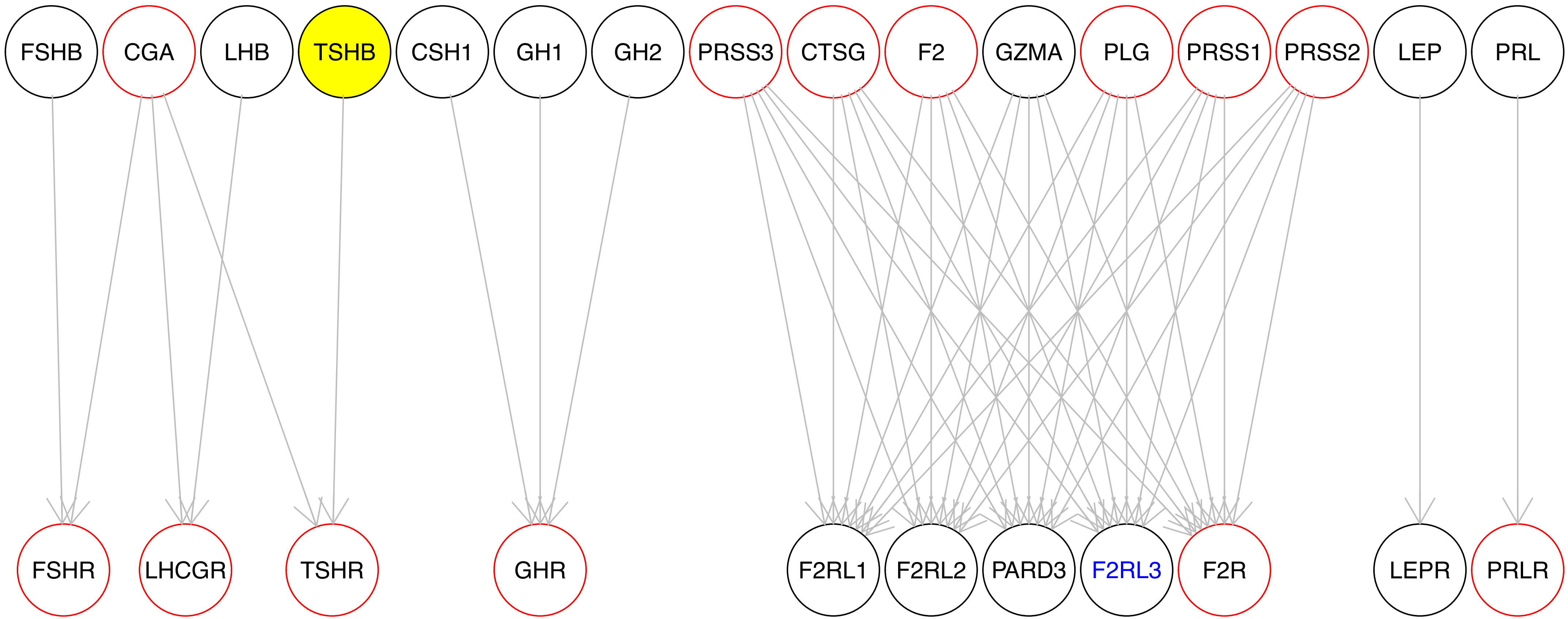

KEGG pathway = Neuroactive ligand−receptor interaction :   tumour = YUMAN :   Yellow Fill = gene variant, Blue Text = expression−survival association, Red Boarder = drug

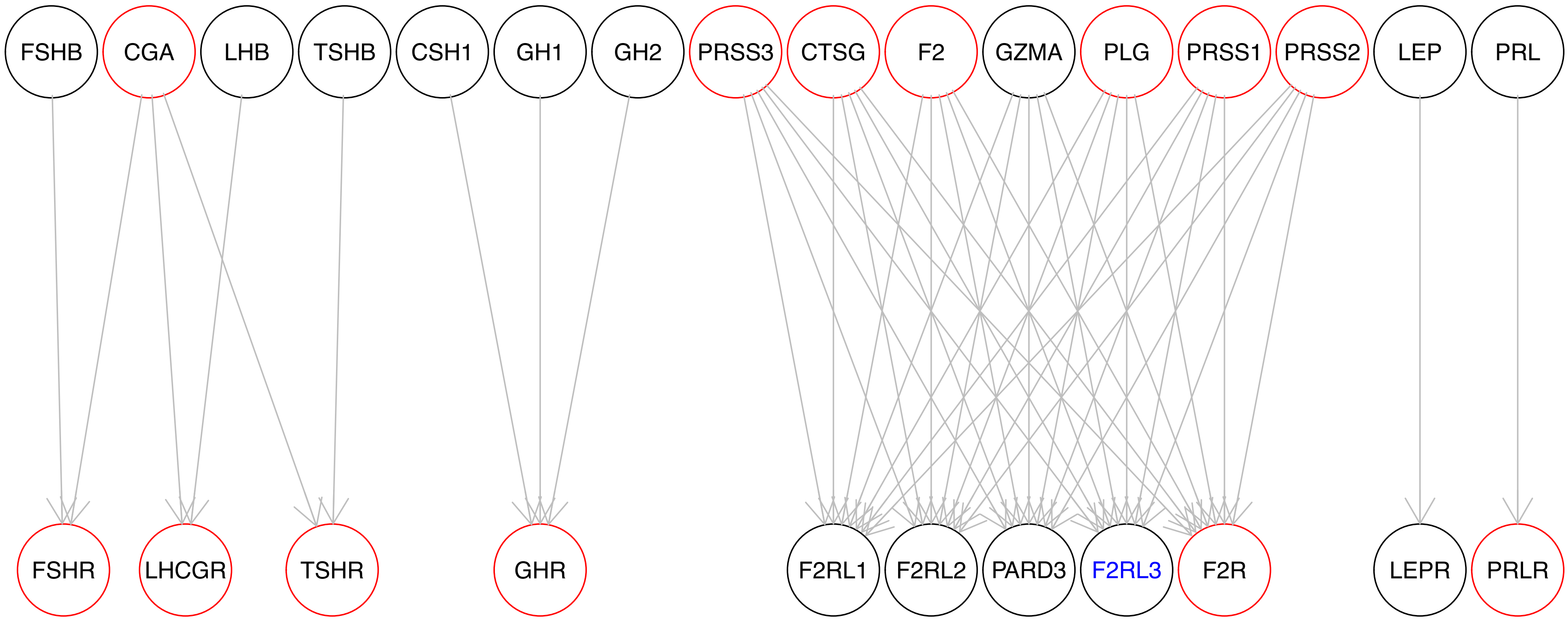

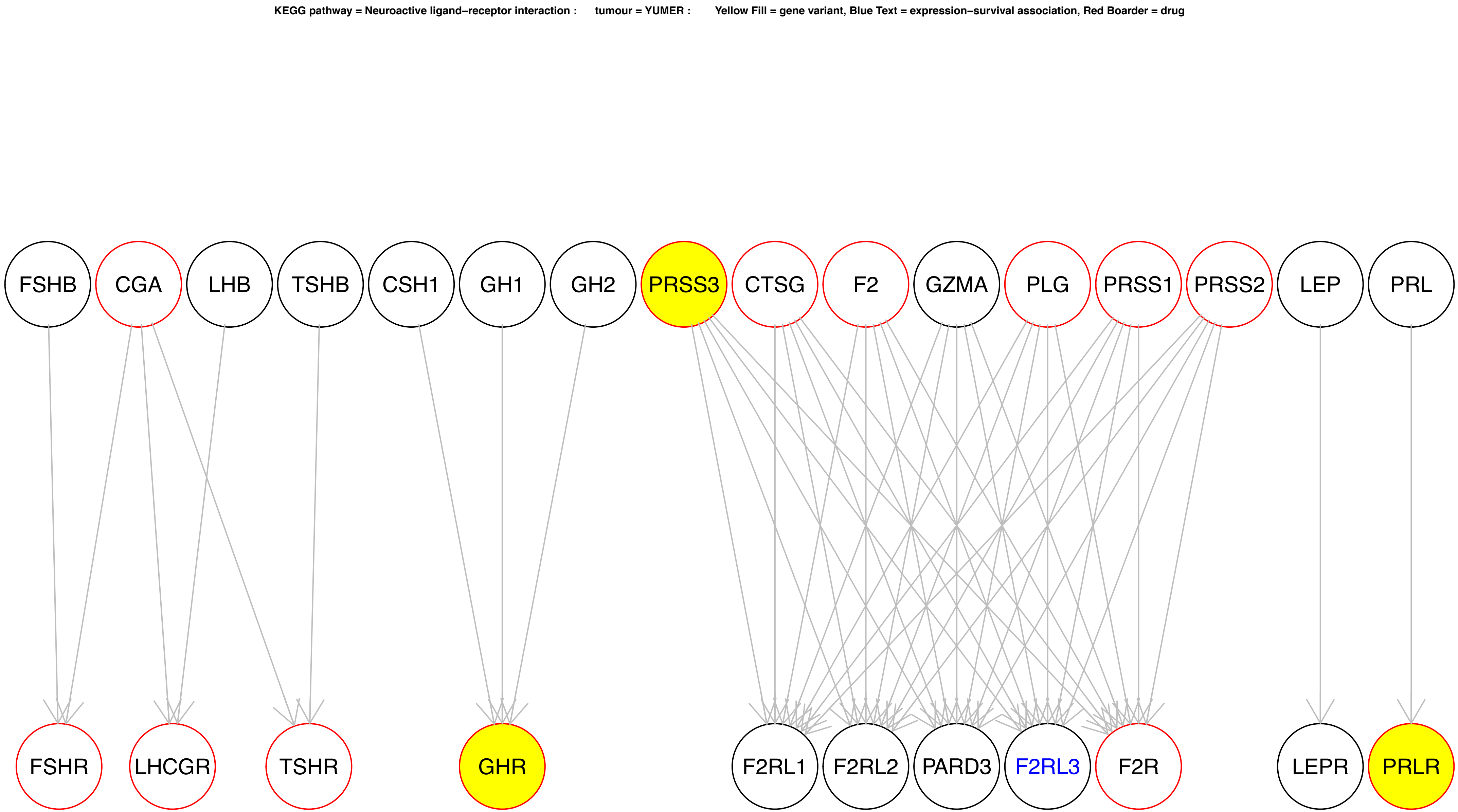
KEGG pathway = Neuroactive ligand−receptor interaction :   tumour = YUMER :   Yellow Fill = gene variant, Blue Text = expression−survival association, Red Boarder = drug
FSHB
CGA
LHB
TSHB
CSH1
GH1
GH2
PRSS3
CTSG
F2
GZMA
PLG
PRSS1
PRSS2
LEP
PRL
FSHR
LHCGR
TSHR
GHR
F2RL1
F2RL2
PARD3
F2RL3
F2R
LEPR
PRLR

KEGG pathway = Neuroactive ligand−receptor interaction : tumour = YUMOOK : Yellow Fill = gene variant, Blue Text = expression−survival association, Red Boarder = drug

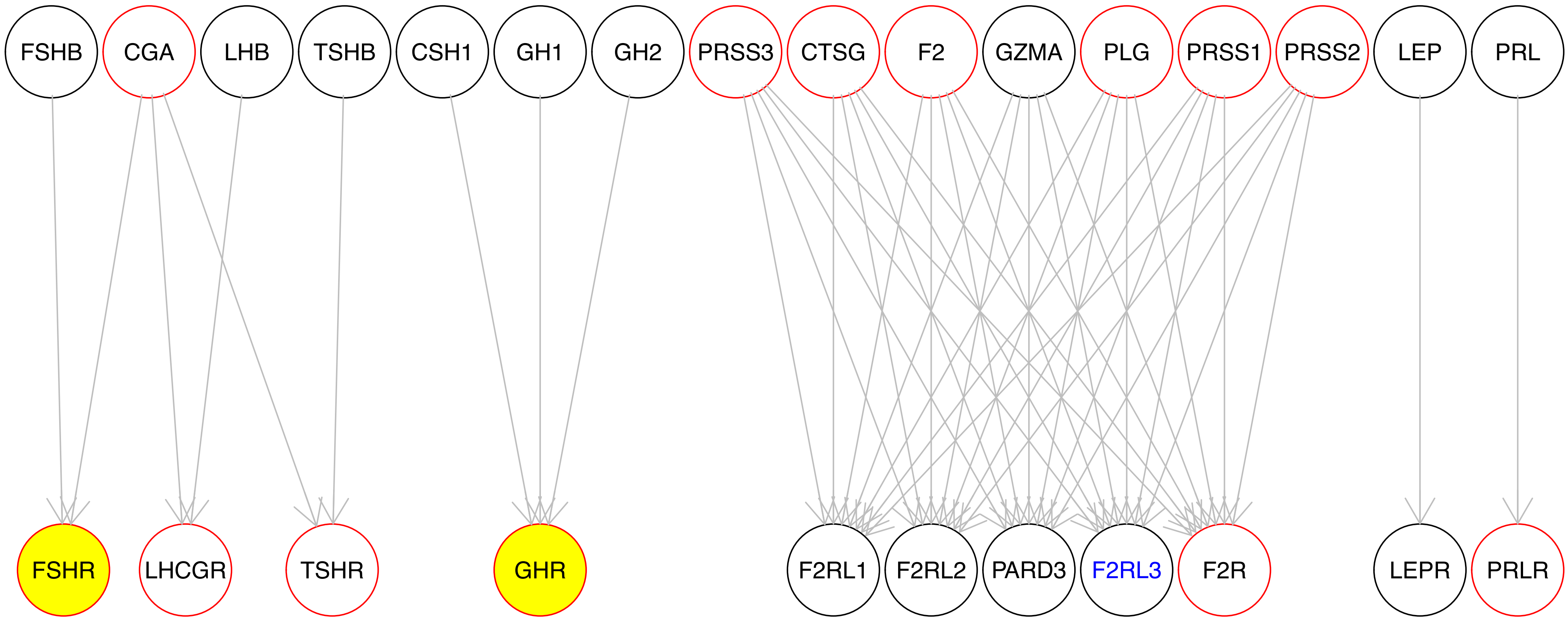

KEGG pathway = Neuroactive ligand−receptor interaction : tumour = YUMOYA : Yellow Fill = gene variant, Blue Text = expression−survival association, Red Boarder = drug

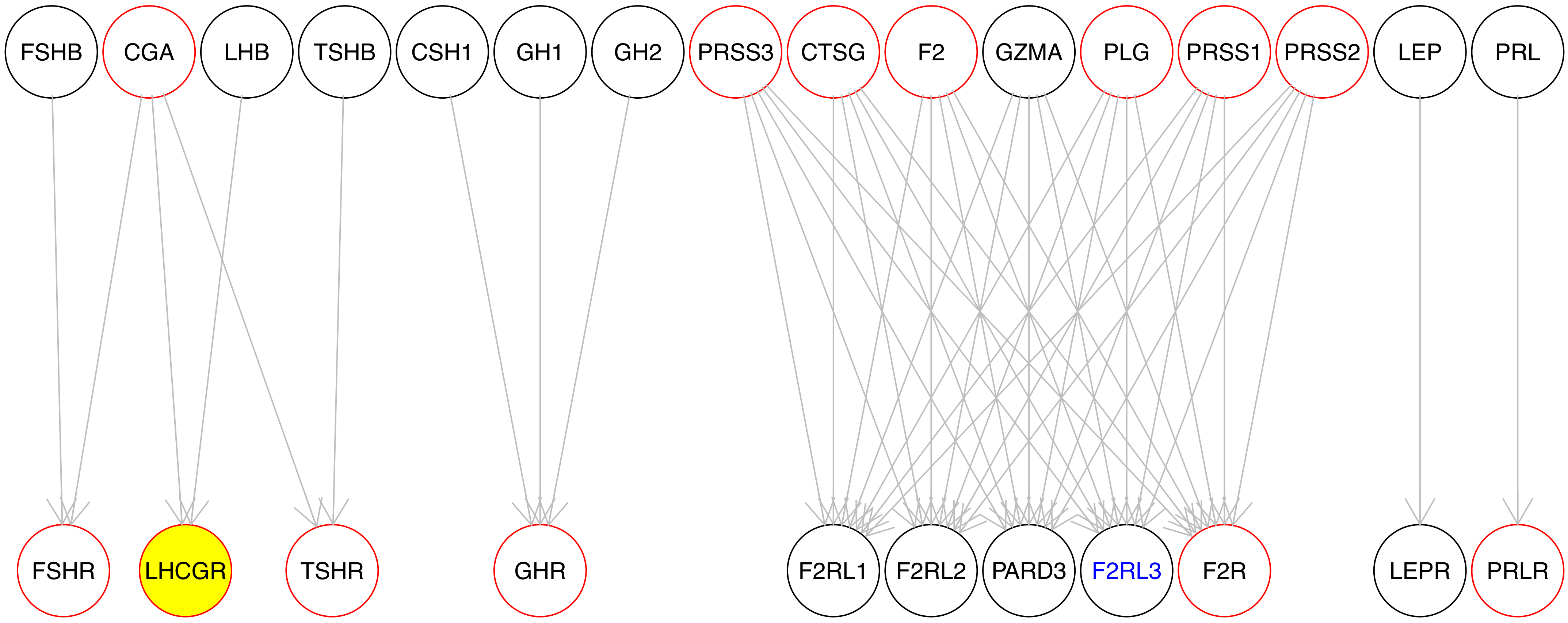

KEGG pathway = Neuroactive ligand−receptor interaction :   tumour = YUMUT :   Yellow Fill = gene variant, Blue Text = expression−survival association, Red Boarder = drug

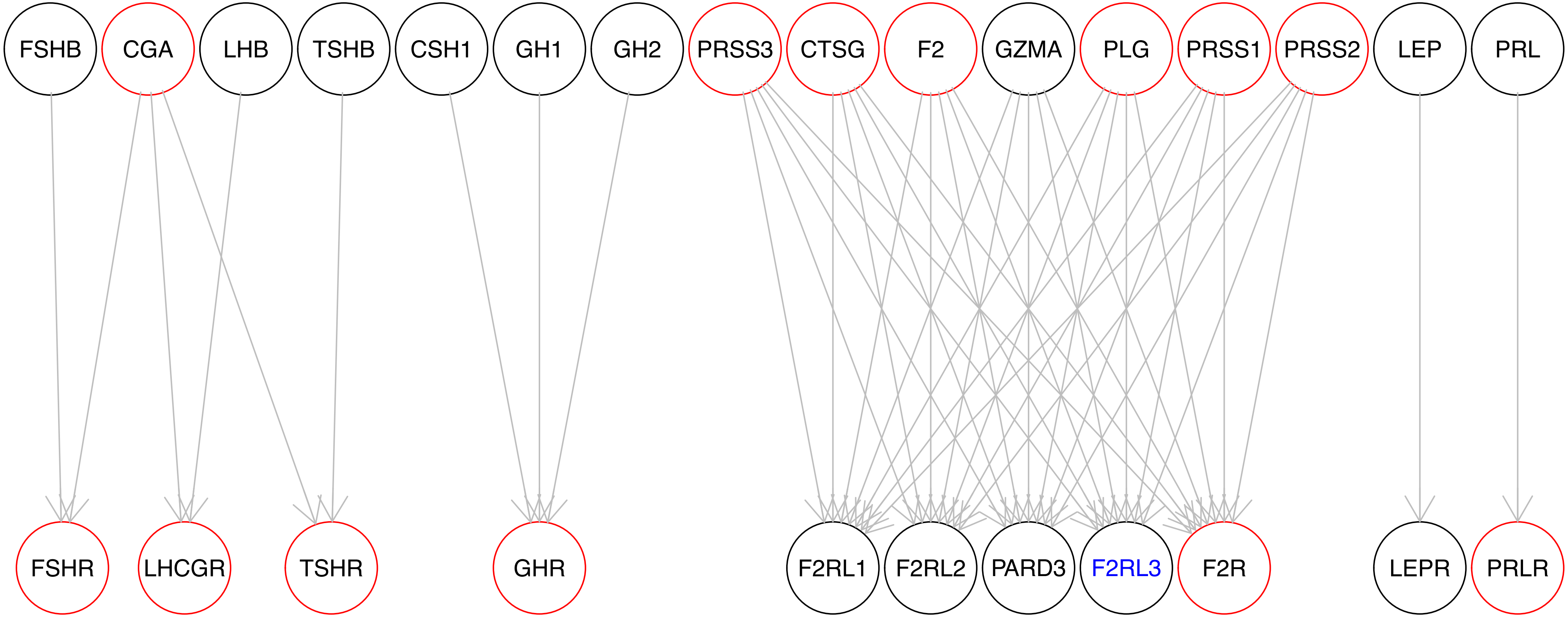

KEGG pathway = Neuroactive ligand−receptor interaction : tumour = YUNACK : Yellow Fill = gene variant, Blue Text = expression−survival association, Red Boarder = drug

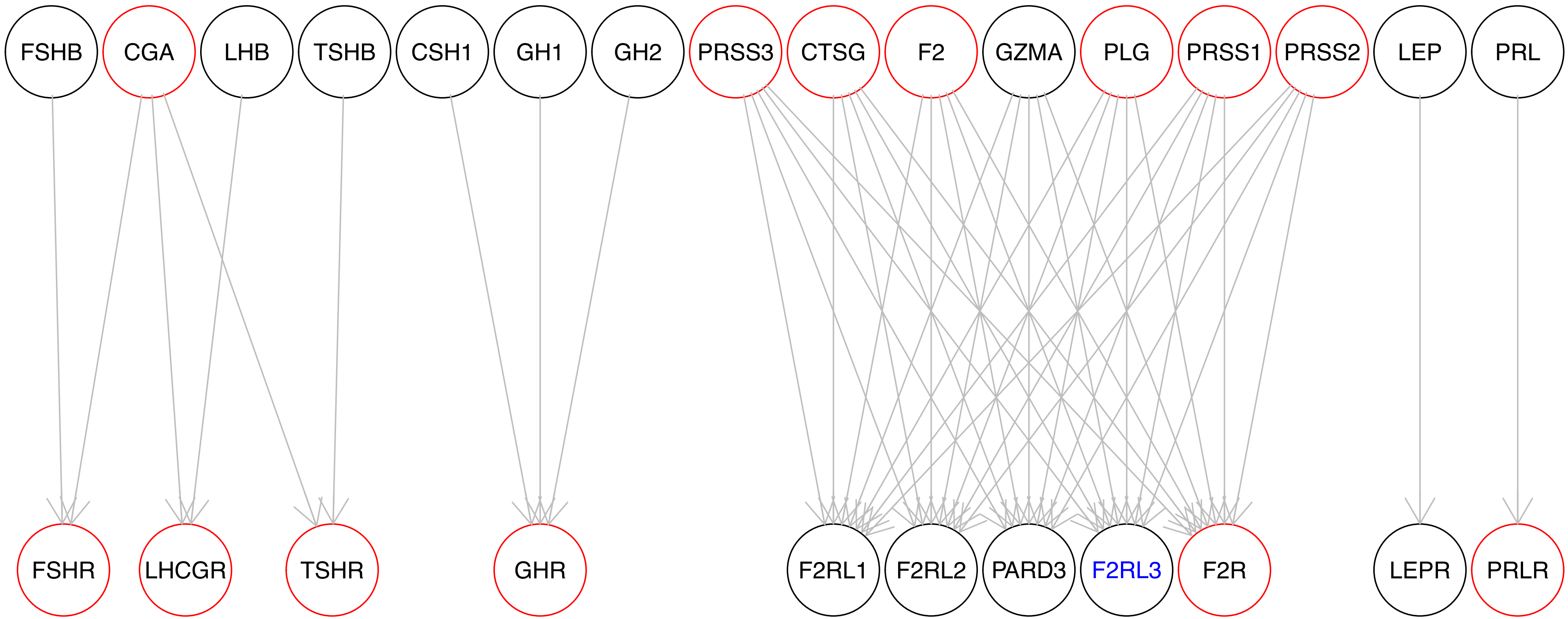

KEGG pathway = Neuroactive ligand−receptor interaction :   tumour = YUNEON :   Yellow Fill = gene variant, Blue Text = expression−survival association, Red Boarder = drug

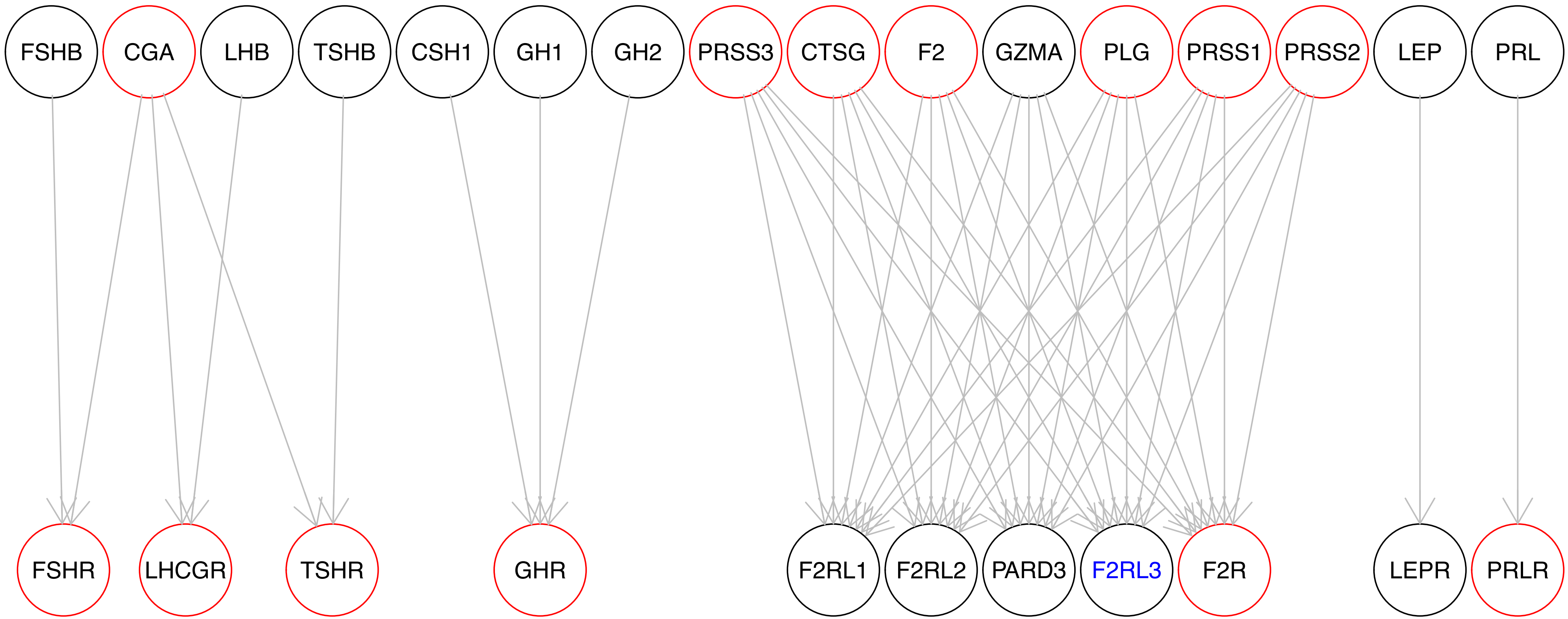

KEGG pathway = Neuroactive ligand−receptor interaction :   tumour = YUNOCA :   Yellow Fill = gene variant, Blue Text = expression−survival association, Red Boarder = drug

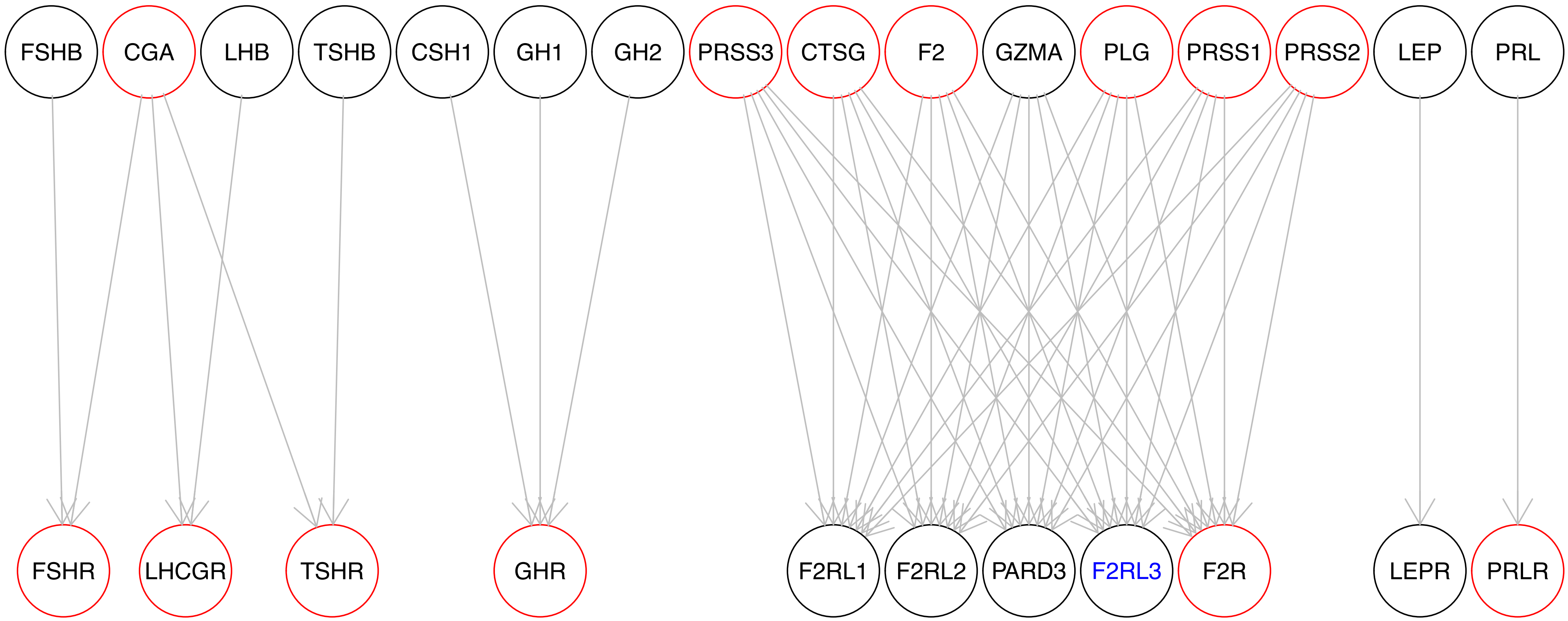

KEGG pathway = Neuroactive ligand−receptor interaction : tumour = YUNUFF : Yellow Fill = gene variant, Blue Text = expression−survival association, Red Boarder = drug

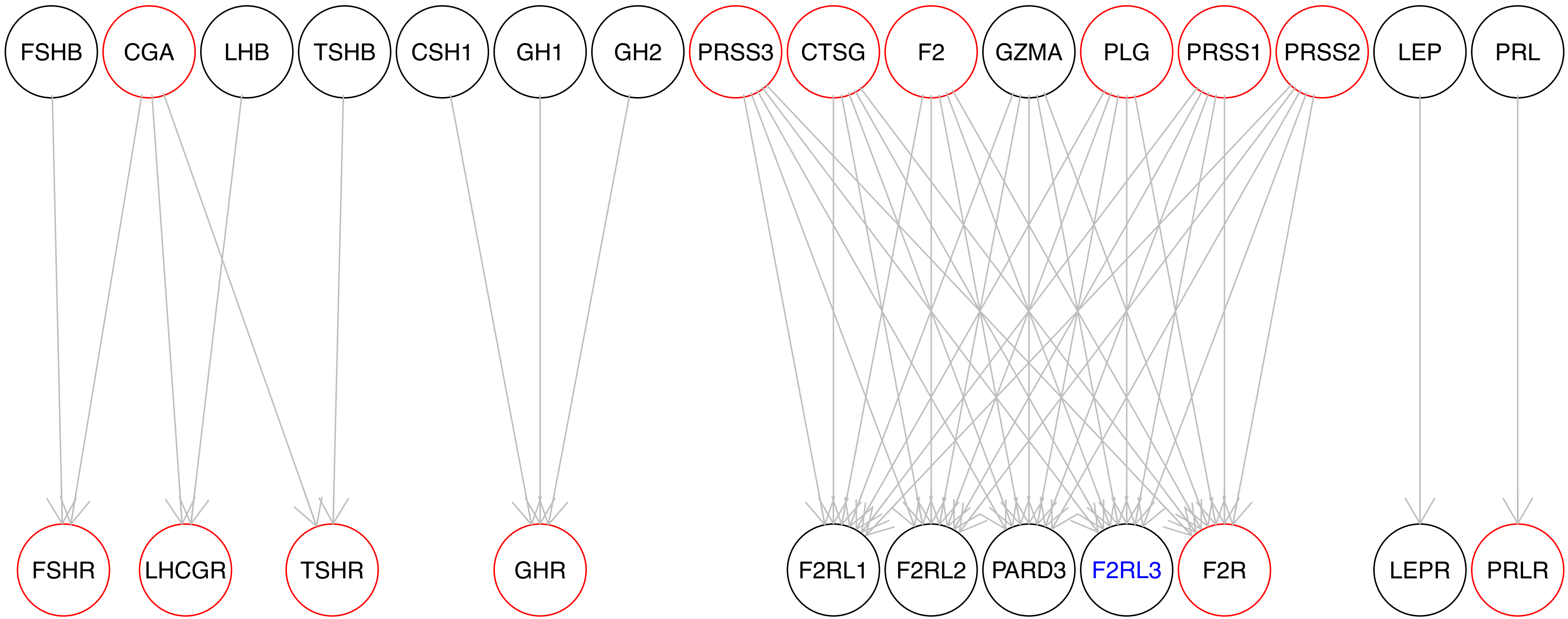

KEGG pathway = Neuroactive ligand−receptor interaction :   tumour = YUNUVO :   Yellow Fill = gene variant, Blue Text = expression−survival association, Red Boarder = drug

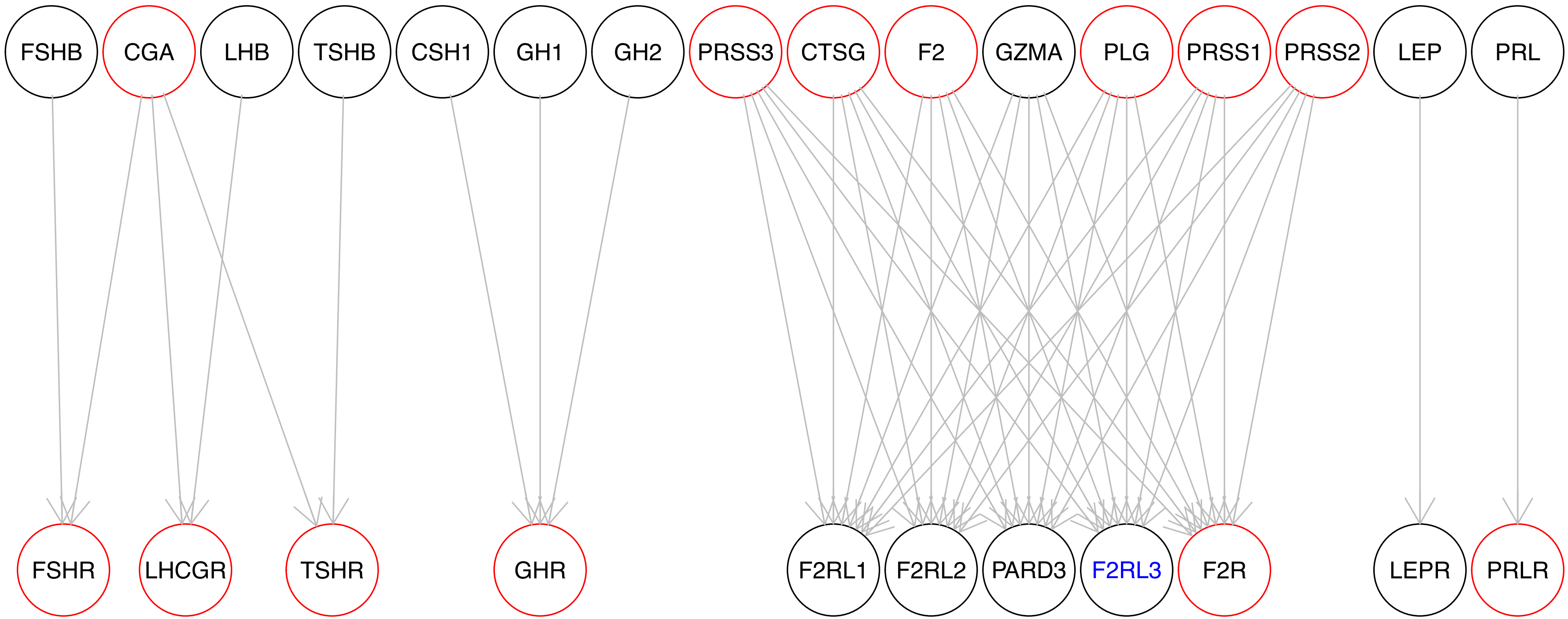

KEGG pathway = Neuroactive ligand−receptor interaction :   tumour = YUPADI :   Yellow Fill = gene variant, Blue Text = expression−survival association, Red Boarder = drug

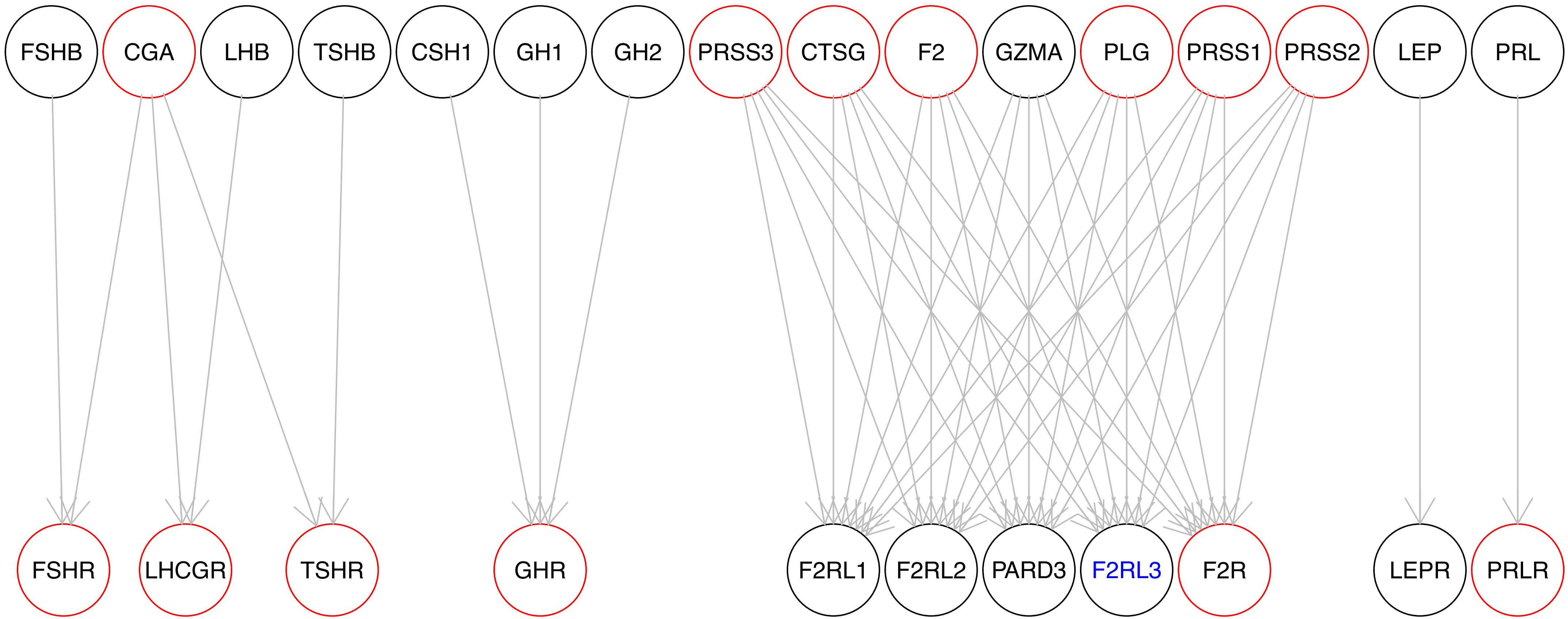

KEGG pathway = Neuroactive ligand−receptor interaction : tumour = YUPAF : Yellow Fill = gene variant, Blue Text = expression−survival association, Red Boarder = drug

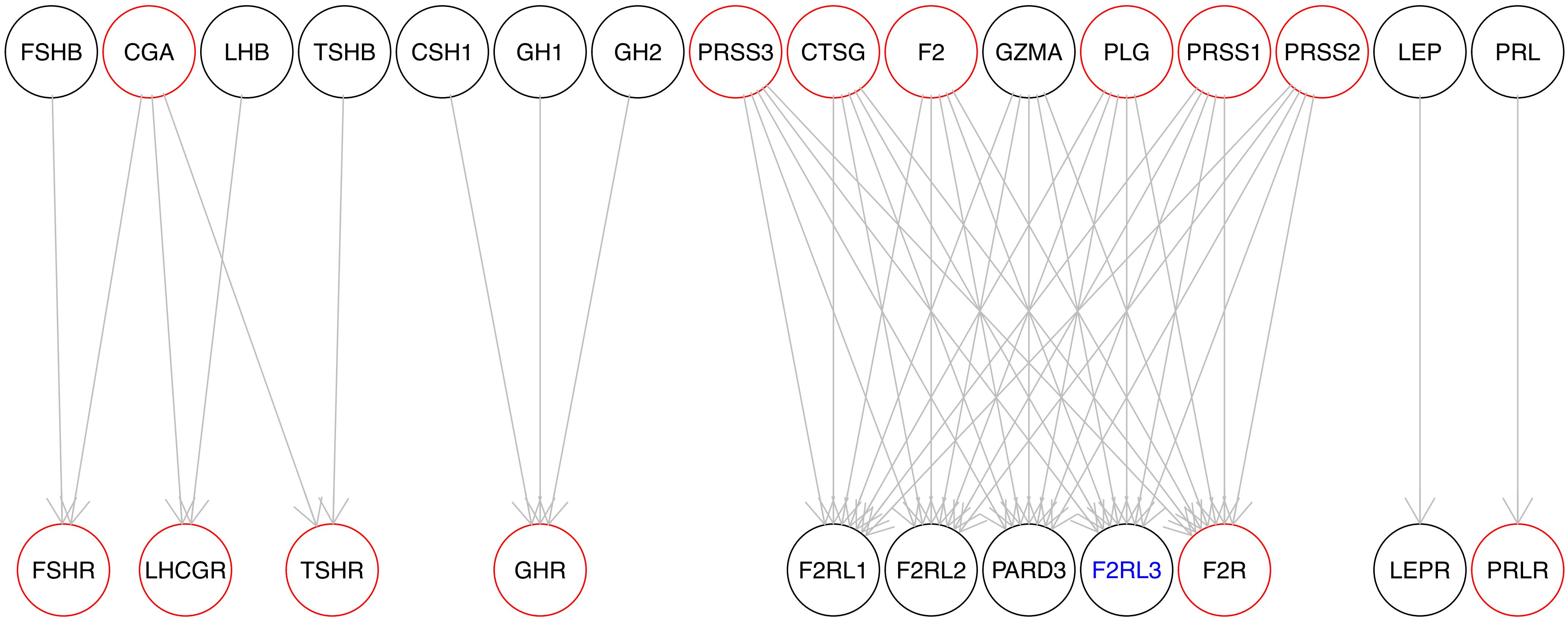

KEGG pathway = Neuroactive ligand−receptor interaction : tumour = YUPAL : Yellow Fill = gene variant, Blue Text = expression−survival association, Red Boarder = drug

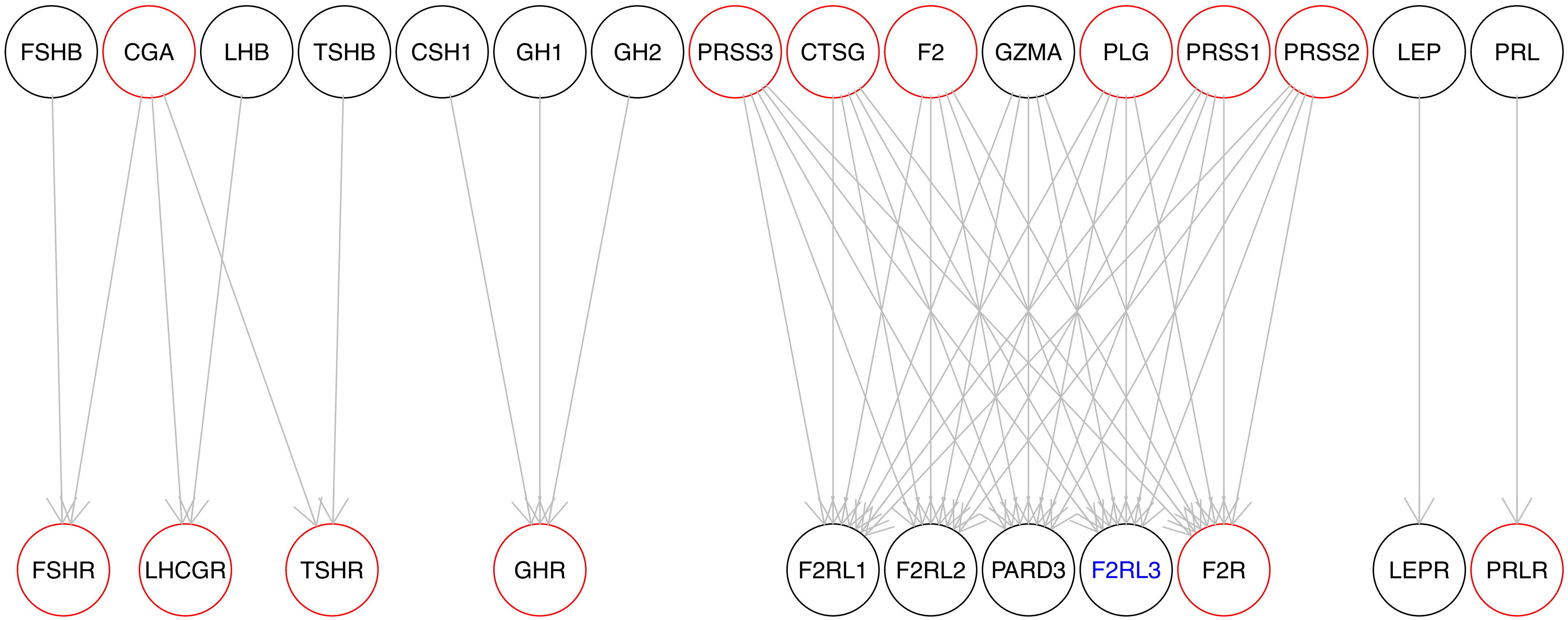

KEGG pathway = Neuroactive ligand−receptor interaction : tumour = YUPAT : Yellow Fill = gene variant, Blue Text = expression−survival association, Red Boarder = drug

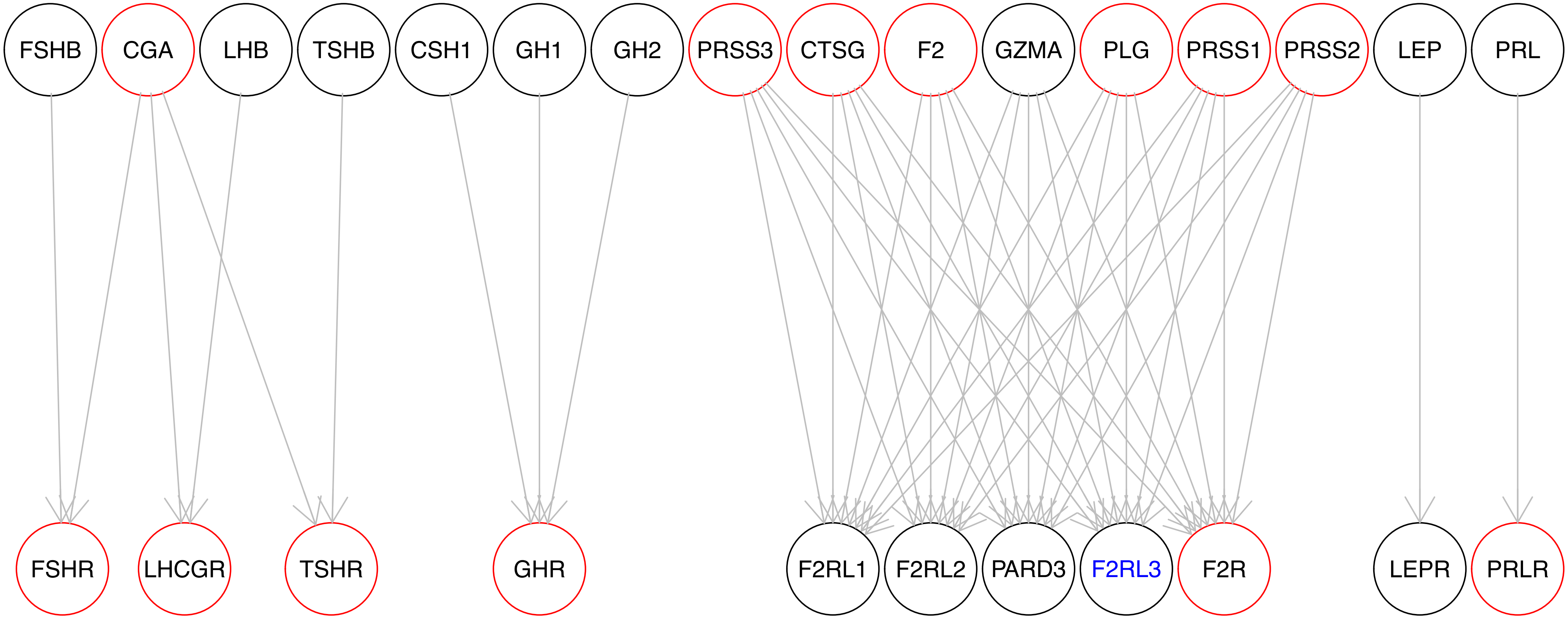

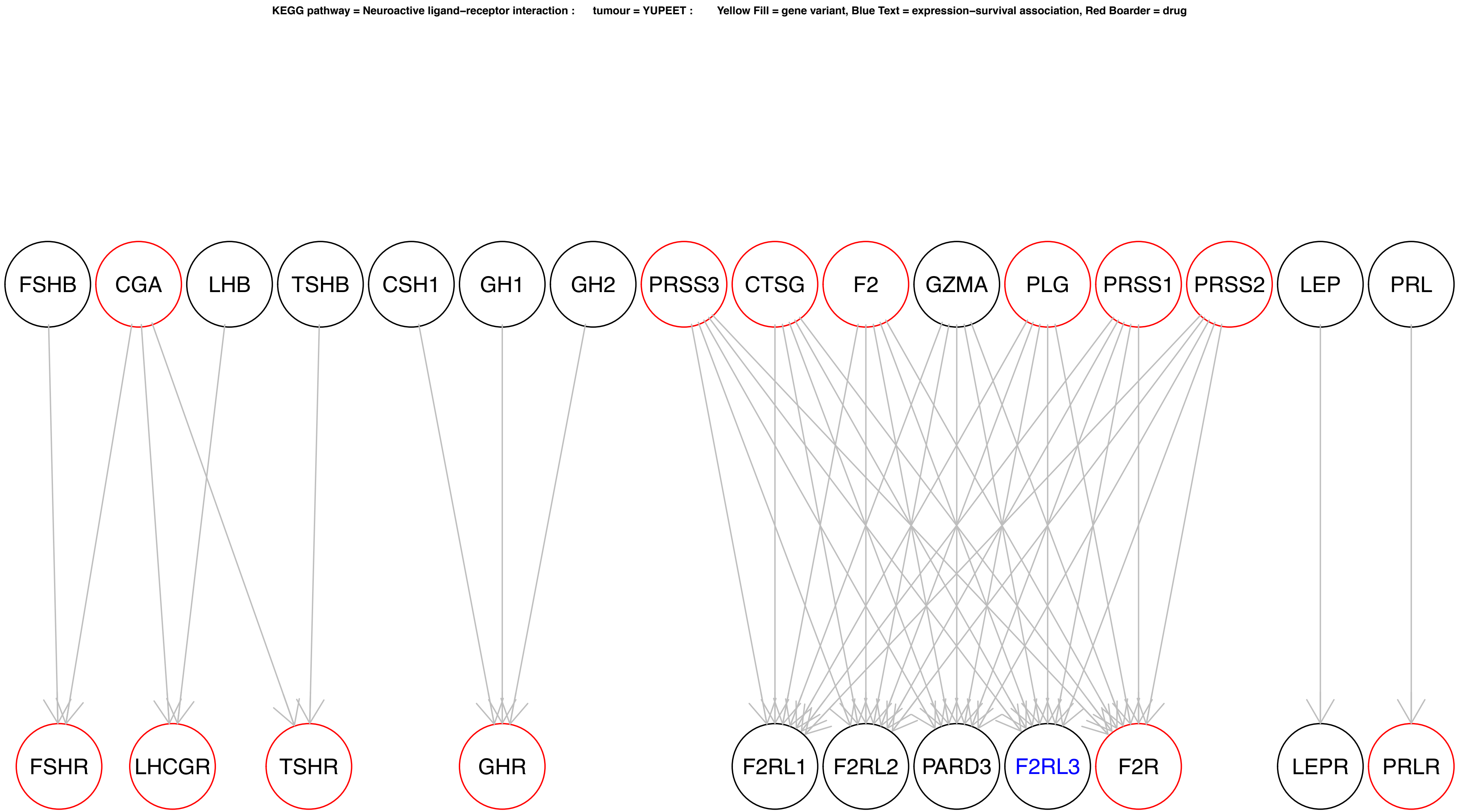
KEGG pathway = Neuroactive ligand−receptor interaction : tumour = YUPEET : Yellow Fill = gene variant, Blue Text = expression−survival association, Red Boarder = drug
FSHB
CGA
LHB
TSHB
CSH1
GH1
GH2
PRSS3
CTSG
F2
GZMA
PLG
PRSS1
PRSS2
LEP
PRL
FSHR
LHCGR
TSHR
GHR
F2RL1
F2RL2
PARD3
F2RL3
F2R
LEPR
PRLR

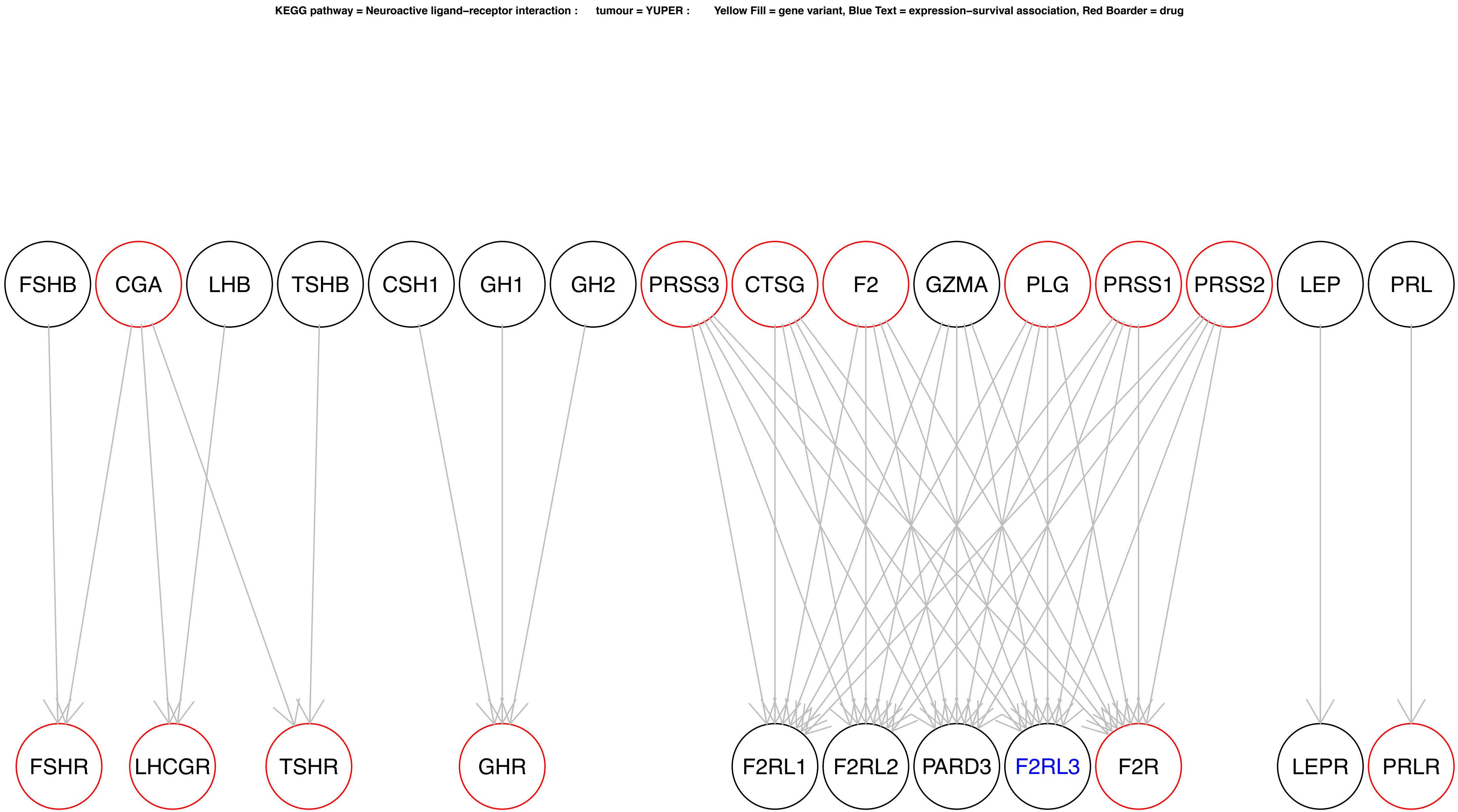

KEGG pathway = Neuroactive ligand−receptor interaction : tumour = YUPER : Yellow Fill = gene variant, Blue Text = expression−survival association, Red Boarder = drug
FSHB
CGA
LHB
TSHB
CSH1
GH1
GH2
PRSS3
CTSG
F2
GZMA
PLG
PRSS1
PRSS2
LEP
PRL
FSHR
LHCGR
TSHR
GHR
F2RL1
F2RL2
PARD3
F2RL3
F2R
LEPR
PRLR

KEGG pathway = Neuroactive ligand−receptor interaction : tumour = YUPORCH : Yellow Fill = gene variant, Blue Text = expression−survival association, Red Boarder = drug

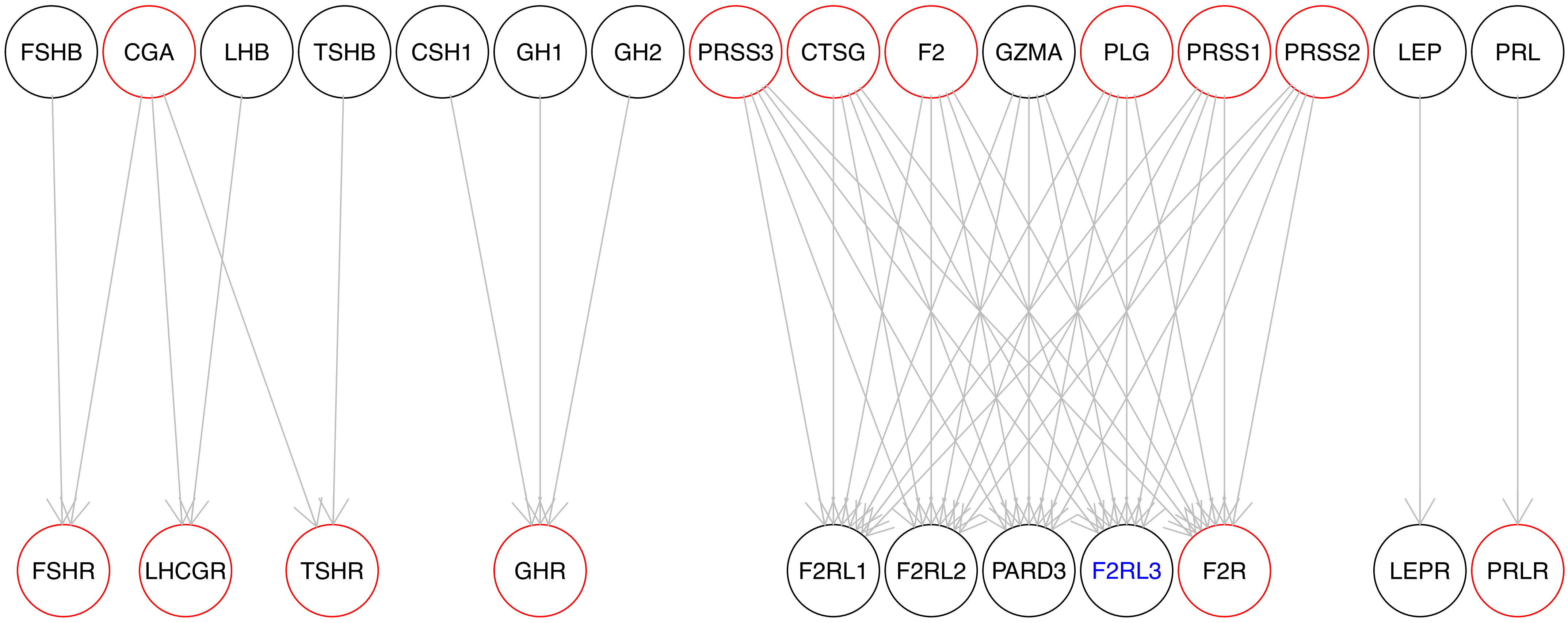

KEGG pathway = Neuroactive ligand−receptor interaction :   tumour = YUPRO :   Yellow Fill = gene variant, Blue Text = expression−survival association, Red Boarder = drug

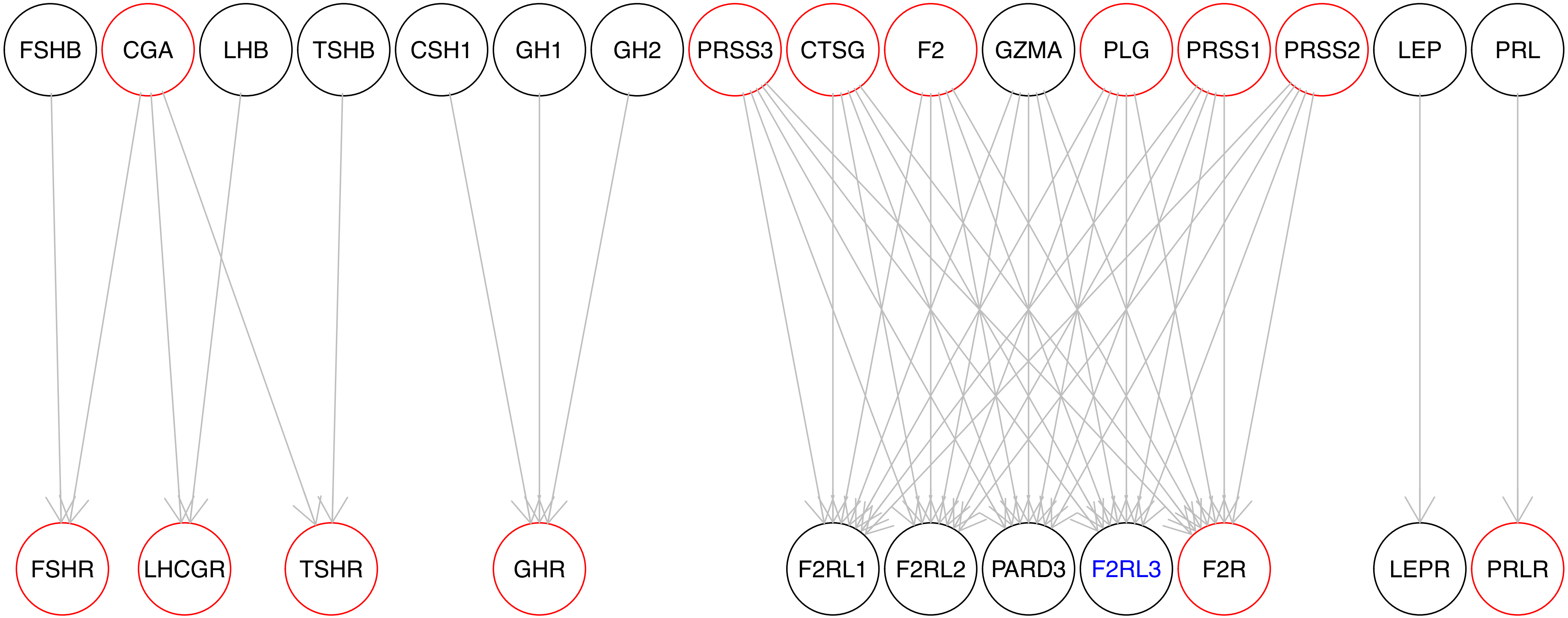

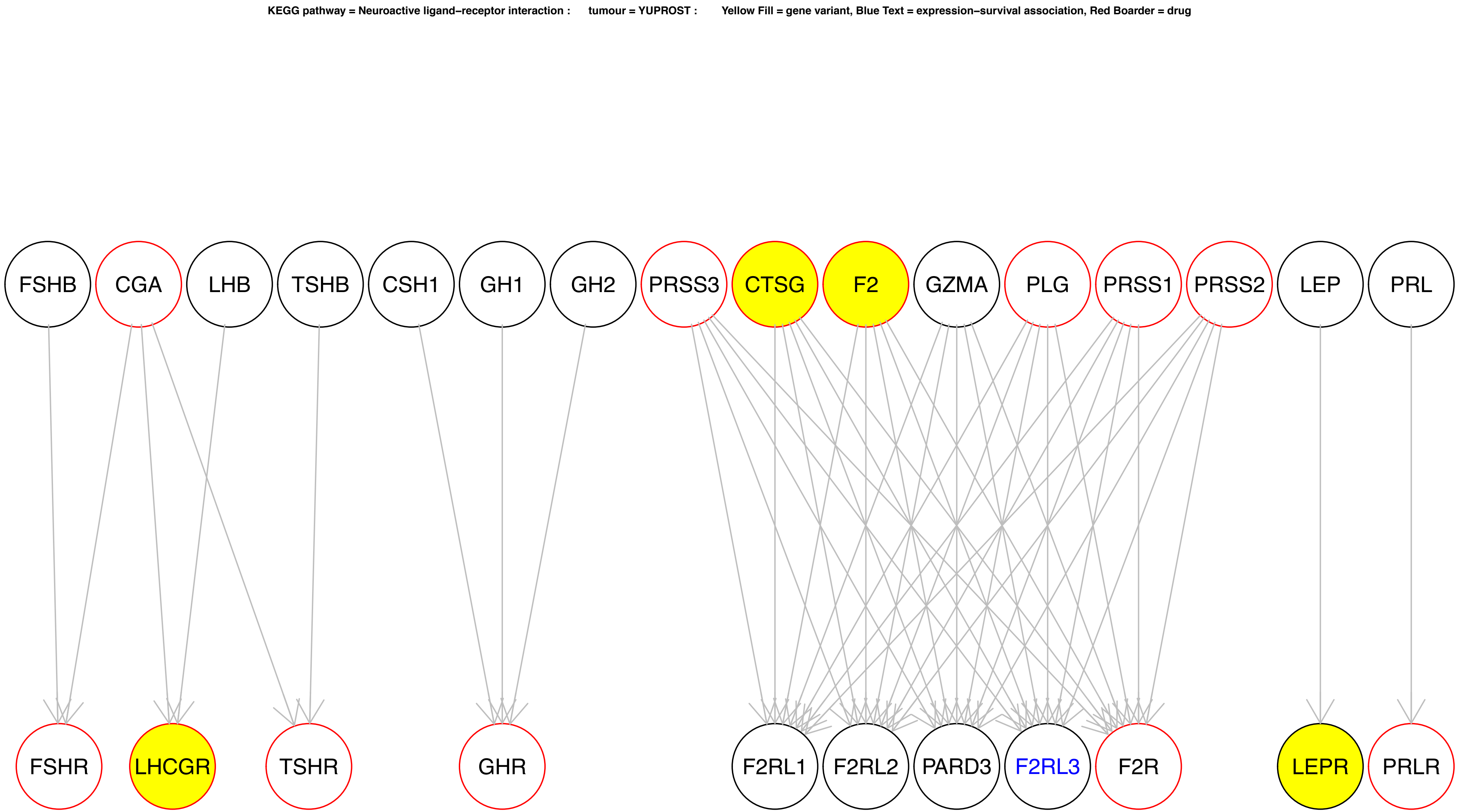
KEGG pathway = Neuroactive ligand−receptor interaction :
tumour = YUPROST :
Yellow Fill = gene variant, Blue Text = expression−survival association, Red Boarder = drug
FSHB
CGA
LHB
TSHB
CSH1
GH1
GH2
PRSS3
CTSG
F2
GZMA
PLG
PRSS1
PRSS2
LEP
PRL
FSHR
LHCGR
TSHR
GHR
F2RL1
F2RL2
PARD3
F2RL3
F2R
LEPR
PRLR

KEGG pathway = Neuroactive ligand−receptor interaction : tumour = YURDE : Yellow Fill = gene variant, Blue Text = expression−survival association, Red Boarder = drug

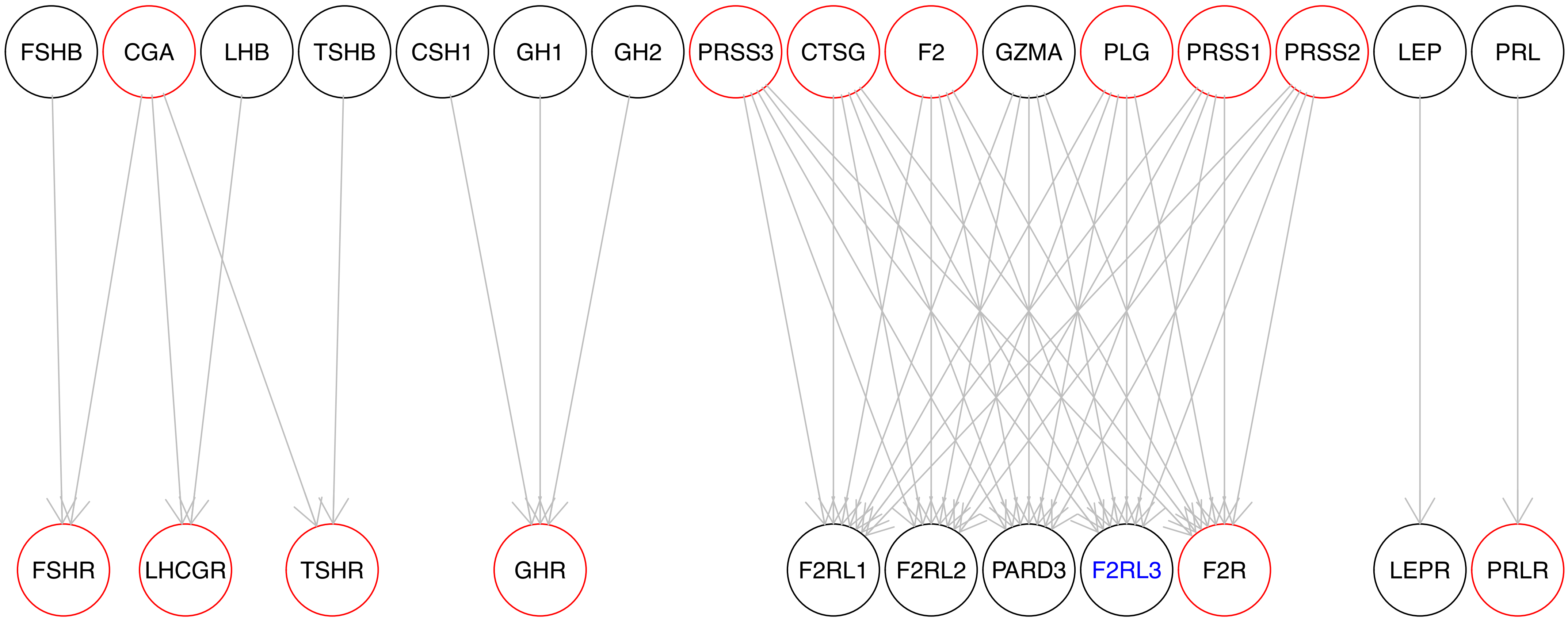

KEGG pathway = Neuroactive ligand−receptor interaction : tumour = YURED : Yellow Fill = gene variant, Blue Text = expression−survival association, Red Boarder = drug

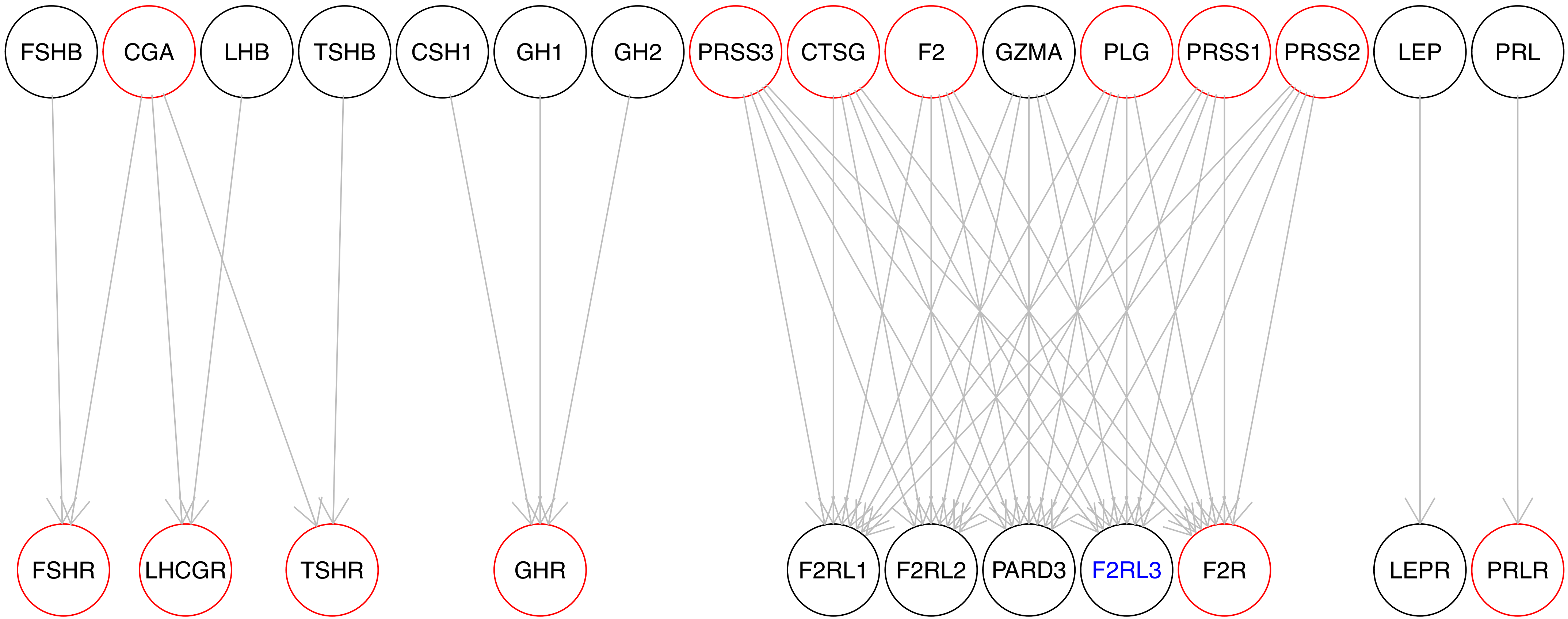

KEGG pathway = Neuroactive ligand−receptor interaction : tumour = YURIDA : Yellow Fill = gene variant, Blue Text = expression−survival association, Red Boarder = drug

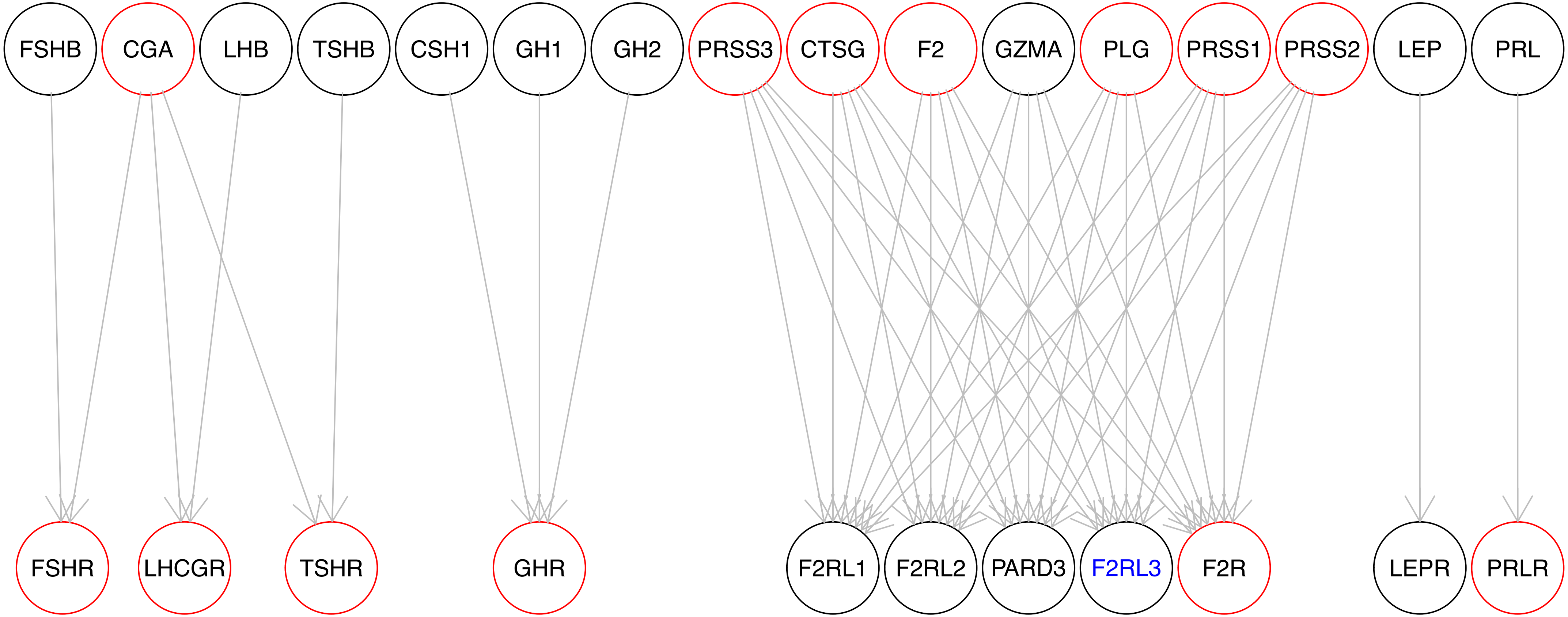

KEGG pathway = Neuroactive ligand−receptor interaction : tumour = YURIF : Yellow Fill = gene variant, Blue Text = expression−survival association, Red Boarder = drug

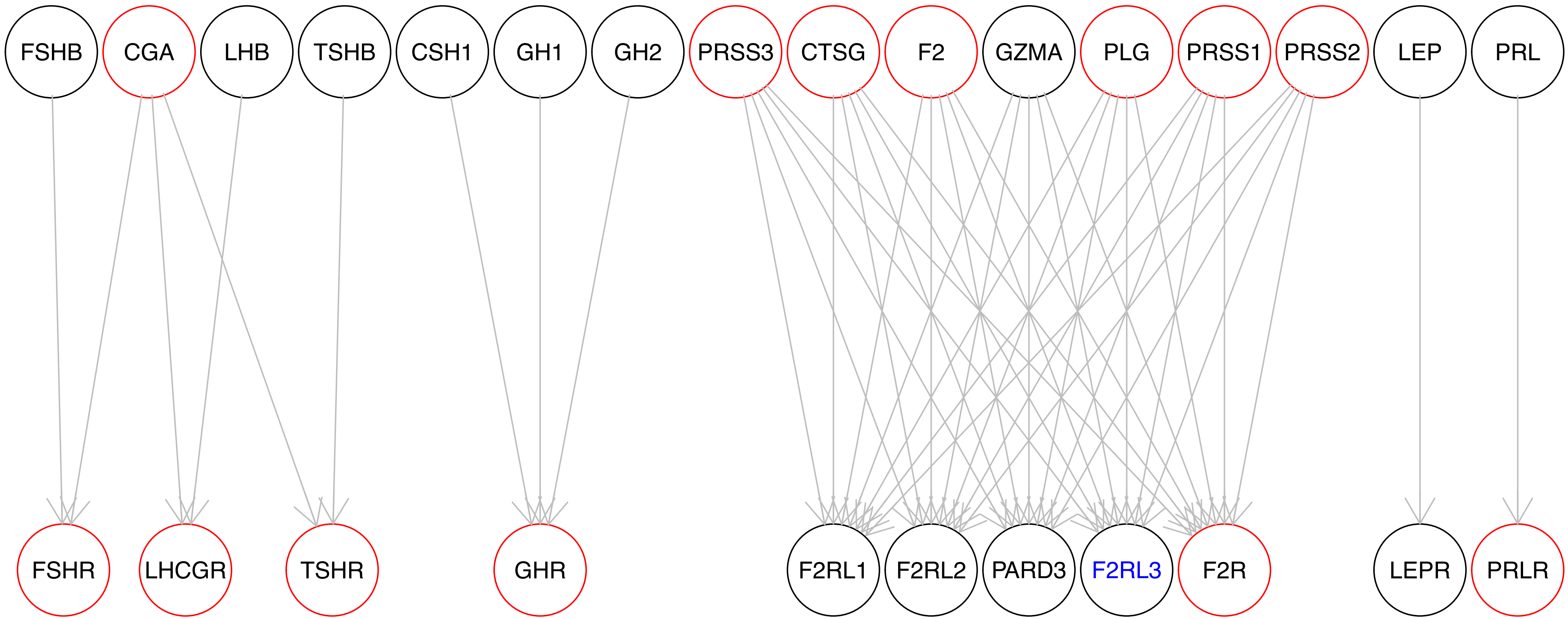

KEGG pathway = Neuroactive ligand−receptor interaction : tumour = YURKEN : Yellow Fill = gene variant, Blue Text = expression−survival association, Red Boarder = drug

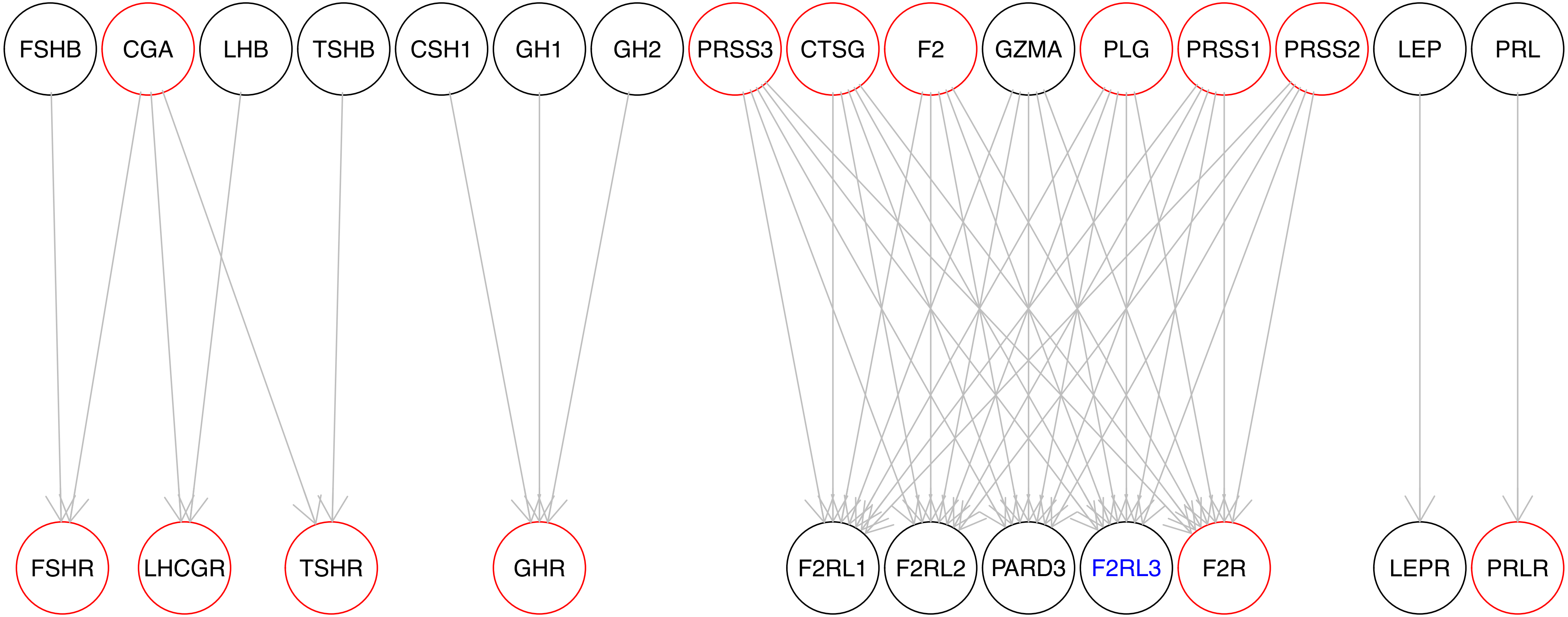

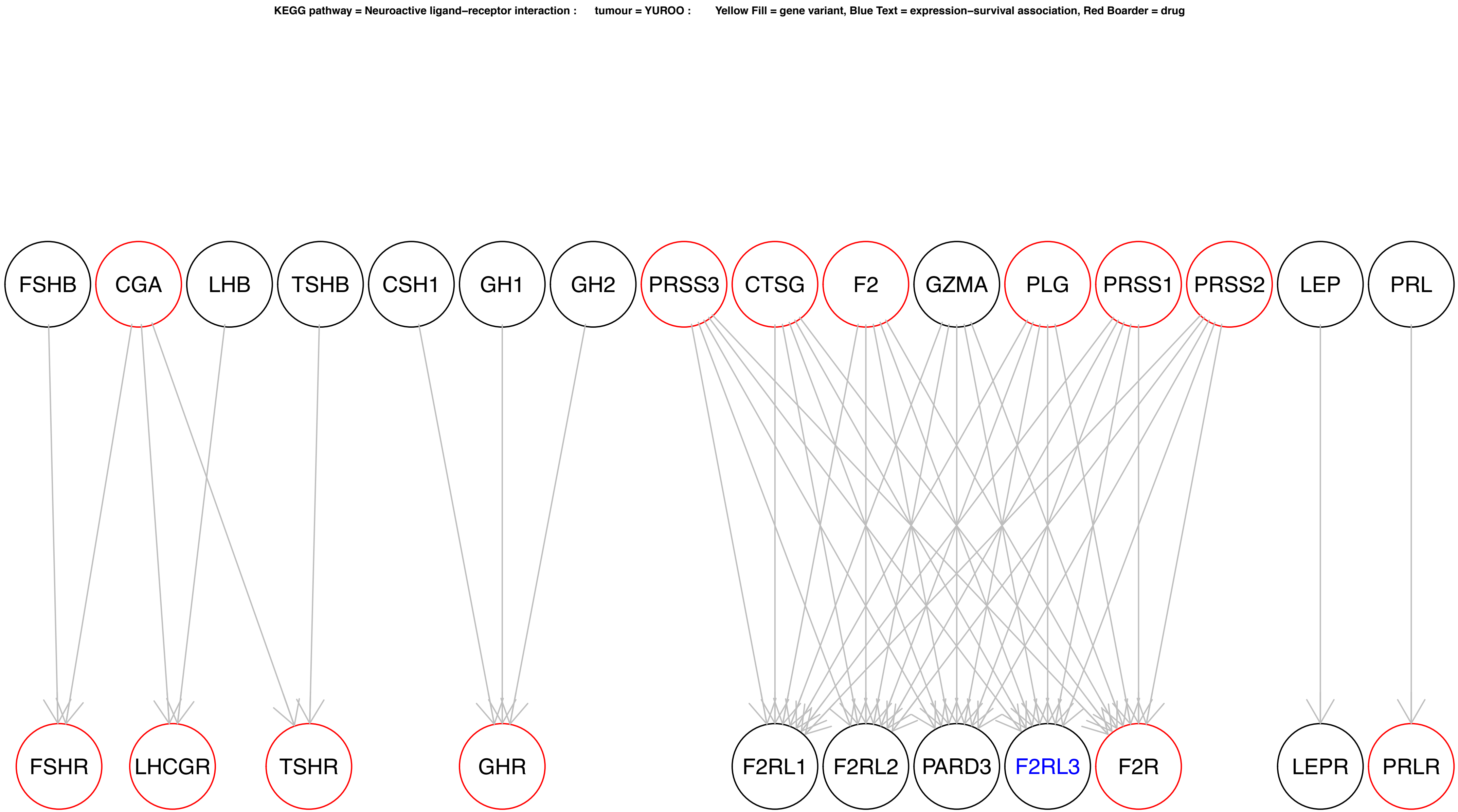
KEGG pathway = Neuroactive ligand−receptor interaction : tumour = YUROO : Yellow Fill = gene variant, Blue Text = expression−survival association, Red Boarder = drug
FSHB
CGA
LHB
TSHB
CSH1
GH1
GH2
PRSS3
CTSG
F2
GZMA
PLG
PRSS1
PRSS2
LEP
PRL
FSHR
LHCGR
TSHR
GHR
F2RL1
F2RL2
PARD3
F2RL3
F2R
LEPR
PRLR

KEGG pathway = Neuroactive ligand−receptor interaction :   tumour = YURTHE :   Yellow Fill = gene variant, Blue Text = expression−survival association, Red Boarder = drug

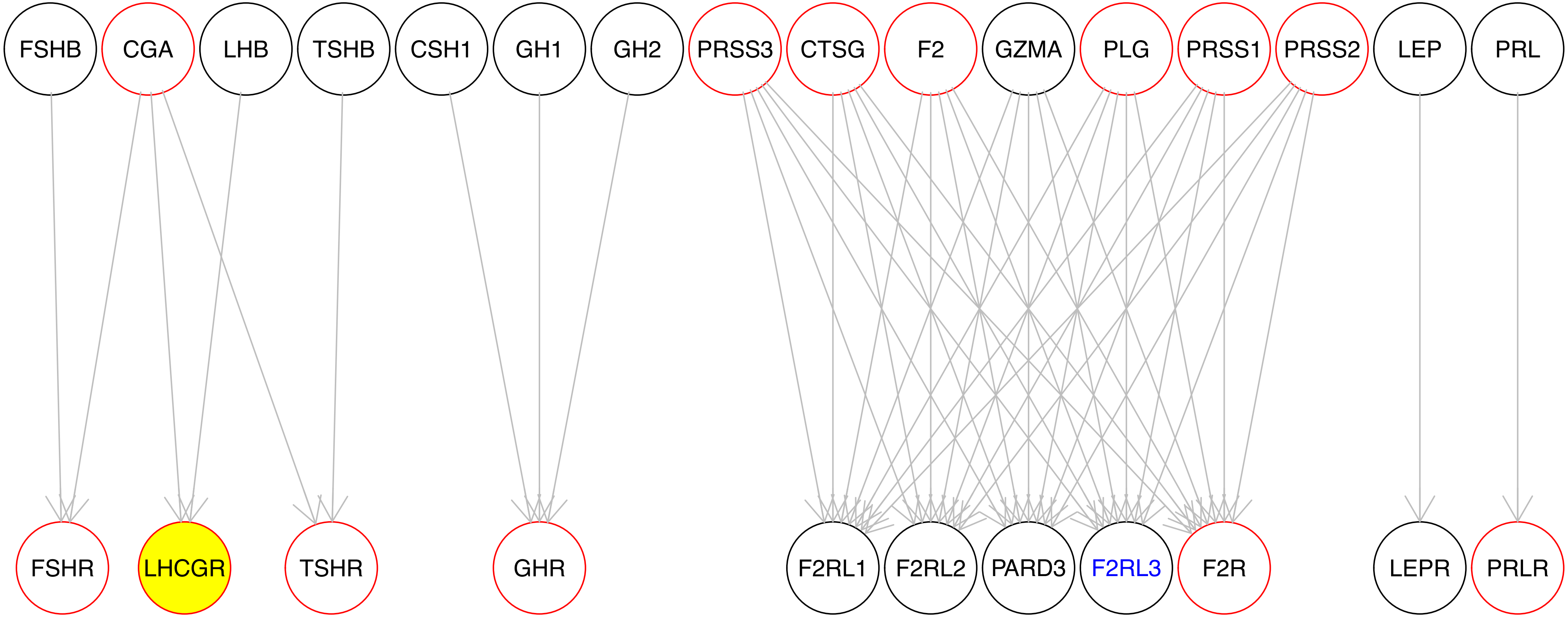

KEGG pathway = Neuroactive ligand−receptor interaction :   tumour = YURUB :   Yellow Fill = gene variant, Blue Text = expression−survival association, Red Boarder = drug

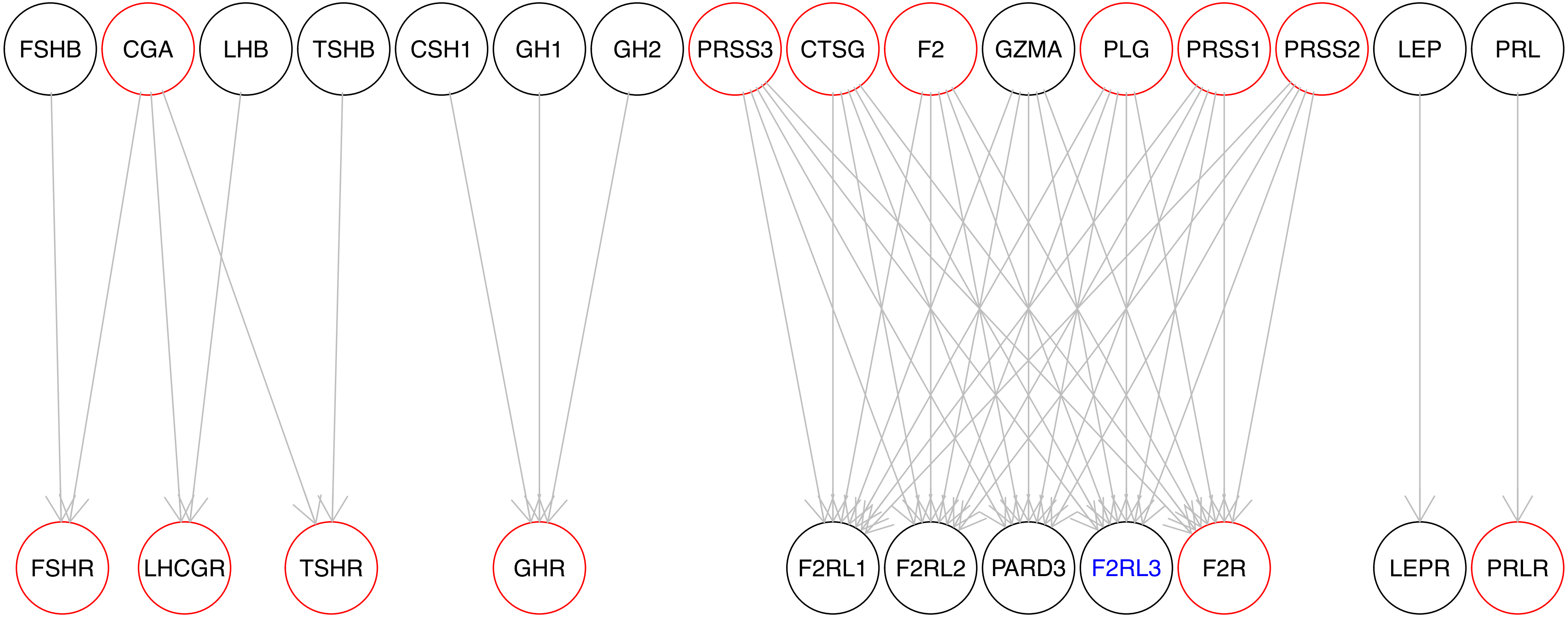

KEGG pathway = Neuroactive ligand−receptor interaction :   tumour = YURUS :   Yellow Fill = gene variant, Blue Text = expression−survival association, Red Boarder = drug

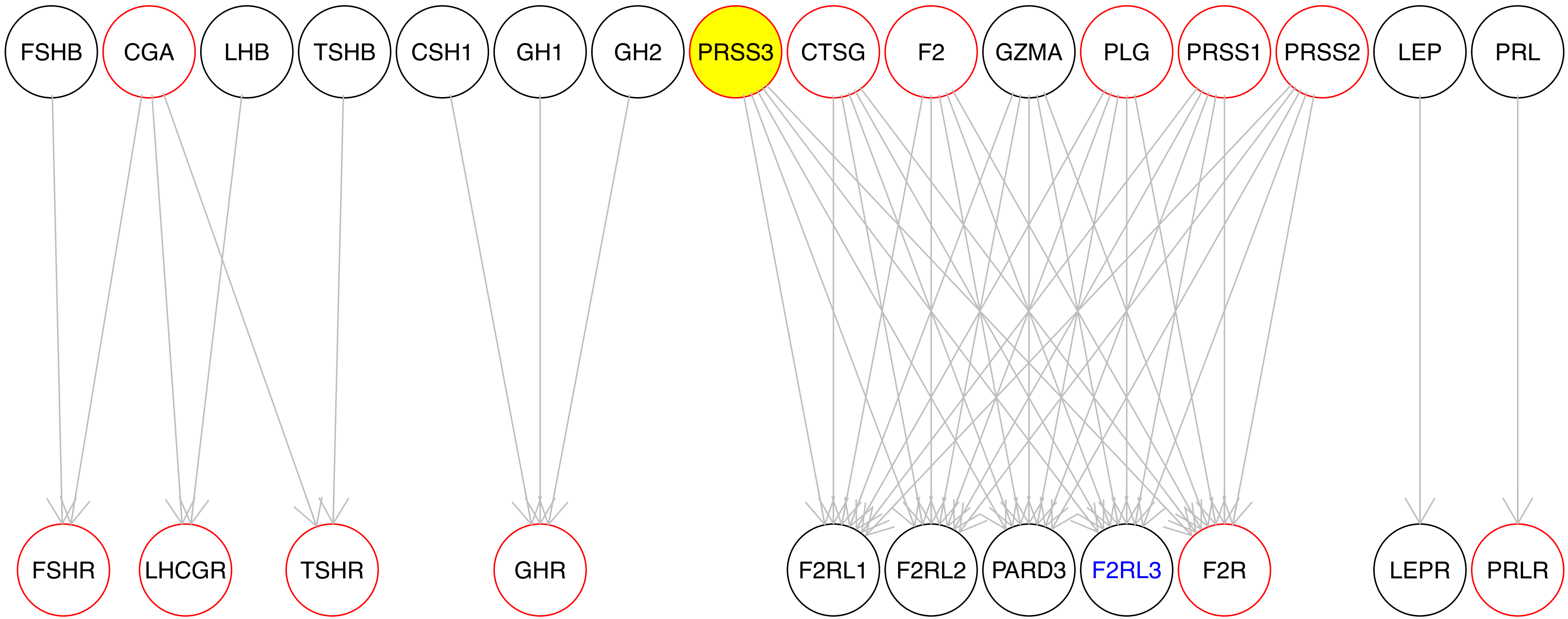

KEGG pathway = Neuroactive ligand−receptor interaction :   tumour = YUSAG :   Yellow Fill = gene variant, Blue Text = expression−survival association, Red Boarder = drug

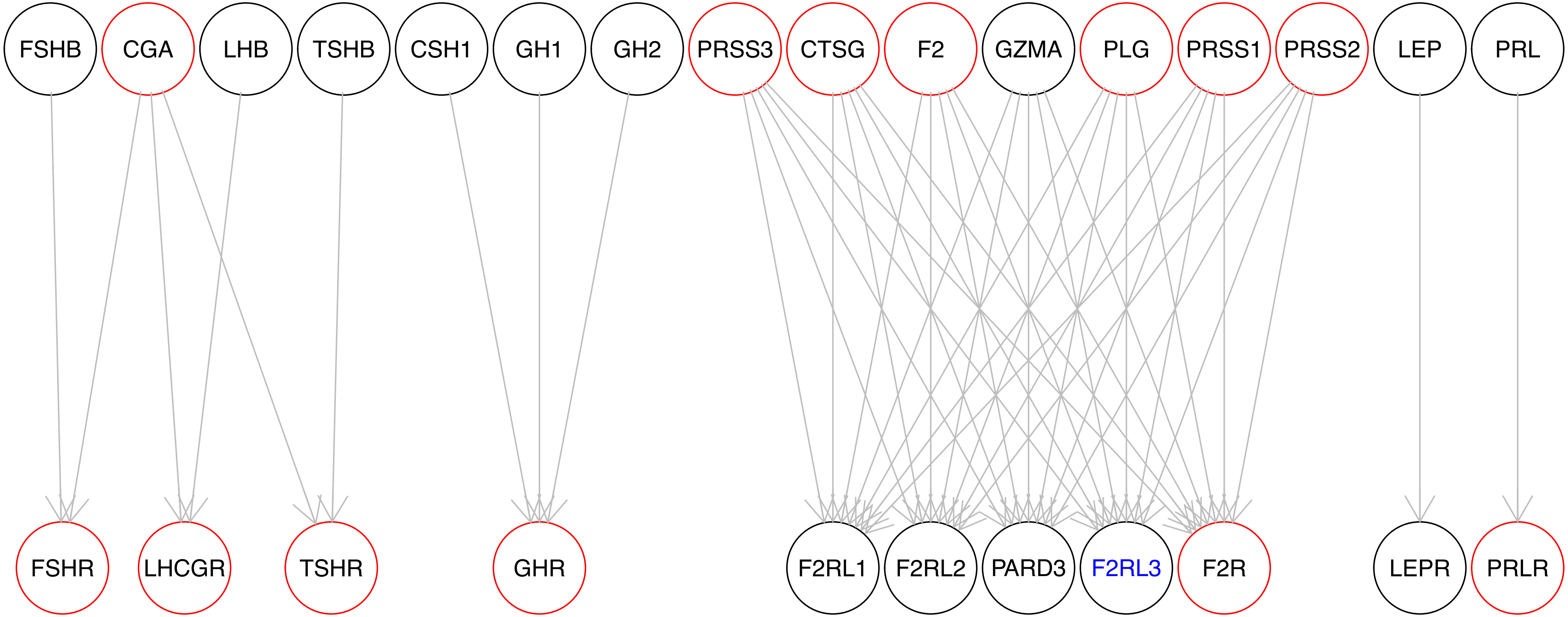

KEGG pathway = Neuroactive ligand−receptor interaction : tumour = YUSAN : Yellow Fill = gene variant, Blue Text = expression−survival association, Red Boarder = drug

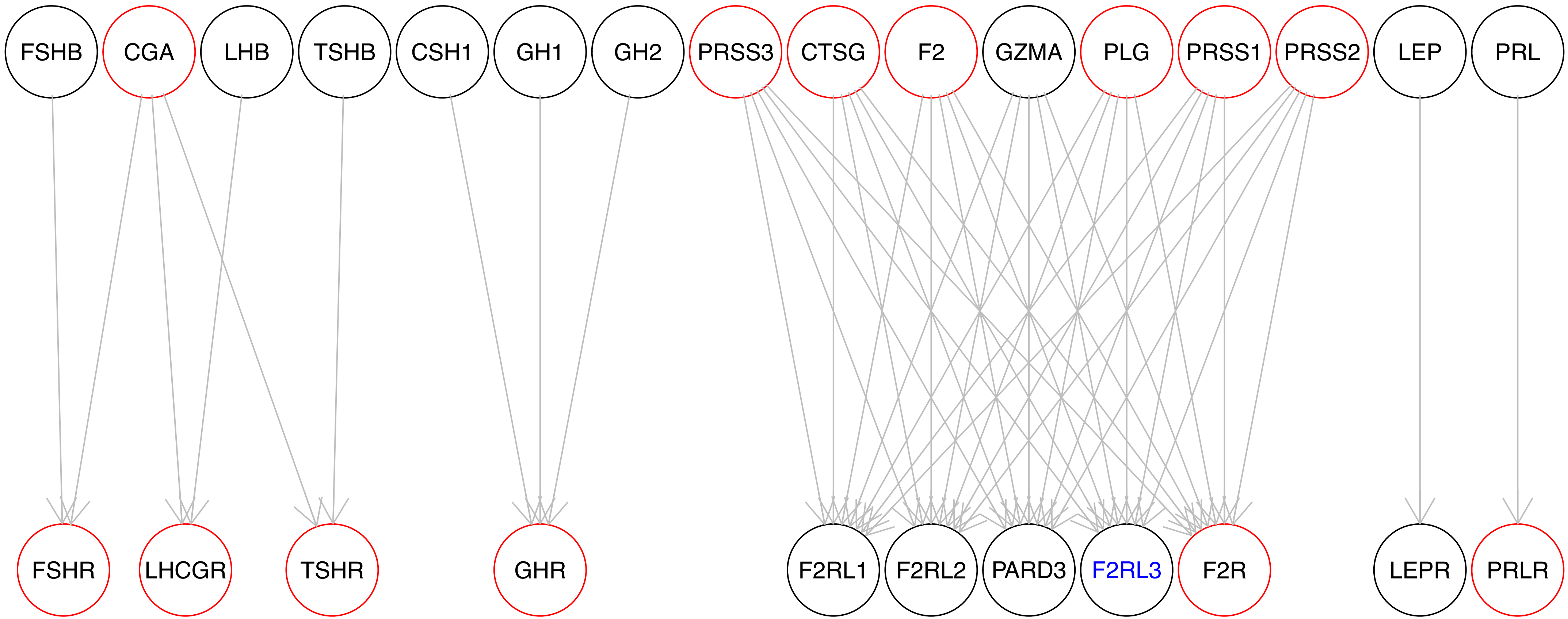

KEGG pathway = Neuroactive ligand−receptor interaction : tumour = YUSARI : Yellow Fill = gene variant, Blue Text = expression−survival association, Red Boarder = drug

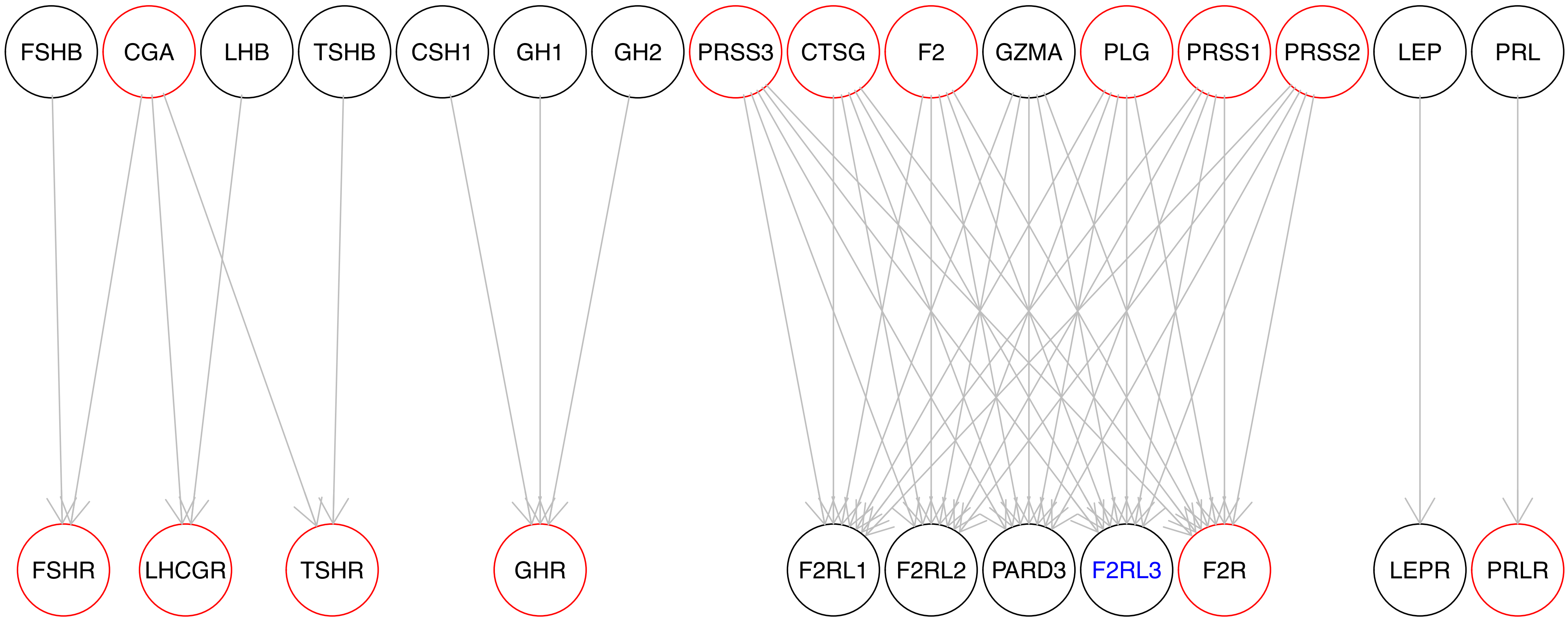

KEGG pathway = Neuroactive ligand−receptor interaction : tumour = YUSCO : Yellow Fill = gene variant, Blue Text = expression−survival association, Red Boarder = drug

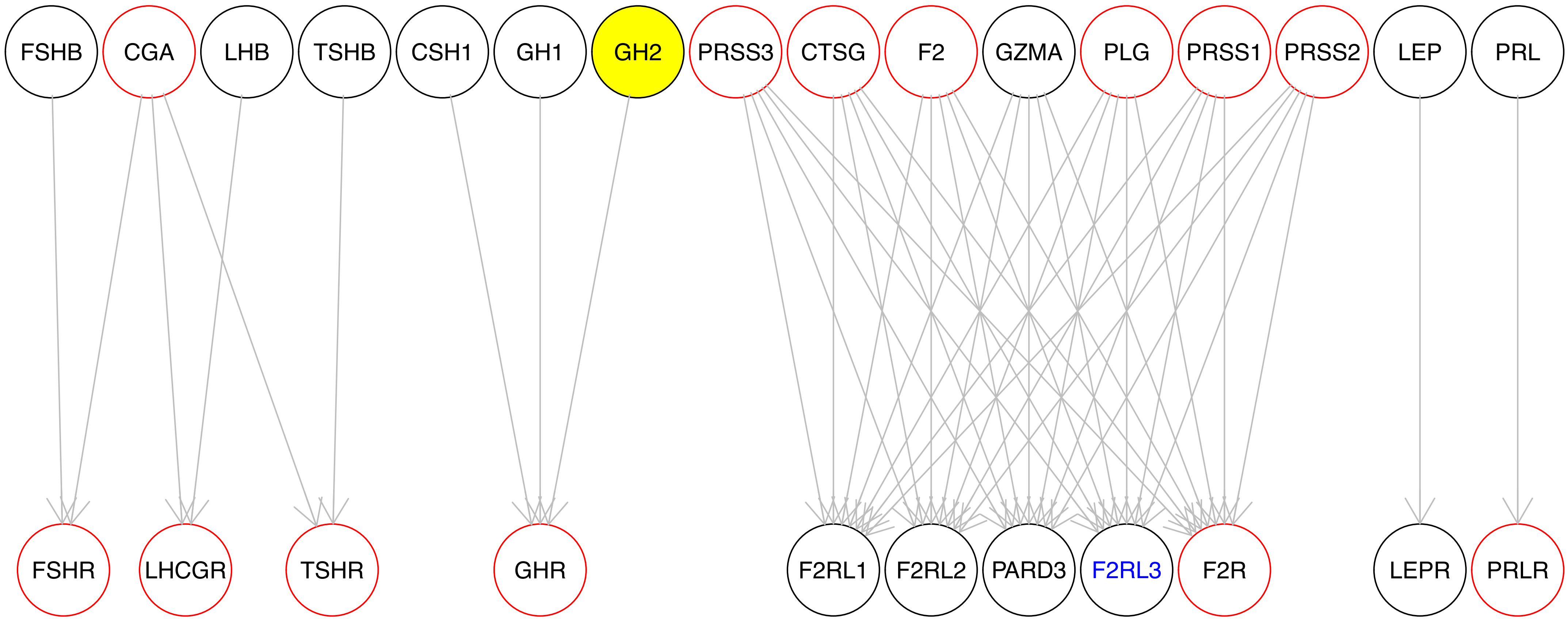

KEGG pathway = Neuroactive ligand−receptor interaction :   tumour = YUSEL :   Yellow Fill = gene variant, Blue Text = expression−survival association, Red Boarder = drug

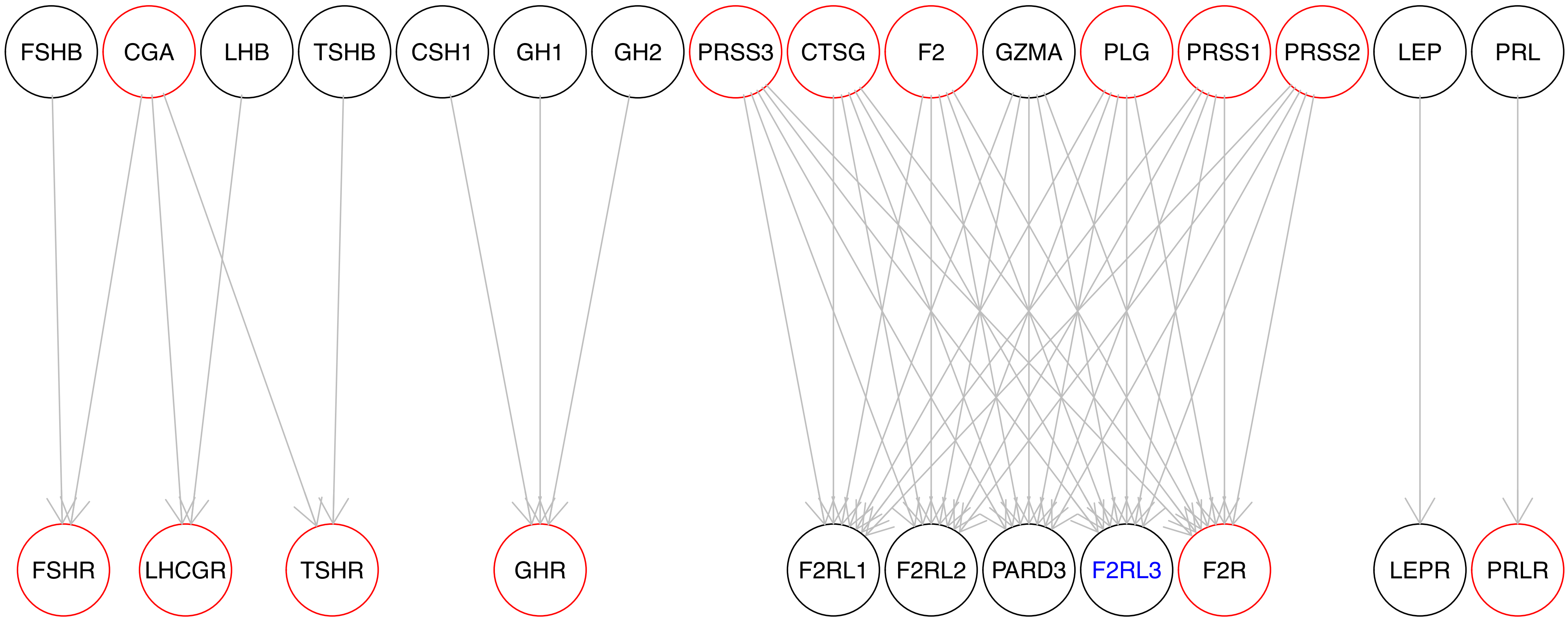

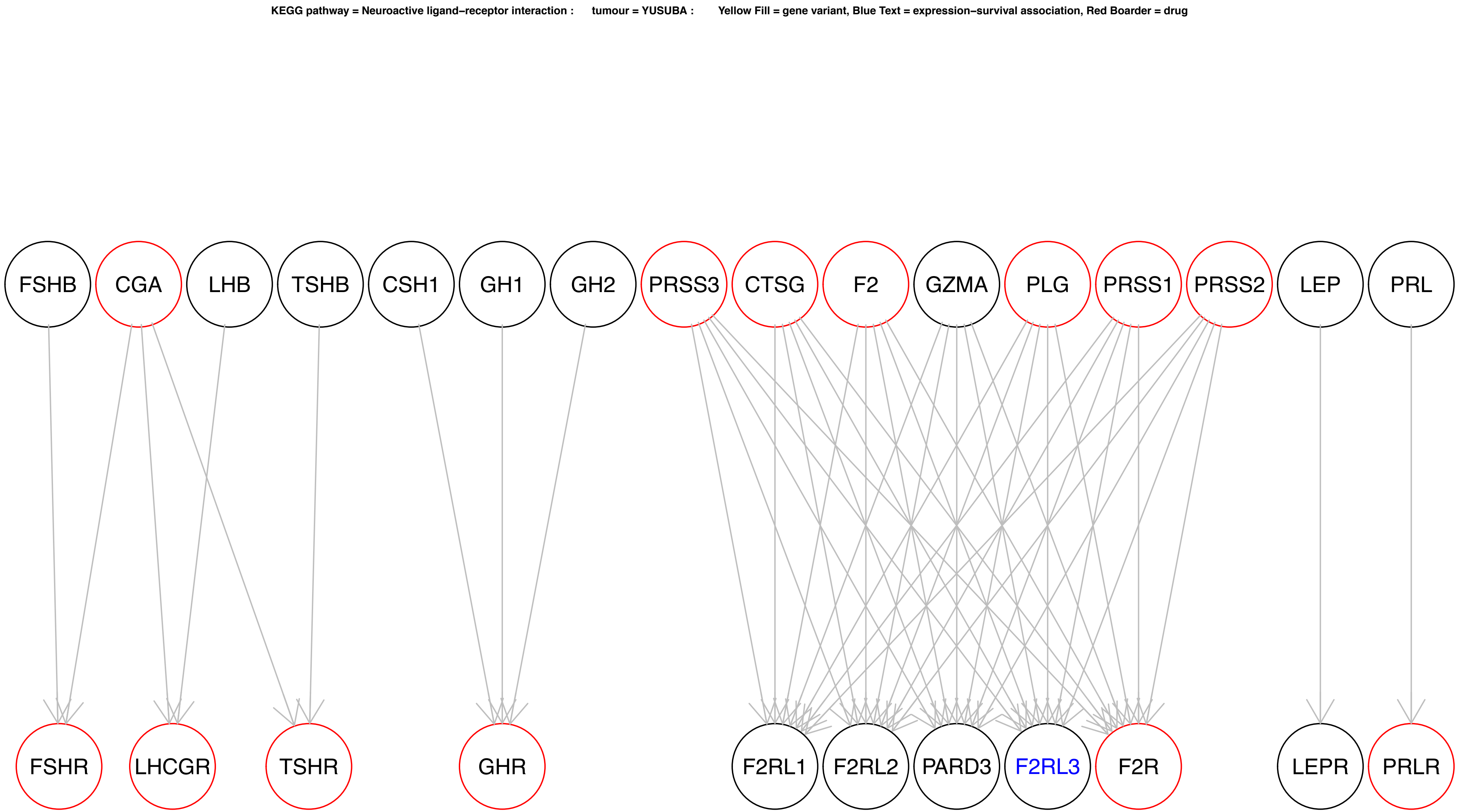

KEGG pathway = Neuroactive ligand−receptor interaction : tumour = YUSUBA : Yellow Fill = gene variant, Blue Text = expression−survival association, Red Boarder = drug
FSHB
CGA
LHB
TSHB
CSH1
GH1
GH2
PRSS3
CTSG
F2
GZMA
PLG
PRSS1
PRSS2
LEP
PRL
FSHR
LHCGR
TSHR
GHR
F2RL1
F2RL2
PARD3
F2RL3
F2R
LEPR
PRLR

KEGG pathway = Neuroactive ligand−receptor interaction : tumour = YUSWI : Yellow Fill = gene variant, Blue Text = expression−survival association, Red Boarder = drug

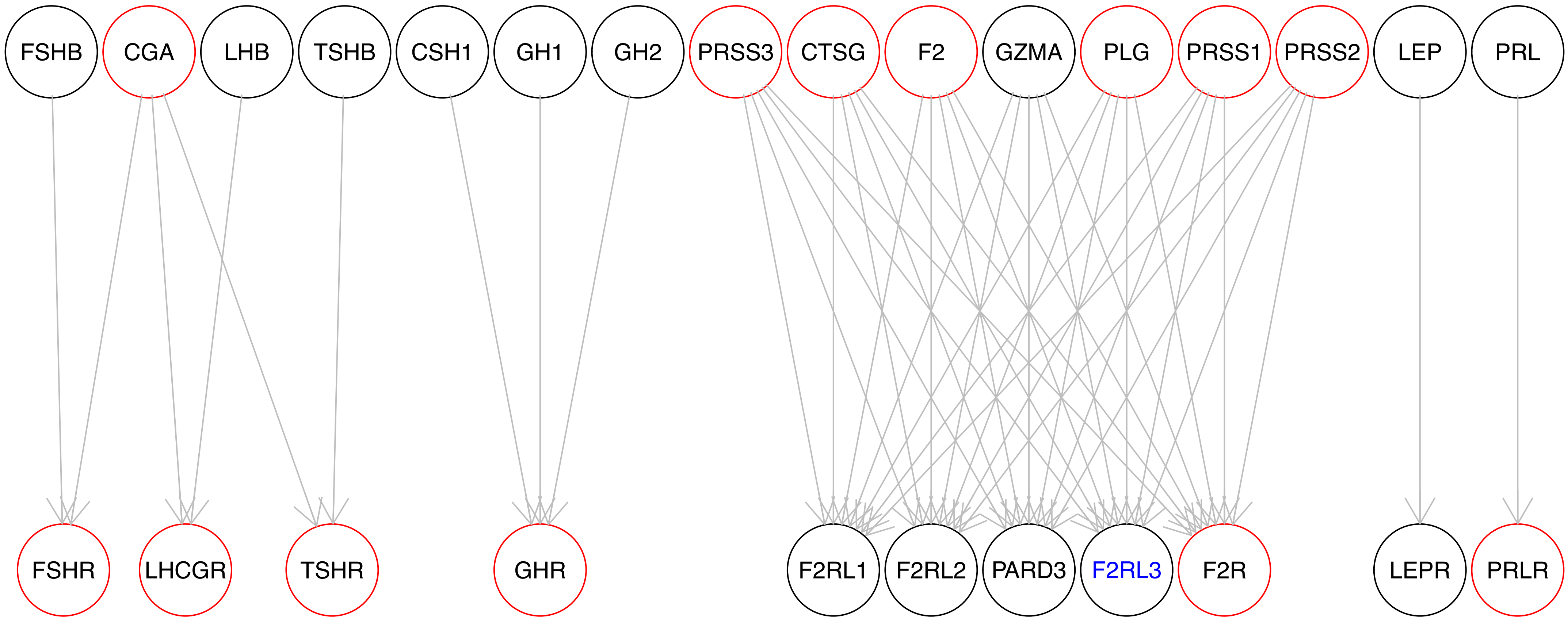

KEGG pathway = Neuroactive ligand−receptor interaction :   tumour = YUTEPA :   Yellow Fill = gene variant, Blue Text = expression−survival association, Red Boarder = drug

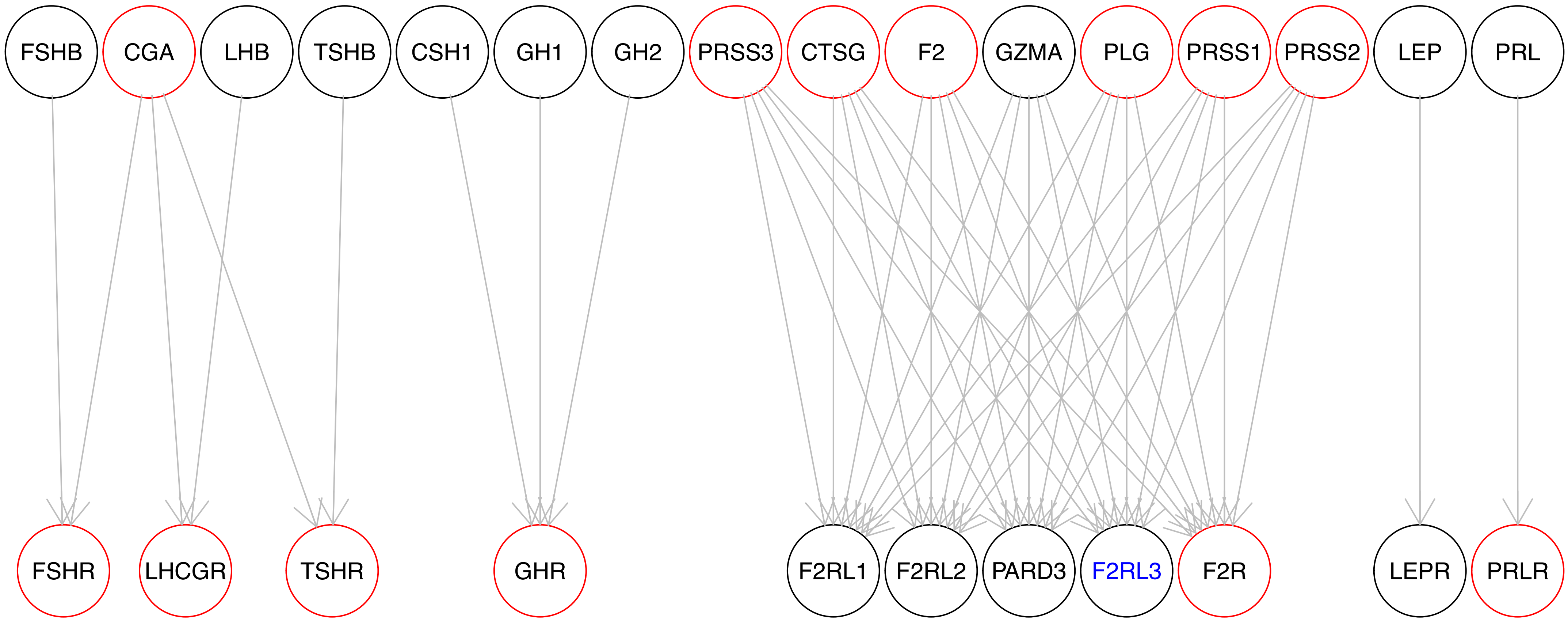

KEGG pathway = Neuroactive ligand−receptor interaction : tumour = YUTER : Yellow Fill = gene variant, Blue Text = expression−survival association, Red Boarder = drug

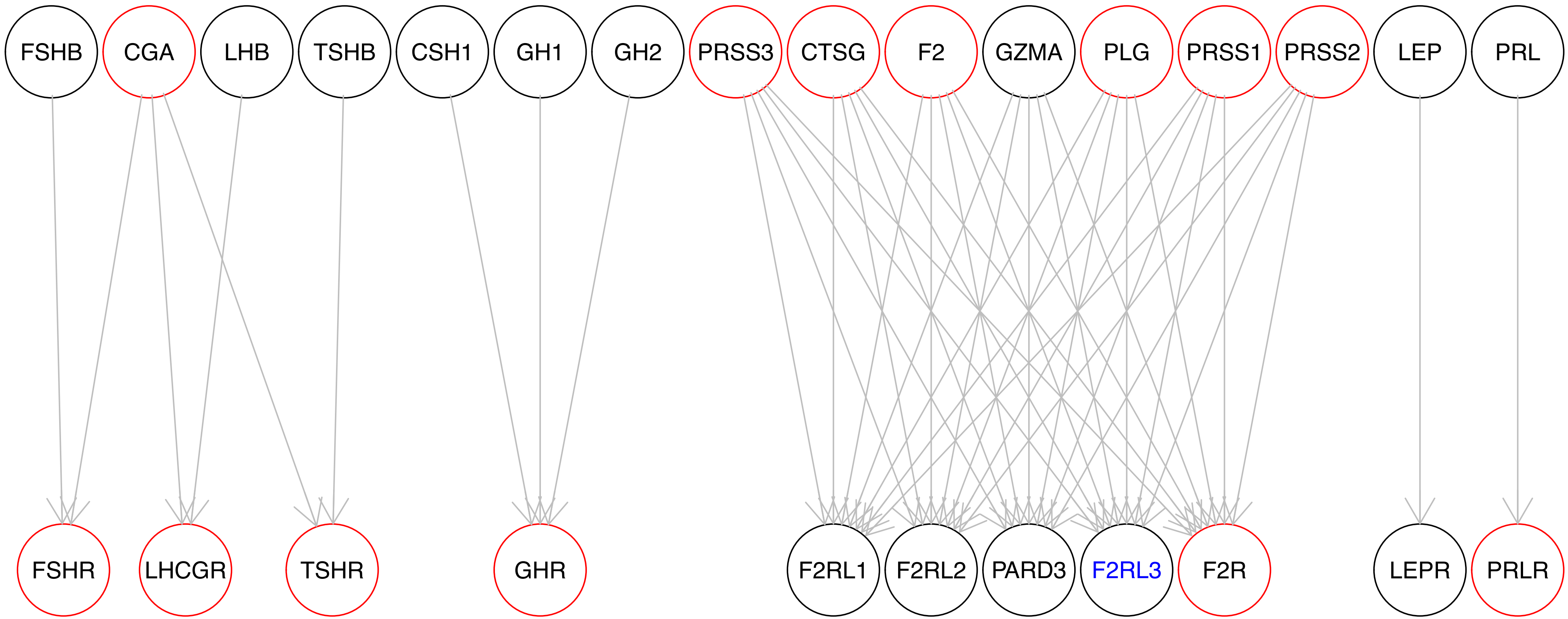

KEGG pathway = Neuroactive ligand−receptor interaction : tumour = YUTOGS : Yellow Fill = gene variant, Blue Text = expression−survival association, Red Boarder = drug

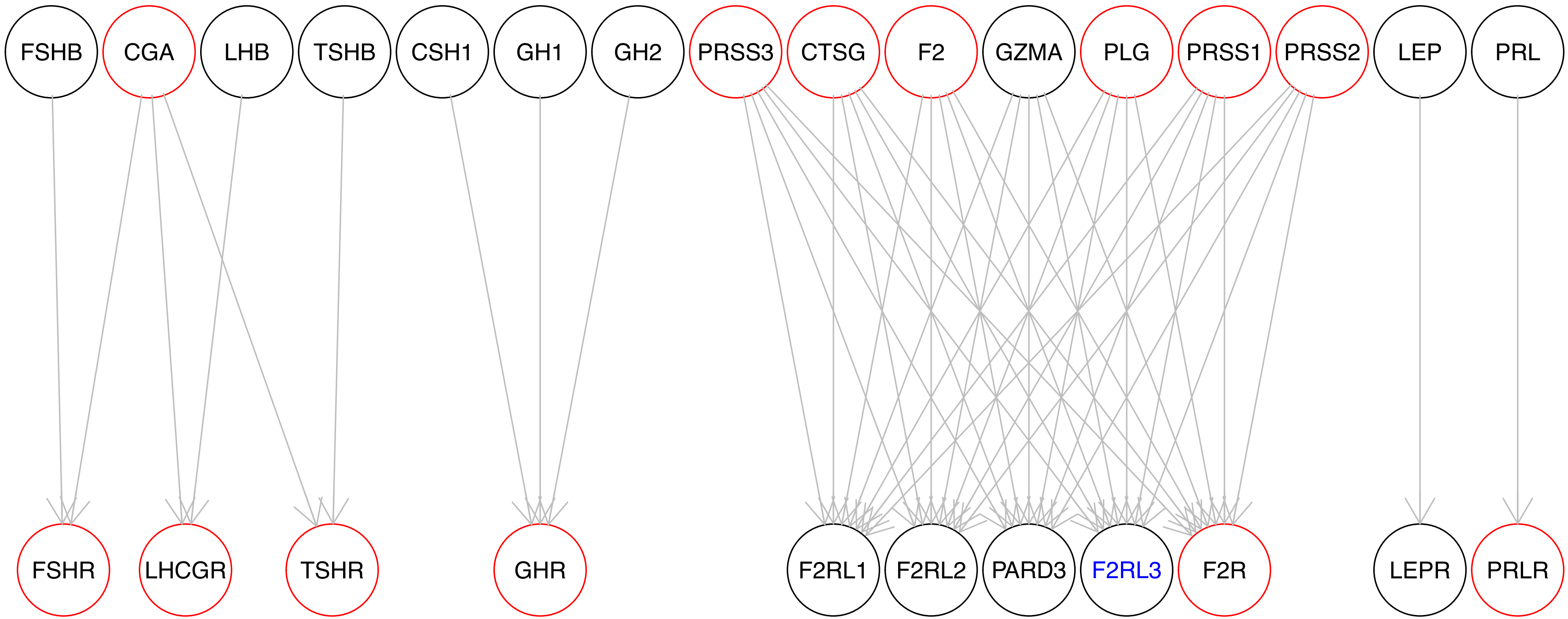

KEGG pathway = Neuroactive ligand−receptor interaction : tumour = YUTOLL : Yellow Fill = gene variant, Blue Text = expression−survival association, Red Boarder = drug

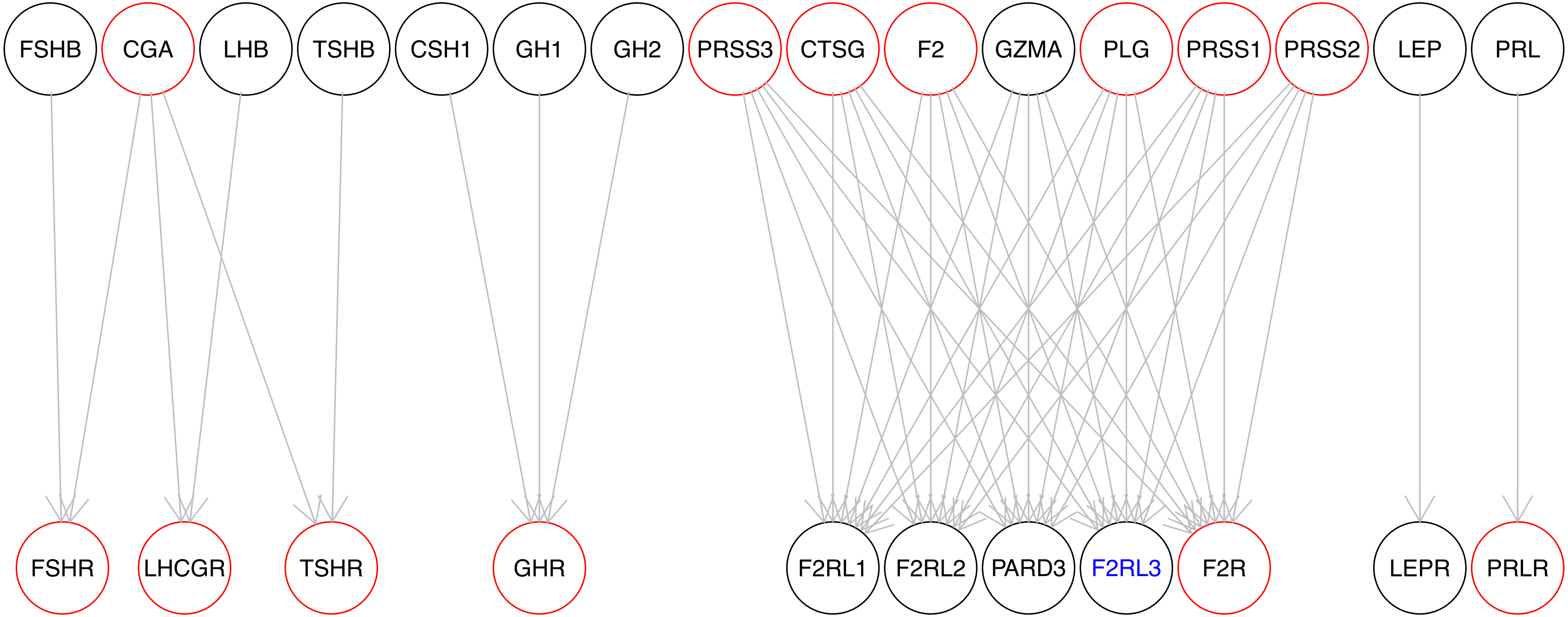

KEGG pathway = Neuroactive ligand−receptor interaction :   tumour = YUVAIL :   Yellow Fill = gene variant, Blue Text = expression−survival association, Red Boarder = drug

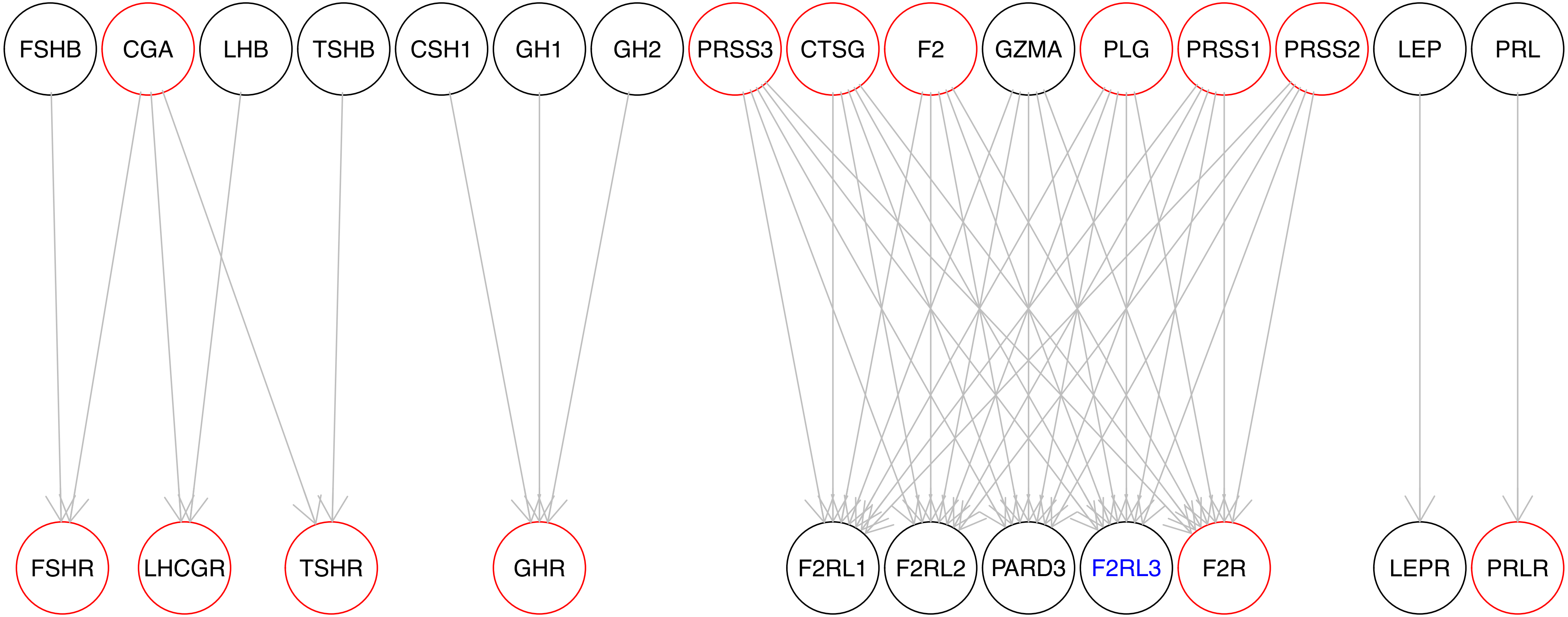

KEGG pathway = Neuroactive ligand−receptor interaction :   tumour = YUWAGE :   Yellow Fill = gene variant, Blue Text = expression−survival association, Red Boarder = drug

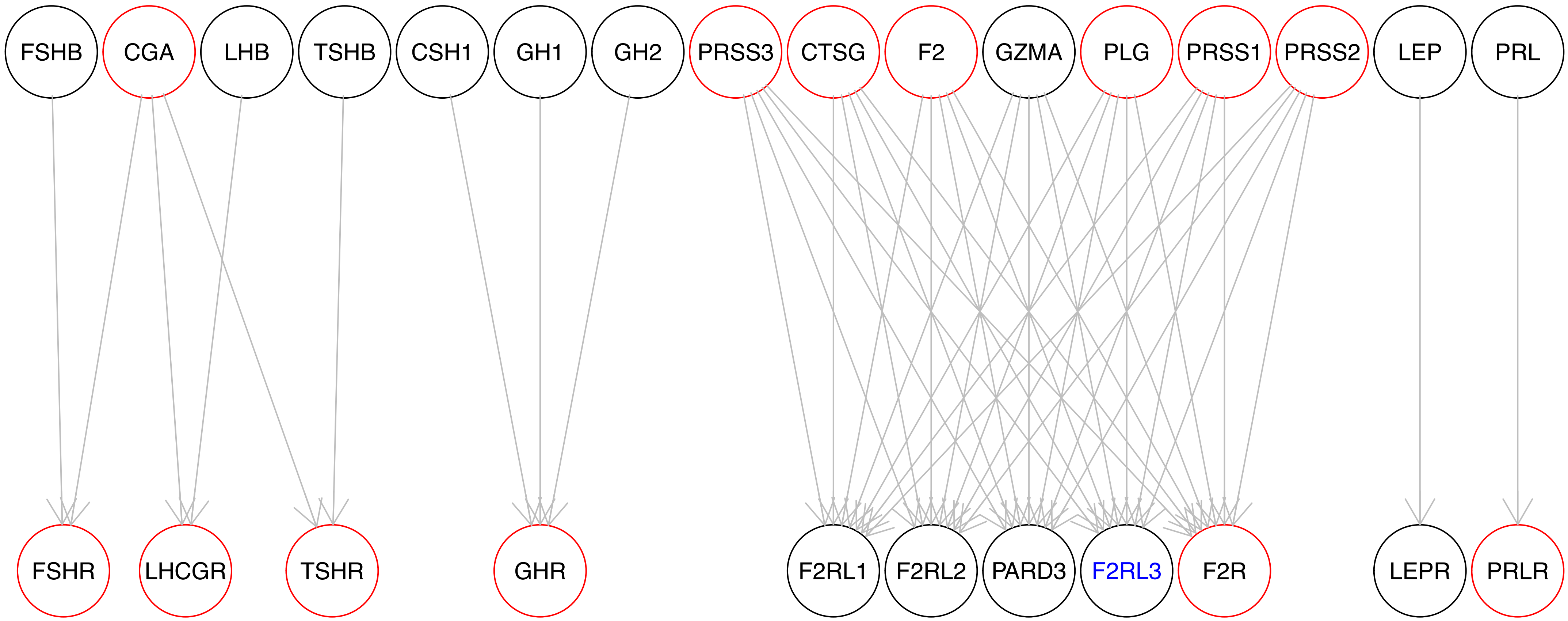

KEGG pathway = Neuroactive ligand−receptor interaction :   tumour = YUWALI :   Yellow Fill = gene variant, Blue Text = expression−survival association, Red Boarder = drug

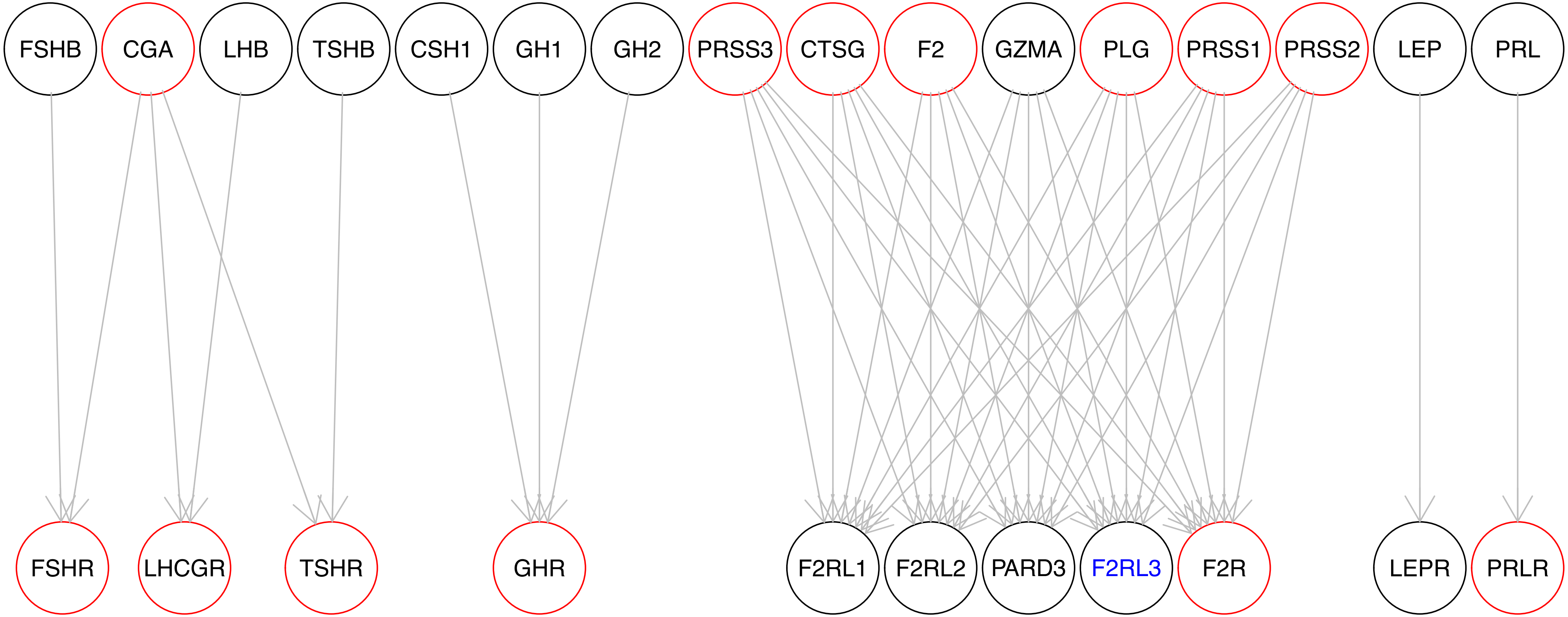

KEGG pathway = Neuroactive ligand−receptor interaction : tumour = YUWAND : Yellow Fill = gene variant, Blue Text = expression−survival association, Red Boarder = drug

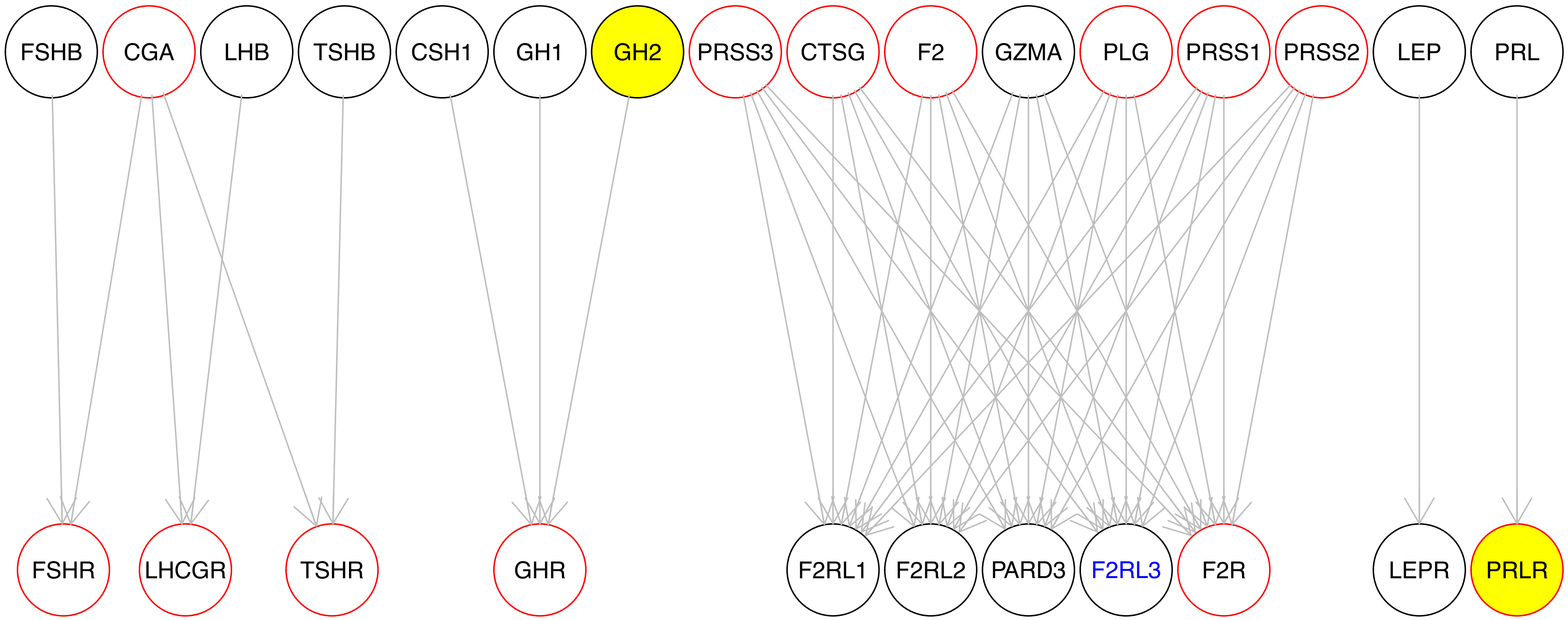

KEGG pathway = Neuroactive ligand−receptor interaction : tumour = YUWHIM : Yellow Fill = gene variant, Blue Text = expression−survival association, Red Boarder = drug

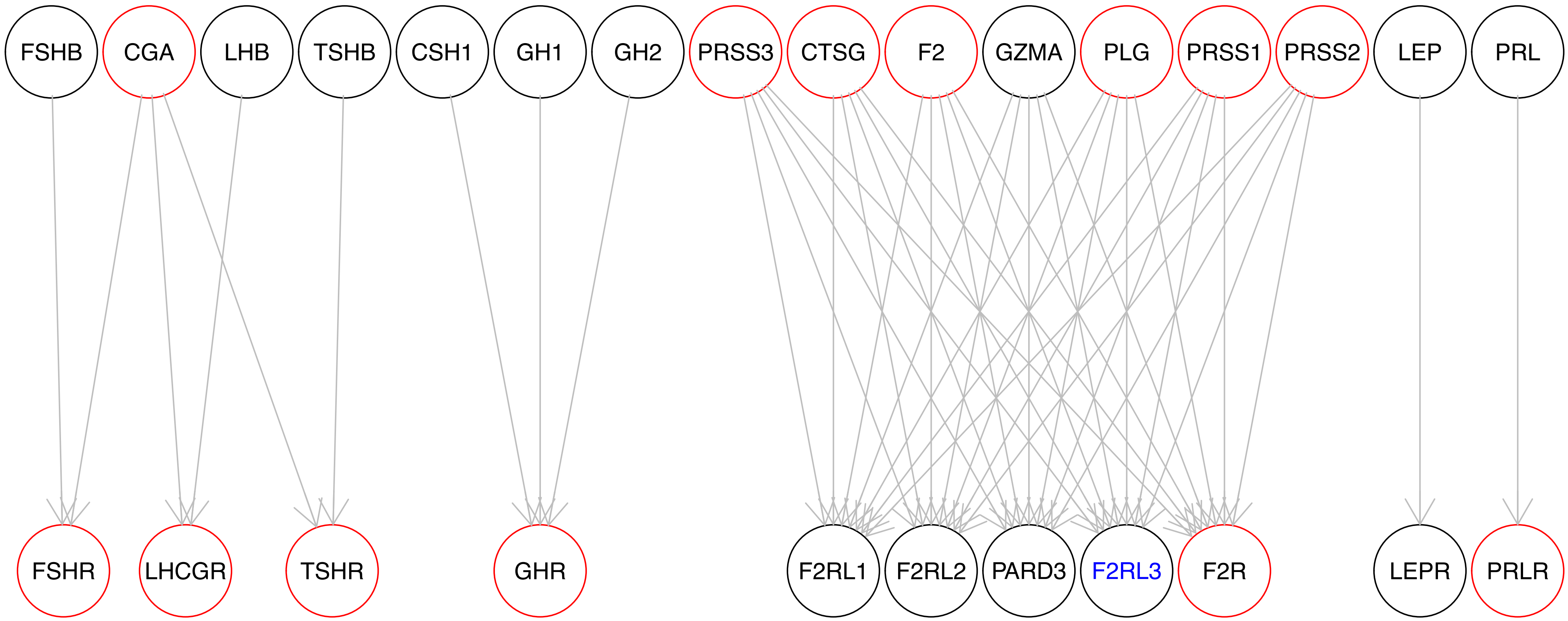

KEGG pathway = Neuroactive ligand−receptor interaction :   tumour = YUZEAL :   Yellow Fill = gene variant, Blue Text = expression−survival association, Red Boarder = drug

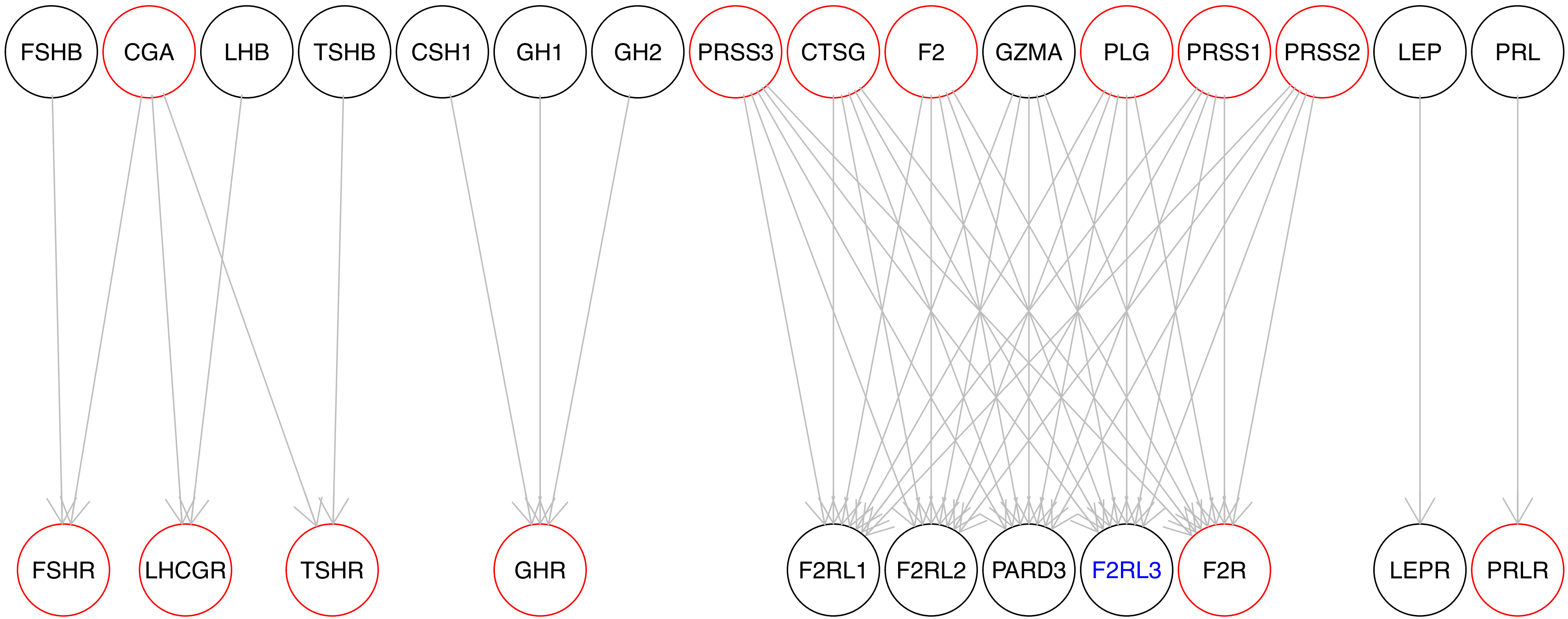

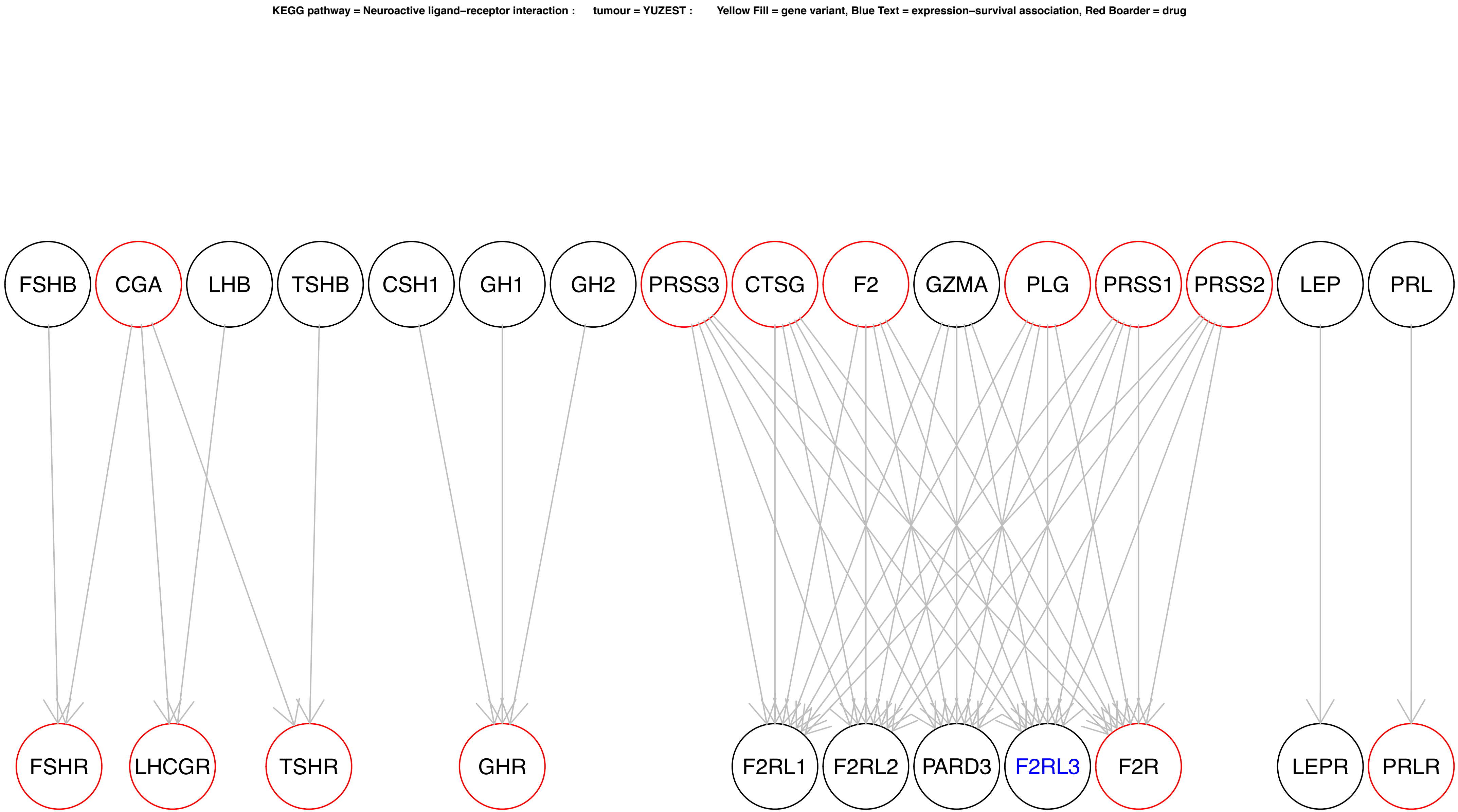
KEGG pathway = Neuroactive ligand−receptor interaction :   tumour = YUZEST :   Yellow Fill = gene variant, Blue Text = expression−survival association, Red Boarder = drug
FSHB
CGA
LHB
TSHB
CSH1
GH1
GH2
PRSS3
CTSG
F2
GZMA
PLG
PRSS1
PRSS2
LEP
PRL
FSHR
LHCGR
TSHR
GHR
F2RL1
F2RL2
PARD3
F2RL3
F2R
LEPR
PRLR

KEGG pathway = Neuroactive ligand−receptor interaction : tumour = YUZINO : Yellow Fill = gene variant, Blue Text = expression−survival association, Red Boarder = drug

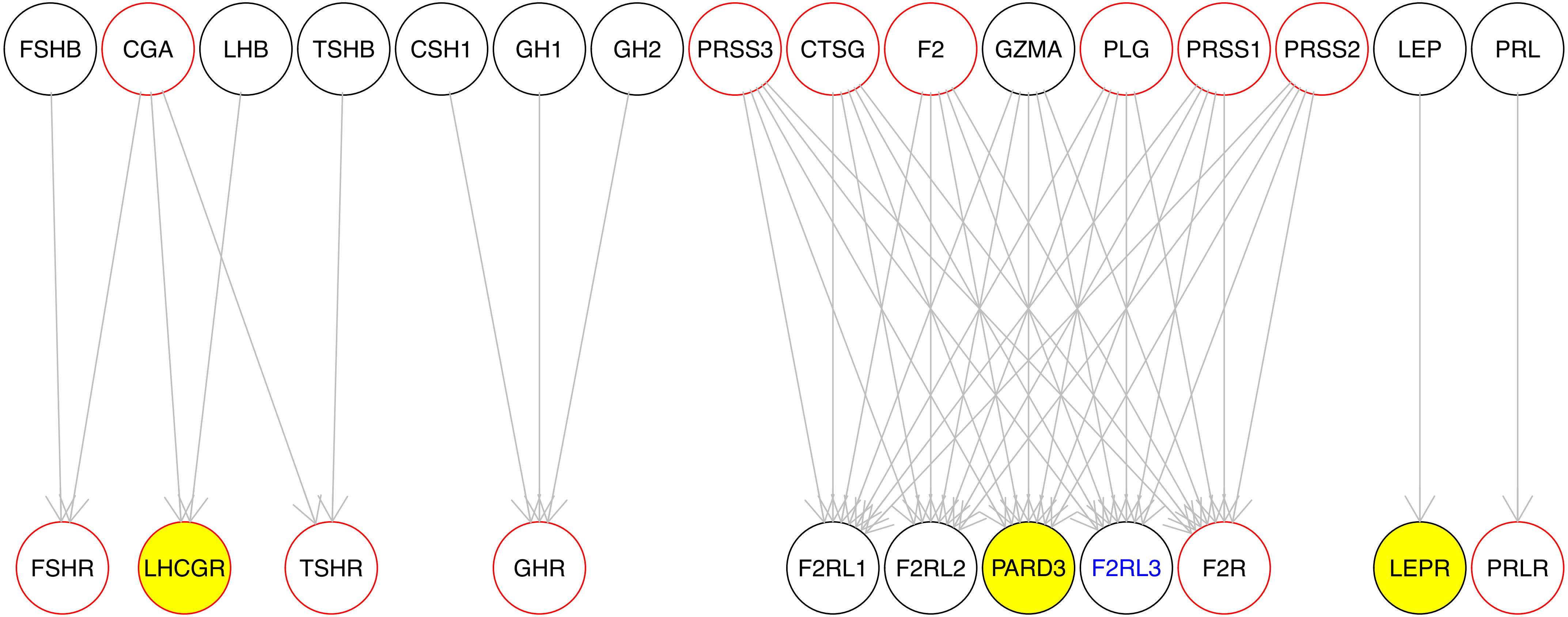

Supplement: Supplementary file 2 [file 51894_Print_Presentation2.PDF]
