# Supplementary material for: MelanomaDB: A Web Tool for Integrative Analysis of Melanoma Genomic Information to Identify Disease-Associated Molecular Pathways
Source: Front Oncol. 2013 Jul 16;3:184. doi: 10.3389/fonc.2013.00184 (PMC3712543; doi:10.3389/fonc.2013.00184)
Supplement: Supplementary file 7 [file 51894_Print_DataSheet5.DOCX]

#**************************************************

# R scripts used for for generation of Figure 2A.

# First data was read from the clipboard into an object "a", a histogram object was generated, then a modified histogram was plotted.

a<-read.delim(pipe("pbpaste"))

sss<-(hist(a[,2], breaks=100)

plot(sss$count~sss$mids, log="xy", type='h', lwd=2.5, lend=2, col="red", xlab="number of tumours (/310)", ylab="number of genes with alterations")

#**************************************************

#**************************************************

# R scripts used for for generation of Figure 2B.

# First data was read from the clipboard into an object "a", vectors representing the number of tumours (/310) with mutations and total exon length were assigned, and a scatterplot was generated.

a<-read.delim(pipe("pbpaste"))

GL<-a[,2]

tn<-a[,3]

plot(tn,GL,log="xy",col="black",xlab="number of tumours (/310) with mutation in gene",ylab="total exon length of gene in base pairs",xlim=c(1,200),ylim=range(GL),pch=1)

par(new=TRUE)

plot(lowess(GL[-2]~tn[-2],f=0.1),col="red", type="l",log="xy",xlim=c(1,200),ylim=range(GL),xlab="",ylab="")

#**************************************************

#**************************************************

# R scripts used for for generation of Figure 2C.

# First data was read from the clipboard into an object "a", then a simple histogram was plotted.

a<-read.delim(pipe("pbpaste"))

hist(a[,2], breaks=100, main="",xlab="number of genes with alterations per tumour",cex=2)

#**************************************************

#**************************************************

# R script used for generation of pathway diagrams.

#************first define heatmap.csm() function, a modification of heatmap.2() from the plots package

heatmap.csm<-function (x, Rowv = TRUE, Colv = if (symm) "Rowv" else TRUE,

distfun = dist, hclustfun = hclust, dendrogram = c("both",

"row", "column", "none"), symm = FALSE, scale = c("none",

"row", "column"), na.rm = TRUE, revC = identical(Colv,

"Rowv"), add.expr, breaks, col = "heat.colors", colsep,

rowsep, sepcolor = "white", sepwidth = c(0.05, 0.05), cellnote,

notecex = 1, notecol = "cyan", na.color = par("bg"), trace = c("column",

"row", "both", "none"), tracecol = "cyan", hline = median(breaks),

vline = median(breaks), linecol = tracecol, margins = c(5,

5), ColSideColors, RowSideColors, cexRow = 0.2 + 1/log10(nr),

cexCol = 0.2 + 1/log10(nc), labRow = NULL, labCol = NULL,

key = TRUE, keysize = 1.5, density.info = c("histogram",

"density", "none"), denscol = tracecol, symkey = min(x <

0, na.rm = TRUE), densadj = 0.25, main = NULL, xlab = NULL,

ylab = NULL, ...)

{

scale01 <- function(x, low = min(x), high = max(x)) {

x <- (x - low)/(high - low)

x

}

scale <- if (symm && missing(scale))

"none"

else match.arg(scale)

dendrogram <- match.arg(dendrogram)

trace <- match.arg(trace)

density.info <- match.arg(density.info)

if (!missing(breaks) && (scale != "none"))

warning("Using scale=\"row\" or scale=\"column\" when breaks are",

"specified can produce unpredictable results.", "Please consider using only one or the other.")

if ((Colv == "Rowv") && (!isTRUE(Rowv) || is.null(Rowv)))

Colv <- FALSE

if (length(di <- dim(x)) != 2 || !is.numeric(x))

stop("`x' must be a numeric matrix")

nr <- di[1]

nc <- di[2]

if (nr <= 1 || nc <= 1)

stop("`x' must have at least 2 rows and 2 columns")

if (!is.numeric(margins) || length(margins) != 2)

stop("`margins' must be a numeric vector of length 2")

if (missing(cellnote))

cellnote <- matrix("", ncol = ncol(x), nrow = nrow(x))

if (!inherits(Rowv, "dendrogram")) {

if (((!isTRUE(Rowv)) || (is.null(Rowv))) && (dendrogram %in%

c("both", "row"))) {

if (is.logical(Colv) && (Colv))

dendrogram <- "column"

else dedrogram <- "none"

warning("Discrepancy: Rowv is FALSE, while dendrogram is `",

dendrogram, "'. Omitting row dendogram.")

}

}

if (!inherits(Colv, "dendrogram")) {

if (((!isTRUE(Colv)) || (is.null(Colv))) && (dendrogram %in%

c("both", "column"))) {

if (is.logical(Rowv) && (Rowv))

dendrogram <- "row"

else dendrogram <- "none"

warning("Discrepancy: Colv is FALSE, while dendrogram is `",

dendrogram, "'. Omitting column dendogram.")

}

}

if (inherits(Rowv, "dendrogram")) {

ddr <- Rowv

rowInd <- order.dendrogram(ddr)

}

else if (is.integer(Rowv)) {

hcr <- hclustfun(distfun(x))

ddr <- as.dendrogram(hcr)

ddr <- reorder(ddr, Rowv)

rowInd <- order.dendrogram(ddr)

if (nr != length(rowInd))

stop("row dendrogram ordering gave index of wrong length")

}

else if (isTRUE(Rowv)) {

Rowv <- rowMeans(x, na.rm = na.rm)

hcr <- hclustfun(distfun(x))

ddr <- as.dendrogram(hcr)

ddr <- reorder(ddr, Rowv)

rowInd <- order.dendrogram(ddr)

if (nr != length(rowInd))

stop("row dendrogram ordering gave index of wrong length")

}

else {

rowInd <- nr:1

}

if (inherits(Colv, "dendrogram")) {

ddc <- Colv

colInd <- order.dendrogram(ddc)

}

else if (identical(Colv, "Rowv")) {

if (nr != nc)

stop("Colv = \"Rowv\" but nrow(x) != ncol(x)")

if (exists("ddr")) {

ddc <- ddr

colInd <- order.dendrogram(ddc)

}

else colInd <- rowInd

}

else if (is.integer(Colv)) {

hcc <- hclustfun(distfun(if (symm)

x

else t(x)))

ddc <- as.dendrogram(hcc)

ddc <- reorder(ddc, Colv)

colInd <- order.dendrogram(ddc)

if (nc != length(colInd))

stop("column dendrogram ordering gave index of wrong length")

}

else if (isTRUE(Colv)) {

Colv <- colMeans(x, na.rm = na.rm)

hcc <- hclustfun(distfun(if (symm)

x

else t(x)))

ddc <- as.dendrogram(hcc)

ddc <- reorder(ddc, Colv)

colInd <- order.dendrogram(ddc)

if (nc != length(colInd))

stop("column dendrogram ordering gave index of wrong length")

}

else {

colInd <- 1:nc

}

x <- x[rowInd, colInd]

x.unscaled <- x

cellnote <- cellnote[rowInd, colInd]

if (is.null(labRow))

labRow <- if (is.null(rownames(x)))

(1:nr)[rowInd]

else rownames(x)

else labRow <- labRow[rowInd]

if (is.null(labCol))

labCol <- if (is.null(colnames(x)))

(1:nc)[colInd]

else colnames(x)

else labCol <- labCol[colInd]

if (scale == "row") {

x <- sweep(x, 1, rowMeans(x, na.rm = na.rm))

sx <- apply(x, 1, sd, na.rm = na.rm)

x <- sweep(x, 1, sx, "/")

}

else if (scale == "column") {

x <- sweep(x, 2, colMeans(x, na.rm = na.rm))

sx <- apply(x, 2, sd, na.rm = na.rm)

x <- sweep(x, 2, sx, "/")

}

if (missing(breaks) || is.null(breaks) || length(breaks) <

1)

if (missing(col))

breaks <- 16

else breaks <- length(col) + 1

if (length(breaks) == 1) {

breaks <- seq(min(x, na.rm = na.rm), max(x, na.rm = na.rm),

length = breaks)

}

nbr <- length(breaks)

ncol <- length(breaks) - 1

if (class(col) == "function")

col <- col(ncol)

else if (is.character(col) && length(col) == 1)

col <- do.call(col, list(ncol))

min.breaks <- min(breaks)

max.breaks <- max(breaks)

x[] <- ifelse(x < min.breaks, min.breaks, x)

x[] <- ifelse(x > max.breaks, max.breaks, x)

lmat <- rbind(4:3, 2:1)

lhei <- lwid <- c(keysize, 4)

if (!missing(ColSideColors)) {

# if (!is.character(ColSideColors) || length(ColSideColors) !=

# nc)

# stop("'ColSideColors' must be a character vector of length ncol(x)")

if(is.null(nrow(ColSideColors))){

lmat <- rbind(lmat[1, ] + 1, c(NA, 1), lmat[2, ] + 1)

lhei <- c(lhei[1], 0.2, lhei[2])

}

else{

lmat <- rbind(lmat[1, ] + nrow(ColSideColors),

t(matrix(as.numeric(unlist(strsplit(paste("NA",1:nrow(ColSideColors))," "))),2,nrow(ColSideColors))),

lmat[2, ] + nrow(ColSideColors))

# lhei <- c(lhei[1], 0.2*nrow(ColSideColors), lhei[2])

lhei <- c(lhei[1], rep(0.2,nrow(ColSideColors)), lhei[2])

}

}

if (!missing(RowSideColors)) {

if (!is.character(RowSideColors) || length(RowSideColors) !=

nr)

stop("'RowSideColors' must be a character vector of length nrow(x)")

lmat <- cbind(lmat[, 1] + 1, c(rep(NA, nrow(lmat) - 1),

1), lmat[, 2] + 1)

lwid <- c(lwid[1], 0.2, lwid[2])

}

lmat[is.na(lmat)] <- 0

op <- par(no.readonly = TRUE)

on.exit(par(op))

layout(lmat, widths = lwid, heights = lhei, respect = FALSE)

if (!missing(RowSideColors)) {

par(mar = c(margins[1], 0, 0, 0.5))

image(rbind(1:nr), col = RowSideColors[rowInd], axes = FALSE)

}

if (!missing(ColSideColors)) {

if(is.null(nrow(ColSideColors))) {

par(mar = c(0.5, 0, 0, margins[2]))

image(cbind(1:nc), col = ColSideColors[colInd], axes = FALSE)

}

else{

for(kk in 1:nrow(ColSideColors)){

par(mar = c(0.5, 0, 0, margins[2]))

image(cbind(1:nc), col = ColSideColors[kk,colInd], axes = FALSE)

axis(4, 0, labels = rownames(ColSideColors)[kk], las = 2,

line = -0.5, tick = 0, cex.axis = cexCol)

}

}

}

par(mar = c(margins[1], 0, 0, margins[2]))

if (!symm || scale != "none") {

x <- t(x)

cellnote <- t(cellnote)

}

if (revC) {

iy <- nr:1

ddr <- rev(ddr)

x <- x[, iy]

cellnote <- cellnote[, iy]

}

else iy <- 1:nr

image(1:nc, 1:nr, x, xlim = 0.5 + c(0, nc), ylim = 0.5 +

c(0, nr), axes = FALSE, xlab = "", ylab = "", col = col,

breaks = breaks, ...)

if (!invalid(na.color) & any(is.na(x))) {

mmat <- ifelse(is.na(x), 1, NA)

image(1:nc, 1:nr, mmat, axes = FALSE, xlab = "", ylab = "",

col = na.color, add = TRUE)

}

axis(1, 1:nc, labels = labCol, las = 2, line = -0.5, tick = 0,

cex.axis = cexCol)

if (!is.null(xlab))

mtext(xlab, side = 1, line = margins[1] - 1.25)

axis(4, iy, labels = labRow, las = 2, line = -0.5, tick = 0,

cex.axis = cexRow)

if (!is.null(ylab))

mtext(ylab, side = 4, line = margins[2] - 1.25)

if (!missing(add.expr))

eval(substitute(add.expr))

if (!missing(colsep))

for (csep in colsep) rect(xleft = csep + 0.5, ybottom = rep(0,

length(csep)), xright = csep + 0.5 + sepwidth[1],

ytop = rep(ncol(x) + 1, csep), lty = 1, lwd = 1,

col = sepcolor, border = sepcolor)

if (!missing(rowsep))

for (rsep in rowsep) rect(xleft = 0, ybottom = (ncol(x) +

1 - rsep) - 0.5, xright = ncol(x) + 1, ytop = (ncol(x) +

1 - rsep) - 0.5 - sepwidth[2], lty = 1, lwd = 1,

col = sepcolor, border = sepcolor)

min.scale <- min(breaks)

max.scale <- max(breaks)

x.scaled <- scale01(t(x), min.scale, max.scale)

if (trace %in% c("both", "column")) {

for (i in colInd) {

if (!is.null(vline)) {

vline.vals <- scale01(vline, min.scale, max.scale)

abline(v = i - 0.5 + vline.vals, col = linecol,

lty = 2)

}

xv <- rep(i, nrow(x.scaled)) + x.scaled[, i] - 0.5

xv <- c(xv[1], xv)

yv <- 1:length(xv) - 0.5

lines(x = xv, y = yv, lwd = 1, col = tracecol, type = "s")

}

}

if (trace %in% c("both", "row")) {

for (i in rowInd) {

if (!is.null(hline)) {

hline.vals <- scale01(hline, min.scale, max.scale)

abline(h = i + hline, col = linecol, lty = 2)

}

yv <- rep(i, ncol(x.scaled)) + x.scaled[i, ] - 0.5

yv <- rev(c(yv[1], yv))

xv <- length(yv):1 - 0.5

lines(x = xv, y = yv, lwd = 1, col = tracecol, type = "s")

}

}

if (!missing(cellnote))

text(x = c(row(cellnote)), y = c(col(cellnote)), labels = c(cellnote),

col = notecol, cex = notecex)

par(mar = c(margins[1], 0, 0, 0))

if (dendrogram %in% c("both", "row")) {

plot(ddr, horiz = TRUE, axes = FALSE, yaxs = "i", leaflab = "none")

}

else plot.new()

par(mar = c(0, 0, if (!is.null(main)) 5 else 0, margins[2]))

if (dendrogram %in% c("both", "column")) {

plot(ddc, axes = FALSE, xaxs = "i", leaflab = "none")

}

else plot.new()

if (!is.null(main))

title(main, cex.main = 1.5 * op[["cex.main"]])

if (key) {

par(mar = c(5, 4, 2, 1), cex = 0.75)

if (symkey) {

max.raw <- max(abs(x), na.rm = TRUE)

min.raw <- -max.raw

}

else {

min.raw <- min(x, na.rm = TRUE)

max.raw <- max(x, na.rm = TRUE)

}

z <- seq(min.raw, max.raw, length = length(col))

image(z = matrix(z, ncol = 1), col = col, breaks = breaks,

xaxt = "n", yaxt = "n")

par(usr = c(0, 1, 0, 1))

lv <- pretty(breaks)

xv <- scale01(as.numeric(lv), min.raw, max.raw)

axis(1, at = xv, labels = lv)

if (scale == "row")

mtext(side = 1, "Row Z-Score", line = 2)

else if (scale == "column")

mtext(side = 1, "Column Z-Score", line = 2)

else mtext(side = 1, "Value", line = 2)

if (density.info == "density") {

dens <- density(x, adjust = densadj, na.rm = TRUE)

omit <- dens$x < min(breaks) | dens$x > max(breaks)

dens$x <- dens$x[-omit]

dens$y <- dens$y[-omit]

dens$x <- scale01(dens$x, min.raw, max.raw)

lines(dens$x, dens$y/max(dens$y) * 0.95, col = denscol,

lwd = 1)

axis(2, at = pretty(dens$y)/max(dens$y) * 0.95, pretty(dens$y))

title("Color Key\nand Density Plot")

par(cex = 0.5)

mtext(side = 2, "Density", line = 2)

}

else if (density.info == "histogram") {

h <- hist(x, plot = FALSE, breaks = breaks)

hx <- scale01(breaks, min.raw, max.raw)

hy <- c(h$counts, h$counts[length(h$counts)])

lines(hx, hy/max(hy) * 0.95, lwd = 1, type = "s",

col = denscol)

axis(2, at = pretty(hy)/max(hy) * 0.95, pretty(hy))

title("Color Key\nand Histogram")

par(cex = 0.5)

mtext(side = 2, "Count", line = 2)

}

else title("Color Key")

}

else plot.new()

invisible(list(rowInd = rowInd, colInd = colInd))

}

#************have now defined heatmap.csm function****************

# load packages

library(gplots)

library(graphite)

library(Rgraphviz)

library(affy)

# read in variant data from tab-delimietd text file containing row and column headers, columns are all the tumours in the study, rows are all the genes (lablled by OGS) that have variants in the tumour (variant = 1, non varient = 0) as well as, below these genes, added in all genes in any of the KEGG pathways not included above based on having a variant in at least one tumour (obviosuly, all of these genes for all tumours will be 0).

FileChoice<-file.choose() # choose input file

tumour.variants<- read.delim(FileChoice, skip=0, sep="\t", as.is=TRUE, header=TRUE) # (must be in same order as npnew, below)

rownames(tumour.variants)<-tumour.variants[,1]

tumour.variants<-tumour.variants[,-1]

tumour.variants[1:5,1:5] # to check

tumour.names<-colnames(tumour.variants)

# read in annotation data for tumours for the genes in this pathway (genes expected to be in same order as in the variant data file read into the tumour.variants object above).

FileChoice<-file.choose()

ann<-read.delim(FileChoice, skip=0, sep="\t", as.is=TRUE, header=TRUE)

rownames(ann)<-ann[,1]

ann<-ann[,-1]

ann[1:50,] # to check

# trial file is called "~2a~tumour_annotations_May2013drugbank.txt"

# read in KEGG parhways from a tab-delimited text file. Two columns, pathway in column 1 and gene (OGS) of pathway member in column 2

FileChoice<-file.choose()

kegg.paths<-read.delim(FileChoice, skip=0, sep="\t", as.is=TRUE, header=TRUE)

kegg.paths[1:5,] # to check

kegg.paths.names<-unique(kegg.paths[,1])

# define an object of for number of tumours

nrk<-length(kegg.paths.names)

# configuration switch for which pathways to use, or to use all

# pathway.choice<-"all"

pathway.choice<-c("MAPK signaling pathway")

if(pathway.choice=="all") {pathway.choice<-kegg.paths.names} #IGNORE WARNGIN MESSAGE

lpc<-length(pathway.choice)

# transpose and process the tumour variants matrix

t.tumour.variants<-t(tumour.variants)

t.tumour.variants[1:5,1:5] # to check

# define an object of for number of tumours

nrt<-nrow(t.tumour.variants)

# ============================================

# main pathway loop starts here and runs to end of this script

# ============================================

for(pathway.count in 1:lpc){

pathway <- kegg[[pathway.choice[pathway.count]]]

print(paste("pathway count", pathway.count,"of",lpc))

ogs.pathway<-convertIdentifiers(pathway, "symbol")

ogs.pathway@nodes<-kegg.paths[kegg.paths$KEGG.PATHWAY== ogs.pathway@title,2] #trim pathway down to my predefined node set, from the pathway file loaded into object kegg.paths

# get the tumour variants for just the pathway we are plotting

zz<-t.tumour.variants[,colnames(t.tumour.variants)%in%ogs.pathway@nodes]

zz<-zz[,match(ogs.pathway@nodes,colnames(zz))] # reorder to match pathway nodes

# get the annotations for just the pathway we are plotting

ant<-ann[rownames(ann)%in%ogs.pathway@nodes,]

ant<-ant[match(ogs.pathway@nodes,rownames(ant)),] # reorder to match pathway nodes

# to set up pathway diagram annotation

nAttrs <- list() #

z<-nodes(ogs.pathway) #

names(z)<-nodes(ogs.pathway) #

nAttrs$label <- z #

# to set up annotated heatmap annotation and clustering

# Set up colours for plotting heatmap annotations

aa<-c("lightgrey","red")

bb<-c("lightgrey","yellow")

cc<-c("lightgrey","orange", "red")

dd<-c("lightgrey","blue")

ee<-c("lightgrey","brown")

ff<-c("lightgrey","purple")

cn<-rbind(aa[ant$Drug.Target], bb[ant$Druggability], cc[ant$Variant.Incidence], dd[ant$Survival.Expression.Association],ee[ant$Cancer.Gene.Census],ff[ant$Melanoma.Driver])

rownames(cn)<-colnames(ant)

#define clustering functions for plotting

my.dist <- function(x) dist(x, method="binary")

#one of "euclidean", "maximum", "manhattan", "canberra", "binary" or "minkowski"

my.hclust <- function(d) hclust(d, method="single")

# one of "ward", "single", "complete", "average", "mcquitty", "median" or "centroid"

# set up to print to pdf

pdf(paste("tumour analysis KEGG",ogs.pathway@title,date()),width=12,height=8)

mainp=paste("KEGG pathway =",ogs.pathway@title)

# first annotated heatmap

heatmap.csm(zz,col=colorpanel(2,low="grey",high="blue"), main=mainp, ColSideColors=cn, distfun =my.dist, hclustfun=my.hclust,Colv=TRUE,scale="none", key=FALSE, density.info="density", trace="none",cexRow=0.1, cexCol=0.2, margins = c(10, 10))

#then prepaer for and plot waterfall plot

q<-colSums(zz)

s<-sort(q, decreasing = TRUE)

#build geometric series from mutation frequency ranking

u<-rbind(sort(colSums(zz), decreasing = TRUE),Reduce("%/%",rep(2,(ncol(zz)-1)),init=2^(ncol(zz)-1),accum=TRUE))

# obtain gene order forplotting

rankgene<- rank(q, ties.method = "first")

numtumours<-nrow(zz)

tscore<-vector(mode="numeric",length=nrow(zz))

names( tscore)<-rownames(zz)

for(count in 1:nrow(zz)){

#for each tumour

v<-zz[count,]

w<-v[v==1]

nw<-names(w)

tscore[count]<-sum(u[2,colnames(u)%in%nw])

}

ranktumour<- rank(tscore, ties.method = "first")

zzz<-zz[order(ranktumour, decreasing = TRUE), order(rankgene, decreasing = TRUE)]

cn<-cn[,order(rankgene, decreasing = TRUE)] #reorder annotations

heatmap.csm(zzz,col=colorpanel(2,low="grey",high="blue"), Rowv=FALSE, main=mainp, ColSideColors=cn, distfun =my.dist, hclustfun=my.hclust,Colv=FALSE,scale="none", key=FALSE, density.info="density", trace="none",cexRow=0.1, cexCol=0.5, margins = c(10, 10))

for(tumour.count in 1:nrt){

print(paste("tumour", tumour.count,"of",nrt))

# plot annotated pathway diagram

zzr<-zz[tumour.count,] # vector to indicate those gene that have mutations in this tumor in this pathway

zzr[zzr==1]<-7 # to allow yellow (7) fill to identify variants in each tumour

zzl<-ant$Drug.Target # vector to indicate those gene that have drugged in this tumor in this pathway

zzl[zzl==2]<-"red"

zzs<-ant$Survival.Expression.Association # vector to indicate those gene that have patient survival associations

zzs[zzs==2]<-"blue"

names(zzr)<-nodes(ogs.pathway)

names(zzl)<-nodes(ogs.pathway)

names(zzs)<-nodes(ogs.pathway)

nAttrs$fillcolor<-zzr

nAttrs$color<-zzl

nAttrs$fontcolor<-zzs

g <- pathwayGraph(ogs.pathway)

maine=paste(mainp,": tumour =",tumour.names[tumour.count], ": Yellow Fill = gene variant, Blue Text = expression-survival association, Red Boarder = drug")

plot(g, main=maine, cex.main=0.5, nodeAttrs = nAttrs, recipEdges = "combined", attrs = list(node = list(height = 4, width = 4, fontsize=32),edge = list(color = "grey")))

}

dev.off()

}# end of main pathway loop

#**************

#**************

# R script below is to perform gene set enrichment analysis using the GATHER web tool (http://gather.genome.duke.edu) to identify any KEGG pathways for which genes somatically altered in each tumour were significantly enriched.

options(warn=-1)

print("For GATHER analysis what is FDR cutoff? (reccommend conservative starting point is 0.001)")

Gcut<-as.numeric(readLines(con = stdin(), n = 1, ok = TRUE))

print("What name for output file?")

fileOut<-as.character(readLines(con = stdin(), n = 1, ok = TRUE))

#Read in the data file an sort it ***by tumour***

FileChoice1<-file.choose()

bbb<-read.delim(FileChoice1,header=FALSE,skip=1,sep="\t", as.is = TRUE) #read in the network

a<-bbb[,1]; b<-bbb[,2]

print("Data read in OK")

#Order by tumours

dd <- transform(data.frame(a,b), a = factor(a))

e <- dd[ do.call(order, dd) ,] #e is the sorted data frame

print("Data reordered OK")

#Count numbers of genes

e$num_children<-0 # set up third column to which we will add number of genes

e$hist<-0 # set up third column to indicate first occurances for use later in histogram

colnames(e)<-c("P","C","num_genes","hist")

UP<-unique(e$P) # get a non-redundant list of the tumours

for (cycle1 in 1:length(UP)) { # cycle through the non-redundant tumours adding number of genes to column 3 as you go

print(paste("Cycling through non-redundant tumours to get number of genes",cycle1,"of",length(UP)))

e[e$P==UP[cycle1],3]<-length(e[e$P==UP[cycle1],3])

}

e[1,4]<-1

for (cycleX in 2:length(e[,1])) { # cycle through e setting e$hist to one on last occurances of tumour

print(paste("Cycling through e$hist to get last tumour occurance",cycleX,"of",length(e[,1])))

if(e[cycleX,1]!=e[cycleX-1,1]) {

e[cycleX,4]<-1

}}

hi<-e[e$hist==1,3]

hist(hi, breaks = 1:max(hi),density=20, main="histogram of #genes", xlab="#genes")

print("")

print("Based on the histogram, what is the minimum number of genes you want to work on? (note that the Gather querry will only work is less than 500 genes)")

Cmin<-as.numeric(readLines(con = stdin(), n = 1, ok = TRUE))

print("")

#Filter so that tumours only remain if they have ≥ Cmin genes

e_filt<-e[e$num_children>=Cmin,]

UP_filt<-unique(e_filt$P) # get a non-redundant list of the filtered tumours

library(hgu133a2.db) #get OGS of focus_P

UP_filt2<-as.character(UP_filt)

db<-mget(UP_filt2, hgu133a2SYMBOL, ifnotfound=NA) #get OGSs

dd<-data.frame(cbind(UP_filt2, db))

for (cycle2 in 1:length(UP_filt)) { # cycle through the non-redundant tumours to generate tumour and gene lists for Gather analysis

focus_P<-UP_filt[cycle2]

focus_children<-e_filt[e_filt$P==focus_P,2]

nnc<-length(focus_children)

print("")

print(paste("Looking up annotations of the", nnc, "genes of tumuor ",focus_P, "(number ", cycle2, "of ", length(UP_filt), ")"))

focus_Pname<-dd[dd$UP_filt2==focus_P,2]

dest<-paste("<<< ", nnc, " genes of ",focus_P, " : ", focus_Pname, " >>>")

#Convert the probeID list into a character string with probes separated by "+"

t<-as.character(focus_children[1])

for (xx in 2:length(focus_children)) {

t<-paste(t,focus_children[xx],sep = "+")

}

#Prepare the script strnigs to make requests to ***Gather***

v_GO<-paste("http://gather.genome.duke.edu/?cmd=report&gene_box=",t,"&tax_id=9606&annot_type=gene_ontology&network=0&homologs=0",sep = "")

v_KE<-paste("http://gather.genome.duke.edu/?cmd=report&gene_box=",t,"&tax_id=9606&annot_type=kegg&network=0&homologs=0",sep = "")

v_TF<-paste("http://gather.genome.duke.edu/?cmd=report&gene_box=",t,"&tax_id=9606&annot_type=transfac&network=0&homologs=0",sep = "")

v_PR<-paste("http://gather.genome.duke.edu/?cmd=report&gene_box=",t,"&tax_id=9606&annot_type=proteins&network=0&homologs=0",sep = "")

v_MI<-paste("http://gather.genome.duke.edu/?cmd=report&gene_box=",t,"&tax_id=9606&annot_type=mirna&network=0&homologs=0",sep = "")

v_CH<-paste("http://gather.genome.duke.edu/?cmd=report&gene_box=",t,"&tax_id=9606&annot_type=maploc&network=0&homologs=0",sep = "")

#Make the querries - obviously, YOU NEED TO BE ON LINE

print("Querrying Gather for Gene_Ontology")

q_GO<-read.delim(v_GO)

print("Querrying Gather for KEGG")

q_KE<-read.delim(v_KE)

print("Querrying Gather for Transfac")

q_TF<-read.delim(v_TF)

print("Querrying Gather for Protein-Protein interactions")

q_PR<-read.delim(v_PR)

print("Querrying Gather for miRNAs")

q_MI<-read.delim(v_MI)

print("Querrying Gather for Chromosomal Location")

q_CH<-read.delim(v_CH)

print("")

print("")

#Reformat Gether output to retain only esssential columns

print("")

print("Reformatting Gather for Gene_Ontology")

q_GO$FDR<-exp(-q_GO$FE..neg.ln.FDR.)

t_GO<-q_GO[q_GO$FDR<=Gcut,c(2,3,8,12,13)]

t_GO<-cbind(rep(as.character(focus_P),nrow(t_GO)),rep(nnc,nrow(t_GO)),t_GO)

colnames(t_GO)[1]<-paste(dest,"*****Gene_Ontology*****")

print("Reformatting Gather for KEGG")

q_KE$FDR<-exp(-q_KE$FE..neg.ln.FDR.)

t_KE<-q_KE[q_KE$FDR<=Gcut,c(2,3,8,12,13)]

t_KE<-cbind(rep(as.character(focus_P),nrow(t_KE)),rep(nnc,nrow(t_KE)),t_KE)

colnames(t_KE)[1]<-paste(dest,"*****KEGG*****")

print("Reformatting Gather for Transfac")

q_TF$FDR<-exp(-q_TF$FE..neg.ln.FDR.)

t_TF<-q_TF[q_TF$FDR<=Gcut,c(2,3,8,12,13)]

t_TF<-cbind(rep(as.character(focus_P),nrow(t_TF)),rep(nnc,nrow(t_TF)),t_TF)

colnames(t_TF)[1]<-paste(dest,"*****TRANSFAC*****")

print("Reformatting Gather for Protein-Protein interactions")

q_PR$FDR<-exp(-q_PR$FE..neg.ln.FDR.)

t_PR<-q_PR[q_PR$FDR<=Gcut,c(2,3,8,12,13)]

t_PR<-cbind(rep(as.character(focus_P),nrow(t_PR)),rep(nnc,nrow(t_PR)),t_PR)

colnames(t_PR)[1]<-paste(dest,"*****Protein_Binding*****")

print("Reformatting Gather for miRNAs")

q_MI$FDR<-exp(-q_MI$FE..neg.ln.FDR.)

t_MI<-q_MI[q_MI$FDR<=Gcut,c(2,3,8,12,13)]

t_MI<-cbind(rep(as.character(focus_P),nrow(t_MI)),rep(nnc,nrow(t_MI)),t_MI)

colnames(t_MI)[1]<-paste(dest,"*****miRNA*****")

print("Reformatting Gather for Chromosome Location")

q_CH$FDR<-exp(-q_CH$FE..neg.ln.FDR.)

t_CH<-q_CH[q_CH$FDR<=Gcut,c(2,3,8,12,13)]

t_CH<-cbind(rep(as.character(focus_P),nrow(t_CH)),rep(nnc,nrow(t_CH)),t_CH)

colnames(t_CH)[1]<-paste(dest,"*****Chromosome Location*****")

print("")

print("")

#Appending to the tab-delimited text output file

print("Appending to output file")

write.table(t_GO,file=fileOut, sep="\t", row.names=FALSE, col.names= TRUE,append=TRUE, quote = FALSE)

write.table(t_KE,file=fileOut, sep="\t", row.names=FALSE, col.names= TRUE,append=TRUE, quote = FALSE)

write.table(t_TF,file=fileOut, sep="\t", row.names=FALSE, col.names= TRUE,append=TRUE, quote = FALSE)

write.table(t_PR,file=fileOut, sep="\t", row.names=FALSE, col.names= TRUE,append=TRUE, quote = FALSE)

write.table(t_MI,file=fileOut, sep="\t", row.names=FALSE, col.names= TRUE,append=TRUE, quote = FALSE)

write.table(t_CH,file=fileOut, sep="\t", row.names=FALSE, col.names= TRUE,append=TRUE, quote = FALSE)

write.table("",file=fileOut, sep="\t", row.names=FALSE, col.names=FALSE,append=TRUE, quote = FALSE)

write.table("",file=fileOut, sep="\t", row.names=FALSE, col.names=FALSE,append=TRUE, quote = FALSE)

write.table("",file=fileOut, sep="\t", row.names=FALSE, col.names=FALSE,append=TRUE, quote = FALSE)

}
